# A flexible representation of omic knowledge for thorough analysis of microarray data

**DOI:** 10.1186/1746-4811-2-5

**Published:** 2006-03-02

**Authors:** Yoshikazu Hasegawa, Motoaki Seki, Yoshiki Mochizuki, Naohiko Heida, Katsura Hirosawa, Naoki Okamoto, Tetsuya Sakurai, Masakazu Satou, Kenji Akiyama, Kei Iida, Kisik Lee, Shigehiko Kanaya, Taku Demura, Kazuo Shinozaki, Akihiko Konagaya, Tetsuro Toyoda

**Affiliations:** 1 Phenome Informatics Team, Functional Genomics Research Group, Genomic Sciences Center, RIKEN, Suehiro, Tsurumi, Yokohama, Kanagawa, Japan; 2 Plant Functional Genomics Research Team, Functional Genomics Research Group, Genomic Sciences Center, RIKEN, Suehiro, Tsurumi, Yokohama, Kanagawa, Japan; 3 NEC Infomatec Systems Ltd, Sakato, Takatsu, Kawasaki, Kanagawa, Japan; 4 Integrated Genome Informatics Research Unit, Metabolomics Group, Plant Science Center, RIKEN, Suehiro, Tsurumi, Yokohama, Kanagawa, Japan; 5 Faculty of Bio-Science, Nagahama Institute of Bio-Science and Technology, Tamura, Nagahama, Shiga, Japan; 6 IT technology research institute, Taehung Telcom co., Ltd., Dangsan-dong 3-ga 402, Youngdungpo-gu, Seoul, South Korea; 7 Laboratory of Comparative Genomics, Department of Bioinformatics and Genomics, Graduate School of Information Science, NARA Institute of Science and Technology, Takayama, Ikoma, Nara, Japan; 8 Morphoregulation Research Team, Plant Productivity Systems Research Group, Plant Science Center, RIKEN, Suehiro, Tsurumi, Yokohama, Kanagawa, Japan; 9 Plant Science Center, RIKEN, Suehiro, Tsurumi, Yokohama, Kanagawa, Japan; 10 Advanced Genome Information Technology Research Group, Genomic Sciences Center, RIKEN Suehiro, Tsurumi, Yokohama, Kanagawa, Japan

## Abstract

**Background:**

In order to understand microarray data reasonably in the context of other existing biological knowledge, it is necessary to conduct a thorough examination of the data utilizing every aspect of available omic knowledge libraries. So far, a number of bioinformatics tools have been developed. However, each of them is restricted to deal with one type of omic knowledge, e.g., pathways, interactions or gene ontology. Now that the varieties of omic knowledge are expanding, analysis tools need a way to deal with any type of omic knowledge. Hence, we have designed the Omic Space Markup Language (OSML) that can represent a wide range of omic knowledge, and also, we have developed a tool named GSCope3, which can statistically analyze microarray data in comparison with the OSML-formatted omic knowledge data.

**Results:**

In order to test the applicability of OSML to represent a variety of omic knowledge specifically useful for analysis of *Arabidopsis thaliana *microarray data, we have constructed a Biological Knowledge Library (BiKLi) by converting eight different types of omic knowledge into OSML-formatted datasets. We applied GSCope3 and BiKLi to previously reported *A. thaliana *microarray data, so as to extract any additional insights from the data. As a result, we have discovered a new insight that lignin formation resists drought stress and activates transcription of many water channel genes to oppose drought stress; and most of the 20S proteasome subunit genes show similar expression profiles under drought stress. In addition to this novel discovery, similar findings previously reported were also quickly confirmed using GSCope3 and BiKLi.

**Conclusion:**

GSCope3 can statistically analyze microarray data in the context of any OSML-represented omic knowledge. OSML is not restricted to a specific data type structure, but it can represent a wide range of omic knowledge. It allows us to convert new types of omic knowledge into datasets that can be used for microarray data analysis with GSCope3. In addition to BiKLi, by collecting various types of omic knowledge as OSML libraries, it becomes possible for us to conduct detailed thorough analysis from various biological viewpoints. GSCope3 and BiKLi are available for academic users at our web site .

## Background

Since microarray analysis was first developed as a technique for analyzing gene expression simultaneously [1,2], functional investigation of genes has been actively carried out using microarrays and novel findings have been obtained. However, there is always a possibility that some gene functions to be discovered are overlooked by biologists analyzing the microarray data, because the amount of gene expression information detected by microarray is so vast that it is difficult to analyze the obtained data fully. Therefore, various methods and tools for analyzing microarray data have been developed, especially comparing microarray data with biological knowledge [3-6]. The importance of gene expression in biological networks (for example, metabolic pathways) is noted [7,8]. Dahlquist et al. [4] have developed a tool which can display the gene expression profiles of microarray data on biological networks.

Regarding conceptually structured ontology of gene functions, the Gene Ontology Consortium [9] is providing a set of structured vocabularies for specific biological domains, which can be used to describe gene products in any organism. Doniger et al. [5] have developed a tool which can display the gene expression profile of microarray data on a directed acyclic graph of Gene Ontology (GO). On the other hand, Thimm et al. [6] have developed a tool which can display the gene expression of microarray data on metabolic pathways and other biological processes. GeneSpring (Silicon Genetics, Redwood City, CA, USA) can display microarray data on the figure of a gene positioned on a genome. However, these tools give priority to the display of a certain type of data and cannot analyze microarray data from multiple view points. It is desired that various form of biological knowledge are represented by a flexible language and can be used for microarray analyses by a single universal tool.

**Figure 1:**
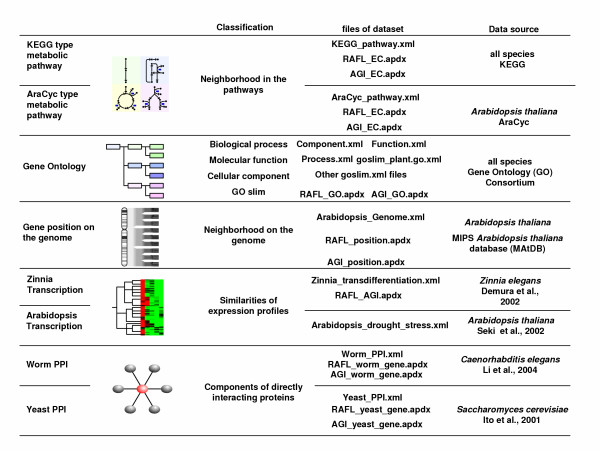
**Contents of Biological Knowledge Library (BiKLi)**. The BiKLi is constructed by biological knowledge from eight public databases and literature.

**Figure 2:**
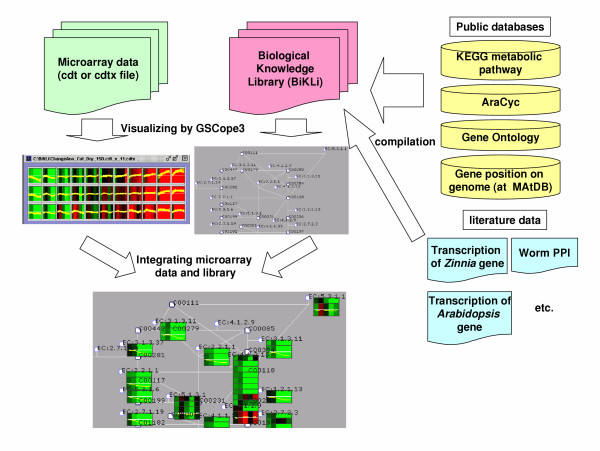
**Integration of the Biological Knowledge Library (BiKLi) and microarray data**. The BiKLi is constructed by biological knowledge from public databases and literature (right). The BiKLi can be accessed using OSML Editor, which allows integration of the BiKLi and microarray data. OSML Editor is used to display the library (centre grey display). Enzymes are shown in a light blue circle frame. The metabolite is shown with black square frame. The microarray probe corresponding to the enzyme is shown as a rectangular head under the enzyme. The microarray data in cdtx format is graphed on the left central display. Each cluster is shown at this display. We can integrate, display and this array and BiKLi data. (The combination of the array and BiKLi data is presented.) (bottom display)

A number of bioinformatics tools have been developed. However, they are restricted to deal with only a few types of omic knowledge, e.g., pathways, interactions or gene ontology. Now that the varieties of omic knowledge are expanding, analysis tools need a way to handle any type of omic knowledge. Hence, we have designed the Omic Space Markup Language (OSML) format which is able to convey various elements of biological knowledge [10]. OSML format is highly flexible, can describe a wide range of biological knowledge, and is designed to allow users to prepare their own data in the OSML format [10]. In order to show, through examples, that OSML format data can express the metabolic pathway, directed acyclic graphs of GO terms, gene positions on genome, and protein-protein interaction, we have constructed a Biological Knowledge Library (BiKLi) by converting various biological knowledge and information sources into OSML format (Figure 1, Figure 2). BiKLi is available with GSCope3 for academic and non-profit users . To confirm the effectiveness of BiKLi and GSCope3, we have analyzed the microarray data of *A. thaliana *under drought stress treatments by applying BiKLi and GSCope3 [11]. The statistical ranking function provided in GSCope3 is so useful that the metabolic pathway, genome area, or GO relating to a specific gene expression pattern can be easily discovered. Moreover, users can perform various analyses by using GSCope3 using their own original data represented by OSML.

Plant growth, viability, and fertilization are greatly affected by environmental stresses, such as drought. Plants respond and adapt to these stresses in order to survive. These stresses induce various biochemical and physiological responses in plants, which are followed by a change of gene expression. For this reason, analysis of gene expression under stress is important in plant molecular biology, biochemistry, and physiology. Hence, some biologists have studied gene expression in plants under these stressful conditions [12-15]. Moreover, studies on expression profiling under stress conditions using microarray technology have been published [16-18]. As mentioned above, the analysis of gene expression under stress is very important and many biologists are interested in this study. Hence, we analyzed the microarray data of *A. thaliana *under stress.

In this paper, we report and present the results of 7 K RIKEN *Arabidopsis *Full-Length (RAFL) cDNA microarray analysis under drought stress treatments [18] by using BiKLi and GSCope3, and further discuss the utility of the BiKLi for microarray data analysis.

## Results and discussion

### KEGG type metabolic pathway

Additional File 1 shows a list of significant correlations between the "functional Class" and clusters formed by batch-learning self-organizing maps (BL-SOM) [19] of the expression profile of microarray probes under drought conditions). In this table, header means as follows: A is the number of probes in the selected pathway ("functional Class") and the selected cluster, B is the number of probes out of the selected pathway and in the selected cluster, C is the number of probes in the selected pathway and out of the selected cluster, D is the number of probes out of the selected pathway and the selected cluster, P is the probability function, P' is Bonferroni Corrected P, and N is number of pathway ("functional Class"). The colour of expression means as follows: red is up-regulation while green is down-regulation. A higher colour chroma denotes higher value. The "functional Class" shows a sub-pathway in the metabolic pathway.

**Figure 3:**
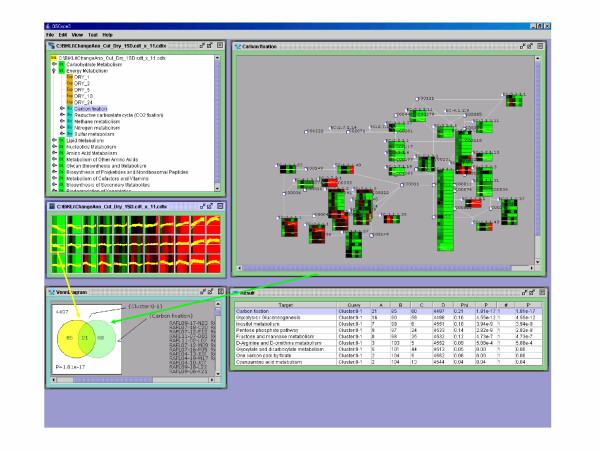
**Visualization of the relationship between the "carbon fixation pathway" and the gene expression profile which reduces the transcription**. The whole screen shows cluster ID 0-1 (the yellow frame on the left) after it was specified and ranked. The upper left display screen shows the data of the first principal ingredient set to 11 and clustered by BL-SOM in OSML Editor. The colour of the classified clusters indicates the mean values of the probe data included in the cluster in each experiment. Each yellow line in the cluster represents an expression profile of the probes in the cluster. The upper right display screen shows the details of the hierarchy of OSML data. The middle right display screen is the "carbon fixation pathway" by OSML Editor and the array probe and array data correspond to specific enzymes and array probes, respectively. (Pathway View) The enzyme is presented as a light blue circle frame and the microarray probe corresponding to the enzyme is shown under each corresponding enzyme. The metabolites are shown in black square frame. The display illustrates the microarray data in the framed array probe. The bottom display screen provides details on the ranking results of clustering from the upper left display (cluster ID 0-1). The ranking is displayed on the right and the left is a Venn diagram of "carbon fixation pathway" and the probes (cluster 0-1). The "carbon fixation pathway" shown in the upper left corner of Figure 3 is at the top of the ranking. In the "carbon fixation pathway", 21 out of 81 probes are included in cluster 0-1.

A strong correlation was discovered between the "carbon fixation pathway" and gene expression profile which reduced the transcription after some time passed (cluster ID is 0-1 and 0-0) (Additional File 1), especially the genes classified in cluster 0-1 and became concentrated at the "Calvin cycle"(Figure 3). This signifies an existing relationship between the "carbon fixation pathway" and down-regulated genes under drought stress conditions. Indeed, Seki et al. [18] have found out that many photosynthesis-related genes are down-regulated by drought stress.

A significant correlation between "starch and sucrose metabolism" and the gene expression profile which enhanced the transcription after a period of time was revealed (cluster ID is 10-2) (Additional File 1). Common genes between this "functional Class" and this cluster are polygalacturonase, glycosyl hydrolase family 32 (beta-fructosidase) (EC:3.2.1.26, At1g62660), sucrose synthase (EC:2.4.1.13, At3g43190), and trehalose-6-phosphate phosphatase (EC:3.1.3.12, At4g12430). Cushman and Bohnert [20] have suggested that disaccharides (e.g. sucrose, raffinose, or trehalose) probably function as osmolytes in protecting cells from dehydration. Seki et al. [18] have noted that galactinol synthase, raffinose synthase, sucrose synthase, and trehalose-6-phosphate synthase genes are up-regulated genes under drought stress conditions. Moreover, Seki et al. [18] have discussed the correlation between the data and the findings by Cushman and Bohnert [20].

In this study, we discovered that BiKLi and GSCope3 determined in a fast and efficient way the same findings which Seki et al. had obtained [18].

### AraCyc type metabolic pathway

The KEGG type and AraCyc type metabolic pathways are biochemically different from each other. KEGG type metabolic pathway is focused on all organisms. On the other hand, AraCyc type metabolic pathway is focused on *Arabidopsis thaliana *only. Moreover, metabolic pathway can divide or branch into various patterns. Therefore, the two type pathways are not combined in this study.

Additional File 2 shows a list of significant correlations between the "functional Class" and the clusters formed by BL-SOM of the microarray probes' expression profile under drought conditions. Similar to the analysis using the KEGG type metabolic pathway dataset, a strong correlation was found between the "Calvin cycle" and the gene expression profile which later caused down-regulation of transcription (cluster ID is 0-1 and 0-0) (Additional File 2).

Another significant correlation was reported between the "serine-isocitrate lyase pathway" and the gene expression profile, which showed a weak peak two hours after the drought condition started (cluster ID is 3-0) (Additional File 2). Each gene encodes four enzymes: alanine-glyoxylate aminotransferase (EC:2.6.1.45, At2g13360), malate dehydrogenase (EC:1.1.1.37, At1g04410, At3g47520), phosphoenolpyruvate carboxylase (EC:4.1.1.31, At2g42600), and glycine hydroxymethyltransferase (EC 2.1.2.1, At4g32520). The enzymes act in the "serine-isocitrate lyase pathway" and are classified in the same cluster. However, the "serine-isocitrate lyase pathway" utilizes one carbon compound, such as formaldehyde in bacteria [21]. In addition, these detected enzymes do not act as regulated enzymes, such as hydroxypyruvate reductase (EC:1.1.1.81), glycerate kinase (EC:2.7.1.31), malate-CoA ligase (EC:6.2.1.9), or malyl-CoA lyase (EC:4.1.3.24) in the "serine-isocitrate lyase pathway". Therefore, the correlation between "serine-isocitrate lyase pathway" and the genes with weak expression profile may not be important. However, these detected enzymes produce malate from L-glycine. Perhaps, the reason these genes are up-regulated after drought conditions started is because the drought stress induces malate biosynthesis from glycine, not the induction of "serine-isocitrate lyase pathway".

Another significant correlation was revealed between the "lignin biosynthesis" or the "suberin biosynthesis" and the gene expression profile which peaked two hours after introducing drought conditions (cluster ID is 6-2) (Additional File 2). Lignification of the cell wall and secondary wall formation make the cell strong [22]. Therefore, it has been suggested that transcription of these genes is induced during drought stress because lignin synthesis can cause resistance to drought stress. In fact, lignin formation is observed at sites of wounding or pathogen attack, apparently in an effort to strengthen the wall at these sites of damage [23]. Perhaps, lignin formation takes place in *A. thaliana *to resist drought stress.

In this study, we discovered that BiKLi and GSCope3 efficiently determined similar findings which Seki et al. had obtained [18]. Moreover, the detection of novel information using BiKLi and GSCope3 revealed lignin formation in *A. thaliana *in order to resist drought stress.

### Directed acyclic graphs of GO terms

**Figure 4:**
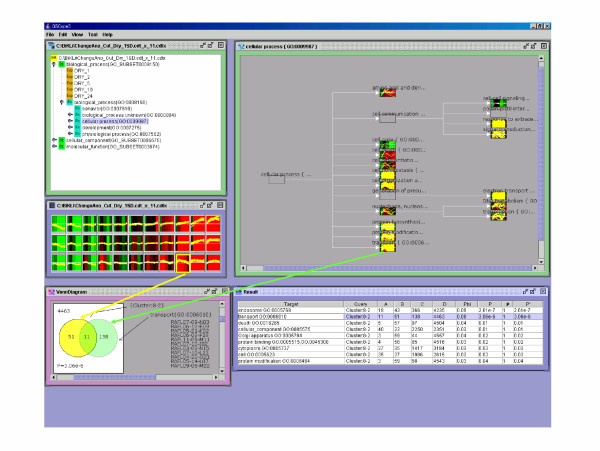
**Visualization of the relationship between transport (GO:0006810) and the gene expression profile which shows a strong peak two hours after introduction of drought stress**. Fig. 3 demonstrates the array probe and array data corresponding to GO terms and the array probes, respectively and the ranked GO terms. The upper right display screen shows the details of the hierarchy of OSML data. The middle left display screen is the first principal ingredient set to 11 and clustered by BL-SOM in OSML Editor. Goslim_plant.2003.xml in the upper right corner is displayed by "Functional Class View" in OSML Editor. The GO terms are in rectangular frames. The lower display provides the details on the ranking results (Ranking View) from information in the upper left frame (cluster ID 8-2). "Transport (GO:0006810)" is in second place. Eleven probes out of 149 in the GO:0006810 are included in cluster 8-2.

The Gene Ontology (GO) Consortium provides directed acyclic graphs of GO terms. However, these graphs are very large, so we attempted to use the Plant GO slim data for the microarray data analysis. Additional File 3 shows a list of significant correlations between the "functional Class" and the clusters formed by BL-SOM of the microarray probes' expression profile during drought conditions. A strong correlation was discovered between the "transport (GO:0006810)" (Figure 4) and the gene expression profile which showed a strong peak after two hours of drought conditions (cluster ID is 8-2) (Additional File 3).

In this study, many water channel genes, classified as transports (GO:0006810), are connected to the expression profile, which increases the transcription until two hours after drought conditions started, then subsequently decreases the transcription. These water channel genes are plasma membrane intrinsic protein 2A (At3g53420), transmembrane protein (MIP family) (At4g00430), plasma membrane intrinsic protein 2C (At2g37180), putative plasma membrane aquaporin (At3g54820), plasma membrane intrinsic protein 1A (At3g61430), and plasma membrane intrinsic protein 1c (At1g01620). It is thought that transcription of these genes become active to oppose drought stress. Perhaps, the transcription peak of these genes is two hours after drought stress begins. It is considered that transcription of these genes decreases and returns to the usual state two hours after changing to drought conditions because the necessity for transcription is lost.

Another significant correlation between the "development (GO:0007275)" and gene expression profile, which increased transcription two hours after drought conditions began, was noted (cluster ID is 10-1) (Additional File 3). These genes are late embryogenesis abundant (LEA) protein family gene (At4g02380), a late embryogenesis abundant protein LEA-like gene (At5g06760), and non-apical meristem (NAM) family protein gene (At4g27410). Seki et al. [18] found that the LEA protein genes are induced by drought stress, which is supported by the fact that LEA proteins are involved in protecting macromolecules [24].

In this study, using BiKLi and GSCope3, we discovered the same findings which Seki et al. had obtained [18]. The result was the activation of LEA protein genes by drought stress. Moreover, the novel information detected was the induced transcription of many water channel genes to oppose drought stress.

### Gene position on genome

**Figure 5:**
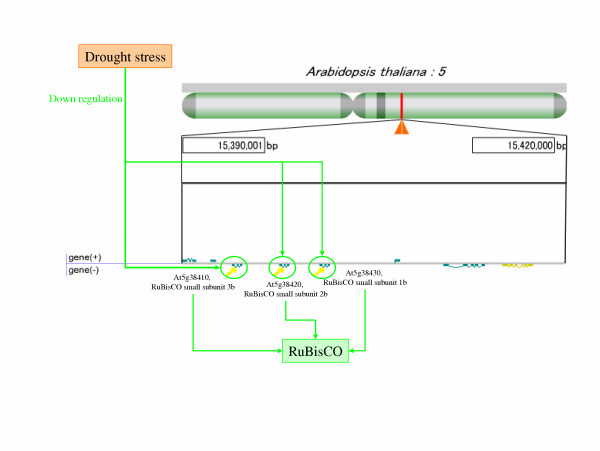
**Decrease of RuBisCO gene expression by drought stress**. The area of 15,390,001_15,420,000 bp in chromosome 5 is enlarged. Three RuBisCO small subunit genes stand on an identical strand. These three genes are probably controlled by drought stress similarly and are expressed together.

The analysis whether genes with similar expression profiles localizes at neighbourhoods on the genome or not is possible by using gene position data on the genome. Additional File 4 shows a list of significant correlations between the "functional Class" and the clusters formed by BL-SOM of the microarray probes' expression profile during drought conditions. A strong correlation was found between the area of 15,390,001–15,420,000 bp in chromosome 5 and the gene expression profile which decreased transcription after some time passed (cluster ID is 0-0) (Additional File 4). In this area, three ribulose 1,5-bisphosphate carboxylase/oxygenase (RuBisCO) small subunit genes exist [25]. It is likely that these RuBisCO genes are co-regulated in the neighbourhood area because they have similar expression profiles and form the same enzymes (Figure 5).

**Figure 6:**
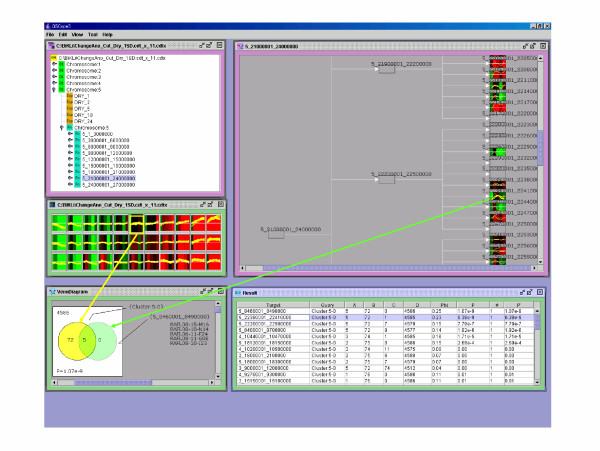
**Visualization of the relationship between the area of 22,680,001–22,710,000 bp in chromosome 5 and the gene expression profile which moderately increases transcription**. The array probe and the array data correspond to the section of every 30,000 bp and the array probe, respectively and then sections are ranked. The middle left display is the first principal ingredient set to 11 and clustered by BL-SOM in OSML Editor. (Graph View) In the upper right display the Arabidopsis_genome.xml is displayed by "Functional Class View" in OSML Editor. The rectangular frame indicates the classification of every 3,000,000 bp, every 300,000 bp, and every 30,000 bp. The display at the bottom provides details on the ranking results (Ranking View) of clustering from the data in the upper left display (clustering ID 5-0). "Chr: 5_15390001-15420000 bp" is in second place. Five probes out of six are included in cluster 5-0.

**Figure 7:**
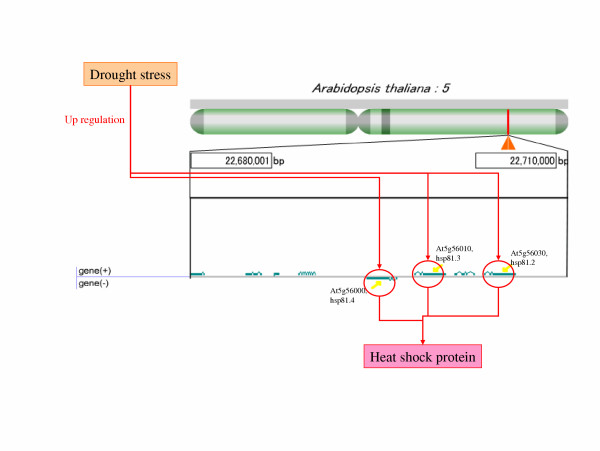
**Increase in gene expression of heat shock protein 90 (hsp90) genes by drought stress**. The area of 22,680,001_22,710,000 bp in chromosome 5 is enlarged. Two of the three hsp90 genes stand close together on an identical strand and others exist on a different strand. These three genes are probably controlled similarly by drought stress and are expressed together.

The correlation between the area of 22,680,001–22,710,000 bp in chromosome 5 and the gene expression profile, which moderately increased the transcription after a period of time, was considered significant (cluster ID is 5-0) (Additional File 4). Heat shock protein 81.4 (hsp81.4) (At5g56000), heat shock protein 90 (perhaps hsp81.3) (At5g56010), and heat shock protein 81.2 (hsp81.2) (At5g56030) existed in this area and they were classified in the same cluster (Figure 6). It has been suggested that these genes have similar expression profiles and there is a possibility of a similar control affecting these genes (Figure 7), although Milioni and Hatzopoulos [26] already revealed that these genes are clustered within the 15 kb genomic region on chromosome 5.

### Protein-protein interaction and *A. thaliana *gene expression

The information in protein-protein interaction is useful for examining the relationships between units of protein, and between ligand and receptor. In this study, only the relationship of the expressions between 20S proteasome genes was elucidated, specifically evident in *A. thaliana*. However, by using the information on protein-protein interaction in other organisms, the association of the expressions between the unclear genes in *A. thaliana *can be clarified. In this study, the details on protein-protein interaction on *Caenorhabditis elegans*, as well as the protein-protein interaction on *Saccharomyces cerevisiae *were built into BiKLi. It indicates that GSCope3 is capable of organizing the information on protein-protein interaction. In this study, we present the result on *C. elegans *protein-protein interaction (worm PPI) dataset.

**Figure 8:**
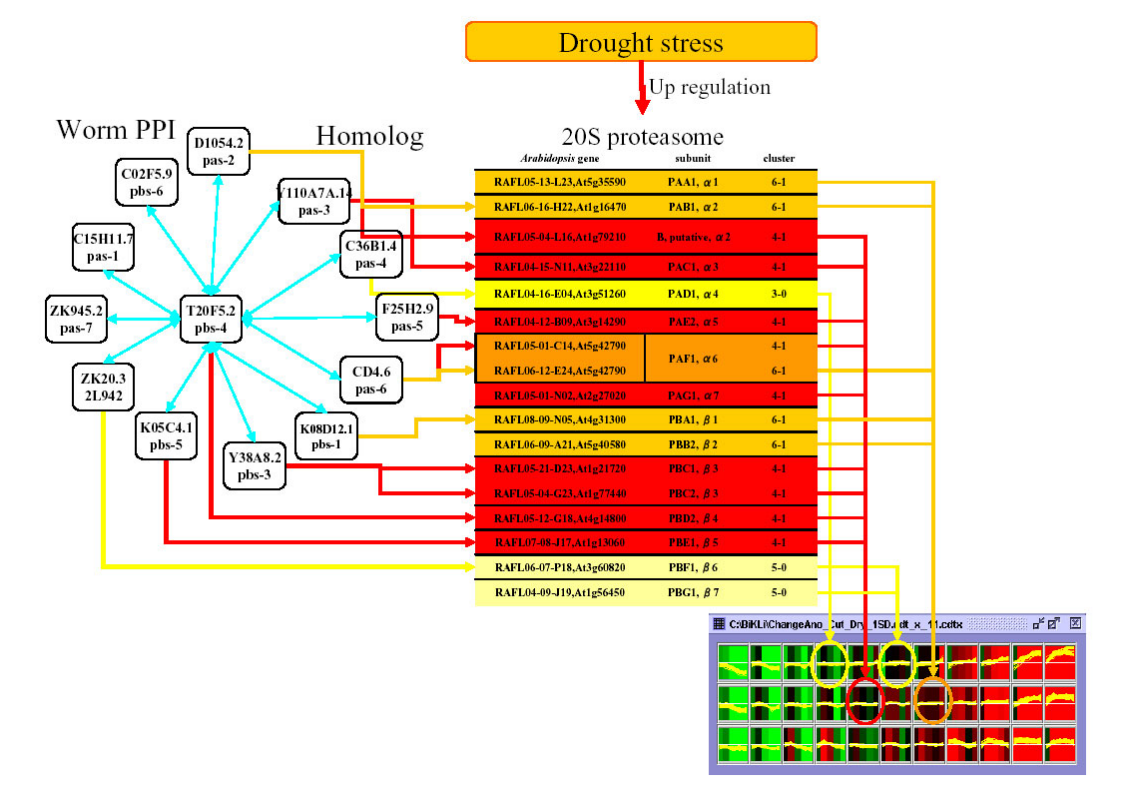
**The relationship between 20S proteasome family genes included in Worm PPI and the microarray data of *A. thaliana *gene**. The Worm gene T20F5.2 (pbs-4)-based interaction is shown on the left. In the centre, the relationship between *A. thaliana *genes, 20S proteasome subunits, and Worm genes is displayed. From the left, the gene (RAFL clone, ORF), subunit, and cluster ID of RAFL clone are indicated in the table. A line corresponding to the Worm gene is drawn on the left. The Graph View of the clustered array data is displayed on the right. The lines show the relationship between clusters and genes. Cluster 3-0 is shown in yellow, 5-0 in maize, 4-1 in red, and 6-1 in orange.

**Figure 9:**
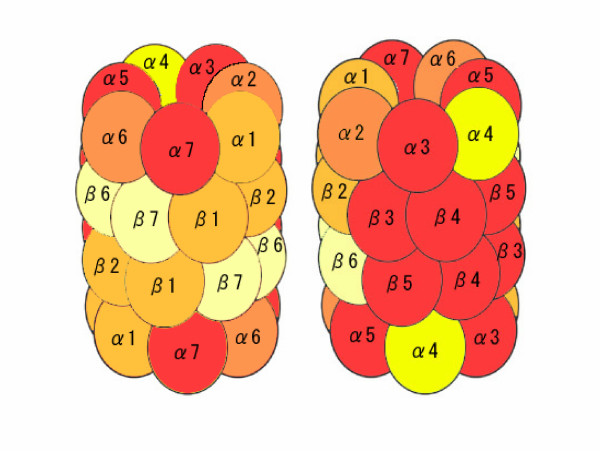
**The relationship of gene expression and subunit of 20S proteasome**. This figure shows a section of genes classified by using BL-SOM clustering based on microarray data corresponding to a structure of 20S proteasome complex. α3, α5, α7, β3, β4, and β5 included in cluster 4-1 are shown in red. α1, β1, and β2 included in 6-1 are in orange. α1 and α6 included in cluster 4-1 and 6-1 are in bright orange. α4 included in cluster 3-0 is shown in yellow and β6 and β7 included in cluster 5-0 are shown in maize.

Additional File 5 shows a list of significant correlations between the "functional Class" and the clusters formed by BL-SOM of the microarray probes' expression profile during drought conditions. A significant correlation was found between the "functional Class" which centred on the peptidase (T20F5.2, Y38A8.2) or proteasome component (C15H11.7, D1054.2) and the gene expression profile which showed a weak peak after two hours of drought conditions (cluster ID is 4-1). All probes included in these functional Classes and cluster 4-1 were cDNA clones of 20S proteasome subunit genes. Furthermore, most cDNA clones of 20S proteasome genes were included in cluster 6-1 or 4-1 (Figure 8, 9), which suggested that genes of 20S proteasome subunits have similar expression profiles and receive similar control. Exceptionally, the expression profiles of PAD1 (At3g51260), PBF1 (At3g60820), and PBG1 (At1g56450), which encoded for the α4, β6, and β7 subunits, respectively showed expression profiles different from the majority. It has been reported that the 20S proteasome α4 subunit of *A. thaliana *complements the 20S proteasome α4 subunit and α3 subunit of yeast [27], which suggests a complementary working relationship of α4 subunit with other subunits. Therefore, PAD1 possibly receives a different control, although most 20S proteasome subunit genes receive a similar control. However, further investigation is necessary to determine if the expression patterns of PAD1, PBF1, and PBG1 are different from the expression patterns of other 20S proteasome genes.

In this study, BiKLi and GSCope3 detected the novel information on 20S proteasome subunit genes showing similar expression profiles under drought stress.

### Comparison between gene expression of *A. thaliana *and *Z. elegans*

A comparison between different microarray data can be facilitated by using GSCope3. In addition, if the genes between different organisms relate, a comparison of the microarray data between different organisms becomes possible.

Demura et al. [28] tried to compare gene expressions between *Z. elegans *and *Poplar tremula *× *P. tremuloides *clone T89, where the *A. thaliana *gene corresponded to the *Z. elegans *gene. Demura et al. [28] also examined the change in gene expression in the transdifferentiation of mesophyll cells into xylem cells in *Z. elegans*.

This experiment is quite difficult to perform on *A. thaliana*. In order to investigate the change in gene expression in the transdifferentiation of mesophyll cells into xylem cells in *A. thaliana*, comparison between *Z. elegans *and *A. thaliana *is practical. In this study, the comparison was made in order to confirm the relationship between gene expressions in the transdifferentiation of mesophyll cells into xylem cells and the response against drought stress. Furthermore, it presents an example of a feasible comparison of the microarray experiment using different organisms.

The gene expression profiles were divided into 24 subgroups. We compared the classification of the genes by BL-SOM using the microarray data of Seki et al. [18] with the genes based on the microarray data of Demura et al. [28].

Additional File 6 shows a list of the significant correlations between the "functional Class" and the clusters formed by BL-SOM of the microarray probes' expression profile during drought conditions. A significant correlation was discovered between the gene expression profiles with decreased transcription two hours after the drought condition started (cluster ID is 1-2) and the subclass with up-regulated genes during stage one (Additional File 6). Stage one corresponds to the functional dedifferentiation process during which mesophyll cells lose their photosynthetic capacity and acquire a new multidifferentiation potency [27]. The genes included in the category are 5-methyltetrahydropteroyltriglutamate-homocysteine S-methyltransferase (At5g17920), hydroxymethyltransferase (At4g13930), and putative WD-40 repeat auxin-dependent protein ARCA (At1g48630). These genes may be key genes between drought stress response and transdifferentiation. Unfortunately, there are no novel discoveries because the *A. thaliana *gene and *Z. elegans *gene exhibit little association. If more *A. thaliana *genes associate to *Z. elegans *genes, then likeness and difference of gene expression mechanisms between transdifferentiation and stress response could be analyzed in detail.

## Conclusion

It is proven in this study, through microarray experiment, that the relationship between gene expression and biological phenomenon is easily discovered by using the BiKLi and GSCope3. In order to speculate any relationship between a specific gene's expression pattern and the specific biological phenomenon, some researchers compare an individual gene with the biological phenomenon. However, this kind of comparison is not enough because one biological phenomenon is almost always caused by a combination of two or more genes.

In the analysis using the BiKLi and GSCope3, we use cross-tabulation in conducting statistical process. We divide the genes related to a specific biological phenomenon and other genes, and genes that have a specific expression profile and other genes. Thus, we can statistically detect the significance of a specific biological phenomenon and genes which have specific expression profiles. We therefore conclude that this analysis is more accurate.

Although we have re-analyzed the microarray data used in other analysis, we hope that when the new microarray data is assessed, many new findings may be revealed. As we look to a wider analysis in the future, we plan to enhance the content of the BiKLi.

Additionally, we are developing Genome-Phenome Superhighway (GPS) [29] and TraitMap system [30] which can use the BiKLi. The GPS has a biological network data and offers an environment where gene information can be retrieved. This network data contains biomolecular relationship data which is generated from co-citation frequencies of gene names and from arbitrary key-concept terms in literature. We plan to add the data in the BiKLi to the GPS in order to strengthen the GPS and TraitMap system in the future.

## Methods

### Construction of the biological knowledge library

#### KEGG type metabolic pathway dataset

We made a KEGG type metabolic pathway Omic Space Markup Language (OSML) file using Knowledge editor [31], which is referred to the KEGG database (Release 26.0) [32] and LIGAND database (Release 29.0) [33]. The metabolic pathway data, especially the enzyme reactions, was constructed according to Michaels's expression [34]. The KEGG type metabolic pathway OSML data was constructed by "functional Class" ("functional Class" is one of the elements in the OSML) [10], which illustrated each sub-pathway wherein the metabolic pathway is constructed by various sub-pathways. The enzyme and the metabolic compound were described as the "omicElement" ("omicElement" is one of the elements in OSML) [10] in the "functional Class". The enzyme code (EC) was used to show the enzyme in the metabolic pathway. For the purpose of connecting a gene and an enzyme, the use of an appendix file [35], wherein a gene corresponds to an enzyme in GSCope3, was acceptable. Therefore, we made an appendix file where the RAFL clone's code corresponded to EC in order to analyze *A. thaliana *microarray data. We made this appendix file by following this procedure: The information regarding the correspondence between the open reading frame (orf) of *A. thaliana*, which was shown as *Arabidopsis *Genome Initiative (AGI) code [36], and EC were acquired from the LIGAND database and AraCyc (version 2.1) [37]. Then, the information regarding correspondence between the RAFL clone and AGI code was acquired from the RIKEN *Arabidopsis *Genome Encyclopaedia (RARGE) [38]. An appendix file that showed the microarray probe corresponding to the enzyme was completed using this information. Moreover, we prepared an appendix file for the association between the AGI code and EC. The KEGG type metabolic pathway data set was composed of a KEGG type metabolic pathways OSML file (KEGG_pathway.xml), an appendix file which showed the RAFL clone's code referring to EC (RAFL_EC.apdx), and an appendix file which illustrated the AGI code corresponding to EC (AGI_EC.apdx) (Figure 1).

If revisions of the data are necessary in BiKLi, users can rewrite the OSML data of BiKLi. Moreover, we are planning to improve GSCope3 so that data of BiKLi can be modified by using Graphical User Interface (GUI).

#### AraCyc type metabolic pathway dataset

We made an AraCyc type metabolic pathway OSML file which referred to the AraCyc data (version 2.1) in the TAIR database [37]. The enzymes and *A. thaliana *genes of each metabolic pathway were merely enumerated in the AraCyc data. The AraCyc type metabolic pathway OSML file was constructed by some "functional Classes" as the KEGG type metabolic pathway OSML file which demonstrated each sub-pathway. Therefore, the AraCyc type metabolic pathway OSML file was made so that the enzyme would be included in the "functional Class" as "omicElement". The AraCyc type metabolic pathway dataset was composed of an AraCyc type metabolic pathways OSML file (AraCyc_pathway.xml), an appendix file showing the RAFL clone's code matched to EC (RAFL_EC.apdx), and an appendix file that illustrating the AGI code corresponding to EC (AGI_EC.apdx) (Figure 1).

#### Directed acyclic graphs of GO terms dataset

Directed acyclic graphs of GO terms OSML file were made by referring to the flat files of gene ontology (GO), which were made by the GO consortium [9]. The graphs were composed of terms from molecular function ontology, biological process ontology, and cellular component ontology. Moreover, compact directed acyclic graphs of GO terms ("GO slim") made by some research laboratories were transferred to directed acyclic graphs of GO terms data. We made an appendix file wherein the RAFL clone corresponded to a GO term by following these procedures: Information regarding the correspondence between AGI code and GO term were acquired from the gene ontology annotations data in the TAIR [39]. An appendix file, which showed the microarray probe consistent with the GO term, was made using this information. Additionally, we prepared an appendix file to show the association between the AGI code and GO term. The directed acyclic graphs of GO terms dataset was composed of directed acyclic graphs of GO terms OSML files (Component.xml, Function.xml, Process.xml, Goslim_plant_2003.xml, etc.), an appendix file which presented the RAFL clone's code relating to the GO term (RAFL_GO.apdx), and an appendix file which showed the AGI code relating to the GO term (AGI_GO.apdx) (Figure 1).

#### Gene position on A. thaliana genome dataset

The information about the gene position on the *A. thaliana *genome was acquired from the MIPS *Arabidopsis thaliana *database (MAtDB) (version 110204) [40]. The gene position OSML file has five "datasets" (a "dataset" is one of the elements in OSML) [10] corresponding to each of the five chromosomes. Each "dataset" was divided by some "functional Classes" corresponding to the area of 3,000,000 base pair (bp), 300,000 bp, and 30,000 bp. The "functional Class" demonstrated by the narrow area was described by the succession implication "part of" included in the "functional Class" pertaining to the large area. The gene position appendix file was made by describing the relation between the gene and the 30,000 bp area on the genome. The gene's position on the *A. thaliana *genome dataset was composed of an *Arabidopsis *genome OSML file (Arabidopsis_Genome.xml), an appendix file showing that the RAFL clone's code pointed to the gene position on the genome divided by 30,000 bp (RAFL_position.apdx) and an appendix file presenting the AGI code corresponding to the gene position on the genome divided by 30,000 bp (AGI_position.apdx) (Figure 1).

#### Yeast PPI dataset

Information about the *Saccharomyces cerevisiae *(yeast) protein-protein interaction (PPI) data was acquired from the paper "A comprehensive two-hybrid analysis to explore the yeast protein interactome" [41]. The PPI information in the yeast PPI OSML was described as follows: It was assumed that the protein name was synonymous with the gene name, so the protein name was shown by the systematic orf name, e.g. YLR163C etc. One protein referred to one "functional Class" and was described as an "omicElement" in that "functional Class". Other proteins that interact with the protein were described as other "omicElements", which showed whether they are members in the "functional Class". The information regarding the association between a gene of yeast *and A. thaliana *was acquired from the Homologene database at the National Center for Biotechnology Information (NCBI) [42]. The yeast gene was described with gene symbols offered in the Homologene database. Therefore, the *Saccharomyces *genome database (SGD) [43] was used to connect the gene symbol and the systematic orf name. The yeast PPI dataset was composed of a yeast PPI OSML file (yeast_PPI.xml), an appendix file which illustrated the RAFL clone's code matched to the yeast systematic orf name (RAFL_yeast_gene.apdx) and an appendix file which presented the AGI code corresponds matched to the yeast systematic orf name (AGI_yeast_gene.apdx) (Figure 1).

#### Worm PPI dataset

The *Caenorhabditis elegans *(worm) PPI data was acquired from the paper "A map of the interactome network of the metazoan *C. elegans*" [44]. The PPI information was placed in a worm PPI OSML by the following procedure: It was assumed that the protein name was synonymous with the gene name, so the protein was shown by the cosmid based gene names, e.g. F07D10.1, etc. One protein corresponded to one "functional Class". The other proteins that interact with the protein were described as an "omicElement", which demonstrated whether they are members in the "functional Class". The information regarding the correspondence between a worm gene *and A. thaliana *was acquired from the Homologene database. The worm gene was described with gene symbols in data presented by the Homologene. Therefore, the WormBase [45] explained the correspondence between the gene symbol and the cosmid based gene name. The worm PPI dataset was composed of a worm PPI OSML file (Worm_PPI.xml), an appendix file matching the RAFL clone's code with the worm cosmid based gene name (RAFL_worm_gene.apdx), and an appendix file about the AGI code matched to worm cosmid based gene name (AGI_worm_gene.apdx) (Figure 1).

#### A. thaliana transcription dataset

**Figure 10:**
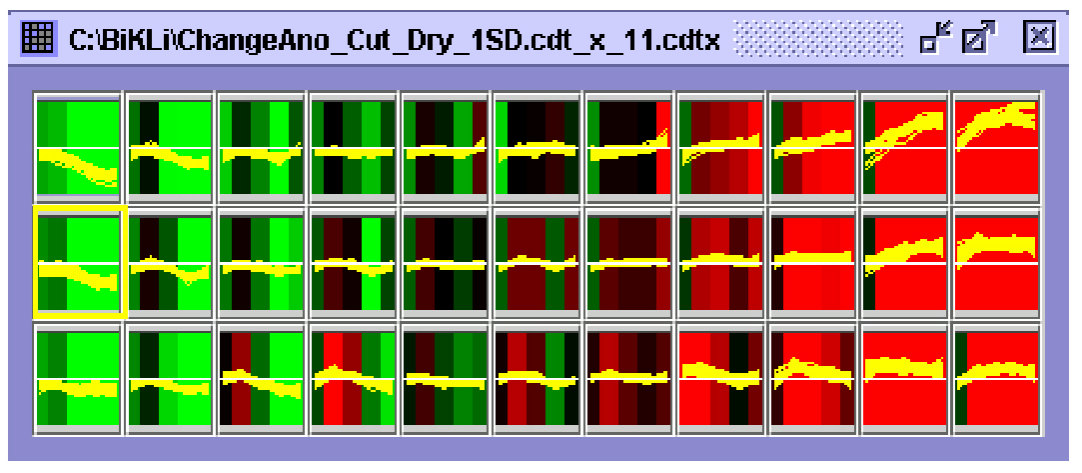
**The appearance of BL-SOM clustering by GraphView**. Each cluster is shown in the figure. The first principal ingredient ID of the BL-SOM cluster is shown as 0 to 10 from the right. The second principal ingredient is shown as 0 to 3 from the top. The cluster ID is described as the first principal ingredient ID- the second principal ingredient ID. The cluster framed in yellow becomes ID 0-1. The colour of the classified clusters indicates the mean values of the probe data included in the cluster in each experiment. Each yellow line in the cluster is a graph of the expression profile of all the probes included in the cluster.

An *A. thaliana *transcription OSML file was made according to the following procedure: The *A. thaliana *microarray data under drought stress was clustered by batch-learning self-organizing maps (BL-SOM) [19] (Figure 10). The first principal ingredient was set at eleven. An OSML file was made so that each of the 33 clusters corresponded to each "functional Class" and RAFL cDNA clones included in the same cluster were included in the same "functional Class" as the "omicElement". The genes with similar expression profiles in drought stress and in other conditions became possible by using this dataset. An *A. thaliana *transcription dataset was composed of an *A. thaliana *transcription during drought stress OSML file (Arabidopsis_drought_stress.xml) (Figure 1). In the future, we would intend to make more OSML files from other microarray data to enhance this dataset.

#### Z. elegans transcription dataset

Demura et al. [28] reported on the clustering data of a *Zinnia elegans *gene during transdifferentiation of mesophyll cells into xylem cells and the correspondence between data of *Z. elegans *genes and *A. thaliana *genes (AGI code). The *Z. elegans *genes were divided into 24 subgroups in the clustering. An OSML file was made so that these 24 subgroups could be set to "functional Class" and the AGI code corresponding to the *Z. elegans *gene included in this subgroup was set to "omicElement", which was contained in this "functional Class". The *Z. elegans *transcription dataset was composed of a *Z. elegans *transcription during transdifferentiation OSML file (Zinnia_transdifferentiation.xml) and an appendix file of the RAFL clone's code corresponding to AGI code (RAFL_AGI.apdx) (Figure 1).

### Microarray data

We used the microarray data generated by Seki et al. [18]. In the investigation that analyzed gene expression profiles, mRNAs were isolated from *A. thaliana*, which were subjected to dehydration, cold, or high-salinity stress treatments as "experimental" group and no treatments for the "reference". We used the data from the dehydration stress treatments experiment. They utilized a Stanford type microarray, which consists of 7000 cDNA sequences representing RIKEN *A. thaliana *full-length (RAFL) cDNA clones [46] isolated from full-length cDNA libraries [47]. In this paper, the microarray data that was obtained was processed by the following methods: Background fluorescence was calculated on the basis of the fluorescence signal of negative control genes. Spots showing a signal value under the value of the fluorescence signal of negative control genes (+ standard deviation (SD)) in both the Cy3 ("reference" sample) and Cy5 ("experimental" sample) channels were not considered for analysis. Then each spot value of Cy5/Cy3 was calculated. To normalize the hybridization signals generated from different samples, external controls were used. Lastly, the log_2 _values of each spot and mean values of each probe were calculated.

### Direction of GSCope3

A GSCope3 program produced by Toyoda et al. is used to analyze microarray data [11]. GSCope3 is programmed in JAVA, therefore, it can be used in multiple platforms including Windows and Mac OS. We open this program to the public through a website [11] and academic users are able to freely download and use this program. GSCope3 can BL-SOM cluster the microarray data which is in cdt format [48]. At present, the manual of GSCope3 is being made, however methods of using GSCope3 and microarray data analysis are described in the online tutorial of GSCope3 [49]. Briefly, one OSML file in the BiKLi dataset is opened by GSCope3. Each sub-network in the dataset is expressed as a "functional Class". For example, each sub-pathway is categorized by "functional Class" in the metabolic pathway dataset and each GO term is classified by "functional Class" in the directed acyclic graphs of the GO terms dataset. Then, a suitable appendix file is opened by GSCope3. Each microarray probe is displayed by shape corresponding to each gene, protein, GO term, or gene position on the dataset by using an appendix file [35], in which the microarray probes are written and compared. Afterwards, if the microarray data in cdt [48] format or cdtx format [35] is opened by GSCope3, the association between the microarray data and microarray probes on the dataset (OSML and appendix file) is analyzed and the data value is displayed corresponding to the probe (Figure 2).

### Functional class (network) ranking by GSCope3

After the probes are selected, the correlation of the probes and the "functional Class" can be examined in GSCope3. The ranking of the number of probes, which is analyzed by cross-tabulation using Fisher's test and included in the "functional Class", is classified into four groups: objective, not objective, selected, and not selected.

The "functional Class", which includes the selected probes, is ranked by GSCope3. Moreover, the degree of correlation between the selected probes and "functional Class" is revealed (Additional Files 1, 2, 3, 4, 5, 6). In this paper, we have examined the correlation between the probes of each cluster after BL-SOM clustering and each "functional Class".

### Batch-learning self-organizing maps (BL-SOM) clustering by GSCope3 and the procedure for deciding the best classification value of BL-SOM clustering

BL-SOM is an improved method of the original SOM [50] with regard to the fact that the initial weight vectors are set by principal component analysis. The learning process is designed to be independent of the order of input vectors, hence, the result is reproducible [18]. So, it is applicable to many fields of bioinformatics [51,52].

The figure 10 shows the appearance of BL-SOM clustering of the microarray data. The first principal ingredient ID of BL-SOM cluster is shown as a number from 0 to 10 from the left horizontally. The second principal ingredient ID is the number from 0 to 3 vertically from the upper part. The cluster ID is described in the order of first principal ingredient ID then -second principal ingredient ID. For example, the section first from the left and second from the top is shown as cluster ID 0-1 (Figure 10).

To decide the best classification value of BL-SOM clustering, the following procedure was done: The microarray data was clustered by GSCope3 using the number of the first principal ingredient, ranging from three to twenty. The mean value of each cluster's average radius, which is the squared distances of the points from the centre of the cluster [53], and the explained variability [54] were calculated and plotted (Additional File 7). We decided that the eleven first principal ingredients were the best classification values because the mean value of each cluster's average radius was comparatively small and the explained variability was comparatively large.

## Competing interests

The author(s) declare that they have no competing interests.

## Authors' contributions

YH created and supervised the Biological Knowledge Library (BiKLi). YH analyzed the microarray data by using BiKli and GSCope3. YH is the overall author of this paper. MS, and KS provided the microarray data of *Arabidopsis thaliana*. They also reviewed and revised this paper. YM, and KH participated in the conception of Omic Space Markup Language (OSML) and designed a part of BiKLi, especially the KEGG type metabolic pathway. NH developed websites for the tutorial of GSCope3 and GSCope3. NO participated in the creation of OSML and developed GSCope3. TS, MS, KA, and KI provided the microarray data of *A. thaliana *and modified the data. KL created a part of BiKLi, especially the Directed acyclic graphs of GO terms. SK provided the program of Batch-leaning self-organizing maps (BL-SOM) clustering. TD provided microarray data of *Zinnia elegans *and the correlation to *Zinnia elegans *and *A. thaliana *genes. AK managed the writing of the manuscript. TT is the chief of the OSML creation. In addition, he participated in the design and coordination of the study, supervised in writing the manuscript, and wrote a part of the abstract and background.

## Supplementary Material

Additional File 1 **Supplementary Table 1**. Ranking result of significant correlations between the "functional Class" of the KEGG type metabolic pathway and clusters formed by BL-SOM of the expression profile of microarray probes under drought conditions by using GSCope3 Click here for file

Additional File 2 **Supplementary Table 2**. Ranking result of significant correlations between the "functional Class" of the AraCyc type metabolic pathway and the clusters formed by BL-SOM of the microarray probes of expression profile under drought conditions Click here for file

Additional File 3 **Supplementary Table 3**. Ranking result of significant correlations between the "functional Class" of the Directed acyclic graphs of GO terms and the clusters formed by BL-SOM of the microarray probes of expression profile under drought conditions Click here for file

Additional File 4 **Supplementary Table 4**. Ranking result of significant correlations between the "functional Class" of the Gene position on genome and the clusters formed by BL-SOM of the microarray probes of expression profile under drought conditions Click here for file

Additional File 5 **Supplementary Table 5**. Ranking result of significant correlations between the "functional Class" of the "Protein-protein interaction and *Arabidopsis thaliana *gene expression" and the clusters formed by BL-SOM of the microarray probes of expression profile under drought conditions Click here for file

Additional File 6 **Supplementary Table 6**. Ranking result of significant correlations between the "functional Class" of the "Comparison between gene expression of *Arabidopsis thaliana *and *Zinia elegans*" and the clusters formed by BL-SOM of the microarray probes of expression profile under drought conditions Click here for file

Additional File 7 **The relationship between the number of the first principal ingredient in the BL-SOM clusters and the index of clustering**. The first principal ingredient = 3 to 20 (clustering 3 to 120) of BL-SOM clustering of the microarray data under drought stress and the index of clustering (the "mean value of each cluster's average radius" and "explained variability") is plotted. It is used for analysis because the cluster with X = 11, clustering = 33, separated enough. Click here for file
