# Supplementary material for: A flexible representation of omic knowledge for thorough analysis of microarray data
Source: Plant Methods. 2006 Mar 2;2:5. doi: 10.1186/1746-4811-2-5 (PMC1421397; doi:10.1186/1746-4811-2-5)
Supplement: Additional File 1 — Supplementary Table 1. Ranking result of significant correlations between the "functional Class" of the KEGG type metabolic pathway and clusters formed by BL-SOM of the expression profile of microarray probes under drought conditions by using GSCope3 [file 1746-4811-2-5-S1.HTML]

|  |  |  |  |  |  |  |  |  |  |  |  |
| --- | --- | --- | --- | --- | --- | --- | --- | --- | --- | --- | --- |
| Date: | | 2005/06/24 | | | | | | | | | |
| Method: | | Fisher test | | | | | | | | | |
| Cut off P-value: | | 0.05 | | | | | | | | | |
| Target dataset(s): | | Carbohydrate Metabolism  Energy Metabolism  Biodegradation of Xenobiotics  Lipid Metabolism  Metabolism of Other Amino Acids  Amino Acid Metabolism  Metabolism of Cofactors and Vitamins  Glycan Biosynthesis and Metabolism  Biosynthesis of Secondary Metabolites  Biosynthesis of Polyketides and Nonribosomal Peptides  Nucleotide Metabolism | | | | | | | | | |
| Query dataset(s): | | SOM Cluster | | | | | | | | | |
  | | | | | | | | | | | || Alkaloid biosynthesis II | | |  |  | A | B | C | D | P | P' | N |
|  | Cluster:6-2 | |  |  | 3 | 173 | 4 | 4483 | 0.0016539665 | 0.0082698325 | 5 |
|  |  | RAFL04-13-B02 | At2g37040 / phenylalanine ammonia lyase (PAL1) | |  |  |  |  |  | | --- | --- | --- | --- | --- | |  |  |  |  |  | | EC:4.3.1.5 | | | | | | |
|  |  | RAFL09-11-L22 | At3g53260 / phenylalanine ammonia-lyase (PAL2) | |  |  |  |  |  | | --- | --- | --- | --- | --- | |  |  |  |  |  | | EC:4.3.1.5 | | | | | | |
|  |  | RAFL04-16-D08 | At3g53260 / phenylalanine ammonia-lyase (PAL2) | |  |  |  |  |  | | --- | --- | --- | --- | --- | |  |  |  |  |  | | EC:4.3.1.5 | | | | | | |
| Inositol phosphate metabolism | | |  |  | A | B | C | D | P | P' | N |
|  | Cluster:2-2 | |  |  | 2 | 51 | 5 | 4605 | 0.0025667707 | 0.015400624 | 6 |
|  |  | RAFL09-15-K07 | At4g39800 / myo-inositol-1-phosphate synthase | |  |  |  |  |  | | --- | --- | --- | --- | --- | |  |  |  |  |  | | EC:5.5.1.4 | | | | | | |
|  |  | RAFL09-14-L01 | At4g39800 / myo-inositol-1-phosphate synthase | |  |  |  |  |  | | --- | --- | --- | --- | --- | |  |  |  |  |  | | EC:5.5.1.4 | | | | | | |
| Cyanoamino acid metabolism | | |  |  | A | B | C | D | P | P' | N |
|  | Cluster:8-2 | |  |  | 2 | 60 | 13 | 4588 | 0.016341258 | 0.16341259 | 10 |
|  |  | RAFL02-08-C20 | At4g08790 / nitrilase 1 like protein | |  |  |  |  |  | | --- | --- | --- | --- | --- | |  |  |  |  |  | | EC:4.2.1.84 ,EC:3.5.5.1 | | | | | | |
|  |  | RAFL08-18-I10 | At3g47000 / glycosyl hydrolase family 3 | |  |  |  |  |  | | --- | --- | --- | --- | --- | |  |  |  |  |  | | EC:3.2.1.21 | | | | | | |
|  | Cluster:0-1 | |  |  | 2 | 104 | 13 | 4544 | 0.04433326 | 0.4433326 | 10 |
|  |  | RAFL05-04-O06 | At4g37930 / glycine hydroxymethyltransferase like protein | |  |  |  |  |  | | --- | --- | --- | --- | --- | |  |  |  |  |  | | EC:2.1.2.1 | | | | | | |
|  |  | RAFL09-15-K15 | At3g18080 / glycosyl hydrolase family 1 | |  |  |  |  |  | | --- | --- | --- | --- | --- | |  |  |  |  |  | | EC:3.2.1.21 | | | | | | |
|  | Cluster:8-0 | |  |  | 2 | 107 | 13 | 4541 | 0.04663169 | 0.4663169 | 10 |
|  |  | RAFL06-13-E03 | At3g44300 / nitrilase 2 | |  |  |  |  |  | | --- | --- | --- | --- | --- | |  |  |  |  |  | | EC:3.5.5.1 | | | | | | |
|  |  | RAFL08-10-H06 | At3g44300 / nitrilase 2 | |  |  |  |  |  | | --- | --- | --- | --- | --- | |  |  |  |  |  | | EC:3.5.5.1 | | | | | | |
| Purine metabolism | | |  |  | A | B | C | D | P | P' | N |
|  | Cluster:2-2 | |  |  | 3 | 50 | 31 | 4579 | 0.0064710197 | 0.11000734 | 17 |
|  |  | RAFL04-20-F22 | At5g47840 / expressed protein | |  |  |  |  |  | | --- | --- | --- | --- | --- | |  |  |  |  |  | | EC:2.7.4.3 | | | | | | |
|  |  | RAFL07-10-P11 | At3g22890 / ATP sulfurylase -related | |  |  |  |  |  | | --- | --- | --- | --- | --- | |  |  |  |  |  | | EC:2.7.7.4 | | | | | | |
|  |  | RAFL07-12-E10 | At3g22890 / ATP sulfurylase -related | |  |  |  |  |  | | --- | --- | --- | --- | --- | |  |  |  |  |  | | EC:2.7.7.4 | | | | | | |
|  | Cluster:2-1 | |  |  | 5 | 239 | 29 | 4390 | 0.03021746 | 0.51369685 | 17 |
|  |  | RAFL04-18-P17 | At5g35170 / adenylate kinase -related protein | |  |  |  |  |  | | --- | --- | --- | --- | --- | |  |  |  |  |  | | EC:2.7.4.3 | | | | | | |
|  |  | RAFL04-12-O11 | At3g27740 / carbamoyl-phosphate synthase (glutamine-hydrolyzing) (glutamine-dependent carbamoyl-phosphate synthase) small subunit | |  |  |  |  |  | | --- | --- | --- | --- | --- | |  |  |  |  |  | | EC:6.3.5.2 | | | | | | |
|  |  | RAFL04-13-M20 | At4g11010 / nucleoside diphosphate kinase 3 (ndpk3) | |  |  |  |  |  | | --- | --- | --- | --- | --- | |  |  |  |  |  | | EC:2.7.4.6 | | | | | | |
|  |  | RAFL04-17-H07 | At3g57610 / adenylosuccinate synthetase | |  |  |  |  |  | | --- | --- | --- | --- | --- | |  |  |  |  |  | | EC:6.3.4.4 | | | | | | |
|  |  | RAFL06-13-H08 | At1g32440 / pyruvate kinase, putative | |  |  |  |  |  | | --- | --- | --- | --- | --- | |  |  |  |  |  | | EC:2.7.1.40 | | | | | | |
| Pyruvate metabolism | | |  |  | A | B | C | D | P | P' | N |
|  | Cluster:9-0 | |  |  | 4 | 28 | 51 | 4580 | 4.876191E-4 | 0.008777143 | 18 |
|  |  | RAFL04-09-D07 | At1g54100 / aldehyde dehydrogenase, putative (ALDH) | |  |  |  |  |  | | --- | --- | --- | --- | --- | |  |  |  |  |  | | EC:1.2.1.3 | | | | | | |
|  |  | RAFL05-21-E06 | At1g54100 / aldehyde dehydrogenase, putative (ALDH) | |  |  |  |  |  | | --- | --- | --- | --- | --- | |  |  |  |  |  | | EC:1.2.1.3 | | | | | | |
|  |  | RAFL08-15-L09 | At1g54100 / aldehyde dehydrogenase, putative (ALDH) | |  |  |  |  |  | | --- | --- | --- | --- | --- | |  |  |  |  |  | | EC:1.2.1.3 | | | | | | |
|  |  | RAFL08-09-C23 | At1g54100 / aldehyde dehydrogenase, putative (ALDH) | |  |  |  |  |  | | --- | --- | --- | --- | --- | |  |  |  |  |  | | EC:1.2.1.3 | | | | | | |
|  | Cluster:4-1 | |  |  | 9 | 301 | 46 | 4307 | 0.009480812 | 0.17065461 | 18 |
|  |  | RAFL09-11-F09 | At1g30120 / pyruvate dehydrogenase E1 beta subunit -related | |  |  |  |  |  | | --- | --- | --- | --- | --- | |  |  |  |  |  | | EC:1.2.4.1 | | | | | | |
|  |  | RAFL09-12-A19 | At3g13930 / acetyltransferase -related | |  |  |  |  |  | | --- | --- | --- | --- | --- | |  |  |  |  |  | | EC:2.3.1.12 | | | | | | |
|  |  | RAFL05-17-D15 | At1g11840 / glyoxalase I, putative (lactoylglutathione lyase) | |  |  |  |  |  | | --- | --- | --- | --- | --- | |  |  |  |  |  | | EC:4.4.1.5 | | | | | | |
|  |  | RAFL06-08-D06 | At2g34590 / pyruvate dehydrogenase E1 beta subunit -related | |  |  |  |  |  | | --- | --- | --- | --- | --- | |  |  |  |  |  | | EC:1.2.4.1 | | | | | | |
|  |  | RAFL09-16-O16 | At1g01090 / pyruvate dehydrogenase E1 alpha subunit | |  |  |  |  |  | | --- | --- | --- | --- | --- | |  |  |  |  |  | | EC:1.2.4.1 | | | | | | |
|  |  | RAFL11-03-L09 | At5g56350 / pyruvate kinase, putative | |  |  |  |  |  | | --- | --- | --- | --- | --- | |  |  |  |  |  | | EC:2.7.1.40 | | | | | | |
|  |  | RAFL11-03-C02 | At1g48030 / dihydrolipoamide dehydrogenase, mitochondrial (lipoamide dehydrogenase) (mtlpd1) | |  |  |  |  |  | | --- | --- | --- | --- | --- | |  |  |  |  |  | | EC:1.8.1.4 | | | | | | |
|  |  | RAFL07-16-E16 | At3g13930 / acetyltransferase -related | |  |  |  |  |  | | --- | --- | --- | --- | --- | |  |  |  |  |  | | EC:2.3.1.12 | | | | | | |
|  |  | RAFL05-18-D04 | At5g36880 / acetyl-CoA synthetase (acetate-CoA ligase), putative | |  |  |  |  |  | | --- | --- | --- | --- | --- | |  |  |  |  |  | | EC:6.2.1.1 | | | | | | |
|  | Cluster:3-0 | |  |  | 7 | 226 | 48 | 4382 | 0.018589124 | 0.33460423 | 18 |
|  |  | RAFL05-21-P13 | At2g42600 / phosphoenolpyruvate carboxylase | |  |  |  |  |  | | --- | --- | --- | --- | --- | |  |  |  |  |  | | EC:4.1.1.49 ,EC:4.1.1.32 ,EC:4.1.1.31 | | | | | | |
|  |  | RAFL06-07-J21 | At1g04410 / malate dehydrogenase, cytosolic, putative | |  |  |  |  |  | | --- | --- | --- | --- | --- | |  |  |  |  |  | | EC:1.1.1.37 ,EC:1.1.99.16 | | | | | | |
|  |  | RAFL06-08-D17 | At5g43940 / alcohol dehydrogenase class III (glutathione-dependent formaldehyde dehydrogenase) (GSH-FDH) (ADHIII) | |  |  |  |  |  | | --- | --- | --- | --- | --- | |  |  |  |  |  | | EC:1.2.1.1 | | | | | | |
|  |  | RAFL07-17-M04 | At1g04410 / malate dehydrogenase, cytosolic, putative | |  |  |  |  |  | | --- | --- | --- | --- | --- | |  |  |  |  |  | | EC:1.1.1.37 ,EC:1.1.99.16 | | | | | | |
|  |  | RAFL07-10-F16 | At3g13930 / acetyltransferase -related | |  |  |  |  |  | | --- | --- | --- | --- | --- | |  |  |  |  |  | | EC:2.3.1.12 | | | | | | |
|  |  | RAFL09-09-M02 | At3g47520 / malate dehydrogenase (NAD), chloroplast, putative | |  |  |  |  |  | | --- | --- | --- | --- | --- | |  |  |  |  |  | | EC:1.1.1.37 ,EC:1.1.99.16 | | | | | | |
|  |  | RAFL09-17-A09 | At5g35360 / acetyl-CoA carboxylase | |  |  |  |  |  | | --- | --- | --- | --- | --- | |  |  |  |  |  | | EC:6.4.1.2 | | | | | | |
| Valine, leucine and isoleucine degradation | | |  |  | A | B | C | D | P | P' | N |
|  | Cluster:9-0 | |  |  | 4 | 28 | 10 | 4621 | 1.7435473E-6 | 1.5691925E-5 | 9 |
|  |  | RAFL04-09-D07 | At1g54100 / aldehyde dehydrogenase, putative (ALDH) | |  |  |  |  |  | | --- | --- | --- | --- | --- | |  |  |  |  |  | | EC:1.2.1.3 | | | | | | |
|  |  | RAFL05-21-E06 | At1g54100 / aldehyde dehydrogenase, putative (ALDH) | |  |  |  |  |  | | --- | --- | --- | --- | --- | |  |  |  |  |  | | EC:1.2.1.3 | | | | | | |
|  |  | RAFL08-15-L09 | At1g54100 / aldehyde dehydrogenase, putative (ALDH) | |  |  |  |  |  | | --- | --- | --- | --- | --- | |  |  |  |  |  | | EC:1.2.1.3 | | | | | | |
|  |  | RAFL08-09-C23 | At1g54100 / aldehyde dehydrogenase, putative (ALDH) | |  |  |  |  |  | | --- | --- | --- | --- | --- | |  |  |  |  |  | | EC:1.2.1.3 | | | | | | |
| Fatty acid biosynthesis (path 2) | | |  |  | A | B | C | D | P | P' | N |
|  | Cluster:6-1 | |  |  | 2 | 313 | 2 | 4346 | 0.024916494 | 0.074749485 | 3 |
|  |  | RAFL07-15-A09 | At3g06860 / fatty acid multifunctional protein (AtMFP2) | |  |  |  |  |  | | --- | --- | --- | --- | --- | |  |  |  |  |  | | EC:4.2.1.17 | | | | | | |
|  |  | RAFL11-10-D22 | At5g43280 / enoyl-CoA hydratase/isomerase family | |  |  |  |  |  | | --- | --- | --- | --- | --- | |  |  |  |  |  | | EC:4.2.1.17 | | | | | | |
| Glycolysis / Gluconeogenesis | | |  |  | A | B | C | D | P | P' | N |
|  | Cluster:0-1 | |  |  | 16 | 90 | 59 | 4498 | 4.556285E-12 | 9.568198E-11 | 21 |
|  |  | RAFL07-07-I23 | At1g56190 / phosphoglycerate kinase -related | |  |  |  |  |  | | --- | --- | --- | --- | --- | |  |  |  |  |  | | EC:2.7.2.3 | | | | | | |
|  |  | RAFL07-18-C20 | At2g21330 / fructose-bisphosphate aldolase, putative | |  |  |  |  |  | | --- | --- | --- | --- | --- | |  |  |  |  |  | | EC:4.1.2.13 | | | | | | |
|  |  | RAFL04-19-O21 | At4g38970 / fructose-bisphosphate aldolase, putative | |  |  |  |  |  | | --- | --- | --- | --- | --- | |  |  |  |  |  | | EC:4.1.2.13 | | | | | | |
|  |  | RAFL09-17-N23 | At2g21330 / fructose-bisphosphate aldolase, putative | |  |  |  |  |  | | --- | --- | --- | --- | --- | |  |  |  |  |  | | EC:4.1.2.13 | | | | | | |
|  |  | RAFL07-14-L16 | At3g12780 / phosphoglycerate kinase -related | |  |  |  |  |  | | --- | --- | --- | --- | --- | |  |  |  |  |  | | EC:2.7.2.3 | | | | | | |
|  |  | RAFL09-15-L04 | At3g12780 / phosphoglycerate kinase -related | |  |  |  |  |  | | --- | --- | --- | --- | --- | |  |  |  |  |  | | EC:2.7.2.3 | | | | | | |
|  |  | RAFL05-07-J06 | At1g42970 / glyceraldehyde-3-phosphate dehydrogenase | |  |  |  |  |  | | --- | --- | --- | --- | --- | |  |  |  |  |  | | EC:1.2.1.12 | | | | | | |
|  |  | RAFL04-09-D24 | At1g42970 / glyceraldehyde-3-phosphate dehydrogenase | |  |  |  |  |  | | --- | --- | --- | --- | --- | |  |  |  |  |  | | EC:1.2.1.12 | | | | | | |
|  |  | RAFL09-18-L22 | At3g12780 / phosphoglycerate kinase -related | |  |  |  |  |  | | --- | --- | --- | --- | --- | |  |  |  |  |  | | EC:2.7.2.3 | | | | | | |
|  |  | RAFL07-12-M09 | At2g21330 / fructose-bisphosphate aldolase, putative | |  |  |  |  |  | | --- | --- | --- | --- | --- | |  |  |  |  |  | | EC:4.1.2.13 | | | | | | |
|  |  | RAFL08-18-C10 | At2g21330 / fructose-bisphosphate aldolase, putative | |  |  |  |  |  | | --- | --- | --- | --- | --- | |  |  |  |  |  | | EC:4.1.2.13 | | | | | | |
|  |  | RAFL04-13-J02 | At3g54050 / fructose-bisphosphatase precursor | |  |  |  |  |  | | --- | --- | --- | --- | --- | |  |  |  |  |  | | EC:3.1.3.11 | | | | | | |
|  |  | RAFL07-12-E12 | At2g21330 / fructose-bisphosphate aldolase, putative | |  |  |  |  |  | | --- | --- | --- | --- | --- | |  |  |  |  |  | | EC:4.1.2.13 | | | | | | |
|  |  | RAFL07-18-J01 | At2g21330 / fructose-bisphosphate aldolase, putative | |  |  |  |  |  | | --- | --- | --- | --- | --- | |  |  |  |  |  | | EC:4.1.2.13 | | | | | | |
|  |  | RAFL04-15-A14 | At1g12900 / calcium-binding protein, calreticulin -related | |  |  |  |  |  | | --- | --- | --- | --- | --- | |  |  |  |  |  | | EC:1.2.1.12 | | | | | | |
|  |  | RAFL07-16-P05 | At3g12780 / phosphoglycerate kinase -related | |  |  |  |  |  | | --- | --- | --- | --- | --- | |  |  |  |  |  | | EC:2.7.2.3 | | | | | | |
|  | Cluster:9-0 | |  |  | 4 | 28 | 71 | 4560 | 0.0015783928 | 0.03314625 | 21 |
|  |  | RAFL04-09-D07 | At1g54100 / aldehyde dehydrogenase, putative (ALDH) | |  |  |  |  |  | | --- | --- | --- | --- | --- | |  |  |  |  |  | | EC:1.2.1.3 | | | | | | |
|  |  | RAFL05-21-E06 | At1g54100 / aldehyde dehydrogenase, putative (ALDH) | |  |  |  |  |  | | --- | --- | --- | --- | --- | |  |  |  |  |  | | EC:1.2.1.3 | | | | | | |
|  |  | RAFL08-15-L09 | At1g54100 / aldehyde dehydrogenase, putative (ALDH) | |  |  |  |  |  | | --- | --- | --- | --- | --- | |  |  |  |  |  | | EC:1.2.1.3 | | | | | | |
|  |  | RAFL08-09-C23 | At1g54100 / aldehyde dehydrogenase, putative (ALDH) | |  |  |  |  |  | | --- | --- | --- | --- | --- | |  |  |  |  |  | | EC:1.2.1.3 | | | | | | |
|  | Cluster:3-1 | |  |  | 8 | 208 | 67 | 4380 | 0.021701496 | 0.4557314 | 21 |
|  |  | RAFL07-10-P13 | At1g09780 / 2,3-bisphosphoglycerate-independent phosphoglycerate mutase -related | |  |  |  |  |  | | --- | --- | --- | --- | --- | |  |  |  |  |  | | EC:5.4.2.1 | | | | | | |
|  |  | RAFL09-12-D13 | At5g52920 / pyruvate kinase, putative | |  |  |  |  |  | | --- | --- | --- | --- | --- | |  |  |  |  |  | | EC:2.7.1.40 | | | | | | |
|  |  | RAFL05-05-G17 | At3g25860 / dihydrolipoamide S-acetyltransferase | |  |  |  |  |  | | --- | --- | --- | --- | --- | |  |  |  |  |  | | EC:2.3.1.12 | | | | | | |
|  |  | RAFL04-13-O10 | At2g29560 / enolase (2-phospho-D-glycerate hydroylase) -related | |  |  |  |  |  | | --- | --- | --- | --- | --- | |  |  |  |  |  | | EC:4.2.1.11 | | | | | | |
|  |  | RAFL05-11-L02 | At4g25900 / aldose 1-epimerase family | |  |  |  |  |  | | --- | --- | --- | --- | --- | |  |  |  |  |  | | EC:5.1.3.3 | | | | | | |
|  |  | RAFL05-01-I24 | At3g55440 / triosephosphate isomerase, cytosolic, putative | |  |  |  |  |  | | --- | --- | --- | --- | --- | |  |  |  |  |  | | EC:5.3.1.1 | | | | | | |
|  |  | RAFL04-17-I11 | At3g13930 / acetyltransferase -related | |  |  |  |  |  | | --- | --- | --- | --- | --- | |  |  |  |  |  | | EC:2.3.1.12 | | | | | | |
|  |  | RAFL05-21-G03 | At3g22960 / pyruvate kinase, putative | |  |  |  |  |  | | --- | --- | --- | --- | --- | |  |  |  |  |  | | EC:2.7.1.40 | | | | | | |
| Arginine and proline metabolism | | |  |  | A | B | C | D | P | P' | N |
|  | Cluster:9-0 | |  |  | 4 | 28 | 40 | 4591 | 2.0464069E-4 | 0.0042974544 | 21 |
|  |  | RAFL04-09-D07 | At1g54100 / aldehyde dehydrogenase, putative (ALDH) | |  |  |  |  |  | | --- | --- | --- | --- | --- | |  |  |  |  |  | | EC:1.2.1.3 ,EC:1.5.1.12 | | | | | | |
|  |  | RAFL05-21-E06 | At1g54100 / aldehyde dehydrogenase, putative (ALDH) | |  |  |  |  |  | | --- | --- | --- | --- | --- | |  |  |  |  |  | | EC:1.2.1.3 ,EC:1.5.1.12 | | | | | | |
|  |  | RAFL08-15-L09 | At1g54100 / aldehyde dehydrogenase, putative (ALDH) | |  |  |  |  |  | | --- | --- | --- | --- | --- | |  |  |  |  |  | | EC:1.2.1.3 ,EC:1.5.1.12 | | | | | | |
|  |  | RAFL08-09-C23 | At1g54100 / aldehyde dehydrogenase, putative (ALDH) | |  |  |  |  |  | | --- | --- | --- | --- | --- | |  |  |  |  |  | | EC:1.2.1.3 ,EC:1.5.1.12 | | | | | | |
| Biotin metabolism | | |  |  | A | B | C | D | P | P' | N |
|  | Cluster:7-0 | |  |  | 2 | 245 | 2 | 4414 | 0.015618202 | 0.046854608 | 3 |
|  |  | RAFL07-12-J17 | At3g48780 / serine C-palmitoyltransferase, putative | |  |  |  |  |  | | --- | --- | --- | --- | --- | |  |  |  |  |  | | EC:2.3.1.47 | | | | | | |
|  |  | RAFL05-21-O04 | At3g08860 / alanine--glyoxylate aminotransferase (beta-alanine-pyruvate aminotransferase/AGT), putative | |  |  |  |  |  | | --- | --- | --- | --- | --- | |  |  |  |  |  | | EC:2.6.1.62 | | | | | | |
|  | Cluster:9-0 | |  |  | 1 | 31 | 3 | 4628 | 0.027177518 | 0.08153255 | 3 |
|  |  | RAFL05-08-B14 | At2g38400 / alanine--glyoxylate aminotransferase (beta-alanine-pyruvate aminotransferase/AGT), putative | |  |  |  |  |  | | --- | --- | --- | --- | --- | |  |  |  |  |  | | EC:2.6.1.62 | | | | | | |
| Taurine and hypotaurine metabolism | | |  |  | A | B | C | D | P | P' | N |
|  | Cluster:2-2 | |  |  | 1 | 52 | 0 | 4610 | 0.011366073 | 0.011366073 | 1 |
|  |  | RAFL04-16-J21 | At1g65960 / glutamate decarboxylase 2 (GAD 2) | |  |  |  |  |  | | --- | --- | --- | --- | --- | |  |  |  |  |  | | EC:4.1.1.15 | | | | | | |
| Inositol metabolism | | |  |  | A | B | C | D | P | P' | N |
|  | Cluster:0-1 | |  |  | 7 | 99 | 6 | 4551 | 3.9462105E-9 | 2.3677263E-8 | 6 |
|  |  | RAFL07-18-C20 | At2g21330 / fructose-bisphosphate aldolase, putative | |  |  |  |  |  | | --- | --- | --- | --- | --- | |  |  |  |  |  | | EC:4.1.2.13 | | | | | | |
|  |  | RAFL04-19-O21 | At4g38970 / fructose-bisphosphate aldolase, putative | |  |  |  |  |  | | --- | --- | --- | --- | --- | |  |  |  |  |  | | EC:4.1.2.13 | | | | | | |
|  |  | RAFL09-17-N23 | At2g21330 / fructose-bisphosphate aldolase, putative | |  |  |  |  |  | | --- | --- | --- | --- | --- | |  |  |  |  |  | | EC:4.1.2.13 | | | | | | |
|  |  | RAFL07-12-M09 | At2g21330 / fructose-bisphosphate aldolase, putative | |  |  |  |  |  | | --- | --- | --- | --- | --- | |  |  |  |  |  | | EC:4.1.2.13 | | | | | | |
|  |  | RAFL08-18-C10 | At2g21330 / fructose-bisphosphate aldolase, putative | |  |  |  |  |  | | --- | --- | --- | --- | --- | |  |  |  |  |  | | EC:4.1.2.13 | | | | | | |
|  |  | RAFL07-12-E12 | At2g21330 / fructose-bisphosphate aldolase, putative | |  |  |  |  |  | | --- | --- | --- | --- | --- | |  |  |  |  |  | | EC:4.1.2.13 | | | | | | |
|  |  | RAFL07-18-J01 | At2g21330 / fructose-bisphosphate aldolase, putative | |  |  |  |  |  | | --- | --- | --- | --- | --- | |  |  |  |  |  | | EC:4.1.2.13 | | | | | | |
| Erythromycin biosynthesis | | |  |  | A | B | C | D | P | P' | N |
|  | Cluster:3-2 | |  |  | 2 | 35 | 8 | 4618 | 0.0026489347 | 0.018542543 | 7 |
|  |  | RAFL09-16-F08 | At3g23820 / NAD-dependent epimerase/dehydratase family | |  |  |  |  |  | | --- | --- | --- | --- | --- | |  |  |  |  |  | | EC:4.2.1.46 | | | | | | |
|  |  | RAFL09-07-D12 | At3g23820 / NAD-dependent epimerase/dehydratase family | |  |  |  |  |  | | --- | --- | --- | --- | --- | |  |  |  |  |  | | EC:4.2.1.46 | | | | | | |
|  | Cluster:5-2 | |  |  | 2 | 124 | 8 | 4529 | 0.028284373 | 0.19799061 | 7 |
|  |  | RAFL07-11-C21 | At4g30440 / nucleotide sugar epimerase family | |  |  |  |  |  | | --- | --- | --- | --- | --- | |  |  |  |  |  | | EC:4.2.1.46 | | | | | | |
|  |  | RAFL04-09-G05 | At1g50450 / expressed protein | |  |  |  |  |  | | --- | --- | --- | --- | --- | |  |  |  |  |  | | EC:4.2.1.46 | | | | | | |
| Sterol biosynthesis | | |  |  | A | B | C | D | P | P' | N |
|  | Cluster:0-2 | |  |  | 2 | 77 | 13 | 4571 | 0.025799962 | 0.2837996 | 11 |
|  |  | RAFL04-15-A04 | At4g15560 / DEF (CLA1) protein | |  |  |  |  |  | | --- | --- | --- | --- | --- | |  |  |  |  |  | | EC:2.2.1.7 | | | | | | |
|  |  | RAFL07-12-F08 | At5g17230 / phytoene synthase (geranylgeranyl-diphosphate geranylgeranyl transferase)(PSY) | |  |  |  |  |  | | --- | --- | --- | --- | --- | |  |  |  |  |  | | EC:2.5.1.32 | | | | | | |
| Glutamate metabolism | | |  |  | A | B | C | D | P | P' | N |
|  | Cluster:9-0 | |  |  | 5 | 27 | 42 | 4589 | 1.3746837E-5 | 2.7493676E-4 | 20 |
|  |  | RAFL05-08-B14 | At2g38400 / alanine--glyoxylate aminotransferase (beta-alanine-pyruvate aminotransferase/AGT), putative | |  |  |  |  |  | | --- | --- | --- | --- | --- | |  |  |  |  |  | | EC:2.6.1.19 | | | | | | |
|  |  | RAFL04-09-D07 | At1g54100 / aldehyde dehydrogenase, putative (ALDH) | |  |  |  |  |  | | --- | --- | --- | --- | --- | |  |  |  |  |  | | EC:1.5.1.12 | | | | | | |
|  |  | RAFL05-21-E06 | At1g54100 / aldehyde dehydrogenase, putative (ALDH) | |  |  |  |  |  | | --- | --- | --- | --- | --- | |  |  |  |  |  | | EC:1.5.1.12 | | | | | | |
|  |  | RAFL08-15-L09 | At1g54100 / aldehyde dehydrogenase, putative (ALDH) | |  |  |  |  |  | | --- | --- | --- | --- | --- | |  |  |  |  |  | | EC:1.5.1.12 | | | | | | |
|  |  | RAFL08-09-C23 | At1g54100 / aldehyde dehydrogenase, putative (ALDH) | |  |  |  |  |  | | --- | --- | --- | --- | --- | |  |  |  |  |  | | EC:1.5.1.12 | | | | | | |
|  | Cluster:9-2 | |  |  | 4 | 63 | 43 | 4553 | 0.004365906 | 0.08731812 | 20 |
|  |  | RAFL08-11-N01 | At4g34710 / arginine decarboxylase SPE2 | |  |  |  |  |  | | --- | --- | --- | --- | --- | |  |  |  |  |  | | EC:4.1.1.19 | | | | | | |
|  |  | RAFL08-17-D17 | At3g53180 / nodulin / glutamate-ammonia ligase - like protein | |  |  |  |  |  | | --- | --- | --- | --- | --- | |  |  |  |  |  | | EC:6.3.1.2 | | | | | | |
|  |  | RAFL09-13-D07 | At4g34710 / arginine decarboxylase SPE2 | |  |  |  |  |  | | --- | --- | --- | --- | --- | |  |  |  |  |  | | EC:4.1.1.19 | | | | | | |
|  |  | RAFL06-09-F14 | At3g53180 / nodulin / glutamate-ammonia ligase - like protein | |  |  |  |  |  | | --- | --- | --- | --- | --- | |  |  |  |  |  | | EC:6.3.1.2 | | | | | | |
| Nitrogen metabolism | | |  |  | A | B | C | D | P | P' | N |
|  | Cluster:5-2 | |  |  | 4 | 122 | 38 | 4499 | 0.025769636 | 0.56693196 | 22 |
|  |  | RAFL09-09-P06 | At3g53260 / phenylalanine ammonia-lyase (PAL2) | |  |  |  |  |  | | --- | --- | --- | --- | --- | |  |  |  |  |  | | EC:4.3.1.5 | | | | | | |
|  |  | RAFL11-09-K10 | At1g37130 / nitrate reductase 2 (NR2) | |  |  |  |  |  | | --- | --- | --- | --- | --- | |  |  |  |  |  | | EC:1.7.1.1 | | | | | | |
|  |  | RAFL09-13-L09 | At1g37130 / nitrate reductase 2 (NR2) | |  |  |  |  |  | | --- | --- | --- | --- | --- | |  |  |  |  |  | | EC:1.7.1.1 | | | | | | |
|  |  | RAFL09-11-J22 | At1g37130 / nitrate reductase 2 (NR2) | |  |  |  |  |  | | --- | --- | --- | --- | --- | |  |  |  |  |  | | EC:1.7.1.1 | | | | | | |
|  | Cluster:0-0 | |  |  | 2 | 34 | 40 | 4587 | 0.04113549 | 0.90498084 | 22 |
|  |  | RAFL06-13-B01 | At3g01500 / carbonic anhydrase, chloroplast precursor | |  |  |  |  |  | | --- | --- | --- | --- | --- | |  |  |  |  |  | | EC:4.2.1.1 | | | | | | |
|  |  | RAFL06-11-K17 | At3g01500 / carbonic anhydrase, chloroplast precursor | |  |  |  |  |  | | --- | --- | --- | --- | --- | |  |  |  |  |  | | EC:4.2.1.1 | | | | | | |
| Selenoamino acid metabolism | | |  |  | A | B | C | D | P | P' | N |
|  | Cluster:3-2 | |  |  | 4 | 33 | 24 | 4602 | 5.997704E-5 | 7.797015E-4 | 13 |
|  |  | RAFL09-06-N12 | At3g23810 / S-adenosyl-L-homocysteinas -related | |  |  |  |  |  | | --- | --- | --- | --- | --- | |  |  |  |  |  | | EC:3.3.1.1 | | | | | | |
|  |  | RAFL09-13-P13 | At3g23810 / S-adenosyl-L-homocysteinas -related | |  |  |  |  |  | | --- | --- | --- | --- | --- | |  |  |  |  |  | | EC:3.3.1.1 | | | | | | |
|  |  | RAFL07-09-L01 | At3g23810 / S-adenosyl-L-homocysteinas -related | |  |  |  |  |  | | --- | --- | --- | --- | --- | |  |  |  |  |  | | EC:3.3.1.1 | | | | | | |
|  |  | RAFL09-10-M18 | At3g23810 / S-adenosyl-L-homocysteinas -related | |  |  |  |  |  | | --- | --- | --- | --- | --- | |  |  |  |  |  | | EC:3.3.1.1 | | | | | | |
|  | Cluster:2-2 | |  |  | 2 | 51 | 26 | 4584 | 0.039688654 | 0.5159525 | 13 |
|  |  | RAFL07-10-P11 | At3g22890 / ATP sulfurylase -related | |  |  |  |  |  | | --- | --- | --- | --- | --- | |  |  |  |  |  | | EC:2.7.7.4 | | | | | | |
|  |  | RAFL07-12-E10 | At3g22890 / ATP sulfurylase -related | |  |  |  |  |  | | --- | --- | --- | --- | --- | |  |  |  |  |  | | EC:2.7.7.4 | | | | | | |
|  | Cluster:4-2 | |  |  | 3 | 132 | 25 | 4503 | 0.04587297 | 0.59634864 | 13 |
|  |  | RAFL05-08-P23 | At3g59980 / expressed protein | |  |  |  |  |  | | --- | --- | --- | --- | --- | |  |  |  |  |  | | EC:6.1.1.10 | | | | | | |
|  |  | RAFL11-03-C19 | At3g22890 / ATP sulfurylase -related | |  |  |  |  |  | | --- | --- | --- | --- | --- | |  |  |  |  |  | | EC:2.7.7.4 | | | | | | |
|  |  | RAFL04-17-C12 | At1g02500 / s-adenosylmethionine synthetase | |  |  |  |  |  | | --- | --- | --- | --- | --- | |  |  |  |  |  | | EC:2.5.1.6 | | | | | | |
| Galactose metabolism | | |  |  | A | B | C | D | P | P' | N |
|  | Cluster:3-2 | |  |  | 2 | 35 | 34 | 4592 | 0.032590393 | 0.68439823 | 21 |
|  |  | RAFL09-16-F08 | At3g23820 / NAD-dependent epimerase/dehydratase family | |  |  |  |  |  | | --- | --- | --- | --- | --- | |  |  |  |  |  | | EC:5.1.3.2 | | | | | | |
|  |  | RAFL09-07-D12 | At3g23820 / NAD-dependent epimerase/dehydratase family | |  |  |  |  |  | | --- | --- | --- | --- | --- | |  |  |  |  |  | | EC:5.1.3.2 | | | | | | |
| Propanoate metabolism | | |  |  | A | B | C | D | P | P' | N |
|  | Cluster:9-0 | |  |  | 5 | 27 | 18 | 4613 | 3.387656E-7 | 4.7427184E-6 | 14 |
|  |  | RAFL05-08-B14 | At2g38400 / alanine--glyoxylate aminotransferase (beta-alanine-pyruvate aminotransferase/AGT), putative | |  |  |  |  |  | | --- | --- | --- | --- | --- | |  |  |  |  |  | | EC:2.6.1.19 | | | | | | |
|  |  | RAFL04-09-D07 | At1g54100 / aldehyde dehydrogenase, putative (ALDH) | |  |  |  |  |  | | --- | --- | --- | --- | --- | |  |  |  |  |  | | EC:1.2.1.3 | | | | | | |
|  |  | RAFL05-21-E06 | At1g54100 / aldehyde dehydrogenase, putative (ALDH) | |  |  |  |  |  | | --- | --- | --- | --- | --- | |  |  |  |  |  | | EC:1.2.1.3 | | | | | | |
|  |  | RAFL08-15-L09 | At1g54100 / aldehyde dehydrogenase, putative (ALDH) | |  |  |  |  |  | | --- | --- | --- | --- | --- | |  |  |  |  |  | | EC:1.2.1.3 | | | | | | |
|  |  | RAFL08-09-C23 | At1g54100 / aldehyde dehydrogenase, putative (ALDH) | |  |  |  |  |  | | --- | --- | --- | --- | --- | |  |  |  |  |  | | EC:1.2.1.3 | | | | | | |
| Sulfur metabolism | | |  |  | A | B | C | D | P | P' | N |
|  | Cluster:2-2 | |  |  | 3 | 50 | 15 | 4595 | 0.0010030596 | 0.012036716 | 12 |
|  |  | RAFL04-17-H16 | At3g13110 / serine acetyltransferase (Sat-1) | |  |  |  |  |  | | --- | --- | --- | --- | --- | |  |  |  |  |  | | EC:2.3.1.30 | | | | | | |
|  |  | RAFL07-10-P11 | At3g22890 / ATP sulfurylase -related | |  |  |  |  |  | | --- | --- | --- | --- | --- | |  |  |  |  |  | | EC:2.7.7.4 | | | | | | |
|  |  | RAFL07-12-E10 | At3g22890 / ATP sulfurylase -related | |  |  |  |  |  | | --- | --- | --- | --- | --- | |  |  |  |  |  | | EC:2.7.7.4 | | | | | | |
| Pentose phosphate pathway | | |  |  | A | B | C | D | P | P' | N |
|  | Cluster:0-1 | |  |  | 9 | 97 | 24 | 4533 | 2.8207568E-8 | 4.5132109E-7 | 16 |
|  |  | RAFL07-18-C20 | At2g21330 / fructose-bisphosphate aldolase, putative | |  |  |  |  |  | | --- | --- | --- | --- | --- | |  |  |  |  |  | | EC:4.1.2.13 | | | | | | |
|  |  | RAFL04-19-O21 | At4g38970 / fructose-bisphosphate aldolase, putative | |  |  |  |  |  | | --- | --- | --- | --- | --- | |  |  |  |  |  | | EC:4.1.2.13 | | | | | | |
|  |  | RAFL09-17-N23 | At2g21330 / fructose-bisphosphate aldolase, putative | |  |  |  |  |  | | --- | --- | --- | --- | --- | |  |  |  |  |  | | EC:4.1.2.13 | | | | | | |
|  |  | RAFL07-12-M09 | At2g21330 / fructose-bisphosphate aldolase, putative | |  |  |  |  |  | | --- | --- | --- | --- | --- | |  |  |  |  |  | | EC:4.1.2.13 | | | | | | |
|  |  | RAFL08-18-C10 | At2g21330 / fructose-bisphosphate aldolase, putative | |  |  |  |  |  | | --- | --- | --- | --- | --- | |  |  |  |  |  | | EC:4.1.2.13 | | | | | | |
|  |  | RAFL04-13-J02 | At3g54050 / fructose-bisphosphatase precursor | |  |  |  |  |  | | --- | --- | --- | --- | --- | |  |  |  |  |  | | EC:3.1.3.11 | | | | | | |
|  |  | RAFL07-12-E12 | At2g21330 / fructose-bisphosphate aldolase, putative | |  |  |  |  |  | | --- | --- | --- | --- | --- | |  |  |  |  |  | | EC:4.1.2.13 | | | | | | |
|  |  | RAFL07-18-J01 | At2g21330 / fructose-bisphosphate aldolase, putative | |  |  |  |  |  | | --- | --- | --- | --- | --- | |  |  |  |  |  | | EC:4.1.2.13 | | | | | | |
|  |  | RAFL04-10-J07 | At3g04790 / ribose 5-phosphate isomerase -related | |  |  |  |  |  | | --- | --- | --- | --- | --- | |  |  |  |  |  | | EC:5.3.1.6 | | | | | | |
|  | Cluster:1-2 | |  |  | 4 | 170 | 29 | 4460 | 0.032959472 | 0.52735156 | 16 |
|  |  | RAFL07-12-L15 | At1g12000 / pyrophosphate-fructose-6-phosphate 1-phosphotransferase -related | |  |  |  |  |  | | --- | --- | --- | --- | --- | |  |  |  |  |  | | EC:2.7.1.11 | | | | | | |
|  |  | RAFL05-21-I19 | At1g43670 / fructose 1,6-bisphosphatase -related | |  |  |  |  |  | | --- | --- | --- | --- | --- | |  |  |  |  |  | | EC:3.1.3.11 | | | | | | |
|  |  | RAFL07-15-F22 | At1g20950 / pyrophosphate-dependent phosphofructokinase alpha subunit -related | |  |  |  |  |  | | --- | --- | --- | --- | --- | |  |  |  |  |  | | EC:2.7.1.11 | | | | | | |
|  |  | RAFL05-13-B09 | At5g03300 / pfkB type carbohydrate kinase protein family | |  |  |  |  |  | | --- | --- | --- | --- | --- | |  |  |  |  |  | | EC:2.7.1.11 | | | | | | |
| Peptidoglycan biosynthesis | | |  |  | A | B | C | D | P | P' | N |
|  | Cluster:9-2 | |  |  | 2 | 65 | 2 | 4594 | 0.0011979077 | 0.0035937233 | 3 |
|  |  | RAFL08-17-D17 | At3g53180 / nodulin / glutamate-ammonia ligase - like protein | |  |  |  |  |  | | --- | --- | --- | --- | --- | |  |  |  |  |  | | EC:6.3.1.2 | | | | | | |
|  |  | RAFL06-09-F14 | At3g53180 / nodulin / glutamate-ammonia ligase - like protein | |  |  |  |  |  | | --- | --- | --- | --- | --- | |  |  |  |  |  | | EC:6.3.1.2 | | | | | | |
|  | Cluster:2-2 | |  |  | 1 | 52 | 3 | 4607 | 0.044709165 | 0.13412748 | 3 |
|  |  | RAFL04-16-N11 | At5g35630 / glutamate-ammonia ligase (EC 6.3.1.2) precursor, chloroplast (clone lambdaAtgsl1) (pir||S18600) | |  |  |  |  |  | | --- | --- | --- | --- | --- | |  |  |  |  |  | | EC:6.3.1.2 | | | | | | |
| Phenylalanine metabolism | | |  |  | A | B | C | D | P | P' | N |
|  | Cluster:9-1 | |  |  | 5 | 91 | 32 | 4535 | 8.6172705E-4 | 0.015511087 | 18 |
|  |  | RAFL05-19-H07 | At5g11520 / aspartate aminotransferase, chloroplast (transaminase A/Asp3) | |  |  |  |  |  | | --- | --- | --- | --- | --- | |  |  |  |  |  | | EC:2.6.1.1 | | | | | | |
|  |  | RAFL04-20-P19 | At3g49110 / peroxidase | |  |  |  |  |  | | --- | --- | --- | --- | --- | |  |  |  |  |  | | EC:1.11.1.7 | | | | | | |
|  |  | RAFL11-09-O05 | At1g06570 / 4-hydroxyphenylpyruvate dioxygenase (HPD) | |  |  |  |  |  | | --- | --- | --- | --- | --- | |  |  |  |  |  | | EC:1.13.11.27 | | | | | | |
|  |  | RAFL11-12-C18 | At1g06570 / 4-hydroxyphenylpyruvate dioxygenase (HPD) | |  |  |  |  |  | | --- | --- | --- | --- | --- | |  |  |  |  |  | | EC:1.13.11.27 | | | | | | |
|  |  | RAFL09-07-G15 | At3g49120 / peroxidase, putative | |  |  |  |  |  | | --- | --- | --- | --- | --- | |  |  |  |  |  | | EC:1.11.1.7 | | | | | | |
|  | Cluster:6-2 | |  |  | 4 | 172 | 33 | 4454 | 0.0491507 | 0.8847126 | 18 |
|  |  | RAFL02-07-M07 | At1g08980 / amidase | |  |  |  |  |  | | --- | --- | --- | --- | --- | |  |  |  |  |  | | EC:3.5.1.4 | | | | | | |
|  |  | RAFL04-13-B02 | At2g37040 / phenylalanine ammonia lyase (PAL1) | |  |  |  |  |  | | --- | --- | --- | --- | --- | |  |  |  |  |  | | EC:4.3.1.5 | | | | | | |
|  |  | RAFL09-11-L22 | At3g53260 / phenylalanine ammonia-lyase (PAL2) | |  |  |  |  |  | | --- | --- | --- | --- | --- | |  |  |  |  |  | | EC:4.3.1.5 | | | | | | |
|  |  | RAFL04-16-D08 | At3g53260 / phenylalanine ammonia-lyase (PAL2) | |  |  |  |  |  | | --- | --- | --- | --- | --- | |  |  |  |  |  | | EC:4.3.1.5 | | | | | | |
| Methane metabolism | | |  |  | A | B | C | D | P | P' | N |
|  | Cluster:6-0 | |  |  | 3 | 137 | 20 | 4503 | 0.030230032 | 0.42322046 | 14 |
|  |  | RAFL09-13-G24 | At1g20620 / catalase 3 | |  |  |  |  |  | | --- | --- | --- | --- | --- | |  |  |  |  |  | | EC:1.11.1.6 | | | | | | |
|  |  | RAFL06-08-C18 | At4g37520 / peroxidase, putative | |  |  |  |  |  | | --- | --- | --- | --- | --- | |  |  |  |  |  | | EC:1.11.1.7 | | | | | | |
|  |  | RAFL05-18-P09 | At1g71695 / peroxidase, putative | |  |  |  |  |  | | --- | --- | --- | --- | --- | |  |  |  |  |  | | EC:1.11.1.7 | | | | | | |
| Glyoxylate and dicarboxylate metabolism | | |  |  | A | B | C | D | P | P' | N |
|  | Cluster:0-0 | |  |  | 15 | 21 | 34 | 4593 | 9.474795E-22 | 1.5159671E-20 | 16 |
|  |  | RAFL09-16-C21 | At5g38410 / ribulose bisphosphate carboxylase small chain 3b precursor (RuBisCO small subunit 3b) (sp|P10798) | |  |  |  |  |  | | --- | --- | --- | --- | --- | |  |  |  |  |  | | EC:4.1.1.39 | | | | | | |
|  |  | RAFL09-09-K05 | At5g38410 / ribulose bisphosphate carboxylase small chain 3b precursor (RuBisCO small subunit 3b) (sp|P10798) | |  |  |  |  |  | | --- | --- | --- | --- | --- | |  |  |  |  |  | | EC:4.1.1.39 | | | | | | |
|  |  | RAFL11-03-H09 | At1g67090 / ribulose-bisphosphate carboxylase small unit -related | |  |  |  |  |  | | --- | --- | --- | --- | --- | |  |  |  |  |  | | EC:4.1.1.39 | | | | | | |
|  |  | RAFL06-10-O15 | At5g38420 / ribulose bisphosphate carboxylase small chain 2b precursor (RuBisCO small subunit 2b) (sp|P10797) | |  |  |  |  |  | | --- | --- | --- | --- | --- | |  |  |  |  |  | | EC:4.1.1.39 | | | | | | |
|  |  | RAFL04-15-J15 | At5g38410 / ribulose bisphosphate carboxylase small chain 3b precursor (RuBisCO small subunit 3b) (sp|P10798) | |  |  |  |  |  | | --- | --- | --- | --- | --- | |  |  |  |  |  | | EC:4.1.1.39 | | | | | | |
|  |  | RAFL08-17-J10 | At5g38420 / ribulose bisphosphate carboxylase small chain 2b precursor (RuBisCO small subunit 2b) (sp|P10797) | |  |  |  |  |  | | --- | --- | --- | --- | --- | |  |  |  |  |  | | EC:4.1.1.39 | | | | | | |
|  |  | RAFL06-07-I02 | At1g67090 / ribulose-bisphosphate carboxylase small unit -related | |  |  |  |  |  | | --- | --- | --- | --- | --- | |  |  |  |  |  | | EC:4.1.1.39 | | | | | | |
|  |  | RAFL09-09-L07 | At1g67090 / ribulose-bisphosphate carboxylase small unit -related | |  |  |  |  |  | | --- | --- | --- | --- | --- | |  |  |  |  |  | | EC:4.1.1.39 | | | | | | |
|  |  | RAFL07-14-L17 | At5g38420 / ribulose bisphosphate carboxylase small chain 2b precursor (RuBisCO small subunit 2b) (sp|P10797) | |  |  |  |  |  | | --- | --- | --- | --- | --- | |  |  |  |  |  | | EC:4.1.1.39 | | | | | | |
|  |  | RAFL06-13-H11 | At5g38430 / ribulose bisphosphate carboxylase small chain 1b precursor (RuBisCO small subunit 1b) (sp|P10796) | |  |  |  |  |  | | --- | --- | --- | --- | --- | |  |  |  |  |  | | EC:4.1.1.39 | | | | | | |
|  |  | RAFL06-14-L16 | At5g38430 / ribulose bisphosphate carboxylase small chain 1b precursor (RuBisCO small subunit 1b) (sp|P10796) | |  |  |  |  |  | | --- | --- | --- | --- | --- | |  |  |  |  |  | | EC:4.1.1.39 | | | | | | |
|  |  | RAFL07-11-L12 | At5g38420 / ribulose bisphosphate carboxylase small chain 2b precursor (RuBisCO small subunit 2b) (sp|P10797) | |  |  |  |  |  | | --- | --- | --- | --- | --- | |  |  |  |  |  | | EC:4.1.1.39 | | | | | | |
|  |  | RAFL06-14-C14 | At1g67090 / ribulose-bisphosphate carboxylase small unit -related | |  |  |  |  |  | | --- | --- | --- | --- | --- | |  |  |  |  |  | | EC:4.1.1.39 | | | | | | |
|  |  | RAFL06-14-C19 | At5g38410 / ribulose bisphosphate carboxylase small chain 3b precursor (RuBisCO small subunit 3b) (sp|P10798) | |  |  |  |  |  | | --- | --- | --- | --- | --- | |  |  |  |  |  | | EC:4.1.1.39 | | | | | | |
|  |  | RAFL06-08-L09 | At5g38420 / ribulose bisphosphate carboxylase small chain 2b precursor (RuBisCO small subunit 2b) (sp|P10797) | |  |  |  |  |  | | --- | --- | --- | --- | --- | |  |  |  |  |  | | EC:4.1.1.39 | | | | | | |
|  | Cluster:1-0 | |  |  | 6 | 143 | 43 | 4471 | 0.0043278704 | 0.06924593 | 16 |
|  |  | RAFL04-13-N06 | At1g68010 / glycerate dehydrogenase (NADH-dependent hydroxypyruvate reductase) (HPR) | |  |  |  |  |  | | --- | --- | --- | --- | --- | |  |  |  |  |  | | EC:1.1.1.81 ,EC:1.1.1.29 | | | | | | |
|  |  | RAFL11-05-D24 | At1g67090 / ribulose-bisphosphate carboxylase small unit -related | |  |  |  |  |  | | --- | --- | --- | --- | --- | |  |  |  |  |  | | EC:4.1.1.39 | | | | | | |
|  |  | RAFL06-14-K21 | At5g36700 / phosphoglycolate phosphatase, putative | |  |  |  |  |  | | --- | --- | --- | --- | --- | |  |  |  |  |  | | EC:3.1.3.18 | | | | | | |
|  |  | RAFL08-15-E10 | At3g14420 / glycolate oxidase -related | |  |  |  |  |  | | --- | --- | --- | --- | --- | |  |  |  |  |  | | EC:1.1.3.15 | | | | | | |
|  |  | RAFL05-03-H12 | At5g09660 / malate dehydrogenase, glyoxysomal | |  |  |  |  |  | | --- | --- | --- | --- | --- | |  |  |  |  |  | | EC:1.1.1.37 | | | | | | |
|  |  | RAFL09-13-P20 | At3g14420 / glycolate oxidase -related | |  |  |  |  |  | | --- | --- | --- | --- | --- | |  |  |  |  |  | | EC:1.1.3.15 | | | | | | |
|  | Cluster:0-1 | |  |  | 5 | 101 | 44 | 4513 | 0.0047566555 | 0.07610649 | 16 |
|  |  | RAFL11-02-L02 | At1g67090 / ribulose-bisphosphate carboxylase small unit -related | |  |  |  |  |  | | --- | --- | --- | --- | --- | |  |  |  |  |  | | EC:4.1.1.39 | | | | | | |
|  |  | RAFL03-06-F08 | At1g67090 / ribulose-bisphosphate carboxylase small unit -related | |  |  |  |  |  | | --- | --- | --- | --- | --- | |  |  |  |  |  | | EC:4.1.1.39 | | | | | | |
|  |  | RAFL09-06-K21 | At1g67090 / ribulose-bisphosphate carboxylase small unit -related | |  |  |  |  |  | | --- | --- | --- | --- | --- | |  |  |  |  |  | | EC:4.1.1.39 | | | | | | |
|  |  | RAFL11-07-D01 | At5g38410 / ribulose bisphosphate carboxylase small chain 3b precursor (RuBisCO small subunit 3b) (sp|P10798) | |  |  |  |  |  | | --- | --- | --- | --- | --- | |  |  |  |  |  | | EC:4.1.1.39 | | | | | | |
|  |  | RAFL09-06-P15 | At1g67090 / ribulose-bisphosphate carboxylase small unit -related | |  |  |  |  |  | | --- | --- | --- | --- | --- | |  |  |  |  |  | | EC:4.1.1.39 | | | | | | |
| Benzoate degradation via CoA ligation | | |  |  | A | B | C | D | P | P' | N |
|  | Cluster:8-0 | |  |  | 4 | 105 | 10 | 4544 | 2.3608547E-4 | 0.0016525983 | 7 |
|  |  | RAFL06-13-H12 | At3g51840 / acyl-coA dehydrogenase | |  |  |  |  |  | | --- | --- | --- | --- | --- | |  |  |  |  |  | | EC:1.3.99.7 | | | | | | |
|  |  | RAFL06-13-E03 | At3g44300 / nitrilase 2 | |  |  |  |  |  | | --- | --- | --- | --- | --- | |  |  |  |  |  | | EC:3.5.5.1 | | | | | | |
|  |  | RAFL04-12-F14 | At3g27380 / succinate dehydrogenase, iron-sulphur subunit, mitochondrial (sdh2-1) | |  |  |  |  |  | | --- | --- | --- | --- | --- | |  |  |  |  |  | | EC:1.3.99.1 | | | | | | |
|  |  | RAFL08-10-H06 | At3g44300 / nitrilase 2 | |  |  |  |  |  | | --- | --- | --- | --- | --- | |  |  |  |  |  | | EC:3.5.5.1 | | | | | | |
|  | Cluster:6-1 | |  |  | 4 | 311 | 10 | 4338 | 0.011896291 | 0.08327404 | 7 |
|  |  | RAFL09-14-D16 | At5g22300 / Nitrilase 4 (sp P46011) | |  |  |  |  |  | | --- | --- | --- | --- | --- | |  |  |  |  |  | | EC:4.2.1.84 ,EC:3.5.5.1 | | | | | | |
|  |  | RAFL05-17-A04 | At5g09600 / expressed protein | |  |  |  |  |  | | --- | --- | --- | --- | --- | |  |  |  |  |  | | EC:1.3.99.1 | | | | | | |
|  |  | RAFL07-15-A09 | At3g06860 / fatty acid multifunctional protein (AtMFP2) | |  |  |  |  |  | | --- | --- | --- | --- | --- | |  |  |  |  |  | | EC:4.2.1.17 | | | | | | |
|  |  | RAFL11-10-D22 | At5g43280 / enoyl-CoA hydratase/isomerase family | |  |  |  |  |  | | --- | --- | --- | --- | --- | |  |  |  |  |  | | EC:4.2.1.17 | | | | | | |
| Aminosugars metabolism | | |  |  | A | B | C | D | P | P' | N |
|  | Cluster:1-1 | |  |  | 2 | 103 | 3 | 4555 | 0.004804865 | 0.01921946 | 4 |
|  |  | RAFL09-18-H10 | At5g19220 / glucose-1-phosphate adenylyltransferase, large subunit 1, chloroplast (ADP-glucose pyrophosphorylase) (ADG2) (APL1) | |  |  |  |  |  | | --- | --- | --- | --- | --- | |  |  |  |  |  | | EC:2.7.7.23 | | | | | | |
|  |  | RAFL09-13-M20 | At1g31070 / UDP-N-acetylglucosamine pyrophosphorylase-related protein | |  |  |  |  |  | | --- | --- | --- | --- | --- | |  |  |  |  |  | | EC:2.7.7.23 | | | | | | |
| D-Arginine and D-ornithine metabolism | | |  |  | A | B | C | D | P | P' | N |
|  | Cluster:0-1 | |  |  | 3 | 103 | 5 | 4552 | 5.885334E-4 | 0.0035312006 | 6 |
|  |  | RAFL05-07-J06 | At1g42970 / glyceraldehyde-3-phosphate dehydrogenase | |  |  |  |  |  | | --- | --- | --- | --- | --- | |  |  |  |  |  | | EC:1.2.1.12 | | | | | | |
|  |  | RAFL04-09-D24 | At1g42970 / glyceraldehyde-3-phosphate dehydrogenase | |  |  |  |  |  | | --- | --- | --- | --- | --- | |  |  |  |  |  | | EC:1.2.1.12 | | | | | | |
|  |  | RAFL04-15-A14 | At1g12900 / calcium-binding protein, calreticulin -related | |  |  |  |  |  | | --- | --- | --- | --- | --- | |  |  |  |  |  | | EC:1.2.1.12 | | | | | | |
| Fructose and mannose metabolism | | |  |  | A | B | C | D | P | P' | N |
|  | Cluster:0-1 | |  |  | 8 | 98 | 25 | 4532 | 4.73974E-7 | 6.161662E-6 | 13 |
|  |  | RAFL07-18-C20 | At2g21330 / fructose-bisphosphate aldolase, putative | |  |  |  |  |  | | --- | --- | --- | --- | --- | |  |  |  |  |  | | EC:4.1.2.13 | | | | | | |
|  |  | RAFL04-19-O21 | At4g38970 / fructose-bisphosphate aldolase, putative | |  |  |  |  |  | | --- | --- | --- | --- | --- | |  |  |  |  |  | | EC:4.1.2.13 | | | | | | |
|  |  | RAFL09-17-N23 | At2g21330 / fructose-bisphosphate aldolase, putative | |  |  |  |  |  | | --- | --- | --- | --- | --- | |  |  |  |  |  | | EC:4.1.2.13 | | | | | | |
|  |  | RAFL07-12-M09 | At2g21330 / fructose-bisphosphate aldolase, putative | |  |  |  |  |  | | --- | --- | --- | --- | --- | |  |  |  |  |  | | EC:4.1.2.13 | | | | | | |
|  |  | RAFL08-18-C10 | At2g21330 / fructose-bisphosphate aldolase, putative | |  |  |  |  |  | | --- | --- | --- | --- | --- | |  |  |  |  |  | | EC:4.1.2.13 | | | | | | |
|  |  | RAFL04-13-J02 | At3g54050 / fructose-bisphosphatase precursor | |  |  |  |  |  | | --- | --- | --- | --- | --- | |  |  |  |  |  | | EC:3.1.3.11 | | | | | | |
|  |  | RAFL07-12-E12 | At2g21330 / fructose-bisphosphate aldolase, putative | |  |  |  |  |  | | --- | --- | --- | --- | --- | |  |  |  |  |  | | EC:4.1.2.13 | | | | | | |
|  |  | RAFL07-18-J01 | At2g21330 / fructose-bisphosphate aldolase, putative | |  |  |  |  |  | | --- | --- | --- | --- | --- | |  |  |  |  |  | | EC:4.1.2.13 | | | | | | |
|  | Cluster:1-2 | |  |  | 5 | 169 | 28 | 4461 | 0.0069361012 | 0.09016931 | 13 |
|  |  | RAFL04-09-G20 | At2g21170 / triosephosphate isomerase, chloroplast, putative | |  |  |  |  |  | | --- | --- | --- | --- | --- | |  |  |  |  |  | | EC:5.3.1.1 | | | | | | |
|  |  | RAFL07-12-L15 | At1g12000 / pyrophosphate-fructose-6-phosphate 1-phosphotransferase -related | |  |  |  |  |  | | --- | --- | --- | --- | --- | |  |  |  |  |  | | EC:2.7.1.90 ,EC:2.7.1.11 | | | | | | |
|  |  | RAFL05-21-I19 | At1g43670 / fructose 1,6-bisphosphatase -related | |  |  |  |  |  | | --- | --- | --- | --- | --- | |  |  |  |  |  | | EC:3.1.3.11 | | | | | | |
|  |  | RAFL07-15-F22 | At1g20950 / pyrophosphate-dependent phosphofructokinase alpha subunit -related | |  |  |  |  |  | | --- | --- | --- | --- | --- | |  |  |  |  |  | | EC:2.7.1.90 ,EC:2.7.1.11 | | | | | | |
|  |  | RAFL05-13-B09 | At5g03300 / pfkB type carbohydrate kinase protein family | |  |  |  |  |  | | --- | --- | --- | --- | --- | |  |  |  |  |  | | EC:2.7.1.11 | | | | | | |
|  | Cluster:3-2 | |  |  | 2 | 35 | 31 | 4595 | 0.027721709 | 0.3603822 | 13 |
|  |  | RAFL09-16-F08 | At3g23820 / NAD-dependent epimerase/dehydratase family | |  |  |  |  |  | | --- | --- | --- | --- | --- | |  |  |  |  |  | | EC:4.2.1.47 | | | | | | |
|  |  | RAFL09-07-D12 | At3g23820 / NAD-dependent epimerase/dehydratase family | |  |  |  |  |  | | --- | --- | --- | --- | --- | |  |  |  |  |  | | EC:4.2.1.47 | | | | | | |
| Tyrosine metabolism | | |  |  | A | B | C | D | P | P' | N |
|  | Cluster:9-1 | |  |  | 4 | 92 | 25 | 4542 | 0.0027000385 | 0.03780054 | 14 |
|  |  | RAFL05-19-H07 | At5g11520 / aspartate aminotransferase, chloroplast (transaminase A/Asp3) | |  |  |  |  |  | | --- | --- | --- | --- | --- | |  |  |  |  |  | | EC:2.6.1.1 | | | | | | |
|  |  | RAFL07-16-P10 | At1g77120 / alcohol dehydrogenase (ADH) | |  |  |  |  |  | | --- | --- | --- | --- | --- | |  |  |  |  |  | | EC:1.1.1.1 | | | | | | |
|  |  | RAFL11-09-O05 | At1g06570 / 4-hydroxyphenylpyruvate dioxygenase (HPD) | |  |  |  |  |  | | --- | --- | --- | --- | --- | |  |  |  |  |  | | EC:1.13.11.27 | | | | | | |
|  |  | RAFL11-12-C18 | At1g06570 / 4-hydroxyphenylpyruvate dioxygenase (HPD) | |  |  |  |  |  | | --- | --- | --- | --- | --- | |  |  |  |  |  | | EC:1.13.11.27 | | | | | | |
|  | Cluster:6-2 | |  |  | 4 | 172 | 25 | 4462 | 0.02227629 | 0.31186807 | 14 |
|  |  | RAFL02-07-O01 | At2g24270 / NADP-dependent glyceraldehyde-3-phosphate dehydrogenase, putative | |  |  |  |  |  | | --- | --- | --- | --- | --- | |  |  |  |  |  | | EC:1.2.1.16 | | | | | | |
|  |  | RAFL04-13-B02 | At2g37040 / phenylalanine ammonia lyase (PAL1) | |  |  |  |  |  | | --- | --- | --- | --- | --- | |  |  |  |  |  | | EC:4.3.1.5 | | | | | | |
|  |  | RAFL09-11-L22 | At3g53260 / phenylalanine ammonia-lyase (PAL2) | |  |  |  |  |  | | --- | --- | --- | --- | --- | |  |  |  |  |  | | EC:4.3.1.5 | | | | | | |
|  |  | RAFL04-16-D08 | At3g53260 / phenylalanine ammonia-lyase (PAL2) | |  |  |  |  |  | | --- | --- | --- | --- | --- | |  |  |  |  |  | | EC:4.3.1.5 | | | | | | |
|  | Cluster:5-1 | |  |  | 5 | 277 | 24 | 4357 | 0.028004097 | 0.39205736 | 14 |
|  |  | RAFL08-15-H03 | At1g79440 / succinate-semialdehyde dehydrogenase, putative (SSDH) | |  |  |  |  |  | | --- | --- | --- | --- | --- | |  |  |  |  |  | | EC:1.2.1.16 | | | | | | |
|  |  | RAFL05-18-D02 | At3g43670 / amine oxidase -related protein | |  |  |  |  |  | | --- | --- | --- | --- | --- | |  |  |  |  |  | | EC:1.4.3.6 | | | | | | |
|  |  | RAFL05-14-M18 | At1g12050 / fumarylacetoacetate hydrolase-related protein | |  |  |  |  |  | | --- | --- | --- | --- | --- | |  |  |  |  |  | | EC:3.7.1.2 | | | | | | |
|  |  | RAFL08-16-B22 | At1g11840 / glyoxalase I, putative (lactoylglutathione lyase) | |  |  |  |  |  | | --- | --- | --- | --- | --- | |  |  |  |  |  | | EC:1.13.11.27 | | | | | | |
|  |  | RAFL09-07-G14 | At1g67280 / glyoxalase I, putative (lactoylglutathione lyase) | |  |  |  |  |  | | --- | --- | --- | --- | --- | |  |  |  |  |  | | EC:1.13.11.27 | | | | | | |
| Synthesis and degradation of ketone bodies | | |  |  | A | B | C | D | P | P' | N |
|  | Cluster:10-2 | |  |  | 1 | 108 | 1 | 4553 | 0.0462095 | 0.092419 | 2 |
|  |  | RAFL06-10-M04 | At2g26800 / hydroxymethylglutaryl-CoA lyase -related | |  |  |  |  |  | | --- | --- | --- | --- | --- | |  |  |  |  |  | | EC:4.1.3.4 | | | | | | |
| Urea cycle and metabolism of amino groups | | |  |  | A | B | C | D | P | P' | N |
|  | Cluster:9-0 | |  |  | 4 | 28 | 18 | 4613 | 1.2259562E-5 | 1.4711474E-4 | 12 |
|  |  | RAFL04-09-D07 | At1g54100 / aldehyde dehydrogenase, putative (ALDH) | |  |  |  |  |  | | --- | --- | --- | --- | --- | |  |  |  |  |  | | EC:1.2.1.41 | | | | | | |
|  |  | RAFL05-21-E06 | At1g54100 / aldehyde dehydrogenase, putative (ALDH) | |  |  |  |  |  | | --- | --- | --- | --- | --- | |  |  |  |  |  | | EC:1.2.1.41 | | | | | | |
|  |  | RAFL08-15-L09 | At1g54100 / aldehyde dehydrogenase, putative (ALDH) | |  |  |  |  |  | | --- | --- | --- | --- | --- | |  |  |  |  |  | | EC:1.2.1.41 | | | | | | |
|  |  | RAFL08-09-C23 | At1g54100 / aldehyde dehydrogenase, putative (ALDH) | |  |  |  |  |  | | --- | --- | --- | --- | --- | |  |  |  |  |  | | EC:1.2.1.41 | | | | | | |
|  | Cluster:3-1 | |  |  | 5 | 211 | 17 | 4430 | 0.0028076097 | 0.033691317 | 12 |
|  |  | RAFL04-16-G24 | At1g80600 / acetylornithine aminotransferase, mitochondrial (acetylornithine transaminase/AOTA/ACOAT), putative | |  |  |  |  |  | | --- | --- | --- | --- | --- | |  |  |  |  |  | | EC:2.6.1.13 ,EC:2.6.1.11 | | | | | | |
|  |  | RAFL06-16-I04 | At5g19530 / spermine synthase (ACL5) | |  |  |  |  |  | | --- | --- | --- | --- | --- | |  |  |  |  |  | | EC:2.5.1.22 | | | | | | |
|  |  | RAFL07-16-F16 | At3g20330 / aspartate carbamoyltransferase precursor (aspartate transcarbamylase) | |  |  |  |  |  | | --- | --- | --- | --- | --- | |  |  |  |  |  | | EC:2.1.3.3 | | | | | | |
|  |  | RAFL07-18-A10 | At2g37500 / glutamate/ornithine acetyltransferase -related | |  |  |  |  |  | | --- | --- | --- | --- | --- | |  |  |  |  |  | | EC:2.3.1.35 ,EC:2.3.1.1 | | | | | | |
|  |  | RAFL07-08-L02 | At5g10920 / argininosuccinate lyase (AtArgH) | |  |  |  |  |  | | --- | --- | --- | --- | --- | |  |  |  |  |  | | EC:4.3.2.1 | | | | | | |
| Ubiquinone biosynthesis | | |  |  | A | B | C | D | P | P' | N |
|  | Cluster:4-1 | |  |  | 5 | 305 | 6 | 4347 | 4.162242E-4 | 0.0016648968 | 4 |
|  |  | RAFL05-04-N24 | At3g18410 / expressed protein | |  |  |  |  |  | | --- | --- | --- | --- | --- | |  |  |  |  |  | | EC:1.6.5.3 | | | | | | |
|  |  | RAFL09-18-I01 | At5g08530 / NADH-ubiquinone oxidoreductase (mitochondrial), putative | |  |  |  |  |  | | --- | --- | --- | --- | --- | |  |  |  |  |  | | EC:1.6.5.3 | | | | | | |
|  |  | RAFL09-10-O11 | At3g12260 / expressed protein | |  |  |  |  |  | | --- | --- | --- | --- | --- | |  |  |  |  |  | | EC:1.6.5.3 | | | | | | |
|  |  | RAFL11-02-J20 | At3g12260 / expressed protein | |  |  |  |  |  | | --- | --- | --- | --- | --- | |  |  |  |  |  | | EC:1.6.5.3 | | | | | | |
|  |  | RAFL06-08-D19 | At5g37510 / NADH dehydrogenase (ubiquinone), mitochondrial, putative | |  |  |  |  |  | | --- | --- | --- | --- | --- | |  |  |  |  |  | | EC:1.6.5.3 | | | | | | |
|  | Cluster:6-1 | |  |  | 3 | 312 | 8 | 4340 | 0.033555806 | 0.13422322 | 4 |
|  |  | RAFL07-07-N09 | At5g37510 / NADH dehydrogenase (ubiquinone), mitochondrial, putative | |  |  |  |  |  | | --- | --- | --- | --- | --- | |  |  |  |  |  | | EC:1.6.5.3 | | | | | | |
|  |  | RAFL05-08-F21 | At3g03100 / expressed protein | |  |  |  |  |  | | --- | --- | --- | --- | --- | |  |  |  |  |  | | EC:1.6.5.3 | | | | | | |
|  |  | RAFL06-10-E05 | At1g16700 / NADH:ubiquinone oxidoreductase -related | |  |  |  |  |  | | --- | --- | --- | --- | --- | |  |  |  |  |  | | EC:1.6.5.3 | | | | | | |
|  | Cluster:6-0 | |  |  | 2 | 138 | 9 | 4514 | 0.04121896 | 0.16487584 | 4 |
|  |  | RAFL05-17-L16 | At1g79010 / NADH dehydrogenase -related | |  |  |  |  |  | | --- | --- | --- | --- | --- | |  |  |  |  |  | | EC:1.6.5.3 | | | | | | |
|  |  | RAFL08-12-A05 | At5g11770 / NADH dehydrogenase (ubiquinone) | |  |  |  |  |  | | --- | --- | --- | --- | --- | |  |  |  |  |  | | EC:1.6.5.3 | | | | | | |
| Pentose and glucuronate interconversions | | |  |  | A | B | C | D | P | P' | N |
|  | Cluster:3-0 | |  |  | 3 | 230 | 12 | 4418 | 0.03588357 | 0.3588357 | 10 |
|  |  | RAFL05-21-O08 | At5g61410 / ribulose-5-phosphate-3-epimerase | |  |  |  |  |  | | --- | --- | --- | --- | --- | |  |  |  |  |  | | EC:5.1.3.1 | | | | | | |
|  |  | RAFL09-15-M18 | At3g03250 / UDP-glucose pyrophosphorylase -related | |  |  |  |  |  | | --- | --- | --- | --- | --- | |  |  |  |  |  | | EC:2.7.7.9 | | | | | | |
|  |  | RAFL11-02-F16 | At3g01850 / D-ribulose-5-phosphate 3-epimerase -related | |  |  |  |  |  | | --- | --- | --- | --- | --- | |  |  |  |  |  | | EC:5.1.3.1 | | | | | | |
| Phenylalanine, tyrosine and tryptophan biosynthesis | | |  |  | A | B | C | D | P | P' | N |
|  | Cluster:3-1 | |  |  | 5 | 211 | 23 | 4424 | 0.00835746 | 0.108646974 | 13 |
|  |  | RAFL05-13-G12 | At1g07780 / phosphoribosylanthranilate isomerase (PAI1) | |  |  |  |  |  | | --- | --- | --- | --- | --- | |  |  |  |  |  | | EC:5.3.1.24 | | | | | | |
|  |  | RAFL04-13-O10 | At2g29560 / enolase (2-phospho-D-glycerate hydroylase) -related | |  |  |  |  |  | | --- | --- | --- | --- | --- | |  |  |  |  |  | | EC:4.2.1.11 | | | | | | |
|  |  | RAFL05-16-L22 | At5g48220 / indole-3-glycerol phosphate synthase (IGPS), putative | |  |  |  |  |  | | --- | --- | --- | --- | --- | |  |  |  |  |  | | EC:4.1.1.48 | | | | | | |
|  |  | RAFL04-10-L08 | At4g34200 / D-3-phosphoglycerate dehydrogenase (3-PGDH), putative | |  |  |  |  |  | | --- | --- | --- | --- | --- | |  |  |  |  |  | | EC:2.6.1.9 | | | | | | |
|  |  | RAFL09-09-E22 | At4g39280 / phenylalanyl-trna synthetase - like protein | |  |  |  |  |  | | --- | --- | --- | --- | --- | |  |  |  |  |  | | EC:6.1.1.20 | | | | | | |
| Ascorbate and aldarate metabolism | | |  |  | A | B | C | D | P | P' | N |
|  | Cluster:9-0 | |  |  | 4 | 28 | 6 | 4625 | 3.7289774E-7 | 2.6102844E-6 | 7 |
|  |  | RAFL04-09-D07 | At1g54100 / aldehyde dehydrogenase, putative (ALDH) | |  |  |  |  |  | | --- | --- | --- | --- | --- | |  |  |  |  |  | | EC:1.2.1.3 | | | | | | |
|  |  | RAFL05-21-E06 | At1g54100 / aldehyde dehydrogenase, putative (ALDH) | |  |  |  |  |  | | --- | --- | --- | --- | --- | |  |  |  |  |  | | EC:1.2.1.3 | | | | | | |
|  |  | RAFL08-15-L09 | At1g54100 / aldehyde dehydrogenase, putative (ALDH) | |  |  |  |  |  | | --- | --- | --- | --- | --- | |  |  |  |  |  | | EC:1.2.1.3 | | | | | | |
|  |  | RAFL08-09-C23 | At1g54100 / aldehyde dehydrogenase, putative (ALDH) | |  |  |  |  |  | | --- | --- | --- | --- | --- | |  |  |  |  |  | | EC:1.2.1.3 | | | | | | |
| 1,2-Dichloroethane degradation | | |  |  | A | B | C | D | P | P' | N |
|  | Cluster:9-0 | |  |  | 4 | 28 | 2 | 4629 | 2.7153972E-8 | 8.146192E-8 | 3 |
|  |  | RAFL04-09-D07 | At1g54100 / aldehyde dehydrogenase, putative (ALDH) | |  |  |  |  |  | | --- | --- | --- | --- | --- | |  |  |  |  |  | | EC:1.2.1.3 | | | | | | |
|  |  | RAFL05-21-E06 | At1g54100 / aldehyde dehydrogenase, putative (ALDH) | |  |  |  |  |  | | --- | --- | --- | --- | --- | |  |  |  |  |  | | EC:1.2.1.3 | | | | | | |
|  |  | RAFL08-15-L09 | At1g54100 / aldehyde dehydrogenase, putative (ALDH) | |  |  |  |  |  | | --- | --- | --- | --- | --- | |  |  |  |  |  | | EC:1.2.1.3 | | | | | | |
|  |  | RAFL08-09-C23 | At1g54100 / aldehyde dehydrogenase, putative (ALDH) | |  |  |  |  |  | | --- | --- | --- | --- | --- | |  |  |  |  |  | | EC:1.2.1.3 | | | | | | |
| Indole and ipecac alkaloid biosynthesis | | |  |  | A | B | C | D | P | P' | N |
|  | Cluster:9-1 | |  |  | 1 | 95 | 0 | 4567 | 0.020587604 | 0.020587604 | 1 |
|  |  | RAFL05-09-P03 | At1g74020 / strictosidine synthase family | |  |  |  |  |  | | --- | --- | --- | --- | --- | |  |  |  |  |  | | EC:4.3.3.2 | | | | | | |
| Glutathione metabolism | | |  |  | A | B | C | D | P | P' | N |
|  | Cluster:2-0 | |  |  | 5 | 145 | 23 | 4490 | 0.0017399205 | 0.033058487 | 19 |
|  |  | RAFL09-11-A18 | At1g65930 / isocitrate dehydrogenase (NADP+), putative | |  |  |  |  |  | | --- | --- | --- | --- | --- | |  |  |  |  |  | | EC:1.1.1.42 | | | | | | |
|  |  | RAFL03-05-I07 | At4g02520 / glutathione transferase, putative | |  |  |  |  |  | | --- | --- | --- | --- | --- | |  |  |  |  |  | | EC:2.5.1.18 | | | | | | |
|  |  | RAFL09-07-F20 | At1g65930 / isocitrate dehydrogenase (NADP+), putative | |  |  |  |  |  | | --- | --- | --- | --- | --- | |  |  |  |  |  | | EC:1.1.1.42 | | | | | | |
|  |  | RAFL09-06-L20 | At1g65930 / isocitrate dehydrogenase (NADP+), putative | |  |  |  |  |  | | --- | --- | --- | --- | --- | |  |  |  |  |  | | EC:1.1.1.42 | | | | | | |
|  |  | RAFL03-05-B08 | At2g30860 / glutathione transferase, putative | |  |  |  |  |  | | --- | --- | --- | --- | --- | |  |  |  |  |  | | EC:2.5.1.18 | | | | | | |
| Streptomycin biosynthesis | | |  |  | A | B | C | D | P | P' | N |
|  | Cluster:3-2 | |  |  | 2 | 35 | 12 | 4614 | 0.0052507473 | 0.04200598 | 8 |
|  |  | RAFL09-16-F08 | At3g23820 / NAD-dependent epimerase/dehydratase family | |  |  |  |  |  | | --- | --- | --- | --- | --- | |  |  |  |  |  | | EC:4.2.1.46 | | | | | | |
|  |  | RAFL09-07-D12 | At3g23820 / NAD-dependent epimerase/dehydratase family | |  |  |  |  |  | | --- | --- | --- | --- | --- | |  |  |  |  |  | | EC:4.2.1.46 | | | | | | |
|  | Cluster:2-2 | |  |  | 2 | 51 | 12 | 4598 | 0.010570335 | 0.08456268 | 8 |
|  |  | RAFL09-15-K07 | At4g39800 / myo-inositol-1-phosphate synthase | |  |  |  |  |  | | --- | --- | --- | --- | --- | |  |  |  |  |  | | EC:5.5.1.4 | | | | | | |
|  |  | RAFL09-14-L01 | At4g39800 / myo-inositol-1-phosphate synthase | |  |  |  |  |  | | --- | --- | --- | --- | --- | |  |  |  |  |  | | EC:5.5.1.4 | | | | | | |
| Alanine and aspartate metabolism | | |  |  | A | B | C | D | P | P' | N |
|  | Cluster:2-1 | |  |  | 4 | 240 | 22 | 4397 | 0.04413268 | 0.7061229 | 16 |
|  |  | RAFL09-09-I19 | At1g23310 / alanine aminotransferase -related | |  |  |  |  |  | | --- | --- | --- | --- | --- | |  |  |  |  |  | | EC:2.6.1.2 | | | | | | |
|  |  | RAFL05-07-N11 | At1g70580 / alanine aminotransferase, putative | |  |  |  |  |  | | --- | --- | --- | --- | --- | |  |  |  |  |  | | EC:2.6.1.2 | | | | | | |
|  |  | RAFL04-17-H07 | At3g57610 / adenylosuccinate synthetase | |  |  |  |  |  | | --- | --- | --- | --- | --- | |  |  |  |  |  | | EC:6.3.4.4 | | | | | | |
|  |  | RAFL09-16-K01 | At1g23310 / alanine aminotransferase -related | |  |  |  |  |  | | --- | --- | --- | --- | --- | |  |  |  |  |  | | EC:2.6.1.2 | | | | | | |
| One carbon pool by folate | | |  |  | A | B | C | D | P | P' | N |
|  | Cluster:0-1 | |  |  | 2 | 104 | 5 | 4552 | 0.0099780755 | 0.049890377 | 5 |
|  |  | RAFL06-13-H16 | At1g11860 / aminomethyltransferase-related precursor protein | |  |  |  |  |  | | --- | --- | --- | --- | --- | |  |  |  |  |  | | EC:2.1.2.10 | | | | | | |
|  |  | RAFL05-04-O06 | At4g37930 / glycine hydroxymethyltransferase like protein | |  |  |  |  |  | | --- | --- | --- | --- | --- | |  |  |  |  |  | | EC:2.1.2.1 | | | | | | |
|  | Cluster:1-2 | |  |  | 2 | 172 | 5 | 4484 | 0.025693169 | 0.12846585 | 5 |
|  |  | RAFL09-11-K06 | At4g13930 / hydroxymethyltransferase | |  |  |  |  |  | | --- | --- | --- | --- | --- | |  |  |  |  |  | | EC:2.1.2.1 | | | | | | |
|  |  | RAFL09-16-M15 | At1g11860 / aminomethyltransferase-related precursor protein | |  |  |  |  |  | | --- | --- | --- | --- | --- | |  |  |  |  |  | | EC:2.1.2.10 | | | | | | |
| Alkaloid biosynthesis I | | |  |  | A | B | C | D | P | P' | N |
|  | Cluster:8-0 | |  |  | 2 | 107 | 7 | 4547 | 0.017509907 | 0.10505944 | 6 |
|  |  | RAFL05-09-B02 | At5g53970 / aminotransferase, putative | |  |  |  |  |  | | --- | --- | --- | --- | --- | |  |  |  |  |  | | EC:2.6.1.5 | | | | | | |
|  |  | RAFL07-10-M07 | At5g53970 / aminotransferase, putative | |  |  |  |  |  | | --- | --- | --- | --- | --- | |  |  |  |  |  | | EC:2.6.1.5 | | | | | | |
|  | Cluster:6-1 | |  |  | 3 | 312 | 6 | 4342 | 0.018891884 | 0.1133513 | 6 |
|  |  | RAFL04-17-L05 | At5g19550 / aspartate aminotransferase, cytoplasmic isozyme 1 (transaminase A/Asp2) | |  |  |  |  |  | | --- | --- | --- | --- | --- | |  |  |  |  |  | | EC:2.6.1.1 | | | | | | |
|  |  | RAFL06-11-D08 | At5g36160 / tyrosine aminotransferase-related protein | |  |  |  |  |  | | --- | --- | --- | --- | --- | |  |  |  |  |  | | EC:2.6.1.5 | | | | | | |
|  |  | RAFL11-09-A12 | At5g19550 / aspartate aminotransferase, cytoplasmic isozyme 1 (transaminase A/Asp2) | |  |  |  |  |  | | --- | --- | --- | --- | --- | |  |  |  |  |  | | EC:2.6.1.1 | | | | | | |
| Starch and sucrose metabolism | | |  |  | A | B | C | D | P | P' | N |
|  | Cluster:3-2 | |  |  | 4 | 33 | 54 | 4572 | 0.0010484793 | 0.023066545 | 22 |
|  |  | RAFL08-10-H13 | At5g07830 / glycosyl hydrolase family 79 (endo-beta-glucuronidase/heparanase) | |  |  |  |  |  | | --- | --- | --- | --- | --- | |  |  |  |  |  | | EC:3.2.1.31 | | | | | | |
|  |  | RAFL07-12-E11 | At3g02230 / reversibly glycosylated polypeptide-1 | |  |  |  |  |  | | --- | --- | --- | --- | --- | |  |  |  |  |  | | EC:2.4.1.12 | | | | | | |
|  |  | RAFL05-16-L21 | At4g15210 / glycosyl hydrolase family 14 (beta-amylase) | |  |  |  |  |  | | --- | --- | --- | --- | --- | |  |  |  |  |  | | EC:3.2.1.2 | | | | | | |
|  |  | RAFL04-14-G14 | At1g26560 / glycosyl hydrolase family 1 | |  |  |  |  |  | | --- | --- | --- | --- | --- | |  |  |  |  |  | | EC:3.2.1.21 | | | | | | |
|  | Cluster:5-2 | |  |  | 5 | 121 | 53 | 4484 | 0.019437116 | 0.42761654 | 22 |
|  |  | RAFL09-14-L23 | At5g11720 / glycosyl hydrolase family 31 | |  |  |  |  |  | | --- | --- | --- | --- | --- | |  |  |  |  |  | | EC:3.2.1.20 | | | | | | |
|  |  | RAFL05-21-E11 | At5g64740 / cellulose synthase, catalytic subunit, putative | |  |  |  |  |  | | --- | --- | --- | --- | --- | |  |  |  |  |  | | EC:2.4.1.12 | | | | | | |
|  |  | RAFL07-09-M15 | At3g29360 / UDP-glucose dehydrogenase -related | |  |  |  |  |  | | --- | --- | --- | --- | --- | |  |  |  |  |  | | EC:1.1.1.22 | | | | | | |
|  |  | RAFL05-03-E09 | At5g64860 / glycosyl hydrolase family 77 (4-alpha-glucanotransferase) | |  |  |  |  |  | | --- | --- | --- | --- | --- | |  |  |  |  |  | | EC:2.4.1.25 | | | | | | |
|  |  | RAFL05-18-N23 | At2g35650 / glycosyltransferase family 2 | |  |  |  |  |  | | --- | --- | --- | --- | --- | |  |  |  |  |  | | EC:2.4.1.12 | | | | | | |
|  | Cluster:2-2 | |  |  | 3 | 50 | 55 | 4555 | 0.027576564 | 0.6066844 | 22 |
|  |  | RAFL06-10-O06 | At1g53840 / pectinesterase family | |  |  |  |  |  | | --- | --- | --- | --- | --- | |  |  |  |  |  | | EC:3.1.1.11 | | | | | | |
|  |  | RAFL06-16-M17 | At4g17090 / glycosyl hydrolase family 14 (beta-amylase) | |  |  |  |  |  | | --- | --- | --- | --- | --- | |  |  |  |  |  | | EC:3.2.1.2 | | | | | | |
|  |  | RAFL05-07-J12 | At1g66430 / pfkB type carbohydrate kinase protein family | |  |  |  |  |  | | --- | --- | --- | --- | --- | |  |  |  |  |  | | EC:2.7.1.4 | | | | | | |
|  | Cluster:10-2 | |  |  | 4 | 105 | 54 | 4500 | 0.04569159 | 1.0052149 | 22 |
|  |  | RAFL08-15-K01 | At1g62660 / glycosyl hydrolase family 32 | |  |  |  |  |  | | --- | --- | --- | --- | --- | |  |  |  |  |  | | EC:3.2.1.26 | | | | | | |
|  |  | RAFL08-13-K06 | At1g62660 / glycosyl hydrolase family 32 | |  |  |  |  |  | | --- | --- | --- | --- | --- | |  |  |  |  |  | | EC:3.2.1.26 | | | | | | |
|  |  | RAFL07-14-D12 | At4g12430 / trehalose-6-phosphate phosphatase, putative | |  |  |  |  |  | | --- | --- | --- | --- | --- | |  |  |  |  |  | | EC:3.1.3.12 | | | | | | |
|  |  | RAFL05-07-J05 | At3g43190 / sucrose synthase (UDP-glucose-fructose glucosyltransferase/sucrose-UDP glucosyltransferase), putative | |  |  |  |  |  | | --- | --- | --- | --- | --- | |  |  |  |  |  | | EC:2.4.1.13 | | | | | | |
| Nucleotide sugars metabolism | | |  |  | A | B | C | D | P | P' | N |
|  | Cluster:3-2 | |  |  | 2 | 35 | 20 | 4606 | 0.012808084 | 0.17931317 | 14 |
|  |  | RAFL09-16-F08 | At3g23820 / NAD-dependent epimerase/dehydratase family | |  |  |  |  |  | | --- | --- | --- | --- | --- | |  |  |  |  |  | | EC:4.2.1.46 ,EC:5.1.3.2 | | | | | | |
|  |  | RAFL09-07-D12 | At3g23820 / NAD-dependent epimerase/dehydratase family | |  |  |  |  |  | | --- | --- | --- | --- | --- | |  |  |  |  |  | | EC:4.2.1.46 ,EC:5.1.3.2 | | | | | | |
|  | Cluster:5-2 | |  |  | 3 | 123 | 19 | 4518 | 0.020386456 | 0.28541037 | 14 |
|  |  | RAFL07-11-C21 | At4g30440 / nucleotide sugar epimerase family | |  |  |  |  |  | | --- | --- | --- | --- | --- | |  |  |  |  |  | | EC:4.2.1.46 ,EC:5.1.3.2 | | | | | | |
|  |  | RAFL04-09-G05 | At1g50450 / expressed protein | |  |  |  |  |  | | --- | --- | --- | --- | --- | |  |  |  |  |  | | EC:4.2.1.46 | | | | | | |
|  |  | RAFL07-09-M15 | At3g29360 / UDP-glucose dehydrogenase -related | |  |  |  |  |  | | --- | --- | --- | --- | --- | |  |  |  |  |  | | EC:1.1.1.22 | | | | | | |
| Glycine, serine and threonine metabolism | | |  |  | A | B | C | D | P | P' | N |
|  | Cluster:0-2 | |  |  | 8 | 71 | 34 | 4550 | 3.512669E-7 | 5.6202703E-6 | 16 |
|  |  | RAFL09-09-C13 | At4g33010 / glycine dehydrogenase (decarboxylating) (glycine decarboxylase/glycine cleavage system P-protein), putative | |  |  |  |  |  | | --- | --- | --- | --- | --- | |  |  |  |  |  | | EC:1.4.4.2 | | | | | | |
|  |  | RAFL09-06-E16 | At2g26080 / glycine dehydrogenase (decarboxylating) (glycine decarboxylase/glycine cleavage system P-protein), putative | |  |  |  |  |  | | --- | --- | --- | --- | --- | |  |  |  |  |  | | EC:1.4.4.2 | | | | | | |
|  |  | RAFL07-18-E18 | At4g33010 / glycine dehydrogenase (decarboxylating) (glycine decarboxylase/glycine cleavage system P-protein), putative | |  |  |  |  |  | | --- | --- | --- | --- | --- | |  |  |  |  |  | | EC:1.4.4.2 | | | | | | |
|  |  | RAFL08-11-O04 | At4g33010 / glycine dehydrogenase (decarboxylating) (glycine decarboxylase/glycine cleavage system P-protein), putative | |  |  |  |  |  | | --- | --- | --- | --- | --- | |  |  |  |  |  | | EC:1.4.4.2 | | | | | | |
|  |  | RAFL11-06-P03 | At4g33010 / glycine dehydrogenase (decarboxylating) (glycine decarboxylase/glycine cleavage system P-protein), putative | |  |  |  |  |  | | --- | --- | --- | --- | --- | |  |  |  |  |  | | EC:1.4.4.2 | | | | | | |
|  |  | RAFL06-13-I09 | At3g01120 / cystathionine gamma-synthase -related | |  |  |  |  |  | | --- | --- | --- | --- | --- | |  |  |  |  |  | | EC:2.5.1.48 | | | | | | |
|  |  | RAFL07-10-O06 | At4g33010 / glycine dehydrogenase (decarboxylating) (glycine decarboxylase/glycine cleavage system P-protein), putative | |  |  |  |  |  | | --- | --- | --- | --- | --- | |  |  |  |  |  | | EC:1.4.4.2 | | | | | | |
|  |  | RAFL11-06-F06 | At4g33010 / glycine dehydrogenase (decarboxylating) (glycine decarboxylase/glycine cleavage system P-protein), putative | |  |  |  |  |  | | --- | --- | --- | --- | --- | |  |  |  |  |  | | EC:1.4.4.2 | | | | | | |
| Bile acid biosynthesis | | |  |  | A | B | C | D | P | P' | N |
|  | Cluster:9-0 | |  |  | 4 | 28 | 5 | 4626 | 2.2481943E-7 | 1.3489166E-6 | 6 |
|  |  | RAFL04-09-D07 | At1g54100 / aldehyde dehydrogenase, putative (ALDH) | |  |  |  |  |  | | --- | --- | --- | --- | --- | |  |  |  |  |  | | EC:1.2.1.3 | | | | | | |
|  |  | RAFL05-21-E06 | At1g54100 / aldehyde dehydrogenase, putative (ALDH) | |  |  |  |  |  | | --- | --- | --- | --- | --- | |  |  |  |  |  | | EC:1.2.1.3 | | | | | | |
|  |  | RAFL08-15-L09 | At1g54100 / aldehyde dehydrogenase, putative (ALDH) | |  |  |  |  |  | | --- | --- | --- | --- | --- | |  |  |  |  |  | | EC:1.2.1.3 | | | | | | |
|  |  | RAFL08-09-C23 | At1g54100 / aldehyde dehydrogenase, putative (ALDH) | |  |  |  |  |  | | --- | --- | --- | --- | --- | |  |  |  |  |  | | EC:1.2.1.3 | | | | | | |
| Porphyrin and chlorophyll metabolism | | |  |  | A | B | C | D | P | P' | N |
|  | Cluster:3-2 | |  |  | 5 | 32 | 16 | 4610 | 4.413796E-7 | 4.8551756E-6 | 11 |
|  |  | RAFL08-10-H13 | At5g07830 / glycosyl hydrolase family 79 (endo-beta-glucuronidase/heparanase) | |  |  |  |  |  | | --- | --- | --- | --- | --- | |  |  |  |  |  | | EC:3.2.1.31 | | | | | | |
|  |  | RAFL09-06-N12 | At3g23810 / S-adenosyl-L-homocysteinas -related | |  |  |  |  |  | | --- | --- | --- | --- | --- | |  |  |  |  |  | | EC:4.3.1.8 | | | | | | |
|  |  | RAFL09-13-P13 | At3g23810 / S-adenosyl-L-homocysteinas -related | |  |  |  |  |  | | --- | --- | --- | --- | --- | |  |  |  |  |  | | EC:4.3.1.8 | | | | | | |
|  |  | RAFL07-09-L01 | At3g23810 / S-adenosyl-L-homocysteinas -related | |  |  |  |  |  | | --- | --- | --- | --- | --- | |  |  |  |  |  | | EC:4.3.1.8 | | | | | | |
|  |  | RAFL09-10-M18 | At3g23810 / S-adenosyl-L-homocysteinas -related | |  |  |  |  |  | | --- | --- | --- | --- | --- | |  |  |  |  |  | | EC:4.3.1.8 | | | | | | |
| Prostaglandin and leukotriene metabolism | | |  |  | A | B | C | D | P | P' | N |
|  | Cluster:2-2 | |  |  | 2 | 51 | 1 | 4609 | 3.7755756E-4 | 7.551151E-4 | 2 |
|  |  | RAFL05-12-G03 | At5g42650 / allene oxide synthase / cytochrome P450 74A | |  |  |  |  |  | | --- | --- | --- | --- | --- | |  |  |  |  |  | | EC:4.2.1.92 | | | | | | |
|  |  | RAFL06-10-H13 | At5g42650 / allene oxide synthase / cytochrome P450 74A | |  |  |  |  |  | | --- | --- | --- | --- | --- | |  |  |  |  |  | | EC:4.2.1.92 | | | | | | |
| Clavulanic acid biosynthesis | | |  |  | A | B | C | D | P | P' | N |
|  | Cluster:1-0 | |  |  | 1 | 148 | 0 | 4514 | 0.031953678 | 0.031953678 | 1 |
|  |  | RAFL05-18-H22 | At4g08870 / arginase -related | |  |  |  |  |  | | --- | --- | --- | --- | --- | |  |  |  |  |  | | EC:3.5.3.11 | | | | | | |
| Tryptophan metabolism | | |  |  | A | B | C | D | P | P' | N |
|  | Cluster:9-0 | |  |  | 4 | 28 | 20 | 4611 | 1.7637874E-5 | 2.2929236E-4 | 13 |
|  |  | RAFL04-09-D07 | At1g54100 / aldehyde dehydrogenase, putative (ALDH) | |  |  |  |  |  | | --- | --- | --- | --- | --- | |  |  |  |  |  | | EC:1.2.1.3 | | | | | | |
|  |  | RAFL05-21-E06 | At1g54100 / aldehyde dehydrogenase, putative (ALDH) | |  |  |  |  |  | | --- | --- | --- | --- | --- | |  |  |  |  |  | | EC:1.2.1.3 | | | | | | |
|  |  | RAFL08-15-L09 | At1g54100 / aldehyde dehydrogenase, putative (ALDH) | |  |  |  |  |  | | --- | --- | --- | --- | --- | |  |  |  |  |  | | EC:1.2.1.3 | | | | | | |
|  |  | RAFL08-09-C23 | At1g54100 / aldehyde dehydrogenase, putative (ALDH) | |  |  |  |  |  | | --- | --- | --- | --- | --- | |  |  |  |  |  | | EC:1.2.1.3 | | | | | | |
|  | Cluster:8-0 | |  |  | 4 | 105 | 20 | 4534 | 0.0020926541 | 0.027204502 | 13 |
|  |  | RAFL03-05-E06 | At3g48000 / mitochondrial aldehyde dehydrogenase, putative (ALDH) | |  |  |  |  |  | | --- | --- | --- | --- | --- | |  |  |  |  |  | | EC:1.2.1.3 | | | | | | |
|  |  | RAFL06-13-H12 | At3g51840 / acyl-coA dehydrogenase | |  |  |  |  |  | | --- | --- | --- | --- | --- | |  |  |  |  |  | | EC:1.3.99.7 | | | | | | |
|  |  | RAFL06-13-E03 | At3g44300 / nitrilase 2 | |  |  |  |  |  | | --- | --- | --- | --- | --- | |  |  |  |  |  | | EC:3.5.5.1 | | | | | | |
|  |  | RAFL08-10-H06 | At3g44300 / nitrilase 2 | |  |  |  |  |  | | --- | --- | --- | --- | --- | |  |  |  |  |  | | EC:3.5.5.1 | | | | | | |
|  | Cluster:6-0 | |  |  | 3 | 137 | 21 | 4502 | 0.033808403 | 0.4395092 | 13 |
|  |  | RAFL07-09-I01 | At5g65750 / 2-oxoglutarate dehydrogenase, E1 component | |  |  |  |  |  | | --- | --- | --- | --- | --- | |  |  |  |  |  | | EC:1.2.4.2 | | | | | | |
|  |  | RAFL09-13-G24 | At1g20620 / catalase 3 | |  |  |  |  |  | | --- | --- | --- | --- | --- | |  |  |  |  |  | | EC:1.11.1.6 | | | | | | |
|  |  | RAFL05-12-I22 | At3g44310 / nitrilase 1 | |  |  |  |  |  | | --- | --- | --- | --- | --- | |  |  |  |  |  | | EC:3.5.5.1 | | | | | | |
| Methionine metabolism | | |  |  | A | B | C | D | P | P' | N |
|  | Cluster:3-2 | |  |  | 4 | 33 | 19 | 4607 | 2.668534E-5 | 2.1348272E-4 | 8 |
|  |  | RAFL09-06-N12 | At3g23810 / S-adenosyl-L-homocysteinas -related | |  |  |  |  |  | | --- | --- | --- | --- | --- | |  |  |  |  |  | | EC:3.3.1.1 | | | | | | |
|  |  | RAFL09-13-P13 | At3g23810 / S-adenosyl-L-homocysteinas -related | |  |  |  |  |  | | --- | --- | --- | --- | --- | |  |  |  |  |  | | EC:3.3.1.1 | | | | | | |
|  |  | RAFL07-09-L01 | At3g23810 / S-adenosyl-L-homocysteinas -related | |  |  |  |  |  | | --- | --- | --- | --- | --- | |  |  |  |  |  | | EC:3.3.1.1 | | | | | | |
|  |  | RAFL09-10-M18 | At3g23810 / S-adenosyl-L-homocysteinas -related | |  |  |  |  |  | | --- | --- | --- | --- | --- | |  |  |  |  |  | | EC:3.3.1.1 | | | | | | |
|  | Cluster:1-2 | |  |  | 6 | 168 | 17 | 4472 | 1.4723212E-4 | 0.0011778569 | 8 |
|  |  | RAFL09-11-C22 | At5g17920 / 5-methyltetrahydropteroyltriglutamate--homocysteine S-methyltransferase | |  |  |  |  |  | | --- | --- | --- | --- | --- | |  |  |  |  |  | | EC:2.1.1.14 | | | | | | |
|  |  | RAFL07-08-E09 | At5g49030 / isoleucyl-tRNA synthetase | |  |  |  |  |  | | --- | --- | --- | --- | --- | |  |  |  |  |  | | EC:6.1.1.10 | | | | | | |
|  |  | RAFL06-12-D05 | At5g17920 / 5-methyltetrahydropteroyltriglutamate--homocysteine S-methyltransferase | |  |  |  |  |  | | --- | --- | --- | --- | --- | |  |  |  |  |  | | EC:2.1.1.14 | | | | | | |
|  |  | RAFL11-01-K15 | At5g17920 / 5-methyltetrahydropteroyltriglutamate--homocysteine S-methyltransferase | |  |  |  |  |  | | --- | --- | --- | --- | --- | |  |  |  |  |  | | EC:2.1.1.14 | | | | | | |
|  |  | RAFL09-10-C09 | At5g17920 / 5-methyltetrahydropteroyltriglutamate--homocysteine S-methyltransferase | |  |  |  |  |  | | --- | --- | --- | --- | --- | |  |  |  |  |  | | EC:2.1.1.14 | | | | | | |
|  |  | RAFL11-06-L17 | At5g17920 / 5-methyltetrahydropteroyltriglutamate--homocysteine S-methyltransferase | |  |  |  |  |  | | --- | --- | --- | --- | --- | |  |  |  |  |  | | EC:2.1.1.14 | | | | | | |
|  | Cluster:0-2 | |  |  | 3 | 76 | 20 | 4564 | 0.0064938474 | 0.05195078 | 8 |
|  |  | RAFL09-09-A21 | At5g17920 / 5-methyltetrahydropteroyltriglutamate--homocysteine S-methyltransferase | |  |  |  |  |  | | --- | --- | --- | --- | --- | |  |  |  |  |  | | EC:2.1.1.14 | | | | | | |
|  |  | RAFL06-13-I09 | At3g01120 / cystathionine gamma-synthase -related | |  |  |  |  |  | | --- | --- | --- | --- | --- | |  |  |  |  |  | | EC:2.5.1.48 | | | | | | |
|  |  | RAFL08-16-E05 | At5g17920 / 5-methyltetrahydropteroyltriglutamate--homocysteine S-methyltransferase | |  |  |  |  |  | | --- | --- | --- | --- | --- | |  |  |  |  |  | | EC:2.1.1.14 | | | | | | |
| Butanoate metabolism | | |  |  | A | B | C | D | P | P' | N |
|  | Cluster:9-0 | |  |  | 5 | 27 | 28 | 4603 | 2.2763622E-6 | 3.6421796E-5 | 16 |
|  |  | RAFL05-08-B14 | At2g38400 / alanine--glyoxylate aminotransferase (beta-alanine-pyruvate aminotransferase/AGT), putative | |  |  |  |  |  | | --- | --- | --- | --- | --- | |  |  |  |  |  | | EC:2.6.1.19 | | | | | | |
|  |  | RAFL04-09-D07 | At1g54100 / aldehyde dehydrogenase, putative (ALDH) | |  |  |  |  |  | | --- | --- | --- | --- | --- | |  |  |  |  |  | | EC:1.2.1.3 | | | | | | |
|  |  | RAFL05-21-E06 | At1g54100 / aldehyde dehydrogenase, putative (ALDH) | |  |  |  |  |  | | --- | --- | --- | --- | --- | |  |  |  |  |  | | EC:1.2.1.3 | | | | | | |
|  |  | RAFL08-15-L09 | At1g54100 / aldehyde dehydrogenase, putative (ALDH) | |  |  |  |  |  | | --- | --- | --- | --- | --- | |  |  |  |  |  | | EC:1.2.1.3 | | | | | | |
|  |  | RAFL08-09-C23 | At1g54100 / aldehyde dehydrogenase, putative (ALDH) | |  |  |  |  |  | | --- | --- | --- | --- | --- | |  |  |  |  |  | | EC:1.2.1.3 | | | | | | |
|  | Cluster:10-2 | |  |  | 3 | 106 | 30 | 4524 | 0.040794313 | 0.652709 | 16 |
|  |  | RAFL08-15-A08 | At4g34710 / arginine decarboxylase SPE2 | |  |  |  |  |  | | --- | --- | --- | --- | --- | |  |  |  |  |  | | EC:4.1.1.19 | | | | | | |
|  |  | RAFL04-13-O07 | At3g22200 / 4-aminobutyrate aminotransferase (gamma-amino-N-butyrate transaminase/GABA transaminase/beta-alanine--oxoglutarate aminotransferase) | |  |  |  |  |  | | --- | --- | --- | --- | --- | |  |  |  |  |  | | EC:2.6.1.19 | | | | | | |
|  |  | RAFL06-10-M04 | At2g26800 / hydroxymethylglutaryl-CoA lyase -related | |  |  |  |  |  | | --- | --- | --- | --- | --- | |  |  |  |  |  | | EC:4.1.3.4 | | | | | | |
| Sphingophospholipid biosynthesis | | |  |  | A | B | C | D | P | P' | N |
|  | Cluster:5-2 | |  |  | 1 | 125 | 0 | 4537 | 0.027021231 | 0.027021231 | 1 |
|  |  | RAFL05-21-D15 | At1g13560 / aminoalcoholphosphotransferase | |  |  |  |  |  | | --- | --- | --- | --- | --- | |  |  |  |  |  | | EC:2.7.8.1 | | | | | | |
| Citrate cycle (TCA cycle) | | |  |  | A | B | C | D | P | P' | N |
|  | Cluster:3-0 | |  |  | 6 | 227 | 30 | 4400 | 0.008043733 | 0.13674346 | 17 |
|  |  | RAFL07-14-B18 | At2g47510 / fumarase -related | |  |  |  |  |  | | --- | --- | --- | --- | --- | |  |  |  |  |  | | EC:4.2.1.2 | | | | | | |
|  |  | RAFL05-21-P13 | At2g42600 / phosphoenolpyruvate carboxylase | |  |  |  |  |  | | --- | --- | --- | --- | --- | |  |  |  |  |  | | EC:4.1.1.49 ,EC:4.1.1.32 | | | | | | |
|  |  | RAFL06-07-J21 | At1g04410 / malate dehydrogenase, cytosolic, putative | |  |  |  |  |  | | --- | --- | --- | --- | --- | |  |  |  |  |  | | EC:1.1.1.37 | | | | | | |
|  |  | RAFL07-17-M04 | At1g04410 / malate dehydrogenase, cytosolic, putative | |  |  |  |  |  | | --- | --- | --- | --- | --- | |  |  |  |  |  | | EC:1.1.1.37 | | | | | | |
|  |  | RAFL09-09-M02 | At3g47520 / malate dehydrogenase (NAD), chloroplast, putative | |  |  |  |  |  | | --- | --- | --- | --- | --- | |  |  |  |  |  | | EC:1.1.1.37 | | | | | | |
|  |  | RAFL05-15-O22 | At2g20420 / succinyl-CoA ligase beta subunit | |  |  |  |  |  | | --- | --- | --- | --- | --- | |  |  |  |  |  | | EC:6.2.1.4 | | | | | | |
| Histidine metabolism | | |  |  | A | B | C | D | P | P' | N |
|  | Cluster:9-0 | |  |  | 4 | 28 | 12 | 4619 | 3.139684E-6 | 3.4536522E-5 | 11 |
|  |  | RAFL04-09-D07 | At1g54100 / aldehyde dehydrogenase, putative (ALDH) | |  |  |  |  |  | | --- | --- | --- | --- | --- | |  |  |  |  |  | | EC:1.2.1.3 | | | | | | |
|  |  | RAFL05-21-E06 | At1g54100 / aldehyde dehydrogenase, putative (ALDH) | |  |  |  |  |  | | --- | --- | --- | --- | --- | |  |  |  |  |  | | EC:1.2.1.3 | | | | | | |
|  |  | RAFL08-15-L09 | At1g54100 / aldehyde dehydrogenase, putative (ALDH) | |  |  |  |  |  | | --- | --- | --- | --- | --- | |  |  |  |  |  | | EC:1.2.1.3 | | | | | | |
|  |  | RAFL08-09-C23 | At1g54100 / aldehyde dehydrogenase, putative (ALDH) | |  |  |  |  |  | | --- | --- | --- | --- | --- | |  |  |  |  |  | | EC:1.2.1.3 | | | | | | |
|  | Cluster:8-0 | |  |  | 3 | 106 | 13 | 4541 | 0.0055737486 | 0.061311234 | 11 |
|  |  | RAFL03-05-E06 | At3g48000 / mitochondrial aldehyde dehydrogenase, putative (ALDH) | |  |  |  |  |  | | --- | --- | --- | --- | --- | |  |  |  |  |  | | EC:1.2.1.3 | | | | | | |
|  |  | RAFL05-09-B02 | At5g53970 / aminotransferase, putative | |  |  |  |  |  | | --- | --- | --- | --- | --- | |  |  |  |  |  | | EC:2.6.1.9 | | | | | | |
|  |  | RAFL07-10-M07 | At5g53970 / aminotransferase, putative | |  |  |  |  |  | | --- | --- | --- | --- | --- | |  |  |  |  |  | | EC:2.6.1.9 | | | | | | |
| Glycerolipid metabolism | | |  |  | A | B | C | D | P | P' | N |
|  | Cluster:9-0 | |  |  | 4 | 28 | 36 | 4595 | 1.404465E-4 | 0.0029493764 | 21 |
|  |  | RAFL04-09-D07 | At1g54100 / aldehyde dehydrogenase, putative (ALDH) | |  |  |  |  |  | | --- | --- | --- | --- | --- | |  |  |  |  |  | | EC:1.2.1.3 | | | | | | |
|  |  | RAFL05-21-E06 | At1g54100 / aldehyde dehydrogenase, putative (ALDH) | |  |  |  |  |  | | --- | --- | --- | --- | --- | |  |  |  |  |  | | EC:1.2.1.3 | | | | | | |
|  |  | RAFL08-15-L09 | At1g54100 / aldehyde dehydrogenase, putative (ALDH) | |  |  |  |  |  | | --- | --- | --- | --- | --- | |  |  |  |  |  | | EC:1.2.1.3 | | | | | | |
|  |  | RAFL08-09-C23 | At1g54100 / aldehyde dehydrogenase, putative (ALDH) | |  |  |  |  |  | | --- | --- | --- | --- | --- | |  |  |  |  |  | | EC:1.2.1.3 | | | | | | |
|  | Cluster:9-1 | |  |  | 3 | 93 | 37 | 4530 | 0.048277758 | 1.0138329 | 21 |
|  |  | RAFL07-16-P10 | At1g77120 / alcohol dehydrogenase (ADH) | |  |  |  |  |  | | --- | --- | --- | --- | --- | |  |  |  |  |  | | EC:1.1.1.1 | | | | | | |
|  |  | RAFL05-18-O21 | At2g30550 / lipase (class 3) family | |  |  |  |  |  | | --- | --- | --- | --- | --- | |  |  |  |  |  | | EC:3.1.1.3 | | | | | | |
|  |  | RAFL08-09-J19 | At1g02660 / lipase (class 3) family | |  |  |  |  |  | | --- | --- | --- | --- | --- | |  |  |  |  |  | | EC:3.1.1.3 | | | | | | |
| Caprolactam degradation | | |  |  | A | B | C | D | P | P' | N |
|  | Cluster:2-2 | |  |  | 1 | 52 | 0 | 4610 | 0.011366073 | 0.011366073 | 1 |
|  |  | RAFL04-09-D16 | At5g24420 / 6-phosphogluconolactonase-related protein | |  |  |  |  |  | | --- | --- | --- | --- | --- | |  |  |  |  |  | | EC:3.1.1.17 | | | | | | |
| Fatty acid metabolism | | |  |  | A | B | C | D | P | P' | N |
|  | Cluster:9-0 | |  |  | 4 | 28 | 19 | 4612 | 1.4769203E-5 | 2.2153804E-4 | 15 |
|  |  | RAFL04-09-D07 | At1g54100 / aldehyde dehydrogenase, putative (ALDH) | |  |  |  |  |  | | --- | --- | --- | --- | --- | |  |  |  |  |  | | EC:1.2.1.3 | | | | | | |
|  |  | RAFL05-21-E06 | At1g54100 / aldehyde dehydrogenase, putative (ALDH) | |  |  |  |  |  | | --- | --- | --- | --- | --- | |  |  |  |  |  | | EC:1.2.1.3 | | | | | | |
|  |  | RAFL08-15-L09 | At1g54100 / aldehyde dehydrogenase, putative (ALDH) | |  |  |  |  |  | | --- | --- | --- | --- | --- | |  |  |  |  |  | | EC:1.2.1.3 | | | | | | |
|  |  | RAFL08-09-C23 | At1g54100 / aldehyde dehydrogenase, putative (ALDH) | |  |  |  |  |  | | --- | --- | --- | --- | --- | |  |  |  |  |  | | EC:1.2.1.3 | | | | | | |
| Carbon fixation | | |  |  | A | B | C | D | P | P' | N |
|  | Cluster:0-0 | |  |  | 15 | 21 | 66 | 4561 | 4.2700217E-18 | 8.113041E-17 | 19 |
|  |  | RAFL09-16-C21 | At5g38410 / ribulose bisphosphate carboxylase small chain 3b precursor (RuBisCO small subunit 3b) (sp|P10798) | |  |  |  |  |  | | --- | --- | --- | --- | --- | |  |  |  |  |  | | EC:4.1.1.39 | | | | | | |
|  |  | RAFL09-09-K05 | At5g38410 / ribulose bisphosphate carboxylase small chain 3b precursor (RuBisCO small subunit 3b) (sp|P10798) | |  |  |  |  |  | | --- | --- | --- | --- | --- | |  |  |  |  |  | | EC:4.1.1.39 | | | | | | |
|  |  | RAFL11-03-H09 | At1g67090 / ribulose-bisphosphate carboxylase small unit -related | |  |  |  |  |  | | --- | --- | --- | --- | --- | |  |  |  |  |  | | EC:4.1.1.39 | | | | | | |
|  |  | RAFL06-10-O15 | At5g38420 / ribulose bisphosphate carboxylase small chain 2b precursor (RuBisCO small subunit 2b) (sp|P10797) | |  |  |  |  |  | | --- | --- | --- | --- | --- | |  |  |  |  |  | | EC:4.1.1.39 | | | | | | |
|  |  | RAFL04-15-J15 | At5g38410 / ribulose bisphosphate carboxylase small chain 3b precursor (RuBisCO small subunit 3b) (sp|P10798) | |  |  |  |  |  | | --- | --- | --- | --- | --- | |  |  |  |  |  | | EC:4.1.1.39 | | | | | | |
|  |  | RAFL08-17-J10 | At5g38420 / ribulose bisphosphate carboxylase small chain 2b precursor (RuBisCO small subunit 2b) (sp|P10797) | |  |  |  |  |  | | --- | --- | --- | --- | --- | |  |  |  |  |  | | EC:4.1.1.39 | | | | | | |
|  |  | RAFL06-07-I02 | At1g67090 / ribulose-bisphosphate carboxylase small unit -related | |  |  |  |  |  | | --- | --- | --- | --- | --- | |  |  |  |  |  | | EC:4.1.1.39 | | | | | | |
|  |  | RAFL09-09-L07 | At1g67090 / ribulose-bisphosphate carboxylase small unit -related | |  |  |  |  |  | | --- | --- | --- | --- | --- | |  |  |  |  |  | | EC:4.1.1.39 | | | | | | |
|  |  | RAFL07-14-L17 | At5g38420 / ribulose bisphosphate carboxylase small chain 2b precursor (RuBisCO small subunit 2b) (sp|P10797) | |  |  |  |  |  | | --- | --- | --- | --- | --- | |  |  |  |  |  | | EC:4.1.1.39 | | | | | | |
|  |  | RAFL06-13-H11 | At5g38430 / ribulose bisphosphate carboxylase small chain 1b precursor (RuBisCO small subunit 1b) (sp|P10796) | |  |  |  |  |  | | --- | --- | --- | --- | --- | |  |  |  |  |  | | EC:4.1.1.39 | | | | | | |
|  |  | RAFL06-14-L16 | At5g38430 / ribulose bisphosphate carboxylase small chain 1b precursor (RuBisCO small subunit 1b) (sp|P10796) | |  |  |  |  |  | | --- | --- | --- | --- | --- | |  |  |  |  |  | | EC:4.1.1.39 | | | | | | |
|  |  | RAFL07-11-L12 | At5g38420 / ribulose bisphosphate carboxylase small chain 2b precursor (RuBisCO small subunit 2b) (sp|P10797) | |  |  |  |  |  | | --- | --- | --- | --- | --- | |  |  |  |  |  | | EC:4.1.1.39 | | | | | | |
|  |  | RAFL06-14-C14 | At1g67090 / ribulose-bisphosphate carboxylase small unit -related | |  |  |  |  |  | | --- | --- | --- | --- | --- | |  |  |  |  |  | | EC:4.1.1.39 | | | | | | |
|  |  | RAFL06-14-C19 | At5g38410 / ribulose bisphosphate carboxylase small chain 3b precursor (RuBisCO small subunit 3b) (sp|P10798) | |  |  |  |  |  | | --- | --- | --- | --- | --- | |  |  |  |  |  | | EC:4.1.1.39 | | | | | | |
|  |  | RAFL06-08-L09 | At5g38420 / ribulose bisphosphate carboxylase small chain 2b precursor (RuBisCO small subunit 2b) (sp|P10797) | |  |  |  |  |  | | --- | --- | --- | --- | --- | |  |  |  |  |  | | EC:4.1.1.39 | | | | | | |
|  | Cluster:0-1 | |  |  | 21 | 85 | 60 | 4497 | 1.8197178E-17 | 3.4574636E-16 | 19 |
|  |  | RAFL11-02-L02 | At1g67090 / ribulose-bisphosphate carboxylase small unit -related | |  |  |  |  |  | | --- | --- | --- | --- | --- | |  |  |  |  |  | | EC:4.1.1.39 | | | | | | |
|  |  | RAFL07-07-I23 | At1g56190 / phosphoglycerate kinase -related | |  |  |  |  |  | | --- | --- | --- | --- | --- | |  |  |  |  |  | | EC:2.7.2.3 | | | | | | |
|  |  | RAFL07-18-C20 | At2g21330 / fructose-bisphosphate aldolase, putative | |  |  |  |  |  | | --- | --- | --- | --- | --- | |  |  |  |  |  | | EC:4.1.2.13 | | | | | | |
|  |  | RAFL04-19-O21 | At4g38970 / fructose-bisphosphate aldolase, putative | |  |  |  |  |  | | --- | --- | --- | --- | --- | |  |  |  |  |  | | EC:4.1.2.13 | | | | | | |
|  |  | RAFL09-17-N23 | At2g21330 / fructose-bisphosphate aldolase, putative | |  |  |  |  |  | | --- | --- | --- | --- | --- | |  |  |  |  |  | | EC:4.1.2.13 | | | | | | |
|  |  | RAFL07-14-L16 | At3g12780 / phosphoglycerate kinase -related | |  |  |  |  |  | | --- | --- | --- | --- | --- | |  |  |  |  |  | | EC:2.7.2.3 | | | | | | |
|  |  | RAFL09-15-L04 | At3g12780 / phosphoglycerate kinase -related | |  |  |  |  |  | | --- | --- | --- | --- | --- | |  |  |  |  |  | | EC:2.7.2.3 | | | | | | |
|  |  | RAFL04-19-M17 | At1g32060 / phosphoribulokinase precursor | |  |  |  |  |  | | --- | --- | --- | --- | --- | |  |  |  |  |  | | EC:2.7.1.19 | | | | | | |
|  |  | RAFL03-06-F08 | At1g67090 / ribulose-bisphosphate carboxylase small unit -related | |  |  |  |  |  | | --- | --- | --- | --- | --- | |  |  |  |  |  | | EC:4.1.1.39 | | | | | | |
|  |  | RAFL09-18-L22 | At3g12780 / phosphoglycerate kinase -related | |  |  |  |  |  | | --- | --- | --- | --- | --- | |  |  |  |  |  | | EC:2.7.2.3 | | | | | | |
|  |  | RAFL07-12-M09 | At2g21330 / fructose-bisphosphate aldolase, putative | |  |  |  |  |  | | --- | --- | --- | --- | --- | |  |  |  |  |  | | EC:4.1.2.13 | | | | | | |
|  |  | RAFL08-18-C10 | At2g21330 / fructose-bisphosphate aldolase, putative | |  |  |  |  |  | | --- | --- | --- | --- | --- | |  |  |  |  |  | | EC:4.1.2.13 | | | | | | |
|  |  | RAFL04-13-J02 | At3g54050 / fructose-bisphosphatase precursor | |  |  |  |  |  | | --- | --- | --- | --- | --- | |  |  |  |  |  | | EC:3.1.3.11 | | | | | | |
|  |  | RAFL09-06-K21 | At1g67090 / ribulose-bisphosphate carboxylase small unit -related | |  |  |  |  |  | | --- | --- | --- | --- | --- | |  |  |  |  |  | | EC:4.1.1.39 | | | | | | |
|  |  | RAFL07-12-E12 | At2g21330 / fructose-bisphosphate aldolase, putative | |  |  |  |  |  | | --- | --- | --- | --- | --- | |  |  |  |  |  | | EC:4.1.2.13 | | | | | | |
|  |  | RAFL07-18-J01 | At2g21330 / fructose-bisphosphate aldolase, putative | |  |  |  |  |  | | --- | --- | --- | --- | --- | |  |  |  |  |  | | EC:4.1.2.13 | | | | | | |
|  |  | RAFL11-07-D01 | At5g38410 / ribulose bisphosphate carboxylase small chain 3b precursor (RuBisCO small subunit 3b) (sp|P10798) | |  |  |  |  |  | | --- | --- | --- | --- | --- | |  |  |  |  |  | | EC:4.1.1.39 | | | | | | |
|  |  | RAFL04-10-J07 | At3g04790 / ribose 5-phosphate isomerase -related | |  |  |  |  |  | | --- | --- | --- | --- | --- | |  |  |  |  |  | | EC:5.3.1.6 | | | | | | |
|  |  | RAFL09-06-P15 | At1g67090 / ribulose-bisphosphate carboxylase small unit -related | |  |  |  |  |  | | --- | --- | --- | --- | --- | |  |  |  |  |  | | EC:4.1.1.39 | | | | | | |
|  |  | RAFL05-12-O19 | At3g55800 / sedoheptulose-bisphosphatase precursor | |  |  |  |  |  | | --- | --- | --- | --- | --- | |  |  |  |  |  | | EC:3.1.3.37 | | | | | | |
|  |  | RAFL07-16-P05 | At3g12780 / phosphoglycerate kinase -related | |  |  |  |  |  | | --- | --- | --- | --- | --- | |  |  |  |  |  | | EC:2.7.2.3 | | | | | | |
|  | Cluster:3-0 | |  |  | 9 | 224 | 72 | 4358 | 0.018764498 | 0.35652548 | 19 |
|  |  | RAFL05-21-O08 | At5g61410 / ribulose-5-phosphate-3-epimerase | |  |  |  |  |  | | --- | --- | --- | --- | --- | |  |  |  |  |  | | EC:5.1.3.1 | | | | | | |
|  |  | RAFL05-21-P13 | At2g42600 / phosphoenolpyruvate carboxylase | |  |  |  |  |  | | --- | --- | --- | --- | --- | |  |  |  |  |  | | EC:4.1.1.49 ,EC:4.1.1.31 | | | | | | |
|  |  | RAFL06-11-B16 | At3g52930 / fructose-bisphosphate aldolase, putative | |  |  |  |  |  | | --- | --- | --- | --- | --- | |  |  |  |  |  | | EC:4.1.2.13 | | | | | | |
|  |  | RAFL04-17-F02 | At2g01140 / fructose-bisphosphate aldolase, putative | |  |  |  |  |  | | --- | --- | --- | --- | --- | |  |  |  |  |  | | EC:4.1.2.13 | | | | | | |
|  |  | RAFL11-02-F16 | At3g01850 / D-ribulose-5-phosphate 3-epimerase -related | |  |  |  |  |  | | --- | --- | --- | --- | --- | |  |  |  |  |  | | EC:5.1.3.1 | | | | | | |
|  |  | RAFL06-07-J21 | At1g04410 / malate dehydrogenase, cytosolic, putative | |  |  |  |  |  | | --- | --- | --- | --- | --- | |  |  |  |  |  | | EC:1.1.1.37 | | | | | | |
|  |  | RAFL09-07-B08 | At2g30970 / aspartate aminotransferase, mitochondrial (transaminase A/Asp1) | |  |  |  |  |  | | --- | --- | --- | --- | --- | |  |  |  |  |  | | EC:2.6.1.1 | | | | | | |
|  |  | RAFL07-17-M04 | At1g04410 / malate dehydrogenase, cytosolic, putative | |  |  |  |  |  | | --- | --- | --- | --- | --- | |  |  |  |  |  | | EC:1.1.1.37 | | | | | | |
|  |  | RAFL09-09-M02 | At3g47520 / malate dehydrogenase (NAD), chloroplast, putative | |  |  |  |  |  | | --- | --- | --- | --- | --- | |  |  |  |  |  | | EC:1.1.1.37 | | | | | | |
| Riboflavin metabolism | | |  |  | A | B | C | D | P | P' | N |
|  | Cluster:1-1 | |  |  | 1 | 104 | 1 | 4557 | 0.04453306 | 0.08906612 | 2 |
|  |  | RAFL08-12-F18 | At2g44050 / 6,7-dimethyl-8-ribityllumazine synthase precursor | |  |  |  |  |  | | --- | --- | --- | --- | --- | |  |  |  |  |  | | EC:2.5.1.9 | | | | | | |
| Reductive carboxylate cycle (CO2 fixation) | | |  |  | A | B | C | D | P | P' | N |
|  | Cluster:3-0 | |  |  | 5 | 228 | 21 | 4409 | 0.0082831625 | 0.12424744 | 15 |
|  |  | RAFL07-14-B18 | At2g47510 / fumarase -related | |  |  |  |  |  | | --- | --- | --- | --- | --- | |  |  |  |  |  | | EC:4.2.1.2 | | | | | | |
|  |  | RAFL05-21-P13 | At2g42600 / phosphoenolpyruvate carboxylase | |  |  |  |  |  | | --- | --- | --- | --- | --- | |  |  |  |  |  | | EC:4.1.1.31 | | | | | | |
|  |  | RAFL06-07-J21 | At1g04410 / malate dehydrogenase, cytosolic, putative | |  |  |  |  |  | | --- | --- | --- | --- | --- | |  |  |  |  |  | | EC:1.1.1.37 | | | | | | |
|  |  | RAFL07-17-M04 | At1g04410 / malate dehydrogenase, cytosolic, putative | |  |  |  |  |  | | --- | --- | --- | --- | --- | |  |  |  |  |  | | EC:1.1.1.37 | | | | | | |
|  |  | RAFL09-09-M02 | At3g47520 / malate dehydrogenase (NAD), chloroplast, putative | |  |  |  |  |  | | --- | --- | --- | --- | --- | |  |  |  |  |  | | EC:1.1.1.37 | | | | | | |
|  | Cluster:2-0 | |  |  | 3 | 147 | 23 | 4490 | 0.04939301 | 0.74089515 | 15 |
|  |  | RAFL09-11-A18 | At1g65930 / isocitrate dehydrogenase (NADP+), putative | |  |  |  |  |  | | --- | --- | --- | --- | --- | |  |  |  |  |  | | EC:1.1.1.42 | | | | | | |
|  |  | RAFL09-07-F20 | At1g65930 / isocitrate dehydrogenase (NADP+), putative | |  |  |  |  |  | | --- | --- | --- | --- | --- | |  |  |  |  |  | | EC:1.1.1.42 | | | | | | |
|  |  | RAFL09-06-L20 | At1g65930 / isocitrate dehydrogenase (NADP+), putative | |  |  |  |  |  | | --- | --- | --- | --- | --- | |  |  |  |  |  | | EC:1.1.1.42 | | | | | | |
| Pyrimidine metabolism | | |  |  | A | B | C | D | P | P' | N |
|  | Cluster:2-1 | |  |  | 3 | 241 | 12 | 4407 | 0.040360164 | 0.40360165 | 10 |
|  |  | RAFL04-18-P17 | At5g35170 / adenylate kinase -related protein | |  |  |  |  |  | | --- | --- | --- | --- | --- | |  |  |  |  |  | | EC:2.7.4.9 | | | | | | |
|  |  | RAFL04-12-O11 | At3g27740 / carbamoyl-phosphate synthase (glutamine-hydrolyzing) (glutamine-dependent carbamoyl-phosphate synthase) small subunit | |  |  |  |  |  | | --- | --- | --- | --- | --- | |  |  |  |  |  | | EC:6.3.5.5 | | | | | | |
|  |  | RAFL04-13-M20 | At4g11010 / nucleoside diphosphate kinase 3 (ndpk3) | |  |  |  |  |  | | --- | --- | --- | --- | --- | |  |  |  |  |  | | EC:2.7.4.6 | | | | | | |
| Cysteine metabolism | | |  |  | A | B | C | D | P | P' | N |
|  | Cluster:9-1 | |  |  | 3 | 93 | 11 | 4556 | 0.0026107915 | 0.026107915 | 10 |
|  |  | RAFL05-19-H07 | At5g11520 / aspartate aminotransferase, chloroplast (transaminase A/Asp3) | |  |  |  |  |  | | --- | --- | --- | --- | --- | |  |  |  |  |  | | EC:2.6.1.1 | | | | | | |
|  |  | RAFL11-02-N11 | At1g64660 / methionine/cystathionine gamma lyase -related | |  |  |  |  |  | | --- | --- | --- | --- | --- | |  |  |  |  |  | | EC:4.4.1.8 | | | | | | |
|  |  | RAFL05-18-H15 | At1g64660 / methionine/cystathionine gamma lyase -related | |  |  |  |  |  | | --- | --- | --- | --- | --- | |  |  |  |  |  | | EC:4.4.1.8 | | | | | | |
| beta-Alanine metabolism | | |  |  | A | B | C | D | P | P' | N |
|  | Cluster:9-0 | |  |  | 5 | 27 | 17 | 4614 | 2.664087E-7 | 3.1969046E-6 | 12 |
|  |  | RAFL05-08-B14 | At2g38400 / alanine--glyoxylate aminotransferase (beta-alanine-pyruvate aminotransferase/AGT), putative | |  |  |  |  |  | | --- | --- | --- | --- | --- | |  |  |  |  |  | | EC:2.6.1.19 | | | | | | |
|  |  | RAFL04-09-D07 | At1g54100 / aldehyde dehydrogenase, putative (ALDH) | |  |  |  |  |  | | --- | --- | --- | --- | --- | |  |  |  |  |  | | EC:1.2.1.3 | | | | | | |
|  |  | RAFL05-21-E06 | At1g54100 / aldehyde dehydrogenase, putative (ALDH) | |  |  |  |  |  | | --- | --- | --- | --- | --- | |  |  |  |  |  | | EC:1.2.1.3 | | | | | | |
|  |  | RAFL08-15-L09 | At1g54100 / aldehyde dehydrogenase, putative (ALDH) | |  |  |  |  |  | | --- | --- | --- | --- | --- | |  |  |  |  |  | | EC:1.2.1.3 | | | | | | |
|  |  | RAFL08-09-C23 | At1g54100 / aldehyde dehydrogenase, putative (ALDH) | |  |  |  |  |  | | --- | --- | --- | --- | --- | |  |  |  |  |  | | EC:1.2.1.3 | | | | | | |
| Flavonoids, stilbene and lignin biosynthesis | | |  |  | A | B | C | D | P | P' | N |
|  | Cluster:9-0 | |  |  | 3 | 29 | 33 | 4598 | 0.0017977278 | 0.03415683 | 19 |
|  |  | RAFL06-15-H16 | At1g09500 / cinnamyl-alcohol dehydrogenase (CAD) family | |  |  |  |  |  | | --- | --- | --- | --- | --- | |  |  |  |  |  | | EC:1.1.1.195 | | | | | | |
|  |  | RAFL05-18-A06 | At1g09500 / cinnamyl-alcohol dehydrogenase (CAD) family | |  |  |  |  |  | | --- | --- | --- | --- | --- | |  |  |  |  |  | | EC:1.1.1.195 | | | | | | |
|  |  | RAFL05-14-E15 | At2g33590 / cinnamoyl-CoA reductase family | |  |  |  |  |  | | --- | --- | --- | --- | --- | |  |  |  |  |  | | EC:1.2.1.44 | | | | | | |
|  | Cluster:9-1 | |  |  | 4 | 92 | 32 | 4535 | 0.0060000676 | 0.11400128 | 19 |
|  |  | RAFL04-20-P19 | At3g49110 / peroxidase | |  |  |  |  |  | | --- | --- | --- | --- | --- | |  |  |  |  |  | | EC:1.11.1.7 | | | | | | |
|  |  | RAFL04-13-E17 | At5g20230 / plastocyanin-like domain containing protein | |  |  |  |  |  | | --- | --- | --- | --- | --- | |  |  |  |  |  | | EC:2.1.1.68 | | | | | | |
|  |  | RAFL05-12-N20 | At4g30470 / cinnamoyl-CoA reductase-related | |  |  |  |  |  | | --- | --- | --- | --- | --- | |  |  |  |  |  | | EC:1.2.1.44 | | | | | | |
|  |  | RAFL09-07-G15 | At3g49120 / peroxidase, putative | |  |  |  |  |  | | --- | --- | --- | --- | --- | |  |  |  |  |  | | EC:1.11.1.7 | | | | | | |
| Thiamine metabolism | | |  |  | A | B | C | D | P | P' | N |
|  | Cluster:8-0 | |  |  | 1 | 108 | 1 | 4553 | 0.0462095 | 0.092419 | 2 |
|  |  | RAFL07-08-G02 | At3g04080 / apyrase (Atapy1) | |  |  |  |  |  | | --- | --- | --- | --- | --- | |  |  |  |  |  | | EC:3.6.1.15 | | | | | | |
| Lysine degradation | | |  |  | A | B | C | D | P | P' | N |
|  | Cluster:9-0 | |  |  | 4 | 28 | 12 | 4619 | 3.139684E-6 | 3.4536522E-5 | 11 |
|  |  | RAFL04-09-D07 | At1g54100 / aldehyde dehydrogenase, putative (ALDH) | |  |  |  |  |  | | --- | --- | --- | --- | --- | |  |  |  |  |  | | EC:1.2.1.3 | | | | | | |
|  |  | RAFL05-21-E06 | At1g54100 / aldehyde dehydrogenase, putative (ALDH) | |  |  |  |  |  | | --- | --- | --- | --- | --- | |  |  |  |  |  | | EC:1.2.1.3 | | | | | | |
|  |  | RAFL08-15-L09 | At1g54100 / aldehyde dehydrogenase, putative (ALDH) | |  |  |  |  |  | | --- | --- | --- | --- | --- | |  |  |  |  |  | | EC:1.2.1.3 | | | | | | |
|  |  | RAFL08-09-C23 | At1g54100 / aldehyde dehydrogenase, putative (ALDH) | |  |  |  |  |  | | --- | --- | --- | --- | --- | |  |  |  |  |  | | EC:1.2.1.3 | | | | | | |
|  | | | | | | | | | | | |
| Cluster:9-2 | | |  |  | A | B | C | D | P | P' | N |
|  | Peptidoglycan biosynthesis | |  |  | 2 | 65 | 2 | 4594 | 0.0011979077 | 0.0035937233 | 3 |
|  |  | RAFL08-17-D17 | At3g53180 / nodulin / glutamate-ammonia ligase - like protein | |  |  |  |  |  | | --- | --- | --- | --- | --- | |  |  |  |  |  | | EC:6.3.1.2 | | | | | | |
|  |  | RAFL06-09-F14 | At3g53180 / nodulin / glutamate-ammonia ligase - like protein | |  |  |  |  |  | | --- | --- | --- | --- | --- | |  |  |  |  |  | | EC:6.3.1.2 | | | | | | |
|  | Glutamate metabolism | |  |  | 4 | 63 | 43 | 4553 | 0.004365906 | 0.08731812 | 20 |
|  |  | RAFL08-11-N01 | At4g34710 / arginine decarboxylase SPE2 | |  |  |  |  |  | | --- | --- | --- | --- | --- | |  |  |  |  |  | | EC:4.1.1.19 | | | | | | |
|  |  | RAFL08-17-D17 | At3g53180 / nodulin / glutamate-ammonia ligase - like protein | |  |  |  |  |  | | --- | --- | --- | --- | --- | |  |  |  |  |  | | EC:6.3.1.2 | | | | | | |
|  |  | RAFL09-13-D07 | At4g34710 / arginine decarboxylase SPE2 | |  |  |  |  |  | | --- | --- | --- | --- | --- | |  |  |  |  |  | | EC:4.1.1.19 | | | | | | |
|  |  | RAFL06-09-F14 | At3g53180 / nodulin / glutamate-ammonia ligase - like protein | |  |  |  |  |  | | --- | --- | --- | --- | --- | |  |  |  |  |  | | EC:6.3.1.2 | | | | | | |
| Cluster:6-0 | | |  |  | A | B | C | D | P | P' | N |
|  | Methane metabolism | |  |  | 3 | 137 | 20 | 4503 | 0.030230032 | 0.42322046 | 14 |
|  |  | RAFL09-13-G24 | At1g20620 / catalase 3 | |  |  |  |  |  | | --- | --- | --- | --- | --- | |  |  |  |  |  | | EC:1.11.1.6 | | | | | | |
|  |  | RAFL06-08-C18 | At4g37520 / peroxidase, putative | |  |  |  |  |  | | --- | --- | --- | --- | --- | |  |  |  |  |  | | EC:1.11.1.7 | | | | | | |
|  |  | RAFL05-18-P09 | At1g71695 / peroxidase, putative | |  |  |  |  |  | | --- | --- | --- | --- | --- | |  |  |  |  |  | | EC:1.11.1.7 | | | | | | |
|  | Tryptophan metabolism | |  |  | 3 | 137 | 21 | 4502 | 0.033808403 | 0.4395092 | 13 |
|  |  | RAFL07-09-I01 | At5g65750 / 2-oxoglutarate dehydrogenase, E1 component | |  |  |  |  |  | | --- | --- | --- | --- | --- | |  |  |  |  |  | | EC:1.2.4.2 | | | | | | |
|  |  | RAFL09-13-G24 | At1g20620 / catalase 3 | |  |  |  |  |  | | --- | --- | --- | --- | --- | |  |  |  |  |  | | EC:1.11.1.6 | | | | | | |
|  |  | RAFL05-12-I22 | At3g44310 / nitrilase 1 | |  |  |  |  |  | | --- | --- | --- | --- | --- | |  |  |  |  |  | | EC:3.5.5.1 | | | | | | |
|  | Ubiquinone biosynthesis | |  |  | 2 | 138 | 9 | 4514 | 0.04121896 | 0.16487584 | 4 |
|  |  | RAFL05-17-L16 | At1g79010 / NADH dehydrogenase -related | |  |  |  |  |  | | --- | --- | --- | --- | --- | |  |  |  |  |  | | EC:1.6.5.3 | | | | | | |
|  |  | RAFL08-12-A05 | At5g11770 / NADH dehydrogenase (ubiquinone) | |  |  |  |  |  | | --- | --- | --- | --- | --- | |  |  |  |  |  | | EC:1.6.5.3 | | | | | | |
| Cluster:0-0 | | |  |  | A | B | C | D | P | P' | N |
|  | Glyoxylate and dicarboxylate metabolism | |  |  | 15 | 21 | 34 | 4593 | 9.474795E-22 | 1.5159671E-20 | 16 |
|  |  | RAFL09-16-C21 | At5g38410 / ribulose bisphosphate carboxylase small chain 3b precursor (RuBisCO small subunit 3b) (sp|P10798) | |  |  |  |  |  | | --- | --- | --- | --- | --- | |  |  |  |  |  | | EC:4.1.1.39 | | | | | | |
|  |  | RAFL09-09-K05 | At5g38410 / ribulose bisphosphate carboxylase small chain 3b precursor (RuBisCO small subunit 3b) (sp|P10798) | |  |  |  |  |  | | --- | --- | --- | --- | --- | |  |  |  |  |  | | EC:4.1.1.39 | | | | | | |
|  |  | RAFL11-03-H09 | At1g67090 / ribulose-bisphosphate carboxylase small unit -related | |  |  |  |  |  | | --- | --- | --- | --- | --- | |  |  |  |  |  | | EC:4.1.1.39 | | | | | | |
|  |  | RAFL06-10-O15 | At5g38420 / ribulose bisphosphate carboxylase small chain 2b precursor (RuBisCO small subunit 2b) (sp|P10797) | |  |  |  |  |  | | --- | --- | --- | --- | --- | |  |  |  |  |  | | EC:4.1.1.39 | | | | | | |
|  |  | RAFL04-15-J15 | At5g38410 / ribulose bisphosphate carboxylase small chain 3b precursor (RuBisCO small subunit 3b) (sp|P10798) | |  |  |  |  |  | | --- | --- | --- | --- | --- | |  |  |  |  |  | | EC:4.1.1.39 | | | | | | |
|  |  | RAFL08-17-J10 | At5g38420 / ribulose bisphosphate carboxylase small chain 2b precursor (RuBisCO small subunit 2b) (sp|P10797) | |  |  |  |  |  | | --- | --- | --- | --- | --- | |  |  |  |  |  | | EC:4.1.1.39 | | | | | | |
|  |  | RAFL06-07-I02 | At1g67090 / ribulose-bisphosphate carboxylase small unit -related | |  |  |  |  |  | | --- | --- | --- | --- | --- | |  |  |  |  |  | | EC:4.1.1.39 | | | | | | |
|  |  | RAFL09-09-L07 | At1g67090 / ribulose-bisphosphate carboxylase small unit -related | |  |  |  |  |  | | --- | --- | --- | --- | --- | |  |  |  |  |  | | EC:4.1.1.39 | | | | | | |
|  |  | RAFL07-14-L17 | At5g38420 / ribulose bisphosphate carboxylase small chain 2b precursor (RuBisCO small subunit 2b) (sp|P10797) | |  |  |  |  |  | | --- | --- | --- | --- | --- | |  |  |  |  |  | | EC:4.1.1.39 | | | | | | |
|  |  | RAFL06-13-H11 | At5g38430 / ribulose bisphosphate carboxylase small chain 1b precursor (RuBisCO small subunit 1b) (sp|P10796) | |  |  |  |  |  | | --- | --- | --- | --- | --- | |  |  |  |  |  | | EC:4.1.1.39 | | | | | | |
|  |  | RAFL06-14-L16 | At5g38430 / ribulose bisphosphate carboxylase small chain 1b precursor (RuBisCO small subunit 1b) (sp|P10796) | |  |  |  |  |  | | --- | --- | --- | --- | --- | |  |  |  |  |  | | EC:4.1.1.39 | | | | | | |
|  |  | RAFL07-11-L12 | At5g38420 / ribulose bisphosphate carboxylase small chain 2b precursor (RuBisCO small subunit 2b) (sp|P10797) | |  |  |  |  |  | | --- | --- | --- | --- | --- | |  |  |  |  |  | | EC:4.1.1.39 | | | | | | |
|  |  | RAFL06-14-C14 | At1g67090 / ribulose-bisphosphate carboxylase small unit -related | |  |  |  |  |  | | --- | --- | --- | --- | --- | |  |  |  |  |  | | EC:4.1.1.39 | | | | | | |
|  |  | RAFL06-14-C19 | At5g38410 / ribulose bisphosphate carboxylase small chain 3b precursor (RuBisCO small subunit 3b) (sp|P10798) | |  |  |  |  |  | | --- | --- | --- | --- | --- | |  |  |  |  |  | | EC:4.1.1.39 | | | | | | |
|  |  | RAFL06-08-L09 | At5g38420 / ribulose bisphosphate carboxylase small chain 2b precursor (RuBisCO small subunit 2b) (sp|P10797) | |  |  |  |  |  | | --- | --- | --- | --- | --- | |  |  |  |  |  | | EC:4.1.1.39 | | | | | | |
|  | Carbon fixation | |  |  | 15 | 21 | 66 | 4561 | 4.2700217E-18 | 8.113041E-17 | 19 |
|  |  | RAFL09-16-C21 | At5g38410 / ribulose bisphosphate carboxylase small chain 3b precursor (RuBisCO small subunit 3b) (sp|P10798) | |  |  |  |  |  | | --- | --- | --- | --- | --- | |  |  |  |  |  | | EC:4.1.1.39 | | | | | | |
|  |  | RAFL09-09-K05 | At5g38410 / ribulose bisphosphate carboxylase small chain 3b precursor (RuBisCO small subunit 3b) (sp|P10798) | |  |  |  |  |  | | --- | --- | --- | --- | --- | |  |  |  |  |  | | EC:4.1.1.39 | | | | | | |
|  |  | RAFL11-03-H09 | At1g67090 / ribulose-bisphosphate carboxylase small unit -related | |  |  |  |  |  | | --- | --- | --- | --- | --- | |  |  |  |  |  | | EC:4.1.1.39 | | | | | | |
|  |  | RAFL06-10-O15 | At5g38420 / ribulose bisphosphate carboxylase small chain 2b precursor (RuBisCO small subunit 2b) (sp|P10797) | |  |  |  |  |  | | --- | --- | --- | --- | --- | |  |  |  |  |  | | EC:4.1.1.39 | | | | | | |
|  |  | RAFL04-15-J15 | At5g38410 / ribulose bisphosphate carboxylase small chain 3b precursor (RuBisCO small subunit 3b) (sp|P10798) | |  |  |  |  |  | | --- | --- | --- | --- | --- | |  |  |  |  |  | | EC:4.1.1.39 | | | | | | |
|  |  | RAFL08-17-J10 | At5g38420 / ribulose bisphosphate carboxylase small chain 2b precursor (RuBisCO small subunit 2b) (sp|P10797) | |  |  |  |  |  | | --- | --- | --- | --- | --- | |  |  |  |  |  | | EC:4.1.1.39 | | | | | | |
|  |  | RAFL06-07-I02 | At1g67090 / ribulose-bisphosphate carboxylase small unit -related | |  |  |  |  |  | | --- | --- | --- | --- | --- | |  |  |  |  |  | | EC:4.1.1.39 | | | | | | |
|  |  | RAFL09-09-L07 | At1g67090 / ribulose-bisphosphate carboxylase small unit -related | |  |  |  |  |  | | --- | --- | --- | --- | --- | |  |  |  |  |  | | EC:4.1.1.39 | | | | | | |
|  |  | RAFL07-14-L17 | At5g38420 / ribulose bisphosphate carboxylase small chain 2b precursor (RuBisCO small subunit 2b) (sp|P10797) | |  |  |  |  |  | | --- | --- | --- | --- | --- | |  |  |  |  |  | | EC:4.1.1.39 | | | | | | |
|  |  | RAFL06-13-H11 | At5g38430 / ribulose bisphosphate carboxylase small chain 1b precursor (RuBisCO small subunit 1b) (sp|P10796) | |  |  |  |  |  | | --- | --- | --- | --- | --- | |  |  |  |  |  | | EC:4.1.1.39 | | | | | | |
|  |  | RAFL06-14-L16 | At5g38430 / ribulose bisphosphate carboxylase small chain 1b precursor (RuBisCO small subunit 1b) (sp|P10796) | |  |  |  |  |  | | --- | --- | --- | --- | --- | |  |  |  |  |  | | EC:4.1.1.39 | | | | | | |
|  |  | RAFL07-11-L12 | At5g38420 / ribulose bisphosphate carboxylase small chain 2b precursor (RuBisCO small subunit 2b) (sp|P10797) | |  |  |  |  |  | | --- | --- | --- | --- | --- | |  |  |  |  |  | | EC:4.1.1.39 | | | | | | |
|  |  | RAFL06-14-C14 | At1g67090 / ribulose-bisphosphate carboxylase small unit -related | |  |  |  |  |  | | --- | --- | --- | --- | --- | |  |  |  |  |  | | EC:4.1.1.39 | | | | | | |
|  |  | RAFL06-14-C19 | At5g38410 / ribulose bisphosphate carboxylase small chain 3b precursor (RuBisCO small subunit 3b) (sp|P10798) | |  |  |  |  |  | | --- | --- | --- | --- | --- | |  |  |  |  |  | | EC:4.1.1.39 | | | | | | |
|  |  | RAFL06-08-L09 | At5g38420 / ribulose bisphosphate carboxylase small chain 2b precursor (RuBisCO small subunit 2b) (sp|P10797) | |  |  |  |  |  | | --- | --- | --- | --- | --- | |  |  |  |  |  | | EC:4.1.1.39 | | | | | | |
|  | Nitrogen metabolism | |  |  | 2 | 34 | 40 | 4587 | 0.04113549 | 0.90498084 | 22 |
|  |  | RAFL06-13-B01 | At3g01500 / carbonic anhydrase, chloroplast precursor | |  |  |  |  |  | | --- | --- | --- | --- | --- | |  |  |  |  |  | | EC:4.2.1.1 | | | | | | |
|  |  | RAFL06-11-K17 | At3g01500 / carbonic anhydrase, chloroplast precursor | |  |  |  |  |  | | --- | --- | --- | --- | --- | |  |  |  |  |  | | EC:4.2.1.1 | | | | | | |
| Cluster:0-1 | | |  |  | A | B | C | D | P | P' | N |
|  | Carbon fixation | |  |  | 21 | 85 | 60 | 4497 | 1.8197178E-17 | 3.4574636E-16 | 19 |
|  |  | RAFL11-02-L02 | At1g67090 / ribulose-bisphosphate carboxylase small unit -related | |  |  |  |  |  | | --- | --- | --- | --- | --- | |  |  |  |  |  | | EC:4.1.1.39 | | | | | | |
|  |  | RAFL07-07-I23 | At1g56190 / phosphoglycerate kinase -related | |  |  |  |  |  | | --- | --- | --- | --- | --- | |  |  |  |  |  | | EC:2.7.2.3 | | | | | | |
|  |  | RAFL07-18-C20 | At2g21330 / fructose-bisphosphate aldolase, putative | |  |  |  |  |  | | --- | --- | --- | --- | --- | |  |  |  |  |  | | EC:4.1.2.13 | | | | | | |
|  |  | RAFL04-19-O21 | At4g38970 / fructose-bisphosphate aldolase, putative | |  |  |  |  |  | | --- | --- | --- | --- | --- | |  |  |  |  |  | | EC:4.1.2.13 | | | | | | |
|  |  | RAFL09-17-N23 | At2g21330 / fructose-bisphosphate aldolase, putative | |  |  |  |  |  | | --- | --- | --- | --- | --- | |  |  |  |  |  | | EC:4.1.2.13 | | | | | | |
|  |  | RAFL07-14-L16 | At3g12780 / phosphoglycerate kinase -related | |  |  |  |  |  | | --- | --- | --- | --- | --- | |  |  |  |  |  | | EC:2.7.2.3 | | | | | | |
|  |  | RAFL09-15-L04 | At3g12780 / phosphoglycerate kinase -related | |  |  |  |  |  | | --- | --- | --- | --- | --- | |  |  |  |  |  | | EC:2.7.2.3 | | | | | | |
|  |  | RAFL04-19-M17 | At1g32060 / phosphoribulokinase precursor | |  |  |  |  |  | | --- | --- | --- | --- | --- | |  |  |  |  |  | | EC:2.7.1.19 | | | | | | |
|  |  | RAFL03-06-F08 | At1g67090 / ribulose-bisphosphate carboxylase small unit -related | |  |  |  |  |  | | --- | --- | --- | --- | --- | |  |  |  |  |  | | EC:4.1.1.39 | | | | | | |
|  |  | RAFL09-18-L22 | At3g12780 / phosphoglycerate kinase -related | |  |  |  |  |  | | --- | --- | --- | --- | --- | |  |  |  |  |  | | EC:2.7.2.3 | | | | | | |
|  |  | RAFL07-12-M09 | At2g21330 / fructose-bisphosphate aldolase, putative | |  |  |  |  |  | | --- | --- | --- | --- | --- | |  |  |  |  |  | | EC:4.1.2.13 | | | | | | |
|  |  | RAFL08-18-C10 | At2g21330 / fructose-bisphosphate aldolase, putative | |  |  |  |  |  | | --- | --- | --- | --- | --- | |  |  |  |  |  | | EC:4.1.2.13 | | | | | | |
|  |  | RAFL04-13-J02 | At3g54050 / fructose-bisphosphatase precursor | |  |  |  |  |  | | --- | --- | --- | --- | --- | |  |  |  |  |  | | EC:3.1.3.11 | | | | | | |
|  |  | RAFL09-06-K21 | At1g67090 / ribulose-bisphosphate carboxylase small unit -related | |  |  |  |  |  | | --- | --- | --- | --- | --- | |  |  |  |  |  | | EC:4.1.1.39 | | | | | | |
|  |  | RAFL07-12-E12 | At2g21330 / fructose-bisphosphate aldolase, putative | |  |  |  |  |  | | --- | --- | --- | --- | --- | |  |  |  |  |  | | EC:4.1.2.13 | | | | | | |
|  |  | RAFL07-18-J01 | At2g21330 / fructose-bisphosphate aldolase, putative | |  |  |  |  |  | | --- | --- | --- | --- | --- | |  |  |  |  |  | | EC:4.1.2.13 | | | | | | |
|  |  | RAFL11-07-D01 | At5g38410 / ribulose bisphosphate carboxylase small chain 3b precursor (RuBisCO small subunit 3b) (sp|P10798) | |  |  |  |  |  | | --- | --- | --- | --- | --- | |  |  |  |  |  | | EC:4.1.1.39 | | | | | | |
|  |  | RAFL04-10-J07 | At3g04790 / ribose 5-phosphate isomerase -related | |  |  |  |  |  | | --- | --- | --- | --- | --- | |  |  |  |  |  | | EC:5.3.1.6 | | | | | | |
|  |  | RAFL09-06-P15 | At1g67090 / ribulose-bisphosphate carboxylase small unit -related | |  |  |  |  |  | | --- | --- | --- | --- | --- | |  |  |  |  |  | | EC:4.1.1.39 | | | | | | |
|  |  | RAFL05-12-O19 | At3g55800 / sedoheptulose-bisphosphatase precursor | |  |  |  |  |  | | --- | --- | --- | --- | --- | |  |  |  |  |  | | EC:3.1.3.37 | | | | | | |
|  |  | RAFL07-16-P05 | At3g12780 / phosphoglycerate kinase -related | |  |  |  |  |  | | --- | --- | --- | --- | --- | |  |  |  |  |  | | EC:2.7.2.3 | | | | | | |
|  | Glycolysis / Gluconeogenesis | |  |  | 16 | 90 | 59 | 4498 | 4.556285E-12 | 9.568198E-11 | 21 |
|  |  | RAFL07-07-I23 | At1g56190 / phosphoglycerate kinase -related | |  |  |  |  |  | | --- | --- | --- | --- | --- | |  |  |  |  |  | | EC:2.7.2.3 | | | | | | |
|  |  | RAFL07-18-C20 | At2g21330 / fructose-bisphosphate aldolase, putative | |  |  |  |  |  | | --- | --- | --- | --- | --- | |  |  |  |  |  | | EC:4.1.2.13 | | | | | | |
|  |  | RAFL04-19-O21 | At4g38970 / fructose-bisphosphate aldolase, putative | |  |  |  |  |  | | --- | --- | --- | --- | --- | |  |  |  |  |  | | EC:4.1.2.13 | | | | | | |
|  |  | RAFL09-17-N23 | At2g21330 / fructose-bisphosphate aldolase, putative | |  |  |  |  |  | | --- | --- | --- | --- | --- | |  |  |  |  |  | | EC:4.1.2.13 | | | | | | |
|  |  | RAFL07-14-L16 | At3g12780 / phosphoglycerate kinase -related | |  |  |  |  |  | | --- | --- | --- | --- | --- | |  |  |  |  |  | | EC:2.7.2.3 | | | | | | |
|  |  | RAFL09-15-L04 | At3g12780 / phosphoglycerate kinase -related | |  |  |  |  |  | | --- | --- | --- | --- | --- | |  |  |  |  |  | | EC:2.7.2.3 | | | | | | |
|  |  | RAFL05-07-J06 | At1g42970 / glyceraldehyde-3-phosphate dehydrogenase | |  |  |  |  |  | | --- | --- | --- | --- | --- | |  |  |  |  |  | | EC:1.2.1.12 | | | | | | |
|  |  | RAFL04-09-D24 | At1g42970 / glyceraldehyde-3-phosphate dehydrogenase | |  |  |  |  |  | | --- | --- | --- | --- | --- | |  |  |  |  |  | | EC:1.2.1.12 | | | | | | |
|  |  | RAFL09-18-L22 | At3g12780 / phosphoglycerate kinase -related | |  |  |  |  |  | | --- | --- | --- | --- | --- | |  |  |  |  |  | | EC:2.7.2.3 | | | | | | |
|  |  | RAFL07-12-M09 | At2g21330 / fructose-bisphosphate aldolase, putative | |  |  |  |  |  | | --- | --- | --- | --- | --- | |  |  |  |  |  | | EC:4.1.2.13 | | | | | | |
|  |  | RAFL08-18-C10 | At2g21330 / fructose-bisphosphate aldolase, putative | |  |  |  |  |  | | --- | --- | --- | --- | --- | |  |  |  |  |  | | EC:4.1.2.13 | | | | | | |
|  |  | RAFL04-13-J02 | At3g54050 / fructose-bisphosphatase precursor | |  |  |  |  |  | | --- | --- | --- | --- | --- | |  |  |  |  |  | | EC:3.1.3.11 | | | | | | |
|  |  | RAFL07-12-E12 | At2g21330 / fructose-bisphosphate aldolase, putative | |  |  |  |  |  | | --- | --- | --- | --- | --- | |  |  |  |  |  | | EC:4.1.2.13 | | | | | | |
|  |  | RAFL07-18-J01 | At2g21330 / fructose-bisphosphate aldolase, putative | |  |  |  |  |  | | --- | --- | --- | --- | --- | |  |  |  |  |  | | EC:4.1.2.13 | | | | | | |
|  |  | RAFL04-15-A14 | At1g12900 / calcium-binding protein, calreticulin -related | |  |  |  |  |  | | --- | --- | --- | --- | --- | |  |  |  |  |  | | EC:1.2.1.12 | | | | | | |
|  |  | RAFL07-16-P05 | At3g12780 / phosphoglycerate kinase -related | |  |  |  |  |  | | --- | --- | --- | --- | --- | |  |  |  |  |  | | EC:2.7.2.3 | | | | | | |
|  | Inositol metabolism | |  |  | 7 | 99 | 6 | 4551 | 3.9462105E-9 | 2.3677263E-8 | 6 |
|  |  | RAFL07-18-C20 | At2g21330 / fructose-bisphosphate aldolase, putative | |  |  |  |  |  | | --- | --- | --- | --- | --- | |  |  |  |  |  | | EC:4.1.2.13 | | | | | | |
|  |  | RAFL04-19-O21 | At4g38970 / fructose-bisphosphate aldolase, putative | |  |  |  |  |  | | --- | --- | --- | --- | --- | |  |  |  |  |  | | EC:4.1.2.13 | | | | | | |
|  |  | RAFL09-17-N23 | At2g21330 / fructose-bisphosphate aldolase, putative | |  |  |  |  |  | | --- | --- | --- | --- | --- | |  |  |  |  |  | | EC:4.1.2.13 | | | | | | |
|  |  | RAFL07-12-M09 | At2g21330 / fructose-bisphosphate aldolase, putative | |  |  |  |  |  | | --- | --- | --- | --- | --- | |  |  |  |  |  | | EC:4.1.2.13 | | | | | | |
|  |  | RAFL08-18-C10 | At2g21330 / fructose-bisphosphate aldolase, putative | |  |  |  |  |  | | --- | --- | --- | --- | --- | |  |  |  |  |  | | EC:4.1.2.13 | | | | | | |
|  |  | RAFL07-12-E12 | At2g21330 / fructose-bisphosphate aldolase, putative | |  |  |  |  |  | | --- | --- | --- | --- | --- | |  |  |  |  |  | | EC:4.1.2.13 | | | | | | |
|  |  | RAFL07-18-J01 | At2g21330 / fructose-bisphosphate aldolase, putative | |  |  |  |  |  | | --- | --- | --- | --- | --- | |  |  |  |  |  | | EC:4.1.2.13 | | | | | | |
|  | Pentose phosphate pathway | |  |  | 9 | 97 | 24 | 4533 | 2.8207568E-8 | 4.5132109E-7 | 16 |
|  |  | RAFL07-18-C20 | At2g21330 / fructose-bisphosphate aldolase, putative | |  |  |  |  |  | | --- | --- | --- | --- | --- | |  |  |  |  |  | | EC:4.1.2.13 | | | | | | |
|  |  | RAFL04-19-O21 | At4g38970 / fructose-bisphosphate aldolase, putative | |  |  |  |  |  | | --- | --- | --- | --- | --- | |  |  |  |  |  | | EC:4.1.2.13 | | | | | | |
|  |  | RAFL09-17-N23 | At2g21330 / fructose-bisphosphate aldolase, putative | |  |  |  |  |  | | --- | --- | --- | --- | --- | |  |  |  |  |  | | EC:4.1.2.13 | | | | | | |
|  |  | RAFL07-12-M09 | At2g21330 / fructose-bisphosphate aldolase, putative | |  |  |  |  |  | | --- | --- | --- | --- | --- | |  |  |  |  |  | | EC:4.1.2.13 | | | | | | |
|  |  | RAFL08-18-C10 | At2g21330 / fructose-bisphosphate aldolase, putative | |  |  |  |  |  | | --- | --- | --- | --- | --- | |  |  |  |  |  | | EC:4.1.2.13 | | | | | | |
|  |  | RAFL04-13-J02 | At3g54050 / fructose-bisphosphatase precursor | |  |  |  |  |  | | --- | --- | --- | --- | --- | |  |  |  |  |  | | EC:3.1.3.11 | | | | | | |
|  |  | RAFL07-12-E12 | At2g21330 / fructose-bisphosphate aldolase, putative | |  |  |  |  |  | | --- | --- | --- | --- | --- | |  |  |  |  |  | | EC:4.1.2.13 | | | | | | |
|  |  | RAFL07-18-J01 | At2g21330 / fructose-bisphosphate aldolase, putative | |  |  |  |  |  | | --- | --- | --- | --- | --- | |  |  |  |  |  | | EC:4.1.2.13 | | | | | | |
|  |  | RAFL04-10-J07 | At3g04790 / ribose 5-phosphate isomerase -related | |  |  |  |  |  | | --- | --- | --- | --- | --- | |  |  |  |  |  | | EC:5.3.1.6 | | | | | | |
|  | Fructose and mannose metabolism | |  |  | 8 | 98 | 25 | 4532 | 4.73974E-7 | 6.161662E-6 | 13 |
|  |  | RAFL07-18-C20 | At2g21330 / fructose-bisphosphate aldolase, putative | |  |  |  |  |  | | --- | --- | --- | --- | --- | |  |  |  |  |  | | EC:4.1.2.13 | | | | | | |
|  |  | RAFL04-19-O21 | At4g38970 / fructose-bisphosphate aldolase, putative | |  |  |  |  |  | | --- | --- | --- | --- | --- | |  |  |  |  |  | | EC:4.1.2.13 | | | | | | |
|  |  | RAFL09-17-N23 | At2g21330 / fructose-bisphosphate aldolase, putative | |  |  |  |  |  | | --- | --- | --- | --- | --- | |  |  |  |  |  | | EC:4.1.2.13 | | | | | | |
|  |  | RAFL07-12-M09 | At2g21330 / fructose-bisphosphate aldolase, putative | |  |  |  |  |  | | --- | --- | --- | --- | --- | |  |  |  |  |  | | EC:4.1.2.13 | | | | | | |
|  |  | RAFL08-18-C10 | At2g21330 / fructose-bisphosphate aldolase, putative | |  |  |  |  |  | | --- | --- | --- | --- | --- | |  |  |  |  |  | | EC:4.1.2.13 | | | | | | |
|  |  | RAFL04-13-J02 | At3g54050 / fructose-bisphosphatase precursor | |  |  |  |  |  | | --- | --- | --- | --- | --- | |  |  |  |  |  | | EC:3.1.3.11 | | | | | | |
|  |  | RAFL07-12-E12 | At2g21330 / fructose-bisphosphate aldolase, putative | |  |  |  |  |  | | --- | --- | --- | --- | --- | |  |  |  |  |  | | EC:4.1.2.13 | | | | | | |
|  |  | RAFL07-18-J01 | At2g21330 / fructose-bisphosphate aldolase, putative | |  |  |  |  |  | | --- | --- | --- | --- | --- | |  |  |  |  |  | | EC:4.1.2.13 | | | | | | |
|  | D-Arginine and D-ornithine metabolism | |  |  | 3 | 103 | 5 | 4552 | 5.885334E-4 | 0.0035312006 | 6 |
|  |  | RAFL05-07-J06 | At1g42970 / glyceraldehyde-3-phosphate dehydrogenase | |  |  |  |  |  | | --- | --- | --- | --- | --- | |  |  |  |  |  | | EC:1.2.1.12 | | | | | | |
|  |  | RAFL04-09-D24 | At1g42970 / glyceraldehyde-3-phosphate dehydrogenase | |  |  |  |  |  | | --- | --- | --- | --- | --- | |  |  |  |  |  | | EC:1.2.1.12 | | | | | | |
|  |  | RAFL04-15-A14 | At1g12900 / calcium-binding protein, calreticulin -related | |  |  |  |  |  | | --- | --- | --- | --- | --- | |  |  |  |  |  | | EC:1.2.1.12 | | | | | | |
|  | Glyoxylate and dicarboxylate metabolism | |  |  | 5 | 101 | 44 | 4513 | 0.0047566555 | 0.07610649 | 16 |
|  |  | RAFL11-02-L02 | At1g67090 / ribulose-bisphosphate carboxylase small unit -related | |  |  |  |  |  | | --- | --- | --- | --- | --- | |  |  |  |  |  | | EC:4.1.1.39 | | | | | | |
|  |  | RAFL03-06-F08 | At1g67090 / ribulose-bisphosphate carboxylase small unit -related | |  |  |  |  |  | | --- | --- | --- | --- | --- | |  |  |  |  |  | | EC:4.1.1.39 | | | | | | |
|  |  | RAFL09-06-K21 | At1g67090 / ribulose-bisphosphate carboxylase small unit -related | |  |  |  |  |  | | --- | --- | --- | --- | --- | |  |  |  |  |  | | EC:4.1.1.39 | | | | | | |
|  |  | RAFL11-07-D01 | At5g38410 / ribulose bisphosphate carboxylase small chain 3b precursor (RuBisCO small subunit 3b) (sp|P10798) | |  |  |  |  |  | | --- | --- | --- | --- | --- | |  |  |  |  |  | | EC:4.1.1.39 | | | | | | |
|  |  | RAFL09-06-P15 | At1g67090 / ribulose-bisphosphate carboxylase small unit -related | |  |  |  |  |  | | --- | --- | --- | --- | --- | |  |  |  |  |  | | EC:4.1.1.39 | | | | | | |
|  | One carbon pool by folate | |  |  | 2 | 104 | 5 | 4552 | 0.0099780755 | 0.049890377 | 5 |
|  |  | RAFL06-13-H16 | At1g11860 / aminomethyltransferase-related precursor protein | |  |  |  |  |  | | --- | --- | --- | --- | --- | |  |  |  |  |  | | EC:2.1.2.10 | | | | | | |
|  |  | RAFL05-04-O06 | At4g37930 / glycine hydroxymethyltransferase like protein | |  |  |  |  |  | | --- | --- | --- | --- | --- | |  |  |  |  |  | | EC:2.1.2.1 | | | | | | |
|  | Cyanoamino acid metabolism | |  |  | 2 | 104 | 13 | 4544 | 0.04433326 | 0.4433326 | 10 |
|  |  | RAFL05-04-O06 | At4g37930 / glycine hydroxymethyltransferase like protein | |  |  |  |  |  | | --- | --- | --- | --- | --- | |  |  |  |  |  | | EC:2.1.2.1 | | | | | | |
|  |  | RAFL09-15-K15 | At3g18080 / glycosyl hydrolase family 1 | |  |  |  |  |  | | --- | --- | --- | --- | --- | |  |  |  |  |  | | EC:3.2.1.21 | | | | | | |
| Cluster:7-0 | | |  |  | A | B | C | D | P | P' | N |
|  | Biotin metabolism | |  |  | 2 | 245 | 2 | 4414 | 0.015618202 | 0.046854608 | 3 |
|  |  | RAFL07-12-J17 | At3g48780 / serine C-palmitoyltransferase, putative | |  |  |  |  |  | | --- | --- | --- | --- | --- | |  |  |  |  |  | | EC:2.3.1.47 | | | | | | |
|  |  | RAFL05-21-O04 | At3g08860 / alanine--glyoxylate aminotransferase (beta-alanine-pyruvate aminotransferase/AGT), putative | |  |  |  |  |  | | --- | --- | --- | --- | --- | |  |  |  |  |  | | EC:2.6.1.62 | | | | | | |
| Cluster:3-1 | | |  |  | A | B | C | D | P | P' | N |
|  | Urea cycle and metabolism of amino groups | |  |  | 5 | 211 | 17 | 4430 | 0.0028076097 | 0.033691317 | 12 |
|  |  | RAFL04-16-G24 | At1g80600 / acetylornithine aminotransferase, mitochondrial (acetylornithine transaminase/AOTA/ACOAT), putative | |  |  |  |  |  | | --- | --- | --- | --- | --- | |  |  |  |  |  | | EC:2.6.1.13 ,EC:2.6.1.11 | | | | | | |
|  |  | RAFL06-16-I04 | At5g19530 / spermine synthase (ACL5) | |  |  |  |  |  | | --- | --- | --- | --- | --- | |  |  |  |  |  | | EC:2.5.1.22 | | | | | | |
|  |  | RAFL07-16-F16 | At3g20330 / aspartate carbamoyltransferase precursor (aspartate transcarbamylase) | |  |  |  |  |  | | --- | --- | --- | --- | --- | |  |  |  |  |  | | EC:2.1.3.3 | | | | | | |
|  |  | RAFL07-18-A10 | At2g37500 / glutamate/ornithine acetyltransferase -related | |  |  |  |  |  | | --- | --- | --- | --- | --- | |  |  |  |  |  | | EC:2.3.1.35 ,EC:2.3.1.1 | | | | | | |
|  |  | RAFL07-08-L02 | At5g10920 / argininosuccinate lyase (AtArgH) | |  |  |  |  |  | | --- | --- | --- | --- | --- | |  |  |  |  |  | | EC:4.3.2.1 | | | | | | |
|  | Phenylalanine, tyrosine and tryptophan biosynthesis | |  |  | 5 | 211 | 23 | 4424 | 0.00835746 | 0.108646974 | 13 |
|  |  | RAFL05-13-G12 | At1g07780 / phosphoribosylanthranilate isomerase (PAI1) | |  |  |  |  |  | | --- | --- | --- | --- | --- | |  |  |  |  |  | | EC:5.3.1.24 | | | | | | |
|  |  | RAFL04-13-O10 | At2g29560 / enolase (2-phospho-D-glycerate hydroylase) -related | |  |  |  |  |  | | --- | --- | --- | --- | --- | |  |  |  |  |  | | EC:4.2.1.11 | | | | | | |
|  |  | RAFL05-16-L22 | At5g48220 / indole-3-glycerol phosphate synthase (IGPS), putative | |  |  |  |  |  | | --- | --- | --- | --- | --- | |  |  |  |  |  | | EC:4.1.1.48 | | | | | | |
|  |  | RAFL04-10-L08 | At4g34200 / D-3-phosphoglycerate dehydrogenase (3-PGDH), putative | |  |  |  |  |  | | --- | --- | --- | --- | --- | |  |  |  |  |  | | EC:2.6.1.9 | | | | | | |
|  |  | RAFL09-09-E22 | At4g39280 / phenylalanyl-trna synthetase - like protein | |  |  |  |  |  | | --- | --- | --- | --- | --- | |  |  |  |  |  | | EC:6.1.1.20 | | | | | | |
|  | Glycolysis / Gluconeogenesis | |  |  | 8 | 208 | 67 | 4380 | 0.021701496 | 0.4557314 | 21 |
|  |  | RAFL07-10-P13 | At1g09780 / 2,3-bisphosphoglycerate-independent phosphoglycerate mutase -related | |  |  |  |  |  | | --- | --- | --- | --- | --- | |  |  |  |  |  | | EC:5.4.2.1 | | | | | | |
|  |  | RAFL09-12-D13 | At5g52920 / pyruvate kinase, putative | |  |  |  |  |  | | --- | --- | --- | --- | --- | |  |  |  |  |  | | EC:2.7.1.40 | | | | | | |
|  |  | RAFL05-05-G17 | At3g25860 / dihydrolipoamide S-acetyltransferase | |  |  |  |  |  | | --- | --- | --- | --- | --- | |  |  |  |  |  | | EC:2.3.1.12 | | | | | | |
|  |  | RAFL04-13-O10 | At2g29560 / enolase (2-phospho-D-glycerate hydroylase) -related | |  |  |  |  |  | | --- | --- | --- | --- | --- | |  |  |  |  |  | | EC:4.2.1.11 | | | | | | |
|  |  | RAFL05-11-L02 | At4g25900 / aldose 1-epimerase family | |  |  |  |  |  | | --- | --- | --- | --- | --- | |  |  |  |  |  | | EC:5.1.3.3 | | | | | | |
|  |  | RAFL05-01-I24 | At3g55440 / triosephosphate isomerase, cytosolic, putative | |  |  |  |  |  | | --- | --- | --- | --- | --- | |  |  |  |  |  | | EC:5.3.1.1 | | | | | | |
|  |  | RAFL04-17-I11 | At3g13930 / acetyltransferase -related | |  |  |  |  |  | | --- | --- | --- | --- | --- | |  |  |  |  |  | | EC:2.3.1.12 | | | | | | |
|  |  | RAFL05-21-G03 | At3g22960 / pyruvate kinase, putative | |  |  |  |  |  | | --- | --- | --- | --- | --- | |  |  |  |  |  | | EC:2.7.1.40 | | | | | | |
| Cluster:3-2 | | |  |  | A | B | C | D | P | P' | N |
|  | Porphyrin and chlorophyll metabolism | |  |  | 5 | 32 | 16 | 4610 | 4.413796E-7 | 4.8551756E-6 | 11 |
|  |  | RAFL08-10-H13 | At5g07830 / glycosyl hydrolase family 79 (endo-beta-glucuronidase/heparanase) | |  |  |  |  |  | | --- | --- | --- | --- | --- | |  |  |  |  |  | | EC:3.2.1.31 | | | | | | |
|  |  | RAFL09-06-N12 | At3g23810 / S-adenosyl-L-homocysteinas -related | |  |  |  |  |  | | --- | --- | --- | --- | --- | |  |  |  |  |  | | EC:4.3.1.8 | | | | | | |
|  |  | RAFL09-13-P13 | At3g23810 / S-adenosyl-L-homocysteinas -related | |  |  |  |  |  | | --- | --- | --- | --- | --- | |  |  |  |  |  | | EC:4.3.1.8 | | | | | | |
|  |  | RAFL07-09-L01 | At3g23810 / S-adenosyl-L-homocysteinas -related | |  |  |  |  |  | | --- | --- | --- | --- | --- | |  |  |  |  |  | | EC:4.3.1.8 | | | | | | |
|  |  | RAFL09-10-M18 | At3g23810 / S-adenosyl-L-homocysteinas -related | |  |  |  |  |  | | --- | --- | --- | --- | --- | |  |  |  |  |  | | EC:4.3.1.8 | | | | | | |
|  | Methionine metabolism | |  |  | 4 | 33 | 19 | 4607 | 2.668534E-5 | 2.1348272E-4 | 8 |
|  |  | RAFL09-06-N12 | At3g23810 / S-adenosyl-L-homocysteinas -related | |  |  |  |  |  | | --- | --- | --- | --- | --- | |  |  |  |  |  | | EC:3.3.1.1 | | | | | | |
|  |  | RAFL09-13-P13 | At3g23810 / S-adenosyl-L-homocysteinas -related | |  |  |  |  |  | | --- | --- | --- | --- | --- | |  |  |  |  |  | | EC:3.3.1.1 | | | | | | |
|  |  | RAFL07-09-L01 | At3g23810 / S-adenosyl-L-homocysteinas -related | |  |  |  |  |  | | --- | --- | --- | --- | --- | |  |  |  |  |  | | EC:3.3.1.1 | | | | | | |
|  |  | RAFL09-10-M18 | At3g23810 / S-adenosyl-L-homocysteinas -related | |  |  |  |  |  | | --- | --- | --- | --- | --- | |  |  |  |  |  | | EC:3.3.1.1 | | | | | | |
|  | Selenoamino acid metabolism | |  |  | 4 | 33 | 24 | 4602 | 5.997704E-5 | 7.797015E-4 | 13 |
|  |  | RAFL09-06-N12 | At3g23810 / S-adenosyl-L-homocysteinas -related | |  |  |  |  |  | | --- | --- | --- | --- | --- | |  |  |  |  |  | | EC:3.3.1.1 | | | | | | |
|  |  | RAFL09-13-P13 | At3g23810 / S-adenosyl-L-homocysteinas -related | |  |  |  |  |  | | --- | --- | --- | --- | --- | |  |  |  |  |  | | EC:3.3.1.1 | | | | | | |
|  |  | RAFL07-09-L01 | At3g23810 / S-adenosyl-L-homocysteinas -related | |  |  |  |  |  | | --- | --- | --- | --- | --- | |  |  |  |  |  | | EC:3.3.1.1 | | | | | | |
|  |  | RAFL09-10-M18 | At3g23810 / S-adenosyl-L-homocysteinas -related | |  |  |  |  |  | | --- | --- | --- | --- | --- | |  |  |  |  |  | | EC:3.3.1.1 | | | | | | |
|  | Starch and sucrose metabolism | |  |  | 4 | 33 | 54 | 4572 | 0.0010484793 | 0.023066545 | 22 |
|  |  | RAFL08-10-H13 | At5g07830 / glycosyl hydrolase family 79 (endo-beta-glucuronidase/heparanase) | |  |  |  |  |  | | --- | --- | --- | --- | --- | |  |  |  |  |  | | EC:3.2.1.31 | | | | | | |
|  |  | RAFL07-12-E11 | At3g02230 / reversibly glycosylated polypeptide-1 | |  |  |  |  |  | | --- | --- | --- | --- | --- | |  |  |  |  |  | | EC:2.4.1.12 | | | | | | |
|  |  | RAFL05-16-L21 | At4g15210 / glycosyl hydrolase family 14 (beta-amylase) | |  |  |  |  |  | | --- | --- | --- | --- | --- | |  |  |  |  |  | | EC:3.2.1.2 | | | | | | |
|  |  | RAFL04-14-G14 | At1g26560 / glycosyl hydrolase family 1 | |  |  |  |  |  | | --- | --- | --- | --- | --- | |  |  |  |  |  | | EC:3.2.1.21 | | | | | | |
|  | Erythromycin biosynthesis | |  |  | 2 | 35 | 8 | 4618 | 0.0026489347 | 0.018542543 | 7 |
|  |  | RAFL09-16-F08 | At3g23820 / NAD-dependent epimerase/dehydratase family | |  |  |  |  |  | | --- | --- | --- | --- | --- | |  |  |  |  |  | | EC:4.2.1.46 | | | | | | |
|  |  | RAFL09-07-D12 | At3g23820 / NAD-dependent epimerase/dehydratase family | |  |  |  |  |  | | --- | --- | --- | --- | --- | |  |  |  |  |  | | EC:4.2.1.46 | | | | | | |
|  | Streptomycin biosynthesis | |  |  | 2 | 35 | 12 | 4614 | 0.0052507473 | 0.04200598 | 8 |
|  |  | RAFL09-16-F08 | At3g23820 / NAD-dependent epimerase/dehydratase family | |  |  |  |  |  | | --- | --- | --- | --- | --- | |  |  |  |  |  | | EC:4.2.1.46 | | | | | | |
|  |  | RAFL09-07-D12 | At3g23820 / NAD-dependent epimerase/dehydratase family | |  |  |  |  |  | | --- | --- | --- | --- | --- | |  |  |  |  |  | | EC:4.2.1.46 | | | | | | |
|  | Nucleotide sugars metabolism | |  |  | 2 | 35 | 20 | 4606 | 0.012808084 | 0.17931317 | 14 |
|  |  | RAFL09-16-F08 | At3g23820 / NAD-dependent epimerase/dehydratase family | |  |  |  |  |  | | --- | --- | --- | --- | --- | |  |  |  |  |  | | EC:4.2.1.46 ,EC:5.1.3.2 | | | | | | |
|  |  | RAFL09-07-D12 | At3g23820 / NAD-dependent epimerase/dehydratase family | |  |  |  |  |  | | --- | --- | --- | --- | --- | |  |  |  |  |  | | EC:4.2.1.46 ,EC:5.1.3.2 | | | | | | |
|  | Fructose and mannose metabolism | |  |  | 2 | 35 | 31 | 4595 | 0.027721709 | 0.3603822 | 13 |
|  |  | RAFL09-16-F08 | At3g23820 / NAD-dependent epimerase/dehydratase family | |  |  |  |  |  | | --- | --- | --- | --- | --- | |  |  |  |  |  | | EC:4.2.1.47 | | | | | | |
|  |  | RAFL09-07-D12 | At3g23820 / NAD-dependent epimerase/dehydratase family | |  |  |  |  |  | | --- | --- | --- | --- | --- | |  |  |  |  |  | | EC:4.2.1.47 | | | | | | |
|  | Galactose metabolism | |  |  | 2 | 35 | 34 | 4592 | 0.032590393 | 0.68439823 | 21 |
|  |  | RAFL09-16-F08 | At3g23820 / NAD-dependent epimerase/dehydratase family | |  |  |  |  |  | | --- | --- | --- | --- | --- | |  |  |  |  |  | | EC:5.1.3.2 | | | | | | |
|  |  | RAFL09-07-D12 | At3g23820 / NAD-dependent epimerase/dehydratase family | |  |  |  |  |  | | --- | --- | --- | --- | --- | |  |  |  |  |  | | EC:5.1.3.2 | | | | | | |
| Cluster:6-2 | | |  |  | A | B | C | D | P | P' | N |
|  | Alkaloid biosynthesis II | |  |  | 3 | 173 | 4 | 4483 | 0.0016539665 | 0.0082698325 | 5 |
|  |  | RAFL04-13-B02 | At2g37040 / phenylalanine ammonia lyase (PAL1) | |  |  |  |  |  | | --- | --- | --- | --- | --- | |  |  |  |  |  | | EC:4.3.1.5 | | | | | | |
|  |  | RAFL09-11-L22 | At3g53260 / phenylalanine ammonia-lyase (PAL2) | |  |  |  |  |  | | --- | --- | --- | --- | --- | |  |  |  |  |  | | EC:4.3.1.5 | | | | | | |
|  |  | RAFL04-16-D08 | At3g53260 / phenylalanine ammonia-lyase (PAL2) | |  |  |  |  |  | | --- | --- | --- | --- | --- | |  |  |  |  |  | | EC:4.3.1.5 | | | | | | |
|  | Tyrosine metabolism | |  |  | 4 | 172 | 25 | 4462 | 0.02227629 | 0.31186807 | 14 |
|  |  | RAFL02-07-O01 | At2g24270 / NADP-dependent glyceraldehyde-3-phosphate dehydrogenase, putative | |  |  |  |  |  | | --- | --- | --- | --- | --- | |  |  |  |  |  | | EC:1.2.1.16 | | | | | | |
|  |  | RAFL04-13-B02 | At2g37040 / phenylalanine ammonia lyase (PAL1) | |  |  |  |  |  | | --- | --- | --- | --- | --- | |  |  |  |  |  | | EC:4.3.1.5 | | | | | | |
|  |  | RAFL09-11-L22 | At3g53260 / phenylalanine ammonia-lyase (PAL2) | |  |  |  |  |  | | --- | --- | --- | --- | --- | |  |  |  |  |  | | EC:4.3.1.5 | | | | | | |
|  |  | RAFL04-16-D08 | At3g53260 / phenylalanine ammonia-lyase (PAL2) | |  |  |  |  |  | | --- | --- | --- | --- | --- | |  |  |  |  |  | | EC:4.3.1.5 | | | | | | |
|  | Phenylalanine metabolism | |  |  | 4 | 172 | 33 | 4454 | 0.0491507 | 0.8847126 | 18 |
|  |  | RAFL02-07-M07 | At1g08980 / amidase | |  |  |  |  |  | | --- | --- | --- | --- | --- | |  |  |  |  |  | | EC:3.5.1.4 | | | | | | |
|  |  | RAFL04-13-B02 | At2g37040 / phenylalanine ammonia lyase (PAL1) | |  |  |  |  |  | | --- | --- | --- | --- | --- | |  |  |  |  |  | | EC:4.3.1.5 | | | | | | |
|  |  | RAFL09-11-L22 | At3g53260 / phenylalanine ammonia-lyase (PAL2) | |  |  |  |  |  | | --- | --- | --- | --- | --- | |  |  |  |  |  | | EC:4.3.1.5 | | | | | | |
|  |  | RAFL04-16-D08 | At3g53260 / phenylalanine ammonia-lyase (PAL2) | |  |  |  |  |  | | --- | --- | --- | --- | --- | |  |  |  |  |  | | EC:4.3.1.5 | | | | | | |
| Cluster:1-1 | | |  |  | A | B | C | D | P | P' | N |
|  | Aminosugars metabolism | |  |  | 2 | 103 | 3 | 4555 | 0.004804865 | 0.01921946 | 4 |
|  |  | RAFL09-18-H10 | At5g19220 / glucose-1-phosphate adenylyltransferase, large subunit 1, chloroplast (ADP-glucose pyrophosphorylase) (ADG2) (APL1) | |  |  |  |  |  | | --- | --- | --- | --- | --- | |  |  |  |  |  | | EC:2.7.7.23 | | | | | | |
|  |  | RAFL09-13-M20 | At1g31070 / UDP-N-acetylglucosamine pyrophosphorylase-related protein | |  |  |  |  |  | | --- | --- | --- | --- | --- | |  |  |  |  |  | | EC:2.7.7.23 | | | | | | |
|  | Riboflavin metabolism | |  |  | 1 | 104 | 1 | 4557 | 0.04453306 | 0.08906612 | 2 |
|  |  | RAFL08-12-F18 | At2g44050 / 6,7-dimethyl-8-ribityllumazine synthase precursor | |  |  |  |  |  | | --- | --- | --- | --- | --- | |  |  |  |  |  | | EC:2.5.1.9 | | | | | | |
| Cluster:5-1 | | |  |  | A | B | C | D | P | P' | N |
|  | Tyrosine metabolism | |  |  | 5 | 277 | 24 | 4357 | 0.028004097 | 0.39205736 | 14 |
|  |  | RAFL08-15-H03 | At1g79440 / succinate-semialdehyde dehydrogenase, putative (SSDH) | |  |  |  |  |  | | --- | --- | --- | --- | --- | |  |  |  |  |  | | EC:1.2.1.16 | | | | | | |
|  |  | RAFL05-18-D02 | At3g43670 / amine oxidase -related protein | |  |  |  |  |  | | --- | --- | --- | --- | --- | |  |  |  |  |  | | EC:1.4.3.6 | | | | | | |
|  |  | RAFL05-14-M18 | At1g12050 / fumarylacetoacetate hydrolase-related protein | |  |  |  |  |  | | --- | --- | --- | --- | --- | |  |  |  |  |  | | EC:3.7.1.2 | | | | | | |
|  |  | RAFL08-16-B22 | At1g11840 / glyoxalase I, putative (lactoylglutathione lyase) | |  |  |  |  |  | | --- | --- | --- | --- | --- | |  |  |  |  |  | | EC:1.13.11.27 | | | | | | |
|  |  | RAFL09-07-G14 | At1g67280 / glyoxalase I, putative (lactoylglutathione lyase) | |  |  |  |  |  | | --- | --- | --- | --- | --- | |  |  |  |  |  | | EC:1.13.11.27 | | | | | | |
| Cluster:1-2 | | |  |  | A | B | C | D | P | P' | N |
|  | Methionine metabolism | |  |  | 6 | 168 | 17 | 4472 | 1.4723212E-4 | 0.0011778569 | 8 |
|  |  | RAFL09-11-C22 | At5g17920 / 5-methyltetrahydropteroyltriglutamate--homocysteine S-methyltransferase | |  |  |  |  |  | | --- | --- | --- | --- | --- | |  |  |  |  |  | | EC:2.1.1.14 | | | | | | |
|  |  | RAFL07-08-E09 | At5g49030 / isoleucyl-tRNA synthetase | |  |  |  |  |  | | --- | --- | --- | --- | --- | |  |  |  |  |  | | EC:6.1.1.10 | | | | | | |
|  |  | RAFL06-12-D05 | At5g17920 / 5-methyltetrahydropteroyltriglutamate--homocysteine S-methyltransferase | |  |  |  |  |  | | --- | --- | --- | --- | --- | |  |  |  |  |  | | EC:2.1.1.14 | | | | | | |
|  |  | RAFL11-01-K15 | At5g17920 / 5-methyltetrahydropteroyltriglutamate--homocysteine S-methyltransferase | |  |  |  |  |  | | --- | --- | --- | --- | --- | |  |  |  |  |  | | EC:2.1.1.14 | | | | | | |
|  |  | RAFL09-10-C09 | At5g17920 / 5-methyltetrahydropteroyltriglutamate--homocysteine S-methyltransferase | |  |  |  |  |  | | --- | --- | --- | --- | --- | |  |  |  |  |  | | EC:2.1.1.14 | | | | | | |
|  |  | RAFL11-06-L17 | At5g17920 / 5-methyltetrahydropteroyltriglutamate--homocysteine S-methyltransferase | |  |  |  |  |  | | --- | --- | --- | --- | --- | |  |  |  |  |  | | EC:2.1.1.14 | | | | | | |
|  | Fructose and mannose metabolism | |  |  | 5 | 169 | 28 | 4461 | 0.0069361012 | 0.09016931 | 13 |
|  |  | RAFL04-09-G20 | At2g21170 / triosephosphate isomerase, chloroplast, putative | |  |  |  |  |  | | --- | --- | --- | --- | --- | |  |  |  |  |  | | EC:5.3.1.1 | | | | | | |
|  |  | RAFL07-12-L15 | At1g12000 / pyrophosphate-fructose-6-phosphate 1-phosphotransferase -related | |  |  |  |  |  | | --- | --- | --- | --- | --- | |  |  |  |  |  | | EC:2.7.1.90 ,EC:2.7.1.11 | | | | | | |
|  |  | RAFL05-21-I19 | At1g43670 / fructose 1,6-bisphosphatase -related | |  |  |  |  |  | | --- | --- | --- | --- | --- | |  |  |  |  |  | | EC:3.1.3.11 | | | | | | |
|  |  | RAFL07-15-F22 | At1g20950 / pyrophosphate-dependent phosphofructokinase alpha subunit -related | |  |  |  |  |  | | --- | --- | --- | --- | --- | |  |  |  |  |  | | EC:2.7.1.90 ,EC:2.7.1.11 | | | | | | |
|  |  | RAFL05-13-B09 | At5g03300 / pfkB type carbohydrate kinase protein family | |  |  |  |  |  | | --- | --- | --- | --- | --- | |  |  |  |  |  | | EC:2.7.1.11 | | | | | | |
|  | One carbon pool by folate | |  |  | 2 | 172 | 5 | 4484 | 0.025693169 | 0.12846585 | 5 |
|  |  | RAFL09-11-K06 | At4g13930 / hydroxymethyltransferase | |  |  |  |  |  | | --- | --- | --- | --- | --- | |  |  |  |  |  | | EC:2.1.2.1 | | | | | | |
|  |  | RAFL09-16-M15 | At1g11860 / aminomethyltransferase-related precursor protein | |  |  |  |  |  | | --- | --- | --- | --- | --- | |  |  |  |  |  | | EC:2.1.2.10 | | | | | | |
|  | Pentose phosphate pathway | |  |  | 4 | 170 | 29 | 4460 | 0.032959472 | 0.52735156 | 16 |
|  |  | RAFL07-12-L15 | At1g12000 / pyrophosphate-fructose-6-phosphate 1-phosphotransferase -related | |  |  |  |  |  | | --- | --- | --- | --- | --- | |  |  |  |  |  | | EC:2.7.1.11 | | | | | | |
|  |  | RAFL05-21-I19 | At1g43670 / fructose 1,6-bisphosphatase -related | |  |  |  |  |  | | --- | --- | --- | --- | --- | |  |  |  |  |  | | EC:3.1.3.11 | | | | | | |
|  |  | RAFL07-15-F22 | At1g20950 / pyrophosphate-dependent phosphofructokinase alpha subunit -related | |  |  |  |  |  | | --- | --- | --- | --- | --- | |  |  |  |  |  | | EC:2.7.1.11 | | | | | | |
|  |  | RAFL05-13-B09 | At5g03300 / pfkB type carbohydrate kinase protein family | |  |  |  |  |  | | --- | --- | --- | --- | --- | |  |  |  |  |  | | EC:2.7.1.11 | | | | | | |
| Cluster:9-1 | | |  |  | A | B | C | D | P | P' | N |
|  | Phenylalanine metabolism | |  |  | 5 | 91 | 32 | 4535 | 8.6172705E-4 | 0.015511087 | 18 |
|  |  | RAFL05-19-H07 | At5g11520 / aspartate aminotransferase, chloroplast (transaminase A/Asp3) | |  |  |  |  |  | | --- | --- | --- | --- | --- | |  |  |  |  |  | | EC:2.6.1.1 | | | | | | |
|  |  | RAFL04-20-P19 | At3g49110 / peroxidase | |  |  |  |  |  | | --- | --- | --- | --- | --- | |  |  |  |  |  | | EC:1.11.1.7 | | | | | | |
|  |  | RAFL11-09-O05 | At1g06570 / 4-hydroxyphenylpyruvate dioxygenase (HPD) | |  |  |  |  |  | | --- | --- | --- | --- | --- | |  |  |  |  |  | | EC:1.13.11.27 | | | | | | |
|  |  | RAFL11-12-C18 | At1g06570 / 4-hydroxyphenylpyruvate dioxygenase (HPD) | |  |  |  |  |  | | --- | --- | --- | --- | --- | |  |  |  |  |  | | EC:1.13.11.27 | | | | | | |
|  |  | RAFL09-07-G15 | At3g49120 / peroxidase, putative | |  |  |  |  |  | | --- | --- | --- | --- | --- | |  |  |  |  |  | | EC:1.11.1.7 | | | | | | |
|  | Cysteine metabolism | |  |  | 3 | 93 | 11 | 4556 | 0.0026107915 | 0.026107915 | 10 |
|  |  | RAFL05-19-H07 | At5g11520 / aspartate aminotransferase, chloroplast (transaminase A/Asp3) | |  |  |  |  |  | | --- | --- | --- | --- | --- | |  |  |  |  |  | | EC:2.6.1.1 | | | | | | |
|  |  | RAFL11-02-N11 | At1g64660 / methionine/cystathionine gamma lyase -related | |  |  |  |  |  | | --- | --- | --- | --- | --- | |  |  |  |  |  | | EC:4.4.1.8 | | | | | | |
|  |  | RAFL05-18-H15 | At1g64660 / methionine/cystathionine gamma lyase -related | |  |  |  |  |  | | --- | --- | --- | --- | --- | |  |  |  |  |  | | EC:4.4.1.8 | | | | | | |
|  | Tyrosine metabolism | |  |  | 4 | 92 | 25 | 4542 | 0.0027000385 | 0.03780054 | 14 |
|  |  | RAFL05-19-H07 | At5g11520 / aspartate aminotransferase, chloroplast (transaminase A/Asp3) | |  |  |  |  |  | | --- | --- | --- | --- | --- | |  |  |  |  |  | | EC:2.6.1.1 | | | | | | |
|  |  | RAFL07-16-P10 | At1g77120 / alcohol dehydrogenase (ADH) | |  |  |  |  |  | | --- | --- | --- | --- | --- | |  |  |  |  |  | | EC:1.1.1.1 | | | | | | |
|  |  | RAFL11-09-O05 | At1g06570 / 4-hydroxyphenylpyruvate dioxygenase (HPD) | |  |  |  |  |  | | --- | --- | --- | --- | --- | |  |  |  |  |  | | EC:1.13.11.27 | | | | | | |
|  |  | RAFL11-12-C18 | At1g06570 / 4-hydroxyphenylpyruvate dioxygenase (HPD) | |  |  |  |  |  | | --- | --- | --- | --- | --- | |  |  |  |  |  | | EC:1.13.11.27 | | | | | | |
|  | Flavonoids, stilbene and lignin biosynthesis | |  |  | 4 | 92 | 32 | 4535 | 0.0060000676 | 0.11400128 | 19 |
|  |  | RAFL04-20-P19 | At3g49110 / peroxidase | |  |  |  |  |  | | --- | --- | --- | --- | --- | |  |  |  |  |  | | EC:1.11.1.7 | | | | | | |
|  |  | RAFL04-13-E17 | At5g20230 / plastocyanin-like domain containing protein | |  |  |  |  |  | | --- | --- | --- | --- | --- | |  |  |  |  |  | | EC:2.1.1.68 | | | | | | |
|  |  | RAFL05-12-N20 | At4g30470 / cinnamoyl-CoA reductase-related | |  |  |  |  |  | | --- | --- | --- | --- | --- | |  |  |  |  |  | | EC:1.2.1.44 | | | | | | |
|  |  | RAFL09-07-G15 | At3g49120 / peroxidase, putative | |  |  |  |  |  | | --- | --- | --- | --- | --- | |  |  |  |  |  | | EC:1.11.1.7 | | | | | | |
|  | Indole and ipecac alkaloid biosynthesis | |  |  | 1 | 95 | 0 | 4567 | 0.020587604 | 0.020587604 | 1 |
|  |  | RAFL05-09-P03 | At1g74020 / strictosidine synthase family | |  |  |  |  |  | | --- | --- | --- | --- | --- | |  |  |  |  |  | | EC:4.3.3.2 | | | | | | |
|  | Glycerolipid metabolism | |  |  | 3 | 93 | 37 | 4530 | 0.048277758 | 1.0138329 | 21 |
|  |  | RAFL07-16-P10 | At1g77120 / alcohol dehydrogenase (ADH) | |  |  |  |  |  | | --- | --- | --- | --- | --- | |  |  |  |  |  | | EC:1.1.1.1 | | | | | | |
|  |  | RAFL05-18-O21 | At2g30550 / lipase (class 3) family | |  |  |  |  |  | | --- | --- | --- | --- | --- | |  |  |  |  |  | | EC:3.1.1.3 | | | | | | |
|  |  | RAFL08-09-J19 | At1g02660 / lipase (class 3) family | |  |  |  |  |  | | --- | --- | --- | --- | --- | |  |  |  |  |  | | EC:3.1.1.3 | | | | | | |
| Cluster:4-2 | | |  |  | A | B | C | D | P | P' | N |
|  | Selenoamino acid metabolism | |  |  | 3 | 132 | 25 | 4503 | 0.04587297 | 0.59634864 | 13 |
|  |  | RAFL05-08-P23 | At3g59980 / expressed protein | |  |  |  |  |  | | --- | --- | --- | --- | --- | |  |  |  |  |  | | EC:6.1.1.10 | | | | | | |
|  |  | RAFL11-03-C19 | At3g22890 / ATP sulfurylase -related | |  |  |  |  |  | | --- | --- | --- | --- | --- | |  |  |  |  |  | | EC:2.7.7.4 | | | | | | |
|  |  | RAFL04-17-C12 | At1g02500 / s-adenosylmethionine synthetase | |  |  |  |  |  | | --- | --- | --- | --- | --- | |  |  |  |  |  | | EC:2.5.1.6 | | | | | | |
| Cluster:2-0 | | |  |  | A | B | C | D | P | P' | N |
|  | Glutathione metabolism | |  |  | 5 | 145 | 23 | 4490 | 0.0017399205 | 0.033058487 | 19 |
|  |  | RAFL09-11-A18 | At1g65930 / isocitrate dehydrogenase (NADP+), putative | |  |  |  |  |  | | --- | --- | --- | --- | --- | |  |  |  |  |  | | EC:1.1.1.42 | | | | | | |
|  |  | RAFL03-05-I07 | At4g02520 / glutathione transferase, putative | |  |  |  |  |  | | --- | --- | --- | --- | --- | |  |  |  |  |  | | EC:2.5.1.18 | | | | | | |
|  |  | RAFL09-07-F20 | At1g65930 / isocitrate dehydrogenase (NADP+), putative | |  |  |  |  |  | | --- | --- | --- | --- | --- | |  |  |  |  |  | | EC:1.1.1.42 | | | | | | |
|  |  | RAFL09-06-L20 | At1g65930 / isocitrate dehydrogenase (NADP+), putative | |  |  |  |  |  | | --- | --- | --- | --- | --- | |  |  |  |  |  | | EC:1.1.1.42 | | | | | | |
|  |  | RAFL03-05-B08 | At2g30860 / glutathione transferase, putative | |  |  |  |  |  | | --- | --- | --- | --- | --- | |  |  |  |  |  | | EC:2.5.1.18 | | | | | | |
|  | Reductive carboxylate cycle (CO2 fixation) | |  |  | 3 | 147 | 23 | 4490 | 0.04939301 | 0.74089515 | 15 |
|  |  | RAFL09-11-A18 | At1g65930 / isocitrate dehydrogenase (NADP+), putative | |  |  |  |  |  | | --- | --- | --- | --- | --- | |  |  |  |  |  | | EC:1.1.1.42 | | | | | | |
|  |  | RAFL09-07-F20 | At1g65930 / isocitrate dehydrogenase (NADP+), putative | |  |  |  |  |  | | --- | --- | --- | --- | --- | |  |  |  |  |  | | EC:1.1.1.42 | | | | | | |
|  |  | RAFL09-06-L20 | At1g65930 / isocitrate dehydrogenase (NADP+), putative | |  |  |  |  |  | | --- | --- | --- | --- | --- | |  |  |  |  |  | | EC:1.1.1.42 | | | | | | |
| Cluster:3-0 | | |  |  | A | B | C | D | P | P' | N |
|  | Citrate cycle (TCA cycle) | |  |  | 6 | 227 | 30 | 4400 | 0.008043733 | 0.13674346 | 17 |
|  |  | RAFL07-14-B18 | At2g47510 / fumarase -related | |  |  |  |  |  | | --- | --- | --- | --- | --- | |  |  |  |  |  | | EC:4.2.1.2 | | | | | | |
|  |  | RAFL05-21-P13 | At2g42600 / phosphoenolpyruvate carboxylase | |  |  |  |  |  | | --- | --- | --- | --- | --- | |  |  |  |  |  | | EC:4.1.1.49 ,EC:4.1.1.32 | | | | | | |
|  |  | RAFL06-07-J21 | At1g04410 / malate dehydrogenase, cytosolic, putative | |  |  |  |  |  | | --- | --- | --- | --- | --- | |  |  |  |  |  | | EC:1.1.1.37 | | | | | | |
|  |  | RAFL07-17-M04 | At1g04410 / malate dehydrogenase, cytosolic, putative | |  |  |  |  |  | | --- | --- | --- | --- | --- | |  |  |  |  |  | | EC:1.1.1.37 | | | | | | |
|  |  | RAFL09-09-M02 | At3g47520 / malate dehydrogenase (NAD), chloroplast, putative | |  |  |  |  |  | | --- | --- | --- | --- | --- | |  |  |  |  |  | | EC:1.1.1.37 | | | | | | |
|  |  | RAFL05-15-O22 | At2g20420 / succinyl-CoA ligase beta subunit | |  |  |  |  |  | | --- | --- | --- | --- | --- | |  |  |  |  |  | | EC:6.2.1.4 | | | | | | |
|  | Reductive carboxylate cycle (CO2 fixation) | |  |  | 5 | 228 | 21 | 4409 | 0.0082831625 | 0.12424744 | 15 |
|  |  | RAFL07-14-B18 | At2g47510 / fumarase -related | |  |  |  |  |  | | --- | --- | --- | --- | --- | |  |  |  |  |  | | EC:4.2.1.2 | | | | | | |
|  |  | RAFL05-21-P13 | At2g42600 / phosphoenolpyruvate carboxylase | |  |  |  |  |  | | --- | --- | --- | --- | --- | |  |  |  |  |  | | EC:4.1.1.31 | | | | | | |
|  |  | RAFL06-07-J21 | At1g04410 / malate dehydrogenase, cytosolic, putative | |  |  |  |  |  | | --- | --- | --- | --- | --- | |  |  |  |  |  | | EC:1.1.1.37 | | | | | | |
|  |  | RAFL07-17-M04 | At1g04410 / malate dehydrogenase, cytosolic, putative | |  |  |  |  |  | | --- | --- | --- | --- | --- | |  |  |  |  |  | | EC:1.1.1.37 | | | | | | |
|  |  | RAFL09-09-M02 | At3g47520 / malate dehydrogenase (NAD), chloroplast, putative | |  |  |  |  |  | | --- | --- | --- | --- | --- | |  |  |  |  |  | | EC:1.1.1.37 | | | | | | |
|  | Pyruvate metabolism | |  |  | 7 | 226 | 48 | 4382 | 0.018589124 | 0.33460423 | 18 |
|  |  | RAFL05-21-P13 | At2g42600 / phosphoenolpyruvate carboxylase | |  |  |  |  |  | | --- | --- | --- | --- | --- | |  |  |  |  |  | | EC:4.1.1.49 ,EC:4.1.1.32 ,EC:4.1.1.31 | | | | | | |
|  |  | RAFL06-07-J21 | At1g04410 / malate dehydrogenase, cytosolic, putative | |  |  |  |  |  | | --- | --- | --- | --- | --- | |  |  |  |  |  | | EC:1.1.1.37 ,EC:1.1.99.16 | | | | | | |
|  |  | RAFL06-08-D17 | At5g43940 / alcohol dehydrogenase class III (glutathione-dependent formaldehyde dehydrogenase) (GSH-FDH) (ADHIII) | |  |  |  |  |  | | --- | --- | --- | --- | --- | |  |  |  |  |  | | EC:1.2.1.1 | | | | | | |
|  |  | RAFL07-17-M04 | At1g04410 / malate dehydrogenase, cytosolic, putative | |  |  |  |  |  | | --- | --- | --- | --- | --- | |  |  |  |  |  | | EC:1.1.1.37 ,EC:1.1.99.16 | | | | | | |
|  |  | RAFL07-10-F16 | At3g13930 / acetyltransferase -related | |  |  |  |  |  | | --- | --- | --- | --- | --- | |  |  |  |  |  | | EC:2.3.1.12 | | | | | | |
|  |  | RAFL09-09-M02 | At3g47520 / malate dehydrogenase (NAD), chloroplast, putative | |  |  |  |  |  | | --- | --- | --- | --- | --- | |  |  |  |  |  | | EC:1.1.1.37 ,EC:1.1.99.16 | | | | | | |
|  |  | RAFL09-17-A09 | At5g35360 / acetyl-CoA carboxylase | |  |  |  |  |  | | --- | --- | --- | --- | --- | |  |  |  |  |  | | EC:6.4.1.2 | | | | | | |
|  | Carbon fixation | |  |  | 9 | 224 | 72 | 4358 | 0.018764498 | 0.35652548 | 19 |
|  |  | RAFL05-21-O08 | At5g61410 / ribulose-5-phosphate-3-epimerase | |  |  |  |  |  | | --- | --- | --- | --- | --- | |  |  |  |  |  | | EC:5.1.3.1 | | | | | | |
|  |  | RAFL05-21-P13 | At2g42600 / phosphoenolpyruvate carboxylase | |  |  |  |  |  | | --- | --- | --- | --- | --- | |  |  |  |  |  | | EC:4.1.1.49 ,EC:4.1.1.31 | | | | | | |
|  |  | RAFL06-11-B16 | At3g52930 / fructose-bisphosphate aldolase, putative | |  |  |  |  |  | | --- | --- | --- | --- | --- | |  |  |  |  |  | | EC:4.1.2.13 | | | | | | |
|  |  | RAFL04-17-F02 | At2g01140 / fructose-bisphosphate aldolase, putative | |  |  |  |  |  | | --- | --- | --- | --- | --- | |  |  |  |  |  | | EC:4.1.2.13 | | | | | | |
|  |  | RAFL11-02-F16 | At3g01850 / D-ribulose-5-phosphate 3-epimerase -related | |  |  |  |  |  | | --- | --- | --- | --- | --- | |  |  |  |  |  | | EC:5.1.3.1 | | | | | | |
|  |  | RAFL06-07-J21 | At1g04410 / malate dehydrogenase, cytosolic, putative | |  |  |  |  |  | | --- | --- | --- | --- | --- | |  |  |  |  |  | | EC:1.1.1.37 | | | | | | |
|  |  | RAFL09-07-B08 | At2g30970 / aspartate aminotransferase, mitochondrial (transaminase A/Asp1) | |  |  |  |  |  | | --- | --- | --- | --- | --- | |  |  |  |  |  | | EC:2.6.1.1 | | | | | | |
|  |  | RAFL07-17-M04 | At1g04410 / malate dehydrogenase, cytosolic, putative | |  |  |  |  |  | | --- | --- | --- | --- | --- | |  |  |  |  |  | | EC:1.1.1.37 | | | | | | |
|  |  | RAFL09-09-M02 | At3g47520 / malate dehydrogenase (NAD), chloroplast, putative | |  |  |  |  |  | | --- | --- | --- | --- | --- | |  |  |  |  |  | | EC:1.1.1.37 | | | | | | |
|  | Pentose and glucuronate interconversions | |  |  | 3 | 230 | 12 | 4418 | 0.03588357 | 0.3588357 | 10 |
|  |  | RAFL05-21-O08 | At5g61410 / ribulose-5-phosphate-3-epimerase | |  |  |  |  |  | | --- | --- | --- | --- | --- | |  |  |  |  |  | | EC:5.1.3.1 | | | | | | |
|  |  | RAFL09-15-M18 | At3g03250 / UDP-glucose pyrophosphorylase -related | |  |  |  |  |  | | --- | --- | --- | --- | --- | |  |  |  |  |  | | EC:2.7.7.9 | | | | | | |
|  |  | RAFL11-02-F16 | At3g01850 / D-ribulose-5-phosphate 3-epimerase -related | |  |  |  |  |  | | --- | --- | --- | --- | --- | |  |  |  |  |  | | EC:5.1.3.1 | | | | | | |
| Cluster:2-2 | | |  |  | A | B | C | D | P | P' | N |
|  | Prostaglandin and leukotriene metabolism | |  |  | 2 | 51 | 1 | 4609 | 3.7755756E-4 | 7.551151E-4 | 2 |
|  |  | RAFL05-12-G03 | At5g42650 / allene oxide synthase / cytochrome P450 74A | |  |  |  |  |  | | --- | --- | --- | --- | --- | |  |  |  |  |  | | EC:4.2.1.92 | | | | | | |
|  |  | RAFL06-10-H13 | At5g42650 / allene oxide synthase / cytochrome P450 74A | |  |  |  |  |  | | --- | --- | --- | --- | --- | |  |  |  |  |  | | EC:4.2.1.92 | | | | | | |
|  | Sulfur metabolism | |  |  | 3 | 50 | 15 | 4595 | 0.0010030596 | 0.012036716 | 12 |
|  |  | RAFL04-17-H16 | At3g13110 / serine acetyltransferase (Sat-1) | |  |  |  |  |  | | --- | --- | --- | --- | --- | |  |  |  |  |  | | EC:2.3.1.30 | | | | | | |
|  |  | RAFL07-10-P11 | At3g22890 / ATP sulfurylase -related | |  |  |  |  |  | | --- | --- | --- | --- | --- | |  |  |  |  |  | | EC:2.7.7.4 | | | | | | |
|  |  | RAFL07-12-E10 | At3g22890 / ATP sulfurylase -related | |  |  |  |  |  | | --- | --- | --- | --- | --- | |  |  |  |  |  | | EC:2.7.7.4 | | | | | | |
|  | Inositol phosphate metabolism | |  |  | 2 | 51 | 5 | 4605 | 0.0025667707 | 0.015400624 | 6 |
|  |  | RAFL09-15-K07 | At4g39800 / myo-inositol-1-phosphate synthase | |  |  |  |  |  | | --- | --- | --- | --- | --- | |  |  |  |  |  | | EC:5.5.1.4 | | | | | | |
|  |  | RAFL09-14-L01 | At4g39800 / myo-inositol-1-phosphate synthase | |  |  |  |  |  | | --- | --- | --- | --- | --- | |  |  |  |  |  | | EC:5.5.1.4 | | | | | | |
|  | Purine metabolism | |  |  | 3 | 50 | 31 | 4579 | 0.0064710197 | 0.11000734 | 17 |
|  |  | RAFL04-20-F22 | At5g47840 / expressed protein | |  |  |  |  |  | | --- | --- | --- | --- | --- | |  |  |  |  |  | | EC:2.7.4.3 | | | | | | |
|  |  | RAFL07-10-P11 | At3g22890 / ATP sulfurylase -related | |  |  |  |  |  | | --- | --- | --- | --- | --- | |  |  |  |  |  | | EC:2.7.7.4 | | | | | | |
|  |  | RAFL07-12-E10 | At3g22890 / ATP sulfurylase -related | |  |  |  |  |  | | --- | --- | --- | --- | --- | |  |  |  |  |  | | EC:2.7.7.4 | | | | | | |
|  | Streptomycin biosynthesis | |  |  | 2 | 51 | 12 | 4598 | 0.010570335 | 0.08456268 | 8 |
|  |  | RAFL09-15-K07 | At4g39800 / myo-inositol-1-phosphate synthase | |  |  |  |  |  | | --- | --- | --- | --- | --- | |  |  |  |  |  | | EC:5.5.1.4 | | | | | | |
|  |  | RAFL09-14-L01 | At4g39800 / myo-inositol-1-phosphate synthase | |  |  |  |  |  | | --- | --- | --- | --- | --- | |  |  |  |  |  | | EC:5.5.1.4 | | | | | | |
|  | Taurine and hypotaurine metabolism | |  |  | 1 | 52 | 0 | 4610 | 0.011366073 | 0.011366073 | 1 |
|  |  | RAFL04-16-J21 | At1g65960 / glutamate decarboxylase 2 (GAD 2) | |  |  |  |  |  | | --- | --- | --- | --- | --- | |  |  |  |  |  | | EC:4.1.1.15 | | | | | | |
|  | Caprolactam degradation | |  |  | 1 | 52 | 0 | 4610 | 0.011366073 | 0.011366073 | 1 |
|  |  | RAFL04-09-D16 | At5g24420 / 6-phosphogluconolactonase-related protein | |  |  |  |  |  | | --- | --- | --- | --- | --- | |  |  |  |  |  | | EC:3.1.1.17 | | | | | | |
|  | Starch and sucrose metabolism | |  |  | 3 | 50 | 55 | 4555 | 0.027576564 | 0.6066844 | 22 |
|  |  | RAFL06-10-O06 | At1g53840 / pectinesterase family | |  |  |  |  |  | | --- | --- | --- | --- | --- | |  |  |  |  |  | | EC:3.1.1.11 | | | | | | |
|  |  | RAFL06-16-M17 | At4g17090 / glycosyl hydrolase family 14 (beta-amylase) | |  |  |  |  |  | | --- | --- | --- | --- | --- | |  |  |  |  |  | | EC:3.2.1.2 | | | | | | |
|  |  | RAFL05-07-J12 | At1g66430 / pfkB type carbohydrate kinase protein family | |  |  |  |  |  | | --- | --- | --- | --- | --- | |  |  |  |  |  | | EC:2.7.1.4 | | | | | | |
|  | Selenoamino acid metabolism | |  |  | 2 | 51 | 26 | 4584 | 0.039688654 | 0.5159525 | 13 |
|  |  | RAFL07-10-P11 | At3g22890 / ATP sulfurylase -related | |  |  |  |  |  | | --- | --- | --- | --- | --- | |  |  |  |  |  | | EC:2.7.7.4 | | | | | | |
|  |  | RAFL07-12-E10 | At3g22890 / ATP sulfurylase -related | |  |  |  |  |  | | --- | --- | --- | --- | --- | |  |  |  |  |  | | EC:2.7.7.4 | | | | | | |
|  | Peptidoglycan biosynthesis | |  |  | 1 | 52 | 3 | 4607 | 0.044709165 | 0.13412748 | 3 |
|  |  | RAFL04-16-N11 | At5g35630 / glutamate-ammonia ligase (EC 6.3.1.2) precursor, chloroplast (clone lambdaAtgsl1) (pir||S18600) | |  |  |  |  |  | | --- | --- | --- | --- | --- | |  |  |  |  |  | | EC:6.3.1.2 | | | | | | |
| Cluster:1-0 | | |  |  | A | B | C | D | P | P' | N |
|  | Glyoxylate and dicarboxylate metabolism | |  |  | 6 | 143 | 43 | 4471 | 0.0043278704 | 0.06924593 | 16 |
|  |  | RAFL04-13-N06 | At1g68010 / glycerate dehydrogenase (NADH-dependent hydroxypyruvate reductase) (HPR) | |  |  |  |  |  | | --- | --- | --- | --- | --- | |  |  |  |  |  | | EC:1.1.1.81 ,EC:1.1.1.29 | | | | | | |
|  |  | RAFL11-05-D24 | At1g67090 / ribulose-bisphosphate carboxylase small unit -related | |  |  |  |  |  | | --- | --- | --- | --- | --- | |  |  |  |  |  | | EC:4.1.1.39 | | | | | | |
|  |  | RAFL06-14-K21 | At5g36700 / phosphoglycolate phosphatase, putative | |  |  |  |  |  | | --- | --- | --- | --- | --- | |  |  |  |  |  | | EC:3.1.3.18 | | | | | | |
|  |  | RAFL08-15-E10 | At3g14420 / glycolate oxidase -related | |  |  |  |  |  | | --- | --- | --- | --- | --- | |  |  |  |  |  | | EC:1.1.3.15 | | | | | | |
|  |  | RAFL05-03-H12 | At5g09660 / malate dehydrogenase, glyoxysomal | |  |  |  |  |  | | --- | --- | --- | --- | --- | |  |  |  |  |  | | EC:1.1.1.37 | | | | | | |
|  |  | RAFL09-13-P20 | At3g14420 / glycolate oxidase -related | |  |  |  |  |  | | --- | --- | --- | --- | --- | |  |  |  |  |  | | EC:1.1.3.15 | | | | | | |
|  | Clavulanic acid biosynthesis | |  |  | 1 | 148 | 0 | 4514 | 0.031953678 | 0.031953678 | 1 |
|  |  | RAFL05-18-H22 | At4g08870 / arginase -related | |  |  |  |  |  | | --- | --- | --- | --- | --- | |  |  |  |  |  | | EC:3.5.3.11 | | | | | | |
| Cluster:8-0 | | |  |  | A | B | C | D | P | P' | N |
|  | Benzoate degradation via CoA ligation | |  |  | 4 | 105 | 10 | 4544 | 2.3608547E-4 | 0.0016525983 | 7 |
|  |  | RAFL06-13-H12 | At3g51840 / acyl-coA dehydrogenase | |  |  |  |  |  | | --- | --- | --- | --- | --- | |  |  |  |  |  | | EC:1.3.99.7 | | | | | | |
|  |  | RAFL06-13-E03 | At3g44300 / nitrilase 2 | |  |  |  |  |  | | --- | --- | --- | --- | --- | |  |  |  |  |  | | EC:3.5.5.1 | | | | | | |
|  |  | RAFL04-12-F14 | At3g27380 / succinate dehydrogenase, iron-sulphur subunit, mitochondrial (sdh2-1) | |  |  |  |  |  | | --- | --- | --- | --- | --- | |  |  |  |  |  | | EC:1.3.99.1 | | | | | | |
|  |  | RAFL08-10-H06 | At3g44300 / nitrilase 2 | |  |  |  |  |  | | --- | --- | --- | --- | --- | |  |  |  |  |  | | EC:3.5.5.1 | | | | | | |
|  | Tryptophan metabolism | |  |  | 4 | 105 | 20 | 4534 | 0.0020926541 | 0.027204502 | 13 |
|  |  | RAFL03-05-E06 | At3g48000 / mitochondrial aldehyde dehydrogenase, putative (ALDH) | |  |  |  |  |  | | --- | --- | --- | --- | --- | |  |  |  |  |  | | EC:1.2.1.3 | | | | | | |
|  |  | RAFL06-13-H12 | At3g51840 / acyl-coA dehydrogenase | |  |  |  |  |  | | --- | --- | --- | --- | --- | |  |  |  |  |  | | EC:1.3.99.7 | | | | | | |
|  |  | RAFL06-13-E03 | At3g44300 / nitrilase 2 | |  |  |  |  |  | | --- | --- | --- | --- | --- | |  |  |  |  |  | | EC:3.5.5.1 | | | | | | |
|  |  | RAFL08-10-H06 | At3g44300 / nitrilase 2 | |  |  |  |  |  | | --- | --- | --- | --- | --- | |  |  |  |  |  | | EC:3.5.5.1 | | | | | | |
|  | Histidine metabolism | |  |  | 3 | 106 | 13 | 4541 | 0.0055737486 | 0.061311234 | 11 |
|  |  | RAFL03-05-E06 | At3g48000 / mitochondrial aldehyde dehydrogenase, putative (ALDH) | |  |  |  |  |  | | --- | --- | --- | --- | --- | |  |  |  |  |  | | EC:1.2.1.3 | | | | | | |
|  |  | RAFL05-09-B02 | At5g53970 / aminotransferase, putative | |  |  |  |  |  | | --- | --- | --- | --- | --- | |  |  |  |  |  | | EC:2.6.1.9 | | | | | | |
|  |  | RAFL07-10-M07 | At5g53970 / aminotransferase, putative | |  |  |  |  |  | | --- | --- | --- | --- | --- | |  |  |  |  |  | | EC:2.6.1.9 | | | | | | |
|  | Alkaloid biosynthesis I | |  |  | 2 | 107 | 7 | 4547 | 0.017509907 | 0.10505944 | 6 |
|  |  | RAFL05-09-B02 | At5g53970 / aminotransferase, putative | |  |  |  |  |  | | --- | --- | --- | --- | --- | |  |  |  |  |  | | EC:2.6.1.5 | | | | | | |
|  |  | RAFL07-10-M07 | At5g53970 / aminotransferase, putative | |  |  |  |  |  | | --- | --- | --- | --- | --- | |  |  |  |  |  | | EC:2.6.1.5 | | | | | | |
|  | Thiamine metabolism | |  |  | 1 | 108 | 1 | 4553 | 0.0462095 | 0.092419 | 2 |
|  |  | RAFL07-08-G02 | At3g04080 / apyrase (Atapy1) | |  |  |  |  |  | | --- | --- | --- | --- | --- | |  |  |  |  |  | | EC:3.6.1.15 | | | | | | |
|  | Cyanoamino acid metabolism | |  |  | 2 | 107 | 13 | 4541 | 0.04663169 | 0.4663169 | 10 |
|  |  | RAFL06-13-E03 | At3g44300 / nitrilase 2 | |  |  |  |  |  | | --- | --- | --- | --- | --- | |  |  |  |  |  | | EC:3.5.5.1 | | | | | | |
|  |  | RAFL08-10-H06 | At3g44300 / nitrilase 2 | |  |  |  |  |  | | --- | --- | --- | --- | --- | |  |  |  |  |  | | EC:3.5.5.1 | | | | | | |
| Cluster:9-0 | | |  |  | A | B | C | D | P | P' | N |
|  | 1,2-Dichloroethane degradation | |  |  | 4 | 28 | 2 | 4629 | 2.7153972E-8 | 8.146192E-8 | 3 |
|  |  | RAFL04-09-D07 | At1g54100 / aldehyde dehydrogenase, putative (ALDH) | |  |  |  |  |  | | --- | --- | --- | --- | --- | |  |  |  |  |  | | EC:1.2.1.3 | | | | | | |
|  |  | RAFL05-21-E06 | At1g54100 / aldehyde dehydrogenase, putative (ALDH) | |  |  |  |  |  | | --- | --- | --- | --- | --- | |  |  |  |  |  | | EC:1.2.1.3 | | | | | | |
|  |  | RAFL08-15-L09 | At1g54100 / aldehyde dehydrogenase, putative (ALDH) | |  |  |  |  |  | | --- | --- | --- | --- | --- | |  |  |  |  |  | | EC:1.2.1.3 | | | | | | |
|  |  | RAFL08-09-C23 | At1g54100 / aldehyde dehydrogenase, putative (ALDH) | |  |  |  |  |  | | --- | --- | --- | --- | --- | |  |  |  |  |  | | EC:1.2.1.3 | | | | | | |
|  | Bile acid biosynthesis | |  |  | 4 | 28 | 5 | 4626 | 2.2481943E-7 | 1.3489166E-6 | 6 |
|  |  | RAFL04-09-D07 | At1g54100 / aldehyde dehydrogenase, putative (ALDH) | |  |  |  |  |  | | --- | --- | --- | --- | --- | |  |  |  |  |  | | EC:1.2.1.3 | | | | | | |
|  |  | RAFL05-21-E06 | At1g54100 / aldehyde dehydrogenase, putative (ALDH) | |  |  |  |  |  | | --- | --- | --- | --- | --- | |  |  |  |  |  | | EC:1.2.1.3 | | | | | | |
|  |  | RAFL08-15-L09 | At1g54100 / aldehyde dehydrogenase, putative (ALDH) | |  |  |  |  |  | | --- | --- | --- | --- | --- | |  |  |  |  |  | | EC:1.2.1.3 | | | | | | |
|  |  | RAFL08-09-C23 | At1g54100 / aldehyde dehydrogenase, putative (ALDH) | |  |  |  |  |  | | --- | --- | --- | --- | --- | |  |  |  |  |  | | EC:1.2.1.3 | | | | | | |
|  | beta-Alanine metabolism | |  |  | 5 | 27 | 17 | 4614 | 2.664087E-7 | 3.1969046E-6 | 12 |
|  |  | RAFL05-08-B14 | At2g38400 / alanine--glyoxylate aminotransferase (beta-alanine-pyruvate aminotransferase/AGT), putative | |  |  |  |  |  | | --- | --- | --- | --- | --- | |  |  |  |  |  | | EC:2.6.1.19 | | | | | | |
|  |  | RAFL04-09-D07 | At1g54100 / aldehyde dehydrogenase, putative (ALDH) | |  |  |  |  |  | | --- | --- | --- | --- | --- | |  |  |  |  |  | | EC:1.2.1.3 | | | | | | |
|  |  | RAFL05-21-E06 | At1g54100 / aldehyde dehydrogenase, putative (ALDH) | |  |  |  |  |  | | --- | --- | --- | --- | --- | |  |  |  |  |  | | EC:1.2.1.3 | | | | | | |
|  |  | RAFL08-15-L09 | At1g54100 / aldehyde dehydrogenase, putative (ALDH) | |  |  |  |  |  | | --- | --- | --- | --- | --- | |  |  |  |  |  | | EC:1.2.1.3 | | | | | | |
|  |  | RAFL08-09-C23 | At1g54100 / aldehyde dehydrogenase, putative (ALDH) | |  |  |  |  |  | | --- | --- | --- | --- | --- | |  |  |  |  |  | | EC:1.2.1.3 | | | | | | |
|  | Propanoate metabolism | |  |  | 5 | 27 | 18 | 4613 | 3.387656E-7 | 4.7427184E-6 | 14 |
|  |  | RAFL05-08-B14 | At2g38400 / alanine--glyoxylate aminotransferase (beta-alanine-pyruvate aminotransferase/AGT), putative | |  |  |  |  |  | | --- | --- | --- | --- | --- | |  |  |  |  |  | | EC:2.6.1.19 | | | | | | |
|  |  | RAFL04-09-D07 | At1g54100 / aldehyde dehydrogenase, putative (ALDH) | |  |  |  |  |  | | --- | --- | --- | --- | --- | |  |  |  |  |  | | EC:1.2.1.3 | | | | | | |
|  |  | RAFL05-21-E06 | At1g54100 / aldehyde dehydrogenase, putative (ALDH) | |  |  |  |  |  | | --- | --- | --- | --- | --- | |  |  |  |  |  | | EC:1.2.1.3 | | | | | | |
|  |  | RAFL08-15-L09 | At1g54100 / aldehyde dehydrogenase, putative (ALDH) | |  |  |  |  |  | | --- | --- | --- | --- | --- | |  |  |  |  |  | | EC:1.2.1.3 | | | | | | |
|  |  | RAFL08-09-C23 | At1g54100 / aldehyde dehydrogenase, putative (ALDH) | |  |  |  |  |  | | --- | --- | --- | --- | --- | |  |  |  |  |  | | EC:1.2.1.3 | | | | | | |
|  | Ascorbate and aldarate metabolism | |  |  | 4 | 28 | 6 | 4625 | 3.7289774E-7 | 2.6102844E-6 | 7 |
|  |  | RAFL04-09-D07 | At1g54100 / aldehyde dehydrogenase, putative (ALDH) | |  |  |  |  |  | | --- | --- | --- | --- | --- | |  |  |  |  |  | | EC:1.2.1.3 | | | | | | |
|  |  | RAFL05-21-E06 | At1g54100 / aldehyde dehydrogenase, putative (ALDH) | |  |  |  |  |  | | --- | --- | --- | --- | --- | |  |  |  |  |  | | EC:1.2.1.3 | | | | | | |
|  |  | RAFL08-15-L09 | At1g54100 / aldehyde dehydrogenase, putative (ALDH) | |  |  |  |  |  | | --- | --- | --- | --- | --- | |  |  |  |  |  | | EC:1.2.1.3 | | | | | | |
|  |  | RAFL08-09-C23 | At1g54100 / aldehyde dehydrogenase, putative (ALDH) | |  |  |  |  |  | | --- | --- | --- | --- | --- | |  |  |  |  |  | | EC:1.2.1.3 | | | | | | |
|  | Valine, leucine and isoleucine degradation | |  |  | 4 | 28 | 10 | 4621 | 1.7435473E-6 | 1.5691925E-5 | 9 |
|  |  | RAFL04-09-D07 | At1g54100 / aldehyde dehydrogenase, putative (ALDH) | |  |  |  |  |  | | --- | --- | --- | --- | --- | |  |  |  |  |  | | EC:1.2.1.3 | | | | | | |
|  |  | RAFL05-21-E06 | At1g54100 / aldehyde dehydrogenase, putative (ALDH) | |  |  |  |  |  | | --- | --- | --- | --- | --- | |  |  |  |  |  | | EC:1.2.1.3 | | | | | | |
|  |  | RAFL08-15-L09 | At1g54100 / aldehyde dehydrogenase, putative (ALDH) | |  |  |  |  |  | | --- | --- | --- | --- | --- | |  |  |  |  |  | | EC:1.2.1.3 | | | | | | |
|  |  | RAFL08-09-C23 | At1g54100 / aldehyde dehydrogenase, putative (ALDH) | |  |  |  |  |  | | --- | --- | --- | --- | --- | |  |  |  |  |  | | EC:1.2.1.3 | | | | | | |
|  | Butanoate metabolism | |  |  | 5 | 27 | 28 | 4603 | 2.2763622E-6 | 3.6421796E-5 | 16 |
|  |  | RAFL05-08-B14 | At2g38400 / alanine--glyoxylate aminotransferase (beta-alanine-pyruvate aminotransferase/AGT), putative | |  |  |  |  |  | | --- | --- | --- | --- | --- | |  |  |  |  |  | | EC:2.6.1.19 | | | | | | |
|  |  | RAFL04-09-D07 | At1g54100 / aldehyde dehydrogenase, putative (ALDH) | |  |  |  |  |  | | --- | --- | --- | --- | --- | |  |  |  |  |  | | EC:1.2.1.3 | | | | | | |
|  |  | RAFL05-21-E06 | At1g54100 / aldehyde dehydrogenase, putative (ALDH) | |  |  |  |  |  | | --- | --- | --- | --- | --- | |  |  |  |  |  | | EC:1.2.1.3 | | | | | | |
|  |  | RAFL08-15-L09 | At1g54100 / aldehyde dehydrogenase, putative (ALDH) | |  |  |  |  |  | | --- | --- | --- | --- | --- | |  |  |  |  |  | | EC:1.2.1.3 | | | | | | |
|  |  | RAFL08-09-C23 | At1g54100 / aldehyde dehydrogenase, putative (ALDH) | |  |  |  |  |  | | --- | --- | --- | --- | --- | |  |  |  |  |  | | EC:1.2.1.3 | | | | | | |
|  | Lysine degradation | |  |  | 4 | 28 | 12 | 4619 | 3.139684E-6 | 3.4536522E-5 | 11 |
|  |  | RAFL04-09-D07 | At1g54100 / aldehyde dehydrogenase, putative (ALDH) | |  |  |  |  |  | | --- | --- | --- | --- | --- | |  |  |  |  |  | | EC:1.2.1.3 | | | | | | |
|  |  | RAFL05-21-E06 | At1g54100 / aldehyde dehydrogenase, putative (ALDH) | |  |  |  |  |  | | --- | --- | --- | --- | --- | |  |  |  |  |  | | EC:1.2.1.3 | | | | | | |
|  |  | RAFL08-15-L09 | At1g54100 / aldehyde dehydrogenase, putative (ALDH) | |  |  |  |  |  | | --- | --- | --- | --- | --- | |  |  |  |  |  | | EC:1.2.1.3 | | | | | | |
|  |  | RAFL08-09-C23 | At1g54100 / aldehyde dehydrogenase, putative (ALDH) | |  |  |  |  |  | | --- | --- | --- | --- | --- | |  |  |  |  |  | | EC:1.2.1.3 | | | | | | |
|  | Histidine metabolism | |  |  | 4 | 28 | 12 | 4619 | 3.139684E-6 | 3.4536522E-5 | 11 |
|  |  | RAFL04-09-D07 | At1g54100 / aldehyde dehydrogenase, putative (ALDH) | |  |  |  |  |  | | --- | --- | --- | --- | --- | |  |  |  |  |  | | EC:1.2.1.3 | | | | | | |
|  |  | RAFL05-21-E06 | At1g54100 / aldehyde dehydrogenase, putative (ALDH) | |  |  |  |  |  | | --- | --- | --- | --- | --- | |  |  |  |  |  | | EC:1.2.1.3 | | | | | | |
|  |  | RAFL08-15-L09 | At1g54100 / aldehyde dehydrogenase, putative (ALDH) | |  |  |  |  |  | | --- | --- | --- | --- | --- | |  |  |  |  |  | | EC:1.2.1.3 | | | | | | |
|  |  | RAFL08-09-C23 | At1g54100 / aldehyde dehydrogenase, putative (ALDH) | |  |  |  |  |  | | --- | --- | --- | --- | --- | |  |  |  |  |  | | EC:1.2.1.3 | | | | | | |
|  | Urea cycle and metabolism of amino groups | |  |  | 4 | 28 | 18 | 4613 | 1.2259562E-5 | 1.4711474E-4 | 12 |
|  |  | RAFL04-09-D07 | At1g54100 / aldehyde dehydrogenase, putative (ALDH) | |  |  |  |  |  | | --- | --- | --- | --- | --- | |  |  |  |  |  | | EC:1.2.1.41 | | | | | | |
|  |  | RAFL05-21-E06 | At1g54100 / aldehyde dehydrogenase, putative (ALDH) | |  |  |  |  |  | | --- | --- | --- | --- | --- | |  |  |  |  |  | | EC:1.2.1.41 | | | | | | |
|  |  | RAFL08-15-L09 | At1g54100 / aldehyde dehydrogenase, putative (ALDH) | |  |  |  |  |  | | --- | --- | --- | --- | --- | |  |  |  |  |  | | EC:1.2.1.41 | | | | | | |
|  |  | RAFL08-09-C23 | At1g54100 / aldehyde dehydrogenase, putative (ALDH) | |  |  |  |  |  | | --- | --- | --- | --- | --- | |  |  |  |  |  | | EC:1.2.1.41 | | | | | | |
|  | Glutamate metabolism | |  |  | 5 | 27 | 42 | 4589 | 1.3746837E-5 | 2.7493676E-4 | 20 |
|  |  | RAFL05-08-B14 | At2g38400 / alanine--glyoxylate aminotransferase (beta-alanine-pyruvate aminotransferase/AGT), putative | |  |  |  |  |  | | --- | --- | --- | --- | --- | |  |  |  |  |  | | EC:2.6.1.19 | | | | | | |
|  |  | RAFL04-09-D07 | At1g54100 / aldehyde dehydrogenase, putative (ALDH) | |  |  |  |  |  | | --- | --- | --- | --- | --- | |  |  |  |  |  | | EC:1.5.1.12 | | | | | | |
|  |  | RAFL05-21-E06 | At1g54100 / aldehyde dehydrogenase, putative (ALDH) | |  |  |  |  |  | | --- | --- | --- | --- | --- | |  |  |  |  |  | | EC:1.5.1.12 | | | | | | |
|  |  | RAFL08-15-L09 | At1g54100 / aldehyde dehydrogenase, putative (ALDH) | |  |  |  |  |  | | --- | --- | --- | --- | --- | |  |  |  |  |  | | EC:1.5.1.12 | | | | | | |
|  |  | RAFL08-09-C23 | At1g54100 / aldehyde dehydrogenase, putative (ALDH) | |  |  |  |  |  | | --- | --- | --- | --- | --- | |  |  |  |  |  | | EC:1.5.1.12 | | | | | | |
|  | Fatty acid metabolism | |  |  | 4 | 28 | 19 | 4612 | 1.4769203E-5 | 2.2153804E-4 | 15 |
|  |  | RAFL04-09-D07 | At1g54100 / aldehyde dehydrogenase, putative (ALDH) | |  |  |  |  |  | | --- | --- | --- | --- | --- | |  |  |  |  |  | | EC:1.2.1.3 | | | | | | |
|  |  | RAFL05-21-E06 | At1g54100 / aldehyde dehydrogenase, putative (ALDH) | |  |  |  |  |  | | --- | --- | --- | --- | --- | |  |  |  |  |  | | EC:1.2.1.3 | | | | | | |
|  |  | RAFL08-15-L09 | At1g54100 / aldehyde dehydrogenase, putative (ALDH) | |  |  |  |  |  | | --- | --- | --- | --- | --- | |  |  |  |  |  | | EC:1.2.1.3 | | | | | | |
|  |  | RAFL08-09-C23 | At1g54100 / aldehyde dehydrogenase, putative (ALDH) | |  |  |  |  |  | | --- | --- | --- | --- | --- | |  |  |  |  |  | | EC:1.2.1.3 | | | | | | |
|  | Tryptophan metabolism | |  |  | 4 | 28 | 20 | 4611 | 1.7637874E-5 | 2.2929236E-4 | 13 |
|  |  | RAFL04-09-D07 | At1g54100 / aldehyde dehydrogenase, putative (ALDH) | |  |  |  |  |  | | --- | --- | --- | --- | --- | |  |  |  |  |  | | EC:1.2.1.3 | | | | | | |
|  |  | RAFL05-21-E06 | At1g54100 / aldehyde dehydrogenase, putative (ALDH) | |  |  |  |  |  | | --- | --- | --- | --- | --- | |  |  |  |  |  | | EC:1.2.1.3 | | | | | | |
|  |  | RAFL08-15-L09 | At1g54100 / aldehyde dehydrogenase, putative (ALDH) | |  |  |  |  |  | | --- | --- | --- | --- | --- | |  |  |  |  |  | | EC:1.2.1.3 | | | | | | |
|  |  | RAFL08-09-C23 | At1g54100 / aldehyde dehydrogenase, putative (ALDH) | |  |  |  |  |  | | --- | --- | --- | --- | --- | |  |  |  |  |  | | EC:1.2.1.3 | | | | | | |
|  | Glycerolipid metabolism | |  |  | 4 | 28 | 36 | 4595 | 1.404465E-4 | 0.0029493764 | 21 |
|  |  | RAFL04-09-D07 | At1g54100 / aldehyde dehydrogenase, putative (ALDH) | |  |  |  |  |  | | --- | --- | --- | --- | --- | |  |  |  |  |  | | EC:1.2.1.3 | | | | | | |
|  |  | RAFL05-21-E06 | At1g54100 / aldehyde dehydrogenase, putative (ALDH) | |  |  |  |  |  | | --- | --- | --- | --- | --- | |  |  |  |  |  | | EC:1.2.1.3 | | | | | | |
|  |  | RAFL08-15-L09 | At1g54100 / aldehyde dehydrogenase, putative (ALDH) | |  |  |  |  |  | | --- | --- | --- | --- | --- | |  |  |  |  |  | | EC:1.2.1.3 | | | | | | |
|  |  | RAFL08-09-C23 | At1g54100 / aldehyde dehydrogenase, putative (ALDH) | |  |  |  |  |  | | --- | --- | --- | --- | --- | |  |  |  |  |  | | EC:1.2.1.3 | | | | | | |
|  | Arginine and proline metabolism | |  |  | 4 | 28 | 40 | 4591 | 2.0464069E-4 | 0.0042974544 | 21 |
|  |  | RAFL04-09-D07 | At1g54100 / aldehyde dehydrogenase, putative (ALDH) | |  |  |  |  |  | | --- | --- | --- | --- | --- | |  |  |  |  |  | | EC:1.2.1.3 ,EC:1.5.1.12 | | | | | | |
|  |  | RAFL05-21-E06 | At1g54100 / aldehyde dehydrogenase, putative (ALDH) | |  |  |  |  |  | | --- | --- | --- | --- | --- | |  |  |  |  |  | | EC:1.2.1.3 ,EC:1.5.1.12 | | | | | | |
|  |  | RAFL08-15-L09 | At1g54100 / aldehyde dehydrogenase, putative (ALDH) | |  |  |  |  |  | | --- | --- | --- | --- | --- | |  |  |  |  |  | | EC:1.2.1.3 ,EC:1.5.1.12 | | | | | | |
|  |  | RAFL08-09-C23 | At1g54100 / aldehyde dehydrogenase, putative (ALDH) | |  |  |  |  |  | | --- | --- | --- | --- | --- | |  |  |  |  |  | | EC:1.2.1.3 ,EC:1.5.1.12 | | | | | | |
|  | Pyruvate metabolism | |  |  | 4 | 28 | 51 | 4580 | 4.876191E-4 | 0.008777143 | 18 |
|  |  | RAFL04-09-D07 | At1g54100 / aldehyde dehydrogenase, putative (ALDH) | |  |  |  |  |  | | --- | --- | --- | --- | --- | |  |  |  |  |  | | EC:1.2.1.3 | | | | | | |
|  |  | RAFL05-21-E06 | At1g54100 / aldehyde dehydrogenase, putative (ALDH) | |  |  |  |  |  | | --- | --- | --- | --- | --- | |  |  |  |  |  | | EC:1.2.1.3 | | | | | | |
|  |  | RAFL08-15-L09 | At1g54100 / aldehyde dehydrogenase, putative (ALDH) | |  |  |  |  |  | | --- | --- | --- | --- | --- | |  |  |  |  |  | | EC:1.2.1.3 | | | | | | |
|  |  | RAFL08-09-C23 | At1g54100 / aldehyde dehydrogenase, putative (ALDH) | |  |  |  |  |  | | --- | --- | --- | --- | --- | |  |  |  |  |  | | EC:1.2.1.3 | | | | | | |
|  | Glycolysis / Gluconeogenesis | |  |  | 4 | 28 | 71 | 4560 | 0.0015783928 | 0.03314625 | 21 |
|  |  | RAFL04-09-D07 | At1g54100 / aldehyde dehydrogenase, putative (ALDH) | |  |  |  |  |  | | --- | --- | --- | --- | --- | |  |  |  |  |  | | EC:1.2.1.3 | | | | | | |
|  |  | RAFL05-21-E06 | At1g54100 / aldehyde dehydrogenase, putative (ALDH) | |  |  |  |  |  | | --- | --- | --- | --- | --- | |  |  |  |  |  | | EC:1.2.1.3 | | | | | | |
|  |  | RAFL08-15-L09 | At1g54100 / aldehyde dehydrogenase, putative (ALDH) | |  |  |  |  |  | | --- | --- | --- | --- | --- | |  |  |  |  |  | | EC:1.2.1.3 | | | | | | |
|  |  | RAFL08-09-C23 | At1g54100 / aldehyde dehydrogenase, putative (ALDH) | |  |  |  |  |  | | --- | --- | --- | --- | --- | |  |  |  |  |  | | EC:1.2.1.3 | | | | | | |
|  | Flavonoids, stilbene and lignin biosynthesis | |  |  | 3 | 29 | 33 | 4598 | 0.0017977278 | 0.03415683 | 19 |
|  |  | RAFL06-15-H16 | At1g09500 / cinnamyl-alcohol dehydrogenase (CAD) family | |  |  |  |  |  | | --- | --- | --- | --- | --- | |  |  |  |  |  | | EC:1.1.1.195 | | | | | | |
|  |  | RAFL05-18-A06 | At1g09500 / cinnamyl-alcohol dehydrogenase (CAD) family | |  |  |  |  |  | | --- | --- | --- | --- | --- | |  |  |  |  |  | | EC:1.1.1.195 | | | | | | |
|  |  | RAFL05-14-E15 | At2g33590 / cinnamoyl-CoA reductase family | |  |  |  |  |  | | --- | --- | --- | --- | --- | |  |  |  |  |  | | EC:1.2.1.44 | | | | | | |
|  | Biotin metabolism | |  |  | 1 | 31 | 3 | 4628 | 0.027177518 | 0.08153255 | 3 |
|  |  | RAFL05-08-B14 | At2g38400 / alanine--glyoxylate aminotransferase (beta-alanine-pyruvate aminotransferase/AGT), putative | |  |  |  |  |  | | --- | --- | --- | --- | --- | |  |  |  |  |  | | EC:2.6.1.62 | | | | | | |
| Cluster:6-1 | | |  |  | A | B | C | D | P | P' | N |
|  | Benzoate degradation via CoA ligation | |  |  | 4 | 311 | 10 | 4338 | 0.011896291 | 0.08327404 | 7 |
|  |  | RAFL09-14-D16 | At5g22300 / Nitrilase 4 (sp P46011) | |  |  |  |  |  | | --- | --- | --- | --- | --- | |  |  |  |  |  | | EC:4.2.1.84 ,EC:3.5.5.1 | | | | | | |
|  |  | RAFL05-17-A04 | At5g09600 / expressed protein | |  |  |  |  |  | | --- | --- | --- | --- | --- | |  |  |  |  |  | | EC:1.3.99.1 | | | | | | |
|  |  | RAFL07-15-A09 | At3g06860 / fatty acid multifunctional protein (AtMFP2) | |  |  |  |  |  | | --- | --- | --- | --- | --- | |  |  |  |  |  | | EC:4.2.1.17 | | | | | | |
|  |  | RAFL11-10-D22 | At5g43280 / enoyl-CoA hydratase/isomerase family | |  |  |  |  |  | | --- | --- | --- | --- | --- | |  |  |  |  |  | | EC:4.2.1.17 | | | | | | |
|  | Alkaloid biosynthesis I | |  |  | 3 | 312 | 6 | 4342 | 0.018891884 | 0.1133513 | 6 |
|  |  | RAFL04-17-L05 | At5g19550 / aspartate aminotransferase, cytoplasmic isozyme 1 (transaminase A/Asp2) | |  |  |  |  |  | | --- | --- | --- | --- | --- | |  |  |  |  |  | | EC:2.6.1.1 | | | | | | |
|  |  | RAFL06-11-D08 | At5g36160 / tyrosine aminotransferase-related protein | |  |  |  |  |  | | --- | --- | --- | --- | --- | |  |  |  |  |  | | EC:2.6.1.5 | | | | | | |
|  |  | RAFL11-09-A12 | At5g19550 / aspartate aminotransferase, cytoplasmic isozyme 1 (transaminase A/Asp2) | |  |  |  |  |  | | --- | --- | --- | --- | --- | |  |  |  |  |  | | EC:2.6.1.1 | | | | | | |
|  | Fatty acid biosynthesis (path 2) | |  |  | 2 | 313 | 2 | 4346 | 0.024916494 | 0.074749485 | 3 |
|  |  | RAFL07-15-A09 | At3g06860 / fatty acid multifunctional protein (AtMFP2) | |  |  |  |  |  | | --- | --- | --- | --- | --- | |  |  |  |  |  | | EC:4.2.1.17 | | | | | | |
|  |  | RAFL11-10-D22 | At5g43280 / enoyl-CoA hydratase/isomerase family | |  |  |  |  |  | | --- | --- | --- | --- | --- | |  |  |  |  |  | | EC:4.2.1.17 | | | | | | |
|  | Ubiquinone biosynthesis | |  |  | 3 | 312 | 8 | 4340 | 0.033555806 | 0.13422322 | 4 |
|  |  | RAFL07-07-N09 | At5g37510 / NADH dehydrogenase (ubiquinone), mitochondrial, putative | |  |  |  |  |  | | --- | --- | --- | --- | --- | |  |  |  |  |  | | EC:1.6.5.3 | | | | | | |
|  |  | RAFL05-08-F21 | At3g03100 / expressed protein | |  |  |  |  |  | | --- | --- | --- | --- | --- | |  |  |  |  |  | | EC:1.6.5.3 | | | | | | |
|  |  | RAFL06-10-E05 | At1g16700 / NADH:ubiquinone oxidoreductase -related | |  |  |  |  |  | | --- | --- | --- | --- | --- | |  |  |  |  |  | | EC:1.6.5.3 | | | | | | |
| Cluster:0-2 | | |  |  | A | B | C | D | P | P' | N |
|  | Glycine, serine and threonine metabolism | |  |  | 8 | 71 | 34 | 4550 | 3.512669E-7 | 5.6202703E-6 | 16 |
|  |  | RAFL09-09-C13 | At4g33010 / glycine dehydrogenase (decarboxylating) (glycine decarboxylase/glycine cleavage system P-protein), putative | |  |  |  |  |  | | --- | --- | --- | --- | --- | |  |  |  |  |  | | EC:1.4.4.2 | | | | | | |
|  |  | RAFL09-06-E16 | At2g26080 / glycine dehydrogenase (decarboxylating) (glycine decarboxylase/glycine cleavage system P-protein), putative | |  |  |  |  |  | | --- | --- | --- | --- | --- | |  |  |  |  |  | | EC:1.4.4.2 | | | | | | |
|  |  | RAFL07-18-E18 | At4g33010 / glycine dehydrogenase (decarboxylating) (glycine decarboxylase/glycine cleavage system P-protein), putative | |  |  |  |  |  | | --- | --- | --- | --- | --- | |  |  |  |  |  | | EC:1.4.4.2 | | | | | | |
|  |  | RAFL08-11-O04 | At4g33010 / glycine dehydrogenase (decarboxylating) (glycine decarboxylase/glycine cleavage system P-protein), putative | |  |  |  |  |  | | --- | --- | --- | --- | --- | |  |  |  |  |  | | EC:1.4.4.2 | | | | | | |
|  |  | RAFL11-06-P03 | At4g33010 / glycine dehydrogenase (decarboxylating) (glycine decarboxylase/glycine cleavage system P-protein), putative | |  |  |  |  |  | | --- | --- | --- | --- | --- | |  |  |  |  |  | | EC:1.4.4.2 | | | | | | |
|  |  | RAFL06-13-I09 | At3g01120 / cystathionine gamma-synthase -related | |  |  |  |  |  | | --- | --- | --- | --- | --- | |  |  |  |  |  | | EC:2.5.1.48 | | | | | | |
|  |  | RAFL07-10-O06 | At4g33010 / glycine dehydrogenase (decarboxylating) (glycine decarboxylase/glycine cleavage system P-protein), putative | |  |  |  |  |  | | --- | --- | --- | --- | --- | |  |  |  |  |  | | EC:1.4.4.2 | | | | | | |
|  |  | RAFL11-06-F06 | At4g33010 / glycine dehydrogenase (decarboxylating) (glycine decarboxylase/glycine cleavage system P-protein), putative | |  |  |  |  |  | | --- | --- | --- | --- | --- | |  |  |  |  |  | | EC:1.4.4.2 | | | | | | |
|  | Methionine metabolism | |  |  | 3 | 76 | 20 | 4564 | 0.0064938474 | 0.05195078 | 8 |
|  |  | RAFL09-09-A21 | At5g17920 / 5-methyltetrahydropteroyltriglutamate--homocysteine S-methyltransferase | |  |  |  |  |  | | --- | --- | --- | --- | --- | |  |  |  |  |  | | EC:2.1.1.14 | | | | | | |
|  |  | RAFL06-13-I09 | At3g01120 / cystathionine gamma-synthase -related | |  |  |  |  |  | | --- | --- | --- | --- | --- | |  |  |  |  |  | | EC:2.5.1.48 | | | | | | |
|  |  | RAFL08-16-E05 | At5g17920 / 5-methyltetrahydropteroyltriglutamate--homocysteine S-methyltransferase | |  |  |  |  |  | | --- | --- | --- | --- | --- | |  |  |  |  |  | | EC:2.1.1.14 | | | | | | |
|  | Sterol biosynthesis | |  |  | 2 | 77 | 13 | 4571 | 0.025799962 | 0.2837996 | 11 |
|  |  | RAFL04-15-A04 | At4g15560 / DEF (CLA1) protein | |  |  |  |  |  | | --- | --- | --- | --- | --- | |  |  |  |  |  | | EC:2.2.1.7 | | | | | | |
|  |  | RAFL07-12-F08 | At5g17230 / phytoene synthase (geranylgeranyl-diphosphate geranylgeranyl transferase)(PSY) | |  |  |  |  |  | | --- | --- | --- | --- | --- | |  |  |  |  |  | | EC:2.5.1.32 | | | | | | |
| Cluster:4-1 | | |  |  | A | B | C | D | P | P' | N |
|  | Ubiquinone biosynthesis | |  |  | 5 | 305 | 6 | 4347 | 4.162242E-4 | 0.0016648968 | 4 |
|  |  | RAFL05-04-N24 | At3g18410 / expressed protein | |  |  |  |  |  | | --- | --- | --- | --- | --- | |  |  |  |  |  | | EC:1.6.5.3 | | | | | | |
|  |  | RAFL09-18-I01 | At5g08530 / NADH-ubiquinone oxidoreductase (mitochondrial), putative | |  |  |  |  |  | | --- | --- | --- | --- | --- | |  |  |  |  |  | | EC:1.6.5.3 | | | | | | |
|  |  | RAFL09-10-O11 | At3g12260 / expressed protein | |  |  |  |  |  | | --- | --- | --- | --- | --- | |  |  |  |  |  | | EC:1.6.5.3 | | | | | | |
|  |  | RAFL11-02-J20 | At3g12260 / expressed protein | |  |  |  |  |  | | --- | --- | --- | --- | --- | |  |  |  |  |  | | EC:1.6.5.3 | | | | | | |
|  |  | RAFL06-08-D19 | At5g37510 / NADH dehydrogenase (ubiquinone), mitochondrial, putative | |  |  |  |  |  | | --- | --- | --- | --- | --- | |  |  |  |  |  | | EC:1.6.5.3 | | | | | | |
|  | Pyruvate metabolism | |  |  | 9 | 301 | 46 | 4307 | 0.009480812 | 0.17065461 | 18 |
|  |  | RAFL09-11-F09 | At1g30120 / pyruvate dehydrogenase E1 beta subunit -related | |  |  |  |  |  | | --- | --- | --- | --- | --- | |  |  |  |  |  | | EC:1.2.4.1 | | | | | | |
|  |  | RAFL09-12-A19 | At3g13930 / acetyltransferase -related | |  |  |  |  |  | | --- | --- | --- | --- | --- | |  |  |  |  |  | | EC:2.3.1.12 | | | | | | |
|  |  | RAFL05-17-D15 | At1g11840 / glyoxalase I, putative (lactoylglutathione lyase) | |  |  |  |  |  | | --- | --- | --- | --- | --- | |  |  |  |  |  | | EC:4.4.1.5 | | | | | | |
|  |  | RAFL06-08-D06 | At2g34590 / pyruvate dehydrogenase E1 beta subunit -related | |  |  |  |  |  | | --- | --- | --- | --- | --- | |  |  |  |  |  | | EC:1.2.4.1 | | | | | | |
|  |  | RAFL09-16-O16 | At1g01090 / pyruvate dehydrogenase E1 alpha subunit | |  |  |  |  |  | | --- | --- | --- | --- | --- | |  |  |  |  |  | | EC:1.2.4.1 | | | | | | |
|  |  | RAFL11-03-L09 | At5g56350 / pyruvate kinase, putative | |  |  |  |  |  | | --- | --- | --- | --- | --- | |  |  |  |  |  | | EC:2.7.1.40 | | | | | | |
|  |  | RAFL11-03-C02 | At1g48030 / dihydrolipoamide dehydrogenase, mitochondrial (lipoamide dehydrogenase) (mtlpd1) | |  |  |  |  |  | | --- | --- | --- | --- | --- | |  |  |  |  |  | | EC:1.8.1.4 | | | | | | |
|  |  | RAFL07-16-E16 | At3g13930 / acetyltransferase -related | |  |  |  |  |  | | --- | --- | --- | --- | --- | |  |  |  |  |  | | EC:2.3.1.12 | | | | | | |
|  |  | RAFL05-18-D04 | At5g36880 / acetyl-CoA synthetase (acetate-CoA ligase), putative | |  |  |  |  |  | | --- | --- | --- | --- | --- | |  |  |  |  |  | | EC:6.2.1.1 | | | | | | |
| Cluster:8-2 | | |  |  | A | B | C | D | P | P' | N |
|  | Cyanoamino acid metabolism | |  |  | 2 | 60 | 13 | 4588 | 0.016341258 | 0.16341259 | 10 |
|  |  | RAFL02-08-C20 | At4g08790 / nitrilase 1 like protein | |  |  |  |  |  | | --- | --- | --- | --- | --- | |  |  |  |  |  | | EC:4.2.1.84 ,EC:3.5.5.1 | | | | | | |
|  |  | RAFL08-18-I10 | At3g47000 / glycosyl hydrolase family 3 | |  |  |  |  |  | | --- | --- | --- | --- | --- | |  |  |  |  |  | | EC:3.2.1.21 | | | | | | |
| Cluster:2-1 | | |  |  | A | B | C | D | P | P' | N |
|  | Purine metabolism | |  |  | 5 | 239 | 29 | 4390 | 0.03021746 | 0.51369685 | 17 |
|  |  | RAFL04-18-P17 | At5g35170 / adenylate kinase -related protein | |  |  |  |  |  | | --- | --- | --- | --- | --- | |  |  |  |  |  | | EC:2.7.4.3 | | | | | | |
|  |  | RAFL04-12-O11 | At3g27740 / carbamoyl-phosphate synthase (glutamine-hydrolyzing) (glutamine-dependent carbamoyl-phosphate synthase) small subunit | |  |  |  |  |  | | --- | --- | --- | --- | --- | |  |  |  |  |  | | EC:6.3.5.2 | | | | | | |
|  |  | RAFL04-13-M20 | At4g11010 / nucleoside diphosphate kinase 3 (ndpk3) | |  |  |  |  |  | | --- | --- | --- | --- | --- | |  |  |  |  |  | | EC:2.7.4.6 | | | | | | |
|  |  | RAFL04-17-H07 | At3g57610 / adenylosuccinate synthetase | |  |  |  |  |  | | --- | --- | --- | --- | --- | |  |  |  |  |  | | EC:6.3.4.4 | | | | | | |
|  |  | RAFL06-13-H08 | At1g32440 / pyruvate kinase, putative | |  |  |  |  |  | | --- | --- | --- | --- | --- | |  |  |  |  |  | | EC:2.7.1.40 | | | | | | |
|  | Pyrimidine metabolism | |  |  | 3 | 241 | 12 | 4407 | 0.040360164 | 0.40360165 | 10 |
|  |  | RAFL04-18-P17 | At5g35170 / adenylate kinase -related protein | |  |  |  |  |  | | --- | --- | --- | --- | --- | |  |  |  |  |  | | EC:2.7.4.9 | | | | | | |
|  |  | RAFL04-12-O11 | At3g27740 / carbamoyl-phosphate synthase (glutamine-hydrolyzing) (glutamine-dependent carbamoyl-phosphate synthase) small subunit | |  |  |  |  |  | | --- | --- | --- | --- | --- | |  |  |  |  |  | | EC:6.3.5.5 | | | | | | |
|  |  | RAFL04-13-M20 | At4g11010 / nucleoside diphosphate kinase 3 (ndpk3) | |  |  |  |  |  | | --- | --- | --- | --- | --- | |  |  |  |  |  | | EC:2.7.4.6 | | | | | | |
|  | Alanine and aspartate metabolism | |  |  | 4 | 240 | 22 | 4397 | 0.04413268 | 0.7061229 | 16 |
|  |  | RAFL09-09-I19 | At1g23310 / alanine aminotransferase -related | |  |  |  |  |  | | --- | --- | --- | --- | --- | |  |  |  |  |  | | EC:2.6.1.2 | | | | | | |
|  |  | RAFL05-07-N11 | At1g70580 / alanine aminotransferase, putative | |  |  |  |  |  | | --- | --- | --- | --- | --- | |  |  |  |  |  | | EC:2.6.1.2 | | | | | | |
|  |  | RAFL04-17-H07 | At3g57610 / adenylosuccinate synthetase | |  |  |  |  |  | | --- | --- | --- | --- | --- | |  |  |  |  |  | | EC:6.3.4.4 | | | | | | |
|  |  | RAFL09-16-K01 | At1g23310 / alanine aminotransferase -related | |  |  |  |  |  | | --- | --- | --- | --- | --- | |  |  |  |  |  | | EC:2.6.1.2 | | | | | | |
| Cluster:10-2 | | |  |  | A | B | C | D | P | P' | N |
|  | Butanoate metabolism | |  |  | 3 | 106 | 30 | 4524 | 0.040794313 | 0.652709 | 16 |
|  |  | RAFL08-15-A08 | At4g34710 / arginine decarboxylase SPE2 | |  |  |  |  |  | | --- | --- | --- | --- | --- | |  |  |  |  |  | | EC:4.1.1.19 | | | | | | |
|  |  | RAFL04-13-O07 | At3g22200 / 4-aminobutyrate aminotransferase (gamma-amino-N-butyrate transaminase/GABA transaminase/beta-alanine--oxoglutarate aminotransferase) | |  |  |  |  |  | | --- | --- | --- | --- | --- | |  |  |  |  |  | | EC:2.6.1.19 | | | | | | |
|  |  | RAFL06-10-M04 | At2g26800 / hydroxymethylglutaryl-CoA lyase -related | |  |  |  |  |  | | --- | --- | --- | --- | --- | |  |  |  |  |  | | EC:4.1.3.4 | | | | | | |
|  | Starch and sucrose metabolism | |  |  | 4 | 105 | 54 | 4500 | 0.04569159 | 1.0052149 | 22 |
|  |  | RAFL08-15-K01 | At1g62660 / glycosyl hydrolase family 32 | |  |  |  |  |  | | --- | --- | --- | --- | --- | |  |  |  |  |  | | EC:3.2.1.26 | | | | | | |
|  |  | RAFL08-13-K06 | At1g62660 / glycosyl hydrolase family 32 | |  |  |  |  |  | | --- | --- | --- | --- | --- | |  |  |  |  |  | | EC:3.2.1.26 | | | | | | |
|  |  | RAFL07-14-D12 | At4g12430 / trehalose-6-phosphate phosphatase, putative | |  |  |  |  |  | | --- | --- | --- | --- | --- | |  |  |  |  |  | | EC:3.1.3.12 | | | | | | |
|  |  | RAFL05-07-J05 | At3g43190 / sucrose synthase (UDP-glucose-fructose glucosyltransferase/sucrose-UDP glucosyltransferase), putative | |  |  |  |  |  | | --- | --- | --- | --- | --- | |  |  |  |  |  | | EC:2.4.1.13 | | | | | | |
|  | Synthesis and degradation of ketone bodies | |  |  | 1 | 108 | 1 | 4553 | 0.0462095 | 0.092419 | 2 |
|  |  | RAFL06-10-M04 | At2g26800 / hydroxymethylglutaryl-CoA lyase -related | |  |  |  |  |  | | --- | --- | --- | --- | --- | |  |  |  |  |  | | EC:4.1.3.4 | | | | | | |
| Cluster:5-2 | | |  |  | A | B | C | D | P | P' | N |
|  | Starch and sucrose metabolism | |  |  | 5 | 121 | 53 | 4484 | 0.019437116 | 0.42761654 | 22 |
|  |  | RAFL09-14-L23 | At5g11720 / glycosyl hydrolase family 31 | |  |  |  |  |  | | --- | --- | --- | --- | --- | |  |  |  |  |  | | EC:3.2.1.20 | | | | | | |
|  |  | RAFL05-21-E11 | At5g64740 / cellulose synthase, catalytic subunit, putative | |  |  |  |  |  | | --- | --- | --- | --- | --- | |  |  |  |  |  | | EC:2.4.1.12 | | | | | | |
|  |  | RAFL07-09-M15 | At3g29360 / UDP-glucose dehydrogenase -related | |  |  |  |  |  | | --- | --- | --- | --- | --- | |  |  |  |  |  | | EC:1.1.1.22 | | | | | | |
|  |  | RAFL05-03-E09 | At5g64860 / glycosyl hydrolase family 77 (4-alpha-glucanotransferase) | |  |  |  |  |  | | --- | --- | --- | --- | --- | |  |  |  |  |  | | EC:2.4.1.25 | | | | | | |
|  |  | RAFL05-18-N23 | At2g35650 / glycosyltransferase family 2 | |  |  |  |  |  | | --- | --- | --- | --- | --- | |  |  |  |  |  | | EC:2.4.1.12 | | | | | | |
|  | Nucleotide sugars metabolism | |  |  | 3 | 123 | 19 | 4518 | 0.020386456 | 0.28541037 | 14 |
|  |  | RAFL07-11-C21 | At4g30440 / nucleotide sugar epimerase family | |  |  |  |  |  | | --- | --- | --- | --- | --- | |  |  |  |  |  | | EC:4.2.1.46 ,EC:5.1.3.2 | | | | | | |
|  |  | RAFL04-09-G05 | At1g50450 / expressed protein | |  |  |  |  |  | | --- | --- | --- | --- | --- | |  |  |  |  |  | | EC:4.2.1.46 | | | | | | |
|  |  | RAFL07-09-M15 | At3g29360 / UDP-glucose dehydrogenase -related | |  |  |  |  |  | | --- | --- | --- | --- | --- | |  |  |  |  |  | | EC:1.1.1.22 | | | | | | |
|  | Nitrogen metabolism | |  |  | 4 | 122 | 38 | 4499 | 0.025769636 | 0.56693196 | 22 |
|  |  | RAFL09-09-P06 | At3g53260 / phenylalanine ammonia-lyase (PAL2) | |  |  |  |  |  | | --- | --- | --- | --- | --- | |  |  |  |  |  | | EC:4.3.1.5 | | | | | | |
|  |  | RAFL11-09-K10 | At1g37130 / nitrate reductase 2 (NR2) | |  |  |  |  |  | | --- | --- | --- | --- | --- | |  |  |  |  |  | | EC:1.7.1.1 | | | | | | |
|  |  | RAFL09-13-L09 | At1g37130 / nitrate reductase 2 (NR2) | |  |  |  |  |  | | --- | --- | --- | --- | --- | |  |  |  |  |  | | EC:1.7.1.1 | | | | | | |
|  |  | RAFL09-11-J22 | At1g37130 / nitrate reductase 2 (NR2) | |  |  |  |  |  | | --- | --- | --- | --- | --- | |  |  |  |  |  | | EC:1.7.1.1 | | | | | | |
|  | Sphingophospholipid biosynthesis | |  |  | 1 | 125 | 0 | 4537 | 0.027021231 | 0.027021231 | 1 |
|  |  | RAFL05-21-D15 | At1g13560 / aminoalcoholphosphotransferase | |  |  |  |  |  | | --- | --- | --- | --- | --- | |  |  |  |  |  | | EC:2.7.8.1 | | | | | | |
|  | Erythromycin biosynthesis | |  |  | 2 | 124 | 8 | 4529 | 0.028284373 | 0.19799061 | 7 |
|  |  | RAFL07-11-C21 | At4g30440 / nucleotide sugar epimerase family | |  |  |  |  |  | | --- | --- | --- | --- | --- | |  |  |  |  |  | | EC:4.2.1.46 | | | | | | |
|  |  | RAFL04-09-G05 | At1g50450 / expressed protein | |  |  |  |  |  | | --- | --- | --- | --- | --- | |  |  |  |  |  | | EC:4.2.1.46 | | | | | | |
