# Supplementary material for: A flexible representation of omic knowledge for thorough analysis of microarray data
Source: Plant Methods. 2006 Mar 2;2:5. doi: 10.1186/1746-4811-2-5 (PMC1421397; doi:10.1186/1746-4811-2-5)
Supplement: Additional File 2 — Supplementary Table 2. Ranking result of significant correlations between the "functional Class" of the AraCyc type metabolic pathway and the clusters formed by BL-SOM of the microarray probes of expression profile under drought conditions [file 1746-4811-2-5-S2.HTML]

|  |  |  |  |  |  |  |  |  |  |  |  |
| --- | --- | --- | --- | --- | --- | --- | --- | --- | --- | --- | --- |
| Date: | | 2005/06/24 | | | | | | | | | |
| Method: | | Fisher test | | | | | | | | | |
| Cut off P-value: | | 0.05 | | | | | | | | | |
| Target dataset(s): | | AraCyc pathways | | | | | | | | | |
| Query dataset(s): | | SOM Cluster | | | | | | | | | |
  | | | | | | | | | | | || salicylic acid biosynthesis | | |  |  | A | B | C | D | P | P' | N |
|  | Cluster:6-2 | |  |  | 3 | 173 | 2 | 4485 | 4.998933E-4 | 0.0014996799 | 3 |
|  |  | RAFL09-11-L22 | At3g53260 / phenylalanine ammonia-lyase (PAL2) | |  |  |  |  |  | | --- | --- | --- | --- | --- | |  |  |  |  |  | | EC:4.3.1.5  phenylalanine ammonia-lyase | | | | | | |
|  |  | RAFL04-13-B02 | At2g37040 / phenylalanine ammonia lyase (PAL1) | |  |  |  |  |  | | --- | --- | --- | --- | --- | |  |  |  |  |  | | EC:4.3.1.5  phenylalanine ammonia-lyase | | | | | | |
|  |  | RAFL04-16-D08 | At3g53260 / phenylalanine ammonia-lyase (PAL2) | |  |  |  |  |  | | --- | --- | --- | --- | --- | |  |  |  |  |  | | EC:4.3.1.5  phenylalanine ammonia-lyase | | | | | | |
| gluconeogenesis | | |  |  | A | B | C | D | P | P' | N |
|  | Cluster:0-1 | |  |  | 16 | 90 | 31 | 4526 | 1.345045E-15 | 2.152072E-14 | 16 |
|  |  | RAFL07-18-C20 | At2g21330 / fructose-bisphosphate aldolase, putative | |  |  |  |  |  | | --- | --- | --- | --- | --- | |  |  |  |  |  | | EC:4.1.2.13  fructose-bisphosphate aldolase | | | | | | |
|  |  | RAFL09-15-L04 | At3g12780 / phosphoglycerate kinase -related | |  |  |  |  |  | | --- | --- | --- | --- | --- | |  |  |  |  |  | | EC:2.7.2.3  phosphoglycerate kinase | | | | | | |
|  |  | RAFL07-14-L16 | At3g12780 / phosphoglycerate kinase -related | |  |  |  |  |  | | --- | --- | --- | --- | --- | |  |  |  |  |  | | EC:2.7.2.3  phosphoglycerate kinase | | | | | | |
|  |  | RAFL08-18-C10 | At2g21330 / fructose-bisphosphate aldolase, putative | |  |  |  |  |  | | --- | --- | --- | --- | --- | |  |  |  |  |  | | EC:4.1.2.13  fructose-bisphosphate aldolase | | | | | | |
|  |  | RAFL07-12-E12 | At2g21330 / fructose-bisphosphate aldolase, putative | |  |  |  |  |  | | --- | --- | --- | --- | --- | |  |  |  |  |  | | EC:4.1.2.13  fructose-bisphosphate aldolase | | | | | | |
|  |  | RAFL07-07-I23 | At1g56190 / phosphoglycerate kinase -related | |  |  |  |  |  | | --- | --- | --- | --- | --- | |  |  |  |  |  | | EC:2.7.2.3  phosphoglycerate kinase | | | | | | |
|  |  | RAFL04-13-J02 | At3g54050 / fructose-bisphosphatase precursor | |  |  |  |  |  | | --- | --- | --- | --- | --- | |  |  |  |  |  | | EC:3.1.3.11  fructose-bisphosphatase//phosphoric ester hydrolase | | | | | | |
|  |  | RAFL07-12-M09 | At2g21330 / fructose-bisphosphate aldolase, putative | |  |  |  |  |  | | --- | --- | --- | --- | --- | |  |  |  |  |  | | EC:4.1.2.13  fructose-bisphosphate aldolase | | | | | | |
|  |  | RAFL04-09-D24 | At1g42970 / glyceraldehyde-3-phosphate dehydrogenase | |  |  |  |  |  | | --- | --- | --- | --- | --- | |  |  |  |  |  | | EC:1.2.1.12  glyceraldehyde-3-phosphate dehydrogenase | | | | | | |
|  |  | RAFL07-16-P05 | At3g12780 / phosphoglycerate kinase -related | |  |  |  |  |  | | --- | --- | --- | --- | --- | |  |  |  |  |  | | EC:2.7.2.3  phosphoglycerate kinase | | | | | | |
|  |  | RAFL09-18-L22 | At3g12780 / phosphoglycerate kinase -related | |  |  |  |  |  | | --- | --- | --- | --- | --- | |  |  |  |  |  | | EC:2.7.2.3  phosphoglycerate kinase | | | | | | |
|  |  | RAFL04-19-O21 | At4g38970 / fructose-bisphosphate aldolase, putative | |  |  |  |  |  | | --- | --- | --- | --- | --- | |  |  |  |  |  | | EC:4.1.2.13  fructose-bisphosphate aldolase | | | | | | |
|  |  | RAFL09-17-N23 | At2g21330 / fructose-bisphosphate aldolase, putative | |  |  |  |  |  | | --- | --- | --- | --- | --- | |  |  |  |  |  | | EC:4.1.2.13  fructose-bisphosphate aldolase | | | | | | |
|  |  | RAFL04-15-A14 | At1g12900 / calcium-binding protein, calreticulin -related | |  |  |  |  |  | | --- | --- | --- | --- | --- | |  |  |  |  |  | | EC:1.2.1.12  glyceraldehyde-3-phosphate dehydrogenase | | | | | | |
|  |  | RAFL07-18-J01 | At2g21330 / fructose-bisphosphate aldolase, putative | |  |  |  |  |  | | --- | --- | --- | --- | --- | |  |  |  |  |  | | EC:4.1.2.13  fructose-bisphosphate aldolase | | | | | | |
|  |  | RAFL05-07-J06 | At1g42970 / glyceraldehyde-3-phosphate dehydrogenase | |  |  |  |  |  | | --- | --- | --- | --- | --- | |  |  |  |  |  | | EC:1.2.1.12  glyceraldehyde-3-phosphate dehydrogenase | | | | | | |
|  | Cluster:3-0 | |  |  | 7 | 226 | 40 | 4390 | 0.008070272 | 0.12912436 | 16 |
|  |  | RAFL07-17-M04 | At1g04410 / malate dehydrogenase, cytosolic, putative | |  |  |  |  |  | | --- | --- | --- | --- | --- | |  |  |  |  |  | | EC:1.1.1.37  malate dehydrogenase | | | | | | |
|  |  | RAFL06-07-J21 | At1g04410 / malate dehydrogenase, cytosolic, putative | |  |  |  |  |  | | --- | --- | --- | --- | --- | |  |  |  |  |  | | EC:1.1.1.37  malate dehydrogenase | | | | | | |
|  |  | RAFL05-21-P13 | At2g42600 / phosphoenolpyruvate carboxylase | |  |  |  |  |  | | --- | --- | --- | --- | --- | |  |  |  |  |  | | EC:4.1.1.49 ,EC:4.1.1.31  phosphoenolpyruvate carboxykinase (ATP)  phosphoenolpyruvate carboxylase | | | | | | |
|  |  | RAFL09-09-M02 | At3g47520 / malate dehydrogenase (NAD), chloroplast, putative | |  |  |  |  |  | | --- | --- | --- | --- | --- | |  |  |  |  |  | | EC:1.1.1.37  malate dehydrogenase | | | | | | |
|  |  | RAFL04-17-F02 | At2g01140 / fructose-bisphosphate aldolase, putative | |  |  |  |  |  | | --- | --- | --- | --- | --- | |  |  |  |  |  | | EC:4.1.2.13  fructose-bisphosphate aldolase | | | | | | |
|  |  | RAFL06-14-K01 | At3g04120 / glyceraldehyde-3-phosphate dehydrogenase C subunit (GapC) | |  |  |  |  |  | | --- | --- | --- | --- | --- | |  |  |  |  |  | | EC:1.2.1.12  glyceraldehyde-3-phosphate dehydrogenase | | | | | | |
|  |  | RAFL06-11-B16 | At3g52930 / fructose-bisphosphate aldolase, putative | |  |  |  |  |  | | --- | --- | --- | --- | --- | |  |  |  |  |  | | EC:4.1.2.13  fructose-bisphosphate aldolase | | | | | | |
| nitrate assimilation pathway | | |  |  | A | B | C | D | P | P' | N |
|  | Cluster:5-2 | |  |  | 3 | 123 | 8 | 4529 | 0.0027120202 | 0.021696161 | 8 |
|  |  | RAFL09-13-L09 | At1g37130 / nitrate reductase 2 (NR2) | |  |  |  |  |  | | --- | --- | --- | --- | --- | |  |  |  |  |  | | EC:1.7.1.1  nitrate reductase | | | | | | |
|  |  | RAFL09-11-J22 | At1g37130 / nitrate reductase 2 (NR2) | |  |  |  |  |  | | --- | --- | --- | --- | --- | |  |  |  |  |  | | EC:1.7.1.1  nitrate reductase | | | | | | |
|  |  | RAFL11-09-K10 | At1g37130 / nitrate reductase 2 (NR2) | |  |  |  |  |  | | --- | --- | --- | --- | --- | |  |  |  |  |  | | EC:1.7.1.1  nitrate reductase | | | | | | |
|  | Cluster:9-2 | |  |  | 2 | 65 | 9 | 4587 | 0.010289272 | 0.08231418 | 8 |
|  |  | RAFL08-17-D17 | At3g53180 / nodulin / glutamate-ammonia ligase - like protein | |  |  |  |  |  | | --- | --- | --- | --- | --- | |  |  |  |  |  | | EC:6.3.1.2  glutamate-ammonia ligase | | | | | | |
|  |  | RAFL06-09-F14 | At3g53180 / nodulin / glutamate-ammonia ligase - like protein | |  |  |  |  |  | | --- | --- | --- | --- | --- | |  |  |  |  |  | | EC:6.3.1.2  glutamate-ammonia ligase | | | | | | |
| sorbitol fermentation | | |  |  | A | B | C | D | P | P' | N |
|  | Cluster:0-1 | |  |  | 15 | 91 | 29 | 4528 | 1.08628985E-14 | 1.6294348E-13 | 15 |
|  |  | RAFL07-18-C20 | At2g21330 / fructose-bisphosphate aldolase, putative | |  |  |  |  |  | | --- | --- | --- | --- | --- | |  |  |  |  |  | | EC:4.1.2.13  fructose-bisphosphate aldolase | | | | | | |
|  |  | RAFL09-15-L04 | At3g12780 / phosphoglycerate kinase -related | |  |  |  |  |  | | --- | --- | --- | --- | --- | |  |  |  |  |  | | EC:2.7.2.3  phosphoglycerate kinase | | | | | | |
|  |  | RAFL07-14-L16 | At3g12780 / phosphoglycerate kinase -related | |  |  |  |  |  | | --- | --- | --- | --- | --- | |  |  |  |  |  | | EC:2.7.2.3  phosphoglycerate kinase | | | | | | |
|  |  | RAFL08-18-C10 | At2g21330 / fructose-bisphosphate aldolase, putative | |  |  |  |  |  | | --- | --- | --- | --- | --- | |  |  |  |  |  | | EC:4.1.2.13  fructose-bisphosphate aldolase | | | | | | |
|  |  | RAFL07-12-E12 | At2g21330 / fructose-bisphosphate aldolase, putative | |  |  |  |  |  | | --- | --- | --- | --- | --- | |  |  |  |  |  | | EC:4.1.2.13  fructose-bisphosphate aldolase | | | | | | |
|  |  | RAFL07-07-I23 | At1g56190 / phosphoglycerate kinase -related | |  |  |  |  |  | | --- | --- | --- | --- | --- | |  |  |  |  |  | | EC:2.7.2.3  phosphoglycerate kinase | | | | | | |
|  |  | RAFL07-12-M09 | At2g21330 / fructose-bisphosphate aldolase, putative | |  |  |  |  |  | | --- | --- | --- | --- | --- | |  |  |  |  |  | | EC:4.1.2.13  fructose-bisphosphate aldolase | | | | | | |
|  |  | RAFL04-09-D24 | At1g42970 / glyceraldehyde-3-phosphate dehydrogenase | |  |  |  |  |  | | --- | --- | --- | --- | --- | |  |  |  |  |  | | EC:1.2.1.12  glyceraldehyde-3-phosphate dehydrogenase | | | | | | |
|  |  | RAFL07-16-P05 | At3g12780 / phosphoglycerate kinase -related | |  |  |  |  |  | | --- | --- | --- | --- | --- | |  |  |  |  |  | | EC:2.7.2.3  phosphoglycerate kinase | | | | | | |
|  |  | RAFL09-18-L22 | At3g12780 / phosphoglycerate kinase -related | |  |  |  |  |  | | --- | --- | --- | --- | --- | |  |  |  |  |  | | EC:2.7.2.3  phosphoglycerate kinase | | | | | | |
|  |  | RAFL04-19-O21 | At4g38970 / fructose-bisphosphate aldolase, putative | |  |  |  |  |  | | --- | --- | --- | --- | --- | |  |  |  |  |  | | EC:4.1.2.13  fructose-bisphosphate aldolase | | | | | | |
|  |  | RAFL09-17-N23 | At2g21330 / fructose-bisphosphate aldolase, putative | |  |  |  |  |  | | --- | --- | --- | --- | --- | |  |  |  |  |  | | EC:4.1.2.13  fructose-bisphosphate aldolase | | | | | | |
|  |  | RAFL04-15-A14 | At1g12900 / calcium-binding protein, calreticulin -related | |  |  |  |  |  | | --- | --- | --- | --- | --- | |  |  |  |  |  | | EC:1.2.1.12  glyceraldehyde-3-phosphate dehydrogenase | | | | | | |
|  |  | RAFL07-18-J01 | At2g21330 / fructose-bisphosphate aldolase, putative | |  |  |  |  |  | | --- | --- | --- | --- | --- | |  |  |  |  |  | | EC:4.1.2.13  fructose-bisphosphate aldolase | | | | | | |
|  |  | RAFL05-07-J06 | At1g42970 / glyceraldehyde-3-phosphate dehydrogenase | |  |  |  |  |  | | --- | --- | --- | --- | --- | |  |  |  |  |  | | EC:1.2.1.12  glyceraldehyde-3-phosphate dehydrogenase | | | | | | |
|  | Cluster:1-2 | |  |  | 5 | 169 | 39 | 4450 | 0.0228522 | 0.34278297 | 15 |
|  |  | RAFL05-13-B09 | At5g03300 / pfkB type carbohydrate kinase protein family | |  |  |  |  |  | | --- | --- | --- | --- | --- | |  |  |  |  |  | | EC:2.7.1.11  6-phosphofructokinase | | | | | | |
|  |  | RAFL04-09-G20 | At2g21170 / triosephosphate isomerase, chloroplast, putative | |  |  |  |  |  | | --- | --- | --- | --- | --- | |  |  |  |  |  | | EC:5.3.1.1  triose-phosphate isomerase | | | | | | |
|  |  | RAFL07-12-L15 | At1g12000 / pyrophosphate-fructose-6-phosphate 1-phosphotransferase -related | |  |  |  |  |  | | --- | --- | --- | --- | --- | |  |  |  |  |  | | EC:2.7.1.11  6-phosphofructokinase | | | | | | |
|  |  | RAFL07-15-F22 | At1g20950 / pyrophosphate-dependent phosphofructokinase alpha subunit -related | |  |  |  |  |  | | --- | --- | --- | --- | --- | |  |  |  |  |  | | EC:2.7.1.11  6-phosphofructokinase | | | | | | |
|  |  | RAFL06-13-M02 | At1g13440 / glyceraldehyde-3-phosphate dehydrogenase -related | |  |  |  |  |  | | --- | --- | --- | --- | --- | |  |  |  |  |  | | EC:1.2.1.12  glyceraldehyde-3-phosphate dehydrogenase | | | | | | |
| formaldehyde assimilation I (serine pathway) | | |  |  | A | B | C | D | P | P' | N |
|  | Cluster:3-0 | |  |  | 7 | 226 | 16 | 4414 | 8.757928E-5 | 0.0010509514 | 12 |
|  |  | RAFL07-17-M04 | At1g04410 / malate dehydrogenase, cytosolic, putative | |  |  |  |  |  | | --- | --- | --- | --- | --- | |  |  |  |  |  | | EC:1.1.1.37  malate dehydrogenase | | | | | | |
|  |  | RAFL05-10-L06 | At2g13360 / alanine-glyoxylate aminotransferase | |  |  |  |  |  | | --- | --- | --- | --- | --- | |  |  |  |  |  | | EC:2.6.1.45  unknown | | | | | | |
|  |  | RAFL06-07-J21 | At1g04410 / malate dehydrogenase, cytosolic, putative | |  |  |  |  |  | | --- | --- | --- | --- | --- | |  |  |  |  |  | | EC:1.1.1.37  malate dehydrogenase | | | | | | |
|  |  | RAFL05-21-P13 | At2g42600 / phosphoenolpyruvate carboxylase | |  |  |  |  |  | | --- | --- | --- | --- | --- | |  |  |  |  |  | | EC:4.1.1.31  phosphoenolpyruvate carboxylase | | | | | | |
|  |  | RAFL09-09-M02 | At3g47520 / malate dehydrogenase (NAD), chloroplast, putative | |  |  |  |  |  | | --- | --- | --- | --- | --- | |  |  |  |  |  | | EC:1.1.1.37  malate dehydrogenase | | | | | | |
|  |  | RAFL05-18-J16 | At2g13360 / alanine-glyoxylate aminotransferase | |  |  |  |  |  | | --- | --- | --- | --- | --- | |  |  |  |  |  | | EC:2.6.1.45  unknown | | | | | | |
|  |  | RAFL05-02-A17 | At4g32520 / glycine hydroxymethyltransferase (EC 2.1.2.1) - like protein | |  |  |  |  |  | | --- | --- | --- | --- | --- | |  |  |  |  |  | | EC:2.1.2.1  glycine hydroxymethyltransferase | | | | | | |
| aspartate degradation I | | |  |  | A | B | C | D | P | P' | N |
|  | Cluster:3-0 | |  |  | 2 | 231 | 3 | 4427 | 0.02249133 | 0.067473985 | 3 |
|  |  | RAFL05-21-P13 | At2g42600 / phosphoenolpyruvate carboxylase | |  |  |  |  |  | | --- | --- | --- | --- | --- | |  |  |  |  |  | | EC:4.1.1.32  phosphoenolpyruvate carboxykinase | | | | | | |
|  |  | RAFL09-07-B08 | At2g30970 / aspartate aminotransferase, mitochondrial (transaminase A/Asp1) | |  |  |  |  |  | | --- | --- | --- | --- | --- | |  |  |  |  |  | | EC:2.6.1.1  aspartate transaminase | | | | | | |
|  | Cluster:6-1 | |  |  | 2 | 313 | 3 | 4345 | 0.039689664 | 0.11906899 | 3 |
|  |  | RAFL04-17-L05 | At5g19550 / aspartate aminotransferase, cytoplasmic isozyme 1 (transaminase A/Asp2) | |  |  |  |  |  | | --- | --- | --- | --- | --- | |  |  |  |  |  | | EC:2.6.1.1  aspartate transaminase | | | | | | |
|  |  | RAFL11-09-A12 | At5g19550 / aspartate aminotransferase, cytoplasmic isozyme 1 (transaminase A/Asp2) | |  |  |  |  |  | | --- | --- | --- | --- | --- | |  |  |  |  |  | | EC:2.6.1.1  aspartate transaminase | | | | | | |
| TCA cycle variation VII | | |  |  | A | B | C | D | P | P' | N |
|  | Cluster:3-0 | |  |  | 5 | 228 | 6 | 4424 | 1.0765596E-4 | 7.535917E-4 | 7 |
|  |  | RAFL07-17-M04 | At1g04410 / malate dehydrogenase, cytosolic, putative | |  |  |  |  |  | | --- | --- | --- | --- | --- | |  |  |  |  |  | | EC:1.1.1.37  malate dehydrogenase | | | | | | |
|  |  | RAFL06-07-J21 | At1g04410 / malate dehydrogenase, cytosolic, putative | |  |  |  |  |  | | --- | --- | --- | --- | --- | |  |  |  |  |  | | EC:1.1.1.37  malate dehydrogenase | | | | | | |
|  |  | RAFL05-21-P13 | At2g42600 / phosphoenolpyruvate carboxylase | |  |  |  |  |  | | --- | --- | --- | --- | --- | |  |  |  |  |  | | EC:4.1.1.32  phosphoenolpyruvate carboxykinase | | | | | | |
|  |  | RAFL09-09-M02 | At3g47520 / malate dehydrogenase (NAD), chloroplast, putative | |  |  |  |  |  | | --- | --- | --- | --- | --- | |  |  |  |  |  | | EC:1.1.1.37  malate dehydrogenase | | | | | | |
|  |  | RAFL07-14-B18 | At2g47510 / fumarase -related | |  |  |  |  |  | | --- | --- | --- | --- | --- | |  |  |  |  |  | | EC:4.2.1.2  adenylosuccinate lyase | | | | | | |
| tryptophan biosynthesis | | |  |  | A | B | C | D | P | P' | N |
|  | Cluster:6-2 | |  |  | 2 | 174 | 5 | 4482 | 0.026250903 | 0.13125451 | 5 |
|  |  | RAFL05-01-B07 | At3g57880 / C2 domain-containing protein | |  |  |  |  |  | | --- | --- | --- | --- | --- | |  |  |  |  |  | | EC:2.4.2.18  anthranilate phosphoribosyltransferase | | | | | | |
|  |  | RAFL06-16-J02 | At4g30530 / glutamine amidotransferase class-I domain-containing protein | |  |  |  |  |  | | --- | --- | --- | --- | --- | |  |  |  |  |  | | EC:4.1.3.27  GMP synthase (glutamine-hydrolyzing) | | | | | | |
|  | Cluster:3-1 | |  |  | 2 | 214 | 5 | 4442 | 0.03844968 | 0.1922484 | 5 |
|  |  | RAFL05-16-L22 | At5g48220 / indole-3-glycerol phosphate synthase (IGPS), putative | |  |  |  |  |  | | --- | --- | --- | --- | --- | |  |  |  |  |  | | EC:4.1.1.48  indole-3-glycerol-phosphate synthase | | | | | | |
|  |  | RAFL05-13-G12 | At1g07780 / phosphoribosylanthranilate isomerase (PAI1) | |  |  |  |  |  | | --- | --- | --- | --- | --- | |  |  |  |  |  | | EC:5.3.1.24  phosphoribosylanthranilate isomerase | | | | | | |
| arginine degradation IX | | |  |  | A | B | C | D | P | P' | N |
|  | Cluster:9-0 | |  |  | 4 | 28 | 9 | 4622 | 1.2514063E-6 | 1.2514062E-5 | 10 |
|  |  | RAFL05-21-E06 | At1g54100 / aldehyde dehydrogenase, putative (ALDH) | |  |  |  |  |  | | --- | --- | --- | --- | --- | |  |  |  |  |  | | EC:1.5.1.12  1-pyrroline-5-carboxylate dehydrogenase//aldehyde dehydrogenase | | | | | | |
|  |  | RAFL04-09-D07 | At1g54100 / aldehyde dehydrogenase, putative (ALDH) | |  |  |  |  |  | | --- | --- | --- | --- | --- | |  |  |  |  |  | | EC:1.5.1.12  1-pyrroline-5-carboxylate dehydrogenase//aldehyde dehydrogenase | | | | | | |
|  |  | RAFL08-15-L09 | At1g54100 / aldehyde dehydrogenase, putative (ALDH) | |  |  |  |  |  | | --- | --- | --- | --- | --- | |  |  |  |  |  | | EC:1.5.1.12  1-pyrroline-5-carboxylate dehydrogenase//aldehyde dehydrogenase | | | | | | |
|  |  | RAFL08-09-C23 | At1g54100 / aldehyde dehydrogenase, putative (ALDH) | |  |  |  |  |  | | --- | --- | --- | --- | --- | |  |  |  |  |  | | EC:1.5.1.12  1-pyrroline-5-carboxylate dehydrogenase//aldehyde dehydrogenase | | | | | | |
| mannitol degradation | | |  |  | A | B | C | D | P | P' | N |
|  | Cluster:0-1 | |  |  | 7 | 99 | 7 | 4550 | 7.745682E-9 | 3.8728412E-8 | 5 |
|  |  | RAFL07-18-C20 | At2g21330 / fructose-bisphosphate aldolase, putative | |  |  |  |  |  | | --- | --- | --- | --- | --- | |  |  |  |  |  | | EC:4.1.2.13  fructose-bisphosphate aldolase | | | | | | |
|  |  | RAFL08-18-C10 | At2g21330 / fructose-bisphosphate aldolase, putative | |  |  |  |  |  | | --- | --- | --- | --- | --- | |  |  |  |  |  | | EC:4.1.2.13  fructose-bisphosphate aldolase | | | | | | |
|  |  | RAFL07-12-E12 | At2g21330 / fructose-bisphosphate aldolase, putative | |  |  |  |  |  | | --- | --- | --- | --- | --- | |  |  |  |  |  | | EC:4.1.2.13  fructose-bisphosphate aldolase | | | | | | |
|  |  | RAFL07-12-M09 | At2g21330 / fructose-bisphosphate aldolase, putative | |  |  |  |  |  | | --- | --- | --- | --- | --- | |  |  |  |  |  | | EC:4.1.2.13  fructose-bisphosphate aldolase | | | | | | |
|  |  | RAFL04-19-O21 | At4g38970 / fructose-bisphosphate aldolase, putative | |  |  |  |  |  | | --- | --- | --- | --- | --- | |  |  |  |  |  | | EC:4.1.2.13  fructose-bisphosphate aldolase | | | | | | |
|  |  | RAFL09-17-N23 | At2g21330 / fructose-bisphosphate aldolase, putative | |  |  |  |  |  | | --- | --- | --- | --- | --- | |  |  |  |  |  | | EC:4.1.2.13  fructose-bisphosphate aldolase | | | | | | |
|  |  | RAFL07-18-J01 | At2g21330 / fructose-bisphosphate aldolase, putative | |  |  |  |  |  | | --- | --- | --- | --- | --- | |  |  |  |  |  | | EC:4.1.2.13  fructose-bisphosphate aldolase | | | | | | |
|  | Cluster:1-2 | |  |  | 3 | 171 | 11 | 4478 | 0.013720686 | 0.068603426 | 5 |
|  |  | RAFL05-13-B09 | At5g03300 / pfkB type carbohydrate kinase protein family | |  |  |  |  |  | | --- | --- | --- | --- | --- | |  |  |  |  |  | | EC:2.7.1.11  6-phosphofructokinase | | | | | | |
|  |  | RAFL07-12-L15 | At1g12000 / pyrophosphate-fructose-6-phosphate 1-phosphotransferase -related | |  |  |  |  |  | | --- | --- | --- | --- | --- | |  |  |  |  |  | | EC:2.7.1.11  6-phosphofructokinase | | | | | | |
|  |  | RAFL07-15-F22 | At1g20950 / pyrophosphate-dependent phosphofructokinase alpha subunit -related | |  |  |  |  |  | | --- | --- | --- | --- | --- | |  |  |  |  |  | | EC:2.7.1.11  6-phosphofructokinase | | | | | | |
| glycolysis I | | |  |  | A | B | C | D | P | P' | N |
|  | Cluster:0-1 | |  |  | 15 | 91 | 28 | 4529 | 7.293992E-15 | 1.09409884E-13 | 15 |
|  |  | RAFL07-18-C20 | At2g21330 / fructose-bisphosphate aldolase, putative | |  |  |  |  |  | | --- | --- | --- | --- | --- | |  |  |  |  |  | | EC:4.1.2.13  fructose-bisphosphate aldolase | | | | | | |
|  |  | RAFL09-15-L04 | At3g12780 / phosphoglycerate kinase -related | |  |  |  |  |  | | --- | --- | --- | --- | --- | |  |  |  |  |  | | EC:2.7.2.3  phosphoglycerate kinase | | | | | | |
|  |  | RAFL07-14-L16 | At3g12780 / phosphoglycerate kinase -related | |  |  |  |  |  | | --- | --- | --- | --- | --- | |  |  |  |  |  | | EC:2.7.2.3  phosphoglycerate kinase | | | | | | |
|  |  | RAFL08-18-C10 | At2g21330 / fructose-bisphosphate aldolase, putative | |  |  |  |  |  | | --- | --- | --- | --- | --- | |  |  |  |  |  | | EC:4.1.2.13  fructose-bisphosphate aldolase | | | | | | |
|  |  | RAFL07-12-E12 | At2g21330 / fructose-bisphosphate aldolase, putative | |  |  |  |  |  | | --- | --- | --- | --- | --- | |  |  |  |  |  | | EC:4.1.2.13  fructose-bisphosphate aldolase | | | | | | |
|  |  | RAFL07-07-I23 | At1g56190 / phosphoglycerate kinase -related | |  |  |  |  |  | | --- | --- | --- | --- | --- | |  |  |  |  |  | | EC:2.7.2.3  phosphoglycerate kinase | | | | | | |
|  |  | RAFL07-12-M09 | At2g21330 / fructose-bisphosphate aldolase, putative | |  |  |  |  |  | | --- | --- | --- | --- | --- | |  |  |  |  |  | | EC:4.1.2.13  fructose-bisphosphate aldolase | | | | | | |
|  |  | RAFL04-09-D24 | At1g42970 / glyceraldehyde-3-phosphate dehydrogenase | |  |  |  |  |  | | --- | --- | --- | --- | --- | |  |  |  |  |  | | EC:1.2.1.12  glyceraldehyde-3-phosphate dehydrogenase | | | | | | |
|  |  | RAFL07-16-P05 | At3g12780 / phosphoglycerate kinase -related | |  |  |  |  |  | | --- | --- | --- | --- | --- | |  |  |  |  |  | | EC:2.7.2.3  phosphoglycerate kinase | | | | | | |
|  |  | RAFL09-18-L22 | At3g12780 / phosphoglycerate kinase -related | |  |  |  |  |  | | --- | --- | --- | --- | --- | |  |  |  |  |  | | EC:2.7.2.3  phosphoglycerate kinase | | | | | | |
|  |  | RAFL04-19-O21 | At4g38970 / fructose-bisphosphate aldolase, putative | |  |  |  |  |  | | --- | --- | --- | --- | --- | |  |  |  |  |  | | EC:4.1.2.13  fructose-bisphosphate aldolase | | | | | | |
|  |  | RAFL09-17-N23 | At2g21330 / fructose-bisphosphate aldolase, putative | |  |  |  |  |  | | --- | --- | --- | --- | --- | |  |  |  |  |  | | EC:4.1.2.13  fructose-bisphosphate aldolase | | | | | | |
|  |  | RAFL04-15-A14 | At1g12900 / calcium-binding protein, calreticulin -related | |  |  |  |  |  | | --- | --- | --- | --- | --- | |  |  |  |  |  | | EC:1.2.1.12  glyceraldehyde-3-phosphate dehydrogenase | | | | | | |
|  |  | RAFL07-18-J01 | At2g21330 / fructose-bisphosphate aldolase, putative | |  |  |  |  |  | | --- | --- | --- | --- | --- | |  |  |  |  |  | | EC:4.1.2.13  fructose-bisphosphate aldolase | | | | | | |
|  |  | RAFL05-07-J06 | At1g42970 / glyceraldehyde-3-phosphate dehydrogenase | |  |  |  |  |  | | --- | --- | --- | --- | --- | |  |  |  |  |  | | EC:1.2.1.12  glyceraldehyde-3-phosphate dehydrogenase | | | | | | |
|  | Cluster:1-2 | |  |  | 5 | 169 | 38 | 4451 | 0.020866273 | 0.3129941 | 15 |
|  |  | RAFL05-13-B09 | At5g03300 / pfkB type carbohydrate kinase protein family | |  |  |  |  |  | | --- | --- | --- | --- | --- | |  |  |  |  |  | | EC:2.7.1.11  6-phosphofructokinase | | | | | | |
|  |  | RAFL04-09-G20 | At2g21170 / triosephosphate isomerase, chloroplast, putative | |  |  |  |  |  | | --- | --- | --- | --- | --- | |  |  |  |  |  | | EC:5.3.1.1  triose-phosphate isomerase | | | | | | |
|  |  | RAFL07-12-L15 | At1g12000 / pyrophosphate-fructose-6-phosphate 1-phosphotransferase -related | |  |  |  |  |  | | --- | --- | --- | --- | --- | |  |  |  |  |  | | EC:2.7.1.11  6-phosphofructokinase | | | | | | |
|  |  | RAFL07-15-F22 | At1g20950 / pyrophosphate-dependent phosphofructokinase alpha subunit -related | |  |  |  |  |  | | --- | --- | --- | --- | --- | |  |  |  |  |  | | EC:2.7.1.11  6-phosphofructokinase | | | | | | |
|  |  | RAFL06-13-M02 | At1g13440 / glyceraldehyde-3-phosphate dehydrogenase -related | |  |  |  |  |  | | --- | --- | --- | --- | --- | |  |  |  |  |  | | EC:1.2.1.12  glyceraldehyde-3-phosphate dehydrogenase | | | | | | |
|  | Cluster:3-1 | |  |  | 5 | 211 | 38 | 4409 | 0.04685452 | 0.7028178 | 15 |
|  |  | RAFL07-10-P13 | At1g09780 / 2,3-bisphosphoglycerate-independent phosphoglycerate mutase -related | |  |  |  |  |  | | --- | --- | --- | --- | --- | |  |  |  |  |  | | EC:5.4.2.1  2,3-bisphosphoglycerate-independent phosphoglycerate mutase | | | | | | |
|  |  | RAFL05-01-I24 | At3g55440 / triosephosphate isomerase, cytosolic, putative | |  |  |  |  |  | | --- | --- | --- | --- | --- | |  |  |  |  |  | | EC:5.3.1.1  triose-phosphate isomerase | | | | | | |
|  |  | RAFL05-21-G03 | At3g22960 / pyruvate kinase, putative | |  |  |  |  |  | | --- | --- | --- | --- | --- | |  |  |  |  |  | | EC:2.7.1.40  pyruvate kinase | | | | | | |
|  |  | RAFL04-13-O10 | At2g29560 / enolase (2-phospho-D-glycerate hydroylase) -related | |  |  |  |  |  | | --- | --- | --- | --- | --- | |  |  |  |  |  | | EC:4.2.1.11  phosphopyruvate hydratase | | | | | | |
|  |  | RAFL09-12-D13 | At5g52920 / pyruvate kinase, putative | |  |  |  |  |  | | --- | --- | --- | --- | --- | |  |  |  |  |  | | EC:2.7.1.40  pyruvate kinase | | | | | | |
| trehalose biosynthesis II | | |  |  | A | B | C | D | P | P' | N |
|  | Cluster:10-2 | |  |  | 1 | 108 | 1 | 4553 | 0.0462095 | 0.092419 | 2 |
|  |  | RAFL07-14-D12 | At4g12430 / trehalose-6-phosphate phosphatase, putative | |  |  |  |  |  | | --- | --- | --- | --- | --- | |  |  |  |  |  | | EC:3.1.3.12  trehalose-phosphatase | | | | | | |
| triacylglycerol degradation | | |  |  | A | B | C | D | P | P' | N |
|  | Cluster:9-1 | |  |  | 2 | 94 | 6 | 4561 | 0.0108338045 | 0.06500283 | 6 |
|  |  | RAFL08-09-J19 | At1g02660 / lipase (class 3) family | |  |  |  |  |  | | --- | --- | --- | --- | --- | |  |  |  |  |  | | EC:3.1.1.3  triacylglycerol lipase | | | | | | |
|  |  | RAFL05-18-O21 | At2g30550 / lipase (class 3) family | |  |  |  |  |  | | --- | --- | --- | --- | --- | |  |  |  |  |  | | EC:3.1.1.3  triacylglycerol lipase | | | | | | |
|  | Cluster:10-2 | |  |  | 2 | 107 | 6 | 4548 | 0.013828159 | 0.08296896 | 6 |
|  |  | RAFL11-07-N24 | At1g10740 / lipase -related | |  |  |  |  |  | | --- | --- | --- | --- | --- | |  |  |  |  |  | | EC:3.1.1.3  triacylglycerol lipase | | | | | | |
|  |  | RAFL08-08-G07 | At1g73920 / lipase family | |  |  |  |  |  | | --- | --- | --- | --- | --- | |  |  |  |  |  | | EC:3.1.1.3  triacylglycerol lipase | | | | | | |
| GDP-mannose metabolism | | |  |  | A | B | C | D | P | P' | N |
|  | Cluster:3-1 | |  |  | 2 | 214 | 1 | 4446 | 0.0062126224 | 0.012425245 | 2 |
|  |  | RAFL08-14-D02 | At2g39770 / GDP-mannose pyrophosphorylase | |  |  |  |  |  | | --- | --- | --- | --- | --- | |  |  |  |  |  | | EC:2.7.7.22  unknown | | | | | | |
|  |  | RAFL08-13-A07 | At2g45790 / phosphomannomutase -related | |  |  |  |  |  | | --- | --- | --- | --- | --- | |  |  |  |  |  | | EC:5.4.2.8  phosphomannomutase | | | | | | |
|  | Cluster:2-2 | |  |  | 1 | 52 | 2 | 4608 | 0.033719275 | 0.06743855 | 2 |
|  |  | RAFL09-13-E08 | At2g39770 / GDP-mannose pyrophosphorylase | |  |  |  |  |  | | --- | --- | --- | --- | --- | |  |  |  |  |  | | EC:2.7.7.22  unknown | | | | | | |
| TCA cycle variation IV | | |  |  | A | B | C | D | P | P' | N |
|  | Cluster:3-0 | |  |  | 5 | 228 | 19 | 4411 | 0.0058060726 | 0.08709109 | 15 |
|  |  | RAFL07-17-M04 | At1g04410 / malate dehydrogenase, cytosolic, putative | |  |  |  |  |  | | --- | --- | --- | --- | --- | |  |  |  |  |  | | EC:1.1.1.37  malate dehydrogenase | | | | | | |
|  |  | RAFL06-07-J21 | At1g04410 / malate dehydrogenase, cytosolic, putative | |  |  |  |  |  | | --- | --- | --- | --- | --- | |  |  |  |  |  | | EC:1.1.1.37  malate dehydrogenase | | | | | | |
|  |  | RAFL05-21-P13 | At2g42600 / phosphoenolpyruvate carboxylase | |  |  |  |  |  | | --- | --- | --- | --- | --- | |  |  |  |  |  | | EC:4.1.1.32  phosphoenolpyruvate carboxykinase | | | | | | |
|  |  | RAFL09-09-M02 | At3g47520 / malate dehydrogenase (NAD), chloroplast, putative | |  |  |  |  |  | | --- | --- | --- | --- | --- | |  |  |  |  |  | | EC:1.1.1.37  malate dehydrogenase | | | | | | |
|  |  | RAFL07-14-B18 | At2g47510 / fumarase -related | |  |  |  |  |  | | --- | --- | --- | --- | --- | |  |  |  |  |  | | EC:4.2.1.2  adenylosuccinate lyase | | | | | | |
|  | Cluster:2-0 | |  |  | 3 | 147 | 21 | 4492 | 0.040271647 | 0.6040747 | 15 |
|  |  | RAFL09-06-L20 | At1g65930 / isocitrate dehydrogenase (NADP+), putative | |  |  |  |  |  | | --- | --- | --- | --- | --- | |  |  |  |  |  | | EC:1.1.1.42  3-isopropylmalate dehydrogenase | | | | | | |
|  |  | RAFL09-07-F20 | At1g65930 / isocitrate dehydrogenase (NADP+), putative | |  |  |  |  |  | | --- | --- | --- | --- | --- | |  |  |  |  |  | | EC:1.1.1.42  3-isopropylmalate dehydrogenase | | | | | | |
|  |  | RAFL09-11-A18 | At1g65930 / isocitrate dehydrogenase (NADP+), putative | |  |  |  |  |  | | --- | --- | --- | --- | --- | |  |  |  |  |  | | EC:1.1.1.42  3-isopropylmalate dehydrogenase | | | | | | |
| TCA cycle -- aerobic respiration | | |  |  | A | B | C | D | P | P' | N |
|  | Cluster:3-0 | |  |  | 4 | 229 | 24 | 4406 | 0.048447587 | 0.87205654 | 18 |
|  |  | RAFL07-17-M04 | At1g04410 / malate dehydrogenase, cytosolic, putative | |  |  |  |  |  | | --- | --- | --- | --- | --- | |  |  |  |  |  | | EC:1.1.1.37 ,EC:1.1.99.16  malate dehydrogenase | | | | | | |
|  |  | RAFL06-07-J21 | At1g04410 / malate dehydrogenase, cytosolic, putative | |  |  |  |  |  | | --- | --- | --- | --- | --- | |  |  |  |  |  | | EC:1.1.1.37 ,EC:1.1.99.16  malate dehydrogenase | | | | | | |
|  |  | RAFL09-09-M02 | At3g47520 / malate dehydrogenase (NAD), chloroplast, putative | |  |  |  |  |  | | --- | --- | --- | --- | --- | |  |  |  |  |  | | EC:1.1.1.37 ,EC:1.1.99.16  malate dehydrogenase | | | | | | |
|  |  | RAFL07-14-B18 | At2g47510 / fumarase -related | |  |  |  |  |  | | --- | --- | --- | --- | --- | |  |  |  |  |  | | EC:4.2.1.2  adenylosuccinate lyase | | | | | | |
| lactose degradation IV | | |  |  | A | B | C | D | P | P' | N |
|  | Cluster:3-2 | |  |  | 2 | 35 | 23 | 4603 | 0.0163878 | 0.2785926 | 17 |
|  |  | RAFL09-16-F08 | At3g23820 / NAD-dependent epimerase/dehydratase family | |  |  |  |  |  | | --- | --- | --- | --- | --- | |  |  |  |  |  | | EC:5.1.3.2  UDP-glucose 4-epimerase | | | | | | |
|  |  | RAFL09-07-D12 | At3g23820 / NAD-dependent epimerase/dehydratase family | |  |  |  |  |  | | --- | --- | --- | --- | --- | |  |  |  |  |  | | EC:5.1.3.2  UDP-glucose 4-epimerase | | | | | | |
| arginine degradation II | | |  |  | A | B | C | D | P | P' | N |
|  | Cluster:9-2 | |  |  | 2 | 65 | 8 | 4588 | 0.008496844 | 0.06797475 | 8 |
|  |  | RAFL09-13-D07 | At4g34710 / arginine decarboxylase SPE2 | |  |  |  |  |  | | --- | --- | --- | --- | --- | |  |  |  |  |  | | EC:4.1.1.19  arginine decarboxylase | | | | | | |
|  |  | RAFL08-11-N01 | At4g34710 / arginine decarboxylase SPE2 | |  |  |  |  |  | | --- | --- | --- | --- | --- | |  |  |  |  |  | | EC:4.1.1.19  arginine decarboxylase | | | | | | |
|  | Cluster:10-2 | |  |  | 2 | 107 | 8 | 4546 | 0.021556646 | 0.17245317 | 8 |
|  |  | RAFL08-15-A08 | At4g34710 / arginine decarboxylase SPE2 | |  |  |  |  |  | | --- | --- | --- | --- | --- | |  |  |  |  |  | | EC:4.1.1.19  arginine decarboxylase | | | | | | |
|  |  | RAFL04-13-O07 | At3g22200 / 4-aminobutyrate aminotransferase (gamma-amino-N-butyrate transaminase/GABA transaminase/beta-alanine--oxoglutarate aminotransferase) | |  |  |  |  |  | | --- | --- | --- | --- | --- | |  |  |  |  |  | | EC:2.6.1.19  4-aminobutyrate transaminase//alanine-glyoxylate transaminase | | | | | | |
| leucine degradation II | | |  |  | A | B | C | D | P | P' | N |
|  | Cluster:10-2 | |  |  | 1 | 108 | 1 | 4553 | 0.0462095 | 0.092419 | 2 |
|  |  | RAFL06-10-M04 | At2g26800 / hydroxymethylglutaryl-CoA lyase -related | |  |  |  |  |  | | --- | --- | --- | --- | --- | |  |  |  |  |  | | EC:4.1.3.4  hydroxymethylglutaryl-CoA lyase | | | | | | |
| phenylpropanoid biosynthesis | | |  |  | A | B | C | D | P | P' | N |
|  | Cluster:9-1 | |  |  | 1 | 95 | 1 | 4566 | 0.040755685 | 0.08151137 | 2 |
|  |  | RAFL04-13-E17 | At5g20230 / plastocyanin-like domain containing protein | |  |  |  |  |  | | --- | --- | --- | --- | --- | |  |  |  |  |  | | EC:2.1.1.68  caffeic acid /5-hydroxyferulic acid o-methyltransferase | | | | | | |
| octane oxidation | | |  |  | A | B | C | D | P | P' | N |
|  | Cluster:9-0 | |  |  | 4 | 28 | 8 | 4623 | 8.7054286E-7 | 7.834886E-6 | 9 |
|  |  | RAFL05-21-E06 | At1g54100 / aldehyde dehydrogenase, putative (ALDH) | |  |  |  |  |  | | --- | --- | --- | --- | --- | |  |  |  |  |  | | EC:1.2.1.3  unknown | | | | | | |
|  |  | RAFL04-09-D07 | At1g54100 / aldehyde dehydrogenase, putative (ALDH) | |  |  |  |  |  | | --- | --- | --- | --- | --- | |  |  |  |  |  | | EC:1.2.1.3  unknown | | | | | | |
|  |  | RAFL08-15-L09 | At1g54100 / aldehyde dehydrogenase, putative (ALDH) | |  |  |  |  |  | | --- | --- | --- | --- | --- | |  |  |  |  |  | | EC:1.2.1.3  unknown | | | | | | |
|  |  | RAFL08-09-C23 | At1g54100 / aldehyde dehydrogenase, putative (ALDH) | |  |  |  |  |  | | --- | --- | --- | --- | --- | |  |  |  |  |  | | EC:1.2.1.3  unknown | | | | | | |
| lipoxygenase pathway | | |  |  | A | B | C | D | P | P' | N |
|  | Cluster:2-2 | |  |  | 2 | 51 | 5 | 4605 | 0.0025667707 | 0.015400624 | 6 |
|  |  | RAFL05-12-G03 | At5g42650 / allene oxide synthase / cytochrome P450 74A | |  |  |  |  |  | | --- | --- | --- | --- | --- | |  |  |  |  |  | | EC:4.2.1.92  allene oxide synthase | | | | | | |
|  |  | RAFL06-10-H13 | At5g42650 / allene oxide synthase / cytochrome P450 74A | |  |  |  |  |  | | --- | --- | --- | --- | --- | |  |  |  |  |  | | EC:4.2.1.92  allene oxide synthase | | | | | | |
| arginine degradation XII | | |  |  | A | B | C | D | P | P' | N |
|  | Cluster:9-2 | |  |  | 2 | 65 | 4 | 4592 | 0.0029394836 | 0.014697418 | 5 |
|  |  | RAFL09-13-D07 | At4g34710 / arginine decarboxylase SPE2 | |  |  |  |  |  | | --- | --- | --- | --- | --- | |  |  |  |  |  | | EC:4.1.1.19  arginine decarboxylase | | | | | | |
|  |  | RAFL08-11-N01 | At4g34710 / arginine decarboxylase SPE2 | |  |  |  |  |  | | --- | --- | --- | --- | --- | |  |  |  |  |  | | EC:4.1.1.19  arginine decarboxylase | | | | | | |
| β-D-glucuronide degradation | | |  |  | A | B | C | D | P | P' | N |
|  | Cluster:3-2 | |  |  | 1 | 36 | 0 | 4626 | 0.007934806 | 0.007934806 | 1 |
|  |  | RAFL08-10-H13 | At5g07830 / glycosyl hydrolase family 79 (endo-beta-glucuronidase/heparanase) | |  |  |  |  |  | | --- | --- | --- | --- | --- | |  |  |  |  |  | | EC:3.2.1.31  beta-glucuronidase | | | | | | |
| de novo biosynthesis of pyrimidine deoxyribonucleotides | | |  |  | A | B | C | D | P | P' | N |
|  | Cluster:2-1 | |  |  | 2 | 242 | 2 | 4417 | 0.01525385 | 0.045761548 | 3 |
|  |  | RAFL04-13-M20 | At4g11010 / nucleoside diphosphate kinase 3 (ndpk3) | |  |  |  |  |  | | --- | --- | --- | --- | --- | |  |  |  |  |  | | EC:2.7.4.6  nucleoside-diphosphate kinase | | | | | | |
|  |  | RAFL04-18-P17 | At5g35170 / adenylate kinase -related protein | |  |  |  |  |  | | --- | --- | --- | --- | --- | |  |  |  |  |  | | EC:2.7.4.9  guanylate kinase | | | | | | |
| pyridoxal 5'-phosphate biosynthesis | | |  |  | A | B | C | D | P | P' | N |
|  | Cluster:0-2 | |  |  | 1 | 78 | 1 | 4583 | 0.03360031 | 0.06720062 | 2 |
|  |  | RAFL04-15-A04 | At4g15560 / DEF (CLA1) protein | |  |  |  |  |  | | --- | --- | --- | --- | --- | |  |  |  |  |  | | EC:4.1.3.37  1-deoxy-D-xylulose-5-phosphate synthase | | | | | | |
| de novo biosynthesis of pyrimidine ribonucleotides | | |  |  | A | B | C | D | P | P' | N |
|  | Cluster:2-0 | |  |  | 2 | 148 | 6 | 4507 | 0.025339978 | 0.12669988 | 5 |
|  |  | RAFL04-20-D06 | At4g09320 / nucleoside-diphosphate kinase | |  |  |  |  |  | | --- | --- | --- | --- | --- | |  |  |  |  |  | | EC:2.7.4.6  nucleoside-diphosphate kinase | | | | | | |
|  |  | RAFL09-12-G17 | At1g29900 / carbamoylphosphate synthetase -related | |  |  |  |  |  | | --- | --- | --- | --- | --- | |  |  |  |  |  | | EC:6.3.5.5  carbamoyl-phosphate synthase | | | | | | |
|  | Cluster:3-1 | |  |  | 2 | 214 | 6 | 4441 | 0.049727194 | 0.24863598 | 5 |
|  |  | RAFL07-16-F16 | At3g20330 / aspartate carbamoyltransferase precursor (aspartate transcarbamylase) | |  |  |  |  |  | | --- | --- | --- | --- | --- | |  |  |  |  |  | | EC:2.1.3.2  unknown | | | | | | |
|  |  | RAFL05-01-N17 | At5g67220 / nitrogen regulation protein family (NIFR3) | |  |  |  |  |  | | --- | --- | --- | --- | --- | |  |  |  |  |  | | EC:1.3.3.1  dihydroorotate dehydrogenase | | | | | | |
| asparagine biosynthesis I | | |  |  | A | B | C | D | P | P' | N |
|  | Cluster:6-1 | |  |  | 2 | 313 | 3 | 4345 | 0.039689664 | 0.15875866 | 4 |
|  |  | RAFL04-17-L05 | At5g19550 / aspartate aminotransferase, cytoplasmic isozyme 1 (transaminase A/Asp2) | |  |  |  |  |  | | --- | --- | --- | --- | --- | |  |  |  |  |  | | EC:2.6.1.1  aspartate transaminase | | | | | | |
|  |  | RAFL11-09-A12 | At5g19550 / aspartate aminotransferase, cytoplasmic isozyme 1 (transaminase A/Asp2) | |  |  |  |  |  | | --- | --- | --- | --- | --- | |  |  |  |  |  | | EC:2.6.1.1  aspartate transaminase | | | | | | |
| aspartate degradation II | | |  |  | A | B | C | D | P | P' | N |
|  | Cluster:3-0 | |  |  | 4 | 229 | 6 | 4424 | 0.0010050355 | 0.0060302126 | 6 |
|  |  | RAFL07-17-M04 | At1g04410 / malate dehydrogenase, cytosolic, putative | |  |  |  |  |  | | --- | --- | --- | --- | --- | |  |  |  |  |  | | EC:1.1.1.37  malate dehydrogenase | | | | | | |
|  |  | RAFL06-07-J21 | At1g04410 / malate dehydrogenase, cytosolic, putative | |  |  |  |  |  | | --- | --- | --- | --- | --- | |  |  |  |  |  | | EC:1.1.1.37  malate dehydrogenase | | | | | | |
|  |  | RAFL09-09-M02 | At3g47520 / malate dehydrogenase (NAD), chloroplast, putative | |  |  |  |  |  | | --- | --- | --- | --- | --- | |  |  |  |  |  | | EC:1.1.1.37  malate dehydrogenase | | | | | | |
|  |  | RAFL09-07-B08 | At2g30970 / aspartate aminotransferase, mitochondrial (transaminase A/Asp1) | |  |  |  |  |  | | --- | --- | --- | --- | --- | |  |  |  |  |  | | EC:2.6.1.1  1-aminocyclopropane-1-carboxylate synthase | | | | | | |
| acetate fermentation | | |  |  | A | B | C | D | P | P' | N |
|  | Cluster:0-1 | |  |  | 15 | 91 | 36 | 4521 | 1.3226566E-13 | 2.645313E-12 | 20 |
|  |  | RAFL07-18-C20 | At2g21330 / fructose-bisphosphate aldolase, putative | |  |  |  |  |  | | --- | --- | --- | --- | --- | |  |  |  |  |  | | EC:4.1.2.13  fructose-bisphosphate aldolase | | | | | | |
|  |  | RAFL09-15-L04 | At3g12780 / phosphoglycerate kinase -related | |  |  |  |  |  | | --- | --- | --- | --- | --- | |  |  |  |  |  | | EC:2.7.2.3  phosphoglycerate kinase | | | | | | |
|  |  | RAFL07-14-L16 | At3g12780 / phosphoglycerate kinase -related | |  |  |  |  |  | | --- | --- | --- | --- | --- | |  |  |  |  |  | | EC:2.7.2.3  phosphoglycerate kinase | | | | | | |
|  |  | RAFL08-18-C10 | At2g21330 / fructose-bisphosphate aldolase, putative | |  |  |  |  |  | | --- | --- | --- | --- | --- | |  |  |  |  |  | | EC:4.1.2.13  fructose-bisphosphate aldolase | | | | | | |
|  |  | RAFL07-12-E12 | At2g21330 / fructose-bisphosphate aldolase, putative | |  |  |  |  |  | | --- | --- | --- | --- | --- | |  |  |  |  |  | | EC:4.1.2.13  fructose-bisphosphate aldolase | | | | | | |
|  |  | RAFL07-07-I23 | At1g56190 / phosphoglycerate kinase -related | |  |  |  |  |  | | --- | --- | --- | --- | --- | |  |  |  |  |  | | EC:2.7.2.3  phosphoglycerate kinase | | | | | | |
|  |  | RAFL07-12-M09 | At2g21330 / fructose-bisphosphate aldolase, putative | |  |  |  |  |  | | --- | --- | --- | --- | --- | |  |  |  |  |  | | EC:4.1.2.13  fructose-bisphosphate aldolase | | | | | | |
|  |  | RAFL04-09-D24 | At1g42970 / glyceraldehyde-3-phosphate dehydrogenase | |  |  |  |  |  | | --- | --- | --- | --- | --- | |  |  |  |  |  | | EC:1.2.1.12  glyceraldehyde-3-phosphate dehydrogenase | | | | | | |
|  |  | RAFL07-16-P05 | At3g12780 / phosphoglycerate kinase -related | |  |  |  |  |  | | --- | --- | --- | --- | --- | |  |  |  |  |  | | EC:2.7.2.3  phosphoglycerate kinase | | | | | | |
|  |  | RAFL09-18-L22 | At3g12780 / phosphoglycerate kinase -related | |  |  |  |  |  | | --- | --- | --- | --- | --- | |  |  |  |  |  | | EC:2.7.2.3  phosphoglycerate kinase | | | | | | |
|  |  | RAFL04-19-O21 | At4g38970 / fructose-bisphosphate aldolase, putative | |  |  |  |  |  | | --- | --- | --- | --- | --- | |  |  |  |  |  | | EC:4.1.2.13  fructose-bisphosphate aldolase | | | | | | |
|  |  | RAFL09-17-N23 | At2g21330 / fructose-bisphosphate aldolase, putative | |  |  |  |  |  | | --- | --- | --- | --- | --- | |  |  |  |  |  | | EC:4.1.2.13  fructose-bisphosphate aldolase | | | | | | |
|  |  | RAFL04-15-A14 | At1g12900 / calcium-binding protein, calreticulin -related | |  |  |  |  |  | | --- | --- | --- | --- | --- | |  |  |  |  |  | | EC:1.2.1.12  glyceraldehyde-3-phosphate dehydrogenase | | | | | | |
|  |  | RAFL07-18-J01 | At2g21330 / fructose-bisphosphate aldolase, putative | |  |  |  |  |  | | --- | --- | --- | --- | --- | |  |  |  |  |  | | EC:4.1.2.13  fructose-bisphosphate aldolase | | | | | | |
|  |  | RAFL05-07-J06 | At1g42970 / glyceraldehyde-3-phosphate dehydrogenase | |  |  |  |  |  | | --- | --- | --- | --- | --- | |  |  |  |  |  | | EC:1.2.1.12  glyceraldehyde-3-phosphate dehydrogenase | | | | | | |
|  | Cluster:1-2 | |  |  | 5 | 169 | 46 | 4443 | 0.04018175 | 0.803635 | 20 |
|  |  | RAFL05-13-B09 | At5g03300 / pfkB type carbohydrate kinase protein family | |  |  |  |  |  | | --- | --- | --- | --- | --- | |  |  |  |  |  | | EC:2.7.1.11  6-phosphofructokinase | | | | | | |
|  |  | RAFL04-09-G20 | At2g21170 / triosephosphate isomerase, chloroplast, putative | |  |  |  |  |  | | --- | --- | --- | --- | --- | |  |  |  |  |  | | EC:5.3.1.1  triose-phosphate isomerase | | | | | | |
|  |  | RAFL07-12-L15 | At1g12000 / pyrophosphate-fructose-6-phosphate 1-phosphotransferase -related | |  |  |  |  |  | | --- | --- | --- | --- | --- | |  |  |  |  |  | | EC:2.7.1.11  6-phosphofructokinase | | | | | | |
|  |  | RAFL07-15-F22 | At1g20950 / pyrophosphate-dependent phosphofructokinase alpha subunit -related | |  |  |  |  |  | | --- | --- | --- | --- | --- | |  |  |  |  |  | | EC:2.7.1.11  6-phosphofructokinase | | | | | | |
|  |  | RAFL06-13-M02 | At1g13440 / glyceraldehyde-3-phosphate dehydrogenase -related | |  |  |  |  |  | | --- | --- | --- | --- | --- | |  |  |  |  |  | | EC:1.2.1.12  glyceraldehyde-3-phosphate dehydrogenase | | | | | | |
| riboflavin and FMN and FAD biosynthesis | | |  |  | A | B | C | D | P | P' | N |
|  | Cluster:1-1 | |  |  | 1 | 104 | 1 | 4557 | 0.04453306 | 0.08906612 | 2 |
|  |  | RAFL08-12-F18 | At2g44050 / 6,7-dimethyl-8-ribityllumazine synthase precursor | |  |  |  |  |  | | --- | --- | --- | --- | --- | |  |  |  |  |  | | EC:2.5.1.9  riboflavin synthase | | | | | | |
| flavonoid biosynthesis | | |  |  | A | B | C | D | P | P' | N |
|  | Cluster:8-1 | |  |  | 2 | 160 | 1 | 4500 | 0.0035169823 | 0.0070339646 | 2 |
|  |  | RAFL04-16-O21 | At5g24530 / oxidoreductase, 2OG-Fe(II) oxygenase family | |  |  |  |  |  | | --- | --- | --- | --- | --- | |  |  |  |  |  | | EC:1.14.11.9  flavanone 3-hydroxylase||isopenicillin-N synthase | | | | | | |
|  |  | RAFL05-17-O13 | At3g21230 / 4-coumarate:CoA ligase (4-coumaroyl-CoA synthase) (4CL), putative | |  |  |  |  |  | | --- | --- | --- | --- | --- | |  |  |  |  |  | | EC:6.2.1.12  4-coumarate-CoA ligase | | | | | | |
| glycolysis IV | | |  |  | A | B | C | D | P | P' | N |
|  | Cluster:0-1 | |  |  | 15 | 91 | 29 | 4528 | 1.08628985E-14 | 1.7380638E-13 | 16 |
|  |  | RAFL07-18-C20 | At2g21330 / fructose-bisphosphate aldolase, putative | |  |  |  |  |  | | --- | --- | --- | --- | --- | |  |  |  |  |  | | EC:4.1.2.13  fructose-bisphosphate aldolase | | | | | | |
|  |  | RAFL09-15-L04 | At3g12780 / phosphoglycerate kinase -related | |  |  |  |  |  | | --- | --- | --- | --- | --- | |  |  |  |  |  | | EC:2.7.2.3  phosphoglycerate kinase | | | | | | |
|  |  | RAFL07-14-L16 | At3g12780 / phosphoglycerate kinase -related | |  |  |  |  |  | | --- | --- | --- | --- | --- | |  |  |  |  |  | | EC:2.7.2.3  phosphoglycerate kinase | | | | | | |
|  |  | RAFL08-18-C10 | At2g21330 / fructose-bisphosphate aldolase, putative | |  |  |  |  |  | | --- | --- | --- | --- | --- | |  |  |  |  |  | | EC:4.1.2.13  fructose-bisphosphate aldolase | | | | | | |
|  |  | RAFL07-12-E12 | At2g21330 / fructose-bisphosphate aldolase, putative | |  |  |  |  |  | | --- | --- | --- | --- | --- | |  |  |  |  |  | | EC:4.1.2.13  fructose-bisphosphate aldolase | | | | | | |
|  |  | RAFL07-07-I23 | At1g56190 / phosphoglycerate kinase -related | |  |  |  |  |  | | --- | --- | --- | --- | --- | |  |  |  |  |  | | EC:2.7.2.3  phosphoglycerate kinase | | | | | | |
|  |  | RAFL07-12-M09 | At2g21330 / fructose-bisphosphate aldolase, putative | |  |  |  |  |  | | --- | --- | --- | --- | --- | |  |  |  |  |  | | EC:4.1.2.13  fructose-bisphosphate aldolase | | | | | | |
|  |  | RAFL04-09-D24 | At1g42970 / glyceraldehyde-3-phosphate dehydrogenase | |  |  |  |  |  | | --- | --- | --- | --- | --- | |  |  |  |  |  | | EC:1.2.1.12 ,EC:1.2.1.9  glyceraldehyde-3-phosphate dehydrogenase  glyceraldehyde-3-phosphate dehydrogenase (NADP+)//glyceraldehyde-3-phosphate dehydrogenase | | | | | | |
|  |  | RAFL07-16-P05 | At3g12780 / phosphoglycerate kinase -related | |  |  |  |  |  | | --- | --- | --- | --- | --- | |  |  |  |  |  | | EC:2.7.2.3  phosphoglycerate kinase | | | | | | |
|  |  | RAFL09-18-L22 | At3g12780 / phosphoglycerate kinase -related | |  |  |  |  |  | | --- | --- | --- | --- | --- | |  |  |  |  |  | | EC:2.7.2.3  phosphoglycerate kinase | | | | | | |
|  |  | RAFL04-19-O21 | At4g38970 / fructose-bisphosphate aldolase, putative | |  |  |  |  |  | | --- | --- | --- | --- | --- | |  |  |  |  |  | | EC:4.1.2.13  fructose-bisphosphate aldolase | | | | | | |
|  |  | RAFL09-17-N23 | At2g21330 / fructose-bisphosphate aldolase, putative | |  |  |  |  |  | | --- | --- | --- | --- | --- | |  |  |  |  |  | | EC:4.1.2.13  fructose-bisphosphate aldolase | | | | | | |
|  |  | RAFL04-15-A14 | At1g12900 / calcium-binding protein, calreticulin -related | |  |  |  |  |  | | --- | --- | --- | --- | --- | |  |  |  |  |  | | EC:1.2.1.12  glyceraldehyde-3-phosphate dehydrogenase | | | | | | |
|  |  | RAFL07-18-J01 | At2g21330 / fructose-bisphosphate aldolase, putative | |  |  |  |  |  | | --- | --- | --- | --- | --- | |  |  |  |  |  | | EC:4.1.2.13  fructose-bisphosphate aldolase | | | | | | |
|  |  | RAFL05-07-J06 | At1g42970 / glyceraldehyde-3-phosphate dehydrogenase | |  |  |  |  |  | | --- | --- | --- | --- | --- | |  |  |  |  |  | | EC:1.2.1.12 ,EC:1.2.1.9  glyceraldehyde-3-phosphate dehydrogenase  glyceraldehyde-3-phosphate dehydrogenase (NADP+)//glyceraldehyde-3-phosphate dehydrogenase | | | | | | |
|  | Cluster:1-2 | |  |  | 5 | 169 | 39 | 4450 | 0.0228522 | 0.3656352 | 16 |
|  |  | RAFL05-13-B09 | At5g03300 / pfkB type carbohydrate kinase protein family | |  |  |  |  |  | | --- | --- | --- | --- | --- | |  |  |  |  |  | | EC:2.7.1.11  6-phosphofructokinase | | | | | | |
|  |  | RAFL04-09-G20 | At2g21170 / triosephosphate isomerase, chloroplast, putative | |  |  |  |  |  | | --- | --- | --- | --- | --- | |  |  |  |  |  | | EC:5.3.1.1  triose-phosphate isomerase | | | | | | |
|  |  | RAFL07-12-L15 | At1g12000 / pyrophosphate-fructose-6-phosphate 1-phosphotransferase -related | |  |  |  |  |  | | --- | --- | --- | --- | --- | |  |  |  |  |  | | EC:2.7.1.90 ,EC:2.7.1.11  6-phosphofructokinase  unknown | | | | | | |
|  |  | RAFL07-15-F22 | At1g20950 / pyrophosphate-dependent phosphofructokinase alpha subunit -related | |  |  |  |  |  | | --- | --- | --- | --- | --- | |  |  |  |  |  | | EC:2.7.1.90 ,EC:2.7.1.11  6-phosphofructokinase  unknown | | | | | | |
|  |  | RAFL06-13-M02 | At1g13440 / glyceraldehyde-3-phosphate dehydrogenase -related | |  |  |  |  |  | | --- | --- | --- | --- | --- | |  |  |  |  |  | | EC:1.2.1.12  glyceraldehyde-3-phosphate dehydrogenase | | | | | | |
| glyceraldehyde 3-phosphate degradation | | |  |  | A | B | C | D | P | P' | N |
|  | Cluster:0-1 | |  |  | 8 | 98 | 16 | 4541 | 2.9754789E-8 | 3.8681225E-7 | 13 |
|  |  | RAFL09-15-L04 | At3g12780 / phosphoglycerate kinase -related | |  |  |  |  |  | | --- | --- | --- | --- | --- | |  |  |  |  |  | | EC:2.7.2.3  phosphoglycerate kinase | | | | | | |
|  |  | RAFL07-14-L16 | At3g12780 / phosphoglycerate kinase -related | |  |  |  |  |  | | --- | --- | --- | --- | --- | |  |  |  |  |  | | EC:2.7.2.3  phosphoglycerate kinase | | | | | | |
|  |  | RAFL07-07-I23 | At1g56190 / phosphoglycerate kinase -related | |  |  |  |  |  | | --- | --- | --- | --- | --- | |  |  |  |  |  | | EC:2.7.2.3  phosphoglycerate kinase | | | | | | |
|  |  | RAFL04-09-D24 | At1g42970 / glyceraldehyde-3-phosphate dehydrogenase | |  |  |  |  |  | | --- | --- | --- | --- | --- | |  |  |  |  |  | | EC:1.2.1.12  glyceraldehyde-3-phosphate dehydrogenase | | | | | | |
|  |  | RAFL07-16-P05 | At3g12780 / phosphoglycerate kinase -related | |  |  |  |  |  | | --- | --- | --- | --- | --- | |  |  |  |  |  | | EC:2.7.2.3  phosphoglycerate kinase | | | | | | |
|  |  | RAFL09-18-L22 | At3g12780 / phosphoglycerate kinase -related | |  |  |  |  |  | | --- | --- | --- | --- | --- | |  |  |  |  |  | | EC:2.7.2.3  phosphoglycerate kinase | | | | | | |
|  |  | RAFL04-15-A14 | At1g12900 / calcium-binding protein, calreticulin -related | |  |  |  |  |  | | --- | --- | --- | --- | --- | |  |  |  |  |  | | EC:1.2.1.12  glyceraldehyde-3-phosphate dehydrogenase | | | | | | |
|  |  | RAFL05-07-J06 | At1g42970 / glyceraldehyde-3-phosphate dehydrogenase | |  |  |  |  |  | | --- | --- | --- | --- | --- | |  |  |  |  |  | | EC:1.2.1.12  glyceraldehyde-3-phosphate dehydrogenase | | | | | | |
| alanine biosynthesis II | | |  |  | A | B | C | D | P | P' | N |
|  | Cluster:2-1 | |  |  | 3 | 241 | 4 | 4415 | 0.0042335736 | 0.016934294 | 4 |
|  |  | RAFL09-09-I19 | At1g23310 / alanine aminotransferase -related | |  |  |  |  |  | | --- | --- | --- | --- | --- | |  |  |  |  |  | | EC:2.6.1.2  alanine transaminase | | | | | | |
|  |  | RAFL09-16-K01 | At1g23310 / alanine aminotransferase -related | |  |  |  |  |  | | --- | --- | --- | --- | --- | |  |  |  |  |  | | EC:2.6.1.2  alanine transaminase | | | | | | |
|  |  | RAFL05-07-N11 | At1g70580 / alanine aminotransferase, putative | |  |  |  |  |  | | --- | --- | --- | --- | --- | |  |  |  |  |  | | EC:2.6.1.2  alanine transaminase | | | | | | |
|  | Cluster:1-2 | |  |  | 2 | 172 | 5 | 4484 | 0.025693169 | 0.102772675 | 4 |
|  |  | RAFL05-14-F03 | At1g23310 / alanine aminotransferase -related | |  |  |  |  |  | | --- | --- | --- | --- | --- | |  |  |  |  |  | | EC:2.6.1.2  alanine transaminase | | | | | | |
|  |  | RAFL07-12-E03 | At1g23310 / alanine aminotransferase -related | |  |  |  |  |  | | --- | --- | --- | --- | --- | |  |  |  |  |  | | EC:2.6.1.2  alanine transaminase | | | | | | |
| methylglyoxal pathway | | |  |  | A | B | C | D | P | P' | N |
|  | Cluster:5-1 | |  |  | 2 | 280 | 4 | 4377 | 0.046489924 | 0.23244964 | 5 |
|  |  | RAFL08-16-B22 | At1g11840 / glyoxalase I, putative (lactoylglutathione lyase) | |  |  |  |  |  | | --- | --- | --- | --- | --- | |  |  |  |  |  | | EC:4.4.1.5  4-hydroxyphenylpyruvate dioxygenase | | | | | | |
|  |  | RAFL09-07-G14 | At1g67280 / glyoxalase I, putative (lactoylglutathione lyase) | |  |  |  |  |  | | --- | --- | --- | --- | --- | |  |  |  |  |  | | EC:4.4.1.5  4-hydroxyphenylpyruvate dioxygenase | | | | | | |
| carotenoid biosynthesis | | |  |  | A | B | C | D | P | P' | N |
|  | Cluster:0-2 | |  |  | 1 | 78 | 0 | 4584 | 0.016941883 | 0.016941883 | 1 |
|  |  | RAFL07-12-F08 | At5g17230 / phytoene synthase (geranylgeranyl-diphosphate geranylgeranyl transferase)(PSY) | |  |  |  |  |  | | --- | --- | --- | --- | --- | |  |  |  |  |  | | EC:2.5.1.32  geranylgeranyl-diphosphate geranylgeranyltransferase | | | | | | |
| dissimilatory sulfate reduction | | |  |  | A | B | C | D | P | P' | N |
|  | Cluster:2-2 | |  |  | 2 | 51 | 5 | 4605 | 0.0025667707 | 0.015400624 | 6 |
|  |  | RAFL07-10-P11 | At3g22890 / ATP sulfurylase -related | |  |  |  |  |  | | --- | --- | --- | --- | --- | |  |  |  |  |  | | EC:2.7.7.4  ATP sulfurylase | | | | | | |
|  |  | RAFL07-12-E10 | At3g22890 / ATP sulfurylase -related | |  |  |  |  |  | | --- | --- | --- | --- | --- | |  |  |  |  |  | | EC:2.7.7.4  ATP sulfurylase | | | | | | |
| vitamin E biosynthesis | | |  |  | A | B | C | D | P | P' | N |
|  | Cluster:9-1 | |  |  | 2 | 94 | 4 | 4563 | 0.005961956 | 0.023847824 | 4 |
|  |  | RAFL11-09-O05 | At1g06570 / 4-hydroxyphenylpyruvate dioxygenase (HPD) | |  |  |  |  |  | | --- | --- | --- | --- | --- | |  |  |  |  |  | | EC:1.13.11.27  4-hydroxyphenylpyruvate dioxygenase | | | | | | |
|  |  | RAFL11-12-C18 | At1g06570 / 4-hydroxyphenylpyruvate dioxygenase (HPD) | |  |  |  |  |  | | --- | --- | --- | --- | --- | |  |  |  |  |  | | EC:1.13.11.27  4-hydroxyphenylpyruvate dioxygenase | | | | | | |
|  | Cluster:9-0 | |  |  | 1 | 31 | 5 | 4626 | 0.04049657 | 0.16198628 | 4 |
|  |  | RAFL05-14-F20 | At1g06570 / 4-hydroxyphenylpyruvate dioxygenase (HPD) | |  |  |  |  |  | | --- | --- | --- | --- | --- | |  |  |  |  |  | | EC:1.13.11.27  4-hydroxyphenylpyruvate dioxygenase | | | | | | |
|  | Cluster:5-1 | |  |  | 2 | 280 | 4 | 4377 | 0.046489924 | 0.1859597 | 4 |
|  |  | RAFL08-16-B22 | At1g11840 / glyoxalase I, putative (lactoylglutathione lyase) | |  |  |  |  |  | | --- | --- | --- | --- | --- | |  |  |  |  |  | | EC:1.13.11.27  4-hydroxyphenylpyruvate dioxygenase | | | | | | |
|  |  | RAFL09-07-G14 | At1g67280 / glyoxalase I, putative (lactoylglutathione lyase) | |  |  |  |  |  | | --- | --- | --- | --- | --- | |  |  |  |  |  | | EC:1.13.11.27  4-hydroxyphenylpyruvate dioxygenase | | | | | | |
| ascorbate biosynthesis | | |  |  | A | B | C | D | P | P' | N |
|  | Cluster:3-1 | |  |  | 3 | 213 | 1 | 4446 | 3.7887847E-4 | 7.5775693E-4 | 2 |
|  |  | RAFL08-14-D02 | At2g39770 / GDP-mannose pyrophosphorylase | |  |  |  |  |  | | --- | --- | --- | --- | --- | |  |  |  |  |  | | EC:2.7.7.13  GDP-D-mannose pyrophosphorylase | | | | | | |
|  |  | RAFL08-13-A07 | At2g45790 / phosphomannomutase -related | |  |  |  |  |  | | --- | --- | --- | --- | --- | |  |  |  |  |  | | EC:5.4.2.8  phosphomannomutase | | | | | | |
|  |  | RAFL04-15-P16 | At5g28840 / NAD-dependent epimerase/dehydratase family | |  |  |  |  |  | | --- | --- | --- | --- | --- | |  |  |  |  |  | | EC:5.1.3.18  GDP-D-mannose-3,5-epimerase | | | | | | |
|  | Cluster:2-2 | |  |  | 1 | 52 | 3 | 4607 | 0.044709165 | 0.08941833 | 2 |
|  |  | RAFL09-13-E08 | At2g39770 / GDP-mannose pyrophosphorylase | |  |  |  |  |  | | --- | --- | --- | --- | --- | |  |  |  |  |  | | EC:2.7.7.13  GDP-D-mannose pyrophosphorylase | | | | | | |
| TCA cycle variation II | | |  |  | A | B | C | D | P | P' | N |
|  | Cluster:3-0 | |  |  | 5 | 228 | 4 | 4426 | 3.1898064E-5 | 1.5949032E-4 | 5 |
|  |  | RAFL07-17-M04 | At1g04410 / malate dehydrogenase, cytosolic, putative | |  |  |  |  |  | | --- | --- | --- | --- | --- | |  |  |  |  |  | | EC:1.1.1.37  malate dehydrogenase | | | | | | |
|  |  | RAFL06-07-J21 | At1g04410 / malate dehydrogenase, cytosolic, putative | |  |  |  |  |  | | --- | --- | --- | --- | --- | |  |  |  |  |  | | EC:1.1.1.37  malate dehydrogenase | | | | | | |
|  |  | RAFL05-21-P13 | At2g42600 / phosphoenolpyruvate carboxylase | |  |  |  |  |  | | --- | --- | --- | --- | --- | |  |  |  |  |  | | EC:4.1.1.31  phosphoenolpyruvate carboxylase | | | | | | |
|  |  | RAFL09-09-M02 | At3g47520 / malate dehydrogenase (NAD), chloroplast, putative | |  |  |  |  |  | | --- | --- | --- | --- | --- | |  |  |  |  |  | | EC:1.1.1.37  malate dehydrogenase | | | | | | |
|  |  | RAFL07-14-B18 | At2g47510 / fumarase -related | |  |  |  |  |  | | --- | --- | --- | --- | --- | |  |  |  |  |  | | EC:4.2.1.2  adenylosuccinate lyase | | | | | | |
| phenylpropanoid pathway, initial reactions | | |  |  | A | B | C | D | P | P' | N |
|  | Cluster:6-2 | |  |  | 3 | 173 | 3 | 4484 | 9.720464E-4 | 0.0038881856 | 4 |
|  |  | RAFL09-11-L22 | At3g53260 / phenylalanine ammonia-lyase (PAL2) | |  |  |  |  |  | | --- | --- | --- | --- | --- | |  |  |  |  |  | | EC:4.3.1.5  phenylalanine ammonia-lyase | | | | | | |
|  |  | RAFL04-13-B02 | At2g37040 / phenylalanine ammonia lyase (PAL1) | |  |  |  |  |  | | --- | --- | --- | --- | --- | |  |  |  |  |  | | EC:4.3.1.5  phenylalanine ammonia-lyase | | | | | | |
|  |  | RAFL04-16-D08 | At3g53260 / phenylalanine ammonia-lyase (PAL2) | |  |  |  |  |  | | --- | --- | --- | --- | --- | |  |  |  |  |  | | EC:4.3.1.5  phenylalanine ammonia-lyase | | | | | | |
| fatty acid elongation -- saturated | | |  |  | A | B | C | D | P | P' | N |
|  | Cluster:6-2 | |  |  | 2 | 174 | 7 | 4480 | 0.042826395 | 0.34261116 | 8 |
|  |  | RAFL05-10-D10 | At5g43760 / beta-ketoacyl-CoA synthase, putative | |  |  |  |  |  | | --- | --- | --- | --- | --- | |  |  |  |  |  | | EC:2.3.1.41  3-oxoacyl-[acyl-carrier protein] synthase | | | | | | |
|  |  | RAFL02-03-L07 | At2g26250 / beta-ketoacyl-CoA synthase family (FIDDLEHEAD) (FDH) | |  |  |  |  |  | | --- | --- | --- | --- | --- | |  |  |  |  |  | | EC:2.3.1.41  3-oxoacyl-[acyl-carrier protein] synthase | | | | | | |
| ribitol degradation | | |  |  | A | B | C | D | P | P' | N |
|  | Cluster:3-0 | |  |  | 2 | 231 | 1 | 4429 | 0.007213332 | 0.014426664 | 2 |
|  |  | RAFL05-21-O08 | At5g61410 / ribulose-5-phosphate-3-epimerase | |  |  |  |  |  | | --- | --- | --- | --- | --- | |  |  |  |  |  | | EC:5.1.3.1  ribulose-phosphate 3-epimerase | | | | | | |
|  |  | RAFL11-02-F16 | At3g01850 / D-ribulose-5-phosphate 3-epimerase -related | |  |  |  |  |  | | --- | --- | --- | --- | --- | |  |  |  |  |  | | EC:5.1.3.1  ribulose-phosphate 3-epimerase | | | | | | |
| respiration (anaerobic)-- electron donors reaction list | | |  |  | A | B | C | D | P | P' | N |
|  | Cluster:4-1 | |  |  | 5 | 305 | 7 | 4346 | 6.7494786E-4 | 0.0033747396 | 5 |
|  |  | RAFL05-04-N24 | At3g18410 / expressed protein | |  |  |  |  |  | | --- | --- | --- | --- | --- | |  |  |  |  |  | | EC:1.6.5.3  NADH dehydrogenase (ubiquinone) | | | | | | |
|  |  | RAFL11-02-J20 | At3g12260 / expressed protein | |  |  |  |  |  | | --- | --- | --- | --- | --- | |  |  |  |  |  | | EC:1.6.5.3  NADH dehydrogenase (ubiquinone) | | | | | | |
|  |  | RAFL09-10-O11 | At3g12260 / expressed protein | |  |  |  |  |  | | --- | --- | --- | --- | --- | |  |  |  |  |  | | EC:1.6.5.3  NADH dehydrogenase (ubiquinone) | | | | | | |
|  |  | RAFL09-18-I01 | At5g08530 / NADH-ubiquinone oxidoreductase (mitochondrial), putative | |  |  |  |  |  | | --- | --- | --- | --- | --- | |  |  |  |  |  | | EC:1.6.5.3 ,EC:1.18.99.1  NADH dehydrogenase (ubiquinone) | | | | | | |
|  |  | RAFL06-08-D19 | At5g37510 / NADH dehydrogenase (ubiquinone), mitochondrial, putative | |  |  |  |  |  | | --- | --- | --- | --- | --- | |  |  |  |  |  | | EC:1.2.1.2 ,EC:1.6.5.3  NADH dehydrogenase (ubiquinone)  formate dehydrogenase | | | | | | |
|  | Cluster:6-1 | |  |  | 3 | 312 | 9 | 4339 | 0.042557452 | 0.21278727 | 5 |
|  |  | RAFL06-10-E05 | At1g16700 / NADH:ubiquinone oxidoreductase -related | |  |  |  |  |  | | --- | --- | --- | --- | --- | |  |  |  |  |  | | EC:1.6.5.3  NADH dehydrogenase (ubiquinone) | | | | | | |
|  |  | RAFL05-08-F21 | At3g03100 / expressed protein | |  |  |  |  |  | | --- | --- | --- | --- | --- | |  |  |  |  |  | | EC:1.6.5.3  NADH dehydrogenase (ubiquinone) | | | | | | |
|  |  | RAFL07-07-N09 | At5g37510 / NADH dehydrogenase (ubiquinone), mitochondrial, putative | |  |  |  |  |  | | --- | --- | --- | --- | --- | |  |  |  |  |  | | EC:1.2.1.2 ,EC:1.6.5.3  NADH dehydrogenase (ubiquinone)  formate dehydrogenase | | | | | | |
|  | Cluster:6-0 | |  |  | 2 | 138 | 10 | 4513 | 0.048507567 | 0.24253784 | 5 |
|  |  | RAFL08-12-A05 | At5g11770 / NADH dehydrogenase (ubiquinone) | |  |  |  |  |  | | --- | --- | --- | --- | --- | |  |  |  |  |  | | EC:1.6.5.3  NADH dehydrogenase (ubiquinone) | | | | | | |
|  |  | RAFL05-17-L16 | At1g79010 / NADH dehydrogenase -related | |  |  |  |  |  | | --- | --- | --- | --- | --- | |  |  |  |  |  | | EC:1.6.5.3  NADH dehydrogenase (ubiquinone) | | | | | | |
| sorbitol degradation | | |  |  | A | B | C | D | P | P' | N |
|  | Cluster:0-1 | |  |  | 7 | 99 | 7 | 4550 | 7.745682E-9 | 3.8728412E-8 | 5 |
|  |  | RAFL07-18-C20 | At2g21330 / fructose-bisphosphate aldolase, putative | |  |  |  |  |  | | --- | --- | --- | --- | --- | |  |  |  |  |  | | EC:4.1.2.13  fructose-bisphosphate aldolase | | | | | | |
|  |  | RAFL08-18-C10 | At2g21330 / fructose-bisphosphate aldolase, putative | |  |  |  |  |  | | --- | --- | --- | --- | --- | |  |  |  |  |  | | EC:4.1.2.13  fructose-bisphosphate aldolase | | | | | | |
|  |  | RAFL07-12-E12 | At2g21330 / fructose-bisphosphate aldolase, putative | |  |  |  |  |  | | --- | --- | --- | --- | --- | |  |  |  |  |  | | EC:4.1.2.13  fructose-bisphosphate aldolase | | | | | | |
|  |  | RAFL07-12-M09 | At2g21330 / fructose-bisphosphate aldolase, putative | |  |  |  |  |  | | --- | --- | --- | --- | --- | |  |  |  |  |  | | EC:4.1.2.13  fructose-bisphosphate aldolase | | | | | | |
|  |  | RAFL04-19-O21 | At4g38970 / fructose-bisphosphate aldolase, putative | |  |  |  |  |  | | --- | --- | --- | --- | --- | |  |  |  |  |  | | EC:4.1.2.13  fructose-bisphosphate aldolase | | | | | | |
|  |  | RAFL09-17-N23 | At2g21330 / fructose-bisphosphate aldolase, putative | |  |  |  |  |  | | --- | --- | --- | --- | --- | |  |  |  |  |  | | EC:4.1.2.13  fructose-bisphosphate aldolase | | | | | | |
|  |  | RAFL07-18-J01 | At2g21330 / fructose-bisphosphate aldolase, putative | |  |  |  |  |  | | --- | --- | --- | --- | --- | |  |  |  |  |  | | EC:4.1.2.13  fructose-bisphosphate aldolase | | | | | | |
|  | Cluster:1-2 | |  |  | 3 | 171 | 11 | 4478 | 0.013720686 | 0.068603426 | 5 |
|  |  | RAFL05-13-B09 | At5g03300 / pfkB type carbohydrate kinase protein family | |  |  |  |  |  | | --- | --- | --- | --- | --- | |  |  |  |  |  | | EC:2.7.1.11  6-phosphofructokinase | | | | | | |
|  |  | RAFL07-12-L15 | At1g12000 / pyrophosphate-fructose-6-phosphate 1-phosphotransferase -related | |  |  |  |  |  | | --- | --- | --- | --- | --- | |  |  |  |  |  | | EC:2.7.1.11  6-phosphofructokinase | | | | | | |
|  |  | RAFL07-15-F22 | At1g20950 / pyrophosphate-dependent phosphofructokinase alpha subunit -related | |  |  |  |  |  | | --- | --- | --- | --- | --- | |  |  |  |  |  | | EC:2.7.1.11  6-phosphofructokinase | | | | | | |
| (deoxy)ribose phosphate degradation | | |  |  | A | B | C | D | P | P' | N |
|  | Cluster:0-2 | |  |  | 1 | 78 | 1 | 4583 | 0.03360031 | 0.06720062 | 2 |
|  |  | RAFL07-12-I18 | At2g45290 / transketolase precursor -related | |  |  |  |  |  | | --- | --- | --- | --- | --- | |  |  |  |  |  | | EC:2.2.1.1  transketolase | | | | | | |
| non-phosphorylated glucose degradation | | |  |  | A | B | C | D | P | P' | N |
|  | Cluster:9-0 | |  |  | 4 | 28 | 13 | 4618 | 4.0860064E-6 | 4.0860064E-5 | 10 |
|  |  | RAFL05-21-E06 | At1g54100 / aldehyde dehydrogenase, putative (ALDH) | |  |  |  |  |  | | --- | --- | --- | --- | --- | |  |  |  |  |  | | EC:1.2.1.3  unknown | | | | | | |
|  |  | RAFL04-09-D07 | At1g54100 / aldehyde dehydrogenase, putative (ALDH) | |  |  |  |  |  | | --- | --- | --- | --- | --- | |  |  |  |  |  | | EC:1.2.1.3  unknown | | | | | | |
|  |  | RAFL08-15-L09 | At1g54100 / aldehyde dehydrogenase, putative (ALDH) | |  |  |  |  |  | | --- | --- | --- | --- | --- | |  |  |  |  |  | | EC:1.2.1.3  unknown | | | | | | |
|  |  | RAFL08-09-C23 | At1g54100 / aldehyde dehydrogenase, putative (ALDH) | |  |  |  |  |  | | --- | --- | --- | --- | --- | |  |  |  |  |  | | EC:1.2.1.3  unknown | | | | | | |
|  | Cluster:3-1 | |  |  | 4 | 212 | 13 | 4434 | 0.006616924 | 0.06616924 | 10 |
|  |  | RAFL05-11-L02 | At4g25900 / aldose 1-epimerase family | |  |  |  |  |  | | --- | --- | --- | --- | --- | |  |  |  |  |  | | EC:5.1.3.3  aldose 1-epimerase | | | | | | |
|  |  | RAFL05-21-G03 | At3g22960 / pyruvate kinase, putative | |  |  |  |  |  | | --- | --- | --- | --- | --- | |  |  |  |  |  | | EC:2.7.1.40  pyruvate kinase | | | | | | |
|  |  | RAFL04-13-O10 | At2g29560 / enolase (2-phospho-D-glycerate hydroylase) -related | |  |  |  |  |  | | --- | --- | --- | --- | --- | |  |  |  |  |  | | EC:4.2.1.11  phosphopyruvate hydratase | | | | | | |
|  |  | RAFL09-12-D13 | At5g52920 / pyruvate kinase, putative | |  |  |  |  |  | | --- | --- | --- | --- | --- | |  |  |  |  |  | | EC:2.7.1.40  pyruvate kinase | | | | | | |
| glycerol metabolism | | |  |  | A | B | C | D | P | P' | N |
|  | Cluster:8-2 | |  |  | 1 | 61 | 2 | 4599 | 0.0393688 | 0.1181064 | 3 |
|  |  | RAFL06-11-H07 | At1g80460 / glycerol kinase -related | |  |  |  |  |  | | --- | --- | --- | --- | --- | |  |  |  |  |  | | EC:2.7.1.30  glycerol kinase | | | | | | |
| colanic acid building blocks biosynthesis | | |  |  | A | B | C | D | P | P' | N |
|  | Cluster:3-2 | |  |  | 2 | 35 | 22 | 4604 | 0.015151827 | 0.2272774 | 15 |
|  |  | RAFL09-16-F08 | At3g23820 / NAD-dependent epimerase/dehydratase family | |  |  |  |  |  | | --- | --- | --- | --- | --- | |  |  |  |  |  | | EC:4.2.1.47 ,EC:5.1.3.2  UDP-glucose 4-epimerase  3-beta-hydroxy-delta5-steroid dehydrogenase | | | | | | |
|  |  | RAFL09-07-D12 | At3g23820 / NAD-dependent epimerase/dehydratase family | |  |  |  |  |  | | --- | --- | --- | --- | --- | |  |  |  |  |  | | EC:4.2.1.47 ,EC:5.1.3.2  UDP-glucose 4-epimerase  3-beta-hydroxy-delta5-steroid dehydrogenase | | | | | | |
| isoleucine biosynthesis I | | |  |  | A | B | C | D | P | P' | N |
|  | Cluster:4-0 | |  |  | 2 | 238 | 5 | 4418 | 0.046670057 | 0.23335029 | 5 |
|  |  | RAFL06-16-L13 | At5g54960 / pyruvate decarboxylase (gb|AAB16855.1) | |  |  |  |  |  | | --- | --- | --- | --- | --- | |  |  |  |  |  | | EC:4.1.3.18  acetolactate synthase | | | | | | |
|  |  | RAFL09-18-P18 | At3g48560 / acetolactate synthase | |  |  |  |  |  | | --- | --- | --- | --- | --- | |  |  |  |  |  | | EC:4.1.3.18  acetolactate synthase | | | | | | |
|  | Cluster:2-1 | |  |  | 2 | 242 | 5 | 4414 | 0.04810191 | 0.24050954 | 5 |
|  |  | RAFL02-01-G08 | At3g58610 / ketol-acid reductoisomerase | |  |  |  |  |  | | --- | --- | --- | --- | --- | |  |  |  |  |  | | EC:1.1.1.86  ketol-acid reductoisomerase | | | | | | |
|  |  | RAFL04-13-D06 | At3g23940 / dihydroxyacid dehydratase -related | |  |  |  |  |  | | --- | --- | --- | --- | --- | |  |  |  |  |  | | EC:4.2.1.9  unknown | | | | | | |
| lysine biosynthesis I | | |  |  | A | B | C | D | P | P' | N |
|  | Cluster:2-1 | |  |  | 2 | 242 | 3 | 4416 | 0.024550742 | 0.098202966 | 4 |
|  |  | RAFL06-12-N06 | At3g53580 / diaminopimelate epimerase - like protein | |  |  |  |  |  | | --- | --- | --- | --- | --- | |  |  |  |  |  | | EC:5.1.1.7  diaminopimelate epimerase | | | | | | |
|  |  | RAFL06-15-B04 | At1g14810 / aspartate-semialdehyde dehydrogenase -related | |  |  |  |  |  | | --- | --- | --- | --- | --- | |  |  |  |  |  | | EC:1.2.1.11  aspartate-semialdehyde dehydrogenase | | | | | | |
| glycine degradation I | | |  |  | A | B | C | D | P | P' | N |
|  | Cluster:0-2 | |  |  | 7 | 72 | 5 | 4579 | 2.2650871E-10 | 1.1325436E-9 | 5 |
|  |  | RAFL11-06-P03 | At4g33010 / glycine dehydrogenase (decarboxylating) (glycine decarboxylase/glycine cleavage system P-protein), putative | |  |  |  |  |  | | --- | --- | --- | --- | --- | |  |  |  |  |  | | EC:1.4.4.2  glycine dehydrogenase (decarboxylating) | | | | | | |
|  |  | RAFL07-10-O06 | At4g33010 / glycine dehydrogenase (decarboxylating) (glycine decarboxylase/glycine cleavage system P-protein), putative | |  |  |  |  |  | | --- | --- | --- | --- | --- | |  |  |  |  |  | | EC:1.4.4.2  glycine dehydrogenase (decarboxylating) | | | | | | |
|  |  | RAFL09-06-E16 | At2g26080 / glycine dehydrogenase (decarboxylating) (glycine decarboxylase/glycine cleavage system P-protein), putative | |  |  |  |  |  | | --- | --- | --- | --- | --- | |  |  |  |  |  | | EC:1.4.4.2  glycine dehydrogenase (decarboxylating) | | | | | | |
|  |  | RAFL08-11-O04 | At4g33010 / glycine dehydrogenase (decarboxylating) (glycine decarboxylase/glycine cleavage system P-protein), putative | |  |  |  |  |  | | --- | --- | --- | --- | --- | |  |  |  |  |  | | EC:1.4.4.2  glycine dehydrogenase (decarboxylating) | | | | | | |
|  |  | RAFL11-06-F06 | At4g33010 / glycine dehydrogenase (decarboxylating) (glycine decarboxylase/glycine cleavage system P-protein), putative | |  |  |  |  |  | | --- | --- | --- | --- | --- | |  |  |  |  |  | | EC:1.4.4.2  glycine dehydrogenase (decarboxylating) | | | | | | |
|  |  | RAFL07-18-E18 | At4g33010 / glycine dehydrogenase (decarboxylating) (glycine decarboxylase/glycine cleavage system P-protein), putative | |  |  |  |  |  | | --- | --- | --- | --- | --- | |  |  |  |  |  | | EC:1.4.4.2  glycine dehydrogenase (decarboxylating) | | | | | | |
|  |  | RAFL09-09-C13 | At4g33010 / glycine dehydrogenase (decarboxylating) (glycine decarboxylase/glycine cleavage system P-protein), putative | |  |  |  |  |  | | --- | --- | --- | --- | --- | |  |  |  |  |  | | EC:1.4.4.2  glycine dehydrogenase (decarboxylating) | | | | | | |
|  | Cluster:0-1 | |  |  | 2 | 104 | 10 | 4547 | 0.029122537 | 0.14561269 | 5 |
|  |  | RAFL06-13-H16 | At1g11860 / aminomethyltransferase-related precursor protein | |  |  |  |  |  | | --- | --- | --- | --- | --- | |  |  |  |  |  | | EC:2.1.2.10  aminomethyltransferase | | | | | | |
|  |  | RAFL05-04-M03 | At1g32470 / glycine cleavage system H protein precursor -related | |  |  |  |  |  | | --- | --- | --- | --- | --- | |  |  |  |  |  | | EC:1.4.4.2  glycine dehydrogenase (decarboxylating) | | | | | | |
| cysteine biosynthesis II | | |  |  | A | B | C | D | P | P' | N |
|  | Cluster:3-1 | |  |  | 2 | 214 | 0 | 4447 | 0.0021362621 | 0.0021362621 | 1 |
|  |  | RAFL04-10-L08 | At4g34200 / D-3-phosphoglycerate dehydrogenase (3-PGDH), putative | |  |  |  |  |  | | --- | --- | --- | --- | --- | |  |  |  |  |  | | EC:1.1.1.95  phosphoglycerate dehydrogenase | | | | | | |
|  |  | RAFL04-18-D17 | At4g35630 / phosphoserine aminotransferase | |  |  |  |  |  | | --- | --- | --- | --- | --- | |  |  |  |  |  | | EC:2.6.1.52  triacylglycerol lipase | | | | | | |
| phenylalanine degradation I | | |  |  | A | B | C | D | P | P' | N |
|  | Cluster:3-0 | |  |  | 5 | 228 | 15 | 4415 | 0.0024938595 | 0.027432455 | 11 |
|  |  | RAFL07-17-M04 | At1g04410 / malate dehydrogenase, cytosolic, putative | |  |  |  |  |  | | --- | --- | --- | --- | --- | |  |  |  |  |  | | EC:1.1.1.37  malate dehydrogenase | | | | | | |
|  |  | RAFL06-07-J21 | At1g04410 / malate dehydrogenase, cytosolic, putative | |  |  |  |  |  | | --- | --- | --- | --- | --- | |  |  |  |  |  | | EC:1.1.1.37  malate dehydrogenase | | | | | | |
|  |  | RAFL05-21-P13 | At2g42600 / phosphoenolpyruvate carboxylase | |  |  |  |  |  | | --- | --- | --- | --- | --- | |  |  |  |  |  | | EC:4.1.1.32  phosphoenolpyruvate carboxykinase | | | | | | |
|  |  | RAFL09-09-M02 | At3g47520 / malate dehydrogenase (NAD), chloroplast, putative | |  |  |  |  |  | | --- | --- | --- | --- | --- | |  |  |  |  |  | | EC:1.1.1.37  malate dehydrogenase | | | | | | |
|  |  | RAFL07-14-B18 | At2g47510 / fumarase -related | |  |  |  |  |  | | --- | --- | --- | --- | --- | |  |  |  |  |  | | EC:4.2.1.2  adenylosuccinate lyase | | | | | | |
| aspartate biosynthesis and degradation | | |  |  | A | B | C | D | P | P' | N |
|  | Cluster:6-1 | |  |  | 2 | 313 | 2 | 4346 | 0.024916494 | 0.074749485 | 3 |
|  |  | RAFL04-17-L05 | At5g19550 / aspartate aminotransferase, cytoplasmic isozyme 1 (transaminase A/Asp2) | |  |  |  |  |  | | --- | --- | --- | --- | --- | |  |  |  |  |  | | EC:2.6.1.1  1-aminocyclopropane-1-carboxylate synthase | | | | | | |
|  |  | RAFL11-09-A12 | At5g19550 / aspartate aminotransferase, cytoplasmic isozyme 1 (transaminase A/Asp2) | |  |  |  |  |  | | --- | --- | --- | --- | --- | |  |  |  |  |  | | EC:2.6.1.1  1-aminocyclopropane-1-carboxylate synthase | | | | | | |
| proline biosynthesis I | | |  |  | A | B | C | D | P | P' | N |
|  | Cluster:9-0 | |  |  | 4 | 28 | 5 | 4626 | 2.2481943E-7 | 1.1240971E-6 | 5 |
|  |  | RAFL05-21-E06 | At1g54100 / aldehyde dehydrogenase, putative (ALDH) | |  |  |  |  |  | | --- | --- | --- | --- | --- | |  |  |  |  |  | | EC:1.2.1.41  1-pyrroline-5-carboxylate dehydrogenase | | | | | | |
|  |  | RAFL04-09-D07 | At1g54100 / aldehyde dehydrogenase, putative (ALDH) | |  |  |  |  |  | | --- | --- | --- | --- | --- | |  |  |  |  |  | | EC:1.2.1.41  1-pyrroline-5-carboxylate dehydrogenase | | | | | | |
|  |  | RAFL08-15-L09 | At1g54100 / aldehyde dehydrogenase, putative (ALDH) | |  |  |  |  |  | | --- | --- | --- | --- | --- | |  |  |  |  |  | | EC:1.2.1.41  1-pyrroline-5-carboxylate dehydrogenase | | | | | | |
|  |  | RAFL08-09-C23 | At1g54100 / aldehyde dehydrogenase, putative (ALDH) | |  |  |  |  |  | | --- | --- | --- | --- | --- | |  |  |  |  |  | | EC:1.2.1.41  1-pyrroline-5-carboxylate dehydrogenase | | | | | | |
| fructose degradation (anaerobic) | | |  |  | A | B | C | D | P | P' | N |
|  | Cluster:0-1 | |  |  | 15 | 91 | 29 | 4528 | 1.08628985E-14 | 1.6294348E-13 | 15 |
|  |  | RAFL07-18-C20 | At2g21330 / fructose-bisphosphate aldolase, putative | |  |  |  |  |  | | --- | --- | --- | --- | --- | |  |  |  |  |  | | EC:4.1.2.13  fructose-bisphosphate aldolase | | | | | | |
|  |  | RAFL09-15-L04 | At3g12780 / phosphoglycerate kinase -related | |  |  |  |  |  | | --- | --- | --- | --- | --- | |  |  |  |  |  | | EC:2.7.2.3  phosphoglycerate kinase | | | | | | |
|  |  | RAFL07-14-L16 | At3g12780 / phosphoglycerate kinase -related | |  |  |  |  |  | | --- | --- | --- | --- | --- | |  |  |  |  |  | | EC:2.7.2.3  phosphoglycerate kinase | | | | | | |
|  |  | RAFL08-18-C10 | At2g21330 / fructose-bisphosphate aldolase, putative | |  |  |  |  |  | | --- | --- | --- | --- | --- | |  |  |  |  |  | | EC:4.1.2.13  fructose-bisphosphate aldolase | | | | | | |
|  |  | RAFL07-12-E12 | At2g21330 / fructose-bisphosphate aldolase, putative | |  |  |  |  |  | | --- | --- | --- | --- | --- | |  |  |  |  |  | | EC:4.1.2.13  fructose-bisphosphate aldolase | | | | | | |
|  |  | RAFL07-07-I23 | At1g56190 / phosphoglycerate kinase -related | |  |  |  |  |  | | --- | --- | --- | --- | --- | |  |  |  |  |  | | EC:2.7.2.3  phosphoglycerate kinase | | | | | | |
|  |  | RAFL07-12-M09 | At2g21330 / fructose-bisphosphate aldolase, putative | |  |  |  |  |  | | --- | --- | --- | --- | --- | |  |  |  |  |  | | EC:4.1.2.13  fructose-bisphosphate aldolase | | | | | | |
|  |  | RAFL04-09-D24 | At1g42970 / glyceraldehyde-3-phosphate dehydrogenase | |  |  |  |  |  | | --- | --- | --- | --- | --- | |  |  |  |  |  | | EC:1.2.1.12  glyceraldehyde-3-phosphate dehydrogenase | | | | | | |
|  |  | RAFL07-16-P05 | At3g12780 / phosphoglycerate kinase -related | |  |  |  |  |  | | --- | --- | --- | --- | --- | |  |  |  |  |  | | EC:2.7.2.3  phosphoglycerate kinase | | | | | | |
|  |  | RAFL09-18-L22 | At3g12780 / phosphoglycerate kinase -related | |  |  |  |  |  | | --- | --- | --- | --- | --- | |  |  |  |  |  | | EC:2.7.2.3  phosphoglycerate kinase | | | | | | |
|  |  | RAFL04-19-O21 | At4g38970 / fructose-bisphosphate aldolase, putative | |  |  |  |  |  | | --- | --- | --- | --- | --- | |  |  |  |  |  | | EC:4.1.2.13  fructose-bisphosphate aldolase | | | | | | |
|  |  | RAFL09-17-N23 | At2g21330 / fructose-bisphosphate aldolase, putative | |  |  |  |  |  | | --- | --- | --- | --- | --- | |  |  |  |  |  | | EC:4.1.2.13  fructose-bisphosphate aldolase | | | | | | |
|  |  | RAFL04-15-A14 | At1g12900 / calcium-binding protein, calreticulin -related | |  |  |  |  |  | | --- | --- | --- | --- | --- | |  |  |  |  |  | | EC:1.2.1.12  glyceraldehyde-3-phosphate dehydrogenase | | | | | | |
|  |  | RAFL07-18-J01 | At2g21330 / fructose-bisphosphate aldolase, putative | |  |  |  |  |  | | --- | --- | --- | --- | --- | |  |  |  |  |  | | EC:4.1.2.13  fructose-bisphosphate aldolase | | | | | | |
|  |  | RAFL05-07-J06 | At1g42970 / glyceraldehyde-3-phosphate dehydrogenase | |  |  |  |  |  | | --- | --- | --- | --- | --- | |  |  |  |  |  | | EC:1.2.1.12  glyceraldehyde-3-phosphate dehydrogenase | | | | | | |
|  | Cluster:1-2 | |  |  | 5 | 169 | 39 | 4450 | 0.0228522 | 0.34278297 | 15 |
|  |  | RAFL05-13-B09 | At5g03300 / pfkB type carbohydrate kinase protein family | |  |  |  |  |  | | --- | --- | --- | --- | --- | |  |  |  |  |  | | EC:2.7.1.11  6-phosphofructokinase | | | | | | |
|  |  | RAFL04-09-G20 | At2g21170 / triosephosphate isomerase, chloroplast, putative | |  |  |  |  |  | | --- | --- | --- | --- | --- | |  |  |  |  |  | | EC:5.3.1.1  triose-phosphate isomerase | | | | | | |
|  |  | RAFL07-12-L15 | At1g12000 / pyrophosphate-fructose-6-phosphate 1-phosphotransferase -related | |  |  |  |  |  | | --- | --- | --- | --- | --- | |  |  |  |  |  | | EC:2.7.1.11  6-phosphofructokinase | | | | | | |
|  |  | RAFL07-15-F22 | At1g20950 / pyrophosphate-dependent phosphofructokinase alpha subunit -related | |  |  |  |  |  | | --- | --- | --- | --- | --- | |  |  |  |  |  | | EC:2.7.1.11  6-phosphofructokinase | | | | | | |
|  |  | RAFL06-13-M02 | At1g13440 / glyceraldehyde-3-phosphate dehydrogenase -related | |  |  |  |  |  | | --- | --- | --- | --- | --- | |  |  |  |  |  | | EC:1.2.1.12  glyceraldehyde-3-phosphate dehydrogenase | | | | | | |
| alanine degradation III | | |  |  | A | B | C | D | P | P' | N |
|  | Cluster:2-1 | |  |  | 3 | 241 | 4 | 4415 | 0.0042335736 | 0.016934294 | 4 |
|  |  | RAFL09-09-I19 | At1g23310 / alanine aminotransferase -related | |  |  |  |  |  | | --- | --- | --- | --- | --- | |  |  |  |  |  | | EC:2.6.1.2  alanine transaminase | | | | | | |
|  |  | RAFL09-16-K01 | At1g23310 / alanine aminotransferase -related | |  |  |  |  |  | | --- | --- | --- | --- | --- | |  |  |  |  |  | | EC:2.6.1.2  alanine transaminase | | | | | | |
|  |  | RAFL05-07-N11 | At1g70580 / alanine aminotransferase, putative | |  |  |  |  |  | | --- | --- | --- | --- | --- | |  |  |  |  |  | | EC:2.6.1.2  alanine transaminase | | | | | | |
|  | Cluster:1-2 | |  |  | 2 | 172 | 5 | 4484 | 0.025693169 | 0.102772675 | 4 |
|  |  | RAFL05-14-F03 | At1g23310 / alanine aminotransferase -related | |  |  |  |  |  | | --- | --- | --- | --- | --- | |  |  |  |  |  | | EC:2.6.1.2  alanine transaminase | | | | | | |
|  |  | RAFL07-12-E03 | At1g23310 / alanine aminotransferase -related | |  |  |  |  |  | | --- | --- | --- | --- | --- | |  |  |  |  |  | | EC:2.6.1.2  alanine transaminase | | | | | | |
| serine-isocitrate lyase pathway | | |  |  | A | B | C | D | P | P' | N |
|  | Cluster:3-0 | |  |  | 7 | 226 | 24 | 4406 | 6.6646485E-4 | 0.011996367 | 18 |
|  |  | RAFL07-17-M04 | At1g04410 / malate dehydrogenase, cytosolic, putative | |  |  |  |  |  | | --- | --- | --- | --- | --- | |  |  |  |  |  | | EC:1.1.1.37  malate dehydrogenase | | | | | | |
|  |  | RAFL05-10-L06 | At2g13360 / alanine-glyoxylate aminotransferase | |  |  |  |  |  | | --- | --- | --- | --- | --- | |  |  |  |  |  | | EC:2.6.1.45  unknown | | | | | | |
|  |  | RAFL06-07-J21 | At1g04410 / malate dehydrogenase, cytosolic, putative | |  |  |  |  |  | | --- | --- | --- | --- | --- | |  |  |  |  |  | | EC:1.1.1.37  malate dehydrogenase | | | | | | |
|  |  | RAFL05-21-P13 | At2g42600 / phosphoenolpyruvate carboxylase | |  |  |  |  |  | | --- | --- | --- | --- | --- | |  |  |  |  |  | | EC:4.1.1.31  phosphoenolpyruvate carboxylase | | | | | | |
|  |  | RAFL09-09-M02 | At3g47520 / malate dehydrogenase (NAD), chloroplast, putative | |  |  |  |  |  | | --- | --- | --- | --- | --- | |  |  |  |  |  | | EC:1.1.1.37  malate dehydrogenase | | | | | | |
|  |  | RAFL05-18-J16 | At2g13360 / alanine-glyoxylate aminotransferase | |  |  |  |  |  | | --- | --- | --- | --- | --- | |  |  |  |  |  | | EC:2.6.1.45  unknown | | | | | | |
|  |  | RAFL05-02-A17 | At4g32520 / glycine hydroxymethyltransferase (EC 2.1.2.1) - like protein | |  |  |  |  |  | | --- | --- | --- | --- | --- | |  |  |  |  |  | | EC:2.1.2.1  glycine hydroxymethyltransferase | | | | | | |
| trehalose biosynthesis I | | |  |  | A | B | C | D | P | P' | N |
|  | Cluster:10-1 | |  |  | 1 | 42 | 3 | 4617 | 0.03639058 | 0.14556232 | 4 |
|  |  | RAFL05-13-B06 | At2g18700 / glycosyltransferase family 20 | |  |  |  |  |  | | --- | --- | --- | --- | --- | |  |  |  |  |  | | EC:2.4.1.15  alpha,alpha-trehalose-phosphate synthase (UDP-forming) | | | | | | |
| superpathway of serine and glycine biosynthesis II | | |  |  | A | B | C | D | P | P' | N |
|  | Cluster:1-2 | |  |  | 3 | 171 | 13 | 4476 | 0.019986069 | 0.17987463 | 9 |
|  |  | RAFL05-14-F03 | At1g23310 / alanine aminotransferase -related | |  |  |  |  |  | | --- | --- | --- | --- | --- | |  |  |  |  |  | | EC:2.6.1.4  alanine transaminase | | | | | | |
|  |  | RAFL09-11-K06 | At4g13930 / hydroxymethyltransferase | |  |  |  |  |  | | --- | --- | --- | --- | --- | |  |  |  |  |  | | EC:2.1.2.1  glycine hydroxymethyltransferase | | | | | | |
|  |  | RAFL07-12-E03 | At1g23310 / alanine aminotransferase -related | |  |  |  |  |  | | --- | --- | --- | --- | --- | |  |  |  |  |  | | EC:2.6.1.4  alanine transaminase | | | | | | |
|  | Cluster:3-0 | |  |  | 3 | 230 | 13 | 4417 | 0.04257905 | 0.38321143 | 9 |
|  |  | RAFL05-10-L06 | At2g13360 / alanine-glyoxylate aminotransferase | |  |  |  |  |  | | --- | --- | --- | --- | --- | |  |  |  |  |  | | EC:2.6.1.4  alanine transaminase | | | | | | |
|  |  | RAFL05-18-J16 | At2g13360 / alanine-glyoxylate aminotransferase | |  |  |  |  |  | | --- | --- | --- | --- | --- | |  |  |  |  |  | | EC:2.6.1.4  alanine transaminase | | | | | | |
|  |  | RAFL05-02-A17 | At4g32520 / glycine hydroxymethyltransferase (EC 2.1.2.1) - like protein | |  |  |  |  |  | | --- | --- | --- | --- | --- | |  |  |  |  |  | | EC:2.1.2.1  glycine hydroxymethyltransferase | | | | | | |
|  | Cluster:2-1 | |  |  | 3 | 241 | 13 | 4406 | 0.04780889 | 0.43028 | 9 |
|  |  | RAFL09-09-I19 | At1g23310 / alanine aminotransferase -related | |  |  |  |  |  | | --- | --- | --- | --- | --- | |  |  |  |  |  | | EC:2.6.1.4  alanine transaminase | | | | | | |
|  |  | RAFL09-16-K01 | At1g23310 / alanine aminotransferase -related | |  |  |  |  |  | | --- | --- | --- | --- | --- | |  |  |  |  |  | | EC:2.6.1.4  alanine transaminase | | | | | | |
|  |  | RAFL05-07-N11 | At1g70580 / alanine aminotransferase, putative | |  |  |  |  |  | | --- | --- | --- | --- | --- | |  |  |  |  |  | | EC:2.6.1.4  alanine transaminase | | | | | | |
| fatty acid oxidation pathway II | | |  |  | A | B | C | D | P | P' | N |
|  | Cluster:9-0 | |  |  | 4 | 28 | 2 | 4629 | 2.7153972E-8 | 8.146192E-8 | 3 |
|  |  | RAFL05-21-E06 | At1g54100 / aldehyde dehydrogenase, putative (ALDH) | |  |  |  |  |  | | --- | --- | --- | --- | --- | |  |  |  |  |  | | EC:1.2.1.3  unknown | | | | | | |
|  |  | RAFL04-09-D07 | At1g54100 / aldehyde dehydrogenase, putative (ALDH) | |  |  |  |  |  | | --- | --- | --- | --- | --- | |  |  |  |  |  | | EC:1.2.1.3  unknown | | | | | | |
|  |  | RAFL08-15-L09 | At1g54100 / aldehyde dehydrogenase, putative (ALDH) | |  |  |  |  |  | | --- | --- | --- | --- | --- | |  |  |  |  |  | | EC:1.2.1.3  unknown | | | | | | |
|  |  | RAFL08-09-C23 | At1g54100 / aldehyde dehydrogenase, putative (ALDH) | |  |  |  |  |  | | --- | --- | --- | --- | --- | |  |  |  |  |  | | EC:1.2.1.3  unknown | | | | | | |
| arginine degradation III | | |  |  | A | B | C | D | P | P' | N |
|  | Cluster:9-2 | |  |  | 2 | 65 | 8 | 4588 | 0.008496844 | 0.06797475 | 8 |
|  |  | RAFL09-13-D07 | At4g34710 / arginine decarboxylase SPE2 | |  |  |  |  |  | | --- | --- | --- | --- | --- | |  |  |  |  |  | | EC:4.1.1.19  arginine decarboxylase | | | | | | |
|  |  | RAFL08-11-N01 | At4g34710 / arginine decarboxylase SPE2 | |  |  |  |  |  | | --- | --- | --- | --- | --- | |  |  |  |  |  | | EC:4.1.1.19  arginine decarboxylase | | | | | | |
|  | Cluster:10-2 | |  |  | 2 | 107 | 8 | 4546 | 0.021556646 | 0.17245317 | 8 |
|  |  | RAFL08-15-A08 | At4g34710 / arginine decarboxylase SPE2 | |  |  |  |  |  | | --- | --- | --- | --- | --- | |  |  |  |  |  | | EC:4.1.1.19  arginine decarboxylase | | | | | | |
|  |  | RAFL04-13-O07 | At3g22200 / 4-aminobutyrate aminotransferase (gamma-amino-N-butyrate transaminase/GABA transaminase/beta-alanine--oxoglutarate aminotransferase) | |  |  |  |  |  | | --- | --- | --- | --- | --- | |  |  |  |  |  | | EC:2.6.1.19  4-aminobutyrate transaminase | | | | | | |
| tryptophan degradation III | | |  |  | A | B | C | D | P | P' | N |
|  | Cluster:3-1 | |  |  | 1 | 215 | 0 | 4447 | 0.04632211 | 0.04632211 | 1 |
|  |  | RAFL08-11-A03 | At5g48230 / acetyl-CoA C-acetyltransferase (acetoacetyl-coenzyme A thiolase), putative | |  |  |  |  |  | | --- | --- | --- | --- | --- | |  |  |  |  |  | | EC:2.3.1.9  acetyl-CoA C-acetyltransferase | | | | | | |
| lignin biosynthesis | | |  |  | A | B | C | D | P | P' | N |
|  | Cluster:9-0 | |  |  | 3 | 29 | 18 | 4613 | 3.591211E-4 | 0.004309453 | 12 |
|  |  | RAFL05-18-A06 | At1g09500 / cinnamyl-alcohol dehydrogenase (CAD) family | |  |  |  |  |  | | --- | --- | --- | --- | --- | |  |  |  |  |  | | EC:1.1.1.195  cinnamyl alcohol dehydrogenase | | | | | | |
|  |  | RAFL06-15-H16 | At1g09500 / cinnamyl-alcohol dehydrogenase (CAD) family | |  |  |  |  |  | | --- | --- | --- | --- | --- | |  |  |  |  |  | | EC:1.1.1.195  cinnamyl alcohol dehydrogenase | | | | | | |
|  |  | RAFL05-14-E15 | At2g33590 / cinnamoyl-CoA reductase family | |  |  |  |  |  | | --- | --- | --- | --- | --- | |  |  |  |  |  | | EC:1.2.1.44  cinnamoyl coenzyme A reductase | | | | | | |
|  | Cluster:6-2 | |  |  | 4 | 172 | 17 | 4470 | 0.0070927707 | 0.08511325 | 12 |
|  |  | RAFL09-11-L22 | At3g53260 / phenylalanine ammonia-lyase (PAL2) | |  |  |  |  |  | | --- | --- | --- | --- | --- | |  |  |  |  |  | | EC:4.3.1.5  phenylalanine ammonia-lyase | | | | | | |
|  |  | RAFL04-13-B02 | At2g37040 / phenylalanine ammonia lyase (PAL1) | |  |  |  |  |  | | --- | --- | --- | --- | --- | |  |  |  |  |  | | EC:4.3.1.5  phenylalanine ammonia-lyase | | | | | | |
|  |  | RAFL04-16-D08 | At3g53260 / phenylalanine ammonia-lyase (PAL2) | |  |  |  |  |  | | --- | --- | --- | --- | --- | |  |  |  |  |  | | EC:4.3.1.5  phenylalanine ammonia-lyase | | | | | | |
|  |  | RAFL04-17-C19 | At4g34050 / caffeoyl-CoA 3-O-methyltransferase | |  |  |  |  |  | | --- | --- | --- | --- | --- | |  |  |  |  |  | | EC:2.1.1.104  caffeoyl-CoA O-methyltransferase | | | | | | |
| polyamine biosynthesis I | | |  |  | A | B | C | D | P | P' | N |
|  | Cluster:9-2 | |  |  | 2 | 65 | 9 | 4587 | 0.010289272 | 0.08231418 | 8 |
|  |  | RAFL09-13-D07 | At4g34710 / arginine decarboxylase SPE2 | |  |  |  |  |  | | --- | --- | --- | --- | --- | |  |  |  |  |  | | EC:4.1.1.19  arginine decarboxylase | | | | | | |
|  |  | RAFL08-11-N01 | At4g34710 / arginine decarboxylase SPE2 | |  |  |  |  |  | | --- | --- | --- | --- | --- | |  |  |  |  |  | | EC:4.1.1.19  arginine decarboxylase | | | | | | |
|  | Cluster:10-2 | |  |  | 2 | 107 | 9 | 4545 | 0.025949622 | 0.20759697 | 8 |
|  |  | RAFL05-15-A16 | At5g53120 / spermidine synthase | |  |  |  |  |  | | --- | --- | --- | --- | --- | |  |  |  |  |  | | EC:2.5.1.16  spermidine synthase | | | | | | |
|  |  | RAFL08-15-A08 | At4g34710 / arginine decarboxylase SPE2 | |  |  |  |  |  | | --- | --- | --- | --- | --- | |  |  |  |  |  | | EC:4.1.1.19  arginine decarboxylase | | | | | | |
| tyrosine degradation | | |  |  | A | B | C | D | P | P' | N |
|  | Cluster:9-1 | |  |  | 2 | 94 | 10 | 4557 | 0.024206378 | 0.19365102 | 8 |
|  |  | RAFL11-09-O05 | At1g06570 / 4-hydroxyphenylpyruvate dioxygenase (HPD) | |  |  |  |  |  | | --- | --- | --- | --- | --- | |  |  |  |  |  | | EC:1.13.11.27  4-hydroxyphenylpyruvate dioxygenase | | | | | | |
|  |  | RAFL11-12-C18 | At1g06570 / 4-hydroxyphenylpyruvate dioxygenase (HPD) | |  |  |  |  |  | | --- | --- | --- | --- | --- | |  |  |  |  |  | | EC:1.13.11.27  4-hydroxyphenylpyruvate dioxygenase | | | | | | |
|  | Cluster:8-0 | |  |  | 2 | 107 | 10 | 4544 | 0.03067075 | 0.245366 | 8 |
|  |  | RAFL07-10-M07 | At5g53970 / aminotransferase, putative | |  |  |  |  |  | | --- | --- | --- | --- | --- | |  |  |  |  |  | | EC:2.6.1.5  tyrosine transaminase | | | | | | |
|  |  | RAFL05-09-B02 | At5g53970 / aminotransferase, putative | |  |  |  |  |  | | --- | --- | --- | --- | --- | |  |  |  |  |  | | EC:2.6.1.5  tyrosine transaminase | | | | | | |
|  | Cluster:5-1 | |  |  | 3 | 279 | 9 | 4372 | 0.03202454 | 0.25619632 | 8 |
|  |  | RAFL08-16-B22 | At1g11840 / glyoxalase I, putative (lactoylglutathione lyase) | |  |  |  |  |  | | --- | --- | --- | --- | --- | |  |  |  |  |  | | EC:1.13.11.27  4-hydroxyphenylpyruvate dioxygenase | | | | | | |
|  |  | RAFL09-07-G14 | At1g67280 / glyoxalase I, putative (lactoylglutathione lyase) | |  |  |  |  |  | | --- | --- | --- | --- | --- | |  |  |  |  |  | | EC:1.13.11.27  4-hydroxyphenylpyruvate dioxygenase | | | | | | |
|  |  | RAFL05-14-M18 | At1g12050 / fumarylacetoacetate hydrolase-related protein | |  |  |  |  |  | | --- | --- | --- | --- | --- | |  |  |  |  |  | | EC:3.7.1.2  fumarylacetoacetase | | | | | | |
| spermine biosynthesis I | | |  |  | A | B | C | D | P | P' | N |
|  | Cluster:9-2 | |  |  | 2 | 65 | 9 | 4587 | 0.010289272 | 0.08231418 | 8 |
|  |  | RAFL09-13-D07 | At4g34710 / arginine decarboxylase SPE2 | |  |  |  |  |  | | --- | --- | --- | --- | --- | |  |  |  |  |  | | EC:4.1.1.19  arginine decarboxylase | | | | | | |
|  |  | RAFL08-11-N01 | At4g34710 / arginine decarboxylase SPE2 | |  |  |  |  |  | | --- | --- | --- | --- | --- | |  |  |  |  |  | | EC:4.1.1.19  arginine decarboxylase | | | | | | |
|  | Cluster:10-2 | |  |  | 2 | 107 | 9 | 4545 | 0.025949622 | 0.20759697 | 8 |
|  |  | RAFL05-15-A16 | At5g53120 / spermidine synthase | |  |  |  |  |  | | --- | --- | --- | --- | --- | |  |  |  |  |  | | EC:2.5.1.16  spermidine synthase | | | | | | |
|  |  | RAFL08-15-A08 | At4g34710 / arginine decarboxylase SPE2 | |  |  |  |  |  | | --- | --- | --- | --- | --- | |  |  |  |  |  | | EC:4.1.1.19  arginine decarboxylase | | | | | | |
| acrylonitrile degradation | | |  |  | A | B | C | D | P | P' | N |
|  | Cluster:8-2 | |  |  | 1 | 61 | 2 | 4599 | 0.0393688 | 0.1181064 | 3 |
|  |  | RAFL02-08-C20 | At4g08790 / nitrilase 1 like protein | |  |  |  |  |  | | --- | --- | --- | --- | --- | |  |  |  |  |  | | EC:4.2.1.84  hydrolase, acting on carbon-nitrogen (but not peptide) bonds | | | | | | |
| jasmonic acid biosynthesis | | |  |  | A | B | C | D | P | P' | N |
|  | Cluster:2-2 | |  |  | 2 | 51 | 7 | 4603 | 0.0043364847 | 0.034691878 | 8 |
|  |  | RAFL05-12-G03 | At5g42650 / allene oxide synthase / cytochrome P450 74A | |  |  |  |  |  | | --- | --- | --- | --- | --- | |  |  |  |  |  | | EC:4.2.1.92  allene oxide synthase | | | | | | |
|  |  | RAFL06-10-H13 | At5g42650 / allene oxide synthase / cytochrome P450 74A | |  |  |  |  |  | | --- | --- | --- | --- | --- | |  |  |  |  |  | | EC:4.2.1.92  allene oxide synthase | | | | | | |
| dTDP-rhamnose biosynthesis | | |  |  | A | B | C | D | P | P' | N |
|  | Cluster:3-2 | |  |  | 2 | 35 | 7 | 4619 | 0.0021297743 | 0.012778646 | 6 |
|  |  | RAFL09-16-F08 | At3g23820 / NAD-dependent epimerase/dehydratase family | |  |  |  |  |  | | --- | --- | --- | --- | --- | |  |  |  |  |  | | EC:4.2.1.46  dTDP-glucose 4,6-dehydratase | | | | | | |
|  |  | RAFL09-07-D12 | At3g23820 / NAD-dependent epimerase/dehydratase family | |  |  |  |  |  | | --- | --- | --- | --- | --- | |  |  |  |  |  | | EC:4.2.1.46  dTDP-glucose 4,6-dehydratase | | | | | | |
|  | Cluster:5-2 | |  |  | 2 | 124 | 7 | 4530 | 0.023029873 | 0.13817924 | 6 |
|  |  | RAFL04-09-G05 | At1g50450 / expressed protein | |  |  |  |  |  | | --- | --- | --- | --- | --- | |  |  |  |  |  | | EC:4.2.1.46  dTDP-glucose 4,6-dehydratase | | | | | | |
|  |  | RAFL07-11-C21 | At4g30440 / nucleotide sugar epimerase family | |  |  |  |  |  | | --- | --- | --- | --- | --- | |  |  |  |  |  | | EC:4.2.1.46  dTDP-glucose 4,6-dehydratase | | | | | | |
| serine biosynthesis | | |  |  | A | B | C | D | P | P' | N |
|  | Cluster:3-1 | |  |  | 2 | 214 | 0 | 4447 | 0.0021362621 | 0.0021362621 | 1 |
|  |  | RAFL04-10-L08 | At4g34200 / D-3-phosphoglycerate dehydrogenase (3-PGDH), putative | |  |  |  |  |  | | --- | --- | --- | --- | --- | |  |  |  |  |  | | EC:1.1.1.95  phosphoglycerate dehydrogenase | | | | | | |
|  |  | RAFL04-18-D17 | At4g35630 / phosphoserine aminotransferase | |  |  |  |  |  | | --- | --- | --- | --- | --- | |  |  |  |  |  | | EC:2.6.1.52  triacylglycerol lipase | | | | | | |
| sucrose degradation III | | |  |  | A | B | C | D | P | P' | N |
|  | Cluster:9-0 | |  |  | 2 | 30 | 17 | 4614 | 0.0072546974 | 0.094311066 | 13 |
|  |  | RAFL05-18-M07 | At4g02280 / sucrose synthase (UDP-glucose-fructose glucosyltransferase/sucrose-UDP glucosyltransferase), putative | |  |  |  |  |  | | --- | --- | --- | --- | --- | |  |  |  |  |  | | EC:2.4.1.13  UDP-glycosyltransferase | | | | | | |
|  |  | RAFL08-10-G22 | At4g02280 / sucrose synthase (UDP-glucose-fructose glucosyltransferase/sucrose-UDP glucosyltransferase), putative | |  |  |  |  |  | | --- | --- | --- | --- | --- | |  |  |  |  |  | | EC:2.4.1.13  UDP-glycosyltransferase | | | | | | |
|  | Cluster:10-2 | |  |  | 3 | 106 | 16 | 4538 | 0.009166159 | 0.11916006 | 13 |
|  |  | RAFL05-07-J05 | At3g43190 / sucrose synthase (UDP-glucose-fructose glucosyltransferase/sucrose-UDP glucosyltransferase), putative | |  |  |  |  |  | | --- | --- | --- | --- | --- | |  |  |  |  |  | | EC:2.4.1.13  UDP-glycosyltransferase | | | | | | |
|  |  | RAFL08-15-K01 | At1g62660 / glycosyl hydrolase family 32 | |  |  |  |  |  | | --- | --- | --- | --- | --- | |  |  |  |  |  | | EC:3.2.1.26  unknown | | | | | | |
|  |  | RAFL08-13-K06 | At1g62660 / glycosyl hydrolase family 32 | |  |  |  |  |  | | --- | --- | --- | --- | --- | |  |  |  |  |  | | EC:3.2.1.26  unknown | | | | | | |
| asparagine degradation I | | |  |  | A | B | C | D | P | P' | N |
|  | Cluster:3-0 | |  |  | 3 | 230 | 3 | 4427 | 0.0022016333 | 0.0066048997 | 3 |
|  |  | RAFL05-21-P13 | At2g42600 / phosphoenolpyruvate carboxylase | |  |  |  |  |  | | --- | --- | --- | --- | --- | |  |  |  |  |  | | EC:4.1.1.32  phosphoenolpyruvate carboxylase | | | | | | |
|  |  | RAFL09-07-B08 | At2g30970 / aspartate aminotransferase, mitochondrial (transaminase A/Asp1) | |  |  |  |  |  | | --- | --- | --- | --- | --- | |  |  |  |  |  | | EC:2.6.1.1  1-aminocyclopropane-1-carboxylate synthase | | | | | | |
|  |  | RAFL04-20-H11 | At5g08100 / asparaginase | |  |  |  |  |  | | --- | --- | --- | --- | --- | |  |  |  |  |  | | EC:3.5.1.1  asparaginase | | | | | | |
| glutamine biosynthesis I | | |  |  | A | B | C | D | P | P' | N |
|  | Cluster:9-2 | |  |  | 2 | 65 | 2 | 4594 | 0.0011979077 | 0.0035937233 | 3 |
|  |  | RAFL08-17-D17 | At3g53180 / nodulin / glutamate-ammonia ligase - like protein | |  |  |  |  |  | | --- | --- | --- | --- | --- | |  |  |  |  |  | | EC:6.3.1.2  glutamate-ammonia ligase | | | | | | |
|  |  | RAFL06-09-F14 | At3g53180 / nodulin / glutamate-ammonia ligase - like protein | |  |  |  |  |  | | --- | --- | --- | --- | --- | |  |  |  |  |  | | EC:6.3.1.2  glutamate-ammonia ligase | | | | | | |
|  | Cluster:2-2 | |  |  | 1 | 52 | 3 | 4607 | 0.044709165 | 0.13412748 | 3 |
|  |  | RAFL04-16-N11 | At5g35630 / glutamate-ammonia ligase (EC 6.3.1.2) precursor, chloroplast (clone lambdaAtgsl1) (pir||S18600) | |  |  |  |  |  | | --- | --- | --- | --- | --- | |  |  |  |  |  | | EC:6.3.1.2  glutamate-ammonia ligase | | | | | | |
| arginine degradation V | | |  |  | A | B | C | D | P | P' | N |
|  | Cluster:9-2 | |  |  | 2 | 65 | 8 | 4588 | 0.008496844 | 0.06797475 | 8 |
|  |  | RAFL09-13-D07 | At4g34710 / arginine decarboxylase SPE2 | |  |  |  |  |  | | --- | --- | --- | --- | --- | |  |  |  |  |  | | EC:1.13.12.1  arginine decarboxylase | | | | | | |
|  |  | RAFL08-11-N01 | At4g34710 / arginine decarboxylase SPE2 | |  |  |  |  |  | | --- | --- | --- | --- | --- | |  |  |  |  |  | | EC:1.13.12.1  arginine decarboxylase | | | | | | |
|  | Cluster:10-2 | |  |  | 2 | 107 | 8 | 4546 | 0.021556646 | 0.17245317 | 8 |
|  |  | RAFL08-15-A08 | At4g34710 / arginine decarboxylase SPE2 | |  |  |  |  |  | | --- | --- | --- | --- | --- | |  |  |  |  |  | | EC:1.13.12.1  arginine decarboxylase | | | | | | |
|  |  | RAFL04-13-O07 | At3g22200 / 4-aminobutyrate aminotransferase (gamma-amino-N-butyrate transaminase/GABA transaminase/beta-alanine--oxoglutarate aminotransferase) | |  |  |  |  |  | | --- | --- | --- | --- | --- | |  |  |  |  |  | | EC:2.6.1.19  4-aminobutyrate transaminase//alanine-glyoxylate transaminase | | | | | | |
| threonine degradation | | |  |  | A | B | C | D | P | P' | N |
|  | Cluster:4-0 | |  |  | 3 | 237 | 7 | 4416 | 0.012340463 | 0.07404278 | 6 |
|  |  | RAFL04-17-J20 | At1g49670 / oxidoreductase, zinc-binding dehydrogenase family | |  |  |  |  |  | | --- | --- | --- | --- | --- | |  |  |  |  |  | | EC:1.1.1.103  L-threonine 3-dehydrogenase//alcohol dehydrogenase, zinc-dependent | | | | | | |
|  |  | RAFL05-19-C06 | At3g56460 / oxidoreductase, zinc-binding dehydrogenase family | |  |  |  |  |  | | --- | --- | --- | --- | --- | |  |  |  |  |  | | EC:1.1.1.103  L-threonine 3-dehydrogenase//alcohol dehydrogenase, zinc-dependent | | | | | | |
|  |  | RAFL04-18-H23 | At1g08110 / glyoxalase I, putative (lactoylglutathione lyase) | |  |  |  |  |  | | --- | --- | --- | --- | --- | |  |  |  |  |  | | EC:4.4.1.5  4-hydroxyphenylpyruvate dioxygenase | | | | | | |
|  | Cluster:8-0 | |  |  | 2 | 107 | 8 | 4546 | 0.021556646 | 0.12933987 | 6 |
|  |  | RAFL05-21-N06 | At4g13010 / oxidoreductase, zinc-binding dehydrogenase family | |  |  |  |  |  | | --- | --- | --- | --- | --- | |  |  |  |  |  | | EC:1.1.1.103  L-threonine 3-dehydrogenase//alcohol dehydrogenase, zinc-dependent | | | | | | |
|  |  | RAFL09-09-E18 | At2g32090 / glyoxalase family protein (lactoylglutathione lyase family protein) | |  |  |  |  |  | | --- | --- | --- | --- | --- | |  |  |  |  |  | | EC:4.4.1.5  4-hydroxyphenylpyruvate dioxygenase | | | | | | |
| 4-hydroxyproline degradation | | |  |  | A | B | C | D | P | P' | N |
|  | Cluster:9-0 | |  |  | 4 | 28 | 8 | 4623 | 8.7054286E-7 | 7.834886E-6 | 9 |
|  |  | RAFL05-21-E06 | At1g54100 / aldehyde dehydrogenase, putative (ALDH) | |  |  |  |  |  | | --- | --- | --- | --- | --- | |  |  |  |  |  | | EC:1.5.1.12  1-pyrroline-5-carboxylate dehydrogenase//aldehyde dehydrogenase | | | | | | |
|  |  | RAFL04-09-D07 | At1g54100 / aldehyde dehydrogenase, putative (ALDH) | |  |  |  |  |  | | --- | --- | --- | --- | --- | |  |  |  |  |  | | EC:1.5.1.12  1-pyrroline-5-carboxylate dehydrogenase//aldehyde dehydrogenase | | | | | | |
|  |  | RAFL08-15-L09 | At1g54100 / aldehyde dehydrogenase, putative (ALDH) | |  |  |  |  |  | | --- | --- | --- | --- | --- | |  |  |  |  |  | | EC:1.5.1.12  1-pyrroline-5-carboxylate dehydrogenase//aldehyde dehydrogenase | | | | | | |
|  |  | RAFL08-09-C23 | At1g54100 / aldehyde dehydrogenase, putative (ALDH) | |  |  |  |  |  | | --- | --- | --- | --- | --- | |  |  |  |  |  | | EC:1.5.1.12  1-pyrroline-5-carboxylate dehydrogenase//aldehyde dehydrogenase | | | | | | |
| glutamate degradation I | | |  |  | A | B | C | D | P | P' | N |
|  | Cluster:9-0 | |  |  | 1 | 31 | 5 | 4626 | 0.04049657 | 0.2429794 | 6 |
|  |  | RAFL05-08-B14 | At2g38400 / alanine--glyoxylate aminotransferase (beta-alanine-pyruvate aminotransferase/AGT), putative | |  |  |  |  |  | | --- | --- | --- | --- | --- | |  |  |  |  |  | | EC:2.6.1.19  4-aminobutyrate transaminase//alanine-glyoxylate transaminase | | | | | | |
| de novo biosynthesis of purine nucleotides II | | |  |  | A | B | C | D | P | P' | N |
|  | Cluster:3-0 | |  |  | 2 | 231 | 5 | 4425 | 0.044206142 | 0.2210307 | 5 |
|  |  | RAFL07-14-B18 | At2g47510 / fumarase -related | |  |  |  |  |  | | --- | --- | --- | --- | --- | |  |  |  |  |  | | EC:4.3.2.2  adenylosuccinate lyase | | | | | | |
|  |  | RAFL05-15-O22 | At2g20420 / succinyl-CoA ligase beta subunit | |  |  |  |  |  | | --- | --- | --- | --- | --- | |  |  |  |  |  | | EC:6.3.4.13  phosphoribosylamine-glycine ligase | | | | | | |
|  | Cluster:2-1 | |  |  | 2 | 242 | 5 | 4414 | 0.04810191 | 0.24050954 | 5 |
|  |  | RAFL04-17-H07 | At3g57610 / adenylosuccinate synthetase | |  |  |  |  |  | | --- | --- | --- | --- | --- | |  |  |  |  |  | | EC:6.3.4.4  adenylosuccinate synthase | | | | | | |
|  |  | RAFL04-12-O11 | At3g27740 / carbamoyl-phosphate synthase (glutamine-hydrolyzing) (glutamine-dependent carbamoyl-phosphate synthase) small subunit | |  |  |  |  |  | | --- | --- | --- | --- | --- | |  |  |  |  |  | | EC:6.3.5.2  GMP synthase (glutamine-hydrolyzing) | | | | | | |
| proline degradation I | | |  |  | A | B | C | D | P | P' | N |
|  | Cluster:9-0 | |  |  | 4 | 28 | 8 | 4623 | 8.7054286E-7 | 7.834886E-6 | 9 |
|  |  | RAFL05-21-E06 | At1g54100 / aldehyde dehydrogenase, putative (ALDH) | |  |  |  |  |  | | --- | --- | --- | --- | --- | |  |  |  |  |  | | EC:1.5.1.12  1-pyrroline-5-carboxylate dehydrogenase//aldehyde dehydrogenase | | | | | | |
|  |  | RAFL04-09-D07 | At1g54100 / aldehyde dehydrogenase, putative (ALDH) | |  |  |  |  |  | | --- | --- | --- | --- | --- | |  |  |  |  |  | | EC:1.5.1.12  1-pyrroline-5-carboxylate dehydrogenase//aldehyde dehydrogenase | | | | | | |
|  |  | RAFL08-15-L09 | At1g54100 / aldehyde dehydrogenase, putative (ALDH) | |  |  |  |  |  | | --- | --- | --- | --- | --- | |  |  |  |  |  | | EC:1.5.1.12  1-pyrroline-5-carboxylate dehydrogenase//aldehyde dehydrogenase | | | | | | |
|  |  | RAFL08-09-C23 | At1g54100 / aldehyde dehydrogenase, putative (ALDH) | |  |  |  |  |  | | --- | --- | --- | --- | --- | |  |  |  |  |  | | EC:1.5.1.12  1-pyrroline-5-carboxylate dehydrogenase//aldehyde dehydrogenase | | | | | | |
| arginine degradation VIII | | |  |  | A | B | C | D | P | P' | N |
|  | Cluster:4-1 | |  |  | 2 | 308 | 2 | 4351 | 0.024166461 | 0.07249938 | 3 |
|  |  | RAFL05-09-P07 | At4g29120 / expressed protein | |  |  |  |  |  | | --- | --- | --- | --- | --- | |  |  |  |  |  | | EC:1.5.1.2  disulfide oxidoreductase | | | | | | |
|  |  | RAFL06-14-H08 | At5g14800 / pyrroline-5-carboxylate reductase | |  |  |  |  |  | | --- | --- | --- | --- | --- | |  |  |  |  |  | | EC:1.5.1.2  disulfide oxidoreductase | | | | | | |
| histidine biosynthesis I | | |  |  | A | B | C | D | P | P' | N |
|  | Cluster:8-0 | |  |  | 2 | 107 | 6 | 4548 | 0.013828159 | 0.096797116 | 7 |
|  |  | RAFL07-10-M07 | At5g53970 / aminotransferase, putative | |  |  |  |  |  | | --- | --- | --- | --- | --- | |  |  |  |  |  | | EC:2.6.1.9  histidinol-phosphate transaminase | | | | | | |
|  |  | RAFL05-09-B02 | At5g53970 / aminotransferase, putative | |  |  |  |  |  | | --- | --- | --- | --- | --- | |  |  |  |  |  | | EC:2.6.1.9  histidinol-phosphate transaminase | | | | | | |
| glycosylglyceride biosynthesis | | |  |  | A | B | C | D | P | P' | N |
|  | Cluster:8-2 | |  |  | 1 | 61 | 2 | 4599 | 0.0393688 | 0.1181064 | 3 |
|  |  | RAFL04-20-J18 | At4g31780 / 1,2-diacylglycerol 3-beta-galactosyltransferase (UDP-galactose:diacylglycerol galactosyltransferase) (MGDG synthase) (MGD1), putative | |  |  |  |  |  | | --- | --- | --- | --- | --- | |  |  |  |  |  | | EC:2.4.1.46  UDP-galactose:DAG galactosyltransferase | | | | | | |
| polyamine biosynthesis III | | |  |  | A | B | C | D | P | P' | N |
|  | Cluster:9-2 | |  |  | 2 | 65 | 8 | 4588 | 0.008496844 | 0.059477907 | 7 |
|  |  | RAFL09-13-D07 | At4g34710 / arginine decarboxylase SPE2 | |  |  |  |  |  | | --- | --- | --- | --- | --- | |  |  |  |  |  | | EC:4.1.1.19  arginine decarboxylase | | | | | | |
|  |  | RAFL08-11-N01 | At4g34710 / arginine decarboxylase SPE2 | |  |  |  |  |  | | --- | --- | --- | --- | --- | |  |  |  |  |  | | EC:4.1.1.19  arginine decarboxylase | | | | | | |
|  | Cluster:10-2 | |  |  | 2 | 107 | 8 | 4546 | 0.021556646 | 0.15089652 | 7 |
|  |  | RAFL05-15-A16 | At5g53120 / spermidine synthase | |  |  |  |  |  | | --- | --- | --- | --- | --- | |  |  |  |  |  | | EC:2.5.1.16  spermidine synthase | | | | | | |
|  |  | RAFL08-15-A08 | At4g34710 / arginine decarboxylase SPE2 | |  |  |  |  |  | | --- | --- | --- | --- | --- | |  |  |  |  |  | | EC:4.1.1.19  arginine decarboxylase | | | | | | |
|  | Cluster:8-1 | |  |  | 2 | 160 | 8 | 4493 | 0.044944704 | 0.31461293 | 7 |
|  |  | RAFL04-17-I02 | At2g16500 / arginine decarboxylase | |  |  |  |  |  | | --- | --- | --- | --- | --- | |  |  |  |  |  | | EC:4.1.1.19  arginine decarboxylase | | | | | | |
|  |  | RAFL05-09-J16 | At5g53120 / spermidine synthase | |  |  |  |  |  | | --- | --- | --- | --- | --- | |  |  |  |  |  | | EC:2.5.1.16  spermidine synthase | | | | | | |
| phenylalanine biosynthesis II | | |  |  | A | B | C | D | P | P' | N |
|  | Cluster:8-0 | |  |  | 2 | 107 | 4 | 4550 | 0.007638084 | 0.038190417 | 5 |
|  |  | RAFL07-10-M07 | At5g53970 / aminotransferase, putative | |  |  |  |  |  | | --- | --- | --- | --- | --- | |  |  |  |  |  | | EC:2.6.1.5 ,EC:2.6.1.9  1-aminocyclopropane-1-carboxylate synthase  phosphoglycerate dehydrogenase | | | | | | |
|  |  | RAFL05-09-B02 | At5g53970 / aminotransferase, putative | |  |  |  |  |  | | --- | --- | --- | --- | --- | |  |  |  |  |  | | EC:2.6.1.5 ,EC:2.6.1.9  1-aminocyclopropane-1-carboxylate synthase  phosphoglycerate dehydrogenase | | | | | | |
| glyoxylate cycle | | |  |  | A | B | C | D | P | P' | N |
|  | Cluster:3-0 | |  |  | 3 | 230 | 11 | 4419 | 0.029778728 | 0.327566 | 11 |
|  |  | RAFL07-17-M04 | At1g04410 / malate dehydrogenase, cytosolic, putative | |  |  |  |  |  | | --- | --- | --- | --- | --- | |  |  |  |  |  | | EC:1.1.1.37  malate dehydrogenase | | | | | | |
|  |  | RAFL06-07-J21 | At1g04410 / malate dehydrogenase, cytosolic, putative | |  |  |  |  |  | | --- | --- | --- | --- | --- | |  |  |  |  |  | | EC:1.1.1.37  malate dehydrogenase | | | | | | |
|  |  | RAFL09-09-M02 | At3g47520 / malate dehydrogenase (NAD), chloroplast, putative | |  |  |  |  |  | | --- | --- | --- | --- | --- | |  |  |  |  |  | | EC:1.1.1.37  malate dehydrogenase | | | | | | |
| polyamine biosynthesis II | | |  |  | A | B | C | D | P | P' | N |
|  | Cluster:9-2 | |  |  | 2 | 65 | 8 | 4588 | 0.008496844 | 0.059477907 | 7 |
|  |  | RAFL09-13-D07 | At4g34710 / arginine decarboxylase SPE2 | |  |  |  |  |  | | --- | --- | --- | --- | --- | |  |  |  |  |  | | EC:4.1.1.19  arginine decarboxylase | | | | | | |
|  |  | RAFL08-11-N01 | At4g34710 / arginine decarboxylase SPE2 | |  |  |  |  |  | | --- | --- | --- | --- | --- | |  |  |  |  |  | | EC:4.1.1.19  arginine decarboxylase | | | | | | |
|  | Cluster:10-2 | |  |  | 2 | 107 | 8 | 4546 | 0.021556646 | 0.15089652 | 7 |
|  |  | RAFL05-15-A16 | At5g53120 / spermidine synthase | |  |  |  |  |  | | --- | --- | --- | --- | --- | |  |  |  |  |  | | EC:2.5.1.16  spermidine synthase | | | | | | |
|  |  | RAFL08-15-A08 | At4g34710 / arginine decarboxylase SPE2 | |  |  |  |  |  | | --- | --- | --- | --- | --- | |  |  |  |  |  | | EC:4.1.1.19  arginine decarboxylase | | | | | | |
|  | Cluster:8-1 | |  |  | 2 | 160 | 8 | 4493 | 0.044944704 | 0.31461293 | 7 |
|  |  | RAFL04-17-I02 | At2g16500 / arginine decarboxylase | |  |  |  |  |  | | --- | --- | --- | --- | --- | |  |  |  |  |  | | EC:4.1.1.19  arginine decarboxylase | | | | | | |
|  |  | RAFL05-09-J16 | At5g53120 / spermidine synthase | |  |  |  |  |  | | --- | --- | --- | --- | --- | |  |  |  |  |  | | EC:2.5.1.16  spermidine synthase | | | | | | |
| pyridoxal 5'-phosphate salvage pathway | | |  |  | A | B | C | D | P | P' | N |
|  | Cluster:4-2 | |  |  | 1 | 134 | 0 | 4528 | 0.028951319 | 0.028951319 | 1 |
|  |  | RAFL05-16-L10 | At5g49970 / expressed protein | |  |  |  |  |  | | --- | --- | --- | --- | --- | |  |  |  |  |  | | EC:1.4.3.5  pyridoxamine-phosphate oxidase | | | | | | |
| homocysteine and cysteine interconversion | | |  |  | A | B | C | D | P | P' | N |
|  | Cluster:9-1 | |  |  | 2 | 94 | 0 | 4567 | 4.1952432E-4 | 4.1952432E-4 | 1 |
|  |  | RAFL05-18-H15 | At1g64660 / methionine/cystathionine gamma lyase -related | |  |  |  |  |  | | --- | --- | --- | --- | --- | |  |  |  |  |  | | EC:4.4.1.8  cystathionine beta-lyase | | | | | | |
|  |  | RAFL11-02-N11 | At1g64660 / methionine/cystathionine gamma lyase -related | |  |  |  |  |  | | --- | --- | --- | --- | --- | |  |  |  |  |  | | EC:4.4.1.8  cystathionine beta-lyase | | | | | | |
| methylglyoxal degradation | | |  |  | A | B | C | D | P | P' | N |
|  | Cluster:5-1 | |  |  | 2 | 280 | 4 | 4377 | 0.046489924 | 0.23244964 | 5 |
|  |  | RAFL08-16-B22 | At1g11840 / glyoxalase I, putative (lactoylglutathione lyase) | |  |  |  |  |  | | --- | --- | --- | --- | --- | |  |  |  |  |  | | EC:4.4.1.5  4-hydroxyphenylpyruvate dioxygenase | | | | | | |
|  |  | RAFL09-07-G14 | At1g67280 / glyoxalase I, putative (lactoylglutathione lyase) | |  |  |  |  |  | | --- | --- | --- | --- | --- | |  |  |  |  |  | | EC:4.4.1.5  4-hydroxyphenylpyruvate dioxygenase | | | | | | |
| lactate oxidation | | |  |  | A | B | C | D | P | P' | N |
|  | Cluster:4-1 | |  |  | 5 | 305 | 9 | 4344 | 0.001527034 | 0.012216272 | 8 |
|  |  | RAFL07-16-E16 | At3g13930 / acetyltransferase -related | |  |  |  |  |  | | --- | --- | --- | --- | --- | |  |  |  |  |  | | EC:2.3.1.12  unknown | | | | | | |
|  |  | RAFL06-08-D06 | At2g34590 / pyruvate dehydrogenase E1 beta subunit -related | |  |  |  |  |  | | --- | --- | --- | --- | --- | |  |  |  |  |  | | EC:1.2.4.1  pyruvate decarboxylase | | | | | | |
|  |  | RAFL09-12-A19 | At3g13930 / acetyltransferase -related | |  |  |  |  |  | | --- | --- | --- | --- | --- | |  |  |  |  |  | | EC:2.3.1.12  unknown | | | | | | |
|  |  | RAFL09-11-F09 | At1g30120 / pyruvate dehydrogenase E1 beta subunit -related | |  |  |  |  |  | | --- | --- | --- | --- | --- | |  |  |  |  |  | | EC:1.2.4.1  pyruvate decarboxylase | | | | | | |
|  |  | RAFL09-16-O16 | At1g01090 / pyruvate dehydrogenase E1 alpha subunit | |  |  |  |  |  | | --- | --- | --- | --- | --- | |  |  |  |  |  | | EC:1.2.4.1  pyruvate decarboxylase | | | | | | |
| biosynthesis of proto- and siroheme | | |  |  | A | B | C | D | P | P' | N |
|  | Cluster:3-2 | |  |  | 4 | 33 | 11 | 4615 | 4.3046944E-6 | 3.4437555E-5 | 8 |
|  |  | RAFL09-06-N12 | At3g23810 / S-adenosyl-L-homocysteinas -related | |  |  |  |  |  | | --- | --- | --- | --- | --- | |  |  |  |  |  | | EC:4.3.1.8  adenosylhomocysteinase | | | | | | |
|  |  | RAFL07-09-L01 | At3g23810 / S-adenosyl-L-homocysteinas -related | |  |  |  |  |  | | --- | --- | --- | --- | --- | |  |  |  |  |  | | EC:4.3.1.8  adenosylhomocysteinase | | | | | | |
|  |  | RAFL09-13-P13 | At3g23810 / S-adenosyl-L-homocysteinas -related | |  |  |  |  |  | | --- | --- | --- | --- | --- | |  |  |  |  |  | | EC:4.3.1.8  adenosylhomocysteinase | | | | | | |
|  |  | RAFL09-10-M18 | At3g23810 / S-adenosyl-L-homocysteinas -related | |  |  |  |  |  | | --- | --- | --- | --- | --- | |  |  |  |  |  | | EC:4.3.1.8  adenosylhomocysteinase | | | | | | |
|  | Cluster:2-1 | |  |  | 3 | 241 | 12 | 4407 | 0.040360164 | 0.3228813 | 8 |
|  |  | RAFL04-17-P12 | At4g13940 / adenosylhomocysteinase | |  |  |  |  |  | | --- | --- | --- | --- | --- | |  |  |  |  |  | | EC:4.3.1.8  adenosylhomocysteinase | | | | | | |
|  |  | RAFL05-10-J03 | At1g69740 / porphobilinogen synthase (delta-aminolevulinic acid dehydratase), putative | |  |  |  |  |  | | --- | --- | --- | --- | --- | |  |  |  |  |  | | EC:4.2.1.24  porphobilinogen synthase | | | | | | |
|  |  | RAFL04-15-N01 | At1g69740 / porphobilinogen synthase (delta-aminolevulinic acid dehydratase), putative | |  |  |  |  |  | | --- | --- | --- | --- | --- | |  |  |  |  |  | | EC:4.2.1.24  porphobilinogen synthase | | | | | | |
| leucine degradation I | | |  |  | A | B | C | D | P | P' | N |
|  | Cluster:10-2 | |  |  | 1 | 108 | 1 | 4553 | 0.0462095 | 0.092419 | 2 |
|  |  | RAFL06-10-M04 | At2g26800 / hydroxymethylglutaryl-CoA lyase -related | |  |  |  |  |  | | --- | --- | --- | --- | --- | |  |  |  |  |  | | EC:4.1.3.4  hydroxymethylglutaryl-CoA lyase | | | | | | |
| glycerol degradation II | | |  |  | A | B | C | D | P | P' | N |
|  | Cluster:0-1 | |  |  | 8 | 98 | 24 | 4533 | 3.6590941E-7 | 5.4886414E-6 | 15 |
|  |  | RAFL09-15-L04 | At3g12780 / phosphoglycerate kinase -related | |  |  |  |  |  | | --- | --- | --- | --- | --- | |  |  |  |  |  | | EC:2.7.2.3  phosphoglycerate kinase | | | | | | |
|  |  | RAFL07-14-L16 | At3g12780 / phosphoglycerate kinase -related | |  |  |  |  |  | | --- | --- | --- | --- | --- | |  |  |  |  |  | | EC:2.7.2.3  phosphoglycerate kinase | | | | | | |
|  |  | RAFL07-07-I23 | At1g56190 / phosphoglycerate kinase -related | |  |  |  |  |  | | --- | --- | --- | --- | --- | |  |  |  |  |  | | EC:2.7.2.3  phosphoglycerate kinase | | | | | | |
|  |  | RAFL04-09-D24 | At1g42970 / glyceraldehyde-3-phosphate dehydrogenase | |  |  |  |  |  | | --- | --- | --- | --- | --- | |  |  |  |  |  | | EC:1.2.1.12  glyceraldehyde-3-phosphate dehydrogenase | | | | | | |
|  |  | RAFL07-16-P05 | At3g12780 / phosphoglycerate kinase -related | |  |  |  |  |  | | --- | --- | --- | --- | --- | |  |  |  |  |  | | EC:2.7.2.3  phosphoglycerate kinase | | | | | | |
|  |  | RAFL09-18-L22 | At3g12780 / phosphoglycerate kinase -related | |  |  |  |  |  | | --- | --- | --- | --- | --- | |  |  |  |  |  | | EC:2.7.2.3  phosphoglycerate kinase | | | | | | |
|  |  | RAFL04-15-A14 | At1g12900 / calcium-binding protein, calreticulin -related | |  |  |  |  |  | | --- | --- | --- | --- | --- | |  |  |  |  |  | | EC:1.2.1.12  glyceraldehyde-3-phosphate dehydrogenase | | | | | | |
|  |  | RAFL05-07-J06 | At1g42970 / glyceraldehyde-3-phosphate dehydrogenase | |  |  |  |  |  | | --- | --- | --- | --- | --- | |  |  |  |  |  | | EC:1.2.1.12  glyceraldehyde-3-phosphate dehydrogenase | | | | | | |
|  | Cluster:3-1 | |  |  | 6 | 210 | 26 | 4421 | 0.0030328825 | 0.045493238 | 15 |
|  |  | RAFL07-10-P13 | At1g09780 / 2,3-bisphosphoglycerate-independent phosphoglycerate mutase -related | |  |  |  |  |  | | --- | --- | --- | --- | --- | |  |  |  |  |  | | EC:5.4.2.1  2,3-bisphosphoglycerate-independent phosphoglycerate mutase | | | | | | |
|  |  | RAFL05-01-I24 | At3g55440 / triosephosphate isomerase, cytosolic, putative | |  |  |  |  |  | | --- | --- | --- | --- | --- | |  |  |  |  |  | | EC:5.3.1.1  triose-phosphate isomerase | | | | | | |
|  |  | RAFL05-21-G03 | At3g22960 / pyruvate kinase, putative | |  |  |  |  |  | | --- | --- | --- | --- | --- | |  |  |  |  |  | | EC:2.7.1.40  pyruvate kinase | | | | | | |
|  |  | RAFL04-13-O10 | At2g29560 / enolase (2-phospho-D-glycerate hydroylase) -related | |  |  |  |  |  | | --- | --- | --- | --- | --- | |  |  |  |  |  | | EC:4.2.1.11  phosphopyruvate hydratase | | | | | | |
|  |  | RAFL09-12-D13 | At5g52920 / pyruvate kinase, putative | |  |  |  |  |  | | --- | --- | --- | --- | --- | |  |  |  |  |  | | EC:2.7.1.40  pyruvate kinase | | | | | | |
|  |  | RAFL07-09-L18 | At2g40690 / glycerol-3-phosphate dehydrogenase | |  |  |  |  |  | | --- | --- | --- | --- | --- | |  |  |  |  |  | | EC:1.1.1.8  glycerol-3-phosphate dehydrogenase (NAD+) | | | | | | |
| TCA cycle variation VIII | | |  |  | A | B | C | D | P | P' | N |
|  | Cluster:3-0 | |  |  | 5 | 228 | 23 | 4407 | 0.011410674 | 0.19398145 | 17 |
|  |  | RAFL07-17-M04 | At1g04410 / malate dehydrogenase, cytosolic, putative | |  |  |  |  |  | | --- | --- | --- | --- | --- | |  |  |  |  |  | | EC:1.1.1.37  malate dehydrogenase | | | | | | |
|  |  | RAFL06-07-J21 | At1g04410 / malate dehydrogenase, cytosolic, putative | |  |  |  |  |  | | --- | --- | --- | --- | --- | |  |  |  |  |  | | EC:1.1.1.37  malate dehydrogenase | | | | | | |
|  |  | RAFL05-21-P13 | At2g42600 / phosphoenolpyruvate carboxylase | |  |  |  |  |  | | --- | --- | --- | --- | --- | |  |  |  |  |  | | EC:4.1.1.32  phosphoenolpyruvate carboxykinase | | | | | | |
|  |  | RAFL09-09-M02 | At3g47520 / malate dehydrogenase (NAD), chloroplast, putative | |  |  |  |  |  | | --- | --- | --- | --- | --- | |  |  |  |  |  | | EC:1.1.1.37  malate dehydrogenase | | | | | | |
|  |  | RAFL07-14-B18 | At2g47510 / fumarase -related | |  |  |  |  |  | | --- | --- | --- | --- | --- | |  |  |  |  |  | | EC:4.2.1.2  adenylosuccinate lyase | | | | | | |
| galactose degradation I | | |  |  | A | B | C | D | P | P' | N |
|  | Cluster:3-2 | |  |  | 2 | 35 | 17 | 4609 | 0.009623891 | 0.13473447 | 14 |
|  |  | RAFL09-16-F08 | At3g23820 / NAD-dependent epimerase/dehydratase family | |  |  |  |  |  | | --- | --- | --- | --- | --- | |  |  |  |  |  | | EC:5.1.3.2  UDP-glucose 4-epimerase | | | | | | |
|  |  | RAFL09-07-D12 | At3g23820 / NAD-dependent epimerase/dehydratase family | |  |  |  |  |  | | --- | --- | --- | --- | --- | |  |  |  |  |  | | EC:5.1.3.2  UDP-glucose 4-epimerase | | | | | | |
| UDP-N-acetylglucosamine biosynthesis | | |  |  | A | B | C | D | P | P' | N |
|  | Cluster:1-1 | |  |  | 2 | 103 | 2 | 4556 | 0.0029258758 | 0.008777628 | 3 |
|  |  | RAFL09-13-M20 | At1g31070 / UDP-N-acetylglucosamine pyrophosphorylase-related protein | |  |  |  |  |  | | --- | --- | --- | --- | --- | |  |  |  |  |  | | EC:2.7.7.23  UDP-N-acetylglucosamine diphosphorylase | | | | | | |
|  |  | RAFL09-18-H10 | At5g19220 / glucose-1-phosphate adenylyltransferase, large subunit 1, chloroplast (ADP-glucose pyrophosphorylase) (ADG2) (APL1) | |  |  |  |  |  | | --- | --- | --- | --- | --- | |  |  |  |  |  | | EC:2.7.7.23  UDP-N-acetylglucosamine diphosphorylase | | | | | | |
| cyclopropane fatty acid (CFA) biosynthesis | | |  |  | A | B | C | D | P | P' | N |
|  | Cluster:2-1 | |  |  | 2 | 242 | 1 | 4418 | 0.00789916 | 0.01579832 | 2 |
|  |  | RAFL07-08-E22 | At3g23530 / cyclopropane synthase, putative | |  |  |  |  |  | | --- | --- | --- | --- | --- | |  |  |  |  |  | | EC:2.1.1.79  amine oxidase | | | | | | |
|  |  | RAFL07-18-J07 | At3g23530 / cyclopropane synthase, putative | |  |  |  |  |  | | --- | --- | --- | --- | --- | |  |  |  |  |  | | EC:2.1.1.79  amine oxidase | | | | | | |
| arginine biosynthesis I | | |  |  | A | B | C | D | P | P' | N |
|  | Cluster:3-1 | |  |  | 4 | 212 | 2 | 4445 | 6.244364E-5 | 1.8733094E-4 | 3 |
|  |  | RAFL07-16-F16 | At3g20330 / aspartate carbamoyltransferase precursor (aspartate transcarbamylase) | |  |  |  |  |  | | --- | --- | --- | --- | --- | |  |  |  |  |  | | EC:2.1.3.3  ornithine carbamoyltransferase | | | | | | |
|  |  | RAFL07-18-A10 | At2g37500 / glutamate/ornithine acetyltransferase -related | |  |  |  |  |  | | --- | --- | --- | --- | --- | |  |  |  |  |  | | EC:2.3.1.35 ,EC:2.3.1.1  glutamate N-acetyltransferase  acetylglutamate kinase | | | | | | |
|  |  | RAFL07-08-L02 | At5g10920 / argininosuccinate lyase (AtArgH) | |  |  |  |  |  | | --- | --- | --- | --- | --- | |  |  |  |  |  | | EC:4.3.2.1  adenylosuccinate lyase | | | | | | |
|  |  | RAFL04-16-G24 | At1g80600 / acetylornithine aminotransferase, mitochondrial (acetylornithine transaminase/AOTA/ACOAT), putative | |  |  |  |  |  | | --- | --- | --- | --- | --- | |  |  |  |  |  | | EC:2.6.1.11  acetylornithine transaminase | | | | | | |
| peptidoglycan biosynthesis | | |  |  | A | B | C | D | P | P' | N |
|  | Cluster:1-1 | |  |  | 2 | 103 | 1 | 4557 | 0.0014847745 | 0.002969549 | 2 |
|  |  | RAFL09-13-M20 | At1g31070 / UDP-N-acetylglucosamine pyrophosphorylase-related protein | |  |  |  |  |  | | --- | --- | --- | --- | --- | |  |  |  |  |  | | EC:2.7.7.23  UDP-N-acetylglucosamine diphosphorylase | | | | | | |
|  |  | RAFL09-18-H10 | At5g19220 / glucose-1-phosphate adenylyltransferase, large subunit 1, chloroplast (ADP-glucose pyrophosphorylase) (ADG2) (APL1) | |  |  |  |  |  | | --- | --- | --- | --- | --- | |  |  |  |  |  | | EC:2.7.7.23  UDP-N-acetylglucosamine diphosphorylase | | | | | | |
| ammonia assimilation cycle | | |  |  | A | B | C | D | P | P' | N |
|  | Cluster:9-2 | |  |  | 2 | 65 | 2 | 4594 | 0.0011979077 | 0.0035937233 | 3 |
|  |  | RAFL08-17-D17 | At3g53180 / nodulin / glutamate-ammonia ligase - like protein | |  |  |  |  |  | | --- | --- | --- | --- | --- | |  |  |  |  |  | | EC:6.3.1.2  glutamate-ammonia ligase | | | | | | |
|  |  | RAFL06-09-F14 | At3g53180 / nodulin / glutamate-ammonia ligase - like protein | |  |  |  |  |  | | --- | --- | --- | --- | --- | |  |  |  |  |  | | EC:6.3.1.2  glutamate-ammonia ligase | | | | | | |
|  | Cluster:2-2 | |  |  | 1 | 52 | 3 | 4607 | 0.044709165 | 0.13412748 | 3 |
|  |  | RAFL04-16-N11 | At5g35630 / glutamate-ammonia ligase (EC 6.3.1.2) precursor, chloroplast (clone lambdaAtgsl1) (pir||S18600) | |  |  |  |  |  | | --- | --- | --- | --- | --- | |  |  |  |  |  | | EC:6.3.1.2  glutamate-ammonia ligase | | | | | | |
| homogalacturonan degradation | | |  |  | A | B | C | D | P | P' | N |
|  | Cluster:2-2 | |  |  | 1 | 52 | 1 | 4609 | 0.022605369 | 0.045210738 | 2 |
|  |  | RAFL06-10-O06 | At1g53840 / pectinesterase family | |  |  |  |  |  | | --- | --- | --- | --- | --- | |  |  |  |  |  | | EC:3.1.1.11  pectin methylesterase | | | | | | |
|  | Cluster:0-1 | |  |  | 1 | 105 | 1 | 4556 | 0.044952307 | 0.089904614 | 2 |
|  |  | RAFL09-12-M04 | At3g14310 / pectin methylesterase -related | |  |  |  |  |  | | --- | --- | --- | --- | --- | |  |  |  |  |  | | EC:3.1.1.11  pectin methylesterase | | | | | | |
| sucrose biosynthesis | | |  |  | A | B | C | D | P | P' | N |
|  | Cluster:9-0 | |  |  | 2 | 30 | 10 | 4621 | 0.0028851912 | 0.02596672 | 9 |
|  |  | RAFL05-18-M07 | At4g02280 / sucrose synthase (UDP-glucose-fructose glucosyltransferase/sucrose-UDP glucosyltransferase), putative | |  |  |  |  |  | | --- | --- | --- | --- | --- | |  |  |  |  |  | | EC:2.4.1.13  UDP-glycosyltransferase | | | | | | |
|  |  | RAFL08-10-G22 | At4g02280 / sucrose synthase (UDP-glucose-fructose glucosyltransferase/sucrose-UDP glucosyltransferase), putative | |  |  |  |  |  | | --- | --- | --- | --- | --- | |  |  |  |  |  | | EC:2.4.1.13  UDP-glycosyltransferase | | | | | | |
|  | Cluster:4-1 | |  |  | 3 | 307 | 9 | 4344 | 0.040857635 | 0.36771873 | 9 |
|  |  | RAFL07-13-K21 | At2g35840 / expressed protein | |  |  |  |  |  | | --- | --- | --- | --- | --- | |  |  |  |  |  | | EC:3.1.3.24  sucrose-phosphatase | | | | | | |
|  |  | RAFL09-15-M21 | At5g17310 / UDP-glucose pyrophosphorylase | |  |  |  |  |  | | --- | --- | --- | --- | --- | |  |  |  |  |  | | EC:2.7.7.9  UDP-N-acetylglucosamine diphosphorylase | | | | | | |
|  |  | RAFL09-12-G22 | At5g17310 / UDP-glucose pyrophosphorylase | |  |  |  |  |  | | --- | --- | --- | --- | --- | |  |  |  |  |  | | EC:2.7.7.9  UDP-N-acetylglucosamine diphosphorylase | | | | | | |
| UDP-glucose conversion | | |  |  | A | B | C | D | P | P' | N |
|  | Cluster:3-0 | |  |  | 5 | 228 | 21 | 4409 | 0.0082831625 | 0.1325306 | 16 |
|  |  | RAFL07-11-P20 | At4g01480 / inorganic phosphatase -related | |  |  |  |  |  | | --- | --- | --- | --- | --- | |  |  |  |  |  | | EC:3.6.1.1  unknown | | | | | | |
|  |  | RAFL05-16-O24 | At4g35250 / vestitone reductase-related | |  |  |  |  |  | | --- | --- | --- | --- | --- | |  |  |  |  |  | | EC:5.1.3.2  UDP-glucose 4-epimerase | | | | | | |
|  |  | RAFL09-15-M18 | At3g03250 / UDP-glucose pyrophosphorylase -related | |  |  |  |  |  | | --- | --- | --- | --- | --- | |  |  |  |  |  | | EC:2.7.7.9  UDP-N-acetylglucosamine diphosphorylase | | | | | | |
|  |  | RAFL07-18-D08 | At1g15690 / inorganic pyrophosphatase -related | |  |  |  |  |  | | --- | --- | --- | --- | --- | |  |  |  |  |  | | EC:3.6.1.1  unknown | | | | | | |
|  |  | RAFL05-18-A12 | At1g15690 / inorganic pyrophosphatase -related | |  |  |  |  |  | | --- | --- | --- | --- | --- | |  |  |  |  |  | | EC:3.6.1.1  unknown | | | | | | |
|  | Cluster:3-2 | |  |  | 2 | 35 | 24 | 4602 | 0.017665556 | 0.2826489 | 16 |
|  |  | RAFL09-16-F08 | At3g23820 / NAD-dependent epimerase/dehydratase family | |  |  |  |  |  | | --- | --- | --- | --- | --- | |  |  |  |  |  | | EC:5.1.3.2  UDP-glucose 4-epimerase | | | | | | |
|  |  | RAFL09-07-D12 | At3g23820 / NAD-dependent epimerase/dehydratase family | |  |  |  |  |  | | --- | --- | --- | --- | --- | |  |  |  |  |  | | EC:5.1.3.2  UDP-glucose 4-epimerase | | | | | | |
| fatty acid elongation -- unsaturated | | |  |  | A | B | C | D | P | P' | N |
|  | Cluster:6-2 | |  |  | 2 | 174 | 6 | 4481 | 0.034143474 | 0.23900433 | 7 |
|  |  | RAFL05-10-D10 | At5g43760 / beta-ketoacyl-CoA synthase, putative | |  |  |  |  |  | | --- | --- | --- | --- | --- | |  |  |  |  |  | | EC:2.3.1.41  3-oxoacyl-[acyl-carrier protein] synthase | | | | | | |
|  |  | RAFL02-03-L07 | At2g26250 / beta-ketoacyl-CoA synthase family (FIDDLEHEAD) (FDH) | |  |  |  |  |  | | --- | --- | --- | --- | --- | |  |  |  |  |  | | EC:2.3.1.41  3-oxoacyl-[acyl-carrier protein] synthase | | | | | | |
| acetyl-CoA assimilation | | |  |  | A | B | C | D | P | P' | N |
|  | Cluster:3-0 | |  |  | 5 | 228 | 16 | 4414 | 0.0031419091 | 0.04084482 | 13 |
|  |  | RAFL07-17-M04 | At1g04410 / malate dehydrogenase, cytosolic, putative | |  |  |  |  |  | | --- | --- | --- | --- | --- | |  |  |  |  |  | | EC:1.1.1.37  malate dehydrogenase | | | | | | |
|  |  | RAFL06-07-J21 | At1g04410 / malate dehydrogenase, cytosolic, putative | |  |  |  |  |  | | --- | --- | --- | --- | --- | |  |  |  |  |  | | EC:1.1.1.37  malate dehydrogenase | | | | | | |
|  |  | RAFL05-21-P13 | At2g42600 / phosphoenolpyruvate carboxylase | |  |  |  |  |  | | --- | --- | --- | --- | --- | |  |  |  |  |  | | EC:4.1.1.31  phosphoenolpyruvate carboxylase | | | | | | |
|  |  | RAFL09-09-M02 | At3g47520 / malate dehydrogenase (NAD), chloroplast, putative | |  |  |  |  |  | | --- | --- | --- | --- | --- | |  |  |  |  |  | | EC:1.1.1.37  malate dehydrogenase | | | | | | |
|  |  | RAFL07-14-B18 | At2g47510 / fumarase -related | |  |  |  |  |  | | --- | --- | --- | --- | --- | |  |  |  |  |  | | EC:4.2.1.2  adenylosuccinate lyase | | | | | | |
| isoleucine degradation I | | |  |  | A | B | C | D | P | P' | N |
|  | Cluster:6-1 | |  |  | 2 | 313 | 1 | 4347 | 0.013038642 | 0.026077284 | 2 |
|  |  | RAFL07-15-A09 | At3g06860 / fatty acid multifunctional protein (AtMFP2) | |  |  |  |  |  | | --- | --- | --- | --- | --- | |  |  |  |  |  | | EC:4.2.1.17  enoyl-CoA hydratase | | | | | | |
|  |  | RAFL11-10-D22 | At5g43280 / enoyl-CoA hydratase/isomerase family | |  |  |  |  |  | | --- | --- | --- | --- | --- | |  |  |  |  |  | | EC:4.2.1.17  enoyl-CoA hydratase | | | | | | |
| cyanate degradation | | |  |  | A | B | C | D | P | P' | N |
|  | Cluster:0-0 | |  |  | 2 | 34 | 3 | 4624 | 5.7119486E-4 | 0.0022847794 | 4 |
|  |  | RAFL06-13-B01 | At3g01500 / carbonic anhydrase, chloroplast precursor | |  |  |  |  |  | | --- | --- | --- | --- | --- | |  |  |  |  |  | | EC:4.2.1.1  carbonate dehydratase | | | | | | |
|  |  | RAFL06-11-K17 | At3g01500 / carbonic anhydrase, chloroplast precursor | |  |  |  |  |  | | --- | --- | --- | --- | --- | |  |  |  |  |  | | EC:4.2.1.1  carbonate dehydratase | | | | | | |
| plastoquinone biosynthesis | | |  |  | A | B | C | D | P | P' | N |
|  | Cluster:9-1 | |  |  | 2 | 94 | 4 | 4563 | 0.005961956 | 0.023847824 | 4 |
|  |  | RAFL11-09-O05 | At1g06570 / 4-hydroxyphenylpyruvate dioxygenase (HPD) | |  |  |  |  |  | | --- | --- | --- | --- | --- | |  |  |  |  |  | | EC:1.13.11.27  4-hydroxyphenylpyruvate dioxygenase | | | | | | |
|  |  | RAFL11-12-C18 | At1g06570 / 4-hydroxyphenylpyruvate dioxygenase (HPD) | |  |  |  |  |  | | --- | --- | --- | --- | --- | |  |  |  |  |  | | EC:1.13.11.27  4-hydroxyphenylpyruvate dioxygenase | | | | | | |
|  | Cluster:9-0 | |  |  | 1 | 31 | 5 | 4626 | 0.04049657 | 0.16198628 | 4 |
|  |  | RAFL05-14-F20 | At1g06570 / 4-hydroxyphenylpyruvate dioxygenase (HPD) | |  |  |  |  |  | | --- | --- | --- | --- | --- | |  |  |  |  |  | | EC:1.13.11.27  4-hydroxyphenylpyruvate dioxygenase | | | | | | |
|  | Cluster:5-1 | |  |  | 2 | 280 | 4 | 4377 | 0.046489924 | 0.1859597 | 4 |
|  |  | RAFL08-16-B22 | At1g11840 / glyoxalase I, putative (lactoylglutathione lyase) | |  |  |  |  |  | | --- | --- | --- | --- | --- | |  |  |  |  |  | | EC:1.13.11.27  4-hydroxyphenylpyruvate dioxygenase | | | | | | |
|  |  | RAFL09-07-G14 | At1g67280 / glyoxalase I, putative (lactoylglutathione lyase) | |  |  |  |  |  | | --- | --- | --- | --- | --- | |  |  |  |  |  | | EC:1.13.11.27  4-hydroxyphenylpyruvate dioxygenase | | | | | | |
| pantothenate biosynthesis | | |  |  | A | B | C | D | P | P' | N |
|  | Cluster:3-1 | |  |  | 1 | 215 | 0 | 4447 | 0.04632211 | 0.04632211 | 1 |
|  |  | RAFL07-09-L18 | At2g40690 / glycerol-3-phosphate dehydrogenase | |  |  |  |  |  | | --- | --- | --- | --- | --- | |  |  |  |  |  | | EC:1.1.1.169  2-dehydropantoate 2-reductase | | | | | | |
| photorespiration | | |  |  | A | B | C | D | P | P' | N |
|  | Cluster:1-0 | |  |  | 4 | 145 | 17 | 4497 | 0.0039202836 | 0.043123122 | 11 |
|  |  | RAFL09-13-P20 | At3g14420 / glycolate oxidase -related | |  |  |  |  |  | | --- | --- | --- | --- | --- | |  |  |  |  |  | | EC:1.1.3.15  glycolate oxidase | | | | | | |
|  |  | RAFL06-14-K21 | At5g36700 / phosphoglycolate phosphatase, putative | |  |  |  |  |  | | --- | --- | --- | --- | --- | |  |  |  |  |  | | EC:3.1.3.18  phosphoglycolate phosphatase | | | | | | |
|  |  | RAFL04-13-N06 | At1g68010 / glycerate dehydrogenase (NADH-dependent hydroxypyruvate reductase) (HPR) | |  |  |  |  |  | | --- | --- | --- | --- | --- | |  |  |  |  |  | | EC:1.1.1.29  phosphoglycerate dehydrogenase | | | | | | |
|  |  | RAFL08-15-E10 | At3g14420 / glycolate oxidase -related | |  |  |  |  |  | | --- | --- | --- | --- | --- | |  |  |  |  |  | | EC:1.1.3.15  glycolate oxidase | | | | | | |
|  | Cluster:1-2 | |  |  | 3 | 171 | 18 | 4471 | 0.0414438 | 0.45588177 | 11 |
|  |  | RAFL05-14-F03 | At1g23310 / alanine aminotransferase -related | |  |  |  |  |  | | --- | --- | --- | --- | --- | |  |  |  |  |  | | EC:2.6.1.4  alanine transaminase | | | | | | |
|  |  | RAFL09-11-K06 | At4g13930 / hydroxymethyltransferase | |  |  |  |  |  | | --- | --- | --- | --- | --- | |  |  |  |  |  | | EC:2.1.2.1  glycine hydroxymethyltransferase | | | | | | |
|  |  | RAFL07-12-E03 | At1g23310 / alanine aminotransferase -related | |  |  |  |  |  | | --- | --- | --- | --- | --- | |  |  |  |  |  | | EC:2.6.1.4  alanine transaminase | | | | | | |
| proline degradation II | | |  |  | A | B | C | D | P | P' | N |
|  | Cluster:9-0 | |  |  | 4 | 28 | 8 | 4623 | 8.7054286E-7 | 7.834886E-6 | 9 |
|  |  | RAFL05-21-E06 | At1g54100 / aldehyde dehydrogenase, putative (ALDH) | |  |  |  |  |  | | --- | --- | --- | --- | --- | |  |  |  |  |  | | EC:1.5.1.12  1-pyrroline-5-carboxylate dehydrogenase//aldehyde dehydrogenase | | | | | | |
|  |  | RAFL04-09-D07 | At1g54100 / aldehyde dehydrogenase, putative (ALDH) | |  |  |  |  |  | | --- | --- | --- | --- | --- | |  |  |  |  |  | | EC:1.5.1.12  1-pyrroline-5-carboxylate dehydrogenase//aldehyde dehydrogenase | | | | | | |
|  |  | RAFL08-15-L09 | At1g54100 / aldehyde dehydrogenase, putative (ALDH) | |  |  |  |  |  | | --- | --- | --- | --- | --- | |  |  |  |  |  | | EC:1.5.1.12  1-pyrroline-5-carboxylate dehydrogenase//aldehyde dehydrogenase | | | | | | |
|  |  | RAFL08-09-C23 | At1g54100 / aldehyde dehydrogenase, putative (ALDH) | |  |  |  |  |  | | --- | --- | --- | --- | --- | |  |  |  |  |  | | EC:1.5.1.12  1-pyrroline-5-carboxylate dehydrogenase//aldehyde dehydrogenase | | | | | | |
| mixed acid fermentation | | |  |  | A | B | C | D | P | P' | N |
|  | Cluster:3-0 | |  |  | 5 | 228 | 12 | 4418 | 0.0011257539 | 0.012383292 | 11 |
|  |  | RAFL07-17-M04 | At1g04410 / malate dehydrogenase, cytosolic, putative | |  |  |  |  |  | | --- | --- | --- | --- | --- | |  |  |  |  |  | | EC:1.1.1.37  malate dehydrogenase | | | | | | |
|  |  | RAFL06-07-J21 | At1g04410 / malate dehydrogenase, cytosolic, putative | |  |  |  |  |  | | --- | --- | --- | --- | --- | |  |  |  |  |  | | EC:1.1.1.37  malate dehydrogenase | | | | | | |
|  |  | RAFL05-21-P13 | At2g42600 / phosphoenolpyruvate carboxylase | |  |  |  |  |  | | --- | --- | --- | --- | --- | |  |  |  |  |  | | EC:4.1.1.31  phosphoenolpyruvate carboxylase | | | | | | |
|  |  | RAFL09-09-M02 | At3g47520 / malate dehydrogenase (NAD), chloroplast, putative | |  |  |  |  |  | | --- | --- | --- | --- | --- | |  |  |  |  |  | | EC:1.1.1.37  malate dehydrogenase | | | | | | |
|  |  | RAFL07-14-B18 | At2g47510 / fumarase -related | |  |  |  |  |  | | --- | --- | --- | --- | --- | |  |  |  |  |  | | EC:4.2.1.2  adenylosuccinate lyase | | | | | | |
| methionine biosynthesis II | | |  |  | A | B | C | D | P | P' | N |
|  | Cluster:1-2 | |  |  | 5 | 169 | 5 | 4484 | 1.4791213E-5 | 5.9164853E-5 | 4 |
|  |  | RAFL06-12-D05 | At5g17920 / 5-methyltetrahydropteroyltriglutamate--homocysteine S-methyltransferase | |  |  |  |  |  | | --- | --- | --- | --- | --- | |  |  |  |  |  | | EC:2.1.1.14  5-methyltetrahydropteroyltriglutamate-homocysteine S-methyltransferase | | | | | | |
|  |  | RAFL11-01-K15 | At5g17920 / 5-methyltetrahydropteroyltriglutamate--homocysteine S-methyltransferase | |  |  |  |  |  | | --- | --- | --- | --- | --- | |  |  |  |  |  | | EC:2.1.1.14  5-methyltetrahydropteroyltriglutamate-homocysteine S-methyltransferase | | | | | | |
|  |  | RAFL09-11-C22 | At5g17920 / 5-methyltetrahydropteroyltriglutamate--homocysteine S-methyltransferase | |  |  |  |  |  | | --- | --- | --- | --- | --- | |  |  |  |  |  | | EC:2.1.1.14  5-methyltetrahydropteroyltriglutamate-homocysteine S-methyltransferase | | | | | | |
|  |  | RAFL11-06-L17 | At5g17920 / 5-methyltetrahydropteroyltriglutamate--homocysteine S-methyltransferase | |  |  |  |  |  | | --- | --- | --- | --- | --- | |  |  |  |  |  | | EC:2.1.1.14  5-methyltetrahydropteroyltriglutamate-homocysteine S-methyltransferase | | | | | | |
|  |  | RAFL09-10-C09 | At5g17920 / 5-methyltetrahydropteroyltriglutamate--homocysteine S-methyltransferase | |  |  |  |  |  | | --- | --- | --- | --- | --- | |  |  |  |  |  | | EC:2.1.1.14  5-methyltetrahydropteroyltriglutamate-homocysteine S-methyltransferase | | | | | | |
|  | Cluster:0-2 | |  |  | 2 | 77 | 8 | 4576 | 0.01167852 | 0.04671408 | 4 |
|  |  | RAFL08-16-E05 | At5g17920 / 5-methyltetrahydropteroyltriglutamate--homocysteine S-methyltransferase | |  |  |  |  |  | | --- | --- | --- | --- | --- | |  |  |  |  |  | | EC:2.1.1.14  5-methyltetrahydropteroyltriglutamate-homocysteine S-methyltransferase | | | | | | |
|  |  | RAFL09-09-A21 | At5g17920 / 5-methyltetrahydropteroyltriglutamate--homocysteine S-methyltransferase | |  |  |  |  |  | | --- | --- | --- | --- | --- | |  |  |  |  |  | | EC:2.1.1.14  5-methyltetrahydropteroyltriglutamate-homocysteine S-methyltransferase | | | | | | |
|  | Cluster:9-1 | |  |  | 2 | 94 | 8 | 4559 | 0.016951112 | 0.06780445 | 4 |
|  |  | RAFL05-18-H15 | At1g64660 / methionine/cystathionine gamma lyase -related | |  |  |  |  |  | | --- | --- | --- | --- | --- | |  |  |  |  |  | | EC:4.4.1.8  cystathionine beta-lyase | | | | | | |
|  |  | RAFL11-02-N11 | At1g64660 / methionine/cystathionine gamma lyase -related | |  |  |  |  |  | | --- | --- | --- | --- | --- | |  |  |  |  |  | | EC:4.4.1.8  cystathionine beta-lyase | | | | | | |
| fatty acid oxidation pathway III | | |  |  | A | B | C | D | P | P' | N |
|  | Cluster:9-0 | |  |  | 4 | 28 | 2 | 4629 | 2.7153972E-8 | 8.146192E-8 | 3 |
|  |  | RAFL05-21-E06 | At1g54100 / aldehyde dehydrogenase, putative (ALDH) | |  |  |  |  |  | | --- | --- | --- | --- | --- | |  |  |  |  |  | | EC:1.2.1.3  unknown | | | | | | |
|  |  | RAFL04-09-D07 | At1g54100 / aldehyde dehydrogenase, putative (ALDH) | |  |  |  |  |  | | --- | --- | --- | --- | --- | |  |  |  |  |  | | EC:1.2.1.3  unknown | | | | | | |
|  |  | RAFL08-15-L09 | At1g54100 / aldehyde dehydrogenase, putative (ALDH) | |  |  |  |  |  | | --- | --- | --- | --- | --- | |  |  |  |  |  | | EC:1.2.1.3  unknown | | | | | | |
|  |  | RAFL08-09-C23 | At1g54100 / aldehyde dehydrogenase, putative (ALDH) | |  |  |  |  |  | | --- | --- | --- | --- | --- | |  |  |  |  |  | | EC:1.2.1.3  unknown | | | | | | |
| fatty acid oxidation pathway | | |  |  | A | B | C | D | P | P' | N |
|  | Cluster:6-1 | |  |  | 3 | 312 | 5 | 4343 | 0.013248402 | 0.07949041 | 6 |
|  |  | RAFL07-15-A09 | At3g06860 / fatty acid multifunctional protein (AtMFP2) | |  |  |  |  |  | | --- | --- | --- | --- | --- | |  |  |  |  |  | | EC:4.2.1.17  enoyl-CoA hydratase | | | | | | |
|  |  | RAFL11-10-D22 | At5g43280 / enoyl-CoA hydratase/isomerase family | |  |  |  |  |  | | --- | --- | --- | --- | --- | |  |  |  |  |  | | EC:4.2.1.17  enoyl-CoA hydratase | | | | | | |
|  |  | RAFL04-20-G12 | At3g05970 / AMP-binding protein, putative | |  |  |  |  |  | | --- | --- | --- | --- | --- | |  |  |  |  |  | | EC:6.2.1.3  long-chain-fatty-acid-CoA ligase | | | | | | |
| isoleucine degradation III | | |  |  | A | B | C | D | P | P' | N |
|  | Cluster:6-1 | |  |  | 2 | 313 | 1 | 4347 | 0.013038642 | 0.026077284 | 2 |
|  |  | RAFL07-15-A09 | At3g06860 / fatty acid multifunctional protein (AtMFP2) | |  |  |  |  |  | | --- | --- | --- | --- | --- | |  |  |  |  |  | | EC:4.2.1.17  enoyl-CoA hydratase | | | | | | |
|  |  | RAFL11-10-D22 | At5g43280 / enoyl-CoA hydratase/isomerase family | |  |  |  |  |  | | --- | --- | --- | --- | --- | |  |  |  |  |  | | EC:4.2.1.17  enoyl-CoA hydratase | | | | | | |
| IAA biosynthesis I | | |  |  | A | B | C | D | P | P' | N |
|  | Cluster:8-0 | |  |  | 2 | 107 | 3 | 4551 | 0.005170766 | 0.020683063 | 4 |
|  |  | RAFL08-10-H06 | At3g44300 / nitrilase 2 | |  |  |  |  |  | | --- | --- | --- | --- | --- | |  |  |  |  |  | | EC:3.5.5.1  hydrolase, acting on carbon-nitrogen (but not peptide) bonds | | | | | | |
|  |  | RAFL06-13-E03 | At3g44300 / nitrilase 2 | |  |  |  |  |  | | --- | --- | --- | --- | --- | |  |  |  |  |  | | EC:3.5.5.1  hydrolase, acting on carbon-nitrogen (but not peptide) bonds | | | | | | |
| biotin biosynthesis I | | |  |  | A | B | C | D | P | P' | N |
|  | Cluster:7-0 | |  |  | 2 | 245 | 2 | 4414 | 0.015618202 | 0.046854608 | 3 |
|  |  | RAFL05-21-O04 | At3g08860 / alanine--glyoxylate aminotransferase (beta-alanine-pyruvate aminotransferase/AGT), putative | |  |  |  |  |  | | --- | --- | --- | --- | --- | |  |  |  |  |  | | EC:2.6.1.62  4-aminobutyrate transaminase//alanine-glyoxylate transaminase | | | | | | |
|  |  | RAFL07-12-J17 | At3g48780 / serine C-palmitoyltransferase, putative | |  |  |  |  |  | | --- | --- | --- | --- | --- | |  |  |  |  |  | | EC:2.3.1.47  8-amino-7-oxononanoate synthase | | | | | | |
|  | Cluster:9-0 | |  |  | 1 | 31 | 3 | 4628 | 0.027177518 | 0.08153255 | 3 |
|  |  | RAFL05-08-B14 | At2g38400 / alanine--glyoxylate aminotransferase (beta-alanine-pyruvate aminotransferase/AGT), putative | |  |  |  |  |  | | --- | --- | --- | --- | --- | |  |  |  |  |  | | EC:2.6.1.62  4-aminobutyrate transaminase//alanine-glyoxylate transaminase | | | | | | |
| sulfate assimilation III | | |  |  | A | B | C | D | P | P' | N |
|  | Cluster:1-2 | |  |  | 6 | 168 | 18 | 4471 | 1.9029251E-4 | 0.0020932176 | 11 |
|  |  | RAFL06-12-D05 | At5g17920 / 5-methyltetrahydropteroyltriglutamate--homocysteine S-methyltransferase | |  |  |  |  |  | | --- | --- | --- | --- | --- | |  |  |  |  |  | | EC:2.1.1.14  5-methyltetrahydropteroyltriglutamate-homocysteine S-methyltransferase | | | | | | |
|  |  | RAFL11-01-K15 | At5g17920 / 5-methyltetrahydropteroyltriglutamate--homocysteine S-methyltransferase | |  |  |  |  |  | | --- | --- | --- | --- | --- | |  |  |  |  |  | | EC:2.1.1.14  5-methyltetrahydropteroyltriglutamate-homocysteine S-methyltransferase | | | | | | |
|  |  | RAFL09-11-C22 | At5g17920 / 5-methyltetrahydropteroyltriglutamate--homocysteine S-methyltransferase | |  |  |  |  |  | | --- | --- | --- | --- | --- | |  |  |  |  |  | | EC:2.1.1.14  5-methyltetrahydropteroyltriglutamate-homocysteine S-methyltransferase | | | | | | |
|  |  | RAFL11-06-L17 | At5g17920 / 5-methyltetrahydropteroyltriglutamate--homocysteine S-methyltransferase | |  |  |  |  |  | | --- | --- | --- | --- | --- | |  |  |  |  |  | | EC:2.1.1.14  5-methyltetrahydropteroyltriglutamate-homocysteine S-methyltransferase | | | | | | |
|  |  | RAFL11-10-I18 | At1g19920 / sulfate adenylyltransferase | |  |  |  |  |  | | --- | --- | --- | --- | --- | |  |  |  |  |  | | EC:2.7.7.4  ATP sulfurylase | | | | | | |
|  |  | RAFL09-10-C09 | At5g17920 / 5-methyltetrahydropteroyltriglutamate--homocysteine S-methyltransferase | |  |  |  |  |  | | --- | --- | --- | --- | --- | |  |  |  |  |  | | EC:2.1.1.14  5-methyltetrahydropteroyltriglutamate-homocysteine S-methyltransferase | | | | | | |
|  | Cluster:2-2 | |  |  | 3 | 50 | 21 | 4589 | 0.0023710139 | 0.026081152 | 11 |
|  |  | RAFL04-17-H16 | At3g13110 / serine acetyltransferase (Sat-1) | |  |  |  |  |  | | --- | --- | --- | --- | --- | |  |  |  |  |  | | EC:2.3.1.30  serine O-acetyltransferase | | | | | | |
|  |  | RAFL07-10-P11 | At3g22890 / ATP sulfurylase -related | |  |  |  |  |  | | --- | --- | --- | --- | --- | |  |  |  |  |  | | EC:2.7.7.4  ATP sulfurylase | | | | | | |
|  |  | RAFL07-12-E10 | At3g22890 / ATP sulfurylase -related | |  |  |  |  |  | | --- | --- | --- | --- | --- | |  |  |  |  |  | | EC:2.7.7.4  ATP sulfurylase | | | | | | |
| suberin biosynthesis | | |  |  | A | B | C | D | P | P' | N |
|  | Cluster:6-2 | |  |  | 4 | 172 | 7 | 4480 | 5.2568526E-4 | 0.0031541116 | 6 |
|  |  | RAFL09-11-L22 | At3g53260 / phenylalanine ammonia-lyase (PAL2) | |  |  |  |  |  | | --- | --- | --- | --- | --- | |  |  |  |  |  | | EC:4.3.1.5  phenylalanine ammonia-lyase | | | | | | |
|  |  | RAFL04-13-B02 | At2g37040 / phenylalanine ammonia lyase (PAL1) | |  |  |  |  |  | | --- | --- | --- | --- | --- | |  |  |  |  |  | | EC:4.3.1.5  phenylalanine ammonia-lyase | | | | | | |
|  |  | RAFL04-16-D08 | At3g53260 / phenylalanine ammonia-lyase (PAL2) | |  |  |  |  |  | | --- | --- | --- | --- | --- | |  |  |  |  |  | | EC:4.3.1.5  phenylalanine ammonia-lyase | | | | | | |
|  |  | RAFL04-17-C19 | At4g34050 / caffeoyl-CoA 3-O-methyltransferase | |  |  |  |  |  | | --- | --- | --- | --- | --- | |  |  |  |  |  | | EC:2.1.1.104  caffeoyl-CoA O-methyltransferase | | | | | | |
|  | Cluster:7-0 | |  |  | 3 | 244 | 8 | 4408 | 0.017647006 | 0.10588203 | 6 |
|  |  | RAFL06-11-J16 | At2g30490 / cytochrome P450 73 / trans-cinnamate 4-monooxygenase / cinnamate-4-hydroxylase (CYP73) (C4H) | |  |  |  |  |  | | --- | --- | --- | --- | --- | |  |  |  |  |  | | EC:1.14.13.11  cinnamate 4-hydroxylase | | | | | | |
|  |  | RAFL09-15-A15 | At4g26220 / caffeoyl-CoA 3-O-methyltransferase, putative | |  |  |  |  |  | | --- | --- | --- | --- | --- | |  |  |  |  |  | | EC:2.1.1.104  caffeoyl-CoA O-methyltransferase | | | | | | |
|  |  | RAFL06-07-M15 | At5g54160 / O-methyltransferase 1 | |  |  |  |  |  | | --- | --- | --- | --- | --- | |  |  |  |  |  | | EC:2.1.1.68  caffeic acid /5-hydroxyferulic acid o-methyltransferase | | | | | | |
| cellulose biosynthesis | | |  |  | A | B | C | D | P | P' | N |
|  | Cluster:3-1 | |  |  | 5 | 211 | 9 | 4438 | 2.892519E-4 | 0.0023140153 | 8 |
|  |  | RAFL05-02-P19 | At5g64740 / cellulose synthase, catalytic subunit, putative | |  |  |  |  |  | | --- | --- | --- | --- | --- | |  |  |  |  |  | | EC:2.4.1.12  cellulose synthase (UDP-forming) | | | | | | |
|  |  | RAFL05-19-M03 | At5g05170 / cellulose synthase, catalytic subunit (Ath-B) | |  |  |  |  |  | | --- | --- | --- | --- | --- | |  |  |  |  |  | | EC:2.4.1.12  cellulose synthase (UDP-forming) | | | | | | |
|  |  | RAFL09-10-K01 | At5g22740 / glycosyltransferase family 2 | |  |  |  |  |  | | --- | --- | --- | --- | --- | |  |  |  |  |  | | EC:2.4.1.12  cellulose synthase (UDP-forming) | | | | | | |
|  |  | RAFL04-19-E02 | At4g32410 / cellulose synthase, catalytic subunit, putative | |  |  |  |  |  | | --- | --- | --- | --- | --- | |  |  |  |  |  | | EC:2.4.1.12  cellulose synthase (UDP-forming) | | | | | | |
|  |  | RAFL05-02-G06 | At5g49720 / glycosyl hydrolase family 9 (endo-1,4-beta-glucanase) | |  |  |  |  |  | | --- | --- | --- | --- | --- | |  |  |  |  |  | | EC:3.2.1.4  1,4-b-D-glucan 4-glucanohydrolase | | | | | | |
|  | Cluster:7-2 | |  |  | 2 | 62 | 12 | 4587 | 0.015175772 | 0.121406175 | 8 |
|  |  | RAFL05-09-M07 | At5g64740 / cellulose synthase, catalytic subunit, putative | |  |  |  |  |  | | --- | --- | --- | --- | --- | |  |  |  |  |  | | EC:2.4.1.12  cellulose synthase (UDP-forming) | | | | | | |
|  |  | RAFL09-16-P14 | At3g03050 / cellulose synthase family | |  |  |  |  |  | | --- | --- | --- | --- | --- | |  |  |  |  |  | | EC:2.4.1.12  cellulose synthase (UDP-forming) | | | | | | |
| xylose degradation | | |  |  | A | B | C | D | P | P' | N |
|  | Cluster:8-0 | |  |  | 1 | 108 | 1 | 4553 | 0.0462095 | 0.092419 | 2 |
|  |  | RAFL05-15-K08 | At5g57655 / expressed protein | |  |  |  |  |  | | --- | --- | --- | --- | --- | |  |  |  |  |  | | EC:5.3.1.5  xylose isomerase | | | | | | |
| glutamine degradation III | | |  |  | A | B | C | D | P | P' | N |
|  | Cluster:3-0 | |  |  | 2 | 231 | 4 | 4426 | 0.032636177 | 0.1305447 | 4 |
|  |  | RAFL07-14-B18 | At2g47510 / fumarase -related | |  |  |  |  |  | | --- | --- | --- | --- | --- | |  |  |  |  |  | | EC:4.3.1.1  fumarate hydratase | | | | | | |
|  |  | RAFL09-07-B08 | At2g30970 / aspartate aminotransferase, mitochondrial (transaminase A/Asp1) | |  |  |  |  |  | | --- | --- | --- | --- | --- | |  |  |  |  |  | | EC:2.6.1.1  1-aminocyclopropane-1-carboxylate synthase | | | | | | |
| cysteine biosynthesis I | | |  |  | A | B | C | D | P | P' | N |
|  | Cluster:2-0 | |  |  | 2 | 148 | 4 | 4509 | 0.014161414 | 0.07080707 | 5 |
|  |  | RAFL05-10-D20 | At3g59760 / cysteine synthase, mitochondrial (O-acetylserine (thiol)-lyase/O-acetylserine sulfhydrylase), putative | |  |  |  |  |  | | --- | --- | --- | --- | --- | |  |  |  |  |  | | EC:4.2.99.8  cystathionine beta-synthase//lyase | | | | | | |
|  |  | RAFL07-18-N19 | At3g61440 / cysteine synthase (O-acetylserine (thiol)-lyase/O-acetylserine sulfhydrylase/AtcysC1) | |  |  |  |  |  | | --- | --- | --- | --- | --- | |  |  |  |  |  | | EC:4.2.99.8  cystathionine beta-synthase//lyase | | | | | | |
| methionine degradation I | | |  |  | A | B | C | D | P | P' | N |
|  | Cluster:3-2 | |  |  | 4 | 33 | 6 | 4620 | 6.813379E-7 | 3.4066893E-6 | 5 |
|  |  | RAFL09-06-N12 | At3g23810 / S-adenosyl-L-homocysteinas -related | |  |  |  |  |  | | --- | --- | --- | --- | --- | |  |  |  |  |  | | EC:3.3.1.1  adenosylhomocysteinase | | | | | | |
|  |  | RAFL07-09-L01 | At3g23810 / S-adenosyl-L-homocysteinas -related | |  |  |  |  |  | | --- | --- | --- | --- | --- | |  |  |  |  |  | | EC:3.3.1.1  adenosylhomocysteinase | | | | | | |
|  |  | RAFL09-13-P13 | At3g23810 / S-adenosyl-L-homocysteinas -related | |  |  |  |  |  | | --- | --- | --- | --- | --- | |  |  |  |  |  | | EC:3.3.1.1  adenosylhomocysteinase | | | | | | |
|  |  | RAFL09-10-M18 | At3g23810 / S-adenosyl-L-homocysteinas -related | |  |  |  |  |  | | --- | --- | --- | --- | --- | |  |  |  |  |  | | EC:3.3.1.1  adenosylhomocysteinase | | | | | | |
|  | Cluster:1-0 | |  |  | 2 | 147 | 8 | 4506 | 0.038570926 | 0.19285463 | 5 |
|  |  | RAFL02-08-J05 | At3g17390 / s-adenosylmethionine synthetase -related | |  |  |  |  |  | | --- | --- | --- | --- | --- | |  |  |  |  |  | | EC:2.5.1.6  methionine adenosyltransferase | | | | | | |
|  |  | RAFL05-12-C12 | At3g17390 / s-adenosylmethionine synthetase -related | |  |  |  |  |  | | --- | --- | --- | --- | --- | |  |  |  |  |  | | EC:2.5.1.6  methionine adenosyltransferase | | | | | | |
| citrulline degradation | | |  |  | A | B | C | D | P | P' | N |
|  | Cluster:3-1 | |  |  | 1 | 215 | 0 | 4447 | 0.04632211 | 0.04632211 | 1 |
|  |  | RAFL07-16-F16 | At3g20330 / aspartate carbamoyltransferase precursor (aspartate transcarbamylase) | |  |  |  |  |  | | --- | --- | --- | --- | --- | |  |  |  |  |  | | EC:2.1.3.3  ornithine carbamoyltransferase | | | | | | |
| valine biosynthesis | | |  |  | A | B | C | D | P | P' | N |
|  | Cluster:4-0 | |  |  | 2 | 238 | 5 | 4418 | 0.046670057 | 0.23335029 | 5 |
|  |  | RAFL06-16-L13 | At5g54960 / pyruvate decarboxylase (gb|AAB16855.1) | |  |  |  |  |  | | --- | --- | --- | --- | --- | |  |  |  |  |  | | EC:4.1.3.18  acetolactate synthase | | | | | | |
|  |  | RAFL09-18-P18 | At3g48560 / acetolactate synthase | |  |  |  |  |  | | --- | --- | --- | --- | --- | |  |  |  |  |  | | EC:4.1.3.18  acetolactate synthase | | | | | | |
|  | Cluster:2-1 | |  |  | 2 | 242 | 5 | 4414 | 0.04810191 | 0.24050954 | 5 |
|  |  | RAFL02-01-G08 | At3g58610 / ketol-acid reductoisomerase | |  |  |  |  |  | | --- | --- | --- | --- | --- | |  |  |  |  |  | | EC:1.1.1.86  ketol-acid reductoisomerase | | | | | | |
|  |  | RAFL04-13-D06 | At3g23940 / dihydroxyacid dehydratase -related | |  |  |  |  |  | | --- | --- | --- | --- | --- | |  |  |  |  |  | | EC:4.2.1.9  unknown | | | | | | |
| Calvin cycle | | |  |  | A | B | C | D | P | P' | N |
|  | Cluster:0-1 | |  |  | 21 | 85 | 31 | 4526 | 4.2892147E-22 | 4.7181364E-21 | 11 |
|  |  | RAFL07-18-C20 | At2g21330 / fructose-bisphosphate aldolase, putative | |  |  |  |  |  | | --- | --- | --- | --- | --- | |  |  |  |  |  | | EC:4.1.2.13  fructose-bisphosphate aldolase | | | | | | |
|  |  | RAFL09-15-L04 | At3g12780 / phosphoglycerate kinase -related | |  |  |  |  |  | | --- | --- | --- | --- | --- | |  |  |  |  |  | | EC:2.7.2.3  phosphoglycerate kinase | | | | | | |
|  |  | RAFL07-14-L16 | At3g12780 / phosphoglycerate kinase -related | |  |  |  |  |  | | --- | --- | --- | --- | --- | |  |  |  |  |  | | EC:2.7.2.3  phosphoglycerate kinase | | | | | | |
|  |  | RAFL08-18-C10 | At2g21330 / fructose-bisphosphate aldolase, putative | |  |  |  |  |  | | --- | --- | --- | --- | --- | |  |  |  |  |  | | EC:4.1.2.13  fructose-bisphosphate aldolase | | | | | | |
|  |  | RAFL07-12-E12 | At2g21330 / fructose-bisphosphate aldolase, putative | |  |  |  |  |  | | --- | --- | --- | --- | --- | |  |  |  |  |  | | EC:4.1.2.13  fructose-bisphosphate aldolase | | | | | | |
|  |  | RAFL07-07-I23 | At1g56190 / phosphoglycerate kinase -related | |  |  |  |  |  | | --- | --- | --- | --- | --- | |  |  |  |  |  | | EC:2.7.2.3  phosphoglycerate kinase | | | | | | |
|  |  | RAFL04-13-J02 | At3g54050 / fructose-bisphosphatase precursor | |  |  |  |  |  | | --- | --- | --- | --- | --- | |  |  |  |  |  | | EC:3.1.3.11  fructose-bisphosphatase//phosphoric ester hydrolase | | | | | | |
|  |  | RAFL07-12-M09 | At2g21330 / fructose-bisphosphate aldolase, putative | |  |  |  |  |  | | --- | --- | --- | --- | --- | |  |  |  |  |  | | EC:4.1.2.13  fructose-bisphosphate aldolase | | | | | | |
|  |  | RAFL07-16-P05 | At3g12780 / phosphoglycerate kinase -related | |  |  |  |  |  | | --- | --- | --- | --- | --- | |  |  |  |  |  | | EC:2.7.2.3  phosphoglycerate kinase | | | | | | |
|  |  | RAFL09-06-P15 | At1g67090 / ribulose-bisphosphate carboxylase small unit -related | |  |  |  |  |  | | --- | --- | --- | --- | --- | |  |  |  |  |  | | EC:4.1.1.39  ribulose-bisphosphate carboxylase | | | | | | |
|  |  | RAFL09-18-L22 | At3g12780 / phosphoglycerate kinase -related | |  |  |  |  |  | | --- | --- | --- | --- | --- | |  |  |  |  |  | | EC:2.7.2.3  phosphoglycerate kinase | | | | | | |
|  |  | RAFL04-19-O21 | At4g38970 / fructose-bisphosphate aldolase, putative | |  |  |  |  |  | | --- | --- | --- | --- | --- | |  |  |  |  |  | | EC:4.1.2.13  fructose-bisphosphate aldolase | | | | | | |
|  |  | RAFL09-17-N23 | At2g21330 / fructose-bisphosphate aldolase, putative | |  |  |  |  |  | | --- | --- | --- | --- | --- | |  |  |  |  |  | | EC:4.1.2.13  fructose-bisphosphate aldolase | | | | | | |
|  |  | RAFL05-12-O19 | At3g55800 / sedoheptulose-bisphosphatase precursor | |  |  |  |  |  | | --- | --- | --- | --- | --- | |  |  |  |  |  | | EC:3.1.3.37  unknown | | | | | | |
|  |  | RAFL04-19-M17 | At1g32060 / phosphoribulokinase precursor | |  |  |  |  |  | | --- | --- | --- | --- | --- | |  |  |  |  |  | | EC:2.7.1.19  phosphoribulokinase | | | | | | |
|  |  | RAFL04-10-J07 | At3g04790 / ribose 5-phosphate isomerase -related | |  |  |  |  |  | | --- | --- | --- | --- | --- | |  |  |  |  |  | | EC:5.3.1.6  ribose-5-phosphate isomerase | | | | | | |
|  |  | RAFL07-18-J01 | At2g21330 / fructose-bisphosphate aldolase, putative | |  |  |  |  |  | | --- | --- | --- | --- | --- | |  |  |  |  |  | | EC:4.1.2.13  fructose-bisphosphate aldolase | | | | | | |
|  |  | RAFL09-06-K21 | At1g67090 / ribulose-bisphosphate carboxylase small unit -related | |  |  |  |  |  | | --- | --- | --- | --- | --- | |  |  |  |  |  | | EC:4.1.1.39  ribulose-bisphosphate carboxylase | | | | | | |
|  |  | RAFL03-06-F08 | At1g67090 / ribulose-bisphosphate carboxylase small unit -related | |  |  |  |  |  | | --- | --- | --- | --- | --- | |  |  |  |  |  | | EC:4.1.1.39  ribulose-bisphosphate carboxylase | | | | | | |
|  |  | RAFL11-07-D01 | At5g38410 / ribulose bisphosphate carboxylase small chain 3b precursor (RuBisCO small subunit 3b) (sp|P10798) | |  |  |  |  |  | | --- | --- | --- | --- | --- | |  |  |  |  |  | | EC:4.1.1.39  ribulose-bisphosphate carboxylase | | | | | | |
|  |  | RAFL11-02-L02 | At1g67090 / ribulose-bisphosphate carboxylase small unit -related | |  |  |  |  |  | | --- | --- | --- | --- | --- | |  |  |  |  |  | | EC:4.1.1.39  ribulose-bisphosphate carboxylase | | | | | | |
|  | Cluster:0-0 | |  |  | 15 | 21 | 37 | 4590 | 2.6605689E-21 | 2.9266258E-20 | 11 |
|  |  | RAFL06-14-C19 | At5g38410 / ribulose bisphosphate carboxylase small chain 3b precursor (RuBisCO small subunit 3b) (sp|P10798) | |  |  |  |  |  | | --- | --- | --- | --- | --- | |  |  |  |  |  | | EC:4.1.1.39  ribulose-bisphosphate carboxylase | | | | | | |
|  |  | RAFL06-10-O15 | At5g38420 / ribulose bisphosphate carboxylase small chain 2b precursor (RuBisCO small subunit 2b) (sp|P10797) | |  |  |  |  |  | | --- | --- | --- | --- | --- | |  |  |  |  |  | | EC:4.1.1.39  ribulose-bisphosphate carboxylase | | | | | | |
|  |  | RAFL06-13-H11 | At5g38430 / ribulose bisphosphate carboxylase small chain 1b precursor (RuBisCO small subunit 1b) (sp|P10796) | |  |  |  |  |  | | --- | --- | --- | --- | --- | |  |  |  |  |  | | EC:4.1.1.39  ribulose-bisphosphate carboxylase | | | | | | |
|  |  | RAFL09-09-L07 | At1g67090 / ribulose-bisphosphate carboxylase small unit -related | |  |  |  |  |  | | --- | --- | --- | --- | --- | |  |  |  |  |  | | EC:4.1.1.39  ribulose-bisphosphate carboxylase | | | | | | |
|  |  | RAFL06-14-L16 | At5g38430 / ribulose bisphosphate carboxylase small chain 1b precursor (RuBisCO small subunit 1b) (sp|P10796) | |  |  |  |  |  | | --- | --- | --- | --- | --- | |  |  |  |  |  | | EC:4.1.1.39  ribulose-bisphosphate carboxylase | | | | | | |
|  |  | RAFL04-15-J15 | At5g38410 / ribulose bisphosphate carboxylase small chain 3b precursor (RuBisCO small subunit 3b) (sp|P10798) | |  |  |  |  |  | | --- | --- | --- | --- | --- | |  |  |  |  |  | | EC:4.1.1.39  ribulose-bisphosphate carboxylase | | | | | | |
|  |  | RAFL06-08-L09 | At5g38420 / ribulose bisphosphate carboxylase small chain 2b precursor (RuBisCO small subunit 2b) (sp|P10797) | |  |  |  |  |  | | --- | --- | --- | --- | --- | |  |  |  |  |  | | EC:4.1.1.39  ribulose-bisphosphate carboxylase | | | | | | |
|  |  | RAFL07-14-L17 | At5g38420 / ribulose bisphosphate carboxylase small chain 2b precursor (RuBisCO small subunit 2b) (sp|P10797) | |  |  |  |  |  | | --- | --- | --- | --- | --- | |  |  |  |  |  | | EC:4.1.1.39  ribulose-bisphosphate carboxylase | | | | | | |
|  |  | RAFL06-14-C14 | At1g67090 / ribulose-bisphosphate carboxylase small unit -related | |  |  |  |  |  | | --- | --- | --- | --- | --- | |  |  |  |  |  | | EC:4.1.1.39  ribulose-bisphosphate carboxylase | | | | | | |
|  |  | RAFL08-17-J10 | At5g38420 / ribulose bisphosphate carboxylase small chain 2b precursor (RuBisCO small subunit 2b) (sp|P10797) | |  |  |  |  |  | | --- | --- | --- | --- | --- | |  |  |  |  |  | | EC:4.1.1.39  ribulose-bisphosphate carboxylase | | | | | | |
|  |  | RAFL06-07-I02 | At1g67090 / ribulose-bisphosphate carboxylase small unit -related | |  |  |  |  |  | | --- | --- | --- | --- | --- | |  |  |  |  |  | | EC:4.1.1.39  ribulose-bisphosphate carboxylase | | | | | | |
|  |  | RAFL11-03-H09 | At1g67090 / ribulose-bisphosphate carboxylase small unit -related | |  |  |  |  |  | | --- | --- | --- | --- | --- | |  |  |  |  |  | | EC:4.1.1.39  ribulose-bisphosphate carboxylase | | | | | | |
|  |  | RAFL09-16-C21 | At5g38410 / ribulose bisphosphate carboxylase small chain 3b precursor (RuBisCO small subunit 3b) (sp|P10798) | |  |  |  |  |  | | --- | --- | --- | --- | --- | |  |  |  |  |  | | EC:4.1.1.39  ribulose-bisphosphate carboxylase | | | | | | |
|  |  | RAFL09-09-K05 | At5g38410 / ribulose bisphosphate carboxylase small chain 3b precursor (RuBisCO small subunit 3b) (sp|P10798) | |  |  |  |  |  | | --- | --- | --- | --- | --- | |  |  |  |  |  | | EC:4.1.1.39  ribulose-bisphosphate carboxylase | | | | | | |
|  |  | RAFL07-11-L12 | At5g38420 / ribulose bisphosphate carboxylase small chain 2b precursor (RuBisCO small subunit 2b) (sp|P10797) | |  |  |  |  |  | | --- | --- | --- | --- | --- | |  |  |  |  |  | | EC:4.1.1.39  ribulose-bisphosphate carboxylase | | | | | | |
| methionine and <I>S</I>-adenosylmethionine synthesis | | |  |  | A | B | C | D | P | P' | N |
|  | Cluster:1-2 | |  |  | 5 | 169 | 8 | 4481 | 6.892205E-5 | 4.135323E-4 | 6 |
|  |  | RAFL06-12-D05 | At5g17920 / 5-methyltetrahydropteroyltriglutamate--homocysteine S-methyltransferase | |  |  |  |  |  | | --- | --- | --- | --- | --- | |  |  |  |  |  | | EC:2.1.1.14  5-methyltetrahydropteroyltriglutamate-homocysteine S-methyltransferase | | | | | | |
|  |  | RAFL11-01-K15 | At5g17920 / 5-methyltetrahydropteroyltriglutamate--homocysteine S-methyltransferase | |  |  |  |  |  | | --- | --- | --- | --- | --- | |  |  |  |  |  | | EC:2.1.1.14  5-methyltetrahydropteroyltriglutamate-homocysteine S-methyltransferase | | | | | | |
|  |  | RAFL09-11-C22 | At5g17920 / 5-methyltetrahydropteroyltriglutamate--homocysteine S-methyltransferase | |  |  |  |  |  | | --- | --- | --- | --- | --- | |  |  |  |  |  | | EC:2.1.1.14  5-methyltetrahydropteroyltriglutamate-homocysteine S-methyltransferase | | | | | | |
|  |  | RAFL11-06-L17 | At5g17920 / 5-methyltetrahydropteroyltriglutamate--homocysteine S-methyltransferase | |  |  |  |  |  | | --- | --- | --- | --- | --- | |  |  |  |  |  | | EC:2.1.1.14  5-methyltetrahydropteroyltriglutamate-homocysteine S-methyltransferase | | | | | | |
|  |  | RAFL09-10-C09 | At5g17920 / 5-methyltetrahydropteroyltriglutamate--homocysteine S-methyltransferase | |  |  |  |  |  | | --- | --- | --- | --- | --- | |  |  |  |  |  | | EC:2.1.1.14  5-methyltetrahydropteroyltriglutamate-homocysteine S-methyltransferase | | | | | | |
|  | Cluster:0-2 | |  |  | 2 | 77 | 11 | 4573 | 0.019588634 | 0.1175318 | 6 |
|  |  | RAFL08-16-E05 | At5g17920 / 5-methyltetrahydropteroyltriglutamate--homocysteine S-methyltransferase | |  |  |  |  |  | | --- | --- | --- | --- | --- | |  |  |  |  |  | | EC:2.1.1.14  5-methyltetrahydropteroyltriglutamate-homocysteine S-methyltransferase | | | | | | |
|  |  | RAFL09-09-A21 | At5g17920 / 5-methyltetrahydropteroyltriglutamate--homocysteine S-methyltransferase | |  |  |  |  |  | | --- | --- | --- | --- | --- | |  |  |  |  |  | | EC:2.1.1.14  5-methyltetrahydropteroyltriglutamate-homocysteine S-methyltransferase | | | | | | |
| starch degradation | | |  |  | A | B | C | D | P | P' | N |
|  | Cluster:5-2 | |  |  | 2 | 124 | 5 | 4532 | 0.013917747 | 0.069588736 | 5 |
|  |  | RAFL05-03-E09 | At5g64860 / glycosyl hydrolase family 77 (4-alpha-glucanotransferase) | |  |  |  |  |  | | --- | --- | --- | --- | --- | |  |  |  |  |  | | EC:2.4.1.25  disproportionating enzyme | | | | | | |
|  |  | RAFL09-14-L23 | At5g11720 / glycosyl hydrolase family 31 | |  |  |  |  |  | | --- | --- | --- | --- | --- | |  |  |  |  |  | | EC:3.2.1.20  unknown | | | | | | |
|  | Cluster:3-0 | |  |  | 2 | 231 | 5 | 4425 | 0.044206142 | 0.2210307 | 5 |
|  |  | RAFL08-08-F08 | At1g69830 / alpha-amylase (1,4-alpha-D-glucan glucanohydrolase), putative | |  |  |  |  |  | | --- | --- | --- | --- | --- | |  |  |  |  |  | | EC:3.2.1.1  alpha-amylase | | | | | | |
|  |  | RAFL08-09-A13 | At3g29320 / glucan phosphorylase, putative | |  |  |  |  |  | | --- | --- | --- | --- | --- | |  |  |  |  |  | | EC:2.4.1.1  starch phosphorylase | | | | | | |
| sterol biosynthesis | | |  |  | A | B | C | D | P | P' | N |
|  | Cluster:3-1 | |  |  | 2 | 214 | 5 | 4442 | 0.03844968 | 0.1922484 | 5 |
|  |  | RAFL11-10-O06 | At1g50430 / sterol delta-7 reductase (7-dehydrocholesterol reductase) (dwarf5) (DWF5) | |  |  |  |  |  | | --- | --- | --- | --- | --- | |  |  |  |  |  | | EC:1.3.1.21  sterol Δ<sup>7</sup> reductase | | | | | | |
|  |  | RAFL02-01-C03 | At1g76090 / S-adenosyl-methionine-sterol-C-methyltransferase -related | |  |  |  |  |  | | --- | --- | --- | --- | --- | |  |  |  |  |  | | EC:2.1.1.143  24-methylenelophenol-C24-methyltransferase | | | | | | |
|  | Cluster:2-1 | |  |  | 2 | 242 | 5 | 4414 | 0.04810191 | 0.24050954 | 5 |
|  |  | RAFL07-16-C24 | At1g20330 / sterol-C-methyltransferase | |  |  |  |  |  | | --- | --- | --- | --- | --- | |  |  |  |  |  | | EC:2.1.1.143  24-methylenelophenol-C24-methyltransferase | | | | | | |
|  |  | RAFL07-09-C04 | At1g11680 / obtusifoliol 14-demethylase (CYP51) | |  |  |  |  |  | | --- | --- | --- | --- | --- | |  |  |  |  |  | | EC:1.14.13.70  obtusifoliol 14α-demethylase | | | | | | |
| methionine degradation II | | |  |  | A | B | C | D | P | P' | N |
|  | Cluster:9-1 | |  |  | 2 | 94 | 0 | 4567 | 4.1952432E-4 | 4.1952432E-4 | 1 |
|  |  | RAFL05-18-H15 | At1g64660 / methionine/cystathionine gamma lyase -related | |  |  |  |  |  | | --- | --- | --- | --- | --- | |  |  |  |  |  | | EC:4.4.1.11  1-aminocyclopropane-1-carboxylate synthase | | | | | | |
|  |  | RAFL11-02-N11 | At1g64660 / methionine/cystathionine gamma lyase -related | |  |  |  |  |  | | --- | --- | --- | --- | --- | |  |  |  |  |  | | EC:4.4.1.11  1-aminocyclopropane-1-carboxylate synthase | | | | | | |
| NAD phosphorylation and dephosphorylation | | |  |  | A | B | C | D | P | P' | N |
|  | Cluster:3-0 | |  |  | 1 | 232 | 0 | 4430 | 0.049967833 | 0.049967833 | 1 |
|  |  | RAFL05-10-H23 | At1g21640 / expressed protein | |  |  |  |  |  | | --- | --- | --- | --- | --- | |  |  |  |  |  | | EC:2.7.1.23  NAD+ kinase | | | | | | |
| aerobic respiration -- electron donors reaction list | | |  |  | A | B | C | D | P | P' | N |
|  | Cluster:4-1 | |  |  | 5 | 305 | 11 | 4342 | 0.0029831196 | 0.020881835 | 7 |
|  |  | RAFL05-04-N24 | At3g18410 / expressed protein | |  |  |  |  |  | | --- | --- | --- | --- | --- | |  |  |  |  |  | | EC:1.6.5.3  NADH dehydrogenase (ubiquinone) | | | | | | |
|  |  | RAFL11-02-J20 | At3g12260 / expressed protein | |  |  |  |  |  | | --- | --- | --- | --- | --- | |  |  |  |  |  | | EC:1.6.5.3  NADH dehydrogenase (ubiquinone) | | | | | | |
|  |  | RAFL09-10-O11 | At3g12260 / expressed protein | |  |  |  |  |  | | --- | --- | --- | --- | --- | |  |  |  |  |  | | EC:1.6.5.3  NADH dehydrogenase (ubiquinone) | | | | | | |
|  |  | RAFL09-18-I01 | At5g08530 / NADH-ubiquinone oxidoreductase (mitochondrial), putative | |  |  |  |  |  | | --- | --- | --- | --- | --- | |  |  |  |  |  | | EC:1.6.5.3 ,EC:1.18.99.1  NADH dehydrogenase (ubiquinone) | | | | | | |
|  |  | RAFL06-08-D19 | At5g37510 / NADH dehydrogenase (ubiquinone), mitochondrial, putative | |  |  |  |  |  | | --- | --- | --- | --- | --- | |  |  |  |  |  | | EC:1.6.5.3  NADH dehydrogenase (ubiquinone) | | | | | | |
|  | Cluster:6-0 | |  |  | 3 | 137 | 13 | 4510 | 0.011134108 | 0.07793876 | 7 |
|  |  | RAFL06-16-I03 | At5g09600 / expressed protein | |  |  |  |  |  | | --- | --- | --- | --- | --- | |  |  |  |  |  | | EC:1.3.5.1  succinate dehydrogenase (ubiquinone) | | | | | | |
|  |  | RAFL08-12-A05 | At5g11770 / NADH dehydrogenase (ubiquinone) | |  |  |  |  |  | | --- | --- | --- | --- | --- | |  |  |  |  |  | | EC:1.6.5.3  NADH dehydrogenase (ubiquinone) | | | | | | |
|  |  | RAFL05-17-L16 | At1g79010 / NADH dehydrogenase -related | |  |  |  |  |  | | --- | --- | --- | --- | --- | |  |  |  |  |  | | EC:1.6.5.3  NADH dehydrogenase (ubiquinone) | | | | | | |
|  | Cluster:6-1 | |  |  | 4 | 311 | 12 | 4336 | 0.019434473 | 0.13604131 | 7 |
|  |  | RAFL06-10-E05 | At1g16700 / NADH:ubiquinone oxidoreductase -related | |  |  |  |  |  | | --- | --- | --- | --- | --- | |  |  |  |  |  | | EC:1.6.5.3  NADH dehydrogenase (ubiquinone) | | | | | | |
|  |  | RAFL05-08-F21 | At3g03100 / expressed protein | |  |  |  |  |  | | --- | --- | --- | --- | --- | |  |  |  |  |  | | EC:1.6.5.3  NADH dehydrogenase (ubiquinone) | | | | | | |
|  |  | RAFL05-17-A04 | At5g09600 / expressed protein | |  |  |  |  |  | | --- | --- | --- | --- | --- | |  |  |  |  |  | | EC:1.3.5.1  succinate dehydrogenase (ubiquinone) | | | | | | |
|  |  | RAFL07-07-N09 | At5g37510 / NADH dehydrogenase (ubiquinone), mitochondrial, putative | |  |  |  |  |  | | --- | --- | --- | --- | --- | |  |  |  |  |  | | EC:1.6.5.3  NADH dehydrogenase (ubiquinone) | | | | | | |
| GDP-D-rhamnose biosynthesis | | |  |  | A | B | C | D | P | P' | N |
|  | Cluster:3-2 | |  |  | 2 | 35 | 7 | 4619 | 0.0021297743 | 0.010648872 | 5 |
|  |  | RAFL09-16-F08 | At3g23820 / NAD-dependent epimerase/dehydratase family | |  |  |  |  |  | | --- | --- | --- | --- | --- | |  |  |  |  |  | | EC:4.2.1.47  3-beta-hydroxy-delta5-steroid dehydrogenase | | | | | | |
|  |  | RAFL09-07-D12 | At3g23820 / NAD-dependent epimerase/dehydratase family | |  |  |  |  |  | | --- | --- | --- | --- | --- | |  |  |  |  |  | | EC:4.2.1.47  3-beta-hydroxy-delta5-steroid dehydrogenase | | | | | | |
|  | Cluster:3-1 | |  |  | 3 | 213 | 6 | 4441 | 0.0066909385 | 0.03345469 | 5 |
|  |  | RAFL05-07-A06 | At4g33360 / terpene cyclase/mutase-related | |  |  |  |  |  | | --- | --- | --- | --- | --- | |  |  |  |  |  | | EC:4.2.1.47  3-beta-hydroxy-delta5-steroid dehydrogenase | | | | | | |
|  |  | RAFL08-14-D02 | At2g39770 / GDP-mannose pyrophosphorylase | |  |  |  |  |  | | --- | --- | --- | --- | --- | |  |  |  |  |  | | EC:2.7.7.22  unknown | | | | | | |
|  |  | RAFL08-13-A07 | At2g45790 / phosphomannomutase -related | |  |  |  |  |  | | --- | --- | --- | --- | --- | |  |  |  |  |  | | EC:5.4.2.8  phosphomannomutase | | | | | | |
| degradation of short-chain fatty acids | | |  |  | A | B | C | D | P | P' | N |
|  | Cluster:3-1 | |  |  | 1 | 215 | 0 | 4447 | 0.04632211 | 0.04632211 | 1 |
|  |  | RAFL08-11-A03 | At5g48230 / acetyl-CoA C-acetyltransferase (acetoacetyl-coenzyme A thiolase), putative | |  |  |  |  |  | | --- | --- | --- | --- | --- | |  |  |  |  |  | | EC:2.3.1.9  acetyl-CoA C-acetyltransferase | | | | | | |
| non-oxidative branch of the pentose phosphate pathway | | |  |  | A | B | C | D | P | P' | N |
|  | Cluster:3-0 | |  |  | 2 | 231 | 5 | 4425 | 0.044206142 | 0.26523685 | 6 |
|  |  | RAFL05-21-O08 | At5g61410 / ribulose-5-phosphate-3-epimerase | |  |  |  |  |  | | --- | --- | --- | --- | --- | |  |  |  |  |  | | EC:5.1.3.1  ribulose-phosphate 3-epimerase | | | | | | |
|  |  | RAFL11-02-F16 | At3g01850 / D-ribulose-5-phosphate 3-epimerase -related | |  |  |  |  |  | | --- | --- | --- | --- | --- | |  |  |  |  |  | | EC:5.1.3.1  ribulose-phosphate 3-epimerase | | | | | | |
| de novo biosynthesis of purine nucleotides I | | |  |  | A | B | C | D | P | P' | N |
|  | Cluster:2-1 | |  |  | 4 | 240 | 10 | 4409 | 0.0048282314 | 0.043454085 | 9 |
|  |  | RAFL04-17-H07 | At3g57610 / adenylosuccinate synthetase | |  |  |  |  |  | | --- | --- | --- | --- | --- | |  |  |  |  |  | | EC:6.3.4.4  adenylosuccinate synthase | | | | | | |
|  |  | RAFL04-13-M20 | At4g11010 / nucleoside diphosphate kinase 3 (ndpk3) | |  |  |  |  |  | | --- | --- | --- | --- | --- | |  |  |  |  |  | | EC:2.7.4.6  nucleoside-diphosphate kinase | | | | | | |
|  |  | RAFL04-18-P17 | At5g35170 / adenylate kinase -related protein | |  |  |  |  |  | | --- | --- | --- | --- | --- | |  |  |  |  |  | | EC:2.7.4.3  adenylate kinase | | | | | | |
|  |  | RAFL04-12-O11 | At3g27740 / carbamoyl-phosphate synthase (glutamine-hydrolyzing) (glutamine-dependent carbamoyl-phosphate synthase) small subunit | |  |  |  |  |  | | --- | --- | --- | --- | --- | |  |  |  |  |  | | EC:6.3.5.2  GMP synthase (glutamine-hydrolyzing) | | | | | | |
| chlorophyll biosynthesis | | |  |  | A | B | C | D | P | P' | N |
|  | Cluster:3-2 | |  |  | 4 | 33 | 11 | 4615 | 4.3046944E-6 | 3.4437555E-5 | 8 |
|  |  | RAFL09-06-N12 | At3g23810 / S-adenosyl-L-homocysteinas -related | |  |  |  |  |  | | --- | --- | --- | --- | --- | |  |  |  |  |  | | EC:4.3.1.8  adenosylhomocysteinase | | | | | | |
|  |  | RAFL07-09-L01 | At3g23810 / S-adenosyl-L-homocysteinas -related | |  |  |  |  |  | | --- | --- | --- | --- | --- | |  |  |  |  |  | | EC:4.3.1.8  adenosylhomocysteinase | | | | | | |
|  |  | RAFL09-13-P13 | At3g23810 / S-adenosyl-L-homocysteinas -related | |  |  |  |  |  | | --- | --- | --- | --- | --- | |  |  |  |  |  | | EC:4.3.1.8  adenosylhomocysteinase | | | | | | |
|  |  | RAFL09-10-M18 | At3g23810 / S-adenosyl-L-homocysteinas -related | |  |  |  |  |  | | --- | --- | --- | --- | --- | |  |  |  |  |  | | EC:4.3.1.8  adenosylhomocysteinase | | | | | | |
|  | Cluster:2-1 | |  |  | 3 | 241 | 12 | 4407 | 0.040360164 | 0.3228813 | 8 |
|  |  | RAFL04-17-P12 | At4g13940 / adenosylhomocysteinase | |  |  |  |  |  | | --- | --- | --- | --- | --- | |  |  |  |  |  | | EC:4.3.1.8  adenosylhomocysteinase | | | | | | |
|  |  | RAFL05-10-J03 | At1g69740 / porphobilinogen synthase (delta-aminolevulinic acid dehydratase), putative | |  |  |  |  |  | | --- | --- | --- | --- | --- | |  |  |  |  |  | | EC:4.2.1.24  porphobilinogen synthase | | | | | | |
|  |  | RAFL04-15-N01 | At1g69740 / porphobilinogen synthase (delta-aminolevulinic acid dehydratase), putative | |  |  |  |  |  | | --- | --- | --- | --- | --- | |  |  |  |  |  | | EC:4.2.1.24  porphobilinogen synthase | | | | | | |
| tRNA charging pathway | | |  |  | A | B | C | D | P | P' | N |
|  | Cluster:4-2 | |  |  | 4 | 131 | 13 | 4515 | 0.0011926556 | 0.010733901 | 9 |
|  |  | RAFL04-15-H14 | At4g26870 / aspartate-tRNA ligase (aspartyl-tRNA synthetase), putative | |  |  |  |  |  | | --- | --- | --- | --- | --- | |  |  |  |  |  | | EC:6.1.1.12 ,EC:6.1.1.22 ,EC:6.1.1.20 ,EC:6.1.1.6  asparagine-tRNA ligase  lysine-tRNA ligase  phenylalanine-tRNA ligase | | | | | | |
|  |  | RAFL04-19-C07 | At3g62120 / multifunctional aminoacyl-tRNA ligase-related protein | |  |  |  |  |  | | --- | --- | --- | --- | --- | |  |  |  |  |  | | EC:6.1.1.3 ,EC:6.1.1.15  threonine-tRNA ligase  glycine-tRNA ligase | | | | | | |
|  |  | RAFL05-08-P23 | At3g59980 / expressed protein | |  |  |  |  |  | | --- | --- | --- | --- | --- | |  |  |  |  |  | | EC:6.1.1.10 ,EC:6.1.1.20  isoleucine-tRNA ligase//leucine-tRNA ligase  phenylalanine-tRNA ligase | | | | | | |
|  |  | RAFL02-09-F04 | At5g26830 / threonyl-tRNA synthetase | |  |  |  |  |  | | --- | --- | --- | --- | --- | |  |  |  |  |  | | EC:6.1.1.3 ,EC:6.1.1.15 ,EC:6.1.1.14  threonine-tRNA ligase  glycine-tRNA ligase | | | | | | |
|  | Cluster:3-1 | |  |  | 3 | 213 | 14 | 4433 | 0.041229032 | 0.3710613 | 9 |
|  |  | RAFL07-14-N04 | At2g25840 / trytophanyl-tRNA synthetase -related | |  |  |  |  |  | | --- | --- | --- | --- | --- | |  |  |  |  |  | | EC:6.1.1.2  tryptophan-tRNA ligase | | | | | | |
|  |  | RAFL09-09-E22 | At4g39280 / phenylalanyl-trna synthetase - like protein | |  |  |  |  |  | | --- | --- | --- | --- | --- | |  |  |  |  |  | | EC:6.1.1.20  phenylalanine-tRNA ligase | | | | | | |
|  |  | RAFL04-16-L11 | At1g29880 / glycyl tRNA synthetase -related | |  |  |  |  |  | | --- | --- | --- | --- | --- | |  |  |  |  |  | | EC:6.1.1.14  glycine-tRNA ligase | | | | | | |
| thiamine biosynthesis | | |  |  | A | B | C | D | P | P' | N |
|  | Cluster:0-2 | |  |  | 1 | 78 | 2 | 4582 | 0.049979966 | 0.1499399 | 3 |
|  |  | RAFL04-15-A04 | At4g15560 / DEF (CLA1) protein | |  |  |  |  |  | | --- | --- | --- | --- | --- | |  |  |  |  |  | | EC:4.1.3.37  1-deoxy-D-xylulose-5-phosphate synthase | | | | | | |
| aldoxime degradation | | |  |  | A | B | C | D | P | P' | N |
|  | Cluster:8-2 | |  |  | 1 | 61 | 2 | 4599 | 0.0393688 | 0.1181064 | 3 |
|  |  | RAFL02-08-C20 | At4g08790 / nitrilase 1 like protein | |  |  |  |  |  | | --- | --- | --- | --- | --- | |  |  |  |  |  | | EC:4.2.1.84  hydrolase, acting on carbon-nitrogen (but not peptide) bonds | | | | | | |
| D-arabinose degradation II | | |  |  | A | B | C | D | P | P' | N |
|  | Cluster:3-0 | |  |  | 2 | 231 | 1 | 4429 | 0.007213332 | 0.014426664 | 2 |
|  |  | RAFL05-21-O08 | At5g61410 / ribulose-5-phosphate-3-epimerase | |  |  |  |  |  | | --- | --- | --- | --- | --- | |  |  |  |  |  | | EC:5.1.3.1  ribulose-phosphate 3-epimerase | | | | | | |
|  |  | RAFL11-02-F16 | At3g01850 / D-ribulose-5-phosphate 3-epimerase -related | |  |  |  |  |  | | --- | --- | --- | --- | --- | |  |  |  |  |  | | EC:5.1.3.1  ribulose-phosphate 3-epimerase | | | | | | |
|  | | | | | | | | | | | |
| Cluster:6-0 | | |  |  | A | B | C | D | P | P' | N |
|  | aerobic respiration -- electron donors reaction list | |  |  | 3 | 137 | 13 | 4510 | 0.011134108 | 0.07793876 | 7 |
|  |  | RAFL06-16-I03 | At5g09600 / expressed protein | |  |  |  |  |  | | --- | --- | --- | --- | --- | |  |  |  |  |  | | EC:1.3.5.1  succinate dehydrogenase (ubiquinone) | | | | | | |
|  |  | RAFL08-12-A05 | At5g11770 / NADH dehydrogenase (ubiquinone) | |  |  |  |  |  | | --- | --- | --- | --- | --- | |  |  |  |  |  | | EC:1.6.5.3  NADH dehydrogenase (ubiquinone) | | | | | | |
|  |  | RAFL05-17-L16 | At1g79010 / NADH dehydrogenase -related | |  |  |  |  |  | | --- | --- | --- | --- | --- | |  |  |  |  |  | | EC:1.6.5.3  NADH dehydrogenase (ubiquinone) | | | | | | |
|  | respiration (anaerobic)-- electron donors reaction list | |  |  | 2 | 138 | 10 | 4513 | 0.048507567 | 0.24253784 | 5 |
|  |  | RAFL08-12-A05 | At5g11770 / NADH dehydrogenase (ubiquinone) | |  |  |  |  |  | | --- | --- | --- | --- | --- | |  |  |  |  |  | | EC:1.6.5.3  NADH dehydrogenase (ubiquinone) | | | | | | |
|  |  | RAFL05-17-L16 | At1g79010 / NADH dehydrogenase -related | |  |  |  |  |  | | --- | --- | --- | --- | --- | |  |  |  |  |  | | EC:1.6.5.3  NADH dehydrogenase (ubiquinone) | | | | | | |
| Cluster:3-1 | | |  |  | A | B | C | D | P | P' | N |
|  | arginine biosynthesis I | |  |  | 4 | 212 | 2 | 4445 | 6.244364E-5 | 1.8733094E-4 | 3 |
|  |  | RAFL07-16-F16 | At3g20330 / aspartate carbamoyltransferase precursor (aspartate transcarbamylase) | |  |  |  |  |  | | --- | --- | --- | --- | --- | |  |  |  |  |  | | EC:2.1.3.3  ornithine carbamoyltransferase | | | | | | |
|  |  | RAFL07-18-A10 | At2g37500 / glutamate/ornithine acetyltransferase -related | |  |  |  |  |  | | --- | --- | --- | --- | --- | |  |  |  |  |  | | EC:2.3.1.35 ,EC:2.3.1.1  glutamate N-acetyltransferase  acetylglutamate kinase | | | | | | |
|  |  | RAFL07-08-L02 | At5g10920 / argininosuccinate lyase (AtArgH) | |  |  |  |  |  | | --- | --- | --- | --- | --- | |  |  |  |  |  | | EC:4.3.2.1  adenylosuccinate lyase | | | | | | |
|  |  | RAFL04-16-G24 | At1g80600 / acetylornithine aminotransferase, mitochondrial (acetylornithine transaminase/AOTA/ACOAT), putative | |  |  |  |  |  | | --- | --- | --- | --- | --- | |  |  |  |  |  | | EC:2.6.1.11  acetylornithine transaminase | | | | | | |
|  | cellulose biosynthesis | |  |  | 5 | 211 | 9 | 4438 | 2.892519E-4 | 0.0023140153 | 8 |
|  |  | RAFL05-02-P19 | At5g64740 / cellulose synthase, catalytic subunit, putative | |  |  |  |  |  | | --- | --- | --- | --- | --- | |  |  |  |  |  | | EC:2.4.1.12  cellulose synthase (UDP-forming) | | | | | | |
|  |  | RAFL05-19-M03 | At5g05170 / cellulose synthase, catalytic subunit (Ath-B) | |  |  |  |  |  | | --- | --- | --- | --- | --- | |  |  |  |  |  | | EC:2.4.1.12  cellulose synthase (UDP-forming) | | | | | | |
|  |  | RAFL09-10-K01 | At5g22740 / glycosyltransferase family 2 | |  |  |  |  |  | | --- | --- | --- | --- | --- | |  |  |  |  |  | | EC:2.4.1.12  cellulose synthase (UDP-forming) | | | | | | |
|  |  | RAFL04-19-E02 | At4g32410 / cellulose synthase, catalytic subunit, putative | |  |  |  |  |  | | --- | --- | --- | --- | --- | |  |  |  |  |  | | EC:2.4.1.12  cellulose synthase (UDP-forming) | | | | | | |
|  |  | RAFL05-02-G06 | At5g49720 / glycosyl hydrolase family 9 (endo-1,4-beta-glucanase) | |  |  |  |  |  | | --- | --- | --- | --- | --- | |  |  |  |  |  | | EC:3.2.1.4  1,4-b-D-glucan 4-glucanohydrolase | | | | | | |
|  | ascorbate biosynthesis | |  |  | 3 | 213 | 1 | 4446 | 3.7887847E-4 | 7.5775693E-4 | 2 |
|  |  | RAFL08-14-D02 | At2g39770 / GDP-mannose pyrophosphorylase | |  |  |  |  |  | | --- | --- | --- | --- | --- | |  |  |  |  |  | | EC:2.7.7.13  GDP-D-mannose pyrophosphorylase | | | | | | |
|  |  | RAFL08-13-A07 | At2g45790 / phosphomannomutase -related | |  |  |  |  |  | | --- | --- | --- | --- | --- | |  |  |  |  |  | | EC:5.4.2.8  phosphomannomutase | | | | | | |
|  |  | RAFL04-15-P16 | At5g28840 / NAD-dependent epimerase/dehydratase family | |  |  |  |  |  | | --- | --- | --- | --- | --- | |  |  |  |  |  | | EC:5.1.3.18  GDP-D-mannose-3,5-epimerase | | | | | | |
|  | serine biosynthesis | |  |  | 2 | 214 | 0 | 4447 | 0.0021362621 | 0.0021362621 | 1 |
|  |  | RAFL04-10-L08 | At4g34200 / D-3-phosphoglycerate dehydrogenase (3-PGDH), putative | |  |  |  |  |  | | --- | --- | --- | --- | --- | |  |  |  |  |  | | EC:1.1.1.95  phosphoglycerate dehydrogenase | | | | | | |
|  |  | RAFL04-18-D17 | At4g35630 / phosphoserine aminotransferase | |  |  |  |  |  | | --- | --- | --- | --- | --- | |  |  |  |  |  | | EC:2.6.1.52  triacylglycerol lipase | | | | | | |
|  | cysteine biosynthesis II | |  |  | 2 | 214 | 0 | 4447 | 0.0021362621 | 0.0021362621 | 1 |
|  |  | RAFL04-10-L08 | At4g34200 / D-3-phosphoglycerate dehydrogenase (3-PGDH), putative | |  |  |  |  |  | | --- | --- | --- | --- | --- | |  |  |  |  |  | | EC:1.1.1.95  phosphoglycerate dehydrogenase | | | | | | |
|  |  | RAFL04-18-D17 | At4g35630 / phosphoserine aminotransferase | |  |  |  |  |  | | --- | --- | --- | --- | --- | |  |  |  |  |  | | EC:2.6.1.52  triacylglycerol lipase | | | | | | |
|  | glycerol degradation II | |  |  | 6 | 210 | 26 | 4421 | 0.0030328825 | 0.045493238 | 15 |
|  |  | RAFL07-10-P13 | At1g09780 / 2,3-bisphosphoglycerate-independent phosphoglycerate mutase -related | |  |  |  |  |  | | --- | --- | --- | --- | --- | |  |  |  |  |  | | EC:5.4.2.1  2,3-bisphosphoglycerate-independent phosphoglycerate mutase | | | | | | |
|  |  | RAFL05-01-I24 | At3g55440 / triosephosphate isomerase, cytosolic, putative | |  |  |  |  |  | | --- | --- | --- | --- | --- | |  |  |  |  |  | | EC:5.3.1.1  triose-phosphate isomerase | | | | | | |
|  |  | RAFL05-21-G03 | At3g22960 / pyruvate kinase, putative | |  |  |  |  |  | | --- | --- | --- | --- | --- | |  |  |  |  |  | | EC:2.7.1.40  pyruvate kinase | | | | | | |
|  |  | RAFL04-13-O10 | At2g29560 / enolase (2-phospho-D-glycerate hydroylase) -related | |  |  |  |  |  | | --- | --- | --- | --- | --- | |  |  |  |  |  | | EC:4.2.1.11  phosphopyruvate hydratase | | | | | | |
|  |  | RAFL09-12-D13 | At5g52920 / pyruvate kinase, putative | |  |  |  |  |  | | --- | --- | --- | --- | --- | |  |  |  |  |  | | EC:2.7.1.40  pyruvate kinase | | | | | | |
|  |  | RAFL07-09-L18 | At2g40690 / glycerol-3-phosphate dehydrogenase | |  |  |  |  |  | | --- | --- | --- | --- | --- | |  |  |  |  |  | | EC:1.1.1.8  glycerol-3-phosphate dehydrogenase (NAD+) | | | | | | |
|  | GDP-mannose metabolism | |  |  | 2 | 214 | 1 | 4446 | 0.0062126224 | 0.012425245 | 2 |
|  |  | RAFL08-14-D02 | At2g39770 / GDP-mannose pyrophosphorylase | |  |  |  |  |  | | --- | --- | --- | --- | --- | |  |  |  |  |  | | EC:2.7.7.22  unknown | | | | | | |
|  |  | RAFL08-13-A07 | At2g45790 / phosphomannomutase -related | |  |  |  |  |  | | --- | --- | --- | --- | --- | |  |  |  |  |  | | EC:5.4.2.8  phosphomannomutase | | | | | | |
|  | non-phosphorylated glucose degradation | |  |  | 4 | 212 | 13 | 4434 | 0.006616924 | 0.06616924 | 10 |
|  |  | RAFL05-11-L02 | At4g25900 / aldose 1-epimerase family | |  |  |  |  |  | | --- | --- | --- | --- | --- | |  |  |  |  |  | | EC:5.1.3.3  aldose 1-epimerase | | | | | | |
|  |  | RAFL05-21-G03 | At3g22960 / pyruvate kinase, putative | |  |  |  |  |  | | --- | --- | --- | --- | --- | |  |  |  |  |  | | EC:2.7.1.40  pyruvate kinase | | | | | | |
|  |  | RAFL04-13-O10 | At2g29560 / enolase (2-phospho-D-glycerate hydroylase) -related | |  |  |  |  |  | | --- | --- | --- | --- | --- | |  |  |  |  |  | | EC:4.2.1.11  phosphopyruvate hydratase | | | | | | |
|  |  | RAFL09-12-D13 | At5g52920 / pyruvate kinase, putative | |  |  |  |  |  | | --- | --- | --- | --- | --- | |  |  |  |  |  | | EC:2.7.1.40  pyruvate kinase | | | | | | |
|  | GDP-D-rhamnose biosynthesis | |  |  | 3 | 213 | 6 | 4441 | 0.0066909385 | 0.03345469 | 5 |
|  |  | RAFL05-07-A06 | At4g33360 / terpene cyclase/mutase-related | |  |  |  |  |  | | --- | --- | --- | --- | --- | |  |  |  |  |  | | EC:4.2.1.47  3-beta-hydroxy-delta5-steroid dehydrogenase | | | | | | |
|  |  | RAFL08-14-D02 | At2g39770 / GDP-mannose pyrophosphorylase | |  |  |  |  |  | | --- | --- | --- | --- | --- | |  |  |  |  |  | | EC:2.7.7.22  unknown | | | | | | |
|  |  | RAFL08-13-A07 | At2g45790 / phosphomannomutase -related | |  |  |  |  |  | | --- | --- | --- | --- | --- | |  |  |  |  |  | | EC:5.4.2.8  phosphomannomutase | | | | | | |
|  | tryptophan biosynthesis | |  |  | 2 | 214 | 5 | 4442 | 0.03844968 | 0.1922484 | 5 |
|  |  | RAFL05-16-L22 | At5g48220 / indole-3-glycerol phosphate synthase (IGPS), putative | |  |  |  |  |  | | --- | --- | --- | --- | --- | |  |  |  |  |  | | EC:4.1.1.48  indole-3-glycerol-phosphate synthase | | | | | | |
|  |  | RAFL05-13-G12 | At1g07780 / phosphoribosylanthranilate isomerase (PAI1) | |  |  |  |  |  | | --- | --- | --- | --- | --- | |  |  |  |  |  | | EC:5.3.1.24  phosphoribosylanthranilate isomerase | | | | | | |
|  | sterol biosynthesis | |  |  | 2 | 214 | 5 | 4442 | 0.03844968 | 0.1922484 | 5 |
|  |  | RAFL11-10-O06 | At1g50430 / sterol delta-7 reductase (7-dehydrocholesterol reductase) (dwarf5) (DWF5) | |  |  |  |  |  | | --- | --- | --- | --- | --- | |  |  |  |  |  | | EC:1.3.1.21  sterol Δ<sup>7</sup> reductase | | | | | | |
|  |  | RAFL02-01-C03 | At1g76090 / S-adenosyl-methionine-sterol-C-methyltransferase -related | |  |  |  |  |  | | --- | --- | --- | --- | --- | |  |  |  |  |  | | EC:2.1.1.143  24-methylenelophenol-C24-methyltransferase | | | | | | |
|  | tRNA charging pathway | |  |  | 3 | 213 | 14 | 4433 | 0.041229032 | 0.3710613 | 9 |
|  |  | RAFL07-14-N04 | At2g25840 / trytophanyl-tRNA synthetase -related | |  |  |  |  |  | | --- | --- | --- | --- | --- | |  |  |  |  |  | | EC:6.1.1.2  tryptophan-tRNA ligase | | | | | | |
|  |  | RAFL09-09-E22 | At4g39280 / phenylalanyl-trna synthetase - like protein | |  |  |  |  |  | | --- | --- | --- | --- | --- | |  |  |  |  |  | | EC:6.1.1.20  phenylalanine-tRNA ligase | | | | | | |
|  |  | RAFL04-16-L11 | At1g29880 / glycyl tRNA synthetase -related | |  |  |  |  |  | | --- | --- | --- | --- | --- | |  |  |  |  |  | | EC:6.1.1.14  glycine-tRNA ligase | | | | | | |
|  | citrulline degradation | |  |  | 1 | 215 | 0 | 4447 | 0.04632211 | 0.04632211 | 1 |
|  |  | RAFL07-16-F16 | At3g20330 / aspartate carbamoyltransferase precursor (aspartate transcarbamylase) | |  |  |  |  |  | | --- | --- | --- | --- | --- | |  |  |  |  |  | | EC:2.1.3.3  ornithine carbamoyltransferase | | | | | | |
|  | tryptophan degradation III | |  |  | 1 | 215 | 0 | 4447 | 0.04632211 | 0.04632211 | 1 |
|  |  | RAFL08-11-A03 | At5g48230 / acetyl-CoA C-acetyltransferase (acetoacetyl-coenzyme A thiolase), putative | |  |  |  |  |  | | --- | --- | --- | --- | --- | |  |  |  |  |  | | EC:2.3.1.9  acetyl-CoA C-acetyltransferase | | | | | | |
|  | degradation of short-chain fatty acids | |  |  | 1 | 215 | 0 | 4447 | 0.04632211 | 0.04632211 | 1 |
|  |  | RAFL08-11-A03 | At5g48230 / acetyl-CoA C-acetyltransferase (acetoacetyl-coenzyme A thiolase), putative | |  |  |  |  |  | | --- | --- | --- | --- | --- | |  |  |  |  |  | | EC:2.3.1.9  acetyl-CoA C-acetyltransferase | | | | | | |
|  | pantothenate biosynthesis | |  |  | 1 | 215 | 0 | 4447 | 0.04632211 | 0.04632211 | 1 |
|  |  | RAFL07-09-L18 | At2g40690 / glycerol-3-phosphate dehydrogenase | |  |  |  |  |  | | --- | --- | --- | --- | --- | |  |  |  |  |  | | EC:1.1.1.169  2-dehydropantoate 2-reductase | | | | | | |
|  | glycolysis I | |  |  | 5 | 211 | 38 | 4409 | 0.04685452 | 0.7028178 | 15 |
|  |  | RAFL07-10-P13 | At1g09780 / 2,3-bisphosphoglycerate-independent phosphoglycerate mutase -related | |  |  |  |  |  | | --- | --- | --- | --- | --- | |  |  |  |  |  | | EC:5.4.2.1  2,3-bisphosphoglycerate-independent phosphoglycerate mutase | | | | | | |
|  |  | RAFL05-01-I24 | At3g55440 / triosephosphate isomerase, cytosolic, putative | |  |  |  |  |  | | --- | --- | --- | --- | --- | |  |  |  |  |  | | EC:5.3.1.1  triose-phosphate isomerase | | | | | | |
|  |  | RAFL05-21-G03 | At3g22960 / pyruvate kinase, putative | |  |  |  |  |  | | --- | --- | --- | --- | --- | |  |  |  |  |  | | EC:2.7.1.40  pyruvate kinase | | | | | | |
|  |  | RAFL04-13-O10 | At2g29560 / enolase (2-phospho-D-glycerate hydroylase) -related | |  |  |  |  |  | | --- | --- | --- | --- | --- | |  |  |  |  |  | | EC:4.2.1.11  phosphopyruvate hydratase | | | | | | |
|  |  | RAFL09-12-D13 | At5g52920 / pyruvate kinase, putative | |  |  |  |  |  | | --- | --- | --- | --- | --- | |  |  |  |  |  | | EC:2.7.1.40  pyruvate kinase | | | | | | |
|  | de novo biosynthesis of pyrimidine ribonucleotides | |  |  | 2 | 214 | 6 | 4441 | 0.049727194 | 0.24863598 | 5 |
|  |  | RAFL07-16-F16 | At3g20330 / aspartate carbamoyltransferase precursor (aspartate transcarbamylase) | |  |  |  |  |  | | --- | --- | --- | --- | --- | |  |  |  |  |  | | EC:2.1.3.2  unknown | | | | | | |
|  |  | RAFL05-01-N17 | At5g67220 / nitrogen regulation protein family (NIFR3) | |  |  |  |  |  | | --- | --- | --- | --- | --- | |  |  |  |  |  | | EC:1.3.3.1  dihydroorotate dehydrogenase | | | | | | |
| Cluster:8-1 | | |  |  | A | B | C | D | P | P' | N |
|  | flavonoid biosynthesis | |  |  | 2 | 160 | 1 | 4500 | 0.0035169823 | 0.0070339646 | 2 |
|  |  | RAFL04-16-O21 | At5g24530 / oxidoreductase, 2OG-Fe(II) oxygenase family | |  |  |  |  |  | | --- | --- | --- | --- | --- | |  |  |  |  |  | | EC:1.14.11.9  flavanone 3-hydroxylase||isopenicillin-N synthase | | | | | | |
|  |  | RAFL05-17-O13 | At3g21230 / 4-coumarate:CoA ligase (4-coumaroyl-CoA synthase) (4CL), putative | |  |  |  |  |  | | --- | --- | --- | --- | --- | |  |  |  |  |  | | EC:6.2.1.12  4-coumarate-CoA ligase | | | | | | |
|  | polyamine biosynthesis II | |  |  | 2 | 160 | 8 | 4493 | 0.044944704 | 0.31461293 | 7 |
|  |  | RAFL04-17-I02 | At2g16500 / arginine decarboxylase | |  |  |  |  |  | | --- | --- | --- | --- | --- | |  |  |  |  |  | | EC:4.1.1.19  arginine decarboxylase | | | | | | |
|  |  | RAFL05-09-J16 | At5g53120 / spermidine synthase | |  |  |  |  |  | | --- | --- | --- | --- | --- | |  |  |  |  |  | | EC:2.5.1.16  spermidine synthase | | | | | | |
|  | polyamine biosynthesis III | |  |  | 2 | 160 | 8 | 4493 | 0.044944704 | 0.31461293 | 7 |
|  |  | RAFL04-17-I02 | At2g16500 / arginine decarboxylase | |  |  |  |  |  | | --- | --- | --- | --- | --- | |  |  |  |  |  | | EC:4.1.1.19  arginine decarboxylase | | | | | | |
|  |  | RAFL05-09-J16 | At5g53120 / spermidine synthase | |  |  |  |  |  | | --- | --- | --- | --- | --- | |  |  |  |  |  | | EC:2.5.1.16  spermidine synthase | | | | | | |
| Cluster:1-1 | | |  |  | A | B | C | D | P | P' | N |
|  | peptidoglycan biosynthesis | |  |  | 2 | 103 | 1 | 4557 | 0.0014847745 | 0.002969549 | 2 |
|  |  | RAFL09-13-M20 | At1g31070 / UDP-N-acetylglucosamine pyrophosphorylase-related protein | |  |  |  |  |  | | --- | --- | --- | --- | --- | |  |  |  |  |  | | EC:2.7.7.23  UDP-N-acetylglucosamine diphosphorylase | | | | | | |
|  |  | RAFL09-18-H10 | At5g19220 / glucose-1-phosphate adenylyltransferase, large subunit 1, chloroplast (ADP-glucose pyrophosphorylase) (ADG2) (APL1) | |  |  |  |  |  | | --- | --- | --- | --- | --- | |  |  |  |  |  | | EC:2.7.7.23  UDP-N-acetylglucosamine diphosphorylase | | | | | | |
|  | UDP-N-acetylglucosamine biosynthesis | |  |  | 2 | 103 | 2 | 4556 | 0.0029258758 | 0.008777628 | 3 |
|  |  | RAFL09-13-M20 | At1g31070 / UDP-N-acetylglucosamine pyrophosphorylase-related protein | |  |  |  |  |  | | --- | --- | --- | --- | --- | |  |  |  |  |  | | EC:2.7.7.23  UDP-N-acetylglucosamine diphosphorylase | | | | | | |
|  |  | RAFL09-18-H10 | At5g19220 / glucose-1-phosphate adenylyltransferase, large subunit 1, chloroplast (ADP-glucose pyrophosphorylase) (ADG2) (APL1) | |  |  |  |  |  | | --- | --- | --- | --- | --- | |  |  |  |  |  | | EC:2.7.7.23  UDP-N-acetylglucosamine diphosphorylase | | | | | | |
|  | riboflavin and FMN and FAD biosynthesis | |  |  | 1 | 104 | 1 | 4557 | 0.04453306 | 0.08906612 | 2 |
|  |  | RAFL08-12-F18 | At2g44050 / 6,7-dimethyl-8-ribityllumazine synthase precursor | |  |  |  |  |  | | --- | --- | --- | --- | --- | |  |  |  |  |  | | EC:2.5.1.9  riboflavin synthase | | | | | | |
| Cluster:5-2 | | |  |  | A | B | C | D | P | P' | N |
|  | nitrate assimilation pathway | |  |  | 3 | 123 | 8 | 4529 | 0.0027120202 | 0.021696161 | 8 |
|  |  | RAFL09-13-L09 | At1g37130 / nitrate reductase 2 (NR2) | |  |  |  |  |  | | --- | --- | --- | --- | --- | |  |  |  |  |  | | EC:1.7.1.1  nitrate reductase | | | | | | |
|  |  | RAFL09-11-J22 | At1g37130 / nitrate reductase 2 (NR2) | |  |  |  |  |  | | --- | --- | --- | --- | --- | |  |  |  |  |  | | EC:1.7.1.1  nitrate reductase | | | | | | |
|  |  | RAFL11-09-K10 | At1g37130 / nitrate reductase 2 (NR2) | |  |  |  |  |  | | --- | --- | --- | --- | --- | |  |  |  |  |  | | EC:1.7.1.1  nitrate reductase | | | | | | |
|  | starch degradation | |  |  | 2 | 124 | 5 | 4532 | 0.013917747 | 0.069588736 | 5 |
|  |  | RAFL05-03-E09 | At5g64860 / glycosyl hydrolase family 77 (4-alpha-glucanotransferase) | |  |  |  |  |  | | --- | --- | --- | --- | --- | |  |  |  |  |  | | EC:2.4.1.25  disproportionating enzyme | | | | | | |
|  |  | RAFL09-14-L23 | At5g11720 / glycosyl hydrolase family 31 | |  |  |  |  |  | | --- | --- | --- | --- | --- | |  |  |  |  |  | | EC:3.2.1.20  unknown | | | | | | |
|  | dTDP-rhamnose biosynthesis | |  |  | 2 | 124 | 7 | 4530 | 0.023029873 | 0.13817924 | 6 |
|  |  | RAFL04-09-G05 | At1g50450 / expressed protein | |  |  |  |  |  | | --- | --- | --- | --- | --- | |  |  |  |  |  | | EC:4.2.1.46  dTDP-glucose 4,6-dehydratase | | | | | | |
|  |  | RAFL07-11-C21 | At4g30440 / nucleotide sugar epimerase family | |  |  |  |  |  | | --- | --- | --- | --- | --- | |  |  |  |  |  | | EC:4.2.1.46  dTDP-glucose 4,6-dehydratase | | | | | | |
| Cluster:2-0 | | |  |  | A | B | C | D | P | P' | N |
|  | cysteine biosynthesis I | |  |  | 2 | 148 | 4 | 4509 | 0.014161414 | 0.07080707 | 5 |
|  |  | RAFL05-10-D20 | At3g59760 / cysteine synthase, mitochondrial (O-acetylserine (thiol)-lyase/O-acetylserine sulfhydrylase), putative | |  |  |  |  |  | | --- | --- | --- | --- | --- | |  |  |  |  |  | | EC:4.2.99.8  cystathionine beta-synthase//lyase | | | | | | |
|  |  | RAFL07-18-N19 | At3g61440 / cysteine synthase (O-acetylserine (thiol)-lyase/O-acetylserine sulfhydrylase/AtcysC1) | |  |  |  |  |  | | --- | --- | --- | --- | --- | |  |  |  |  |  | | EC:4.2.99.8  cystathionine beta-synthase//lyase | | | | | | |
|  | de novo biosynthesis of pyrimidine ribonucleotides | |  |  | 2 | 148 | 6 | 4507 | 0.025339978 | 0.12669988 | 5 |
|  |  | RAFL04-20-D06 | At4g09320 / nucleoside-diphosphate kinase | |  |  |  |  |  | | --- | --- | --- | --- | --- | |  |  |  |  |  | | EC:2.7.4.6  nucleoside-diphosphate kinase | | | | | | |
|  |  | RAFL09-12-G17 | At1g29900 / carbamoylphosphate synthetase -related | |  |  |  |  |  | | --- | --- | --- | --- | --- | |  |  |  |  |  | | EC:6.3.5.5  carbamoyl-phosphate synthase | | | | | | |
|  | TCA cycle variation IV | |  |  | 3 | 147 | 21 | 4492 | 0.040271647 | 0.6040747 | 15 |
|  |  | RAFL09-06-L20 | At1g65930 / isocitrate dehydrogenase (NADP+), putative | |  |  |  |  |  | | --- | --- | --- | --- | --- | |  |  |  |  |  | | EC:1.1.1.42  3-isopropylmalate dehydrogenase | | | | | | |
|  |  | RAFL09-07-F20 | At1g65930 / isocitrate dehydrogenase (NADP+), putative | |  |  |  |  |  | | --- | --- | --- | --- | --- | |  |  |  |  |  | | EC:1.1.1.42  3-isopropylmalate dehydrogenase | | | | | | |
|  |  | RAFL09-11-A18 | At1g65930 / isocitrate dehydrogenase (NADP+), putative | |  |  |  |  |  | | --- | --- | --- | --- | --- | |  |  |  |  |  | | EC:1.1.1.42  3-isopropylmalate dehydrogenase | | | | | | |
| Cluster:6-1 | | |  |  | A | B | C | D | P | P' | N |
|  | isoleucine degradation III | |  |  | 2 | 313 | 1 | 4347 | 0.013038642 | 0.026077284 | 2 |
|  |  | RAFL07-15-A09 | At3g06860 / fatty acid multifunctional protein (AtMFP2) | |  |  |  |  |  | | --- | --- | --- | --- | --- | |  |  |  |  |  | | EC:4.2.1.17  enoyl-CoA hydratase | | | | | | |
|  |  | RAFL11-10-D22 | At5g43280 / enoyl-CoA hydratase/isomerase family | |  |  |  |  |  | | --- | --- | --- | --- | --- | |  |  |  |  |  | | EC:4.2.1.17  enoyl-CoA hydratase | | | | | | |
|  | isoleucine degradation I | |  |  | 2 | 313 | 1 | 4347 | 0.013038642 | 0.026077284 | 2 |
|  |  | RAFL07-15-A09 | At3g06860 / fatty acid multifunctional protein (AtMFP2) | |  |  |  |  |  | | --- | --- | --- | --- | --- | |  |  |  |  |  | | EC:4.2.1.17  enoyl-CoA hydratase | | | | | | |
|  |  | RAFL11-10-D22 | At5g43280 / enoyl-CoA hydratase/isomerase family | |  |  |  |  |  | | --- | --- | --- | --- | --- | |  |  |  |  |  | | EC:4.2.1.17  enoyl-CoA hydratase | | | | | | |
|  | fatty acid oxidation pathway | |  |  | 3 | 312 | 5 | 4343 | 0.013248402 | 0.07949041 | 6 |
|  |  | RAFL07-15-A09 | At3g06860 / fatty acid multifunctional protein (AtMFP2) | |  |  |  |  |  | | --- | --- | --- | --- | --- | |  |  |  |  |  | | EC:4.2.1.17  enoyl-CoA hydratase | | | | | | |
|  |  | RAFL11-10-D22 | At5g43280 / enoyl-CoA hydratase/isomerase family | |  |  |  |  |  | | --- | --- | --- | --- | --- | |  |  |  |  |  | | EC:4.2.1.17  enoyl-CoA hydratase | | | | | | |
|  |  | RAFL04-20-G12 | At3g05970 / AMP-binding protein, putative | |  |  |  |  |  | | --- | --- | --- | --- | --- | |  |  |  |  |  | | EC:6.2.1.3  long-chain-fatty-acid-CoA ligase | | | | | | |
|  | aerobic respiration -- electron donors reaction list | |  |  | 4 | 311 | 12 | 4336 | 0.019434473 | 0.13604131 | 7 |
|  |  | RAFL06-10-E05 | At1g16700 / NADH:ubiquinone oxidoreductase -related | |  |  |  |  |  | | --- | --- | --- | --- | --- | |  |  |  |  |  | | EC:1.6.5.3  NADH dehydrogenase (ubiquinone) | | | | | | |
|  |  | RAFL05-08-F21 | At3g03100 / expressed protein | |  |  |  |  |  | | --- | --- | --- | --- | --- | |  |  |  |  |  | | EC:1.6.5.3  NADH dehydrogenase (ubiquinone) | | | | | | |
|  |  | RAFL05-17-A04 | At5g09600 / expressed protein | |  |  |  |  |  | | --- | --- | --- | --- | --- | |  |  |  |  |  | | EC:1.3.5.1  succinate dehydrogenase (ubiquinone) | | | | | | |
|  |  | RAFL07-07-N09 | At5g37510 / NADH dehydrogenase (ubiquinone), mitochondrial, putative | |  |  |  |  |  | | --- | --- | --- | --- | --- | |  |  |  |  |  | | EC:1.6.5.3  NADH dehydrogenase (ubiquinone) | | | | | | |
|  | aspartate biosynthesis and degradation | |  |  | 2 | 313 | 2 | 4346 | 0.024916494 | 0.074749485 | 3 |
|  |  | RAFL04-17-L05 | At5g19550 / aspartate aminotransferase, cytoplasmic isozyme 1 (transaminase A/Asp2) | |  |  |  |  |  | | --- | --- | --- | --- | --- | |  |  |  |  |  | | EC:2.6.1.1  1-aminocyclopropane-1-carboxylate synthase | | | | | | |
|  |  | RAFL11-09-A12 | At5g19550 / aspartate aminotransferase, cytoplasmic isozyme 1 (transaminase A/Asp2) | |  |  |  |  |  | | --- | --- | --- | --- | --- | |  |  |  |  |  | | EC:2.6.1.1  1-aminocyclopropane-1-carboxylate synthase | | | | | | |
|  | aspartate degradation I | |  |  | 2 | 313 | 3 | 4345 | 0.039689664 | 0.11906899 | 3 |
|  |  | RAFL04-17-L05 | At5g19550 / aspartate aminotransferase, cytoplasmic isozyme 1 (transaminase A/Asp2) | |  |  |  |  |  | | --- | --- | --- | --- | --- | |  |  |  |  |  | | EC:2.6.1.1  aspartate transaminase | | | | | | |
|  |  | RAFL11-09-A12 | At5g19550 / aspartate aminotransferase, cytoplasmic isozyme 1 (transaminase A/Asp2) | |  |  |  |  |  | | --- | --- | --- | --- | --- | |  |  |  |  |  | | EC:2.6.1.1  aspartate transaminase | | | | | | |
|  | asparagine biosynthesis I | |  |  | 2 | 313 | 3 | 4345 | 0.039689664 | 0.15875866 | 4 |
|  |  | RAFL04-17-L05 | At5g19550 / aspartate aminotransferase, cytoplasmic isozyme 1 (transaminase A/Asp2) | |  |  |  |  |  | | --- | --- | --- | --- | --- | |  |  |  |  |  | | EC:2.6.1.1  aspartate transaminase | | | | | | |
|  |  | RAFL11-09-A12 | At5g19550 / aspartate aminotransferase, cytoplasmic isozyme 1 (transaminase A/Asp2) | |  |  |  |  |  | | --- | --- | --- | --- | --- | |  |  |  |  |  | | EC:2.6.1.1  aspartate transaminase | | | | | | |
|  | respiration (anaerobic)-- electron donors reaction list | |  |  | 3 | 312 | 9 | 4339 | 0.042557452 | 0.21278727 | 5 |
|  |  | RAFL06-10-E05 | At1g16700 / NADH:ubiquinone oxidoreductase -related | |  |  |  |  |  | | --- | --- | --- | --- | --- | |  |  |  |  |  | | EC:1.6.5.3  NADH dehydrogenase (ubiquinone) | | | | | | |
|  |  | RAFL05-08-F21 | At3g03100 / expressed protein | |  |  |  |  |  | | --- | --- | --- | --- | --- | |  |  |  |  |  | | EC:1.6.5.3  NADH dehydrogenase (ubiquinone) | | | | | | |
|  |  | RAFL07-07-N09 | At5g37510 / NADH dehydrogenase (ubiquinone), mitochondrial, putative | |  |  |  |  |  | | --- | --- | --- | --- | --- | |  |  |  |  |  | | EC:1.2.1.2 ,EC:1.6.5.3  NADH dehydrogenase (ubiquinone)  formate dehydrogenase | | | | | | |
| Cluster:0-0 | | |  |  | A | B | C | D | P | P' | N |
|  | Calvin cycle | |  |  | 15 | 21 | 37 | 4590 | 2.6605689E-21 | 2.9266258E-20 | 11 |
|  |  | RAFL06-14-C19 | At5g38410 / ribulose bisphosphate carboxylase small chain 3b precursor (RuBisCO small subunit 3b) (sp|P10798) | |  |  |  |  |  | | --- | --- | --- | --- | --- | |  |  |  |  |  | | EC:4.1.1.39  ribulose-bisphosphate carboxylase | | | | | | |
|  |  | RAFL06-10-O15 | At5g38420 / ribulose bisphosphate carboxylase small chain 2b precursor (RuBisCO small subunit 2b) (sp|P10797) | |  |  |  |  |  | | --- | --- | --- | --- | --- | |  |  |  |  |  | | EC:4.1.1.39  ribulose-bisphosphate carboxylase | | | | | | |
|  |  | RAFL06-13-H11 | At5g38430 / ribulose bisphosphate carboxylase small chain 1b precursor (RuBisCO small subunit 1b) (sp|P10796) | |  |  |  |  |  | | --- | --- | --- | --- | --- | |  |  |  |  |  | | EC:4.1.1.39  ribulose-bisphosphate carboxylase | | | | | | |
|  |  | RAFL09-09-L07 | At1g67090 / ribulose-bisphosphate carboxylase small unit -related | |  |  |  |  |  | | --- | --- | --- | --- | --- | |  |  |  |  |  | | EC:4.1.1.39  ribulose-bisphosphate carboxylase | | | | | | |
|  |  | RAFL06-14-L16 | At5g38430 / ribulose bisphosphate carboxylase small chain 1b precursor (RuBisCO small subunit 1b) (sp|P10796) | |  |  |  |  |  | | --- | --- | --- | --- | --- | |  |  |  |  |  | | EC:4.1.1.39  ribulose-bisphosphate carboxylase | | | | | | |
|  |  | RAFL04-15-J15 | At5g38410 / ribulose bisphosphate carboxylase small chain 3b precursor (RuBisCO small subunit 3b) (sp|P10798) | |  |  |  |  |  | | --- | --- | --- | --- | --- | |  |  |  |  |  | | EC:4.1.1.39  ribulose-bisphosphate carboxylase | | | | | | |
|  |  | RAFL06-08-L09 | At5g38420 / ribulose bisphosphate carboxylase small chain 2b precursor (RuBisCO small subunit 2b) (sp|P10797) | |  |  |  |  |  | | --- | --- | --- | --- | --- | |  |  |  |  |  | | EC:4.1.1.39  ribulose-bisphosphate carboxylase | | | | | | |
|  |  | RAFL07-14-L17 | At5g38420 / ribulose bisphosphate carboxylase small chain 2b precursor (RuBisCO small subunit 2b) (sp|P10797) | |  |  |  |  |  | | --- | --- | --- | --- | --- | |  |  |  |  |  | | EC:4.1.1.39  ribulose-bisphosphate carboxylase | | | | | | |
|  |  | RAFL06-14-C14 | At1g67090 / ribulose-bisphosphate carboxylase small unit -related | |  |  |  |  |  | | --- | --- | --- | --- | --- | |  |  |  |  |  | | EC:4.1.1.39  ribulose-bisphosphate carboxylase | | | | | | |
|  |  | RAFL08-17-J10 | At5g38420 / ribulose bisphosphate carboxylase small chain 2b precursor (RuBisCO small subunit 2b) (sp|P10797) | |  |  |  |  |  | | --- | --- | --- | --- | --- | |  |  |  |  |  | | EC:4.1.1.39  ribulose-bisphosphate carboxylase | | | | | | |
|  |  | RAFL06-07-I02 | At1g67090 / ribulose-bisphosphate carboxylase small unit -related | |  |  |  |  |  | | --- | --- | --- | --- | --- | |  |  |  |  |  | | EC:4.1.1.39  ribulose-bisphosphate carboxylase | | | | | | |
|  |  | RAFL11-03-H09 | At1g67090 / ribulose-bisphosphate carboxylase small unit -related | |  |  |  |  |  | | --- | --- | --- | --- | --- | |  |  |  |  |  | | EC:4.1.1.39  ribulose-bisphosphate carboxylase | | | | | | |
|  |  | RAFL09-16-C21 | At5g38410 / ribulose bisphosphate carboxylase small chain 3b precursor (RuBisCO small subunit 3b) (sp|P10798) | |  |  |  |  |  | | --- | --- | --- | --- | --- | |  |  |  |  |  | | EC:4.1.1.39  ribulose-bisphosphate carboxylase | | | | | | |
|  |  | RAFL09-09-K05 | At5g38410 / ribulose bisphosphate carboxylase small chain 3b precursor (RuBisCO small subunit 3b) (sp|P10798) | |  |  |  |  |  | | --- | --- | --- | --- | --- | |  |  |  |  |  | | EC:4.1.1.39  ribulose-bisphosphate carboxylase | | | | | | |
|  |  | RAFL07-11-L12 | At5g38420 / ribulose bisphosphate carboxylase small chain 2b precursor (RuBisCO small subunit 2b) (sp|P10797) | |  |  |  |  |  | | --- | --- | --- | --- | --- | |  |  |  |  |  | | EC:4.1.1.39  ribulose-bisphosphate carboxylase | | | | | | |
|  | cyanate degradation | |  |  | 2 | 34 | 3 | 4624 | 5.7119486E-4 | 0.0022847794 | 4 |
|  |  | RAFL06-13-B01 | At3g01500 / carbonic anhydrase, chloroplast precursor | |  |  |  |  |  | | --- | --- | --- | --- | --- | |  |  |  |  |  | | EC:4.2.1.1  carbonate dehydratase | | | | | | |
|  |  | RAFL06-11-K17 | At3g01500 / carbonic anhydrase, chloroplast precursor | |  |  |  |  |  | | --- | --- | --- | --- | --- | |  |  |  |  |  | | EC:4.2.1.1  carbonate dehydratase | | | | | | |
| Cluster:4-0 | | |  |  | A | B | C | D | P | P' | N |
|  | threonine degradation | |  |  | 3 | 237 | 7 | 4416 | 0.012340463 | 0.07404278 | 6 |
|  |  | RAFL04-17-J20 | At1g49670 / oxidoreductase, zinc-binding dehydrogenase family | |  |  |  |  |  | | --- | --- | --- | --- | --- | |  |  |  |  |  | | EC:1.1.1.103  L-threonine 3-dehydrogenase//alcohol dehydrogenase, zinc-dependent | | | | | | |
|  |  | RAFL05-19-C06 | At3g56460 / oxidoreductase, zinc-binding dehydrogenase family | |  |  |  |  |  | | --- | --- | --- | --- | --- | |  |  |  |  |  | | EC:1.1.1.103  L-threonine 3-dehydrogenase//alcohol dehydrogenase, zinc-dependent | | | | | | |
|  |  | RAFL04-18-H23 | At1g08110 / glyoxalase I, putative (lactoylglutathione lyase) | |  |  |  |  |  | | --- | --- | --- | --- | --- | |  |  |  |  |  | | EC:4.4.1.5  4-hydroxyphenylpyruvate dioxygenase | | | | | | |
|  | isoleucine biosynthesis I | |  |  | 2 | 238 | 5 | 4418 | 0.046670057 | 0.23335029 | 5 |
|  |  | RAFL06-16-L13 | At5g54960 / pyruvate decarboxylase (gb|AAB16855.1) | |  |  |  |  |  | | --- | --- | --- | --- | --- | |  |  |  |  |  | | EC:4.1.3.18  acetolactate synthase | | | | | | |
|  |  | RAFL09-18-P18 | At3g48560 / acetolactate synthase | |  |  |  |  |  | | --- | --- | --- | --- | --- | |  |  |  |  |  | | EC:4.1.3.18  acetolactate synthase | | | | | | |
|  | valine biosynthesis | |  |  | 2 | 238 | 5 | 4418 | 0.046670057 | 0.23335029 | 5 |
|  |  | RAFL06-16-L13 | At5g54960 / pyruvate decarboxylase (gb|AAB16855.1) | |  |  |  |  |  | | --- | --- | --- | --- | --- | |  |  |  |  |  | | EC:4.1.3.18  acetolactate synthase | | | | | | |
|  |  | RAFL09-18-P18 | At3g48560 / acetolactate synthase | |  |  |  |  |  | | --- | --- | --- | --- | --- | |  |  |  |  |  | | EC:4.1.3.18  acetolactate synthase | | | | | | |
| Cluster:9-1 | | |  |  | A | B | C | D | P | P' | N |
|  | homocysteine and cysteine interconversion | |  |  | 2 | 94 | 0 | 4567 | 4.1952432E-4 | 4.1952432E-4 | 1 |
|  |  | RAFL05-18-H15 | At1g64660 / methionine/cystathionine gamma lyase -related | |  |  |  |  |  | | --- | --- | --- | --- | --- | |  |  |  |  |  | | EC:4.4.1.8  cystathionine beta-lyase | | | | | | |
|  |  | RAFL11-02-N11 | At1g64660 / methionine/cystathionine gamma lyase -related | |  |  |  |  |  | | --- | --- | --- | --- | --- | |  |  |  |  |  | | EC:4.4.1.8  cystathionine beta-lyase | | | | | | |
|  | methionine degradation II | |  |  | 2 | 94 | 0 | 4567 | 4.1952432E-4 | 4.1952432E-4 | 1 |
|  |  | RAFL05-18-H15 | At1g64660 / methionine/cystathionine gamma lyase -related | |  |  |  |  |  | | --- | --- | --- | --- | --- | |  |  |  |  |  | | EC:4.4.1.11  1-aminocyclopropane-1-carboxylate synthase | | | | | | |
|  |  | RAFL11-02-N11 | At1g64660 / methionine/cystathionine gamma lyase -related | |  |  |  |  |  | | --- | --- | --- | --- | --- | |  |  |  |  |  | | EC:4.4.1.11  1-aminocyclopropane-1-carboxylate synthase | | | | | | |
|  | vitamin E biosynthesis | |  |  | 2 | 94 | 4 | 4563 | 0.005961956 | 0.023847824 | 4 |
|  |  | RAFL11-09-O05 | At1g06570 / 4-hydroxyphenylpyruvate dioxygenase (HPD) | |  |  |  |  |  | | --- | --- | --- | --- | --- | |  |  |  |  |  | | EC:1.13.11.27  4-hydroxyphenylpyruvate dioxygenase | | | | | | |
|  |  | RAFL11-12-C18 | At1g06570 / 4-hydroxyphenylpyruvate dioxygenase (HPD) | |  |  |  |  |  | | --- | --- | --- | --- | --- | |  |  |  |  |  | | EC:1.13.11.27  4-hydroxyphenylpyruvate dioxygenase | | | | | | |
|  | plastoquinone biosynthesis | |  |  | 2 | 94 | 4 | 4563 | 0.005961956 | 0.023847824 | 4 |
|  |  | RAFL11-09-O05 | At1g06570 / 4-hydroxyphenylpyruvate dioxygenase (HPD) | |  |  |  |  |  | | --- | --- | --- | --- | --- | |  |  |  |  |  | | EC:1.13.11.27  4-hydroxyphenylpyruvate dioxygenase | | | | | | |
|  |  | RAFL11-12-C18 | At1g06570 / 4-hydroxyphenylpyruvate dioxygenase (HPD) | |  |  |  |  |  | | --- | --- | --- | --- | --- | |  |  |  |  |  | | EC:1.13.11.27  4-hydroxyphenylpyruvate dioxygenase | | | | | | |
|  | triacylglycerol degradation | |  |  | 2 | 94 | 6 | 4561 | 0.0108338045 | 0.06500283 | 6 |
|  |  | RAFL08-09-J19 | At1g02660 / lipase (class 3) family | |  |  |  |  |  | | --- | --- | --- | --- | --- | |  |  |  |  |  | | EC:3.1.1.3  triacylglycerol lipase | | | | | | |
|  |  | RAFL05-18-O21 | At2g30550 / lipase (class 3) family | |  |  |  |  |  | | --- | --- | --- | --- | --- | |  |  |  |  |  | | EC:3.1.1.3  triacylglycerol lipase | | | | | | |
|  | methionine biosynthesis II | |  |  | 2 | 94 | 8 | 4559 | 0.016951112 | 0.06780445 | 4 |
|  |  | RAFL05-18-H15 | At1g64660 / methionine/cystathionine gamma lyase -related | |  |  |  |  |  | | --- | --- | --- | --- | --- | |  |  |  |  |  | | EC:4.4.1.8  cystathionine beta-lyase | | | | | | |
|  |  | RAFL11-02-N11 | At1g64660 / methionine/cystathionine gamma lyase -related | |  |  |  |  |  | | --- | --- | --- | --- | --- | |  |  |  |  |  | | EC:4.4.1.8  cystathionine beta-lyase | | | | | | |
|  | tyrosine degradation | |  |  | 2 | 94 | 10 | 4557 | 0.024206378 | 0.19365102 | 8 |
|  |  | RAFL11-09-O05 | At1g06570 / 4-hydroxyphenylpyruvate dioxygenase (HPD) | |  |  |  |  |  | | --- | --- | --- | --- | --- | |  |  |  |  |  | | EC:1.13.11.27  4-hydroxyphenylpyruvate dioxygenase | | | | | | |
|  |  | RAFL11-12-C18 | At1g06570 / 4-hydroxyphenylpyruvate dioxygenase (HPD) | |  |  |  |  |  | | --- | --- | --- | --- | --- | |  |  |  |  |  | | EC:1.13.11.27  4-hydroxyphenylpyruvate dioxygenase | | | | | | |
|  | phenylpropanoid biosynthesis | |  |  | 1 | 95 | 1 | 4566 | 0.040755685 | 0.08151137 | 2 |
|  |  | RAFL04-13-E17 | At5g20230 / plastocyanin-like domain containing protein | |  |  |  |  |  | | --- | --- | --- | --- | --- | |  |  |  |  |  | | EC:2.1.1.68  caffeic acid /5-hydroxyferulic acid o-methyltransferase | | | | | | |
| Cluster:5-1 | | |  |  | A | B | C | D | P | P' | N |
|  | tyrosine degradation | |  |  | 3 | 279 | 9 | 4372 | 0.03202454 | 0.25619632 | 8 |
|  |  | RAFL08-16-B22 | At1g11840 / glyoxalase I, putative (lactoylglutathione lyase) | |  |  |  |  |  | | --- | --- | --- | --- | --- | |  |  |  |  |  | | EC:1.13.11.27  4-hydroxyphenylpyruvate dioxygenase | | | | | | |
|  |  | RAFL09-07-G14 | At1g67280 / glyoxalase I, putative (lactoylglutathione lyase) | |  |  |  |  |  | | --- | --- | --- | --- | --- | |  |  |  |  |  | | EC:1.13.11.27  4-hydroxyphenylpyruvate dioxygenase | | | | | | |
|  |  | RAFL05-14-M18 | At1g12050 / fumarylacetoacetate hydrolase-related protein | |  |  |  |  |  | | --- | --- | --- | --- | --- | |  |  |  |  |  | | EC:3.7.1.2  fumarylacetoacetase | | | | | | |
|  | methylglyoxal pathway | |  |  | 2 | 280 | 4 | 4377 | 0.046489924 | 0.23244964 | 5 |
|  |  | RAFL08-16-B22 | At1g11840 / glyoxalase I, putative (lactoylglutathione lyase) | |  |  |  |  |  | | --- | --- | --- | --- | --- | |  |  |  |  |  | | EC:4.4.1.5  4-hydroxyphenylpyruvate dioxygenase | | | | | | |
|  |  | RAFL09-07-G14 | At1g67280 / glyoxalase I, putative (lactoylglutathione lyase) | |  |  |  |  |  | | --- | --- | --- | --- | --- | |  |  |  |  |  | | EC:4.4.1.5  4-hydroxyphenylpyruvate dioxygenase | | | | | | |
|  | vitamin E biosynthesis | |  |  | 2 | 280 | 4 | 4377 | 0.046489924 | 0.1859597 | 4 |
|  |  | RAFL08-16-B22 | At1g11840 / glyoxalase I, putative (lactoylglutathione lyase) | |  |  |  |  |  | | --- | --- | --- | --- | --- | |  |  |  |  |  | | EC:1.13.11.27  4-hydroxyphenylpyruvate dioxygenase | | | | | | |
|  |  | RAFL09-07-G14 | At1g67280 / glyoxalase I, putative (lactoylglutathione lyase) | |  |  |  |  |  | | --- | --- | --- | --- | --- | |  |  |  |  |  | | EC:1.13.11.27  4-hydroxyphenylpyruvate dioxygenase | | | | | | |
|  | plastoquinone biosynthesis | |  |  | 2 | 280 | 4 | 4377 | 0.046489924 | 0.1859597 | 4 |
|  |  | RAFL08-16-B22 | At1g11840 / glyoxalase I, putative (lactoylglutathione lyase) | |  |  |  |  |  | | --- | --- | --- | --- | --- | |  |  |  |  |  | | EC:1.13.11.27  4-hydroxyphenylpyruvate dioxygenase | | | | | | |
|  |  | RAFL09-07-G14 | At1g67280 / glyoxalase I, putative (lactoylglutathione lyase) | |  |  |  |  |  | | --- | --- | --- | --- | --- | |  |  |  |  |  | | EC:1.13.11.27  4-hydroxyphenylpyruvate dioxygenase | | | | | | |
|  | methylglyoxal degradation | |  |  | 2 | 280 | 4 | 4377 | 0.046489924 | 0.23244964 | 5 |
|  |  | RAFL08-16-B22 | At1g11840 / glyoxalase I, putative (lactoylglutathione lyase) | |  |  |  |  |  | | --- | --- | --- | --- | --- | |  |  |  |  |  | | EC:4.4.1.5  4-hydroxyphenylpyruvate dioxygenase | | | | | | |
|  |  | RAFL09-07-G14 | At1g67280 / glyoxalase I, putative (lactoylglutathione lyase) | |  |  |  |  |  | | --- | --- | --- | --- | --- | |  |  |  |  |  | | EC:4.4.1.5  4-hydroxyphenylpyruvate dioxygenase | | | | | | |
| Cluster:8-2 | | |  |  | A | B | C | D | P | P' | N |
|  | glycerol metabolism | |  |  | 1 | 61 | 2 | 4599 | 0.0393688 | 0.1181064 | 3 |
|  |  | RAFL06-11-H07 | At1g80460 / glycerol kinase -related | |  |  |  |  |  | | --- | --- | --- | --- | --- | |  |  |  |  |  | | EC:2.7.1.30  glycerol kinase | | | | | | |
|  | aldoxime degradation | |  |  | 1 | 61 | 2 | 4599 | 0.0393688 | 0.1181064 | 3 |
|  |  | RAFL02-08-C20 | At4g08790 / nitrilase 1 like protein | |  |  |  |  |  | | --- | --- | --- | --- | --- | |  |  |  |  |  | | EC:4.2.1.84  hydrolase, acting on carbon-nitrogen (but not peptide) bonds | | | | | | |
|  | acrylonitrile degradation | |  |  | 1 | 61 | 2 | 4599 | 0.0393688 | 0.1181064 | 3 |
|  |  | RAFL02-08-C20 | At4g08790 / nitrilase 1 like protein | |  |  |  |  |  | | --- | --- | --- | --- | --- | |  |  |  |  |  | | EC:4.2.1.84  hydrolase, acting on carbon-nitrogen (but not peptide) bonds | | | | | | |
|  | glycosylglyceride biosynthesis | |  |  | 1 | 61 | 2 | 4599 | 0.0393688 | 0.1181064 | 3 |
|  |  | RAFL04-20-J18 | At4g31780 / 1,2-diacylglycerol 3-beta-galactosyltransferase (UDP-galactose:diacylglycerol galactosyltransferase) (MGDG synthase) (MGD1), putative | |  |  |  |  |  | | --- | --- | --- | --- | --- | |  |  |  |  |  | | EC:2.4.1.46  UDP-galactose:DAG galactosyltransferase | | | | | | |
| Cluster:1-2 | | |  |  | A | B | C | D | P | P' | N |
|  | methionine biosynthesis II | |  |  | 5 | 169 | 5 | 4484 | 1.4791213E-5 | 5.9164853E-5 | 4 |
|  |  | RAFL06-12-D05 | At5g17920 / 5-methyltetrahydropteroyltriglutamate--homocysteine S-methyltransferase | |  |  |  |  |  | | --- | --- | --- | --- | --- | |  |  |  |  |  | | EC:2.1.1.14  5-methyltetrahydropteroyltriglutamate-homocysteine S-methyltransferase | | | | | | |
|  |  | RAFL11-01-K15 | At5g17920 / 5-methyltetrahydropteroyltriglutamate--homocysteine S-methyltransferase | |  |  |  |  |  | | --- | --- | --- | --- | --- | |  |  |  |  |  | | EC:2.1.1.14  5-methyltetrahydropteroyltriglutamate-homocysteine S-methyltransferase | | | | | | |
|  |  | RAFL09-11-C22 | At5g17920 / 5-methyltetrahydropteroyltriglutamate--homocysteine S-methyltransferase | |  |  |  |  |  | | --- | --- | --- | --- | --- | |  |  |  |  |  | | EC:2.1.1.14  5-methyltetrahydropteroyltriglutamate-homocysteine S-methyltransferase | | | | | | |
|  |  | RAFL11-06-L17 | At5g17920 / 5-methyltetrahydropteroyltriglutamate--homocysteine S-methyltransferase | |  |  |  |  |  | | --- | --- | --- | --- | --- | |  |  |  |  |  | | EC:2.1.1.14  5-methyltetrahydropteroyltriglutamate-homocysteine S-methyltransferase | | | | | | |
|  |  | RAFL09-10-C09 | At5g17920 / 5-methyltetrahydropteroyltriglutamate--homocysteine S-methyltransferase | |  |  |  |  |  | | --- | --- | --- | --- | --- | |  |  |  |  |  | | EC:2.1.1.14  5-methyltetrahydropteroyltriglutamate-homocysteine S-methyltransferase | | | | | | |
|  | methionine and <I>S</I>-adenosylmethionine synthesis | |  |  | 5 | 169 | 8 | 4481 | 6.892205E-5 | 4.135323E-4 | 6 |
|  |  | RAFL06-12-D05 | At5g17920 / 5-methyltetrahydropteroyltriglutamate--homocysteine S-methyltransferase | |  |  |  |  |  | | --- | --- | --- | --- | --- | |  |  |  |  |  | | EC:2.1.1.14  5-methyltetrahydropteroyltriglutamate-homocysteine S-methyltransferase | | | | | | |
|  |  | RAFL11-01-K15 | At5g17920 / 5-methyltetrahydropteroyltriglutamate--homocysteine S-methyltransferase | |  |  |  |  |  | | --- | --- | --- | --- | --- | |  |  |  |  |  | | EC:2.1.1.14  5-methyltetrahydropteroyltriglutamate-homocysteine S-methyltransferase | | | | | | |
|  |  | RAFL09-11-C22 | At5g17920 / 5-methyltetrahydropteroyltriglutamate--homocysteine S-methyltransferase | |  |  |  |  |  | | --- | --- | --- | --- | --- | |  |  |  |  |  | | EC:2.1.1.14  5-methyltetrahydropteroyltriglutamate-homocysteine S-methyltransferase | | | | | | |
|  |  | RAFL11-06-L17 | At5g17920 / 5-methyltetrahydropteroyltriglutamate--homocysteine S-methyltransferase | |  |  |  |  |  | | --- | --- | --- | --- | --- | |  |  |  |  |  | | EC:2.1.1.14  5-methyltetrahydropteroyltriglutamate-homocysteine S-methyltransferase | | | | | | |
|  |  | RAFL09-10-C09 | At5g17920 / 5-methyltetrahydropteroyltriglutamate--homocysteine S-methyltransferase | |  |  |  |  |  | | --- | --- | --- | --- | --- | |  |  |  |  |  | | EC:2.1.1.14  5-methyltetrahydropteroyltriglutamate-homocysteine S-methyltransferase | | | | | | |
|  | sulfate assimilation III | |  |  | 6 | 168 | 18 | 4471 | 1.9029251E-4 | 0.0020932176 | 11 |
|  |  | RAFL06-12-D05 | At5g17920 / 5-methyltetrahydropteroyltriglutamate--homocysteine S-methyltransferase | |  |  |  |  |  | | --- | --- | --- | --- | --- | |  |  |  |  |  | | EC:2.1.1.14  5-methyltetrahydropteroyltriglutamate-homocysteine S-methyltransferase | | | | | | |
|  |  | RAFL11-01-K15 | At5g17920 / 5-methyltetrahydropteroyltriglutamate--homocysteine S-methyltransferase | |  |  |  |  |  | | --- | --- | --- | --- | --- | |  |  |  |  |  | | EC:2.1.1.14  5-methyltetrahydropteroyltriglutamate-homocysteine S-methyltransferase | | | | | | |
|  |  | RAFL09-11-C22 | At5g17920 / 5-methyltetrahydropteroyltriglutamate--homocysteine S-methyltransferase | |  |  |  |  |  | | --- | --- | --- | --- | --- | |  |  |  |  |  | | EC:2.1.1.14  5-methyltetrahydropteroyltriglutamate-homocysteine S-methyltransferase | | | | | | |
|  |  | RAFL11-06-L17 | At5g17920 / 5-methyltetrahydropteroyltriglutamate--homocysteine S-methyltransferase | |  |  |  |  |  | | --- | --- | --- | --- | --- | |  |  |  |  |  | | EC:2.1.1.14  5-methyltetrahydropteroyltriglutamate-homocysteine S-methyltransferase | | | | | | |
|  |  | RAFL11-10-I18 | At1g19920 / sulfate adenylyltransferase | |  |  |  |  |  | | --- | --- | --- | --- | --- | |  |  |  |  |  | | EC:2.7.7.4  ATP sulfurylase | | | | | | |
|  |  | RAFL09-10-C09 | At5g17920 / 5-methyltetrahydropteroyltriglutamate--homocysteine S-methyltransferase | |  |  |  |  |  | | --- | --- | --- | --- | --- | |  |  |  |  |  | | EC:2.1.1.14  5-methyltetrahydropteroyltriglutamate-homocysteine S-methyltransferase | | | | | | |
|  | mannitol degradation | |  |  | 3 | 171 | 11 | 4478 | 0.013720686 | 0.068603426 | 5 |
|  |  | RAFL05-13-B09 | At5g03300 / pfkB type carbohydrate kinase protein family | |  |  |  |  |  | | --- | --- | --- | --- | --- | |  |  |  |  |  | | EC:2.7.1.11  6-phosphofructokinase | | | | | | |
|  |  | RAFL07-12-L15 | At1g12000 / pyrophosphate-fructose-6-phosphate 1-phosphotransferase -related | |  |  |  |  |  | | --- | --- | --- | --- | --- | |  |  |  |  |  | | EC:2.7.1.11  6-phosphofructokinase | | | | | | |
|  |  | RAFL07-15-F22 | At1g20950 / pyrophosphate-dependent phosphofructokinase alpha subunit -related | |  |  |  |  |  | | --- | --- | --- | --- | --- | |  |  |  |  |  | | EC:2.7.1.11  6-phosphofructokinase | | | | | | |
|  | sorbitol degradation | |  |  | 3 | 171 | 11 | 4478 | 0.013720686 | 0.068603426 | 5 |
|  |  | RAFL05-13-B09 | At5g03300 / pfkB type carbohydrate kinase protein family | |  |  |  |  |  | | --- | --- | --- | --- | --- | |  |  |  |  |  | | EC:2.7.1.11  6-phosphofructokinase | | | | | | |
|  |  | RAFL07-12-L15 | At1g12000 / pyrophosphate-fructose-6-phosphate 1-phosphotransferase -related | |  |  |  |  |  | | --- | --- | --- | --- | --- | |  |  |  |  |  | | EC:2.7.1.11  6-phosphofructokinase | | | | | | |
|  |  | RAFL07-15-F22 | At1g20950 / pyrophosphate-dependent phosphofructokinase alpha subunit -related | |  |  |  |  |  | | --- | --- | --- | --- | --- | |  |  |  |  |  | | EC:2.7.1.11  6-phosphofructokinase | | | | | | |
|  | superpathway of serine and glycine biosynthesis II | |  |  | 3 | 171 | 13 | 4476 | 0.019986069 | 0.17987463 | 9 |
|  |  | RAFL05-14-F03 | At1g23310 / alanine aminotransferase -related | |  |  |  |  |  | | --- | --- | --- | --- | --- | |  |  |  |  |  | | EC:2.6.1.4  alanine transaminase | | | | | | |
|  |  | RAFL09-11-K06 | At4g13930 / hydroxymethyltransferase | |  |  |  |  |  | | --- | --- | --- | --- | --- | |  |  |  |  |  | | EC:2.1.2.1  glycine hydroxymethyltransferase | | | | | | |
|  |  | RAFL07-12-E03 | At1g23310 / alanine aminotransferase -related | |  |  |  |  |  | | --- | --- | --- | --- | --- | |  |  |  |  |  | | EC:2.6.1.4  alanine transaminase | | | | | | |
|  | glycolysis I | |  |  | 5 | 169 | 38 | 4451 | 0.020866273 | 0.3129941 | 15 |
|  |  | RAFL05-13-B09 | At5g03300 / pfkB type carbohydrate kinase protein family | |  |  |  |  |  | | --- | --- | --- | --- | --- | |  |  |  |  |  | | EC:2.7.1.11  6-phosphofructokinase | | | | | | |
|  |  | RAFL04-09-G20 | At2g21170 / triosephosphate isomerase, chloroplast, putative | |  |  |  |  |  | | --- | --- | --- | --- | --- | |  |  |  |  |  | | EC:5.3.1.1  triose-phosphate isomerase | | | | | | |
|  |  | RAFL07-12-L15 | At1g12000 / pyrophosphate-fructose-6-phosphate 1-phosphotransferase -related | |  |  |  |  |  | | --- | --- | --- | --- | --- | |  |  |  |  |  | | EC:2.7.1.11  6-phosphofructokinase | | | | | | |
|  |  | RAFL07-15-F22 | At1g20950 / pyrophosphate-dependent phosphofructokinase alpha subunit -related | |  |  |  |  |  | | --- | --- | --- | --- | --- | |  |  |  |  |  | | EC:2.7.1.11  6-phosphofructokinase | | | | | | |
|  |  | RAFL06-13-M02 | At1g13440 / glyceraldehyde-3-phosphate dehydrogenase -related | |  |  |  |  |  | | --- | --- | --- | --- | --- | |  |  |  |  |  | | EC:1.2.1.12  glyceraldehyde-3-phosphate dehydrogenase | | | | | | |
|  | glycolysis IV | |  |  | 5 | 169 | 39 | 4450 | 0.0228522 | 0.3656352 | 16 |
|  |  | RAFL05-13-B09 | At5g03300 / pfkB type carbohydrate kinase protein family | |  |  |  |  |  | | --- | --- | --- | --- | --- | |  |  |  |  |  | | EC:2.7.1.11  6-phosphofructokinase | | | | | | |
|  |  | RAFL04-09-G20 | At2g21170 / triosephosphate isomerase, chloroplast, putative | |  |  |  |  |  | | --- | --- | --- | --- | --- | |  |  |  |  |  | | EC:5.3.1.1  triose-phosphate isomerase | | | | | | |
|  |  | RAFL07-12-L15 | At1g12000 / pyrophosphate-fructose-6-phosphate 1-phosphotransferase -related | |  |  |  |  |  | | --- | --- | --- | --- | --- | |  |  |  |  |  | | EC:2.7.1.90 ,EC:2.7.1.11  6-phosphofructokinase  unknown | | | | | | |
|  |  | RAFL07-15-F22 | At1g20950 / pyrophosphate-dependent phosphofructokinase alpha subunit -related | |  |  |  |  |  | | --- | --- | --- | --- | --- | |  |  |  |  |  | | EC:2.7.1.90 ,EC:2.7.1.11  6-phosphofructokinase  unknown | | | | | | |
|  |  | RAFL06-13-M02 | At1g13440 / glyceraldehyde-3-phosphate dehydrogenase -related | |  |  |  |  |  | | --- | --- | --- | --- | --- | |  |  |  |  |  | | EC:1.2.1.12  glyceraldehyde-3-phosphate dehydrogenase | | | | | | |
|  | sorbitol fermentation | |  |  | 5 | 169 | 39 | 4450 | 0.0228522 | 0.34278297 | 15 |
|  |  | RAFL05-13-B09 | At5g03300 / pfkB type carbohydrate kinase protein family | |  |  |  |  |  | | --- | --- | --- | --- | --- | |  |  |  |  |  | | EC:2.7.1.11  6-phosphofructokinase | | | | | | |
|  |  | RAFL04-09-G20 | At2g21170 / triosephosphate isomerase, chloroplast, putative | |  |  |  |  |  | | --- | --- | --- | --- | --- | |  |  |  |  |  | | EC:5.3.1.1  triose-phosphate isomerase | | | | | | |
|  |  | RAFL07-12-L15 | At1g12000 / pyrophosphate-fructose-6-phosphate 1-phosphotransferase -related | |  |  |  |  |  | | --- | --- | --- | --- | --- | |  |  |  |  |  | | EC:2.7.1.11  6-phosphofructokinase | | | | | | |
|  |  | RAFL07-15-F22 | At1g20950 / pyrophosphate-dependent phosphofructokinase alpha subunit -related | |  |  |  |  |  | | --- | --- | --- | --- | --- | |  |  |  |  |  | | EC:2.7.1.11  6-phosphofructokinase | | | | | | |
|  |  | RAFL06-13-M02 | At1g13440 / glyceraldehyde-3-phosphate dehydrogenase -related | |  |  |  |  |  | | --- | --- | --- | --- | --- | |  |  |  |  |  | | EC:1.2.1.12  glyceraldehyde-3-phosphate dehydrogenase | | | | | | |
|  | fructose degradation (anaerobic) | |  |  | 5 | 169 | 39 | 4450 | 0.0228522 | 0.34278297 | 15 |
|  |  | RAFL05-13-B09 | At5g03300 / pfkB type carbohydrate kinase protein family | |  |  |  |  |  | | --- | --- | --- | --- | --- | |  |  |  |  |  | | EC:2.7.1.11  6-phosphofructokinase | | | | | | |
|  |  | RAFL04-09-G20 | At2g21170 / triosephosphate isomerase, chloroplast, putative | |  |  |  |  |  | | --- | --- | --- | --- | --- | |  |  |  |  |  | | EC:5.3.1.1  triose-phosphate isomerase | | | | | | |
|  |  | RAFL07-12-L15 | At1g12000 / pyrophosphate-fructose-6-phosphate 1-phosphotransferase -related | |  |  |  |  |  | | --- | --- | --- | --- | --- | |  |  |  |  |  | | EC:2.7.1.11  6-phosphofructokinase | | | | | | |
|  |  | RAFL07-15-F22 | At1g20950 / pyrophosphate-dependent phosphofructokinase alpha subunit -related | |  |  |  |  |  | | --- | --- | --- | --- | --- | |  |  |  |  |  | | EC:2.7.1.11  6-phosphofructokinase | | | | | | |
|  |  | RAFL06-13-M02 | At1g13440 / glyceraldehyde-3-phosphate dehydrogenase -related | |  |  |  |  |  | | --- | --- | --- | --- | --- | |  |  |  |  |  | | EC:1.2.1.12  glyceraldehyde-3-phosphate dehydrogenase | | | | | | |
|  | alanine degradation III | |  |  | 2 | 172 | 5 | 4484 | 0.025693169 | 0.102772675 | 4 |
|  |  | RAFL05-14-F03 | At1g23310 / alanine aminotransferase -related | |  |  |  |  |  | | --- | --- | --- | --- | --- | |  |  |  |  |  | | EC:2.6.1.2  alanine transaminase | | | | | | |
|  |  | RAFL07-12-E03 | At1g23310 / alanine aminotransferase -related | |  |  |  |  |  | | --- | --- | --- | --- | --- | |  |  |  |  |  | | EC:2.6.1.2  alanine transaminase | | | | | | |
|  | alanine biosynthesis II | |  |  | 2 | 172 | 5 | 4484 | 0.025693169 | 0.102772675 | 4 |
|  |  | RAFL05-14-F03 | At1g23310 / alanine aminotransferase -related | |  |  |  |  |  | | --- | --- | --- | --- | --- | |  |  |  |  |  | | EC:2.6.1.2  alanine transaminase | | | | | | |
|  |  | RAFL07-12-E03 | At1g23310 / alanine aminotransferase -related | |  |  |  |  |  | | --- | --- | --- | --- | --- | |  |  |  |  |  | | EC:2.6.1.2  alanine transaminase | | | | | | |
|  | acetate fermentation | |  |  | 5 | 169 | 46 | 4443 | 0.04018175 | 0.803635 | 20 |
|  |  | RAFL05-13-B09 | At5g03300 / pfkB type carbohydrate kinase protein family | |  |  |  |  |  | | --- | --- | --- | --- | --- | |  |  |  |  |  | | EC:2.7.1.11  6-phosphofructokinase | | | | | | |
|  |  | RAFL04-09-G20 | At2g21170 / triosephosphate isomerase, chloroplast, putative | |  |  |  |  |  | | --- | --- | --- | --- | --- | |  |  |  |  |  | | EC:5.3.1.1  triose-phosphate isomerase | | | | | | |
|  |  | RAFL07-12-L15 | At1g12000 / pyrophosphate-fructose-6-phosphate 1-phosphotransferase -related | |  |  |  |  |  | | --- | --- | --- | --- | --- | |  |  |  |  |  | | EC:2.7.1.11  6-phosphofructokinase | | | | | | |
|  |  | RAFL07-15-F22 | At1g20950 / pyrophosphate-dependent phosphofructokinase alpha subunit -related | |  |  |  |  |  | | --- | --- | --- | --- | --- | |  |  |  |  |  | | EC:2.7.1.11  6-phosphofructokinase | | | | | | |
|  |  | RAFL06-13-M02 | At1g13440 / glyceraldehyde-3-phosphate dehydrogenase -related | |  |  |  |  |  | | --- | --- | --- | --- | --- | |  |  |  |  |  | | EC:1.2.1.12  glyceraldehyde-3-phosphate dehydrogenase | | | | | | |
|  | photorespiration | |  |  | 3 | 171 | 18 | 4471 | 0.0414438 | 0.45588177 | 11 |
|  |  | RAFL05-14-F03 | At1g23310 / alanine aminotransferase -related | |  |  |  |  |  | | --- | --- | --- | --- | --- | |  |  |  |  |  | | EC:2.6.1.4  alanine transaminase | | | | | | |
|  |  | RAFL09-11-K06 | At4g13930 / hydroxymethyltransferase | |  |  |  |  |  | | --- | --- | --- | --- | --- | |  |  |  |  |  | | EC:2.1.2.1  glycine hydroxymethyltransferase | | | | | | |
|  |  | RAFL07-12-E03 | At1g23310 / alanine aminotransferase -related | |  |  |  |  |  | | --- | --- | --- | --- | --- | |  |  |  |  |  | | EC:2.6.1.4  alanine transaminase | | | | | | |
| Cluster:3-0 | | |  |  | A | B | C | D | P | P' | N |
|  | TCA cycle variation II | |  |  | 5 | 228 | 4 | 4426 | 3.1898064E-5 | 1.5949032E-4 | 5 |
|  |  | RAFL07-17-M04 | At1g04410 / malate dehydrogenase, cytosolic, putative | |  |  |  |  |  | | --- | --- | --- | --- | --- | |  |  |  |  |  | | EC:1.1.1.37  malate dehydrogenase | | | | | | |
|  |  | RAFL06-07-J21 | At1g04410 / malate dehydrogenase, cytosolic, putative | |  |  |  |  |  | | --- | --- | --- | --- | --- | |  |  |  |  |  | | EC:1.1.1.37  malate dehydrogenase | | | | | | |
|  |  | RAFL05-21-P13 | At2g42600 / phosphoenolpyruvate carboxylase | |  |  |  |  |  | | --- | --- | --- | --- | --- | |  |  |  |  |  | | EC:4.1.1.31  phosphoenolpyruvate carboxylase | | | | | | |
|  |  | RAFL09-09-M02 | At3g47520 / malate dehydrogenase (NAD), chloroplast, putative | |  |  |  |  |  | | --- | --- | --- | --- | --- | |  |  |  |  |  | | EC:1.1.1.37  malate dehydrogenase | | | | | | |
|  |  | RAFL07-14-B18 | At2g47510 / fumarase -related | |  |  |  |  |  | | --- | --- | --- | --- | --- | |  |  |  |  |  | | EC:4.2.1.2  adenylosuccinate lyase | | | | | | |
|  | formaldehyde assimilation I (serine pathway) | |  |  | 7 | 226 | 16 | 4414 | 8.757928E-5 | 0.0010509514 | 12 |
|  |  | RAFL07-17-M04 | At1g04410 / malate dehydrogenase, cytosolic, putative | |  |  |  |  |  | | --- | --- | --- | --- | --- | |  |  |  |  |  | | EC:1.1.1.37  malate dehydrogenase | | | | | | |
|  |  | RAFL05-10-L06 | At2g13360 / alanine-glyoxylate aminotransferase | |  |  |  |  |  | | --- | --- | --- | --- | --- | |  |  |  |  |  | | EC:2.6.1.45  unknown | | | | | | |
|  |  | RAFL06-07-J21 | At1g04410 / malate dehydrogenase, cytosolic, putative | |  |  |  |  |  | | --- | --- | --- | --- | --- | |  |  |  |  |  | | EC:1.1.1.37  malate dehydrogenase | | | | | | |
|  |  | RAFL05-21-P13 | At2g42600 / phosphoenolpyruvate carboxylase | |  |  |  |  |  | | --- | --- | --- | --- | --- | |  |  |  |  |  | | EC:4.1.1.31  phosphoenolpyruvate carboxylase | | | | | | |
|  |  | RAFL09-09-M02 | At3g47520 / malate dehydrogenase (NAD), chloroplast, putative | |  |  |  |  |  | | --- | --- | --- | --- | --- | |  |  |  |  |  | | EC:1.1.1.37  malate dehydrogenase | | | | | | |
|  |  | RAFL05-18-J16 | At2g13360 / alanine-glyoxylate aminotransferase | |  |  |  |  |  | | --- | --- | --- | --- | --- | |  |  |  |  |  | | EC:2.6.1.45  unknown | | | | | | |
|  |  | RAFL05-02-A17 | At4g32520 / glycine hydroxymethyltransferase (EC 2.1.2.1) - like protein | |  |  |  |  |  | | --- | --- | --- | --- | --- | |  |  |  |  |  | | EC:2.1.2.1  glycine hydroxymethyltransferase | | | | | | |
|  | TCA cycle variation VII | |  |  | 5 | 228 | 6 | 4424 | 1.0765596E-4 | 7.535917E-4 | 7 |
|  |  | RAFL07-17-M04 | At1g04410 / malate dehydrogenase, cytosolic, putative | |  |  |  |  |  | | --- | --- | --- | --- | --- | |  |  |  |  |  | | EC:1.1.1.37  malate dehydrogenase | | | | | | |
|  |  | RAFL06-07-J21 | At1g04410 / malate dehydrogenase, cytosolic, putative | |  |  |  |  |  | | --- | --- | --- | --- | --- | |  |  |  |  |  | | EC:1.1.1.37  malate dehydrogenase | | | | | | |
|  |  | RAFL05-21-P13 | At2g42600 / phosphoenolpyruvate carboxylase | |  |  |  |  |  | | --- | --- | --- | --- | --- | |  |  |  |  |  | | EC:4.1.1.32  phosphoenolpyruvate carboxykinase | | | | | | |
|  |  | RAFL09-09-M02 | At3g47520 / malate dehydrogenase (NAD), chloroplast, putative | |  |  |  |  |  | | --- | --- | --- | --- | --- | |  |  |  |  |  | | EC:1.1.1.37  malate dehydrogenase | | | | | | |
|  |  | RAFL07-14-B18 | At2g47510 / fumarase -related | |  |  |  |  |  | | --- | --- | --- | --- | --- | |  |  |  |  |  | | EC:4.2.1.2  adenylosuccinate lyase | | | | | | |
|  | serine-isocitrate lyase pathway | |  |  | 7 | 226 | 24 | 4406 | 6.6646485E-4 | 0.011996367 | 18 |
|  |  | RAFL07-17-M04 | At1g04410 / malate dehydrogenase, cytosolic, putative | |  |  |  |  |  | | --- | --- | --- | --- | --- | |  |  |  |  |  | | EC:1.1.1.37  malate dehydrogenase | | | | | | |
|  |  | RAFL05-10-L06 | At2g13360 / alanine-glyoxylate aminotransferase | |  |  |  |  |  | | --- | --- | --- | --- | --- | |  |  |  |  |  | | EC:2.6.1.45  unknown | | | | | | |
|  |  | RAFL06-07-J21 | At1g04410 / malate dehydrogenase, cytosolic, putative | |  |  |  |  |  | | --- | --- | --- | --- | --- | |  |  |  |  |  | | EC:1.1.1.37  malate dehydrogenase | | | | | | |
|  |  | RAFL05-21-P13 | At2g42600 / phosphoenolpyruvate carboxylase | |  |  |  |  |  | | --- | --- | --- | --- | --- | |  |  |  |  |  | | EC:4.1.1.31  phosphoenolpyruvate carboxylase | | | | | | |
|  |  | RAFL09-09-M02 | At3g47520 / malate dehydrogenase (NAD), chloroplast, putative | |  |  |  |  |  | | --- | --- | --- | --- | --- | |  |  |  |  |  | | EC:1.1.1.37  malate dehydrogenase | | | | | | |
|  |  | RAFL05-18-J16 | At2g13360 / alanine-glyoxylate aminotransferase | |  |  |  |  |  | | --- | --- | --- | --- | --- | |  |  |  |  |  | | EC:2.6.1.45  unknown | | | | | | |
|  |  | RAFL05-02-A17 | At4g32520 / glycine hydroxymethyltransferase (EC 2.1.2.1) - like protein | |  |  |  |  |  | | --- | --- | --- | --- | --- | |  |  |  |  |  | | EC:2.1.2.1  glycine hydroxymethyltransferase | | | | | | |
|  | aspartate degradation II | |  |  | 4 | 229 | 6 | 4424 | 0.0010050355 | 0.0060302126 | 6 |
|  |  | RAFL07-17-M04 | At1g04410 / malate dehydrogenase, cytosolic, putative | |  |  |  |  |  | | --- | --- | --- | --- | --- | |  |  |  |  |  | | EC:1.1.1.37  malate dehydrogenase | | | | | | |
|  |  | RAFL06-07-J21 | At1g04410 / malate dehydrogenase, cytosolic, putative | |  |  |  |  |  | | --- | --- | --- | --- | --- | |  |  |  |  |  | | EC:1.1.1.37  malate dehydrogenase | | | | | | |
|  |  | RAFL09-09-M02 | At3g47520 / malate dehydrogenase (NAD), chloroplast, putative | |  |  |  |  |  | | --- | --- | --- | --- | --- | |  |  |  |  |  | | EC:1.1.1.37  malate dehydrogenase | | | | | | |
|  |  | RAFL09-07-B08 | At2g30970 / aspartate aminotransferase, mitochondrial (transaminase A/Asp1) | |  |  |  |  |  | | --- | --- | --- | --- | --- | |  |  |  |  |  | | EC:2.6.1.1  1-aminocyclopropane-1-carboxylate synthase | | | | | | |
|  | mixed acid fermentation | |  |  | 5 | 228 | 12 | 4418 | 0.0011257539 | 0.012383292 | 11 |
|  |  | RAFL07-17-M04 | At1g04410 / malate dehydrogenase, cytosolic, putative | |  |  |  |  |  | | --- | --- | --- | --- | --- | |  |  |  |  |  | | EC:1.1.1.37  malate dehydrogenase | | | | | | |
|  |  | RAFL06-07-J21 | At1g04410 / malate dehydrogenase, cytosolic, putative | |  |  |  |  |  | | --- | --- | --- | --- | --- | |  |  |  |  |  | | EC:1.1.1.37  malate dehydrogenase | | | | | | |
|  |  | RAFL05-21-P13 | At2g42600 / phosphoenolpyruvate carboxylase | |  |  |  |  |  | | --- | --- | --- | --- | --- | |  |  |  |  |  | | EC:4.1.1.31  phosphoenolpyruvate carboxylase | | | | | | |
|  |  | RAFL09-09-M02 | At3g47520 / malate dehydrogenase (NAD), chloroplast, putative | |  |  |  |  |  | | --- | --- | --- | --- | --- | |  |  |  |  |  | | EC:1.1.1.37  malate dehydrogenase | | | | | | |
|  |  | RAFL07-14-B18 | At2g47510 / fumarase -related | |  |  |  |  |  | | --- | --- | --- | --- | --- | |  |  |  |  |  | | EC:4.2.1.2  adenylosuccinate lyase | | | | | | |
|  | asparagine degradation I | |  |  | 3 | 230 | 3 | 4427 | 0.0022016333 | 0.0066048997 | 3 |
|  |  | RAFL05-21-P13 | At2g42600 / phosphoenolpyruvate carboxylase | |  |  |  |  |  | | --- | --- | --- | --- | --- | |  |  |  |  |  | | EC:4.1.1.32  phosphoenolpyruvate carboxylase | | | | | | |
|  |  | RAFL09-07-B08 | At2g30970 / aspartate aminotransferase, mitochondrial (transaminase A/Asp1) | |  |  |  |  |  | | --- | --- | --- | --- | --- | |  |  |  |  |  | | EC:2.6.1.1  1-aminocyclopropane-1-carboxylate synthase | | | | | | |
|  |  | RAFL04-20-H11 | At5g08100 / asparaginase | |  |  |  |  |  | | --- | --- | --- | --- | --- | |  |  |  |  |  | | EC:3.5.1.1  asparaginase | | | | | | |
|  | phenylalanine degradation I | |  |  | 5 | 228 | 15 | 4415 | 0.0024938595 | 0.027432455 | 11 |
|  |  | RAFL07-17-M04 | At1g04410 / malate dehydrogenase, cytosolic, putative | |  |  |  |  |  | | --- | --- | --- | --- | --- | |  |  |  |  |  | | EC:1.1.1.37  malate dehydrogenase | | | | | | |
|  |  | RAFL06-07-J21 | At1g04410 / malate dehydrogenase, cytosolic, putative | |  |  |  |  |  | | --- | --- | --- | --- | --- | |  |  |  |  |  | | EC:1.1.1.37  malate dehydrogenase | | | | | | |
|  |  | RAFL05-21-P13 | At2g42600 / phosphoenolpyruvate carboxylase | |  |  |  |  |  | | --- | --- | --- | --- | --- | |  |  |  |  |  | | EC:4.1.1.32  phosphoenolpyruvate carboxykinase | | | | | | |
|  |  | RAFL09-09-M02 | At3g47520 / malate dehydrogenase (NAD), chloroplast, putative | |  |  |  |  |  | | --- | --- | --- | --- | --- | |  |  |  |  |  | | EC:1.1.1.37  malate dehydrogenase | | | | | | |
|  |  | RAFL07-14-B18 | At2g47510 / fumarase -related | |  |  |  |  |  | | --- | --- | --- | --- | --- | |  |  |  |  |  | | EC:4.2.1.2  adenylosuccinate lyase | | | | | | |
|  | acetyl-CoA assimilation | |  |  | 5 | 228 | 16 | 4414 | 0.0031419091 | 0.04084482 | 13 |
|  |  | RAFL07-17-M04 | At1g04410 / malate dehydrogenase, cytosolic, putative | |  |  |  |  |  | | --- | --- | --- | --- | --- | |  |  |  |  |  | | EC:1.1.1.37  malate dehydrogenase | | | | | | |
|  |  | RAFL06-07-J21 | At1g04410 / malate dehydrogenase, cytosolic, putative | |  |  |  |  |  | | --- | --- | --- | --- | --- | |  |  |  |  |  | | EC:1.1.1.37  malate dehydrogenase | | | | | | |
|  |  | RAFL05-21-P13 | At2g42600 / phosphoenolpyruvate carboxylase | |  |  |  |  |  | | --- | --- | --- | --- | --- | |  |  |  |  |  | | EC:4.1.1.31  phosphoenolpyruvate carboxylase | | | | | | |
|  |  | RAFL09-09-M02 | At3g47520 / malate dehydrogenase (NAD), chloroplast, putative | |  |  |  |  |  | | --- | --- | --- | --- | --- | |  |  |  |  |  | | EC:1.1.1.37  malate dehydrogenase | | | | | | |
|  |  | RAFL07-14-B18 | At2g47510 / fumarase -related | |  |  |  |  |  | | --- | --- | --- | --- | --- | |  |  |  |  |  | | EC:4.2.1.2  adenylosuccinate lyase | | | | | | |
|  | TCA cycle variation IV | |  |  | 5 | 228 | 19 | 4411 | 0.0058060726 | 0.08709109 | 15 |
|  |  | RAFL07-17-M04 | At1g04410 / malate dehydrogenase, cytosolic, putative | |  |  |  |  |  | | --- | --- | --- | --- | --- | |  |  |  |  |  | | EC:1.1.1.37  malate dehydrogenase | | | | | | |
|  |  | RAFL06-07-J21 | At1g04410 / malate dehydrogenase, cytosolic, putative | |  |  |  |  |  | | --- | --- | --- | --- | --- | |  |  |  |  |  | | EC:1.1.1.37  malate dehydrogenase | | | | | | |
|  |  | RAFL05-21-P13 | At2g42600 / phosphoenolpyruvate carboxylase | |  |  |  |  |  | | --- | --- | --- | --- | --- | |  |  |  |  |  | | EC:4.1.1.32  phosphoenolpyruvate carboxykinase | | | | | | |
|  |  | RAFL09-09-M02 | At3g47520 / malate dehydrogenase (NAD), chloroplast, putative | |  |  |  |  |  | | --- | --- | --- | --- | --- | |  |  |  |  |  | | EC:1.1.1.37  malate dehydrogenase | | | | | | |
|  |  | RAFL07-14-B18 | At2g47510 / fumarase -related | |  |  |  |  |  | | --- | --- | --- | --- | --- | |  |  |  |  |  | | EC:4.2.1.2  adenylosuccinate lyase | | | | | | |
|  | ribitol degradation | |  |  | 2 | 231 | 1 | 4429 | 0.007213332 | 0.014426664 | 2 |
|  |  | RAFL05-21-O08 | At5g61410 / ribulose-5-phosphate-3-epimerase | |  |  |  |  |  | | --- | --- | --- | --- | --- | |  |  |  |  |  | | EC:5.1.3.1  ribulose-phosphate 3-epimerase | | | | | | |
|  |  | RAFL11-02-F16 | At3g01850 / D-ribulose-5-phosphate 3-epimerase -related | |  |  |  |  |  | | --- | --- | --- | --- | --- | |  |  |  |  |  | | EC:5.1.3.1  ribulose-phosphate 3-epimerase | | | | | | |
|  | D-arabinose degradation II | |  |  | 2 | 231 | 1 | 4429 | 0.007213332 | 0.014426664 | 2 |
|  |  | RAFL05-21-O08 | At5g61410 / ribulose-5-phosphate-3-epimerase | |  |  |  |  |  | | --- | --- | --- | --- | --- | |  |  |  |  |  | | EC:5.1.3.1  ribulose-phosphate 3-epimerase | | | | | | |
|  |  | RAFL11-02-F16 | At3g01850 / D-ribulose-5-phosphate 3-epimerase -related | |  |  |  |  |  | | --- | --- | --- | --- | --- | |  |  |  |  |  | | EC:5.1.3.1  ribulose-phosphate 3-epimerase | | | | | | |
|  | gluconeogenesis | |  |  | 7 | 226 | 40 | 4390 | 0.008070272 | 0.12912436 | 16 |
|  |  | RAFL07-17-M04 | At1g04410 / malate dehydrogenase, cytosolic, putative | |  |  |  |  |  | | --- | --- | --- | --- | --- | |  |  |  |  |  | | EC:1.1.1.37  malate dehydrogenase | | | | | | |
|  |  | RAFL06-07-J21 | At1g04410 / malate dehydrogenase, cytosolic, putative | |  |  |  |  |  | | --- | --- | --- | --- | --- | |  |  |  |  |  | | EC:1.1.1.37  malate dehydrogenase | | | | | | |
|  |  | RAFL05-21-P13 | At2g42600 / phosphoenolpyruvate carboxylase | |  |  |  |  |  | | --- | --- | --- | --- | --- | |  |  |  |  |  | | EC:4.1.1.49 ,EC:4.1.1.31  phosphoenolpyruvate carboxykinase (ATP)  phosphoenolpyruvate carboxylase | | | | | | |
|  |  | RAFL09-09-M02 | At3g47520 / malate dehydrogenase (NAD), chloroplast, putative | |  |  |  |  |  | | --- | --- | --- | --- | --- | |  |  |  |  |  | | EC:1.1.1.37  malate dehydrogenase | | | | | | |
|  |  | RAFL04-17-F02 | At2g01140 / fructose-bisphosphate aldolase, putative | |  |  |  |  |  | | --- | --- | --- | --- | --- | |  |  |  |  |  | | EC:4.1.2.13  fructose-bisphosphate aldolase | | | | | | |
|  |  | RAFL06-14-K01 | At3g04120 / glyceraldehyde-3-phosphate dehydrogenase C subunit (GapC) | |  |  |  |  |  | | --- | --- | --- | --- | --- | |  |  |  |  |  | | EC:1.2.1.12  glyceraldehyde-3-phosphate dehydrogenase | | | | | | |
|  |  | RAFL06-11-B16 | At3g52930 / fructose-bisphosphate aldolase, putative | |  |  |  |  |  | | --- | --- | --- | --- | --- | |  |  |  |  |  | | EC:4.1.2.13  fructose-bisphosphate aldolase | | | | | | |
|  | UDP-glucose conversion | |  |  | 5 | 228 | 21 | 4409 | 0.0082831625 | 0.1325306 | 16 |
|  |  | RAFL07-11-P20 | At4g01480 / inorganic phosphatase -related | |  |  |  |  |  | | --- | --- | --- | --- | --- | |  |  |  |  |  | | EC:3.6.1.1  unknown | | | | | | |
|  |  | RAFL05-16-O24 | At4g35250 / vestitone reductase-related | |  |  |  |  |  | | --- | --- | --- | --- | --- | |  |  |  |  |  | | EC:5.1.3.2  UDP-glucose 4-epimerase | | | | | | |
|  |  | RAFL09-15-M18 | At3g03250 / UDP-glucose pyrophosphorylase -related | |  |  |  |  |  | | --- | --- | --- | --- | --- | |  |  |  |  |  | | EC:2.7.7.9  UDP-N-acetylglucosamine diphosphorylase | | | | | | |
|  |  | RAFL07-18-D08 | At1g15690 / inorganic pyrophosphatase -related | |  |  |  |  |  | | --- | --- | --- | --- | --- | |  |  |  |  |  | | EC:3.6.1.1  unknown | | | | | | |
|  |  | RAFL05-18-A12 | At1g15690 / inorganic pyrophosphatase -related | |  |  |  |  |  | | --- | --- | --- | --- | --- | |  |  |  |  |  | | EC:3.6.1.1  unknown | | | | | | |
|  | TCA cycle variation VIII | |  |  | 5 | 228 | 23 | 4407 | 0.011410674 | 0.19398145 | 17 |
|  |  | RAFL07-17-M04 | At1g04410 / malate dehydrogenase, cytosolic, putative | |  |  |  |  |  | | --- | --- | --- | --- | --- | |  |  |  |  |  | | EC:1.1.1.37  malate dehydrogenase | | | | | | |
|  |  | RAFL06-07-J21 | At1g04410 / malate dehydrogenase, cytosolic, putative | |  |  |  |  |  | | --- | --- | --- | --- | --- | |  |  |  |  |  | | EC:1.1.1.37  malate dehydrogenase | | | | | | |
|  |  | RAFL05-21-P13 | At2g42600 / phosphoenolpyruvate carboxylase | |  |  |  |  |  | | --- | --- | --- | --- | --- | |  |  |  |  |  | | EC:4.1.1.32  phosphoenolpyruvate carboxykinase | | | | | | |
|  |  | RAFL09-09-M02 | At3g47520 / malate dehydrogenase (NAD), chloroplast, putative | |  |  |  |  |  | | --- | --- | --- | --- | --- | |  |  |  |  |  | | EC:1.1.1.37  malate dehydrogenase | | | | | | |
|  |  | RAFL07-14-B18 | At2g47510 / fumarase -related | |  |  |  |  |  | | --- | --- | --- | --- | --- | |  |  |  |  |  | | EC:4.2.1.2  adenylosuccinate lyase | | | | | | |
|  | aspartate degradation I | |  |  | 2 | 231 | 3 | 4427 | 0.02249133 | 0.067473985 | 3 |
|  |  | RAFL05-21-P13 | At2g42600 / phosphoenolpyruvate carboxylase | |  |  |  |  |  | | --- | --- | --- | --- | --- | |  |  |  |  |  | | EC:4.1.1.32  phosphoenolpyruvate carboxykinase | | | | | | |
|  |  | RAFL09-07-B08 | At2g30970 / aspartate aminotransferase, mitochondrial (transaminase A/Asp1) | |  |  |  |  |  | | --- | --- | --- | --- | --- | |  |  |  |  |  | | EC:2.6.1.1  aspartate transaminase | | | | | | |
|  | glyoxylate cycle | |  |  | 3 | 230 | 11 | 4419 | 0.029778728 | 0.327566 | 11 |
|  |  | RAFL07-17-M04 | At1g04410 / malate dehydrogenase, cytosolic, putative | |  |  |  |  |  | | --- | --- | --- | --- | --- | |  |  |  |  |  | | EC:1.1.1.37  malate dehydrogenase | | | | | | |
|  |  | RAFL06-07-J21 | At1g04410 / malate dehydrogenase, cytosolic, putative | |  |  |  |  |  | | --- | --- | --- | --- | --- | |  |  |  |  |  | | EC:1.1.1.37  malate dehydrogenase | | | | | | |
|  |  | RAFL09-09-M02 | At3g47520 / malate dehydrogenase (NAD), chloroplast, putative | |  |  |  |  |  | | --- | --- | --- | --- | --- | |  |  |  |  |  | | EC:1.1.1.37  malate dehydrogenase | | | | | | |
|  | glutamine degradation III | |  |  | 2 | 231 | 4 | 4426 | 0.032636177 | 0.1305447 | 4 |
|  |  | RAFL07-14-B18 | At2g47510 / fumarase -related | |  |  |  |  |  | | --- | --- | --- | --- | --- | |  |  |  |  |  | | EC:4.3.1.1  fumarate hydratase | | | | | | |
|  |  | RAFL09-07-B08 | At2g30970 / aspartate aminotransferase, mitochondrial (transaminase A/Asp1) | |  |  |  |  |  | | --- | --- | --- | --- | --- | |  |  |  |  |  | | EC:2.6.1.1  1-aminocyclopropane-1-carboxylate synthase | | | | | | |
|  | superpathway of serine and glycine biosynthesis II | |  |  | 3 | 230 | 13 | 4417 | 0.04257905 | 0.38321143 | 9 |
|  |  | RAFL05-10-L06 | At2g13360 / alanine-glyoxylate aminotransferase | |  |  |  |  |  | | --- | --- | --- | --- | --- | |  |  |  |  |  | | EC:2.6.1.4  alanine transaminase | | | | | | |
|  |  | RAFL05-18-J16 | At2g13360 / alanine-glyoxylate aminotransferase | |  |  |  |  |  | | --- | --- | --- | --- | --- | |  |  |  |  |  | | EC:2.6.1.4  alanine transaminase | | | | | | |
|  |  | RAFL05-02-A17 | At4g32520 / glycine hydroxymethyltransferase (EC 2.1.2.1) - like protein | |  |  |  |  |  | | --- | --- | --- | --- | --- | |  |  |  |  |  | | EC:2.1.2.1  glycine hydroxymethyltransferase | | | | | | |
|  | non-oxidative branch of the pentose phosphate pathway | |  |  | 2 | 231 | 5 | 4425 | 0.044206142 | 0.26523685 | 6 |
|  |  | RAFL05-21-O08 | At5g61410 / ribulose-5-phosphate-3-epimerase | |  |  |  |  |  | | --- | --- | --- | --- | --- | |  |  |  |  |  | | EC:5.1.3.1  ribulose-phosphate 3-epimerase | | | | | | |
|  |  | RAFL11-02-F16 | At3g01850 / D-ribulose-5-phosphate 3-epimerase -related | |  |  |  |  |  | | --- | --- | --- | --- | --- | |  |  |  |  |  | | EC:5.1.3.1  ribulose-phosphate 3-epimerase | | | | | | |
|  | starch degradation | |  |  | 2 | 231 | 5 | 4425 | 0.044206142 | 0.2210307 | 5 |
|  |  | RAFL08-08-F08 | At1g69830 / alpha-amylase (1,4-alpha-D-glucan glucanohydrolase), putative | |  |  |  |  |  | | --- | --- | --- | --- | --- | |  |  |  |  |  | | EC:3.2.1.1  alpha-amylase | | | | | | |
|  |  | RAFL08-09-A13 | At3g29320 / glucan phosphorylase, putative | |  |  |  |  |  | | --- | --- | --- | --- | --- | |  |  |  |  |  | | EC:2.4.1.1  starch phosphorylase | | | | | | |
|  | de novo biosynthesis of purine nucleotides II | |  |  | 2 | 231 | 5 | 4425 | 0.044206142 | 0.2210307 | 5 |
|  |  | RAFL07-14-B18 | At2g47510 / fumarase -related | |  |  |  |  |  | | --- | --- | --- | --- | --- | |  |  |  |  |  | | EC:4.3.2.2  adenylosuccinate lyase | | | | | | |
|  |  | RAFL05-15-O22 | At2g20420 / succinyl-CoA ligase beta subunit | |  |  |  |  |  | | --- | --- | --- | --- | --- | |  |  |  |  |  | | EC:6.3.4.13  phosphoribosylamine-glycine ligase | | | | | | |
|  | TCA cycle -- aerobic respiration | |  |  | 4 | 229 | 24 | 4406 | 0.048447587 | 0.87205654 | 18 |
|  |  | RAFL07-17-M04 | At1g04410 / malate dehydrogenase, cytosolic, putative | |  |  |  |  |  | | --- | --- | --- | --- | --- | |  |  |  |  |  | | EC:1.1.1.37 ,EC:1.1.99.16  malate dehydrogenase | | | | | | |
|  |  | RAFL06-07-J21 | At1g04410 / malate dehydrogenase, cytosolic, putative | |  |  |  |  |  | | --- | --- | --- | --- | --- | |  |  |  |  |  | | EC:1.1.1.37 ,EC:1.1.99.16  malate dehydrogenase | | | | | | |
|  |  | RAFL09-09-M02 | At3g47520 / malate dehydrogenase (NAD), chloroplast, putative | |  |  |  |  |  | | --- | --- | --- | --- | --- | |  |  |  |  |  | | EC:1.1.1.37 ,EC:1.1.99.16  malate dehydrogenase | | | | | | |
|  |  | RAFL07-14-B18 | At2g47510 / fumarase -related | |  |  |  |  |  | | --- | --- | --- | --- | --- | |  |  |  |  |  | | EC:4.2.1.2  adenylosuccinate lyase | | | | | | |
|  | NAD phosphorylation and dephosphorylation | |  |  | 1 | 232 | 0 | 4430 | 0.049967833 | 0.049967833 | 1 |
|  |  | RAFL05-10-H23 | At1g21640 / expressed protein | |  |  |  |  |  | | --- | --- | --- | --- | --- | |  |  |  |  |  | | EC:2.7.1.23  NAD+ kinase | | | | | | |
| Cluster:0-2 | | |  |  | A | B | C | D | P | P' | N |
|  | glycine degradation I | |  |  | 7 | 72 | 5 | 4579 | 2.2650871E-10 | 1.1325436E-9 | 5 |
|  |  | RAFL11-06-P03 | At4g33010 / glycine dehydrogenase (decarboxylating) (glycine decarboxylase/glycine cleavage system P-protein), putative | |  |  |  |  |  | | --- | --- | --- | --- | --- | |  |  |  |  |  | | EC:1.4.4.2  glycine dehydrogenase (decarboxylating) | | | | | | |
|  |  | RAFL07-10-O06 | At4g33010 / glycine dehydrogenase (decarboxylating) (glycine decarboxylase/glycine cleavage system P-protein), putative | |  |  |  |  |  | | --- | --- | --- | --- | --- | |  |  |  |  |  | | EC:1.4.4.2  glycine dehydrogenase (decarboxylating) | | | | | | |
|  |  | RAFL09-06-E16 | At2g26080 / glycine dehydrogenase (decarboxylating) (glycine decarboxylase/glycine cleavage system P-protein), putative | |  |  |  |  |  | | --- | --- | --- | --- | --- | |  |  |  |  |  | | EC:1.4.4.2  glycine dehydrogenase (decarboxylating) | | | | | | |
|  |  | RAFL08-11-O04 | At4g33010 / glycine dehydrogenase (decarboxylating) (glycine decarboxylase/glycine cleavage system P-protein), putative | |  |  |  |  |  | | --- | --- | --- | --- | --- | |  |  |  |  |  | | EC:1.4.4.2  glycine dehydrogenase (decarboxylating) | | | | | | |
|  |  | RAFL11-06-F06 | At4g33010 / glycine dehydrogenase (decarboxylating) (glycine decarboxylase/glycine cleavage system P-protein), putative | |  |  |  |  |  | | --- | --- | --- | --- | --- | |  |  |  |  |  | | EC:1.4.4.2  glycine dehydrogenase (decarboxylating) | | | | | | |
|  |  | RAFL07-18-E18 | At4g33010 / glycine dehydrogenase (decarboxylating) (glycine decarboxylase/glycine cleavage system P-protein), putative | |  |  |  |  |  | | --- | --- | --- | --- | --- | |  |  |  |  |  | | EC:1.4.4.2  glycine dehydrogenase (decarboxylating) | | | | | | |
|  |  | RAFL09-09-C13 | At4g33010 / glycine dehydrogenase (decarboxylating) (glycine decarboxylase/glycine cleavage system P-protein), putative | |  |  |  |  |  | | --- | --- | --- | --- | --- | |  |  |  |  |  | | EC:1.4.4.2  glycine dehydrogenase (decarboxylating) | | | | | | |
|  | methionine biosynthesis II | |  |  | 2 | 77 | 8 | 4576 | 0.01167852 | 0.04671408 | 4 |
|  |  | RAFL08-16-E05 | At5g17920 / 5-methyltetrahydropteroyltriglutamate--homocysteine S-methyltransferase | |  |  |  |  |  | | --- | --- | --- | --- | --- | |  |  |  |  |  | | EC:2.1.1.14  5-methyltetrahydropteroyltriglutamate-homocysteine S-methyltransferase | | | | | | |
|  |  | RAFL09-09-A21 | At5g17920 / 5-methyltetrahydropteroyltriglutamate--homocysteine S-methyltransferase | |  |  |  |  |  | | --- | --- | --- | --- | --- | |  |  |  |  |  | | EC:2.1.1.14  5-methyltetrahydropteroyltriglutamate-homocysteine S-methyltransferase | | | | | | |
|  | carotenoid biosynthesis | |  |  | 1 | 78 | 0 | 4584 | 0.016941883 | 0.016941883 | 1 |
|  |  | RAFL07-12-F08 | At5g17230 / phytoene synthase (geranylgeranyl-diphosphate geranylgeranyl transferase)(PSY) | |  |  |  |  |  | | --- | --- | --- | --- | --- | |  |  |  |  |  | | EC:2.5.1.32  geranylgeranyl-diphosphate geranylgeranyltransferase | | | | | | |
|  | methionine and <I>S</I>-adenosylmethionine synthesis | |  |  | 2 | 77 | 11 | 4573 | 0.019588634 | 0.1175318 | 6 |
|  |  | RAFL08-16-E05 | At5g17920 / 5-methyltetrahydropteroyltriglutamate--homocysteine S-methyltransferase | |  |  |  |  |  | | --- | --- | --- | --- | --- | |  |  |  |  |  | | EC:2.1.1.14  5-methyltetrahydropteroyltriglutamate-homocysteine S-methyltransferase | | | | | | |
|  |  | RAFL09-09-A21 | At5g17920 / 5-methyltetrahydropteroyltriglutamate--homocysteine S-methyltransferase | |  |  |  |  |  | | --- | --- | --- | --- | --- | |  |  |  |  |  | | EC:2.1.1.14  5-methyltetrahydropteroyltriglutamate-homocysteine S-methyltransferase | | | | | | |
|  | (deoxy)ribose phosphate degradation | |  |  | 1 | 78 | 1 | 4583 | 0.03360031 | 0.06720062 | 2 |
|  |  | RAFL07-12-I18 | At2g45290 / transketolase precursor -related | |  |  |  |  |  | | --- | --- | --- | --- | --- | |  |  |  |  |  | | EC:2.2.1.1  transketolase | | | | | | |
|  | pyridoxal 5'-phosphate biosynthesis | |  |  | 1 | 78 | 1 | 4583 | 0.03360031 | 0.06720062 | 2 |
|  |  | RAFL04-15-A04 | At4g15560 / DEF (CLA1) protein | |  |  |  |  |  | | --- | --- | --- | --- | --- | |  |  |  |  |  | | EC:4.1.3.37  1-deoxy-D-xylulose-5-phosphate synthase | | | | | | |
|  | thiamine biosynthesis | |  |  | 1 | 78 | 2 | 4582 | 0.049979966 | 0.1499399 | 3 |
|  |  | RAFL04-15-A04 | At4g15560 / DEF (CLA1) protein | |  |  |  |  |  | | --- | --- | --- | --- | --- | |  |  |  |  |  | | EC:4.1.3.37  1-deoxy-D-xylulose-5-phosphate synthase | | | | | | |
| Cluster:3-2 | | |  |  | A | B | C | D | P | P' | N |
|  | methionine degradation I | |  |  | 4 | 33 | 6 | 4620 | 6.813379E-7 | 3.4066893E-6 | 5 |
|  |  | RAFL09-06-N12 | At3g23810 / S-adenosyl-L-homocysteinas -related | |  |  |  |  |  | | --- | --- | --- | --- | --- | |  |  |  |  |  | | EC:3.3.1.1  adenosylhomocysteinase | | | | | | |
|  |  | RAFL07-09-L01 | At3g23810 / S-adenosyl-L-homocysteinas -related | |  |  |  |  |  | | --- | --- | --- | --- | --- | |  |  |  |  |  | | EC:3.3.1.1  adenosylhomocysteinase | | | | | | |
|  |  | RAFL09-13-P13 | At3g23810 / S-adenosyl-L-homocysteinas -related | |  |  |  |  |  | | --- | --- | --- | --- | --- | |  |  |  |  |  | | EC:3.3.1.1  adenosylhomocysteinase | | | | | | |
|  |  | RAFL09-10-M18 | At3g23810 / S-adenosyl-L-homocysteinas -related | |  |  |  |  |  | | --- | --- | --- | --- | --- | |  |  |  |  |  | | EC:3.3.1.1  adenosylhomocysteinase | | | | | | |
|  | chlorophyll biosynthesis | |  |  | 4 | 33 | 11 | 4615 | 4.3046944E-6 | 3.4437555E-5 | 8 |
|  |  | RAFL09-06-N12 | At3g23810 / S-adenosyl-L-homocysteinas -related | |  |  |  |  |  | | --- | --- | --- | --- | --- | |  |  |  |  |  | | EC:4.3.1.8  adenosylhomocysteinase | | | | | | |
|  |  | RAFL07-09-L01 | At3g23810 / S-adenosyl-L-homocysteinas -related | |  |  |  |  |  | | --- | --- | --- | --- | --- | |  |  |  |  |  | | EC:4.3.1.8  adenosylhomocysteinase | | | | | | |
|  |  | RAFL09-13-P13 | At3g23810 / S-adenosyl-L-homocysteinas -related | |  |  |  |  |  | | --- | --- | --- | --- | --- | |  |  |  |  |  | | EC:4.3.1.8  adenosylhomocysteinase | | | | | | |
|  |  | RAFL09-10-M18 | At3g23810 / S-adenosyl-L-homocysteinas -related | |  |  |  |  |  | | --- | --- | --- | --- | --- | |  |  |  |  |  | | EC:4.3.1.8  adenosylhomocysteinase | | | | | | |
|  | biosynthesis of proto- and siroheme | |  |  | 4 | 33 | 11 | 4615 | 4.3046944E-6 | 3.4437555E-5 | 8 |
|  |  | RAFL09-06-N12 | At3g23810 / S-adenosyl-L-homocysteinas -related | |  |  |  |  |  | | --- | --- | --- | --- | --- | |  |  |  |  |  | | EC:4.3.1.8  adenosylhomocysteinase | | | | | | |
|  |  | RAFL07-09-L01 | At3g23810 / S-adenosyl-L-homocysteinas -related | |  |  |  |  |  | | --- | --- | --- | --- | --- | |  |  |  |  |  | | EC:4.3.1.8  adenosylhomocysteinase | | | | | | |
|  |  | RAFL09-13-P13 | At3g23810 / S-adenosyl-L-homocysteinas -related | |  |  |  |  |  | | --- | --- | --- | --- | --- | |  |  |  |  |  | | EC:4.3.1.8  adenosylhomocysteinase | | | | | | |
|  |  | RAFL09-10-M18 | At3g23810 / S-adenosyl-L-homocysteinas -related | |  |  |  |  |  | | --- | --- | --- | --- | --- | |  |  |  |  |  | | EC:4.3.1.8  adenosylhomocysteinase | | | | | | |
|  | GDP-D-rhamnose biosynthesis | |  |  | 2 | 35 | 7 | 4619 | 0.0021297743 | 0.010648872 | 5 |
|  |  | RAFL09-16-F08 | At3g23820 / NAD-dependent epimerase/dehydratase family | |  |  |  |  |  | | --- | --- | --- | --- | --- | |  |  |  |  |  | | EC:4.2.1.47  3-beta-hydroxy-delta5-steroid dehydrogenase | | | | | | |
|  |  | RAFL09-07-D12 | At3g23820 / NAD-dependent epimerase/dehydratase family | |  |  |  |  |  | | --- | --- | --- | --- | --- | |  |  |  |  |  | | EC:4.2.1.47  3-beta-hydroxy-delta5-steroid dehydrogenase | | | | | | |
|  | dTDP-rhamnose biosynthesis | |  |  | 2 | 35 | 7 | 4619 | 0.0021297743 | 0.012778646 | 6 |
|  |  | RAFL09-16-F08 | At3g23820 / NAD-dependent epimerase/dehydratase family | |  |  |  |  |  | | --- | --- | --- | --- | --- | |  |  |  |  |  | | EC:4.2.1.46  dTDP-glucose 4,6-dehydratase | | | | | | |
|  |  | RAFL09-07-D12 | At3g23820 / NAD-dependent epimerase/dehydratase family | |  |  |  |  |  | | --- | --- | --- | --- | --- | |  |  |  |  |  | | EC:4.2.1.46  dTDP-glucose 4,6-dehydratase | | | | | | |
|  | β-D-glucuronide degradation | |  |  | 1 | 36 | 0 | 4626 | 0.007934806 | 0.007934806 | 1 |
|  |  | RAFL08-10-H13 | At5g07830 / glycosyl hydrolase family 79 (endo-beta-glucuronidase/heparanase) | |  |  |  |  |  | | --- | --- | --- | --- | --- | |  |  |  |  |  | | EC:3.2.1.31  beta-glucuronidase | | | | | | |
|  | galactose degradation I | |  |  | 2 | 35 | 17 | 4609 | 0.009623891 | 0.13473447 | 14 |
|  |  | RAFL09-16-F08 | At3g23820 / NAD-dependent epimerase/dehydratase family | |  |  |  |  |  | | --- | --- | --- | --- | --- | |  |  |  |  |  | | EC:5.1.3.2  UDP-glucose 4-epimerase | | | | | | |
|  |  | RAFL09-07-D12 | At3g23820 / NAD-dependent epimerase/dehydratase family | |  |  |  |  |  | | --- | --- | --- | --- | --- | |  |  |  |  |  | | EC:5.1.3.2  UDP-glucose 4-epimerase | | | | | | |
|  | colanic acid building blocks biosynthesis | |  |  | 2 | 35 | 22 | 4604 | 0.015151827 | 0.2272774 | 15 |
|  |  | RAFL09-16-F08 | At3g23820 / NAD-dependent epimerase/dehydratase family | |  |  |  |  |  | | --- | --- | --- | --- | --- | |  |  |  |  |  | | EC:4.2.1.47 ,EC:5.1.3.2  UDP-glucose 4-epimerase  3-beta-hydroxy-delta5-steroid dehydrogenase | | | | | | |
|  |  | RAFL09-07-D12 | At3g23820 / NAD-dependent epimerase/dehydratase family | |  |  |  |  |  | | --- | --- | --- | --- | --- | |  |  |  |  |  | | EC:4.2.1.47 ,EC:5.1.3.2  UDP-glucose 4-epimerase  3-beta-hydroxy-delta5-steroid dehydrogenase | | | | | | |
|  | lactose degradation IV | |  |  | 2 | 35 | 23 | 4603 | 0.0163878 | 0.2785926 | 17 |
|  |  | RAFL09-16-F08 | At3g23820 / NAD-dependent epimerase/dehydratase family | |  |  |  |  |  | | --- | --- | --- | --- | --- | |  |  |  |  |  | | EC:5.1.3.2  UDP-glucose 4-epimerase | | | | | | |
|  |  | RAFL09-07-D12 | At3g23820 / NAD-dependent epimerase/dehydratase family | |  |  |  |  |  | | --- | --- | --- | --- | --- | |  |  |  |  |  | | EC:5.1.3.2  UDP-glucose 4-epimerase | | | | | | |
|  | UDP-glucose conversion | |  |  | 2 | 35 | 24 | 4602 | 0.017665556 | 0.2826489 | 16 |
|  |  | RAFL09-16-F08 | At3g23820 / NAD-dependent epimerase/dehydratase family | |  |  |  |  |  | | --- | --- | --- | --- | --- | |  |  |  |  |  | | EC:5.1.3.2  UDP-glucose 4-epimerase | | | | | | |
|  |  | RAFL09-07-D12 | At3g23820 / NAD-dependent epimerase/dehydratase family | |  |  |  |  |  | | --- | --- | --- | --- | --- | |  |  |  |  |  | | EC:5.1.3.2  UDP-glucose 4-epimerase | | | | | | |
| Cluster:6-2 | | |  |  | A | B | C | D | P | P' | N |
|  | salicylic acid biosynthesis | |  |  | 3 | 173 | 2 | 4485 | 4.998933E-4 | 0.0014996799 | 3 |
|  |  | RAFL09-11-L22 | At3g53260 / phenylalanine ammonia-lyase (PAL2) | |  |  |  |  |  | | --- | --- | --- | --- | --- | |  |  |  |  |  | | EC:4.3.1.5  phenylalanine ammonia-lyase | | | | | | |
|  |  | RAFL04-13-B02 | At2g37040 / phenylalanine ammonia lyase (PAL1) | |  |  |  |  |  | | --- | --- | --- | --- | --- | |  |  |  |  |  | | EC:4.3.1.5  phenylalanine ammonia-lyase | | | | | | |
|  |  | RAFL04-16-D08 | At3g53260 / phenylalanine ammonia-lyase (PAL2) | |  |  |  |  |  | | --- | --- | --- | --- | --- | |  |  |  |  |  | | EC:4.3.1.5  phenylalanine ammonia-lyase | | | | | | |
|  | suberin biosynthesis | |  |  | 4 | 172 | 7 | 4480 | 5.2568526E-4 | 0.0031541116 | 6 |
|  |  | RAFL09-11-L22 | At3g53260 / phenylalanine ammonia-lyase (PAL2) | |  |  |  |  |  | | --- | --- | --- | --- | --- | |  |  |  |  |  | | EC:4.3.1.5  phenylalanine ammonia-lyase | | | | | | |
|  |  | RAFL04-13-B02 | At2g37040 / phenylalanine ammonia lyase (PAL1) | |  |  |  |  |  | | --- | --- | --- | --- | --- | |  |  |  |  |  | | EC:4.3.1.5  phenylalanine ammonia-lyase | | | | | | |
|  |  | RAFL04-16-D08 | At3g53260 / phenylalanine ammonia-lyase (PAL2) | |  |  |  |  |  | | --- | --- | --- | --- | --- | |  |  |  |  |  | | EC:4.3.1.5  phenylalanine ammonia-lyase | | | | | | |
|  |  | RAFL04-17-C19 | At4g34050 / caffeoyl-CoA 3-O-methyltransferase | |  |  |  |  |  | | --- | --- | --- | --- | --- | |  |  |  |  |  | | EC:2.1.1.104  caffeoyl-CoA O-methyltransferase | | | | | | |
|  | phenylpropanoid pathway, initial reactions | |  |  | 3 | 173 | 3 | 4484 | 9.720464E-4 | 0.0038881856 | 4 |
|  |  | RAFL09-11-L22 | At3g53260 / phenylalanine ammonia-lyase (PAL2) | |  |  |  |  |  | | --- | --- | --- | --- | --- | |  |  |  |  |  | | EC:4.3.1.5  phenylalanine ammonia-lyase | | | | | | |
|  |  | RAFL04-13-B02 | At2g37040 / phenylalanine ammonia lyase (PAL1) | |  |  |  |  |  | | --- | --- | --- | --- | --- | |  |  |  |  |  | | EC:4.3.1.5  phenylalanine ammonia-lyase | | | | | | |
|  |  | RAFL04-16-D08 | At3g53260 / phenylalanine ammonia-lyase (PAL2) | |  |  |  |  |  | | --- | --- | --- | --- | --- | |  |  |  |  |  | | EC:4.3.1.5  phenylalanine ammonia-lyase | | | | | | |
|  | lignin biosynthesis | |  |  | 4 | 172 | 17 | 4470 | 0.0070927707 | 0.08511325 | 12 |
|  |  | RAFL09-11-L22 | At3g53260 / phenylalanine ammonia-lyase (PAL2) | |  |  |  |  |  | | --- | --- | --- | --- | --- | |  |  |  |  |  | | EC:4.3.1.5  phenylalanine ammonia-lyase | | | | | | |
|  |  | RAFL04-13-B02 | At2g37040 / phenylalanine ammonia lyase (PAL1) | |  |  |  |  |  | | --- | --- | --- | --- | --- | |  |  |  |  |  | | EC:4.3.1.5  phenylalanine ammonia-lyase | | | | | | |
|  |  | RAFL04-16-D08 | At3g53260 / phenylalanine ammonia-lyase (PAL2) | |  |  |  |  |  | | --- | --- | --- | --- | --- | |  |  |  |  |  | | EC:4.3.1.5  phenylalanine ammonia-lyase | | | | | | |
|  |  | RAFL04-17-C19 | At4g34050 / caffeoyl-CoA 3-O-methyltransferase | |  |  |  |  |  | | --- | --- | --- | --- | --- | |  |  |  |  |  | | EC:2.1.1.104  caffeoyl-CoA O-methyltransferase | | | | | | |
|  | tryptophan biosynthesis | |  |  | 2 | 174 | 5 | 4482 | 0.026250903 | 0.13125451 | 5 |
|  |  | RAFL05-01-B07 | At3g57880 / C2 domain-containing protein | |  |  |  |  |  | | --- | --- | --- | --- | --- | |  |  |  |  |  | | EC:2.4.2.18  anthranilate phosphoribosyltransferase | | | | | | |
|  |  | RAFL06-16-J02 | At4g30530 / glutamine amidotransferase class-I domain-containing protein | |  |  |  |  |  | | --- | --- | --- | --- | --- | |  |  |  |  |  | | EC:4.1.3.27  GMP synthase (glutamine-hydrolyzing) | | | | | | |
|  | fatty acid elongation -- unsaturated | |  |  | 2 | 174 | 6 | 4481 | 0.034143474 | 0.23900433 | 7 |
|  |  | RAFL05-10-D10 | At5g43760 / beta-ketoacyl-CoA synthase, putative | |  |  |  |  |  | | --- | --- | --- | --- | --- | |  |  |  |  |  | | EC:2.3.1.41  3-oxoacyl-[acyl-carrier protein] synthase | | | | | | |
|  |  | RAFL02-03-L07 | At2g26250 / beta-ketoacyl-CoA synthase family (FIDDLEHEAD) (FDH) | |  |  |  |  |  | | --- | --- | --- | --- | --- | |  |  |  |  |  | | EC:2.3.1.41  3-oxoacyl-[acyl-carrier protein] synthase | | | | | | |
|  | fatty acid elongation -- saturated | |  |  | 2 | 174 | 7 | 4480 | 0.042826395 | 0.34261116 | 8 |
|  |  | RAFL05-10-D10 | At5g43760 / beta-ketoacyl-CoA synthase, putative | |  |  |  |  |  | | --- | --- | --- | --- | --- | |  |  |  |  |  | | EC:2.3.1.41  3-oxoacyl-[acyl-carrier protein] synthase | | | | | | |
|  |  | RAFL02-03-L07 | At2g26250 / beta-ketoacyl-CoA synthase family (FIDDLEHEAD) (FDH) | |  |  |  |  |  | | --- | --- | --- | --- | --- | |  |  |  |  |  | | EC:2.3.1.41  3-oxoacyl-[acyl-carrier protein] synthase | | | | | | |
| Cluster:7-0 | | |  |  | A | B | C | D | P | P' | N |
|  | biotin biosynthesis I | |  |  | 2 | 245 | 2 | 4414 | 0.015618202 | 0.046854608 | 3 |
|  |  | RAFL05-21-O04 | At3g08860 / alanine--glyoxylate aminotransferase (beta-alanine-pyruvate aminotransferase/AGT), putative | |  |  |  |  |  | | --- | --- | --- | --- | --- | |  |  |  |  |  | | EC:2.6.1.62  4-aminobutyrate transaminase//alanine-glyoxylate transaminase | | | | | | |
|  |  | RAFL07-12-J17 | At3g48780 / serine C-palmitoyltransferase, putative | |  |  |  |  |  | | --- | --- | --- | --- | --- | |  |  |  |  |  | | EC:2.3.1.47  8-amino-7-oxononanoate synthase | | | | | | |
|  | suberin biosynthesis | |  |  | 3 | 244 | 8 | 4408 | 0.017647006 | 0.10588203 | 6 |
|  |  | RAFL06-11-J16 | At2g30490 / cytochrome P450 73 / trans-cinnamate 4-monooxygenase / cinnamate-4-hydroxylase (CYP73) (C4H) | |  |  |  |  |  | | --- | --- | --- | --- | --- | |  |  |  |  |  | | EC:1.14.13.11  cinnamate 4-hydroxylase | | | | | | |
|  |  | RAFL09-15-A15 | At4g26220 / caffeoyl-CoA 3-O-methyltransferase, putative | |  |  |  |  |  | | --- | --- | --- | --- | --- | |  |  |  |  |  | | EC:2.1.1.104  caffeoyl-CoA O-methyltransferase | | | | | | |
|  |  | RAFL06-07-M15 | At5g54160 / O-methyltransferase 1 | |  |  |  |  |  | | --- | --- | --- | --- | --- | |  |  |  |  |  | | EC:2.1.1.68  caffeic acid /5-hydroxyferulic acid o-methyltransferase | | | | | | |
| Cluster:4-2 | | |  |  | A | B | C | D | P | P' | N |
|  | tRNA charging pathway | |  |  | 4 | 131 | 13 | 4515 | 0.0011926556 | 0.010733901 | 9 |
|  |  | RAFL04-15-H14 | At4g26870 / aspartate-tRNA ligase (aspartyl-tRNA synthetase), putative | |  |  |  |  |  | | --- | --- | --- | --- | --- | |  |  |  |  |  | | EC:6.1.1.12 ,EC:6.1.1.22 ,EC:6.1.1.20 ,EC:6.1.1.6  asparagine-tRNA ligase  lysine-tRNA ligase  phenylalanine-tRNA ligase | | | | | | |
|  |  | RAFL04-19-C07 | At3g62120 / multifunctional aminoacyl-tRNA ligase-related protein | |  |  |  |  |  | | --- | --- | --- | --- | --- | |  |  |  |  |  | | EC:6.1.1.3 ,EC:6.1.1.15  threonine-tRNA ligase  glycine-tRNA ligase | | | | | | |
|  |  | RAFL05-08-P23 | At3g59980 / expressed protein | |  |  |  |  |  | | --- | --- | --- | --- | --- | |  |  |  |  |  | | EC:6.1.1.10 ,EC:6.1.1.20  isoleucine-tRNA ligase//leucine-tRNA ligase  phenylalanine-tRNA ligase | | | | | | |
|  |  | RAFL02-09-F04 | At5g26830 / threonyl-tRNA synthetase | |  |  |  |  |  | | --- | --- | --- | --- | --- | |  |  |  |  |  | | EC:6.1.1.3 ,EC:6.1.1.15 ,EC:6.1.1.14  threonine-tRNA ligase  glycine-tRNA ligase | | | | | | |
|  | pyridoxal 5'-phosphate salvage pathway | |  |  | 1 | 134 | 0 | 4528 | 0.028951319 | 0.028951319 | 1 |
|  |  | RAFL05-16-L10 | At5g49970 / expressed protein | |  |  |  |  |  | | --- | --- | --- | --- | --- | |  |  |  |  |  | | EC:1.4.3.5  pyridoxamine-phosphate oxidase | | | | | | |
| Cluster:10-2 | | |  |  | A | B | C | D | P | P' | N |
|  | sucrose degradation III | |  |  | 3 | 106 | 16 | 4538 | 0.009166159 | 0.11916006 | 13 |
|  |  | RAFL05-07-J05 | At3g43190 / sucrose synthase (UDP-glucose-fructose glucosyltransferase/sucrose-UDP glucosyltransferase), putative | |  |  |  |  |  | | --- | --- | --- | --- | --- | |  |  |  |  |  | | EC:2.4.1.13  UDP-glycosyltransferase | | | | | | |
|  |  | RAFL08-15-K01 | At1g62660 / glycosyl hydrolase family 32 | |  |  |  |  |  | | --- | --- | --- | --- | --- | |  |  |  |  |  | | EC:3.2.1.26  unknown | | | | | | |
|  |  | RAFL08-13-K06 | At1g62660 / glycosyl hydrolase family 32 | |  |  |  |  |  | | --- | --- | --- | --- | --- | |  |  |  |  |  | | EC:3.2.1.26  unknown | | | | | | |
|  | triacylglycerol degradation | |  |  | 2 | 107 | 6 | 4548 | 0.013828159 | 0.08296896 | 6 |
|  |  | RAFL11-07-N24 | At1g10740 / lipase -related | |  |  |  |  |  | | --- | --- | --- | --- | --- | |  |  |  |  |  | | EC:3.1.1.3  triacylglycerol lipase | | | | | | |
|  |  | RAFL08-08-G07 | At1g73920 / lipase family | |  |  |  |  |  | | --- | --- | --- | --- | --- | |  |  |  |  |  | | EC:3.1.1.3  triacylglycerol lipase | | | | | | |
|  | arginine degradation II | |  |  | 2 | 107 | 8 | 4546 | 0.021556646 | 0.17245317 | 8 |
|  |  | RAFL08-15-A08 | At4g34710 / arginine decarboxylase SPE2 | |  |  |  |  |  | | --- | --- | --- | --- | --- | |  |  |  |  |  | | EC:4.1.1.19  arginine decarboxylase | | | | | | |
|  |  | RAFL04-13-O07 | At3g22200 / 4-aminobutyrate aminotransferase (gamma-amino-N-butyrate transaminase/GABA transaminase/beta-alanine--oxoglutarate aminotransferase) | |  |  |  |  |  | | --- | --- | --- | --- | --- | |  |  |  |  |  | | EC:2.6.1.19  4-aminobutyrate transaminase//alanine-glyoxylate transaminase | | | | | | |
|  | arginine degradation III | |  |  | 2 | 107 | 8 | 4546 | 0.021556646 | 0.17245317 | 8 |
|  |  | RAFL08-15-A08 | At4g34710 / arginine decarboxylase SPE2 | |  |  |  |  |  | | --- | --- | --- | --- | --- | |  |  |  |  |  | | EC:4.1.1.19  arginine decarboxylase | | | | | | |
|  |  | RAFL04-13-O07 | At3g22200 / 4-aminobutyrate aminotransferase (gamma-amino-N-butyrate transaminase/GABA transaminase/beta-alanine--oxoglutarate aminotransferase) | |  |  |  |  |  | | --- | --- | --- | --- | --- | |  |  |  |  |  | | EC:2.6.1.19  4-aminobutyrate transaminase | | | | | | |
|  | arginine degradation V | |  |  | 2 | 107 | 8 | 4546 | 0.021556646 | 0.17245317 | 8 |
|  |  | RAFL08-15-A08 | At4g34710 / arginine decarboxylase SPE2 | |  |  |  |  |  | | --- | --- | --- | --- | --- | |  |  |  |  |  | | EC:1.13.12.1  arginine decarboxylase | | | | | | |
|  |  | RAFL04-13-O07 | At3g22200 / 4-aminobutyrate aminotransferase (gamma-amino-N-butyrate transaminase/GABA transaminase/beta-alanine--oxoglutarate aminotransferase) | |  |  |  |  |  | | --- | --- | --- | --- | --- | |  |  |  |  |  | | EC:2.6.1.19  4-aminobutyrate transaminase//alanine-glyoxylate transaminase | | | | | | |
|  | polyamine biosynthesis II | |  |  | 2 | 107 | 8 | 4546 | 0.021556646 | 0.15089652 | 7 |
|  |  | RAFL05-15-A16 | At5g53120 / spermidine synthase | |  |  |  |  |  | | --- | --- | --- | --- | --- | |  |  |  |  |  | | EC:2.5.1.16  spermidine synthase | | | | | | |
|  |  | RAFL08-15-A08 | At4g34710 / arginine decarboxylase SPE2 | |  |  |  |  |  | | --- | --- | --- | --- | --- | |  |  |  |  |  | | EC:4.1.1.19  arginine decarboxylase | | | | | | |
|  | polyamine biosynthesis III | |  |  | 2 | 107 | 8 | 4546 | 0.021556646 | 0.15089652 | 7 |
|  |  | RAFL05-15-A16 | At5g53120 / spermidine synthase | |  |  |  |  |  | | --- | --- | --- | --- | --- | |  |  |  |  |  | | EC:2.5.1.16  spermidine synthase | | | | | | |
|  |  | RAFL08-15-A08 | At4g34710 / arginine decarboxylase SPE2 | |  |  |  |  |  | | --- | --- | --- | --- | --- | |  |  |  |  |  | | EC:4.1.1.19  arginine decarboxylase | | | | | | |
|  | spermine biosynthesis I | |  |  | 2 | 107 | 9 | 4545 | 0.025949622 | 0.20759697 | 8 |
|  |  | RAFL05-15-A16 | At5g53120 / spermidine synthase | |  |  |  |  |  | | --- | --- | --- | --- | --- | |  |  |  |  |  | | EC:2.5.1.16  spermidine synthase | | | | | | |
|  |  | RAFL08-15-A08 | At4g34710 / arginine decarboxylase SPE2 | |  |  |  |  |  | | --- | --- | --- | --- | --- | |  |  |  |  |  | | EC:4.1.1.19  arginine decarboxylase | | | | | | |
|  | polyamine biosynthesis I | |  |  | 2 | 107 | 9 | 4545 | 0.025949622 | 0.20759697 | 8 |
|  |  | RAFL05-15-A16 | At5g53120 / spermidine synthase | |  |  |  |  |  | | --- | --- | --- | --- | --- | |  |  |  |  |  | | EC:2.5.1.16  spermidine synthase | | | | | | |
|  |  | RAFL08-15-A08 | At4g34710 / arginine decarboxylase SPE2 | |  |  |  |  |  | | --- | --- | --- | --- | --- | |  |  |  |  |  | | EC:4.1.1.19  arginine decarboxylase | | | | | | |
|  | leucine degradation II | |  |  | 1 | 108 | 1 | 4553 | 0.0462095 | 0.092419 | 2 |
|  |  | RAFL06-10-M04 | At2g26800 / hydroxymethylglutaryl-CoA lyase -related | |  |  |  |  |  | | --- | --- | --- | --- | --- | |  |  |  |  |  | | EC:4.1.3.4  hydroxymethylglutaryl-CoA lyase | | | | | | |
|  | leucine degradation I | |  |  | 1 | 108 | 1 | 4553 | 0.0462095 | 0.092419 | 2 |
|  |  | RAFL06-10-M04 | At2g26800 / hydroxymethylglutaryl-CoA lyase -related | |  |  |  |  |  | | --- | --- | --- | --- | --- | |  |  |  |  |  | | EC:4.1.3.4  hydroxymethylglutaryl-CoA lyase | | | | | | |
|  | trehalose biosynthesis II | |  |  | 1 | 108 | 1 | 4553 | 0.0462095 | 0.092419 | 2 |
|  |  | RAFL07-14-D12 | At4g12430 / trehalose-6-phosphate phosphatase, putative | |  |  |  |  |  | | --- | --- | --- | --- | --- | |  |  |  |  |  | | EC:3.1.3.12  trehalose-phosphatase | | | | | | |
| Cluster:4-1 | | |  |  | A | B | C | D | P | P' | N |
|  | respiration (anaerobic)-- electron donors reaction list | |  |  | 5 | 305 | 7 | 4346 | 6.7494786E-4 | 0.0033747396 | 5 |
|  |  | RAFL05-04-N24 | At3g18410 / expressed protein | |  |  |  |  |  | | --- | --- | --- | --- | --- | |  |  |  |  |  | | EC:1.6.5.3  NADH dehydrogenase (ubiquinone) | | | | | | |
|  |  | RAFL11-02-J20 | At3g12260 / expressed protein | |  |  |  |  |  | | --- | --- | --- | --- | --- | |  |  |  |  |  | | EC:1.6.5.3  NADH dehydrogenase (ubiquinone) | | | | | | |
|  |  | RAFL09-10-O11 | At3g12260 / expressed protein | |  |  |  |  |  | | --- | --- | --- | --- | --- | |  |  |  |  |  | | EC:1.6.5.3  NADH dehydrogenase (ubiquinone) | | | | | | |
|  |  | RAFL09-18-I01 | At5g08530 / NADH-ubiquinone oxidoreductase (mitochondrial), putative | |  |  |  |  |  | | --- | --- | --- | --- | --- | |  |  |  |  |  | | EC:1.6.5.3 ,EC:1.18.99.1  NADH dehydrogenase (ubiquinone) | | | | | | |
|  |  | RAFL06-08-D19 | At5g37510 / NADH dehydrogenase (ubiquinone), mitochondrial, putative | |  |  |  |  |  | | --- | --- | --- | --- | --- | |  |  |  |  |  | | EC:1.2.1.2 ,EC:1.6.5.3  NADH dehydrogenase (ubiquinone)  formate dehydrogenase | | | | | | |
|  | lactate oxidation | |  |  | 5 | 305 | 9 | 4344 | 0.001527034 | 0.012216272 | 8 |
|  |  | RAFL07-16-E16 | At3g13930 / acetyltransferase -related | |  |  |  |  |  | | --- | --- | --- | --- | --- | |  |  |  |  |  | | EC:2.3.1.12  unknown | | | | | | |
|  |  | RAFL06-08-D06 | At2g34590 / pyruvate dehydrogenase E1 beta subunit -related | |  |  |  |  |  | | --- | --- | --- | --- | --- | |  |  |  |  |  | | EC:1.2.4.1  pyruvate decarboxylase | | | | | | |
|  |  | RAFL09-12-A19 | At3g13930 / acetyltransferase -related | |  |  |  |  |  | | --- | --- | --- | --- | --- | |  |  |  |  |  | | EC:2.3.1.12  unknown | | | | | | |
|  |  | RAFL09-11-F09 | At1g30120 / pyruvate dehydrogenase E1 beta subunit -related | |  |  |  |  |  | | --- | --- | --- | --- | --- | |  |  |  |  |  | | EC:1.2.4.1  pyruvate decarboxylase | | | | | | |
|  |  | RAFL09-16-O16 | At1g01090 / pyruvate dehydrogenase E1 alpha subunit | |  |  |  |  |  | | --- | --- | --- | --- | --- | |  |  |  |  |  | | EC:1.2.4.1  pyruvate decarboxylase | | | | | | |
|  | aerobic respiration -- electron donors reaction list | |  |  | 5 | 305 | 11 | 4342 | 0.0029831196 | 0.020881835 | 7 |
|  |  | RAFL05-04-N24 | At3g18410 / expressed protein | |  |  |  |  |  | | --- | --- | --- | --- | --- | |  |  |  |  |  | | EC:1.6.5.3  NADH dehydrogenase (ubiquinone) | | | | | | |
|  |  | RAFL11-02-J20 | At3g12260 / expressed protein | |  |  |  |  |  | | --- | --- | --- | --- | --- | |  |  |  |  |  | | EC:1.6.5.3  NADH dehydrogenase (ubiquinone) | | | | | | |
|  |  | RAFL09-10-O11 | At3g12260 / expressed protein | |  |  |  |  |  | | --- | --- | --- | --- | --- | |  |  |  |  |  | | EC:1.6.5.3  NADH dehydrogenase (ubiquinone) | | | | | | |
|  |  | RAFL09-18-I01 | At5g08530 / NADH-ubiquinone oxidoreductase (mitochondrial), putative | |  |  |  |  |  | | --- | --- | --- | --- | --- | |  |  |  |  |  | | EC:1.6.5.3 ,EC:1.18.99.1  NADH dehydrogenase (ubiquinone) | | | | | | |
|  |  | RAFL06-08-D19 | At5g37510 / NADH dehydrogenase (ubiquinone), mitochondrial, putative | |  |  |  |  |  | | --- | --- | --- | --- | --- | |  |  |  |  |  | | EC:1.6.5.3  NADH dehydrogenase (ubiquinone) | | | | | | |
|  | arginine degradation VIII | |  |  | 2 | 308 | 2 | 4351 | 0.024166461 | 0.07249938 | 3 |
|  |  | RAFL05-09-P07 | At4g29120 / expressed protein | |  |  |  |  |  | | --- | --- | --- | --- | --- | |  |  |  |  |  | | EC:1.5.1.2  disulfide oxidoreductase | | | | | | |
|  |  | RAFL06-14-H08 | At5g14800 / pyrroline-5-carboxylate reductase | |  |  |  |  |  | | --- | --- | --- | --- | --- | |  |  |  |  |  | | EC:1.5.1.2  disulfide oxidoreductase | | | | | | |
|  | sucrose biosynthesis | |  |  | 3 | 307 | 9 | 4344 | 0.040857635 | 0.36771873 | 9 |
|  |  | RAFL07-13-K21 | At2g35840 / expressed protein | |  |  |  |  |  | | --- | --- | --- | --- | --- | |  |  |  |  |  | | EC:3.1.3.24  sucrose-phosphatase | | | | | | |
|  |  | RAFL09-15-M21 | At5g17310 / UDP-glucose pyrophosphorylase | |  |  |  |  |  | | --- | --- | --- | --- | --- | |  |  |  |  |  | | EC:2.7.7.9  UDP-N-acetylglucosamine diphosphorylase | | | | | | |
|  |  | RAFL09-12-G22 | At5g17310 / UDP-glucose pyrophosphorylase | |  |  |  |  |  | | --- | --- | --- | --- | --- | |  |  |  |  |  | | EC:2.7.7.9  UDP-N-acetylglucosamine diphosphorylase | | | | | | |
| Cluster:8-0 | | |  |  | A | B | C | D | P | P' | N |
|  | IAA biosynthesis I | |  |  | 2 | 107 | 3 | 4551 | 0.005170766 | 0.020683063 | 4 |
|  |  | RAFL08-10-H06 | At3g44300 / nitrilase 2 | |  |  |  |  |  | | --- | --- | --- | --- | --- | |  |  |  |  |  | | EC:3.5.5.1  hydrolase, acting on carbon-nitrogen (but not peptide) bonds | | | | | | |
|  |  | RAFL06-13-E03 | At3g44300 / nitrilase 2 | |  |  |  |  |  | | --- | --- | --- | --- | --- | |  |  |  |  |  | | EC:3.5.5.1  hydrolase, acting on carbon-nitrogen (but not peptide) bonds | | | | | | |
|  | phenylalanine biosynthesis II | |  |  | 2 | 107 | 4 | 4550 | 0.007638084 | 0.038190417 | 5 |
|  |  | RAFL07-10-M07 | At5g53970 / aminotransferase, putative | |  |  |  |  |  | | --- | --- | --- | --- | --- | |  |  |  |  |  | | EC:2.6.1.5 ,EC:2.6.1.9  1-aminocyclopropane-1-carboxylate synthase  phosphoglycerate dehydrogenase | | | | | | |
|  |  | RAFL05-09-B02 | At5g53970 / aminotransferase, putative | |  |  |  |  |  | | --- | --- | --- | --- | --- | |  |  |  |  |  | | EC:2.6.1.5 ,EC:2.6.1.9  1-aminocyclopropane-1-carboxylate synthase  phosphoglycerate dehydrogenase | | | | | | |
|  | histidine biosynthesis I | |  |  | 2 | 107 | 6 | 4548 | 0.013828159 | 0.096797116 | 7 |
|  |  | RAFL07-10-M07 | At5g53970 / aminotransferase, putative | |  |  |  |  |  | | --- | --- | --- | --- | --- | |  |  |  |  |  | | EC:2.6.1.9  histidinol-phosphate transaminase | | | | | | |
|  |  | RAFL05-09-B02 | At5g53970 / aminotransferase, putative | |  |  |  |  |  | | --- | --- | --- | --- | --- | |  |  |  |  |  | | EC:2.6.1.9  histidinol-phosphate transaminase | | | | | | |
|  | threonine degradation | |  |  | 2 | 107 | 8 | 4546 | 0.021556646 | 0.12933987 | 6 |
|  |  | RAFL05-21-N06 | At4g13010 / oxidoreductase, zinc-binding dehydrogenase family | |  |  |  |  |  | | --- | --- | --- | --- | --- | |  |  |  |  |  | | EC:1.1.1.103  L-threonine 3-dehydrogenase//alcohol dehydrogenase, zinc-dependent | | | | | | |
|  |  | RAFL09-09-E18 | At2g32090 / glyoxalase family protein (lactoylglutathione lyase family protein) | |  |  |  |  |  | | --- | --- | --- | --- | --- | |  |  |  |  |  | | EC:4.4.1.5  4-hydroxyphenylpyruvate dioxygenase | | | | | | |
|  | tyrosine degradation | |  |  | 2 | 107 | 10 | 4544 | 0.03067075 | 0.245366 | 8 |
|  |  | RAFL07-10-M07 | At5g53970 / aminotransferase, putative | |  |  |  |  |  | | --- | --- | --- | --- | --- | |  |  |  |  |  | | EC:2.6.1.5  tyrosine transaminase | | | | | | |
|  |  | RAFL05-09-B02 | At5g53970 / aminotransferase, putative | |  |  |  |  |  | | --- | --- | --- | --- | --- | |  |  |  |  |  | | EC:2.6.1.5  tyrosine transaminase | | | | | | |
|  | xylose degradation | |  |  | 1 | 108 | 1 | 4553 | 0.0462095 | 0.092419 | 2 |
|  |  | RAFL05-15-K08 | At5g57655 / expressed protein | |  |  |  |  |  | | --- | --- | --- | --- | --- | |  |  |  |  |  | | EC:5.3.1.5  xylose isomerase | | | | | | |
| Cluster:2-1 | | |  |  | A | B | C | D | P | P' | N |
|  | alanine degradation III | |  |  | 3 | 241 | 4 | 4415 | 0.0042335736 | 0.016934294 | 4 |
|  |  | RAFL09-09-I19 | At1g23310 / alanine aminotransferase -related | |  |  |  |  |  | | --- | --- | --- | --- | --- | |  |  |  |  |  | | EC:2.6.1.2  alanine transaminase | | | | | | |
|  |  | RAFL09-16-K01 | At1g23310 / alanine aminotransferase -related | |  |  |  |  |  | | --- | --- | --- | --- | --- | |  |  |  |  |  | | EC:2.6.1.2  alanine transaminase | | | | | | |
|  |  | RAFL05-07-N11 | At1g70580 / alanine aminotransferase, putative | |  |  |  |  |  | | --- | --- | --- | --- | --- | |  |  |  |  |  | | EC:2.6.1.2  alanine transaminase | | | | | | |
|  | alanine biosynthesis II | |  |  | 3 | 241 | 4 | 4415 | 0.0042335736 | 0.016934294 | 4 |
|  |  | RAFL09-09-I19 | At1g23310 / alanine aminotransferase -related | |  |  |  |  |  | | --- | --- | --- | --- | --- | |  |  |  |  |  | | EC:2.6.1.2  alanine transaminase | | | | | | |
|  |  | RAFL09-16-K01 | At1g23310 / alanine aminotransferase -related | |  |  |  |  |  | | --- | --- | --- | --- | --- | |  |  |  |  |  | | EC:2.6.1.2  alanine transaminase | | | | | | |
|  |  | RAFL05-07-N11 | At1g70580 / alanine aminotransferase, putative | |  |  |  |  |  | | --- | --- | --- | --- | --- | |  |  |  |  |  | | EC:2.6.1.2  alanine transaminase | | | | | | |
|  | de novo biosynthesis of purine nucleotides I | |  |  | 4 | 240 | 10 | 4409 | 0.0048282314 | 0.043454085 | 9 |
|  |  | RAFL04-17-H07 | At3g57610 / adenylosuccinate synthetase | |  |  |  |  |  | | --- | --- | --- | --- | --- | |  |  |  |  |  | | EC:6.3.4.4  adenylosuccinate synthase | | | | | | |
|  |  | RAFL04-13-M20 | At4g11010 / nucleoside diphosphate kinase 3 (ndpk3) | |  |  |  |  |  | | --- | --- | --- | --- | --- | |  |  |  |  |  | | EC:2.7.4.6  nucleoside-diphosphate kinase | | | | | | |
|  |  | RAFL04-18-P17 | At5g35170 / adenylate kinase -related protein | |  |  |  |  |  | | --- | --- | --- | --- | --- | |  |  |  |  |  | | EC:2.7.4.3  adenylate kinase | | | | | | |
|  |  | RAFL04-12-O11 | At3g27740 / carbamoyl-phosphate synthase (glutamine-hydrolyzing) (glutamine-dependent carbamoyl-phosphate synthase) small subunit | |  |  |  |  |  | | --- | --- | --- | --- | --- | |  |  |  |  |  | | EC:6.3.5.2  GMP synthase (glutamine-hydrolyzing) | | | | | | |
|  | cyclopropane fatty acid (CFA) biosynthesis | |  |  | 2 | 242 | 1 | 4418 | 0.00789916 | 0.01579832 | 2 |
|  |  | RAFL07-08-E22 | At3g23530 / cyclopropane synthase, putative | |  |  |  |  |  | | --- | --- | --- | --- | --- | |  |  |  |  |  | | EC:2.1.1.79  amine oxidase | | | | | | |
|  |  | RAFL07-18-J07 | At3g23530 / cyclopropane synthase, putative | |  |  |  |  |  | | --- | --- | --- | --- | --- | |  |  |  |  |  | | EC:2.1.1.79  amine oxidase | | | | | | |
|  | de novo biosynthesis of pyrimidine deoxyribonucleotides | |  |  | 2 | 242 | 2 | 4417 | 0.01525385 | 0.045761548 | 3 |
|  |  | RAFL04-13-M20 | At4g11010 / nucleoside diphosphate kinase 3 (ndpk3) | |  |  |  |  |  | | --- | --- | --- | --- | --- | |  |  |  |  |  | | EC:2.7.4.6  nucleoside-diphosphate kinase | | | | | | |
|  |  | RAFL04-18-P17 | At5g35170 / adenylate kinase -related protein | |  |  |  |  |  | | --- | --- | --- | --- | --- | |  |  |  |  |  | | EC:2.7.4.9  guanylate kinase | | | | | | |
|  | lysine biosynthesis I | |  |  | 2 | 242 | 3 | 4416 | 0.024550742 | 0.098202966 | 4 |
|  |  | RAFL06-12-N06 | At3g53580 / diaminopimelate epimerase - like protein | |  |  |  |  |  | | --- | --- | --- | --- | --- | |  |  |  |  |  | | EC:5.1.1.7  diaminopimelate epimerase | | | | | | |
|  |  | RAFL06-15-B04 | At1g14810 / aspartate-semialdehyde dehydrogenase -related | |  |  |  |  |  | | --- | --- | --- | --- | --- | |  |  |  |  |  | | EC:1.2.1.11  aspartate-semialdehyde dehydrogenase | | | | | | |
|  | chlorophyll biosynthesis | |  |  | 3 | 241 | 12 | 4407 | 0.040360164 | 0.3228813 | 8 |
|  |  | RAFL04-17-P12 | At4g13940 / adenosylhomocysteinase | |  |  |  |  |  | | --- | --- | --- | --- | --- | |  |  |  |  |  | | EC:4.3.1.8  adenosylhomocysteinase | | | | | | |
|  |  | RAFL05-10-J03 | At1g69740 / porphobilinogen synthase (delta-aminolevulinic acid dehydratase), putative | |  |  |  |  |  | | --- | --- | --- | --- | --- | |  |  |  |  |  | | EC:4.2.1.24  porphobilinogen synthase | | | | | | |
|  |  | RAFL04-15-N01 | At1g69740 / porphobilinogen synthase (delta-aminolevulinic acid dehydratase), putative | |  |  |  |  |  | | --- | --- | --- | --- | --- | |  |  |  |  |  | | EC:4.2.1.24  porphobilinogen synthase | | | | | | |
|  | biosynthesis of proto- and siroheme | |  |  | 3 | 241 | 12 | 4407 | 0.040360164 | 0.3228813 | 8 |
|  |  | RAFL04-17-P12 | At4g13940 / adenosylhomocysteinase | |  |  |  |  |  | | --- | --- | --- | --- | --- | |  |  |  |  |  | | EC:4.3.1.8  adenosylhomocysteinase | | | | | | |
|  |  | RAFL05-10-J03 | At1g69740 / porphobilinogen synthase (delta-aminolevulinic acid dehydratase), putative | |  |  |  |  |  | | --- | --- | --- | --- | --- | |  |  |  |  |  | | EC:4.2.1.24  porphobilinogen synthase | | | | | | |
|  |  | RAFL04-15-N01 | At1g69740 / porphobilinogen synthase (delta-aminolevulinic acid dehydratase), putative | |  |  |  |  |  | | --- | --- | --- | --- | --- | |  |  |  |  |  | | EC:4.2.1.24  porphobilinogen synthase | | | | | | |
|  | superpathway of serine and glycine biosynthesis II | |  |  | 3 | 241 | 13 | 4406 | 0.04780889 | 0.43028 | 9 |
|  |  | RAFL09-09-I19 | At1g23310 / alanine aminotransferase -related | |  |  |  |  |  | | --- | --- | --- | --- | --- | |  |  |  |  |  | | EC:2.6.1.4  alanine transaminase | | | | | | |
|  |  | RAFL09-16-K01 | At1g23310 / alanine aminotransferase -related | |  |  |  |  |  | | --- | --- | --- | --- | --- | |  |  |  |  |  | | EC:2.6.1.4  alanine transaminase | | | | | | |
|  |  | RAFL05-07-N11 | At1g70580 / alanine aminotransferase, putative | |  |  |  |  |  | | --- | --- | --- | --- | --- | |  |  |  |  |  | | EC:2.6.1.4  alanine transaminase | | | | | | |
|  | isoleucine biosynthesis I | |  |  | 2 | 242 | 5 | 4414 | 0.04810191 | 0.24050954 | 5 |
|  |  | RAFL02-01-G08 | At3g58610 / ketol-acid reductoisomerase | |  |  |  |  |  | | --- | --- | --- | --- | --- | |  |  |  |  |  | | EC:1.1.1.86  ketol-acid reductoisomerase | | | | | | |
|  |  | RAFL04-13-D06 | At3g23940 / dihydroxyacid dehydratase -related | |  |  |  |  |  | | --- | --- | --- | --- | --- | |  |  |  |  |  | | EC:4.2.1.9  unknown | | | | | | |
|  | valine biosynthesis | |  |  | 2 | 242 | 5 | 4414 | 0.04810191 | 0.24050954 | 5 |
|  |  | RAFL02-01-G08 | At3g58610 / ketol-acid reductoisomerase | |  |  |  |  |  | | --- | --- | --- | --- | --- | |  |  |  |  |  | | EC:1.1.1.86  ketol-acid reductoisomerase | | | | | | |
|  |  | RAFL04-13-D06 | At3g23940 / dihydroxyacid dehydratase -related | |  |  |  |  |  | | --- | --- | --- | --- | --- | |  |  |  |  |  | | EC:4.2.1.9  unknown | | | | | | |
|  | de novo biosynthesis of purine nucleotides II | |  |  | 2 | 242 | 5 | 4414 | 0.04810191 | 0.24050954 | 5 |
|  |  | RAFL04-17-H07 | At3g57610 / adenylosuccinate synthetase | |  |  |  |  |  | | --- | --- | --- | --- | --- | |  |  |  |  |  | | EC:6.3.4.4  adenylosuccinate synthase | | | | | | |
|  |  | RAFL04-12-O11 | At3g27740 / carbamoyl-phosphate synthase (glutamine-hydrolyzing) (glutamine-dependent carbamoyl-phosphate synthase) small subunit | |  |  |  |  |  | | --- | --- | --- | --- | --- | |  |  |  |  |  | | EC:6.3.5.2  GMP synthase (glutamine-hydrolyzing) | | | | | | |
|  | sterol biosynthesis | |  |  | 2 | 242 | 5 | 4414 | 0.04810191 | 0.24050954 | 5 |
|  |  | RAFL07-16-C24 | At1g20330 / sterol-C-methyltransferase | |  |  |  |  |  | | --- | --- | --- | --- | --- | |  |  |  |  |  | | EC:2.1.1.143  24-methylenelophenol-C24-methyltransferase | | | | | | |
|  |  | RAFL07-09-C04 | At1g11680 / obtusifoliol 14-demethylase (CYP51) | |  |  |  |  |  | | --- | --- | --- | --- | --- | |  |  |  |  |  | | EC:1.14.13.70  obtusifoliol 14α-demethylase | | | | | | |
| Cluster:2-2 | | |  |  | A | B | C | D | P | P' | N |
|  | sulfate assimilation III | |  |  | 3 | 50 | 21 | 4589 | 0.0023710139 | 0.026081152 | 11 |
|  |  | RAFL04-17-H16 | At3g13110 / serine acetyltransferase (Sat-1) | |  |  |  |  |  | | --- | --- | --- | --- | --- | |  |  |  |  |  | | EC:2.3.1.30  serine O-acetyltransferase | | | | | | |
|  |  | RAFL07-10-P11 | At3g22890 / ATP sulfurylase -related | |  |  |  |  |  | | --- | --- | --- | --- | --- | |  |  |  |  |  | | EC:2.7.7.4  ATP sulfurylase | | | | | | |
|  |  | RAFL07-12-E10 | At3g22890 / ATP sulfurylase -related | |  |  |  |  |  | | --- | --- | --- | --- | --- | |  |  |  |  |  | | EC:2.7.7.4  ATP sulfurylase | | | | | | |
|  | lipoxygenase pathway | |  |  | 2 | 51 | 5 | 4605 | 0.0025667707 | 0.015400624 | 6 |
|  |  | RAFL05-12-G03 | At5g42650 / allene oxide synthase / cytochrome P450 74A | |  |  |  |  |  | | --- | --- | --- | --- | --- | |  |  |  |  |  | | EC:4.2.1.92  allene oxide synthase | | | | | | |
|  |  | RAFL06-10-H13 | At5g42650 / allene oxide synthase / cytochrome P450 74A | |  |  |  |  |  | | --- | --- | --- | --- | --- | |  |  |  |  |  | | EC:4.2.1.92  allene oxide synthase | | | | | | |
|  | dissimilatory sulfate reduction | |  |  | 2 | 51 | 5 | 4605 | 0.0025667707 | 0.015400624 | 6 |
|  |  | RAFL07-10-P11 | At3g22890 / ATP sulfurylase -related | |  |  |  |  |  | | --- | --- | --- | --- | --- | |  |  |  |  |  | | EC:2.7.7.4  ATP sulfurylase | | | | | | |
|  |  | RAFL07-12-E10 | At3g22890 / ATP sulfurylase -related | |  |  |  |  |  | | --- | --- | --- | --- | --- | |  |  |  |  |  | | EC:2.7.7.4  ATP sulfurylase | | | | | | |
|  | jasmonic acid biosynthesis | |  |  | 2 | 51 | 7 | 4603 | 0.0043364847 | 0.034691878 | 8 |
|  |  | RAFL05-12-G03 | At5g42650 / allene oxide synthase / cytochrome P450 74A | |  |  |  |  |  | | --- | --- | --- | --- | --- | |  |  |  |  |  | | EC:4.2.1.92  allene oxide synthase | | | | | | |
|  |  | RAFL06-10-H13 | At5g42650 / allene oxide synthase / cytochrome P450 74A | |  |  |  |  |  | | --- | --- | --- | --- | --- | |  |  |  |  |  | | EC:4.2.1.92  allene oxide synthase | | | | | | |
|  | homogalacturonan degradation | |  |  | 1 | 52 | 1 | 4609 | 0.022605369 | 0.045210738 | 2 |
|  |  | RAFL06-10-O06 | At1g53840 / pectinesterase family | |  |  |  |  |  | | --- | --- | --- | --- | --- | |  |  |  |  |  | | EC:3.1.1.11  pectin methylesterase | | | | | | |
|  | GDP-mannose metabolism | |  |  | 1 | 52 | 2 | 4608 | 0.033719275 | 0.06743855 | 2 |
|  |  | RAFL09-13-E08 | At2g39770 / GDP-mannose pyrophosphorylase | |  |  |  |  |  | | --- | --- | --- | --- | --- | |  |  |  |  |  | | EC:2.7.7.22  unknown | | | | | | |
|  | glutamine biosynthesis I | |  |  | 1 | 52 | 3 | 4607 | 0.044709165 | 0.13412748 | 3 |
|  |  | RAFL04-16-N11 | At5g35630 / glutamate-ammonia ligase (EC 6.3.1.2) precursor, chloroplast (clone lambdaAtgsl1) (pir||S18600) | |  |  |  |  |  | | --- | --- | --- | --- | --- | |  |  |  |  |  | | EC:6.3.1.2  glutamate-ammonia ligase | | | | | | |
|  | ammonia assimilation cycle | |  |  | 1 | 52 | 3 | 4607 | 0.044709165 | 0.13412748 | 3 |
|  |  | RAFL04-16-N11 | At5g35630 / glutamate-ammonia ligase (EC 6.3.1.2) precursor, chloroplast (clone lambdaAtgsl1) (pir||S18600) | |  |  |  |  |  | | --- | --- | --- | --- | --- | |  |  |  |  |  | | EC:6.3.1.2  glutamate-ammonia ligase | | | | | | |
|  | ascorbate biosynthesis | |  |  | 1 | 52 | 3 | 4607 | 0.044709165 | 0.08941833 | 2 |
|  |  | RAFL09-13-E08 | At2g39770 / GDP-mannose pyrophosphorylase | |  |  |  |  |  | | --- | --- | --- | --- | --- | |  |  |  |  |  | | EC:2.7.7.13  GDP-D-mannose pyrophosphorylase | | | | | | |
| Cluster:10-1 | | |  |  | A | B | C | D | P | P' | N |
|  | trehalose biosynthesis I | |  |  | 1 | 42 | 3 | 4617 | 0.03639058 | 0.14556232 | 4 |
|  |  | RAFL05-13-B06 | At2g18700 / glycosyltransferase family 20 | |  |  |  |  |  | | --- | --- | --- | --- | --- | |  |  |  |  |  | | EC:2.4.1.15  alpha,alpha-trehalose-phosphate synthase (UDP-forming) | | | | | | |
| Cluster:7-2 | | |  |  | A | B | C | D | P | P' | N |
|  | cellulose biosynthesis | |  |  | 2 | 62 | 12 | 4587 | 0.015175772 | 0.121406175 | 8 |
|  |  | RAFL05-09-M07 | At5g64740 / cellulose synthase, catalytic subunit, putative | |  |  |  |  |  | | --- | --- | --- | --- | --- | |  |  |  |  |  | | EC:2.4.1.12  cellulose synthase (UDP-forming) | | | | | | |
|  |  | RAFL09-16-P14 | At3g03050 / cellulose synthase family | |  |  |  |  |  | | --- | --- | --- | --- | --- | |  |  |  |  |  | | EC:2.4.1.12  cellulose synthase (UDP-forming) | | | | | | |
| Cluster:1-0 | | |  |  | A | B | C | D | P | P' | N |
|  | photorespiration | |  |  | 4 | 145 | 17 | 4497 | 0.0039202836 | 0.043123122 | 11 |
|  |  | RAFL09-13-P20 | At3g14420 / glycolate oxidase -related | |  |  |  |  |  | | --- | --- | --- | --- | --- | |  |  |  |  |  | | EC:1.1.3.15  glycolate oxidase | | | | | | |
|  |  | RAFL06-14-K21 | At5g36700 / phosphoglycolate phosphatase, putative | |  |  |  |  |  | | --- | --- | --- | --- | --- | |  |  |  |  |  | | EC:3.1.3.18  phosphoglycolate phosphatase | | | | | | |
|  |  | RAFL04-13-N06 | At1g68010 / glycerate dehydrogenase (NADH-dependent hydroxypyruvate reductase) (HPR) | |  |  |  |  |  | | --- | --- | --- | --- | --- | |  |  |  |  |  | | EC:1.1.1.29  phosphoglycerate dehydrogenase | | | | | | |
|  |  | RAFL08-15-E10 | At3g14420 / glycolate oxidase -related | |  |  |  |  |  | | --- | --- | --- | --- | --- | |  |  |  |  |  | | EC:1.1.3.15  glycolate oxidase | | | | | | |
|  | methionine degradation I | |  |  | 2 | 147 | 8 | 4506 | 0.038570926 | 0.19285463 | 5 |
|  |  | RAFL02-08-J05 | At3g17390 / s-adenosylmethionine synthetase -related | |  |  |  |  |  | | --- | --- | --- | --- | --- | |  |  |  |  |  | | EC:2.5.1.6  methionine adenosyltransferase | | | | | | |
|  |  | RAFL05-12-C12 | At3g17390 / s-adenosylmethionine synthetase -related | |  |  |  |  |  | | --- | --- | --- | --- | --- | |  |  |  |  |  | | EC:2.5.1.6  methionine adenosyltransferase | | | | | | |
| Cluster:9-2 | | |  |  | A | B | C | D | P | P' | N |
|  | glutamine biosynthesis I | |  |  | 2 | 65 | 2 | 4594 | 0.0011979077 | 0.0035937233 | 3 |
|  |  | RAFL08-17-D17 | At3g53180 / nodulin / glutamate-ammonia ligase - like protein | |  |  |  |  |  | | --- | --- | --- | --- | --- | |  |  |  |  |  | | EC:6.3.1.2  glutamate-ammonia ligase | | | | | | |
|  |  | RAFL06-09-F14 | At3g53180 / nodulin / glutamate-ammonia ligase - like protein | |  |  |  |  |  | | --- | --- | --- | --- | --- | |  |  |  |  |  | | EC:6.3.1.2  glutamate-ammonia ligase | | | | | | |
|  | ammonia assimilation cycle | |  |  | 2 | 65 | 2 | 4594 | 0.0011979077 | 0.0035937233 | 3 |
|  |  | RAFL08-17-D17 | At3g53180 / nodulin / glutamate-ammonia ligase - like protein | |  |  |  |  |  | | --- | --- | --- | --- | --- | |  |  |  |  |  | | EC:6.3.1.2  glutamate-ammonia ligase | | | | | | |
|  |  | RAFL06-09-F14 | At3g53180 / nodulin / glutamate-ammonia ligase - like protein | |  |  |  |  |  | | --- | --- | --- | --- | --- | |  |  |  |  |  | | EC:6.3.1.2  glutamate-ammonia ligase | | | | | | |
|  | arginine degradation XII | |  |  | 2 | 65 | 4 | 4592 | 0.0029394836 | 0.014697418 | 5 |
|  |  | RAFL09-13-D07 | At4g34710 / arginine decarboxylase SPE2 | |  |  |  |  |  | | --- | --- | --- | --- | --- | |  |  |  |  |  | | EC:4.1.1.19  arginine decarboxylase | | | | | | |
|  |  | RAFL08-11-N01 | At4g34710 / arginine decarboxylase SPE2 | |  |  |  |  |  | | --- | --- | --- | --- | --- | |  |  |  |  |  | | EC:4.1.1.19  arginine decarboxylase | | | | | | |
|  | arginine degradation II | |  |  | 2 | 65 | 8 | 4588 | 0.008496844 | 0.06797475 | 8 |
|  |  | RAFL09-13-D07 | At4g34710 / arginine decarboxylase SPE2 | |  |  |  |  |  | | --- | --- | --- | --- | --- | |  |  |  |  |  | | EC:4.1.1.19  arginine decarboxylase | | | | | | |
|  |  | RAFL08-11-N01 | At4g34710 / arginine decarboxylase SPE2 | |  |  |  |  |  | | --- | --- | --- | --- | --- | |  |  |  |  |  | | EC:4.1.1.19  arginine decarboxylase | | | | | | |
|  | arginine degradation III | |  |  | 2 | 65 | 8 | 4588 | 0.008496844 | 0.06797475 | 8 |
|  |  | RAFL09-13-D07 | At4g34710 / arginine decarboxylase SPE2 | |  |  |  |  |  | | --- | --- | --- | --- | --- | |  |  |  |  |  | | EC:4.1.1.19  arginine decarboxylase | | | | | | |
|  |  | RAFL08-11-N01 | At4g34710 / arginine decarboxylase SPE2 | |  |  |  |  |  | | --- | --- | --- | --- | --- | |  |  |  |  |  | | EC:4.1.1.19  arginine decarboxylase | | | | | | |
|  | arginine degradation V | |  |  | 2 | 65 | 8 | 4588 | 0.008496844 | 0.06797475 | 8 |
|  |  | RAFL09-13-D07 | At4g34710 / arginine decarboxylase SPE2 | |  |  |  |  |  | | --- | --- | --- | --- | --- | |  |  |  |  |  | | EC:1.13.12.1  arginine decarboxylase | | | | | | |
|  |  | RAFL08-11-N01 | At4g34710 / arginine decarboxylase SPE2 | |  |  |  |  |  | | --- | --- | --- | --- | --- | |  |  |  |  |  | | EC:1.13.12.1  arginine decarboxylase | | | | | | |
|  | polyamine biosynthesis II | |  |  | 2 | 65 | 8 | 4588 | 0.008496844 | 0.059477907 | 7 |
|  |  | RAFL09-13-D07 | At4g34710 / arginine decarboxylase SPE2 | |  |  |  |  |  | | --- | --- | --- | --- | --- | |  |  |  |  |  | | EC:4.1.1.19  arginine decarboxylase | | | | | | |
|  |  | RAFL08-11-N01 | At4g34710 / arginine decarboxylase SPE2 | |  |  |  |  |  | | --- | --- | --- | --- | --- | |  |  |  |  |  | | EC:4.1.1.19  arginine decarboxylase | | | | | | |
|  | polyamine biosynthesis III | |  |  | 2 | 65 | 8 | 4588 | 0.008496844 | 0.059477907 | 7 |
|  |  | RAFL09-13-D07 | At4g34710 / arginine decarboxylase SPE2 | |  |  |  |  |  | | --- | --- | --- | --- | --- | |  |  |  |  |  | | EC:4.1.1.19  arginine decarboxylase | | | | | | |
|  |  | RAFL08-11-N01 | At4g34710 / arginine decarboxylase SPE2 | |  |  |  |  |  | | --- | --- | --- | --- | --- | |  |  |  |  |  | | EC:4.1.1.19  arginine decarboxylase | | | | | | |
|  | nitrate assimilation pathway | |  |  | 2 | 65 | 9 | 4587 | 0.010289272 | 0.08231418 | 8 |
|  |  | RAFL08-17-D17 | At3g53180 / nodulin / glutamate-ammonia ligase - like protein | |  |  |  |  |  | | --- | --- | --- | --- | --- | |  |  |  |  |  | | EC:6.3.1.2  glutamate-ammonia ligase | | | | | | |
|  |  | RAFL06-09-F14 | At3g53180 / nodulin / glutamate-ammonia ligase - like protein | |  |  |  |  |  | | --- | --- | --- | --- | --- | |  |  |  |  |  | | EC:6.3.1.2  glutamate-ammonia ligase | | | | | | |
|  | spermine biosynthesis I | |  |  | 2 | 65 | 9 | 4587 | 0.010289272 | 0.08231418 | 8 |
|  |  | RAFL09-13-D07 | At4g34710 / arginine decarboxylase SPE2 | |  |  |  |  |  | | --- | --- | --- | --- | --- | |  |  |  |  |  | | EC:4.1.1.19  arginine decarboxylase | | | | | | |
|  |  | RAFL08-11-N01 | At4g34710 / arginine decarboxylase SPE2 | |  |  |  |  |  | | --- | --- | --- | --- | --- | |  |  |  |  |  | | EC:4.1.1.19  arginine decarboxylase | | | | | | |
|  | polyamine biosynthesis I | |  |  | 2 | 65 | 9 | 4587 | 0.010289272 | 0.08231418 | 8 |
|  |  | RAFL09-13-D07 | At4g34710 / arginine decarboxylase SPE2 | |  |  |  |  |  | | --- | --- | --- | --- | --- | |  |  |  |  |  | | EC:4.1.1.19  arginine decarboxylase | | | | | | |
|  |  | RAFL08-11-N01 | At4g34710 / arginine decarboxylase SPE2 | |  |  |  |  |  | | --- | --- | --- | --- | --- | |  |  |  |  |  | | EC:4.1.1.19  arginine decarboxylase | | | | | | |
| Cluster:0-1 | | |  |  | A | B | C | D | P | P' | N |
|  | Calvin cycle | |  |  | 21 | 85 | 31 | 4526 | 4.2892147E-22 | 4.7181364E-21 | 11 |
|  |  | RAFL07-18-C20 | At2g21330 / fructose-bisphosphate aldolase, putative | |  |  |  |  |  | | --- | --- | --- | --- | --- | |  |  |  |  |  | | EC:4.1.2.13  fructose-bisphosphate aldolase | | | | | | |
|  |  | RAFL09-15-L04 | At3g12780 / phosphoglycerate kinase -related | |  |  |  |  |  | | --- | --- | --- | --- | --- | |  |  |  |  |  | | EC:2.7.2.3  phosphoglycerate kinase | | | | | | |
|  |  | RAFL07-14-L16 | At3g12780 / phosphoglycerate kinase -related | |  |  |  |  |  | | --- | --- | --- | --- | --- | |  |  |  |  |  | | EC:2.7.2.3  phosphoglycerate kinase | | | | | | |
|  |  | RAFL08-18-C10 | At2g21330 / fructose-bisphosphate aldolase, putative | |  |  |  |  |  | | --- | --- | --- | --- | --- | |  |  |  |  |  | | EC:4.1.2.13  fructose-bisphosphate aldolase | | | | | | |
|  |  | RAFL07-12-E12 | At2g21330 / fructose-bisphosphate aldolase, putative | |  |  |  |  |  | | --- | --- | --- | --- | --- | |  |  |  |  |  | | EC:4.1.2.13  fructose-bisphosphate aldolase | | | | | | |
|  |  | RAFL07-07-I23 | At1g56190 / phosphoglycerate kinase -related | |  |  |  |  |  | | --- | --- | --- | --- | --- | |  |  |  |  |  | | EC:2.7.2.3  phosphoglycerate kinase | | | | | | |
|  |  | RAFL04-13-J02 | At3g54050 / fructose-bisphosphatase precursor | |  |  |  |  |  | | --- | --- | --- | --- | --- | |  |  |  |  |  | | EC:3.1.3.11  fructose-bisphosphatase//phosphoric ester hydrolase | | | | | | |
|  |  | RAFL07-12-M09 | At2g21330 / fructose-bisphosphate aldolase, putative | |  |  |  |  |  | | --- | --- | --- | --- | --- | |  |  |  |  |  | | EC:4.1.2.13  fructose-bisphosphate aldolase | | | | | | |
|  |  | RAFL07-16-P05 | At3g12780 / phosphoglycerate kinase -related | |  |  |  |  |  | | --- | --- | --- | --- | --- | |  |  |  |  |  | | EC:2.7.2.3  phosphoglycerate kinase | | | | | | |
|  |  | RAFL09-06-P15 | At1g67090 / ribulose-bisphosphate carboxylase small unit -related | |  |  |  |  |  | | --- | --- | --- | --- | --- | |  |  |  |  |  | | EC:4.1.1.39  ribulose-bisphosphate carboxylase | | | | | | |
|  |  | RAFL09-18-L22 | At3g12780 / phosphoglycerate kinase -related | |  |  |  |  |  | | --- | --- | --- | --- | --- | |  |  |  |  |  | | EC:2.7.2.3  phosphoglycerate kinase | | | | | | |
|  |  | RAFL04-19-O21 | At4g38970 / fructose-bisphosphate aldolase, putative | |  |  |  |  |  | | --- | --- | --- | --- | --- | |  |  |  |  |  | | EC:4.1.2.13  fructose-bisphosphate aldolase | | | | | | |
|  |  | RAFL09-17-N23 | At2g21330 / fructose-bisphosphate aldolase, putative | |  |  |  |  |  | | --- | --- | --- | --- | --- | |  |  |  |  |  | | EC:4.1.2.13  fructose-bisphosphate aldolase | | | | | | |
|  |  | RAFL05-12-O19 | At3g55800 / sedoheptulose-bisphosphatase precursor | |  |  |  |  |  | | --- | --- | --- | --- | --- | |  |  |  |  |  | | EC:3.1.3.37  unknown | | | | | | |
|  |  | RAFL04-19-M17 | At1g32060 / phosphoribulokinase precursor | |  |  |  |  |  | | --- | --- | --- | --- | --- | |  |  |  |  |  | | EC:2.7.1.19  phosphoribulokinase | | | | | | |
|  |  | RAFL04-10-J07 | At3g04790 / ribose 5-phosphate isomerase -related | |  |  |  |  |  | | --- | --- | --- | --- | --- | |  |  |  |  |  | | EC:5.3.1.6  ribose-5-phosphate isomerase | | | | | | |
|  |  | RAFL07-18-J01 | At2g21330 / fructose-bisphosphate aldolase, putative | |  |  |  |  |  | | --- | --- | --- | --- | --- | |  |  |  |  |  | | EC:4.1.2.13  fructose-bisphosphate aldolase | | | | | | |
|  |  | RAFL09-06-K21 | At1g67090 / ribulose-bisphosphate carboxylase small unit -related | |  |  |  |  |  | | --- | --- | --- | --- | --- | |  |  |  |  |  | | EC:4.1.1.39  ribulose-bisphosphate carboxylase | | | | | | |
|  |  | RAFL03-06-F08 | At1g67090 / ribulose-bisphosphate carboxylase small unit -related | |  |  |  |  |  | | --- | --- | --- | --- | --- | |  |  |  |  |  | | EC:4.1.1.39  ribulose-bisphosphate carboxylase | | | | | | |
|  |  | RAFL11-07-D01 | At5g38410 / ribulose bisphosphate carboxylase small chain 3b precursor (RuBisCO small subunit 3b) (sp|P10798) | |  |  |  |  |  | | --- | --- | --- | --- | --- | |  |  |  |  |  | | EC:4.1.1.39  ribulose-bisphosphate carboxylase | | | | | | |
|  |  | RAFL11-02-L02 | At1g67090 / ribulose-bisphosphate carboxylase small unit -related | |  |  |  |  |  | | --- | --- | --- | --- | --- | |  |  |  |  |  | | EC:4.1.1.39  ribulose-bisphosphate carboxylase | | | | | | |
|  | gluconeogenesis | |  |  | 16 | 90 | 31 | 4526 | 1.345045E-15 | 2.152072E-14 | 16 |
|  |  | RAFL07-18-C20 | At2g21330 / fructose-bisphosphate aldolase, putative | |  |  |  |  |  | | --- | --- | --- | --- | --- | |  |  |  |  |  | | EC:4.1.2.13  fructose-bisphosphate aldolase | | | | | | |
|  |  | RAFL09-15-L04 | At3g12780 / phosphoglycerate kinase -related | |  |  |  |  |  | | --- | --- | --- | --- | --- | |  |  |  |  |  | | EC:2.7.2.3  phosphoglycerate kinase | | | | | | |
|  |  | RAFL07-14-L16 | At3g12780 / phosphoglycerate kinase -related | |  |  |  |  |  | | --- | --- | --- | --- | --- | |  |  |  |  |  | | EC:2.7.2.3  phosphoglycerate kinase | | | | | | |
|  |  | RAFL08-18-C10 | At2g21330 / fructose-bisphosphate aldolase, putative | |  |  |  |  |  | | --- | --- | --- | --- | --- | |  |  |  |  |  | | EC:4.1.2.13  fructose-bisphosphate aldolase | | | | | | |
|  |  | RAFL07-12-E12 | At2g21330 / fructose-bisphosphate aldolase, putative | |  |  |  |  |  | | --- | --- | --- | --- | --- | |  |  |  |  |  | | EC:4.1.2.13  fructose-bisphosphate aldolase | | | | | | |
|  |  | RAFL07-07-I23 | At1g56190 / phosphoglycerate kinase -related | |  |  |  |  |  | | --- | --- | --- | --- | --- | |  |  |  |  |  | | EC:2.7.2.3  phosphoglycerate kinase | | | | | | |
|  |  | RAFL04-13-J02 | At3g54050 / fructose-bisphosphatase precursor | |  |  |  |  |  | | --- | --- | --- | --- | --- | |  |  |  |  |  | | EC:3.1.3.11  fructose-bisphosphatase//phosphoric ester hydrolase | | | | | | |
|  |  | RAFL07-12-M09 | At2g21330 / fructose-bisphosphate aldolase, putative | |  |  |  |  |  | | --- | --- | --- | --- | --- | |  |  |  |  |  | | EC:4.1.2.13  fructose-bisphosphate aldolase | | | | | | |
|  |  | RAFL04-09-D24 | At1g42970 / glyceraldehyde-3-phosphate dehydrogenase | |  |  |  |  |  | | --- | --- | --- | --- | --- | |  |  |  |  |  | | EC:1.2.1.12  glyceraldehyde-3-phosphate dehydrogenase | | | | | | |
|  |  | RAFL07-16-P05 | At3g12780 / phosphoglycerate kinase -related | |  |  |  |  |  | | --- | --- | --- | --- | --- | |  |  |  |  |  | | EC:2.7.2.3  phosphoglycerate kinase | | | | | | |
|  |  | RAFL09-18-L22 | At3g12780 / phosphoglycerate kinase -related | |  |  |  |  |  | | --- | --- | --- | --- | --- | |  |  |  |  |  | | EC:2.7.2.3  phosphoglycerate kinase | | | | | | |
|  |  | RAFL04-19-O21 | At4g38970 / fructose-bisphosphate aldolase, putative | |  |  |  |  |  | | --- | --- | --- | --- | --- | |  |  |  |  |  | | EC:4.1.2.13  fructose-bisphosphate aldolase | | | | | | |
|  |  | RAFL09-17-N23 | At2g21330 / fructose-bisphosphate aldolase, putative | |  |  |  |  |  | | --- | --- | --- | --- | --- | |  |  |  |  |  | | EC:4.1.2.13  fructose-bisphosphate aldolase | | | | | | |
|  |  | RAFL04-15-A14 | At1g12900 / calcium-binding protein, calreticulin -related | |  |  |  |  |  | | --- | --- | --- | --- | --- | |  |  |  |  |  | | EC:1.2.1.12  glyceraldehyde-3-phosphate dehydrogenase | | | | | | |
|  |  | RAFL07-18-J01 | At2g21330 / fructose-bisphosphate aldolase, putative | |  |  |  |  |  | | --- | --- | --- | --- | --- | |  |  |  |  |  | | EC:4.1.2.13  fructose-bisphosphate aldolase | | | | | | |
|  |  | RAFL05-07-J06 | At1g42970 / glyceraldehyde-3-phosphate dehydrogenase | |  |  |  |  |  | | --- | --- | --- | --- | --- | |  |  |  |  |  | | EC:1.2.1.12  glyceraldehyde-3-phosphate dehydrogenase | | | | | | |
|  | glycolysis I | |  |  | 15 | 91 | 28 | 4529 | 7.293992E-15 | 1.09409884E-13 | 15 |
|  |  | RAFL07-18-C20 | At2g21330 / fructose-bisphosphate aldolase, putative | |  |  |  |  |  | | --- | --- | --- | --- | --- | |  |  |  |  |  | | EC:4.1.2.13  fructose-bisphosphate aldolase | | | | | | |
|  |  | RAFL09-15-L04 | At3g12780 / phosphoglycerate kinase -related | |  |  |  |  |  | | --- | --- | --- | --- | --- | |  |  |  |  |  | | EC:2.7.2.3  phosphoglycerate kinase | | | | | | |
|  |  | RAFL07-14-L16 | At3g12780 / phosphoglycerate kinase -related | |  |  |  |  |  | | --- | --- | --- | --- | --- | |  |  |  |  |  | | EC:2.7.2.3  phosphoglycerate kinase | | | | | | |
|  |  | RAFL08-18-C10 | At2g21330 / fructose-bisphosphate aldolase, putative | |  |  |  |  |  | | --- | --- | --- | --- | --- | |  |  |  |  |  | | EC:4.1.2.13  fructose-bisphosphate aldolase | | | | | | |
|  |  | RAFL07-12-E12 | At2g21330 / fructose-bisphosphate aldolase, putative | |  |  |  |  |  | | --- | --- | --- | --- | --- | |  |  |  |  |  | | EC:4.1.2.13  fructose-bisphosphate aldolase | | | | | | |
|  |  | RAFL07-07-I23 | At1g56190 / phosphoglycerate kinase -related | |  |  |  |  |  | | --- | --- | --- | --- | --- | |  |  |  |  |  | | EC:2.7.2.3  phosphoglycerate kinase | | | | | | |
|  |  | RAFL07-12-M09 | At2g21330 / fructose-bisphosphate aldolase, putative | |  |  |  |  |  | | --- | --- | --- | --- | --- | |  |  |  |  |  | | EC:4.1.2.13  fructose-bisphosphate aldolase | | | | | | |
|  |  | RAFL04-09-D24 | At1g42970 / glyceraldehyde-3-phosphate dehydrogenase | |  |  |  |  |  | | --- | --- | --- | --- | --- | |  |  |  |  |  | | EC:1.2.1.12  glyceraldehyde-3-phosphate dehydrogenase | | | | | | |
|  |  | RAFL07-16-P05 | At3g12780 / phosphoglycerate kinase -related | |  |  |  |  |  | | --- | --- | --- | --- | --- | |  |  |  |  |  | | EC:2.7.2.3  phosphoglycerate kinase | | | | | | |
|  |  | RAFL09-18-L22 | At3g12780 / phosphoglycerate kinase -related | |  |  |  |  |  | | --- | --- | --- | --- | --- | |  |  |  |  |  | | EC:2.7.2.3  phosphoglycerate kinase | | | | | | |
|  |  | RAFL04-19-O21 | At4g38970 / fructose-bisphosphate aldolase, putative | |  |  |  |  |  | | --- | --- | --- | --- | --- | |  |  |  |  |  | | EC:4.1.2.13  fructose-bisphosphate aldolase | | | | | | |
|  |  | RAFL09-17-N23 | At2g21330 / fructose-bisphosphate aldolase, putative | |  |  |  |  |  | | --- | --- | --- | --- | --- | |  |  |  |  |  | | EC:4.1.2.13  fructose-bisphosphate aldolase | | | | | | |
|  |  | RAFL04-15-A14 | At1g12900 / calcium-binding protein, calreticulin -related | |  |  |  |  |  | | --- | --- | --- | --- | --- | |  |  |  |  |  | | EC:1.2.1.12  glyceraldehyde-3-phosphate dehydrogenase | | | | | | |
|  |  | RAFL07-18-J01 | At2g21330 / fructose-bisphosphate aldolase, putative | |  |  |  |  |  | | --- | --- | --- | --- | --- | |  |  |  |  |  | | EC:4.1.2.13  fructose-bisphosphate aldolase | | | | | | |
|  |  | RAFL05-07-J06 | At1g42970 / glyceraldehyde-3-phosphate dehydrogenase | |  |  |  |  |  | | --- | --- | --- | --- | --- | |  |  |  |  |  | | EC:1.2.1.12  glyceraldehyde-3-phosphate dehydrogenase | | | | | | |
|  | glycolysis IV | |  |  | 15 | 91 | 29 | 4528 | 1.08628985E-14 | 1.7380638E-13 | 16 |
|  |  | RAFL07-18-C20 | At2g21330 / fructose-bisphosphate aldolase, putative | |  |  |  |  |  | | --- | --- | --- | --- | --- | |  |  |  |  |  | | EC:4.1.2.13  fructose-bisphosphate aldolase | | | | | | |
|  |  | RAFL09-15-L04 | At3g12780 / phosphoglycerate kinase -related | |  |  |  |  |  | | --- | --- | --- | --- | --- | |  |  |  |  |  | | EC:2.7.2.3  phosphoglycerate kinase | | | | | | |
|  |  | RAFL07-14-L16 | At3g12780 / phosphoglycerate kinase -related | |  |  |  |  |  | | --- | --- | --- | --- | --- | |  |  |  |  |  | | EC:2.7.2.3  phosphoglycerate kinase | | | | | | |
|  |  | RAFL08-18-C10 | At2g21330 / fructose-bisphosphate aldolase, putative | |  |  |  |  |  | | --- | --- | --- | --- | --- | |  |  |  |  |  | | EC:4.1.2.13  fructose-bisphosphate aldolase | | | | | | |
|  |  | RAFL07-12-E12 | At2g21330 / fructose-bisphosphate aldolase, putative | |  |  |  |  |  | | --- | --- | --- | --- | --- | |  |  |  |  |  | | EC:4.1.2.13  fructose-bisphosphate aldolase | | | | | | |
|  |  | RAFL07-07-I23 | At1g56190 / phosphoglycerate kinase -related | |  |  |  |  |  | | --- | --- | --- | --- | --- | |  |  |  |  |  | | EC:2.7.2.3  phosphoglycerate kinase | | | | | | |
|  |  | RAFL07-12-M09 | At2g21330 / fructose-bisphosphate aldolase, putative | |  |  |  |  |  | | --- | --- | --- | --- | --- | |  |  |  |  |  | | EC:4.1.2.13  fructose-bisphosphate aldolase | | | | | | |
|  |  | RAFL04-09-D24 | At1g42970 / glyceraldehyde-3-phosphate dehydrogenase | |  |  |  |  |  | | --- | --- | --- | --- | --- | |  |  |  |  |  | | EC:1.2.1.12 ,EC:1.2.1.9  glyceraldehyde-3-phosphate dehydrogenase  glyceraldehyde-3-phosphate dehydrogenase (NADP+)//glyceraldehyde-3-phosphate dehydrogenase | | | | | | |
|  |  | RAFL07-16-P05 | At3g12780 / phosphoglycerate kinase -related | |  |  |  |  |  | | --- | --- | --- | --- | --- | |  |  |  |  |  | | EC:2.7.2.3  phosphoglycerate kinase | | | | | | |
|  |  | RAFL09-18-L22 | At3g12780 / phosphoglycerate kinase -related | |  |  |  |  |  | | --- | --- | --- | --- | --- | |  |  |  |  |  | | EC:2.7.2.3  phosphoglycerate kinase | | | | | | |
|  |  | RAFL04-19-O21 | At4g38970 / fructose-bisphosphate aldolase, putative | |  |  |  |  |  | | --- | --- | --- | --- | --- | |  |  |  |  |  | | EC:4.1.2.13  fructose-bisphosphate aldolase | | | | | | |
|  |  | RAFL09-17-N23 | At2g21330 / fructose-bisphosphate aldolase, putative | |  |  |  |  |  | | --- | --- | --- | --- | --- | |  |  |  |  |  | | EC:4.1.2.13  fructose-bisphosphate aldolase | | | | | | |
|  |  | RAFL04-15-A14 | At1g12900 / calcium-binding protein, calreticulin -related | |  |  |  |  |  | | --- | --- | --- | --- | --- | |  |  |  |  |  | | EC:1.2.1.12  glyceraldehyde-3-phosphate dehydrogenase | | | | | | |
|  |  | RAFL07-18-J01 | At2g21330 / fructose-bisphosphate aldolase, putative | |  |  |  |  |  | | --- | --- | --- | --- | --- | |  |  |  |  |  | | EC:4.1.2.13  fructose-bisphosphate aldolase | | | | | | |
|  |  | RAFL05-07-J06 | At1g42970 / glyceraldehyde-3-phosphate dehydrogenase | |  |  |  |  |  | | --- | --- | --- | --- | --- | |  |  |  |  |  | | EC:1.2.1.12 ,EC:1.2.1.9  glyceraldehyde-3-phosphate dehydrogenase  glyceraldehyde-3-phosphate dehydrogenase (NADP+)//glyceraldehyde-3-phosphate dehydrogenase | | | | | | |
|  | sorbitol fermentation | |  |  | 15 | 91 | 29 | 4528 | 1.08628985E-14 | 1.6294348E-13 | 15 |
|  |  | RAFL07-18-C20 | At2g21330 / fructose-bisphosphate aldolase, putative | |  |  |  |  |  | | --- | --- | --- | --- | --- | |  |  |  |  |  | | EC:4.1.2.13  fructose-bisphosphate aldolase | | | | | | |
|  |  | RAFL09-15-L04 | At3g12780 / phosphoglycerate kinase -related | |  |  |  |  |  | | --- | --- | --- | --- | --- | |  |  |  |  |  | | EC:2.7.2.3  phosphoglycerate kinase | | | | | | |
|  |  | RAFL07-14-L16 | At3g12780 / phosphoglycerate kinase -related | |  |  |  |  |  | | --- | --- | --- | --- | --- | |  |  |  |  |  | | EC:2.7.2.3  phosphoglycerate kinase | | | | | | |
|  |  | RAFL08-18-C10 | At2g21330 / fructose-bisphosphate aldolase, putative | |  |  |  |  |  | | --- | --- | --- | --- | --- | |  |  |  |  |  | | EC:4.1.2.13  fructose-bisphosphate aldolase | | | | | | |
|  |  | RAFL07-12-E12 | At2g21330 / fructose-bisphosphate aldolase, putative | |  |  |  |  |  | | --- | --- | --- | --- | --- | |  |  |  |  |  | | EC:4.1.2.13  fructose-bisphosphate aldolase | | | | | | |
|  |  | RAFL07-07-I23 | At1g56190 / phosphoglycerate kinase -related | |  |  |  |  |  | | --- | --- | --- | --- | --- | |  |  |  |  |  | | EC:2.7.2.3  phosphoglycerate kinase | | | | | | |
|  |  | RAFL07-12-M09 | At2g21330 / fructose-bisphosphate aldolase, putative | |  |  |  |  |  | | --- | --- | --- | --- | --- | |  |  |  |  |  | | EC:4.1.2.13  fructose-bisphosphate aldolase | | | | | | |
|  |  | RAFL04-09-D24 | At1g42970 / glyceraldehyde-3-phosphate dehydrogenase | |  |  |  |  |  | | --- | --- | --- | --- | --- | |  |  |  |  |  | | EC:1.2.1.12  glyceraldehyde-3-phosphate dehydrogenase | | | | | | |
|  |  | RAFL07-16-P05 | At3g12780 / phosphoglycerate kinase -related | |  |  |  |  |  | | --- | --- | --- | --- | --- | |  |  |  |  |  | | EC:2.7.2.3  phosphoglycerate kinase | | | | | | |
|  |  | RAFL09-18-L22 | At3g12780 / phosphoglycerate kinase -related | |  |  |  |  |  | | --- | --- | --- | --- | --- | |  |  |  |  |  | | EC:2.7.2.3  phosphoglycerate kinase | | | | | | |
|  |  | RAFL04-19-O21 | At4g38970 / fructose-bisphosphate aldolase, putative | |  |  |  |  |  | | --- | --- | --- | --- | --- | |  |  |  |  |  | | EC:4.1.2.13  fructose-bisphosphate aldolase | | | | | | |
|  |  | RAFL09-17-N23 | At2g21330 / fructose-bisphosphate aldolase, putative | |  |  |  |  |  | | --- | --- | --- | --- | --- | |  |  |  |  |  | | EC:4.1.2.13  fructose-bisphosphate aldolase | | | | | | |
|  |  | RAFL04-15-A14 | At1g12900 / calcium-binding protein, calreticulin -related | |  |  |  |  |  | | --- | --- | --- | --- | --- | |  |  |  |  |  | | EC:1.2.1.12  glyceraldehyde-3-phosphate dehydrogenase | | | | | | |
|  |  | RAFL07-18-J01 | At2g21330 / fructose-bisphosphate aldolase, putative | |  |  |  |  |  | | --- | --- | --- | --- | --- | |  |  |  |  |  | | EC:4.1.2.13  fructose-bisphosphate aldolase | | | | | | |
|  |  | RAFL05-07-J06 | At1g42970 / glyceraldehyde-3-phosphate dehydrogenase | |  |  |  |  |  | | --- | --- | --- | --- | --- | |  |  |  |  |  | | EC:1.2.1.12  glyceraldehyde-3-phosphate dehydrogenase | | | | | | |
|  | fructose degradation (anaerobic) | |  |  | 15 | 91 | 29 | 4528 | 1.08628985E-14 | 1.6294348E-13 | 15 |
|  |  | RAFL07-18-C20 | At2g21330 / fructose-bisphosphate aldolase, putative | |  |  |  |  |  | | --- | --- | --- | --- | --- | |  |  |  |  |  | | EC:4.1.2.13  fructose-bisphosphate aldolase | | | | | | |
|  |  | RAFL09-15-L04 | At3g12780 / phosphoglycerate kinase -related | |  |  |  |  |  | | --- | --- | --- | --- | --- | |  |  |  |  |  | | EC:2.7.2.3  phosphoglycerate kinase | | | | | | |
|  |  | RAFL07-14-L16 | At3g12780 / phosphoglycerate kinase -related | |  |  |  |  |  | | --- | --- | --- | --- | --- | |  |  |  |  |  | | EC:2.7.2.3  phosphoglycerate kinase | | | | | | |
|  |  | RAFL08-18-C10 | At2g21330 / fructose-bisphosphate aldolase, putative | |  |  |  |  |  | | --- | --- | --- | --- | --- | |  |  |  |  |  | | EC:4.1.2.13  fructose-bisphosphate aldolase | | | | | | |
|  |  | RAFL07-12-E12 | At2g21330 / fructose-bisphosphate aldolase, putative | |  |  |  |  |  | | --- | --- | --- | --- | --- | |  |  |  |  |  | | EC:4.1.2.13  fructose-bisphosphate aldolase | | | | | | |
|  |  | RAFL07-07-I23 | At1g56190 / phosphoglycerate kinase -related | |  |  |  |  |  | | --- | --- | --- | --- | --- | |  |  |  |  |  | | EC:2.7.2.3  phosphoglycerate kinase | | | | | | |
|  |  | RAFL07-12-M09 | At2g21330 / fructose-bisphosphate aldolase, putative | |  |  |  |  |  | | --- | --- | --- | --- | --- | |  |  |  |  |  | | EC:4.1.2.13  fructose-bisphosphate aldolase | | | | | | |
|  |  | RAFL04-09-D24 | At1g42970 / glyceraldehyde-3-phosphate dehydrogenase | |  |  |  |  |  | | --- | --- | --- | --- | --- | |  |  |  |  |  | | EC:1.2.1.12  glyceraldehyde-3-phosphate dehydrogenase | | | | | | |
|  |  | RAFL07-16-P05 | At3g12780 / phosphoglycerate kinase -related | |  |  |  |  |  | | --- | --- | --- | --- | --- | |  |  |  |  |  | | EC:2.7.2.3  phosphoglycerate kinase | | | | | | |
|  |  | RAFL09-18-L22 | At3g12780 / phosphoglycerate kinase -related | |  |  |  |  |  | | --- | --- | --- | --- | --- | |  |  |  |  |  | | EC:2.7.2.3  phosphoglycerate kinase | | | | | | |
|  |  | RAFL04-19-O21 | At4g38970 / fructose-bisphosphate aldolase, putative | |  |  |  |  |  | | --- | --- | --- | --- | --- | |  |  |  |  |  | | EC:4.1.2.13  fructose-bisphosphate aldolase | | | | | | |
|  |  | RAFL09-17-N23 | At2g21330 / fructose-bisphosphate aldolase, putative | |  |  |  |  |  | | --- | --- | --- | --- | --- | |  |  |  |  |  | | EC:4.1.2.13  fructose-bisphosphate aldolase | | | | | | |
|  |  | RAFL04-15-A14 | At1g12900 / calcium-binding protein, calreticulin -related | |  |  |  |  |  | | --- | --- | --- | --- | --- | |  |  |  |  |  | | EC:1.2.1.12  glyceraldehyde-3-phosphate dehydrogenase | | | | | | |
|  |  | RAFL07-18-J01 | At2g21330 / fructose-bisphosphate aldolase, putative | |  |  |  |  |  | | --- | --- | --- | --- | --- | |  |  |  |  |  | | EC:4.1.2.13  fructose-bisphosphate aldolase | | | | | | |
|  |  | RAFL05-07-J06 | At1g42970 / glyceraldehyde-3-phosphate dehydrogenase | |  |  |  |  |  | | --- | --- | --- | --- | --- | |  |  |  |  |  | | EC:1.2.1.12  glyceraldehyde-3-phosphate dehydrogenase | | | | | | |
|  | acetate fermentation | |  |  | 15 | 91 | 36 | 4521 | 1.3226566E-13 | 2.645313E-12 | 20 |
|  |  | RAFL07-18-C20 | At2g21330 / fructose-bisphosphate aldolase, putative | |  |  |  |  |  | | --- | --- | --- | --- | --- | |  |  |  |  |  | | EC:4.1.2.13  fructose-bisphosphate aldolase | | | | | | |
|  |  | RAFL09-15-L04 | At3g12780 / phosphoglycerate kinase -related | |  |  |  |  |  | | --- | --- | --- | --- | --- | |  |  |  |  |  | | EC:2.7.2.3  phosphoglycerate kinase | | | | | | |
|  |  | RAFL07-14-L16 | At3g12780 / phosphoglycerate kinase -related | |  |  |  |  |  | | --- | --- | --- | --- | --- | |  |  |  |  |  | | EC:2.7.2.3  phosphoglycerate kinase | | | | | | |
|  |  | RAFL08-18-C10 | At2g21330 / fructose-bisphosphate aldolase, putative | |  |  |  |  |  | | --- | --- | --- | --- | --- | |  |  |  |  |  | | EC:4.1.2.13  fructose-bisphosphate aldolase | | | | | | |
|  |  | RAFL07-12-E12 | At2g21330 / fructose-bisphosphate aldolase, putative | |  |  |  |  |  | | --- | --- | --- | --- | --- | |  |  |  |  |  | | EC:4.1.2.13  fructose-bisphosphate aldolase | | | | | | |
|  |  | RAFL07-07-I23 | At1g56190 / phosphoglycerate kinase -related | |  |  |  |  |  | | --- | --- | --- | --- | --- | |  |  |  |  |  | | EC:2.7.2.3  phosphoglycerate kinase | | | | | | |
|  |  | RAFL07-12-M09 | At2g21330 / fructose-bisphosphate aldolase, putative | |  |  |  |  |  | | --- | --- | --- | --- | --- | |  |  |  |  |  | | EC:4.1.2.13  fructose-bisphosphate aldolase | | | | | | |
|  |  | RAFL04-09-D24 | At1g42970 / glyceraldehyde-3-phosphate dehydrogenase | |  |  |  |  |  | | --- | --- | --- | --- | --- | |  |  |  |  |  | | EC:1.2.1.12  glyceraldehyde-3-phosphate dehydrogenase | | | | | | |
|  |  | RAFL07-16-P05 | At3g12780 / phosphoglycerate kinase -related | |  |  |  |  |  | | --- | --- | --- | --- | --- | |  |  |  |  |  | | EC:2.7.2.3  phosphoglycerate kinase | | | | | | |
|  |  | RAFL09-18-L22 | At3g12780 / phosphoglycerate kinase -related | |  |  |  |  |  | | --- | --- | --- | --- | --- | |  |  |  |  |  | | EC:2.7.2.3  phosphoglycerate kinase | | | | | | |
|  |  | RAFL04-19-O21 | At4g38970 / fructose-bisphosphate aldolase, putative | |  |  |  |  |  | | --- | --- | --- | --- | --- | |  |  |  |  |  | | EC:4.1.2.13  fructose-bisphosphate aldolase | | | | | | |
|  |  | RAFL09-17-N23 | At2g21330 / fructose-bisphosphate aldolase, putative | |  |  |  |  |  | | --- | --- | --- | --- | --- | |  |  |  |  |  | | EC:4.1.2.13  fructose-bisphosphate aldolase | | | | | | |
|  |  | RAFL04-15-A14 | At1g12900 / calcium-binding protein, calreticulin -related | |  |  |  |  |  | | --- | --- | --- | --- | --- | |  |  |  |  |  | | EC:1.2.1.12  glyceraldehyde-3-phosphate dehydrogenase | | | | | | |
|  |  | RAFL07-18-J01 | At2g21330 / fructose-bisphosphate aldolase, putative | |  |  |  |  |  | | --- | --- | --- | --- | --- | |  |  |  |  |  | | EC:4.1.2.13  fructose-bisphosphate aldolase | | | | | | |
|  |  | RAFL05-07-J06 | At1g42970 / glyceraldehyde-3-phosphate dehydrogenase | |  |  |  |  |  | | --- | --- | --- | --- | --- | |  |  |  |  |  | | EC:1.2.1.12  glyceraldehyde-3-phosphate dehydrogenase | | | | | | |
|  | mannitol degradation | |  |  | 7 | 99 | 7 | 4550 | 7.745682E-9 | 3.8728412E-8 | 5 |
|  |  | RAFL07-18-C20 | At2g21330 / fructose-bisphosphate aldolase, putative | |  |  |  |  |  | | --- | --- | --- | --- | --- | |  |  |  |  |  | | EC:4.1.2.13  fructose-bisphosphate aldolase | | | | | | |
|  |  | RAFL08-18-C10 | At2g21330 / fructose-bisphosphate aldolase, putative | |  |  |  |  |  | | --- | --- | --- | --- | --- | |  |  |  |  |  | | EC:4.1.2.13  fructose-bisphosphate aldolase | | | | | | |
|  |  | RAFL07-12-E12 | At2g21330 / fructose-bisphosphate aldolase, putative | |  |  |  |  |  | | --- | --- | --- | --- | --- | |  |  |  |  |  | | EC:4.1.2.13  fructose-bisphosphate aldolase | | | | | | |
|  |  | RAFL07-12-M09 | At2g21330 / fructose-bisphosphate aldolase, putative | |  |  |  |  |  | | --- | --- | --- | --- | --- | |  |  |  |  |  | | EC:4.1.2.13  fructose-bisphosphate aldolase | | | | | | |
|  |  | RAFL04-19-O21 | At4g38970 / fructose-bisphosphate aldolase, putative | |  |  |  |  |  | | --- | --- | --- | --- | --- | |  |  |  |  |  | | EC:4.1.2.13  fructose-bisphosphate aldolase | | | | | | |
|  |  | RAFL09-17-N23 | At2g21330 / fructose-bisphosphate aldolase, putative | |  |  |  |  |  | | --- | --- | --- | --- | --- | |  |  |  |  |  | | EC:4.1.2.13  fructose-bisphosphate aldolase | | | | | | |
|  |  | RAFL07-18-J01 | At2g21330 / fructose-bisphosphate aldolase, putative | |  |  |  |  |  | | --- | --- | --- | --- | --- | |  |  |  |  |  | | EC:4.1.2.13  fructose-bisphosphate aldolase | | | | | | |
|  | sorbitol degradation | |  |  | 7 | 99 | 7 | 4550 | 7.745682E-9 | 3.8728412E-8 | 5 |
|  |  | RAFL07-18-C20 | At2g21330 / fructose-bisphosphate aldolase, putative | |  |  |  |  |  | | --- | --- | --- | --- | --- | |  |  |  |  |  | | EC:4.1.2.13  fructose-bisphosphate aldolase | | | | | | |
|  |  | RAFL08-18-C10 | At2g21330 / fructose-bisphosphate aldolase, putative | |  |  |  |  |  | | --- | --- | --- | --- | --- | |  |  |  |  |  | | EC:4.1.2.13  fructose-bisphosphate aldolase | | | | | | |
|  |  | RAFL07-12-E12 | At2g21330 / fructose-bisphosphate aldolase, putative | |  |  |  |  |  | | --- | --- | --- | --- | --- | |  |  |  |  |  | | EC:4.1.2.13  fructose-bisphosphate aldolase | | | | | | |
|  |  | RAFL07-12-M09 | At2g21330 / fructose-bisphosphate aldolase, putative | |  |  |  |  |  | | --- | --- | --- | --- | --- | |  |  |  |  |  | | EC:4.1.2.13  fructose-bisphosphate aldolase | | | | | | |
|  |  | RAFL04-19-O21 | At4g38970 / fructose-bisphosphate aldolase, putative | |  |  |  |  |  | | --- | --- | --- | --- | --- | |  |  |  |  |  | | EC:4.1.2.13  fructose-bisphosphate aldolase | | | | | | |
|  |  | RAFL09-17-N23 | At2g21330 / fructose-bisphosphate aldolase, putative | |  |  |  |  |  | | --- | --- | --- | --- | --- | |  |  |  |  |  | | EC:4.1.2.13  fructose-bisphosphate aldolase | | | | | | |
|  |  | RAFL07-18-J01 | At2g21330 / fructose-bisphosphate aldolase, putative | |  |  |  |  |  | | --- | --- | --- | --- | --- | |  |  |  |  |  | | EC:4.1.2.13  fructose-bisphosphate aldolase | | | | | | |
|  | glyceraldehyde 3-phosphate degradation | |  |  | 8 | 98 | 16 | 4541 | 2.9754789E-8 | 3.8681225E-7 | 13 |
|  |  | RAFL09-15-L04 | At3g12780 / phosphoglycerate kinase -related | |  |  |  |  |  | | --- | --- | --- | --- | --- | |  |  |  |  |  | | EC:2.7.2.3  phosphoglycerate kinase | | | | | | |
|  |  | RAFL07-14-L16 | At3g12780 / phosphoglycerate kinase -related | |  |  |  |  |  | | --- | --- | --- | --- | --- | |  |  |  |  |  | | EC:2.7.2.3  phosphoglycerate kinase | | | | | | |
|  |  | RAFL07-07-I23 | At1g56190 / phosphoglycerate kinase -related | |  |  |  |  |  | | --- | --- | --- | --- | --- | |  |  |  |  |  | | EC:2.7.2.3  phosphoglycerate kinase | | | | | | |
|  |  | RAFL04-09-D24 | At1g42970 / glyceraldehyde-3-phosphate dehydrogenase | |  |  |  |  |  | | --- | --- | --- | --- | --- | |  |  |  |  |  | | EC:1.2.1.12  glyceraldehyde-3-phosphate dehydrogenase | | | | | | |
|  |  | RAFL07-16-P05 | At3g12780 / phosphoglycerate kinase -related | |  |  |  |  |  | | --- | --- | --- | --- | --- | |  |  |  |  |  | | EC:2.7.2.3  phosphoglycerate kinase | | | | | | |
|  |  | RAFL09-18-L22 | At3g12780 / phosphoglycerate kinase -related | |  |  |  |  |  | | --- | --- | --- | --- | --- | |  |  |  |  |  | | EC:2.7.2.3  phosphoglycerate kinase | | | | | | |
|  |  | RAFL04-15-A14 | At1g12900 / calcium-binding protein, calreticulin -related | |  |  |  |  |  | | --- | --- | --- | --- | --- | |  |  |  |  |  | | EC:1.2.1.12  glyceraldehyde-3-phosphate dehydrogenase | | | | | | |
|  |  | RAFL05-07-J06 | At1g42970 / glyceraldehyde-3-phosphate dehydrogenase | |  |  |  |  |  | | --- | --- | --- | --- | --- | |  |  |  |  |  | | EC:1.2.1.12  glyceraldehyde-3-phosphate dehydrogenase | | | | | | |
|  | glycerol degradation II | |  |  | 8 | 98 | 24 | 4533 | 3.6590941E-7 | 5.4886414E-6 | 15 |
|  |  | RAFL09-15-L04 | At3g12780 / phosphoglycerate kinase -related | |  |  |  |  |  | | --- | --- | --- | --- | --- | |  |  |  |  |  | | EC:2.7.2.3  phosphoglycerate kinase | | | | | | |
|  |  | RAFL07-14-L16 | At3g12780 / phosphoglycerate kinase -related | |  |  |  |  |  | | --- | --- | --- | --- | --- | |  |  |  |  |  | | EC:2.7.2.3  phosphoglycerate kinase | | | | | | |
|  |  | RAFL07-07-I23 | At1g56190 / phosphoglycerate kinase -related | |  |  |  |  |  | | --- | --- | --- | --- | --- | |  |  |  |  |  | | EC:2.7.2.3  phosphoglycerate kinase | | | | | | |
|  |  | RAFL04-09-D24 | At1g42970 / glyceraldehyde-3-phosphate dehydrogenase | |  |  |  |  |  | | --- | --- | --- | --- | --- | |  |  |  |  |  | | EC:1.2.1.12  glyceraldehyde-3-phosphate dehydrogenase | | | | | | |
|  |  | RAFL07-16-P05 | At3g12780 / phosphoglycerate kinase -related | |  |  |  |  |  | | --- | --- | --- | --- | --- | |  |  |  |  |  | | EC:2.7.2.3  phosphoglycerate kinase | | | | | | |
|  |  | RAFL09-18-L22 | At3g12780 / phosphoglycerate kinase -related | |  |  |  |  |  | | --- | --- | --- | --- | --- | |  |  |  |  |  | | EC:2.7.2.3  phosphoglycerate kinase | | | | | | |
|  |  | RAFL04-15-A14 | At1g12900 / calcium-binding protein, calreticulin -related | |  |  |  |  |  | | --- | --- | --- | --- | --- | |  |  |  |  |  | | EC:1.2.1.12  glyceraldehyde-3-phosphate dehydrogenase | | | | | | |
|  |  | RAFL05-07-J06 | At1g42970 / glyceraldehyde-3-phosphate dehydrogenase | |  |  |  |  |  | | --- | --- | --- | --- | --- | |  |  |  |  |  | | EC:1.2.1.12  glyceraldehyde-3-phosphate dehydrogenase | | | | | | |
|  | glycine degradation I | |  |  | 2 | 104 | 10 | 4547 | 0.029122537 | 0.14561269 | 5 |
|  |  | RAFL06-13-H16 | At1g11860 / aminomethyltransferase-related precursor protein | |  |  |  |  |  | | --- | --- | --- | --- | --- | |  |  |  |  |  | | EC:2.1.2.10  aminomethyltransferase | | | | | | |
|  |  | RAFL05-04-M03 | At1g32470 / glycine cleavage system H protein precursor -related | |  |  |  |  |  | | --- | --- | --- | --- | --- | |  |  |  |  |  | | EC:1.4.4.2  glycine dehydrogenase (decarboxylating) | | | | | | |
|  | homogalacturonan degradation | |  |  | 1 | 105 | 1 | 4556 | 0.044952307 | 0.089904614 | 2 |
|  |  | RAFL09-12-M04 | At3g14310 / pectin methylesterase -related | |  |  |  |  |  | | --- | --- | --- | --- | --- | |  |  |  |  |  | | EC:3.1.1.11  pectin methylesterase | | | | | | |
| Cluster:9-0 | | |  |  | A | B | C | D | P | P' | N |
|  | fatty acid oxidation pathway II | |  |  | 4 | 28 | 2 | 4629 | 2.7153972E-8 | 8.146192E-8 | 3 |
|  |  | RAFL05-21-E06 | At1g54100 / aldehyde dehydrogenase, putative (ALDH) | |  |  |  |  |  | | --- | --- | --- | --- | --- | |  |  |  |  |  | | EC:1.2.1.3  unknown | | | | | | |
|  |  | RAFL04-09-D07 | At1g54100 / aldehyde dehydrogenase, putative (ALDH) | |  |  |  |  |  | | --- | --- | --- | --- | --- | |  |  |  |  |  | | EC:1.2.1.3  unknown | | | | | | |
|  |  | RAFL08-15-L09 | At1g54100 / aldehyde dehydrogenase, putative (ALDH) | |  |  |  |  |  | | --- | --- | --- | --- | --- | |  |  |  |  |  | | EC:1.2.1.3  unknown | | | | | | |
|  |  | RAFL08-09-C23 | At1g54100 / aldehyde dehydrogenase, putative (ALDH) | |  |  |  |  |  | | --- | --- | --- | --- | --- | |  |  |  |  |  | | EC:1.2.1.3  unknown | | | | | | |
|  | fatty acid oxidation pathway III | |  |  | 4 | 28 | 2 | 4629 | 2.7153972E-8 | 8.146192E-8 | 3 |
|  |  | RAFL05-21-E06 | At1g54100 / aldehyde dehydrogenase, putative (ALDH) | |  |  |  |  |  | | --- | --- | --- | --- | --- | |  |  |  |  |  | | EC:1.2.1.3  unknown | | | | | | |
|  |  | RAFL04-09-D07 | At1g54100 / aldehyde dehydrogenase, putative (ALDH) | |  |  |  |  |  | | --- | --- | --- | --- | --- | |  |  |  |  |  | | EC:1.2.1.3  unknown | | | | | | |
|  |  | RAFL08-15-L09 | At1g54100 / aldehyde dehydrogenase, putative (ALDH) | |  |  |  |  |  | | --- | --- | --- | --- | --- | |  |  |  |  |  | | EC:1.2.1.3  unknown | | | | | | |
|  |  | RAFL08-09-C23 | At1g54100 / aldehyde dehydrogenase, putative (ALDH) | |  |  |  |  |  | | --- | --- | --- | --- | --- | |  |  |  |  |  | | EC:1.2.1.3  unknown | | | | | | |
|  | proline biosynthesis I | |  |  | 4 | 28 | 5 | 4626 | 2.2481943E-7 | 1.1240971E-6 | 5 |
|  |  | RAFL05-21-E06 | At1g54100 / aldehyde dehydrogenase, putative (ALDH) | |  |  |  |  |  | | --- | --- | --- | --- | --- | |  |  |  |  |  | | EC:1.2.1.41  1-pyrroline-5-carboxylate dehydrogenase | | | | | | |
|  |  | RAFL04-09-D07 | At1g54100 / aldehyde dehydrogenase, putative (ALDH) | |  |  |  |  |  | | --- | --- | --- | --- | --- | |  |  |  |  |  | | EC:1.2.1.41  1-pyrroline-5-carboxylate dehydrogenase | | | | | | |
|  |  | RAFL08-15-L09 | At1g54100 / aldehyde dehydrogenase, putative (ALDH) | |  |  |  |  |  | | --- | --- | --- | --- | --- | |  |  |  |  |  | | EC:1.2.1.41  1-pyrroline-5-carboxylate dehydrogenase | | | | | | |
|  |  | RAFL08-09-C23 | At1g54100 / aldehyde dehydrogenase, putative (ALDH) | |  |  |  |  |  | | --- | --- | --- | --- | --- | |  |  |  |  |  | | EC:1.2.1.41  1-pyrroline-5-carboxylate dehydrogenase | | | | | | |
|  | 4-hydroxyproline degradation | |  |  | 4 | 28 | 8 | 4623 | 8.7054286E-7 | 7.834886E-6 | 9 |
|  |  | RAFL05-21-E06 | At1g54100 / aldehyde dehydrogenase, putative (ALDH) | |  |  |  |  |  | | --- | --- | --- | --- | --- | |  |  |  |  |  | | EC:1.5.1.12  1-pyrroline-5-carboxylate dehydrogenase//aldehyde dehydrogenase | | | | | | |
|  |  | RAFL04-09-D07 | At1g54100 / aldehyde dehydrogenase, putative (ALDH) | |  |  |  |  |  | | --- | --- | --- | --- | --- | |  |  |  |  |  | | EC:1.5.1.12  1-pyrroline-5-carboxylate dehydrogenase//aldehyde dehydrogenase | | | | | | |
|  |  | RAFL08-15-L09 | At1g54100 / aldehyde dehydrogenase, putative (ALDH) | |  |  |  |  |  | | --- | --- | --- | --- | --- | |  |  |  |  |  | | EC:1.5.1.12  1-pyrroline-5-carboxylate dehydrogenase//aldehyde dehydrogenase | | | | | | |
|  |  | RAFL08-09-C23 | At1g54100 / aldehyde dehydrogenase, putative (ALDH) | |  |  |  |  |  | | --- | --- | --- | --- | --- | |  |  |  |  |  | | EC:1.5.1.12  1-pyrroline-5-carboxylate dehydrogenase//aldehyde dehydrogenase | | | | | | |
|  | proline degradation I | |  |  | 4 | 28 | 8 | 4623 | 8.7054286E-7 | 7.834886E-6 | 9 |
|  |  | RAFL05-21-E06 | At1g54100 / aldehyde dehydrogenase, putative (ALDH) | |  |  |  |  |  | | --- | --- | --- | --- | --- | |  |  |  |  |  | | EC:1.5.1.12  1-pyrroline-5-carboxylate dehydrogenase//aldehyde dehydrogenase | | | | | | |
|  |  | RAFL04-09-D07 | At1g54100 / aldehyde dehydrogenase, putative (ALDH) | |  |  |  |  |  | | --- | --- | --- | --- | --- | |  |  |  |  |  | | EC:1.5.1.12  1-pyrroline-5-carboxylate dehydrogenase//aldehyde dehydrogenase | | | | | | |
|  |  | RAFL08-15-L09 | At1g54100 / aldehyde dehydrogenase, putative (ALDH) | |  |  |  |  |  | | --- | --- | --- | --- | --- | |  |  |  |  |  | | EC:1.5.1.12  1-pyrroline-5-carboxylate dehydrogenase//aldehyde dehydrogenase | | | | | | |
|  |  | RAFL08-09-C23 | At1g54100 / aldehyde dehydrogenase, putative (ALDH) | |  |  |  |  |  | | --- | --- | --- | --- | --- | |  |  |  |  |  | | EC:1.5.1.12  1-pyrroline-5-carboxylate dehydrogenase//aldehyde dehydrogenase | | | | | | |
|  | proline degradation II | |  |  | 4 | 28 | 8 | 4623 | 8.7054286E-7 | 7.834886E-6 | 9 |
|  |  | RAFL05-21-E06 | At1g54100 / aldehyde dehydrogenase, putative (ALDH) | |  |  |  |  |  | | --- | --- | --- | --- | --- | |  |  |  |  |  | | EC:1.5.1.12  1-pyrroline-5-carboxylate dehydrogenase//aldehyde dehydrogenase | | | | | | |
|  |  | RAFL04-09-D07 | At1g54100 / aldehyde dehydrogenase, putative (ALDH) | |  |  |  |  |  | | --- | --- | --- | --- | --- | |  |  |  |  |  | | EC:1.5.1.12  1-pyrroline-5-carboxylate dehydrogenase//aldehyde dehydrogenase | | | | | | |
|  |  | RAFL08-15-L09 | At1g54100 / aldehyde dehydrogenase, putative (ALDH) | |  |  |  |  |  | | --- | --- | --- | --- | --- | |  |  |  |  |  | | EC:1.5.1.12  1-pyrroline-5-carboxylate dehydrogenase//aldehyde dehydrogenase | | | | | | |
|  |  | RAFL08-09-C23 | At1g54100 / aldehyde dehydrogenase, putative (ALDH) | |  |  |  |  |  | | --- | --- | --- | --- | --- | |  |  |  |  |  | | EC:1.5.1.12  1-pyrroline-5-carboxylate dehydrogenase//aldehyde dehydrogenase | | | | | | |
|  | octane oxidation | |  |  | 4 | 28 | 8 | 4623 | 8.7054286E-7 | 7.834886E-6 | 9 |
|  |  | RAFL05-21-E06 | At1g54100 / aldehyde dehydrogenase, putative (ALDH) | |  |  |  |  |  | | --- | --- | --- | --- | --- | |  |  |  |  |  | | EC:1.2.1.3  unknown | | | | | | |
|  |  | RAFL04-09-D07 | At1g54100 / aldehyde dehydrogenase, putative (ALDH) | |  |  |  |  |  | | --- | --- | --- | --- | --- | |  |  |  |  |  | | EC:1.2.1.3  unknown | | | | | | |
|  |  | RAFL08-15-L09 | At1g54100 / aldehyde dehydrogenase, putative (ALDH) | |  |  |  |  |  | | --- | --- | --- | --- | --- | |  |  |  |  |  | | EC:1.2.1.3  unknown | | | | | | |
|  |  | RAFL08-09-C23 | At1g54100 / aldehyde dehydrogenase, putative (ALDH) | |  |  |  |  |  | | --- | --- | --- | --- | --- | |  |  |  |  |  | | EC:1.2.1.3  unknown | | | | | | |
|  | arginine degradation IX | |  |  | 4 | 28 | 9 | 4622 | 1.2514063E-6 | 1.2514062E-5 | 10 |
|  |  | RAFL05-21-E06 | At1g54100 / aldehyde dehydrogenase, putative (ALDH) | |  |  |  |  |  | | --- | --- | --- | --- | --- | |  |  |  |  |  | | EC:1.5.1.12  1-pyrroline-5-carboxylate dehydrogenase//aldehyde dehydrogenase | | | | | | |
|  |  | RAFL04-09-D07 | At1g54100 / aldehyde dehydrogenase, putative (ALDH) | |  |  |  |  |  | | --- | --- | --- | --- | --- | |  |  |  |  |  | | EC:1.5.1.12  1-pyrroline-5-carboxylate dehydrogenase//aldehyde dehydrogenase | | | | | | |
|  |  | RAFL08-15-L09 | At1g54100 / aldehyde dehydrogenase, putative (ALDH) | |  |  |  |  |  | | --- | --- | --- | --- | --- | |  |  |  |  |  | | EC:1.5.1.12  1-pyrroline-5-carboxylate dehydrogenase//aldehyde dehydrogenase | | | | | | |
|  |  | RAFL08-09-C23 | At1g54100 / aldehyde dehydrogenase, putative (ALDH) | |  |  |  |  |  | | --- | --- | --- | --- | --- | |  |  |  |  |  | | EC:1.5.1.12  1-pyrroline-5-carboxylate dehydrogenase//aldehyde dehydrogenase | | | | | | |
|  | non-phosphorylated glucose degradation | |  |  | 4 | 28 | 13 | 4618 | 4.0860064E-6 | 4.0860064E-5 | 10 |
|  |  | RAFL05-21-E06 | At1g54100 / aldehyde dehydrogenase, putative (ALDH) | |  |  |  |  |  | | --- | --- | --- | --- | --- | |  |  |  |  |  | | EC:1.2.1.3  unknown | | | | | | |
|  |  | RAFL04-09-D07 | At1g54100 / aldehyde dehydrogenase, putative (ALDH) | |  |  |  |  |  | | --- | --- | --- | --- | --- | |  |  |  |  |  | | EC:1.2.1.3  unknown | | | | | | |
|  |  | RAFL08-15-L09 | At1g54100 / aldehyde dehydrogenase, putative (ALDH) | |  |  |  |  |  | | --- | --- | --- | --- | --- | |  |  |  |  |  | | EC:1.2.1.3  unknown | | | | | | |
|  |  | RAFL08-09-C23 | At1g54100 / aldehyde dehydrogenase, putative (ALDH) | |  |  |  |  |  | | --- | --- | --- | --- | --- | |  |  |  |  |  | | EC:1.2.1.3  unknown | | | | | | |
|  | lignin biosynthesis | |  |  | 3 | 29 | 18 | 4613 | 3.591211E-4 | 0.004309453 | 12 |
|  |  | RAFL05-18-A06 | At1g09500 / cinnamyl-alcohol dehydrogenase (CAD) family | |  |  |  |  |  | | --- | --- | --- | --- | --- | |  |  |  |  |  | | EC:1.1.1.195  cinnamyl alcohol dehydrogenase | | | | | | |
|  |  | RAFL06-15-H16 | At1g09500 / cinnamyl-alcohol dehydrogenase (CAD) family | |  |  |  |  |  | | --- | --- | --- | --- | --- | |  |  |  |  |  | | EC:1.1.1.195  cinnamyl alcohol dehydrogenase | | | | | | |
|  |  | RAFL05-14-E15 | At2g33590 / cinnamoyl-CoA reductase family | |  |  |  |  |  | | --- | --- | --- | --- | --- | |  |  |  |  |  | | EC:1.2.1.44  cinnamoyl coenzyme A reductase | | | | | | |
|  | sucrose biosynthesis | |  |  | 2 | 30 | 10 | 4621 | 0.0028851912 | 0.02596672 | 9 |
|  |  | RAFL05-18-M07 | At4g02280 / sucrose synthase (UDP-glucose-fructose glucosyltransferase/sucrose-UDP glucosyltransferase), putative | |  |  |  |  |  | | --- | --- | --- | --- | --- | |  |  |  |  |  | | EC:2.4.1.13  UDP-glycosyltransferase | | | | | | |
|  |  | RAFL08-10-G22 | At4g02280 / sucrose synthase (UDP-glucose-fructose glucosyltransferase/sucrose-UDP glucosyltransferase), putative | |  |  |  |  |  | | --- | --- | --- | --- | --- | |  |  |  |  |  | | EC:2.4.1.13  UDP-glycosyltransferase | | | | | | |
|  | sucrose degradation III | |  |  | 2 | 30 | 17 | 4614 | 0.0072546974 | 0.094311066 | 13 |
|  |  | RAFL05-18-M07 | At4g02280 / sucrose synthase (UDP-glucose-fructose glucosyltransferase/sucrose-UDP glucosyltransferase), putative | |  |  |  |  |  | | --- | --- | --- | --- | --- | |  |  |  |  |  | | EC:2.4.1.13  UDP-glycosyltransferase | | | | | | |
|  |  | RAFL08-10-G22 | At4g02280 / sucrose synthase (UDP-glucose-fructose glucosyltransferase/sucrose-UDP glucosyltransferase), putative | |  |  |  |  |  | | --- | --- | --- | --- | --- | |  |  |  |  |  | | EC:2.4.1.13  UDP-glycosyltransferase | | | | | | |
|  | biotin biosynthesis I | |  |  | 1 | 31 | 3 | 4628 | 0.027177518 | 0.08153255 | 3 |
|  |  | RAFL05-08-B14 | At2g38400 / alanine--glyoxylate aminotransferase (beta-alanine-pyruvate aminotransferase/AGT), putative | |  |  |  |  |  | | --- | --- | --- | --- | --- | |  |  |  |  |  | | EC:2.6.1.62  4-aminobutyrate transaminase//alanine-glyoxylate transaminase | | | | | | |
|  | glutamate degradation I | |  |  | 1 | 31 | 5 | 4626 | 0.04049657 | 0.2429794 | 6 |
|  |  | RAFL05-08-B14 | At2g38400 / alanine--glyoxylate aminotransferase (beta-alanine-pyruvate aminotransferase/AGT), putative | |  |  |  |  |  | | --- | --- | --- | --- | --- | |  |  |  |  |  | | EC:2.6.1.19  4-aminobutyrate transaminase//alanine-glyoxylate transaminase | | | | | | |
|  | vitamin E biosynthesis | |  |  | 1 | 31 | 5 | 4626 | 0.04049657 | 0.16198628 | 4 |
|  |  | RAFL05-14-F20 | At1g06570 / 4-hydroxyphenylpyruvate dioxygenase (HPD) | |  |  |  |  |  | | --- | --- | --- | --- | --- | |  |  |  |  |  | | EC:1.13.11.27  4-hydroxyphenylpyruvate dioxygenase | | | | | | |
|  | plastoquinone biosynthesis | |  |  | 1 | 31 | 5 | 4626 | 0.04049657 | 0.16198628 | 4 |
|  |  | RAFL05-14-F20 | At1g06570 / 4-hydroxyphenylpyruvate dioxygenase (HPD) | |  |  |  |  |  | | --- | --- | --- | --- | --- | |  |  |  |  |  | | EC:1.13.11.27  4-hydroxyphenylpyruvate dioxygenase | | | | | | |
