# Supplementary material for: A flexible representation of omic knowledge for thorough analysis of microarray data
Source: Plant Methods. 2006 Mar 2;2:5. doi: 10.1186/1746-4811-2-5 (PMC1421397; doi:10.1186/1746-4811-2-5)
Supplement: Additional File 3 — Supplementary Table 3. Ranking result of significant correlations between the "functional Class" of the Directed acyclic graphs of GO terms and the clusters formed by BL-SOM of the microarray probes of expression profile under drought conditions [file 1746-4811-2-5-S3.HTML]

|  |  |  |  |  |  |  |  |  |  |  |  |
| --- | --- | --- | --- | --- | --- | --- | --- | --- | --- | --- | --- |
| Date: | | 2005/06/24 | | | | | | | | | |
| Method: | | Fisher test | | | | | | | | | |
| Cut off P-value: | | 0.05 | | | | | | | | | |
| Target dataset(s): | | biological\_process GO\_SUBSET0008150 cellular\_component GO\_SUBSET0005575 molecular\_function GO\_SUBSET0003674 | | | | | | | | | |
| Query dataset(s): | | SOM Cluster | | | | | | | | | |
  | | | | | | | | | | | || GO:0005515,GO:0045308 | | | protein binding |  | A | B | C | D | P | P' | N |
|  | Cluster:10-2 | |  |  | 7 | 102 | 82 | 4472 | 0.0044802204 | 0.11648573 | 26 |
|  |  | RAFL08-11-G23 | At3g63060 / expressed protein | |  |  |  |  |  | | --- | --- | --- | --- | --- | |  |  |  |  |  | | At3g63060 ,RAFL08-11-G23  circadian clock coupling factor, putative similar to gb:AAK56924 circadian clock coupling factor ZGT {Nicotiana tabacum} | | | | | | |
|  |  | RAFL04-09-C09 | At5g13750 / transporter-related protein | |  |  |  |  |  | | --- | --- | --- | --- | --- | |  |  |  |  |  | | At5g13750 ,RAFL04-09-C09  transporter-related | | | | | | |
|  |  | RAFL05-02-O23 | At3g46450 / SEC14 cytosolic factor, putative | |  |  |  |  |  | | --- | --- | --- | --- | --- | |  |  |  |  |  | | RAFL05-02-O23 ,At3g46450  SEC14 cytosolic factor family protein / phosphoglyceride transfer family protein contains Pfam PF00650 : CRAL/TRIO domain; similar to polyphosphoinositide binding protein Ssh1p (GI:|2739044) {Glycine max} | | | | | | |
|  |  | RAFL06-09-O18 | At5g13750 / transporter-related protein | |  |  |  |  |  | | --- | --- | --- | --- | --- | |  |  |  |  |  | | At5g13750 ,RAFL06-09-O18  transporter-related | | | | | | |
|  |  | RAFL06-09-G08 | At5g58070 / outer membrane lipo protein - like | |  |  |  |  |  | | --- | --- | --- | --- | --- | |  |  |  |  |  | | At5g58070 ,RAFL06-09-G08  lipocalin, putative similar to temperature stress-induced lipocalin [Triticum aestivum] GI:18650668 | | | | | | |
|  |  | RAFL08-12-H04 | At2g26690 / nitrate transporter -related | |  |  |  |  |  | | --- | --- | --- | --- | --- | |  |  |  |  |  | | RAFL08-12-H04 ,At2g26690  nitrate transporter (NTP2) identical to nitrate transporter (ntp2) [Arabidopsis thaliana] GI:4490321 | | | | | | |
|  |  | RAFL04-12-E05 | At1g63010 / expressed protein | |  |  |  |  |  | | --- | --- | --- | --- | --- | |  |  |  |  |  | | At1g63010 ,RAFL04-12-E05  SPX (SYG1/Pho81/XPR1) domain-containing protein contains Pfam profile PF03105: SPX domain | | | | | | |
|  | Cluster:8-2 | |  |  | 4 | 58 | 85 | 4516 | 0.029862033 | 0.7764129 | 26 |
|  |  | RAFL05-14-L07 | At5g60790 / ABC transporter family protein | |  |  |  |  |  | | --- | --- | --- | --- | --- | |  |  |  |  |  | | At5g60790 ,RAFL05-14-L07  ABC transporter family protein similar to ABC transporter homolog PnATH GI:7573600 from [Populus nigra] | | | | | | |
|  |  | RAFL05-16-B15 | At1g69870 / peptide transporter -related | |  |  |  |  |  | | --- | --- | --- | --- | --- | |  |  |  |  |  | | At1g69870 ,RAFL05-16-B15  proton-dependent oligopeptide transport (POT) family protein contains Pfam profile: PF00854 POT family | | | | | | |
|  |  | RAFL04-17-A14 | At2g21050 / amino acid permease, putative | |  |  |  |  |  | | --- | --- | --- | --- | --- | |  |  |  |  |  | | At2g21050 ,RAFL04-17-A14  amino acid permease, putative similar to AUX1 [Arabidopsis thaliana] GI:1531758; contains Pfam profile PF01490: Transmembrane amino acid transporter protein | | | | | | |
|  |  | RAFL07-10-I02 | At5g60790 / ABC transporter family protein | |  |  |  |  |  | | --- | --- | --- | --- | --- | |  |  |  |  |  | | RAFL07-10-I02 ,At5g60790  ABC transporter family protein similar to ABC transporter homolog PnATH GI:7573600 from [Populus nigra] | | | | | | |
| GO:0005975 | | | carbohydrate metabolism |  | A | B | C | D | P | P' | N |
|  | Cluster:1-2 | |  |  | 1 | 173 | 0 | 4489 | 0.037315033 | 0.037315033 | 1 |
|  |  | RAFL09-10-I20 | At1g70940 / auxin transport protein, putative (PIN3) | |  |  |  |  |  | | --- | --- | --- | --- | --- | |  |  |  |  |  | | RAFL09-10-I20 ,At1g70940  auxin transport protein, putative (PIN3) similar to auxin transport protein [Arabidopsis thaliana] gi|5817301|gb|AAD52695 | | | | | | |
| GO:0016265 | | | death |  | A | B | C | D | P | P' | N |
|  | Cluster:2-2 | |  |  | 5 | 48 | 97 | 4513 | 0.005667244 | 0.14734836 | 26 |
|  |  | RAFL05-19-J02 | At1g60260 / glycosyl hydrolase family 1 | |  |  |  |  |  | | --- | --- | --- | --- | --- | |  |  |  |  |  | | At1g60260 ,RAFL05-19-J02  pseudogene, glycosyl hydrolase family 1 contains Pfam PF00232 : Glycosyl hydrolase family 1 domain; TIGRFAM TIGR01233: 6-phospho-beta-galactosidase; similar to amygdalin hydrolase isoform AH I precursor (GI:16757966) [Prunus serotina]; blastp match of 60% identity and 6.4e-121 P-value to GP|12746303|gb|AAK07429.1|AF321287\_1|AF321287 beta-glucosidase {Musa acuminata} | | | | | | |
|  |  | RAFL04-15-F10 | At1g75680 / glycosyl hydrolase family 9 | |  |  |  |  |  | | --- | --- | --- | --- | --- | |  |  |  |  |  | | At1g75680 ,RAFL04-15-F10  glycosyl hydrolase family 9 protein similar to endo-beta-1,4-glucanase GB:AAC12685 GI:3025470 from [Pinus radiata] | | | | | | |
|  |  | RAFL04-09-D16 | At5g24420 / 6-phosphogluconolactonase-related protein | |  |  |  |  |  | | --- | --- | --- | --- | --- | |  |  |  |  |  | | At5g24420 ,RAFL04-09-D16  glucosamine/galactosamine-6-phosphate isomerase-related contains weak similarity to Swiss-Prot:O95336 6-phosphogluconolactonase (EC 3.1.1.31) (6PGL) [Homo sapiens] | | | | | | |
|  |  | RAFL09-14-M16 | At1g64390 / glycosyl hydrolase family 9 (endo-1,4-beta-glucanase) | |  |  |  |  |  | | --- | --- | --- | --- | --- | |  |  |  |  |  | | At1g64390 ,RAFL09-14-M16  endo-1,4-beta-glucanase, putative / cellulase, putative similar to endo-beta-1,4-glucanase GI:4972236 from [Fragaria x ananassa] (Plant Mol. Biol. 40, 323-332 (1999)) | | | | | | |
|  |  | RAFL04-19-M06 | At1g24100 / UDP-glycosyltransferase family | |  |  |  |  |  | | --- | --- | --- | --- | --- | |  |  |  |  |  | | At1g24100 ,RAFL04-19-M06  UDP-glucoronosyl/UDP-glucosyl transferase family protein contains Pfam profile: PF00201 UDP-glucoronosyl and UDP-glucosyl transferase | | | | | | |
|  | Cluster:0-1 | |  |  | 7 | 99 | 95 | 4462 | 0.008086153 | 0.21023999 | 26 |
|  |  | RAFL05-11-D11 | At1g55490 / RuBisCo subunit binding-protein beta subunit/60 kDa chaperonin beta subunit | |  |  |  |  |  | | --- | --- | --- | --- | --- | |  |  |  |  |  | | RAFL05-11-D11 ,At1g55490  RuBisCO subunit binding-protein beta subunit, chloroplast / 60 kDa chaperonin beta subunit / CPN-60 beta identical to SWISS-PROT:P21240- RuBisCO subunit binding-protein beta subunit, chloroplast precursor (60 kDa chaperonin beta subunit, CPN-60 beta) [Arabidopsis thaliana] | | | | | | |
|  |  | RAFL07-10-G07 | At1g52400 / glycosyl hydrolase family 1, beta-glucosidase (BG1) | |  |  |  |  |  | | --- | --- | --- | --- | --- | |  |  |  |  |  | | At1g52400 ,RAFL07-10-G07  glycosyl hydrolase family 1 protein / beta-glucosidase, putative (BG1) contains Pfam PF00232 : Glycosyl hydrolase family 1 domain; TIGRFAM TIGR01233: 6-phospho-beta-galactosidase; identical to GI:6651430 from [Arabidopsis thaliana] | | | | | | |
|  |  | RAFL05-12-O19 | At3g55800 / sedoheptulose-bisphosphatase precursor | |  |  |  |  |  | | --- | --- | --- | --- | --- | |  |  |  |  |  | | At3g55800 ,RAFL05-12-O19  sedoheptulose-1,7-bisphosphatase, chloroplast / sedoheptulose-bisphosphatase identical to SP|P46283 Sedoheptulose-1,7-bisphosphatase, chloroplast precursor (EC 3.1.3.37) (Sedoheptulose-bisphosphatase) (SBPASE) (SED(1,7)P2ASE) {Arabidopsis thaliana} | | | | | | |
|  |  | RAFL05-03-B20 | At1g52400 / glycosyl hydrolase family 1, beta-glucosidase (BG1) | |  |  |  |  |  | | --- | --- | --- | --- | --- | |  |  |  |  |  | | RAFL05-03-B20 ,At1g52400  glycosyl hydrolase family 1 protein / beta-glucosidase, putative (BG1) contains Pfam PF00232 : Glycosyl hydrolase family 1 domain; TIGRFAM TIGR01233: 6-phospho-beta-galactosidase; identical to GI:6651430 from [Arabidopsis thaliana] | | | | | | |
|  |  | RAFL04-19-M17 | At1g32060 / phosphoribulokinase precursor | |  |  |  |  |  | | --- | --- | --- | --- | --- | |  |  |  |  |  | | RAFL04-19-M17 ,At1g32060  phosphoribulokinase (PRK) / phosphopentokinase nearly identical to SP|P25697 Phosphoribulokinase, chloroplast precursor (EC 2.7.1.19) (Phosphopentokinase) (PRKASE) (PRK) {Arabidopsis thaliana} | | | | | | |
|  |  | RAFL04-13-J02 | At3g54050 / fructose-bisphosphatase precursor | |  |  |  |  |  | | --- | --- | --- | --- | --- | |  |  |  |  |  | | At3g54050 ,RAFL04-13-J02  fructose-1,6-bisphosphatase, putative / D-fructose-1,6-bisphosphate 1-phosphohydrolase, putative / FBPase, putative strong similarity to fructose-1,6-bisphosphatase [Brassica napus] GI:289367; identical to SP|P25851 Fructose-1,6-bisphosphatase, chloroplast precursor (EC 3.1.3.11) (D-fructose-1,6-bisphosphate 1-phosphohydrolase) (FBPase) {Arabidopsis thaliana}; contains Pfam profile PF00316: fructose-1,6-bisphosphatase | | | | | | |
|  |  | RAFL09-15-K15 | At3g18080 / glycosyl hydrolase family 1 | |  |  |  |  |  | | --- | --- | --- | --- | --- | |  |  |  |  |  | | At3g18080 ,RAFL09-15-K15  glycosyl hydrolase family 1 protein contains Pfam PF00232 : Glycosyl hydrolase family 1 domain; TIGRFAM TIGR01233: 6-phospho-beta-galactosidase; similar to beta-glucosidase BGQ60 precursor GB:A57512 [Hordeum vulgare]; similar to beta-mannosidase enzyme (GI:17226270) [Lycopersicon esculentum] | | | | | | |
|  | Cluster:3-2 | |  |  | 4 | 33 | 98 | 4528 | 0.008190396 | 0.2129503 | 26 |
|  |  | RAFL09-16-F08 | At3g23820 / NAD-dependent epimerase/dehydratase family | |  |  |  |  |  | | --- | --- | --- | --- | --- | |  |  |  |  |  | | At3g23820 ,RAFL09-16-F08  NAD-dependent epimerase/dehydratase family protein similar to nucleotide sugar epimerase from Vibrio vulnificus GI:3093975 [PID:g3093975], WbnF [Escherichia coli] GI:5739472, CAPI protein {Staphylococcus aureus} SP|P39858; contains Pfam profile: PF01370 NAD dependent epimerase/dehydratase family | | | | | | |
|  |  | RAFL07-11-K21 | At2g32990 / glycosyl hydrolase family 9 | |  |  |  |  |  | | --- | --- | --- | --- | --- | |  |  |  |  |  | | At2g32990 ,RAFL07-11-K21  glycosyl hydrolase family 9 protein similar to endo-beta-1,4-glucanase GI:4972236 from [Fragaria x ananassa] | | | | | | |
|  |  | RAFL04-14-G14 | At1g26560 / glycosyl hydrolase family 1 | |  |  |  |  |  | | --- | --- | --- | --- | --- | |  |  |  |  |  | | At1g26560 ,RAFL04-14-G14  glycosyl hydrolase family 1 protein contains Pfam PF00232 : Glycosyl hydrolase family 1 domain; TIGRFAM TIGR01233: 6-phospho-beta-galactosidase; similar to amygdalin hydrolase isoform AH I precursor (GI:16757966) [Prunus serotina] | | | | | | |
|  |  | RAFL09-07-D12 | At3g23820 / NAD-dependent epimerase/dehydratase family | |  |  |  |  |  | | --- | --- | --- | --- | --- | |  |  |  |  |  | | At3g23820 ,RAFL09-07-D12  NAD-dependent epimerase/dehydratase family protein similar to nucleotide sugar epimerase from Vibrio vulnificus GI:3093975 [PID:g3093975], WbnF [Escherichia coli] GI:5739472, CAPI protein {Staphylococcus aureus} SP|P39858; contains Pfam profile: PF01370 NAD dependent epimerase/dehydratase family | | | | | | |
|  | Cluster:8-2 | |  |  | 5 | 57 | 97 | 4504 | 0.010950954 | 0.2847248 | 26 |
|  |  | RAFL06-11-H07 | At1g80460 / glycerol kinase -related | |  |  |  |  |  | | --- | --- | --- | --- | --- | |  |  |  |  |  | | At1g80460 ,RAFL06-11-H07  glycerol kinase, putative similar to glycerol kinase (ATP:glycerol 3-phosphotransferase, Glycerokinase, GK)[Mycobacterium tuberculosis] Swiss-Prot:O69664 | | | | | | |
|  |  | RAFL11-05-M03 | At5g15650 / reversibly glycosylated polypeptide-3 | |  |  |  |  |  | | --- | --- | --- | --- | --- | |  |  |  |  |  | | At5g15650 ,RAFL11-05-M03  reversibly glycosylated polypeptide-2 (RGP2) identical to reversibly glycosylated polypeptide-2 [Arabidopsis thaliana] GI:2317731 | | | | | | |
|  |  | RAFL08-18-I10 | At3g47000 / glycosyl hydrolase family 3 | |  |  |  |  |  | | --- | --- | --- | --- | --- | |  |  |  |  |  | | At3g47000 ,RAFL08-18-I10  glycosyl hydrolase family 3 protein beta-D-glucan exohydrolase, Nicotiana tabacum, TREMBL:AB017502\_1 | | | | | | |
|  |  | RAFL05-02-G21 | At5g15650 / reversibly glycosylated polypeptide-3 | |  |  |  |  |  | | --- | --- | --- | --- | --- | |  |  |  |  |  | | At5g15650 ,RAFL05-02-G21  reversibly glycosylated polypeptide-2 (RGP2) identical to reversibly glycosylated polypeptide-2 [Arabidopsis thaliana] GI:2317731 | | | | | | |
|  |  | RAFL07-10-L13 | At1g17840 / ABC transporter family protein | |  |  |  |  |  | | --- | --- | --- | --- | --- | |  |  |  |  |  | | RAFL07-10-L13 ,At1g17840  ABC transporter family protein similar to ABC transporter GI:10280532 from [Homo sapiens] | | | | | | |
| GO:0006464 | | | protein modification |  | A | B | C | D | P | P' | N |
|  | Cluster:8-1 | |  |  | 7 | 155 | 54 | 4447 | 0.004826717 | 0.11101449 | 23 |
|  |  | RAFL09-06-I17 | At4g05320 / polyubiquitin UBQ10/SEN3 | |  |  |  |  |  | | --- | --- | --- | --- | --- | |  |  |  |  |  | | At4g05320 ,RAFL09-06-I17  polyubiquitin (UBQ10) (SEN3) senescence-associated protein; identical to GI:870791 | | | | | | |
|  |  | RAFL07-10-D10 | At4g05320 / polyubiquitin UBQ10/SEN3 | |  |  |  |  |  | | --- | --- | --- | --- | --- | |  |  |  |  |  | | At4g05320 ,RAFL07-10-D10  polyubiquitin (UBQ10) (SEN3) senescence-associated protein; identical to GI:870791 | | | | | | |
|  |  | RAFL04-16-G03 | At4g02890 / polyubiquitin (UBQ14) | |  |  |  |  |  | | --- | --- | --- | --- | --- | |  |  |  |  |  | | RAFL04-16-G03 ,At4g02890  polyubiquitin (UBQ14) identical to GI:166795; similar to N. sylvestris hexameric polyubiquitin, GenBank accession number M74101 | | | | | | |
|  |  | RAFL05-04-O09 | At1g55530 / expressed protein | |  |  |  |  |  | | --- | --- | --- | --- | --- | |  |  |  |  |  | | At1g55530 ,RAFL05-04-O09  zinc finger (C3HC4-type RING finger) family protein contains Pfam domain, PF00097: Zinc finger, C3HC4 type (RING finger) | | | | | | |
|  |  | RAFL05-14-O22 | At4g05320 / polyubiquitin UBQ10/SEN3 | |  |  |  |  |  | | --- | --- | --- | --- | --- | |  |  |  |  |  | | At4g05320 ,RAFL05-14-O22  polyubiquitin (UBQ10) (SEN3) senescence-associated protein; identical to GI:870791 | | | | | | |
|  |  | RAFL05-18-E11 | At4g03430 / pre-mRNA splicing factor -related | |  |  |  |  |  | | --- | --- | --- | --- | --- | |  |  |  |  |  | | RAFL05-18-E11 ,At4g03430  pre-mRNA splicing factor-related similar to pre-mRNA splicing factor pre-mRNA splicing factor prp1 (SP:Q12381) [Fission yeast] | | | | | | |
|  |  | RAFL09-09-O15 | At4g05320 / polyubiquitin UBQ10/SEN3 | |  |  |  |  |  | | --- | --- | --- | --- | --- | |  |  |  |  |  | | At4g05320 ,RAFL09-09-O15  polyubiquitin (UBQ10) (SEN3) senescence-associated protein; identical to GI:870791 | | | | | | |
|  | Cluster:8-2 | |  |  | 3 | 59 | 58 | 4543 | 0.046716247 | 1.0744736 | 23 |
|  |  | RAFL06-07-F24 | At4g05050 / polyubiquitin UBQ11 | |  |  |  |  |  | | --- | --- | --- | --- | --- | |  |  |  |  |  | | RAFL06-07-F24 ,At4g05050  polyubiquitin (UBQ11) identical to GI:304117 | | | | | | |
|  |  | RAFL04-14-B14 | At4g05050 / polyubiquitin UBQ11 | |  |  |  |  |  | | --- | --- | --- | --- | --- | |  |  |  |  |  | | At4g05050 ,RAFL04-14-B14  polyubiquitin (UBQ11) identical to GI:304117 | | | | | | |
|  |  | RAFL07-12-F11 | At3g49220 / pectinesterase family | |  |  |  |  |  | | --- | --- | --- | --- | --- | |  |  |  |  |  | | At3g49220 ,RAFL07-12-F11  pectinesterase family protein contains Pfam profile: PF01095 pectinesterase | | | | | | |
| GO:0006118 | | | electron transport |  | A | B | C | D | P | P' | N |
|  | Cluster:0-2 | |  |  | 22 | 57 | 232 | 4352 | 7.7140204E-11 | 2.314206E-9 | 30 |
|  |  | RAFL06-16-F14 | At1g29920 / photosystem II type I chlorophyll a /b binding protein, putative | |  |  |  |  |  | | --- | --- | --- | --- | --- | |  |  |  |  |  | | At1g29920 ,RAFL06-16-F14  chlorophyll A-B binding protein 165/180, chloroplast / LHCII type I CAB-165/180 identical to SP|P04777 Chlorophyll A-B binding protein 165/180, chloroplast precursor (LHCII type I CAB-165/180) (LHCP) {Arabidopsis thaliana}; similar to photosystem II type I chlorophyll a /b binding protein GI:16364 from [Arabidopsis thaliana] | | | | | | |
|  |  | RAFL04-19-B03 | At3g16140 / photosystem I subunit VI precursor | |  |  |  |  |  | | --- | --- | --- | --- | --- | |  |  |  |  |  | | At3g16140 ,RAFL04-19-B03  photosystem I reaction center subunit VI, chloroplast, putative / PSI-H, putative (PSAH1) identical to SP|Q9SUI7; similar to PSI-H precursor [Nicotiana sylvestris] GI:407353; contains Pfam profile PF03244: Photosystem I reaction centre subunit VI | | | | | | |
|  |  | RAFL03-06-D05 | At4g05180 / oxygen-evolving complex protein 16, chloroplast precursor (OEC16) | |  |  |  |  |  | | --- | --- | --- | --- | --- | |  |  |  |  |  | | RAFL03-06-D05 ,At4g05180  oxygen-evolving enhancer protein 3, chloroplast, putative (PSBQ2) identical to SP|Q41932 Oxygen-evolving enhancer protein 3-2, chloroplast precursor (OEE3) (16 kDa subunit of oxygen evolving system of photosystem II) (OEC 16 kDa subunit) {Arabidopsis thaliana}; similar to SP|P12301 Oxygen-evolving enhancer protein 3, chloroplast precursor (OEE3) (16 kDa subunit of oxygen evolving system of photosystem II) (OEC 16 kDa subunit) {Spinacia oleracea}; contains Pfam profile PF05757: Oxygen evolving enhancer protein 3 (PsbQ) | | | | | | |
|  |  | RAFL02-02-L10 | At1g03130 / photosystem I reaction center subunit II precursor -related | |  |  |  |  |  | | --- | --- | --- | --- | --- | |  |  |  |  |  | | RAFL02-02-L10 ,At1g03130  photosystem I reaction center subunit II, chloroplast, putative / photosystem I 20 kDa subunit, putative / PSI-D, putative (PSAD2) similar to SP|P12353 Photosystem I reaction center subunit II, chloroplast precursor (Photosystem I 20 kDa subunit) (PSI-D) {Spinacia oleracea}; contains Pfam profile PF02531: PsaD | | | | | | |
|  |  | RAFL06-07-N01 | At3g14420 / glycolate oxidase -related | |  |  |  |  |  | | --- | --- | --- | --- | --- | |  |  |  |  |  | | At3g14420 ,RAFL06-07-N01  (S)-2-hydroxy-acid oxidase, peroxisomal, putative / glycolate oxidase, putative / short chain alpha-hydroxy acid oxidase, putative similar to (S)-2-hydroxy-acid oxidase, peroxisomal (Glycolate oxidase, GOX) (Short chain alpha-hydroxy acid oxidase) [Spinacia oleracea] SWISS-PROT:P05414 | | | | | | |
|  |  | RAFL09-10-I02 | At1g29930 / light-harvesting chlorophyll a/b binding protein | |  |  |  |  |  | | --- | --- | --- | --- | --- | |  |  |  |  |  | | RAFL09-10-I02 ,At1g29930  chlorophyll A-B binding protein 2, chloroplast / LHCII type I CAB-2 / CAB-140 (CAB2B) identical to SP|P04778 Chlorophyll A-B binding protein 2, chloroplast precursor (LHCII type I CAB-2) (CAB-140) (LHCP) {Arabidopsis thaliana} | | | | | | |
|  |  | RAFL09-06-G16 | At1g31330 / photosystem I subunit III precursor -related | |  |  |  |  |  | | --- | --- | --- | --- | --- | |  |  |  |  |  | | At1g31330 ,RAFL09-06-G16  photosystem I reaction center subunit III family protein contains Pfam profile: PF02507: photosystem I reaction center subunit III | | | | | | |
|  |  | RAFL06-09-H06 | At1g29910 / photosystem II type I chlorophyll a /b binding protein, putative | |  |  |  |  |  | | --- | --- | --- | --- | --- | |  |  |  |  |  | | RAFL06-09-H06 ,At1g29910  chlorophyll A-B binding protein 2, chloroplast / LHCII type I CAB-2 / CAB-140 (CAB2A) identical to SP|P04778 Chlorophyll A-B binding protein 2, chloroplast precursor (LHCII type I CAB-2) (CAB-140) (LHCP) {Arabidopsis thaliana} | | | | | | |
|  |  | RAFL06-10-D15 | At3g56940 / leucine zipper-containing protein AT103 | |  |  |  |  |  | | --- | --- | --- | --- | --- | |  |  |  |  |  | | At3g56940 ,RAFL06-10-D15  dicarboxylate diiron protein, putative (Crd1) similar to leucine-containing zipper protein At103 GP:6911864; contains Pfam profile PF05447: Copper response defect 1 (CRD1) | | | | | | |
|  |  | RAFL05-09-F03 | At1g31330 / photosystem I subunit III precursor -related | |  |  |  |  |  | | --- | --- | --- | --- | --- | |  |  |  |  |  | | At1g31330 ,RAFL05-09-F03  photosystem I reaction center subunit III family protein contains Pfam profile: PF02507: photosystem I reaction center subunit III | | | | | | |
|  |  | RAFL05-16-F08 | At1g31330 / photosystem I subunit III precursor -related | |  |  |  |  |  | | --- | --- | --- | --- | --- | |  |  |  |  |  | | RAFL05-16-F08 ,At1g31330  photosystem I reaction center subunit III family protein contains Pfam profile: PF02507: photosystem I reaction center subunit III | | | | | | |
|  |  | RAFL09-16-K15 | At1g31330 / photosystem I subunit III precursor -related | |  |  |  |  |  | | --- | --- | --- | --- | --- | |  |  |  |  |  | | At1g31330 ,RAFL09-16-K15  photosystem I reaction center subunit III family protein contains Pfam profile: PF02507: photosystem I reaction center subunit III | | | | | | |
|  |  | RAFL06-08-B20 | At5g66190 / ferredoxin--NADP(+) reductase (adrenodoxin reductase), putative | |  |  |  |  |  | | --- | --- | --- | --- | --- | |  |  |  |  |  | | At5g66190 ,RAFL06-08-B20  ferredoxin--NADP(+) reductase, putative / adrenodoxin reductase, putative strong similarity to Ferredoxin--NADP reductase, chloroplast precursor (EC 1.18.1.2) (FNR) from {Pisum sativum} SP|P10933, {Mesembryanthemum crystallinum} SP|P41343, {Spinacia oleracea} SP|P00455; identical to cDNA ferredoxin-NADP+ reductase precursor (petH) GI:5730138 | | | | | | |
|  |  | RAFL02-06-M22 | At5g54270 / light-harvesting chlorophyll a/b binding protein, putative | |  |  |  |  |  | | --- | --- | --- | --- | --- | |  |  |  |  |  | | At5g54270 ,RAFL02-06-M22  chlorophyll A-B binding protein / LHCII type III (LHCB3) identical to Lhcb3 protein [Arabidopsis thaliana] GI:4741952; contains Pfam profile PF00504: Chlorophyll A-B binding protein | | | | | | |
|  |  | RAFL06-07-K01 | At4g12800 / probable photosystem I chain XI precursor | |  |  |  |  |  | | --- | --- | --- | --- | --- | |  |  |  |  |  | | At4g12800 ,RAFL06-07-K01  photosystem I reaction center subunit XI, chloroplast (PSI-L) / PSI subunit V identical to Photosystem I reaction center subunit XI, chloroplast precursor (PSI-L) (PSI subunit V) (Swiss-Prot:Q9SUI4) [Arabidopsis thaliana]; contains Pfam profile PF02605: photosystem I reaction center subunit XI; contains 2 transmembrane domains | | | | | | |
|  |  | RAFL07-15-A19 | At4g02770 / photosystem I reaction center subunit II precursor -related | |  |  |  |  |  | | --- | --- | --- | --- | --- | |  |  |  |  |  | | RAFL07-15-A19 ,At4g02770  photosystem I reaction center subunit II, chloroplast, putative / photosystem I 20 kDa subunit, putative / PSI-D, putative (PSAD1) similar to SP|P12353 Photosystem I reaction center subunit II, chloroplast precursor (Photosystem I 20 kDa subunit) (PSI-D) {Spinacia oleracea}; contains Pfam profile PF02531: PsaD | | | | | | |
|  |  | RAFL11-03-K23 | At1g54500 / rubredoxin -related | |  |  |  |  |  | | --- | --- | --- | --- | --- | |  |  |  |  |  | | At1g54500 ,RAFL11-03-K23  rubredoxin family protein similar to SP|P00270 Rubredoxin (Rd) {Desulfovibrio gigas}; contains Pfam profile PF00301: Rubredoxin | | | | | | |
|  |  | RAFL06-15-B12 | At1g29930 / light-harvesting chlorophyll a/b binding protein | |  |  |  |  |  | | --- | --- | --- | --- | --- | |  |  |  |  |  | | At1g29930 ,RAFL06-15-B12  chlorophyll A-B binding protein 2, chloroplast / LHCII type I CAB-2 / CAB-140 (CAB2B) identical to SP|P04778 Chlorophyll A-B binding protein 2, chloroplast precursor (LHCII type I CAB-2) (CAB-140) (LHCP) {Arabidopsis thaliana} | | | | | | |
|  |  | RAFL02-10-L11 | At5g54270 / light-harvesting chlorophyll a/b binding protein, putative | |  |  |  |  |  | | --- | --- | --- | --- | --- | |  |  |  |  |  | | RAFL02-10-L11 ,At5g54270  chlorophyll A-B binding protein / LHCII type III (LHCB3) identical to Lhcb3 protein [Arabidopsis thaliana] GI:4741952; contains Pfam profile PF00504: Chlorophyll A-B binding protein | | | | | | |
|  |  | RAFL04-09-M24 | At1g54500 / rubredoxin -related | |  |  |  |  |  | | --- | --- | --- | --- | --- | |  |  |  |  |  | | At1g54500 ,RAFL04-09-M24  rubredoxin family protein similar to SP|P00270 Rubredoxin (Rd) {Desulfovibrio gigas}; contains Pfam profile PF00301: Rubredoxin | | | | | | |
|  |  | RAFL06-08-A02 | At1g29920 / photosystem II type I chlorophyll a /b binding protein, putative | |  |  |  |  |  | | --- | --- | --- | --- | --- | |  |  |  |  |  | | At1g29920 ,RAFL06-08-A02  chlorophyll A-B binding protein 165/180, chloroplast / LHCII type I CAB-165/180 identical to SP|P04777 Chlorophyll A-B binding protein 165/180, chloroplast precursor (LHCII type I CAB-165/180) (LHCP) {Arabidopsis thaliana}; similar to photosystem II type I chlorophyll a /b binding protein GI:16364 from [Arabidopsis thaliana] | | | | | | |
|  |  | RAFL06-16-J22 | At4g12800 / probable photosystem I chain XI precursor | |  |  |  |  |  | | --- | --- | --- | --- | --- | |  |  |  |  |  | | At4g12800 ,RAFL06-16-J22  photosystem I reaction center subunit XI, chloroplast (PSI-L) / PSI subunit V identical to Photosystem I reaction center subunit XI, chloroplast precursor (PSI-L) (PSI subunit V) (Swiss-Prot:Q9SUI4) [Arabidopsis thaliana]; contains Pfam profile PF02605: photosystem I reaction center subunit XI; contains 2 transmembrane domains | | | | | | |
|  | Cluster:1-2 | |  |  | 22 | 152 | 232 | 4257 | 2.0486195E-4 | 0.0061458587 | 30 |
|  |  | RAFL09-06-C15 | At5g01530 / light-harvesting chlorophyll a/b binding protein | |  |  |  |  |  | | --- | --- | --- | --- | --- | |  |  |  |  |  | | RAFL09-06-C15 ,At5g01530  chlorophyll A-B binding protein CP29 (LHCB4) identical to CP29 [Arabidopsis thaliana] GI:298036; contains Pfam profile: PF00504 chlorophyll A-B binding protein | | | | | | |
|  |  | RAFL11-06-J20 | At1g29930 / light-harvesting chlorophyll a/b binding protein | |  |  |  |  |  | | --- | --- | --- | --- | --- | |  |  |  |  |  | | At1g29930 ,RAFL11-06-J20  chlorophyll A-B binding protein 2, chloroplast / LHCII type I CAB-2 / CAB-140 (CAB2B) identical to SP|P04778 Chlorophyll A-B binding protein 2, chloroplast precursor (LHCII type I CAB-2) (CAB-140) (LHCP) {Arabidopsis thaliana} | | | | | | |
|  |  | RAFL05-17-F20 | At1g79040 / photosystem II polypeptide -related | |  |  |  |  |  | | --- | --- | --- | --- | --- | |  |  |  |  |  | | At1g79040 ,RAFL05-17-F20  photosystem II 10 kDa polypeptide identical to photosystem II 10 kDa polypeptide, chloroplast [precursor] SP:P27202 from [Arabidopsis thaliana]; contains Pfam profile: PF04725 photosystem II 10 kDa polypeptide PsbR | | | | | | |
|  |  | RAFL09-07-P20 | At5g15350 / plastocyanin-like domain containing protein | |  |  |  |  |  | | --- | --- | --- | --- | --- | |  |  |  |  |  | | At5g15350 ,RAFL09-07-P20  plastocyanin-like domain-containing protein contains plastocyanin-like domain Pfam:PF02298 | | | | | | |
|  |  | RAFL05-11-O16 | At3g61470 / light-harvesting chlorophyll a/b binding protein | |  |  |  |  |  | | --- | --- | --- | --- | --- | |  |  |  |  |  | | RAFL05-11-O16 ,At3g61470  chlorophyll A-B binding protein (LHCA2) identical to Lhca2 protein [Arabidopsis thaliana] GI:4741940; similar to chlorophyll A-B binding protein, chloroplast [Precursor] SP:P13869 from [Petunia hybrida]; contains Pfam profile: PF00504 chlorophyll A-B binding protein | | | | | | |
|  |  | RAFL05-02-P23 | At3g15360 / thioredoxin M-type 4, chloroplast precursor (TRX-M4) | |  |  |  |  |  | | --- | --- | --- | --- | --- | |  |  |  |  |  | | RAFL05-02-P23 ,At3g15360  thioredoxin M-type 4, chloroplast (TRX-M4) nearly identical to SP|Q9SEU6 Thioredoxin M-type 4, chloroplast precursor (TRX-M4) {Arabidopsis thaliana} | | | | | | |
|  |  | RAFL05-01-F04 | At1g30380 / photosystem I subunit X precursor | |  |  |  |  |  | | --- | --- | --- | --- | --- | |  |  |  |  |  | | At1g30380 ,RAFL05-01-F04  photosystem I reaction center subunit psaK, chloroplast, putative / photosystem I subunit X, putative / PSI-K, putative (PSAK) identical to SP|Q9SUI5; strong similarity to SP|P36886 Photosystem I reaction center subunit psaK, chloroplast precursor (Photosystem I subunit X) (PSI-K) (Light-harvesting complex I 7 kDa protein){Hordeum vulgare}; contains Pfam profile PF01241: Photosystem I psaG / psaK | | | | | | |
|  |  | RAFL07-12-B22 | At2g41680 / thioredoxin reductase, putative | |  |  |  |  |  | | --- | --- | --- | --- | --- | |  |  |  |  |  | | RAFL07-12-B22 ,At2g41680  thioredoxin reductase, putative / NADPH-dependent thioredoxin reductase, putative The last 2 exons encode thioredoxin. There is an EST match to exons 5-7, and the distance between exon 7 and exon 8 is only 90bp. It is unlikely this is two separate genes, but more likely a hybrid protein. | | | | | | |
|  |  | RAFL09-10-E05 | At1g12090 / protease inhibitor/seed storage/lipid transfer protein (LTP) family | |  |  |  |  |  | | --- | --- | --- | --- | --- | |  |  |  |  |  | | At1g12090 ,RAFL09-10-E05  protease inhibitor/seed storage/lipid transfer protein (LTP) family protein similar to 14 kDa polypeptide [Catharanthus roseus] GI:407410; contains Pfam protease inhibitor/seed storage/LTP family domain PF00234 | | | | | | |
|  |  | RAFL06-12-K03 | At3g54890 / light-harvesting chlorophyll a/b binding protein | |  |  |  |  |  | | --- | --- | --- | --- | --- | |  |  |  |  |  | | At3g54890 ,RAFL06-12-K03  chlorophyll A-B binding protein / LHCI type I (CAB) identical to chlorophyll A/B-binding protein [Arabidopsis thaliana] GI:16207; contains Pfam profile: PF00504 chlorophyll A-B binding protein | | | | | | |
|  |  | RAFL11-02-M05 | At4g28750 / photosystem I subunit PSI-E - like protein | |  |  |  |  |  | | --- | --- | --- | --- | --- | |  |  |  |  |  | | At4g28750 ,RAFL11-02-M05  photosystem I reaction center subunit IV, chloroplast, putative / PSI-E, putative (PSAE1) identical to SP|Q9S831; similar to SP|P12354 Photosystem I reaction center subunit IV, chloroplast precursor (PSI-E) {Spinacia oleracea}; contains Pfam profile PF02427: Photosystem I reaction centre subunit IV / PsaE | | | | | | |
|  |  | RAFL06-15-G17 | At5g01530 / light-harvesting chlorophyll a/b binding protein | |  |  |  |  |  | | --- | --- | --- | --- | --- | |  |  |  |  |  | | RAFL06-15-G17 ,At5g01530  chlorophyll A-B binding protein CP29 (LHCB4) identical to CP29 [Arabidopsis thaliana] GI:298036; contains Pfam profile: PF00504 chlorophyll A-B binding protein | | | | | | |
|  |  | RAFL08-09-P03 | At1g29930 / light-harvesting chlorophyll a/b binding protein | |  |  |  |  |  | | --- | --- | --- | --- | --- | |  |  |  |  |  | | At1g29930 ,RAFL08-09-P03  chlorophyll A-B binding protein 2, chloroplast / LHCII type I CAB-2 / CAB-140 (CAB2B) identical to SP|P04778 Chlorophyll A-B binding protein 2, chloroplast precursor (LHCII type I CAB-2) (CAB-140) (LHCP) {Arabidopsis thaliana} | | | | | | |
|  |  | RAFL06-16-K04 | At2g34430 / photosystem II type I chlorophyll a /b binding protein | |  |  |  |  |  | | --- | --- | --- | --- | --- | |  |  |  |  |  | | At2g34430 ,RAFL06-16-K04  chlorophyll A-B binding protein / LHCII type I (LHB1B1) identical to photosystem II type I chlorophyll a/b binding protein [Arabidopsis thaliana] GI:16366 | | | | | | |
|  |  | RAFL05-03-F16 | At5g50940 / expressed gene | |  |  |  |  |  | | --- | --- | --- | --- | --- | |  |  |  |  |  | | At5g50940 ,RAFL05-03-F16  expressed protein There's conflicting, but high quality data supporting a variety of gene structures. Each variant is presented here. | | | | | | |
|  |  | RAFL08-10-G06 | At5g01530 / light-harvesting chlorophyll a/b binding protein | |  |  |  |  |  | | --- | --- | --- | --- | --- | |  |  |  |  |  | | RAFL08-10-G06 ,At5g01530  chlorophyll A-B binding protein CP29 (LHCB4) identical to CP29 [Arabidopsis thaliana] GI:298036; contains Pfam profile: PF00504 chlorophyll A-B binding protein | | | | | | |
|  |  | RAFL05-07-G14 | At5g16400 / thioredoxin, putative | |  |  |  |  |  | | --- | --- | --- | --- | --- | |  |  |  |  |  | | At5g16400 ,RAFL05-07-G14  thioredoxin, putative similar to SP|P29450 Thioredoxin F-type, chloroplast precursor (TRX-F) {Pisum sativum}; contains Pfam profile: PF00085 Thioredoxin | | | | | | |
|  |  | RAFL08-10-P13 | At1g29930 / light-harvesting chlorophyll a/b binding protein | |  |  |  |  |  | | --- | --- | --- | --- | --- | |  |  |  |  |  | | At1g29930 ,RAFL08-10-P13  chlorophyll A-B binding protein 2, chloroplast / LHCII type I CAB-2 / CAB-140 (CAB2B) identical to SP|P04778 Chlorophyll A-B binding protein 2, chloroplast precursor (LHCII type I CAB-2) (CAB-140) (LHCP) {Arabidopsis thaliana} | | | | | | |
|  |  | RAFL07-16-C20 | At5g01530 / light-harvesting chlorophyll a/b binding protein | |  |  |  |  |  | | --- | --- | --- | --- | --- | |  |  |  |  |  | | RAFL07-16-C20 ,At5g01530  chlorophyll A-B binding protein CP29 (LHCB4) identical to CP29 [Arabidopsis thaliana] GI:298036; contains Pfam profile: PF00504 chlorophyll A-B binding protein | | | | | | |
|  |  | RAFL06-11-B12 | At5g10840 / endomembrane protein 70, putative | |  |  |  |  |  | | --- | --- | --- | --- | --- | |  |  |  |  |  | | RAFL06-11-B12 ,At5g10840  endomembrane protein 70, putative TM4 family; | | | | | | |
|  |  | RAFL04-15-M14 | At2g20260 / photosystem I reaction center subunit IV -related | |  |  |  |  |  | | --- | --- | --- | --- | --- | |  |  |  |  |  | | At2g20260 ,RAFL04-15-M14  photosystem I reaction center subunit IV, chloroplast, putative / PSI-E, putative (PSAE2) identical to SP|Q9S714; similar to SP|P12354 Photosystem I reaction center subunit IV, chloroplast precursor (PSI-E) {Spinacia oleracea}; contains Pfam profile PF02427: Photosystem I reaction centre subunit IV / PsaE | | | | | | |
|  |  | RAFL11-03-I17 | At5g01530 / light-harvesting chlorophyll a/b binding protein | |  |  |  |  |  | | --- | --- | --- | --- | --- | |  |  |  |  |  | | RAFL11-03-I17 ,At5g01530  chlorophyll A-B binding protein CP29 (LHCB4) identical to CP29 [Arabidopsis thaliana] GI:298036; contains Pfam profile: PF00504 chlorophyll A-B binding protein | | | | | | |
|  | Cluster:0-1 | |  |  | 12 | 94 | 242 | 4315 | 0.014377407 | 0.43132222 | 30 |
|  |  | RAFL04-12-M04 | At5g49730 / ferric reductase-like transmembrane component family | |  |  |  |  |  | | --- | --- | --- | --- | --- | |  |  |  |  |  | | At5g49730 ,RAFL04-12-M04  ferric reductase-like transmembrane component family protein similar to ferric-chelate reductase (FRO1) [Pisum sativum] GI:15341529; contains Pfam profile PF01794: Ferric reductase like transmembrane componenent | | | | | | |
|  |  | RAFL04-20-D12 | At3g16250 / ferredoxin - related | |  |  |  |  |  | | --- | --- | --- | --- | --- | |  |  |  |  |  | | RAFL04-20-D12 ,At3g16250  ferredoxin-related contains Pfam profile: PF00111 2Fe-2S iron-sulfur cluster binding domains | | | | | | |
|  |  | RAFL05-19-N12 | At2g06520 / expressed protein | |  |  |  |  |  | | --- | --- | --- | --- | --- | |  |  |  |  |  | | At2g06520 ,RAFL05-19-N12  membrane protein, putative contains 2 transmembrane domains; | | | | | | |
|  |  | RAFL08-09-L12 | At3g26060 / peroxiredoxin -related | |  |  |  |  |  | | --- | --- | --- | --- | --- | |  |  |  |  |  | | RAFL08-09-L12 ,At3g26060  peroxiredoxin Q, putative similar to peroxiredoxin Q [Sedum lineare] GI:6899842; contains Pfam profile: PF00578 AhpC/TSA (alkyl hydroperoxide reductase and thiol-specific antioxidant) family | | | | | | |
|  |  | RAFL03-06-N04 | At1g55670 / photosystem I subunit V precursor -related | |  |  |  |  |  | | --- | --- | --- | --- | --- | |  |  |  |  |  | | RAFL03-06-N04 ,At1g55670  photosystem I reaction center subunit V, chloroplast, putative / PSI-G, putative (PSAG) identical to SP|Q9S7N7; similar to SP|Q00327 Photosystem I reaction center subunit V, chloroplast precursor (PSI-G) (Photosystem I 9 kDa protein) {Hordeum vulgare}; contains Pfam profile PF01241: Photosystem I psaG / psaK | | | | | | |
|  |  | RAFL05-04-D24 | At1g52230 / photosystem I subunit VI precursor | |  |  |  |  |  | | --- | --- | --- | --- | --- | |  |  |  |  |  | | At1g52230 ,RAFL05-04-D24  photosystem I reaction center subunit VI, chloroplast, putative / PSI-H, putative (PSAH2) identical to SP|Q9SUI6; similar to PSI-H precursor [Nicotiana sylvestris] GI:407355; contains Pfam profile PF03244: Photosystem I reaction centre subunit VI | | | | | | |
|  |  | RAFL02-04-K03 | At1g20340 / plastocyanin | |  |  |  |  |  | | --- | --- | --- | --- | --- | |  |  |  |  |  | | At1g20340 ,RAFL02-04-K03  plastocyanin similar to plastocyanin GI:1865683 from [Arabidopsis thaliana] | | | | | | |
|  |  | RAFL05-16-P12 | At1g67740 / F12A21.13 | |  |  |  |  |  | | --- | --- | --- | --- | --- | |  |  |  |  |  | | RAFL05-16-P12 ,At1g67740  photosystem II core complex proteins psbY, chloroplast (PSBY) / L-arginine metabolising enzyme identical to SP:O49347 Photosystem II core complex proteins psbY, chloroplast precursor (L-arginine metabolising enzyme) (L-AME) [Contains: Photosystem II protein psbY-1 (psbY-A1); Photosystem II protein psbY-2 (psbY-A2)] [Arabidopsis thaliana] | | | | | | |
|  |  | RAFL05-17-G17 | At4g21280 / oxygen-evolving complex protein 16, chloroplast precursor (OEC16) | |  |  |  |  |  | | --- | --- | --- | --- | --- | |  |  |  |  |  | | RAFL05-17-G17 ,At4g21280  oxygen-evolving enhancer protein 3, chloroplast, putative (PSBQ1) (PSBQ) identical to SP|Q9XFT3 Oxygen-evolving enhancer protein 3-1, chloroplast precursor (OEE3) (16 kDa subunit of oxygen evolving system of photosystem II) (OEC 16 kDa subunit) {Arabidopsis thaliana}; similar to SP|P12301 Oxygen-evolving enhancer protein 3, chloroplast precursor (OEE3) (16 kDa subunit of oxygen evolving system of photosystem II) (OEC 16 kDa subunit) {Spinacia oleracea}; contains Pfam profile PF05757: Oxygen evolving enhancer protein 3 (PsbQ) | | | | | | |
|  |  | RAFL07-07-I23 | At1g56190 / phosphoglycerate kinase -related | |  |  |  |  |  | | --- | --- | --- | --- | --- | |  |  |  |  |  | | RAFL07-07-I23 ,At1g56190  phosphoglycerate kinase, putative similar to SP|P41758 Phosphoglycerate kinase, chloroplast precursor (EC 2.7.2.3) {Chlamydomonas reinhardtii}; contains Pfam profile PF00162: phosphoglycerate kinase | | | | | | |
|  |  | RAFL05-01-P13 | At4g02770 / photosystem I reaction center subunit II precursor -related | |  |  |  |  |  | | --- | --- | --- | --- | --- | |  |  |  |  |  | | At4g02770 ,RAFL05-01-P13  photosystem I reaction center subunit II, chloroplast, putative / photosystem I 20 kDa subunit, putative / PSI-D, putative (PSAD1) similar to SP|P12353 Photosystem I reaction center subunit II, chloroplast precursor (Photosystem I 20 kDa subunit) (PSI-D) {Spinacia oleracea}; contains Pfam profile PF02531: PsaD | | | | | | |
|  |  | RAFL06-10-O24 | At1g15820 / chlorophyll a/b-binding protein Lhcb6 | |  |  |  |  |  | | --- | --- | --- | --- | --- | |  |  |  |  |  | | At1g15820 ,RAFL06-10-O24  chlorophyll A-B binding protein, chloroplast (LHCB6) nearly identical to Lhcb6 protein [Arabidopsis thaliana] GI:4741960; contains Pfam profile PF00504: Chlorophyll A-B binding protein | | | | | | |
| GO:0003700,GO:0000130 | | | transcription factor activity |  | A | B | C | D | P | P' | N |
|  | Cluster:0-0 | |  |  | 2 | 34 | 14 | 4613 | 0.006497732 | 0.07147505 | 11 |
|  |  | RAFL07-12-I10 | At5g42010 / expressed protein | |  |  |  |  |  | | --- | --- | --- | --- | --- | |  |  |  |  |  | | At5g42010 ,RAFL07-12-I10  WD-40 repeat family protein contains Pfam PF00400: WD domain, G-beta repeat; similar to WD-repeat protein 5 (WD repeat protein BIG-3) (SP: Q9UGP9) [Homo sapiens] | | | | | | |
|  |  | RAFL07-10-I15 | At5g23060 / expressed protein | |  |  |  |  |  | | --- | --- | --- | --- | --- | |  |  |  |  |  | | At5g23060 ,RAFL07-10-I15  expressed protein | | | | | | |
|  | Cluster:6-2 | |  |  | 3 | 173 | 13 | 4474 | 0.020600509 | 0.2266056 | 11 |
|  |  | RAFL09-13-M21 | At3g15470 / expressed protein | |  |  |  |  |  | | --- | --- | --- | --- | --- | |  |  |  |  |  | | RAFL09-13-M21 ,At3g15470  WD-40 repeat family protein contains Pfam PF00400: WD domain, G-beta repeat; similar to WD-repeat protein 5 (WD repeat protein BIG-3) (SP: Q9UGP9) [Homo sapiens] | | | | | | |
|  |  | RAFL05-08-A21 | At1g53090 / WD-40 repeat protein family | |  |  |  |  |  | | --- | --- | --- | --- | --- | |  |  |  |  |  | | RAFL05-08-A21 ,At1g53090  WD-40 repeat family protein / phytochrome A-related contains 7 WD-40 repeats (PF00400) (1 below cutoff); similar to phytochrome A supressor spa1 (GI:4809171) [Arabidopsis thaliana] | | | | | | |
|  |  | RAFL05-07-J03 | At4g40050 / expressed protein | |  |  |  |  |  | | --- | --- | --- | --- | --- | |  |  |  |  |  | | At4g40050 ,RAFL05-07-J03  expressed protein | | | | | | |
| GO:0007275 | | | development |  | A | B | C | D | P | P' | N |
|  | Cluster:10-1 | |  |  | 5 | 38 | 39 | 4581 | 4.3707958E-5 | 9.178671E-4 | 21 |
|  |  | RAFL09-15-E01 | At4g27410 / No apical meristem (NAM) protein family | |  |  |  |  |  | | --- | --- | --- | --- | --- | |  |  |  |  |  | | At4g27410 ,RAFL09-15-E01  no apical meristem (NAM) family protein (RD26) contains Pfam PF02365: No apical meristem (NAM) domain; Arabidopsis thaliana nap gene,PID:e1234813; identical to cDNA RD26 mRNA for NAM-like protein GI:15375403 | | | | | | |
|  |  | RAFL03-07-M07 | At4g02380 / late embryogenesis abundant protein family | |  |  |  |  |  | | --- | --- | --- | --- | --- | |  |  |  |  |  | | RAFL03-07-M07 ,At4g02380  late embryogenesis abundant 3 family protein / LEA3 family protein similar to several small proteins (~100 aa) that are induced by heat, auxin, ethylene and wounding such as Phaseolus aureus indole-3-acetic acid induced protein ARG (SW:32292); contains Pfam profile PF03242: Late embryogenesis abundant protein | | | | | | |
|  |  | RAFL06-13-N20 | At4g02380 / late embryogenesis abundant protein family | |  |  |  |  |  | | --- | --- | --- | --- | --- | |  |  |  |  |  | | RAFL06-13-N20 ,At4g02380  late embryogenesis abundant 3 family protein / LEA3 family protein similar to several small proteins (~100 aa) that are induced by heat, auxin, ethylene and wounding such as Phaseolus aureus indole-3-acetic acid induced protein ARG (SW:32292); contains Pfam profile PF03242: Late embryogenesis abundant protein | | | | | | |
|  |  | RAFL08-11-C23 | At5g06760 / late embryogenesis abundant protein LEA like | |  |  |  |  |  | | --- | --- | --- | --- | --- | |  |  |  |  |  | | RAFL08-11-C23 ,At5g06760  late embryogenesis abundant group 1 domain-containing protein / LEA group 1 domain-containing protein low similarity to SP|P46515 11 kDa late embryogenesis abundant protein (DS11) {Helianthus annuus}; contains Pfam profile PF03760: Late embryogenesis abundant (LEA) group 1 | | | | | | |
|  |  | RAFL05-21-C17 | At4g27410 / No apical meristem (NAM) protein family | |  |  |  |  |  | | --- | --- | --- | --- | --- | |  |  |  |  |  | | At4g27410 ,RAFL05-21-C17  no apical meristem (NAM) family protein (RD26) contains Pfam PF02365: No apical meristem (NAM) domain; Arabidopsis thaliana nap gene,PID:e1234813; identical to cDNA RD26 mRNA for NAM-like protein GI:15375403 | | | | | | |
| GO:0006950 | | | response to stress |  | A | B | C | D | P | P' | N |
|  | Cluster:2-1 | |  |  | 50 | 194 | 185 | 4234 | 7.7307773E-19 | 1.8553866E-17 | 24 |
|  |  | RAFL09-15-G07 | At3g62250 / ubiquitin extension protein (UBQ5)/40S ribosomal protein S27A (RPS27aC) | |  |  |  |  |  | | --- | --- | --- | --- | --- | |  |  |  |  |  | | RAFL09-15-G07 ,At3g62250  ubiquitin extension protein 5 (UBQ5) / 40S ribosomal protein S27A (RPS27aC) identical to GI:166933, GI:166934 | | | | | | |
|  |  | RAFL07-11-J16 | At3g15950 / expressed protein | |  |  |  |  |  | | --- | --- | --- | --- | --- | |  |  |  |  |  | | RAFL07-11-J16 ,At3g15950  DNA topoisomerase-related similar to DNA topoisomerase IV subunit A (GI:26454107) [Mycoplasma penetrans] | | | | | | |
|  |  | RAFL11-10-K08 | At3g22230 / 60S ribosomal protein L27 (RPL27B) | |  |  |  |  |  | | --- | --- | --- | --- | --- | |  |  |  |  |  | | At3g22230 ,RAFL11-10-K08  60S ribosomal protein L27 (RPL27B) similar to 60S RIBOSOMAL PROTEIN L27 GB:P41101 from [Solanum tuberosum] | | | | | | |
|  |  | RAFL05-17-M03 | At5g56710 / 60S ribosomal protein L31 (RPL31C) | |  |  |  |  |  | | --- | --- | --- | --- | --- | |  |  |  |  |  | | At5g56710 ,RAFL05-17-M03  60S ribosomal protein L31 (RPL31C) | | | | | | |
|  |  | RAFL05-17-L17 | At3g55280 / 60S ribosomal protein L23A (RPL23aB) | |  |  |  |  |  | | --- | --- | --- | --- | --- | |  |  |  |  |  | | RAFL05-17-L17 ,At3g55280  60S ribosomal protein L23A (RPL23aB) various ribosomal L23a proteins | | | | | | |
|  |  | RAFL09-12-B05 | At5g02870 / 60S ribosomal protein L4/L1 (RPL4D) | |  |  |  |  |  | | --- | --- | --- | --- | --- | |  |  |  |  |  | | At5g02870 ,RAFL09-12-B05  60S ribosomal protein L4/L1 (RPL4D) 60S roibosomal protein L4, Arabidopsis thaliana, EMBL:CAA79104 | | | | | | |
|  |  | RAFL07-13-J18 | At2g37270 / 40S ribosomal protein S5 (RPS5A) | |  |  |  |  |  | | --- | --- | --- | --- | --- | |  |  |  |  |  | | At2g37270 ,RAFL07-13-J18  40S ribosomal protein S5 (RPS5A) identical to GP:3043428 | | | | | | |
|  |  | RAFL06-07-H08 | At2g27710 / 60S acidic ribosomal protein P2 (RPP2B) | |  |  |  |  |  | | --- | --- | --- | --- | --- | |  |  |  |  |  | | At2g27710 ,RAFL06-07-H08  60S acidic ribosomal protein P2 (RPP2B) | | | | | | |
|  |  | RAFL05-08-H10 | At4g16720 / 60S ribosomal protein L15 (RPL15A) | |  |  |  |  |  | | --- | --- | --- | --- | --- | |  |  |  |  |  | | RAFL05-08-H10 ,At4g16720  60S ribosomal protein L15 (RPL15A) | | | | | | |
|  |  | RAFL09-12-K01 | At4g00810 / 60S acidic ribosomal protein P1 (RPP1B) | |  |  |  |  |  | | --- | --- | --- | --- | --- | |  |  |  |  |  | | At4g00810 ,RAFL09-12-K01  60S acidic ribosomal protein P1 (RPP1B) similar to acidic ribosomal protein p1 | | | | | | |
|  |  | RAFL05-17-F03 | At2g32060 / 40S ribosomal protein S12 (RPS12C) | |  |  |  |  |  | | --- | --- | --- | --- | --- | |  |  |  |  |  | | RAFL05-17-F03 ,At2g32060  40S ribosomal protein S12 (RPS12C) | | | | | | |
|  |  | RAFL05-03-N22 | At3g15410 / leucine rich repeat protein family | |  |  |  |  |  | | --- | --- | --- | --- | --- | |  |  |  |  |  | | RAFL05-03-N22 ,At3g15410  leucine-rich repeat family protein contains leucine rich-repeat (LRR) domains Pfam:PF00560, INTERPRO:IPR001611; contains similarity to Hcr2-5D [Lycopersicon esculentum] gi|3894393|gb|AAC78596; identical to leucine-rich repeat protein [Arabidopsis thaliana] gi|2760084|emb|CAA76000 | | | | | | |
|  |  | RAFL05-07-H16 | At3g53870 / 40S ribosomal protein S3 (RPS3B) | |  |  |  |  |  | | --- | --- | --- | --- | --- | |  |  |  |  |  | | At3g53870 ,RAFL05-07-H16  40S ribosomal protein S3 (RPS3B) ribosomal protein S3a - Xenopus laevis, PIR:R3XL3A | | | | | | |
|  |  | RAFL07-15-M07 | At1g04480 / 60S ribosomal protein L23 (RPL23A) | |  |  |  |  |  | | --- | --- | --- | --- | --- | |  |  |  |  |  | | At1g04480 ,RAFL07-15-M07  60S ribosomal protein L23 (RPL23A) identical to GB:AAB80655 | | | | | | |
|  |  | RAFL05-14-D21 | At1g18540 / 60S ribosomal protein L6 (RPL6A) | |  |  |  |  |  | | --- | --- | --- | --- | --- | |  |  |  |  |  | | At1g18540 ,RAFL05-14-D21  60S ribosomal protein L6 (RPL6A) similar to 60S ribosomal protein L6 GI:7208784 from [Cicer arietinum] | | | | | | |
|  |  | RAFL08-11-K22 | At1g57660 / 60S ribosomal protein L21 (RPL21E) | |  |  |  |  |  | | --- | --- | --- | --- | --- | |  |  |  |  |  | | At1g57660 ,RAFL08-11-K22  60S ribosomal protein L21 (RPL21E) similar to 60S ribosomal protein L21 GB:Q43291 GI:2851508 from [Arabidopsis thaliana] | | | | | | |
|  |  | RAFL11-12-H04 | At3g25520 / 60S ribosomal protein L5 (RPL5A) | |  |  |  |  |  | | --- | --- | --- | --- | --- | |  |  |  |  |  | | At3g25520 ,RAFL11-12-H04  60S ribosomal protein L5 similar to 60S ribosomal protein L5 GB:P49625 from [Oryza sativa] | | | | | | |
|  |  | RAFL04-18-N22 | At2g44120 / 60S ribosomal protein L7 (RPL7C) | |  |  |  |  |  | | --- | --- | --- | --- | --- | |  |  |  |  |  | | RAFL04-18-N22 ,At2g44120  60S ribosomal protein L7 (RPL7C) | | | | | | |
|  |  | RAFL09-07-D04 | At3g22230 / 60S ribosomal protein L27 (RPL27B) | |  |  |  |  |  | | --- | --- | --- | --- | --- | |  |  |  |  |  | | At3g22230 ,RAFL09-07-D04  60S ribosomal protein L27 (RPL27B) similar to 60S RIBOSOMAL PROTEIN L27 GB:P41101 from [Solanum tuberosum] | | | | | | |
|  |  | RAFL05-18-M20 | At2g19730 / 60S ribosomal protein L28 (RPL28A) | |  |  |  |  |  | | --- | --- | --- | --- | --- | |  |  |  |  |  | | At2g19730 ,RAFL05-18-M20  60S ribosomal protein L28 (RPL28A) | | | | | | |
|  |  | RAFL07-08-E24 | At5g27850 / 60S ribosomal protein L18 (RPL18C) | |  |  |  |  |  | | --- | --- | --- | --- | --- | |  |  |  |  |  | | RAFL07-08-E24 ,At5g27850  60S ribosomal protein L18 (RPL18C) 60S ribosomal protein L18, Arabidopsis thaliana, SWISSPROT:RL18\_ARATH | | | | | | |
|  |  | RAFL11-07-B21 | At3g25520 / 60S ribosomal protein L5 (RPL5A) | |  |  |  |  |  | | --- | --- | --- | --- | --- | |  |  |  |  |  | | At3g25520 ,RAFL11-07-B21  60S ribosomal protein L5 similar to 60S ribosomal protein L5 GB:P49625 from [Oryza sativa] | | | | | | |
|  |  | RAFL04-20-F03 | At5g39740 / 60S ribosomal protein L5 (RPL5B) | |  |  |  |  |  | | --- | --- | --- | --- | --- | |  |  |  |  |  | | RAFL04-20-F03 ,At5g39740  60S ribosomal protein L5 (RPL5B) ribosomal protein L5, rice | | | | | | |
|  |  | RAFL11-05-B21 | At3g54210 / ribosomal protein L17 -related protein | |  |  |  |  |  | | --- | --- | --- | --- | --- | |  |  |  |  |  | | At3g54210 ,RAFL11-05-B21  ribosomal protein L17 family protein contains Pfam profile: PF01196 ribosomal protein L17 | | | | | | |
|  |  | RAFL05-02-K09 | At5g22440 / 60S ribosomal protein L10A (RPL10aC) | |  |  |  |  |  | | --- | --- | --- | --- | --- | |  |  |  |  |  | | RAFL05-02-K09 ,At5g22440  60S ribosomal protein L10A (RPL10aC) | | | | | | |
|  |  | RAFL05-05-M24 | At5g02960 / 40S ribosomal protein S23 (RPS23B) | |  |  |  |  |  | | --- | --- | --- | --- | --- | |  |  |  |  |  | | At5g02960 ,RAFL05-05-M24  40S ribosomal protein S23 (RPS23B) ribosomal protein S23, Fragaria x ananassa, PIR:S56673 | | | | | | |
|  |  | RAFL05-18-P15 | At1g04480 / 60S ribosomal protein L23 (RPL23A) | |  |  |  |  |  | | --- | --- | --- | --- | --- | |  |  |  |  |  | | At1g04480 ,RAFL05-18-P15  60S ribosomal protein L23 (RPL23A) identical to GB:AAB80655 | | | | | | |
|  |  | RAFL04-09-J06 | At1g70600 / 60S ribosomal protein L27A (RPL27aC) | |  |  |  |  |  | | --- | --- | --- | --- | --- | |  |  |  |  |  | | At1g70600 ,RAFL04-09-J06  60S ribosomal protein L27A (RPL27aC) identical to 60S ribosomal protein L27A GB:P49637 [Arabidopsis thaliana] | | | | | | |
|  |  | RAFL04-14-L08 | At3g49010 / 60S ribosomal protein L13 (RPL13B)/breast basic conserved protein 1-related (BBC1) | |  |  |  |  |  | | --- | --- | --- | --- | --- | |  |  |  |  |  | | At3g49010 ,RAFL04-14-L08  60S ribosomal protein L13 (RPL13B) / breast basic conserved protein 1-related (BBC1) | | | | | | |
|  |  | RAFL06-10-J18 | At4g14320 / 60S ribosomal protein L36a/L44 (RPL36aB) | |  |  |  |  |  | | --- | --- | --- | --- | --- | |  |  |  |  |  | | RAFL06-10-J18 ,At4g14320  60S ribosomal protein L36a/L44 (RPL36aB) | | | | | | |
|  |  | RAFL11-09-J03 | At3g54210 / ribosomal protein L17 -related protein | |  |  |  |  |  | | --- | --- | --- | --- | --- | |  |  |  |  |  | | RAFL11-09-J03 ,At3g54210  ribosomal protein L17 family protein contains Pfam profile: PF01196 ribosomal protein L17 | | | | | | |
|  |  | RAFL02-10-A09 | At1g33140 / 60S ribosomal protein L9 (RPL90A/C) | |  |  |  |  |  | | --- | --- | --- | --- | --- | |  |  |  |  |  | | RAFL02-10-A09 ,At1g33140  60S ribosomal protein L9 (RPL90A/C) similar to RIBOSOMAL PROTEIN L9 GB:P49209 from [Arabidopsis thaliana] | | | | | | |
|  |  | RAFL07-14-M14 | At5g02870 / 60S ribosomal protein L4/L1 (RPL4D) | |  |  |  |  |  | | --- | --- | --- | --- | --- | |  |  |  |  |  | | At5g02870 ,RAFL07-14-M14  60S ribosomal protein L4/L1 (RPL4D) 60S roibosomal protein L4, Arabidopsis thaliana, EMBL:CAA79104 | | | | | | |
|  |  | RAFL09-06-A22 | At3g62870 / 60S ribosomal protein L7A (RPL7aB) | |  |  |  |  |  | | --- | --- | --- | --- | --- | |  |  |  |  |  | | RAFL09-06-A22 ,At3g62870  60S ribosomal protein L7A (RPL7aB) 60S RIBOSOMAL PROTEIN L7A - Oryza sativa, SWISSPROT:RL7A\_ORYSA | | | | | | |
|  |  | RAFL04-09-M12 | At4g27090 / 60S ribosomal protein L14 (RPL14B) | |  |  |  |  |  | | --- | --- | --- | --- | --- | |  |  |  |  |  | | At4g27090 ,RAFL04-09-M12  60S ribosomal protein L14 (RPL14B) ribosomal protein L14 - Human,PIR3:JC5954 | | | | | | |
|  |  | RAFL11-10-L18 | At2g36620 / 60S ribosomal protein L24 (RPL24A) | |  |  |  |  |  | | --- | --- | --- | --- | --- | |  |  |  |  |  | | RAFL11-10-L18 ,At2g36620  60S ribosomal protein L24 (RPL24A) | | | | | | |
|  |  | RAFL06-10-E08 | At2g34480 / 60S ribosomal protein L18A (RPL18aB) | |  |  |  |  |  | | --- | --- | --- | --- | --- | |  |  |  |  |  | | At2g34480 ,RAFL06-10-E08  60S ribosomal protein L18A (RPL18aB) | | | | | | |
|  |  | RAFL05-03-L01 | At3g56340 / 40S ribosomal protein S26 homolog | |  |  |  |  |  | | --- | --- | --- | --- | --- | |  |  |  |  |  | | At3g56340 ,RAFL05-03-L01  40S ribosomal protein S26 (RPS26C) several 40S ribosomal protein S26 | | | | | | |
|  |  | RAFL06-08-P20 | At3g07110 / 60S ribosomal protein L13A (RPL13aA) | |  |  |  |  |  | | --- | --- | --- | --- | --- | |  |  |  |  |  | | RAFL06-08-P20 ,At3g07110  60S ribosomal protein L13A (RPL13aA) similar to ribosomal protein L13A GB:O49885 [Lupinus luteus] | | | | | | |
|  |  | RAFL04-10-H14 | At3g49910 / 60S ribosomal protein L26 (RPL26A) | |  |  |  |  |  | | --- | --- | --- | --- | --- | |  |  |  |  |  | | At3g49910 ,RAFL04-10-H14  60S ribosomal protein L26 (RPL26A) 60S RIBOSOMAL PROTEIN L26, Brassica rapa, EMBL:BRD495 | | | | | | |
|  |  | RAFL05-13-M17 | At3g53890 / 40S ribosomal protein S21 homolog | |  |  |  |  |  | | --- | --- | --- | --- | --- | |  |  |  |  |  | | RAFL05-13-M17 ,At3g53890  40S ribosomal protein S21 (RPS21B) ribosomal protein S21, cytosolic - Oryza sativa, PIR:S38357 | | | | | | |
|  |  | RAFL06-11-K09 | At5g03850 / 40S ribosomal protein S28 (RPS28B) | |  |  |  |  |  | | --- | --- | --- | --- | --- | |  |  |  |  |  | | RAFL06-11-K09 ,At5g03850  40S ribosomal protein S28 (RPS28B) ribosomal protein S28, Arabidopsis thaliana, EMBL:ATRP28A | | | | | | |
|  |  | RAFL05-16-K23 | At1g02780 / 60S ribosomal protein L19 (RPL19A) | |  |  |  |  |  | | --- | --- | --- | --- | --- | |  |  |  |  |  | | RAFL05-16-K23 ,At1g02780  60S ribosomal protein L19 (RPL19A) similar to ribosomal protein L19 GI:36127 from [Homo sapiens] | | | | | | |
|  |  | RAFL05-19-H05 | At3g28900 / 60S ribosomal protein L34 (RPL34C) | |  |  |  |  |  | | --- | --- | --- | --- | --- | |  |  |  |  |  | | RAFL05-19-H05 ,At3g28900  60S ribosomal protein L34 (RPL34C) similar to 60S ribosomal protein L34 GB:P41098 [Nicotiana tabacum] | | | | | | |
|  |  | RAFL04-18-P03 | At4g17390 / 60S ribosomal protein L15 (RPL15B) | |  |  |  |  |  | | --- | --- | --- | --- | --- | |  |  |  |  |  | | At4g17390 ,RAFL04-18-P03  60S ribosomal protein L15 (RPL15B) | | | | | | |
|  |  | RAFL05-17-P11 | At3g04920 / 40S ribosomal protein S24 (RPS24A) | |  |  |  |  |  | | --- | --- | --- | --- | --- | |  |  |  |  |  | | At3g04920 ,RAFL05-17-P11  40S ribosomal protein S24 (RPS24A) similar to ribosomal protein S19 GB:445612 [Solanum tuberosum] and similar to ribosomal protein S24 GB:4506703 [Homo sapiens] | | | | | | |
|  |  | RAFL05-13-D18 | At3g04400 / 60S ribosomal protein L23 (RPL23C) | |  |  |  |  |  | | --- | --- | --- | --- | --- | |  |  |  |  |  | | At3g04400 ,RAFL05-13-D18  60S ribosomal protein L23 (RPL23C) similar to ribosomal protein L17 GB:AAA34113.1 from [Nicotiana tabacum] | | | | | | |
|  |  | RAFL06-08-P08 | At3g23390 / 60S ribosomal protein L36a/L44 (RPL36aA) | |  |  |  |  |  | | --- | --- | --- | --- | --- | |  |  |  |  |  | | RAFL06-08-P08 ,At3g23390  60S ribosomal protein L36a/L44 (RPL36aA) similar to ribosomal protein L41 GB:AAA34366 from [Candida maltosa] | | | | | | |
|  |  | RAFL11-06-J19 | At2g01250 / 60S ribosomal protein L7 (RPL7B) | |  |  |  |  |  | | --- | --- | --- | --- | --- | |  |  |  |  |  | | At2g01250 ,RAFL11-06-J19  60S ribosomal protein L7 (RPL7B) | | | | | | |
|  |  | RAFL11-09-C11 | At2g01250 / 60S ribosomal protein L7 (RPL7B) | |  |  |  |  |  | | --- | --- | --- | --- | --- | |  |  |  |  |  | | At2g01250 ,RAFL11-09-C11  60S ribosomal protein L7 (RPL7B) | | | | | | |
|  | Cluster:1-2 | |  |  | 29 | 145 | 206 | 4283 | 6.078509E-9 | 1.4588421E-7 | 24 |
|  |  | RAFL06-16-L14 | At5g48760 / 60S ribosomal protein L13A (RPL13aD) | |  |  |  |  |  | | --- | --- | --- | --- | --- | |  |  |  |  |  | | RAFL06-16-L14 ,At5g48760  60S ribosomal protein L13A (RPL13aD) | | | | | | |
|  |  | RAFL09-07-N01 | At2g04390 / 40S ribosomal protein S17 (RPS17A) | |  |  |  |  |  | | --- | --- | --- | --- | --- | |  |  |  |  |  | | At2g04390 ,RAFL09-07-N01  40S ribosomal protein S17 (RPS17A) | | | | | | |
|  |  | RAFL11-10-E06 | At5g45775 / 60S ribosomal protein L11 (RPL11D) | |  |  |  |  |  | | --- | --- | --- | --- | --- | |  |  |  |  |  | | RAFL11-10-E06 ,At5g45775  60S ribosomal protein L11 (RPL11D) | | | | | | |
|  |  | RAFL05-21-A12 | At4g29060 / expressed protein | |  |  |  |  |  | | --- | --- | --- | --- | --- | |  |  |  |  |  | | RAFL05-21-A12 ,At4g29060  elongation factor Ts family protein similar to SP|P35019 Elongation factor Ts (EF-Ts) {Galdieria sulphuraria}; contains Pfam profiles PF00627: UBA/TS-N domain, PF00889: Elongation factor TS, PF00575: S1 RNA binding domain | | | | | | |
|  |  | RAFL04-10-C15 | At1g09590 / 60S ribosomal protein L21 (RPL21A) | |  |  |  |  |  | | --- | --- | --- | --- | --- | |  |  |  |  |  | | RAFL04-10-C15 ,At1g09590  60S ribosomal protein L21 (RPL21A) Similar to L21 family of ribosomal protein; amino acid sequence is identical to F21M12.8 | | | | | | |
|  |  | RAFL06-10-D03 | At5g67510 / 60S ribosomal protein L26 (RPL26B) | |  |  |  |  |  | | --- | --- | --- | --- | --- | |  |  |  |  |  | | At5g67510 ,RAFL06-10-D03  60S ribosomal protein L26 (RPL26B) | | | | | | |
|  |  | RAFL04-12-O17 | At5g30510 / ribosomal protein S1 | |  |  |  |  |  | | --- | --- | --- | --- | --- | |  |  |  |  |  | | At5g30510 ,RAFL04-12-O17  30S ribosomal protein S1, putative similar to Swiss-Prot:P29344 30S ribosomal protein S1, chloroplast precursor (CS1) [Spinacia oleracea] | | | | | | |
|  |  | RAFL06-08-P05 | At3g25520 / 60S ribosomal protein L5 (RPL5A) | |  |  |  |  |  | | --- | --- | --- | --- | --- | |  |  |  |  |  | | At3g25520 ,RAFL06-08-P05  60S ribosomal protein L5 similar to 60S ribosomal protein L5 GB:P49625 from [Oryza sativa] | | | | | | |
|  |  | RAFL06-11-J01 | At1g09690 / 60S ribosomal protein L21 (RPL21C) | |  |  |  |  |  | | --- | --- | --- | --- | --- | |  |  |  |  |  | | RAFL06-11-J01 ,At1g09690  60S ribosomal protein L21 (RPL21C) Similar to ribosomal protein L21 (gb|L38826). ESTs gb|AA395597,gb|ATTS5197 come from this gene | | | | | | |
|  |  | RAFL05-03-J07 | At1g27400 / 60S ribosomal protein L17 (RPL17A) | |  |  |  |  |  | | --- | --- | --- | --- | --- | |  |  |  |  |  | | At1g27400 ,RAFL05-03-J07  60S ribosomal protein L17 (RPL17A) similar to GB:P51413 from [Arabidopsis thaliana]; similar to ESTs gb|L33542 and gb|AA660016 | | | | | | |
|  |  | RAFL09-15-H22 | At1g05190 / ribosomal protein L6p family | |  |  |  |  |  | | --- | --- | --- | --- | --- | |  |  |  |  |  | | At1g05190 ,RAFL09-15-H22  ribosomal protein L6 family protein Similar to Mycobacterium RlpF (gb|Z84395). ESTs gb|T75785,gb|R30580,gb|T04698 come from this gene | | | | | | |
|  |  | RAFL05-14-H02 | At4g34620 / ribosomal protein S16p family | |  |  |  |  |  | | --- | --- | --- | --- | --- | |  |  |  |  |  | | RAFL05-14-H02 ,At4g34620  ribosomal protein S16 family protein ribosomal protein S16, Neurospora crassa, PIR2:A29927 | | | | | | |
|  |  | RAFL05-03-L24 | At5g60670 / 60S ribosomal protein L12 (RPL12C) | |  |  |  |  |  | | --- | --- | --- | --- | --- | |  |  |  |  |  | | RAFL05-03-L24 ,At5g60670  60S ribosomal protein L12 (RPL12C) 60S RIBOSOMAL PROTEIN L12 (like), Arabidopsis thaliana, PIR:T45883 | | | | | | |
|  |  | RAFL06-08-N13 | At3g49010 / 60S ribosomal protein L13 (RPL13B)/breast basic conserved protein 1-related (BBC1) | |  |  |  |  |  | | --- | --- | --- | --- | --- | |  |  |  |  |  | | At3g49010 ,RAFL06-08-N13  60S ribosomal protein L13 (RPL13B) / breast basic conserved protein 1-related (BBC1) | | | | | | |
|  |  | RAFL05-04-J09 | At5g45775 / 60S ribosomal protein L11 (RPL11D) | |  |  |  |  |  | | --- | --- | --- | --- | --- | |  |  |  |  |  | | At5g45775 ,RAFL05-04-J09  60S ribosomal protein L11 (RPL11D) | | | | | | |
|  |  | RAFL05-10-D01 | At2g05220 / 40S ribosomal protein S17 (RPS17B) | |  |  |  |  |  | | --- | --- | --- | --- | --- | |  |  |  |  |  | | RAFL05-10-D01 ,At2g05220  40S ribosomal protein S17 (RPS17B) | | | | | | |
|  |  | RAFL05-18-H13 | At5g65220 / ribosomal protein L29p family | |  |  |  |  |  | | --- | --- | --- | --- | --- | |  |  |  |  |  | | RAFL05-18-H13 ,At5g65220  ribosomal protein L29 family protein contains Pfam profile PF00831: ribosomal protein L29 | | | | | | |
|  |  | RAFL05-14-G11 | At2g33370 / 60S ribosomal protein L23 (RPL23B) | |  |  |  |  |  | | --- | --- | --- | --- | --- | |  |  |  |  |  | | RAFL05-14-G11 ,At2g33370  60S ribosomal protein L23 (RPL23B) | | | | | | |
|  |  | RAFL06-07-D01 | At2g01250 / 60S ribosomal protein L7 (RPL7B) | |  |  |  |  |  | | --- | --- | --- | --- | --- | |  |  |  |  |  | | At2g01250 ,RAFL06-07-D01  60S ribosomal protein L7 (RPL7B) | | | | | | |
|  |  | RAFL04-12-J05 | At1g32990 / ribosomal protein L11p family | |  |  |  |  |  | | --- | --- | --- | --- | --- | |  |  |  |  |  | | RAFL04-12-J05 ,At1g32990  ribosomal protein L11 family protein similar to chloroplast ribosomal protein L11 GI:21312 from [Spinacia oleracea] | | | | | | |
|  |  | RAFL05-12-L08 | At2g18020 / 60S ribosomal protein L8 (RPL8A) | |  |  |  |  |  | | --- | --- | --- | --- | --- | |  |  |  |  |  | | At2g18020 ,RAFL05-12-L08  60S ribosomal protein L8 (RPL8A) | | | | | | |
|  |  | RAFL04-16-H06 | At1g08360 / 60S ribosomal protein L10A (RPL10aA) | |  |  |  |  |  | | --- | --- | --- | --- | --- | |  |  |  |  |  | | At1g08360 ,RAFL04-16-H06  60S ribosomal protein L10A (RPL10aA) similar to 60S ribosomal protein L10A GB:AAC73045 GI:3860277 from [Arabidopsis thaliana] | | | | | | |
|  |  | RAFL05-04-H07 | At3g27160 / expressed protein | |  |  |  |  |  | | --- | --- | --- | --- | --- | |  |  |  |  |  | | RAFL05-04-H07 ,At3g27160  ribosomal protein S21 family protein contains Pfam profile: PF01165 ribosomal protein S21 | | | | | | |
|  |  | RAFL05-13-G24 | At5g54600 / 50S ribosomal protein L24, chloroplast precursor (CL24) | |  |  |  |  |  | | --- | --- | --- | --- | --- | |  |  |  |  |  | | At5g54600 ,RAFL05-13-G24  50S ribosomal protein L24, chloroplast (CL24) identical to SP|P92959 50S ribosomal protein L24, chloroplast precursor {Arabidopsis thaliana} | | | | | | |
|  |  | RAFL07-16-I10 | At4g34620 / ribosomal protein S16p family | |  |  |  |  |  | | --- | --- | --- | --- | --- | |  |  |  |  |  | | RAFL07-16-I10 ,At4g34620  ribosomal protein S16 family protein ribosomal protein S16, Neurospora crassa, PIR2:A29927 | | | | | | |
|  |  | RAFL09-11-E10 | At1g17220 / translation initiation factor IF-2, chloroplast precursor | |  |  |  |  |  | | --- | --- | --- | --- | --- | |  |  |  |  |  | | RAFL09-11-E10 ,At1g17220  translation initiation factor IF-2, chloroplast, putative similar to SP|P57997|IF2C\_PHAVU Translation initiation factor IF-2, chloroplast precursor (PvIF2cp) {Phaseolus vulgaris} | | | | | | |
|  |  | RAFL04-15-L21 | At1g41880 / 60S ribosomal protein L35a (RPL35aB) | |  |  |  |  |  | | --- | --- | --- | --- | --- | |  |  |  |  |  | | RAFL04-15-L21 ,At1g41880  60S ribosomal protein L35a (RPL35aB) identical to GB:CAB81600 from [Arabidopsis thaliana] | | | | | | |
|  |  | RAFL05-19-N16 | At3g45030 / 40S ribosomal protein S20 (RPS20A) | |  |  |  |  |  | | --- | --- | --- | --- | --- | |  |  |  |  |  | | At3g45030 ,RAFL05-19-N16  40S ribosomal protein S20 (RPS20A) 40S ribsomomal proteinS20, Arabidopsis thaliana, pir:T12992 | | | | | | |
|  |  | RAFL11-12-P09 | At3g54210 / ribosomal protein L17 -related protein | |  |  |  |  |  | | --- | --- | --- | --- | --- | |  |  |  |  |  | | RAFL11-12-P09 ,At3g54210  ribosomal protein L17 family protein contains Pfam profile: PF01196 ribosomal protein L17 | | | | | | |
|  | Cluster:3-0 | |  |  | 31 | 202 | 204 | 4226 | 3.9583412E-7 | 9.500019E-6 | 24 |
|  |  | RAFL07-15-M24 | At1g74050 / 60S ribosomal protein L6 (RPL6C) | |  |  |  |  |  | | --- | --- | --- | --- | --- | |  |  |  |  |  | | At1g74050 ,RAFL07-15-M24  60S ribosomal protein L6 (RPL6C) similar to 60S ribosomal protein L6 (YL 16 like) GB:CAB57309 from [Cyanophora paradoxa] | | | | | | |
|  |  | RAFL04-15-D01 | At3g09200 / 60S acidic ribosomal protein P0 (RPP0B) | |  |  |  |  |  | | --- | --- | --- | --- | --- | |  |  |  |  |  | | At3g09200 ,RAFL04-15-D01  60S acidic ribosomal protein P0 (RPP0B) similar to putative 60S acidic ribosomal protein P0 GB:P50346 [Glycine max] | | | | | | |
|  |  | RAFL09-12-B12 | At4g36130 / 60S ribosomal protein L8 (RPL8C) | |  |  |  |  |  | | --- | --- | --- | --- | --- | |  |  |  |  |  | | At4g36130 ,RAFL09-12-B12  60S ribosomal protein L8 (RPL8C) ribosomal protein L8, cytosolic, tomato, PIR1:R5TOL8 | | | | | | |
|  |  | RAFL11-07-A12 | At5g62300 / 40S ribosomal protein S20 (RPS20C) | |  |  |  |  |  | | --- | --- | --- | --- | --- | |  |  |  |  |  | | RAFL11-07-A12 ,At5g62300  40S ribosomal protein S20 (RPS20C) ribosomal protein S20, Arabidopsis thaliana, PIR:T12992 | | | | | | |
|  |  | RAFL04-15-O08 | At5g46430 / 60S ribosomal protein L32 (RPL32B) | |  |  |  |  |  | | --- | --- | --- | --- | --- | |  |  |  |  |  | | RAFL04-15-O08 ,At5g46430  60S ribosomal protein L32 (RPL32B) | | | | | | |
|  |  | RAFL08-10-G08 | At3g09630 / 60S ribosomal protein L4/L1 (RPL4A) | |  |  |  |  |  | | --- | --- | --- | --- | --- | |  |  |  |  |  | | RAFL08-10-G08 ,At3g09630  60S ribosomal protein L4/L1 (RPL4A) strong similarity to 60S ribosomal protein L1 GB:P49691 | | | | | | |
|  |  | RAFL04-16-M10 | At1g64550 / ABC transporter family protein | |  |  |  |  |  | | --- | --- | --- | --- | --- | |  |  |  |  |  | | RAFL04-16-M10 ,At1g64550  ABC transporter family protein similar to ABC transporter protein GB:AAF31030 GI:6899653 from [Leishmania major] | | | | | | |
|  |  | RAFL09-10-P09 | At4g36130 / 60S ribosomal protein L8 (RPL8C) | |  |  |  |  |  | | --- | --- | --- | --- | --- | |  |  |  |  |  | | At4g36130 ,RAFL09-10-P09  60S ribosomal protein L8 (RPL8C) ribosomal protein L8, cytosolic, tomato, PIR1:R5TOL8 | | | | | | |
|  |  | RAFL02-10-H10 | At3g43980 / 40S ribosomal protein S29 (RPS29A) | |  |  |  |  |  | | --- | --- | --- | --- | --- | |  |  |  |  |  | | At3g43980 ,RAFL02-10-H10  40S ribosomal protein S29 (RPS29A) ribosomal protein S29, rat, PIR:S30298 | | | | | | |
|  |  | RAFL05-19-G10 | At3g24830 / 60S ribosomal protein L13A (RPL13aB) | |  |  |  |  |  | | --- | --- | --- | --- | --- | |  |  |  |  |  | | RAFL05-19-G10 ,At3g24830  60S ribosomal protein L13A (RPL13aB) similar to 60S RIBOSOMAL PROTEIN L13A GB:P35427 from [Rattus norvegicus] | | | | | | |
|  |  | RAFL06-07-B02 | At3g11940 / 40S ribosomal protein S5 (RPS5B) | |  |  |  |  |  | | --- | --- | --- | --- | --- | |  |  |  |  |  | | At3g11940 ,RAFL06-07-B02  40S ribosomal protein S5 (RPS5B) similar to 40S ribosomal protein S5 GB:AAC98068 GI:4056502 from [Arabidopsis thaliana] | | | | | | |
|  |  | RAFL07-16-H23 | At3g16780 / 60S ribosomal protein L19 (RPL19B) | |  |  |  |  |  | | --- | --- | --- | --- | --- | |  |  |  |  |  | | At3g16780 ,RAFL07-16-H23  60S ribosomal protein L19 (RPL19B) similar to ribosomal protein L19 GB:CAA45090 from [Homo sapiens] | | | | | | |
|  |  | RAFL05-07-A02 | At5g47930 / ribosomal protein S27 | |  |  |  |  |  | | --- | --- | --- | --- | --- | |  |  |  |  |  | | RAFL05-07-A02 ,At5g47930  40S ribosomal protein S27 (RPS27D) | | | | | | |
|  |  | RAFL05-14-G01 | At4g02230 / 60S ribosomal protein L19 (RPL19C) | |  |  |  |  |  | | --- | --- | --- | --- | --- | |  |  |  |  |  | | RAFL05-14-G01 ,At4g02230  60S ribosomal protein L19 (RPL19C) similar to L19 from several species | | | | | | |
|  |  | RAFL06-12-J07 | At3g53020 / 60S ribosomal protein L24 (RPL24B) | |  |  |  |  |  | | --- | --- | --- | --- | --- | |  |  |  |  |  | | At3g53020 ,RAFL06-12-J07  60S ribosomal protein L24 (RPL24B) 60S ribosomal protein L24, Arabidopsis thaliana, EMBL:AC006282 | | | | | | |
|  |  | RAFL06-16-N16 | At4g16720 / 60S ribosomal protein L15 (RPL15A) | |  |  |  |  |  | | --- | --- | --- | --- | --- | |  |  |  |  |  | | RAFL06-16-N16 ,At4g16720  60S ribosomal protein L15 (RPL15A) | | | | | | |
|  |  | RAFL04-17-H14 | At3g06700 / 60S ribosomal protein L29 (RPL29A) | |  |  |  |  |  | | --- | --- | --- | --- | --- | |  |  |  |  |  | | RAFL04-17-H14 ,At3g06700  60S ribosomal protein L29 (RPL29A) similar to ribosomal protein L29 GI:7959366 [Panax ginseng] | | | | | | |
|  |  | RAFL04-09-E11 | At5g08180 / ribosomal protein L7Ae family | |  |  |  |  |  | | --- | --- | --- | --- | --- | |  |  |  |  |  | | At5g08180 ,RAFL04-09-E11  ribosomal protein L7Ae/L30e/S12e/Gadd45 family protein Similar to NHP2/L7Ae family proteins, see SWISSPROT:P32495 and PMID:2063628. | | | | | | |
|  |  | RAFL05-09-M22 | At3g49910 / 60S ribosomal protein L26 (RPL26A) | |  |  |  |  |  | | --- | --- | --- | --- | --- | |  |  |  |  |  | | RAFL05-09-M22 ,At3g49910  60S ribosomal protein L26 (RPL26A) 60S RIBOSOMAL PROTEIN L26, Brassica rapa, EMBL:BRD495 | | | | | | |
|  |  | RAFL04-14-I15 | At3g02080 / 40S ribosomal protein S19 (RPS19A) | |  |  |  |  |  | | --- | --- | --- | --- | --- | |  |  |  |  |  | | RAFL04-14-I15 ,At3g02080  40S ribosomal protein S19 (RPS19A) similar to 40S ribosomal protein S19 GB:P40978 [Oryza sativa] | | | | | | |
|  |  | RAFL11-01-C20 | At3g06590 / bHLH protein | |  |  |  |  |  | | --- | --- | --- | --- | --- | |  |  |  |  |  | | RAFL11-01-C20 ,At3g06590  expressed protein | | | | | | |
|  |  | RAFL07-16-A17 | At5g64140 / 40S ribosomal protein S28 (RPS28C) | |  |  |  |  |  | | --- | --- | --- | --- | --- | |  |  |  |  |  | | At5g64140 ,RAFL07-16-A17  40S ribosomal protein S28 (RPS28C) | | | | | | |
|  |  | RAFL07-10-D02 | At1g14320 / 60S ribosomal protein L10 (RPL10A)/Wilm's tumor suppressor protein-related | |  |  |  |  |  | | --- | --- | --- | --- | --- | |  |  |  |  |  | | At1g14320 ,RAFL07-10-D02  60S ribosomal protein L10 (RPL10A) / Wilm's tumor suppressor protein-related similar to tumor suppressor GI:575354 from [Oryza sativa] | | | | | | |
|  |  | RAFL08-13-M06 | At3g11250 / 60S acidic ribosomal protein P0 (RPP0C) | |  |  |  |  |  | | --- | --- | --- | --- | --- | |  |  |  |  |  | | At3g11250 ,RAFL08-13-M06  60S acidic ribosomal protein P0 (RPP0C) similar to 60S acidic ribosomal protein P0 GI:2088654 [Arabidopsis thaliana] | | | | | | |
|  |  | RAFL06-12-M16 | At2g27720 / 60S acidic ribosomal protein P2 (RPP2A) | |  |  |  |  |  | | --- | --- | --- | --- | --- | |  |  |  |  |  | | RAFL06-12-M16 ,At2g27720  60S acidic ribosomal protein P2 (RPP2A) | | | | | | |
|  |  | RAFL05-18-H11 | At5g27700 / ribosomal protein S21 - like | |  |  |  |  |  | | --- | --- | --- | --- | --- | |  |  |  |  |  | | RAFL05-18-H11 ,At5g27700  40S ribosomal protein S21 (RPS21C) ribosomal protein S21, Zea mays, PIR:T03945 | | | | | | |
|  |  | RAFL09-09-O07 | At4g16720 / 60S ribosomal protein L15 (RPL15A) | |  |  |  |  |  | | --- | --- | --- | --- | --- | |  |  |  |  |  | | RAFL09-09-O07 ,At4g16720  60S ribosomal protein L15 (RPL15A) | | | | | | |
|  |  | RAFL09-13-H18 | At1g43170 / 60S ribosomal protein L3 (RPL3A) | |  |  |  |  |  | | --- | --- | --- | --- | --- | |  |  |  |  |  | | At1g43170 ,RAFL09-13-H18  60S ribosomal protein L3 (RPL3A) identical to ribosomal protein GI:166858 from [Arabidopsis thaliana] | | | | | | |
|  |  | RAFL05-14-H24 | At2g21580 / 40S ribosomal protein S25 (RPS25B) | |  |  |  |  |  | | --- | --- | --- | --- | --- | |  |  |  |  |  | | At2g21580 ,RAFL05-14-H24  40S ribosomal protein S25 (RPS25B) | | | | | | |
|  |  | RAFL11-04-H04 | At2g34480 / 60S ribosomal protein L18A (RPL18aB) | |  |  |  |  |  | | --- | --- | --- | --- | --- | |  |  |  |  |  | | At2g34480 ,RAFL11-04-H04  60S ribosomal protein L18A (RPL18aB) | | | | | | |
|  |  | RAFL07-17-K15 | At2g31040 / expressed protein | |  |  |  |  |  | | --- | --- | --- | --- | --- | |  |  |  |  |  | | RAFL07-17-K15 ,At2g31040  ATP synthase protein I -related contains weaks similarity to Swiss-Prot:P08443 ATP synthase protein I [Synechococcus sp.] | | | | | | |
|  | Cluster:2-0 | |  |  | 20 | 130 | 215 | 4298 | 5.0732822E-5 | 0.0012175877 | 24 |
|  |  | RAFL06-08-N09 | At3g63490 / ribosomal protein L1p family | |  |  |  |  |  | | --- | --- | --- | --- | --- | |  |  |  |  |  | | RAFL06-08-N09 ,At3g63490  ribosomal protein L1 family protein ribosomal protein L1, S.oleracea, EMBL:SORPL1 | | | | | | |
|  |  | RAFL09-09-C01 | At2g27530 / 60S ribosomal protein L10A (RPL10aB) | |  |  |  |  |  | | --- | --- | --- | --- | --- | |  |  |  |  |  | | RAFL09-09-C01 ,At2g27530  60S ribosomal protein L10A (RPL10aB) | | | | | | |
|  |  | RAFL04-10-I16 | At1g52300 / 60S ribosomal protein L37 (RPL37B) | |  |  |  |  |  | | --- | --- | --- | --- | --- | |  |  |  |  |  | | RAFL04-10-I16 ,At1g52300  60S ribosomal protein L37 (RPL37B) similar to SP:Q43292 from [Arabidopsis thaliana] | | | | | | |
|  |  | RAFL05-02-L21 | At1g07830 / ribosomal protein L29p family | |  |  |  |  |  | | --- | --- | --- | --- | --- | |  |  |  |  |  | | At1g07830 ,RAFL05-02-L21  ribosomal protein L29 family protein similar to GB:CAA83057 from [Saccharomyces cerevisiae] | | | | | | |
|  |  | RAFL08-12-D04 | At5g20290 / 40S ribosomal protein S8 (RPS8A) | |  |  |  |  |  | | --- | --- | --- | --- | --- | |  |  |  |  |  | | At5g20290 ,RAFL08-12-D04  40S ribosomal protein S8 (RPS8A) ribosomal protein S8 - Zea mays, PIR:T04088 | | | | | | |
|  |  | RAFL03-06-H07 | At1g33140 / 60S ribosomal protein L9 (RPL90A/C) | |  |  |  |  |  | | --- | --- | --- | --- | --- | |  |  |  |  |  | | RAFL03-06-H07 ,At1g33140  60S ribosomal protein L9 (RPL90A/C) similar to RIBOSOMAL PROTEIN L9 GB:P49209 from [Arabidopsis thaliana] | | | | | | |
|  |  | RAFL05-15-M21 | At5g20160 / ribosomal protein L7Ae family | |  |  |  |  |  | | --- | --- | --- | --- | --- | |  |  |  |  |  | | At5g20160 ,RAFL05-15-M21  ribosomal protein L7Ae/L30e/S12e/Gadd45 family protein Similar to NHP2/L7Ae family proteins, see SWISSPROT:P32495 and PMID:2063628. | | | | | | |
|  |  | RAFL04-19-O24 | At5g02610 / 60S ribosomal protein L35 (RPL35D) | |  |  |  |  |  | | --- | --- | --- | --- | --- | |  |  |  |  |  | | RAFL04-19-O24 ,At5g02610  60S ribosomal protein L35 (RPL35D) ribosomal protein L35- cytosolic, Arabidopsis thaliana, PIR:T00549 | | | | | | |
|  |  | RAFL06-15-I18 | At2g40590 / 40S ribosomal protein S26 (RPS26B) | |  |  |  |  |  | | --- | --- | --- | --- | --- | |  |  |  |  |  | | RAFL06-15-I18 ,At2g40590  40S ribosomal protein S26 (RPS26B) | | | | | | |
|  |  | RAFL07-14-H10 | At5g02870 / 60S ribosomal protein L4/L1 (RPL4D) | |  |  |  |  |  | | --- | --- | --- | --- | --- | |  |  |  |  |  | | At5g02870 ,RAFL07-14-H10  60S ribosomal protein L4/L1 (RPL4D) 60S roibosomal protein L4, Arabidopsis thaliana, EMBL:CAA79104 | | | | | | |
|  |  | RAFL09-12-G10 | At4g12600 / ribosomal protein L7Ae family | |  |  |  |  |  | | --- | --- | --- | --- | --- | |  |  |  |  |  | | RAFL09-12-G10 ,At4g12600  ribosomal protein L7Ae/L30e/S12e/Gadd45 family protein Similar to NHP2/L7Ae family proteins, see SWISSPROT:P32495 and PMID:2063628. | | | | | | |
|  |  | RAFL07-18-A18 | At3g23700 / expressed protein | |  |  |  |  |  | | --- | --- | --- | --- | --- | |  |  |  |  |  | | At3g23700 ,RAFL07-18-A18  S1 RNA-binding domain-containing protein contains Pfam domain, PF00575: S1 RNA binding domain | | | | | | |
|  |  | RAFL11-12-P10 | At3g55750 / 60S ribosomal protein L35a (RPL35aD) | |  |  |  |  |  | | --- | --- | --- | --- | --- | |  |  |  |  |  | | RAFL11-12-P10 ,At3g55750  60S ribosomal protein L35a (RPL35aD) ribosomal protein L35a.e.c15, Saccharomyces cerevisiae, PIR:S44069 | | | | | | |
|  |  | RAFL11-12-M17 | At3g25520 / 60S ribosomal protein L5 (RPL5A) | |  |  |  |  |  | | --- | --- | --- | --- | --- | |  |  |  |  |  | | At3g25520 ,RAFL11-12-M17  60S ribosomal protein L5 similar to 60S ribosomal protein L5 GB:P49625 from [Oryza sativa] | | | | | | |
|  |  | RAFL09-17-M14 | At2g27530 / 60S ribosomal protein L10A (RPL10aB) | |  |  |  |  |  | | --- | --- | --- | --- | --- | |  |  |  |  |  | | RAFL09-17-M14 ,At2g27530  60S ribosomal protein L10A (RPL10aB) | | | | | | |
|  |  | RAFL05-01-L16 | At5g11750 / ribosomal protein L19, putative | |  |  |  |  |  | | --- | --- | --- | --- | --- | |  |  |  |  |  | | RAFL05-01-L16 ,At5g11750  ribosomal protein L19 family protein similar to plastid ribosomal protein L19 precursor [Spinacia oleracea] gi|7582403|gb|AAF64312 | | | | | | |
|  |  | RAFL11-13-A04 | At5g02870 / 60S ribosomal protein L4/L1 (RPL4D) | |  |  |  |  |  | | --- | --- | --- | --- | --- | |  |  |  |  |  | | At5g02870 ,RAFL11-13-A04  60S ribosomal protein L4/L1 (RPL4D) 60S roibosomal protein L4, Arabidopsis thaliana, EMBL:CAA79104 | | | | | | |
|  |  | RAFL06-10-L13 | At3g09630 / 60S ribosomal protein L4/L1 (RPL4A) | |  |  |  |  |  | | --- | --- | --- | --- | --- | |  |  |  |  |  | | RAFL06-10-L13 ,At3g09630  60S ribosomal protein L4/L1 (RPL4A) strong similarity to 60S ribosomal protein L1 GB:P49691 | | | | | | |
|  |  | RAFL05-12-K17 | At1g67430 / 60S ribosomal protein L17 (RPL17B) | |  |  |  |  |  | | --- | --- | --- | --- | --- | |  |  |  |  |  | | RAFL05-12-K17 ,At1g67430  60S ribosomal protein L17 (RPL17B) similar to ribosomal protein GI:19101 from [Hordeum vulgare] | | | | | | |
|  |  | RAFL05-15-E14 | At1g70600 / 60S ribosomal protein L27A (RPL27aC) | |  |  |  |  |  | | --- | --- | --- | --- | --- | |  |  |  |  |  | | At1g70600 ,RAFL05-15-E14  60S ribosomal protein L27A (RPL27aC) identical to 60S ribosomal protein L27A GB:P49637 [Arabidopsis thaliana] | | | | | | |
| GO:0019538,GO:0006411 | | | protein metabolism |  | A | B | C | D | P | P' | N |
|  | Cluster:2-1 | |  |  | 70 | 174 | 351 | 4068 | 5.1785943E-20 | 1.4500065E-18 | 28 |
|  |  | RAFL09-15-G07 | At3g62250 / ubiquitin extension protein (UBQ5)/40S ribosomal protein S27A (RPS27aC) | |  |  |  |  |  | | --- | --- | --- | --- | --- | |  |  |  |  |  | | RAFL09-15-G07 ,At3g62250  ubiquitin extension protein 5 (UBQ5) / 40S ribosomal protein S27A (RPS27aC) identical to GI:166933, GI:166934 | | | | | | |
|  |  | RAFL07-11-J16 | At3g15950 / expressed protein | |  |  |  |  |  | | --- | --- | --- | --- | --- | |  |  |  |  |  | | RAFL07-11-J16 ,At3g15950  DNA topoisomerase-related similar to DNA topoisomerase IV subunit A (GI:26454107) [Mycoplasma penetrans] | | | | | | |
|  |  | RAFL08-15-K17 | At5g07090 / 40S ribosomal protein S4 (RPS4B) | |  |  |  |  |  | | --- | --- | --- | --- | --- | |  |  |  |  |  | | At5g07090 ,RAFL08-15-K17  40S ribosomal protein S4 (RPS4B) | | | | | | |
|  |  | RAFL11-10-K08 | At3g22230 / 60S ribosomal protein L27 (RPL27B) | |  |  |  |  |  | | --- | --- | --- | --- | --- | |  |  |  |  |  | | At3g22230 ,RAFL11-10-K08  60S ribosomal protein L27 (RPL27B) similar to 60S RIBOSOMAL PROTEIN L27 GB:P41101 from [Solanum tuberosum] | | | | | | |
|  |  | RAFL05-17-M03 | At5g56710 / 60S ribosomal protein L31 (RPL31C) | |  |  |  |  |  | | --- | --- | --- | --- | --- | |  |  |  |  |  | | At5g56710 ,RAFL05-17-M03  60S ribosomal protein L31 (RPL31C) | | | | | | |
|  |  | RAFL05-17-L17 | At3g55280 / 60S ribosomal protein L23A (RPL23aB) | |  |  |  |  |  | | --- | --- | --- | --- | --- | |  |  |  |  |  | | RAFL05-17-L17 ,At3g55280  60S ribosomal protein L23A (RPL23aB) various ribosomal L23a proteins | | | | | | |
|  |  | RAFL09-12-B05 | At5g02870 / 60S ribosomal protein L4/L1 (RPL4D) | |  |  |  |  |  | | --- | --- | --- | --- | --- | |  |  |  |  |  | | At5g02870 ,RAFL09-12-B05  60S ribosomal protein L4/L1 (RPL4D) 60S roibosomal protein L4, Arabidopsis thaliana, EMBL:CAA79104 | | | | | | |
|  |  | RAFL02-02-F05 | At5g58420 / 40S ribosomal protein S4 (RPS4D) | |  |  |  |  |  | | --- | --- | --- | --- | --- | |  |  |  |  |  | | RAFL02-02-F05 ,At5g58420  40S ribosomal protein S4 (RPS4D) ribosomal protein S4, Arabidopsis thaliana, PIR:T48480 | | | | | | |
|  |  | RAFL07-13-J18 | At2g37270 / 40S ribosomal protein S5 (RPS5A) | |  |  |  |  |  | | --- | --- | --- | --- | --- | |  |  |  |  |  | | At2g37270 ,RAFL07-13-J18  40S ribosomal protein S5 (RPS5A) identical to GP:3043428 | | | | | | |
|  |  | RAFL06-14-I03 | At3g60770 / 40S ribosomal protein S13 (RPS13A) | |  |  |  |  |  | | --- | --- | --- | --- | --- | |  |  |  |  |  | | RAFL06-14-I03 ,At3g60770  40S ribosomal protein S13 (RPS13A) AtRPS13A mRNA for cytoplasmic ribosomal protein S13, Arabidopsis thaliana,AB031739 | | | | | | |
|  |  | RAFL05-08-H10 | At4g16720 / 60S ribosomal protein L15 (RPL15A) | |  |  |  |  |  | | --- | --- | --- | --- | --- | |  |  |  |  |  | | RAFL05-08-H10 ,At4g16720  60S ribosomal protein L15 (RPL15A) | | | | | | |
|  |  | RAFL05-17-F03 | At2g32060 / 40S ribosomal protein S12 (RPS12C) | |  |  |  |  |  | | --- | --- | --- | --- | --- | |  |  |  |  |  | | RAFL05-17-F03 ,At2g32060  40S ribosomal protein S12 (RPS12C) | | | | | | |
|  |  | RAFL06-09-H09 | At3g48930 / 40S ribosomal protein S11 (RPS11A) | |  |  |  |  |  | | --- | --- | --- | --- | --- | |  |  |  |  |  | | At3g48930 ,RAFL06-09-H09  40S ribosomal protein S11 (RPS11A) | | | | | | |
|  |  | RAFL06-15-A09 | At1g59359 / 40S ribosomal protein S2 (RPS2B) | |  |  |  |  |  | | --- | --- | --- | --- | --- | |  |  |  |  |  | | RAFL06-15-A09 ,At1g59359  40S ribosomal protein S2 (RPS2B) similar to ribosomal protein S2 GI:430711 from [Drosophila melanogaster] | | | | | | |
|  |  | RAFL06-08-I04 | At1g07920 / elongation factor 1-alpha (EF-1-alpha) | |  |  |  |  |  | | --- | --- | --- | --- | --- | |  |  |  |  |  | | RAFL06-08-I04 ,At1g07920  elongation factor 1-alpha / EF-1-alpha identical to GB:CAA34456 from [Arabidopsis thaliana] (Plant Mol. Biol. 14 (1), 107-110 (1990)) | | | | | | |
|  |  | RAFL05-07-H16 | At3g53870 / 40S ribosomal protein S3 (RPS3B) | |  |  |  |  |  | | --- | --- | --- | --- | --- | |  |  |  |  |  | | At3g53870 ,RAFL05-07-H16  40S ribosomal protein S3 (RPS3B) ribosomal protein S3a - Xenopus laevis, PIR:R3XL3A | | | | | | |
|  |  | RAFL05-02-G09 | At5g41520 / expressed protein | |  |  |  |  |  | | --- | --- | --- | --- | --- | |  |  |  |  |  | | RAFL05-02-G09 ,At5g41520  40S ribosomal protein S10 (RPS10B) contains similarity to 40S ribosomal protein S10 | | | | | | |
|  |  | RAFL07-15-M07 | At1g04480 / 60S ribosomal protein L23 (RPL23A) | |  |  |  |  |  | | --- | --- | --- | --- | --- | |  |  |  |  |  | | At1g04480 ,RAFL07-15-M07  60S ribosomal protein L23 (RPL23A) identical to GB:AAB80655 | | | | | | |
|  |  | RAFL05-14-D21 | At1g18540 / 60S ribosomal protein L6 (RPL6A) | |  |  |  |  |  | | --- | --- | --- | --- | --- | |  |  |  |  |  | | At1g18540 ,RAFL05-14-D21  60S ribosomal protein L6 (RPL6A) similar to 60S ribosomal protein L6 GI:7208784 from [Cicer arietinum] | | | | | | |
|  |  | RAFL08-11-K22 | At1g57660 / 60S ribosomal protein L21 (RPL21E) | |  |  |  |  |  | | --- | --- | --- | --- | --- | |  |  |  |  |  | | At1g57660 ,RAFL08-11-K22  60S ribosomal protein L21 (RPL21E) similar to 60S ribosomal protein L21 GB:Q43291 GI:2851508 from [Arabidopsis thaliana] | | | | | | |
|  |  | RAFL11-12-H04 | At3g25520 / 60S ribosomal protein L5 (RPL5A) | |  |  |  |  |  | | --- | --- | --- | --- | --- | |  |  |  |  |  | | At3g25520 ,RAFL11-12-H04  60S ribosomal protein L5 similar to 60S ribosomal protein L5 GB:P49625 from [Oryza sativa] | | | | | | |
|  |  | RAFL04-18-N22 | At2g44120 / 60S ribosomal protein L7 (RPL7C) | |  |  |  |  |  | | --- | --- | --- | --- | --- | |  |  |  |  |  | | RAFL04-18-N22 ,At2g44120  60S ribosomal protein L7 (RPL7C) | | | | | | |
|  |  | RAFL09-07-D04 | At3g22230 / 60S ribosomal protein L27 (RPL27B) | |  |  |  |  |  | | --- | --- | --- | --- | --- | |  |  |  |  |  | | At3g22230 ,RAFL09-07-D04  60S ribosomal protein L27 (RPL27B) similar to 60S RIBOSOMAL PROTEIN L27 GB:P41101 from [Solanum tuberosum] | | | | | | |
|  |  | RAFL04-10-F11 | At5g59850 / 40S ribosomal protein S15A (RPS15aF) | |  |  |  |  |  | | --- | --- | --- | --- | --- | |  |  |  |  |  | | At5g59850 ,RAFL04-10-F11  40S ribosomal protein S15A (RPS15aF) cytoplasmic ribosomal protein S15a, Arabidopsis thaliana, EMBL:ATAF1412 | | | | | | |
|  |  | RAFL04-18-N10 | At1g07770 / 40S ribosomal protein S15A (RPS15aA) | |  |  |  |  |  | | --- | --- | --- | --- | --- | |  |  |  |  |  | | RAFL04-18-N10 ,At1g07770  40S ribosomal protein S15A (RPS15aA) identical to GB:AAA61608 from [Arabidopsis thaliana] (Plant Physiol. 106 (1), 401-402 (1994)) | | | | | | |
|  |  | RAFL05-18-M20 | At2g19730 / 60S ribosomal protein L28 (RPL28A) | |  |  |  |  |  | | --- | --- | --- | --- | --- | |  |  |  |  |  | | At2g19730 ,RAFL05-18-M20  60S ribosomal protein L28 (RPL28A) | | | | | | |
|  |  | RAFL07-08-E24 | At5g27850 / 60S ribosomal protein L18 (RPL18C) | |  |  |  |  |  | | --- | --- | --- | --- | --- | |  |  |  |  |  | | RAFL07-08-E24 ,At5g27850  60S ribosomal protein L18 (RPL18C) 60S ribosomal protein L18, Arabidopsis thaliana, SWISSPROT:RL18\_ARATH | | | | | | |
|  |  | RAFL11-07-B21 | At3g25520 / 60S ribosomal protein L5 (RPL5A) | |  |  |  |  |  | | --- | --- | --- | --- | --- | |  |  |  |  |  | | At3g25520 ,RAFL11-07-B21  60S ribosomal protein L5 similar to 60S ribosomal protein L5 GB:P49625 from [Oryza sativa] | | | | | | |
|  |  | RAFL04-20-F03 | At5g39740 / 60S ribosomal protein L5 (RPL5B) | |  |  |  |  |  | | --- | --- | --- | --- | --- | |  |  |  |  |  | | RAFL04-20-F03 ,At5g39740  60S ribosomal protein L5 (RPL5B) ribosomal protein L5, rice | | | | | | |
|  |  | RAFL11-05-B21 | At3g54210 / ribosomal protein L17 -related protein | |  |  |  |  |  | | --- | --- | --- | --- | --- | |  |  |  |  |  | | At3g54210 ,RAFL11-05-B21  ribosomal protein L17 family protein contains Pfam profile: PF01196 ribosomal protein L17 | | | | | | |
|  |  | RAFL05-02-K09 | At5g22440 / 60S ribosomal protein L10A (RPL10aC) | |  |  |  |  |  | | --- | --- | --- | --- | --- | |  |  |  |  |  | | RAFL05-02-K09 ,At5g22440  60S ribosomal protein L10A (RPL10aC) | | | | | | |
|  |  | RAFL02-03-G08 | At4g18730 / 60S ribosomal protein L11 (RPL11C) | |  |  |  |  |  | | --- | --- | --- | --- | --- | |  |  |  |  |  | | RAFL02-03-G08 ,At4g18730  60S ribosomal protein L11 (RPL11C) | | | | | | |
|  |  | RAFL05-05-M24 | At5g02960 / 40S ribosomal protein S23 (RPS23B) | |  |  |  |  |  | | --- | --- | --- | --- | --- | |  |  |  |  |  | | At5g02960 ,RAFL05-05-M24  40S ribosomal protein S23 (RPS23B) ribosomal protein S23, Fragaria x ananassa, PIR:S56673 | | | | | | |
|  |  | RAFL07-13-J05 | At1g07920 / elongation factor 1-alpha (EF-1-alpha) | |  |  |  |  |  | | --- | --- | --- | --- | --- | |  |  |  |  |  | | RAFL07-13-J05 ,At1g07920  elongation factor 1-alpha / EF-1-alpha identical to GB:CAA34456 from [Arabidopsis thaliana] (Plant Mol. Biol. 14 (1), 107-110 (1990)) | | | | | | |
|  |  | RAFL05-18-P15 | At1g04480 / 60S ribosomal protein L23 (RPL23A) | |  |  |  |  |  | | --- | --- | --- | --- | --- | |  |  |  |  |  | | At1g04480 ,RAFL05-18-P15  60S ribosomal protein L23 (RPL23A) identical to GB:AAB80655 | | | | | | |
|  |  | RAFL04-09-J06 | At1g70600 / 60S ribosomal protein L27A (RPL27aC) | |  |  |  |  |  | | --- | --- | --- | --- | --- | |  |  |  |  |  | | At1g70600 ,RAFL04-09-J06  60S ribosomal protein L27A (RPL27aC) identical to 60S ribosomal protein L27A GB:P49637 [Arabidopsis thaliana] | | | | | | |
|  |  | RAFL09-15-M15 | At5g23740 / 40S ribosomal protein S11 (RPS11C) | |  |  |  |  |  | | --- | --- | --- | --- | --- | |  |  |  |  |  | | RAFL09-15-M15 ,At5g23740  40S ribosomal protein S11 (RPS11C) | | | | | | |
|  |  | RAFL05-12-E15 | At2g31610 / 40S ribosomal protein S3 (RPS3A) | |  |  |  |  |  | | --- | --- | --- | --- | --- | |  |  |  |  |  | | RAFL05-12-E15 ,At2g31610  40S ribosomal protein S3 (RPS3A) | | | | | | |
|  |  | RAFL04-14-L08 | At3g49010 / 60S ribosomal protein L13 (RPL13B)/breast basic conserved protein 1-related (BBC1) | |  |  |  |  |  | | --- | --- | --- | --- | --- | |  |  |  |  |  | | At3g49010 ,RAFL04-14-L08  60S ribosomal protein L13 (RPL13B) / breast basic conserved protein 1-related (BBC1) | | | | | | |
|  |  | RAFL06-10-J18 | At4g14320 / 60S ribosomal protein L36a/L44 (RPL36aB) | |  |  |  |  |  | | --- | --- | --- | --- | --- | |  |  |  |  |  | | RAFL06-10-J18 ,At4g14320  60S ribosomal protein L36a/L44 (RPL36aB) | | | | | | |
|  |  | RAFL11-09-J03 | At3g54210 / ribosomal protein L17 -related protein | |  |  |  |  |  | | --- | --- | --- | --- | --- | |  |  |  |  |  | | RAFL11-09-J03 ,At3g54210  ribosomal protein L17 family protein contains Pfam profile: PF01196 ribosomal protein L17 | | | | | | |
|  |  | RAFL02-10-A09 | At1g33140 / 60S ribosomal protein L9 (RPL90A/C) | |  |  |  |  |  | | --- | --- | --- | --- | --- | |  |  |  |  |  | | RAFL02-10-A09 ,At1g33140  60S ribosomal protein L9 (RPL90A/C) similar to RIBOSOMAL PROTEIN L9 GB:P49209 from [Arabidopsis thaliana] | | | | | | |
|  |  | RAFL07-15-K08 | At4g34670 / 40S ribosomal protein S3A (RPS3aB) | |  |  |  |  |  | | --- | --- | --- | --- | --- | |  |  |  |  |  | | RAFL07-15-K08 ,At4g34670  40S ribosomal protein S3A (RPS3aB) | | | | | | |
|  |  | RAFL07-14-M14 | At5g02870 / 60S ribosomal protein L4/L1 (RPL4D) | |  |  |  |  |  | | --- | --- | --- | --- | --- | |  |  |  |  |  | | At5g02870 ,RAFL07-14-M14  60S ribosomal protein L4/L1 (RPL4D) 60S roibosomal protein L4, Arabidopsis thaliana, EMBL:CAA79104 | | | | | | |
|  |  | RAFL09-06-A22 | At3g62870 / 60S ribosomal protein L7A (RPL7aB) | |  |  |  |  |  | | --- | --- | --- | --- | --- | |  |  |  |  |  | | RAFL09-06-A22 ,At3g62870  60S ribosomal protein L7A (RPL7aB) 60S RIBOSOMAL PROTEIN L7A - Oryza sativa, SWISSPROT:RL7A\_ORYSA | | | | | | |
|  |  | RAFL04-09-M12 | At4g27090 / 60S ribosomal protein L14 (RPL14B) | |  |  |  |  |  | | --- | --- | --- | --- | --- | |  |  |  |  |  | | At4g27090 ,RAFL04-09-M12  60S ribosomal protein L14 (RPL14B) ribosomal protein L14 - Human,PIR3:JC5954 | | | | | | |
|  |  | RAFL05-01-G23 | At3g49600 / ubiquitin-specific protease 26 (UBP26) | |  |  |  |  |  | | --- | --- | --- | --- | --- | |  |  |  |  |  | | At3g49600 ,RAFL05-01-G23  ubiquitin-specific protease 26 (UBP26) similar to GI:11993492; RNA binding protein - Homo sapiens, EMBL:AB016089 (N-terminus), several ubiquitin carboxyl-terminal hydrolases from aa pos. 712 | | | | | | |
|  |  | RAFL11-10-L18 | At2g36620 / 60S ribosomal protein L24 (RPL24A) | |  |  |  |  |  | | --- | --- | --- | --- | --- | |  |  |  |  |  | | RAFL11-10-L18 ,At2g36620  60S ribosomal protein L24 (RPL24A) | | | | | | |
|  |  | RAFL02-02-B08 | At3g02540 / RAD23 -related | |  |  |  |  |  | | --- | --- | --- | --- | --- | |  |  |  |  |  | | At3g02540 ,RAFL02-02-B08  ubiquitin family protein contains Pfam profiles PF00240: Ubiquitin family, PF00627: UBA/TS-N domain; | | | | | | |
|  |  | RAFL06-10-E08 | At2g34480 / 60S ribosomal protein L18A (RPL18aB) | |  |  |  |  |  | | --- | --- | --- | --- | --- | |  |  |  |  |  | | At2g34480 ,RAFL06-10-E08  60S ribosomal protein L18A (RPL18aB) | | | | | | |
|  |  | RAFL07-08-L21 | At5g59850 / 40S ribosomal protein S15A (RPS15aF) | |  |  |  |  |  | | --- | --- | --- | --- | --- | |  |  |  |  |  | | At5g59850 ,RAFL07-08-L21  40S ribosomal protein S15A (RPS15aF) cytoplasmic ribosomal protein S15a, Arabidopsis thaliana, EMBL:ATAF1412 | | | | | | |
|  |  | RAFL05-03-L01 | At3g56340 / 40S ribosomal protein S26 homolog | |  |  |  |  |  | | --- | --- | --- | --- | --- | |  |  |  |  |  | | At3g56340 ,RAFL05-03-L01  40S ribosomal protein S26 (RPS26C) several 40S ribosomal protein S26 | | | | | | |
|  |  | RAFL06-08-B09 | At3g11510 / 40S ribosomal protein S14 (RPS14B) | |  |  |  |  |  | | --- | --- | --- | --- | --- | |  |  |  |  |  | | At3g11510 ,RAFL06-08-B09  40S ribosomal protein S14 (RPS14B) similar to 40S ribosomal protein S14 GB:P19950 [Zea mays] | | | | | | |
|  |  | RAFL06-08-P20 | At3g07110 / 60S ribosomal protein L13A (RPL13aA) | |  |  |  |  |  | | --- | --- | --- | --- | --- | |  |  |  |  |  | | RAFL06-08-P20 ,At3g07110  60S ribosomal protein L13A (RPL13aA) similar to ribosomal protein L13A GB:O49885 [Lupinus luteus] | | | | | | |
|  |  | RAFL05-16-H14 | At1g04270 / 40S ribosomal protein S15 (RPS15A) | |  |  |  |  |  | | --- | --- | --- | --- | --- | |  |  |  |  |  | | At1g04270 ,RAFL05-16-H14  40S ribosomal protein S15 (RPS15A) Strong similarity to Oryza 40S ribosomal protein S15. ESTs gb|R29788,gb|ATTS0365 come from this gene | | | | | | |
|  |  | RAFL04-10-H14 | At3g49910 / 60S ribosomal protein L26 (RPL26A) | |  |  |  |  |  | | --- | --- | --- | --- | --- | |  |  |  |  |  | | At3g49910 ,RAFL04-10-H14  60S ribosomal protein L26 (RPL26A) 60S RIBOSOMAL PROTEIN L26, Brassica rapa, EMBL:BRD495 | | | | | | |
|  |  | RAFL05-13-M17 | At3g53890 / 40S ribosomal protein S21 homolog | |  |  |  |  |  | | --- | --- | --- | --- | --- | |  |  |  |  |  | | RAFL05-13-M17 ,At3g53890  40S ribosomal protein S21 (RPS21B) ribosomal protein S21, cytosolic - Oryza sativa, PIR:S38357 | | | | | | |
|  |  | RAFL06-11-K09 | At5g03850 / 40S ribosomal protein S28 (RPS28B) | |  |  |  |  |  | | --- | --- | --- | --- | --- | |  |  |  |  |  | | RAFL06-11-K09 ,At5g03850  40S ribosomal protein S28 (RPS28B) ribosomal protein S28, Arabidopsis thaliana, EMBL:ATRP28A | | | | | | |
|  |  | RAFL05-16-K23 | At1g02780 / 60S ribosomal protein L19 (RPL19A) | |  |  |  |  |  | | --- | --- | --- | --- | --- | |  |  |  |  |  | | RAFL05-16-K23 ,At1g02780  60S ribosomal protein L19 (RPL19A) similar to ribosomal protein L19 GI:36127 from [Homo sapiens] | | | | | | |
|  |  | RAFL05-19-H05 | At3g28900 / 60S ribosomal protein L34 (RPL34C) | |  |  |  |  |  | | --- | --- | --- | --- | --- | |  |  |  |  |  | | RAFL05-19-H05 ,At3g28900  60S ribosomal protein L34 (RPL34C) similar to 60S ribosomal protein L34 GB:P41098 [Nicotiana tabacum] | | | | | | |
|  |  | RAFL04-14-J20 | At2g40660 / methionyl-tRNA synthetase -related | |  |  |  |  |  | | --- | --- | --- | --- | --- | |  |  |  |  |  | | RAFL04-14-J20 ,At2g40660  tRNA-binding region domain-containing protein similar to SP|Q12904 Multisynthetase complex auxiliary component p43 [Contains: Endothelial-monocyte activating polypeptide II (EMAP-II) (Small inducible cytokine subfamily E member 1)] {Homo sapiens}; contains Pfam profile PF01588: Putative tRNA binding domain | | | | | | |
|  |  | RAFL06-08-F22 | At5g13650 / GTP-binding protein typA (tyrosine phosphorylated protein A) | |  |  |  |  |  | | --- | --- | --- | --- | --- | |  |  |  |  |  | | At5g13650 ,RAFL06-08-F22  elongation factor family protein contains Pfam profiles: PF00009 elongation factor Tu GTP binding domain,PF00679 elongation factor G C-terminus, PF03144 elongation factor Tu domain 2 | | | | | | |
|  |  | RAFL04-18-P03 | At4g17390 / 60S ribosomal protein L15 (RPL15B) | |  |  |  |  |  | | --- | --- | --- | --- | --- | |  |  |  |  |  | | At4g17390 ,RAFL04-18-P03  60S ribosomal protein L15 (RPL15B) | | | | | | |
|  |  | RAFL04-19-M20 | At1g58380 / 40S ribosomal protein S2 (RPS2A) | |  |  |  |  |  | | --- | --- | --- | --- | --- | |  |  |  |  |  | | RAFL04-19-M20 ,At1g58380  40S ribosomal protein S2 (RPS2A) similar to ribosomal protein S2 GI:939717 from (Urechis caupo) | | | | | | |
|  |  | RAFL05-17-P11 | At3g04920 / 40S ribosomal protein S24 (RPS24A) | |  |  |  |  |  | | --- | --- | --- | --- | --- | |  |  |  |  |  | | At3g04920 ,RAFL05-17-P11  40S ribosomal protein S24 (RPS24A) similar to ribosomal protein S19 GB:445612 [Solanum tuberosum] and similar to ribosomal protein S24 GB:4506703 [Homo sapiens] | | | | | | |
|  |  | RAFL05-13-D18 | At3g04400 / 60S ribosomal protein L23 (RPL23C) | |  |  |  |  |  | | --- | --- | --- | --- | --- | |  |  |  |  |  | | At3g04400 ,RAFL05-13-D18  60S ribosomal protein L23 (RPL23C) similar to ribosomal protein L17 GB:AAA34113.1 from [Nicotiana tabacum] | | | | | | |
|  |  | RAFL06-08-P08 | At3g23390 / 60S ribosomal protein L36a/L44 (RPL36aA) | |  |  |  |  |  | | --- | --- | --- | --- | --- | |  |  |  |  |  | | RAFL06-08-P08 ,At3g23390  60S ribosomal protein L36a/L44 (RPL36aA) similar to ribosomal protein L41 GB:AAA34366 from [Candida maltosa] | | | | | | |
|  |  | RAFL05-14-A02 | At5g52650 / 40S ribosomal protein S10 (RPS10C) | |  |  |  |  |  | | --- | --- | --- | --- | --- | |  |  |  |  |  | | At5g52650 ,RAFL05-14-A02  40S ribosomal protein S10 (RPS10C) contains similarity to 40S ribosomal protein S10 | | | | | | |
|  |  | RAFL11-06-J19 | At2g01250 / 60S ribosomal protein L7 (RPL7B) | |  |  |  |  |  | | --- | --- | --- | --- | --- | |  |  |  |  |  | | At2g01250 ,RAFL11-06-J19  60S ribosomal protein L7 (RPL7B) | | | | | | |
|  |  | RAFL11-09-C11 | At2g01250 / 60S ribosomal protein L7 (RPL7B) | |  |  |  |  |  | | --- | --- | --- | --- | --- | |  |  |  |  |  | | At2g01250 ,RAFL11-09-C11  60S ribosomal protein L7 (RPL7B) | | | | | | |
|  | Cluster:3-0 | |  |  | 49 | 184 | 372 | 4058 | 1.068473E-8 | 2.9917246E-7 | 28 |
|  |  | RAFL07-15-M24 | At1g74050 / 60S ribosomal protein L6 (RPL6C) | |  |  |  |  |  | | --- | --- | --- | --- | --- | |  |  |  |  |  | | At1g74050 ,RAFL07-15-M24  60S ribosomal protein L6 (RPL6C) similar to 60S ribosomal protein L6 (YL 16 like) GB:CAB57309 from [Cyanophora paradoxa] | | | | | | |
|  |  | RAFL04-15-D01 | At3g09200 / 60S acidic ribosomal protein P0 (RPP0B) | |  |  |  |  |  | | --- | --- | --- | --- | --- | |  |  |  |  |  | | At3g09200 ,RAFL04-15-D01  60S acidic ribosomal protein P0 (RPP0B) similar to putative 60S acidic ribosomal protein P0 GB:P50346 [Glycine max] | | | | | | |
|  |  | RAFL08-11-M03 | At5g60390 / elongation factor 1-alpha (EF-1-alpha) | |  |  |  |  |  | | --- | --- | --- | --- | --- | |  |  |  |  |  | | RAFL08-11-M03 ,At5g60390  elongation factor 1-alpha / EF-1-alpha identical to SWISS-PROT:P13905 elongation factor 1-alpha (EF-1-alpha) [Arabidopsis thaliana] | | | | | | |
|  |  | RAFL09-12-B12 | At4g36130 / 60S ribosomal protein L8 (RPL8C) | |  |  |  |  |  | | --- | --- | --- | --- | --- | |  |  |  |  |  | | At4g36130 ,RAFL09-12-B12  60S ribosomal protein L8 (RPL8C) ribosomal protein L8, cytosolic, tomato, PIR1:R5TOL8 | | | | | | |
|  |  | RAFL11-07-A12 | At5g62300 / 40S ribosomal protein S20 (RPS20C) | |  |  |  |  |  | | --- | --- | --- | --- | --- | |  |  |  |  |  | | RAFL11-07-A12 ,At5g62300  40S ribosomal protein S20 (RPS20C) ribosomal protein S20, Arabidopsis thaliana, PIR:T12992 | | | | | | |
|  |  | RAFL04-15-O08 | At5g46430 / 60S ribosomal protein L32 (RPL32B) | |  |  |  |  |  | | --- | --- | --- | --- | --- | |  |  |  |  |  | | RAFL04-15-O08 ,At5g46430  60S ribosomal protein L32 (RPL32B) | | | | | | |
|  |  | RAFL08-10-G08 | At3g09630 / 60S ribosomal protein L4/L1 (RPL4A) | |  |  |  |  |  | | --- | --- | --- | --- | --- | |  |  |  |  |  | | RAFL08-10-G08 ,At3g09630  60S ribosomal protein L4/L1 (RPL4A) strong similarity to 60S ribosomal protein L1 GB:P49691 | | | | | | |
|  |  | RAFL04-16-M10 | At1g64550 / ABC transporter family protein | |  |  |  |  |  | | --- | --- | --- | --- | --- | |  |  |  |  |  | | RAFL04-16-M10 ,At1g64550  ABC transporter family protein similar to ABC transporter protein GB:AAF31030 GI:6899653 from [Leishmania major] | | | | | | |
|  |  | RAFL09-10-P09 | At4g36130 / 60S ribosomal protein L8 (RPL8C) | |  |  |  |  |  | | --- | --- | --- | --- | --- | |  |  |  |  |  | | At4g36130 ,RAFL09-10-P09  60S ribosomal protein L8 (RPL8C) ribosomal protein L8, cytosolic, tomato, PIR1:R5TOL8 | | | | | | |
|  |  | RAFL02-10-H10 | At3g43980 / 40S ribosomal protein S29 (RPS29A) | |  |  |  |  |  | | --- | --- | --- | --- | --- | |  |  |  |  |  | | At3g43980 ,RAFL02-10-H10  40S ribosomal protein S29 (RPS29A) ribosomal protein S29, rat, PIR:S30298 | | | | | | |
|  |  | RAFL05-07-F05 | At2g17360 / 40S ribosomal protein S4 (RPS4A) | |  |  |  |  |  | | --- | --- | --- | --- | --- | |  |  |  |  |  | | At2g17360 ,RAFL05-07-F05  40S ribosomal protein S4 (RPS4A) contains ribosomal protein S4 signature from residues 8 to 22 | | | | | | |
|  |  | RAFL05-19-G10 | At3g24830 / 60S ribosomal protein L13A (RPL13aB) | |  |  |  |  |  | | --- | --- | --- | --- | --- | |  |  |  |  |  | | RAFL05-19-G10 ,At3g24830  60S ribosomal protein L13A (RPL13aB) similar to 60S RIBOSOMAL PROTEIN L13A GB:P35427 from [Rattus norvegicus] | | | | | | |
|  |  | RAFL06-07-B02 | At3g11940 / 40S ribosomal protein S5 (RPS5B) | |  |  |  |  |  | | --- | --- | --- | --- | --- | |  |  |  |  |  | | At3g11940 ,RAFL06-07-B02  40S ribosomal protein S5 (RPS5B) similar to 40S ribosomal protein S5 GB:AAC98068 GI:4056502 from [Arabidopsis thaliana] | | | | | | |
|  |  | RAFL11-11-E06 | At1g72370 / 40S ribosomal protein SA (RPSaA) | |  |  |  |  |  | | --- | --- | --- | --- | --- | |  |  |  |  |  | | RAFL11-11-E06 ,At1g72370  40S ribosomal protein SA (RPSaA) identical to laminin receptor-like protein GB:U01955 [Arabidopsis thaliana]; identical to cDNA laminin receptor homologue GI:16379 | | | | | | |
|  |  | RAFL07-16-H23 | At3g16780 / 60S ribosomal protein L19 (RPL19B) | |  |  |  |  |  | | --- | --- | --- | --- | --- | |  |  |  |  |  | | At3g16780 ,RAFL07-16-H23  60S ribosomal protein L19 (RPL19B) similar to ribosomal protein L19 GB:CAA45090 from [Homo sapiens] | | | | | | |
|  |  | RAFL05-07-A02 | At5g47930 / ribosomal protein S27 | |  |  |  |  |  | | --- | --- | --- | --- | --- | |  |  |  |  |  | | RAFL05-07-A02 ,At5g47930  40S ribosomal protein S27 (RPS27D) | | | | | | |
|  |  | RAFL08-15-A10 | At5g09510 / 40S ribosomal protein S15 (RPS15D) | |  |  |  |  |  | | --- | --- | --- | --- | --- | |  |  |  |  |  | | RAFL08-15-A10 ,At5g09510  40S ribosomal protein S15 (RPS15D) ribosomal protein S15 - Arabidopsis thaliana, EMBL:Z23161 | | | | | | |
|  |  | RAFL05-14-G01 | At4g02230 / 60S ribosomal protein L19 (RPL19C) | |  |  |  |  |  | | --- | --- | --- | --- | --- | |  |  |  |  |  | | RAFL05-14-G01 ,At4g02230  60S ribosomal protein L19 (RPL19C) similar to L19 from several species | | | | | | |
|  |  | RAFL07-17-D23 | At1g78870 / ubiquitin-conjugating enzyme, putative | |  |  |  |  |  | | --- | --- | --- | --- | --- | |  |  |  |  |  | | At1g78870 ,RAFL07-17-D23  ubiquitin-conjugating enzyme, putative nearly identical to ubiquitin-conjugating enzyme E2 [Catharanthus roseus] GI:5381319; contains Pfam profile PF00179: Ubiquitin-conjugating enzyme | | | | | | |
|  |  | RAFL03-05-E07 | At1g07920 / elongation factor 1-alpha (EF-1-alpha) | |  |  |  |  |  | | --- | --- | --- | --- | --- | |  |  |  |  |  | | RAFL03-05-E07 ,At1g07920  elongation factor 1-alpha / EF-1-alpha identical to GB:CAA34456 from [Arabidopsis thaliana] (Plant Mol. Biol. 14 (1), 107-110 (1990)) | | | | | | |
|  |  | RAFL06-12-J07 | At3g53020 / 60S ribosomal protein L24 (RPL24B) | |  |  |  |  |  | | --- | --- | --- | --- | --- | |  |  |  |  |  | | At3g53020 ,RAFL06-12-J07  60S ribosomal protein L24 (RPL24B) 60S ribosomal protein L24, Arabidopsis thaliana, EMBL:AC006282 | | | | | | |
|  |  | RAFL06-16-N16 | At4g16720 / 60S ribosomal protein L15 (RPL15A) | |  |  |  |  |  | | --- | --- | --- | --- | --- | |  |  |  |  |  | | RAFL06-16-N16 ,At4g16720  60S ribosomal protein L15 (RPL15A) | | | | | | |
|  |  | RAFL04-17-H14 | At3g06700 / 60S ribosomal protein L29 (RPL29A) | |  |  |  |  |  | | --- | --- | --- | --- | --- | |  |  |  |  |  | | RAFL04-17-H14 ,At3g06700  60S ribosomal protein L29 (RPL29A) similar to ribosomal protein L29 GI:7959366 [Panax ginseng] | | | | | | |
|  |  | RAFL04-09-E11 | At5g08180 / ribosomal protein L7Ae family | |  |  |  |  |  | | --- | --- | --- | --- | --- | |  |  |  |  |  | | At5g08180 ,RAFL04-09-E11  ribosomal protein L7Ae/L30e/S12e/Gadd45 family protein Similar to NHP2/L7Ae family proteins, see SWISSPROT:P32495 and PMID:2063628. | | | | | | |
|  |  | RAFL05-09-M22 | At3g49910 / 60S ribosomal protein L26 (RPL26A) | |  |  |  |  |  | | --- | --- | --- | --- | --- | |  |  |  |  |  | | RAFL05-09-M22 ,At3g49910  60S ribosomal protein L26 (RPL26A) 60S RIBOSOMAL PROTEIN L26, Brassica rapa, EMBL:BRD495 | | | | | | |
|  |  | RAFL04-15-O15 | At1g30230 / elongation factor 1-beta -related | |  |  |  |  |  | | --- | --- | --- | --- | --- | |  |  |  |  |  | | RAFL04-15-O15 ,At1g30230  elongation factor 1-beta / EF-1-beta identical to SP|P48006 Elongation factor 1-beta (EF-1-beta) {Arabidopsis thaliana} | | | | | | |
|  |  | RAFL05-13-K07 | At4g09800 / 40S ribosomal protein S18 (RPS18C) | |  |  |  |  |  | | --- | --- | --- | --- | --- | |  |  |  |  |  | | RAFL05-13-K07 ,At4g09800  40S ribosomal protein S18 (RPS18C) | | | | | | |
|  |  | RAFL04-14-I15 | At3g02080 / 40S ribosomal protein S19 (RPS19A) | |  |  |  |  |  | | --- | --- | --- | --- | --- | |  |  |  |  |  | | RAFL04-14-I15 ,At3g02080  40S ribosomal protein S19 (RPS19A) similar to 40S ribosomal protein S19 GB:P40978 [Oryza sativa] | | | | | | |
|  |  | RAFL11-01-C20 | At3g06590 / bHLH protein | |  |  |  |  |  | | --- | --- | --- | --- | --- | |  |  |  |  |  | | RAFL11-01-C20 ,At3g06590  expressed protein | | | | | | |
|  |  | RAFL07-16-A17 | At5g64140 / 40S ribosomal protein S28 (RPS28C) | |  |  |  |  |  | | --- | --- | --- | --- | --- | |  |  |  |  |  | | At5g64140 ,RAFL07-16-A17  40S ribosomal protein S28 (RPS28C) | | | | | | |
|  |  | RAFL07-10-D02 | At1g14320 / 60S ribosomal protein L10 (RPL10A)/Wilm's tumor suppressor protein-related | |  |  |  |  |  | | --- | --- | --- | --- | --- | |  |  |  |  |  | | At1g14320 ,RAFL07-10-D02  60S ribosomal protein L10 (RPL10A) / Wilm's tumor suppressor protein-related similar to tumor suppressor GI:575354 from [Oryza sativa] | | | | | | |
|  |  | RAFL08-13-M06 | At3g11250 / 60S acidic ribosomal protein P0 (RPP0C) | |  |  |  |  |  | | --- | --- | --- | --- | --- | |  |  |  |  |  | | At3g11250 ,RAFL08-13-M06  60S acidic ribosomal protein P0 (RPP0C) similar to 60S acidic ribosomal protein P0 GI:2088654 [Arabidopsis thaliana] | | | | | | |
|  |  | RAFL06-08-M13 | At1g07920 / elongation factor 1-alpha (EF-1-alpha) | |  |  |  |  |  | | --- | --- | --- | --- | --- | |  |  |  |  |  | | RAFL06-08-M13 ,At1g07920  elongation factor 1-alpha / EF-1-alpha identical to GB:CAA34456 from [Arabidopsis thaliana] (Plant Mol. Biol. 14 (1), 107-110 (1990)) | | | | | | |
|  |  | RAFL07-10-F16 | At3g13930 / acetyltransferase -related | |  |  |  |  |  | | --- | --- | --- | --- | --- | |  |  |  |  |  | | At3g13930 ,RAFL07-10-F16  dihydrolipoamide S-acetyltransferase, putative similar to dihydrolipoamide S-acetyltransferase [Zea mays] GI:5669871; contains Pfam profiles PF00198: 2-oxo acid dehydrogenases acyltransferase (catalytic domain), PF00364: Biotin-requiring enzyme, PF02817: e3 binding domain | | | | | | |
|  |  | RAFL05-18-H11 | At5g27700 / ribosomal protein S21 - like | |  |  |  |  |  | | --- | --- | --- | --- | --- | |  |  |  |  |  | | RAFL05-18-H11 ,At5g27700  40S ribosomal protein S21 (RPS21C) ribosomal protein S21, Zea mays, PIR:T03945 | | | | | | |
|  |  | RAFL11-02-J15 | At4g00100 / 40S ribosomal protein S13 (RPS13B) | |  |  |  |  |  | | --- | --- | --- | --- | --- | |  |  |  |  |  | | At4g00100 ,RAFL11-02-J15  40S ribosomal protein S13 (RPS13A) similar to ribosomal protein S13; PF00312 (View Sanger Pfam): ribosomal protein S15; identical to cDNA AtRPS13A mRNA for cytoplasmic ribosomal protein S13 GI:6521011 | | | | | | |
|  |  | RAFL04-20-J09 | At4g25130 / protein-methionine-S-oxide reductase | |  |  |  |  |  | | --- | --- | --- | --- | --- | |  |  |  |  |  | | At4g25130 ,RAFL04-20-J09  peptide methionine sulfoxide reductase, putative strong similarity to SP|P54151 Peptide methionine sulfoxide reductase (EC 1.8.4.6) {Brassica napus}; contains Pfam profile PF01625: Peptide methionine sulfoxide reductase | | | | | | |
|  |  | RAFL09-18-O15 | At2g17360 / 40S ribosomal protein S4 (RPS4A) | |  |  |  |  |  | | --- | --- | --- | --- | --- | |  |  |  |  |  | | RAFL09-18-O15 ,At2g17360  40S ribosomal protein S4 (RPS4A) contains ribosomal protein S4 signature from residues 8 to 22 | | | | | | |
|  |  | RAFL07-08-M19 | At1g09620 / leucyl-tRNA synthetase -related | |  |  |  |  |  | | --- | --- | --- | --- | --- | |  |  |  |  |  | | At1g09620 ,RAFL07-08-M19  tRNA synthetase class I (I, L, M and V) family protein similar to cytosolic leucyl-tRNA synthetase [Candida albicans] GI:9858190; contains Pfam profile PF00133: tRNA synthetases class I (I, L, M and V) | | | | | | |
|  |  | RAFL05-07-M06 | At3g20290 / calcium-binding EF-hand family protein | |  |  |  |  |  | | --- | --- | --- | --- | --- | |  |  |  |  |  | | RAFL05-07-M06 ,At3g20290  calcium-binding EF hand family protein similar to EH-domain containing protein 1 from {Mus musculus} SP|Q9WVK4 and {Homo sapiens} SP|Q9H4M9, receptor-mediated endocytosis 1 from [Caenorhabditis elegans] GI:13487775, GI:13487777, GI:13487779; contains INTERPRO:IPR002048 calcium-binding EF-hand domain | | | | | | |
|  |  | RAFL11-04-A02 | At1g07770 / 40S ribosomal protein S15A (RPS15aA) | |  |  |  |  |  | | --- | --- | --- | --- | --- | |  |  |  |  |  | | At1g07770 ,RAFL11-04-A02  40S ribosomal protein S15A (RPS15aA) identical to GB:AAA61608 from [Arabidopsis thaliana] (Plant Physiol. 106 (1), 401-402 (1994)) | | | | | | |
|  |  | RAFL09-09-O07 | At4g16720 / 60S ribosomal protein L15 (RPL15A) | |  |  |  |  |  | | --- | --- | --- | --- | --- | |  |  |  |  |  | | RAFL09-09-O07 ,At4g16720  60S ribosomal protein L15 (RPL15A) | | | | | | |
|  |  | RAFL04-09-F23 | At2g31610 / 40S ribosomal protein S3 (RPS3A) | |  |  |  |  |  | | --- | --- | --- | --- | --- | |  |  |  |  |  | | RAFL04-09-F23 ,At2g31610  40S ribosomal protein S3 (RPS3A) | | | | | | |
|  |  | RAFL09-13-H18 | At1g43170 / 60S ribosomal protein L3 (RPL3A) | |  |  |  |  |  | | --- | --- | --- | --- | --- | |  |  |  |  |  | | At1g43170 ,RAFL09-13-H18  60S ribosomal protein L3 (RPL3A) identical to ribosomal protein GI:166858 from [Arabidopsis thaliana] | | | | | | |
|  |  | RAFL08-09-E20 | At2g41840 / 40S ribosomal protein S2 (RPS2C) | |  |  |  |  |  | | --- | --- | --- | --- | --- | |  |  |  |  |  | | RAFL08-09-E20 ,At2g41840  40S ribosomal protein S2 (RPS2C) | | | | | | |
|  |  | RAFL09-07-I03 | At5g60390 / elongation factor 1-alpha (EF-1-alpha) | |  |  |  |  |  | | --- | --- | --- | --- | --- | |  |  |  |  |  | | At5g60390 ,RAFL09-07-I03  elongation factor 1-alpha / EF-1-alpha identical to SWISS-PROT:P13905 elongation factor 1-alpha (EF-1-alpha) [Arabidopsis thaliana] | | | | | | |
|  |  | RAFL05-14-H24 | At2g21580 / 40S ribosomal protein S25 (RPS25B) | |  |  |  |  |  | | --- | --- | --- | --- | --- | |  |  |  |  |  | | At2g21580 ,RAFL05-14-H24  40S ribosomal protein S25 (RPS25B) | | | | | | |
|  |  | RAFL11-04-H04 | At2g34480 / 60S ribosomal protein L18A (RPL18aB) | |  |  |  |  |  | | --- | --- | --- | --- | --- | |  |  |  |  |  | | At2g34480 ,RAFL11-04-H04  60S ribosomal protein L18A (RPL18aB) | | | | | | |
|  |  | RAFL07-17-K15 | At2g31040 / expressed protein | |  |  |  |  |  | | --- | --- | --- | --- | --- | |  |  |  |  |  | | RAFL07-17-K15 ,At2g31040  ATP synthase protein I -related contains weaks similarity to Swiss-Prot:P08443 ATP synthase protein I [Synechococcus sp.] | | | | | | |
|  | Cluster:2-0 | |  |  | 36 | 114 | 385 | 4128 | 2.2942839E-8 | 6.4239947E-7 | 28 |
|  |  | RAFL06-08-N09 | At3g63490 / ribosomal protein L1p family | |  |  |  |  |  | | --- | --- | --- | --- | --- | |  |  |  |  |  | | RAFL06-08-N09 ,At3g63490  ribosomal protein L1 family protein ribosomal protein L1, S.oleracea, EMBL:SORPL1 | | | | | | |
|  |  | RAFL09-09-C01 | At2g27530 / 60S ribosomal protein L10A (RPL10aB) | |  |  |  |  |  | | --- | --- | --- | --- | --- | |  |  |  |  |  | | RAFL09-09-C01 ,At2g27530  60S ribosomal protein L10A (RPL10aB) | | | | | | |
|  |  | RAFL04-10-I16 | At1g52300 / 60S ribosomal protein L37 (RPL37B) | |  |  |  |  |  | | --- | --- | --- | --- | --- | |  |  |  |  |  | | RAFL04-10-I16 ,At1g52300  60S ribosomal protein L37 (RPL37B) similar to SP:Q43292 from [Arabidopsis thaliana] | | | | | | |
|  |  | RAFL05-02-L21 | At1g07830 / ribosomal protein L29p family | |  |  |  |  |  | | --- | --- | --- | --- | --- | |  |  |  |  |  | | At1g07830 ,RAFL05-02-L21  ribosomal protein L29 family protein similar to GB:CAA83057 from [Saccharomyces cerevisiae] | | | | | | |
|  |  | RAFL05-16-E22 | At5g60390 / elongation factor 1-alpha (EF-1-alpha) | |  |  |  |  |  | | --- | --- | --- | --- | --- | |  |  |  |  |  | | At5g60390 ,RAFL05-16-E22  elongation factor 1-alpha / EF-1-alpha identical to SWISS-PROT:P13905 elongation factor 1-alpha (EF-1-alpha) [Arabidopsis thaliana] | | | | | | |
|  |  | RAFL08-12-D04 | At5g20290 / 40S ribosomal protein S8 (RPS8A) | |  |  |  |  |  | | --- | --- | --- | --- | --- | |  |  |  |  |  | | At5g20290 ,RAFL08-12-D04  40S ribosomal protein S8 (RPS8A) ribosomal protein S8 - Zea mays, PIR:T04088 | | | | | | |
|  |  | RAFL09-15-C19 | At5g60390 / elongation factor 1-alpha (EF-1-alpha) | |  |  |  |  |  | | --- | --- | --- | --- | --- | |  |  |  |  |  | | At5g60390 ,RAFL09-15-C19  elongation factor 1-alpha / EF-1-alpha identical to SWISS-PROT:P13905 elongation factor 1-alpha (EF-1-alpha) [Arabidopsis thaliana] | | | | | | |
|  |  | RAFL03-06-H07 | At1g33140 / 60S ribosomal protein L9 (RPL90A/C) | |  |  |  |  |  | | --- | --- | --- | --- | --- | |  |  |  |  |  | | RAFL03-06-H07 ,At1g33140  60S ribosomal protein L9 (RPL90A/C) similar to RIBOSOMAL PROTEIN L9 GB:P49209 from [Arabidopsis thaliana] | | | | | | |
|  |  | RAFL07-17-I21 | At1g07930 / elongation factor 1-alpha (EF-1-alpha) | |  |  |  |  |  | | --- | --- | --- | --- | --- | |  |  |  |  |  | | RAFL07-17-I21 ,At1g07930  elongation factor 1-alpha / EF-1-alpha identical to GB:CAA34456 from [Arabidopsis thaliana] (Plant Mol. Biol. 14 (1), 107-110 (1990)) | | | | | | |
|  |  | RAFL05-15-M21 | At5g20160 / ribosomal protein L7Ae family | |  |  |  |  |  | | --- | --- | --- | --- | --- | |  |  |  |  |  | | At5g20160 ,RAFL05-15-M21  ribosomal protein L7Ae/L30e/S12e/Gadd45 family protein Similar to NHP2/L7Ae family proteins, see SWISSPROT:P32495 and PMID:2063628. | | | | | | |
|  |  | RAFL03-08-O03 | At1g07940 / elongation factor 1-alpha (EF-1-alpha) | |  |  |  |  |  | | --- | --- | --- | --- | --- | |  |  |  |  |  | | RAFL03-08-O03 ,At1g07940  elongation factor 1-alpha / EF-1-alpha identical to GB:CAA34456 from [Arabidopsis thaliana] (Plant Mol. Biol. 14 (1), 107-110 (1990)) | | | | | | |
|  |  | RAFL04-19-O24 | At5g02610 / 60S ribosomal protein L35 (RPL35D) | |  |  |  |  |  | | --- | --- | --- | --- | --- | |  |  |  |  |  | | RAFL04-19-O24 ,At5g02610  60S ribosomal protein L35 (RPL35D) ribosomal protein L35- cytosolic, Arabidopsis thaliana, PIR:T00549 | | | | | | |
|  |  | RAFL06-15-I18 | At2g40590 / 40S ribosomal protein S26 (RPS26B) | |  |  |  |  |  | | --- | --- | --- | --- | --- | |  |  |  |  |  | | RAFL06-15-I18 ,At2g40590  40S ribosomal protein S26 (RPS26B) | | | | | | |
|  |  | RAFL03-06-H09 | At3g60770 / 40S ribosomal protein S13 (RPS13A) | |  |  |  |  |  | | --- | --- | --- | --- | --- | |  |  |  |  |  | | RAFL03-06-H09 ,At3g60770  40S ribosomal protein S13 (RPS13A) AtRPS13A mRNA for cytoplasmic ribosomal protein S13, Arabidopsis thaliana,AB031739 | | | | | | |
|  |  | RAFL08-09-P06 | At5g22920 / PGPD14 protein | |  |  |  |  |  | | --- | --- | --- | --- | --- | |  |  |  |  |  | | RAFL08-09-P06 ,At5g22920  zinc finger (C3HC4-type RING finger) family protein contains Pfam profiles:PF05495 CHY zinc finger, PF00097 zinc finger, C3HC4 type (RING finger) | | | | | | |
|  |  | RAFL06-08-H02 | At3g04840 / 40S ribosomal protein S3A (RPS3aA) | |  |  |  |  |  | | --- | --- | --- | --- | --- | |  |  |  |  |  | | At3g04840 ,RAFL06-08-H02  40S ribosomal protein S3A (RPS3aA) similar to 40S ribosomal protein S3A (S phase specific protein GBIS289) GB:P49396 [Brassica rapa] | | | | | | |
|  |  | RAFL07-14-H10 | At5g02870 / 60S ribosomal protein L4/L1 (RPL4D) | |  |  |  |  |  | | --- | --- | --- | --- | --- | |  |  |  |  |  | | At5g02870 ,RAFL07-14-H10  60S ribosomal protein L4/L1 (RPL4D) 60S roibosomal protein L4, Arabidopsis thaliana, EMBL:CAA79104 | | | | | | |
|  |  | RAFL07-17-B02 | At1g59359 / 40S ribosomal protein S2 (RPS2B) | |  |  |  |  |  | | --- | --- | --- | --- | --- | |  |  |  |  |  | | RAFL07-17-B02 ,At1g59359  40S ribosomal protein S2 (RPS2B) similar to ribosomal protein S2 GI:430711 from [Drosophila melanogaster] | | | | | | |
|  |  | RAFL09-12-G10 | At4g12600 / ribosomal protein L7Ae family | |  |  |  |  |  | | --- | --- | --- | --- | --- | |  |  |  |  |  | | RAFL09-12-G10 ,At4g12600  ribosomal protein L7Ae/L30e/S12e/Gadd45 family protein Similar to NHP2/L7Ae family proteins, see SWISSPROT:P32495 and PMID:2063628. | | | | | | |
|  |  | RAFL07-08-G14 | At2g31610 / 40S ribosomal protein S3 (RPS3A) | |  |  |  |  |  | | --- | --- | --- | --- | --- | |  |  |  |  |  | | RAFL07-08-G14 ,At2g31610  40S ribosomal protein S3 (RPS3A) | | | | | | |
|  |  | RAFL07-18-A18 | At3g23700 / expressed protein | |  |  |  |  |  | | --- | --- | --- | --- | --- | |  |  |  |  |  | | At3g23700 ,RAFL07-18-A18  S1 RNA-binding domain-containing protein contains Pfam domain, PF00575: S1 RNA binding domain | | | | | | |
|  |  | RAFL09-13-F11 | At1g07930 / elongation factor 1-alpha (EF-1-alpha) | |  |  |  |  |  | | --- | --- | --- | --- | --- | |  |  |  |  |  | | RAFL09-13-F11 ,At1g07930  elongation factor 1-alpha / EF-1-alpha identical to GB:CAA34456 from [Arabidopsis thaliana] (Plant Mol. Biol. 14 (1), 107-110 (1990)) | | | | | | |
|  |  | RAFL08-09-H17 | At1g07930 / elongation factor 1-alpha (EF-1-alpha) | |  |  |  |  |  | | --- | --- | --- | --- | --- | |  |  |  |  |  | | RAFL08-09-H17 ,At1g07930  elongation factor 1-alpha / EF-1-alpha identical to GB:CAA34456 from [Arabidopsis thaliana] (Plant Mol. Biol. 14 (1), 107-110 (1990)) | | | | | | |
|  |  | RAFL07-09-P18 | At4g31700 / 40S ribosomal protein S6 (RPS6A) | |  |  |  |  |  | | --- | --- | --- | --- | --- | |  |  |  |  |  | | RAFL07-09-P18 ,At4g31700  40S ribosomal protein S6 (RPS6A) ribosomal protein S6, Arabidopsis thaliana, PID:g2662469 | | | | | | |
|  |  | RAFL11-12-P10 | At3g55750 / 60S ribosomal protein L35a (RPL35aD) | |  |  |  |  |  | | --- | --- | --- | --- | --- | |  |  |  |  |  | | RAFL11-12-P10 ,At3g55750  60S ribosomal protein L35a (RPL35aD) ribosomal protein L35a.e.c15, Saccharomyces cerevisiae, PIR:S44069 | | | | | | |
|  |  | RAFL08-18-F01 | At1g07930 / elongation factor 1-alpha (EF-1-alpha) | |  |  |  |  |  | | --- | --- | --- | --- | --- | |  |  |  |  |  | | RAFL08-18-F01 ,At1g07930  elongation factor 1-alpha / EF-1-alpha identical to GB:CAA34456 from [Arabidopsis thaliana] (Plant Mol. Biol. 14 (1), 107-110 (1990)) | | | | | | |
|  |  | RAFL11-12-M17 | At3g25520 / 60S ribosomal protein L5 (RPL5A) | |  |  |  |  |  | | --- | --- | --- | --- | --- | |  |  |  |  |  | | At3g25520 ,RAFL11-12-M17  60S ribosomal protein L5 similar to 60S ribosomal protein L5 GB:P49625 from [Oryza sativa] | | | | | | |
|  |  | RAFL09-17-M14 | At2g27530 / 60S ribosomal protein L10A (RPL10aB) | |  |  |  |  |  | | --- | --- | --- | --- | --- | |  |  |  |  |  | | RAFL09-17-M14 ,At2g27530  60S ribosomal protein L10A (RPL10aB) | | | | | | |
|  |  | RAFL09-09-P17 | At1g72370 / 40S ribosomal protein SA (RPSaA) | |  |  |  |  |  | | --- | --- | --- | --- | --- | |  |  |  |  |  | | RAFL09-09-P17 ,At1g72370  40S ribosomal protein SA (RPSaA) identical to laminin receptor-like protein GB:U01955 [Arabidopsis thaliana]; identical to cDNA laminin receptor homologue GI:16379 | | | | | | |
|  |  | RAFL05-01-L16 | At5g11750 / ribosomal protein L19, putative | |  |  |  |  |  | | --- | --- | --- | --- | --- | |  |  |  |  |  | | RAFL05-01-L16 ,At5g11750  ribosomal protein L19 family protein similar to plastid ribosomal protein L19 precursor [Spinacia oleracea] gi|7582403|gb|AAF64312 | | | | | | |
|  |  | RAFL11-13-A04 | At5g02870 / 60S ribosomal protein L4/L1 (RPL4D) | |  |  |  |  |  | | --- | --- | --- | --- | --- | |  |  |  |  |  | | At5g02870 ,RAFL11-13-A04  60S ribosomal protein L4/L1 (RPL4D) 60S roibosomal protein L4, Arabidopsis thaliana, EMBL:CAA79104 | | | | | | |
|  |  | RAFL06-10-L13 | At3g09630 / 60S ribosomal protein L4/L1 (RPL4A) | |  |  |  |  |  | | --- | --- | --- | --- | --- | |  |  |  |  |  | | RAFL06-10-L13 ,At3g09630  60S ribosomal protein L4/L1 (RPL4A) strong similarity to 60S ribosomal protein L1 GB:P49691 | | | | | | |
|  |  | RAFL07-10-G10 | At1g29880 / glycyl tRNA synthetase -related | |  |  |  |  |  | | --- | --- | --- | --- | --- | |  |  |  |  |  | | RAFL07-10-G10 ,At1g29880  glycyl-tRNA synthetase / glycine--tRNA ligase identical to SP|O23627 Glycyl-tRNA synthetase (EC 6.1.1.14) (Glycine--tRNA ligase) (GlyRS) {Arabidopsis thaliana} | | | | | | |
|  |  | RAFL05-01-F21 | At3g05590 / 60S ribosomal protein L18 (RPL18B) | |  |  |  |  |  | | --- | --- | --- | --- | --- | |  |  |  |  |  | | At3g05590 ,RAFL05-01-F21  60S ribosomal protein L18 (RPL18B) similar to GB:P42791 | | | | | | |
|  |  | RAFL05-12-K17 | At1g67430 / 60S ribosomal protein L17 (RPL17B) | |  |  |  |  |  | | --- | --- | --- | --- | --- | |  |  |  |  |  | | RAFL05-12-K17 ,At1g67430  60S ribosomal protein L17 (RPL17B) similar to ribosomal protein GI:19101 from [Hordeum vulgare] | | | | | | |
|  |  | RAFL05-15-E14 | At1g70600 / 60S ribosomal protein L27A (RPL27aC) | |  |  |  |  |  | | --- | --- | --- | --- | --- | |  |  |  |  |  | | At1g70600 ,RAFL05-15-E14  60S ribosomal protein L27A (RPL27aC) identical to 60S ribosomal protein L27A GB:P49637 [Arabidopsis thaliana] | | | | | | |
|  | Cluster:1-2 | |  |  | 33 | 141 | 388 | 4101 | 3.315521E-5 | 9.283459E-4 | 28 |
|  |  | RAFL06-16-L14 | At5g48760 / 60S ribosomal protein L13A (RPL13aD) | |  |  |  |  |  | | --- | --- | --- | --- | --- | |  |  |  |  |  | | RAFL06-16-L14 ,At5g48760  60S ribosomal protein L13A (RPL13aD) | | | | | | |
|  |  | RAFL09-07-N01 | At2g04390 / 40S ribosomal protein S17 (RPS17A) | |  |  |  |  |  | | --- | --- | --- | --- | --- | |  |  |  |  |  | | At2g04390 ,RAFL09-07-N01  40S ribosomal protein S17 (RPS17A) | | | | | | |
|  |  | RAFL11-10-E06 | At5g45775 / 60S ribosomal protein L11 (RPL11D) | |  |  |  |  |  | | --- | --- | --- | --- | --- | |  |  |  |  |  | | RAFL11-10-E06 ,At5g45775  60S ribosomal protein L11 (RPL11D) | | | | | | |
|  |  | RAFL05-21-A12 | At4g29060 / expressed protein | |  |  |  |  |  | | --- | --- | --- | --- | --- | |  |  |  |  |  | | RAFL05-21-A12 ,At4g29060  elongation factor Ts family protein similar to SP|P35019 Elongation factor Ts (EF-Ts) {Galdieria sulphuraria}; contains Pfam profiles PF00627: UBA/TS-N domain, PF00889: Elongation factor TS, PF00575: S1 RNA binding domain | | | | | | |
|  |  | RAFL07-09-B01 | At4g31700 / 40S ribosomal protein S6 (RPS6A) | |  |  |  |  |  | | --- | --- | --- | --- | --- | |  |  |  |  |  | | RAFL07-09-B01 ,At4g31700  40S ribosomal protein S6 (RPS6A) ribosomal protein S6, Arabidopsis thaliana, PID:g2662469 | | | | | | |
|  |  | RAFL07-08-E09 | At5g49030 / isoleucyl-tRNA synthetase | |  |  |  |  |  | | --- | --- | --- | --- | --- | |  |  |  |  |  | | RAFL07-08-E09 ,At5g49030  tRNA synthetase class I (I, L, M and V) family protein similar to SP|P41972 Isoleucyl-tRNA synthetase (EC 6.1.1.5) (Isoleucine--tRNA ligase) (IleRS) {Staphylococcus aureus}; contains Pfam profile PF00133: tRNA synthetases class I (I, L, M and V) | | | | | | |
|  |  | RAFL04-10-C15 | At1g09590 / 60S ribosomal protein L21 (RPL21A) | |  |  |  |  |  | | --- | --- | --- | --- | --- | |  |  |  |  |  | | RAFL04-10-C15 ,At1g09590  60S ribosomal protein L21 (RPL21A) Similar to L21 family of ribosomal protein; amino acid sequence is identical to F21M12.8 | | | | | | |
|  |  | RAFL06-10-D03 | At5g67510 / 60S ribosomal protein L26 (RPL26B) | |  |  |  |  |  | | --- | --- | --- | --- | --- | |  |  |  |  |  | | At5g67510 ,RAFL06-10-D03  60S ribosomal protein L26 (RPL26B) | | | | | | |
|  |  | RAFL04-12-O17 | At5g30510 / ribosomal protein S1 | |  |  |  |  |  | | --- | --- | --- | --- | --- | |  |  |  |  |  | | At5g30510 ,RAFL04-12-O17  30S ribosomal protein S1, putative similar to Swiss-Prot:P29344 30S ribosomal protein S1, chloroplast precursor (CS1) [Spinacia oleracea] | | | | | | |
|  |  | RAFL06-08-P05 | At3g25520 / 60S ribosomal protein L5 (RPL5A) | |  |  |  |  |  | | --- | --- | --- | --- | --- | |  |  |  |  |  | | At3g25520 ,RAFL06-08-P05  60S ribosomal protein L5 similar to 60S ribosomal protein L5 GB:P49625 from [Oryza sativa] | | | | | | |
|  |  | RAFL06-11-J01 | At1g09690 / 60S ribosomal protein L21 (RPL21C) | |  |  |  |  |  | | --- | --- | --- | --- | --- | |  |  |  |  |  | | RAFL06-11-J01 ,At1g09690  60S ribosomal protein L21 (RPL21C) Similar to ribosomal protein L21 (gb|L38826). ESTs gb|AA395597,gb|ATTS5197 come from this gene | | | | | | |
|  |  | RAFL05-03-J07 | At1g27400 / 60S ribosomal protein L17 (RPL17A) | |  |  |  |  |  | | --- | --- | --- | --- | --- | |  |  |  |  |  | | At1g27400 ,RAFL05-03-J07  60S ribosomal protein L17 (RPL17A) similar to GB:P51413 from [Arabidopsis thaliana]; similar to ESTs gb|L33542 and gb|AA660016 | | | | | | |
|  |  | RAFL09-15-H22 | At1g05190 / ribosomal protein L6p family | |  |  |  |  |  | | --- | --- | --- | --- | --- | |  |  |  |  |  | | At1g05190 ,RAFL09-15-H22  ribosomal protein L6 family protein Similar to Mycobacterium RlpF (gb|Z84395). ESTs gb|T75785,gb|R30580,gb|T04698 come from this gene | | | | | | |
|  |  | RAFL05-14-H02 | At4g34620 / ribosomal protein S16p family | |  |  |  |  |  | | --- | --- | --- | --- | --- | |  |  |  |  |  | | RAFL05-14-H02 ,At4g34620  ribosomal protein S16 family protein ribosomal protein S16, Neurospora crassa, PIR2:A29927 | | | | | | |
|  |  | RAFL05-03-L24 | At5g60670 / 60S ribosomal protein L12 (RPL12C) | |  |  |  |  |  | | --- | --- | --- | --- | --- | |  |  |  |  |  | | RAFL05-03-L24 ,At5g60670  60S ribosomal protein L12 (RPL12C) 60S RIBOSOMAL PROTEIN L12 (like), Arabidopsis thaliana, PIR:T45883 | | | | | | |
|  |  | RAFL06-08-N13 | At3g49010 / 60S ribosomal protein L13 (RPL13B)/breast basic conserved protein 1-related (BBC1) | |  |  |  |  |  | | --- | --- | --- | --- | --- | |  |  |  |  |  | | At3g49010 ,RAFL06-08-N13  60S ribosomal protein L13 (RPL13B) / breast basic conserved protein 1-related (BBC1) | | | | | | |
|  |  | RAFL05-04-J09 | At5g45775 / 60S ribosomal protein L11 (RPL11D) | |  |  |  |  |  | | --- | --- | --- | --- | --- | |  |  |  |  |  | | At5g45775 ,RAFL05-04-J09  60S ribosomal protein L11 (RPL11D) | | | | | | |
|  |  | RAFL05-10-D01 | At2g05220 / 40S ribosomal protein S17 (RPS17B) | |  |  |  |  |  | | --- | --- | --- | --- | --- | |  |  |  |  |  | | RAFL05-10-D01 ,At2g05220  40S ribosomal protein S17 (RPS17B) | | | | | | |
|  |  | RAFL05-18-H13 | At5g65220 / ribosomal protein L29p family | |  |  |  |  |  | | --- | --- | --- | --- | --- | |  |  |  |  |  | | RAFL05-18-H13 ,At5g65220  ribosomal protein L29 family protein contains Pfam profile PF00831: ribosomal protein L29 | | | | | | |
|  |  | RAFL05-14-G11 | At2g33370 / 60S ribosomal protein L23 (RPL23B) | |  |  |  |  |  | | --- | --- | --- | --- | --- | |  |  |  |  |  | | RAFL05-14-G11 ,At2g33370  60S ribosomal protein L23 (RPL23B) | | | | | | |
|  |  | RAFL06-07-D01 | At2g01250 / 60S ribosomal protein L7 (RPL7B) | |  |  |  |  |  | | --- | --- | --- | --- | --- | |  |  |  |  |  | | At2g01250 ,RAFL06-07-D01  60S ribosomal protein L7 (RPL7B) | | | | | | |
|  |  | RAFL04-10-P05 | At4g25740 / 40S ribosomal protein S10 (RPS10A) | |  |  |  |  |  | | --- | --- | --- | --- | --- | |  |  |  |  |  | | At4g25740 ,RAFL04-10-P05  40S ribosomal protein S10 (RPS10A) 40S ribosomal protein S10 - Lumbricus rubellus, PID:e1329701 | | | | | | |
|  |  | RAFL06-13-L16 | At5g07090 / 40S ribosomal protein S4 (RPS4B) | |  |  |  |  |  | | --- | --- | --- | --- | --- | |  |  |  |  |  | | At5g07090 ,RAFL06-13-L16  40S ribosomal protein S4 (RPS4B) | | | | | | |
|  |  | RAFL04-12-J05 | At1g32990 / ribosomal protein L11p family | |  |  |  |  |  | | --- | --- | --- | --- | --- | |  |  |  |  |  | | RAFL04-12-J05 ,At1g32990  ribosomal protein L11 family protein similar to chloroplast ribosomal protein L11 GI:21312 from [Spinacia oleracea] | | | | | | |
|  |  | RAFL05-12-L08 | At2g18020 / 60S ribosomal protein L8 (RPL8A) | |  |  |  |  |  | | --- | --- | --- | --- | --- | |  |  |  |  |  | | At2g18020 ,RAFL05-12-L08  60S ribosomal protein L8 (RPL8A) | | | | | | |
|  |  | RAFL04-16-H06 | At1g08360 / 60S ribosomal protein L10A (RPL10aA) | |  |  |  |  |  | | --- | --- | --- | --- | --- | |  |  |  |  |  | | At1g08360 ,RAFL04-16-H06  60S ribosomal protein L10A (RPL10aA) similar to 60S ribosomal protein L10A GB:AAC73045 GI:3860277 from [Arabidopsis thaliana] | | | | | | |
|  |  | RAFL05-04-H07 | At3g27160 / expressed protein | |  |  |  |  |  | | --- | --- | --- | --- | --- | |  |  |  |  |  | | RAFL05-04-H07 ,At3g27160  ribosomal protein S21 family protein contains Pfam profile: PF01165 ribosomal protein S21 | | | | | | |
|  |  | RAFL05-13-G24 | At5g54600 / 50S ribosomal protein L24, chloroplast precursor (CL24) | |  |  |  |  |  | | --- | --- | --- | --- | --- | |  |  |  |  |  | | At5g54600 ,RAFL05-13-G24  50S ribosomal protein L24, chloroplast (CL24) identical to SP|P92959 50S ribosomal protein L24, chloroplast precursor {Arabidopsis thaliana} | | | | | | |
|  |  | RAFL07-16-I10 | At4g34620 / ribosomal protein S16p family | |  |  |  |  |  | | --- | --- | --- | --- | --- | |  |  |  |  |  | | RAFL07-16-I10 ,At4g34620  ribosomal protein S16 family protein ribosomal protein S16, Neurospora crassa, PIR2:A29927 | | | | | | |
|  |  | RAFL09-11-E10 | At1g17220 / translation initiation factor IF-2, chloroplast precursor | |  |  |  |  |  | | --- | --- | --- | --- | --- | |  |  |  |  |  | | RAFL09-11-E10 ,At1g17220  translation initiation factor IF-2, chloroplast, putative similar to SP|P57997|IF2C\_PHAVU Translation initiation factor IF-2, chloroplast precursor (PvIF2cp) {Phaseolus vulgaris} | | | | | | |
|  |  | RAFL04-15-L21 | At1g41880 / 60S ribosomal protein L35a (RPL35aB) | |  |  |  |  |  | | --- | --- | --- | --- | --- | |  |  |  |  |  | | RAFL04-15-L21 ,At1g41880  60S ribosomal protein L35a (RPL35aB) identical to GB:CAB81600 from [Arabidopsis thaliana] | | | | | | |
|  |  | RAFL05-19-N16 | At3g45030 / 40S ribosomal protein S20 (RPS20A) | |  |  |  |  |  | | --- | --- | --- | --- | --- | |  |  |  |  |  | | At3g45030 ,RAFL05-19-N16  40S ribosomal protein S20 (RPS20A) 40S ribsomomal proteinS20, Arabidopsis thaliana, pir:T12992 | | | | | | |
|  |  | RAFL11-12-P09 | At3g54210 / ribosomal protein L17 -related protein | |  |  |  |  |  | | --- | --- | --- | --- | --- | |  |  |  |  |  | | RAFL11-12-P09 ,At3g54210  ribosomal protein L17 family protein contains Pfam profile: PF01196 ribosomal protein L17 | | | | | | |
| GO:0003677 | | | DNA binding |  | A | B | C | D | P | P' | N |
|  | Cluster:0-2 | |  |  | 30 | 49 | 874 | 3710 | 1.301411E-4 | 0.004294656 | 33 |
|  |  | RAFL04-14-I05 | At3g48200 / expressed protein | |  |  |  |  |  | | --- | --- | --- | --- | --- | |  |  |  |  |  | | At3g48200 ,RAFL04-14-I05  expressed protein | | | | | | |
|  |  | RAFL06-11-B13 | At5g58250 / unknown protein (sp|P72777) -related | |  |  |  |  |  | | --- | --- | --- | --- | --- | |  |  |  |  |  | | At5g58250 ,RAFL06-11-B13  expressed protein | | | | | | |
|  |  | RAFL06-12-C05 | At1g08380 / expressed protein | |  |  |  |  |  | | --- | --- | --- | --- | --- | |  |  |  |  |  | | RAFL06-12-C05 ,At1g08380  expressed protein | | | | | | |
|  |  | RAFL05-19-G04 | At1g54780 / thylakoid lumen 18.3 kDa protein | |  |  |  |  |  | | --- | --- | --- | --- | --- | |  |  |  |  |  | | RAFL05-19-G04 ,At1g54780  thylakoid lumen 18.3 kDa protein SP:Q9ZVL6 | | | | | | |
|  |  | RAFL04-19-B03 | At3g16140 / photosystem I subunit VI precursor | |  |  |  |  |  | | --- | --- | --- | --- | --- | |  |  |  |  |  | | At3g16140 ,RAFL04-19-B03  photosystem I reaction center subunit VI, chloroplast, putative / PSI-H, putative (PSAH1) identical to SP|Q9SUI7; similar to PSI-H precursor [Nicotiana sylvestris] GI:407353; contains Pfam profile PF03244: Photosystem I reaction centre subunit VI | | | | | | |
|  |  | RAFL02-02-L10 | At1g03130 / photosystem I reaction center subunit II precursor -related | |  |  |  |  |  | | --- | --- | --- | --- | --- | |  |  |  |  |  | | RAFL02-02-L10 ,At1g03130  photosystem I reaction center subunit II, chloroplast, putative / photosystem I 20 kDa subunit, putative / PSI-D, putative (PSAD2) similar to SP|P12353 Photosystem I reaction center subunit II, chloroplast precursor (Photosystem I 20 kDa subunit) (PSI-D) {Spinacia oleracea}; contains Pfam profile PF02531: PsaD | | | | | | |
|  |  | RAFL05-18-G05 | At3g62410 / CP12 protein precursor-related protein | |  |  |  |  |  | | --- | --- | --- | --- | --- | |  |  |  |  |  | | RAFL05-18-G05 ,At3g62410  CP12 domain-containing protein contains Pfam domain PF02672: CP12 domain | | | | | | |
|  |  | RAFL05-17-G01 | At1g51400 / photosystem II 5 KD protein | |  |  |  |  |  | | --- | --- | --- | --- | --- | |  |  |  |  |  | | At1g51400 ,RAFL05-17-G01  photosystem II 5 kD protein 100% identical to GI:4836947 (F5D21.10) | | | | | | |
|  |  | RAFL06-12-P20 | At1g28400 / expressed protein | |  |  |  |  |  | | --- | --- | --- | --- | --- | |  |  |  |  |  | | RAFL06-12-P20 ,At1g28400  expressed protein similar to E6 (GI:1000090) [Gossypium barbadense] | | | | | | |
|  |  | RAFL07-14-F21 | At1g54780 / thylakoid lumen 18.3 kDa protein | |  |  |  |  |  | | --- | --- | --- | --- | --- | |  |  |  |  |  | | RAFL07-14-F21 ,At1g54780  thylakoid lumen 18.3 kDa protein SP:Q9ZVL6 | | | | | | |
|  |  | RAFL09-06-G16 | At1g31330 / photosystem I subunit III precursor -related | |  |  |  |  |  | | --- | --- | --- | --- | --- | |  |  |  |  |  | | At1g31330 ,RAFL09-06-G16  photosystem I reaction center subunit III family protein contains Pfam profile: PF02507: photosystem I reaction center subunit III | | | | | | |
|  |  | RAFL06-07-J08 | At3g56360 / expressed protein | |  |  |  |  |  | | --- | --- | --- | --- | --- | |  |  |  |  |  | | RAFL06-07-J08 ,At3g56360  expressed protein unknown protein 110K5.12 - Sorghum bicolor, TREMBL:AF124045\_5 | | | | | | |
|  |  | RAFL04-16-N08 | At5g47110 / Lil3 protein | |  |  |  |  |  | | --- | --- | --- | --- | --- | |  |  |  |  |  | | RAFL04-16-N08 ,At5g47110  lil3 protein, putative similar to Lil3 protein [Arabidopsis thaliana] gi|4741966|gb|AAD28780 | | | | | | |
|  |  | RAFL04-17-P09 | At3g47430 / expressed protein | |  |  |  |  |  | | --- | --- | --- | --- | --- | |  |  |  |  |  | | At3g47430 ,RAFL04-17-P09  peroxisomal biogenesis factor 11 family protein / PEX11 family protein contains Pfam PF05648: Peroxisomal biogenesis factor 11 (PEX11) | | | | | | |
|  |  | RAFL05-09-F03 | At1g31330 / photosystem I subunit III precursor -related | |  |  |  |  |  | | --- | --- | --- | --- | --- | |  |  |  |  |  | | At1g31330 ,RAFL05-09-F03  photosystem I reaction center subunit III family protein contains Pfam profile: PF02507: photosystem I reaction center subunit III | | | | | | |
|  |  | RAFL05-16-F08 | At1g31330 / photosystem I subunit III precursor -related | |  |  |  |  |  | | --- | --- | --- | --- | --- | |  |  |  |  |  | | RAFL05-16-F08 ,At1g31330  photosystem I reaction center subunit III family protein contains Pfam profile: PF02507: photosystem I reaction center subunit III | | | | | | |
|  |  | RAFL06-12-L07 | At1g08380 / expressed protein | |  |  |  |  |  | | --- | --- | --- | --- | --- | |  |  |  |  |  | | At1g08380 ,RAFL06-12-L07  expressed protein | | | | | | |
|  |  | RAFL05-17-G02 | At4g27700 / rhodanese-like domain protein | |  |  |  |  |  | | --- | --- | --- | --- | --- | |  |  |  |  |  | | At4g27700 ,RAFL05-17-G02  rhodanese-like domain-containing protein contains rhodanese-like domain PF00581 | | | | | | |
|  |  | RAFL09-16-K15 | At1g31330 / photosystem I subunit III precursor -related | |  |  |  |  |  | | --- | --- | --- | --- | --- | |  |  |  |  |  | | At1g31330 ,RAFL09-16-K15  photosystem I reaction center subunit III family protein contains Pfam profile: PF02507: photosystem I reaction center subunit III | | | | | | |
|  |  | RAFL04-19-G19 | At1g74730 / expressed protein | |  |  |  |  |  | | --- | --- | --- | --- | --- | |  |  |  |  |  | | At1g74730 ,RAFL04-19-G19  expressed protein | | | | | | |
|  |  | RAFL05-01-I05 | At1g54780 / thylakoid lumen 18.3 kDa protein | |  |  |  |  |  | | --- | --- | --- | --- | --- | |  |  |  |  |  | | RAFL05-01-I05 ,At1g54780  thylakoid lumen 18.3 kDa protein SP:Q9ZVL6 | | | | | | |
|  |  | RAFL06-07-K01 | At4g12800 / probable photosystem I chain XI precursor | |  |  |  |  |  | | --- | --- | --- | --- | --- | |  |  |  |  |  | | At4g12800 ,RAFL06-07-K01  photosystem I reaction center subunit XI, chloroplast (PSI-L) / PSI subunit V identical to Photosystem I reaction center subunit XI, chloroplast precursor (PSI-L) (PSI subunit V) (Swiss-Prot:Q9SUI4) [Arabidopsis thaliana]; contains Pfam profile PF02605: photosystem I reaction center subunit XI; contains 2 transmembrane domains | | | | | | |
|  |  | RAFL05-21-A21 | At4g26850 / expressed protein | |  |  |  |  |  | | --- | --- | --- | --- | --- | |  |  |  |  |  | | RAFL05-21-A21 ,At4g26850  expressed protein | | | | | | |
|  |  | RAFL07-15-A19 | At4g02770 / photosystem I reaction center subunit II precursor -related | |  |  |  |  |  | | --- | --- | --- | --- | --- | |  |  |  |  |  | | RAFL07-15-A19 ,At4g02770  photosystem I reaction center subunit II, chloroplast, putative / photosystem I 20 kDa subunit, putative / PSI-D, putative (PSAD1) similar to SP|P12353 Photosystem I reaction center subunit II, chloroplast precursor (Photosystem I 20 kDa subunit) (PSI-D) {Spinacia oleracea}; contains Pfam profile PF02531: PsaD | | | | | | |
|  |  | RAFL08-12-J08 | At1g14345 / expressed protein | |  |  |  |  |  | | --- | --- | --- | --- | --- | |  |  |  |  |  | | At1g14345 ,RAFL08-12-J08  expressed protein contains one transmembrane domain | | | | | | |
|  |  | RAFL08-15-B05 | At1g08380 / expressed protein | |  |  |  |  |  | | --- | --- | --- | --- | --- | |  |  |  |  |  | | RAFL08-15-B05 ,At1g08380  expressed protein | | | | | | |
|  |  | RAFL06-15-G08 | At1g52220 / expressed protein | |  |  |  |  |  | | --- | --- | --- | --- | --- | |  |  |  |  |  | | At1g52220 ,RAFL06-15-G08  expressed protein | | | | | | |
|  |  | RAFL05-04-A19 | At1g51400 / photosystem II 5 KD protein | |  |  |  |  |  | | --- | --- | --- | --- | --- | |  |  |  |  |  | | At1g51400 ,RAFL05-04-A19  photosystem II 5 kD protein 100% identical to GI:4836947 (F5D21.10) | | | | | | |
|  |  | RAFL06-16-J22 | At4g12800 / probable photosystem I chain XI precursor | |  |  |  |  |  | | --- | --- | --- | --- | --- | |  |  |  |  |  | | At4g12800 ,RAFL06-16-J22  photosystem I reaction center subunit XI, chloroplast (PSI-L) / PSI subunit V identical to Photosystem I reaction center subunit XI, chloroplast precursor (PSI-L) (PSI subunit V) (Swiss-Prot:Q9SUI4) [Arabidopsis thaliana]; contains Pfam profile PF02605: photosystem I reaction center subunit XI; contains 2 transmembrane domains | | | | | | |
|  |  | RAFL04-10-O12 | At1g12250 / chloroplast lumen pentapeptide protein, putative | |  |  |  |  |  | | --- | --- | --- | --- | --- | |  |  |  |  |  | | RAFL04-10-O12 ,At1g12250  thylakoid lumenal protein-related weak similarity to SP|O22160 Thylakoid lumenal 15 kDa protein, chloroplast precursor (p15) {Arabidopsis thaliana}; contains Pfam profile PF00805: Pentapeptide repeats (8 copies) | | | | | | |
|  | Cluster:10-1 | |  |  | 18 | 25 | 886 | 3734 | 6.532788E-4 | 0.0215582 | 33 |
|  |  | RAFL05-03-A05 | At2g42540 / cold-regulated protein (cor15a) | |  |  |  |  |  | | --- | --- | --- | --- | --- | |  |  |  |  |  | | At2g42540 ,RAFL05-03-A05  cold-responsive protein / cold-regulated protein (cor15a) identical to cold-regulated protein cor15a [Arabidopsis thaliana] GI:507149; contains Pfam profile PF02987: Late embryogenesis abundant protein | | | | | | |
|  |  | RAFL08-11-P07 | At5g17460 / expressed protein | |  |  |  |  |  | | --- | --- | --- | --- | --- | |  |  |  |  |  | | At5g17460 ,RAFL08-11-P07  expressed protein | | | | | | |
|  |  | RAFL05-14-I08 | At5g64310 / arabinogalactan-protein (AGP1) | |  |  |  |  |  | | --- | --- | --- | --- | --- | |  |  |  |  |  | | At5g64310 ,RAFL05-14-I08  arabinogalactan-protein (AGP1) identical to gi:3883120 gb:AAC77823 | | | | | | |
|  |  | RAFL05-08-B11 | At3g61060 / F-box protein (lectin-related) | |  |  |  |  |  | | --- | --- | --- | --- | --- | |  |  |  |  |  | | RAFL05-08-B11 ,At3g61060  F-box family protein / lectin-related low similarity to PP2 lectin polypeptide [Cucurbita maxima] GI:410437; contains Pfam profile PF00646: F-box domain | | | | | | |
|  |  | RAFL08-19-H17 | At2g17840 / senescence-associated protein 12 -related | |  |  |  |  |  | | --- | --- | --- | --- | --- | |  |  |  |  |  | | At2g17840 ,RAFL08-19-H17  senescence/dehydration-associated protein-related (ERD7) similar to senescence-associated protein 12 [Hemerocallis hybrid cultivar] gi|3551958|gb|AAC34857; strong similarity to early-responsive to dehydration stress ERD7 protein [Arabidopsis thaliana] gi|15320412|dbj|BAB63916; identical to cDNA ERD7 partial cds GI:15320411 | | | | | | |
|  |  | RAFL05-09-K04 | At5g50360 / expressed protein | |  |  |  |  |  | | --- | --- | --- | --- | --- | |  |  |  |  |  | | At5g50360 ,RAFL05-09-K04  expressed protein | | | | | | |
|  |  | RAFL06-08-N16 | At5g15960 / stress-induced protein KIN1 | |  |  |  |  |  | | --- | --- | --- | --- | --- | |  |  |  |  |  | | At5g15960 ,RAFL06-08-N16  stress-responsive protein (KIN1) / stress-induced protein (KIN1) identical to SP|P18612 Stress-induced KIN1 protein {Arabidopsis thaliana} | | | | | | |
|  |  | RAFL03-07-M07 | At4g02380 / late embryogenesis abundant protein family | |  |  |  |  |  | | --- | --- | --- | --- | --- | |  |  |  |  |  | | RAFL03-07-M07 ,At4g02380  late embryogenesis abundant 3 family protein / LEA3 family protein similar to several small proteins (~100 aa) that are induced by heat, auxin, ethylene and wounding such as Phaseolus aureus indole-3-acetic acid induced protein ARG (SW:32292); contains Pfam profile PF03242: Late embryogenesis abundant protein | | | | | | |
|  |  | RAFL05-08-P17 | At1g20450 / dehydrin (ERD10) | |  |  |  |  |  | | --- | --- | --- | --- | --- | |  |  |  |  |  | | RAFL05-08-P17 ,At1g20450  dehydrin (ERD10) identical to dehydrin ERD10 (Low-temperature-induced protein LTI45) [Arabidopsis thaliana] SWISS-PROT:P42759 | | | | | | |
|  |  | RAFL05-04-C07 | At1g20450 / dehydrin (ERD10) | |  |  |  |  |  | | --- | --- | --- | --- | --- | |  |  |  |  |  | | RAFL05-04-C07 ,At1g20450  dehydrin (ERD10) identical to dehydrin ERD10 (Low-temperature-induced protein LTI45) [Arabidopsis thaliana] SWISS-PROT:P42759 | | | | | | |
|  |  | RAFL09-14-A12 | At1g78070 / expressed protein | |  |  |  |  |  | | --- | --- | --- | --- | --- | |  |  |  |  |  | | At1g78070 ,RAFL09-14-A12  WD-40 repeat family protein contains Pfam profile PF00400: WD domain, G-beta repeat | | | | | | |
|  |  | RAFL04-20-N09 | At1g20440 / dehydrin (COR47) | |  |  |  |  |  | | --- | --- | --- | --- | --- | |  |  |  |  |  | | RAFL04-20-N09 ,At1g20440  dehydrin (COR47) identical to dehydrin COR47 (Cold-induced COR47 protein) [Arabidopsis thaliana] SWISS-PROT:P31168 | | | | | | |
|  |  | RAFL08-18-N19 | At2g22470 / arabinogalactan-protein (AGP2) | |  |  |  |  |  | | --- | --- | --- | --- | --- | |  |  |  |  |  | | RAFL08-18-N19 ,At2g22470  arabinogalactan-protein (AGP2) identical to gi|3883122|gb|AAC77824; supported by cDNA gi|3883121|gb|AF082299 | | | | | | |
|  |  | RAFL06-12-H12 | At5g02020 / expressed protein | |  |  |  |  |  | | --- | --- | --- | --- | --- | |  |  |  |  |  | | At5g02020 ,RAFL06-12-H12  expressed protein | | | | | | |
|  |  | RAFL06-13-N20 | At4g02380 / late embryogenesis abundant protein family | |  |  |  |  |  | | --- | --- | --- | --- | --- | |  |  |  |  |  | | RAFL06-13-N20 ,At4g02380  late embryogenesis abundant 3 family protein / LEA3 family protein similar to several small proteins (~100 aa) that are induced by heat, auxin, ethylene and wounding such as Phaseolus aureus indole-3-acetic acid induced protein ARG (SW:32292); contains Pfam profile PF03242: Late embryogenesis abundant protein | | | | | | |
|  |  | RAFL05-14-G18 | At5g61820 / expressed protein | |  |  |  |  |  | | --- | --- | --- | --- | --- | |  |  |  |  |  | | At5g61820 ,RAFL05-14-G18  expressed protein MtN19, Medicago truncatula, EMBL:MTY15367 | | | | | | |
|  |  | RAFL05-17-B13 | At1g01470 / expressed protein | |  |  |  |  |  | | --- | --- | --- | --- | --- | |  |  |  |  |  | | RAFL05-17-B13 ,At1g01470  late embryogenesis abundant protein, putative / LEA protein, putative similar to SP|P46518 Late embryogenesis abundant protein Lea14-A {Gossypium hirsutum}; contains Pfam profile PF03168: Late embryogenesis abundant protein | | | | | | |
|  |  | RAFL08-11-C23 | At5g06760 / late embryogenesis abundant protein LEA like | |  |  |  |  |  | | --- | --- | --- | --- | --- | |  |  |  |  |  | | RAFL08-11-C23 ,At5g06760  late embryogenesis abundant group 1 domain-containing protein / LEA group 1 domain-containing protein low similarity to SP|P46515 11 kDa late embryogenesis abundant protein (DS11) {Helianthus annuus}; contains Pfam profile PF03760: Late embryogenesis abundant (LEA) group 1 | | | | | | |
|  | Cluster:10-0 | |  |  | 12 | 12 | 892 | 3747 | 7.0534577E-4 | 0.023276411 | 33 |
|  |  | RAFL05-04-I14 | At1g52690 / late embryogenesis abundant (LEA) protein, putative | |  |  |  |  |  | | --- | --- | --- | --- | --- | |  |  |  |  |  | | At1g52690 ,RAFL05-04-I14  late embryogenesis abundant protein, putative / LEA protein, putative similar to SP|P13934 Late embryogenesis abundant protein 76 (LEA 76) {Brassica napus}; contains Pfam profile PF02987: Late embryogenesis abundant protein | | | | | | |
|  |  | RAFL08-13-P06 | At1g52690 / late embryogenesis abundant (LEA) protein, putative | |  |  |  |  |  | | --- | --- | --- | --- | --- | |  |  |  |  |  | | At1g52690 ,RAFL08-13-P06  late embryogenesis abundant protein, putative / LEA protein, putative similar to SP|P13934 Late embryogenesis abundant protein 76 (LEA 76) {Brassica napus}; contains Pfam profile PF02987: Late embryogenesis abundant protein | | | | | | |
|  |  | RAFL05-09-G08 | At3g15670 / late embryogenesis abundant (LEA) protein, putative | |  |  |  |  |  | | --- | --- | --- | --- | --- | |  |  |  |  |  | | At3g15670 ,RAFL05-09-G08  late embryogenesis abundant protein, putative / LEA protein, putative similar to SP|P13934 Late embryogenesis abundant protein 76 (LEA 76) {Brassica napus}; contains Pfam profile PF02987: Late embryogenesis abundant protein | | | | | | |
|  |  | RAFL08-14-E03 | At1g52690 / late embryogenesis abundant (LEA) protein, putative | |  |  |  |  |  | | --- | --- | --- | --- | --- | |  |  |  |  |  | | At1g52690 ,RAFL08-14-E03  late embryogenesis abundant protein, putative / LEA protein, putative similar to SP|P13934 Late embryogenesis abundant protein 76 (LEA 76) {Brassica napus}; contains Pfam profile PF02987: Late embryogenesis abundant protein | | | | | | |
|  |  | RAFL08-13-F10 | At5g03210 / expressed protein | |  |  |  |  |  | | --- | --- | --- | --- | --- | |  |  |  |  |  | | RAFL08-13-F10 ,At5g03210  expressed protein | | | | | | |
|  |  | RAFL05-21-F13 | At1g16850 / expressed protein | |  |  |  |  |  | | --- | --- | --- | --- | --- | |  |  |  |  |  | | At1g16850 ,RAFL05-21-F13  expressed protein | | | | | | |
|  |  | RAFL06-13-J20 | At1g52690 / late embryogenesis abundant (LEA) protein, putative | |  |  |  |  |  | | --- | --- | --- | --- | --- | |  |  |  |  |  | | At1g52690 ,RAFL06-13-J20  late embryogenesis abundant protein, putative / LEA protein, putative similar to SP|P13934 Late embryogenesis abundant protein 76 (LEA 76) {Brassica napus}; contains Pfam profile PF02987: Late embryogenesis abundant protein | | | | | | |
|  |  | RAFL06-10-C16 | At1g05340 / expressed protein | |  |  |  |  |  | | --- | --- | --- | --- | --- | |  |  |  |  |  | | RAFL06-10-C16 ,At1g05340  expressed protein | | | | | | |
|  |  | RAFL04-17-F01 | At5g52310 / low-temperature-induced protein 78 (sp|Q06738) | |  |  |  |  |  | | --- | --- | --- | --- | --- | |  |  |  |  |  | | RAFL04-17-F01 ,At5g52310  low-temperature-responsive protein 78 (LTI78) / desiccation-responsive protein 29A (RD29A) | | | | | | |
|  |  | RAFL05-11-I09 | At5g52300 / low-temperature-induced 65 kD protein (sp|Q04980) | |  |  |  |  |  | | --- | --- | --- | --- | --- | |  |  |  |  |  | | RAFL05-11-I09 ,At5g52300  low-temperature-responsive 65 kD protein (LTI65) / desiccation-responsive protein 29B (RD29B) nearly identical to SP|Q04980 Low-temperature-induced 65 kDa protein (Desiccation-responsive protein 29B) {Arabidopsis thaliana} | | | | | | |
|  |  | RAFL09-17-M11 | At1g20450 / dehydrin (ERD10) | |  |  |  |  |  | | --- | --- | --- | --- | --- | |  |  |  |  |  | | RAFL09-17-M11 ,At1g20450  dehydrin (ERD10) identical to dehydrin ERD10 (Low-temperature-induced protein LTI45) [Arabidopsis thaliana] SWISS-PROT:P42759 | | | | | | |
|  |  | RAFL07-11-M21 | At5g52310 / low-temperature-induced protein 78 (sp|Q06738) | |  |  |  |  |  | | --- | --- | --- | --- | --- | |  |  |  |  |  | | At5g52310 ,RAFL07-11-M21  low-temperature-responsive protein 78 (LTI78) / desiccation-responsive protein 29A (RD29A) | | | | | | |
|  | Cluster:6-0 | |  |  | 38 | 102 | 866 | 3657 | 0.022377433 | 0.7384553 | 33 |
|  |  | RAFL06-15-H18 | At3g26740 / light regulated protein -related | |  |  |  |  |  | | --- | --- | --- | --- | --- | |  |  |  |  |  | | At3g26740 ,RAFL06-15-H18  light responsive protein-related similar to light regulated protein precursor SP:Q03200 [Oryza sativa] (Plant Mol. Biol. 22 (1), 165-170 (1993)), ccr protein GB:S52663 [Citrus X paradisi] (Plant Mol. Biol. 26 (1), 165-173 (1994)) | | | | | | |
|  |  | RAFL09-17-C18 | At5g26260 / expressed protein | |  |  |  |  |  | | --- | --- | --- | --- | --- | |  |  |  |  |  | | At5g26260 ,RAFL09-17-C18  meprin and TRAF homology domain-containing protein / MATH domain-containing protein similar to ubiquitin-specific protease 12 [Arabidopsis thaliana] GI:11993471; contains Pfam profile PF00917: MATH domain | | | | | | |
|  |  | RAFL05-08-E02 | At1g21680 / expressed protein | |  |  |  |  |  | | --- | --- | --- | --- | --- | |  |  |  |  |  | | RAFL05-08-E02 ,At1g21680  expressed protein similar to TolB protein precursor (SP:Q9ZDM5) {Rickettsia prowazekii}; ESTs gb|N96028, gb|F14286, gb|T20680, gb|F14443, gb|AA657300 and gb|N65244 come from this gene | | | | | | |
|  |  | RAFL11-12-C05 | At4g20260 / endomembrane-associated protein | |  |  |  |  |  | | --- | --- | --- | --- | --- | |  |  |  |  |  | | RAFL11-12-C05 ,At4g20260  DREPP plasma membrane polypeptide family protein contains Pfam profile: PF05558 DREPP plasma membrane polypeptide | | | | | | |
|  |  | RAFL08-09-C07 | At3g43520 / expressed protein | |  |  |  |  |  | | --- | --- | --- | --- | --- | |  |  |  |  |  | | At3g43520 ,RAFL08-09-C07  expressed protein contains Pfam profile PF03647: Uncharacterised protein family (UPF0136) | | | | | | |
|  |  | RAFL05-01-C20 | At4g23670 / major latex protein (MLP)-related | |  |  |  |  |  | | --- | --- | --- | --- | --- | |  |  |  |  |  | | At4g23670 ,RAFL05-01-C20  major latex protein-related / MLP-related low similarity to major latex protein {Papaver somniferum}[GI:294060] contains Pfam profile PF00407: Pathogenesis-related protein Bet v I family | | | | | | |
|  |  | RAFL08-08-E16 | At1g80240 / expressed protein | |  |  |  |  |  | | --- | --- | --- | --- | --- | |  |  |  |  |  | | At1g80240 ,RAFL08-08-E16  expressed protein contains Pfam profile PF04862: Protein of unknown function, DUF642 | | | | | | |
|  |  | RAFL05-19-F14 | At2g47960 / expressed protein | |  |  |  |  |  | | --- | --- | --- | --- | --- | |  |  |  |  |  | | RAFL05-19-F14 ,At2g47960  expressed protein | | | | | | |
|  |  | RAFL08-08-J06 | At5g01350 / expressed protein | |  |  |  |  |  | | --- | --- | --- | --- | --- | |  |  |  |  |  | | RAFL08-08-J06 ,At5g01350  expressed protein | | | | | | |
|  |  | RAFL06-15-O20 | At2g38040 / alpha-carboxyltransferase -related | |  |  |  |  |  | | --- | --- | --- | --- | --- | |  |  |  |  |  | | RAFL06-15-O20 ,At2g38040  acetyl co-enzyme A carboxylase carboxyltransferase alpha subunit family contains Pfam profile: PF03255: Acetyl co-enzyme A carboxylase carboxyltransferase alpha subunit | | | | | | |
|  |  | RAFL04-13-A14 | At5g04850 / expressed protein | |  |  |  |  |  | | --- | --- | --- | --- | --- | |  |  |  |  |  | | At5g04850 ,RAFL04-13-A14  SNF7 family protein contains Pfam domain, PF03357: SNF7 family | | | | | | |
|  |  | RAFL11-01-G12 | At1g47420 / expressed protein | |  |  |  |  |  | | --- | --- | --- | --- | --- | |  |  |  |  |  | | At1g47420 ,RAFL11-01-G12  expressed protein identical to hypothetical protein GB:AAD46040 GI:5668814 from [Arabidopsis thaliana] | | | | | | |
|  |  | RAFL02-04-I04 | At5g35570 / axi 1 (auxin-independent growth promoter)-related protein | |  |  |  |  |  | | --- | --- | --- | --- | --- | |  |  |  |  |  | | At5g35570 ,RAFL02-04-I04  expressed protein similar to axi 1 [Nicotiana tabacum] GI:559921; contains Pfam profile PF03138: Plant protein family | | | | | | |
|  |  | RAFL11-05-P19 | At5g14170 / expressed protein | |  |  |  |  |  | | --- | --- | --- | --- | --- | |  |  |  |  |  | | At5g14170 ,RAFL11-05-P19  SWIB complex BAF60b domain-containing protein similar to brahma associated protein 60 kDa [Drosophila melanogaster] GI:3378134, SWI/SNF-related, matrix-associated, actin-dependent regulator of chromatin D1 [Homo sapiens] GI:4566530; contains Pfam profile PF02201: BAF60b domain of the SWIB complex | | | | | | |
|  |  | RAFL08-11-B02 | At3g23080 / expressed protein | |  |  |  |  |  | | --- | --- | --- | --- | --- | |  |  |  |  |  | | RAFL08-11-B02 ,At3g23080  expressed protein weak similarity to SP|Q9UKL6 Phosphatidylcholine transfer protein (PC-TP) {Homo sapiens} | | | | | | |
|  |  | RAFL09-06-O13 | At1g15270 / expressed protein | |  |  |  |  |  | | --- | --- | --- | --- | --- | |  |  |  |  |  | | At1g15270 ,RAFL09-06-O13  expressed protein ESTs gb|AA650895, gb|AA720043 and gb|R29777 come from this gene | | | | | | |
|  |  | RAFL05-19-E09 | At5g59140 / SKP1 family | |  |  |  |  |  | | --- | --- | --- | --- | --- | |  |  |  |  |  | | At5g59140 ,RAFL05-19-E09  SKP1 family protein similar to elongin C, Drosophila melanogaster, GI:2780365 PIR:JC5794; contains Pfam profile PF01466: Skp1 family, dimerisation domain | | | | | | |
|  |  | RAFL07-08-A14 | At3g26890 / expressed protein | |  |  |  |  |  | | --- | --- | --- | --- | --- | |  |  |  |  |  | | RAFL07-08-A14 ,At3g26890  expressed protein | | | | | | |
|  |  | RAFL05-02-P09 | At4g27450 / expressed protein | |  |  |  |  |  | | --- | --- | --- | --- | --- | |  |  |  |  |  | | RAFL05-02-P09 ,At4g27450  expressed protein similar to auxin down-regulated protein ARG10 [Vigna radiata] GI:2970051, wali7 (aluminum-induced protein) [Triticum aestivum] GI:451193 | | | | | | |
|  |  | RAFL08-17-M09 | At3g02200 / expressed protein | |  |  |  |  |  | | --- | --- | --- | --- | --- | |  |  |  |  |  | | At3g02200 ,RAFL08-17-M09  proteasome family protein contains Pfam domain, PF01399: PCI domain | | | | | | |
|  |  | RAFL04-20-C09 | At1g71780 / expressed protein | |  |  |  |  |  | | --- | --- | --- | --- | --- | |  |  |  |  |  | | RAFL04-20-C09 ,At1g71780  expressed protein | | | | | | |
|  |  | RAFL05-01-C15 | At1g30200 / expressed protein | |  |  |  |  |  | | --- | --- | --- | --- | --- | |  |  |  |  |  | | At1g30200 ,RAFL05-01-C15  F-box family protein contains Pfam PF00646: F-box domain; similar to hypothetical protein GI:2832643 from [Arabidopsis thaliana] | | | | | | |
|  |  | RAFL06-15-K18 | At1g54410 / dehydrin protein family | |  |  |  |  |  | | --- | --- | --- | --- | --- | |  |  |  |  |  | | RAFL06-15-K18 ,At1g54410  dehydrin family protein contains Pfam domain, PF00257: Dehydrin | | | | | | |
|  |  | RAFL05-04-A22 | At2g15690 / expressed protein | |  |  |  |  |  | | --- | --- | --- | --- | --- | |  |  |  |  |  | | At2g15690 ,RAFL05-04-A22  pentatricopeptide (PPR) repeat-containing protein contains Pfam profile PF01535: PPR repeat | | | | | | |
|  |  | RAFL02-07-H24 | At2g45740 / expressed protein | |  |  |  |  |  | | --- | --- | --- | --- | --- | |  |  |  |  |  | | At2g45740 ,RAFL02-07-H24  peroxisomal biogenesis factor 11 family protein / PEX11 family protein contains Pfam profile PF05648: Peroxisomal biogenesis factor 11 (PEX11) | | | | | | |
|  |  | RAFL07-18-B18 | At1g68050 / F-box protein FKF1/ADO3, AtFBX2a | |  |  |  |  |  | | --- | --- | --- | --- | --- | |  |  |  |  |  | | At1g68050 ,RAFL07-18-B18  F-box family protein (FKF1) / adagio 3 (ADO3) E3 ubiquitin ligase SCF complex F-box subunit; identical to FKF1 GI:6960305 and Adagio 3 GI:13487072 from [Arabidopsis thaliana]; contains Pfam profiles PF01344: Kelch motif, PF00785: PAC motif and PF00646: F-box domain; contains TIGRfam profile TIGR00229: PAS domain S-boxidentical to cDNA Adagio 3 (ADO3) GI:13487071 | | | | | | |
|  |  | RAFL05-17-F04 | At4g28240 / wound induced protein -related | |  |  |  |  |  | | --- | --- | --- | --- | --- | |  |  |  |  |  | | At4g28240 ,RAFL05-17-F04  wound-responsive protein-related wound-induced protein - tomato (fragment), PIR2:S19773 | | | | | | |
|  |  | RAFL09-17-C08 | At3g48680 / transferase hexapeptide repeat family | |  |  |  |  |  | | --- | --- | --- | --- | --- | |  |  |  |  |  | | At3g48680 ,RAFL09-17-C08  bacterial transferase hexapeptide repeat-containing protein contains Pfam profile PF00132: Bacterial transferase hexapeptide (four repeats); ferripyochelin binding protein - Methanobacterium thermoautotrophicum, EMBL:AE000918.1 | | | | | | |
|  |  | RAFL06-09-E11 | At2g04900 / expressed protein | |  |  |  |  |  | | --- | --- | --- | --- | --- | |  |  |  |  |  | | At2g04900 ,RAFL06-09-E11  expressed protein | | | | | | |
|  |  | RAFL05-13-B18 | At1g56220 / expressed protein | |  |  |  |  |  | | --- | --- | --- | --- | --- | |  |  |  |  |  | | RAFL05-13-B18 ,At1g56220  dormancy/auxin associated family protein similar to Auxin-repressed 12.5 kDa protein (Swiss-Prot:Q05349) [Fragaria ananassa]; similar to auxin-repressed protein (GI:927034) [Fragaria x ananassa]; similar to dormancy-associated protein (GI:2605887) [Pisum sativum] | | | | | | |
|  |  | RAFL07-15-I05 | At4g36980 / expressed protein | |  |  |  |  |  | | --- | --- | --- | --- | --- | |  |  |  |  |  | | At4g36980 ,RAFL07-15-I05  expressed protein | | | | | | |
|  |  | RAFL05-10-H10 | At3g62550 / expressed protein | |  |  |  |  |  | | --- | --- | --- | --- | --- | |  |  |  |  |  | | At3g62550 ,RAFL05-10-H10  universal stress protein (USP) family protein similar to ER6 protein [Lycopersicon esculentum] GI:5669654; contains Pfam profile PF00582: universal stress protein family | | | | | | |
|  |  | RAFL08-16-A20 | At5g19540 / expressed protein | |  |  |  |  |  | | --- | --- | --- | --- | --- | |  |  |  |  |  | | At5g19540 ,RAFL08-16-A20  expressed protein | | | | | | |
|  |  | RAFL06-16-E03 | At4g20260 / endomembrane-associated protein | |  |  |  |  |  | | --- | --- | --- | --- | --- | |  |  |  |  |  | | At4g20260 ,RAFL06-16-E03  DREPP plasma membrane polypeptide family protein contains Pfam profile: PF05558 DREPP plasma membrane polypeptide | | | | | | |
|  |  | RAFL05-04-A24 | At1g80180 / expressed protein | |  |  |  |  |  | | --- | --- | --- | --- | --- | |  |  |  |  |  | | RAFL05-04-A24 ,At1g80180  expressed protein | | | | | | |
|  |  | RAFL03-08-F18 | At1g65720 / expressed protein | |  |  |  |  |  | | --- | --- | --- | --- | --- | |  |  |  |  |  | | At1g65720 ,RAFL03-08-F18  expressed protein | | | | | | |
|  |  | RAFL08-16-H21 | At5g24460 / expressed protein | |  |  |  |  |  | | --- | --- | --- | --- | --- | |  |  |  |  |  | | RAFL08-16-H21 ,At5g24460  expressed protein | | | | | | |
|  |  | RAFL05-02-K03 | At3g26740 / light regulated protein -related | |  |  |  |  |  | | --- | --- | --- | --- | --- | |  |  |  |  |  | | At3g26740 ,RAFL05-02-K03  light responsive protein-related similar to light regulated protein precursor SP:Q03200 [Oryza sativa] (Plant Mol. Biol. 22 (1), 165-170 (1993)), ccr protein GB:S52663 [Citrus X paradisi] (Plant Mol. Biol. 26 (1), 165-173 (1994)) | | | | | | |
|  | Cluster:7-0 | |  |  | 61 | 186 | 843 | 3573 | 0.038244277 | 1.2620611 | 33 |
|  |  | RAFL06-07-G22 | At5g19860 / expressed protein | |  |  |  |  |  | | --- | --- | --- | --- | --- | |  |  |  |  |  | | RAFL06-07-G22 ,At5g19860  expressed protein contains Pfam profile PF04398: Protein of unknown function, DUF538 | | | | | | |
|  |  | RAFL04-09-C05 | At3g62770 / expressed protein | |  |  |  |  |  | | --- | --- | --- | --- | --- | |  |  |  |  |  | | RAFL04-09-C05 ,At3g62770  transport protein-related weak similarity to Gsa12p [Pichia pastoris] GI:18307769; contains 1 WD-40 repeat (PF00400); putative proteins - different species | | | | | | |
|  |  | RAFL09-11-E17 | At4g22220 / iron-sulfur cluster assembly complex protein, putative (ISCU1) | |  |  |  |  |  | | --- | --- | --- | --- | --- | |  |  |  |  |  | | At4g22220 ,RAFL09-11-E17  iron-sulfur cluster assembly complex protein, putative similar to iron-sulfur cluster assembly complex ISCU1 (GI:11545705) [Homo sapiens]; nifU protein homolog YPL135w (GI:15619823) [Saccharomyces cerevisiae] PIR2:S69049 | | | | | | |
|  |  | RAFL05-03-M05 | At5g22120 / expressed protein | |  |  |  |  |  | | --- | --- | --- | --- | --- | |  |  |  |  |  | | RAFL05-03-M05 ,At5g22120  expressed protein | | | | | | |
|  |  | RAFL11-09-C14 | At4g02940 / oxidoreductase, 2OG-Fe(II) oxygenase family | |  |  |  |  |  | | --- | --- | --- | --- | --- | |  |  |  |  |  | | At4g02940 ,RAFL11-09-C14  oxidoreductase, 2OG-Fe(II) oxygenase family protein similar to A. thaliana hypothetical protein T13L16.2, GenBank accession number 2708738; contains Pfam domain PF03171 2OG-Fe(II) oxygenase superfamily | | | | | | |
|  |  | RAFL11-09-I15 | At5g11390 / expressed protein | |  |  |  |  |  | | --- | --- | --- | --- | --- | |  |  |  |  |  | | RAFL11-09-I15 ,At5g11390  expressed protein | | | | | | |
|  |  | RAFL05-21-P18 | At1g36980 / expressed protein | |  |  |  |  |  | | --- | --- | --- | --- | --- | |  |  |  |  |  | | At1g36980 ,RAFL05-21-P18  expressed protein | | | | | | |
|  |  | RAFL05-12-M12 | At1g10150 / expressed protein | |  |  |  |  |  | | --- | --- | --- | --- | --- | |  |  |  |  |  | | RAFL05-12-M12 ,At1g10150  expressed protein similar to ESTs gb|T20511, gb|T45308, gb|H36493, and gb|AA651176 | | | | | | |
|  |  | RAFL08-08-P16 | At4g29790 / expressed protein | |  |  |  |  |  | | --- | --- | --- | --- | --- | |  |  |  |  |  | | RAFL08-08-P16 ,At4g29790  expressed protein | | | | | | |
|  |  | RAFL09-17-N07 | At1g55680 / WD-40 repeat protein family | |  |  |  |  |  | | --- | --- | --- | --- | --- | |  |  |  |  |  | | At1g55680 ,RAFL09-17-N07  WD-40 repeat family protein contains 2 (1 significant) WD-40 repeats (PF0400); similar to Trp-Asp repeat protein (PIR:T40094) [Schizosaccharomyces] | | | | | | |
|  |  | RAFL05-12-F05 | At3g59640 / glycine-rich protein | |  |  |  |  |  | | --- | --- | --- | --- | --- | |  |  |  |  |  | | RAFL05-12-F05 ,At3g59640  glycine-rich protein | | | | | | |
|  |  | RAFL05-12-O20 | At5g24450 / expressed protein | |  |  |  |  |  | | --- | --- | --- | --- | --- | |  |  |  |  |  | | RAFL05-12-O20 ,At5g24450  transcription factor-related low similarity to transcription factor IIIC63 [Homo sapiens] GI:5281316 | | | | | | |
|  |  | RAFL04-16-K10 | At3g09085 / expressed protein | |  |  |  |  |  | | --- | --- | --- | --- | --- | |  |  |  |  |  | | RAFL04-16-K10 ,At3g09085  expressed protein | | | | | | |
|  |  | RAFL03-08-G12 | At5g53330 / proline-rich cell wall protein-related | |  |  |  |  |  | | --- | --- | --- | --- | --- | |  |  |  |  |  | | RAFL03-08-G12 ,At5g53330  expressed protein | | | | | | |
|  |  | RAFL05-17-D02 | At2g43210 / expressed protein | |  |  |  |  |  | | --- | --- | --- | --- | --- | |  |  |  |  |  | | At2g43210 ,RAFL05-17-D02  UBX domain-containing protein contains Pfam profile PF00789: UBX domain | | | | | | |
|  |  | RAFL07-11-H01 | At5g66930 / expressed protein | |  |  |  |  |  | | --- | --- | --- | --- | --- | |  |  |  |  |  | | RAFL07-11-H01 ,At5g66930  expressed protein similar to unknown protein (pir||T38383) | | | | | | |
|  |  | RAFL05-17-J22 | At5g16110 / expressed protein | |  |  |  |  |  | | --- | --- | --- | --- | --- | |  |  |  |  |  | | At5g16110 ,RAFL05-17-J22  expressed protein hypothetical protein T26J14.6 - Arabidopsis thaliana, EMBL:AC011915 | | | | | | |
|  |  | RAFL11-03-E16 | At4g23870 / expressed protein | |  |  |  |  |  | | --- | --- | --- | --- | --- | |  |  |  |  |  | | RAFL11-03-E16 ,At4g23870  expressed protein predicted proteins, Arabidopsis thaliana | | | | | | |
|  |  | RAFL08-19-K02 | At5g42810 / expressed protein | |  |  |  |  |  | | --- | --- | --- | --- | --- | |  |  |  |  |  | | At5g42810 ,RAFL08-19-K02  expressed protein similar to unknown protein (pir||T26506) | | | | | | |
|  |  | RAFL05-16-L05 | At1g67340 / F-box protein family | |  |  |  |  |  | | --- | --- | --- | --- | --- | |  |  |  |  |  | | RAFL05-16-L05 ,At1g67340  zinc finger (MYND type) family protein / F-box family protein | | | | | | |
|  |  | RAFL05-21-F11 | At5g35320 / expressed protein | |  |  |  |  |  | | --- | --- | --- | --- | --- | |  |  |  |  |  | | RAFL05-21-F11 ,At5g35320  expressed protein | | | | | | |
|  |  | RAFL04-14-I08 | At5g65910 / expressed protein | |  |  |  |  |  | | --- | --- | --- | --- | --- | |  |  |  |  |  | | At5g65910 ,RAFL04-14-I08  BSD domain-containing protein contains Pfam profile PF03909: BSD domain | | | | | | |
|  |  | RAFL09-12-K24 | At1g27150 / expressed protein | |  |  |  |  |  | | --- | --- | --- | --- | --- | |  |  |  |  |  | | At1g27150 ,RAFL09-12-K24  expressed protein | | | | | | |
|  |  | RAFL11-09-C22 | At4g28240 / wound induced protein -related | |  |  |  |  |  | | --- | --- | --- | --- | --- | |  |  |  |  |  | | At4g28240 ,RAFL11-09-C22  wound-responsive protein-related wound-induced protein - tomato (fragment), PIR2:S19773 | | | | | | |
|  |  | RAFL03-05-E08 | At3g53990 / expressed protein | |  |  |  |  |  | | --- | --- | --- | --- | --- | |  |  |  |  |  | | At3g53990 ,RAFL03-05-E08  universal stress protein (USP) family protein contains Pfam PF00582: universal stress protein family | | | | | | |
|  |  | RAFL09-09-M04 | At5g19860 / expressed protein | |  |  |  |  |  | | --- | --- | --- | --- | --- | |  |  |  |  |  | | RAFL09-09-M04 ,At5g19860  expressed protein contains Pfam profile PF04398: Protein of unknown function, DUF538 | | | | | | |
|  |  | RAFL05-13-P19 | At5g06280 / expressed protein | |  |  |  |  |  | | --- | --- | --- | --- | --- | |  |  |  |  |  | | At5g06280 ,RAFL05-13-P19  expressed protein similar to unknown protein (pir||T08447) | | | | | | |
|  |  | RAFL04-14-D12 | At5g03230 / expressed protein | |  |  |  |  |  | | --- | --- | --- | --- | --- | |  |  |  |  |  | | At5g03230 ,RAFL04-14-D12  expressed protein contains Pfam profile PF04520: Protein of unknown function, DUF584 | | | | | | |
|  |  | RAFL06-15-J24 | At4g21110 / G10 - like protein | |  |  |  |  |  | | --- | --- | --- | --- | --- | |  |  |  |  |  | | RAFL06-15-J24 ,At4g21110  G10 family protein contains Pfam profile: PF01125 G10 protein | | | | | | |
|  |  | RAFL04-14-A21 | At2g17200 / ubiquitin protein -related | |  |  |  |  |  | | --- | --- | --- | --- | --- | |  |  |  |  |  | | At2g17200 ,RAFL04-14-A21  ubiquitin family protein weak similarity to PLIC-2 (ubiquitin-like type II) [Homo sapiens] GI:9937505; contains Pfam profiles PF00240: Ubiquitin family, PF00627: UBA/TS-N domain | | | | | | |
|  |  | RAFL06-15-K04 | At5g17550 / expressed protein | |  |  |  |  |  | | --- | --- | --- | --- | --- | |  |  |  |  |  | | At5g17550 ,RAFL06-15-K04  peroxisomal protein PEX19 family protein contains Pfam profile: PF04614 Pex19 protein family | | | | | | |
|  |  | RAFL02-10-G21 | At3g53990 / expressed protein | |  |  |  |  |  | | --- | --- | --- | --- | --- | |  |  |  |  |  | | At3g53990 ,RAFL02-10-G21  universal stress protein (USP) family protein contains Pfam PF00582: universal stress protein family | | | | | | |
|  |  | RAFL05-05-O14 | At3g11780 / expressed protein | |  |  |  |  |  | | --- | --- | --- | --- | --- | |  |  |  |  |  | | RAFL05-05-O14 ,At3g11780  MD-2-related lipid recognition domain-containing protein / ML domain-containing protein weak similarity to phosphatidylglycerol/phosphatidylinositol transfer protein [Aspergillus oryzae] GI:10178615; contains Pfam profile PF02221: ML domain | | | | | | |
|  |  | RAFL06-07-O17 | At5g27280 / expressed protein | |  |  |  |  |  | | --- | --- | --- | --- | --- | |  |  |  |  |  | | At5g27280 ,RAFL06-07-O17  zinc finger (DNL type) family protein contains Pfam profile PF05180: DNL zinc finger | | | | | | |
|  |  | RAFL09-16-M08 | At1g70160 / expressed protein | |  |  |  |  |  | | --- | --- | --- | --- | --- | |  |  |  |  |  | | RAFL09-16-M08 ,At1g70160  expressed protein similar to hypothetical protein GI:4455225 from [Arabidopsis thaliana] | | | | | | |
|  |  | RAFL09-06-L15 | At3g60300 / expressed protein | |  |  |  |  |  | | --- | --- | --- | --- | --- | |  |  |  |  |  | | RAFL09-06-L15 ,At3g60300  RWD domain-containing protein contains weak similarity to RING finger protein 25 (RING finger protein AO7) (Swiss-Prot:Q9QZR0) [Mus musculus] | | | | | | |
|  |  | RAFL06-09-D08 | At3g59940 / Kelch repeat containing F-box protein family | |  |  |  |  |  | | --- | --- | --- | --- | --- | |  |  |  |  |  | | RAFL06-09-D08 ,At3g59940  kelch repeat-containing F-box family protein contains Pfam profiles PF01344: Kelch motif, PF00646: F-box domain | | | | | | |
|  |  | RAFL07-12-E04 | At1g03380 / WD-40 repeat protein-related | |  |  |  |  |  | | --- | --- | --- | --- | --- | |  |  |  |  |  | | RAFL07-12-E04 ,At1g03380  expressed protein | | | | | | |
|  |  | RAFL03-04-J10 | At3g07560 / glycine-rich protein | |  |  |  |  |  | | --- | --- | --- | --- | --- | |  |  |  |  |  | | At3g07560 ,RAFL03-04-J10  glycine-rich protein | | | | | | |
|  |  | RAFL05-09-J02 | At5g66052 / expressed protein | |  |  |  |  |  | | --- | --- | --- | --- | --- | |  |  |  |  |  | | At5g66052 ,RAFL05-09-J02  expressed protein | | | | | | |
|  |  | RAFL05-14-I02 | At3g10020 / expressed protein | |  |  |  |  |  | | --- | --- | --- | --- | --- | |  |  |  |  |  | | RAFL05-14-I02 ,At3g10020  expressed protein | | | | | | |
|  |  | RAFL05-21-P21 | At4g29330 / expressed protein | |  |  |  |  |  | | --- | --- | --- | --- | --- | |  |  |  |  |  | | At4g29330 ,RAFL05-21-P21  Der1-like family protein / degradation in the ER-like family protein contains Pfam profile: PF04511 Der1-like family | | | | | | |
|  |  | RAFL05-16-K02 | At5g64170 / expressed protein | |  |  |  |  |  | | --- | --- | --- | --- | --- | |  |  |  |  |  | | At5g64170 ,RAFL05-16-K02  dentin sialophosphoprotein-related contains weak similarity to Swiss-Prot:Q9NZW4 dentin sialophosphoprotein precursor [Homo sapiens] | | | | | | |
|  |  | RAFL05-09-M04 | At1g68980 / pentatricopeptide (PPR) repeat-containing protein | |  |  |  |  |  | | --- | --- | --- | --- | --- | |  |  |  |  |  | | RAFL05-09-M04 ,At1g68980  pentatricopeptide (PPR) repeat-containing protein contains Pfam profile PF01535: PPR repeat | | | | | | |
|  |  | RAFL05-03-B22 | At4g34180 / expressed protein | |  |  |  |  |  | | --- | --- | --- | --- | --- | |  |  |  |  |  | | At4g34180 ,RAFL05-03-B22  cyclase family protein contains Pfam profile: PF04199 putative cyclase | | | | | | |
|  |  | RAFL04-14-A11 | At5g64400 / expressed protein | |  |  |  |  |  | | --- | --- | --- | --- | --- | |  |  |  |  |  | | At5g64400 ,RAFL04-14-A11  expressed protein contains Pfam domain, PF04933: Protein of unknown function (DUF657) | | | | | | |
|  |  | RAFL06-07-I21 | At3g63210 / senescence-associated protein SAG102 | |  |  |  |  |  | | --- | --- | --- | --- | --- | |  |  |  |  |  | | RAFL06-07-I21 ,At3g63210  expressed protein identical to senescence-associated protein SAG102 (GI::22331931) [Arabidopsis thaliana] (unpublished); contains Pfam profile PF04570: Protein of unknown function (DUF581) | | | | | | |
|  |  | RAFL09-16-O10 | At1g50020 / tubulin alpha-6 chain -related | |  |  |  |  |  | | --- | --- | --- | --- | --- | |  |  |  |  |  | | At1g50020 ,RAFL09-16-O10  expressed protein | | | | | | |
|  |  | RAFL04-19-H05 | At1g78790 / expressed protein | |  |  |  |  |  | | --- | --- | --- | --- | --- | |  |  |  |  |  | | At1g78790 ,RAFL04-19-H05  expressed protein | | | | | | |
|  |  | RAFL05-12-G13 | At1g77180 / expressed protein | |  |  |  |  |  | | --- | --- | --- | --- | --- | |  |  |  |  |  | | RAFL05-12-G13 ,At1g77180  chromatin protein family contains Pfam domain, PF02731: SKIP/SNW domain found in chromatin proteins. | | | | | | |
|  |  | RAFL04-09-B05 | At1g09570 / phytochrome A (PHYA) | |  |  |  |  |  | | --- | --- | --- | --- | --- | |  |  |  |  |  | | At1g09570 ,RAFL04-09-B05  phytochrome A (PHYA) identical to SP|P14712 Phytochrome A {Arabidopsis thaliana} | | | | | | |
|  |  | RAFL08-14-C01 | At5g45410 / expressed protein | |  |  |  |  |  | | --- | --- | --- | --- | --- | |  |  |  |  |  | | RAFL08-14-C01 ,At5g45410  expressed protein similar to unknown protein (pir||T05524) | | | | | | |
|  |  | RAFL05-07-I21 | At3g07350 / expressed protein | |  |  |  |  |  | | --- | --- | --- | --- | --- | |  |  |  |  |  | | RAFL05-07-I21 ,At3g07350  expressed protein contains Pfam profile PF04720: Protein of unknown function (DUF506) | | | | | | |
|  |  | RAFL07-17-O09 | At3g51250 / expressed protein | |  |  |  |  |  | | --- | --- | --- | --- | --- | |  |  |  |  |  | | RAFL07-17-O09 ,At3g51250  senescence/dehydration-associated protein-related similar to senescence-associated protein 12 [Hemerocallis hybrid cultivar] gi|3551958|gb|AAC34857; similar to early-responsive to dehydration stress ERD7 protein [Arabidopsis thaliana] gi|15320412|dbj|BAB63916 | | | | | | |
|  |  | RAFL06-07-N14 | At2g05520 / glycine-rich protein (GRP) | |  |  |  |  |  | | --- | --- | --- | --- | --- | |  |  |  |  |  | | RAFL06-07-N14 ,At2g05520  glycine-rich protein (GRP) identical to glycine-rich protein; atGRP (GI:259447) [Arabidopsis thaliana] | | | | | | |
|  |  | RAFL11-01-G07 | At2g48070 / expressed protein | |  |  |  |  |  | | --- | --- | --- | --- | --- | |  |  |  |  |  | | RAFL11-01-G07 ,At2g48070  expressed protein | | | | | | |
|  |  | RAFL05-17-L24 | At2g20740 / expressed protein | |  |  |  |  |  | | --- | --- | --- | --- | --- | |  |  |  |  |  | | RAFL05-17-L24 ,At2g20740  expressed protein | | | | | | |
|  |  | RAFL08-15-K23 | At1g23960 / expressed protein | |  |  |  |  |  | | --- | --- | --- | --- | --- | |  |  |  |  |  | | RAFL08-15-K23 ,At1g23960  expressed protein contains Pfam profile PF04776: Protein of unknown function (DUF626) | | | | | | |
|  |  | RAFL08-10-D20 | At3g13340 / WD-40 repeat protein family | |  |  |  |  |  | | --- | --- | --- | --- | --- | |  |  |  |  |  | | At3g13340 ,RAFL08-10-D20  WD-40 repeat family protein contains Pfam profile: PF00400 WD domain, G-beta repeat (3 copies, 1 significant); similar to Trp-Asp repeat protein (PIR:T40094) [Schizosaccharomyces] | | | | | | |
|  |  | RAFL07-18-C02 | At1g16810 / expressed protein | |  |  |  |  |  | | --- | --- | --- | --- | --- | |  |  |  |  |  | | At1g16810 ,RAFL07-18-C02  expressed protein | | | | | | |
|  |  | RAFL06-13-A22 | At4g04800 / expressed protein | |  |  |  |  |  | | --- | --- | --- | --- | --- | |  |  |  |  |  | | At4g04800 ,RAFL06-13-A22  methionine sulfoxide reductase domain-containing protein / SeIR domain-containing protein low similarity to pilin-like transcription factor [Homo sapiens] GI:5059062, SP|P14930 Peptide methionine sulfoxide reductase msrA/msrB (EC 1.8.4.6) {Neisseria gonorrhoeae}; contains Pfam profile PF01641: SelR domain | | | | | | |
| GO:0009628 | | | response to abiotic stimulus |  | A | B | C | D | P | P' | N |
|  | Cluster:2-1 | |  |  | 4 | 240 | 12 | 4407 | 0.008082359 | 0.08082359 | 10 |
|  |  | RAFL09-09-I19 | At1g23310 / alanine aminotransferase -related | |  |  |  |  |  | | --- | --- | --- | --- | --- | |  |  |  |  |  | | RAFL09-09-I19 ,At1g23310  glutamate:glyoxylate aminotransferase 1 (GGT1) identical to glutamate:glyoxylate aminotransferase 1 [Arabidopsis thaliana] GI:24461827; similar to alanine aminotransferase GI:4730884 from [Oryza sativa]; contains Pfam profile PF00155: aminotransferase, classes I and II | | | | | | |
|  |  | RAFL08-08-P10 | At5g65110 / acyl-CoA oxidase (gb|AAC13497.1) | |  |  |  |  |  | | --- | --- | --- | --- | --- | |  |  |  |  |  | | At5g65110 ,RAFL08-08-P10  acyl-CoA oxidase (ACX2) identical to acyl-CoA oxidase [Arabidopsis thaliana] GI:3044212 | | | | | | |
|  |  | RAFL09-16-K01 | At1g23310 / alanine aminotransferase -related | |  |  |  |  |  | | --- | --- | --- | --- | --- | |  |  |  |  |  | | RAFL09-16-K01 ,At1g23310  glutamate:glyoxylate aminotransferase 1 (GGT1) identical to glutamate:glyoxylate aminotransferase 1 [Arabidopsis thaliana] GI:24461827; similar to alanine aminotransferase GI:4730884 from [Oryza sativa]; contains Pfam profile PF00155: aminotransferase, classes I and II | | | | | | |
|  |  | RAFL05-07-N11 | At1g70580 / alanine aminotransferase, putative | |  |  |  |  |  | | --- | --- | --- | --- | --- | |  |  |  |  |  | | RAFL05-07-N11 ,At1g70580  glutamate:glyoxylate aminotransferase 2 (GGT2) identical to glutamate:glyoxylate aminotransferase 2 [Arabidopsis thaliana] GI:24461829; similar to alanine aminotransferase from Panicum miliaceum [SP|P34106], GI:4730884 from Oryza sativa; contains Pfam profile PF00155: aminotransferase, classes I and II | | | | | | |
|  | Cluster:3-0 | |  |  | 3 | 230 | 13 | 4417 | 0.04257905 | 0.4257905 | 10 |
|  |  | RAFL11-02-M18 | At3g03490 / expressed protein | |  |  |  |  |  | | --- | --- | --- | --- | --- | |  |  |  |  |  | | At3g03490 ,RAFL11-02-M18  peroxisomal protein PEX19 family protein contains Pfam profile: PF04614 Pex19 protein family | | | | | | |
|  |  | RAFL05-18-J16 | At2g13360 / alanine-glyoxylate aminotransferase | |  |  |  |  |  | | --- | --- | --- | --- | --- | |  |  |  |  |  | | RAFL05-18-J16 ,At2g13360  serine-glyoxylate aminotransferase-related similar to serine-glyoxylate aminotransferase (GI:21535798)[Methylobacterium dichloromethanicum; contains TIGRFAM TIGR01364: phosphoserine aminotransferase; contains Pfam PF00266: aminotransferase, class V] | | | | | | |
|  |  | RAFL05-10-L06 | At2g13360 / alanine-glyoxylate aminotransferase | |  |  |  |  |  | | --- | --- | --- | --- | --- | |  |  |  |  |  | | At2g13360 ,RAFL05-10-L06  serine-glyoxylate aminotransferase-related similar to serine-glyoxylate aminotransferase (GI:21535798)[Methylobacterium dichloromethanicum; contains TIGRFAM TIGR01364: phosphoserine aminotransferase; contains Pfam PF00266: aminotransferase, class V] | | | | | | |
| GO:0009058 | | | biosynthesis |  | A | B | C | D | P | P' | N |
|  | Cluster:2-1 | |  |  | 68 | 176 | 295 | 4124 | 1.8855602E-22 | 4.9024567E-21 | 26 |
|  |  | RAFL09-15-G07 | At3g62250 / ubiquitin extension protein (UBQ5)/40S ribosomal protein S27A (RPS27aC) | |  |  |  |  |  | | --- | --- | --- | --- | --- | |  |  |  |  |  | | RAFL09-15-G07 ,At3g62250  ubiquitin extension protein 5 (UBQ5) / 40S ribosomal protein S27A (RPS27aC) identical to GI:166933, GI:166934 | | | | | | |
|  |  | RAFL07-11-J16 | At3g15950 / expressed protein | |  |  |  |  |  | | --- | --- | --- | --- | --- | |  |  |  |  |  | | RAFL07-11-J16 ,At3g15950  DNA topoisomerase-related similar to DNA topoisomerase IV subunit A (GI:26454107) [Mycoplasma penetrans] | | | | | | |
|  |  | RAFL08-15-K17 | At5g07090 / 40S ribosomal protein S4 (RPS4B) | |  |  |  |  |  | | --- | --- | --- | --- | --- | |  |  |  |  |  | | At5g07090 ,RAFL08-15-K17  40S ribosomal protein S4 (RPS4B) | | | | | | |
|  |  | RAFL11-10-K08 | At3g22230 / 60S ribosomal protein L27 (RPL27B) | |  |  |  |  |  | | --- | --- | --- | --- | --- | |  |  |  |  |  | | At3g22230 ,RAFL11-10-K08  60S ribosomal protein L27 (RPL27B) similar to 60S RIBOSOMAL PROTEIN L27 GB:P41101 from [Solanum tuberosum] | | | | | | |
|  |  | RAFL05-17-M03 | At5g56710 / 60S ribosomal protein L31 (RPL31C) | |  |  |  |  |  | | --- | --- | --- | --- | --- | |  |  |  |  |  | | At5g56710 ,RAFL05-17-M03  60S ribosomal protein L31 (RPL31C) | | | | | | |
|  |  | RAFL05-17-L17 | At3g55280 / 60S ribosomal protein L23A (RPL23aB) | |  |  |  |  |  | | --- | --- | --- | --- | --- | |  |  |  |  |  | | RAFL05-17-L17 ,At3g55280  60S ribosomal protein L23A (RPL23aB) various ribosomal L23a proteins | | | | | | |
|  |  | RAFL09-12-B05 | At5g02870 / 60S ribosomal protein L4/L1 (RPL4D) | |  |  |  |  |  | | --- | --- | --- | --- | --- | |  |  |  |  |  | | At5g02870 ,RAFL09-12-B05  60S ribosomal protein L4/L1 (RPL4D) 60S roibosomal protein L4, Arabidopsis thaliana, EMBL:CAA79104 | | | | | | |
|  |  | RAFL02-02-F05 | At5g58420 / 40S ribosomal protein S4 (RPS4D) | |  |  |  |  |  | | --- | --- | --- | --- | --- | |  |  |  |  |  | | RAFL02-02-F05 ,At5g58420  40S ribosomal protein S4 (RPS4D) ribosomal protein S4, Arabidopsis thaliana, PIR:T48480 | | | | | | |
|  |  | RAFL07-13-J18 | At2g37270 / 40S ribosomal protein S5 (RPS5A) | |  |  |  |  |  | | --- | --- | --- | --- | --- | |  |  |  |  |  | | At2g37270 ,RAFL07-13-J18  40S ribosomal protein S5 (RPS5A) identical to GP:3043428 | | | | | | |
|  |  | RAFL06-14-I03 | At3g60770 / 40S ribosomal protein S13 (RPS13A) | |  |  |  |  |  | | --- | --- | --- | --- | --- | |  |  |  |  |  | | RAFL06-14-I03 ,At3g60770  40S ribosomal protein S13 (RPS13A) AtRPS13A mRNA for cytoplasmic ribosomal protein S13, Arabidopsis thaliana,AB031739 | | | | | | |
|  |  | RAFL05-08-H10 | At4g16720 / 60S ribosomal protein L15 (RPL15A) | |  |  |  |  |  | | --- | --- | --- | --- | --- | |  |  |  |  |  | | RAFL05-08-H10 ,At4g16720  60S ribosomal protein L15 (RPL15A) | | | | | | |
|  |  | RAFL05-17-F03 | At2g32060 / 40S ribosomal protein S12 (RPS12C) | |  |  |  |  |  | | --- | --- | --- | --- | --- | |  |  |  |  |  | | RAFL05-17-F03 ,At2g32060  40S ribosomal protein S12 (RPS12C) | | | | | | |
|  |  | RAFL06-09-H09 | At3g48930 / 40S ribosomal protein S11 (RPS11A) | |  |  |  |  |  | | --- | --- | --- | --- | --- | |  |  |  |  |  | | At3g48930 ,RAFL06-09-H09  40S ribosomal protein S11 (RPS11A) | | | | | | |
|  |  | RAFL06-15-A09 | At1g59359 / 40S ribosomal protein S2 (RPS2B) | |  |  |  |  |  | | --- | --- | --- | --- | --- | |  |  |  |  |  | | RAFL06-15-A09 ,At1g59359  40S ribosomal protein S2 (RPS2B) similar to ribosomal protein S2 GI:430711 from [Drosophila melanogaster] | | | | | | |
|  |  | RAFL06-08-I04 | At1g07920 / elongation factor 1-alpha (EF-1-alpha) | |  |  |  |  |  | | --- | --- | --- | --- | --- | |  |  |  |  |  | | RAFL06-08-I04 ,At1g07920  elongation factor 1-alpha / EF-1-alpha identical to GB:CAA34456 from [Arabidopsis thaliana] (Plant Mol. Biol. 14 (1), 107-110 (1990)) | | | | | | |
|  |  | RAFL05-07-H16 | At3g53870 / 40S ribosomal protein S3 (RPS3B) | |  |  |  |  |  | | --- | --- | --- | --- | --- | |  |  |  |  |  | | At3g53870 ,RAFL05-07-H16  40S ribosomal protein S3 (RPS3B) ribosomal protein S3a - Xenopus laevis, PIR:R3XL3A | | | | | | |
|  |  | RAFL05-02-G09 | At5g41520 / expressed protein | |  |  |  |  |  | | --- | --- | --- | --- | --- | |  |  |  |  |  | | RAFL05-02-G09 ,At5g41520  40S ribosomal protein S10 (RPS10B) contains similarity to 40S ribosomal protein S10 | | | | | | |
|  |  | RAFL07-15-M07 | At1g04480 / 60S ribosomal protein L23 (RPL23A) | |  |  |  |  |  | | --- | --- | --- | --- | --- | |  |  |  |  |  | | At1g04480 ,RAFL07-15-M07  60S ribosomal protein L23 (RPL23A) identical to GB:AAB80655 | | | | | | |
|  |  | RAFL05-14-D21 | At1g18540 / 60S ribosomal protein L6 (RPL6A) | |  |  |  |  |  | | --- | --- | --- | --- | --- | |  |  |  |  |  | | At1g18540 ,RAFL05-14-D21  60S ribosomal protein L6 (RPL6A) similar to 60S ribosomal protein L6 GI:7208784 from [Cicer arietinum] | | | | | | |
|  |  | RAFL08-11-K22 | At1g57660 / 60S ribosomal protein L21 (RPL21E) | |  |  |  |  |  | | --- | --- | --- | --- | --- | |  |  |  |  |  | | At1g57660 ,RAFL08-11-K22  60S ribosomal protein L21 (RPL21E) similar to 60S ribosomal protein L21 GB:Q43291 GI:2851508 from [Arabidopsis thaliana] | | | | | | |
|  |  | RAFL11-12-H04 | At3g25520 / 60S ribosomal protein L5 (RPL5A) | |  |  |  |  |  | | --- | --- | --- | --- | --- | |  |  |  |  |  | | At3g25520 ,RAFL11-12-H04  60S ribosomal protein L5 similar to 60S ribosomal protein L5 GB:P49625 from [Oryza sativa] | | | | | | |
|  |  | RAFL04-18-N22 | At2g44120 / 60S ribosomal protein L7 (RPL7C) | |  |  |  |  |  | | --- | --- | --- | --- | --- | |  |  |  |  |  | | RAFL04-18-N22 ,At2g44120  60S ribosomal protein L7 (RPL7C) | | | | | | |
|  |  | RAFL09-07-D04 | At3g22230 / 60S ribosomal protein L27 (RPL27B) | |  |  |  |  |  | | --- | --- | --- | --- | --- | |  |  |  |  |  | | At3g22230 ,RAFL09-07-D04  60S ribosomal protein L27 (RPL27B) similar to 60S RIBOSOMAL PROTEIN L27 GB:P41101 from [Solanum tuberosum] | | | | | | |
|  |  | RAFL04-10-F11 | At5g59850 / 40S ribosomal protein S15A (RPS15aF) | |  |  |  |  |  | | --- | --- | --- | --- | --- | |  |  |  |  |  | | At5g59850 ,RAFL04-10-F11  40S ribosomal protein S15A (RPS15aF) cytoplasmic ribosomal protein S15a, Arabidopsis thaliana, EMBL:ATAF1412 | | | | | | |
|  |  | RAFL04-18-N10 | At1g07770 / 40S ribosomal protein S15A (RPS15aA) | |  |  |  |  |  | | --- | --- | --- | --- | --- | |  |  |  |  |  | | RAFL04-18-N10 ,At1g07770  40S ribosomal protein S15A (RPS15aA) identical to GB:AAA61608 from [Arabidopsis thaliana] (Plant Physiol. 106 (1), 401-402 (1994)) | | | | | | |
|  |  | RAFL05-18-M20 | At2g19730 / 60S ribosomal protein L28 (RPL28A) | |  |  |  |  |  | | --- | --- | --- | --- | --- | |  |  |  |  |  | | At2g19730 ,RAFL05-18-M20  60S ribosomal protein L28 (RPL28A) | | | | | | |
|  |  | RAFL07-08-E24 | At5g27850 / 60S ribosomal protein L18 (RPL18C) | |  |  |  |  |  | | --- | --- | --- | --- | --- | |  |  |  |  |  | | RAFL07-08-E24 ,At5g27850  60S ribosomal protein L18 (RPL18C) 60S ribosomal protein L18, Arabidopsis thaliana, SWISSPROT:RL18\_ARATH | | | | | | |
|  |  | RAFL11-07-B21 | At3g25520 / 60S ribosomal protein L5 (RPL5A) | |  |  |  |  |  | | --- | --- | --- | --- | --- | |  |  |  |  |  | | At3g25520 ,RAFL11-07-B21  60S ribosomal protein L5 similar to 60S ribosomal protein L5 GB:P49625 from [Oryza sativa] | | | | | | |
|  |  | RAFL04-20-F03 | At5g39740 / 60S ribosomal protein L5 (RPL5B) | |  |  |  |  |  | | --- | --- | --- | --- | --- | |  |  |  |  |  | | RAFL04-20-F03 ,At5g39740  60S ribosomal protein L5 (RPL5B) ribosomal protein L5, rice | | | | | | |
|  |  | RAFL11-05-B21 | At3g54210 / ribosomal protein L17 -related protein | |  |  |  |  |  | | --- | --- | --- | --- | --- | |  |  |  |  |  | | At3g54210 ,RAFL11-05-B21  ribosomal protein L17 family protein contains Pfam profile: PF01196 ribosomal protein L17 | | | | | | |
|  |  | RAFL05-02-K09 | At5g22440 / 60S ribosomal protein L10A (RPL10aC) | |  |  |  |  |  | | --- | --- | --- | --- | --- | |  |  |  |  |  | | RAFL05-02-K09 ,At5g22440  60S ribosomal protein L10A (RPL10aC) | | | | | | |
|  |  | RAFL02-03-G08 | At4g18730 / 60S ribosomal protein L11 (RPL11C) | |  |  |  |  |  | | --- | --- | --- | --- | --- | |  |  |  |  |  | | RAFL02-03-G08 ,At4g18730  60S ribosomal protein L11 (RPL11C) | | | | | | |
|  |  | RAFL05-05-M24 | At5g02960 / 40S ribosomal protein S23 (RPS23B) | |  |  |  |  |  | | --- | --- | --- | --- | --- | |  |  |  |  |  | | At5g02960 ,RAFL05-05-M24  40S ribosomal protein S23 (RPS23B) ribosomal protein S23, Fragaria x ananassa, PIR:S56673 | | | | | | |
|  |  | RAFL07-13-J05 | At1g07920 / elongation factor 1-alpha (EF-1-alpha) | |  |  |  |  |  | | --- | --- | --- | --- | --- | |  |  |  |  |  | | RAFL07-13-J05 ,At1g07920  elongation factor 1-alpha / EF-1-alpha identical to GB:CAA34456 from [Arabidopsis thaliana] (Plant Mol. Biol. 14 (1), 107-110 (1990)) | | | | | | |
|  |  | RAFL05-18-P15 | At1g04480 / 60S ribosomal protein L23 (RPL23A) | |  |  |  |  |  | | --- | --- | --- | --- | --- | |  |  |  |  |  | | At1g04480 ,RAFL05-18-P15  60S ribosomal protein L23 (RPL23A) identical to GB:AAB80655 | | | | | | |
|  |  | RAFL04-09-J06 | At1g70600 / 60S ribosomal protein L27A (RPL27aC) | |  |  |  |  |  | | --- | --- | --- | --- | --- | |  |  |  |  |  | | At1g70600 ,RAFL04-09-J06  60S ribosomal protein L27A (RPL27aC) identical to 60S ribosomal protein L27A GB:P49637 [Arabidopsis thaliana] | | | | | | |
|  |  | RAFL09-15-M15 | At5g23740 / 40S ribosomal protein S11 (RPS11C) | |  |  |  |  |  | | --- | --- | --- | --- | --- | |  |  |  |  |  | | RAFL09-15-M15 ,At5g23740  40S ribosomal protein S11 (RPS11C) | | | | | | |
|  |  | RAFL05-12-E15 | At2g31610 / 40S ribosomal protein S3 (RPS3A) | |  |  |  |  |  | | --- | --- | --- | --- | --- | |  |  |  |  |  | | RAFL05-12-E15 ,At2g31610  40S ribosomal protein S3 (RPS3A) | | | | | | |
|  |  | RAFL04-14-L08 | At3g49010 / 60S ribosomal protein L13 (RPL13B)/breast basic conserved protein 1-related (BBC1) | |  |  |  |  |  | | --- | --- | --- | --- | --- | |  |  |  |  |  | | At3g49010 ,RAFL04-14-L08  60S ribosomal protein L13 (RPL13B) / breast basic conserved protein 1-related (BBC1) | | | | | | |
|  |  | RAFL06-10-J18 | At4g14320 / 60S ribosomal protein L36a/L44 (RPL36aB) | |  |  |  |  |  | | --- | --- | --- | --- | --- | |  |  |  |  |  | | RAFL06-10-J18 ,At4g14320  60S ribosomal protein L36a/L44 (RPL36aB) | | | | | | |
|  |  | RAFL11-09-J03 | At3g54210 / ribosomal protein L17 -related protein | |  |  |  |  |  | | --- | --- | --- | --- | --- | |  |  |  |  |  | | RAFL11-09-J03 ,At3g54210  ribosomal protein L17 family protein contains Pfam profile: PF01196 ribosomal protein L17 | | | | | | |
|  |  | RAFL02-10-A09 | At1g33140 / 60S ribosomal protein L9 (RPL90A/C) | |  |  |  |  |  | | --- | --- | --- | --- | --- | |  |  |  |  |  | | RAFL02-10-A09 ,At1g33140  60S ribosomal protein L9 (RPL90A/C) similar to RIBOSOMAL PROTEIN L9 GB:P49209 from [Arabidopsis thaliana] | | | | | | |
|  |  | RAFL07-15-K08 | At4g34670 / 40S ribosomal protein S3A (RPS3aB) | |  |  |  |  |  | | --- | --- | --- | --- | --- | |  |  |  |  |  | | RAFL07-15-K08 ,At4g34670  40S ribosomal protein S3A (RPS3aB) | | | | | | |
|  |  | RAFL07-14-M14 | At5g02870 / 60S ribosomal protein L4/L1 (RPL4D) | |  |  |  |  |  | | --- | --- | --- | --- | --- | |  |  |  |  |  | | At5g02870 ,RAFL07-14-M14  60S ribosomal protein L4/L1 (RPL4D) 60S roibosomal protein L4, Arabidopsis thaliana, EMBL:CAA79104 | | | | | | |
|  |  | RAFL09-06-A22 | At3g62870 / 60S ribosomal protein L7A (RPL7aB) | |  |  |  |  |  | | --- | --- | --- | --- | --- | |  |  |  |  |  | | RAFL09-06-A22 ,At3g62870  60S ribosomal protein L7A (RPL7aB) 60S RIBOSOMAL PROTEIN L7A - Oryza sativa, SWISSPROT:RL7A\_ORYSA | | | | | | |
|  |  | RAFL04-09-M12 | At4g27090 / 60S ribosomal protein L14 (RPL14B) | |  |  |  |  |  | | --- | --- | --- | --- | --- | |  |  |  |  |  | | At4g27090 ,RAFL04-09-M12  60S ribosomal protein L14 (RPL14B) ribosomal protein L14 - Human,PIR3:JC5954 | | | | | | |
|  |  | RAFL11-10-L18 | At2g36620 / 60S ribosomal protein L24 (RPL24A) | |  |  |  |  |  | | --- | --- | --- | --- | --- | |  |  |  |  |  | | RAFL11-10-L18 ,At2g36620  60S ribosomal protein L24 (RPL24A) | | | | | | |
|  |  | RAFL06-10-E08 | At2g34480 / 60S ribosomal protein L18A (RPL18aB) | |  |  |  |  |  | | --- | --- | --- | --- | --- | |  |  |  |  |  | | At2g34480 ,RAFL06-10-E08  60S ribosomal protein L18A (RPL18aB) | | | | | | |
|  |  | RAFL07-08-L21 | At5g59850 / 40S ribosomal protein S15A (RPS15aF) | |  |  |  |  |  | | --- | --- | --- | --- | --- | |  |  |  |  |  | | At5g59850 ,RAFL07-08-L21  40S ribosomal protein S15A (RPS15aF) cytoplasmic ribosomal protein S15a, Arabidopsis thaliana, EMBL:ATAF1412 | | | | | | |
|  |  | RAFL05-03-L01 | At3g56340 / 40S ribosomal protein S26 homolog | |  |  |  |  |  | | --- | --- | --- | --- | --- | |  |  |  |  |  | | At3g56340 ,RAFL05-03-L01  40S ribosomal protein S26 (RPS26C) several 40S ribosomal protein S26 | | | | | | |
|  |  | RAFL06-08-B09 | At3g11510 / 40S ribosomal protein S14 (RPS14B) | |  |  |  |  |  | | --- | --- | --- | --- | --- | |  |  |  |  |  | | At3g11510 ,RAFL06-08-B09  40S ribosomal protein S14 (RPS14B) similar to 40S ribosomal protein S14 GB:P19950 [Zea mays] | | | | | | |
|  |  | RAFL06-08-P20 | At3g07110 / 60S ribosomal protein L13A (RPL13aA) | |  |  |  |  |  | | --- | --- | --- | --- | --- | |  |  |  |  |  | | RAFL06-08-P20 ,At3g07110  60S ribosomal protein L13A (RPL13aA) similar to ribosomal protein L13A GB:O49885 [Lupinus luteus] | | | | | | |
|  |  | RAFL05-16-H14 | At1g04270 / 40S ribosomal protein S15 (RPS15A) | |  |  |  |  |  | | --- | --- | --- | --- | --- | |  |  |  |  |  | | At1g04270 ,RAFL05-16-H14  40S ribosomal protein S15 (RPS15A) Strong similarity to Oryza 40S ribosomal protein S15. ESTs gb|R29788,gb|ATTS0365 come from this gene | | | | | | |
|  |  | RAFL04-10-H14 | At3g49910 / 60S ribosomal protein L26 (RPL26A) | |  |  |  |  |  | | --- | --- | --- | --- | --- | |  |  |  |  |  | | At3g49910 ,RAFL04-10-H14  60S ribosomal protein L26 (RPL26A) 60S RIBOSOMAL PROTEIN L26, Brassica rapa, EMBL:BRD495 | | | | | | |
|  |  | RAFL05-13-M17 | At3g53890 / 40S ribosomal protein S21 homolog | |  |  |  |  |  | | --- | --- | --- | --- | --- | |  |  |  |  |  | | RAFL05-13-M17 ,At3g53890  40S ribosomal protein S21 (RPS21B) ribosomal protein S21, cytosolic - Oryza sativa, PIR:S38357 | | | | | | |
|  |  | RAFL06-11-K09 | At5g03850 / 40S ribosomal protein S28 (RPS28B) | |  |  |  |  |  | | --- | --- | --- | --- | --- | |  |  |  |  |  | | RAFL06-11-K09 ,At5g03850  40S ribosomal protein S28 (RPS28B) ribosomal protein S28, Arabidopsis thaliana, EMBL:ATRP28A | | | | | | |
|  |  | RAFL05-16-K23 | At1g02780 / 60S ribosomal protein L19 (RPL19A) | |  |  |  |  |  | | --- | --- | --- | --- | --- | |  |  |  |  |  | | RAFL05-16-K23 ,At1g02780  60S ribosomal protein L19 (RPL19A) similar to ribosomal protein L19 GI:36127 from [Homo sapiens] | | | | | | |
|  |  | RAFL05-19-H05 | At3g28900 / 60S ribosomal protein L34 (RPL34C) | |  |  |  |  |  | | --- | --- | --- | --- | --- | |  |  |  |  |  | | RAFL05-19-H05 ,At3g28900  60S ribosomal protein L34 (RPL34C) similar to 60S ribosomal protein L34 GB:P41098 [Nicotiana tabacum] | | | | | | |
|  |  | RAFL04-14-J20 | At2g40660 / methionyl-tRNA synthetase -related | |  |  |  |  |  | | --- | --- | --- | --- | --- | |  |  |  |  |  | | RAFL04-14-J20 ,At2g40660  tRNA-binding region domain-containing protein similar to SP|Q12904 Multisynthetase complex auxiliary component p43 [Contains: Endothelial-monocyte activating polypeptide II (EMAP-II) (Small inducible cytokine subfamily E member 1)] {Homo sapiens}; contains Pfam profile PF01588: Putative tRNA binding domain | | | | | | |
|  |  | RAFL06-08-F22 | At5g13650 / GTP-binding protein typA (tyrosine phosphorylated protein A) | |  |  |  |  |  | | --- | --- | --- | --- | --- | |  |  |  |  |  | | At5g13650 ,RAFL06-08-F22  elongation factor family protein contains Pfam profiles: PF00009 elongation factor Tu GTP binding domain,PF00679 elongation factor G C-terminus, PF03144 elongation factor Tu domain 2 | | | | | | |
|  |  | RAFL04-18-P03 | At4g17390 / 60S ribosomal protein L15 (RPL15B) | |  |  |  |  |  | | --- | --- | --- | --- | --- | |  |  |  |  |  | | At4g17390 ,RAFL04-18-P03  60S ribosomal protein L15 (RPL15B) | | | | | | |
|  |  | RAFL04-19-M20 | At1g58380 / 40S ribosomal protein S2 (RPS2A) | |  |  |  |  |  | | --- | --- | --- | --- | --- | |  |  |  |  |  | | RAFL04-19-M20 ,At1g58380  40S ribosomal protein S2 (RPS2A) similar to ribosomal protein S2 GI:939717 from (Urechis caupo) | | | | | | |
|  |  | RAFL05-17-P11 | At3g04920 / 40S ribosomal protein S24 (RPS24A) | |  |  |  |  |  | | --- | --- | --- | --- | --- | |  |  |  |  |  | | At3g04920 ,RAFL05-17-P11  40S ribosomal protein S24 (RPS24A) similar to ribosomal protein S19 GB:445612 [Solanum tuberosum] and similar to ribosomal protein S24 GB:4506703 [Homo sapiens] | | | | | | |
|  |  | RAFL05-13-D18 | At3g04400 / 60S ribosomal protein L23 (RPL23C) | |  |  |  |  |  | | --- | --- | --- | --- | --- | |  |  |  |  |  | | At3g04400 ,RAFL05-13-D18  60S ribosomal protein L23 (RPL23C) similar to ribosomal protein L17 GB:AAA34113.1 from [Nicotiana tabacum] | | | | | | |
|  |  | RAFL06-08-P08 | At3g23390 / 60S ribosomal protein L36a/L44 (RPL36aA) | |  |  |  |  |  | | --- | --- | --- | --- | --- | |  |  |  |  |  | | RAFL06-08-P08 ,At3g23390  60S ribosomal protein L36a/L44 (RPL36aA) similar to ribosomal protein L41 GB:AAA34366 from [Candida maltosa] | | | | | | |
|  |  | RAFL05-14-A02 | At5g52650 / 40S ribosomal protein S10 (RPS10C) | |  |  |  |  |  | | --- | --- | --- | --- | --- | |  |  |  |  |  | | At5g52650 ,RAFL05-14-A02  40S ribosomal protein S10 (RPS10C) contains similarity to 40S ribosomal protein S10 | | | | | | |
|  |  | RAFL11-06-J19 | At2g01250 / 60S ribosomal protein L7 (RPL7B) | |  |  |  |  |  | | --- | --- | --- | --- | --- | |  |  |  |  |  | | At2g01250 ,RAFL11-06-J19  60S ribosomal protein L7 (RPL7B) | | | | | | |
|  |  | RAFL11-09-C11 | At2g01250 / 60S ribosomal protein L7 (RPL7B) | |  |  |  |  |  | | --- | --- | --- | --- | --- | |  |  |  |  |  | | At2g01250 ,RAFL11-09-C11  60S ribosomal protein L7 (RPL7B) | | | | | | |
|  | Cluster:2-0 | |  |  | 36 | 114 | 327 | 4186 | 3.866317E-10 | 1.0052424E-8 | 26 |
|  |  | RAFL06-08-N09 | At3g63490 / ribosomal protein L1p family | |  |  |  |  |  | | --- | --- | --- | --- | --- | |  |  |  |  |  | | RAFL06-08-N09 ,At3g63490  ribosomal protein L1 family protein ribosomal protein L1, S.oleracea, EMBL:SORPL1 | | | | | | |
|  |  | RAFL09-09-C01 | At2g27530 / 60S ribosomal protein L10A (RPL10aB) | |  |  |  |  |  | | --- | --- | --- | --- | --- | |  |  |  |  |  | | RAFL09-09-C01 ,At2g27530  60S ribosomal protein L10A (RPL10aB) | | | | | | |
|  |  | RAFL04-10-I16 | At1g52300 / 60S ribosomal protein L37 (RPL37B) | |  |  |  |  |  | | --- | --- | --- | --- | --- | |  |  |  |  |  | | RAFL04-10-I16 ,At1g52300  60S ribosomal protein L37 (RPL37B) similar to SP:Q43292 from [Arabidopsis thaliana] | | | | | | |
|  |  | RAFL05-02-L21 | At1g07830 / ribosomal protein L29p family | |  |  |  |  |  | | --- | --- | --- | --- | --- | |  |  |  |  |  | | At1g07830 ,RAFL05-02-L21  ribosomal protein L29 family protein similar to GB:CAA83057 from [Saccharomyces cerevisiae] | | | | | | |
|  |  | RAFL05-16-E22 | At5g60390 / elongation factor 1-alpha (EF-1-alpha) | |  |  |  |  |  | | --- | --- | --- | --- | --- | |  |  |  |  |  | | At5g60390 ,RAFL05-16-E22  elongation factor 1-alpha / EF-1-alpha identical to SWISS-PROT:P13905 elongation factor 1-alpha (EF-1-alpha) [Arabidopsis thaliana] | | | | | | |
|  |  | RAFL08-12-D04 | At5g20290 / 40S ribosomal protein S8 (RPS8A) | |  |  |  |  |  | | --- | --- | --- | --- | --- | |  |  |  |  |  | | At5g20290 ,RAFL08-12-D04  40S ribosomal protein S8 (RPS8A) ribosomal protein S8 - Zea mays, PIR:T04088 | | | | | | |
|  |  | RAFL09-15-C19 | At5g60390 / elongation factor 1-alpha (EF-1-alpha) | |  |  |  |  |  | | --- | --- | --- | --- | --- | |  |  |  |  |  | | At5g60390 ,RAFL09-15-C19  elongation factor 1-alpha / EF-1-alpha identical to SWISS-PROT:P13905 elongation factor 1-alpha (EF-1-alpha) [Arabidopsis thaliana] | | | | | | |
|  |  | RAFL03-06-H07 | At1g33140 / 60S ribosomal protein L9 (RPL90A/C) | |  |  |  |  |  | | --- | --- | --- | --- | --- | |  |  |  |  |  | | RAFL03-06-H07 ,At1g33140  60S ribosomal protein L9 (RPL90A/C) similar to RIBOSOMAL PROTEIN L9 GB:P49209 from [Arabidopsis thaliana] | | | | | | |
|  |  | RAFL07-17-I21 | At1g07930 / elongation factor 1-alpha (EF-1-alpha) | |  |  |  |  |  | | --- | --- | --- | --- | --- | |  |  |  |  |  | | RAFL07-17-I21 ,At1g07930  elongation factor 1-alpha / EF-1-alpha identical to GB:CAA34456 from [Arabidopsis thaliana] (Plant Mol. Biol. 14 (1), 107-110 (1990)) | | | | | | |
|  |  | RAFL05-15-M21 | At5g20160 / ribosomal protein L7Ae family | |  |  |  |  |  | | --- | --- | --- | --- | --- | |  |  |  |  |  | | At5g20160 ,RAFL05-15-M21  ribosomal protein L7Ae/L30e/S12e/Gadd45 family protein Similar to NHP2/L7Ae family proteins, see SWISSPROT:P32495 and PMID:2063628. | | | | | | |
|  |  | RAFL03-08-O03 | At1g07940 / elongation factor 1-alpha (EF-1-alpha) | |  |  |  |  |  | | --- | --- | --- | --- | --- | |  |  |  |  |  | | RAFL03-08-O03 ,At1g07940  elongation factor 1-alpha / EF-1-alpha identical to GB:CAA34456 from [Arabidopsis thaliana] (Plant Mol. Biol. 14 (1), 107-110 (1990)) | | | | | | |
|  |  | RAFL04-19-O24 | At5g02610 / 60S ribosomal protein L35 (RPL35D) | |  |  |  |  |  | | --- | --- | --- | --- | --- | |  |  |  |  |  | | RAFL04-19-O24 ,At5g02610  60S ribosomal protein L35 (RPL35D) ribosomal protein L35- cytosolic, Arabidopsis thaliana, PIR:T00549 | | | | | | |
|  |  | RAFL06-15-I18 | At2g40590 / 40S ribosomal protein S26 (RPS26B) | |  |  |  |  |  | | --- | --- | --- | --- | --- | |  |  |  |  |  | | RAFL06-15-I18 ,At2g40590  40S ribosomal protein S26 (RPS26B) | | | | | | |
|  |  | RAFL03-06-H09 | At3g60770 / 40S ribosomal protein S13 (RPS13A) | |  |  |  |  |  | | --- | --- | --- | --- | --- | |  |  |  |  |  | | RAFL03-06-H09 ,At3g60770  40S ribosomal protein S13 (RPS13A) AtRPS13A mRNA for cytoplasmic ribosomal protein S13, Arabidopsis thaliana,AB031739 | | | | | | |
|  |  | RAFL08-09-P06 | At5g22920 / PGPD14 protein | |  |  |  |  |  | | --- | --- | --- | --- | --- | |  |  |  |  |  | | RAFL08-09-P06 ,At5g22920  zinc finger (C3HC4-type RING finger) family protein contains Pfam profiles:PF05495 CHY zinc finger, PF00097 zinc finger, C3HC4 type (RING finger) | | | | | | |
|  |  | RAFL06-08-H02 | At3g04840 / 40S ribosomal protein S3A (RPS3aA) | |  |  |  |  |  | | --- | --- | --- | --- | --- | |  |  |  |  |  | | At3g04840 ,RAFL06-08-H02  40S ribosomal protein S3A (RPS3aA) similar to 40S ribosomal protein S3A (S phase specific protein GBIS289) GB:P49396 [Brassica rapa] | | | | | | |
|  |  | RAFL07-14-H10 | At5g02870 / 60S ribosomal protein L4/L1 (RPL4D) | |  |  |  |  |  | | --- | --- | --- | --- | --- | |  |  |  |  |  | | At5g02870 ,RAFL07-14-H10  60S ribosomal protein L4/L1 (RPL4D) 60S roibosomal protein L4, Arabidopsis thaliana, EMBL:CAA79104 | | | | | | |
|  |  | RAFL07-17-B02 | At1g59359 / 40S ribosomal protein S2 (RPS2B) | |  |  |  |  |  | | --- | --- | --- | --- | --- | |  |  |  |  |  | | RAFL07-17-B02 ,At1g59359  40S ribosomal protein S2 (RPS2B) similar to ribosomal protein S2 GI:430711 from [Drosophila melanogaster] | | | | | | |
|  |  | RAFL09-12-G10 | At4g12600 / ribosomal protein L7Ae family | |  |  |  |  |  | | --- | --- | --- | --- | --- | |  |  |  |  |  | | RAFL09-12-G10 ,At4g12600  ribosomal protein L7Ae/L30e/S12e/Gadd45 family protein Similar to NHP2/L7Ae family proteins, see SWISSPROT:P32495 and PMID:2063628. | | | | | | |
|  |  | RAFL07-08-G14 | At2g31610 / 40S ribosomal protein S3 (RPS3A) | |  |  |  |  |  | | --- | --- | --- | --- | --- | |  |  |  |  |  | | RAFL07-08-G14 ,At2g31610  40S ribosomal protein S3 (RPS3A) | | | | | | |
|  |  | RAFL07-18-A18 | At3g23700 / expressed protein | |  |  |  |  |  | | --- | --- | --- | --- | --- | |  |  |  |  |  | | At3g23700 ,RAFL07-18-A18  S1 RNA-binding domain-containing protein contains Pfam domain, PF00575: S1 RNA binding domain | | | | | | |
|  |  | RAFL09-13-F11 | At1g07930 / elongation factor 1-alpha (EF-1-alpha) | |  |  |  |  |  | | --- | --- | --- | --- | --- | |  |  |  |  |  | | RAFL09-13-F11 ,At1g07930  elongation factor 1-alpha / EF-1-alpha identical to GB:CAA34456 from [Arabidopsis thaliana] (Plant Mol. Biol. 14 (1), 107-110 (1990)) | | | | | | |
|  |  | RAFL08-09-H17 | At1g07930 / elongation factor 1-alpha (EF-1-alpha) | |  |  |  |  |  | | --- | --- | --- | --- | --- | |  |  |  |  |  | | RAFL08-09-H17 ,At1g07930  elongation factor 1-alpha / EF-1-alpha identical to GB:CAA34456 from [Arabidopsis thaliana] (Plant Mol. Biol. 14 (1), 107-110 (1990)) | | | | | | |
|  |  | RAFL07-09-P18 | At4g31700 / 40S ribosomal protein S6 (RPS6A) | |  |  |  |  |  | | --- | --- | --- | --- | --- | |  |  |  |  |  | | RAFL07-09-P18 ,At4g31700  40S ribosomal protein S6 (RPS6A) ribosomal protein S6, Arabidopsis thaliana, PID:g2662469 | | | | | | |
|  |  | RAFL11-12-P10 | At3g55750 / 60S ribosomal protein L35a (RPL35aD) | |  |  |  |  |  | | --- | --- | --- | --- | --- | |  |  |  |  |  | | RAFL11-12-P10 ,At3g55750  60S ribosomal protein L35a (RPL35aD) ribosomal protein L35a.e.c15, Saccharomyces cerevisiae, PIR:S44069 | | | | | | |
|  |  | RAFL08-18-F01 | At1g07930 / elongation factor 1-alpha (EF-1-alpha) | |  |  |  |  |  | | --- | --- | --- | --- | --- | |  |  |  |  |  | | RAFL08-18-F01 ,At1g07930  elongation factor 1-alpha / EF-1-alpha identical to GB:CAA34456 from [Arabidopsis thaliana] (Plant Mol. Biol. 14 (1), 107-110 (1990)) | | | | | | |
|  |  | RAFL11-12-M17 | At3g25520 / 60S ribosomal protein L5 (RPL5A) | |  |  |  |  |  | | --- | --- | --- | --- | --- | |  |  |  |  |  | | At3g25520 ,RAFL11-12-M17  60S ribosomal protein L5 similar to 60S ribosomal protein L5 GB:P49625 from [Oryza sativa] | | | | | | |
|  |  | RAFL09-17-M14 | At2g27530 / 60S ribosomal protein L10A (RPL10aB) | |  |  |  |  |  | | --- | --- | --- | --- | --- | |  |  |  |  |  | | RAFL09-17-M14 ,At2g27530  60S ribosomal protein L10A (RPL10aB) | | | | | | |
|  |  | RAFL09-09-P17 | At1g72370 / 40S ribosomal protein SA (RPSaA) | |  |  |  |  |  | | --- | --- | --- | --- | --- | |  |  |  |  |  | | RAFL09-09-P17 ,At1g72370  40S ribosomal protein SA (RPSaA) identical to laminin receptor-like protein GB:U01955 [Arabidopsis thaliana]; identical to cDNA laminin receptor homologue GI:16379 | | | | | | |
|  |  | RAFL05-01-L16 | At5g11750 / ribosomal protein L19, putative | |  |  |  |  |  | | --- | --- | --- | --- | --- | |  |  |  |  |  | | RAFL05-01-L16 ,At5g11750  ribosomal protein L19 family protein similar to plastid ribosomal protein L19 precursor [Spinacia oleracea] gi|7582403|gb|AAF64312 | | | | | | |
|  |  | RAFL11-13-A04 | At5g02870 / 60S ribosomal protein L4/L1 (RPL4D) | |  |  |  |  |  | | --- | --- | --- | --- | --- | |  |  |  |  |  | | At5g02870 ,RAFL11-13-A04  60S ribosomal protein L4/L1 (RPL4D) 60S roibosomal protein L4, Arabidopsis thaliana, EMBL:CAA79104 | | | | | | |
|  |  | RAFL06-10-L13 | At3g09630 / 60S ribosomal protein L4/L1 (RPL4A) | |  |  |  |  |  | | --- | --- | --- | --- | --- | |  |  |  |  |  | | RAFL06-10-L13 ,At3g09630  60S ribosomal protein L4/L1 (RPL4A) strong similarity to 60S ribosomal protein L1 GB:P49691 | | | | | | |
|  |  | RAFL07-10-G10 | At1g29880 / glycyl tRNA synthetase -related | |  |  |  |  |  | | --- | --- | --- | --- | --- | |  |  |  |  |  | | RAFL07-10-G10 ,At1g29880  glycyl-tRNA synthetase / glycine--tRNA ligase identical to SP|O23627 Glycyl-tRNA synthetase (EC 6.1.1.14) (Glycine--tRNA ligase) (GlyRS) {Arabidopsis thaliana} | | | | | | |
|  |  | RAFL05-01-F21 | At3g05590 / 60S ribosomal protein L18 (RPL18B) | |  |  |  |  |  | | --- | --- | --- | --- | --- | |  |  |  |  |  | | At3g05590 ,RAFL05-01-F21  60S ribosomal protein L18 (RPL18B) similar to GB:P42791 | | | | | | |
|  |  | RAFL05-12-K17 | At1g67430 / 60S ribosomal protein L17 (RPL17B) | |  |  |  |  |  | | --- | --- | --- | --- | --- | |  |  |  |  |  | | RAFL05-12-K17 ,At1g67430  60S ribosomal protein L17 (RPL17B) similar to ribosomal protein GI:19101 from [Hordeum vulgare] | | | | | | |
|  |  | RAFL05-15-E14 | At1g70600 / 60S ribosomal protein L27A (RPL27aC) | |  |  |  |  |  | | --- | --- | --- | --- | --- | |  |  |  |  |  | | At1g70600 ,RAFL05-15-E14  60S ribosomal protein L27A (RPL27aC) identical to 60S ribosomal protein L27A GB:P49637 [Arabidopsis thaliana] | | | | | | |
|  | Cluster:3-0 | |  |  | 47 | 186 | 316 | 4114 | 4.2311096E-10 | 1.10008855E-8 | 26 |
|  |  | RAFL07-15-M24 | At1g74050 / 60S ribosomal protein L6 (RPL6C) | |  |  |  |  |  | | --- | --- | --- | --- | --- | |  |  |  |  |  | | At1g74050 ,RAFL07-15-M24  60S ribosomal protein L6 (RPL6C) similar to 60S ribosomal protein L6 (YL 16 like) GB:CAB57309 from [Cyanophora paradoxa] | | | | | | |
|  |  | RAFL04-15-D01 | At3g09200 / 60S acidic ribosomal protein P0 (RPP0B) | |  |  |  |  |  | | --- | --- | --- | --- | --- | |  |  |  |  |  | | At3g09200 ,RAFL04-15-D01  60S acidic ribosomal protein P0 (RPP0B) similar to putative 60S acidic ribosomal protein P0 GB:P50346 [Glycine max] | | | | | | |
|  |  | RAFL08-11-M03 | At5g60390 / elongation factor 1-alpha (EF-1-alpha) | |  |  |  |  |  | | --- | --- | --- | --- | --- | |  |  |  |  |  | | RAFL08-11-M03 ,At5g60390  elongation factor 1-alpha / EF-1-alpha identical to SWISS-PROT:P13905 elongation factor 1-alpha (EF-1-alpha) [Arabidopsis thaliana] | | | | | | |
|  |  | RAFL09-12-B12 | At4g36130 / 60S ribosomal protein L8 (RPL8C) | |  |  |  |  |  | | --- | --- | --- | --- | --- | |  |  |  |  |  | | At4g36130 ,RAFL09-12-B12  60S ribosomal protein L8 (RPL8C) ribosomal protein L8, cytosolic, tomato, PIR1:R5TOL8 | | | | | | |
|  |  | RAFL11-07-A12 | At5g62300 / 40S ribosomal protein S20 (RPS20C) | |  |  |  |  |  | | --- | --- | --- | --- | --- | |  |  |  |  |  | | RAFL11-07-A12 ,At5g62300  40S ribosomal protein S20 (RPS20C) ribosomal protein S20, Arabidopsis thaliana, PIR:T12992 | | | | | | |
|  |  | RAFL04-15-O08 | At5g46430 / 60S ribosomal protein L32 (RPL32B) | |  |  |  |  |  | | --- | --- | --- | --- | --- | |  |  |  |  |  | | RAFL04-15-O08 ,At5g46430  60S ribosomal protein L32 (RPL32B) | | | | | | |
|  |  | RAFL08-10-G08 | At3g09630 / 60S ribosomal protein L4/L1 (RPL4A) | |  |  |  |  |  | | --- | --- | --- | --- | --- | |  |  |  |  |  | | RAFL08-10-G08 ,At3g09630  60S ribosomal protein L4/L1 (RPL4A) strong similarity to 60S ribosomal protein L1 GB:P49691 | | | | | | |
|  |  | RAFL04-16-M10 | At1g64550 / ABC transporter family protein | |  |  |  |  |  | | --- | --- | --- | --- | --- | |  |  |  |  |  | | RAFL04-16-M10 ,At1g64550  ABC transporter family protein similar to ABC transporter protein GB:AAF31030 GI:6899653 from [Leishmania major] | | | | | | |
|  |  | RAFL09-10-P09 | At4g36130 / 60S ribosomal protein L8 (RPL8C) | |  |  |  |  |  | | --- | --- | --- | --- | --- | |  |  |  |  |  | | At4g36130 ,RAFL09-10-P09  60S ribosomal protein L8 (RPL8C) ribosomal protein L8, cytosolic, tomato, PIR1:R5TOL8 | | | | | | |
|  |  | RAFL02-10-H10 | At3g43980 / 40S ribosomal protein S29 (RPS29A) | |  |  |  |  |  | | --- | --- | --- | --- | --- | |  |  |  |  |  | | At3g43980 ,RAFL02-10-H10  40S ribosomal protein S29 (RPS29A) ribosomal protein S29, rat, PIR:S30298 | | | | | | |
|  |  | RAFL05-07-F05 | At2g17360 / 40S ribosomal protein S4 (RPS4A) | |  |  |  |  |  | | --- | --- | --- | --- | --- | |  |  |  |  |  | | At2g17360 ,RAFL05-07-F05  40S ribosomal protein S4 (RPS4A) contains ribosomal protein S4 signature from residues 8 to 22 | | | | | | |
|  |  | RAFL05-19-G10 | At3g24830 / 60S ribosomal protein L13A (RPL13aB) | |  |  |  |  |  | | --- | --- | --- | --- | --- | |  |  |  |  |  | | RAFL05-19-G10 ,At3g24830  60S ribosomal protein L13A (RPL13aB) similar to 60S RIBOSOMAL PROTEIN L13A GB:P35427 from [Rattus norvegicus] | | | | | | |
|  |  | RAFL06-07-B02 | At3g11940 / 40S ribosomal protein S5 (RPS5B) | |  |  |  |  |  | | --- | --- | --- | --- | --- | |  |  |  |  |  | | At3g11940 ,RAFL06-07-B02  40S ribosomal protein S5 (RPS5B) similar to 40S ribosomal protein S5 GB:AAC98068 GI:4056502 from [Arabidopsis thaliana] | | | | | | |
|  |  | RAFL11-11-E06 | At1g72370 / 40S ribosomal protein SA (RPSaA) | |  |  |  |  |  | | --- | --- | --- | --- | --- | |  |  |  |  |  | | RAFL11-11-E06 ,At1g72370  40S ribosomal protein SA (RPSaA) identical to laminin receptor-like protein GB:U01955 [Arabidopsis thaliana]; identical to cDNA laminin receptor homologue GI:16379 | | | | | | |
|  |  | RAFL07-16-H23 | At3g16780 / 60S ribosomal protein L19 (RPL19B) | |  |  |  |  |  | | --- | --- | --- | --- | --- | |  |  |  |  |  | | At3g16780 ,RAFL07-16-H23  60S ribosomal protein L19 (RPL19B) similar to ribosomal protein L19 GB:CAA45090 from [Homo sapiens] | | | | | | |
|  |  | RAFL05-07-A02 | At5g47930 / ribosomal protein S27 | |  |  |  |  |  | | --- | --- | --- | --- | --- | |  |  |  |  |  | | RAFL05-07-A02 ,At5g47930  40S ribosomal protein S27 (RPS27D) | | | | | | |
|  |  | RAFL08-15-A10 | At5g09510 / 40S ribosomal protein S15 (RPS15D) | |  |  |  |  |  | | --- | --- | --- | --- | --- | |  |  |  |  |  | | RAFL08-15-A10 ,At5g09510  40S ribosomal protein S15 (RPS15D) ribosomal protein S15 - Arabidopsis thaliana, EMBL:Z23161 | | | | | | |
|  |  | RAFL05-14-G01 | At4g02230 / 60S ribosomal protein L19 (RPL19C) | |  |  |  |  |  | | --- | --- | --- | --- | --- | |  |  |  |  |  | | RAFL05-14-G01 ,At4g02230  60S ribosomal protein L19 (RPL19C) similar to L19 from several species | | | | | | |
|  |  | RAFL03-05-E07 | At1g07920 / elongation factor 1-alpha (EF-1-alpha) | |  |  |  |  |  | | --- | --- | --- | --- | --- | |  |  |  |  |  | | RAFL03-05-E07 ,At1g07920  elongation factor 1-alpha / EF-1-alpha identical to GB:CAA34456 from [Arabidopsis thaliana] (Plant Mol. Biol. 14 (1), 107-110 (1990)) | | | | | | |
|  |  | RAFL06-12-J07 | At3g53020 / 60S ribosomal protein L24 (RPL24B) | |  |  |  |  |  | | --- | --- | --- | --- | --- | |  |  |  |  |  | | At3g53020 ,RAFL06-12-J07  60S ribosomal protein L24 (RPL24B) 60S ribosomal protein L24, Arabidopsis thaliana, EMBL:AC006282 | | | | | | |
|  |  | RAFL06-16-N16 | At4g16720 / 60S ribosomal protein L15 (RPL15A) | |  |  |  |  |  | | --- | --- | --- | --- | --- | |  |  |  |  |  | | RAFL06-16-N16 ,At4g16720  60S ribosomal protein L15 (RPL15A) | | | | | | |
|  |  | RAFL04-17-H14 | At3g06700 / 60S ribosomal protein L29 (RPL29A) | |  |  |  |  |  | | --- | --- | --- | --- | --- | |  |  |  |  |  | | RAFL04-17-H14 ,At3g06700  60S ribosomal protein L29 (RPL29A) similar to ribosomal protein L29 GI:7959366 [Panax ginseng] | | | | | | |
|  |  | RAFL04-09-E11 | At5g08180 / ribosomal protein L7Ae family | |  |  |  |  |  | | --- | --- | --- | --- | --- | |  |  |  |  |  | | At5g08180 ,RAFL04-09-E11  ribosomal protein L7Ae/L30e/S12e/Gadd45 family protein Similar to NHP2/L7Ae family proteins, see SWISSPROT:P32495 and PMID:2063628. | | | | | | |
|  |  | RAFL05-09-M22 | At3g49910 / 60S ribosomal protein L26 (RPL26A) | |  |  |  |  |  | | --- | --- | --- | --- | --- | |  |  |  |  |  | | RAFL05-09-M22 ,At3g49910  60S ribosomal protein L26 (RPL26A) 60S RIBOSOMAL PROTEIN L26, Brassica rapa, EMBL:BRD495 | | | | | | |
|  |  | RAFL04-15-O15 | At1g30230 / elongation factor 1-beta -related | |  |  |  |  |  | | --- | --- | --- | --- | --- | |  |  |  |  |  | | RAFL04-15-O15 ,At1g30230  elongation factor 1-beta / EF-1-beta identical to SP|P48006 Elongation factor 1-beta (EF-1-beta) {Arabidopsis thaliana} | | | | | | |
|  |  | RAFL05-13-K07 | At4g09800 / 40S ribosomal protein S18 (RPS18C) | |  |  |  |  |  | | --- | --- | --- | --- | --- | |  |  |  |  |  | | RAFL05-13-K07 ,At4g09800  40S ribosomal protein S18 (RPS18C) | | | | | | |
|  |  | RAFL04-14-I15 | At3g02080 / 40S ribosomal protein S19 (RPS19A) | |  |  |  |  |  | | --- | --- | --- | --- | --- | |  |  |  |  |  | | RAFL04-14-I15 ,At3g02080  40S ribosomal protein S19 (RPS19A) similar to 40S ribosomal protein S19 GB:P40978 [Oryza sativa] | | | | | | |
|  |  | RAFL11-01-C20 | At3g06590 / bHLH protein | |  |  |  |  |  | | --- | --- | --- | --- | --- | |  |  |  |  |  | | RAFL11-01-C20 ,At3g06590  expressed protein | | | | | | |
|  |  | RAFL07-16-A17 | At5g64140 / 40S ribosomal protein S28 (RPS28C) | |  |  |  |  |  | | --- | --- | --- | --- | --- | |  |  |  |  |  | | At5g64140 ,RAFL07-16-A17  40S ribosomal protein S28 (RPS28C) | | | | | | |
|  |  | RAFL07-10-D02 | At1g14320 / 60S ribosomal protein L10 (RPL10A)/Wilm's tumor suppressor protein-related | |  |  |  |  |  | | --- | --- | --- | --- | --- | |  |  |  |  |  | | At1g14320 ,RAFL07-10-D02  60S ribosomal protein L10 (RPL10A) / Wilm's tumor suppressor protein-related similar to tumor suppressor GI:575354 from [Oryza sativa] | | | | | | |
|  |  | RAFL08-13-M06 | At3g11250 / 60S acidic ribosomal protein P0 (RPP0C) | |  |  |  |  |  | | --- | --- | --- | --- | --- | |  |  |  |  |  | | At3g11250 ,RAFL08-13-M06  60S acidic ribosomal protein P0 (RPP0C) similar to 60S acidic ribosomal protein P0 GI:2088654 [Arabidopsis thaliana] | | | | | | |
|  |  | RAFL06-08-M13 | At1g07920 / elongation factor 1-alpha (EF-1-alpha) | |  |  |  |  |  | | --- | --- | --- | --- | --- | |  |  |  |  |  | | RAFL06-08-M13 ,At1g07920  elongation factor 1-alpha / EF-1-alpha identical to GB:CAA34456 from [Arabidopsis thaliana] (Plant Mol. Biol. 14 (1), 107-110 (1990)) | | | | | | |
|  |  | RAFL07-10-F16 | At3g13930 / acetyltransferase -related | |  |  |  |  |  | | --- | --- | --- | --- | --- | |  |  |  |  |  | | At3g13930 ,RAFL07-10-F16  dihydrolipoamide S-acetyltransferase, putative similar to dihydrolipoamide S-acetyltransferase [Zea mays] GI:5669871; contains Pfam profiles PF00198: 2-oxo acid dehydrogenases acyltransferase (catalytic domain), PF00364: Biotin-requiring enzyme, PF02817: e3 binding domain | | | | | | |
|  |  | RAFL05-18-H11 | At5g27700 / ribosomal protein S21 - like | |  |  |  |  |  | | --- | --- | --- | --- | --- | |  |  |  |  |  | | RAFL05-18-H11 ,At5g27700  40S ribosomal protein S21 (RPS21C) ribosomal protein S21, Zea mays, PIR:T03945 | | | | | | |
|  |  | RAFL11-02-J15 | At4g00100 / 40S ribosomal protein S13 (RPS13B) | |  |  |  |  |  | | --- | --- | --- | --- | --- | |  |  |  |  |  | | At4g00100 ,RAFL11-02-J15  40S ribosomal protein S13 (RPS13A) similar to ribosomal protein S13; PF00312 (View Sanger Pfam): ribosomal protein S15; identical to cDNA AtRPS13A mRNA for cytoplasmic ribosomal protein S13 GI:6521011 | | | | | | |
|  |  | RAFL09-18-O15 | At2g17360 / 40S ribosomal protein S4 (RPS4A) | |  |  |  |  |  | | --- | --- | --- | --- | --- | |  |  |  |  |  | | RAFL09-18-O15 ,At2g17360  40S ribosomal protein S4 (RPS4A) contains ribosomal protein S4 signature from residues 8 to 22 | | | | | | |
|  |  | RAFL07-08-M19 | At1g09620 / leucyl-tRNA synthetase -related | |  |  |  |  |  | | --- | --- | --- | --- | --- | |  |  |  |  |  | | At1g09620 ,RAFL07-08-M19  tRNA synthetase class I (I, L, M and V) family protein similar to cytosolic leucyl-tRNA synthetase [Candida albicans] GI:9858190; contains Pfam profile PF00133: tRNA synthetases class I (I, L, M and V) | | | | | | |
|  |  | RAFL05-07-M06 | At3g20290 / calcium-binding EF-hand family protein | |  |  |  |  |  | | --- | --- | --- | --- | --- | |  |  |  |  |  | | RAFL05-07-M06 ,At3g20290  calcium-binding EF hand family protein similar to EH-domain containing protein 1 from {Mus musculus} SP|Q9WVK4 and {Homo sapiens} SP|Q9H4M9, receptor-mediated endocytosis 1 from [Caenorhabditis elegans] GI:13487775, GI:13487777, GI:13487779; contains INTERPRO:IPR002048 calcium-binding EF-hand domain | | | | | | |
|  |  | RAFL11-04-A02 | At1g07770 / 40S ribosomal protein S15A (RPS15aA) | |  |  |  |  |  | | --- | --- | --- | --- | --- | |  |  |  |  |  | | At1g07770 ,RAFL11-04-A02  40S ribosomal protein S15A (RPS15aA) identical to GB:AAA61608 from [Arabidopsis thaliana] (Plant Physiol. 106 (1), 401-402 (1994)) | | | | | | |
|  |  | RAFL09-09-O07 | At4g16720 / 60S ribosomal protein L15 (RPL15A) | |  |  |  |  |  | | --- | --- | --- | --- | --- | |  |  |  |  |  | | RAFL09-09-O07 ,At4g16720  60S ribosomal protein L15 (RPL15A) | | | | | | |
|  |  | RAFL04-09-F23 | At2g31610 / 40S ribosomal protein S3 (RPS3A) | |  |  |  |  |  | | --- | --- | --- | --- | --- | |  |  |  |  |  | | RAFL04-09-F23 ,At2g31610  40S ribosomal protein S3 (RPS3A) | | | | | | |
|  |  | RAFL09-13-H18 | At1g43170 / 60S ribosomal protein L3 (RPL3A) | |  |  |  |  |  | | --- | --- | --- | --- | --- | |  |  |  |  |  | | At1g43170 ,RAFL09-13-H18  60S ribosomal protein L3 (RPL3A) identical to ribosomal protein GI:166858 from [Arabidopsis thaliana] | | | | | | |
|  |  | RAFL08-09-E20 | At2g41840 / 40S ribosomal protein S2 (RPS2C) | |  |  |  |  |  | | --- | --- | --- | --- | --- | |  |  |  |  |  | | RAFL08-09-E20 ,At2g41840  40S ribosomal protein S2 (RPS2C) | | | | | | |
|  |  | RAFL09-07-I03 | At5g60390 / elongation factor 1-alpha (EF-1-alpha) | |  |  |  |  |  | | --- | --- | --- | --- | --- | |  |  |  |  |  | | At5g60390 ,RAFL09-07-I03  elongation factor 1-alpha / EF-1-alpha identical to SWISS-PROT:P13905 elongation factor 1-alpha (EF-1-alpha) [Arabidopsis thaliana] | | | | | | |
|  |  | RAFL05-14-H24 | At2g21580 / 40S ribosomal protein S25 (RPS25B) | |  |  |  |  |  | | --- | --- | --- | --- | --- | |  |  |  |  |  | | At2g21580 ,RAFL05-14-H24  40S ribosomal protein S25 (RPS25B) | | | | | | |
|  |  | RAFL11-04-H04 | At2g34480 / 60S ribosomal protein L18A (RPL18aB) | |  |  |  |  |  | | --- | --- | --- | --- | --- | |  |  |  |  |  | | At2g34480 ,RAFL11-04-H04  60S ribosomal protein L18A (RPL18aB) | | | | | | |
|  |  | RAFL07-17-K15 | At2g31040 / expressed protein | |  |  |  |  |  | | --- | --- | --- | --- | --- | |  |  |  |  |  | | RAFL07-17-K15 ,At2g31040  ATP synthase protein I -related contains weaks similarity to Swiss-Prot:P08443 ATP synthase protein I [Synechococcus sp.] | | | | | | |
|  | Cluster:1-2 | |  |  | 33 | 141 | 330 | 4159 | 1.4985008E-6 | 3.896102E-5 | 26 |
|  |  | RAFL06-16-L14 | At5g48760 / 60S ribosomal protein L13A (RPL13aD) | |  |  |  |  |  | | --- | --- | --- | --- | --- | |  |  |  |  |  | | RAFL06-16-L14 ,At5g48760  60S ribosomal protein L13A (RPL13aD) | | | | | | |
|  |  | RAFL09-07-N01 | At2g04390 / 40S ribosomal protein S17 (RPS17A) | |  |  |  |  |  | | --- | --- | --- | --- | --- | |  |  |  |  |  | | At2g04390 ,RAFL09-07-N01  40S ribosomal protein S17 (RPS17A) | | | | | | |
|  |  | RAFL11-10-E06 | At5g45775 / 60S ribosomal protein L11 (RPL11D) | |  |  |  |  |  | | --- | --- | --- | --- | --- | |  |  |  |  |  | | RAFL11-10-E06 ,At5g45775  60S ribosomal protein L11 (RPL11D) | | | | | | |
|  |  | RAFL05-21-A12 | At4g29060 / expressed protein | |  |  |  |  |  | | --- | --- | --- | --- | --- | |  |  |  |  |  | | RAFL05-21-A12 ,At4g29060  elongation factor Ts family protein similar to SP|P35019 Elongation factor Ts (EF-Ts) {Galdieria sulphuraria}; contains Pfam profiles PF00627: UBA/TS-N domain, PF00889: Elongation factor TS, PF00575: S1 RNA binding domain | | | | | | |
|  |  | RAFL07-09-B01 | At4g31700 / 40S ribosomal protein S6 (RPS6A) | |  |  |  |  |  | | --- | --- | --- | --- | --- | |  |  |  |  |  | | RAFL07-09-B01 ,At4g31700  40S ribosomal protein S6 (RPS6A) ribosomal protein S6, Arabidopsis thaliana, PID:g2662469 | | | | | | |
|  |  | RAFL07-08-E09 | At5g49030 / isoleucyl-tRNA synthetase | |  |  |  |  |  | | --- | --- | --- | --- | --- | |  |  |  |  |  | | RAFL07-08-E09 ,At5g49030  tRNA synthetase class I (I, L, M and V) family protein similar to SP|P41972 Isoleucyl-tRNA synthetase (EC 6.1.1.5) (Isoleucine--tRNA ligase) (IleRS) {Staphylococcus aureus}; contains Pfam profile PF00133: tRNA synthetases class I (I, L, M and V) | | | | | | |
|  |  | RAFL04-10-C15 | At1g09590 / 60S ribosomal protein L21 (RPL21A) | |  |  |  |  |  | | --- | --- | --- | --- | --- | |  |  |  |  |  | | RAFL04-10-C15 ,At1g09590  60S ribosomal protein L21 (RPL21A) Similar to L21 family of ribosomal protein; amino acid sequence is identical to F21M12.8 | | | | | | |
|  |  | RAFL06-10-D03 | At5g67510 / 60S ribosomal protein L26 (RPL26B) | |  |  |  |  |  | | --- | --- | --- | --- | --- | |  |  |  |  |  | | At5g67510 ,RAFL06-10-D03  60S ribosomal protein L26 (RPL26B) | | | | | | |
|  |  | RAFL04-12-O17 | At5g30510 / ribosomal protein S1 | |  |  |  |  |  | | --- | --- | --- | --- | --- | |  |  |  |  |  | | At5g30510 ,RAFL04-12-O17  30S ribosomal protein S1, putative similar to Swiss-Prot:P29344 30S ribosomal protein S1, chloroplast precursor (CS1) [Spinacia oleracea] | | | | | | |
|  |  | RAFL06-08-P05 | At3g25520 / 60S ribosomal protein L5 (RPL5A) | |  |  |  |  |  | | --- | --- | --- | --- | --- | |  |  |  |  |  | | At3g25520 ,RAFL06-08-P05  60S ribosomal protein L5 similar to 60S ribosomal protein L5 GB:P49625 from [Oryza sativa] | | | | | | |
|  |  | RAFL06-11-J01 | At1g09690 / 60S ribosomal protein L21 (RPL21C) | |  |  |  |  |  | | --- | --- | --- | --- | --- | |  |  |  |  |  | | RAFL06-11-J01 ,At1g09690  60S ribosomal protein L21 (RPL21C) Similar to ribosomal protein L21 (gb|L38826). ESTs gb|AA395597,gb|ATTS5197 come from this gene | | | | | | |
|  |  | RAFL05-03-J07 | At1g27400 / 60S ribosomal protein L17 (RPL17A) | |  |  |  |  |  | | --- | --- | --- | --- | --- | |  |  |  |  |  | | At1g27400 ,RAFL05-03-J07  60S ribosomal protein L17 (RPL17A) similar to GB:P51413 from [Arabidopsis thaliana]; similar to ESTs gb|L33542 and gb|AA660016 | | | | | | |
|  |  | RAFL09-15-H22 | At1g05190 / ribosomal protein L6p family | |  |  |  |  |  | | --- | --- | --- | --- | --- | |  |  |  |  |  | | At1g05190 ,RAFL09-15-H22  ribosomal protein L6 family protein Similar to Mycobacterium RlpF (gb|Z84395). ESTs gb|T75785,gb|R30580,gb|T04698 come from this gene | | | | | | |
|  |  | RAFL05-14-H02 | At4g34620 / ribosomal protein S16p family | |  |  |  |  |  | | --- | --- | --- | --- | --- | |  |  |  |  |  | | RAFL05-14-H02 ,At4g34620  ribosomal protein S16 family protein ribosomal protein S16, Neurospora crassa, PIR2:A29927 | | | | | | |
|  |  | RAFL05-03-L24 | At5g60670 / 60S ribosomal protein L12 (RPL12C) | |  |  |  |  |  | | --- | --- | --- | --- | --- | |  |  |  |  |  | | RAFL05-03-L24 ,At5g60670  60S ribosomal protein L12 (RPL12C) 60S RIBOSOMAL PROTEIN L12 (like), Arabidopsis thaliana, PIR:T45883 | | | | | | |
|  |  | RAFL06-08-N13 | At3g49010 / 60S ribosomal protein L13 (RPL13B)/breast basic conserved protein 1-related (BBC1) | |  |  |  |  |  | | --- | --- | --- | --- | --- | |  |  |  |  |  | | At3g49010 ,RAFL06-08-N13  60S ribosomal protein L13 (RPL13B) / breast basic conserved protein 1-related (BBC1) | | | | | | |
|  |  | RAFL05-04-J09 | At5g45775 / 60S ribosomal protein L11 (RPL11D) | |  |  |  |  |  | | --- | --- | --- | --- | --- | |  |  |  |  |  | | At5g45775 ,RAFL05-04-J09  60S ribosomal protein L11 (RPL11D) | | | | | | |
|  |  | RAFL05-10-D01 | At2g05220 / 40S ribosomal protein S17 (RPS17B) | |  |  |  |  |  | | --- | --- | --- | --- | --- | |  |  |  |  |  | | RAFL05-10-D01 ,At2g05220  40S ribosomal protein S17 (RPS17B) | | | | | | |
|  |  | RAFL05-18-H13 | At5g65220 / ribosomal protein L29p family | |  |  |  |  |  | | --- | --- | --- | --- | --- | |  |  |  |  |  | | RAFL05-18-H13 ,At5g65220  ribosomal protein L29 family protein contains Pfam profile PF00831: ribosomal protein L29 | | | | | | |
|  |  | RAFL05-14-G11 | At2g33370 / 60S ribosomal protein L23 (RPL23B) | |  |  |  |  |  | | --- | --- | --- | --- | --- | |  |  |  |  |  | | RAFL05-14-G11 ,At2g33370  60S ribosomal protein L23 (RPL23B) | | | | | | |
|  |  | RAFL06-07-D01 | At2g01250 / 60S ribosomal protein L7 (RPL7B) | |  |  |  |  |  | | --- | --- | --- | --- | --- | |  |  |  |  |  | | At2g01250 ,RAFL06-07-D01  60S ribosomal protein L7 (RPL7B) | | | | | | |
|  |  | RAFL04-10-P05 | At4g25740 / 40S ribosomal protein S10 (RPS10A) | |  |  |  |  |  | | --- | --- | --- | --- | --- | |  |  |  |  |  | | At4g25740 ,RAFL04-10-P05  40S ribosomal protein S10 (RPS10A) 40S ribosomal protein S10 - Lumbricus rubellus, PID:e1329701 | | | | | | |
|  |  | RAFL06-13-L16 | At5g07090 / 40S ribosomal protein S4 (RPS4B) | |  |  |  |  |  | | --- | --- | --- | --- | --- | |  |  |  |  |  | | At5g07090 ,RAFL06-13-L16  40S ribosomal protein S4 (RPS4B) | | | | | | |
|  |  | RAFL04-12-J05 | At1g32990 / ribosomal protein L11p family | |  |  |  |  |  | | --- | --- | --- | --- | --- | |  |  |  |  |  | | RAFL04-12-J05 ,At1g32990  ribosomal protein L11 family protein similar to chloroplast ribosomal protein L11 GI:21312 from [Spinacia oleracea] | | | | | | |
|  |  | RAFL05-12-L08 | At2g18020 / 60S ribosomal protein L8 (RPL8A) | |  |  |  |  |  | | --- | --- | --- | --- | --- | |  |  |  |  |  | | At2g18020 ,RAFL05-12-L08  60S ribosomal protein L8 (RPL8A) | | | | | | |
|  |  | RAFL04-16-H06 | At1g08360 / 60S ribosomal protein L10A (RPL10aA) | |  |  |  |  |  | | --- | --- | --- | --- | --- | |  |  |  |  |  | | At1g08360 ,RAFL04-16-H06  60S ribosomal protein L10A (RPL10aA) similar to 60S ribosomal protein L10A GB:AAC73045 GI:3860277 from [Arabidopsis thaliana] | | | | | | |
|  |  | RAFL05-04-H07 | At3g27160 / expressed protein | |  |  |  |  |  | | --- | --- | --- | --- | --- | |  |  |  |  |  | | RAFL05-04-H07 ,At3g27160  ribosomal protein S21 family protein contains Pfam profile: PF01165 ribosomal protein S21 | | | | | | |
|  |  | RAFL05-13-G24 | At5g54600 / 50S ribosomal protein L24, chloroplast precursor (CL24) | |  |  |  |  |  | | --- | --- | --- | --- | --- | |  |  |  |  |  | | At5g54600 ,RAFL05-13-G24  50S ribosomal protein L24, chloroplast (CL24) identical to SP|P92959 50S ribosomal protein L24, chloroplast precursor {Arabidopsis thaliana} | | | | | | |
|  |  | RAFL07-16-I10 | At4g34620 / ribosomal protein S16p family | |  |  |  |  |  | | --- | --- | --- | --- | --- | |  |  |  |  |  | | RAFL07-16-I10 ,At4g34620  ribosomal protein S16 family protein ribosomal protein S16, Neurospora crassa, PIR2:A29927 | | | | | | |
|  |  | RAFL09-11-E10 | At1g17220 / translation initiation factor IF-2, chloroplast precursor | |  |  |  |  |  | | --- | --- | --- | --- | --- | |  |  |  |  |  | | RAFL09-11-E10 ,At1g17220  translation initiation factor IF-2, chloroplast, putative similar to SP|P57997|IF2C\_PHAVU Translation initiation factor IF-2, chloroplast precursor (PvIF2cp) {Phaseolus vulgaris} | | | | | | |
|  |  | RAFL04-15-L21 | At1g41880 / 60S ribosomal protein L35a (RPL35aB) | |  |  |  |  |  | | --- | --- | --- | --- | --- | |  |  |  |  |  | | RAFL04-15-L21 ,At1g41880  60S ribosomal protein L35a (RPL35aB) identical to GB:CAB81600 from [Arabidopsis thaliana] | | | | | | |
|  |  | RAFL05-19-N16 | At3g45030 / 40S ribosomal protein S20 (RPS20A) | |  |  |  |  |  | | --- | --- | --- | --- | --- | |  |  |  |  |  | | At3g45030 ,RAFL05-19-N16  40S ribosomal protein S20 (RPS20A) 40S ribsomomal proteinS20, Arabidopsis thaliana, pir:T12992 | | | | | | |
|  |  | RAFL11-12-P09 | At3g54210 / ribosomal protein L17 -related protein | |  |  |  |  |  | | --- | --- | --- | --- | --- | |  |  |  |  |  | | RAFL11-12-P09 ,At3g54210  ribosomal protein L17 family protein contains Pfam profile: PF01196 ribosomal protein L17 | | | | | | |
| GO:0005739 | | | mitochondrion |  | A | B | C | D | P | P' | N |
|  | Cluster:10-0 | |  |  | 12 | 12 | 933 | 3706 | 0.0010718829 | 0.035372138 | 33 |
|  |  | RAFL05-04-I14 | At1g52690 / late embryogenesis abundant (LEA) protein, putative | |  |  |  |  |  | | --- | --- | --- | --- | --- | |  |  |  |  |  | | At1g52690 ,RAFL05-04-I14  late embryogenesis abundant protein, putative / LEA protein, putative similar to SP|P13934 Late embryogenesis abundant protein 76 (LEA 76) {Brassica napus}; contains Pfam profile PF02987: Late embryogenesis abundant protein | | | | | | |
|  |  | RAFL05-16-F03 | At1g49450 / transducin / WD-40 repeat protein family | |  |  |  |  |  | | --- | --- | --- | --- | --- | |  |  |  |  |  | | RAFL05-16-F03 ,At1g49450  transducin family protein / WD-40 repeat family protein contains 7 WD-40 repeats (PF00400); similar to En/Spm-like transposon protein GI:2739374 from [Arabidopsis thaliana]; no characterized homologs | | | | | | |
|  |  | RAFL08-13-P06 | At1g52690 / late embryogenesis abundant (LEA) protein, putative | |  |  |  |  |  | | --- | --- | --- | --- | --- | |  |  |  |  |  | | At1g52690 ,RAFL08-13-P06  late embryogenesis abundant protein, putative / LEA protein, putative similar to SP|P13934 Late embryogenesis abundant protein 76 (LEA 76) {Brassica napus}; contains Pfam profile PF02987: Late embryogenesis abundant protein | | | | | | |
|  |  | RAFL05-09-G08 | At3g15670 / late embryogenesis abundant (LEA) protein, putative | |  |  |  |  |  | | --- | --- | --- | --- | --- | |  |  |  |  |  | | At3g15670 ,RAFL05-09-G08  late embryogenesis abundant protein, putative / LEA protein, putative similar to SP|P13934 Late embryogenesis abundant protein 76 (LEA 76) {Brassica napus}; contains Pfam profile PF02987: Late embryogenesis abundant protein | | | | | | |
|  |  | RAFL08-14-E03 | At1g52690 / late embryogenesis abundant (LEA) protein, putative | |  |  |  |  |  | | --- | --- | --- | --- | --- | |  |  |  |  |  | | At1g52690 ,RAFL08-14-E03  late embryogenesis abundant protein, putative / LEA protein, putative similar to SP|P13934 Late embryogenesis abundant protein 76 (LEA 76) {Brassica napus}; contains Pfam profile PF02987: Late embryogenesis abundant protein | | | | | | |
|  |  | RAFL08-16-M12 | At2g33380 / RD20 protein | |  |  |  |  |  | | --- | --- | --- | --- | --- | |  |  |  |  |  | | RAFL08-16-M12 ,At2g33380  calcium-binding RD20 protein (RD20) induced by abscisic acid during dehydration PMID:10965948; putative transmembrane channel protein PMID:10965948; identical to GI:10862968 [Arabidopsis thaliana]; contains EF-hand domain | | | | | | |
|  |  | RAFL06-13-J20 | At1g52690 / late embryogenesis abundant (LEA) protein, putative | |  |  |  |  |  | | --- | --- | --- | --- | --- | |  |  |  |  |  | | At1g52690 ,RAFL06-13-J20  late embryogenesis abundant protein, putative / LEA protein, putative similar to SP|P13934 Late embryogenesis abundant protein 76 (LEA 76) {Brassica napus}; contains Pfam profile PF02987: Late embryogenesis abundant protein | | | | | | |
|  |  | RAFL08-14-A19 | At4g27410 / No apical meristem (NAM) protein family | |  |  |  |  |  | | --- | --- | --- | --- | --- | |  |  |  |  |  | | At4g27410 ,RAFL08-14-A19  no apical meristem (NAM) family protein (RD26) contains Pfam PF02365: No apical meristem (NAM) domain; Arabidopsis thaliana nap gene,PID:e1234813; identical to cDNA RD26 mRNA for NAM-like protein GI:15375403 | | | | | | |
|  |  | RAFL04-17-F01 | At5g52310 / low-temperature-induced protein 78 (sp|Q06738) | |  |  |  |  |  | | --- | --- | --- | --- | --- | |  |  |  |  |  | | RAFL04-17-F01 ,At5g52310  low-temperature-responsive protein 78 (LTI78) / desiccation-responsive protein 29A (RD29A) | | | | | | |
|  |  | RAFL05-11-I09 | At5g52300 / low-temperature-induced 65 kD protein (sp|Q04980) | |  |  |  |  |  | | --- | --- | --- | --- | --- | |  |  |  |  |  | | RAFL05-11-I09 ,At5g52300  low-temperature-responsive 65 kD protein (LTI65) / desiccation-responsive protein 29B (RD29B) nearly identical to SP|Q04980 Low-temperature-induced 65 kDa protein (Desiccation-responsive protein 29B) {Arabidopsis thaliana} | | | | | | |
|  |  | RAFL09-17-M11 | At1g20450 / dehydrin (ERD10) | |  |  |  |  |  | | --- | --- | --- | --- | --- | |  |  |  |  |  | | RAFL09-17-M11 ,At1g20450  dehydrin (ERD10) identical to dehydrin ERD10 (Low-temperature-induced protein LTI45) [Arabidopsis thaliana] SWISS-PROT:P42759 | | | | | | |
|  |  | RAFL07-11-M21 | At5g52310 / low-temperature-induced protein 78 (sp|Q06738) | |  |  |  |  |  | | --- | --- | --- | --- | --- | |  |  |  |  |  | | At5g52310 ,RAFL07-11-M21  low-temperature-responsive protein 78 (LTI78) / desiccation-responsive protein 29A (RD29A) | | | | | | |
|  | Cluster:5-1 | |  |  | 77 | 205 | 868 | 3513 | 0.003572289 | 0.11788554 | 33 |
|  |  | RAFL06-13-O22 | At3g16400 / jacalin lectin family | |  |  |  |  |  | | --- | --- | --- | --- | --- | |  |  |  |  |  | | At3g16400 ,RAFL06-13-O22  jacalin lectin family protein similar to myrosinase-binding protein homolog [Arabidopsis thaliana] GI:2997767, epithiospecifier [Arabidopsis thaliana] GI:16118845; contains Pfam profiles PF01419 jacalin-like lectin family, PF01344 Kelch motif | | | | | | |
|  |  | RAFL05-15-F08 | At5g63860 / UVB-resistance protein UVR8 (gb|AAD43920.1) | |  |  |  |  |  | | --- | --- | --- | --- | --- | |  |  |  |  |  | | RAFL05-15-F08 ,At5g63860  UVB-resistance protein (UVR8) identical to UVB-resistance protein UVR8 (GI:5478530, GB:AAD43920.1) [Arabidopsis thaliana]; contains Pfam 00415: Regulator of chromosome condensation (RCC1) | | | | | | |
|  |  | RAFL08-13-L08 | At2g02160 / expressed protein | |  |  |  |  |  | | --- | --- | --- | --- | --- | |  |  |  |  |  | | RAFL08-13-L08 ,At2g02160  zinc finger (CCCH-type) family protein contains Pfam domain, PF00642: Zinc finger C-x8-C-x5-C-x3-H type (and similar) | | | | | | |
|  |  | RAFL09-13-F09 | At3g51630 / MAP kinase | |  |  |  |  |  | | --- | --- | --- | --- | --- | |  |  |  |  |  | | RAFL09-13-F09 ,At3g51630  protein kinase family protein contains Pfam profile: PF00069 protein kinase domain | | | | | | |
|  |  | RAFL08-09-H04 | At3g16810 / expressed protein | |  |  |  |  |  | | --- | --- | --- | --- | --- | |  |  |  |  |  | | RAFL08-09-H04 ,At3g16810  pumilio/Puf RNA-binding domain-containing protein contains Pfam profile:PF00806 Pumilio-family RNA binding domains | | | | | | |
|  |  | RAFL05-07-A20 | At1g22730 / MA3 domain-containing protein | |  |  |  |  |  | | --- | --- | --- | --- | --- | |  |  |  |  |  | | RAFL05-07-A20 ,At1g22730  MA3 domain-containing protein contains Pfam profile PF02847: MA3 domain; low similarity to programmed cell death 4 protein [Gallus gallus] GI:12958564 | | | | | | |
|  |  | RAFL05-21-L06 | At3g03570 / expressed protein | |  |  |  |  |  | | --- | --- | --- | --- | --- | |  |  |  |  |  | | RAFL05-21-L06 ,At3g03570  expressed protein similar to hypothetical protein GB:CAB38918 [Arabidopsis thaliana] | | | | | | |
|  |  | RAFL02-02-C03 | At3g07720 / Kelch repeats protein family | |  |  |  |  |  | | --- | --- | --- | --- | --- | |  |  |  |  |  | | RAFL02-02-C03 ,At3g07720  kelch repeat-containing protein similar to epithiospecifier (GI:16118838) [Arabidopsis thaliana]; contains Pfam PF01344: Kelch motif (5 repeats) | | | | | | |
|  |  | RAFL05-05-G24 | At5g41810 / expressed protein | |  |  |  |  |  | | --- | --- | --- | --- | --- | |  |  |  |  |  | | At5g41810 ,RAFL05-05-G24  expressed protein | | | | | | |
|  |  | RAFL02-01-D07 | At3g14990 / putative 4-methyl-5(b-hydroxyethyl)-thiazole monophosphate biosynthesis protein | |  |  |  |  |  | | --- | --- | --- | --- | --- | |  |  |  |  |  | | RAFL02-01-D07 ,At3g14990  4-methyl-5(b-hydroxyethyl)-thiazole monophosphate biosynthesis protein, putative supporting cDNA gi|11908017|gb|AF326856.1|; contains Pfam profile PF01965:DJ-1/PfpI family; identical to cDNA EST Athsr7 GI:5281061 | | | | | | |
|  |  | RAFL05-14-J15 | At1g50380 / expressed protein | |  |  |  |  |  | | --- | --- | --- | --- | --- | |  |  |  |  |  | | At1g50380 ,RAFL05-14-J15  prolyl oligopeptidase family protein similar to oligopeptidase B [Leishmania major] GI:4581757; contains Pfam profiles PF00326: prolyl oligopeptidase family, PF02897: Prolyl oligopeptidase, N-terminal beta-propeller domain | | | | | | |
|  |  | RAFL09-18-M19 | At1g33990 / hydrolase, alpha/beta fold family | |  |  |  |  |  | | --- | --- | --- | --- | --- | |  |  |  |  |  | | RAFL09-18-M19 ,At1g33990  hydrolase, alpha/beta fold family protein similar to polyneuridine aldehyde esterase GI:6651393 from [Rauvolfia serpentina], SP|Q40708 PIR7A protein {Oryza sativa}, ethylene-induced esterase [Citrus sinensis] GI:14279437; contains Pfam profile PF00561: hydrolase, alpha/beta fold family | | | | | | |
|  |  | RAFL04-13-J09 | At4g37560 / formamidase - like protein | |  |  |  |  |  | | --- | --- | --- | --- | --- | |  |  |  |  |  | | At4g37560 ,RAFL04-13-J09  formamidase, putative / formamide amidohydrolase, putative similar to SP|Q50228 Formamidase (EC 3.5.1.49) (Formamide amidohydrolase) {Methylophilus methylotrophus}; contains Pfam profile PF03069: Acetamidase/Formamidase family | | | | | | |
|  |  | RAFL08-16-B22 | At1g11840 / glyoxalase I, putative (lactoylglutathione lyase) | |  |  |  |  |  | | --- | --- | --- | --- | --- | |  |  |  |  |  | | RAFL08-16-B22 ,At1g11840  lactoylglutathione lyase, putative / glyoxalase I, putative highly similar to putative lactoylglutathione lyase SP:Q39366 from [Brassica oleracea] | | | | | | |
|  |  | RAFL09-12-J06 | At1g52380 / expressed protein | |  |  |  |  |  | | --- | --- | --- | --- | --- | |  |  |  |  |  | | RAFL09-12-J06 ,At1g52380  Ran-binding protein 1 domain-containing protein / RanBP1 domain-containing protein weak similarity to SP|Q09717 Ran-specific GTPase-activating protein 1 (Ran binding protein 1) (RANBP1) (Spi1-binding protein) {Schizosaccharomyces pombe}; contains Pfam profile PF00638: RanBP1 domain | | | | | | |
|  |  | RAFL05-20-L09 | At3g01770 / DNA-binding protein family | |  |  |  |  |  | | --- | --- | --- | --- | --- | |  |  |  |  |  | | At3g01770 ,RAFL05-20-L09  DNA-binding bromodomain-containing protein contains bromodomain, INTERPRO:IPR001487 | | | | | | |
|  |  | RAFL04-13-L15 | At1g66410 / calmodulin 4 | |  |  |  |  |  | | --- | --- | --- | --- | --- | |  |  |  |  |  | | RAFL04-13-L15 ,At1g66410  calmodulin-1/4 (CAM4) identical to calmodulin [Arabidopsis thaliana] GI:16223; nearly identical to SP|P25854 Calmodulin-1/4 {Arabidopsis thaliana} | | | | | | |
|  |  | RAFL11-11-C06 | At3g57410 / villin 3 fragment | |  |  |  |  |  | | --- | --- | --- | --- | --- | |  |  |  |  |  | | At3g57410 ,RAFL11-11-C06  villin 3 (VLN3) nearly identical to villin 3 (VLN3) [Arabidopsis thaliana] GI:3415117 | | | | | | |
|  |  | RAFL05-07-I06 | At3g14160 / oxidoreductase, 2OG-Fe(II) oxygenase family | |  |  |  |  |  | | --- | --- | --- | --- | --- | |  |  |  |  |  | | RAFL05-07-I06 ,At3g14160  oxidoreductase, 2OG-Fe(II) oxygenase family protein low similarity to alkB protein [Caulobacter crescentus][GI:2055386]; contains Pfam domain PF03171 2OG-Fe(II) oxygenase superfamily | | | | | | |
|  |  | RAFL05-10-E17 | At3g46940 / dUTP pyrophosphatase-related protein | |  |  |  |  |  | | --- | --- | --- | --- | --- | |  |  |  |  |  | | RAFL05-10-E17 ,At3g46940  deoxyuridine 5'-triphosphate nucleotidohydrolase family contains Pfam profile: PF00692 deoxyuridine 5'-triphosphate nucleotidohydrolase | | | | | | |
|  |  | RAFL07-07-F08 | At4g37550 / formamidase - like protein | |  |  |  |  |  | | --- | --- | --- | --- | --- | |  |  |  |  |  | | RAFL07-07-F08 ,At4g37550  formamidase, putative / formamide amidohydrolase, putative similar to SP|Q50228 Formamidase (EC 3.5.1.49) (Formamide amidohydrolase) {Methylophilus methylotrophus}; contains Pfam profile PF03069: Acetamidase/Formamidase family | | | | | | |
|  |  | RAFL05-09-F21 | At4g37210 / tetratricopeptide repeat (TPR)-containing protein | |  |  |  |  |  | | --- | --- | --- | --- | --- | |  |  |  |  |  | | At4g37210 ,RAFL05-09-F21  tetratricopeptide repeat (TPR)-containing protein low similarity to SP|Q02508 Protein HGV2 Halocynthia roretzi; contains Pfam profile PF00515 TPR Domain | | | | | | |
|  |  | RAFL05-11-K24 | At3g23570 / expressed protein | |  |  |  |  |  | | --- | --- | --- | --- | --- | |  |  |  |  |  | | RAFL05-11-K24 ,At3g23570  dienelactone hydrolase family protein similar to SP|Q9ZT66 Endo-1,3;1,4-beta-D-glucanase precursor (EC 3.2.1.-) {Zea mays}; contains Pfam profile: PF01738 dienelactone hydrolase family | | | | | | |
|  |  | RAFL06-07-D07 | At5g20020 / GTP-binding nuclear protein (RAN-2) | |  |  |  |  |  | | --- | --- | --- | --- | --- | |  |  |  |  |  | | RAFL06-07-D07 ,At5g20020  Ras-related GTP-binding nuclear protein (RAN-2) identical to GTP-binding nuclear protein RAN-2 SP:P41917 from [Arabidopsis thaliana] | | | | | | |
|  |  | RAFL08-18-A19 | At1g23260 / ubiquitin-conjugating enzyme family | |  |  |  |  |  | | --- | --- | --- | --- | --- | |  |  |  |  |  | | At1g23260 ,RAFL08-18-A19  ubiquitin-conjugating enzyme family protein similar to TRAF6-regulated IKK activator 1 beta Uev1A [Homo sapiens] GI:10880969; contains Pfam profile PF00179: Ubiquitin-conjugating enzyme | | | | | | |
|  |  | RAFL11-03-N11 | At5g20020 / GTP-binding nuclear protein (RAN-2) | |  |  |  |  |  | | --- | --- | --- | --- | --- | |  |  |  |  |  | | RAFL11-03-N11 ,At5g20020  Ras-related GTP-binding nuclear protein (RAN-2) identical to GTP-binding nuclear protein RAN-2 SP:P41917 from [Arabidopsis thaliana] | | | | | | |
|  |  | RAFL05-16-K03 | At4g36780 / expressed protein | |  |  |  |  |  | | --- | --- | --- | --- | --- | |  |  |  |  |  | | At4g36780 ,RAFL05-16-K03  brassinosteroid signalling positive regulator-related contains similarity to BZR1 protein [Arabidopsis thaliana] gi|20270971|gb|AAM18490 | | | | | | |
|  |  | RAFL04-09-O19 | At5g66420 / expressed protein | |  |  |  |  |  | | --- | --- | --- | --- | --- | |  |  |  |  |  | | RAFL04-09-O19 ,At5g66420  expressed protein | | | | | | |
|  |  | RAFL05-14-E20 | At1g53720 / cyclophilin-RNA interacting protein, putative | |  |  |  |  |  | | --- | --- | --- | --- | --- | |  |  |  |  |  | | At1g53720 ,RAFL05-14-E20  cyclophilin-RNA interacting protein, putative | | | | | | |
|  |  | RAFL04-17-B06 | At5g22060 / DnaJ protein, putative | |  |  |  |  |  | | --- | --- | --- | --- | --- | |  |  |  |  |  | | RAFL04-17-B06 ,At5g22060  DNAJ heat shock protein, putative strong similarity to SP|O60884 DnaJ homolog subfamily A member 2 (Dnj3) Homo sapiens, several plant DnaJ proteins from PGR; contains Pfam profiles PF00226 DnaJ domain, PF00684 DnaJ central domain (4 repeats), PF01556 DnaJ C terminal region | | | | | | |
|  |  | RAFL07-12-H05 | At2g34040 / glycine-rich protein | |  |  |  |  |  | | --- | --- | --- | --- | --- | |  |  |  |  |  | | RAFL07-12-H05 ,At2g34040  apoptosis inhibitory 5 (API5) family protein contains Pfam domain PF05918 Apoptosis inhibitory protein 5 (API5) | | | | | | |
|  |  | RAFL09-14-M02 | At3g16400 / jacalin lectin family | |  |  |  |  |  | | --- | --- | --- | --- | --- | |  |  |  |  |  | | At3g16400 ,RAFL09-14-M02  jacalin lectin family protein similar to myrosinase-binding protein homolog [Arabidopsis thaliana] GI:2997767, epithiospecifier [Arabidopsis thaliana] GI:16118845; contains Pfam profiles PF01419 jacalin-like lectin family, PF01344 Kelch motif | | | | | | |
|  |  | RAFL08-12-I24 | At3g58640 / protein kinase family | |  |  |  |  |  | | --- | --- | --- | --- | --- | |  |  |  |  |  | | RAFL08-12-I24 ,At3g58640  protein kinase family protein contains eukaryotic protein kinase domain, INTERPRO:IPR000719 | | | | | | |
|  |  | RAFL09-12-O15 | At1g54460 / expressed protein | |  |  |  |  |  | | --- | --- | --- | --- | --- | |  |  |  |  |  | | At1g54460 ,RAFL09-12-O15  expressed protein | | | | | | |
|  |  | RAFL05-07-E03 | At3g04810 / protein kinase, putative | |  |  |  |  |  | | --- | --- | --- | --- | --- | |  |  |  |  |  | | RAFL05-07-E03 ,At3g04810  protein kinase, putative similar to LSTK-1-like kinase [Lycopersicon esculentum] GI:15637110; contains Pfam profile: PF00069 Eukaryotic protein kinase domain | | | | | | |
|  |  | RAFL04-19-E01 | At1g11760 / expressed protein | |  |  |  |  |  | | --- | --- | --- | --- | --- | |  |  |  |  |  | | At1g11760 ,RAFL04-19-E01  expressed protein weak similarity to Pfam PF01648: 4'-phosphopantetheinyl transferase superfamily | | | | | | |
|  |  | RAFL06-16-J23 | At5g05970 / transducin / WD-40 repeat protein family | |  |  |  |  |  | | --- | --- | --- | --- | --- | |  |  |  |  |  | | RAFL06-16-J23 ,At5g05970  transducin family protein / WD-40 repeat family protein contains similarity to regulatory protein Nedd1; contains Pfam PF00400: WD domain, G-beta repeat (6 copies, 2 weak)|19804256|gb|AV785466.1|AV785466 | | | | | | |
|  |  | RAFL07-15-D05 | At3g23080 / expressed protein | |  |  |  |  |  | | --- | --- | --- | --- | --- | |  |  |  |  |  | | RAFL07-15-D05 ,At3g23080  expressed protein weak similarity to SP|Q9UKL6 Phosphatidylcholine transfer protein (PC-TP) {Homo sapiens} | | | | | | |
|  |  | RAFL02-10-G13 | At5g51140 / expressed protein | |  |  |  |  |  | | --- | --- | --- | --- | --- | |  |  |  |  |  | | RAFL02-10-G13 ,At5g51140  pseudouridine synthase family protein low similarity to SP|P23851 Ribosomal large subunit pseudouridine synthase C (EC 4.2.1.70) (Pseudouridylate synthase) (Uracil hydrolyase) {Escherichia coli}; contains Pfam profile PF00849: RNA pseudouridylate synthase | | | | | | |
|  |  | RAFL06-11-A21 | At5g58220 / unknown protein (pir||T34863) -related | |  |  |  |  |  | | --- | --- | --- | --- | --- | |  |  |  |  |  | | At5g58220 ,RAFL06-11-A21  expressed protein | | | | | | |
|  |  | RAFL05-05-M08 | At5g62900 / expressed protein | |  |  |  |  |  | | --- | --- | --- | --- | --- | |  |  |  |  |  | | RAFL05-05-M08 ,At5g62900  expressed protein | | | | | | |
|  |  | RAFL07-09-C05 | At2g44260 / expressed protein | |  |  |  |  |  | | --- | --- | --- | --- | --- | |  |  |  |  |  | | At2g44260 ,RAFL07-09-C05  expressed protein | | | | | | |
|  |  | RAFL09-09-K03 | At5g58110 / expressed protein | |  |  |  |  |  | | --- | --- | --- | --- | --- | |  |  |  |  |  | | RAFL09-09-K03 ,At5g58110  expressed protein predicted proteins, Homo sapiens and Drosophila melanogaster | | | | | | |
|  |  | RAFL05-09-K14 | At1g09010 / glycoside hydrolase family 2 | |  |  |  |  |  | | --- | --- | --- | --- | --- | |  |  |  |  |  | | At1g09010 ,RAFL05-09-K14  glycoside hydrolase family 2 protein low similarity to mannosidase [gi:5359712] from Cellulomonas fimi | | | | | | |
|  |  | RAFL05-14-M18 | At1g12050 / fumarylacetoacetate hydrolase-related protein | |  |  |  |  |  | | --- | --- | --- | --- | --- | |  |  |  |  |  | | At1g12050 ,RAFL05-14-M18  fumarylacetoacetase, putative similar to fumarylacetoacetase (Fumarylacetoacetate hydrolase, Beta-diketonase, FAA)[Rattus norvegicus] SWISS-PROT:P25093 | | | | | | |
|  |  | RAFL06-10-D10 | At1g27410 / expressed protein | |  |  |  |  |  | | --- | --- | --- | --- | --- | |  |  |  |  |  | | RAFL06-10-D10 ,At1g27410  DNA cross-link repair protein-related contains weak similarity to Swiss-Prot:P30620 DNA cross-LINK repair protein PSO2/SNM1 [Saccharomyces cerevisiae] | | | | | | |
|  |  | RAFL09-12-F13 | At2g17020 / F-box protein family, AtFBL10 | |  |  |  |  |  | | --- | --- | --- | --- | --- | |  |  |  |  |  | | RAFL09-12-F13 ,At2g17020  F-box family protein (FBL10) contains similarity to F-box protein Partner of Paired GI:10441427 from [Drosophila melanogaster] | | | | | | |
|  |  | RAFL06-13-M05 | At3g07170 / expressed protein | |  |  |  |  |  | | --- | --- | --- | --- | --- | |  |  |  |  |  | | At3g07170 ,RAFL06-13-M05  sterile alpha motif (SAM) domain-containing protein contains Pfam profile PF00536: SAM domain (Sterile alpha motif) | | | | | | |
|  |  | RAFL07-09-O11 | At1g61120 / terpene synthase/cyclase family | |  |  |  |  |  | | --- | --- | --- | --- | --- | |  |  |  |  |  | | RAFL07-09-O11 ,At1g61120  terpene synthase/cyclase family protein similar to S-linalool synthase GI:1491939 from [Clarkia breweri][PMID: 8768373] | | | | | | |
|  |  | RAFL05-20-P12 | At1g26470 / expressed protein | |  |  |  |  |  | | --- | --- | --- | --- | --- | |  |  |  |  |  | | At1g26470 ,RAFL05-20-P12  expressed protein | | | | | | |
|  |  | RAFL04-15-K06 | At5g59450 / scarecrow-like transcription factor 11 (SCL11) | |  |  |  |  |  | | --- | --- | --- | --- | --- | |  |  |  |  |  | | At5g59450 ,RAFL04-15-K06  scarecrow-like transcription factor 11 (SCL11) scarecrow-like 11, Arabidopsis thaliana, EMBL:AF036307 | | | | | | |
|  |  | RAFL07-17-O15 | At5g19180 / RUB-activating enzyme ECR1 | |  |  |  |  |  | | --- | --- | --- | --- | --- | |  |  |  |  |  | | At5g19180 ,RAFL07-17-O15  ubiquitin activating enzyme, putative (ECR1) identical to putative ubiquitin activating enzyme E1 [Arabidopsis thaliana] GI:2952433; similar to NEDD8 activating enzyme [Mus musculus] GI:17061821 | | | | | | |
|  |  | RAFL11-11-A11 | At5g58130 / RRM-containing protein | |  |  |  |  |  | | --- | --- | --- | --- | --- | |  |  |  |  |  | | At5g58130 ,RAFL11-11-A11  RNA recognition motif (RRM)-containing protein | | | | | | |
|  |  | RAFL05-07-E13 | At3g48210 / expressed protein | |  |  |  |  |  | | --- | --- | --- | --- | --- | |  |  |  |  |  | | At3g48210 ,RAFL05-07-E13  expressed protein | | | | | | |
|  |  | RAFL11-06-N05 | At1g07140 / Ran-binding protein (atranbp1a) | |  |  |  |  |  | | --- | --- | --- | --- | --- | |  |  |  |  |  | | RAFL11-06-N05 ,At1g07140  Ran-binding protein 1a (RanBP1a) identical to Ran-binding protein (atranbp1a) GI:2058282 from [Arabidopsis thaliana] | | | | | | |
|  |  | RAFL05-03-L14 | At3g03530 / phosphoesterase family | |  |  |  |  |  | | --- | --- | --- | --- | --- | |  |  |  |  |  | | At3g03530 ,RAFL05-03-L14  phosphoesterase family protein low similarity to SP|P95246 Phospholipase C 2 precursor (EC 3.1.4.3) {Mycobacterium tuberculosis}; contains Pfam profile PF04185: Phosphoesterase family | | | | | | |
|  |  | RAFL04-17-A02 | At1g57870 / shaggy-related protein kinase kappa, putative | |  |  |  |  |  | | --- | --- | --- | --- | --- | |  |  |  |  |  | | At1g57870 ,RAFL04-17-A02  shaggy-related protein kinase kappa, putative / ASK-kappa, putative similar to shaggy-related protein kinase kappa SP:Q39019 GI:717180 from [Arabidopsis thaliana] | | | | | | |
|  |  | RAFL11-01-B05 | At3g19515 / expressed protein | |  |  |  |  |  | | --- | --- | --- | --- | --- | |  |  |  |  |  | | RAFL11-01-B05 ,At3g19515  expressed protein | | | | | | |
|  |  | RAFL05-11-F04 | At5g27320 / expressed protein | |  |  |  |  |  | | --- | --- | --- | --- | --- | |  |  |  |  |  | | RAFL05-11-F04 ,At5g27320  expressed protein similar to PrMC3 [Pinus radiata] GI:5487873 | | | | | | |
|  |  | RAFL05-16-D21 | At1g22970 / expressed protein | |  |  |  |  |  | | --- | --- | --- | --- | --- | |  |  |  |  |  | | RAFL05-16-D21 ,At1g22970  expressed protein | | | | | | |
|  |  | RAFL11-07-C09 | At1g51730 / expressed protein | |  |  |  |  |  | | --- | --- | --- | --- | --- | |  |  |  |  |  | | RAFL11-07-C09 ,At1g51730  RWD domain-containing protein contains Pfam profile PF05773: RWD domain; similar to GCN2 eIF2alpha kinase (GI:6066585) [Mus musculus]; similar to GCN2beta (GI:10764163) [Mus musculus]; similar to RING finger protein 25 (RING finger protein AO7) (Swiss-Prot:Q9QZR0) [Mus musculus]; similar to RING finger protein 25 (Swiss-Prot:Q96BH1) [Homo sapiens] | | | | | | |
|  |  | RAFL04-13-O14 | At1g26270 / phosphatidylinositol 3- and 4-kinase family | |  |  |  |  |  | | --- | --- | --- | --- | --- | |  |  |  |  |  | | At1g26270 ,RAFL04-13-O14  phosphatidylinositol 3- and 4-kinase family protein similar to phosphatidylinositol 4-kinase type-II beta [Homo sapiens] GI:20159767; contains Pfam profile PF00454: Phosphatidylinositol 3- and 4-kinase | | | | | | |
|  |  | RAFL04-12-J09 | At3g03420 / expressed protein | |  |  |  |  |  | | --- | --- | --- | --- | --- | |  |  |  |  |  | | RAFL04-12-J09 ,At3g03420  Ku70-binding family protein similar to Ku70-binding protein GB:AAD31085 GI:4867999 [Homo sapiens]; contains Prosite PS00142: Neutral zinc metallopeptidases, zinc-binding region signature | | | | | | |
|  |  | RAFL11-05-P16 | At5g10410 / expressed protein | |  |  |  |  |  | | --- | --- | --- | --- | --- | |  |  |  |  |  | | RAFL11-05-P16 ,At5g10410  epsin N-terminal homology (ENTH) domain-containing protein / clathrin assembly protein-related contains Pfam PF01417: ENTH domain. ENTH (Epsin N-terminal homology) domain; similar to clathrin assembly protein AP180 (GI:6492344) [Xenopus laevis] | | | | | | |
|  |  | RAFL05-13-F06 | At4g28910 / expressed protein | |  |  |  |  |  | | --- | --- | --- | --- | --- | |  |  |  |  |  | | RAFL05-13-F06 ,At4g28910  expressed protein | | | | | | |
|  |  | RAFL05-08-P13 | At1g22200 / expressed protein | |  |  |  |  |  | | --- | --- | --- | --- | --- | |  |  |  |  |  | | At1g22200 ,RAFL05-08-P13  expressed protein | | | | | | |
|  |  | RAFL08-14-L02 | At5g57580 / calmodulin-binding protein | |  |  |  |  |  | | --- | --- | --- | --- | --- | |  |  |  |  |  | | RAFL08-14-L02 ,At5g57580  calmodulin-binding protein similar to calmodulin-binding protein TCB60 GI:1698548 from [Nicotiana tabacum] | | | | | | |
|  |  | RAFL03-09-A18 | At1g26270 / phosphatidylinositol 3- and 4-kinase family | |  |  |  |  |  | | --- | --- | --- | --- | --- | |  |  |  |  |  | | At1g26270 ,RAFL03-09-A18  phosphatidylinositol 3- and 4-kinase family protein similar to phosphatidylinositol 4-kinase type-II beta [Homo sapiens] GI:20159767; contains Pfam profile PF00454: Phosphatidylinositol 3- and 4-kinase | | | | | | |
|  |  | RAFL08-09-K17 | At2g44670 / senescence-associated protein -related | |  |  |  |  |  | | --- | --- | --- | --- | --- | |  |  |  |  |  | | RAFL08-09-K17 ,At2g44670  senescence-associated protein-related similar to senescence-associated protein SAG102 (GI:22331931) [Arabidopsis thaliana]; | | | | | | |
|  |  | RAFL08-11-D18 | At3g16400 / jacalin lectin family | |  |  |  |  |  | | --- | --- | --- | --- | --- | |  |  |  |  |  | | RAFL08-11-D18 ,At3g16400  jacalin lectin family protein similar to myrosinase-binding protein homolog [Arabidopsis thaliana] GI:2997767, epithiospecifier [Arabidopsis thaliana] GI:16118845; contains Pfam profiles PF01419 jacalin-like lectin family, PF01344 Kelch motif | | | | | | |
|  |  | RAFL05-08-A14 | At3g48090 / disease resistance protein (EDS1) | |  |  |  |  |  | | --- | --- | --- | --- | --- | |  |  |  |  |  | | At3g48090 ,RAFL05-08-A14  disease resistance protein (EDS1) identical to disease resistance protein/lipase homolog EDS1 GI:4454567; contains Pfam profile PF01764: Lipase | | | | | | |
|  |  | RAFL04-19-B11 | At4g30480 / tetratricopeptide repeat (TPR)-containing protein | |  |  |  |  |  | | --- | --- | --- | --- | --- | |  |  |  |  |  | | At4g30480 ,RAFL04-19-B11  tetratricopeptide repeat (TPR)-containing protein similar to SP|Q99614 Tetratricopeptide repeat protein 1 {Homo sapiens}; contains Pfam profile PF00515: TPR Domain | | | | | | |
|  |  | RAFL05-11-F20 | At4g35470 / expressed protein | |  |  |  |  |  | | --- | --- | --- | --- | --- | |  |  |  |  |  | | At4g35470 ,RAFL05-11-F20  leucine-rich repeat family protein similar to Leucine-rich repeat protein SHOC-2 (Ras-binding protein Sur-8) (SP:Q9UQ13 ){Homo sapiens},PIR:T12704; contains Pfam PF00560: Leucine Rich Repeat domains | | | | | | |
|  |  | RAFL04-17-H08 | At3g05320 / expressed protein | |  |  |  |  |  | | --- | --- | --- | --- | --- | |  |  |  |  |  | | At3g05320 ,RAFL04-17-H08  expressed protein | | | | | | |
|  |  | RAFL05-01-I13 | At3g19360 / expressed protein | |  |  |  |  |  | | --- | --- | --- | --- | --- | |  |  |  |  |  | | At3g19360 ,RAFL05-01-I13  zinc finger (CCCH-type) family protein contains Pfam domain, PF00642: Zinc finger C-x8-C-x5-C-x3-H type (and similar) | | | | | | |
|  |  | RAFL09-17-K06 | At3g16400 / jacalin lectin family | |  |  |  |  |  | | --- | --- | --- | --- | --- | |  |  |  |  |  | | At3g16400 ,RAFL09-17-K06  jacalin lectin family protein similar to myrosinase-binding protein homolog [Arabidopsis thaliana] GI:2997767, epithiospecifier [Arabidopsis thaliana] GI:16118845; contains Pfam profiles PF01419 jacalin-like lectin family, PF01344 Kelch motif | | | | | | |
|  |  | RAFL09-11-N12 | At1g09780 / 2,3-bisphosphoglycerate-independent phosphoglycerate mutase -related | |  |  |  |  |  | | --- | --- | --- | --- | --- | |  |  |  |  |  | | At1g09780 ,RAFL09-11-N12  2,3-biphosphoglycerate-independent phosphoglycerate mutase, putative / phosphoglyceromutase, putative strong similarity to SP|Q42908 2,3-bisphosphoglycerate-independent phosphoglycerate mutase (EC 5.4.2.1) (Phosphoglyceromutase) {Mesembryanthemum crystallinum}; contains Pfam profile PF01676: Metalloenzyme superfamily | | | | | | |
|  | Cluster:8-1 | |  |  | 48 | 114 | 897 | 3604 | 0.0037723402 | 0.12448723 | 33 |
|  |  | RAFL11-01-B13 | At5g47650 / MutT/nudix family protein | |  |  |  |  |  | | --- | --- | --- | --- | --- | |  |  |  |  |  | | At5g47650 ,RAFL11-01-B13  MutT/nudix family protein similar to Nucleoside diphosphate-linked moiety X motif 6 (Protein GFG) from {Xenopus laevis} SP|P13420, {Homo sapiens} SP|P53370; contains Pfam profile PF00293: NUDIX domain | | | | | | |
|  |  | RAFL03-08-K18 | At3g51920 / calmodulin 9 | |  |  |  |  |  | | --- | --- | --- | --- | --- | |  |  |  |  |  | | At3g51920 ,RAFL03-08-K18  calmodulin-9 (CAM9) identical to calmodulin 9 GI:5825602 from [Arabidopsis thaliana]; contains Pfam profile PF00036: EF hand | | | | | | |
|  |  | RAFL05-17-D19 | At1g19400 / expressed protein | |  |  |  |  |  | | --- | --- | --- | --- | --- | |  |  |  |  |  | | At1g19400 ,RAFL05-17-D19  expressed protein | | | | | | |
|  |  | RAFL04-13-A13 | At5g51830 / fructokinase, putative | |  |  |  |  |  | | --- | --- | --- | --- | --- | |  |  |  |  |  | | At5g51830 ,RAFL04-13-A13  pfkB-type carbohydrate kinase family protein contains Pfam profile: PF00294 pfkB family carbohydrate kinase | | | | | | |
|  |  | RAFL05-12-H09 | At5g65280 / G protein-coupled receptor-related protein | |  |  |  |  |  | | --- | --- | --- | --- | --- | |  |  |  |  |  | | RAFL05-12-H09 ,At5g65280  lanthionine synthetase C-like family protein contains Pfam domain, PF05147: Lanthionine synthetase C-like protein | | | | | | |
|  |  | RAFL05-07-L17 | At2g46260 / POZ domain protein family | |  |  |  |  |  | | --- | --- | --- | --- | --- | |  |  |  |  |  | | At2g46260 ,RAFL05-07-L17  BTB/POZ domain-containing protein contains Pfam PF00651: BTB/POZ domain; contains Interpro IPR000210/ PS50097: BTBB/POZ domain; similar to POZ/BTB containing-protein AtPOB1 (GI:12006855) [Arabidopsis thaliana]; similar to actinfilin (GI:21667852) [Rattus norvegicus] | | | | | | |
|  |  | RAFL05-17-O13 | At3g21230 / 4-coumarate:CoA ligase (4-coumaroyl-CoA synthase) (4CL), putative | |  |  |  |  |  | | --- | --- | --- | --- | --- | |  |  |  |  |  | | At3g21230 ,RAFL05-17-O13  4-coumarate--CoA ligase, putative / 4-coumaroyl-CoA synthase, putative (4CL) similar to 4CL2 [gi:12229665] and 4CL1 [gi:12229649] from [Arabidopsis thaliana], 4CL1 [gi:12229631] from Nicotiana tabacum | | | | | | |
|  |  | RAFL11-01-P18 | At4g31770 / calcineurin-like phosphoesterase family | |  |  |  |  |  | | --- | --- | --- | --- | --- | |  |  |  |  |  | | At4g31770 ,RAFL11-01-P18  calcineurin-like phosphoesterase family protein contains Pfam profile: PF00149 calcineurin-like phosphoesterase | | | | | | |
|  |  | RAFL05-08-H13 | At1g76710 / SET-domain transcriptional regulator family | |  |  |  |  |  | | --- | --- | --- | --- | --- | |  |  |  |  |  | | RAFL05-08-H13 ,At1g76710  SET domain-containing protein (ASHH1) low similarity to huntingtin interacting protein 1 [Homo sapiens] GI:12697196; contains Pfam profile PF00856: SET domain; identical to cDNA ASH1-like protein 1 (ASHH1) partial cds GI:15488417 | | | | | | |
|  |  | RAFL05-11-G07 | At1g15740 / leucine rich repeat protein-related | |  |  |  |  |  | | --- | --- | --- | --- | --- | |  |  |  |  |  | | RAFL05-11-G07 ,At1g15740  leucine-rich repeat family protein | | | | | | |
|  |  | RAFL05-19-O11 | At4g29190 / expressed protein | |  |  |  |  |  | | --- | --- | --- | --- | --- | |  |  |  |  |  | | At4g29190 ,RAFL05-19-O11  zinc finger (CCCH-type) family protein contains Pfam domain, PF00642: Zinc finger C-x8-C-x5-C-x3-H type (and similar) | | | | | | |
|  |  | RAFL04-17-K07 | At3g48530 / CBS domain containing protein | |  |  |  |  |  | | --- | --- | --- | --- | --- | |  |  |  |  |  | | At3g48530 ,RAFL04-17-K07  CBS domain-containing protein low similarity to SP|Q9UGI9 5'-AMP-activated protein kinase, gamma-3 subunit (AMPK gamma-3 chain) (AMPK gamma3) {Homo sapiens}; contains Pfam profile PF00571: CBS domain | | | | | | |
|  |  | RAFL05-08-G05 | At5g45900 / autophagy 7 (APG7) | |  |  |  |  |  | | --- | --- | --- | --- | --- | |  |  |  |  |  | | RAFL05-08-G05 ,At5g45900  autophagy 7 (APG7) nearly identical to autophagy 7 [Arabidopsis thaliana] GI:19912147; contains Pfam profile PF00899: ThiF family | | | | | | |
|  |  | RAFL04-13-L04 | At5g21990 / expressed protein | |  |  |  |  |  | | --- | --- | --- | --- | --- | |  |  |  |  |  | | At5g21990 ,RAFL04-13-L04  tetratricopeptide repeat (TPR)-containing protein contains Pfam profile PF00515: TPR Domain | | | | | | |
|  |  | RAFL05-14-B17 | At4g29160 / expressed protein | |  |  |  |  |  | | --- | --- | --- | --- | --- | |  |  |  |  |  | | At4g29160 ,RAFL05-14-B17  SNF7 family protein contains Pfam domain, PF03357: SNF7 family | | | | | | |
|  |  | RAFL05-16-H09 | At1g29690 / expressed protein | |  |  |  |  |  | | --- | --- | --- | --- | --- | |  |  |  |  |  | | RAFL05-16-H09 ,At1g29690  expressed protein | | | | | | |
|  |  | RAFL05-07-A18 | At2g23320 / WRKY family transcription factor | |  |  |  |  |  | | --- | --- | --- | --- | --- | |  |  |  |  |  | | At2g23320 ,RAFL05-07-A18  WRKY family transcription factor identical to WRKY DNA-binding protein 15 GI:13506742 from [Arabidopsis thaliana] | | | | | | |
|  |  | RAFL05-13-A18 | At2g47710 / expressed protein | |  |  |  |  |  | | --- | --- | --- | --- | --- | |  |  |  |  |  | | RAFL05-13-A18 ,At2g47710  universal stress protein (USP) family protein similar to ER6 protein [Lycopersicon esculentum] GI:5669654; contains Pfam profile PF00582: universal stress protein family | | | | | | |
|  |  | RAFL05-08-G06 | At5g17380 / 2-hydroxyphytanoyl-CoA lyase-related protein | |  |  |  |  |  | | --- | --- | --- | --- | --- | |  |  |  |  |  | | RAFL05-08-G06 ,At5g17380  pyruvate decarboxylase family protein similar to 2-hydroxyphytanoyl-CoA lyase [Homo sapiens] GI:6273457; contains InterPro entry IPR000399: Pyruvate decarboxylase | | | | | | |
|  |  | RAFL05-08-C18 | At1g53400 / expressed protein | |  |  |  |  |  | | --- | --- | --- | --- | --- | |  |  |  |  |  | | RAFL05-08-C18 ,At1g53400  expressed protein | | | | | | |
|  |  | RAFL05-08-C07 | At3g02140 / expressed protein | |  |  |  |  |  | | --- | --- | --- | --- | --- | |  |  |  |  |  | | RAFL05-08-C07 ,At3g02140  expressed protein | | | | | | |
|  |  | RAFL05-07-H19 | At5g13190 / expressed protein | |  |  |  |  |  | | --- | --- | --- | --- | --- | |  |  |  |  |  | | RAFL05-07-H19 ,At5g13190  expressed protein | | | | | | |
|  |  | RAFL04-17-A20 | At3g52800 / zinc finger - like protein | |  |  |  |  |  | | --- | --- | --- | --- | --- | |  |  |  |  |  | | At3g52800 ,RAFL04-17-A20  zinc finger (AN1-like) family protein contains Pfam domain, PF01428: AN1-like Zinc finger | | | | | | |
|  |  | RAFL05-09-G04 | At1g79340 / putative latex-abundant protein | |  |  |  |  |  | | --- | --- | --- | --- | --- | |  |  |  |  |  | | At1g79340 ,RAFL05-09-G04  latex-abundant protein, putative (AMC7) / caspase family protein similar to latex-abundant protein [Hevea brasiliensis] gb:AAD13216; contains Pfam domain, PF00656: ICE-like protease (caspase) p20 domain | | | | | | |
|  |  | RAFL05-19-N02 | At5g53800 / expressed protein | |  |  |  |  |  | | --- | --- | --- | --- | --- | |  |  |  |  |  | | At5g53800 ,RAFL05-19-N02  expressed protein | | | | | | |
|  |  | RAFL09-07-D10 | At5g22350 / expressed protein | |  |  |  |  |  | | --- | --- | --- | --- | --- | |  |  |  |  |  | | RAFL09-07-D10 ,At5g22350  expressed protein | | | | | | |
|  |  | RAFL05-17-N23 | At5g52200 / expressed protein | |  |  |  |  |  | | --- | --- | --- | --- | --- | |  |  |  |  |  | | RAFL05-17-N23 ,At5g52200  expressed protein | | | | | | |
|  |  | RAFL05-01-K10 | At1g11480 / expressed protein | |  |  |  |  |  | | --- | --- | --- | --- | --- | |  |  |  |  |  | | RAFL05-01-K10 ,At1g11480  eukaryotic translation initiation factor-related contains weak similarity to Swiss-Prot:P23588 eukaryotic translation initiation factor 4B (eIF-4B) [Homo sapiens] | | | | | | |
|  |  | RAFL08-17-C10 | At1g67580 / protein kinase family | |  |  |  |  |  | | --- | --- | --- | --- | --- | |  |  |  |  |  | | At1g67580 ,RAFL08-17-C10  protein kinase family protein contains protein kinase domain, Pfam:PF00069 | | | | | | |
|  |  | RAFL07-10-J07 | At4g31550 / WRKY family transcription factor | |  |  |  |  |  | | --- | --- | --- | --- | --- | |  |  |  |  |  | | RAFL07-10-J07 ,At4g31550  WRKY family transcription factor contains Pfam profile: PF03106 WRKY DNA -binding domain | | | | | | |
|  |  | RAFL11-12-J10 | At2g32800 / protein kinase family | |  |  |  |  |  | | --- | --- | --- | --- | --- | |  |  |  |  |  | | RAFL11-12-J10 ,At2g32800  protein kinase family protein contains dual protein kinase domains, Pfam:PF00069 | | | | | | |
|  |  | RAFL03-05-K01 | At3g04610 / KH domain protein | |  |  |  |  |  | | --- | --- | --- | --- | --- | |  |  |  |  |  | | RAFL03-05-K01 ,At3g04610  KH domain-containing protein similar putative nucleic acid binding protein GB:CAB39665 [Arabidopsis thaliana]; Pfam HMM hit: KH domain family of RNA binding proteins | | | | | | |
|  |  | RAFL06-09-G02 | At5g11680 / expressed protein | |  |  |  |  |  | | --- | --- | --- | --- | --- | |  |  |  |  |  | | RAFL06-09-G02 ,At5g11680  expressed protein predicted proteins, Arabidopsis thaliana | | | | | | |
|  |  | RAFL09-09-M22 | At2g45600 / expressed protein | |  |  |  |  |  | | --- | --- | --- | --- | --- | |  |  |  |  |  | | At2g45600 ,RAFL09-09-M22  expressed protein low similarity to PrMC3 [Pinus radiata] GI:5487873 | | | | | | |
|  |  | RAFL04-13-M21 | At2g02870 / Kelch repeat containing F-box protein family | |  |  |  |  |  | | --- | --- | --- | --- | --- | |  |  |  |  |  | | At2g02870 ,RAFL04-13-M21  kelch repeat-containing F-box family protein weak similarity to Kelch-like protein 5 (Swiss-Prot:Q96PQ7) [Homo sapiens]; contains Pfam profiles PF01344: Kelch motif, PF00646: F-box domain | | | | | | |
|  |  | RAFL05-01-M12 | At1g28330 / dormancy-associated protein -related | |  |  |  |  |  | | --- | --- | --- | --- | --- | |  |  |  |  |  | | RAFL05-01-M12 ,At1g28330  dormancy-associated protein, putative (DRM1) identical to dormancy-associated protein [Arabidopsis thaliana] GI:2995990; similar to dormancy-associated protein GI:2605887 from [Pisum sativum]; contains Pfam profile PF05564: Dormancy/auxin associated protein | | | | | | |
|  |  | RAFL04-16-O21 | At5g24530 / oxidoreductase, 2OG-Fe(II) oxygenase family | |  |  |  |  |  | | --- | --- | --- | --- | --- | |  |  |  |  |  | | At5g24530 ,RAFL04-16-O21  oxidoreductase, 2OG-Fe(II) oxygenase family protein similar to flavanone 3-hydroxylase [Persea americana][GI:727410]; contains PF03171 2OG-Fe(II) oxygenase superfamily domain | | | | | | |
|  |  | RAFL09-15-J03 | At4g29160 / expressed protein | |  |  |  |  |  | | --- | --- | --- | --- | --- | |  |  |  |  |  | | RAFL09-15-J03 ,At4g29160  SNF7 family protein contains Pfam domain, PF03357: SNF7 family | | | | | | |
|  |  | RAFL09-16-C16 | At3g12570 / expressed protein | |  |  |  |  |  | | --- | --- | --- | --- | --- | |  |  |  |  |  | | At3g12570 ,RAFL09-16-C16  expressed protein | | | | | | |
|  |  | RAFL04-19-O12 | At3g57340 / DnaJ protein family | |  |  |  |  |  | | --- | --- | --- | --- | --- | |  |  |  |  |  | | RAFL04-19-O12 ,At3g57340  DNAJ heat shock N-terminal domain-containing protein similar to SP|Q9QYI4 DnaJ homolog subfamily B member 12 Mus musculus; contains Pfam profile PF00226 DnaJ domain | | | | | | |
|  |  | RAFL05-19-M23 | At1g11480 / expressed protein | |  |  |  |  |  | | --- | --- | --- | --- | --- | |  |  |  |  |  | | At1g11480 ,RAFL05-19-M23  eukaryotic translation initiation factor-related contains weak similarity to Swiss-Prot:P23588 eukaryotic translation initiation factor 4B (eIF-4B) [Homo sapiens] | | | | | | |
|  |  | RAFL05-19-P23 | At5g02040 / expressed protein | |  |  |  |  |  | | --- | --- | --- | --- | --- | |  |  |  |  |  | | At5g02040 ,RAFL05-19-P23  prenylated rab acceptor (PRA1) family protein contains Pfam PF03208: PRA1 family protein | | | | | | |
|  |  | RAFL08-11-H15 | At3g50110 / tyrosine phosphatase -related | |  |  |  |  |  | | --- | --- | --- | --- | --- | |  |  |  |  |  | | At3g50110 ,RAFL08-11-H15  phosphatase-related similar to PTEN1 GI:5566292 from [Drosophila melanogaster]; contains prosite evidence: PS00383: Tyrosine specific protein phosphatases active site | | | | | | |
|  |  | RAFL05-13-E18 | At3g13200 / expressed protein | |  |  |  |  |  | | --- | --- | --- | --- | --- | |  |  |  |  |  | | At3g13200 ,RAFL05-13-E18  Cwf15 / Cwc15 cell cycle control family protein contains Pfam profile PF04889: Cwf15/Cwc15 cell cycle control protein; similar to Cell cycle control protein cwf15 (Swiss-Prot:P78794) [Schizosaccharomyces pombe] | | | | | | |
|  |  | RAFL09-10-G07 | At4g18950 / protein kinase - like protein | |  |  |  |  |  | | --- | --- | --- | --- | --- | |  |  |  |  |  | | At4g18950 ,RAFL09-10-G07  ankyrin protein kinase, putative similar to ankyrin-kinase [Medicago truncatula] gi|18700701|gb|AAL78674 | | | | | | |
|  |  | RAFL04-17-A15 | At1g68580 / expressed protein | |  |  |  |  |  | | --- | --- | --- | --- | --- | |  |  |  |  |  | | At1g68580 ,RAFL04-17-A15  agenet domain-containing protein / bromo-adjacent homology (BAH) domain-containing protein contains Pfam profile PF01426: BAH domain and PF05641: Agenet domain | | | | | | |
|  |  | RAFL05-09-B17 | At2g45980 / expressed protein | |  |  |  |  |  | | --- | --- | --- | --- | --- | |  |  |  |  |  | | RAFL05-09-B17 ,At2g45980  expressed protein | | | | | | |
|  |  | RAFL09-17-I16 | At1g15740 / leucine rich repeat protein-related | |  |  |  |  |  | | --- | --- | --- | --- | --- | |  |  |  |  |  | | RAFL09-17-I16 ,At1g15740  leucine-rich repeat family protein | | | | | | |
|  | Cluster:8-0 | |  |  | 33 | 76 | 912 | 3642 | 0.011158572 | 0.36823285 | 33 |
|  |  | RAFL08-10-H06 | At3g44300 / nitrilase 2 | |  |  |  |  |  | | --- | --- | --- | --- | --- | |  |  |  |  |  | | RAFL08-10-H06 ,At3g44300  nitrilase 2 (NIT2) identical to SP|P32962 Nitrilase 2 (EC 3.5.5.1) {Arabidopsis thaliana} | | | | | | |
|  |  | RAFL05-13-M13 | At1g73680 / feebly-related protein | |  |  |  |  |  | | --- | --- | --- | --- | --- | |  |  |  |  |  | | RAFL05-13-M13 ,At1g73680  pathogen-responsive alpha-dioxygenase, putative similar to pathogen-inducible alpha-dioxygenase [Nicotiana attenuata] GI:12539609; contains Pfam profile PF03098: Animal haem peroxidase | | | | | | |
|  |  | RAFL11-05-A02 | At1g27300 / expressed protein | |  |  |  |  |  | | --- | --- | --- | --- | --- | |  |  |  |  |  | | At1g27300 ,RAFL11-05-A02  expressed protein | | | | | | |
|  |  | RAFL05-05-D20 | At5g22290 / No apical meristem (NAM) protein family | |  |  |  |  |  | | --- | --- | --- | --- | --- | |  |  |  |  |  | | RAFL05-05-D20 ,At5g22290  no apical meristem (NAM) family protein contains Pfam PF02365: No apical meristem (NAM) domain | | | | | | |
|  |  | RAFL08-08-L16 | At5g39590 / expressed protein | |  |  |  |  |  | | --- | --- | --- | --- | --- | |  |  |  |  |  | | RAFL08-08-L16 ,At5g39590  expressed protein | | | | | | |
|  |  | RAFL09-09-E18 | At2g32090 / glyoxalase family protein (lactoylglutathione lyase family protein) | |  |  |  |  |  | | --- | --- | --- | --- | --- | |  |  |  |  |  | | RAFL09-09-E18 ,At2g32090  lactoylglutathione lyase family protein / glyoxalase I family protein contains glyoxalase family protein domain, Pfam:PF00903 | | | | | | |
|  |  | RAFL07-10-M07 | At5g53970 / aminotransferase, putative | |  |  |  |  |  | | --- | --- | --- | --- | --- | |  |  |  |  |  | | RAFL07-10-M07 ,At5g53970  aminotransferase, putative similar to nicotianamine aminotransferase from Hordeum vulgare [GI:6498122, GI:6469087]; contains Pfam profile PF00155 aminotransferase, classes I and II | | | | | | |
|  |  | RAFL08-09-C10 | At3g14560 / expressed protein | |  |  |  |  |  | | --- | --- | --- | --- | --- | |  |  |  |  |  | | At3g14560 ,RAFL08-09-C10  expressed protein | | | | | | |
|  |  | RAFL05-09-B02 | At5g53970 / aminotransferase, putative | |  |  |  |  |  | | --- | --- | --- | --- | --- | |  |  |  |  |  | | At5g53970 ,RAFL05-09-B02  aminotransferase, putative similar to nicotianamine aminotransferase from Hordeum vulgare [GI:6498122, GI:6469087]; contains Pfam profile PF00155 aminotransferase, classes I and II | | | | | | |
|  |  | RAFL05-13-B03 | At5g43850 / expressed protein | |  |  |  |  |  | | --- | --- | --- | --- | --- | |  |  |  |  |  | | RAFL05-13-B03 ,At5g43850  acireductone dioxygenase (ARD/ARD') family protein similar to iron-deficiency induced gene [Hordeum vulgare] GI:14522834, SIPL [Homo sapiens] GI:16551383; contains Pfam profile PF03079: ARD/ARD' family | | | | | | |
|  |  | RAFL11-01-A10 | At3g62650 / expressed protein | |  |  |  |  |  | | --- | --- | --- | --- | --- | |  |  |  |  |  | | RAFL11-01-A10 ,At3g62650  expressed protein putative mitochondrial carrier protein At2g47490 - Arabidopsis thaliana, EMBL:AC002535 | | | | | | |
|  |  | RAFL02-09-O22 | At1g03610 / expressed protein | |  |  |  |  |  | | --- | --- | --- | --- | --- | |  |  |  |  |  | | RAFL02-09-O22 ,At1g03610  expressed protein | | | | | | |
|  |  | RAFL05-19-D06 | At5g39590 / expressed protein | |  |  |  |  |  | | --- | --- | --- | --- | --- | |  |  |  |  |  | | At5g39590 ,RAFL05-19-D06  expressed protein | | | | | | |
|  |  | RAFL07-17-B18 | At5g56750 / Ndr family | |  |  |  |  |  | | --- | --- | --- | --- | --- | |  |  |  |  |  | | RAFL07-17-B18 ,At5g56750  Ndr family protein similar to SP|O23969 Pollen specific protein SF21 {Helianthus annuus}; contains Pfam profile PF03096: Ndr family | | | | | | |
|  |  | RAFL08-15-L13 | At3g48690 / expressed protein | |  |  |  |  |  | | --- | --- | --- | --- | --- | |  |  |  |  |  | | At3g48690 ,RAFL08-15-L13  expressed protein similar to PrMC3 [Pinus radiata] GI:5487873 | | | | | | |
|  |  | RAFL05-21-N06 | At4g13010 / oxidoreductase, zinc-binding dehydrogenase family | |  |  |  |  |  | | --- | --- | --- | --- | --- | |  |  |  |  |  | | At4g13010 ,RAFL05-21-N06  oxidoreductase, zinc-binding dehydrogenase family protein low similarity to probable NADP-dependent oxidoreductase (zeta-crystallin homolog) P1 [SP|Q39172][gi:886428] and P2 [SP|Q39173][gi:886430]; contains Pfam profile PF00107: oxidoreductase, zinc-binding dehydrogenase family | | | | | | |
|  |  | RAFL05-05-N17 | At5g41040 / transferase family | |  |  |  |  |  | | --- | --- | --- | --- | --- | |  |  |  |  |  | | At5g41040 ,RAFL05-05-N17  transferase family protein similar to hypersensitivity-related gene product HSR201 - Nicotiana tabacum, EMBL:X95343; contains Pfam transferase family domain PF00248 | | | | | | |
|  |  | RAFL02-09-F24 | At5g66170 / senescence-associated protein | |  |  |  |  |  | | --- | --- | --- | --- | --- | |  |  |  |  |  | | RAFL02-09-F24 ,At5g66170  senescence-associated family protein contains similarity to ketoconazole resistant protein GI:928938 and senescence-associated protein GI:1046268 from [Arabidopsis thaliana] | | | | | | |
|  |  | RAFL04-19-E09 | At5g23750 / expressed protein | |  |  |  |  |  | | --- | --- | --- | --- | --- | |  |  |  |  |  | | At5g23750 ,RAFL04-19-E09  remorin family protein contains Pfam domain, PF03766: Remorin, N-terminal region; contains Pfam domain, PF03763: Remorin, C-terminal region | | | | | | |
|  |  | RAFL08-13-I12 | At5g56380 / expressed protein | |  |  |  |  |  | | --- | --- | --- | --- | --- | |  |  |  |  |  | | At5g56380 ,RAFL08-13-I12  F-box family protein similar to unknown protein (emb|CAB62440.1); contains Pfam profile PF00646: F-box domain | | | | | | |
|  |  | RAFL06-13-E03 | At3g44300 / nitrilase 2 | |  |  |  |  |  | | --- | --- | --- | --- | --- | |  |  |  |  |  | | RAFL06-13-E03 ,At3g44300  nitrilase 2 (NIT2) identical to SP|P32962 Nitrilase 2 (EC 3.5.5.1) {Arabidopsis thaliana} | | | | | | |
|  |  | RAFL08-19-L12 | At2g01540 / C2 domain-containing protein | |  |  |  |  |  | | --- | --- | --- | --- | --- | |  |  |  |  |  | | RAFL08-19-L12 ,At2g01540  C2 domain-containing protein similar to zinc finger and C2 domain protein GI:9957238 from [Arabidopsis thaliana] | | | | | | |
|  |  | RAFL07-11-G07 | At5g63190 / MA3 domain-containing protein | |  |  |  |  |  | | --- | --- | --- | --- | --- | |  |  |  |  |  | | At5g63190 ,RAFL07-11-G07  MA3 domain-containing protein low similarity to programmed cell death 4 protein [Gallus gallus] GI:12958564; contains Pfam profile PF02847: MA3 domain | | | | | | |
|  |  | RAFL05-15-C16 | At4g29820 / mRNA cleavage factor subunit - like protein | |  |  |  |  |  | | --- | --- | --- | --- | --- | |  |  |  |  |  | | At4g29820 ,RAFL05-15-C16  expressed protein | | | | | | |
|  |  | RAFL08-13-B15 | At3g01590 / aldose 1-epimerase family | |  |  |  |  |  | | --- | --- | --- | --- | --- | |  |  |  |  |  | | RAFL08-13-B15 ,At3g01590  aldose 1-epimerase family protein similar to apospory-associated protein C; APOC [Chlamydomonas reinhardtii] GI:6970044 Pfam profile PF01263: Aldose 1-epimerase | | | | | | |
|  |  | RAFL04-10-G08 | At5g63190 / MA3 domain-containing protein | |  |  |  |  |  | | --- | --- | --- | --- | --- | |  |  |  |  |  | | At5g63190 ,RAFL04-10-G08  MA3 domain-containing protein low similarity to programmed cell death 4 protein [Gallus gallus] GI:12958564; contains Pfam profile PF02847: MA3 domain | | | | | | |
|  |  | RAFL09-06-N19 | At3g57540 / expressed protein | |  |  |  |  |  | | --- | --- | --- | --- | --- | |  |  |  |  |  | | RAFL09-06-N19 ,At3g57540  remorin family protein contains Pfam domain, PF03763: Remorin, C-terminal region | | | | | | |
|  |  | RAFL07-14-G13 | At4g24220 / expressed protein | |  |  |  |  |  | | --- | --- | --- | --- | --- | |  |  |  |  |  | | RAFL07-14-G13 ,At4g24220  expressed protein protein induced upon wounding - Arabidopsis thaliana, PID:e257749 | | | | | | |
|  |  | RAFL09-13-J20 | At1g69410 / Eukaryotic initiation factor 5A -related | |  |  |  |  |  | | --- | --- | --- | --- | --- | |  |  |  |  |  | | RAFL09-13-J20 ,At1g69410  eukaryotic translation initiation factor 5A, putative / eIF-5A, putative strong similarity to eukaryotic initiation factor 5A (2) (Nicotiana plumbaginifolia) GI:19702, SP|Q9AXQ6| Eukaryotic translation initiation factor 5A-1 (eIF-5A 1) {Lycopersicon esculentum} | | | | | | |
|  |  | RAFL05-03-P08 | At3g09390 / metallothionein-related protein | |  |  |  |  |  | | --- | --- | --- | --- | --- | |  |  |  |  |  | | At3g09390 ,RAFL05-03-P08  metallothionein protein, putative (MT2A) identical to Swiss-Prot:P25860 metallothionein-like protein 2A (MT-2A) (MT-K) (MT-1G) [Arabidopsis thaliana] | | | | | | |
|  |  | RAFL09-07-A12 | At5g10930 / CBL-interacting protein kinase 5 | |  |  |  |  |  | | --- | --- | --- | --- | --- | |  |  |  |  |  | | RAFL09-07-A12 ,At5g10930  CBL-interacting protein kinase 5 (CIPK5) identical to CBL-interacting protein kinase 5 GP|9280632|gb|AAF86504 [Arabidopsis thaliana] | | | | | | |
|  |  | RAFL08-11-M15 | At1g69360 / expressed protein | |  |  |  |  |  | | --- | --- | --- | --- | --- | |  |  |  |  |  | | RAFL08-11-M15 ,At1g69360  expressed protein | | | | | | |
|  |  | RAFL05-18-O03 | At3g21270 / Dof zinc finger protein | |  |  |  |  |  | | --- | --- | --- | --- | --- | |  |  |  |  |  | | At3g21270 ,RAFL05-18-O03  Dof-type zinc finger domain-containing protein (ADOF2) identical to Dof zinc finger protein ADOF2 GI:3608263 from [Arabidopsis thaliana]; identical to cDNA adof2 mRNA for Dof zinc finger protein GI:3608262; contains Pfam profile PF02701: Dof domain, zinc finger | | | | | | |
|  | Cluster:10-2 | |  |  | 33 | 76 | 912 | 3642 | 0.011158572 | 0.36823285 | 33 |
|  |  | RAFL02-10-M19 | At2g43760 / molybdopterin synthase large subunit -related | |  |  |  |  |  | | --- | --- | --- | --- | --- | |  |  |  |  |  | | RAFL02-10-M19 ,At2g43760  molybdopterin biosynthesis MoaE family protein contains Pfam profile: PF02391 molybdopterin converting factor, subunit 2 | | | | | | |
|  |  | RAFL11-09-C12 | At1g07600 / metallothionein-related protein | |  |  |  |  |  | | --- | --- | --- | --- | --- | |  |  |  |  |  | | At1g07600 ,RAFL11-09-C12  metallothionein-like protein 1A (MT-1A) (MT-Q) (MT-2) identical to Metallothionein-like protein 1A (MT-1A) (MT-Q) (MT-2) SP:P43392 from (Arabidopsis thaliana) | | | | | | |
|  |  | RAFL02-09-E22 | At1g54290 / protein translation factor Sui1 homolog -related | |  |  |  |  |  | | --- | --- | --- | --- | --- | |  |  |  |  |  | | RAFL02-09-E22 ,At1g54290  eukaryotic translation initiation factor SUI1, putative similar to P|P32911 Protein translation factor SUI1 {Saccharomyces cerevisiae}; contains Pfam profile PF01253: Translation initiation factor SUI1 | | | | | | |
|  |  | RAFL06-09-D22 | At3g06780 / glycine-rich protein | |  |  |  |  |  | | --- | --- | --- | --- | --- | |  |  |  |  |  | | At3g06780 ,RAFL06-09-D22  glycine-rich protein | | | | | | |
|  |  | RAFL09-15-D18 | At4g30960 / CBL-interacting protein kinase 6 | |  |  |  |  |  | | --- | --- | --- | --- | --- | |  |  |  |  |  | | RAFL09-15-D18 ,At4g30960  CBL-interacting protein kinase 6 (CIPK6) identical to CBL-interacting protein kinase 6 [Arabidopsis thaliana] gi|9280634|gb|AAF86505 | | | | | | |
|  |  | RAFL05-01-L22 | At1g68620 / expressed protein | |  |  |  |  |  | | --- | --- | --- | --- | --- | |  |  |  |  |  | | RAFL05-01-L22 ,At1g68620  expressed protein similar to PrMC3 [Pinus radiata] GI:5487873 | | | | | | |
|  |  | RAFL02-07-F13 | At3g51130 / expressed protein | |  |  |  |  |  | | --- | --- | --- | --- | --- | |  |  |  |  |  | | RAFL02-07-F13 ,At3g51130  expressed protein contains Pfam PF03676: Uncharacterised protein family (UPF0183) | | | | | | |
|  |  | RAFL11-01-F16 | At1g07600 / metallothionein-related protein | |  |  |  |  |  | | --- | --- | --- | --- | --- | |  |  |  |  |  | | At1g07600 ,RAFL11-01-F16  metallothionein-like protein 1A (MT-1A) (MT-Q) (MT-2) identical to Metallothionein-like protein 1A (MT-1A) (MT-Q) (MT-2) SP:P43392 from (Arabidopsis thaliana) | | | | | | |
|  |  | RAFL03-03-L03 | At5g35460 / expressed protein | |  |  |  |  |  | | --- | --- | --- | --- | --- | |  |  |  |  |  | | At5g35460 ,RAFL03-03-L03  expressed protein | | | | | | |
|  |  | RAFL09-06-C22 | At3g48990 / AMP-dependent synthetase and ligase family | |  |  |  |  |  | | --- | --- | --- | --- | --- | |  |  |  |  |  | | At3g48990 ,RAFL09-06-C22  AMP-dependent synthetase and ligase family protein similar to peroxisomal-coenzyme A synthetase (FAT2) [gi:586339] from Saccharomyces cerevisiae; contains Pfam AMP-binding enzyme domain PF00501; identical to cDNA; identical to cDNA adenosine monophosphate binding protein 3 AMPBP3 (AMPBP3)GI:20799714 | | | | | | |
|  |  | RAFL08-15-A08 | At4g34710 / arginine decarboxylase SPE2 | |  |  |  |  |  | | --- | --- | --- | --- | --- | |  |  |  |  |  | | RAFL08-15-A08 ,At4g34710  arginine decarboxylase 2 (SPE2) identical to SP|O23141 Arginine decarboxylase 2 (EC 4.1.1.19) (ARGDC 2) (ADC 2) (ADC-N) {Arabidopsis thaliana} | | | | | | |
|  |  | RAFL05-02-O23 | At3g46450 / SEC14 cytosolic factor, putative | |  |  |  |  |  | | --- | --- | --- | --- | --- | |  |  |  |  |  | | RAFL05-02-O23 ,At3g46450  SEC14 cytosolic factor family protein / phosphoglyceride transfer family protein contains Pfam PF00650 : CRAL/TRIO domain; similar to polyphosphoinositide binding protein Ssh1p (GI:|2739044) {Glycine max} | | | | | | |
|  |  | RAFL09-10-N09 | At4g11570 / haloacid dehalogenase-like hydrolase family | |  |  |  |  |  | | --- | --- | --- | --- | --- | |  |  |  |  |  | | RAFL09-10-N09 ,At4g11570  haloacid dehalogenase-like hydrolase family protein similar to genetic modifier [Zea mays] GI:10444400; contains InterPro accession IPR005834: Haloacid dehalogenase-like hydrolase | | | | | | |
|  |  | RAFL09-16-G02 | At1g23440 / expressed protein | |  |  |  |  |  | | --- | --- | --- | --- | --- | |  |  |  |  |  | | RAFL09-16-G02 ,At1g23440  pyrrolidone-carboxylate peptidase family protein similar to Pyrrolidone-carboxylate peptidase (Swiss-Prot:O58321) [Pyrococcus horikoshii]; contains Prosite PS00141: Eukaryotic and viral aspartyl proteases active site | | | | | | |
|  |  | RAFL06-09-G08 | At5g58070 / outer membrane lipo protein - like | |  |  |  |  |  | | --- | --- | --- | --- | --- | |  |  |  |  |  | | At5g58070 ,RAFL06-09-G08  lipocalin, putative similar to temperature stress-induced lipocalin [Triticum aestivum] GI:18650668 | | | | | | |
|  |  | RAFL06-12-A17 | At3g24740 / expressed protein | |  |  |  |  |  | | --- | --- | --- | --- | --- | |  |  |  |  |  | | RAFL06-12-A17 ,At3g24740  expressed protein | | | | | | |
|  |  | RAFL09-18-I05 | At5g09620 / octicosapeptide/Phox/Bem1p (PB1) domain-containing protein | |  |  |  |  |  | | --- | --- | --- | --- | --- | |  |  |  |  |  | | At5g09620 ,RAFL09-18-I05  octicosapeptide/Phox/Bem1p (PB1) domain-containing protein predicted proteins, Arabidopsis thaliana and Drosophila melanogaster contains Pfam profile PF00564: PB1 domain | | | | | | |
|  |  | RAFL05-20-B01 | At2g40000 / nematode-resistance protein -related | |  |  |  |  |  | | --- | --- | --- | --- | --- | |  |  |  |  |  | | At2g40000 ,RAFL05-20-B01  expressed protein | | | | | | |
|  |  | RAFL05-17-C10 | At4g03420 / expressed protein | |  |  |  |  |  | | --- | --- | --- | --- | --- | |  |  |  |  |  | | At4g03420 ,RAFL05-17-C10  expressed protein | | | | | | |
|  |  | RAFL05-08-H09 | At1g28200 / GRAM domain-containing protein | |  |  |  |  |  | | --- | --- | --- | --- | --- | |  |  |  |  |  | | RAFL05-08-H09 ,At1g28200  GRAM domain-containing protein / ABA-responsive protein-related similar to ABA-responsive protein [Hordeum vulgare] GI:4103635; contains Pfam profile PF02893: GRAM domain | | | | | | |
|  |  | RAFL08-10-C08 | At5g62540 / ubiquitin-conjugating enzyme 3 (UBC3) | |  |  |  |  |  | | --- | --- | --- | --- | --- | |  |  |  |  |  | | RAFL08-10-C08 ,At5g62540  ubiquitin-conjugating enzyme 3 (UBC3) E2; identical to gi:431261, SP:P42746 | | | | | | |
|  |  | RAFL08-12-E20 | At3g48990 / AMP-dependent synthetase and ligase family | |  |  |  |  |  | | --- | --- | --- | --- | --- | |  |  |  |  |  | | At3g48990 ,RAFL08-12-E20  AMP-dependent synthetase and ligase family protein similar to peroxisomal-coenzyme A synthetase (FAT2) [gi:586339] from Saccharomyces cerevisiae; contains Pfam AMP-binding enzyme domain PF00501; identical to cDNA; identical to cDNA adenosine monophosphate binding protein 3 AMPBP3 (AMPBP3)GI:20799714 | | | | | | |
|  |  | RAFL05-07-K11 | At1g51140 / bHLH protein family | |  |  |  |  |  | | --- | --- | --- | --- | --- | |  |  |  |  |  | | At1g51140 ,RAFL05-07-K11  basic helix-loop-helix (bHLH) family protein contains Pfam profile: PF00010 helix-loop-helix DNA-binding domain | | | | | | |
|  |  | RAFL05-11-E04 | At3g51090 / expressed protein | |  |  |  |  |  | | --- | --- | --- | --- | --- | |  |  |  |  |  | | RAFL05-11-E04 ,At3g51090  expressed protein | | | | | | |
|  |  | RAFL05-05-J23 | At1g08570 / thioredoxin family | |  |  |  |  |  | | --- | --- | --- | --- | --- | |  |  |  |  |  | | At1g08570 ,RAFL05-05-J23  thioredoxin family protein contains Pfam profile: PF00085 Thioredoxin; similar to ESTs gb|T46281, gb|R83933, gb|N65879, emb|F14466, gb|N96726, gb|AA042340, and emb|Z18150 | | | | | | |
|  |  | RAFL05-09-N17 | At1g79270 / expressed protein | |  |  |  |  |  | | --- | --- | --- | --- | --- | |  |  |  |  |  | | RAFL05-09-N17 ,At1g79270  expressed protein contains Pfam profile PF04146: YT521-B-like family | | | | | | |
|  |  | RAFL08-12-C22 | At5g37300 / expressed protein | |  |  |  |  |  | | --- | --- | --- | --- | --- | |  |  |  |  |  | | RAFL08-12-C22 ,At5g37300  expressed protein | | | | | | |
|  |  | RAFL05-17-I08 | At2g43020 / amine oxidase family | |  |  |  |  |  | | --- | --- | --- | --- | --- | |  |  |  |  |  | | At2g43020 ,RAFL05-17-I08  amine oxidase family protein similar to polyamine oxidase SP:O64411 [Zea mays]; contains Pfam profile PF01593 amine oxidase, flavin-containing | | | | | | |
|  |  | RAFL08-15-F02 | At4g11570 / haloacid dehalogenase-like hydrolase family | |  |  |  |  |  | | --- | --- | --- | --- | --- | |  |  |  |  |  | | At4g11570 ,RAFL08-15-F02  haloacid dehalogenase-like hydrolase family protein similar to genetic modifier [Zea mays] GI:10444400; contains InterPro accession IPR005834: Haloacid dehalogenase-like hydrolase | | | | | | |
|  |  | RAFL09-11-B18 | At3g16990 / seed maturation protein -related | |  |  |  |  |  | | --- | --- | --- | --- | --- | |  |  |  |  |  | | At3g16990 ,RAFL09-11-B18  TENA/THI-4 family protein contains Pfam profile: PF03070 TENA/THI-4 family | | | | | | |
|  |  | RAFL09-06-F02 | At5g13760 / expressed protein | |  |  |  |  |  | | --- | --- | --- | --- | --- | |  |  |  |  |  | | RAFL09-06-F02 ,At5g13760  expressed protein similar to unknown protein (gb AAF63775.1) | | | | | | |
|  |  | RAFL08-08-G07 | At1g73920 / lipase family | |  |  |  |  |  | | --- | --- | --- | --- | --- | |  |  |  |  |  | | At1g73920 ,RAFL08-08-G07  lipase family protein similar to lipase GB:CAA74737 [SP|O46108] from [Drosophila melanogaster] | | | | | | |
|  |  | RAFL08-15-N01 | At4g38810 / calcium-binding EF-hand family protein | |  |  |  |  |  | | --- | --- | --- | --- | --- | |  |  |  |  |  | | RAFL08-15-N01 ,At4g38810  calcium-binding EF hand family protein contains INTERPRO:IPR002048 calcium-binding EF-hand domain | | | | | | |
|  | Cluster:6-2 | |  |  | 49 | 127 | 896 | 3591 | 0.012911535 | 0.42608064 | 33 |
|  |  | RAFL04-20-F09 | At4g19003 / expressed protein | |  |  |  |  |  | | --- | --- | --- | --- | --- | |  |  |  |  |  | | RAFL04-20-F09 ,At4g19003  expressed protein contains Pfam PF05871: Eukaryotic protein of unknown function (DUF852) | | | | | | |
|  |  | RAFL05-02-B05 | At4g24060 / Dof zinc finger protein | |  |  |  |  |  | | --- | --- | --- | --- | --- | |  |  |  |  |  | | At4g24060 ,RAFL05-02-B05  Dof-type zinc finger domain-containing protein Dof zinc finger protein - Oryza sativa,PID:d1042342 | | | | | | |
|  |  | RAFL04-17-F05 | At2g38730 / peptidyl-prolyl cis-trans isomerase -related | |  |  |  |  |  | | --- | --- | --- | --- | --- | |  |  |  |  |  | | At2g38730 ,RAFL04-17-F05  peptidyl-prolyl cis-trans isomerase, putative / cyclophilin, putative / rotamase, putative similar to cyclophilin [Homo sapiens] gi|3647230|gb|AAC60793 | | | | | | |
|  |  | RAFL04-17-N02 | At1g50370 / serine/threonine protein phosphatase | |  |  |  |  |  | | --- | --- | --- | --- | --- | |  |  |  |  |  | | RAFL04-17-N02 ,At1g50370  serine/threonine protein phosphatase, putative nearly identical to serine/threonine protein phosphatase [Arabidopsis thaliana] GI:14582206 | | | | | | |
|  |  | RAFL04-15-L12 | At5g48930 / anthranilate N-hydroxycinnamoyl/benzoyltransferase family | |  |  |  |  |  | | --- | --- | --- | --- | --- | |  |  |  |  |  | | RAFL04-15-L12 ,At5g48930  transferase family protein similar to anthranilate N-hydroxycinnamoyl/benzoyltransferase from Dianthus caryophyllus [GI:3288180, GI:2239091]; contains Pfam profile PF02458 transferase family | | | | | | |
|  |  | RAFL05-14-A14 | At1g48210 / serine/threonine protein kinase, putative | |  |  |  |  |  | | --- | --- | --- | --- | --- | |  |  |  |  |  | | RAFL05-14-A14 ,At1g48210  serine/threonine protein kinase, putative similar to Pto kinase interactor 1 [Lycopersicon esculentum] gi|3668069|gb|AAC61805; contains protein kinase domain, Pfam:PF00069 | | | | | | |
|  |  | RAFL09-17-B18 | At3g16270 / expressed protein | |  |  |  |  |  | | --- | --- | --- | --- | --- | |  |  |  |  |  | | RAFL09-17-B18 ,At3g16270  expressed protein gene model | | | | | | |
|  |  | RAFL05-09-G11 | At2g45260 / expressed protein | |  |  |  |  |  | | --- | --- | --- | --- | --- | |  |  |  |  |  | | RAFL05-09-G11 ,At2g45260  expressed protein contains Pfam profile PF04859: Plant protein of unknown function (DUF641 | | | | | | |
|  |  | RAFL05-07-G20 | At5g16080 / expressed protein | |  |  |  |  |  | | --- | --- | --- | --- | --- | |  |  |  |  |  | | RAFL05-07-G20 ,At5g16080  expressed protein similar to PrMC3 [Pinus radiata] GI:5487873 | | | | | | |
|  |  | RAFL05-16-P14 | At5g13880 / expressed protein | |  |  |  |  |  | | --- | --- | --- | --- | --- | |  |  |  |  |  | | At5g13880 ,RAFL05-16-P14  expressed protein | | | | | | |
|  |  | RAFL05-09-L07 | At1g70100 / expressed protein | |  |  |  |  |  | | --- | --- | --- | --- | --- | |  |  |  |  |  | | RAFL05-09-L07 ,At1g70100  expressed protein | | | | | | |
|  |  | RAFL05-18-B19 | At2g34770 / fatty acid hydroxylase (FAH1) | |  |  |  |  |  | | --- | --- | --- | --- | --- | |  |  |  |  |  | | RAFL05-18-B19 ,At2g34770  fatty acid hydroxylase (FAH1) identical to fatty acid hydroxylase Fah1p GB:AF021804 GI:2736147 from [Arabidopsis thaliana] | | | | | | |
|  |  | RAFL02-02-H09 | At5g53300 / ubiquitin-conjugating enzyme 10 (UBC10) | |  |  |  |  |  | | --- | --- | --- | --- | --- | |  |  |  |  |  | | At5g53300 ,RAFL02-02-H09  ubiquitin-conjugating enzyme 10 (UBC10) E2; identical to gi:297877, SP:P35133 | | | | | | |
|  |  | RAFL08-12-G20 | At5g47420 / expressed protein | |  |  |  |  |  | | --- | --- | --- | --- | --- | |  |  |  |  |  | | RAFL08-12-G20 ,At5g47420  expressed protein contains Pfam domain, PF01987: Protein of unknown function | | | | | | |
|  |  | RAFL04-09-C19 | At2g04520 / translation initiation factor eIF-1A -related | |  |  |  |  |  | | --- | --- | --- | --- | --- | |  |  |  |  |  | | At2g04520 ,RAFL04-09-C19  eukaryotic translation initiation factor 1A, putative / eIF-1A, putative / eIF-4C, putative strong similarity to translation initiation factor (eIF-1A) [Beta vulgaris] GI:17977975; contains Pfam profile PF01176: Eukaryotic initiation factor 1A | | | | | | |
|  |  | RAFL09-12-H09 | At5g04550 / expressed protein | |  |  |  |  |  | | --- | --- | --- | --- | --- | |  |  |  |  |  | | At5g04550 ,RAFL09-12-H09  expressed protein contains Pfam domain PF05003: protein of unknown function (DUF668) | | | | | | |
|  |  | RAFL05-11-N02 | At3g52560 / ubiquitin-conjugating enzyme family | |  |  |  |  |  | | --- | --- | --- | --- | --- | |  |  |  |  |  | | At3g52560 ,RAFL05-11-N02  ubiquitin-conjugating enzyme family protein similar to DNA-binding protein CROC-1B [Homo sapiens] GI:1066082; contains Pfam profile PF00179: Ubiquitin-conjugating enzyme | | | | | | |
|  |  | RAFL11-06-D23 | At1g65860 / flavin-containing monooxygenase (FMO) family | |  |  |  |  |  | | --- | --- | --- | --- | --- | |  |  |  |  |  | | At1g65860 ,RAFL11-06-D23  flavin-containing monooxygenase family protein / FMO family protein similar to flavin-containing monooxygenase FMO3 (dimethylaniline monoxygenase (N-oxide forming) 3) GI:349533 [SP|P32417] from Oryctolagus cuniculus, [SP|P97501] from Mus musculus; contains Pfam profile PF00743 Flavin-binding monooxygenase-like domain | | | | | | |
|  |  | RAFL09-06-F17 | At3g61580 / delta-8 sphingolipid desaturase (SLD1) | |  |  |  |  |  | | --- | --- | --- | --- | --- | |  |  |  |  |  | | RAFL09-06-F17 ,At3g61580  delta-8 sphingolipid desaturase (SLD1) identical to delta-8 sphingolipid desaturase GI:3819710 from [Arabidopsis thaliana]; contains Pfam profile PF00487: Fatty acid desaturase; contains Pfam profile PF00173: Heme/Steroid binding domain | | | | | | |
|  |  | RAFL05-15-H17 | At4g26100 / casein kinase, putative | |  |  |  |  |  | | --- | --- | --- | --- | --- | |  |  |  |  |  | | RAFL05-15-H17 ,At4g26100  casein kinase, putative similar to casein kinase I, delta isoform [Arabidopsis thaliana] SWISS-PROT:P42158; contains protein kinase domain, Pfam:PF00069 | | | | | | |
|  |  | RAFL05-07-O18 | At2g24360 / serine/threonine/tyrosine kinase, putative | |  |  |  |  |  | | --- | --- | --- | --- | --- | |  |  |  |  |  | | At2g24360 ,RAFL05-07-O18  serine/threonine/tyrosine kinase, putative similar to serine/threonine/tyrosine kinase [Arachis hypogaea] gi|13124865|gb|AAK11734 | | | | | | |
|  |  | RAFL08-17-B19 | At1g33390 / helicase domain-containing protein | |  |  |  |  |  | | --- | --- | --- | --- | --- | |  |  |  |  |  | | At1g33390 ,RAFL08-17-B19  helicase domain-containing protein similar to kurz protein [Drosophila melanogaster] GI:5869803; contains Pfam profiles PF04408: Helicase associated domain (HA2), PF00271: Helicase conserved C-terminal domain | | | | | | |
|  |  | RAFL08-10-N22 | At2g05940 / protein kinase, putative | |  |  |  |  |  | | --- | --- | --- | --- | --- | |  |  |  |  |  | | RAFL08-10-N22 ,At2g05940  protein kinase, putative similar to auxin-regulated dual specificity cytosolic kinase [Lycopersicon esculentum] gi|14484938|gb|AAK62821 | | | | | | |
|  |  | RAFL02-06-A10 | At2g18690 / expressed protein | |  |  |  |  |  | | --- | --- | --- | --- | --- | |  |  |  |  |  | | At2g18690 ,RAFL02-06-A10  expressed protein | | | | | | |
|  |  | RAFL04-20-M06 | At2g27260 / expressed protein | |  |  |  |  |  | | --- | --- | --- | --- | --- | |  |  |  |  |  | | At2g27260 ,RAFL04-20-M06  expressed protein | | | | | | |
|  |  | RAFL05-05-F06 | At3g45100 / n-acetylglucosaminyl-phosphatidylinositol biosynthetic protein, putative | |  |  |  |  |  | | --- | --- | --- | --- | --- | |  |  |  |  |  | | At3g45100 ,RAFL05-05-F06  N-acetylglucosaminyl-phosphatidylinositol biosynthetic protein, putative similar to PIG-A from Mus musculus [gi:577723[, Homo sapiens [SP|P37287]; contains Pfam glycosyl transferase, group 1 family protein domain PF00534 | | | | | | |
|  |  | RAFL05-09-N21 | At5g10540 / oligopeptidase A - like protein | |  |  |  |  |  | | --- | --- | --- | --- | --- | |  |  |  |  |  | | At5g10540 ,RAFL05-09-N21  peptidase M3 family protein / thimet oligopeptidase family protein similar to SP|P27237 Oligopeptidase A (EC 3.4.24.70) {Salmonella typhimurium}; contains Pfam profile PF01432: Peptidase family M3 | | | | | | |
|  |  | RAFL04-15-J07 | At2g29970 / expressed protein | |  |  |  |  |  | | --- | --- | --- | --- | --- | |  |  |  |  |  | | RAFL04-15-J07 ,At2g29970  heat shock protein-related contains similarity to 101 kDa heat shock protein; HSP101 [Triticum aestivum] gi|11561808|gb|AAC83689 | | | | | | |
|  |  | RAFL05-07-N24 | At3g61260 / DNA-binding protein -related | |  |  |  |  |  | | --- | --- | --- | --- | --- | |  |  |  |  |  | | At3g61260 ,RAFL05-07-N24  DNA-binding family protein / remorin family protein similar to DNA-binding protein gi|601843 [Arabidopsis thaliana], remorin [Solanum tuberosum] GI:1881585; contains Pfam profiles PF03763: Remorin C-terminal region, PF03766: Remorin N-terminal region | | | | | | |
|  |  | RAFL05-07-I02 | At5g51940 / DNA-directed RNA polymerase II subunit-related protein | |  |  |  |  |  | | --- | --- | --- | --- | --- | |  |  |  |  |  | | RAFL05-07-I02 ,At5g51940  DNA-directed RNA polymerase II, putative similar to SP|O88828 DNA-directed RNA polymerase II 14.4 kDa polypeptide (EC 2.7.7.6) (RPB6) (RPB14.4) {Rattus norvegicus}; contains Pfam profile PF01192: RNA polymerases K / 14 to 18 kDa subunit | | | | | | |
|  |  | RAFL05-07-O04 | At1g07250 / glycosyltransferase family | |  |  |  |  |  | | --- | --- | --- | --- | --- | |  |  |  |  |  | | At1g07250 ,RAFL05-07-O04  UDP-glucoronosyl/UDP-glucosyl transferase family protein similar to UDP-glucose glucosyltransferase GI:453245 from [Manihot esculenta] | | | | | | |
|  |  | RAFL08-17-D06 | At3g51850 / calcium-dependent protein kinase, putative (CDPK) | |  |  |  |  |  | | --- | --- | --- | --- | --- | |  |  |  |  |  | | At3g51850 ,RAFL08-17-D06  calcium-dependent protein kinase, putative / CDPK, putative similar to calcium-dependent protein kinase [Arabidopsis thaliana] gi|836942|gb|AAA67655; contains protein kinase domain, Pfam:PF00069; contains EF hand domain (calcium-binding EF-hand), Pfam:PF00036, INTERPRO:IPR002048 | | | | | | |
|  |  | RAFL05-07-J03 | At4g40050 / expressed protein | |  |  |  |  |  | | --- | --- | --- | --- | --- | |  |  |  |  |  | | At4g40050 ,RAFL05-07-J03  expressed protein | | | | | | |
|  |  | RAFL02-07-M07 | At1g08980 / amidase | |  |  |  |  |  | | --- | --- | --- | --- | --- | |  |  |  |  |  | | RAFL02-07-M07 ,At1g08980  amidase family protein similar to component of chloroplast outer membrane translocon Toc64 [Pisum sativum] GI:7453538; contains Pfam profile PF01425: Amidase; supporting cDNA gi|11493701|gb|AF202077.1|AF202077 | | | | | | |
|  |  | RAFL04-15-F14 | At5g26751 / shaggy-related protein kinase alpha | |  |  |  |  |  | | --- | --- | --- | --- | --- | |  |  |  |  |  | | At5g26751 ,RAFL04-15-F14  shaggy-related protein kinase alpha / ASK-alpha (ASK1) identical to shaggy-related protein kinase alpha SP:P43288 GI:460832 from [Arabidopsis thaliana] | | | | | | |
|  |  | RAFL02-08-J13 | At5g37830 / 5-oxoprolinase -related protein | |  |  |  |  |  | | --- | --- | --- | --- | --- | |  |  |  |  |  | | RAFL02-08-J13 ,At5g37830  hydantoinase/oxoprolinase family protein contains Pfam profiles: PF02538 hydantoinase B/oxoprolinase, PF01968 hydantoinase/oxoprolinase, PF05378 hydantoinase/oxoprolinase N-terminal region | | | | | | |
|  |  | RAFL08-18-J08 | At3g05840 / shaggy-related protein kinase gamma | |  |  |  |  |  | | --- | --- | --- | --- | --- | |  |  |  |  |  | | At3g05840 ,RAFL08-18-J08  shaggy-related protein kinase gamma / ASK-gamma (ASK3) identical to shaggy-related protein kinase gamma SP:P43289 GI:456509 from [Arabidopsis thaliana] | | | | | | |
|  |  | RAFL05-13-K22 | At4g14300 / heterogeneous nuclear ribonucleoprotein (hnRNP), putative | |  |  |  |  |  | | --- | --- | --- | --- | --- | |  |  |  |  |  | | At4g14300 ,RAFL05-13-K22  heterogeneous nuclear ribonucleoprotein, putative / hnRNP, putative | | | | | | |
|  |  | RAFL04-13-G15 | At1g16170 / expressed protein | |  |  |  |  |  | | --- | --- | --- | --- | --- | |  |  |  |  |  | | At1g16170 ,RAFL04-13-G15  expressed protein | | | | | | |
|  |  | RAFL05-09-N19 | At2g25850 / poly(A) polymerase -related | |  |  |  |  |  | | --- | --- | --- | --- | --- | |  |  |  |  |  | | At2g25850 ,RAFL05-09-N19  nucleotidyltransferase family protein contains Pfam profiles: PF01909 nucleotidyltransferase domain, PF04926 poly(A) polymerase predicted RNA binding domain; identical to cDNA GI:31747890 | | | | | | |
|  |  | RAFL06-15-J06 | At5g64130 / expressed protein | |  |  |  |  |  | | --- | --- | --- | --- | --- | |  |  |  |  |  | | RAFL06-15-J06 ,At5g64130  expressed protein | | | | | | |
|  |  | RAFL05-08-O16 | At2g17870 / glycine-rich, zinc-finger DNA-binding protein -related | |  |  |  |  |  | | --- | --- | --- | --- | --- | |  |  |  |  |  | | RAFL05-08-O16 ,At2g17870  cold-shock DNA-binding family protein contains Pfam domains, PF00313: 'Cold-shock' DNA-binding domain and PF00098: Zinc knuckle | | | | | | |
|  |  | RAFL05-19-K18 | At5g19180 / RUB-activating enzyme ECR1 | |  |  |  |  |  | | --- | --- | --- | --- | --- | |  |  |  |  |  | | RAFL05-19-K18 ,At5g19180  ubiquitin activating enzyme, putative (ECR1) identical to putative ubiquitin activating enzyme E1 [Arabidopsis thaliana] GI:2952433; similar to NEDD8 activating enzyme [Mus musculus] GI:17061821 | | | | | | |
|  |  | RAFL04-17-J19 | At1g07110 / fructose-2,6-bisphosphatase -related | |  |  |  |  |  | | --- | --- | --- | --- | --- | |  |  |  |  |  | | RAFL04-17-J19 ,At1g07110  fructose-6-phosphate 2-kinase / fructose-2,6-bisphosphatase (F2KP) identical to fructose-6-phosphate 2-kinase/fructose-2,6-bisphosphatase (F2KP) [Arabidopsis thaliana] GI:13096098 | | | | | | |
|  |  | RAFL05-01-F03 | At5g38590 / F-box protein family | |  |  |  |  |  | | --- | --- | --- | --- | --- | |  |  |  |  |  | | At5g38590 ,RAFL05-01-F03  F-box family protein contains F-box domain Pfam:PF00646 | | | | | | |
|  |  | RAFL05-17-H03 | At2g25430 / expressed protein | |  |  |  |  |  | | --- | --- | --- | --- | --- | |  |  |  |  |  | | RAFL05-17-H03 ,At2g25430  epsin N-terminal homology (ENTH) domain-containing protein contains Pfam PF01417: ENTH domain. ENTH (Epsin N-terminal homology) domain; | | | | | | |
|  |  | RAFL06-16-O17 | At5g41810 / expressed protein | |  |  |  |  |  | | --- | --- | --- | --- | --- | |  |  |  |  |  | | At5g41810 ,RAFL06-16-O17  expressed protein | | | | | | |
|  |  | RAFL05-19-E13 | At1g61730 / expressed protein | |  |  |  |  |  | | --- | --- | --- | --- | --- | |  |  |  |  |  | | At1g61730 ,RAFL05-19-E13  DNA-binding storekeeper protein-related contains Pfam profile: PF04504 protein of unknown function, DUF573; similar to storekeeper protein GI:14268476 [Solanum tuberosum] | | | | | | |
|  |  | RAFL05-07-M03 | At1g01800 / short-chain dehydrogenase/reductase family protein | |  |  |  |  |  | | --- | --- | --- | --- | --- | |  |  |  |  |  | | At1g01800 ,RAFL05-07-M03  short-chain dehydrogenase/reductase (SDR) family protein similar to carbonyl reductase GI:1049108 from [Mus musculus] | | | | | | |
|  | Cluster:9-2 | |  |  | 22 | 45 | 923 | 3673 | 0.013835912 | 0.45658508 | 33 |
|  |  | RAFL05-18-H12 | At1g80840 / WRKY family transcription factor | |  |  |  |  |  | | --- | --- | --- | --- | --- | |  |  |  |  |  | | RAFL05-18-H12 ,At1g80840  WRKY family transcription factor similar to WRKY transcription factor GB:BAA87058 GI:6472585 from [Nicotiana tabacum] | | | | | | |
|  |  | RAFL05-19-E19 | At2g30250 / WRKY family transcription factor | |  |  |  |  |  | | --- | --- | --- | --- | --- | |  |  |  |  |  | | RAFL05-19-E19 ,At2g30250  WRKY family transcription factor | | | | | | |
|  |  | RAFL08-11-N01 | At4g34710 / arginine decarboxylase SPE2 | |  |  |  |  |  | | --- | --- | --- | --- | --- | |  |  |  |  |  | | At4g34710 ,RAFL08-11-N01  arginine decarboxylase 2 (SPE2) identical to SP|O23141 Arginine decarboxylase 2 (EC 4.1.1.19) (ARGDC 2) (ADC 2) (ADC-N) {Arabidopsis thaliana} | | | | | | |
|  |  | RAFL08-08-L20 | At1g56600 / galactinol synthase, putative | |  |  |  |  |  | | --- | --- | --- | --- | --- | |  |  |  |  |  | | At1g56600 ,RAFL08-08-L20  galactinol synthase, putative similar to galactinol synthase, isoform GolS-1 GI:5608497 from [Ajuga reptans] | | | | | | |
|  |  | RAFL05-19-I05 | At1g01720 / No apical meristem (NAM) protein family | |  |  |  |  |  | | --- | --- | --- | --- | --- | |  |  |  |  |  | | RAFL05-19-I05 ,At1g01720  no apical meristem (NAM) family protein contains Pfam PF02365: No apical meristem (NAM) domain; similar to NAC domain protein NAM GB:AAD17313 GI:4325282 from [Arabidopsis thaliana] | | | | | | |
|  |  | RAFL04-10-D13 | At2g23120 / expressed protein | |  |  |  |  |  | | --- | --- | --- | --- | --- | |  |  |  |  |  | | RAFL04-10-D13 ,At2g23120  expressed protein | | | | | | |
|  |  | RAFL09-13-D07 | At4g34710 / arginine decarboxylase SPE2 | |  |  |  |  |  | | --- | --- | --- | --- | --- | |  |  |  |  |  | | RAFL09-13-D07 ,At4g34710  arginine decarboxylase 2 (SPE2) identical to SP|O23141 Arginine decarboxylase 2 (EC 4.1.1.19) (ARGDC 2) (ADC 2) (ADC-N) {Arabidopsis thaliana} | | | | | | |
|  |  | RAFL09-11-P17 | At4g25690 / expressed protein | |  |  |  |  |  | | --- | --- | --- | --- | --- | |  |  |  |  |  | | At4g25690 ,RAFL09-11-P17  expressed protein | | | | | | |
|  |  | RAFL02-07-L08 | At5g61160 / transferase family | |  |  |  |  |  | | --- | --- | --- | --- | --- | |  |  |  |  |  | | At5g61160 ,RAFL02-07-L08  transferase family protein similar to anthocyanin 5-aromatic acyltransferase from Gentiana triflora GI:4185599, malonyl CoA:anthocyanin 5-O-glucoside-6'''-O-malonyltransferase from Perilla frutescens GI:17980232, Salvia splendens GI:17980234; contains Pfam profile PF02458 transferase family | | | | | | |
|  |  | RAFL05-09-I02 | At5g23340 / expressed protein | |  |  |  |  |  | | --- | --- | --- | --- | --- | |  |  |  |  |  | | At5g23340 ,RAFL05-09-I02  expressed protein | | | | | | |
|  |  | RAFL05-13-D04 | At2g37970 / expressed protein | |  |  |  |  |  | | --- | --- | --- | --- | --- | |  |  |  |  |  | | At2g37970 ,RAFL05-13-D04  SOUL heme-binding family protein weak similarity to SOUL protein [Mus musculus] GI:4886906; contains Pfam profile PF04832: SOUL heme-binding protein | | | | | | |
|  |  | RAFL11-06-L09 | At2g40000 / nematode-resistance protein -related | |  |  |  |  |  | | --- | --- | --- | --- | --- | |  |  |  |  |  | | At2g40000 ,RAFL11-06-L09  expressed protein | | | | | | |
|  |  | RAFL05-14-A21 | At3g59350 / serine/threonine protein kinase, putative | |  |  |  |  |  | | --- | --- | --- | --- | --- | |  |  |  |  |  | | RAFL05-14-A21 ,At3g59350  serine/threonine protein kinase, putative similar to Pto kinase interactor 1 (Pti1)[Lycopersicon esculentum] gi|3668069|gb|AAC61805 | | | | | | |
|  |  | RAFL07-07-G15 | At1g01720 / No apical meristem (NAM) protein family | |  |  |  |  |  | | --- | --- | --- | --- | --- | |  |  |  |  |  | | RAFL07-07-G15 ,At1g01720  no apical meristem (NAM) family protein contains Pfam PF02365: No apical meristem (NAM) domain; similar to NAC domain protein NAM GB:AAD17313 GI:4325282 from [Arabidopsis thaliana] | | | | | | |
|  |  | RAFL07-13-C20 | At3g10300 / calcium-binding EF-hand family protein | |  |  |  |  |  | | --- | --- | --- | --- | --- | |  |  |  |  |  | | At3g10300 ,RAFL07-13-C20  calcium-binding EF hand family protein low similarity to SP|P12815 Programmed cell death protein 6 (Probable calcium-binding protein ALG-2) {Mus musculus}; contains INTERPRO:IPR002048 calcium-binding EF-hand domain | | | | | | |
|  |  | RAFL03-05-I09 | At1g76180 / dehydrin -related | |  |  |  |  |  | | --- | --- | --- | --- | --- | |  |  |  |  |  | | RAFL03-05-I09 ,At1g76180  dehydrin (ERD14) identical to SP|P42763 Dehydrin ERD14 {Arabidopsis thaliana} | | | | | | |
|  |  | RAFL05-09-M02 | At5g06320 / harpin-induced protein 1 family (NDR1/HIN1-like protein 3) | |  |  |  |  |  | | --- | --- | --- | --- | --- | |  |  |  |  |  | | RAFL05-09-M02 ,At5g06320  harpin-induced family protein / HIN1 family protein / harpin-responsive family protein / NDR1/HIN1-like protein 3 similar to harpin-induced protein hin1 (GI:1619321)[Nicotiana tabacum] | | | | | | |
|  |  | RAFL05-01-H22 | At4g18280 / glycine-rich cell wall protein-related | |  |  |  |  |  | | --- | --- | --- | --- | --- | |  |  |  |  |  | | At4g18280 ,RAFL05-01-H22  glycine-rich cell wall protein-related glycine-rich protein 1.0 precursor, Phaseolus vulgaris, PIR1:S01821 | | | | | | |
|  |  | RAFL04-17-M22 | At1g73390 / expressed protein | |  |  |  |  |  | | --- | --- | --- | --- | --- | |  |  |  |  |  | | At1g73390 ,RAFL04-17-M22  expressed protein | | | | | | |
|  |  | RAFL05-03-K03 | At4g25670 / expressed protein | |  |  |  |  |  | | --- | --- | --- | --- | --- | |  |  |  |  |  | | RAFL05-03-K03 ,At4g25670  expressed protein | | | | | | |
|  |  | RAFL05-04-E02 | At5g54940 / translation initiation factor-related protein | |  |  |  |  |  | | --- | --- | --- | --- | --- | |  |  |  |  |  | | At5g54940 ,RAFL05-04-E02  eukaryotic translation initiation factor SUI1, putative similar to SP|P32911 Protein translation factor SUI1 {Saccharomyces cerevisiae}; contains Pfam profile PF01253: Translation initiation factor SUI1 | | | | | | |
|  |  | RAFL04-12-F24 | At1g51090 / heavy-metal-associated domain-containing protein | |  |  |  |  |  | | --- | --- | --- | --- | --- | |  |  |  |  |  | | At1g51090 ,RAFL04-12-F24  heavy-metal-associated domain-containing protein contains Pfam profile PF00403: Heavy-metal-associated domain | | | | | | |
|  | Cluster:7-0 | |  |  | 63 | 184 | 882 | 3534 | 0.041663494 | 1.3748953 | 33 |
|  |  | RAFL08-13-K20 | At4g26000 / KH domain protein | |  |  |  |  |  | | --- | --- | --- | --- | --- | |  |  |  |  |  | | RAFL08-13-K20 ,At4g26000  KH domain-containing protein single-stranded nucleic acid-binding protein CBP - mouse, PIR2:S78515 | | | | | | |
|  |  | RAFL05-16-L16 | At3g10770 / expressed protein | |  |  |  |  |  | | --- | --- | --- | --- | --- | |  |  |  |  |  | | At3g10770 ,RAFL05-16-L16  expressed protein | | | | | | |
|  |  | RAFL05-03-M05 | At5g22120 / expressed protein | |  |  |  |  |  | | --- | --- | --- | --- | --- | |  |  |  |  |  | | RAFL05-03-M05 ,At5g22120  expressed protein | | | | | | |
|  |  | RAFL05-04-C19 | At3g50910 / expressed protein | |  |  |  |  |  | | --- | --- | --- | --- | --- | |  |  |  |  |  | | RAFL05-04-C19 ,At3g50910  expressed protein | | | | | | |
|  |  | RAFL06-16-H21 | At1g15340 / expressed protein | |  |  |  |  |  | | --- | --- | --- | --- | --- | |  |  |  |  |  | | RAFL06-16-H21 ,At1g15340  methyl-CpG-binding domain-containing protein contains Pfam profile PF01429: Methyl-CpG binding domain | | | | | | |
|  |  | RAFL05-21-P18 | At1g36980 / expressed protein | |  |  |  |  |  | | --- | --- | --- | --- | --- | |  |  |  |  |  | | At1g36980 ,RAFL05-21-P18  expressed protein | | | | | | |
|  |  | RAFL04-15-B18 | At2g46900 / expressed protein | |  |  |  |  |  | | --- | --- | --- | --- | --- | |  |  |  |  |  | | RAFL04-15-B18 ,At2g46900  expressed protein contains Pfam profile PF04910: Protein of unknown function, DUF654 | | | | | | |
|  |  | RAFL04-12-P17 | At4g05530 / short-chain dehydrogenase/reductase family protein | |  |  |  |  |  | | --- | --- | --- | --- | --- | |  |  |  |  |  | | At4g05530 ,RAFL04-12-P17  short-chain dehydrogenase/reductase (SDR) family protein similar to peroxisomal short-chain alcohol dehydrogenase GI:4105190 from [Homo sapiens] | | | | | | |
|  |  | RAFL03-08-N24 | At4g14430 / enoyl-CoA hydratase/isomerase family | |  |  |  |  |  | | --- | --- | --- | --- | --- | |  |  |  |  |  | | RAFL03-08-N24 ,At4g14430  enoyl-CoA hydratase/isomerase family protein low siimilarity to 2-cyclohexenylcarbonyl CoA isomerase [Streptomyces collinus] GI:8133118, enoyl-CoA isomerase [Escherichia coli] GI:2764829; contains Pfam profile PF00378 enoyl-CoA hydratase/isomerase family protein | | | | | | |
|  |  | RAFL05-07-C05 | At5g38650 / expressed protein | |  |  |  |  |  | | --- | --- | --- | --- | --- | |  |  |  |  |  | | RAFL05-07-C05 ,At5g38650  proteasome maturation factor UMP1 family protein contains Pfam profile PF05348: Proteasome maturation factor UMP1 | | | | | | |
|  |  | RAFL07-13-O20 | At1g72710 / casein kinase, putative | |  |  |  |  |  | | --- | --- | --- | --- | --- | |  |  |  |  |  | | RAFL07-13-O20 ,At1g72710  casein kinase, putative similar to casein kinase I, delta isoform [Arabidopsis thaliana] SWISS-PROT:P42158 | | | | | | |
|  |  | RAFL09-07-D20 | At5g05100 / expressed protein | |  |  |  |  |  | | --- | --- | --- | --- | --- | |  |  |  |  |  | | RAFL09-07-D20 ,At5g05100  expressed protein | | | | | | |
|  |  | RAFL05-02-G23 | At5g62540 / ubiquitin-conjugating enzyme 3 (UBC3) | |  |  |  |  |  | | --- | --- | --- | --- | --- | |  |  |  |  |  | | RAFL05-02-G23 ,At5g62540  ubiquitin-conjugating enzyme 3 (UBC3) E2; identical to gi:431261, SP:P42746 | | | | | | |
|  |  | RAFL05-12-O20 | At5g24450 / expressed protein | |  |  |  |  |  | | --- | --- | --- | --- | --- | |  |  |  |  |  | | RAFL05-12-O20 ,At5g24450  transcription factor-related low similarity to transcription factor IIIC63 [Homo sapiens] GI:5281316 | | | | | | |
|  |  | RAFL04-16-K10 | At3g09085 / expressed protein | |  |  |  |  |  | | --- | --- | --- | --- | --- | |  |  |  |  |  | | RAFL04-16-K10 ,At3g09085  expressed protein | | | | | | |
|  |  | RAFL03-08-G12 | At5g53330 / proline-rich cell wall protein-related | |  |  |  |  |  | | --- | --- | --- | --- | --- | |  |  |  |  |  | | RAFL03-08-G12 ,At5g53330  expressed protein | | | | | | |
|  |  | RAFL05-17-D02 | At2g43210 / expressed protein | |  |  |  |  |  | | --- | --- | --- | --- | --- | |  |  |  |  |  | | At2g43210 ,RAFL05-17-D02  UBX domain-containing protein contains Pfam profile PF00789: UBX domain | | | | | | |
|  |  | RAFL06-09-E01 | At3g12490 / cysteine proteinase inhibitor -related | |  |  |  |  |  | | --- | --- | --- | --- | --- | |  |  |  |  |  | | RAFL06-09-E01 ,At3g12490  cysteine protease inhibitor, putative / cystatin, putative similar to PRLI-interacting factor M [Arabidopsis thaliana] GI:11139270, cysteine proteinase inhibitor [Brassica rapa] GI:762785; contains Pfam profile PF00031: Cystatin domain | | | | | | |
|  |  | RAFL07-11-H01 | At5g66930 / expressed protein | |  |  |  |  |  | | --- | --- | --- | --- | --- | |  |  |  |  |  | | RAFL07-11-H01 ,At5g66930  expressed protein similar to unknown protein (pir||T38383) | | | | | | |
|  |  | RAFL09-18-M24 | At2g35680 / expressed protein | |  |  |  |  |  | | --- | --- | --- | --- | --- | |  |  |  |  |  | | At2g35680 ,RAFL09-18-M24  dual specificity protein phosphatase family protein contains Pfam profile: PF00782 dual specificity phosphatase, catalytic domain | | | | | | |
|  |  | RAFL11-03-E16 | At4g23870 / expressed protein | |  |  |  |  |  | | --- | --- | --- | --- | --- | |  |  |  |  |  | | RAFL11-03-E16 ,At4g23870  expressed protein predicted proteins, Arabidopsis thaliana | | | | | | |
|  |  | RAFL08-19-K02 | At5g42810 / expressed protein | |  |  |  |  |  | | --- | --- | --- | --- | --- | |  |  |  |  |  | | At5g42810 ,RAFL08-19-K02  expressed protein similar to unknown protein (pir||T26506) | | | | | | |
|  |  | RAFL05-21-F11 | At5g35320 / expressed protein | |  |  |  |  |  | | --- | --- | --- | --- | --- | |  |  |  |  |  | | RAFL05-21-F11 ,At5g35320  expressed protein | | | | | | |
|  |  | RAFL04-14-I08 | At5g65910 / expressed protein | |  |  |  |  |  | | --- | --- | --- | --- | --- | |  |  |  |  |  | | At5g65910 ,RAFL04-14-I08  BSD domain-containing protein contains Pfam profile PF03909: BSD domain | | | | | | |
|  |  | RAFL09-12-K24 | At1g27150 / expressed protein | |  |  |  |  |  | | --- | --- | --- | --- | --- | |  |  |  |  |  | | At1g27150 ,RAFL09-12-K24  expressed protein | | | | | | |
|  |  | RAFL03-05-E08 | At3g53990 / expressed protein | |  |  |  |  |  | | --- | --- | --- | --- | --- | |  |  |  |  |  | | At3g53990 ,RAFL03-05-E08  universal stress protein (USP) family protein contains Pfam PF00582: universal stress protein family | | | | | | |
|  |  | RAFL05-02-F07 | At5g58350 / protein kinase family | |  |  |  |  |  | | --- | --- | --- | --- | --- | |  |  |  |  |  | | RAFL05-02-F07 ,At5g58350  protein kinase family protein contains protein kinase domain, Pfam:PF00069 | | | | | | |
|  |  | RAFL04-14-D12 | At5g03230 / expressed protein | |  |  |  |  |  | | --- | --- | --- | --- | --- | |  |  |  |  |  | | At5g03230 ,RAFL04-14-D12  expressed protein contains Pfam profile PF04520: Protein of unknown function, DUF584 | | | | | | |
|  |  | RAFL08-11-J20 | At1g01780 / LIM domain protein -related | |  |  |  |  |  | | --- | --- | --- | --- | --- | |  |  |  |  |  | | At1g01780 ,RAFL08-11-J20  LIM domain-containing protein similar to PGPS/D1 [Petunia x hybrida] GI:4105772, LIM domain protein PLIM1 [Nicotiana tabacum] GI:5932418; contains Pfam profile PF00412: LIM domain | | | | | | |
|  |  | RAFL11-06-B10 | At1g54270 / eukaryotic translation initiation factor 4A-2 (eIF4A-2) | |  |  |  |  |  | | --- | --- | --- | --- | --- | |  |  |  |  |  | | RAFL11-06-B10 ,At1g54270  eukaryotic translation initiation factor 4A-2 / eIF-4A-2 similar to eukaryotic translation initiation factor 4A GI:19696 from [Nicotiana plumbaginifolia] | | | | | | |
|  |  | RAFL09-16-I09 | At5g20280 / sucrose-phosphate synthase, putative | |  |  |  |  |  | | --- | --- | --- | --- | --- | |  |  |  |  |  | | At5g20280 ,RAFL09-16-I09  sucrose-phosphate synthase, putative similar to sucrose-phosphate synthase (EC 2.4.1.14) isoform 1 - Citrus unshiu, EMBL:AB005023 | | | | | | |
|  |  | RAFL05-14-A08 | At5g63030 / glutaredoxin, putative | |  |  |  |  |  | | --- | --- | --- | --- | --- | |  |  |  |  |  | | At5g63030 ,RAFL05-14-A08  glutaredoxin, putative similar to glutaredoxin [Ricinus communis] gi|1732424|emb|CAA89699 | | | | | | |
|  |  | RAFL02-10-G21 | At3g53990 / expressed protein | |  |  |  |  |  | | --- | --- | --- | --- | --- | |  |  |  |  |  | | At3g53990 ,RAFL02-10-G21  universal stress protein (USP) family protein contains Pfam PF00582: universal stress protein family | | | | | | |
|  |  | RAFL03-01-H02 | At1g35190 / oxidoreductase, 2OG-Fe(II) oxygenase family | |  |  |  |  |  | | --- | --- | --- | --- | --- | |  |  |  |  |  | | RAFL03-01-H02 ,At1g35190  oxidoreductase, 2OG-Fe(II) oxygenase family protein low similarity to hyoscyamine 6-dioxygenase hydroxylase from Hyoscyamus niger [GB:P24397][SP|P24397], Atropa belladona [gi:4996123]; contains Pfam domain PF03171, 2OG-Fe(II) oxygenase superfamily | | | | | | |
|  |  | RAFL09-07-L11 | At5g41990 / protein kinase family | |  |  |  |  |  | | --- | --- | --- | --- | --- | |  |  |  |  |  | | RAFL09-07-L11 ,At5g41990  protein kinase family protein contains protein kinase domain, Pfam:PF00069 | | | | | | |
|  |  | RAFL07-14-P03 | At5g59420 / oxysterol-binding protein - like | |  |  |  |  |  | | --- | --- | --- | --- | --- | |  |  |  |  |  | | At5g59420 ,RAFL07-14-P03  oxysterol-binding family protein similar to SP|P16258 Oxysterol-binding protein 1 {Oryctolagus cuniculus}; contains Pfam profile PF01237: Oxysterol-binding protein | | | | | | |
|  |  | RAFL06-09-D08 | At3g59940 / Kelch repeat containing F-box protein family | |  |  |  |  |  | | --- | --- | --- | --- | --- | |  |  |  |  |  | | RAFL06-09-D08 ,At3g59940  kelch repeat-containing F-box family protein contains Pfam profiles PF01344: Kelch motif, PF00646: F-box domain | | | | | | |
|  |  | RAFL11-03-C04 | At4g19860 / hypothetical protein | |  |  |  |  |  | | --- | --- | --- | --- | --- | |  |  |  |  |  | | At4g19860 ,RAFL11-03-C04  lecithin:cholesterol acyltransferase family protein / LACT family protein similar to lysosomal phospholipase A2 [Mus musculus] GI:18699602; contains Pfam profile PF02450: Lecithin:cholesterol acyltransferase (phosphatidylcholine-sterol acyltransferase) | | | | | | |
|  |  | RAFL05-07-A07 | At2g36950 / heavy-metal-associated domain-containing protein | |  |  |  |  |  | | --- | --- | --- | --- | --- | |  |  |  |  |  | | At2g36950 ,RAFL05-07-A07  heavy-metal-associated domain-containing protein nearly identical to farnesylated protein ATFP2 [GI:4097545] Pfam profile PF00403: Heavy-metal-associated domain | | | | | | |
|  |  | RAFL05-09-J02 | At5g66052 / expressed protein | |  |  |  |  |  | | --- | --- | --- | --- | --- | |  |  |  |  |  | | At5g66052 ,RAFL05-09-J02  expressed protein | | | | | | |
|  |  | RAFL05-14-I02 | At3g10020 / expressed protein | |  |  |  |  |  | | --- | --- | --- | --- | --- | |  |  |  |  |  | | RAFL05-14-I02 ,At3g10020  expressed protein | | | | | | |
|  |  | RAFL04-20-P03 | At5g58380 / CBL-interacting protein kinase 10 | |  |  |  |  |  | | --- | --- | --- | --- | --- | |  |  |  |  |  | | At5g58380 ,RAFL04-20-P03  CBL-interacting protein kinase 10 (CIPK10) identical to CBL-interacting protein kinase 10 [Arabidopsis thaliana] gi|13249119|gb|AAK16685; contains Pfam profiles PF00069: Protein kinase domain and PF03822: NAF domain; identical to cDNA CBL-interacting protein kinase 10 (CIPK10) GI:13249118 | | | | | | |
|  |  | RAFL05-21-P21 | At4g29330 / expressed protein | |  |  |  |  |  | | --- | --- | --- | --- | --- | |  |  |  |  |  | | At4g29330 ,RAFL05-21-P21  Der1-like family protein / degradation in the ER-like family protein contains Pfam profile: PF04511 Der1-like family | | | | | | |
|  |  | RAFL07-13-L20 | At1g67580 / protein kinase family | |  |  |  |  |  | | --- | --- | --- | --- | --- | |  |  |  |  |  | | RAFL07-13-L20 ,At1g67580  protein kinase family protein contains protein kinase domain, Pfam:PF00069 | | | | | | |
|  |  | RAFL05-16-K02 | At5g64170 / expressed protein | |  |  |  |  |  | | --- | --- | --- | --- | --- | |  |  |  |  |  | | At5g64170 ,RAFL05-16-K02  dentin sialophosphoprotein-related contains weak similarity to Swiss-Prot:Q9NZW4 dentin sialophosphoprotein precursor [Homo sapiens] | | | | | | |
|  |  | RAFL06-12-N20 | At3g16190 / isochorismatase hydrolase family | |  |  |  |  |  | | --- | --- | --- | --- | --- | |  |  |  |  |  | | RAFL06-12-N20 ,At3g16190  isochorismatase hydrolase family protein low similarity to SP|P32400 N-carbamoylsarcosine amidase (EC 3.5.1.59) (N-carbamoylsarcosine amidohydrolase) {Arthrobacter sp}; contains Pfam profile PF00857: isochorismatase family protein | | | | | | |
|  |  | RAFL05-11-B19 | At1g07530 / scarecrow-like transcription factor 14 (SCL14) | |  |  |  |  |  | | --- | --- | --- | --- | --- | |  |  |  |  |  | | At1g07530 ,RAFL05-11-B19  scarecrow-like transcription factor 14 (SCL14) identical to GB:AAD24412 from [Arabidopsis thaliana] (Plant J. 18 (1), 111-119 (1999)) | | | | | | |
|  |  | RAFL06-11-N07 | At4g24690 / ubiquitin-associated (UBA)/PB1 domain-containing protein | |  |  |  |  |  | | --- | --- | --- | --- | --- | |  |  |  |  |  | | RAFL06-11-N07 ,At4g24690  ubiquitin-associated (UBA)/TS-N domain-containing protein / octicosapeptide/Phox/Bemp1 (PB1) domain-containing protein contains Pfam profiles PF00627: Ubiquitin-associated (UBA)/TS-N domain, PF00569: Zinc finger ZZ type domain, PF00564: PB1 domain | | | | | | |
|  |  | RAFL09-14-B02 | At2g22240 / myo-inositol 1-phosphate synthase -related | |  |  |  |  |  | | --- | --- | --- | --- | --- | |  |  |  |  |  | | At2g22240 ,RAFL09-14-B02  inositol-3-phosphate synthase isozyme 2 / myo-inositol-1-phosphate synthase 2 / MI-1-P synthase 2 / IPS 2 identical to SP|Q38862 Myo-inositol-1-phosphate synthase isozyme 2 (EC 5.5.1.4) (MI-1-P synthase 2) (IPS 2) {Arabidopsis thaliana} | | | | | | |
|  |  | RAFL04-19-H05 | At1g78790 / expressed protein | |  |  |  |  |  | | --- | --- | --- | --- | --- | |  |  |  |  |  | | At1g78790 ,RAFL04-19-H05  expressed protein | | | | | | |
|  |  | RAFL08-14-C01 | At5g45410 / expressed protein | |  |  |  |  |  | | --- | --- | --- | --- | --- | |  |  |  |  |  | | RAFL08-14-C01 ,At5g45410  expressed protein similar to unknown protein (pir||T05524) | | | | | | |
|  |  | RAFL05-07-I21 | At3g07350 / expressed protein | |  |  |  |  |  | | --- | --- | --- | --- | --- | |  |  |  |  |  | | RAFL05-07-I21 ,At3g07350  expressed protein contains Pfam profile PF04720: Protein of unknown function (DUF506) | | | | | | |
|  |  | RAFL05-09-B20 | At3g08590 / 2,3-bisphosphoglycerate-independent phosphoglycerate mutase -related | |  |  |  |  |  | | --- | --- | --- | --- | --- | |  |  |  |  |  | | RAFL05-09-B20 ,At3g08590  2,3-biphosphoglycerate-independent phosphoglycerate mutase, putative / phosphoglyceromutase, putative strong similarity to SP|Q42908 2,3-bisphosphoglycerate-independent phosphoglycerate mutase (EC 5.4.2.1) (Phosphoglyceromutase) {Mesembryanthemum crystallinum}; contains Pfam profile PF01676: Metalloenzyme superfamily | | | | | | |
|  |  | RAFL07-17-O09 | At3g51250 / expressed protein | |  |  |  |  |  | | --- | --- | --- | --- | --- | |  |  |  |  |  | | RAFL07-17-O09 ,At3g51250  senescence/dehydration-associated protein-related similar to senescence-associated protein 12 [Hemerocallis hybrid cultivar] gi|3551958|gb|AAC34857; similar to early-responsive to dehydration stress ERD7 protein [Arabidopsis thaliana] gi|15320412|dbj|BAB63916 | | | | | | |
|  |  | RAFL09-16-J07 | At5g06600 / ubiquitin-specific protease 12 (UBP12) | |  |  |  |  |  | | --- | --- | --- | --- | --- | |  |  |  |  |  | | At5g06600 ,RAFL09-16-J07  ubiquitin-specific protease 12 (UBP12) almost identical to ubiquitin-specific protease 12 GI:11993471 [Arabidopsis thaliana], one amino acid difference | | | | | | |
|  |  | RAFL06-08-K09 | At1g23190 / phosphoglucomutase -related | |  |  |  |  |  | | --- | --- | --- | --- | --- | |  |  |  |  |  | | RAFL06-08-K09 ,At1g23190  phosphoglucomutase, cytoplasmic, putative / glucose phosphomutase, putative strong similarity to SP|P93805 Phosphoglucomutase, cytoplasmic 2 (EC 5.4.2.2) (Glucose phosphomutase 2) (PGM 2) {Zea mays}; contains InterPro accession IPR006352: Phosphoglucosamine mutase | | | | | | |
|  |  | RAFL07-09-J05 | At1g51710 / ubiquitin-specific protease 6 (UBP6), putative | |  |  |  |  |  | | --- | --- | --- | --- | --- | |  |  |  |  |  | | RAFL07-09-J05 ,At1g51710  ubiquitin-specific protease 6, putative (UBP6) similar to GI:11993465 | | | | | | |
|  |  | RAFL08-15-K23 | At1g23960 / expressed protein | |  |  |  |  |  | | --- | --- | --- | --- | --- | |  |  |  |  |  | | RAFL08-15-K23 ,At1g23960  expressed protein contains Pfam profile PF04776: Protein of unknown function (DUF626) | | | | | | |
|  |  | RAFL07-18-C02 | At1g16810 / expressed protein | |  |  |  |  |  | | --- | --- | --- | --- | --- | |  |  |  |  |  | | At1g16810 ,RAFL07-18-C02  expressed protein | | | | | | |
|  |  | RAFL06-08-O03 | At4g34890 / xanthine dehydrogenase, putative | |  |  |  |  |  | | --- | --- | --- | --- | --- | |  |  |  |  |  | | RAFL06-08-O03 ,At4g34890  xanthine dehydrogenase, putative similar to xanthine dehydrogenase from Gallus gallus, PIR:XOCHDH [SP|P47990]; contains Pfam profile PF02738 Aldehyde oxidase and xanthine dehydrogenase, molybdopterin binding domain | | | | | | |
|  |  | RAFL05-21-E21 | At5g15860 / Carboxylesterase-related protein | |  |  |  |  |  | | --- | --- | --- | --- | --- | |  |  |  |  |  | | RAFL05-21-E21 ,At5g15860  expressed protein | | | | | | |
|  |  | RAFL11-10-D21 | At5g57630 / CBL-interacting protein kinase 21 | |  |  |  |  |  | | --- | --- | --- | --- | --- | |  |  |  |  |  | | RAFL11-10-D21 ,At5g57630  CBL-interacting protein kinase 21, putative (CIPK21) identical to CBL-interacting protein kinase 21 [Arabidopsis thaliana] gi|14334390|gb|AAK59696 | | | | | | |
|  |  | RAFL04-16-A04 | At3g02470 / S-adenosylmethionine decarboxylase | |  |  |  |  |  | | --- | --- | --- | --- | --- | |  |  |  |  |  | | At3g02470 ,RAFL04-16-A04  adenosylmethionine decarboxylase family protein contains Pfam profile: PF01536 adenosylmethionine decarboxylase | | | | | | |
| GO:0009987 | | | cellular process |  | A | B | C | D | P | P' | N |
|  | Cluster:2-1 | |  |  | 119 | 125 | 1347 | 3072 | 6.8750077E-9 | 2.2687524E-7 | 33 |
|  |  | RAFL09-15-G07 | At3g62250 / ubiquitin extension protein (UBQ5)/40S ribosomal protein S27A (RPS27aC) | |  |  |  |  |  | | --- | --- | --- | --- | --- | |  |  |  |  |  | | RAFL09-15-G07 ,At3g62250  ubiquitin extension protein 5 (UBQ5) / 40S ribosomal protein S27A (RPS27aC) identical to GI:166933, GI:166934 | | | | | | |
|  |  | RAFL07-11-J16 | At3g15950 / expressed protein | |  |  |  |  |  | | --- | --- | --- | --- | --- | |  |  |  |  |  | | RAFL07-11-J16 ,At3g15950  DNA topoisomerase-related similar to DNA topoisomerase IV subunit A (GI:26454107) [Mycoplasma penetrans] | | | | | | |
|  |  | RAFL08-13-A03 | At1g63010 / expressed protein | |  |  |  |  |  | | --- | --- | --- | --- | --- | |  |  |  |  |  | | At1g63010 ,RAFL08-13-A03  SPX (SYG1/Pho81/XPR1) domain-containing protein contains Pfam profile PF03105: SPX domain | | | | | | |
|  |  | RAFL09-07-O12 | At3g26520 / tonoplast intrinsic protein, putative | |  |  |  |  |  | | --- | --- | --- | --- | --- | |  |  |  |  |  | | At3g26520 ,RAFL09-07-O12  tonoplast intrinsic protein, putative similar to tonoplast intrinsic protein GI:5081419 from [Brassica napus] | | | | | | |
|  |  | RAFL08-15-K17 | At5g07090 / 40S ribosomal protein S4 (RPS4B) | |  |  |  |  |  | | --- | --- | --- | --- | --- | |  |  |  |  |  | | At5g07090 ,RAFL08-15-K17  40S ribosomal protein S4 (RPS4B) | | | | | | |
|  |  | RAFL09-14-E24 | At1g74910 / ADP-glucose pyrophosphorylase family | |  |  |  |  |  | | --- | --- | --- | --- | --- | |  |  |  |  |  | | At1g74910 ,RAFL09-14-E24  ADP-glucose pyrophosphorylase family protein contains Pfam profile PF00483: Nucleotidyl transferase; low similarity to mannose-1-phosphate guanylyltransferase [Hypocrea jecorina] GI:3323397 | | | | | | |
|  |  | RAFL11-10-K08 | At3g22230 / 60S ribosomal protein L27 (RPL27B) | |  |  |  |  |  | | --- | --- | --- | --- | --- | |  |  |  |  |  | | At3g22230 ,RAFL11-10-K08  60S ribosomal protein L27 (RPL27B) similar to 60S RIBOSOMAL PROTEIN L27 GB:P41101 from [Solanum tuberosum] | | | | | | |
|  |  | RAFL05-17-M03 | At5g56710 / 60S ribosomal protein L31 (RPL31C) | |  |  |  |  |  | | --- | --- | --- | --- | --- | |  |  |  |  |  | | At5g56710 ,RAFL05-17-M03  60S ribosomal protein L31 (RPL31C) | | | | | | |
|  |  | RAFL05-17-L17 | At3g55280 / 60S ribosomal protein L23A (RPL23aB) | |  |  |  |  |  | | --- | --- | --- | --- | --- | |  |  |  |  |  | | RAFL05-17-L17 ,At3g55280  60S ribosomal protein L23A (RPL23aB) various ribosomal L23a proteins | | | | | | |
|  |  | RAFL09-12-B05 | At5g02870 / 60S ribosomal protein L4/L1 (RPL4D) | |  |  |  |  |  | | --- | --- | --- | --- | --- | |  |  |  |  |  | | At5g02870 ,RAFL09-12-B05  60S ribosomal protein L4/L1 (RPL4D) 60S roibosomal protein L4, Arabidopsis thaliana, EMBL:CAA79104 | | | | | | |
|  |  | RAFL02-02-F05 | At5g58420 / 40S ribosomal protein S4 (RPS4D) | |  |  |  |  |  | | --- | --- | --- | --- | --- | |  |  |  |  |  | | RAFL02-02-F05 ,At5g58420  40S ribosomal protein S4 (RPS4D) ribosomal protein S4, Arabidopsis thaliana, PIR:T48480 | | | | | | |
|  |  | RAFL08-10-A13 | At5g04590 / sulphite reductase | |  |  |  |  |  | | --- | --- | --- | --- | --- | |  |  |  |  |  | | At5g04590 ,RAFL08-10-A13  sulfite reductase / ferredoxin (SIR) identical to sulfite reductase [Arabidopsis thaliana] GI:804953, GI:2584721 | | | | | | |
|  |  | RAFL11-01-L20 | At4g28750 / photosystem I subunit PSI-E - like protein | |  |  |  |  |  | | --- | --- | --- | --- | --- | |  |  |  |  |  | | RAFL11-01-L20 ,At4g28750  photosystem I reaction center subunit IV, chloroplast, putative / PSI-E, putative (PSAE1) identical to SP|Q9S831; similar to SP|P12354 Photosystem I reaction center subunit IV, chloroplast precursor (PSI-E) {Spinacia oleracea}; contains Pfam profile PF02427: Photosystem I reaction centre subunit IV / PsaE | | | | | | |
|  |  | RAFL05-21-L03 | At3g23400 / plastid-lipid associated protein PAP/fibrillin family | |  |  |  |  |  | | --- | --- | --- | --- | --- | |  |  |  |  |  | | RAFL05-21-L03 ,At3g23400  plastid-lipid associated protein PAP / fibrillin family protein contains Pfam profile PF04755: PAP\_fibrillin | | | | | | |
|  |  | RAFL07-13-J18 | At2g37270 / 40S ribosomal protein S5 (RPS5A) | |  |  |  |  |  | | --- | --- | --- | --- | --- | |  |  |  |  |  | | At2g37270 ,RAFL07-13-J18  40S ribosomal protein S5 (RPS5A) identical to GP:3043428 | | | | | | |
|  |  | RAFL07-13-K09 | At5g11800 / K+ efflux antiporter, putative (KEA6) | |  |  |  |  |  | | --- | --- | --- | --- | --- | |  |  |  |  |  | | RAFL07-13-K09 ,At5g11800  K+ efflux antiporter, putative (KEA6) Monovalent cation:proton antiporter family 2 (CPA2 family) member, PMID:11500563; similar to glutathione-regulated potassium-efflux system protein KEFB, Escherichia coli, SWISSPROT:P45522 | | | | | | |
|  |  | RAFL04-12-O11 | At3g27740 / carbamoyl-phosphate synthase (glutamine-hydrolyzing) (glutamine-dependent carbamoyl-phosphate synthase) small subunit | |  |  |  |  |  | | --- | --- | --- | --- | --- | |  |  |  |  |  | | At3g27740 ,RAFL04-12-O11  carbamoyl-phosphate synthase [glutamine-hydrolyzing] (CARA) / glutamine-dependent carbamoyl-phosphate synthase small subunit identical to carbamoyl phosphate synthetase small subunit GI:2462781 [Arabidopsis thaliana] | | | | | | |
|  |  | RAFL06-14-I03 | At3g60770 / 40S ribosomal protein S13 (RPS13A) | |  |  |  |  |  | | --- | --- | --- | --- | --- | |  |  |  |  |  | | RAFL06-14-I03 ,At3g60770  40S ribosomal protein S13 (RPS13A) AtRPS13A mRNA for cytoplasmic ribosomal protein S13, Arabidopsis thaliana,AB031739 | | | | | | |
|  |  | RAFL09-17-G12 | At2g15620 / ferredoxin--nitrite reductase | |  |  |  |  |  | | --- | --- | --- | --- | --- | |  |  |  |  |  | | RAFL09-17-G12 ,At2g15620  ferredoxin--nitrite reductase, putative strong similarity to ferredoxin--nitrite reductase [Nicotiana tabacum] GI:19893; contains Pfam profiles PF03460: Nitrite/Sulfite reductase ferredoxin-like half domain, PF01077: Nitrite and sulphite reductase 4Fe-4S domain | | | | | | |
|  |  | RAFL09-07-C06 | At2g34430 / photosystem II type I chlorophyll a /b binding protein | |  |  |  |  |  | | --- | --- | --- | --- | --- | |  |  |  |  |  | | At2g34430 ,RAFL09-07-C06  chlorophyll A-B binding protein / LHCII type I (LHB1B1) identical to photosystem II type I chlorophyll a/b binding protein [Arabidopsis thaliana] GI:16366 | | | | | | |
|  |  | RAFL05-08-H10 | At4g16720 / 60S ribosomal protein L15 (RPL15A) | |  |  |  |  |  | | --- | --- | --- | --- | --- | |  |  |  |  |  | | RAFL05-08-H10 ,At4g16720  60S ribosomal protein L15 (RPL15A) | | | | | | |
|  |  | RAFL05-17-F03 | At2g32060 / 40S ribosomal protein S12 (RPS12C) | |  |  |  |  |  | | --- | --- | --- | --- | --- | |  |  |  |  |  | | RAFL05-17-F03 ,At2g32060  40S ribosomal protein S12 (RPS12C) | | | | | | |
|  |  | RAFL06-09-H09 | At3g48930 / 40S ribosomal protein S11 (RPS11A) | |  |  |  |  |  | | --- | --- | --- | --- | --- | |  |  |  |  |  | | At3g48930 ,RAFL06-09-H09  40S ribosomal protein S11 (RPS11A) | | | | | | |
|  |  | RAFL06-15-A09 | At1g59359 / 40S ribosomal protein S2 (RPS2B) | |  |  |  |  |  | | --- | --- | --- | --- | --- | |  |  |  |  |  | | RAFL06-15-A09 ,At1g59359  40S ribosomal protein S2 (RPS2B) similar to ribosomal protein S2 GI:430711 from [Drosophila melanogaster] | | | | | | |
|  |  | RAFL06-08-I04 | At1g07920 / elongation factor 1-alpha (EF-1-alpha) | |  |  |  |  |  | | --- | --- | --- | --- | --- | |  |  |  |  |  | | RAFL06-08-I04 ,At1g07920  elongation factor 1-alpha / EF-1-alpha identical to GB:CAA34456 from [Arabidopsis thaliana] (Plant Mol. Biol. 14 (1), 107-110 (1990)) | | | | | | |
|  |  | RAFL05-03-N22 | At3g15410 / leucine rich repeat protein family | |  |  |  |  |  | | --- | --- | --- | --- | --- | |  |  |  |  |  | | RAFL05-03-N22 ,At3g15410  leucine-rich repeat family protein contains leucine rich-repeat (LRR) domains Pfam:PF00560, INTERPRO:IPR001611; contains similarity to Hcr2-5D [Lycopersicon esculentum] gi|3894393|gb|AAC78596; identical to leucine-rich repeat protein [Arabidopsis thaliana] gi|2760084|emb|CAA76000 | | | | | | |
|  |  | RAFL05-07-H16 | At3g53870 / 40S ribosomal protein S3 (RPS3B) | |  |  |  |  |  | | --- | --- | --- | --- | --- | |  |  |  |  |  | | At3g53870 ,RAFL05-07-H16  40S ribosomal protein S3 (RPS3B) ribosomal protein S3a - Xenopus laevis, PIR:R3XL3A | | | | | | |
|  |  | RAFL05-02-G09 | At5g41520 / expressed protein | |  |  |  |  |  | | --- | --- | --- | --- | --- | |  |  |  |  |  | | RAFL05-02-G09 ,At5g41520  40S ribosomal protein S10 (RPS10B) contains similarity to 40S ribosomal protein S10 | | | | | | |
|  |  | RAFL04-13-M20 | At4g11010 / nucleoside diphosphate kinase 3 (ndpk3) | |  |  |  |  |  | | --- | --- | --- | --- | --- | |  |  |  |  |  | | At4g11010 ,RAFL04-13-M20  nucleoside diphosphate kinase 3, mitochondrial (NDK3) identical to Nucleoside diphosphate kinase III, mitochondrial precursor (NDK III) (NDP kinase III) (NDPK III) (SP:O49203) [Arabidopsis thaliana]; contains Pfam PF00334 : Nucleoside diphosphate kinase domain; | | | | | | |
|  |  | RAFL07-15-M07 | At1g04480 / 60S ribosomal protein L23 (RPL23A) | |  |  |  |  |  | | --- | --- | --- | --- | --- | |  |  |  |  |  | | At1g04480 ,RAFL07-15-M07  60S ribosomal protein L23 (RPL23A) identical to GB:AAB80655 | | | | | | |
|  |  | RAFL07-17-P17 | At2g15620 / ferredoxin--nitrite reductase | |  |  |  |  |  | | --- | --- | --- | --- | --- | |  |  |  |  |  | | RAFL07-17-P17 ,At2g15620  ferredoxin--nitrite reductase, putative strong similarity to ferredoxin--nitrite reductase [Nicotiana tabacum] GI:19893; contains Pfam profiles PF03460: Nitrite/Sulfite reductase ferredoxin-like half domain, PF01077: Nitrite and sulphite reductase 4Fe-4S domain | | | | | | |
|  |  | RAFL05-14-D21 | At1g18540 / 60S ribosomal protein L6 (RPL6A) | |  |  |  |  |  | | --- | --- | --- | --- | --- | |  |  |  |  |  | | At1g18540 ,RAFL05-14-D21  60S ribosomal protein L6 (RPL6A) similar to 60S ribosomal protein L6 GI:7208784 from [Cicer arietinum] | | | | | | |
|  |  | RAFL08-11-K22 | At1g57660 / 60S ribosomal protein L21 (RPL21E) | |  |  |  |  |  | | --- | --- | --- | --- | --- | |  |  |  |  |  | | At1g57660 ,RAFL08-11-K22  60S ribosomal protein L21 (RPL21E) similar to 60S ribosomal protein L21 GB:Q43291 GI:2851508 from [Arabidopsis thaliana] | | | | | | |
|  |  | RAFL11-12-H04 | At3g25520 / 60S ribosomal protein L5 (RPL5A) | |  |  |  |  |  | | --- | --- | --- | --- | --- | |  |  |  |  |  | | At3g25520 ,RAFL11-12-H04  60S ribosomal protein L5 similar to 60S ribosomal protein L5 GB:P49625 from [Oryza sativa] | | | | | | |
|  |  | RAFL04-18-N22 | At2g44120 / 60S ribosomal protein L7 (RPL7C) | |  |  |  |  |  | | --- | --- | --- | --- | --- | |  |  |  |  |  | | RAFL04-18-N22 ,At2g44120  60S ribosomal protein L7 (RPL7C) | | | | | | |
|  |  | RAFL04-16-H12 | At1g67560 / lipoxygenase family | |  |  |  |  |  | | --- | --- | --- | --- | --- | |  |  |  |  |  | | At1g67560 ,RAFL04-16-H12  lipoxygenase family protein similar to 13-lipoxygenase GB:CAA65269 [Solanum tuberosum], gi:1654140 [Lycopersicon esculentum] | | | | | | |
|  |  | RAFL08-10-I20 | At1g52410 / myosin-related protein | |  |  |  |  |  | | --- | --- | --- | --- | --- | |  |  |  |  |  | | At1g52410 ,RAFL08-10-I20  caldesmon-related weak similarity to Caldesmon (CDM) (Swiss-Prot:P12957) [Gallus gallus] | | | | | | |
|  |  | RAFL04-13-I18 | At5g67070 / expressed protein | |  |  |  |  |  | | --- | --- | --- | --- | --- | |  |  |  |  |  | | RAFL04-13-I18 ,At5g67070  rapid alkalinization factor (RALF) family protein similar to RALF precursor [Nicotiana tabacum] GI:16566316 | | | | | | |
|  |  | RAFL07-08-E18 | At1g32550 / ferredoxin family | |  |  |  |  |  | | --- | --- | --- | --- | --- | |  |  |  |  |  | | At1g32550 ,RAFL07-08-E18  ferredoxin family protein similar to ferredoxin from Synechocystis sp. [GI:48019]; contains Pfam profile PF00111 2Fe-2S iron-sulfur cluster binding domain | | | | | | |
|  |  | RAFL06-13-D11 | At3g14120 / expressed protein | |  |  |  |  |  | | --- | --- | --- | --- | --- | |  |  |  |  |  | | At3g14120 ,RAFL06-13-D11  expressed protein similar to Nuclear pore complex protein Nup107 (Nucleoporin Nup107) (107 kDa nucleoporin) (p105) (Swiss-Prot:P52590) [Rattus norvegicus] | | | | | | |
|  |  | RAFL02-01-G08 | At3g58610 / ketol-acid reductoisomerase | |  |  |  |  |  | | --- | --- | --- | --- | --- | |  |  |  |  |  | | RAFL02-01-G08 ,At3g58610  ketol-acid reductoisomerase identical to ketol-acid reductoisomerase, chloroplast precursor (EC 1.1.1.86) (Acetohydroxy-acid reductoisomerase) (Alpha-keto-beta-hydroxylacil reductoisomerase) (Swiss-Prot:Q05758) [Arabidopsis thaliana] | | | | | | |
|  |  | RAFL09-07-D04 | At3g22230 / 60S ribosomal protein L27 (RPL27B) | |  |  |  |  |  | | --- | --- | --- | --- | --- | |  |  |  |  |  | | At3g22230 ,RAFL09-07-D04  60S ribosomal protein L27 (RPL27B) similar to 60S RIBOSOMAL PROTEIN L27 GB:P41101 from [Solanum tuberosum] | | | | | | |
|  |  | RAFL04-10-F11 | At5g59850 / 40S ribosomal protein S15A (RPS15aF) | |  |  |  |  |  | | --- | --- | --- | --- | --- | |  |  |  |  |  | | At5g59850 ,RAFL04-10-F11  40S ribosomal protein S15A (RPS15aF) cytoplasmic ribosomal protein S15a, Arabidopsis thaliana, EMBL:ATAF1412 | | | | | | |
|  |  | RAFL04-15-K13 | At5g66510 / transferase hexapeptide repeat family | |  |  |  |  |  | | --- | --- | --- | --- | --- | |  |  |  |  |  | | At5g66510 ,RAFL04-15-K13  bacterial transferase hexapeptide repeat-containing protein contains Pfam profile PF00132: Bacterial transferase hexapeptide (four repeats) | | | | | | |
|  |  | RAFL04-18-N10 | At1g07770 / 40S ribosomal protein S15A (RPS15aA) | |  |  |  |  |  | | --- | --- | --- | --- | --- | |  |  |  |  |  | | RAFL04-18-N10 ,At1g07770  40S ribosomal protein S15A (RPS15aA) identical to GB:AAA61608 from [Arabidopsis thaliana] (Plant Physiol. 106 (1), 401-402 (1994)) | | | | | | |
|  |  | RAFL05-18-M20 | At2g19730 / 60S ribosomal protein L28 (RPL28A) | |  |  |  |  |  | | --- | --- | --- | --- | --- | |  |  |  |  |  | | At2g19730 ,RAFL05-18-M20  60S ribosomal protein L28 (RPL28A) | | | | | | |
|  |  | RAFL07-10-A20 | At3g54010 / peptidylprolyl isomerase (pasticcino 1) | |  |  |  |  |  | | --- | --- | --- | --- | --- | |  |  |  |  |  | | RAFL07-10-A20 ,At3g54010  peptidyl-prolyl cis-trans isomerase, putative / FK506-binding protein, putative / pasticcino 1-D (PAS1-D) nearly identical to pasticcino 1-D [Arabidopsis thaliana] GI:3080740 | | | | | | |
|  |  | RAFL07-08-E24 | At5g27850 / 60S ribosomal protein L18 (RPL18C) | |  |  |  |  |  | | --- | --- | --- | --- | --- | |  |  |  |  |  | | RAFL07-08-E24 ,At5g27850  60S ribosomal protein L18 (RPL18C) 60S ribosomal protein L18, Arabidopsis thaliana, SWISSPROT:RL18\_ARATH | | | | | | |
|  |  | RAFL06-15-B04 | At1g14810 / aspartate-semialdehyde dehydrogenase -related | |  |  |  |  |  | | --- | --- | --- | --- | --- | |  |  |  |  |  | | At1g14810 ,RAFL06-15-B04  semialdehyde dehydrogenase family protein similar to SP:O31219 Aspartate-semialdehyde dehydrogenase (EC 1.2.1.11) (ASA dehydrogenase) (ASADH) {Legionella pneumophila}; contains Pfam profiles PF02774: Semialdehyde dehydrogenase dimerisation domain, PF01118: Semialdehyde dehydrogenase NAD binding domain | | | | | | |
|  |  | RAFL05-21-C12 | At4g13430 / aconitase family | |  |  |  |  |  | | --- | --- | --- | --- | --- | |  |  |  |  |  | | RAFL05-21-C12 ,At4g13430  aconitase family protein / aconitate hydratase family protein contains Pfam profile PF00330: Aconitase family (aconitate hydratase | | | | | | |
|  |  | RAFL11-07-B21 | At3g25520 / 60S ribosomal protein L5 (RPL5A) | |  |  |  |  |  | | --- | --- | --- | --- | --- | |  |  |  |  |  | | At3g25520 ,RAFL11-07-B21  60S ribosomal protein L5 similar to 60S ribosomal protein L5 GB:P49625 from [Oryza sativa] | | | | | | |
|  |  | RAFL06-12-N06 | At3g53580 / diaminopimelate epimerase - like protein | |  |  |  |  |  | | --- | --- | --- | --- | --- | |  |  |  |  |  | | At3g53580 ,RAFL06-12-N06  diaminopimelate epimerase family protein contains Pfam profile PF01678: Diaminopimelate epimerase | | | | | | |
|  |  | RAFL04-20-F03 | At5g39740 / 60S ribosomal protein L5 (RPL5B) | |  |  |  |  |  | | --- | --- | --- | --- | --- | |  |  |  |  |  | | RAFL04-20-F03 ,At5g39740  60S ribosomal protein L5 (RPL5B) ribosomal protein L5, rice | | | | | | |
|  |  | RAFL11-05-B21 | At3g54210 / ribosomal protein L17 -related protein | |  |  |  |  |  | | --- | --- | --- | --- | --- | |  |  |  |  |  | | At3g54210 ,RAFL11-05-B21  ribosomal protein L17 family protein contains Pfam profile: PF01196 ribosomal protein L17 | | | | | | |
|  |  | RAFL07-18-J24 | At3g02690 / expressed integral membrane protein | |  |  |  |  |  | | --- | --- | --- | --- | --- | |  |  |  |  |  | | RAFL07-18-J24 ,At3g02690  integral membrane family protein similar to PecM protein (GI:5852331) {Vogesella indigofera} and PecM protein (SP:P42194) [Erwinia chrysanthemi] | | | | | | |
|  |  | RAFL06-15-P19 | At2g31750 / glycosyltransferase family | |  |  |  |  |  | | --- | --- | --- | --- | --- | |  |  |  |  |  | | At2g31750 ,RAFL06-15-P19  UDP-glucoronosyl/UDP-glucosyl transferase family protein contains Pfam profile: PF00201 UDP-glucoronosyl and UDP-glucosyl transferase | | | | | | |
|  |  | RAFL05-02-K09 | At5g22440 / 60S ribosomal protein L10A (RPL10aC) | |  |  |  |  |  | | --- | --- | --- | --- | --- | |  |  |  |  |  | | RAFL05-02-K09 ,At5g22440  60S ribosomal protein L10A (RPL10aC) | | | | | | |
|  |  | RAFL02-03-G08 | At4g18730 / 60S ribosomal protein L11 (RPL11C) | |  |  |  |  |  | | --- | --- | --- | --- | --- | |  |  |  |  |  | | RAFL02-03-G08 ,At4g18730  60S ribosomal protein L11 (RPL11C) | | | | | | |
|  |  | RAFL05-05-M24 | At5g02960 / 40S ribosomal protein S23 (RPS23B) | |  |  |  |  |  | | --- | --- | --- | --- | --- | |  |  |  |  |  | | At5g02960 ,RAFL05-05-M24  40S ribosomal protein S23 (RPS23B) ribosomal protein S23, Fragaria x ananassa, PIR:S56673 | | | | | | |
|  |  | RAFL05-10-B02 | At5g64460 / ZW10-related protein | |  |  |  |  |  | | --- | --- | --- | --- | --- | |  |  |  |  |  | | At5g64460 ,RAFL05-10-B02  expressed protein | | | | | | |
|  |  | RAFL04-13-D06 | At3g23940 / dihydroxyacid dehydratase -related | |  |  |  |  |  | | --- | --- | --- | --- | --- | |  |  |  |  |  | | RAFL04-13-D06 ,At3g23940  dehydratase family contains Pfam profile: PF00920 dehydratase family | | | | | | |
|  |  | RAFL06-15-H04 | At2g43750 / cysteine synthase, chloroplast (O-acetylserine (thiol)-lyase/O-acetylserine sulfhydrylase/cpACS1) | |  |  |  |  |  | | --- | --- | --- | --- | --- | |  |  |  |  |  | | At2g43750 ,RAFL06-15-H04  cysteine synthase, chloroplast / O-acetylserine (thiol)-lyase / O-acetylserine sulfhydrylase / cpACS1 (OASB) identical to SP|P47999 Cysteine synthase, chloroplast precursor (EC 4.2.99.8) (O-acetylserine sulfhydrylase) (O-acetylserine (Thiol)-lyase) (cpACS1) {Arabidopsis thaliana}; identical to cDNA O-acetylserine lyase (At.OAS.7-4) GI:6983575 | | | | | | |
|  |  | RAFL07-13-J05 | At1g07920 / elongation factor 1-alpha (EF-1-alpha) | |  |  |  |  |  | | --- | --- | --- | --- | --- | |  |  |  |  |  | | RAFL07-13-J05 ,At1g07920  elongation factor 1-alpha / EF-1-alpha identical to GB:CAA34456 from [Arabidopsis thaliana] (Plant Mol. Biol. 14 (1), 107-110 (1990)) | | | | | | |
|  |  | RAFL04-16-K24 | At2g44870 / expressed protein | |  |  |  |  |  | | --- | --- | --- | --- | --- | |  |  |  |  |  | | At2g44870 ,RAFL04-16-K24  expressed protein | | | | | | |
|  |  | RAFL05-18-P15 | At1g04480 / 60S ribosomal protein L23 (RPL23A) | |  |  |  |  |  | | --- | --- | --- | --- | --- | |  |  |  |  |  | | At1g04480 ,RAFL05-18-P15  60S ribosomal protein L23 (RPL23A) identical to GB:AAB80655 | | | | | | |
|  |  | RAFL07-18-J07 | At3g23530 / cyclopropane synthase, putative | |  |  |  |  |  | | --- | --- | --- | --- | --- | |  |  |  |  |  | | RAFL07-18-J07 ,At3g23530  cyclopropane fatty acid synthase, putative / CPA-FA synthase, putative similar to cyclopropane synthase [Sterculia foetida] GI:21069167; contains Pfam profiles PF02353: Cyclopropane-fatty-acyl-phospholipid synthase, PF01593: amine oxidase, flavin-containing | | | | | | |
|  |  | RAFL04-09-J06 | At1g70600 / 60S ribosomal protein L27A (RPL27aC) | |  |  |  |  |  | | --- | --- | --- | --- | --- | |  |  |  |  |  | | At1g70600 ,RAFL04-09-J06  60S ribosomal protein L27A (RPL27aC) identical to 60S ribosomal protein L27A GB:P49637 [Arabidopsis thaliana] | | | | | | |
|  |  | RAFL09-15-M15 | At5g23740 / 40S ribosomal protein S11 (RPS11C) | |  |  |  |  |  | | --- | --- | --- | --- | --- | |  |  |  |  |  | | RAFL09-15-M15 ,At5g23740  40S ribosomal protein S11 (RPS11C) | | | | | | |
|  |  | RAFL11-02-I08 | At1g50320 / thioredoxin x | |  |  |  |  |  | | --- | --- | --- | --- | --- | |  |  |  |  |  | | At1g50320 ,RAFL11-02-I08  thioredoxin x nearly identical to thioredoxin x GB:AAF15952 GI:6539616 from [Arabidopsis thaliana] | | | | | | |
|  |  | RAFL05-12-E15 | At2g31610 / 40S ribosomal protein S3 (RPS3A) | |  |  |  |  |  | | --- | --- | --- | --- | --- | |  |  |  |  |  | | RAFL05-12-E15 ,At2g31610  40S ribosomal protein S3 (RPS3A) | | | | | | |
|  |  | RAFL04-14-L08 | At3g49010 / 60S ribosomal protein L13 (RPL13B)/breast basic conserved protein 1-related (BBC1) | |  |  |  |  |  | | --- | --- | --- | --- | --- | |  |  |  |  |  | | At3g49010 ,RAFL04-14-L08  60S ribosomal protein L13 (RPL13B) / breast basic conserved protein 1-related (BBC1) | | | | | | |
|  |  | RAFL08-09-J20 | At5g01530 / light-harvesting chlorophyll a/b binding protein | |  |  |  |  |  | | --- | --- | --- | --- | --- | |  |  |  |  |  | | RAFL08-09-J20 ,At5g01530  chlorophyll A-B binding protein CP29 (LHCB4) identical to CP29 [Arabidopsis thaliana] GI:298036; contains Pfam profile: PF00504 chlorophyll A-B binding protein | | | | | | |
|  |  | RAFL04-20-F17 | At1g02150 / pentatricopeptide (PPR) repeat-containing protein | |  |  |  |  |  | | --- | --- | --- | --- | --- | |  |  |  |  |  | | At1g02150 ,RAFL04-20-F17  pentatricopeptide (PPR) repeat-containing protein low similiarity to DNA-binding protein [Triticum aestivum] GI:6958202; contains Pfam profile: PF01535 PPR repeat | | | | | | |
|  |  | RAFL06-10-J18 | At4g14320 / 60S ribosomal protein L36a/L44 (RPL36aB) | |  |  |  |  |  | | --- | --- | --- | --- | --- | |  |  |  |  |  | | RAFL06-10-J18 ,At4g14320  60S ribosomal protein L36a/L44 (RPL36aB) | | | | | | |
|  |  | RAFL11-09-J03 | At3g54210 / ribosomal protein L17 -related protein | |  |  |  |  |  | | --- | --- | --- | --- | --- | |  |  |  |  |  | | RAFL11-09-J03 ,At3g54210  ribosomal protein L17 family protein contains Pfam profile: PF01196 ribosomal protein L17 | | | | | | |
|  |  | RAFL02-10-A09 | At1g33140 / 60S ribosomal protein L9 (RPL90A/C) | |  |  |  |  |  | | --- | --- | --- | --- | --- | |  |  |  |  |  | | RAFL02-10-A09 ,At1g33140  60S ribosomal protein L9 (RPL90A/C) similar to RIBOSOMAL PROTEIN L9 GB:P49209 from [Arabidopsis thaliana] | | | | | | |
|  |  | RAFL07-15-K08 | At4g34670 / 40S ribosomal protein S3A (RPS3aB) | |  |  |  |  |  | | --- | --- | --- | --- | --- | |  |  |  |  |  | | RAFL07-15-K08 ,At4g34670  40S ribosomal protein S3A (RPS3aB) | | | | | | |
|  |  | RAFL04-18-P17 | At5g35170 / adenylate kinase -related protein | |  |  |  |  |  | | --- | --- | --- | --- | --- | |  |  |  |  |  | | At5g35170 ,RAFL04-18-P17  adenylate kinase family protein contains Pfam profile: PF00406 adenylate kinase | | | | | | |
|  |  | RAFL07-14-M14 | At5g02870 / 60S ribosomal protein L4/L1 (RPL4D) | |  |  |  |  |  | | --- | --- | --- | --- | --- | |  |  |  |  |  | | At5g02870 ,RAFL07-14-M14  60S ribosomal protein L4/L1 (RPL4D) 60S roibosomal protein L4, Arabidopsis thaliana, EMBL:CAA79104 | | | | | | |
|  |  | RAFL09-06-A22 | At3g62870 / 60S ribosomal protein L7A (RPL7aB) | |  |  |  |  |  | | --- | --- | --- | --- | --- | |  |  |  |  |  | | RAFL09-06-A22 ,At3g62870  60S ribosomal protein L7A (RPL7aB) 60S RIBOSOMAL PROTEIN L7A - Oryza sativa, SWISSPROT:RL7A\_ORYSA | | | | | | |
|  |  | RAFL04-09-M12 | At4g27090 / 60S ribosomal protein L14 (RPL14B) | |  |  |  |  |  | | --- | --- | --- | --- | --- | |  |  |  |  |  | | At4g27090 ,RAFL04-09-M12  60S ribosomal protein L14 (RPL14B) ribosomal protein L14 - Human,PIR3:JC5954 | | | | | | |
|  |  | RAFL05-01-G23 | At3g49600 / ubiquitin-specific protease 26 (UBP26) | |  |  |  |  |  | | --- | --- | --- | --- | --- | |  |  |  |  |  | | At3g49600 ,RAFL05-01-G23  ubiquitin-specific protease 26 (UBP26) similar to GI:11993492; RNA binding protein - Homo sapiens, EMBL:AB016089 (N-terminus), several ubiquitin carboxyl-terminal hydrolases from aa pos. 712 | | | | | | |
|  |  | RAFL11-10-L18 | At2g36620 / 60S ribosomal protein L24 (RPL24A) | |  |  |  |  |  | | --- | --- | --- | --- | --- | |  |  |  |  |  | | RAFL11-10-L18 ,At2g36620  60S ribosomal protein L24 (RPL24A) | | | | | | |
|  |  | RAFL02-02-B08 | At3g02540 / RAD23 -related | |  |  |  |  |  | | --- | --- | --- | --- | --- | |  |  |  |  |  | | At3g02540 ,RAFL02-02-B08  ubiquitin family protein contains Pfam profiles PF00240: Ubiquitin family, PF00627: UBA/TS-N domain; | | | | | | |
|  |  | RAFL05-10-D12 | At5g47770 / farnesyl pyrophosphate synthetase 1 (FPS1) (farnesyl diphosphate synthase 1) | |  |  |  |  |  | | --- | --- | --- | --- | --- | |  |  |  |  |  | | RAFL05-10-D12 ,At5g47770  farnesyl pyrophosphate synthetase 1, mitochondrial (FPS1) / FPP synthetase 1 / farnesyl diphosphate synthase 1 identical to SP|Q09152 Farnesyl pyrophosphate synthetase 1, mitochondrial precursor (FPP synthetase 1) (FPS 1) (Farnesyl diphosphate synthetase 1) [Includes: Dimethylallyltransferase (EC 2.5.1.1); Geranyltranstransferase (EC 2.5.1.10)] {Arabidopsis thaliana} | | | | | | |
|  |  | RAFL02-09-C19 | At5g13630 / cobalamin biosynthesis protein | |  |  |  |  |  | | --- | --- | --- | --- | --- | |  |  |  |  |  | | At5g13630 ,RAFL02-09-C19  magnesium-chelatase subunit chlH, chloroplast, putative / Mg-protoporphyrin IX chelatase, putative (CHLH) nearly identical to magnesium chelatase subunit GI:1154627 from [Arabidopsis thaliana]; contains Pfam profile: PF02514 CobN/magnesium chelatase family protein | | | | | | |
|  |  | RAFL02-02-A04 | At3g54890 / light-harvesting chlorophyll a/b binding protein | |  |  |  |  |  | | --- | --- | --- | --- | --- | |  |  |  |  |  | | At3g54890 ,RAFL02-02-A04  chlorophyll A-B binding protein / LHCI type I (CAB) identical to chlorophyll A/B-binding protein [Arabidopsis thaliana] GI:16207; contains Pfam profile: PF00504 chlorophyll A-B binding protein | | | | | | |
|  |  | RAFL06-10-E08 | At2g34480 / 60S ribosomal protein L18A (RPL18aB) | |  |  |  |  |  | | --- | --- | --- | --- | --- | |  |  |  |  |  | | At2g34480 ,RAFL06-10-E08  60S ribosomal protein L18A (RPL18aB) | | | | | | |
|  |  | RAFL06-08-F07 | At4g35100 / aquaporin, putative | |  |  |  |  |  | | --- | --- | --- | --- | --- | |  |  |  |  |  | | At4g35100 ,RAFL06-08-F07  plasma membrane intrinsic protein (SIMIP) nearly identical to plasma membrane intrinsic protein [Arabidopsis thaliana] GI:2306917 | | | | | | |
|  |  | RAFL07-08-L21 | At5g59850 / 40S ribosomal protein S15A (RPS15aF) | |  |  |  |  |  | | --- | --- | --- | --- | --- | |  |  |  |  |  | | At5g59850 ,RAFL07-08-L21  40S ribosomal protein S15A (RPS15aF) cytoplasmic ribosomal protein S15a, Arabidopsis thaliana, EMBL:ATAF1412 | | | | | | |
|  |  | RAFL05-03-L01 | At3g56340 / 40S ribosomal protein S26 homolog | |  |  |  |  |  | | --- | --- | --- | --- | --- | |  |  |  |  |  | | At3g56340 ,RAFL05-03-L01  40S ribosomal protein S26 (RPS26C) several 40S ribosomal protein S26 | | | | | | |
|  |  | RAFL05-19-M17 | At5g51010 / expressed protein | |  |  |  |  |  | | --- | --- | --- | --- | --- | |  |  |  |  |  | | RAFL05-19-M17 ,At5g51010  rubredoxin family protein similar to SP|P04170 Rubredoxin (Rd) {Desulfovibrio desulfuricans}; contains Pfam profile PF00301: Rubredoxin | | | | | | |
|  |  | RAFL06-08-B09 | At3g11510 / 40S ribosomal protein S14 (RPS14B) | |  |  |  |  |  | | --- | --- | --- | --- | --- | |  |  |  |  |  | | At3g11510 ,RAFL06-08-B09  40S ribosomal protein S14 (RPS14B) similar to 40S ribosomal protein S14 GB:P19950 [Zea mays] | | | | | | |
|  |  | RAFL06-08-P20 | At3g07110 / 60S ribosomal protein L13A (RPL13aA) | |  |  |  |  |  | | --- | --- | --- | --- | --- | |  |  |  |  |  | | RAFL06-08-P20 ,At3g07110  60S ribosomal protein L13A (RPL13aA) similar to ribosomal protein L13A GB:O49885 [Lupinus luteus] | | | | | | |
|  |  | RAFL05-16-H14 | At1g04270 / 40S ribosomal protein S15 (RPS15A) | |  |  |  |  |  | | --- | --- | --- | --- | --- | |  |  |  |  |  | | At1g04270 ,RAFL05-16-H14  40S ribosomal protein S15 (RPS15A) Strong similarity to Oryza 40S ribosomal protein S15. ESTs gb|R29788,gb|ATTS0365 come from this gene | | | | | | |
|  |  | RAFL11-02-N24 | At4g15480 / UDP-glycosyltransferase family | |  |  |  |  |  | | --- | --- | --- | --- | --- | |  |  |  |  |  | | At4g15480 ,RAFL11-02-N24  UDP-glucoronosyl/UDP-glucosyl transferase family protein contains Pfam profile: PF00201 UDP-glucoronosyl and UDP-glucosyl transferase | | | | | | |
|  |  | RAFL04-10-H14 | At3g49910 / 60S ribosomal protein L26 (RPL26A) | |  |  |  |  |  | | --- | --- | --- | --- | --- | |  |  |  |  |  | | At3g49910 ,RAFL04-10-H14  60S ribosomal protein L26 (RPL26A) 60S RIBOSOMAL PROTEIN L26, Brassica rapa, EMBL:BRD495 | | | | | | |
|  |  | RAFL05-13-M17 | At3g53890 / 40S ribosomal protein S21 homolog | |  |  |  |  |  | | --- | --- | --- | --- | --- | |  |  |  |  |  | | RAFL05-13-M17 ,At3g53890  40S ribosomal protein S21 (RPS21B) ribosomal protein S21, cytosolic - Oryza sativa, PIR:S38357 | | | | | | |
|  |  | RAFL06-11-K09 | At5g03850 / 40S ribosomal protein S28 (RPS28B) | |  |  |  |  |  | | --- | --- | --- | --- | --- | |  |  |  |  |  | | RAFL06-11-K09 ,At5g03850  40S ribosomal protein S28 (RPS28B) ribosomal protein S28, Arabidopsis thaliana, EMBL:ATRP28A | | | | | | |
|  |  | RAFL05-16-K23 | At1g02780 / 60S ribosomal protein L19 (RPL19A) | |  |  |  |  |  | | --- | --- | --- | --- | --- | |  |  |  |  |  | | RAFL05-16-K23 ,At1g02780  60S ribosomal protein L19 (RPL19A) similar to ribosomal protein L19 GI:36127 from [Homo sapiens] | | | | | | |
|  |  | RAFL05-19-H05 | At3g28900 / 60S ribosomal protein L34 (RPL34C) | |  |  |  |  |  | | --- | --- | --- | --- | --- | |  |  |  |  |  | | RAFL05-19-H05 ,At3g28900  60S ribosomal protein L34 (RPL34C) similar to 60S ribosomal protein L34 GB:P41098 [Nicotiana tabacum] | | | | | | |
|  |  | RAFL05-10-F15 | At1g71900 / expressed protein | |  |  |  |  |  | | --- | --- | --- | --- | --- | |  |  |  |  |  | | RAFL05-10-F15 ,At1g71900  expressed protein | | | | | | |
|  |  | RAFL04-14-J20 | At2g40660 / methionyl-tRNA synthetase -related | |  |  |  |  |  | | --- | --- | --- | --- | --- | |  |  |  |  |  | | RAFL04-14-J20 ,At2g40660  tRNA-binding region domain-containing protein similar to SP|Q12904 Multisynthetase complex auxiliary component p43 [Contains: Endothelial-monocyte activating polypeptide II (EMAP-II) (Small inducible cytokine subfamily E member 1)] {Homo sapiens}; contains Pfam profile PF01588: Putative tRNA binding domain | | | | | | |
|  |  | RAFL06-08-F22 | At5g13650 / GTP-binding protein typA (tyrosine phosphorylated protein A) | |  |  |  |  |  | | --- | --- | --- | --- | --- | |  |  |  |  |  | | At5g13650 ,RAFL06-08-F22  elongation factor family protein contains Pfam profiles: PF00009 elongation factor Tu GTP binding domain,PF00679 elongation factor G C-terminus, PF03144 elongation factor Tu domain 2 | | | | | | |
|  |  | RAFL04-18-P03 | At4g17390 / 60S ribosomal protein L15 (RPL15B) | |  |  |  |  |  | | --- | --- | --- | --- | --- | |  |  |  |  |  | | At4g17390 ,RAFL04-18-P03  60S ribosomal protein L15 (RPL15B) | | | | | | |
|  |  | RAFL05-10-I23 | At1g16720 / expressed protein | |  |  |  |  |  | | --- | --- | --- | --- | --- | |  |  |  |  |  | | At1g16720 ,RAFL05-10-I23  expressed protein | | | | | | |
|  |  | RAFL04-19-M20 | At1g58380 / 40S ribosomal protein S2 (RPS2A) | |  |  |  |  |  | | --- | --- | --- | --- | --- | |  |  |  |  |  | | RAFL04-19-M20 ,At1g58380  40S ribosomal protein S2 (RPS2A) similar to ribosomal protein S2 GI:939717 from (Urechis caupo) | | | | | | |
|  |  | RAFL05-17-P11 | At3g04920 / 40S ribosomal protein S24 (RPS24A) | |  |  |  |  |  | | --- | --- | --- | --- | --- | |  |  |  |  |  | | At3g04920 ,RAFL05-17-P11  40S ribosomal protein S24 (RPS24A) similar to ribosomal protein S19 GB:445612 [Solanum tuberosum] and similar to ribosomal protein S24 GB:4506703 [Homo sapiens] | | | | | | |
|  |  | RAFL05-13-D18 | At3g04400 / 60S ribosomal protein L23 (RPL23C) | |  |  |  |  |  | | --- | --- | --- | --- | --- | |  |  |  |  |  | | At3g04400 ,RAFL05-13-D18  60S ribosomal protein L23 (RPL23C) similar to ribosomal protein L17 GB:AAA34113.1 from [Nicotiana tabacum] | | | | | | |
|  |  | RAFL06-08-P08 | At3g23390 / 60S ribosomal protein L36a/L44 (RPL36aA) | |  |  |  |  |  | | --- | --- | --- | --- | --- | |  |  |  |  |  | | RAFL06-08-P08 ,At3g23390  60S ribosomal protein L36a/L44 (RPL36aA) similar to ribosomal protein L41 GB:AAA34366 from [Candida maltosa] | | | | | | |
|  |  | RAFL05-14-A02 | At5g52650 / 40S ribosomal protein S10 (RPS10C) | |  |  |  |  |  | | --- | --- | --- | --- | --- | |  |  |  |  |  | | At5g52650 ,RAFL05-14-A02  40S ribosomal protein S10 (RPS10C) contains similarity to 40S ribosomal protein S10 | | | | | | |
|  |  | RAFL07-17-J24 | At1g71880 / sucrose transporter SUC1 (sucrose-proton symporter) | |  |  |  |  |  | | --- | --- | --- | --- | --- | |  |  |  |  |  | | At1g71880 ,RAFL07-17-J24  sucrose transporter / sucrose-proton symporter (SUC1) identical to sucrose-proton symporter SUC1 [Arabidopsis thaliana] GI:407094 | | | | | | |
|  |  | RAFL07-08-E22 | At3g23530 / cyclopropane synthase, putative | |  |  |  |  |  | | --- | --- | --- | --- | --- | |  |  |  |  |  | | RAFL07-08-E22 ,At3g23530  cyclopropane fatty acid synthase, putative / CPA-FA synthase, putative similar to cyclopropane synthase [Sterculia foetida] GI:21069167; contains Pfam profiles PF02353: Cyclopropane-fatty-acyl-phospholipid synthase, PF01593: amine oxidase, flavin-containing | | | | | | |
|  |  | RAFL11-06-J19 | At2g01250 / 60S ribosomal protein L7 (RPL7B) | |  |  |  |  |  | | --- | --- | --- | --- | --- | |  |  |  |  |  | | At2g01250 ,RAFL11-06-J19  60S ribosomal protein L7 (RPL7B) | | | | | | |
|  |  | RAFL07-10-K10 | At2g15620 / ferredoxin--nitrite reductase | |  |  |  |  |  | | --- | --- | --- | --- | --- | |  |  |  |  |  | | RAFL07-10-K10 ,At2g15620  ferredoxin--nitrite reductase, putative strong similarity to ferredoxin--nitrite reductase [Nicotiana tabacum] GI:19893; contains Pfam profiles PF03460: Nitrite/Sulfite reductase ferredoxin-like half domain, PF01077: Nitrite and sulphite reductase 4Fe-4S domain | | | | | | |
|  |  | RAFL04-16-M03 | At3g20790 / expressed protein | |  |  |  |  |  | | --- | --- | --- | --- | --- | |  |  |  |  |  | | At3g20790 ,RAFL04-16-M03  oxidoreductase family protein weak similarity to SP|Q07982 Glucose--fructose oxidoreductase precursor (EC 1.1.99.28) {Zymomonas mobilis}; contains Pfam profiles PF01408: Oxidoreductase family NAD-binding Rossmann fold, PF02894: Oxidoreductase family C-terminal alpha/beta domain | | | | | | |
|  |  | RAFL11-09-C11 | At2g01250 / 60S ribosomal protein L7 (RPL7B) | |  |  |  |  |  | | --- | --- | --- | --- | --- | |  |  |  |  |  | | At2g01250 ,RAFL11-09-C11  60S ribosomal protein L7 (RPL7B) | | | | | | |
|  |  | RAFL08-13-H16 | At2g15620 / ferredoxin--nitrite reductase | |  |  |  |  |  | | --- | --- | --- | --- | --- | |  |  |  |  |  | | RAFL08-13-H16 ,At2g15620  ferredoxin--nitrite reductase, putative strong similarity to ferredoxin--nitrite reductase [Nicotiana tabacum] GI:19893; contains Pfam profiles PF03460: Nitrite/Sulfite reductase ferredoxin-like half domain, PF01077: Nitrite and sulphite reductase 4Fe-4S domain | | | | | | |
|  |  | RAFL05-08-L19 | At3g43540 / expressed protein | |  |  |  |  |  | | --- | --- | --- | --- | --- | |  |  |  |  |  | | At3g43540 ,RAFL05-08-L19  expressed protein hypothetical protein slr1699 - Synechocystis sp. (strain PCC 6803), PIR:S75306 | | | | | | |
|  | Cluster:3-0 | |  |  | 102 | 131 | 1364 | 3066 | 6.215158E-5 | 0.002051002 | 33 |
|  |  | RAFL07-15-M24 | At1g74050 / 60S ribosomal protein L6 (RPL6C) | |  |  |  |  |  | | --- | --- | --- | --- | --- | |  |  |  |  |  | | At1g74050 ,RAFL07-15-M24  60S ribosomal protein L6 (RPL6C) similar to 60S ribosomal protein L6 (YL 16 like) GB:CAB57309 from [Cyanophora paradoxa] | | | | | | |
|  |  | RAFL05-18-A12 | At1g15690 / inorganic pyrophosphatase -related | |  |  |  |  |  | | --- | --- | --- | --- | --- | |  |  |  |  |  | | At1g15690 ,RAFL05-18-A12  pyrophosphate-energized vacuolar membrane proton pump / pyrophosphate-energized inorganic pyrophosphatase (AVP-3) identical to pyrophosphate-energized vacuolar membrane proton pump (pyrophosphate-energized inorganic pyrophosphatase) SP:P31414 from [Arabidopsis thaliana] | | | | | | |
|  |  | RAFL04-20-E04 | At3g14100 / oligouridylate binding protein (UBP1), putative | |  |  |  |  |  | | --- | --- | --- | --- | --- | |  |  |  |  |  | | RAFL04-20-E04 ,At3g14100  oligouridylate-binding protein, putative similar to GB:CAB75429 (GI:6996560) from [Nicotiana plumbaginifolia], contains Pfam profiles: PF00076 RNA recognition motif (3 copies) | | | | | | |
|  |  | RAFL11-02-L09 | At2g26510 / membrane transporter -related | |  |  |  |  |  | | --- | --- | --- | --- | --- | |  |  |  |  |  | | At2g26510 ,RAFL11-02-L09  xanthine/uracil permease family protein contains Pfam profile: PF00860 permease family | | | | | | |
|  |  | RAFL04-15-D01 | At3g09200 / 60S acidic ribosomal protein P0 (RPP0B) | |  |  |  |  |  | | --- | --- | --- | --- | --- | |  |  |  |  |  | | At3g09200 ,RAFL04-15-D01  60S acidic ribosomal protein P0 (RPP0B) similar to putative 60S acidic ribosomal protein P0 GB:P50346 [Glycine max] | | | | | | |
|  |  | RAFL07-10-I07 | At4g22690 / cytochrome P450 family | |  |  |  |  |  | | --- | --- | --- | --- | --- | |  |  |  |  |  | | At4g22690 ,RAFL07-10-I07  cytochrome P450 family protein flavonoid 3',5'-hydroxylase Hf1, Petunia x hybrida, PIR2:S38985 | | | | | | |
|  |  | RAFL08-11-M03 | At5g60390 / elongation factor 1-alpha (EF-1-alpha) | |  |  |  |  |  | | --- | --- | --- | --- | --- | |  |  |  |  |  | | RAFL08-11-M03 ,At5g60390  elongation factor 1-alpha / EF-1-alpha identical to SWISS-PROT:P13905 elongation factor 1-alpha (EF-1-alpha) [Arabidopsis thaliana] | | | | | | |
|  |  | RAFL09-12-B12 | At4g36130 / 60S ribosomal protein L8 (RPL8C) | |  |  |  |  |  | | --- | --- | --- | --- | --- | |  |  |  |  |  | | At4g36130 ,RAFL09-12-B12  60S ribosomal protein L8 (RPL8C) ribosomal protein L8, cytosolic, tomato, PIR1:R5TOL8 | | | | | | |
|  |  | RAFL09-09-M02 | At3g47520 / malate dehydrogenase (NAD), chloroplast, putative | |  |  |  |  |  | | --- | --- | --- | --- | --- | |  |  |  |  |  | | At3g47520 ,RAFL09-09-M02  malate dehydrogenase [NAD], chloroplast (MDH) identical to chloroplast NAD-malate dehydrogenase [Arabidopsis thaliana] GI:3256066; contains InterPro entry IPR001236: Lactate/malate dehydrogenase; contains Pfam profiles PF00056: lactate/malate dehydrogenase, NAD binding domain and PF02866: lactate/malate dehydrogenase, alpha/beta C-terminal domain | | | | | | |
|  |  | RAFL04-17-E12 | At1g71810 / expressed protein | |  |  |  |  |  | | --- | --- | --- | --- | --- | |  |  |  |  |  | | At1g71810 ,RAFL04-17-E12  ABC1 family protein contains Pfam domain, PF03109: ABC1 family | | | | | | |
|  |  | RAFL09-07-K24 | At2g34420 / photosystem II type I chlorophyll a /b binding protein | |  |  |  |  |  | | --- | --- | --- | --- | --- | |  |  |  |  |  | | At2g34420 ,RAFL09-07-K24  chlorophyll A-B binding protein / LHCII type I (LHB1B2) identical to GB:X64460 photosystem II type I chlorophyll a/b binding protein [Arabidopsis thaliana] GI:16364 | | | | | | |
|  |  | RAFL05-10-H23 | At1g21640 / expressed protein | |  |  |  |  |  | | --- | --- | --- | --- | --- | |  |  |  |  |  | | At1g21640 ,RAFL05-10-H23  ATP-NAD kinase family protein contains similarity to NAD kinase [Homo sapiens] gi|20070086|gb|AAM01195; contains Pfam domain, PF01513: ATP-NAD kinase | | | | | | |
|  |  | RAFL04-10-K01 | At2g43090 / aconitase family | |  |  |  |  |  | | --- | --- | --- | --- | --- | |  |  |  |  |  | | At2g43090 ,RAFL04-10-K01  aconitase C-terminal domain-containing protein contains Pfam profile PF00694: Aconitase C-terminal domain | | | | | | |
|  |  | RAFL11-07-A12 | At5g62300 / 40S ribosomal protein S20 (RPS20C) | |  |  |  |  |  | | --- | --- | --- | --- | --- | |  |  |  |  |  | | RAFL11-07-A12 ,At5g62300  40S ribosomal protein S20 (RPS20C) ribosomal protein S20, Arabidopsis thaliana, PIR:T12992 | | | | | | |
|  |  | RAFL04-15-O08 | At5g46430 / 60S ribosomal protein L32 (RPL32B) | |  |  |  |  |  | | --- | --- | --- | --- | --- | |  |  |  |  |  | | RAFL04-15-O08 ,At5g46430  60S ribosomal protein L32 (RPL32B) | | | | | | |
|  |  | RAFL08-10-G08 | At3g09630 / 60S ribosomal protein L4/L1 (RPL4A) | |  |  |  |  |  | | --- | --- | --- | --- | --- | |  |  |  |  |  | | RAFL08-10-G08 ,At3g09630  60S ribosomal protein L4/L1 (RPL4A) strong similarity to 60S ribosomal protein L1 GB:P49691 | | | | | | |
|  |  | RAFL04-16-M10 | At1g64550 / ABC transporter family protein | |  |  |  |  |  | | --- | --- | --- | --- | --- | |  |  |  |  |  | | RAFL04-16-M10 ,At1g64550  ABC transporter family protein similar to ABC transporter protein GB:AAF31030 GI:6899653 from [Leishmania major] | | | | | | |
|  |  | RAFL06-16-O18 | At5g48810 / cytochrome b5, putative | |  |  |  |  |  | | --- | --- | --- | --- | --- | |  |  |  |  |  | | At5g48810 ,RAFL06-16-O18  cytochrome b5 identical to cytochrome b5 [Arabidopsis thaliana] GI:4240122; strong similarity to Cytochrome B5 SP:P49098 from [Nicotiana tabacum] | | | | | | |
|  |  | RAFL09-10-P09 | At4g36130 / 60S ribosomal protein L8 (RPL8C) | |  |  |  |  |  | | --- | --- | --- | --- | --- | |  |  |  |  |  | | At4g36130 ,RAFL09-10-P09  60S ribosomal protein L8 (RPL8C) ribosomal protein L8, cytosolic, tomato, PIR1:R5TOL8 | | | | | | |
|  |  | RAFL02-10-H10 | At3g43980 / 40S ribosomal protein S29 (RPS29A) | |  |  |  |  |  | | --- | --- | --- | --- | --- | |  |  |  |  |  | | At3g43980 ,RAFL02-10-H10  40S ribosomal protein S29 (RPS29A) ribosomal protein S29, rat, PIR:S30298 | | | | | | |
|  |  | RAFL04-18-B19 | At3g24503 / aldehyde dehydrogenase (ALDH1a) | |  |  |  |  |  | | --- | --- | --- | --- | --- | |  |  |  |  |  | | At3g24503 ,RAFL04-18-B19  aldehyde dehydrogenase (ALDH1a) identical to aldehyde dehydrogenase ALDH1a [Arabidopsis thaliana] gi|20530143|gb|AAM27004 | | | | | | |
|  |  | RAFL05-07-F05 | At2g17360 / 40S ribosomal protein S4 (RPS4A) | |  |  |  |  |  | | --- | --- | --- | --- | --- | |  |  |  |  |  | | At2g17360 ,RAFL05-07-F05  40S ribosomal protein S4 (RPS4A) contains ribosomal protein S4 signature from residues 8 to 22 | | | | | | |
|  |  | RAFL03-01-G10 | At4g38500 / expressed protein | |  |  |  |  |  | | --- | --- | --- | --- | --- | |  |  |  |  |  | | At4g38500 ,RAFL03-01-G10  expressed protein contains Pfam profile: PF04765 protein of unknown function (DUF616) | | | | | | |
|  |  | RAFL09-07-B08 | At2g30970 / aspartate aminotransferase, mitochondrial (transaminase A/Asp1) | |  |  |  |  |  | | --- | --- | --- | --- | --- | |  |  |  |  |  | | At2g30970 ,RAFL09-07-B08  aspartate aminotransferase, mitochondrial / transaminase A (ASP1) identical to SP|P46643 Aspartate aminotransferase, mitochondrial precursor (EC 2.6.1.1) (Transaminase A) {Arabidopsis thaliana} | | | | | | |
|  |  | RAFL09-09-M09 | At5g14040 / mitochondrial phosphate transporter | |  |  |  |  |  | | --- | --- | --- | --- | --- | |  |  |  |  |  | | At5g14040 ,RAFL09-09-M09  mitochondrial phosphate transporter identical to mitochondrial phosphate transporter GI:3318617 from [Arabidopsis thaliana] | | | | | | |
|  |  | RAFL05-19-G10 | At3g24830 / 60S ribosomal protein L13A (RPL13aB) | |  |  |  |  |  | | --- | --- | --- | --- | --- | |  |  |  |  |  | | RAFL05-19-G10 ,At3g24830  60S ribosomal protein L13A (RPL13aB) similar to 60S RIBOSOMAL PROTEIN L13A GB:P35427 from [Rattus norvegicus] | | | | | | |
|  |  | RAFL06-07-B02 | At3g11940 / 40S ribosomal protein S5 (RPS5B) | |  |  |  |  |  | | --- | --- | --- | --- | --- | |  |  |  |  |  | | At3g11940 ,RAFL06-07-B02  40S ribosomal protein S5 (RPS5B) similar to 40S ribosomal protein S5 GB:AAC98068 GI:4056502 from [Arabidopsis thaliana] | | | | | | |
|  |  | RAFL11-11-E06 | At1g72370 / 40S ribosomal protein SA (RPSaA) | |  |  |  |  |  | | --- | --- | --- | --- | --- | |  |  |  |  |  | | RAFL11-11-E06 ,At1g72370  40S ribosomal protein SA (RPSaA) identical to laminin receptor-like protein GB:U01955 [Arabidopsis thaliana]; identical to cDNA laminin receptor homologue GI:16379 | | | | | | |
|  |  | RAFL07-16-H23 | At3g16780 / 60S ribosomal protein L19 (RPL19B) | |  |  |  |  |  | | --- | --- | --- | --- | --- | |  |  |  |  |  | | At3g16780 ,RAFL07-16-H23  60S ribosomal protein L19 (RPL19B) similar to ribosomal protein L19 GB:CAA45090 from [Homo sapiens] | | | | | | |
|  |  | RAFL07-12-L01 | At4g34870 / peptidylprolyl isomerase (cyclophilin) | |  |  |  |  |  | | --- | --- | --- | --- | --- | |  |  |  |  |  | | At4g34870 ,RAFL07-12-L01  peptidyl-prolyl cis-trans isomerase / cyclophilin (CYP1) / rotamase identical to cyclophilin (CYP1) gi|992643|gb|AAA75512; similar to peptidyl-prolyl cis-trans isomerase, PPIase (cyclophilin, cyclosporin A-binding protein) [Catharanthus roseus] SWISS-PROT:Q39613 | | | | | | |
|  |  | RAFL09-18-A11 | At5g19760 / mitochondrial 2-oxoglutarate/malate translocator, putative | |  |  |  |  |  | | --- | --- | --- | --- | --- | |  |  |  |  |  | | RAFL09-18-A11 ,At5g19760  dicarboxylate/tricarboxylate carrier (DTC) identical to dicarboxylate/tricarboxylate carrier [Arabidopsis thaliana] GI:19913113 | | | | | | |
|  |  | RAFL05-07-A02 | At5g47930 / ribosomal protein S27 | |  |  |  |  |  | | --- | --- | --- | --- | --- | |  |  |  |  |  | | RAFL05-07-A02 ,At5g47930  40S ribosomal protein S27 (RPS27D) | | | | | | |
|  |  | RAFL04-15-L14 | At3g06350 / dehydroquinate dehydratase/shikimate dehydrogenase, putative | |  |  |  |  |  | | --- | --- | --- | --- | --- | |  |  |  |  |  | | RAFL04-15-L14 ,At3g06350  dehydroquinate dehydratase, putative / shikimate dehydrogenase, putative similar to dehydroquinate dehydratase/shikimate dehydrogenase [Nicotiana tabacum][GI:535771], dehydroquinate dehydratase/shikimate:NADP oxidoreductase [Lycopersicon esculentum][GI:3169883] | | | | | | |
|  |  | RAFL05-08-M23 | At1g03860 / prohibitin 2 -related | |  |  |  |  |  | | --- | --- | --- | --- | --- | |  |  |  |  |  | | RAFL05-08-M23 ,At1g03860  prohibitin, putative similar to SP|P24142 Prohibitin (B-cell receptor associated protein 32) (BAP 32) {Rattus norvegicus}; contains Pfam profile PF01145: SPFH domain / Band 7 family | | | | | | |
|  |  | RAFL05-16-K13 | At2g47320 / peptidyl-prolyl cis-trans isomerase -related | |  |  |  |  |  | | --- | --- | --- | --- | --- | |  |  |  |  |  | | At2g47320 ,RAFL05-16-K13  peptidyl-prolyl cis-trans isomerase cyclophilin-type family protein contains Pfam domain, PF00160: peptidyl-prolyl cis-trans isomerase, cyclophilin-type | | | | | | |
|  |  | RAFL04-14-I21 | At1g78580 / alpha,alpha-trehalose-phosphate synthase, UDP-forming (trehalose-6-phosphate synthase/UDP-glucose-glucosephosphate glucosyltransferase), putative | |  |  |  |  |  | | --- | --- | --- | --- | --- | |  |  |  |  |  | | At1g78580 ,RAFL04-14-I21  alpha, alpha-trehalose-phosphate synthase, UDP-forming, putative / trehalose-6-phosphate synthase, putative / UDP-glucose-glucosephosphate glucosyltransferase, putative similar to trehalose-6-phosphate synthase SL-TPS/P [Selaginella lepidophylla] GI:4100325; contains Pfam profiles PF00982: Glycosyltransferase family 20, PF02358: Trehalose-phosphatase | | | | | | |
|  |  | RAFL05-17-A01 | At3g52300 / expressed protein | |  |  |  |  |  | | --- | --- | --- | --- | --- | |  |  |  |  |  | | RAFL05-17-A01 ,At3g52300  ATP synthase D chain-related contains weak similarity to ATP synthase D chain, mitochondrial (EC 3.6.3.14) (Swiss-Prot:P31399) [Rattus norvegicus] | | | | | | |
|  |  | RAFL08-15-A10 | At5g09510 / 40S ribosomal protein S15 (RPS15D) | |  |  |  |  |  | | --- | --- | --- | --- | --- | |  |  |  |  |  | | RAFL08-15-A10 ,At5g09510  40S ribosomal protein S15 (RPS15D) ribosomal protein S15 - Arabidopsis thaliana, EMBL:Z23161 | | | | | | |
|  |  | RAFL05-10-L03 | At5g20500 / glutaredoxin, putative | |  |  |  |  |  | | --- | --- | --- | --- | --- | |  |  |  |  |  | | RAFL05-10-L03 ,At5g20500  glutaredoxin, putative similar to glutaredoxin [Populus tremula x Populus tremuloides] gi|19548658|gb|AAL90750 | | | | | | |
|  |  | RAFL05-14-G01 | At4g02230 / 60S ribosomal protein L19 (RPL19C) | |  |  |  |  |  | | --- | --- | --- | --- | --- | |  |  |  |  |  | | RAFL05-14-G01 ,At4g02230  60S ribosomal protein L19 (RPL19C) similar to L19 from several species | | | | | | |
|  |  | RAFL05-09-F24 | At3g46560 / small zinc finger-related protein TIM9 | |  |  |  |  |  | | --- | --- | --- | --- | --- | |  |  |  |  |  | | RAFL05-09-F24 ,At3g46560  mitochondrial import inner membrane translocase (TIM9) identical to mitochondrial import inner membrane translocase subunit Tim9 [Arabidopsis thaliana] Swiss-Prot:Q9XGX9; contains Pfam domain, PF02953: Tim10/DDP family zinc finger | | | | | | |
|  |  | RAFL07-17-D23 | At1g78870 / ubiquitin-conjugating enzyme, putative | |  |  |  |  |  | | --- | --- | --- | --- | --- | |  |  |  |  |  | | At1g78870 ,RAFL07-17-D23  ubiquitin-conjugating enzyme, putative nearly identical to ubiquitin-conjugating enzyme E2 [Catharanthus roseus] GI:5381319; contains Pfam profile PF00179: Ubiquitin-conjugating enzyme | | | | | | |
|  |  | RAFL03-05-E07 | At1g07920 / elongation factor 1-alpha (EF-1-alpha) | |  |  |  |  |  | | --- | --- | --- | --- | --- | |  |  |  |  |  | | RAFL03-05-E07 ,At1g07920  elongation factor 1-alpha / EF-1-alpha identical to GB:CAA34456 from [Arabidopsis thaliana] (Plant Mol. Biol. 14 (1), 107-110 (1990)) | | | | | | |
|  |  | RAFL05-21-A22 | At3g20670 / histone H2A, putative | |  |  |  |  |  | | --- | --- | --- | --- | --- | |  |  |  |  |  | | At3g20670 ,RAFL05-21-A22  histone H2A, putative strong similarity to histone H2A GB:AAF64418 GI:7595337 from Arabidopsis thaliana, Triticum aestivum GI:536892; contains Pfam profile PF00125 Core histone H2A/H2B/H3/H4 | | | | | | |
|  |  | RAFL06-12-J07 | At3g53020 / 60S ribosomal protein L24 (RPL24B) | |  |  |  |  |  | | --- | --- | --- | --- | --- | |  |  |  |  |  | | At3g53020 ,RAFL06-12-J07  60S ribosomal protein L24 (RPL24B) 60S ribosomal protein L24, Arabidopsis thaliana, EMBL:AC006282 | | | | | | |
|  |  | RAFL06-16-N16 | At4g16720 / 60S ribosomal protein L15 (RPL15A) | |  |  |  |  |  | | --- | --- | --- | --- | --- | |  |  |  |  |  | | RAFL06-16-N16 ,At4g16720  60S ribosomal protein L15 (RPL15A) | | | | | | |
|  |  | RAFL11-05-A06 | At5g64500 / expressed protein | |  |  |  |  |  | | --- | --- | --- | --- | --- | |  |  |  |  |  | | RAFL11-05-A06 ,At5g64500  membrane protein-related contains weak similarity to spinster type IV (GI:12003976) [Drosophila melanogaster] | | | | | | |
|  |  | RAFL04-17-H14 | At3g06700 / 60S ribosomal protein L29 (RPL29A) | |  |  |  |  |  | | --- | --- | --- | --- | --- | |  |  |  |  |  | | RAFL04-17-H14 ,At3g06700  60S ribosomal protein L29 (RPL29A) similar to ribosomal protein L29 GI:7959366 [Panax ginseng] | | | | | | |
|  |  | RAFL04-09-E11 | At5g08180 / ribosomal protein L7Ae family | |  |  |  |  |  | | --- | --- | --- | --- | --- | |  |  |  |  |  | | At5g08180 ,RAFL04-09-E11  ribosomal protein L7Ae/L30e/S12e/Gadd45 family protein Similar to NHP2/L7Ae family proteins, see SWISSPROT:P32495 and PMID:2063628. | | | | | | |
|  |  | RAFL05-09-M22 | At3g49910 / 60S ribosomal protein L26 (RPL26A) | |  |  |  |  |  | | --- | --- | --- | --- | --- | |  |  |  |  |  | | RAFL05-09-M22 ,At3g49910  60S ribosomal protein L26 (RPL26A) 60S RIBOSOMAL PROTEIN L26, Brassica rapa, EMBL:BRD495 | | | | | | |
|  |  | RAFL06-09-O23 | At3g15000 / expressed protein | |  |  |  |  |  | | --- | --- | --- | --- | --- | |  |  |  |  |  | | RAFL06-09-O23 ,At3g15000  expressed protein similar to DAG protein (required for chloroplast differentiation and palisade development) GB:Q38732 [Antirrhinum majus] | | | | | | |
|  |  | RAFL04-15-O15 | At1g30230 / elongation factor 1-beta -related | |  |  |  |  |  | | --- | --- | --- | --- | --- | |  |  |  |  |  | | RAFL04-15-O15 ,At1g30230  elongation factor 1-beta / EF-1-beta identical to SP|P48006 Elongation factor 1-beta (EF-1-beta) {Arabidopsis thaliana} | | | | | | |
|  |  | RAFL04-13-N15 | At5g48810 / cytochrome b5, putative | |  |  |  |  |  | | --- | --- | --- | --- | --- | |  |  |  |  |  | | At5g48810 ,RAFL04-13-N15  cytochrome b5 identical to cytochrome b5 [Arabidopsis thaliana] GI:4240122; strong similarity to Cytochrome B5 SP:P49098 from [Nicotiana tabacum] | | | | | | |
|  |  | RAFL04-17-E10 | At1g12770 / pentatricopeptide (PPR) repeat-containing protein | |  |  |  |  |  | | --- | --- | --- | --- | --- | |  |  |  |  |  | | At1g12770 ,RAFL04-17-E10  DEAD/DEAH box helicase family protein / pentatricopeptide (PPR) repeat-containing protein contains Pfam profiles: PF00271 helicase conserved C-terminal domain, PF01535 PPR repeat, PF00270: DEAD/DEAH box helicase | | | | | | |
|  |  | RAFL05-13-K07 | At4g09800 / 40S ribosomal protein S18 (RPS18C) | |  |  |  |  |  | | --- | --- | --- | --- | --- | |  |  |  |  |  | | RAFL05-13-K07 ,At4g09800  40S ribosomal protein S18 (RPS18C) | | | | | | |
|  |  | RAFL05-16-O24 | At4g35250 / vestitone reductase-related | |  |  |  |  |  | | --- | --- | --- | --- | --- | |  |  |  |  |  | | RAFL05-16-O24 ,At4g35250  vestitone reductase-related low similarity to vestitone reductase [Medicago sativa subsp. sativa] GI:973249 | | | | | | |
|  |  | RAFL07-11-P20 | At4g01480 / inorganic phosphatase -related | |  |  |  |  |  | | --- | --- | --- | --- | --- | |  |  |  |  |  | | RAFL07-11-P20 ,At4g01480  inorganic pyrophosphatase, putative [soluble] / pyrophosphate phospho-hydrolase, putative / PPase, putative strong similarity to SP|Q43187 Soluble inorganic pyrophosphatase (EC 3.6.1.1) (Pyrophosphate phospho- hydrolase) (PPase) {Solanum tuberosum}; contains Pfam profile PF00719: inorganic pyrophosphatase | | | | | | |
|  |  | RAFL04-14-I15 | At3g02080 / 40S ribosomal protein S19 (RPS19A) | |  |  |  |  |  | | --- | --- | --- | --- | --- | |  |  |  |  |  | | RAFL04-14-I15 ,At3g02080  40S ribosomal protein S19 (RPS19A) similar to 40S ribosomal protein S19 GB:P40978 [Oryza sativa] | | | | | | |
|  |  | RAFL06-14-K01 | At3g04120 / glyceraldehyde-3-phosphate dehydrogenase C subunit (GapC) | |  |  |  |  |  | | --- | --- | --- | --- | --- | |  |  |  |  |  | | RAFL06-14-K01 ,At3g04120  glyceraldehyde-3-phosphate dehydrogenase, cytosolic (GAPC) / NAD-dependent glyceraldehyde-3-phosphate dehydrogenase identical to SP|P25858 Glyceraldehyde 3-phosphate dehydrogenase, cytosolic (EC 1.2.1.12) {Arabidopsis thaliana} | | | | | | |
|  |  | RAFL11-01-C20 | At3g06590 / bHLH protein | |  |  |  |  |  | | --- | --- | --- | --- | --- | |  |  |  |  |  | | RAFL11-01-C20 ,At3g06590  expressed protein | | | | | | |
|  |  | RAFL07-16-A17 | At5g64140 / 40S ribosomal protein S28 (RPS28C) | |  |  |  |  |  | | --- | --- | --- | --- | --- | |  |  |  |  |  | | At5g64140 ,RAFL07-16-A17  40S ribosomal protein S28 (RPS28C) | | | | | | |
|  |  | RAFL07-10-D02 | At1g14320 / 60S ribosomal protein L10 (RPL10A)/Wilm's tumor suppressor protein-related | |  |  |  |  |  | | --- | --- | --- | --- | --- | |  |  |  |  |  | | At1g14320 ,RAFL07-10-D02  60S ribosomal protein L10 (RPL10A) / Wilm's tumor suppressor protein-related similar to tumor suppressor GI:575354 from [Oryza sativa] | | | | | | |
|  |  | RAFL08-13-M06 | At3g11250 / 60S acidic ribosomal protein P0 (RPP0C) | |  |  |  |  |  | | --- | --- | --- | --- | --- | |  |  |  |  |  | | At3g11250 ,RAFL08-13-M06  60S acidic ribosomal protein P0 (RPP0C) similar to 60S acidic ribosomal protein P0 GI:2088654 [Arabidopsis thaliana] | | | | | | |
|  |  | RAFL05-15-O22 | At2g20420 / succinyl-CoA ligase beta subunit | |  |  |  |  |  | | --- | --- | --- | --- | --- | |  |  |  |  |  | | RAFL05-15-O22 ,At2g20420  succinyl-CoA ligase [GDP-forming] beta-chain, mitochondrial, putative / succinyl-CoA synthetase, beta chain, putative / SCS-beta, putative identical to SP|O82662 Succinyl-CoA ligase [GDP-forming] beta-chain, mitochondrial precursor (EC 6.2.1.4) (Succinyl-CoA synthetase, beta chain) (SCS- beta) {Arabidopsis thaliana}; similar to SP|O97580 Succinyl-CoA ligase [ADP-forming] beta-chain, mitochondrial precursor (EC 6.2.1.5) {Sus scrofa}; contains Pfam profiles PF00549: CoA-ligase, PF02222: ATP-grasp domain | | | | | | |
|  |  | RAFL04-17-F02 | At2g01140 / fructose-bisphosphate aldolase, putative | |  |  |  |  |  | | --- | --- | --- | --- | --- | |  |  |  |  |  | | RAFL04-17-F02 ,At2g01140  fructose-bisphosphate aldolase, putative similar to plastidic aldolase NPALDP1 from Nicotiana paniculata [GI:4827251]; contains Pfam profile PF00274 Fructose-bisphosphate aldolase class-I | | | | | | |
|  |  | RAFL06-08-M13 | At1g07920 / elongation factor 1-alpha (EF-1-alpha) | |  |  |  |  |  | | --- | --- | --- | --- | --- | |  |  |  |  |  | | RAFL06-08-M13 ,At1g07920  elongation factor 1-alpha / EF-1-alpha identical to GB:CAA34456 from [Arabidopsis thaliana] (Plant Mol. Biol. 14 (1), 107-110 (1990)) | | | | | | |
|  |  | RAFL07-10-F16 | At3g13930 / acetyltransferase -related | |  |  |  |  |  | | --- | --- | --- | --- | --- | |  |  |  |  |  | | At3g13930 ,RAFL07-10-F16  dihydrolipoamide S-acetyltransferase, putative similar to dihydrolipoamide S-acetyltransferase [Zea mays] GI:5669871; contains Pfam profiles PF00198: 2-oxo acid dehydrogenases acyltransferase (catalytic domain), PF00364: Biotin-requiring enzyme, PF02817: e3 binding domain | | | | | | |
|  |  | RAFL06-08-I18 | At2g34420 / photosystem II type I chlorophyll a /b binding protein | |  |  |  |  |  | | --- | --- | --- | --- | --- | |  |  |  |  |  | | At2g34420 ,RAFL06-08-I18  chlorophyll A-B binding protein / LHCII type I (LHB1B2) identical to GB:X64460 photosystem II type I chlorophyll a/b binding protein [Arabidopsis thaliana] GI:16364 | | | | | | |
|  |  | RAFL05-21-M03 | At1g32500 / expressed protein | |  |  |  |  |  | | --- | --- | --- | --- | --- | |  |  |  |  |  | | RAFL05-21-M03 ,At1g32500  ATP-binding-cassette transporter, putative similar to ATP-binding-cassette transporter (ABC1) described in PMID:11156608 | | | | | | |
|  |  | RAFL07-10-I06 | At5g08670 / H+-transporting ATP synthase beta chain (mitochondrial) -related | |  |  |  |  |  | | --- | --- | --- | --- | --- | |  |  |  |  |  | | At5g08670 ,RAFL07-10-I06  ATP synthase beta chain 1, mitochondrial identical to SP|P83483 ATP synthase beta chain 1, mitochondrial precursor (EC 3.6.3.14) {Arabidopsis thaliana}; strong similarity to SP|P17614 ATP synthase beta chain, mitochondrial precursor (EC 3.6.3.14) {Nicotiana plumbaginifolia}; contains Pfam profiles PF00006: ATP synthase alpha/beta family nucleotide-binding domain, PF00306: ATP synthase ab C terminal, PF02874: ATP synthase alpha/beta family beta-barrel domain; supporting cDNA gi|26452102|dbj|AK118538.1| | | | | | | |
|  |  | RAFL05-18-H11 | At5g27700 / ribosomal protein S21 - like | |  |  |  |  |  | | --- | --- | --- | --- | --- | |  |  |  |  |  | | RAFL05-18-H11 ,At5g27700  40S ribosomal protein S21 (RPS21C) ribosomal protein S21, Zea mays, PIR:T03945 | | | | | | |
|  |  | RAFL07-17-M20 | At1g67840 / ATP-binding region, ATPase-like domain-containing protein | |  |  |  |  |  | | --- | --- | --- | --- | --- | |  |  |  |  |  | | At1g67840 ,RAFL07-17-M20  ATP-binding region, ATPase-like domain-containing protein contains Pfam profile PF02518: ATPase, histidine kinase-, DNA gyrase B-, and HSP90-like domain protein; similar to ESTs gb|AI995396.1, dbj|AV557393.1, and dbj|AV557055.1 | | | | | | |
|  |  | RAFL11-02-J15 | At4g00100 / 40S ribosomal protein S13 (RPS13B) | |  |  |  |  |  | | --- | --- | --- | --- | --- | |  |  |  |  |  | | At4g00100 ,RAFL11-02-J15  40S ribosomal protein S13 (RPS13A) similar to ribosomal protein S13; PF00312 (View Sanger Pfam): ribosomal protein S15; identical to cDNA AtRPS13A mRNA for cytoplasmic ribosomal protein S13 GI:6521011 | | | | | | |
|  |  | RAFL07-18-D08 | At1g15690 / inorganic pyrophosphatase -related | |  |  |  |  |  | | --- | --- | --- | --- | --- | |  |  |  |  |  | | At1g15690 ,RAFL07-18-D08  pyrophosphate-energized vacuolar membrane proton pump / pyrophosphate-energized inorganic pyrophosphatase (AVP-3) identical to pyrophosphate-energized vacuolar membrane proton pump (pyrophosphate-energized inorganic pyrophosphatase) SP:P31414 from [Arabidopsis thaliana] | | | | | | |
|  |  | RAFL07-18-J05 | At3g09210 / expressed protein | |  |  |  |  |  | | --- | --- | --- | --- | --- | |  |  |  |  |  | | At3g09210 ,RAFL07-18-J05  KOW domain-containing transcription factor family protein ; est match | | | | | | |
|  |  | RAFL04-20-J09 | At4g25130 / protein-methionine-S-oxide reductase | |  |  |  |  |  | | --- | --- | --- | --- | --- | |  |  |  |  |  | | At4g25130 ,RAFL04-20-J09  peptide methionine sulfoxide reductase, putative strong similarity to SP|P54151 Peptide methionine sulfoxide reductase (EC 1.8.4.6) {Brassica napus}; contains Pfam profile PF01625: Peptide methionine sulfoxide reductase | | | | | | |
|  |  | RAFL09-18-O15 | At2g17360 / 40S ribosomal protein S4 (RPS4A) | |  |  |  |  |  | | --- | --- | --- | --- | --- | |  |  |  |  |  | | RAFL09-18-O15 ,At2g17360  40S ribosomal protein S4 (RPS4A) contains ribosomal protein S4 signature from residues 8 to 22 | | | | | | |
|  |  | RAFL06-07-A07 | At5g53140 / protein phosphatase 2C (PP2C), putative | |  |  |  |  |  | | --- | --- | --- | --- | --- | |  |  |  |  |  | | RAFL06-07-A07 ,At5g53140  protein phosphatase 2C, putative / PP2C, putative | | | | | | |
|  |  | RAFL07-08-M19 | At1g09620 / leucyl-tRNA synthetase -related | |  |  |  |  |  | | --- | --- | --- | --- | --- | |  |  |  |  |  | | At1g09620 ,RAFL07-08-M19  tRNA synthetase class I (I, L, M and V) family protein similar to cytosolic leucyl-tRNA synthetase [Candida albicans] GI:9858190; contains Pfam profile PF00133: tRNA synthetases class I (I, L, M and V) | | | | | | |
|  |  | RAFL04-16-A18 | At1g51650 / epsilon subunit of mitochondrial F1-ATPase | |  |  |  |  |  | | --- | --- | --- | --- | --- | |  |  |  |  |  | | RAFL04-16-A18 ,At1g51650  ATP synthase epsilon chain, mitochondrial identical to ATP synthase epsilon chain, mitochondrial SP:Q96253 from [Arabidopsis thaliana] | | | | | | |
|  |  | RAFL05-07-M06 | At3g20290 / calcium-binding EF-hand family protein | |  |  |  |  |  | | --- | --- | --- | --- | --- | |  |  |  |  |  | | RAFL05-07-M06 ,At3g20290  calcium-binding EF hand family protein similar to EH-domain containing protein 1 from {Mus musculus} SP|Q9WVK4 and {Homo sapiens} SP|Q9H4M9, receptor-mediated endocytosis 1 from [Caenorhabditis elegans] GI:13487775, GI:13487777, GI:13487779; contains INTERPRO:IPR002048 calcium-binding EF-hand domain | | | | | | |
|  |  | RAFL07-07-L08 | At1g33080 / MATE efflux protein family | |  |  |  |  |  | | --- | --- | --- | --- | --- | |  |  |  |  |  | | RAFL07-07-L08 ,At1g33080  MATE efflux family protein similar to ripening regulated protein DDTFR18 [Lycopersicon esculentum] GI:12231296; contains Pfam profile PF01554: Uncharacterized membrane protein family | | | | | | |
|  |  | RAFL07-14-B18 | At2g47510 / fumarase -related | |  |  |  |  |  | | --- | --- | --- | --- | --- | |  |  |  |  |  | | At2g47510 ,RAFL07-14-B18  fumarate hydratase, putative / fumarase, putative similar to SP|P55250 Fumarate hydratase, mitochondrial precursor (EC 4.2.1.2) (Fumarase) {Rhizopus oryzae}; contains Pfam profile PF00206: Lyase | | | | | | |
|  |  | RAFL05-02-J02 | At1g08490 / nitrogen fixation protein. putative (NifS) | |  |  |  |  |  | | --- | --- | --- | --- | --- | |  |  |  |  |  | | RAFL05-02-J02 ,At1g08490  cysteine desulfurase, putative similar to nitrogen fixation protein (nifS) GB:D64004 GI:1001701 from [Synechocystis sp]; contains TIGRFAM TIGR01364: phosphoserine aminotransferase; contains Pfam PF00266: aminotransferase, class V | | | | | | |
|  |  | RAFL11-04-A02 | At1g07770 / 40S ribosomal protein S15A (RPS15aA) | |  |  |  |  |  | | --- | --- | --- | --- | --- | |  |  |  |  |  | | At1g07770 ,RAFL11-04-A02  40S ribosomal protein S15A (RPS15aA) identical to GB:AAA61608 from [Arabidopsis thaliana] (Plant Physiol. 106 (1), 401-402 (1994)) | | | | | | |
|  |  | RAFL09-09-O07 | At4g16720 / 60S ribosomal protein L15 (RPL15A) | |  |  |  |  |  | | --- | --- | --- | --- | --- | |  |  |  |  |  | | RAFL09-09-O07 ,At4g16720  60S ribosomal protein L15 (RPL15A) | | | | | | |
|  |  | RAFL04-09-F23 | At2g31610 / 40S ribosomal protein S3 (RPS3A) | |  |  |  |  |  | | --- | --- | --- | --- | --- | |  |  |  |  |  | | RAFL04-09-F23 ,At2g31610  40S ribosomal protein S3 (RPS3A) | | | | | | |
|  |  | RAFL04-18-P18 | At2g33040 / mitochondrial F1-ATPase, gamma subunit (ATP3\_ARATH) | |  |  |  |  |  | | --- | --- | --- | --- | --- | |  |  |  |  |  | | At2g33040 ,RAFL04-18-P18  ATP synthase gamma chain, mitochondrial (ATPC) identical to SP|Q96250 ATP synthase gamma chain, mitochondrial precursor (EC 3.6.3.14) {Arabidopsis thaliana}; contains Pfam profile: PF00231 ATP synthase | | | | | | |
|  |  | RAFL09-13-H18 | At1g43170 / 60S ribosomal protein L3 (RPL3A) | |  |  |  |  |  | | --- | --- | --- | --- | --- | |  |  |  |  |  | | At1g43170 ,RAFL09-13-H18  60S ribosomal protein L3 (RPL3A) identical to ribosomal protein GI:166858 from [Arabidopsis thaliana] | | | | | | |
|  |  | RAFL04-20-I07 | At3g23670 / phragmoplast-associated kinesin-related protein, putative | |  |  |  |  |  | | --- | --- | --- | --- | --- | |  |  |  |  |  | | At3g23670 ,RAFL04-20-I07  phragmoplast-associated kinesin-related protein, putative similar to kinesin like protein GB:CAB10194 from [Arabidopsis thaliana] | | | | | | |
|  |  | RAFL08-09-E20 | At2g41840 / 40S ribosomal protein S2 (RPS2C) | |  |  |  |  |  | | --- | --- | --- | --- | --- | |  |  |  |  |  | | RAFL08-09-E20 ,At2g41840  40S ribosomal protein S2 (RPS2C) | | | | | | |
|  |  | RAFL09-07-I03 | At5g60390 / elongation factor 1-alpha (EF-1-alpha) | |  |  |  |  |  | | --- | --- | --- | --- | --- | |  |  |  |  |  | | At5g60390 ,RAFL09-07-I03  elongation factor 1-alpha / EF-1-alpha identical to SWISS-PROT:P13905 elongation factor 1-alpha (EF-1-alpha) [Arabidopsis thaliana] | | | | | | |
|  |  | RAFL05-16-I03 | At3g50440 / hydrolase, alpha/beta fold family | |  |  |  |  |  | | --- | --- | --- | --- | --- | |  |  |  |  |  | | RAFL05-16-I03 ,At3g50440  hydrolase, alpha/beta fold family protein similar to ethylene-induced esterase [Citrus sinensis] GI:14279437, polyneuridine aldehyde esterase [Rauvolfia serpentina] GI:6651393; contains Pfam profile PF00561: hydrolase, alpha/beta fold family | | | | | | |
[truncated: 4,128,205 more chars]
