# Supplementary material for: A flexible representation of omic knowledge for thorough analysis of microarray data
Source: Plant Methods. 2006 Mar 2;2:5. doi: 10.1186/1746-4811-2-5 (PMC1421397; doi:10.1186/1746-4811-2-5)
Supplement: Additional File 4 — Supplementary Table 4. Ranking result of significant correlations between the "functional Class" of the Gene position on genome and the clusters formed by BL-SOM of the microarray probes of expression profile under drought conditions [file 1746-4811-2-5-S4.HTML]

|  |  |  |  |  |  |  |  |  |  |  |  |
| --- | --- | --- | --- | --- | --- | --- | --- | --- | --- | --- | --- |
| Date: | | 2005/06/24 | | | | | | | | | |
| Method: | | Fisher test | | | | | | | | | |
| Cut off P-value: | | 0.05 | | | | | | | | | |
| Target dataset(s): | | Chromosome:5  Chromosome:3  Chromosome:4  Chromosome:1  Chromosome:2 | | | | | | | | | |
| Query dataset(s): | | SOM Cluster | | | | | | | | | |
  | | | | | | | | | | | || 3\_20040001\_20070000 | | |  |  | A | B | C | D | P | P' | N |
|  | Cluster:5-2 | |  |  | 1 | 125 | 0 | 4537 | 0.027021231 | 0.027021231 | 1 |
|  |  | RAFL08-12-E04 | At3g54110 / uncoupling protein (ucp/PUMP) | |  |  |  |  |  | | --- | --- | --- | --- | --- | |  |  |  |  |  | | At3g54110 ,RAFL08-12-E04  plant uncoupling mitochondrial protein (PUMP) identical to plant uncoupling mitochondrial protein [Arabidopsis thaliana] GI:3115108 | | | | | | |
| 5\_26340001\_26370000 | | |  |  | A | B | C | D | P | P' | N |
|  | Cluster:9-1 | |  |  | 1 | 95 | 1 | 4566 | 0.040755685 | 0.08151137 | 2 |
|  |  | RAFL09-11-J12 | At5g66760 / succinate dehydrogenase (ubiquinone), putative | |  |  |  |  |  | | --- | --- | --- | --- | --- | |  |  |  |  |  | | RAFL09-11-J12 ,At5g66760  succinate dehydrogenase [ubiquinone] flavoprotein subunit, mitochondrial / flavoprotein subunit of complex II identical to SP|O82663 Succinate dehydrogenase [ubiquinone] flavoprotein subunit, mitochondrial (EC 1.3.5.1) (FP) (Flavoprotein subunit of complex II) {Arabidopsis thaliana} | | | | | | |
| 3\_2460001\_2490000 | | |  |  | A | B | C | D | P | P' | N |
|  | Cluster:6-2 | |  |  | 2 | 174 | 1 | 4486 | 0.0041446616 | 0.008289323 | 2 |
|  |  | RAFL04-16-E03 | At3g07790 / expressed protein | |  |  |  |  |  | | --- | --- | --- | --- | --- | |  |  |  |  |  | | RAFL04-16-E03 ,At3g07790  DGCR14-related similar to DGCR14 protein (DiGeorge syndrome critical region 14) (ES2 protein) (Swiss-Prot:Q96DF8) [Homo sapiens] | | | | | | |
|  |  | RAFL08-16-C13 | At3g07780 / expressed protein | |  |  |  |  |  | | --- | --- | --- | --- | --- | |  |  |  |  |  | | RAFL08-16-C13 ,At3g07780  expressed protein | | | | | | |
| 5\_5520001\_5550000 | | |  |  | A | B | C | D | P | P' | N |
|  | Cluster:8-0 | |  |  | 1 | 108 | 0 | 4554 | 0.02337551 | 0.02337551 | 1 |
|  |  | RAFL06-12-I17 | At5g16840 / RRM-containing protein | |  |  |  |  |  | | --- | --- | --- | --- | --- | |  |  |  |  |  | | At5g16840 ,RAFL06-12-I17  RNA recognition motif (RRM)-containing protein predicted proteins - Arabidopsis thaliana | | | | | | |
| 2\_12360001\_12390000 | | |  |  | A | B | C | D | P | P' | N |
|  | Cluster:7-0 | |  |  | 2 | 245 | 0 | 4416 | 0.002795081 | 0.002795081 | 1 |
|  |  | RAFL07-14-N08 | At2g28910 / expressed protein | |  |  |  |  |  | | --- | --- | --- | --- | --- | |  |  |  |  |  | | At2g28910 ,RAFL07-14-N08  CAX-interacting protein 4 (CAXIP4) contains Pfam domain PF00098: Zinc knuckle; identical to cDNA CAX-interacting protein 4 GI:27651998 | | | | | | |
|  |  | RAFL08-16-B05 | At2g28910 / expressed protein | |  |  |  |  |  | | --- | --- | --- | --- | --- | |  |  |  |  |  | | At2g28910 ,RAFL08-16-B05  CAX-interacting protein 4 (CAXIP4) contains Pfam domain PF00098: Zinc knuckle; identical to cDNA CAX-interacting protein 4 GI:27651998 | | | | | | |
| 5\_26220001\_26250000 | | |  |  | A | B | C | D | P | P' | N |
|  | Cluster:9-0 | |  |  | 1 | 31 | 2 | 4629 | 0.020451 | 0.061353 | 3 |
|  |  | RAFL05-03-I09 | At5g66400 / dehydrin RAB18-related protein (sp P30185) | |  |  |  |  |  | | --- | --- | --- | --- | --- | |  |  |  |  |  | | RAFL05-03-I09 ,At5g66400  dehydrin (RAB18) nearly identical to SP|P30185 Dehydrin Rab18 {Arabidopsis thaliana} | | | | | | |
| 4\_13830001\_13860000 | | |  |  | A | B | C | D | P | P' | N |
|  | Cluster:9-1 | |  |  | 1 | 95 | 1 | 4566 | 0.040755685 | 0.08151137 | 2 |
|  |  | RAFL05-12-N20 | At4g30470 / cinnamoyl-CoA reductase-related | |  |  |  |  |  | | --- | --- | --- | --- | --- | |  |  |  |  |  | | RAFL05-12-N20 ,At4g30470  cinnamoyl-CoA reductase-related similar to cinnamoyl-CoA reductase from Pinus taeda [GI:17978649], Saccharum officinarum [GI:3341511] | | | | | | |
| 4\_900001\_1200000 | | |  |  | A | B | C | D | P | P' | N |
|  | Cluster:9-0 | |  |  | 2 | 30 | 15 | 4616 | 0.005819355 | 0.075651616 | 13 |
|  |  | RAFL08-10-G22 | At4g02280 / sucrose synthase (UDP-glucose-fructose glucosyltransferase/sucrose-UDP glucosyltransferase), putative | |  |  |  |  |  | | --- | --- | --- | --- | --- | |  |  |  |  |  | | RAFL08-10-G22 ,At4g02280  sucrose synthase, putative / sucrose-UDP glucosyltransferase, putative strong similarity to sucrose synthase GI:6682841 from [Citrus unshiu] | | | | | | |
|  |  | RAFL05-18-M07 | At4g02280 / sucrose synthase (UDP-glucose-fructose glucosyltransferase/sucrose-UDP glucosyltransferase), putative | |  |  |  |  |  | | --- | --- | --- | --- | --- | |  |  |  |  |  | | At4g02280 ,RAFL05-18-M07  sucrose synthase, putative / sucrose-UDP glucosyltransferase, putative strong similarity to sucrose synthase GI:6682841 from [Citrus unshiu] | | | | | | |
|  | Cluster:10-1 | |  |  | 2 | 41 | 15 | 4605 | 0.01034811 | 0.13452543 | 13 |
|  |  | RAFL03-07-M07 | At4g02380 / late embryogenesis abundant protein family | |  |  |  |  |  | | --- | --- | --- | --- | --- | |  |  |  |  |  | | RAFL03-07-M07 ,At4g02380  late embryogenesis abundant 3 family protein / LEA3 family protein similar to several small proteins (~100 aa) that are induced by heat, auxin, ethylene and wounding such as Phaseolus aureus indole-3-acetic acid induced protein ARG (SW:32292); contains Pfam profile PF03242: Late embryogenesis abundant protein | | | | | | |
|  |  | RAFL06-13-N20 | At4g02380 / late embryogenesis abundant protein family | |  |  |  |  |  | | --- | --- | --- | --- | --- | |  |  |  |  |  | | RAFL06-13-N20 ,At4g02380  late embryogenesis abundant 3 family protein / LEA3 family protein similar to several small proteins (~100 aa) that are induced by heat, auxin, ethylene and wounding such as Phaseolus aureus indole-3-acetic acid induced protein ARG (SW:32292); contains Pfam profile PF03242: Late embryogenesis abundant protein | | | | | | |
| 2\_12150001\_12180000 | | |  |  | A | B | C | D | P | P' | N |
|  | Cluster:3-1 | |  |  | 1 | 215 | 0 | 4447 | 0.04632211 | 0.04632211 | 1 |
|  |  | RAFL08-11-H19 | At2g28550 / AP2 domain transcription factor RAP2.7 | |  |  |  |  |  | | --- | --- | --- | --- | --- | |  |  |  |  |  | | RAFL08-11-H19 ,At2g28550  AP2 domain-containing transcription factor RAP2.7 (RAP2.7) nearly identical to AP2 domain transcription factor RAP2.7 (GI:2281639) [Arabidopsis thaliana] | | | | | | |
| 4\_7170001\_7200000 | | |  |  | A | B | C | D | P | P' | N |
|  | Cluster:6-2 | |  |  | 1 | 175 | 0 | 4487 | 0.03774394 | 0.03774394 | 1 |
|  |  | RAFL05-13-K22 | At4g14300 / heterogeneous nuclear ribonucleoprotein (hnRNP), putative | |  |  |  |  |  | | --- | --- | --- | --- | --- | |  |  |  |  |  | | At4g14300 ,RAFL05-13-K22  heterogeneous nuclear ribonucleoprotein, putative / hnRNP, putative | | | | | | |
| 5\_10950001\_10980000 | | |  |  | A | B | C | D | P | P' | N |
|  | Cluster:3-0 | |  |  | 1 | 232 | 0 | 4430 | 0.049967833 | 0.049967833 | 1 |
|  |  | RAFL05-18-K01 | At5g29000 / expressed protein | |  |  |  |  |  | | --- | --- | --- | --- | --- | |  |  |  |  |  | | At5g29000 ,RAFL05-18-K01  myb family transcription factor contains Pfam profile: PF00249 myb-like DNA-binding domain | | | | | | |
| 2\_15360001\_15390000 | | |  |  | A | B | C | D | P | P' | N |
|  | Cluster:9-1 | |  |  | 1 | 95 | 0 | 4567 | 0.020587604 | 0.020587604 | 1 |
|  |  | RAFL05-18-H16 | At2g36780 / UDP-glycosyltransferase family | |  |  |  |  |  | | --- | --- | --- | --- | --- | |  |  |  |  |  | | At2g36780 ,RAFL05-18-H16  UDP-glucoronosyl/UDP-glucosyl transferase family protein contains Pfam profile: PF00201 UDP-glucoronosyl and UDP-glucosyl transferase | | | | | | |
| 4\_17430001\_17460000 | | |  |  | A | B | C | D | P | P' | N |
|  | Cluster:2-2 | |  |  | 2 | 51 | 0 | 4610 | 1.267773E-4 | 1.267773E-4 | 1 |
|  |  | RAFL09-15-K07 | At4g39800 / myo-inositol-1-phosphate synthase | |  |  |  |  |  | | --- | --- | --- | --- | --- | |  |  |  |  |  | | At4g39800 ,RAFL09-15-K07  inositol-3-phosphate synthase isozyme 1 / myo-inositol-1-phosphate synthase 1 / MI-1-P synthase 1 / IPS 1 identical to SP|P42801 Inositol-3-phosphate synthase isozyme 1 (EC 5.5.1.4) (Myo-inositol-1- phosphate synthase 1) (MI-1-P synthase 1) (IPS 1) {Arabidopsis thaliana} | | | | | | |
|  |  | RAFL09-14-L01 | At4g39800 / myo-inositol-1-phosphate synthase | |  |  |  |  |  | | --- | --- | --- | --- | --- | |  |  |  |  |  | | RAFL09-14-L01 ,At4g39800  inositol-3-phosphate synthase isozyme 1 / myo-inositol-1-phosphate synthase 1 / MI-1-P synthase 1 / IPS 1 identical to SP|P42801 Inositol-3-phosphate synthase isozyme 1 (EC 5.5.1.4) (Myo-inositol-1- phosphate synthase 1) (MI-1-P synthase 1) (IPS 1) {Arabidopsis thaliana} | | | | | | |
| 3\_18420001\_18450000 | | |  |  | A | B | C | D | P | P' | N |
|  | Cluster:1-0 | |  |  | 1 | 148 | 0 | 4514 | 0.031953678 | 0.031953678 | 1 |
|  |  | RAFL06-09-J08 | At3g49720 / expressed protein | |  |  |  |  |  | | --- | --- | --- | --- | --- | |  |  |  |  |  | | RAFL06-09-J08 ,At3g49720  expressed protein | | | | | | |
| 1\_2460001\_2490000 | | |  |  | A | B | C | D | P | P' | N |
|  | Cluster:2-0 | |  |  | 1 | 149 | 0 | 4513 | 0.03216813 | 0.03216813 | 1 |
|  |  | RAFL03-08-O03 | At1g07940 / elongation factor 1-alpha (EF-1-alpha) | |  |  |  |  |  | | --- | --- | --- | --- | --- | |  |  |  |  |  | | RAFL03-08-O03 ,At1g07940  elongation factor 1-alpha / EF-1-alpha identical to GB:CAA34456 from [Arabidopsis thaliana] (Plant Mol. Biol. 14 (1), 107-110 (1990)) | | | | | | |
| 3\_17820001\_17850000 | | |  |  | A | B | C | D | P | P' | N |
|  | Cluster:0-2 | |  |  | 1 | 78 | 0 | 4584 | 0.016941883 | 0.016941883 | 1 |
|  |  | RAFL04-14-I05 | At3g48200 / expressed protein | |  |  |  |  |  | | --- | --- | --- | --- | --- | |  |  |  |  |  | | At3g48200 ,RAFL04-14-I05  expressed protein | | | | | | |
| 3\_19560001\_19590000 | | |  |  | A | B | C | D | P | P' | N |
|  | Cluster:8-1 | |  |  | 1 | 161 | 0 | 4501 | 0.034741584 | 0.034741584 | 1 |
|  |  | RAFL04-17-A20 | At3g52800 / zinc finger - like protein | |  |  |  |  |  | | --- | --- | --- | --- | --- | |  |  |  |  |  | | At3g52800 ,RAFL04-17-A20  zinc finger (AN1-like) family protein contains Pfam domain, PF01428: AN1-like Zinc finger | | | | | | |
| 4\_1800001\_2100000 | | |  |  | A | B | C | D | P | P' | N |
|  | Cluster:10-2 | |  |  | 1 | 108 | 0 | 4554 | 0.02337551 | 0.02337551 | 1 |
|  |  | RAFL07-15-N09 | At4g04020 / plastid-lipid associated protein PAP/fibrillin, putative | |  |  |  |  |  | | --- | --- | --- | --- | --- | |  |  |  |  |  | | RAFL07-15-N09 ,At4g04020  plastid-lipid associated protein PAP, putative / fibrillin, putative strong similarity to plastid-lipid associated proteins PAP1 GI:14248554, PAP2 GI:14248556 from [Brassica rapa], fibrillin [Brassica napus] GI:4139097; contains Pfam profile PF04755: PAP\_fibrillin | | | | | | |
| Chromosome:4 | | |  |  | A | B | C | D | P | P' | N |
|  | Cluster:8-1 | |  |  | 33 | 129 | 599 | 3902 | 0.0137234 | 0.45287222 | 33 |
|  |  | RAFL08-18-B11 | At4g36990 / heat shock transcription factor 4 (HSF4) | |  |  |  |  |  | | --- | --- | --- | --- | --- | |  |  |  |  |  | | RAFL08-18-B11 ,At4g36990  heat shock factor protein 4 (HSF4) / heat shock transcription factor 4 (HSTF4) identical to heat shock transcription factor 4 (HSF4) SP:Q96320 from [Arabidopsis thaliana] | | | | | | |
|  |  | RAFL05-14-K07 | At4g27680 / expressed protein | |  |  |  |  |  | | --- | --- | --- | --- | --- | |  |  |  |  |  | | RAFL05-14-K07 ,At4g27680  MSP1 protein, putative / intramitochondrial sorting protein, putative similar to Swiss-Prot:P28737 MSP1 protein (TAT-binding homolog 4) [Saccharomyces cerevisiae]; contains Pfam domain, PF00004: ATPase, AAA family | | | | | | |
|  |  | RAFL07-10-J07 | At4g31550 / WRKY family transcription factor | |  |  |  |  |  | | --- | --- | --- | --- | --- | |  |  |  |  |  | | RAFL07-10-J07 ,At4g31550  WRKY family transcription factor contains Pfam profile: PF03106 WRKY DNA -binding domain | | | | | | |
|  |  | RAFL09-17-E21 | At4g19410 / pectinacetylesterase, putative | |  |  |  |  |  | | --- | --- | --- | --- | --- | |  |  |  |  |  | | RAFL09-17-E21 ,At4g19410  pectinacetylesterase, putative similar to pectinacetylesterase precursor GI:1431629 from [Vigna radiata] | | | | | | |
|  |  | RAFL05-14-B17 | At4g29160 / expressed protein | |  |  |  |  |  | | --- | --- | --- | --- | --- | |  |  |  |  |  | | At4g29160 ,RAFL05-14-B17  SNF7 family protein contains Pfam domain, PF03357: SNF7 family | | | | | | |
|  |  | RAFL04-16-G03 | At4g02890 / polyubiquitin (UBQ14) | |  |  |  |  |  | | --- | --- | --- | --- | --- | |  |  |  |  |  | | RAFL04-16-G03 ,At4g02890  polyubiquitin (UBQ14) identical to GI:166795; similar to N. sylvestris hexameric polyubiquitin, GenBank accession number M74101 | | | | | | |
|  |  | RAFL09-10-G07 | At4g18950 / protein kinase - like protein | |  |  |  |  |  | | --- | --- | --- | --- | --- | |  |  |  |  |  | | At4g18950 ,RAFL09-10-G07  ankyrin protein kinase, putative similar to ankyrin-kinase [Medicago truncatula] gi|18700701|gb|AAL78674 | | | | | | |
|  |  | RAFL05-08-J06 | At4g19640 / GTP-binding protein, putative | |  |  |  |  |  | | --- | --- | --- | --- | --- | |  |  |  |  |  | | At4g19640 ,RAFL05-08-J06  Ras-related GTP-binding protein, putative similar to GTP-binding protein RAB5A GI:1370178 from [Lotus japonicus] | | | | | | |
|  |  | RAFL05-18-E11 | At4g03430 / pre-mRNA splicing factor -related | |  |  |  |  |  | | --- | --- | --- | --- | --- | |  |  |  |  |  | | RAFL05-18-E11 ,At4g03430  pre-mRNA splicing factor-related similar to pre-mRNA splicing factor pre-mRNA splicing factor prp1 (SP:Q12381) [Fission yeast] | | | | | | |
|  |  | RAFL09-11-C11 | At4g12040 / expressed protein | |  |  |  |  |  | | --- | --- | --- | --- | --- | |  |  |  |  |  | | RAFL09-11-C11 ,At4g12040  zinc finger (AN1-like) family protein contains Pfam domains, PF01428: AN1-like Zinc finger and PF01754: A20-like zinc finger | | | | | | |
|  |  | RAFL05-07-H10 | At4g30990 / hypothetical protein | |  |  |  |  |  | | --- | --- | --- | --- | --- | |  |  |  |  |  | | At4g30990 ,RAFL05-07-H10  expressed protein ; expression supported by MPSS | | | | | | |
|  |  | RAFL09-09-O15 | At4g05320 / polyubiquitin UBQ10/SEN3 | |  |  |  |  |  | | --- | --- | --- | --- | --- | |  |  |  |  |  | | At4g05320 ,RAFL09-09-O15  polyubiquitin (UBQ10) (SEN3) senescence-associated protein; identical to GI:870791 | | | | | | |
|  |  | RAFL09-06-I17 | At4g05320 / polyubiquitin UBQ10/SEN3 | |  |  |  |  |  | | --- | --- | --- | --- | --- | |  |  |  |  |  | | At4g05320 ,RAFL09-06-I17  polyubiquitin (UBQ10) (SEN3) senescence-associated protein; identical to GI:870791 | | | | | | |
|  |  | RAFL04-17-I16 | At4g02380 / late embryogenesis abundant protein family | |  |  |  |  |  | | --- | --- | --- | --- | --- | |  |  |  |  |  | | RAFL04-17-I16 ,At4g02380  late embryogenesis abundant 3 family protein / LEA3 family protein similar to several small proteins (~100 aa) that are induced by heat, auxin, ethylene and wounding such as Phaseolus aureus indole-3-acetic acid induced protein ARG (SW:32292); contains Pfam profile PF03242: Late embryogenesis abundant protein | | | | | | |
|  |  | RAFL11-02-D14 | At4g36730 / G-box-binding factor 1 | |  |  |  |  |  | | --- | --- | --- | --- | --- | |  |  |  |  |  | | At4g36730 ,RAFL11-02-D14  G-box binding factor 1 (GBF1) identical to G-box binding factor 1 SP:P42774 from [Arabidopsis thaliana]; contains Pfam profile: PF00170 bZIP transcription factor | | | | | | |
|  |  | RAFL09-14-I01 | At4g33920 / expressed protein | |  |  |  |  |  | | --- | --- | --- | --- | --- | |  |  |  |  |  | | At4g33920 ,RAFL09-14-I01  protein phosphatase 2C family protein / PP2C family protein similar to Ser/Thr protein phosphatase 2C (PP2C6) (GI:15020818) [Arabidopsis thaliana]; similar to protein phosphatase 2C (GI:3608412) [Mesembryanthemum crystallinum]; contains Pfam PF00481 : Protein phosphatase 2C domain | | | | | | |
|  |  | RAFL08-09-D17 | At4g11220 / expressed protein | |  |  |  |  |  | | --- | --- | --- | --- | --- | |  |  |  |  |  | | At4g11220 ,RAFL08-09-D17  reticulon family protein (RTNLB2) similar to SP|Q64548 Reticulon 1 (Neuroendocrine-specific protein) {Rattus norvegicus}; contains Pfam profile PF02453: Reticulon | | | | | | |
|  |  | RAFL05-14-O22 | At4g05320 / polyubiquitin UBQ10/SEN3 | |  |  |  |  |  | | --- | --- | --- | --- | --- | |  |  |  |  |  | | At4g05320 ,RAFL05-14-O22  polyubiquitin (UBQ10) (SEN3) senescence-associated protein; identical to GI:870791 | | | | | | |
|  |  | RAFL05-19-O11 | At4g29190 / expressed protein | |  |  |  |  |  | | --- | --- | --- | --- | --- | |  |  |  |  |  | | At4g29190 ,RAFL05-19-O11  zinc finger (CCCH-type) family protein contains Pfam domain, PF00642: Zinc finger C-x8-C-x5-C-x3-H type (and similar) | | | | | | |
|  |  | RAFL04-12-D15 | At4g38360 / expressed protein | |  |  |  |  |  | | --- | --- | --- | --- | --- | |  |  |  |  |  | | RAFL04-12-D15 ,At4g38360  expressed protein contains Pfam profile PF03619: Domain of unknown function | | | | | | |
|  |  | RAFL02-09-C13 | At4g28240 / wound induced protein -related | |  |  |  |  |  | | --- | --- | --- | --- | --- | |  |  |  |  |  | | At4g28240 ,RAFL02-09-C13  wound-responsive protein-related wound-induced protein - tomato (fragment), PIR2:S19773 | | | | | | |
|  |  | RAFL04-12-N22 | At4g25170 / expressed protein | |  |  |  |  |  | | --- | --- | --- | --- | --- | |  |  |  |  |  | | At4g25170 ,RAFL04-12-N22  expressed protein | | | | | | |
|  |  | RAFL05-07-L01 | At4g24400 / CBL-interacting protein kinase 8 | |  |  |  |  |  | | --- | --- | --- | --- | --- | |  |  |  |  |  | | RAFL05-07-L01 ,At4g24400  CBL-interacting protein kinase 8 (CIPK8) identical to CBL-interacting protein kinase 8 [Arabidopsis thaliana] GP|13249115|gb|AAK16683; contains Pfam profiles PF00069: Protein kinase domain and PF03822: NAF domain | | | | | | |
|  |  | RAFL08-18-J23 | At4g25650 / Rieske (2Fe-2S) domain-containing protein | |  |  |  |  |  | | --- | --- | --- | --- | --- | |  |  |  |  |  | | RAFL08-18-J23 ,At4g25650  Rieske [2Fe-2S] domain-containing protein similar to cell death suppressor protein lls1 from Zea mays [gi:1935909], Rieske iron-sulfur protein Tic55 from Pisum sativum [gi:2764524]; contains Pfam PF00355 Rieske [2Fe-2S] domain | | | | | | |
|  |  | RAFL09-12-B15 | At4g30600 / signal recognition particle receptor-related protein | |  |  |  |  |  | | --- | --- | --- | --- | --- | |  |  |  |  |  | | At4g30600 ,RAFL09-12-B15  signal recognition particle receptor alpha subunit family protein similar to Signal recognition particle receptor alpha subunit (SR-alpha) (Docking protein alpha) (DP-alpha) (SP:P08240) [Homo sapiens}; similar to Signal recognition particle receptor alpha subunit (SR-alpha) (Docking protein alpha) (DP-alpha) (SP:P06625) [Canis familiaris}; contains Pfam PF04086: Signal recognition particle, alpha subunit, N-terminal; contains Pfam PF00448: SRP54-type protein, GTPase domain | | | | | | |
|  |  | RAFL08-16-G17 | At4g17500 / ethylene responsive element binding factor 1 (frameshift !) | |  |  |  |  |  | | --- | --- | --- | --- | --- | |  |  |  |  |  | | RAFL08-16-G17 ,At4g17500  ethylene-responsive element-binding protein 1 (ERF1) / EREBP-2 protein identical to SP|O80337 Ethylene responsive element binding factor 1 (EREBP-2 protein) [Arabidopsis thaliana]; a false single bp exon was added to circumvent a single basepair insertion in the genomic sequence, supported by cDNA/genome alignment. | | | | | | |
|  |  | RAFL07-10-D10 | At4g05320 / polyubiquitin UBQ10/SEN3 | |  |  |  |  |  | | --- | --- | --- | --- | --- | |  |  |  |  |  | | At4g05320 ,RAFL07-10-D10  polyubiquitin (UBQ10) (SEN3) senescence-associated protein; identical to GI:870791 | | | | | | |
|  |  | RAFL05-16-P22 | At4g05150 / octicosapeptide/Phox/Bem1p (PB1) domain-containing protein | |  |  |  |  |  | | --- | --- | --- | --- | --- | |  |  |  |  |  | | At4g05150 ,RAFL05-16-P22  octicosapeptide/Phox/Bem1p (PB1) domain-containing protein various predicted proteins contains Pfam profile PF00564: PB1 domain | | | | | | |
|  |  | RAFL05-18-A07 | At4g27500 / proton pump interactor | |  |  |  |  |  | | --- | --- | --- | --- | --- | |  |  |  |  |  | | RAFL05-18-A07 ,At4g27500  expressed protein non-consensus GA donor splice site at exon 6 | | | | | | |
|  |  | RAFL09-15-J03 | At4g29160 / expressed protein | |  |  |  |  |  | | --- | --- | --- | --- | --- | |  |  |  |  |  | | RAFL09-15-J03 ,At4g29160  SNF7 family protein contains Pfam domain, PF03357: SNF7 family | | | | | | |
|  |  | RAFL05-14-J24 | At4g26750 / proline-rich protein family | |  |  |  |  |  | | --- | --- | --- | --- | --- | |  |  |  |  |  | | RAFL05-14-J24 ,At4g26750  hydroxyproline-rich glycoprotein family protein | | | | | | |
|  |  | RAFL11-01-P18 | At4g31770 / calcineurin-like phosphoesterase family | |  |  |  |  |  | | --- | --- | --- | --- | --- | |  |  |  |  |  | | At4g31770 ,RAFL11-01-P18  calcineurin-like phosphoesterase family protein contains Pfam profile: PF00149 calcineurin-like phosphoesterase | | | | | | |
|  |  | RAFL08-16-I23 | At4g01120 / G-box binding factor(bZIP protein), putative | |  |  |  |  |  | | --- | --- | --- | --- | --- | |  |  |  |  |  | | RAFL08-16-I23 ,At4g01120  G-box binding factor 2 (GBF2) identical to G-box binding factor 2 (GBF2) SP:P42775 from [Arabidopsis thaliana];contains Pfam profile: PF00170 bZIP transcription factor | | | | | | |
| 4\_5100001\_5130000 | | |  |  | A | B | C | D | P | P' | N |
|  | Cluster:9-1 | |  |  | 1 | 95 | 0 | 4567 | 0.020587604 | 0.020587604 | 1 |
|  |  | RAFL06-16-C13 | At4g09760 / choline kinase GmCK2p -related protein | |  |  |  |  |  | | --- | --- | --- | --- | --- | |  |  |  |  |  | | At4g09760 ,RAFL06-16-C13  choline kinase, putative similar to GmCK2p choline kinase gi|1438881|gb|AAC49375 | | | | | | |
| 5\_21540001\_21570000 | | |  |  | A | B | C | D | P | P' | N |
|  | Cluster:8-1 | |  |  | 1 | 161 | 0 | 4501 | 0.034741584 | 0.034741584 | 1 |
|  |  | RAFL05-19-N02 | At5g53800 / expressed protein | |  |  |  |  |  | | --- | --- | --- | --- | --- | |  |  |  |  |  | | At5g53800 ,RAFL05-19-N02  expressed protein | | | | | | |
| 2\_7770001\_7800000 | | |  |  | A | B | C | D | P | P' | N |
|  | Cluster:8-0 | |  |  | 1 | 108 | 1 | 4553 | 0.0462095 | 0.092419 | 2 |
|  |  | RAFL05-20-P13 | At2g18050 / histone H1 | |  |  |  |  |  | | --- | --- | --- | --- | --- | |  |  |  |  |  | | RAFL05-20-P13 ,At2g18050  histone H1-3 (HIS1-3) similar to histone H1 [Lycopersicon pennellii] SWISS-PROT:P40267; identical to cDNA histone H1-3 (His1-3) GI:1809314, histone H1-3 [Arabidopsis thaliana] GI:1809305 | | | | | | |
| 4\_10770001\_10800000 | | |  |  | A | B | C | D | P | P' | N |
|  | Cluster:5-2 | |  |  | 1 | 125 | 0 | 4537 | 0.027021231 | 0.027021231 | 1 |
|  |  | RAFL04-20-J03 | At4g22380 / Ribosomal protein L7Ae family | |  |  |  |  |  | | --- | --- | --- | --- | --- | |  |  |  |  |  | | At4g22380 ,RAFL04-20-J03  ribosomal protein L7Ae/L30e/S12e/Gadd45 family protein Similar to NHP2/L7Ae family proteins, see SWISSPROT:P32495 and PMID:2063628. | | | | | | |
| 5\_16140001\_16170000 | | |  |  | A | B | C | D | P | P' | N |
|  | Cluster:8-0 | |  |  | 1 | 108 | 0 | 4554 | 0.02337551 | 0.02337551 | 1 |
|  |  | RAFL05-05-N17 | At5g41040 / transferase family | |  |  |  |  |  | | --- | --- | --- | --- | --- | |  |  |  |  |  | | At5g41040 ,RAFL05-05-N17  transferase family protein similar to hypersensitivity-related gene product HSR201 - Nicotiana tabacum, EMBL:X95343; contains Pfam transferase family domain PF00248 | | | | | | |
| 1\_18000001\_21000000 | | |  |  | A | B | C | D | P | P' | N |
|  | Cluster:0-2 | |  |  | 9 | 70 | 115 | 4469 | 2.1293285E-4 | 0.006387986 | 30 |
|  |  | RAFL04-17-G02 | At1g56340 / calreticulin 1 (CRT1) | |  |  |  |  |  | | --- | --- | --- | --- | --- | |  |  |  |  |  | | At1g56340 ,RAFL04-17-G02  calreticulin 1 (CRT1) identical to calreticulin (crt1) GI:2052379 [Arabidopsis thaliana] | | | | | | |
|  |  | RAFL06-15-G08 | At1g52220 / expressed protein | |  |  |  |  |  | | --- | --- | --- | --- | --- | |  |  |  |  |  | | At1g52220 ,RAFL06-15-G08  expressed protein | | | | | | |
|  |  | RAFL05-17-G01 | At1g51400 / photosystem II 5 KD protein | |  |  |  |  |  | | --- | --- | --- | --- | --- | |  |  |  |  |  | | At1g51400 ,RAFL05-17-G01  photosystem II 5 kD protein 100% identical to GI:4836947 (F5D21.10) | | | | | | |
|  |  | RAFL04-09-M24 | At1g54500 / rubredoxin -related | |  |  |  |  |  | | --- | --- | --- | --- | --- | |  |  |  |  |  | | At1g54500 ,RAFL04-09-M24  rubredoxin family protein similar to SP|P00270 Rubredoxin (Rd) {Desulfovibrio gigas}; contains Pfam profile PF00301: Rubredoxin | | | | | | |
|  |  | RAFL05-19-G04 | At1g54780 / thylakoid lumen 18.3 kDa protein | |  |  |  |  |  | | --- | --- | --- | --- | --- | |  |  |  |  |  | | RAFL05-19-G04 ,At1g54780  thylakoid lumen 18.3 kDa protein SP:Q9ZVL6 | | | | | | |
|  |  | RAFL05-04-A19 | At1g51400 / photosystem II 5 KD protein | |  |  |  |  |  | | --- | --- | --- | --- | --- | |  |  |  |  |  | | At1g51400 ,RAFL05-04-A19  photosystem II 5 kD protein 100% identical to GI:4836947 (F5D21.10) | | | | | | |
|  |  | RAFL05-01-I05 | At1g54780 / thylakoid lumen 18.3 kDa protein | |  |  |  |  |  | | --- | --- | --- | --- | --- | |  |  |  |  |  | | RAFL05-01-I05 ,At1g54780  thylakoid lumen 18.3 kDa protein SP:Q9ZVL6 | | | | | | |
|  |  | RAFL07-14-F21 | At1g54780 / thylakoid lumen 18.3 kDa protein | |  |  |  |  |  | | --- | --- | --- | --- | --- | |  |  |  |  |  | | RAFL07-14-F21 ,At1g54780  thylakoid lumen 18.3 kDa protein SP:Q9ZVL6 | | | | | | |
|  |  | RAFL11-03-K23 | At1g54500 / rubredoxin -related | |  |  |  |  |  | | --- | --- | --- | --- | --- | |  |  |  |  |  | | At1g54500 ,RAFL11-03-K23  rubredoxin family protein similar to SP|P00270 Rubredoxin (Rd) {Desulfovibrio gigas}; contains Pfam profile PF00301: Rubredoxin | | | | | | |
|  | Cluster:0-1 | |  |  | 9 | 97 | 115 | 4442 | 0.0018587124 | 0.05576137 | 30 |
|  |  | RAFL03-06-N04 | At1g55670 / photosystem I subunit V precursor -related | |  |  |  |  |  | | --- | --- | --- | --- | --- | |  |  |  |  |  | | RAFL03-06-N04 ,At1g55670  photosystem I reaction center subunit V, chloroplast, putative / PSI-G, putative (PSAG) identical to SP|Q9S7N7; similar to SP|Q00327 Photosystem I reaction center subunit V, chloroplast precursor (PSI-G) (Photosystem I 9 kDa protein) {Hordeum vulgare}; contains Pfam profile PF01241: Photosystem I psaG / psaK | | | | | | |
|  |  | RAFL05-04-D24 | At1g52230 / photosystem I subunit VI precursor | |  |  |  |  |  | | --- | --- | --- | --- | --- | |  |  |  |  |  | | At1g52230 ,RAFL05-04-D24  photosystem I reaction center subunit VI, chloroplast, putative / PSI-H, putative (PSAH2) identical to SP|Q9SUI6; similar to PSI-H precursor [Nicotiana sylvestris] GI:407355; contains Pfam profile PF03244: Photosystem I reaction centre subunit VI | | | | | | |
|  |  | RAFL06-07-I03 | At1g50900 / expressed protein | |  |  |  |  |  | | --- | --- | --- | --- | --- | |  |  |  |  |  | | At1g50900 ,RAFL06-07-I03  expressed protein | | | | | | |
|  |  | RAFL05-03-B20 | At1g52400 / glycosyl hydrolase family 1, beta-glucosidase (BG1) | |  |  |  |  |  | | --- | --- | --- | --- | --- | |  |  |  |  |  | | RAFL05-03-B20 ,At1g52400  glycosyl hydrolase family 1 protein / beta-glucosidase, putative (BG1) contains Pfam PF00232 : Glycosyl hydrolase family 1 domain; TIGRFAM TIGR01233: 6-phospho-beta-galactosidase; identical to GI:6651430 from [Arabidopsis thaliana] | | | | | | |
|  |  | RAFL05-11-D11 | At1g55490 / RuBisCo subunit binding-protein beta subunit/60 kDa chaperonin beta subunit | |  |  |  |  |  | | --- | --- | --- | --- | --- | |  |  |  |  |  | | RAFL05-11-D11 ,At1g55490  RuBisCO subunit binding-protein beta subunit, chloroplast / 60 kDa chaperonin beta subunit / CPN-60 beta identical to SWISS-PROT:P21240- RuBisCO subunit binding-protein beta subunit, chloroplast precursor (60 kDa chaperonin beta subunit, CPN-60 beta) [Arabidopsis thaliana] | | | | | | |
|  |  | RAFL07-11-O20 | At1g52980 / GTP-binding protein -related | |  |  |  |  |  | | --- | --- | --- | --- | --- | |  |  |  |  |  | | At1g52980 ,RAFL07-11-O20  GTP-binding family protein contains Pfam domain, PF01926: GTPase of unknown function | | | | | | |
|  |  | RAFL07-10-G07 | At1g52400 / glycosyl hydrolase family 1, beta-glucosidase (BG1) | |  |  |  |  |  | | --- | --- | --- | --- | --- | |  |  |  |  |  | | At1g52400 ,RAFL07-10-G07  glycosyl hydrolase family 1 protein / beta-glucosidase, putative (BG1) contains Pfam PF00232 : Glycosyl hydrolase family 1 domain; TIGRFAM TIGR01233: 6-phospho-beta-galactosidase; identical to GI:6651430 from [Arabidopsis thaliana] | | | | | | |
|  |  | RAFL07-07-I23 | At1g56190 / phosphoglycerate kinase -related | |  |  |  |  |  | | --- | --- | --- | --- | --- | |  |  |  |  |  | | RAFL07-07-I23 ,At1g56190  phosphoglycerate kinase, putative similar to SP|P41758 Phosphoglycerate kinase, chloroplast precursor (EC 2.7.2.3) {Chlamydomonas reinhardtii}; contains Pfam profile PF00162: phosphoglycerate kinase | | | | | | |
|  |  | RAFL07-11-K16 | At1g52000 / jacalin lectin family | |  |  |  |  |  | | --- | --- | --- | --- | --- | |  |  |  |  |  | | At1g52000 ,RAFL07-11-K16  jacalin lectin family protein similar to myrosinase binding protein [Brassica napus] GI:1711296, myrosinase-binding protein homolog [Arabidopsis thaliana] GI:2997767; contains Pfam profile: PF01419 jacalin-like lectin domain | | | | | | |
|  | Cluster:10-0 | |  |  | 4 | 20 | 120 | 4519 | 0.0033508611 | 0.10052583 | 30 |
|  |  | RAFL05-04-I14 | At1g52690 / late embryogenesis abundant (LEA) protein, putative | |  |  |  |  |  | | --- | --- | --- | --- | --- | |  |  |  |  |  | | At1g52690 ,RAFL05-04-I14  late embryogenesis abundant protein, putative / LEA protein, putative similar to SP|P13934 Late embryogenesis abundant protein 76 (LEA 76) {Brassica napus}; contains Pfam profile PF02987: Late embryogenesis abundant protein | | | | | | |
|  |  | RAFL08-14-E03 | At1g52690 / late embryogenesis abundant (LEA) protein, putative | |  |  |  |  |  | | --- | --- | --- | --- | --- | |  |  |  |  |  | | At1g52690 ,RAFL08-14-E03  late embryogenesis abundant protein, putative / LEA protein, putative similar to SP|P13934 Late embryogenesis abundant protein 76 (LEA 76) {Brassica napus}; contains Pfam profile PF02987: Late embryogenesis abundant protein | | | | | | |
|  |  | RAFL06-13-J20 | At1g52690 / late embryogenesis abundant (LEA) protein, putative | |  |  |  |  |  | | --- | --- | --- | --- | --- | |  |  |  |  |  | | At1g52690 ,RAFL06-13-J20  late embryogenesis abundant protein, putative / LEA protein, putative similar to SP|P13934 Late embryogenesis abundant protein 76 (LEA 76) {Brassica napus}; contains Pfam profile PF02987: Late embryogenesis abundant protein | | | | | | |
|  |  | RAFL08-13-P06 | At1g52690 / late embryogenesis abundant (LEA) protein, putative | |  |  |  |  |  | | --- | --- | --- | --- | --- | |  |  |  |  |  | | At1g52690 ,RAFL08-13-P06  late embryogenesis abundant protein, putative / LEA protein, putative similar to SP|P13934 Late embryogenesis abundant protein 76 (LEA 76) {Brassica napus}; contains Pfam profile PF02987: Late embryogenesis abundant protein | | | | | | |
|  | Cluster:9-0 | |  |  | 4 | 28 | 120 | 4511 | 0.00962857 | 0.2888571 | 30 |
|  |  | RAFL05-21-E06 | At1g54100 / aldehyde dehydrogenase, putative (ALDH) | |  |  |  |  |  | | --- | --- | --- | --- | --- | |  |  |  |  |  | | At1g54100 ,RAFL05-21-E06  aldehyde dehydrogenase, putative / antiquitin, putative strong similarity to SP|Q41247 Aldehyde dehydrogenase family 7 member A1 (EC 1.2.1.3) (Antiquitin 1) (Brassica turgor-responsive/drought-induced gene 26 protein) (Btg-26) {Brassica napus}; similar to turgor-responsive protein 26G (aldehyde dehydrogenase family 7 member A1) [Pisum sativum] SWISS-PROT:P25795 | | | | | | |
|  |  | RAFL04-09-D07 | At1g54100 / aldehyde dehydrogenase, putative (ALDH) | |  |  |  |  |  | | --- | --- | --- | --- | --- | |  |  |  |  |  | | RAFL04-09-D07 ,At1g54100  aldehyde dehydrogenase, putative / antiquitin, putative strong similarity to SP|Q41247 Aldehyde dehydrogenase family 7 member A1 (EC 1.2.1.3) (Antiquitin 1) (Brassica turgor-responsive/drought-induced gene 26 protein) (Btg-26) {Brassica napus}; similar to turgor-responsive protein 26G (aldehyde dehydrogenase family 7 member A1) [Pisum sativum] SWISS-PROT:P25795 | | | | | | |
|  |  | RAFL08-09-C23 | At1g54100 / aldehyde dehydrogenase, putative (ALDH) | |  |  |  |  |  | | --- | --- | --- | --- | --- | |  |  |  |  |  | | RAFL08-09-C23 ,At1g54100  aldehyde dehydrogenase, putative / antiquitin, putative strong similarity to SP|Q41247 Aldehyde dehydrogenase family 7 member A1 (EC 1.2.1.3) (Antiquitin 1) (Brassica turgor-responsive/drought-induced gene 26 protein) (Btg-26) {Brassica napus}; similar to turgor-responsive protein 26G (aldehyde dehydrogenase family 7 member A1) [Pisum sativum] SWISS-PROT:P25795 | | | | | | |
|  |  | RAFL08-15-L09 | At1g54100 / aldehyde dehydrogenase, putative (ALDH) | |  |  |  |  |  | | --- | --- | --- | --- | --- | |  |  |  |  |  | | RAFL08-15-L09 ,At1g54100  aldehyde dehydrogenase, putative / antiquitin, putative strong similarity to SP|Q41247 Aldehyde dehydrogenase family 7 member A1 (EC 1.2.1.3) (Antiquitin 1) (Brassica turgor-responsive/drought-induced gene 26 protein) (Btg-26) {Brassica napus}; similar to turgor-responsive protein 26G (aldehyde dehydrogenase family 7 member A1) [Pisum sativum] SWISS-PROT:P25795 | | | | | | |
| 1\_6930001\_6960000 | | |  |  | A | B | C | D | P | P' | N |
|  | Cluster:1-0 | |  |  | 1 | 148 | 0 | 4514 | 0.031953678 | 0.031953678 | 1 |
|  |  | RAFL09-11-P11 | At1g20010 / tubulin beta-5 chain (TUB5) | |  |  |  |  |  | | --- | --- | --- | --- | --- | |  |  |  |  |  | | RAFL09-11-P11 ,At1g20010  tubulin beta-5 chain (TUB5) nearly identical to SP|P29513 Tubulin beta-5 chain {Arabidopsis thaliana} | | | | | | |
| 2\_270001\_300000 | | |  |  | A | B | C | D | P | P' | N |
|  | Cluster:2-0 | |  |  | 1 | 149 | 0 | 4513 | 0.03216813 | 0.03216813 | 1 |
|  |  | RAFL06-10-E23 | At2g01660 / expressed protein | |  |  |  |  |  | | --- | --- | --- | --- | --- | |  |  |  |  |  | | At2g01660 ,RAFL06-10-E23  33 kDa secretory protein-related contains Pfam PF01657: Domain of unknown function, duplicated in 33 KDa secretory proteins | | | | | | |
| 4\_5400001\_5700000 | | |  |  | A | B | C | D | P | P' | N |
|  | Cluster:3-0 | |  |  | 2 | 231 | 4 | 4426 | 0.032636177 | 0.1305447 | 4 |
|  |  | RAFL08-18-K16 | At4g10480 / alpha NAC -related | |  |  |  |  |  | | --- | --- | --- | --- | --- | |  |  |  |  |  | | RAFL08-18-K16 ,At4g10480  nascent polypeptide associated complex alpha chain protein, putative / alpha-NAC, putative similar to alpha-NAC, non-muscle form [Mus musculus] GI:1666690; contains Pfam profiles PF01849: NAC domain, PF00627: UBA/TS-N domain | | | | | | |
|  |  | RAFL05-12-D01 | At4g10480 / alpha NAC -related | |  |  |  |  |  | | --- | --- | --- | --- | --- | |  |  |  |  |  | | RAFL05-12-D01 ,At4g10480  nascent polypeptide associated complex alpha chain protein, putative / alpha-NAC, putative similar to alpha-NAC, non-muscle form [Mus musculus] GI:1666690; contains Pfam profiles PF01849: NAC domain, PF00627: UBA/TS-N domain | | | | | | |
|  | Cluster:2-1 | |  |  | 2 | 242 | 4 | 4415 | 0.0355681 | 0.1422724 | 4 |
|  |  | RAFL04-13-M20 | At4g11010 / nucleoside diphosphate kinase 3 (ndpk3) | |  |  |  |  |  | | --- | --- | --- | --- | --- | |  |  |  |  |  | | At4g11010 ,RAFL04-13-M20  nucleoside diphosphate kinase 3, mitochondrial (NDK3) identical to Nucleoside diphosphate kinase III, mitochondrial precursor (NDK III) (NDP kinase III) (NDPK III) (SP:O49203) [Arabidopsis thaliana]; contains Pfam PF00334 : Nucleoside diphosphate kinase domain; | | | | | | |
|  |  | RAFL05-21-M05 | At4g10480 / alpha NAC -related | |  |  |  |  |  | | --- | --- | --- | --- | --- | |  |  |  |  |  | | At4g10480 ,RAFL05-21-M05  nascent polypeptide associated complex alpha chain protein, putative / alpha-NAC, putative similar to alpha-NAC, non-muscle form [Mus musculus] GI:1666690; contains Pfam profiles PF01849: NAC domain, PF00627: UBA/TS-N domain | | | | | | |
| 2\_17820001\_17850000 | | |  |  | A | B | C | D | P | P' | N |
|  | Cluster:10-2 | |  |  | 1 | 108 | 1 | 4553 | 0.0462095 | 0.092419 | 2 |
|  |  | RAFL05-17-I08 | At2g43020 / amine oxidase family | |  |  |  |  |  | | --- | --- | --- | --- | --- | |  |  |  |  |  | | At2g43020 ,RAFL05-17-I08  amine oxidase family protein similar to polyamine oxidase SP:O64411 [Zea mays]; contains Pfam profile PF01593 amine oxidase, flavin-containing | | | | | | |
| 5\_7440001\_7470000 | | |  |  | A | B | C | D | P | P' | N |
|  | Cluster:10-2 | |  |  | 1 | 108 | 0 | 4554 | 0.02337551 | 0.02337551 | 1 |
|  |  | RAFL09-17-P13 | At5g22500 / male sterility 2-related protein (emb|CAA68191.1) | |  |  |  |  |  | | --- | --- | --- | --- | --- | |  |  |  |  |  | | At5g22500 ,RAFL09-17-P13  acyl CoA reductase, putative / male-sterility protein, putative similar to acyl CoA reductase [Simmondsia chinensis] GI:5020215; contains Pfam profile PF03015: Male sterility protein; identical to cDNA male sterility 2-like protein GI:1491614 | | | | | | |
| 2\_12570001\_12600000 | | |  |  | A | B | C | D | P | P' | N |
|  | Cluster:9-0 | |  |  | 1 | 31 | 3 | 4628 | 0.027177518 | 0.10871007 | 4 |
|  |  | RAFL04-17-K13 | At2g29460 / glutathione transferase, putative | |  |  |  |  |  | | --- | --- | --- | --- | --- | |  |  |  |  |  | | RAFL04-17-K13 ,At2g29460  glutathione S-transferase, putative | | | | | | |
| 5\_23250001\_23280000 | | |  |  | A | B | C | D | P | P' | N |
|  | Cluster:0-2 | |  |  | 1 | 78 | 2 | 4582 | 0.049979966 | 0.1499399 | 3 |
|  |  | RAFL06-11-B13 | At5g58250 / unknown protein (sp|P72777) -related | |  |  |  |  |  | | --- | --- | --- | --- | --- | |  |  |  |  |  | | At5g58250 ,RAFL06-11-B13  expressed protein | | | | | | |
| 3\_3000001\_6000000 | | |  |  | A | B | C | D | P | P' | N |
|  | Cluster:1-0 | |  |  | 13 | 136 | 172 | 4342 | 0.007977094 | 0.255267 | 32 |
|  |  | RAFL06-08-E07 | At3g11630 / 2-cys peroxiredoxin BAS1 precursor (thiol-specific antioxidant protein) -related | |  |  |  |  |  | | --- | --- | --- | --- | --- | |  |  |  |  |  | | At3g11630 ,RAFL06-08-E07  2-cys peroxiredoxin, chloroplast (BAS1) identical to SP|Q96291 2-cys peroxiredoxin BAS1, chloroplast precursor {Arabidopsis thaliana}; contains Pfam profile: PF00578 AhpC/TSA (alkyl hydroperoxide reductase and thiol-specific antioxidant) family | | | | | | |
|  |  | RAFL09-11-D17 | At3g12780 / phosphoglycerate kinase -related | |  |  |  |  |  | | --- | --- | --- | --- | --- | |  |  |  |  |  | | RAFL09-11-D17 ,At3g12780  phosphoglycerate kinase, putative similar to SP|P41758 Phosphoglycerate kinase, chloroplast precursor (EC 2.7.2.3) {Chlamydomonas reinhardtii}; contains Pfam profile PF00162: phosphoglycerate kinase | | | | | | |
|  |  | RAFL02-08-J05 | At3g17390 / s-adenosylmethionine synthetase -related | |  |  |  |  |  | | --- | --- | --- | --- | --- | |  |  |  |  |  | | RAFL02-08-J05 ,At3g17390  S-adenosylmethionine synthetase, putative similar to S-adenosylmethionine synthetase 2 (Methionine adenosyltransferase 2, AdoMet synthetase 2) [Catharanthus roseus] SWISS-PROT:Q96552 | | | | | | |
|  |  | RAFL06-16-H23 | At3g15030 / TCP family transcription factor, putative | |  |  |  |  |  | | --- | --- | --- | --- | --- | |  |  |  |  |  | | At3g15030 ,RAFL06-16-H23  TCP family transcription factor, putative similar to TCP3 GB:AAC24010 [Arabidopsis thaliana] | | | | | | |
|  |  | RAFL08-15-E10 | At3g14420 / glycolate oxidase -related | |  |  |  |  |  | | --- | --- | --- | --- | --- | |  |  |  |  |  | | At3g14420 ,RAFL08-15-E10  (S)-2-hydroxy-acid oxidase, peroxisomal, putative / glycolate oxidase, putative / short chain alpha-hydroxy acid oxidase, putative similar to (S)-2-hydroxy-acid oxidase, peroxisomal (Glycolate oxidase, GOX) (Short chain alpha-hydroxy acid oxidase) [Spinacia oleracea] SWISS-PROT:P05414 | | | | | | |
|  |  | RAFL05-01-K06 | At3g11170 / omega-3 fatty acid desaturase, chloroplast precursor (FAD7) | |  |  |  |  |  | | --- | --- | --- | --- | --- | |  |  |  |  |  | | RAFL05-01-K06 ,At3g11170  omega-3 fatty acid desaturase, chloroplast (FAD7) (FADD) identical to omega-3 fatty acid desaturase, chloroplast precursor SP:P46310 [Arabidopsis thaliana (Mouse-ear cress)]; identical to Pfam profile PF00487: Fatty acid desaturase; identical to cDNA plastid fatty acid desaturase GI:809491 | | | | | | |
|  |  | RAFL05-12-C12 | At3g17390 / s-adenosylmethionine synthetase -related | |  |  |  |  |  | | --- | --- | --- | --- | --- | |  |  |  |  |  | | RAFL05-12-C12 ,At3g17390  S-adenosylmethionine synthetase, putative similar to S-adenosylmethionine synthetase 2 (Methionine adenosyltransferase 2, AdoMet synthetase 2) [Catharanthus roseus] SWISS-PROT:Q96552 | | | | | | |
|  |  | RAFL08-11-A20 | At3g15850 / fatty acid desaturase family protein | |  |  |  |  |  | | --- | --- | --- | --- | --- | |  |  |  |  |  | | RAFL08-11-A20 ,At3g15850  fatty acid desaturase family protein similar to delta 9 acyl-lipid desaturase (ADS1) GI:2970034 from [Arabidopsis thaliana] | | | | | | |
|  |  | RAFL09-09-J21 | At3g11700 / expressed protein | |  |  |  |  |  | | --- | --- | --- | --- | --- | |  |  |  |  |  | | RAFL09-09-J21 ,At3g11700  beta-Ig-H3 domain-containing protein / fasciclin domain-containing protein contains Pfam profile PF02469: Fasciclin domain | | | | | | |
|  |  | RAFL09-13-P20 | At3g14420 / glycolate oxidase -related | |  |  |  |  |  | | --- | --- | --- | --- | --- | |  |  |  |  |  | | At3g14420 ,RAFL09-13-P20  (S)-2-hydroxy-acid oxidase, peroxisomal, putative / glycolate oxidase, putative / short chain alpha-hydroxy acid oxidase, putative similar to (S)-2-hydroxy-acid oxidase, peroxisomal (Glycolate oxidase, GOX) (Short chain alpha-hydroxy acid oxidase) [Spinacia oleracea] SWISS-PROT:P05414 | | | | | | |
|  |  | RAFL05-04-C24 | At3g13120 / chloroplast 30S ribosomal protein S10, putative | |  |  |  |  |  | | --- | --- | --- | --- | --- | |  |  |  |  |  | | At3g13120 ,RAFL05-04-C24  30S ribosomal protein S10, chloroplast, putative similar to 30S ribosomal protein S10 GB:P02364 [Escherichia coli] (est matches suggest the N-terminal extension) | | | | | | |
|  |  | RAFL04-09-O21 | At3g16370 / GDSL-motif lipase/hydrolase protein | |  |  |  |  |  | | --- | --- | --- | --- | --- | |  |  |  |  |  | | RAFL04-09-O21 ,At3g16370  GDSL-motif lipase/hydrolase family protein similar to family II lipases EXL3 GI:15054386, EXL1 GI:15054382, EXL2 GI:15054384 from [Arabidopsis thaliana]; contains Pfam profile: PF00657 Lipase Acylhydrolase with GDSL-like motif | | | | | | |
|  |  | RAFL04-20-A09 | At3g16370 / GDSL-motif lipase/hydrolase protein | |  |  |  |  |  | | --- | --- | --- | --- | --- | |  |  |  |  |  | | RAFL04-20-A09 ,At3g16370  GDSL-motif lipase/hydrolase family protein similar to family II lipases EXL3 GI:15054386, EXL1 GI:15054382, EXL2 GI:15054384 from [Arabidopsis thaliana]; contains Pfam profile: PF00657 Lipase Acylhydrolase with GDSL-like motif | | | | | | |
| 5\_26100001\_26400000 | | |  |  | A | B | C | D | P | P' | N |
|  | Cluster:9-0 | |  |  | 2 | 30 | 19 | 4612 | 0.008833496 | 0.12366895 | 14 |
|  |  | RAFL05-03-I09 | At5g66400 / dehydrin RAB18-related protein (sp P30185) | |  |  |  |  |  | | --- | --- | --- | --- | --- | |  |  |  |  |  | | RAFL05-03-I09 ,At5g66400  dehydrin (RAB18) nearly identical to SP|P30185 Dehydrin Rab18 {Arabidopsis thaliana} | | | | | | |
|  |  | RAFL08-10-E21 | At5g66780 / expressed protein | |  |  |  |  |  | | --- | --- | --- | --- | --- | |  |  |  |  |  | | At5g66780 ,RAFL08-10-E21  expressed protein | | | | | | |
|  | Cluster:4-0 | |  |  | 4 | 236 | 17 | 4406 | 0.020494834 | 0.28692767 | 14 |
|  |  | RAFL04-20-L08 | At5g66040 / senescence-associated protein | |  |  |  |  |  | | --- | --- | --- | --- | --- | |  |  |  |  |  | | RAFL04-20-L08 ,At5g66040  senescence-associated family protein almost identical to ketoconazole resistant protein GI:928938 from [Arabidopsis thaliana] full-length cDNA: Ceres:101608. | | | | | | |
|  |  | RAFL08-08-F13 | At5g66055 / expressed protein | |  |  |  |  |  | | --- | --- | --- | --- | --- | |  |  |  |  |  | | At5g66055 ,RAFL08-08-F13  ankyrin repeat protein / AKRP (AKR) identical to ankyrin repeat protein (AKRP) [Arabidopsis thaliana] SWISS-PROT:Q05753 | | | | | | |
|  |  | RAFL06-10-I09 | At5g66290 / hypothetical protein | |  |  |  |  |  | | --- | --- | --- | --- | --- | |  |  |  |  |  | | RAFL06-10-I09 ,At5g66290  expressed protein | | | | | | |
|  |  | RAFL04-20-B17 | At5g66590 / pathogenesis-related protein family | |  |  |  |  |  | | --- | --- | --- | --- | --- | |  |  |  |  |  | | RAFL04-20-B17 ,At5g66590  allergen V5/Tpx-1-related family protein contains similarity to SP|Q41495 STS14 protein precursor {Solanum tuberosum}; contains Pfam profile PF00188: SCP-like extracellular protein | | | | | | |
| 5\_18120001\_18150000 | | |  |  | A | B | C | D | P | P' | N |
|  | Cluster:5-0 | |  |  | 2 | 75 | 0 | 4586 | 2.6919477E-4 | 2.6919477E-4 | 1 |
|  |  | RAFL07-11-O11 | At5g45430 / protein kinase, putative | |  |  |  |  |  | | --- | --- | --- | --- | --- | |  |  |  |  |  | | At5g45430 ,RAFL07-11-O11  protein kinase, putative contains similarity to male germ cell-associated kinase [Homo sapiens] gi|23268497|gb|AAN16405 | | | | | | |
|  |  | RAFL07-18-J19 | At5g45500 / expressed protein | |  |  |  |  |  | | --- | --- | --- | --- | --- | |  |  |  |  |  | | At5g45500 ,RAFL07-18-J19  expressed protein weak similarity to resistance complex protein I2C-2 [Lycopersicon esculentum] GI:2258317 | | | | | | |
| 5\_20460001\_20490000 | | |  |  | A | B | C | D | P | P' | N |
|  | Cluster:9-1 | |  |  | 2 | 94 | 0 | 4567 | 4.1952432E-4 | 4.1952432E-4 | 1 |
|  |  | RAFL09-15-D15 | At5g51070 / ATP-dependent Clp protease ATP-binding subunit (ClpD), ERD1 protein precursor | |  |  |  |  |  | | --- | --- | --- | --- | --- | |  |  |  |  |  | | At5g51070 ,RAFL09-15-D15  ATP-dependent Clp protease ATP-binding subunit (ClpD), (ERD1) SAG15/ERD1; identical to ERD1 protein GI:497629, SP:P42762 from [Arabidopsis thaliana]; contains Pfam profile PF02861: Clp amino terminal domain | | | | | | |
|  |  | RAFL05-05-I08 | At5g51070 / ATP-dependent Clp protease ATP-binding subunit (ClpD), ERD1 protein precursor | |  |  |  |  |  | | --- | --- | --- | --- | --- | |  |  |  |  |  | | At5g51070 ,RAFL05-05-I08  ATP-dependent Clp protease ATP-binding subunit (ClpD), (ERD1) SAG15/ERD1; identical to ERD1 protein GI:497629, SP:P42762 from [Arabidopsis thaliana]; contains Pfam profile PF02861: Clp amino terminal domain | | | | | | |
| 4\_11970001\_12000000 | | |  |  | A | B | C | D | P | P' | N |
|  | Cluster:6-0 | |  |  | 1 | 139 | 0 | 4523 | 0.03002359 | 0.03002359 | 1 |
|  |  | RAFL04-10-E08 | At4g25500 / arginine/serine-rich splicing factor RSp40 | |  |  |  |  |  | | --- | --- | --- | --- | --- | |  |  |  |  |  | | At4g25500 ,RAFL04-10-E08  arginine/serine-rich splicing factor RSP40 (RSP40) identical to SP|P92965 Arginine/serine-rich splicing factor RSP40 {Arabidopsis thaliana} | | | | | | |
| 1\_27900001\_27930000 | | |  |  | A | B | C | D | P | P' | N |
|  | Cluster:2-2 | |  |  | 1 | 52 | 1 | 4609 | 0.022605369 | 0.045210738 | 2 |
|  |  | RAFL04-15-A17 | At1g75350 / chloroplast 50S ribosomal protein L31 -related | |  |  |  |  |  | | --- | --- | --- | --- | --- | |  |  |  |  |  | | RAFL04-15-A17 ,At1g75350  ribosomal protein L31 family protein similar to SP:O46917 from [Guillardia theta] | | | | | | |
| 3\_17340001\_17370000 | | |  |  | A | B | C | D | P | P' | N |
|  | Cluster:0-1 | |  |  | 1 | 105 | 1 | 4556 | 0.044952307 | 0.089904614 | 2 |
|  |  | RAFL04-15-K17 | At3g47070 / expressed protein | |  |  |  |  |  | | --- | --- | --- | --- | --- | |  |  |  |  |  | | At3g47070 ,RAFL04-15-K17  expressed protein | | | | | | |
| 1\_23280001\_23310000 | | |  |  | A | B | C | D | P | P' | N |
|  | Cluster:9-1 | |  |  | 1 | 95 | 0 | 4567 | 0.020587604 | 0.020587604 | 1 |
|  |  | RAFL05-08-D17 | At1g63720 / expressed protein | |  |  |  |  |  | | --- | --- | --- | --- | --- | |  |  |  |  |  | | RAFL05-08-D17 ,At1g63720  expressed protein similar to putative protein GB:CAA18164 [Arabidopsis thaliana] | | | | | | |
| 5\_14010001\_14040000 | | |  |  | A | B | C | D | P | P' | N |
|  | Cluster:1-1 | |  |  | 1 | 104 | 0 | 4558 | 0.022517692 | 0.022517692 | 1 |
|  |  | RAFL07-14-E07 | At5g36290 / transmembrane protein FT27/PFT27-related | |  |  |  |  |  | | --- | --- | --- | --- | --- | |  |  |  |  |  | | At5g36290 ,RAFL07-14-E07  expressed protein contains Pfam profile PF01169: Uncharacterized protein family UPF0016 | | | | | | |
| 5\_5070001\_5100000 | | |  |  | A | B | C | D | P | P' | N |
|  | Cluster:8-2 | |  |  | 2 | 60 | 0 | 4601 | 1.7397379E-4 | 1.7397379E-4 | 1 |
|  |  | RAFL05-02-G21 | At5g15650 / reversibly glycosylated polypeptide-3 | |  |  |  |  |  | | --- | --- | --- | --- | --- | |  |  |  |  |  | | At5g15650 ,RAFL05-02-G21  reversibly glycosylated polypeptide-2 (RGP2) identical to reversibly glycosylated polypeptide-2 [Arabidopsis thaliana] GI:2317731 | | | | | | |
|  |  | RAFL11-05-M03 | At5g15650 / reversibly glycosylated polypeptide-3 | |  |  |  |  |  | | --- | --- | --- | --- | --- | |  |  |  |  |  | | At5g15650 ,RAFL11-05-M03  reversibly glycosylated polypeptide-2 (RGP2) identical to reversibly glycosylated polypeptide-2 [Arabidopsis thaliana] GI:2317731 | | | | | | |
| 1\_28620001\_28650000 | | |  |  | A | B | C | D | P | P' | N |
|  | Cluster:9-1 | |  |  | 1 | 95 | 0 | 4567 | 0.020587604 | 0.020587604 | 1 |
|  |  | RAFL07-16-P10 | At1g77120 / alcohol dehydrogenase (ADH) | |  |  |  |  |  | | --- | --- | --- | --- | --- | |  |  |  |  |  | | RAFL07-16-P10 ,At1g77120  alcohol dehydrogenase (ADH) identical to alcohol dehydrogenase GI:469467 from (Arabidopsis thaliana) | | | | | | |
| 2\_15720001\_15750000 | | |  |  | A | B | C | D | P | P' | N |
|  | Cluster:0-1 | |  |  | 1 | 105 | 0 | 4557 | 0.022732146 | 0.022732146 | 1 |
|  |  | RAFL04-16-P06 | At2g37660 / expressed protein | |  |  |  |  |  | | --- | --- | --- | --- | --- | |  |  |  |  |  | | At2g37660 ,RAFL04-16-P06  expressed protein | | | | | | |
| 1\_28860001\_28890000 | | |  |  | A | B | C | D | P | P' | N |
|  | Cluster:3-2 | |  |  | 1 | 36 | 0 | 4626 | 0.007934806 | 0.007934806 | 1 |
|  |  | RAFL09-07-M15 | At1g77760 / nitrate reductase 1 (NR1) | |  |  |  |  |  | | --- | --- | --- | --- | --- | |  |  |  |  |  | | RAFL09-07-M15 ,At1g77760  nitrate reductase 1 (NR1) identical to SP|P11832 Nitrate reductase 1 (formerly EC 1.6.6.1) (NR1){Arabidopsis thaliana} | | | | | | |
| 5\_4200001\_4230000 | | |  |  | A | B | C | D | P | P' | N |
|  | Cluster:8-1 | |  |  | 1 | 161 | 0 | 4501 | 0.034741584 | 0.034741584 | 1 |
|  |  | RAFL05-07-H19 | At5g13190 / expressed protein | |  |  |  |  |  | | --- | --- | --- | --- | --- | |  |  |  |  |  | | RAFL05-07-H19 ,At5g13190  expressed protein | | | | | | |
| 4\_15510001\_15540000 | | |  |  | A | B | C | D | P | P' | N |
|  | Cluster:9-2 | |  |  | 2 | 65 | 5 | 4591 | 0.004077173 | 0.024463037 | 6 |
|  |  | RAFL08-11-N01 | At4g34710 / arginine decarboxylase SPE2 | |  |  |  |  |  | | --- | --- | --- | --- | --- | |  |  |  |  |  | | At4g34710 ,RAFL08-11-N01  arginine decarboxylase 2 (SPE2) identical to SP|O23141 Arginine decarboxylase 2 (EC 4.1.1.19) (ARGDC 2) (ADC 2) (ADC-N) {Arabidopsis thaliana} | | | | | | |
|  |  | RAFL09-13-D07 | At4g34710 / arginine decarboxylase SPE2 | |  |  |  |  |  | | --- | --- | --- | --- | --- | |  |  |  |  |  | | RAFL09-13-D07 ,At4g34710  arginine decarboxylase 2 (SPE2) identical to SP|O23141 Arginine decarboxylase 2 (EC 4.1.1.19) (ARGDC 2) (ADC 2) (ADC-N) {Arabidopsis thaliana} | | | | | | |
| 4\_6000001\_9000000 | | |  |  | A | B | C | D | P | P' | N |
|  | Cluster:1-0 | |  |  | 9 | 140 | 86 | 4428 | 0.0030741743 | 0.079928525 | 26 |
|  |  | RAFL11-05-E03 | At4g17600 / Lil3 protein | |  |  |  |  |  | | --- | --- | --- | --- | --- | |  |  |  |  |  | | RAFL11-05-E03 ,At4g17600  lil3 protein identical to Lil3 protein [Arabidopsis thaliana] gi|4741966|gb|AAD28780 | | | | | | |
|  |  | RAFL11-11-M03 | At4g17600 / Lil3 protein | |  |  |  |  |  | | --- | --- | --- | --- | --- | |  |  |  |  |  | | RAFL11-11-M03 ,At4g17600  lil3 protein identical to Lil3 protein [Arabidopsis thaliana] gi|4741966|gb|AAD28780 | | | | | | |
|  |  | RAFL06-10-B16 | At4g17560 / ribosomal protein L19, putative | |  |  |  |  |  | | --- | --- | --- | --- | --- | |  |  |  |  |  | | At4g17560 ,RAFL06-10-B16  ribosomal protein L19 family protein similar to plastid ribosomal protein L19 precursor [Spinacia oleracea] gi|7582403|gb|AAF64312 | | | | | | |
|  |  | RAFL05-17-O20 | At4g12030 / bile acid:sodium symporter family | |  |  |  |  |  | | --- | --- | --- | --- | --- | |  |  |  |  |  | | RAFL05-17-O20 ,At4g12030  bile acid:sodium symporter family protein low similarity to SP|Q12908 Ileal sodium/bile acid cotransporter {Homo sapiens}; contains Pfam profile PF01758: Sodium Bile acid symporter family | | | | | | |
|  |  | RAFL04-17-B18 | At4g13840 / transferase family | |  |  |  |  |  | | --- | --- | --- | --- | --- | |  |  |  |  |  | | RAFL04-17-B18 ,At4g13840  transferase family protein low similarity to acetyl-CoA:benzylalcohol acetyltranferase [Clarkia concinna] GI:6166328; contains Pfam profile PF02458: Transferase family | | | | | | |
|  |  | RAFL06-12-P08 | At4g18030 / dehydration-induced protein family | |  |  |  |  |  | | --- | --- | --- | --- | --- | |  |  |  |  |  | | At4g18030 ,RAFL06-12-P08  dehydration-responsive family protein similar to early-responsive to dehydration stress ERD3 protein [Arabidopsis thaliana] GI:15320410; contains Pfam profile PF03141: Putative methyltransferase | | | | | | |
|  |  | RAFL09-09-D17 | At4g14960 / tubulin alpha-6 chain (TUA6) | |  |  |  |  |  | | --- | --- | --- | --- | --- | |  |  |  |  |  | | At4g14960 ,RAFL09-09-D17  tubulin alpha-6 chain (TUA6) nearly identical to SP|P29511 Tubulin alpha-6 chain {Arabidopsis thaliana} | | | | | | |
|  |  | RAFL09-06-N13 | At4g14960 / tubulin alpha-6 chain (TUA6) | |  |  |  |  |  | | --- | --- | --- | --- | --- | |  |  |  |  |  | | At4g14960 ,RAFL09-06-N13  tubulin alpha-6 chain (TUA6) nearly identical to SP|P29511 Tubulin alpha-6 chain {Arabidopsis thaliana} | | | | | | |
|  |  | RAFL11-07-D18 | At4g16370 / isp4 like protein | |  |  |  |  |  | | --- | --- | --- | --- | --- | |  |  |  |  |  | | At4g16370 ,RAFL11-07-D18  oligopeptide transporter OPT family protein similar to oligopeptide transporter Opt1p [Candida albicans] GI:2367386; contains Pfam profile PF03169: OPT oligopeptide transporter protein | | | | | | |
|  | Cluster:1-2 | |  |  | 9 | 165 | 86 | 4403 | 0.00847757 | 0.22041681 | 26 |
|  |  | RAFL04-16-C09 | At4g17520 / nuclear RNA binding protein, putative | |  |  |  |  |  | | --- | --- | --- | --- | --- | |  |  |  |  |  | | RAFL04-16-C09 ,At4g17520  nuclear RNA-binding protein, putative similar to nuclear RNA binding protein GI:6492264 from [Arabidopsis thaliana] | | | | | | |
|  |  | RAFL09-11-K06 | At4g13930 / hydroxymethyltransferase | |  |  |  |  |  | | --- | --- | --- | --- | --- | |  |  |  |  |  | | At4g13930 ,RAFL09-11-K06  glycine hydroxymethyltransferase, putative / serine hydroxymethyltransferase, putative / serine/threonine aldolase, putative similar to serine hydroxymethyltransferase [Chlamydomonas reinhardtii] GI:17066746; contains Pfam profile PF00464: serine hydroxymethyltransferase | | | | | | |
|  |  | RAFL09-18-P13 | At4g14040 / selenium-binding protein like | |  |  |  |  |  | | --- | --- | --- | --- | --- | |  |  |  |  |  | | At4g14040 ,RAFL09-18-P13  selenium-binding protein, putative contains Pfam profile PF05694: 56kDa selenium binding protein (SBP56); similar to Putative selenium-binding protein (Swiss-Prot:O23264) [Arabidopsis thaliana]; similar to selenium binding protein (GI:15485232) [Arabidopsis thaliana] | | | | | | |
|  |  | RAFL11-07-F10 | At4g14040 / selenium-binding protein like | |  |  |  |  |  | | --- | --- | --- | --- | --- | |  |  |  |  |  | | At4g14040 ,RAFL11-07-F10  selenium-binding protein, putative contains Pfam profile PF05694: 56kDa selenium binding protein (SBP56); similar to Putative selenium-binding protein (Swiss-Prot:O23264) [Arabidopsis thaliana]; similar to selenium binding protein (GI:15485232) [Arabidopsis thaliana] | | | | | | |
|  |  | RAFL09-12-K14 | At4g14040 / selenium-binding protein like | |  |  |  |  |  | | --- | --- | --- | --- | --- | |  |  |  |  |  | | At4g14040 ,RAFL09-12-K14  selenium-binding protein, putative contains Pfam profile PF05694: 56kDa selenium binding protein (SBP56); similar to Putative selenium-binding protein (Swiss-Prot:O23264) [Arabidopsis thaliana]; similar to selenium binding protein (GI:15485232) [Arabidopsis thaliana] | | | | | | |
|  |  | RAFL09-14-M21 | At4g16370 / isp4 like protein | |  |  |  |  |  | | --- | --- | --- | --- | --- | |  |  |  |  |  | | At4g16370 ,RAFL09-14-M21  oligopeptide transporter OPT family protein similar to oligopeptide transporter Opt1p [Candida albicans] GI:2367386; contains Pfam profile PF03169: OPT oligopeptide transporter protein | | | | | | |
|  |  | RAFL04-09-I24 | At4g15540 / nodulin MtN21 - related | |  |  |  |  |  | | --- | --- | --- | --- | --- | |  |  |  |  |  | | At4g15540 ,RAFL04-09-I24  nodulin-related low similarity to MtN21 [Medicago truncatula] GI:2598575 | | | | | | |
|  |  | RAFL05-01-M18 | At4g13510 / ammonium transport protein (AMT1) | |  |  |  |  |  | | --- | --- | --- | --- | --- | |  |  |  |  |  | | RAFL05-01-M18 ,At4g13510  ammonium transporter 1, member 1 (AMT1.1) identical to SP|P54144 High affinity ammonium transporter (AtAMT1;1) {Arabidopsis thaliana} | | | | | | |
|  |  | RAFL09-16-C12 | At4g14040 / selenium-binding protein like | |  |  |  |  |  | | --- | --- | --- | --- | --- | |  |  |  |  |  | | At4g14040 ,RAFL09-16-C12  selenium-binding protein, putative contains Pfam profile PF05694: 56kDa selenium binding protein (SBP56); similar to Putative selenium-binding protein (Swiss-Prot:O23264) [Arabidopsis thaliana]; similar to selenium binding protein (GI:15485232) [Arabidopsis thaliana] | | | | | | |
| 3\_5940001\_5970000 | | |  |  | A | B | C | D | P | P' | N |
|  | Cluster:1-0 | |  |  | 2 | 147 | 0 | 4514 | 0.0010144025 | 0.0010144025 | 1 |
|  |  | RAFL02-08-J05 | At3g17390 / s-adenosylmethionine synthetase -related | |  |  |  |  |  | | --- | --- | --- | --- | --- | |  |  |  |  |  | | RAFL02-08-J05 ,At3g17390  S-adenosylmethionine synthetase, putative similar to S-adenosylmethionine synthetase 2 (Methionine adenosyltransferase 2, AdoMet synthetase 2) [Catharanthus roseus] SWISS-PROT:Q96552 | | | | | | |
|  |  | RAFL05-12-C12 | At3g17390 / s-adenosylmethionine synthetase -related | |  |  |  |  |  | | --- | --- | --- | --- | --- | |  |  |  |  |  | | RAFL05-12-C12 ,At3g17390  S-adenosylmethionine synthetase, putative similar to S-adenosylmethionine synthetase 2 (Methionine adenosyltransferase 2, AdoMet synthetase 2) [Catharanthus roseus] SWISS-PROT:Q96552 | | | | | | |
| 4\_14850001\_14880000 | | |  |  | A | B | C | D | P | P' | N |
|  | Cluster:8-0 | |  |  | 1 | 108 | 0 | 4554 | 0.02337551 | 0.02337551 | 1 |
|  |  | RAFL05-09-J06 | At4g32940 / gamma-VPE (vacuolar processing enzyme) | |  |  |  |  |  | | --- | --- | --- | --- | --- | |  |  |  |  |  | | RAFL05-09-J06 ,At4g32940  vacuolar processing enzyme gamma / gamma-VPE nearly identical to SP|Q39119 Vacuolar processing enzyme, gamma-isozyme precursor (EC 3.4.22.-) (Gamma-VPE) {Arabidopsis thaliana} | | | | | | |
| 5\_360001\_390000 | | |  |  | A | B | C | D | P | P' | N |
|  | Cluster:10-1 | |  |  | 1 | 42 | 0 | 4620 | 0.009221531 | 0.009221531 | 1 |
|  |  | RAFL06-12-H12 | At5g02020 / expressed protein | |  |  |  |  |  | | --- | --- | --- | --- | --- | |  |  |  |  |  | | At5g02020 ,RAFL06-12-H12  expressed protein | | | | | | |
| 4\_4590001\_4620000 | | |  |  | A | B | C | D | P | P' | N |
|  | Cluster:1-0 | |  |  | 1 | 148 | 0 | 4514 | 0.031953678 | 0.031953678 | 1 |
|  |  | RAFL05-18-H22 | At4g08870 / arginase -related | |  |  |  |  |  | | --- | --- | --- | --- | --- | |  |  |  |  |  | | At4g08870 ,RAFL05-18-H22  arginase, putative similar to Swiss-Prot:P46637 arginase (EC 3.5.3.1) [Arabidopsis thaliana] | | | | | | |
| 5\_7470001\_7500000 | | |  |  | A | B | C | D | P | P' | N |
|  | Cluster:0-1 | |  |  | 1 | 105 | 0 | 4557 | 0.022732146 | 0.022732146 | 1 |
|  |  | RAFL04-09-L14 | At5g22580 / expressed protein | |  |  |  |  |  | | --- | --- | --- | --- | --- | |  |  |  |  |  | | RAFL04-09-L14 ,At5g22580  expressed protein | | | | | | |
| 5\_25590001\_25620000 | | |  |  | A | B | C | D | P | P' | N |
|  | Cluster:7-2 | |  |  | 1 | 63 | 2 | 4597 | 0.040621255 | 0.12186376 | 3 |
|  |  | RAFL05-09-M07 | At5g64740 / cellulose synthase, catalytic subunit, putative | |  |  |  |  |  | | --- | --- | --- | --- | --- | |  |  |  |  |  | | RAFL05-09-M07 ,At5g64740  cellulose synthase, catalytic subunit, putative similar to gi:2827141 cellulose synthase catalytic subunit (Ath-A), Arabidopsis thaliana | | | | | | |
| 2\_1710001\_1740000 | | |  |  | A | B | C | D | P | P' | N |
|  | Cluster:6-0 | |  |  | 1 | 139 | 0 | 4523 | 0.03002359 | 0.03002359 | 1 |
|  |  | RAFL06-09-E11 | At2g04900 / expressed protein | |  |  |  |  |  | | --- | --- | --- | --- | --- | |  |  |  |  |  | | At2g04900 ,RAFL06-09-E11  expressed protein | | | | | | |
| 4\_10380001\_10410000 | | |  |  | A | B | C | D | P | P' | N |
|  | Cluster:9-1 | |  |  | 1 | 95 | 0 | 4567 | 0.020587604 | 0.020587604 | 1 |
|  |  | RAFL09-07-E08 | At4g21440 / myb family protein | |  |  |  |  |  | | --- | --- | --- | --- | --- | |  |  |  |  |  | | At4g21440 ,RAFL09-07-E08  myb family transcription factor (MYB102) contains Pfam profile: PF00249 myb-like DNA-binding domain | | | | | | |
| 1\_21690001\_21720000 | | |  |  | A | B | C | D | P | P' | N |
|  | Cluster:6-0 | |  |  | 1 | 139 | 0 | 4523 | 0.03002359 | 0.03002359 | 1 |
|  |  | RAFL09-17-A17 | At1g59900 / pyruvate dehydrogenase e1 alpha subunit -related | |  |  |  |  |  | | --- | --- | --- | --- | --- | |  |  |  |  |  | | At1g59900 ,RAFL09-17-A17  pyruvate dehydrogenase E1 component alpha subunit, mitochondrial (PDHE1-A) identical to SP|P52901 Pyruvate dehydrogenase E1 component alpha subunit, mitochondrial precursor (EC 1.2.4.1) (PDHE1-A) {Arabidopsis thaliana} | | | | | | |
| 3\_15780001\_15810000 | | |  |  | A | B | C | D | P | P' | N |
|  | Cluster:3-0 | |  |  | 1 | 232 | 0 | 4430 | 0.049967833 | 0.049967833 | 1 |
|  |  | RAFL02-10-H10 | At3g43980 / 40S ribosomal protein S29 (RPS29A) | |  |  |  |  |  | | --- | --- | --- | --- | --- | |  |  |  |  |  | | At3g43980 ,RAFL02-10-H10  40S ribosomal protein S29 (RPS29A) ribosomal protein S29, rat, PIR:S30298 | | | | | | |
| 3\_17460001\_17490000 | | |  |  | A | B | C | D | P | P' | N |
|  | Cluster:0-2 | |  |  | 1 | 78 | 1 | 4583 | 0.03360031 | 0.06720062 | 2 |
|  |  | RAFL04-17-P09 | At3g47430 / expressed protein | |  |  |  |  |  | | --- | --- | --- | --- | --- | |  |  |  |  |  | | At3g47430 ,RAFL04-17-P09  peroxisomal biogenesis factor 11 family protein / PEX11 family protein contains Pfam PF05648: Peroxisomal biogenesis factor 11 (PEX11) | | | | | | |
| 1\_3060001\_3090000 | | |  |  | A | B | C | D | P | P' | N |
|  | Cluster:9-0 | |  |  | 2 | 30 | 1 | 4630 | 1.3631E-4 | 2.7262E-4 | 2 |
|  |  | RAFL06-15-H16 | At1g09500 / cinnamyl-alcohol dehydrogenase (CAD) family | |  |  |  |  |  | | --- | --- | --- | --- | --- | |  |  |  |  |  | | At1g09500 ,RAFL06-15-H16  cinnamyl-alcohol dehydrogenase family / CAD family similar to cinnamyl alcohol dehydrogenase, Eucalyptus gunnii [gi:1143445], CPRD14 protein, Vigna unguiculata [gi:1854445] | | | | | | |
|  |  | RAFL05-18-A06 | At1g09500 / cinnamyl-alcohol dehydrogenase (CAD) family | |  |  |  |  |  | | --- | --- | --- | --- | --- | |  |  |  |  |  | | At1g09500 ,RAFL05-18-A06  cinnamyl-alcohol dehydrogenase family / CAD family similar to cinnamyl alcohol dehydrogenase, Eucalyptus gunnii [gi:1143445], CPRD14 protein, Vigna unguiculata [gi:1854445] | | | | | | |
| 3\_4350001\_4380000 | | |  |  | A | B | C | D | P | P' | N |
|  | Cluster:6-1 | |  |  | 2 | 313 | 1 | 4347 | 0.013038642 | 0.026077284 | 2 |
|  |  | RAFL05-05-P01 | At3g13410 / expressed protein | |  |  |  |  |  | | --- | --- | --- | --- | --- | |  |  |  |  |  | | At3g13410 ,RAFL05-05-P01  expressed protein | | | | | | |
|  |  | RAFL05-10-K07 | At3g13445 / transcription initiation factor TFIID-1 (TATA sequence-binding protein 1) | |  |  |  |  |  | | --- | --- | --- | --- | --- | |  |  |  |  |  | | RAFL05-10-K07 ,At3g13445  transcription initiation factor IID-1 (TFIID-1) / TATA-box factor 1 / TATA sequence-binding protein 1 (TBP1) identical to Swiss-Prot:P28147 transcription initiation factor TFIID-1 (TATA-box factor 1)(TATA sequence-binding protein 1) (TBP-1) [Arabidopsis thaliana] | | | | | | |
| 4\_13470001\_13500000 | | |  |  | A | B | C | D | P | P' | N |
|  | Cluster:3-0 | |  |  | 1 | 232 | 0 | 4430 | 0.049967833 | 0.049967833 | 1 |
|  |  | RAFL05-02-E04 | At4g29590 / expressed protein | |  |  |  |  |  | | --- | --- | --- | --- | --- | |  |  |  |  |  | | At4g29590 ,RAFL05-02-E04  expressed protein | | | | | | |
| 2\_16050001\_16080000 | | |  |  | A | B | C | D | P | P' | N |
|  | Cluster:9-1 | |  |  | 1 | 95 | 1 | 4566 | 0.040755685 | 0.08151137 | 2 |
|  |  | RAFL06-07-J20 | At2g38530 / nonspecific lipid transfer protein 2 (LTP 2) | |  |  |  |  |  | | --- | --- | --- | --- | --- | |  |  |  |  |  | | RAFL06-07-J20 ,At2g38530  nonspecific lipid transfer protein 2 (LTP2) identical to nonspecific lipid-transfer protein 2 from Arabidopsis thaliana [SP|Q9S7I3]; contains Pfam protease inhibitor/seed storage/LTP family domain PF00234 | | | | | | |
| 3\_4710001\_4740000 | | |  |  | A | B | C | D | P | P' | N |
|  | Cluster:0-0 | |  |  | 1 | 35 | 0 | 4627 | 0.0077203517 | 0.0077203517 | 1 |
|  |  | RAFL09-06-D04 | At3g14210 / myrosinase-associated protein, putative | |  |  |  |  |  | | --- | --- | --- | --- | --- | |  |  |  |  |  | | RAFL09-06-D04 ,At3g14210  myrosinase-associated protein, putative similar to GB:CAA71238 from [Brassica napus]; contains Pfam profile:PF00657 Lipase/Acylhydrolase with GDSL-like motif | | | | | | |
| 1\_18450001\_18480000 | | |  |  | A | B | C | D | P | P' | N |
|  | Cluster:0-1 | |  |  | 1 | 105 | 0 | 4557 | 0.022732146 | 0.022732146 | 1 |
|  |  | RAFL06-07-I03 | At1g50900 / expressed protein | |  |  |  |  |  | | --- | --- | --- | --- | --- | |  |  |  |  |  | | At1g50900 ,RAFL06-07-I03  expressed protein | | | | | | |
| 3\_20700001\_20730000 | | |  |  | A | B | C | D | P | P' | N |
|  | Cluster:0-1 | |  |  | 1 | 105 | 1 | 4556 | 0.044952307 | 0.089904614 | 2 |
|  |  | RAFL05-12-O19 | At3g55800 / sedoheptulose-bisphosphatase precursor | |  |  |  |  |  | | --- | --- | --- | --- | --- | |  |  |  |  |  | | At3g55800 ,RAFL05-12-O19  sedoheptulose-1,7-bisphosphatase, chloroplast / sedoheptulose-bisphosphatase identical to SP|P46283 Sedoheptulose-1,7-bisphosphatase, chloroplast precursor (EC 3.1.3.37) (Sedoheptulose-bisphosphatase) (SBPASE) (SED(1,7)P2ASE) {Arabidopsis thaliana} | | | | | | |
| 5\_21420001\_21450000 | | |  |  | A | B | C | D | P | P' | N |
|  | Cluster:1-2 | |  |  | 1 | 173 | 0 | 4489 | 0.037315033 | 0.037315033 | 1 |
|  |  | RAFL04-18-D08 | At5g53490 / thylakoid lumenal 17.4 kD pentapeptide repeat family protein, chloroplast precursor | |  |  |  |  |  | | --- | --- | --- | --- | --- | |  |  |  |  |  | | At5g53490 ,RAFL04-18-D08  thylakoid lumenal 17.4 kDa protein, chloroplast identical to SP:P81760 Thylakoid lumenal 17.4 kDa protein, chloroplast precursor (P17.4) {Arabidopsis thaliana} | | | | | | |
| 1\_10710001\_10740000 | | |  |  | A | B | C | D | P | P' | N |
|  | Cluster:7-2 | |  |  | 2 | 62 | 2 | 4597 | 0.0010932032 | 0.0032796096 | 3 |
|  |  | RAFL04-12-K17 | At1g30360 / ERD4 protein | |  |  |  |  |  | | --- | --- | --- | --- | --- | |  |  |  |  |  | | At1g30360 ,RAFL04-12-K17  early-responsive to dehydration stress protein (ERD4) nearly identical to ERD4 protein (early-responsive to dehydration stress) [Arabidopsis thaliana] GI:15375406; contains Pfam profile PF02714: Domain of unknown function DUF221 | | | | | | |
|  |  | RAFL11-06-I11 | At1g30360 / ERD4 protein | |  |  |  |  |  | | --- | --- | --- | --- | --- | |  |  |  |  |  | | At1g30360 ,RAFL11-06-I11  early-responsive to dehydration stress protein (ERD4) nearly identical to ERD4 protein (early-responsive to dehydration stress) [Arabidopsis thaliana] GI:15375406; contains Pfam profile PF02714: Domain of unknown function DUF221 | | | | | | |
| 5\_7830001\_7860000 | | |  |  | A | B | C | D | P | P' | N |
|  | Cluster:9-2 | |  |  | 1 | 66 | 1 | 4595 | 0.028533451 | 0.057066903 | 2 |
|  |  | RAFL05-09-I02 | At5g23340 / expressed protein | |  |  |  |  |  | | --- | --- | --- | --- | --- | |  |  |  |  |  | | At5g23340 ,RAFL05-09-I02  expressed protein | | | | | | |
| 5\_16590001\_16620000 | | |  |  | A | B | C | D | P | P' | N |
|  | Cluster:5-0 | |  |  | 1 | 76 | 1 | 4585 | 0.032756753 | 0.06551351 | 2 |
|  |  | RAFL05-04-D03 | At5g42270 / FtsH protease, putative | |  |  |  |  |  | | --- | --- | --- | --- | --- | |  |  |  |  |  | | RAFL05-04-D03 ,At5g42270  FtsH protease, putative similar to FtsH protease GI:13183728 from [Medicago sativa] | | | | | | |
| 2\_1800001\_2100000 | | |  |  | A | B | C | D | P | P' | N |
|  | Cluster:5-0 | |  |  | 2 | 75 | 6 | 4580 | 0.00706652 | 0.04239912 | 6 |
|  |  | RAFL04-14-C14 | At2g05100 / light-harvesting chlorophyll a/b binding protein | |  |  |  |  |  | | --- | --- | --- | --- | --- | |  |  |  |  |  | | RAFL04-14-C14 ,At2g05100  chlorophyll A-B binding protein / LHCII type II (LHCB2.1) (LHCB2.3) identical to Lhcb2 protein [Arabidopsis thaliana] GI:4741948, GI:4741944; contains Pfam profile PF00504: Chlorophyll A-B binding protein | | | | | | |
|  |  | RAFL05-01-K24 | At2g05620 / expressed protein | |  |  |  |  |  | | --- | --- | --- | --- | --- | |  |  |  |  |  | | RAFL05-01-K24 ,At2g05620  expressed protein | | | | | | |
| 5\_2910001\_2940000 | | |  |  | A | B | C | D | P | P' | N |
|  | Cluster:8-2 | |  |  | 1 | 61 | 0 | 4601 | 0.013296161 | 0.013296161 | 1 |
|  |  | RAFL06-07-I05 | At5g09440 / expressed protein | |  |  |  |  |  | | --- | --- | --- | --- | --- | |  |  |  |  |  | | At5g09440 ,RAFL06-07-I05  phosphate-responsive protein, putative similar to phi-1 (phosphate-induced gene) [Nicotiana tabacum] GI:3759184; contains Pfam profile PF04674: Phosphate-induced protein 1 conserved region | | | | | | |
| 2\_11100001\_11400000 | | |  |  | A | B | C | D | P | P' | N |
|  | Cluster:10-2 | |  |  | 2 | 107 | 11 | 4543 | 0.035702586 | 0.35702586 | 10 |
|  |  | RAFL08-12-H04 | At2g26690 / nitrate transporter -related | |  |  |  |  |  | | --- | --- | --- | --- | --- | |  |  |  |  |  | | RAFL08-12-H04 ,At2g26690  nitrate transporter (NTP2) identical to nitrate transporter (ntp2) [Arabidopsis thaliana] GI:4490321 | | | | | | |
|  |  | RAFL06-10-M04 | At2g26800 / hydroxymethylglutaryl-CoA lyase -related | |  |  |  |  |  | | --- | --- | --- | --- | --- | |  |  |  |  |  | | At2g26800 ,RAFL06-10-M04  hydroxymethylglutaryl-CoA lyase, putative / 3-hydroxy-3-methylglutarate-CoA lyase, putative / HMG-CoA lyase, putative similar to SP|P35915 Hydroxymethylglutaryl-CoA lyase (EC 4.1.3.4) (HMG-CoA lyase) {Gallus gallus}; contains Pfam profile PF00682: HMGL-like | | | | | | |
| 2\_13590001\_13620000 | | |  |  | A | B | C | D | P | P' | N |
|  | Cluster:8-0 | |  |  | 1 | 108 | 1 | 4553 | 0.0462095 | 0.092419 | 2 |
|  |  | RAFL09-09-E18 | At2g32090 / glyoxalase family protein (lactoylglutathione lyase family protein) | |  |  |  |  |  | | --- | --- | --- | --- | --- | |  |  |  |  |  | | RAFL09-09-E18 ,At2g32090  lactoylglutathione lyase family protein / glyoxalase I family protein contains glyoxalase family protein domain, Pfam:PF00903 | | | | | | |
| 1\_25080001\_25110000 | | |  |  | A | B | C | D | P | P' | N |
|  | Cluster:2-2 | |  |  | 1 | 52 | 2 | 4608 | 0.033719275 | 0.10115783 | 3 |
|  |  | RAFL05-04-I18 | At1g67850 / F12A21.2 | |  |  |  |  |  | | --- | --- | --- | --- | --- | |  |  |  |  |  | | RAFL05-04-I18 ,At1g67850  expressed protein contains Pfam profile PF05212: Protein of unknown function (DUF707) | | | | | | |
| 5\_630001\_660000 | | |  |  | A | B | C | D | P | P' | N |
|  | Cluster:2-0 | |  |  | 2 | 148 | 4 | 4509 | 0.014161414 | 0.056645654 | 4 |
|  |  | RAFL07-14-H10 | At5g02870 / 60S ribosomal protein L4/L1 (RPL4D) | |  |  |  |  |  | | --- | --- | --- | --- | --- | |  |  |  |  |  | | At5g02870 ,RAFL07-14-H10  60S ribosomal protein L4/L1 (RPL4D) 60S roibosomal protein L4, Arabidopsis thaliana, EMBL:CAA79104 | | | | | | |
|  |  | RAFL11-13-A04 | At5g02870 / 60S ribosomal protein L4/L1 (RPL4D) | |  |  |  |  |  | | --- | --- | --- | --- | --- | |  |  |  |  |  | | At5g02870 ,RAFL11-13-A04  60S ribosomal protein L4/L1 (RPL4D) 60S roibosomal protein L4, Arabidopsis thaliana, EMBL:CAA79104 | | | | | | |
|  | Cluster:2-1 | |  |  | 2 | 242 | 4 | 4415 | 0.0355681 | 0.1422724 | 4 |
|  |  | RAFL07-14-M14 | At5g02870 / 60S ribosomal protein L4/L1 (RPL4D) | |  |  |  |  |  | | --- | --- | --- | --- | --- | |  |  |  |  |  | | At5g02870 ,RAFL07-14-M14  60S ribosomal protein L4/L1 (RPL4D) 60S roibosomal protein L4, Arabidopsis thaliana, EMBL:CAA79104 | | | | | | |
|  |  | RAFL09-12-B05 | At5g02870 / 60S ribosomal protein L4/L1 (RPL4D) | |  |  |  |  |  | | --- | --- | --- | --- | --- | |  |  |  |  |  | | At5g02870 ,RAFL09-12-B05  60S ribosomal protein L4/L1 (RPL4D) 60S roibosomal protein L4, Arabidopsis thaliana, EMBL:CAA79104 | | | | | | |
| 5\_22200001\_22500000 | | |  |  | A | B | C | D | P | P' | N |
|  | Cluster:5-0 | |  |  | 5 | 72 | 7 | 4579 | 7.7957714E-7 | 6.236617E-6 | 8 |
|  |  | RAFL05-16-L15 | At5g56010 / heat shock protein, putative | |  |  |  |  |  | | --- | --- | --- | --- | --- | |  |  |  |  |  | | RAFL05-16-L15 ,At5g56010  heat shock protein, putative strong similarity to SP|P55737 Heat shock protein 81-2 (HSP81-2) {Arabidopsis thaliana}; contains Pfam profiles PF02518: ATPase, histidine kinase-, DNA gyrase B-, and HSP90-like domain protein, PF00183: Hsp90 protein | | | | | | |
|  |  | RAFL09-06-O18 | At5g56030 / heat shock protein 81-2 (HSP81-2) | |  |  |  |  |  | | --- | --- | --- | --- | --- | |  |  |  |  |  | | RAFL09-06-O18 ,At5g56030  heat shock protein 81-2 (HSP81-2) nearly identical to SP|P55737 Heat shock protein 81-2 (HSP81-2) {Arabidopsis thaliana} | | | | | | |
|  |  | RAFL07-13-H08 | At5g56010 / heat shock protein, putative | |  |  |  |  |  | | --- | --- | --- | --- | --- | |  |  |  |  |  | | RAFL07-13-H08 ,At5g56010  heat shock protein, putative strong similarity to SP|P55737 Heat shock protein 81-2 (HSP81-2) {Arabidopsis thaliana}; contains Pfam profiles PF02518: ATPase, histidine kinase-, DNA gyrase B-, and HSP90-like domain protein, PF00183: Hsp90 protein | | | | | | |
|  |  | RAFL11-03-D07 | At5g56000 / heat shock protein 81.4 (hsp81.4) | |  |  |  |  |  | | --- | --- | --- | --- | --- | |  |  |  |  |  | | RAFL11-03-D07 ,At5g56000  heat shock protein 81-4 (HSP81-4) nearly identical to heat shock protein hsp81.4 [Arabidopsis thaliana] GI:1906828; contains Pfam profiles PF02518: ATPase, histidine kinase-, DNA gyrase B-, and HSP90-like domain protein, PF00183: Hsp90 protein | | | | | | |
|  |  | RAFL04-15-M13 | At5g56030 / heat shock protein 81-2 (HSP81-2) | |  |  |  |  |  | | --- | --- | --- | --- | --- | |  |  |  |  |  | | RAFL04-15-M13 ,At5g56030  heat shock protein 81-2 (HSP81-2) nearly identical to SP|P55737 Heat shock protein 81-2 (HSP81-2) {Arabidopsis thaliana} | | | | | | |
| 1\_24630001\_24660000 | | |  |  | A | B | C | D | P | P' | N |
|  | Cluster:1-0 | |  |  | 1 | 148 | 0 | 4514 | 0.031953678 | 0.031953678 | 1 |
|  |  | RAFL06-12-I20 | At1g66970 / expressed protein | |  |  |  |  |  | | --- | --- | --- | --- | --- | |  |  |  |  |  | | At1g66970 ,RAFL06-12-I20  glycerophosphoryl diester phosphodiesterase family protein contains Pfam PF03009 : Glycerophosphoryl diester phosphodiesterase family | | | | | | |
| 2\_11190001\_11220000 | | |  |  | A | B | C | D | P | P' | N |
|  | Cluster:0-2 | |  |  | 1 | 78 | 0 | 4584 | 0.016941883 | 0.016941883 | 1 |
|  |  | RAFL05-03-B18 | At2g26500 / expressed protein | |  |  |  |  |  | | --- | --- | --- | --- | --- | |  |  |  |  |  | | RAFL05-03-B18 ,At2g26500  cytochrome b6f complex subunit (petM), putative nearly identical to cytochrome b6f complex subunit (GI:3090403) [Arabidopsis thaliana]; alternative splice forms exist | | | | | | |
| 3\_23040001\_23070000 | | |  |  | A | B | C | D | P | P' | N |
|  | Cluster:9-2 | |  |  | 1 | 66 | 2 | 4594 | 0.042497892 | 0.12749368 | 3 |
|  |  | RAFL06-13-K18 | At3g62260 / protein phosphatase 2C (PP2C), putative | |  |  |  |  |  | | --- | --- | --- | --- | --- | |  |  |  |  |  | | RAFL06-13-K18 ,At3g62260  protein phosphatase 2C, putative / PP2C, putative phosphoprotein phosphatase (EC 3.1.3.16) 1A-alpha - Homo sapiens, PIR:S22423 | | | | | | |
| 5\_7200001\_7500000 | | |  |  | A | B | C | D | P | P' | N |
|  | Cluster:5-1 | |  |  | 3 | 279 | 10 | 4371 | 0.039814703 | 0.39814702 | 10 |
|  |  | RAFL04-17-B06 | At5g22060 / DnaJ protein, putative | |  |  |  |  |  | | --- | --- | --- | --- | --- | |  |  |  |  |  | | RAFL04-17-B06 ,At5g22060  DNAJ heat shock protein, putative strong similarity to SP|O60884 DnaJ homolog subfamily A member 2 (Dnj3) Homo sapiens, several plant DnaJ proteins from PGR; contains Pfam profiles PF00226 DnaJ domain, PF00684 DnaJ central domain (4 repeats), PF01556 DnaJ C terminal region | | | | | | |
|  |  | RAFL07-08-I10 | At5g22460 / esterase/lipase/thioesterase family | |  |  |  |  |  | | --- | --- | --- | --- | --- | |  |  |  |  |  | | At5g22460 ,RAFL07-08-I10  esterase/lipase/thioesterase family protein low similarity to 2-hydroxy-6-oxo-6-phenylhexa-2,4-dienoate hydrolase [Rhodococcus sp. RHA1] GI:8978311, SP|Q02104 Lipase 1 precursor (EC 3.1.1.3) (Triacylglycerol lipase) {Psychrobacter immobilis}; contains Interpro entry IPR000379 | | | | | | |
|  |  | RAFL05-08-E17 | At5g22330 / Ruv DNA-helicase-related protein | |  |  |  |  |  | | --- | --- | --- | --- | --- | |  |  |  |  |  | | At5g22330 ,RAFL05-08-E17  TATA box-binding protein-interacting protein-related similar to TATA box-binding protein-interacting protein SP:O35753 from [ Mus musculus] | | | | | | |
| 1\_4770001\_4800000 | | |  |  | A | B | C | D | P | P' | N |
|  | Cluster:9-1 | |  |  | 1 | 95 | 1 | 4566 | 0.040755685 | 0.08151137 | 2 |
|  |  | RAFL05-05-E24 | At1g13990 / expressed protein | |  |  |  |  |  | | --- | --- | --- | --- | --- | |  |  |  |  |  | | At1g13990 ,RAFL05-05-E24  expressed protein | | | | | | |
| 1\_19290001\_19320000 | | |  |  | A | B | C | D | P | P' | N |
|  | Cluster:3-1 | |  |  | 1 | 215 | 0 | 4447 | 0.04632211 | 0.04632211 | 1 |
|  |  | RAFL04-14-M12 | At1g52930 / expressed protein | |  |  |  |  |  | | --- | --- | --- | --- | --- | |  |  |  |  |  | | RAFL04-14-M12 ,At1g52930  brix domain-containing protein contains Pfam domain, PF04427: Brix domain | | | | | | |
| 1\_2430001\_2460000 | | |  |  | A | B | C | D | P | P' | N |
|  | Cluster:2-0 | |  |  | 4 | 146 | 5 | 4508 | 1.1431223E-4 | 4.572489E-4 | 4 |
|  |  | RAFL08-18-F01 | At1g07930 / elongation factor 1-alpha (EF-1-alpha) | |  |  |  |  |  | | --- | --- | --- | --- | --- | |  |  |  |  |  | | RAFL08-18-F01 ,At1g07930  elongation factor 1-alpha / EF-1-alpha identical to GB:CAA34456 from [Arabidopsis thaliana] (Plant Mol. Biol. 14 (1), 107-110 (1990)) | | | | | | |
|  |  | RAFL09-13-F11 | At1g07930 / elongation factor 1-alpha (EF-1-alpha) | |  |  |  |  |  | | --- | --- | --- | --- | --- | |  |  |  |  |  | | RAFL09-13-F11 ,At1g07930  elongation factor 1-alpha / EF-1-alpha identical to GB:CAA34456 from [Arabidopsis thaliana] (Plant Mol. Biol. 14 (1), 107-110 (1990)) | | | | | | |
|  |  | RAFL07-17-I21 | At1g07930 / elongation factor 1-alpha (EF-1-alpha) | |  |  |  |  |  | | --- | --- | --- | --- | --- | |  |  |  |  |  | | RAFL07-17-I21 ,At1g07930  elongation factor 1-alpha / EF-1-alpha identical to GB:CAA34456 from [Arabidopsis thaliana] (Plant Mol. Biol. 14 (1), 107-110 (1990)) | | | | | | |
|  |  | RAFL08-09-H17 | At1g07930 / elongation factor 1-alpha (EF-1-alpha) | |  |  |  |  |  | | --- | --- | --- | --- | --- | |  |  |  |  |  | | RAFL08-09-H17 ,At1g07930  elongation factor 1-alpha / EF-1-alpha identical to GB:CAA34456 from [Arabidopsis thaliana] (Plant Mol. Biol. 14 (1), 107-110 (1990)) | | | | | | |
| 1\_25410001\_25440000 | | |  |  | A | B | C | D | P | P' | N |
|  | Cluster:10-2 | |  |  | 1 | 108 | 1 | 4553 | 0.0462095 | 0.092419 | 2 |
|  |  | RAFL05-01-L22 | At1g68620 / expressed protein | |  |  |  |  |  | | --- | --- | --- | --- | --- | |  |  |  |  |  | | RAFL05-01-L22 ,At1g68620  expressed protein similar to PrMC3 [Pinus radiata] GI:5487873 | | | | | | |
| 4\_12870001\_12900000 | | |  |  | A | B | C | D | P | P' | N |
|  | Cluster:6-2 | |  |  | 1 | 175 | 0 | 4487 | 0.03774394 | 0.03774394 | 1 |
|  |  | RAFL04-14-N02 | At4g27960 / ubiquitin-conjugating enzyme 9 (UBC9) | |  |  |  |  |  | | --- | --- | --- | --- | --- | |  |  |  |  |  | | At4g27960 ,RAFL04-14-N02  ubiquitin-conjugating enzyme E2-17 kDa 9 (UBC9) E2; identical to gi:297883, SP:P35132; identical to cDNA UBC9 for ubiquitin conjugating enzyme homolog GI:297883 | | | | | | |
| 5\_24660001\_24690000 | | |  |  | A | B | C | D | P | P' | N |
|  | Cluster:4-2 | |  |  | 1 | 134 | 0 | 4528 | 0.028951319 | 0.028951319 | 1 |
|  |  | RAFL06-13-P21 | At5g62130 / expressed protein | |  |  |  |  |  | | --- | --- | --- | --- | --- | |  |  |  |  |  | | At5g62130 ,RAFL06-13-P21  Per1-like protein-related | | | | | | |
| 1\_15990001\_16020000 | | |  |  | A | B | C | D | P | P' | N |
|  | Cluster:1-2 | |  |  | 1 | 173 | 0 | 4489 | 0.037315033 | 0.037315033 | 1 |
|  |  | RAFL05-21-I19 | At1g43670 / fructose 1,6-bisphosphatase -related | |  |  |  |  |  | | --- | --- | --- | --- | --- | |  |  |  |  |  | | RAFL05-21-I19 ,At1g43670  fructose-1,6-bisphosphatase, putative / D-fructose-1,6-bisphosphate 1-phosphohydrolase, putative / FBPase, putative very strong similarity to SP|P46267 Fructose-1,6-bisphosphatase, cytosolic (EC 3.1.3.11) (D-fructose-1,6- bisphosphate 1-phosphohydrolase) (FBPase) {Brassica napus}; contains Pfam profile PF00316: fructose-1,6-bisphosphatase | | | | | | |
| 1\_16200001\_16500000 | | |  |  | A | B | C | D | P | P' | N |
|  | Cluster:0-1 | |  |  | 2 | 104 | 3 | 4554 | 0.004895142 | 0.019580567 | 4 |
|  |  | RAFL05-18-I22 | At1g44575 / photosystem II 22kDa protein -related | |  |  |  |  |  | | --- | --- | --- | --- | --- | |  |  |  |  |  | | At1g44575 ,RAFL05-18-I22  photosystem II 22kDa protein, chloroplast / CP22 (PSBS) identical to photosystem II 22 kDa protein, chloroplast [precursor] SP:Q9XF91 from [Arabidopsis thaliana]; contains Pfam profile PF00504: Chlorophyll A-B binding protein | | | | | | |
|  |  | RAFL06-13-A08 | At1g44575 / photosystem II 22kDa protein -related | |  |  |  |  |  | | --- | --- | --- | --- | --- | |  |  |  |  |  | | RAFL06-13-A08 ,At1g44575  photosystem II 22kDa protein, chloroplast / CP22 (PSBS) identical to photosystem II 22 kDa protein, chloroplast [precursor] SP:Q9XF91 from [Arabidopsis thaliana]; contains Pfam profile PF00504: Chlorophyll A-B binding protein | | | | | | |
| 3\_19950001\_19980000 | | |  |  | A | B | C | D | P | P' | N |
|  | Cluster:2-1 | |  |  | 2 | 242 | 1 | 4418 | 0.00789916 | 0.01579832 | 2 |
|  |  | RAFL05-07-H16 | At3g53870 / 40S ribosomal protein S3 (RPS3B) | |  |  |  |  |  | | --- | --- | --- | --- | --- | |  |  |  |  |  | | At3g53870 ,RAFL05-07-H16  40S ribosomal protein S3 (RPS3B) ribosomal protein S3a - Xenopus laevis, PIR:R3XL3A | | | | | | |
|  |  | RAFL05-13-M17 | At3g53890 / 40S ribosomal protein S21 homolog | |  |  |  |  |  | | --- | --- | --- | --- | --- | |  |  |  |  |  | | RAFL05-13-M17 ,At3g53890  40S ribosomal protein S21 (RPS21B) ribosomal protein S21, cytosolic - Oryza sativa, PIR:S38357 | | | | | | |
| 1\_26970001\_27000000 | | |  |  | A | B | C | D | P | P' | N |
|  | Cluster:0-0 | |  |  | 1 | 35 | 0 | 4627 | 0.0077203517 | 0.0077203517 | 1 |
|  |  | RAFL06-08-C15 | At1g72610 / germin-like protein (AtGER1) | |  |  |  |  |  | | --- | --- | --- | --- | --- | |  |  |  |  |  | | RAFL06-08-C15 ,At1g72610  germin-like protein (GER1) identical to germin-like protein subfamily 3 member 1 SP|P94040; contains Pfam profile: PF01072 Germin family | | | | | | |
| 4\_2100001\_2400000 | | |  |  | A | B | C | D | P | P' | N |
|  | Cluster:2-2 | |  |  | 1 | 52 | 1 | 4609 | 0.022605369 | 0.045210738 | 2 |
|  |  | RAFL04-12-C20 | At4g04330 / expressed protein | |  |  |  |  |  | | --- | --- | --- | --- | --- | |  |  |  |  |  | | RAFL04-12-C20 ,At4g04330  expressed protein | | | | | | |
| 3\_19230001\_19260000 | | |  |  | A | B | C | D | P | P' | N |
|  | Cluster:8-0 | |  |  | 1 | 108 | 1 | 4553 | 0.0462095 | 0.092419 | 2 |
|  |  | RAFL06-13-H12 | At3g51840 / acyl-coA dehydrogenase | |  |  |  |  |  | | --- | --- | --- | --- | --- | |  |  |  |  |  | | At3g51840 ,RAFL06-13-H12  short-chain acyl-CoA oxidase identical to Short-chain acyl CoA oxidase [Arabidopsis thaliana] GI:5478795; contains InterPro entry IPR006089: Acyl-CoA dehydrogenase | | | | | | |
| 1\_5910001\_5940000 | | |  |  | A | B | C | D | P | P' | N |
|  | Cluster:2-2 | |  |  | 1 | 52 | 1 | 4609 | 0.022605369 | 0.045210738 | 2 |
|  |  | RAFL11-07-B20 | At1g17330 / hypothetical protein | |  |  |  |  |  | | --- | --- | --- | --- | --- | |  |  |  |  |  | | RAFL11-07-B20 ,At1g17330  metal-dependent phosphohydrolase HD domain-containing protein-related | | | | | | |
| 5\_16800001\_17100000 | | |  |  | A | B | C | D | P | P' | N |
|  | Cluster:2-2 | |  |  | 2 | 51 | 11 | 4599 | 0.009126297 | 0.07301038 | 8 |
|  |  | RAFL06-10-H13 | At5g42650 / allene oxide synthase / cytochrome P450 74A | |  |  |  |  |  | | --- | --- | --- | --- | --- | |  |  |  |  |  | | RAFL06-10-H13 ,At5g42650  allene oxide synthase (AOS) / hydroperoxide dehydrase / cytochrome P450 74A (CYP74A) identical to Allene oxide synthase, chloroplast precursor (Hydroperoxide dehydrase) (Cytochrome P450 74A) (SP:Q96242) {Arabidopsis thaliana} | | | | | | |
|  |  | RAFL05-12-G03 | At5g42650 / allene oxide synthase / cytochrome P450 74A | |  |  |  |  |  | | --- | --- | --- | --- | --- | |  |  |  |  |  | | At5g42650 ,RAFL05-12-G03  allene oxide synthase (AOS) / hydroperoxide dehydrase / cytochrome P450 74A (CYP74A) identical to Allene oxide synthase, chloroplast precursor (Hydroperoxide dehydrase) (Cytochrome P450 74A) (SP:Q96242) {Arabidopsis thaliana} | | | | | | |
| 3\_19380001\_19410000 | | |  |  | A | B | C | D | P | P' | N |
|  | Cluster:3-0 | |  |  | 1 | 232 | 0 | 4430 | 0.049967833 | 0.049967833 | 1 |
|  |  | RAFL05-17-A01 | At3g52300 / expressed protein | |  |  |  |  |  | | --- | --- | --- | --- | --- | |  |  |  |  |  | | RAFL05-17-A01 ,At3g52300  ATP synthase D chain-related contains weak similarity to ATP synthase D chain, mitochondrial (EC 3.6.3.14) (Swiss-Prot:P31399) [Rattus norvegicus] | | | | | | |
| 5\_16800001\_16830000 | | |  |  | A | B | C | D | P | P' | N |
|  | Cluster:2-2 | |  |  | 2 | 51 | 0 | 4610 | 1.267773E-4 | 1.267773E-4 | 1 |
|  |  | RAFL06-10-H13 | At5g42650 / allene oxide synthase / cytochrome P450 74A | |  |  |  |  |  | | --- | --- | --- | --- | --- | |  |  |  |  |  | | RAFL06-10-H13 ,At5g42650  allene oxide synthase (AOS) / hydroperoxide dehydrase / cytochrome P450 74A (CYP74A) identical to Allene oxide synthase, chloroplast precursor (Hydroperoxide dehydrase) (Cytochrome P450 74A) (SP:Q96242) {Arabidopsis thaliana} | | | | | | |
|  |  | RAFL05-12-G03 | At5g42650 / allene oxide synthase / cytochrome P450 74A | |  |  |  |  |  | | --- | --- | --- | --- | --- | |  |  |  |  |  | | At5g42650 ,RAFL05-12-G03  allene oxide synthase (AOS) / hydroperoxide dehydrase / cytochrome P450 74A (CYP74A) identical to Allene oxide synthase, chloroplast precursor (Hydroperoxide dehydrase) (Cytochrome P450 74A) (SP:Q96242) {Arabidopsis thaliana} | | | | | | |
| 5\_19530001\_19560000 | | |  |  | A | B | C | D | P | P' | N |
|  | Cluster:6-2 | |  |  | 1 | 175 | 0 | 4487 | 0.03774394 | 0.03774394 | 1 |
|  |  | RAFL04-15-L12 | At5g48930 / anthranilate N-hydroxycinnamoyl/benzoyltransferase family | |  |  |  |  |  | | --- | --- | --- | --- | --- | |  |  |  |  |  | | RAFL04-15-L12 ,At5g48930  transferase family protein similar to anthranilate N-hydroxycinnamoyl/benzoyltransferase from Dianthus caryophyllus [GI:3288180, GI:2239091]; contains Pfam profile PF02458 transferase family | | | | | | |
| 2\_3000001\_6000000 | | |  |  | A | B | C | D | P | P' | N |
|  | Cluster:0-0 | |  |  | 2 | 34 | 9 | 4618 | 0.003051236 | 0.024409888 | 8 |
|  |  | RAFL06-16-M15 | At2g10940 / protease inhibitor/seed storage/lipid transfer protein (LTP) family | |  |  |  |  |  | | --- | --- | --- | --- | --- | |  |  |  |  |  | | At2g10940 ,RAFL06-16-M15  protease inhibitor/seed storage/lipid transfer protein (LTP) family protein similar to proline-rich cell wall protein [Medicago sativa] GI:3818416; contains Pfam profile PF00234 Protease inhibitor/seed storage/LTP family | | | | | | |
|  |  | RAFL06-08-D09 | At2g10940 / protease inhibitor/seed storage/lipid transfer protein (LTP) family | |  |  |  |  |  | | --- | --- | --- | --- | --- | |  |  |  |  |  | | At2g10940 ,RAFL06-08-D09  protease inhibitor/seed storage/lipid transfer protein (LTP) family protein similar to proline-rich cell wall protein [Medicago sativa] GI:3818416; contains Pfam profile PF00234 Protease inhibitor/seed storage/LTP family | | | | | | |
| 5\_24600001\_24630000 | | |  |  | A | B | C | D | P | P' | N |
|  | Cluster:6-2 | |  |  | 3 | 173 | 1 | 4486 | 2.0567402E-4 | 4.1134804E-4 | 2 |
|  |  | RAFL05-05-M22 | At5g61990 / pentatricopeptide (PPR) repeat-containing protein | |  |  |  |  |  | | --- | --- | --- | --- | --- | |  |  |  |  |  | | At5g61990 ,RAFL05-05-M22  pentatricopeptide (PPR) repeat-containing protein contains Pfam profile PF01535: PPR repeat | | | | | | |
|  |  | RAFL07-10-G12 | At5g62010 / unknown similar to AGI | |  |  |  |  |  | | --- | --- | --- | --- | --- | |  |  |  |  |  | | At5g62010 ,RAFL07-10-G12  http://rarge.gsc.riken.go.jp/microarray/microarray\_data2.pl?ALL\_EX=on&NC\_FLAG=display&LOG=on&ID=RAFL07-10-G12 | | | | | | |
|  |  | RAFL04-09-J03 | At5g62010 / unknown similar to AGI | |  |  |  |  |  | | --- | --- | --- | --- | --- | |  |  |  |  |  | | At5g62010 ,RAFL04-09-J03  http://rarge.gsc.riken.go.jp/microarray/microarray\_data2.pl?ALL\_EX=on&NC\_FLAG=display&LOG=on&ID=RAFL04-09-J03 | | | | | | |
| 5\_12360001\_12390000 | | |  |  | A | B | C | D | P | P' | N |
|  | Cluster:1-2 | |  |  | 1 | 173 | 0 | 4489 | 0.037315033 | 0.037315033 | 1 |
|  |  | RAFL06-07-E13 | At5g33320 / triose phosphate/phosphate translocator, putative | |  |  |  |  |  | | --- | --- | --- | --- | --- | |  |  |  |  |  | | At5g33320 ,RAFL06-07-E13  triose phosphate/phosphate translocator, putative similar to SWISS-PROT:P52178 triose phosphate/phosphate translocator [Cauliflower] {Brassica oleracea} | | | | | | |
| 1\_3810001\_3840000 | | |  |  | A | B | C | D | P | P' | N |
|  | Cluster:9-1 | |  |  | 1 | 95 | 0 | 4567 | 0.020587604 | 0.020587604 | 1 |
|  |  | RAFL08-08-I15 | At1g11360 / expressed protein | |  |  |  |  |  | | --- | --- | --- | --- | --- | |  |  |  |  |  | | RAFL08-08-I15 ,At1g11360  universal stress protein (USP) family protein contains Pfam PF00582: universal stress protein family domain; similar to ethylene-responsive ER6 protein (GI:5669654) [Lycopersicon esculentum] | | | | | | |
| 3\_22650001\_22680000 | | |  |  | A | B | C | D | P | P' | N |
|  | Cluster:5-0 | |  |  | 1 | 76 | 2 | 4584 | 0.04873567 | 0.146207 | 3 |
|  |  | RAFL07-08-A21 | At3g61220 / short-chain dehydrogenase/reductase family protein | |  |  |  |  |  | | --- | --- | --- | --- | --- | |  |  |  |  |  | | At3g61220 ,RAFL07-08-A21  short-chain dehydrogenase/reductase (SDR) family protein similar to carbonyl reductase GI:1049108 from [Mus musculus] | | | | | | |
| 5\_24030001\_24060000 | | |  |  | A | B | C | D | P | P' | N |
|  | Cluster:2-0 | |  |  | 1 | 149 | 0 | 4513 | 0.03216813 | 0.03216813 | 1 |
|  |  | RAFL06-16-A10 | At5g60490 / fasciclin-like arabinogalactan-protein (FLA12) | |  |  |  |  |  | | --- | --- | --- | --- | --- | |  |  |  |  |  | | RAFL06-16-A10 ,At5g60490  fasciclin-like arabinogalactan-protein (FLA12) | | | | | | |
| 1\_5490001\_5520000 | | |  |  | A | B | C | D | P | P' | N |
|  | Cluster:8-0 | |  |  | 1 | 108 | 1 | 4553 | 0.0462095 | 0.092419 | 2 |
|  |  | RAFL05-14-C07 | At1g16030 / heat shock protein hsp70b | |  |  |  |  |  | | --- | --- | --- | --- | --- | |  |  |  |  |  | | RAFL05-14-C07 ,At1g16030  heat shock protein 70, putative / HSP70, putative similar to heat shock protein hsp70 GI:1771478 from [Pisum sativum] | | | | | | |
| 2\_9510001\_9540000 | | |  |  | A | B | C | D | P | P' | N |
|  | Cluster:9-2 | |  |  | 1 | 66 | 2 | 4594 | 0.042497892 | 0.12749368 | 3 |
|  |  | RAFL05-09-N09 | At2g22500 / mitochondrial carrier protein family | |  |  |  |  |  | | --- | --- | --- | --- | --- | |  |  |  |  |  | | At2g22500 ,RAFL05-09-N09  mitochondrial substrate carrier family protein contains Pfam profile: PF00153 mitochondrial carrier protein | | | | | | |
| 4\_8700001\_9000000 | | |  |  | A | B | C | D | P | P' | N |
|  | Cluster:1-0 | |  |  | 4 | 145 | 12 | 4502 | 0.0013500572 | 0.016200686 | 12 |
|  |  | RAFL11-05-E03 | At4g17600 / Lil3 protein | |  |  |  |  |  | | --- | --- | --- | --- | --- | |  |  |  |  |  | | RAFL11-05-E03 ,At4g17600  lil3 protein identical to Lil3 protein [Arabidopsis thaliana] gi|4741966|gb|AAD28780 | | | | | | |
|  |  | RAFL11-11-M03 | At4g17600 / Lil3 protein | |  |  |  |  |  | | --- | --- | --- | --- | --- | |  |  |  |  |  | | RAFL11-11-M03 ,At4g17600  lil3 protein identical to Lil3 protein [Arabidopsis thaliana] gi|4741966|gb|AAD28780 | | | | | | |
|  |  | RAFL06-10-B16 | At4g17560 / ribosomal protein L19, putative | |  |  |  |  |  | | --- | --- | --- | --- | --- | |  |  |  |  |  | | At4g17560 ,RAFL06-10-B16  ribosomal protein L19 family protein similar to plastid ribosomal protein L19 precursor [Spinacia oleracea] gi|7582403|gb|AAF64312 | | | | | | |
|  |  | RAFL06-12-P08 | At4g18030 / dehydration-induced protein family | |  |  |  |  |  | | --- | --- | --- | --- | --- | |  |  |  |  |  | | At4g18030 ,RAFL06-12-P08  dehydration-responsive family protein similar to early-responsive to dehydration stress ERD3 protein [Arabidopsis thaliana] GI:15320410; contains Pfam profile PF03141: Putative methyltransferase | | | | | | |
| 3\_22050001\_22080000 | | |  |  | A | B | C | D | P | P' | N |
|  | Cluster:6-0 | |  |  | 1 | 139 | 0 | 4523 | 0.03002359 | 0.03002359 | 1 |
|  |  | RAFL04-17-C18 | At3g59690 / expressed protein | |  |  |  |  |  | | --- | --- | --- | --- | --- | |  |  |  |  |  | | At3g59690 ,RAFL04-17-C18  calmodulin-binding family protein similar to SF16 protein [Helianthus annuus] GI:560150; contains Pfam profile PF00612: IQ calmodulin-binding motif | | | | | | |
| 4\_1500001\_1530000 | | |  |  | A | B | C | D | P | P' | N |
|  | Cluster:10-2 | |  |  | 1 | 108 | 1 | 4553 | 0.0462095 | 0.092419 | 2 |
|  |  | RAFL05-17-C10 | At4g03420 / expressed protein | |  |  |  |  |  | | --- | --- | --- | --- | --- | |  |  |  |  |  | | At4g03420 ,RAFL05-17-C10  expressed protein | | | | | | |
| 4\_16200001\_16500000 | | |  |  | A | B | C | D | P | P' | N |
|  | Cluster:0-1 | |  |  | 2 | 104 | 13 | 4544 | 0.04433326 | 0.39899936 | 9 |
|  |  | RAFL04-14-H12 | At4g36540 / expressed protein | |  |  |  |  |  | | --- | --- | --- | --- | --- | |  |  |  |  |  | | At4g36540 ,RAFL04-14-H12  basic helix-loop-helix (bHLH) family protein contains Pfam domain, PF00010: Helix-loop-helix DNA-binding domain | | | | | | |
|  |  | RAFL07-16-J11 | At4g36870 / BEL1-like homeobox 2 protein (BLH2) | |  |  |  |  |  | | --- | --- | --- | --- | --- | |  |  |  |  |  | | RAFL07-16-J11 ,At4g36870  BEL1-like homeobox 2 protein (BLH2) | | | | | | |
| 1\_9300001\_9330000 | | |  |  | A | B | C | D | P | P' | N |
|  | Cluster:5-0 | |  |  | 1 | 76 | 1 | 4585 | 0.032756753 | 0.06551351 | 2 |
|  |  | RAFL06-09-N14 | At1g26920 / expressed protein | |  |  |  |  |  | | --- | --- | --- | --- | --- | |  |  |  |  |  | | RAFL06-09-N14 ,At1g26920  expressed protein Location of EST 228A16T7A, gb|N65686 | | | | | | |
| 1\_14100001\_14400000 | | |  |  | A | B | C | D | P | P' | N |
|  | Cluster:5-2 | |  |  | 3 | 123 | 3 | 4534 | 3.6307445E-4 | 0.0014522978 | 4 |
|  |  | RAFL09-13-L09 | At1g37130 / nitrate reductase 2 (NR2) | |  |  |  |  |  | | --- | --- | --- | --- | --- | |  |  |  |  |  | | At1g37130 ,RAFL09-13-L09  nitrate reductase 2 (NR2) identical to SP|P11035 Nitrate reductase 2 (formerly EC 1.6.6.1) (NR2) {Arabidopsis thaliana} | | | | | | |
|  |  | RAFL09-11-J22 | At1g37130 / nitrate reductase 2 (NR2) | |  |  |  |  |  | | --- | --- | --- | --- | --- | |  |  |  |  |  | | At1g37130 ,RAFL09-11-J22  nitrate reductase 2 (NR2) identical to SP|P11035 Nitrate reductase 2 (formerly EC 1.6.6.1) (NR2) {Arabidopsis thaliana} | | | | | | |
|  |  | RAFL11-09-K10 | At1g37130 / nitrate reductase 2 (NR2) | |  |  |  |  |  | | --- | --- | --- | --- | --- | |  |  |  |  |  | | At1g37130 ,RAFL11-09-K10  nitrate reductase 2 (NR2) identical to SP|P11035 Nitrate reductase 2 (formerly EC 1.6.6.1) (NR2) {Arabidopsis thaliana} | | | | | | |
| 2\_2100001\_2400000 | | |  |  | A | B | C | D | P | P' | N |
|  | Cluster:7-2 | |  |  | 1 | 63 | 2 | 4597 | 0.040621255 | 0.12186376 | 3 |
|  |  | RAFL06-16-J10 | At2g06050 / 12-oxophytodienoate reductase (OPR3)(DDE1) | |  |  |  |  |  | | --- | --- | --- | --- | --- | |  |  |  |  |  | | RAFL06-16-J10 ,At2g06050  12-oxophytodienoate reductase (OPR3) / delayed dehiscence1 (DDE1) nearly identical to DELAYED DEHISCENCE1 [GI:7688991] and to OPR3 [GI:10242314]; contains Pfam profile PF00724:oxidoreductase, FAD/FMN-binding; identical to cDNA OPDA-reductase homolog GI:5059114 | | | | | | |
| 1\_15660001\_15690000 | | |  |  | A | B | C | D | P | P' | N |
|  | Cluster:9-2 | |  |  | 1 | 66 | 0 | 4596 | 0.014368433 | 0.014368433 | 1 |
|  |  | RAFL05-18-N16 | At1g42990 / bZIP family transcription factor | |  |  |  |  |  | | --- | --- | --- | --- | --- | |  |  |  |  |  | | At1g42990 ,RAFL05-18-N16  bZIP transcription factor family protein contains Pfam profile: PF00170: bZIP transcription factor | | | | | | |
| 4\_780001\_810000 | | |  |  | A | B | C | D | P | P' | N |
|  | Cluster:5-2 | |  |  | 1 | 125 | 0 | 4537 | 0.027021231 | 0.027021231 | 1 |
|  |  | RAFL05-14-A18 | At4g01850 / S-adenosylmethionine synthase 2 | |  |  |  |  |  | | --- | --- | --- | --- | --- | |  |  |  |  |  | | At4g01850 ,RAFL05-14-A18  S-adenosylmethionine synthetase 2 (SAM2) identical to S-adenosylmethionine synthetase 2 (Methionine adenosyltransferase 2, AdoMet synthetase 2) [Arabidopsis thaliana] SWISS-PROT:P17562 | | | | | | |
| 2\_17610001\_17640000 | | |  |  | A | B | C | D | P | P' | N |
|  | Cluster:5-0 | |  |  | 1 | 76 | 1 | 4585 | 0.032756753 | 0.06551351 | 2 |
|  |  | RAFL05-11-I16 | At2g42490 / copper amine oxidase -related | |  |  |  |  |  | | --- | --- | --- | --- | --- | |  |  |  |  |  | | At2g42490 ,RAFL05-11-I16  copper amine oxidase, putative similar to copper methylamine oxidase precursor (MAOXII) [Arthrobacter sp.] SWISS-PROT:Q07123 | | | | | | |
| 4\_14040001\_14070000 | | |  |  | A | B | C | D | P | P' | N |
|  | Cluster:8-1 | |  |  | 1 | 161 | 0 | 4501 | 0.034741584 | 0.034741584 | 1 |
|  |  | RAFL05-07-H10 | At4g30990 / hypothetical protein | |  |  |  |  |  | | --- | --- | --- | --- | --- | |  |  |  |  |  | | At4g30990 ,RAFL05-07-H10  expressed protein ; expression supported by MPSS | | | | | | |
| 5\_2700001\_2730000 | | |  |  | A | B | C | D | P | P' | N |
|  | Cluster:3-1 | |  |  | 2 | 214 | 0 | 4447 | 0.0021362621 | 0.0021362621 | 1 |
|  |  | RAFL04-13-O20 | At5g08420 / rev interacting protein mis3 - like | |  |  |  |  |  | | --- | --- | --- | --- | --- | |  |  |  |  |  | | At5g08420 ,RAFL04-13-O20  expressed protein | | | | | | |
|  |  | RAFL04-20-G24 | At5g08400 / expressed protein | |  |  |  |  |  | | --- | --- | --- | --- | --- | |  |  |  |  |  | | RAFL04-20-G24 ,At5g08400  expressed protein predicted proteins, Arabidopsis thaliana and Synechocystis sp. | | | | | | |
| 5\_21900001\_22200000 | | |  |  | A | B | C | D | P | P' | N |
|  | Cluster:4-0 | |  |  | 5 | 235 | 12 | 4411 | 0.0012869332 | 0.009008532 | 7 |
|  |  | RAFL06-07-B07 | At5g55190 / GTP-binding protein atran3, putative | |  |  |  |  |  | | --- | --- | --- | --- | --- | |  |  |  |  |  | | RAFL06-07-B07 ,At5g55190  Ras-related GTP-binding protein (RAN3) identical to atran3 [Arabidopsis thaliana] GI:2058280 | | | | | | |
|  |  | RAFL05-17-L19 | At5g55160 / ubiquitin-like protein SMT3, putative | |  |  |  |  |  | | --- | --- | --- | --- | --- | |  |  |  |  |  | | At5g55160 ,RAFL05-17-L19  small ubiquitin-like modifier 2 (SUMO) similar to ubiquitin-like protein SMT3 SP:P55852 from [Arabidopsis thaliana]; identical to cDNA small ubiquitin-like modifier 2 (SUMO) GI:22652843; contains Pfam profile PF00240: Ubiquitin family | | | | | | |
|  |  | RAFL06-15-K24 | At5g54750 / transport protein particle component Bet3p-related protein | |  |  |  |  |  | | --- | --- | --- | --- | --- | |  |  |  |  |  | | RAFL06-15-K24 ,At5g54750  transport protein particle (TRAPP) component Bet3, putative similar to SP|P36149 Transport protein particle 22 kDa subunit (TRAPP 22 kDa subunit) {Saccharomyces cerevisiae}; contains Pfam profile PF04051: Transport protein particle (TRAPP) component, Bet3 | | | | | | |
|  |  | RAFL06-16-L13 | At5g54960 / pyruvate decarboxylase (gb|AAB16855.1) | |  |  |  |  |  | | --- | --- | --- | --- | --- | |  |  |  |  |  | | RAFL06-16-L13 ,At5g54960  pyruvate decarboxylase, putative strong similarity to pyruvate decarboxylase 1 [Vitis vinifera] GI:10732644; contains InterPro entry IPR000399: Pyruvate decarboxylase | | | | | | |
|  |  | RAFL05-17-C16 | At5g55190 / GTP-binding protein atran3, putative | |  |  |  |  |  | | --- | --- | --- | --- | --- | |  |  |  |  |  | | RAFL05-17-C16 ,At5g55190  Ras-related GTP-binding protein (RAN3) identical to atran3 [Arabidopsis thaliana] GI:2058280 | | | | | | |
|  | Cluster:7-1 | |  |  | 4 | 261 | 13 | 4385 | 0.0134790605 | 0.09435342 | 7 |
|  |  | RAFL11-06-H06 | At5g54650 / formin homology 2 (FH2) domain-containing protein | |  |  |  |  |  | | --- | --- | --- | --- | --- | |  |  |  |  |  | | RAFL11-06-H06 ,At5g54650  formin homology 2 domain-containing protein / FH2 domain-containing protein contains formin homology 2 domain, Pfam:PF02181 | | | | | | |
|  |  | RAFL06-13-G07 | At5g54855 / expressed protein | |  |  |  |  |  | | --- | --- | --- | --- | --- | |  |  |  |  |  | | At5g54855 ,RAFL06-13-G07  pollen Ole e 1 allergen and extensin family protein contains Pfam domain, PF01190: Pollen proteins Ole e I family | | | | | | |
|  |  | RAFL09-12-N24 | At5g54810 / tryptophan synthase, beta subunit 1 (TSB1) | |  |  |  |  |  | | --- | --- | --- | --- | --- | |  |  |  |  |  | | At5g54810 ,RAFL09-12-N24  tryptophan synthase, beta subunit 1 (TSB1) identical to SP|P14671 | | | | | | |
|  |  | RAFL05-07-E06 | At5g55100 / expressed protein | |  |  |  |  |  | | --- | --- | --- | --- | --- | |  |  |  |  |  | | RAFL05-07-E06 ,At5g55100  SWAP (Suppressor-of-White-APricot)/surp domain-containing protein contains Pfam domain PF01805: Surp module | | | | | | |
| 1\_25380001\_25410000 | | |  |  | A | B | C | D | P | P' | N |
|  | Cluster:8-1 | |  |  | 1 | 161 | 0 | 4501 | 0.034741584 | 0.034741584 | 1 |
|  |  | RAFL04-17-A15 | At1g68580 / expressed protein | |  |  |  |  |  | | --- | --- | --- | --- | --- | |  |  |  |  |  | | At1g68580 ,RAFL04-17-A15  agenet domain-containing protein / bromo-adjacent homology (BAH) domain-containing protein contains Pfam profile PF01426: BAH domain and PF05641: Agenet domain | | | | | | |
| 4\_16200001\_16230000 | | |  |  | A | B | C | D | P | P' | N |
|  | Cluster:0-1 | |  |  | 1 | 105 | 0 | 4557 | 0.022732146 | 0.022732146 | 1 |
|  |  | RAFL04-14-H12 | At4g36540 / expressed protein | |  |  |  |  |  | | --- | --- | --- | --- | --- | |  |  |  |  |  | | At4g36540 ,RAFL04-14-H12  basic helix-loop-helix (bHLH) family protein contains Pfam domain, PF00010: Helix-loop-helix DNA-binding domain | | | | | | |
| 3\_19140001\_19170000 | | |  |  | A | B | C | D | P | P' | N |
|  | Cluster:5-0 | |  |  | 1 | 76 | 1 | 4585 | 0.032756753 | 0.06551351 | 2 |
|  |  | RAFL06-12-D06 | At3g51600 / nonspecific lipid transfer protein 5 (LTP 5) | |  |  |  |  |  | | --- | --- | --- | --- | --- | |  |  |  |  |  | | At3g51600 ,RAFL06-12-D06  nonspecific lipid transfer protein 5 (LTP5) identical to SP|Q9XFS7 Nonspecific lipid-transfer protein 5 (LTP 5) {Arabidopsis thaliana} | | | | | | |
| 4\_16800001\_16830000 | | |  |  | A | B | C | D | P | P' | N |
|  | Cluster:8-0 | |  |  | 3 | 106 | 0 | 4554 | 1.243132E-5 | 1.243132E-5 | 1 |
|  |  | RAFL08-11-O11 | At4g37980 / mannitol dehydrogenase (ELI3-1), putative | |  |  |  |  |  | | --- | --- | --- | --- | --- | |  |  |  |  |  | | At4g37980 ,RAFL08-11-O11  mannitol dehydrogenase, putative (ELI3-1) identical to GI:16267 | | | | | | |
|  |  | RAFL08-08-M18 | At4g37980 / mannitol dehydrogenase (ELI3-1), putative | |  |  |  |  |  | | --- | --- | --- | --- | --- | |  |  |  |  |  | | At4g37980 ,RAFL08-08-M18  mannitol dehydrogenase, putative (ELI3-1) identical to GI:16267 | | | | | | |
|  |  | RAFL09-16-M04 | At4g37980 / mannitol dehydrogenase (ELI3-1), putative | |  |  |  |  |  | | --- | --- | --- | --- | --- | |  |  |  |  |  | | At4g37980 ,RAFL09-16-M04  mannitol dehydrogenase, putative (ELI3-1) identical to GI:16267 | | | | | | |
| 3\_6960001\_6990000 | | |  |  | A | B | C | D | P | P' | N |
|  | Cluster:3-1 | |  |  | 1 | 215 | 0 | 4447 | 0.04632211 | 0.04632211 | 1 |
|  |  | RAFL08-12-L05 | At3g20000 / membrane import protein -related | |  |  |  |  |  | | --- | --- | --- | --- | --- | |  |  |  |  |  | | RAFL08-12-L05 ,At3g20000  porin family protein low similarity to haymaker protein [Mus musculus] GI:17834089, mitochondrial outer membrane protein MOM35 [Mus musculus] GI:6650562; contains Pfam profile PF01459: Eukaryotic porin | | | | | | |
| 5\_18240001\_18270000 | | |  |  | A | B | C | D | P | P' | N |
|  | Cluster:2-2 | |  |  | 1 | 52 | 0 | 4610 | 0.011366073 | 0.011366073 | 1 |
|  |  | RAFL05-01-H15 | At5g45680 / immunophilin / FKBP-type peptidyl-prolyl cis-trans isomerase | |  |  |  |  |  | | --- | --- | --- | --- | --- | |  |  |  |  |  | | RAFL05-01-H15 ,At5g45680  FK506-binding protein 1 (FKBP13) identical to Probable FKBP-type peptidyl-prolyl cis-trans isomerase 3, chloroplast precursor (Ppiase) (Rotamase) (SP:Q9SCY2) / FK506 binding protein 1 (GI:21535744) [Arabidopsis thaliana]; contains Pfam PF00254: peptidyl-prolyl cis-trans isomerase, FKBP-type | | | | | | |
| 1\_10500001\_10800000 | | |  |  | A | B | C | D | P | P' | N |
|  | Cluster:7-2 | |  |  | 2 | 62 | 7 | 4592 | 0.00627458 | 0.04392206 | 7 |
|  |  | RAFL04-12-K17 | At1g30360 / ERD4 protein | |  |  |  |  |  | | --- | --- | --- | --- | --- | |  |  |  |  |  | | At1g30360 ,RAFL04-12-K17  early-responsive to dehydration stress protein (ERD4) nearly identical to ERD4 protein (early-responsive to dehydration stress) [Arabidopsis thaliana] GI:15375406; contains Pfam profile PF02714: Domain of unknown function DUF221 | | | | | | |
|  |  | RAFL11-06-I11 | At1g30360 / ERD4 protein | |  |  |  |  |  | | --- | --- | --- | --- | --- | |  |  |  |  |  | | At1g30360 ,RAFL11-06-I11  early-responsive to dehydration stress protein (ERD4) nearly identical to ERD4 protein (early-responsive to dehydration stress) [Arabidopsis thaliana] GI:15375406; contains Pfam profile PF02714: Domain of unknown function DUF221 | | | | | | |
| 5\_8550001\_8580000 | | |  |  | A | B | C | D | P | P' | N |
|  | Cluster:8-1 | |  |  | 1 | 161 | 0 | 4501 | 0.034741584 | 0.034741584 | 1 |
|  |  | RAFL04-17-N23 | At5g24930 / CONSTANS B-box zinc finger family protein | |  |  |  |  |  | | --- | --- | --- | --- | --- | |  |  |  |  |  | | RAFL04-17-N23 ,At5g24930  zinc finger (B-box type) family protein similar to CONSTANS-like protein 1 GI:4091804 from [Malus x domestica] | | | | | | |
| 4\_5040001\_5070000 | | |  |  | A | B | C | D | P | P' | N |
|  | Cluster:0-1 | |  |  | 1 | 105 | 0 | 4557 | 0.022732146 | 0.022732146 | 1 |
|  |  | RAFL06-07-K18 | At4g09650 / H+-transporting ATP synthase-related protein | |  |  |  |  |  | | --- | --- | --- | --- | --- | |  |  |  |  |  | | At4g09650 ,RAFL06-07-K18  ATP synthase delta chain, chloroplast, putative / H(+)-transporting two-sector ATPase, delta (OSCP) subunit, putative similar to SP|P32980 ATP synthase delta chain, chloroplast precursor (EC 3.6.3.14) {Nicotiana tabacum}; contains Pfam profile PF00213: ATP synthase F1, delta subunit | | | | | | |
| 3\_19710001\_19740000 | | |  |  | A | B | C | D | P | P' | N |
|  | Cluster:9-2 | |  |  | 2 | 65 | 0 | 4596 | 2.034141E-4 | 2.034141E-4 | 1 |
|  |  | RAFL08-17-D17 | At3g53180 / nodulin / glutamate-ammonia ligase - like protein | |  |  |  |  |  | | --- | --- | --- | --- | --- | |  |  |  |  |  | | RAFL08-17-D17 ,At3g53180  glutamine synthetase, putative similar to glutamine synthetase (glutamate--ammonia ligase) [Bacillus subtilis] SWISS-PROT:P12425 | | | | | | |
|  |  | RAFL06-09-F14 | At3g53180 / nodulin / glutamate-ammonia ligase - like protein | |  |  |  |  |  | | --- | --- | --- | --- | --- | |  |  |  |  |  | | RAFL06-09-F14 ,At3g53180  glutamine synthetase, putative similar to glutamine synthetase (glutamate--ammonia ligase) [Bacillus subtilis] SWISS-PROT:P12425 | | | | | | |
| 3\_6000001\_9000000 | | |  |  | A | B | C | D | P | P' | N |
|  | Cluster:3-2 | |  |  | 7 | 30 | 123 | 4503 | 5.706903E-5 | 0.0015979329 | 28 |
|  |  | RAFL09-06-N12 | At3g23810 / S-adenosyl-L-homocysteinas -related | |  |  |  |  |  | | --- | --- | --- | --- | --- | |  |  |  |  |  | | At3g23810 ,RAFL09-06-N12  adenosylhomocysteinase, putative / S-adenosyl-L-homocysteine hydrolase, putative / AdoHcyase, putative strong similarity to SP|P50248|SAHH\_TOBAC Adenosylhomocysteinase (EC 3.3.1.1) (S-adenosyl-L-homocysteine hydrolase) (AdoHcyase) {Nicotiana sylvestris}; contains Pfam profile PF00670: S-adenosyl-L-homocysteine hydrolase, NAD binding domain | | | | | | |
|  |  | RAFL09-13-P13 | At3g23810 / S-adenosyl-L-homocysteinas -related | |  |  |  |  |  | | --- | --- | --- | --- | --- | |  |  |  |  |  | | RAFL09-13-P13 ,At3g23810  adenosylhomocysteinase, putative / S-adenosyl-L-homocysteine hydrolase, putative / AdoHcyase, putative strong similarity to SP|P50248|SAHH\_TOBAC Adenosylhomocysteinase (EC 3.3.1.1) (S-adenosyl-L-homocysteine hydrolase) (AdoHcyase) {Nicotiana sylvestris}; contains Pfam profile PF00670: S-adenosyl-L-homocysteine hydrolase, NAD binding domain | | | | | | |
|  |  | RAFL07-09-L01 | At3g23810 / S-adenosyl-L-homocysteinas -related | |  |  |  |  |  | | --- | --- | --- | --- | --- | |  |  |  |  |  | | RAFL07-09-L01 ,At3g23810  adenosylhomocysteinase, putative / S-adenosyl-L-homocysteine hydrolase, putative / AdoHcyase, putative strong similarity to SP|P50248|SAHH\_TOBAC Adenosylhomocysteinase (EC 3.3.1.1) (S-adenosyl-L-homocysteine hydrolase) (AdoHcyase) {Nicotiana sylvestris}; contains Pfam profile PF00670: S-adenosyl-L-homocysteine hydrolase, NAD binding domain | | | | | | |
|  |  | RAFL05-02-I24 | At3g20390 / translational inhibitor protein -related | |  |  |  |  |  | | --- | --- | --- | --- | --- | |  |  |  |  |  | | At3g20390 ,RAFL05-02-I24  endoribonuclease L-PSP family protein contains Pfam domain PF01042: Endoribonuclease L-PSP | | | | | | |
|  |  | RAFL09-16-F08 | At3g23820 / NAD-dependent epimerase/dehydratase family | |  |  |  |  |  | | --- | --- | --- | --- | --- | |  |  |  |  |  | | At3g23820 ,RAFL09-16-F08  NAD-dependent epimerase/dehydratase family protein similar to nucleotide sugar epimerase from Vibrio vulnificus GI:3093975 [PID:g3093975], WbnF [Escherichia coli] GI:5739472, CAPI protein {Staphylococcus aureus} SP|P39858; contains Pfam profile: PF01370 NAD dependent epimerase/dehydratase family | | | | | | |
|  |  | RAFL09-07-D12 | At3g23820 / NAD-dependent epimerase/dehydratase family | |  |  |  |  |  | | --- | --- | --- | --- | --- | |  |  |  |  |  | | At3g23820 ,RAFL09-07-D12  NAD-dependent epimerase/dehydratase family protein similar to nucleotide sugar epimerase from Vibrio vulnificus GI:3093975 [PID:g3093975], WbnF [Escherichia coli] GI:5739472, CAPI protein {Staphylococcus aureus} SP|P39858; contains Pfam profile: PF01370 NAD dependent epimerase/dehydratase family | | | | | | |
|  |  | RAFL09-10-M18 | At3g23810 / S-adenosyl-L-homocysteinas -related | |  |  |  |  |  | | --- | --- | --- | --- | --- | |  |  |  |  |  | | RAFL09-10-M18 ,At3g23810  adenosylhomocysteinase, putative / S-adenosyl-L-homocysteine hydrolase, putative / AdoHcyase, putative strong similarity to SP|P50248|SAHH\_TOBAC Adenosylhomocysteinase (EC 3.3.1.1) (S-adenosyl-L-homocysteine hydrolase) (AdoHcyase) {Nicotiana sylvestris}; contains Pfam profile PF00670: S-adenosyl-L-homocysteine hydrolase, NAD binding domain | | | | | | |
|  | Cluster:1-1 | |  |  | 7 | 98 | 123 | 4435 | 0.026404485 | 0.7393256 | 28 |
|  |  | RAFL05-17-A02 | At3g20810 / expressed protein | |  |  |  |  |  | | --- | --- | --- | --- | --- | |  |  |  |  |  | | At3g20810 ,RAFL05-17-A02  transcription factor jumonji (jmjC) domain-containing protein contains Pfam domain PF02373: jmjC domain | | | | | | |
|  |  | RAFL07-14-J18 | At3g23530 / cyclopropane synthase, putative | |  |  |  |  |  | | --- | --- | --- | --- | --- | |  |  |  |  |  | | RAFL07-14-J18 ,At3g23530  cyclopropane fatty acid synthase, putative / CPA-FA synthase, putative similar to cyclopropane synthase [Sterculia foetida] GI:21069167; contains Pfam profiles PF02353: Cyclopropane-fatty-acyl-phospholipid synthase, PF01593: amine oxidase, flavin-containing | | | | | | |
|  |  | RAFL09-10-N16 | At3g18000 / phosphoethanolamine N-methyltransferase, putative | |  |  |  |  |  | | --- | --- | --- | --- | --- | |  |  |  |  |  | | RAFL09-10-N16 ,At3g18000  phosphoethanolamine N-methyltransferase 1 / PEAMT 1 (NMT1) identical to Phosphoethanolamine N-methyltransferase 1 (EC 2.1.1.103) (PEAMT 1) (AtNMT1) (SP:Q9FR44){Arabidopsis thaliana}; strong similarity to phosphoethanolamine N-methyltransferase from [Spinacia oleracea] GI:7407189, [Triticum aestivum] GI:17887465; contains Pfam profile PF01209: methlytransferase, UbiE/COQ5 family | | | | | | |
|  |  | RAFL05-19-J04 | At3g24430 / mrp protein -related | |  |  |  |  |  | | --- | --- | --- | --- | --- | |  |  |  |  |  | | RAFL05-19-J04 ,At3g24430  expressed protein contains Pfam profile PF01883: Domain of unknown function | | | | | | |
|  |  | RAFL04-14-G15 | At3g22780 / DNA binding protein -related | |  |  |  |  |  | | --- | --- | --- | --- | --- | |  |  |  |  |  | | RAFL04-14-G15 ,At3g22780  CXC domain protein (TSO1) identical to CXC domain protein TSO1 [Arabidopsis thaliana] GI:7767425 | | | | | | |
|  |  | RAFL07-18-P15 | At3g22120 / protease inhibitor/seed storage/lipid transfer protein (LTP) family | |  |  |  |  |  | | --- | --- | --- | --- | --- | |  |  |  |  |  | | At3g22120 ,RAFL07-18-P15  protease inhibitor/seed storage/lipid transfer protein (LTP) family protein similar to SP|Q00451|PRF1\_LYCES 36.4 kDa proline-rich protein Lycopersicon esculentum, proline-rich cell wall protein [Medicago sativa] GI:3818416; contains Pfam profile PF00234 Protease inhibitor/seed storage/LTP family | | | | | | |
|  |  | RAFL05-17-H07 | At3g17840 / leucine-rich repeat transmembrane protein kinase, putative | |  |  |  |  |  | | --- | --- | --- | --- | --- | |  |  |  |  |  | | At3g17840 ,RAFL05-17-H07  leucine-rich repeat transmembrane protein kinase, putative similar to receptor kinase GB:AAA33715 from [Petunia integrifolia] | | | | | | |
|  | Cluster:2-1 | |  |  | 12 | 232 | 118 | 4301 | 0.0452302 | 1.2664456 | 28 |
|  |  | RAFL07-18-J07 | At3g23530 / cyclopropane synthase, putative | |  |  |  |  |  | | --- | --- | --- | --- | --- | |  |  |  |  |  | | RAFL07-18-J07 ,At3g23530  cyclopropane fatty acid synthase, putative / CPA-FA synthase, putative similar to cyclopropane synthase [Sterculia foetida] GI:21069167; contains Pfam profiles PF02353: Cyclopropane-fatty-acyl-phospholipid synthase, PF01593: amine oxidase, flavin-containing | | | | | | |
|  |  | RAFL04-13-D06 | At3g23940 / dihydroxyacid dehydratase -related | |  |  |  |  |  | | --- | --- | --- | --- | --- | |  |  |  |  |  | | RAFL04-13-D06 ,At3g23940  dehydratase family contains Pfam profile: PF00920 dehydratase family | | | | | | |
|  |  | RAFL09-07-D04 | At3g22230 / 60S ribosomal protein L27 (RPL27B) | |  |  |  |  |  | | --- | --- | --- | --- | --- | |  |  |  |  |  | | At3g22230 ,RAFL09-07-D04  60S ribosomal protein L27 (RPL27B) similar to 60S RIBOSOMAL PROTEIN L27 GB:P41101 from [Solanum tuberosum] | | | | | | |
|  |  | RAFL06-16-M11 | At3g18780 / actin 2 | |  |  |  |  |  | | --- | --- | --- | --- | --- | |  |  |  |  |  | | At3g18780 ,RAFL06-16-M11  actin 2 (ACT2) identical to SP|Q96292 Actin 2 {Arabidopsis thaliana}; nearly identical to SP|Q96293 Actin 8 [Arabidopsis thaliana] GI:1669387 and to At1g49240 | | | | | | |
|  |  | RAFL07-08-E22 | At3g23530 / cyclopropane synthase, putative | |  |  |  |  |  | | --- | --- | --- | --- | --- | |  |  |  |  |  | | RAFL07-08-E22 ,At3g23530  cyclopropane fatty acid synthase, putative / CPA-FA synthase, putative similar to cyclopropane synthase [Sterculia foetida] GI:21069167; contains Pfam profiles PF02353: Cyclopropane-fatty-acyl-phospholipid synthase, PF01593: amine oxidase, flavin-containing | | | | | | |
|  |  | RAFL06-08-P08 | At3g23390 / 60S ribosomal protein L36a/L44 (RPL36aA) | |  |  |  |  |  | | --- | --- | --- | --- | --- | |  |  |  |  |  | | RAFL06-08-P08 ,At3g23390  60S ribosomal protein L36a/L44 (RPL36aA) similar to ribosomal protein L41 GB:AAA34366 from [Candida maltosa] | | | | | | |
|  |  | RAFL05-12-K04 | At3g23050 / auxin-responsive protein IAA7 (Indoleacetic acid-induced protein 7) | |  |  |  |  |  | | --- | --- | --- | --- | --- | |  |  |  |  |  | | RAFL05-12-K04 ,At3g23050  auxin-responsive protein / indoleacetic acid-induced protein 7 (IAA7) identical to SP|Q38825|AXI7\_ARATH Auxin-responsive protein IAA7 (Indoleacetic acid-induced protein 7) | | | | | | |
|  |  | RAFL05-21-L03 | At3g23400 / plastid-lipid associated protein PAP/fibrillin family | |  |  |  |  |  | | --- | --- | --- | --- | --- | |  |  |  |  |  | | RAFL05-21-L03 ,At3g23400  plastid-lipid associated protein PAP / fibrillin family protein contains Pfam profile PF04755: PAP\_fibrillin | | | | | | |
|  |  | RAFL05-21-D08 | At3g18490 / chloroplast nucleoid DNA-binding protein -related | |  |  |  |  |  | | --- | --- | --- | --- | --- | |  |  |  |  |  | | At3g18490 ,RAFL05-21-D08  aspartyl protease family protein contains Pfam domain, PF00026: eukaryotic aspartyl protease | | | | | | |
|  |  | RAFL04-16-M03 | At3g20790 / expressed protein | |  |  |  |  |  | | --- | --- | --- | --- | --- | |  |  |  |  |  | | At3g20790 ,RAFL04-16-M03  oxidoreductase family protein weak similarity to SP|Q07982 Glucose--fructose oxidoreductase precursor (EC 1.1.99.28) {Zymomonas mobilis}; contains Pfam profiles PF01408: Oxidoreductase family NAD-binding Rossmann fold, PF02894: Oxidoreductase family C-terminal alpha/beta domain | | | | | | |
|  |  | RAFL07-12-K04 | At3g20050 / T-complex protein 1, alpha subunit/chaperonin | |  |  |  |  |  | | --- | --- | --- | --- | --- | |  |  |  |  |  | | RAFL07-12-K04 ,At3g20050  T-complex protein 1 alpha subunit / TCP-1-alpha / chaperonin (CCT1) identical to SWISS-PROT:P28769- T-complex protein 1, alpha subunit (TCP-1-alpha) [Arabidopsis thaliana] | | | | | | |
|  |  | RAFL11-10-K08 | At3g22230 / 60S ribosomal protein L27 (RPL27B) | |  |  |  |  |  | | --- | --- | --- | --- | --- | |  |  |  |  |  | | At3g22230 ,RAFL11-10-K08  60S ribosomal protein L27 (RPL27B) similar to 60S RIBOSOMAL PROTEIN L27 GB:P41101 from [Solanum tuberosum] | | | | | | |
| 3\_5580001\_5610000 | | |  |  | A | B | C | D | P | P' | N |
|  | Cluster:2-0 | |  |  | 2 | 148 | 2 | 4511 | 0.00591059 | 0.017731769 | 3 |
|  |  | RAFL04-15-D12 | At3g16460 / jacalin lectin family | |  |  |  |  |  | | --- | --- | --- | --- | --- | |  |  |  |  |  | | At3g16460 ,RAFL04-15-D12  jacalin lectin family protein contains Pfam profile: PF01419 jacalin-like lectin domain; similar to myrosinase binding protein [Brassica napus] GI:1711296, GI:1655824, myrosinase-binding protein homolog [Arabidopsis thaliana] GI:2997767; contains Pfam profile PF01419 jacalin-like lectin family | | | | | | |
|  |  | RAFL03-03-J03 | At3g16450 / jacalin lectin family | |  |  |  |  |  | | --- | --- | --- | --- | --- | |  |  |  |  |  | | At3g16450 ,RAFL03-03-J03  jacalin lectin family protein similar to SP|Q9SAV1 Myrosinase binding protein-like At1g52030 {Arabidopsis thaliana}; contains Pfam profile: PF01419 jacalin-like lectin domain | | | | | | |
| 5\_25620001\_25650000 | | |  |  | A | B | C | D | P | P' | N |
|  | Cluster:5-2 | |  |  | 1 | 125 | 0 | 4537 | 0.027021231 | 0.027021231 | 1 |
|  |  | RAFL05-03-E09 | At5g64860 / glycosyl hydrolase family 77 (4-alpha-glucanotransferase) | |  |  |  |  |  | | --- | --- | --- | --- | --- | |  |  |  |  |  | | At5g64860 ,RAFL05-03-E09  4-alpha-glucanotransferase, putative / disproportionating enzyme, putative similar to 4-alpha-glucanotransferase SP:Q06801 from [Solanum tuberosum] | | | | | | |
| 3\_4200001\_4230000 | | |  |  | A | B | C | D | P | P' | N |
|  | Cluster:2-2 | |  |  | 1 | 52 | 1 | 4609 | 0.022605369 | 0.045210738 | 2 |
|  |  | RAFL04-17-H16 | At3g13110 / serine acetyltransferase (Sat-1) | |  |  |  |  |  | | --- | --- | --- | --- | --- | |  |  |  |  |  | | RAFL04-17-H16 ,At3g13110  serine O-acetyltransferase (SAT-1) identical to serine acetyltransferase (Sat-1) GI:1184048 [Arabidopsis thaliana] | | | | | | |
| 1\_7650001\_7680000 | | |  |  | A | B | C | D | P | P' | N |
|  | Cluster:10-1 | |  |  | 1 | 42 | 0 | 4620 | 0.009221531 | 0.009221531 | 1 |
|  |  | RAFL08-15-M21 | At1g21790 / expressed protein | |  |  |  |  |  | | --- | --- | --- | --- | --- | |  |  |  |  |  | | At1g21790 ,RAFL08-15-M21  expressed protein | | | | | | |
| 4\_8550001\_8580000 | | |  |  | A | B | C | D | P | P' | N |
|  | Cluster:2-2 | |  |  | 1 | 52 | 0 | 4610 | 0.011366073 | 0.011366073 | 1 |
|  |  | RAFL06-16-M17 | At4g17090 / glycosyl hydrolase family 14 (beta-amylase) | |  |  |  |  |  | | --- | --- | --- | --- | --- | |  |  |  |  |  | | At4g17090 ,RAFL06-16-M17  beta-amylase (CT-BMY) / 1,4-alpha-D-glucan maltohydrolase identical to beta-amylase enzyme GI:6065749 from [Arabidopsis thaliana] | | | | | | |
| 3\_15600001\_15630000 | | |  |  | A | B | C | D | P | P' | N |
|  | Cluster:3-2 | |  |  | 1 | 36 | 0 | 4626 | 0.007934806 | 0.007934806 | 1 |
|  |  | RAFL05-09-N18 | At3g43720 / protease inhibitor/seed storage/lipid transfer protein (LTP) family | |  |  |  |  |  | | --- | --- | --- | --- | --- | |  |  |  |  |  | | RAFL05-09-N18 ,At3g43720  protease inhibitor/seed storage/lipid transfer protein (LTP) family protein contains Pfam protease inhibitor/seed storage/LTP family domain PF00234 | | | | | | |
| 2\_18720001\_18750000 | | |  |  | A | B | C | D | P | P' | N |
|  | Cluster:8-1 | |  |  | 1 | 161 | 0 | 4501 | 0.034741584 | 0.034741584 | 1 |
|  |  | RAFL09-09-M22 | At2g45600 / expressed protein | |  |  |  |  |  | | --- | --- | --- | --- | --- | |  |  |  |  |  | | At2g45600 ,RAFL09-09-M22  expressed protein low similarity to PrMC3 [Pinus radiata] GI:5487873 | | | | | | |
| 4\_14940001\_14970000 | | |  |  | A | B | C | D | P | P' | N |
|  | Cluster:9-0 | |  |  | 1 | 31 | 0 | 4631 | 0.0068625347 | 0.0068625347 | 1 |
|  |  | RAFL05-02-O17 | At4g33150 / lysine-ketoglutarate reductase/saccharopine | |  |  |  |  |  | | --- | --- | --- | --- | --- | |  |  |  |  |  | | At4g33150 ,RAFL05-02-O17  lysine-ketoglutarate reductase/saccharopine dehydrogenase bifunctional enzyme identical to lysine-ketoglutarate reductase/saccharopine dehydrogenase GI:2052508 from [Arabidopsis thaliana] | | | | | | |
| 2\_17400001\_17700000 | | |  |  | A | B | C | D | P | P' | N |
|  | Cluster:3-0 | |  |  | 3 | 230 | 12 | 4418 | 0.03588357 | 0.39471924 | 11 |
|  |  | RAFL06-09-K10 | At2g41960 / expressed protein | |  |  |  |  |  | | --- | --- | --- | --- | --- | |  |  |  |  |  | | At2g41960 ,RAFL06-09-K10  expressed protein | | | | | | |
|  |  | RAFL05-21-P13 | At2g42600 / phosphoenolpyruvate carboxylase | |  |  |  |  |  | | --- | --- | --- | --- | --- | |  |  |  |  |  | | At2g42600 ,RAFL05-21-P13  phosphoenolpyruvate carboxylase, putative / PEP carboxylase, putative (PPC2) strong similarity to phosphoenolpyruvate carboxylase [Brassica napus] GI:507808; contains Pfam profile PF00311: phosphoenolpyruvate carboxylase | | | | | | |
|  |  | RAFL08-09-E20 | At2g41840 / 40S ribosomal protein S2 (RPS2C) | |  |  |  |  |  | | --- | --- | --- | --- | --- | |  |  |  |  |  | | RAFL08-09-E20 ,At2g41840  40S ribosomal protein S2 (RPS2C) | | | | | | |
|  | Cluster:7-1 | |  |  | 3 | 262 | 12 | 4386 | 0.049681213 | 0.54649335 | 11 |
|  |  | RAFL09-10-C07 | At2g42030 / C3HC4-type zinc finger protein family | |  |  |  |  |  | | --- | --- | --- | --- | --- | |  |  |  |  |  | | At2g42030 ,RAFL09-10-C07  zinc finger (C3HC4-type RING finger) family protein contains Pfam profile: PF00097 zinc finger, C3HC4 type (RING finger) | | | | | | |
|  |  | RAFL08-11-N21 | At2g42270 / U5 small nuclear ribonucleoprotein helicase, putative | |  |  |  |  |  | | --- | --- | --- | --- | --- | |  |  |  |  |  | | RAFL08-11-N21 ,At2g42270  U5 small nuclear ribonucleoprotein helicase, putative | | | | | | |
|  |  | RAFL05-02-C24 | At2g41900 / CCCH-type zinc finger protein -related | |  |  |  |  |  | | --- | --- | --- | --- | --- | |  |  |  |  |  | | At2g41900 ,RAFL05-02-C24  zinc finger (CCCH-type) family protein contains Pfam domain, PF00642: Zinc finger C-x8-C-x5-C-x3-H type (and similar) and Pfam domain, PF00023: Ankyrin repeat | | | | | | |
| 5\_2100001\_2400000 | | |  |  | A | B | C | D | P | P' | N |
|  | Cluster:2-1 | |  |  | 3 | 241 | 9 | 4410 | 0.021903817 | 0.19713435 | 9 |
|  |  | RAFL08-15-K17 | At5g07090 / 40S ribosomal protein S4 (RPS4B) | |  |  |  |  |  | | --- | --- | --- | --- | --- | |  |  |  |  |  | | At5g07090 ,RAFL08-15-K17  40S ribosomal protein S4 (RPS4B) | | | | | | |
|  |  | RAFL08-14-N17 | At5g07020 / proline-rich protein family | |  |  |  |  |  | | --- | --- | --- | --- | --- | |  |  |  |  |  | | RAFL08-14-N17 ,At5g07020  proline-rich family protein | | | | | | |
|  |  | RAFL05-12-E19 | At5g07340 / calnexin, putative | |  |  |  |  |  | | --- | --- | --- | --- | --- | |  |  |  |  |  | | At5g07340 ,RAFL05-12-E19  calnexin, putative identical to calnexin homolog 2 from Arabidopsis thaliana [SP|Q38798], strong similarity to calnexin homolog 1, Arabidopsis thaliana, EMBL:AT08315 [SP|P29402]; contains Pfam profile PF00262 calreticulin family | | | | | | |
|  | Cluster:1-1 | |  |  | 2 | 103 | 10 | 4548 | 0.028613875 | 0.25752488 | 9 |
|  |  | RAFL04-15-E20 | At5g07240 / expressed protein | |  |  |  |  |  | | --- | --- | --- | --- | --- | |  |  |  |  |  | | At5g07240 ,RAFL04-15-E20  calmodulin-binding family protein contains Pfam profile PF00612: IQ calmodulin-binding motif | | | | | | |
|  |  | RAFL04-10-L09 | At5g07030 / nucleoid DNA-binding-related protein | |  |  |  |  |  | | --- | --- | --- | --- | --- | |  |  |  |  |  | | RAFL04-10-L09 ,At5g07030  aspartyl protease family protein contains Pfam profile:PF00026 eukaryotic aspartyl protease | | | | | | |
| 4\_4740001\_4770000 | | |  |  | A | B | C | D | P | P' | N |
|  | Cluster:1-2 | |  |  | 1 | 173 | 0 | 4489 | 0.037315033 | 0.037315033 | 1 |
|  |  | RAFL04-15-N07 | At4g09040 / RNA recognition motif (RRM) - containing protein | |  |  |  |  |  | | --- | --- | --- | --- | --- | |  |  |  |  |  | | At4g09040 ,RAFL04-15-N07  RNA recognition motif (RRM)-containing protein low similarity to enhancer binding protein-1; EBP1 [Entamoeba histolytica] GI:8163877, SP|P19682 28 kDa ribonucleoprotein, chloroplast precursor (28RNP) {Nicotiana sylvestris}; contains InterPro entry IPR000504: RNA-binding region RNP-1 (RNA recognition motif) (RRM) | | | | | | |
| 3\_17580001\_17610000 | | |  |  | A | B | C | D | P | P' | N |
|  | Cluster:5-2 | |  |  | 1 | 125 | 0 | 4537 | 0.027021231 | 0.027021231 | 1 |
|  |  | RAFL05-04-L01 | At3g47670 / pectinesterase family | |  |  |  |  |  | | --- | --- | --- | --- | --- | |  |  |  |  |  | | At3g47670 ,RAFL05-04-L01  invertase/pectin methylesterase inhibitor family protein similar to pectinesterase from Arabidopsis thaliana SP|Q43867, Phaseolus vulgaris SP|Q43111; contains Pfam profile PF04043: Plant invertase/pectin methylesterase inhibitor | | | | | | |
| 3\_20400001\_20700000 | | |  |  | A | B | C | D | P | P' | N |
|  | Cluster:3-2 | |  |  | 2 | 35 | 12 | 4614 | 0.0052507473 | 0.063008964 | 12 |
|  |  | RAFL06-08-F13 | At3g55360 / 3-oxo-5-alpha-steroid 4-dehydrogenase (steroid 5-alpha-reductase) family | |  |  |  |  |  | | --- | --- | --- | --- | --- | |  |  |  |  |  | | At3g55360 ,RAFL06-08-F13  3-oxo-5-alpha-steroid 4-dehydrogenase family protein / steroid 5-alpha-reductase family protein similar to synaptic glycoprotein SC2 spliced variant from Homo sapiens [EMBL:AF038958], SC2 from Rattus sp. [gi:256994]; contains Pfam 3-oxo-5-alpha-steroid 4-dehydrogenase domain PF02544 | | | | | | |
|  |  | RAFL05-07-N10 | At3g55130 / ABC transporter family protein | |  |  |  |  |  | | --- | --- | --- | --- | --- | |  |  |  |  |  | | At3g55130 ,RAFL05-07-N10  ABC transporter family protein breast cancer resistance protein 1 BCRP1, Mus musculus, EMBL:NP\_036050 | | | | | | |
| 1\_20310001\_20340000 | | |  |  | A | B | C | D | P | P' | N |
|  | Cluster:8-1 | |  |  | 1 | 161 | 0 | 4501 | 0.034741584 | 0.034741584 | 1 |
|  |  | RAFL05-04-O09 | At1g55530 / expressed protein | |  |  |  |  |  | | --- | --- | --- | --- | --- | |  |  |  |  |  | | At1g55530 ,RAFL05-04-O09  zinc finger (C3HC4-type RING finger) family protein contains Pfam domain, PF00097: Zinc finger, C3HC4 type (RING finger) | | | | | | |
| 3\_20760001\_20790000 | | |  |  | A | B | C | D | P | P' | N |
|  | Cluster:7-2 | |  |  | 1 | 63 | 0 | 4599 | 0.013725069 | 0.013725069 | 1 |
|  |  | RAFL08-18-O11 | At3g55980 / expressed protein | |  |  |  |  |  | | --- | --- | --- | --- | --- | |  |  |  |  |  | | At3g55980 ,RAFL08-18-O11  zinc finger (CCCH-type) family protein contains Pfam domain, PF00642: Zinc finger C-x8-C-x5-C-x3-H type (and similar) and Pfam domain, PF00023: Ankyrin repeat | | | | | | |
| 1\_27300001\_27600000 | | |  |  | A | B | C | D | P | P' | N |
|  | Cluster:4-0 | |  |  | 3 | 237 | 9 | 4414 | 0.020962995 | 0.20962994 | 10 |
|  |  | RAFL09-14-N04 | At1g73590 / auxin transporter splice variant b -related | |  |  |  |  |  | | --- | --- | --- | --- | --- | |  |  |  |  |  | | At1g73590 ,RAFL09-14-N04  auxin efflux carrier protein, putative (PIN1) identical to putative auxin efflux carrier protein; AtPIN1 [Arabidopsis thaliana] GI:4151319; contains Pfam profile PF03547: Auxin Efflux Carrier | | | | | | |
|  |  | RAFL04-17-F10 | At1g74230 / glycine-rich RNA-binding protein | |  |  |  |  |  | | --- | --- | --- | --- | --- | |  |  |  |  |  | | RAFL04-17-F10 ,At1g74230  glycine-rich RNA-binding protein similar to RNA-binding protein GB:S46286 from [Nicotiana sylvestris] | | | | | | |
|  |  | RAFL11-02-J06 | At1g74050 / 60S ribosomal protein L6 (RPL6C) | |  |  |  |  |  | | --- | --- | --- | --- | --- | |  |  |  |  |  | | At1g74050 ,RAFL11-02-J06  60S ribosomal protein L6 (RPL6C) similar to 60S ribosomal protein L6 (YL 16 like) GB:CAB57309 from [Cyanophora paradoxa] | | | | | | |
| 5\_20790001\_20820000 | | |  |  | A | B | C | D | P | P' | N |
|  | Cluster:6-2 | |  |  | 1 | 175 | 0 | 4487 | 0.03774394 | 0.03774394 | 1 |
|  |  | RAFL05-07-I02 | At5g51940 / DNA-directed RNA polymerase II subunit-related protein | |  |  |  |  |  | | --- | --- | --- | --- | --- | |  |  |  |  |  | | RAFL05-07-I02 ,At5g51940  DNA-directed RNA polymerase II, putative similar to SP|O88828 DNA-directed RNA polymerase II 14.4 kDa polypeptide (EC 2.7.7.6) (RPB6) (RPB14.4) {Rattus norvegicus}; contains Pfam profile PF01192: RNA polymerases K / 14 to 18 kDa subunit | | | | | | |
| 1\_23250001\_23280000 | | |  |  | A | B | C | D | P | P' | N |
|  | Cluster:2-0 | |  |  | 1 | 149 | 0 | 4513 | 0.03216813 | 0.03216813 | 1 |
|  |  | RAFL06-07-N12 | At1g63690 / expressed protein | |  |  |  |  |  | | --- | --- | --- | --- | --- | |  |  |  |  |  | | At1g63690 ,RAFL06-07-N12  protease-associated (PA) domain-containing protein contains protease associated (PA) domain, Pfam:PF02225 | | | | | | |
| 3\_930001\_960000 | | |  |  | A | B | C | D | P | P' | N |
|  | Cluster:1-1 | |  |  | 1 | 104 | 1 | 4557 | 0.04453306 | 0.08906612 | 2 |
|  |  | RAFL11-05-E10 | At3g03770 / leucine-rich repeat transmembrane protein kinase, putative | |  |  |  |  |  | | --- | --- | --- | --- | --- | |  |  |  |  |  | | At3g03770 ,RAFL11-05-E10  leucine-rich repeat transmembrane protein kinase, putative may contain C-terminal ser/thr protein kinase domain, similar to serine/threonine protein kinase Pto GB:AAB47421 [Lycopersicon esculentum] | | | | | | |
| 5\_22380001\_22410000 | | |  |  | A | B | C | D | P | P' | N |
|  | Cluster:5-0 | |  |  | 5 | 72 | 1 | 4585 | 6.383303E-9 | 1.2766606E-8 | 2 |
|  |  | RAFL05-16-L15 | At5g56010 / heat shock protein, putative | |  |  |  |  |  | | --- | --- | --- | --- | --- | |  |  |  |  |  | | RAFL05-16-L15 ,At5g56010  heat shock protein, putative strong similarity to SP|P55737 Heat shock protein 81-2 (HSP81-2) {Arabidopsis thaliana}; contains Pfam profiles PF02518: ATPase, histidine kinase-, DNA gyrase B-, and HSP90-like domain protein, PF00183: Hsp90 protein | | | | | | |
|  |  | RAFL09-06-O18 | At5g56030 / heat shock protein 81-2 (HSP81-2) | |  |  |  |  |  | | --- | --- | --- | --- | --- | |  |  |  |  |  | | RAFL09-06-O18 ,At5g56030  heat shock protein 81-2 (HSP81-2) nearly identical to SP|P55737 Heat shock protein 81-2 (HSP81-2) {Arabidopsis thaliana} | | | | | | |
|  |  | RAFL07-13-H08 | At5g56010 / heat shock protein, putative | |  |  |  |  |  | | --- | --- | --- | --- | --- | |  |  |  |  |  | | RAFL07-13-H08 ,At5g56010  heat shock protein, putative strong similarity to SP|P55737 Heat shock protein 81-2 (HSP81-2) {Arabidopsis thaliana}; contains Pfam profiles PF02518: ATPase, histidine kinase-, DNA gyrase B-, and HSP90-like domain protein, PF00183: Hsp90 protein | | | | | | |
|  |  | RAFL11-03-D07 | At5g56000 / heat shock protein 81.4 (hsp81.4) | |  |  |  |  |  | | --- | --- | --- | --- | --- | |  |  |  |  |  | | RAFL11-03-D07 ,At5g56000  heat shock protein 81-4 (HSP81-4) nearly identical to heat shock protein hsp81.4 [Arabidopsis thaliana] GI:1906828; contains Pfam profiles PF02518: ATPase, histidine kinase-, DNA gyrase B-, and HSP90-like domain protein, PF00183: Hsp90 protein | | | | | | |
|  |  | RAFL04-15-M13 | At5g56030 / heat shock protein 81-2 (HSP81-2) | |  |  |  |  |  | | --- | --- | --- | --- | --- | |  |  |  |  |  | | RAFL04-15-M13 ,At5g56030  heat shock protein 81-2 (HSP81-2) nearly identical to SP|P55737 Heat shock protein 81-2 (HSP81-2) {Arabidopsis thaliana} | | | | | | |
| 2\_11280001\_11310000 | | |  |  | A | B | C | D | P | P' | N |
|  | Cluster:10-2 | |  |  | 1 | 108 | 1 | 4553 | 0.0462095 | 0.092419 | 2 |
|  |  | RAFL08-12-H04 | At2g26690 / nitrate transporter -related | |  |  |  |  |  | | --- | --- | --- | --- | --- | |  |  |  |  |  | | RAFL08-12-H04 ,At2g26690  nitrate transporter (NTP2) identical to nitrate transporter (ntp2) [Arabidopsis thaliana] GI:4490321 | | | | | | |
| 1\_17370001\_17400000 | | |  |  | A | B | C | D | P | P' | N |
|  | Cluster:6-2 | |  |  | 1 | 175 | 0 | 4487 | 0.03774394 | 0.03774394 | 1 |
|  |  | RAFL05-14-A14 | At1g48210 / serine/threonine protein kinase, putative | |  |  |  |  |  | | --- | --- | --- | --- | --- | |  |  |  |  |  | | RAFL05-14-A14 ,At1g48210  serine/threonine protein kinase, putative similar to Pto kinase interactor 1 [Lycopersicon esculentum] gi|3668069|gb|AAC61805; contains protein kinase domain, Pfam:PF00069 | | | | | | |
| 5\_15000001\_18000000 | | |  |  | A | B | C | D | P | P' | N |
|  | Cluster:0-0 | |  |  | 14 | 22 | 93 | 4534 | 1.1793967E-14 | 3.1843712E-13 | 27 |
|  |  | RAFL06-14-C19 | At5g38410 / ribulose bisphosphate carboxylase small chain 3b precursor (RuBisCO small subunit 3b) (sp|P10798) | |  |  |  |  |  | | --- | --- | --- | --- | --- | |  |  |  |  |  | | At5g38410 ,RAFL06-14-C19  ribulose bisphosphate carboxylase small chain 3B / RuBisCO small subunit 3B (RBCS-3B) (ATS3B) identical to SP|P10798 Ribulose bisphosphate carboxylase small chain 3B, chloroplast precursor (EC 4.1.1.39) (RuBisCO small subunit 3B) {Arabidopsis thaliana} | | | | | | |
|  |  | RAFL09-16-C21 | At5g38410 / ribulose bisphosphate carboxylase small chain 3b precursor (RuBisCO small subunit 3b) (sp|P10798) | |  |  |  |  |  | | --- | --- | --- | --- | --- | |  |  |  |  |  | | At5g38410 ,RAFL09-16-C21  ribulose bisphosphate carboxylase small chain 3B / RuBisCO small subunit 3B (RBCS-3B) (ATS3B) identical to SP|P10798 Ribulose bisphosphate carboxylase small chain 3B, chloroplast precursor (EC 4.1.1.39) (RuBisCO small subunit 3B) {Arabidopsis thaliana} | | | | | | |
|  |  | RAFL06-13-H11 | At5g38430 / ribulose bisphosphate carboxylase small chain 1b precursor (RuBisCO small subunit 1b) (sp|P10796) | |  |  |  |  |  | | --- | --- | --- | --- | --- | |  |  |  |  |  | | RAFL06-13-H11 ,At5g38430  ribulose bisphosphate carboxylase small chain 1B / RuBisCO small subunit 1B (RBCS-1B) (ATS1B) identical to SP|P10796 Ribulose bisphosphate carboxylase small chain 1B, chloroplast precursor (EC 4.1.1.39) (RuBisCO small subunit 1B) {Arabidopsis thaliana} | | | | | | |
|  |  | RAFL04-15-J15 | At5g38410 / ribulose bisphosphate carboxylase small chain 3b precursor (RuBisCO small subunit 3b) (sp|P10798) | |  |  |  |  |  | | --- | --- | --- | --- | --- | |  |  |  |  |  | | At5g38410 ,RAFL04-15-J15  ribulose bisphosphate carboxylase small chain 3B / RuBisCO small subunit 3B (RBCS-3B) (ATS3B) identical to SP|P10798 Ribulose bisphosphate carboxylase small chain 3B, chloroplast precursor (EC 4.1.1.39) (RuBisCO small subunit 3B) {Arabidopsis thaliana} | | | | | | |
|  |  | RAFL06-10-C06 | At5g42530 / expressed protein | |  |  |  |  |  | | --- | --- | --- | --- | --- | |  |  |  |  |  | | At5g42530 ,RAFL06-10-C06  expressed protein | | | | | | |
|  |  | RAFL06-10-O15 | At5g38420 / ribulose bisphosphate carboxylase small chain 2b precursor (RuBisCO small subunit 2b) (sp|P10797) | |  |  |  |  |  | | --- | --- | --- | --- | --- | |  |  |  |  |  | | At5g38420 ,RAFL06-10-O15  ribulose bisphosphate carboxylase small chain 2B / RuBisCO small subunit 2B (RBCS-2B) (ATS2B) identical to SP|P10797 Ribulose bisphosphate carboxylase small chain 2B, chloroplast precursor (EC 4.1.1.39) (RuBisCO small subunit 2B) {Arabidopsis thaliana} | | | | | | |
|  |  | RAFL07-12-I10 | At5g42010 / expressed protein | |  |  |  |  |  | | --- | --- | --- | --- | --- | |  |  |  |  |  | | At5g42010 ,RAFL07-12-I10  WD-40 repeat family protein contains Pfam PF00400: WD domain, G-beta repeat; similar to WD-repeat protein 5 (WD repeat protein BIG-3) (SP: Q9UGP9) [Homo sapiens] | | | | | | |
|  |  | RAFL08-17-J10 | At5g38420 / ribulose bisphosphate carboxylase small chain 2b precursor (RuBisCO small subunit 2b) (sp|P10797) | |  |  |  |  |  | | --- | --- | --- | --- | --- | |  |  |  |  |  | | At5g38420 ,RAFL08-17-J10  ribulose bisphosphate carboxylase small chain 2B / RuBisCO small subunit 2B (RBCS-2B) (ATS2B) identical to SP|P10797 Ribulose bisphosphate carboxylase small chain 2B, chloroplast precursor (EC 4.1.1.39) (RuBisCO small subunit 2B) {Arabidopsis thaliana} | | | | | | |
|  |  | RAFL06-08-L09 | At5g38420 / ribulose bisphosphate carboxylase small chain 2b precursor (RuBisCO small subunit 2b) (sp|P10797) | |  |  |  |  |  | | --- | --- | --- | --- | --- | |  |  |  |  |  | | At5g38420 ,RAFL06-08-L09  ribulose bisphosphate carboxylase small chain 2B / RuBisCO small subunit 2B (RBCS-2B) (ATS2B) identical to SP|P10797 Ribulose bisphosphate carboxylase small chain 2B, chloroplast precursor (EC 4.1.1.39) (RuBisCO small subunit 2B) {Arabidopsis thaliana} | | | | | | |
|  |  | RAFL06-14-L16 | At5g38430 / ribulose bisphosphate carboxylase small chain 1b precursor (RuBisCO small subunit 1b) (sp|P10796) | |  |  |  |  |  | | --- | --- | --- | --- | --- | |  |  |  |  |  | | At5g38430 ,RAFL06-14-L16  ribulose bisphosphate carboxylase small chain 1B / RuBisCO small subunit 1B (RBCS-1B) (ATS1B) identical to SP|P10796 Ribulose bisphosphate carboxylase small chain 1B, chloroplast precursor (EC 4.1.1.39) (RuBisCO small subunit 1B) {Arabidopsis thaliana} | | | | | | |
|  |  | RAFL09-09-K05 | At5g38410 / ribulose bisphosphate carboxylase small chain 3b precursor (RuBisCO small subunit 3b) (sp|P10798) | |  |  |  |  |  | | --- | --- | --- | --- | --- | |  |  |  |  |  | | RAFL09-09-K05 ,At5g38410  ribulose bisphosphate carboxylase small chain 3B / RuBisCO small subunit 3B (RBCS-3B) (ATS3B) identical to SP|P10798 Ribulose bisphosphate carboxylase small chain 3B, chloroplast precursor (EC 4.1.1.39) (RuBisCO small subunit 3B) {Arabidopsis thaliana} | | | | | | |
|  |  | RAFL07-11-L12 | At5g38420 / ribulose bisphosphate carboxylase small chain 2b precursor (RuBisCO small subunit 2b) (sp|P10797) | |  |  |  |  |  | | --- | --- | --- | --- | --- | |  |  |  |  |  | | At5g38420 ,RAFL07-11-L12  ribulose bisphosphate carboxylase small chain 2B / RuBisCO small subunit 2B (RBCS-2B) (ATS2B) identical to SP|P10797 Ribulose bisphosphate carboxylase small chain 2B, chloroplast precursor (EC 4.1.1.39) (RuBisCO small subunit 2B) {Arabidopsis thaliana} | | | | | | |
|  |  | RAFL07-14-L17 | At5g38420 / ribulose bisphosphate carboxylase small chain 2b precursor (RuBisCO small subunit 2b) (sp|P10797) | |  |  |  |  |  | | --- | --- | --- | --- | --- | |  |  |  |  |  | | At5g38420 ,RAFL07-14-L17  ribulose bisphosphate carboxylase small chain 2B / RuBisCO small subunit 2B (RBCS-2B) (ATS2B) identical to SP|P10797 Ribulose bisphosphate carboxylase small chain 2B, chloroplast precursor (EC 4.1.1.39) (RuBisCO small subunit 2B) {Arabidopsis thaliana} | | | | | | |
|  |  | RAFL04-13-P21 | At5g42530 / expressed protein | |  |  |  |  |  | | --- | --- | --- | --- | --- | |  |  |  |  |  | | At5g42530 ,RAFL04-13-P21  expressed protein | | | | | | |
|  | Cluster:6-1 | |  |  | 14 | 301 | 93 | 4255 | 0.016745122 | 0.45211828 | 27 |
|  |  | RAFL05-10-P24 | At5g40890 / CLC-a chloride channel protein | |  |  |  |  |  | | --- | --- | --- | --- | --- | |  |  |  |  |  | | At5g40890 ,RAFL05-10-P24  chloride channel protein (CLC-a) identical to GI:1742952 (gb|AAC05742.1) | | | | | | |
|  |  | RAFL05-14-B01 | At5g42980 / thioredoxin H-type 3 (TRX-H-3) | |  |  |  |  |  | | --- | --- | --- | --- | --- | |  |  |  |  |  | | RAFL05-14-B01 ,At5g42980  thioredoxin H-type 3 (TRX-H-3) (GIF1) identical to SP|Q42403 Thioredoxin H-type 3 (TRX-H-3) {Arabidopsis thaliana}; identical to cDNA (GIF1) mRNA for thioredoxin GI:992961 | | | | | | |
|  |  | RAFL09-06-P04 | At5g39730 / avirulence induced gene (AIG) - like protein | |  |  |  |  |  | | --- | --- | --- | --- | --- | |  |  |  |  |  | | At5g39730 ,RAFL09-06-P04  avirulence-responsive protein-related / avirulence induced gene (AIG) protein-related similar to SP|P54121 AIG2 protein {Arabidopsis thaliana} | | | | | | |
|  |  | RAFL05-14-O08 | At5g39950 / thioredoxin H-type 2 (TRX-H-2) | |  |  |  |  |  | | --- | --- | --- | --- | --- | |  |  |  |  |  | | At5g39950 ,RAFL05-14-O08  thioredoxin H-type 2 (TRX-H-2) (Gif2) identical to SP|Q38879 Thioredoxin H-type 2 (TRX-H-2) {Arabidopsis thaliana}; identical to cDNA (Gif2) mRNA for thioredoxin GI:992963 | | | | | | |
|  |  | RAFL04-20-N11 | At5g41370 / TFIIH basal transcription factor complex helicase XPB subunit, putative | |  |  |  |  |  | | --- | --- | --- | --- | --- | |  |  |  |  |  | | At5g41370 ,RAFL04-20-N11  DNA repair protein, putative / TFIIH basal transcription factor complex helicase XPB subunit, putative (XPB1) contains Pfam profile PF00271:Helicase conserved C-terminal domain; identical to cDNA putative DNA repair protein (XPB1) GI:10314019 | | | | | | |
|  |  | RAFL06-09-A21 | At5g40580 / 20S proteasome beta subunit B (PBB2) | |  |  |  |  |  | | --- | --- | --- | --- | --- | |  |  |  |  |  | | At5g40580 ,RAFL06-09-A21  20S proteasome beta subunit B (PBB2) (PRCFC) identical to 20S proteasome beta subunit PBB2 [Arabidopsis thaliana] GI:3421104, cDNA proteasome subunit prcfc GI:2511575 | | | | | | |
|  |  | RAFL07-12-M14 | At5g42390 / pitrilysin | |  |  |  |  |  | | --- | --- | --- | --- | --- | |  |  |  |  |  | | At5g42390 ,RAFL07-12-M14  metalloendopeptidase identical to chloroplast processing enzyme metalloendopeptidase [Arabidopsis thaliana] gi|2827039|gb|AAC39482 | | | | | | |
|  |  | RAFL06-16-H05 | At5g40730 / arabinogalactan-protein (AGP24) | |  |  |  |  |  | | --- | --- | --- | --- | --- | |  |  |  |  |  | | RAFL06-16-H05 ,At5g40730  arabinogalactan-protein (AGP24) | | | | | | |
|  |  | RAFL09-12-L08 | At5g38470 / DNA repair protein RAD23, putative | |  |  |  |  |  | | --- | --- | --- | --- | --- | |  |  |  |  |  | | RAFL09-12-L08 ,At5g38470  DNA repair protein RAD23, putative similar to DNA repair by nucleotide excision (NER) RAD23 protein, isoform I GI:1914683 from [Daucus carota] | | | | | | |
|  |  | RAFL06-12-E24 | At5g42790 / 20S proteasome alpha subunit F1 (PAF1) | |  |  |  |  |  | | --- | --- | --- | --- | --- | |  |  |  |  |  | | At5g42790 ,RAFL06-12-E24  20S proteasome alpha subunit F1 (PAF1) (gb|AAC32062.1) | | | | | | |
|  |  | RAFL11-01-O04 | At5g42520 / expressed protein | |  |  |  |  |  | | --- | --- | --- | --- | --- | |  |  |  |  |  | | RAFL11-01-O04 ,At5g42520  expressed protein | | | | | | |
|  |  | RAFL11-10-D22 | At5g43280 / enoyl-CoA hydratase/isomerase family | |  |  |  |  |  | | --- | --- | --- | --- | --- | |  |  |  |  |  | | RAFL11-10-D22 ,At5g43280  enoyl-CoA hydratase/isomerase family protein similar to Delta 3,5-delta2,4-dienoyl-CoA isomerase, mitochondrial (ECH1) from Rattus norvegicus [SP|Q62651], from Homo sapiens [SP|Q13011]; contains Pfam profile PF00378 enoyl-CoA hydratase/isomerase family protein | | | | | | |
|  |  | RAFL05-07-C04 | At5g40740 / expressed protein | |  |  |  |  |  | | --- | --- | --- | --- | --- | |  |  |  |  |  | | RAFL05-07-C04 ,At5g40740  expressed protein | | | | | | |
|  |  | RAFL04-13-C05 | At5g43780 / ATP sulfurylase precursor (gb|AAD26634.1) | |  |  |  |  |  | | --- | --- | --- | --- | --- | |  |  |  |  |  | | At5g43780 ,RAFL04-13-C05  sulfate adenylyltransferase 4 / ATP-sulfurylase 4 (APS4) identical to ATP sulfurylase precursor (APS4) [Arabidopsis thaliana] GI:4633131 | | | | | | |
|  | Cluster:8-0 | |  |  | 6 | 103 | 101 | 4453 | 0.03824088 | 1.0325037 | 27 |
|  |  | RAFL05-19-D06 | At5g39590 / expressed protein | |  |  |  |  |  | | --- | --- | --- | --- | --- | |  |  |  |  |  | | At5g39590 ,RAFL05-19-D06  expressed protein | | | | | | |
|  |  | RAFL05-13-B03 | At5g43850 / expressed protein | |  |  |  |  |  | | --- | --- | --- | --- | --- | |  |  |  |  |  | | RAFL05-13-B03 ,At5g43850  acireductone dioxygenase (ARD/ARD') family protein similar to iron-deficiency induced gene [Hordeum vulgare] GI:14522834, SIPL [Homo sapiens] GI:16551383; contains Pfam profile PF03079: ARD/ARD' family | | | | | | |
|  |  | RAFL05-05-N17 | At5g41040 / transferase family | |  |  |  |  |  | | --- | --- | --- | --- | --- | |  |  |  |  |  | | At5g41040 ,RAFL05-05-N17  transferase family protein similar to hypersensitivity-related gene product HSR201 - Nicotiana tabacum, EMBL:X95343; contains Pfam transferase family domain PF00248 | | | | | | |
|  |  | RAFL08-08-L16 | At5g39590 / expressed protein | |  |  |  |  |  | | --- | --- | --- | --- | --- | |  |  |  |  |  | | RAFL08-08-L16 ,At5g39590  expressed protein | | | | | | |
|  |  | RAFL04-17-M08 | At5g43450 / 2-oxoglutarate-dependent dioxygenase, putative | |  |  |  |  |  | | --- | --- | --- | --- | --- | |  |  |  |  |  | | RAFL04-17-M08 ,At5g43450  2-oxoglutarate-dependent dioxygenase, putative similar to 2A6 (GI:599622) and tomato ethylene synthesis regulatory protein E8 (SP|P10967) | | | | | | |
|  |  | RAFL06-15-G18 | At5g42380 / calmodulin-related protein, putative | |  |  |  |  |  | | --- | --- | --- | --- | --- | |  |  |  |  |  | | RAFL06-15-G18 ,At5g42380  calmodulin-related protein, putative similar to regulator of gene silencing calmodulin-related protein GI:12963415 from [Nicotiana tabacum] | | | | | | |
| 5\_900001\_930000 | | |  |  | A | B | C | D | P | P' | N |
|  | Cluster:8-1 | |  |  | 1 | 161 | 0 | 4501 | 0.034741584 | 0.034741584 | 1 |
|  |  | RAFL05-04-E03 | At5g03560 / expressed protein | |  |  |  |  |  | | --- | --- | --- | --- | --- | |  |  |  |  |  | | At5g03560 ,RAFL05-04-E03  expressed protein | | | | | | |
| 1\_9750001\_9780000 | | |  |  | A | B | C | D | P | P' | N |
|  | Cluster:6-2 | |  |  | 1 | 175 | 0 | 4487 | 0.03774394 | 0.03774394 | 1 |
|  |  | RAFL03-05-O03 | At1g28050 / CONSTANS B-box zinc finger family protein | |  |  |  |  |  | | --- | --- | --- | --- | --- | |  |  |  |  |  | | At1g28050 ,RAFL03-05-O03  zinc finger (B-box type) family protein | | | | | | |
| 4\_8700001\_8730000 | | |  |  | A | B | C | D | P | P' | N |
|  | Cluster:8-1 | |  |  | 1 | 161 | 0 | 4501 | 0.034741584 | 0.034741584 | 1 |
|  |  | RAFL08-16-G17 | At4g17500 / ethylene responsive element binding factor 1 (frameshift !) | |  |  |  |  |  | | --- | --- | --- | --- | --- | |  |  |  |  |  | | RAFL08-16-G17 ,At4g17500  ethylene-responsive element-binding protein 1 (ERF1) / EREBP-2 protein identical to SP|O80337 Ethylene responsive element binding factor 1 (EREBP-2 protein) [Arabidopsis thaliana]; a false single bp exon was added to circumvent a single basepair insertion in the genomic sequence, supported by cDNA/genome alignment. | | | | | | |
| 4\_16560001\_16590000 | | |  |  | A | B | C | D | P | P' | N |
|  | Cluster:8-0 | |  |  | 1 | 108 | 0 | 4554 | 0.02337551 | 0.02337551 | 1 |
|  |  | RAFL05-02-B21 | At4g37430 / cytochrome P450 91A2 | |  |  |  |  |  | | --- | --- | --- | --- | --- | |  |  |  |  |  | | RAFL05-02-B21 ,At4g37430  cytochrome P450 81F1 (CYP81F1) (CYP91A2) identical to cytochrome P450 81F1 (91A2) (SP:O65790) [Arabidopsis thaliana] | | | | | | |
| 2\_12000001\_12030000 | | |  |  | A | B | C | D | P | P' | N |
|  | Cluster:8-1 | |  |  | 1 | 161 | 0 | 4501 | 0.034741584 | 0.034741584 | 1 |
|  |  | RAFL09-12-N06 | At2g28305 / expressed protein | |  |  |  |  |  | | --- | --- | --- | --- | --- | |  |  |  |  |  | | At2g28305 ,RAFL09-12-N06  expressed protein contains Pfam profile PF03641: decarboxylase family protein | | | | | | |
| 5\_13710001\_13740000 | | |  |  | A | B | C | D | P | P' | N |
|  | Cluster:6-2 | |  |  | 1 | 175 | 0 | 4487 | 0.03774394 | 0.03774394 | 1 |
|  |  | RAFL05-02-H10 | At5g35840 / phytochrome C (PHYC) | |  |  |  |  |  | | --- | --- | --- | --- | --- | |  |  |  |  |  | | RAFL05-02-H10 ,At5g35840  phytochrome C (PHYC) identical to SP|P14714 Phytochrome C {Arabidopsis thaliana} | | | | | | |
| 1\_7920001\_7950000 | | |  |  | A | B | C | D | P | P' | N |
|  | Cluster:3-1 | |  |  | 1 | 215 | 0 | 4447 | 0.04632211 | 0.04632211 | 1 |
|  |  | RAFL04-18-L02 | At1g22500 / RING-H2 zinc finger protein ATL5 -related | |  |  |  |  |  | | --- | --- | --- | --- | --- | |  |  |  |  |  | | At1g22500 ,RAFL04-18-L02  zinc finger (C3HC4-type RING finger) family protein contains Pfam profile: PF00097: Zinc finger, C3HC4 type (RING finger) | | | | | | |
| 1\_18900001\_19200000 | | |  |  | A | B | C | D | P | P' | N |
|  | Cluster:0-1 | |  |  | 4 | 102 | 7 | 4550 | 7.364662E-5 | 5.155263E-4 | 7 |
|  |  | RAFL05-04-D24 | At1g52230 / photosystem I subunit VI precursor | |  |  |  |  |  | | --- | --- | --- | --- | --- | |  |  |  |  |  | | At1g52230 ,RAFL05-04-D24  photosystem I reaction center subunit VI, chloroplast, putative / PSI-H, putative (PSAH2) identical to SP|Q9SUI6; similar to PSI-H precursor [Nicotiana sylvestris] GI:407355; contains Pfam profile PF03244: Photosystem I reaction centre subunit VI | | | | | | |
|  |  | RAFL05-03-B20 | At1g52400 / glycosyl hydrolase family 1, beta-glucosidase (BG1) | |  |  |  |  |  | | --- | --- | --- | --- | --- | |  |  |  |  |  | | RAFL05-03-B20 ,At1g52400  glycosyl hydrolase family 1 protein / beta-glucosidase, putative (BG1) contains Pfam PF00232 : Glycosyl hydrolase family 1 domain; TIGRFAM TIGR01233: 6-phospho-beta-galactosidase; identical to GI:6651430 from [Arabidopsis thaliana] | | | | | | |
|  |  | RAFL07-10-G07 | At1g52400 / glycosyl hydrolase family 1, beta-glucosidase (BG1) | |  |  |  |  |  | | --- | --- | --- | --- | --- | |  |  |  |  |  | | At1g52400 ,RAFL07-10-G07  glycosyl hydrolase family 1 protein / beta-glucosidase, putative (BG1) contains Pfam PF00232 : Glycosyl hydrolase family 1 domain; TIGRFAM TIGR01233: 6-phospho-beta-galactosidase; identical to GI:6651430 from [Arabidopsis thaliana] | | | | | | |
|  |  | RAFL07-11-K16 | At1g52000 / jacalin lectin family | |  |  |  |  |  | | --- | --- | --- | --- | --- | |  |  |  |  |  | | At1g52000 ,RAFL07-11-K16  jacalin lectin family protein similar to myrosinase binding protein [Brassica napus] GI:1711296, myrosinase-binding protein homolog [Arabidopsis thaliana] GI:2997767; contains Pfam profile: PF01419 jacalin-like lectin domain | | | | | | |
|  | Cluster:1-0 | |  |  | 2 | 147 | 9 | 4505 | 0.04617144 | 0.32320008 | 7 |
|  |  | RAFL08-09-I24 | At1g52040 / jacalin lectin family | |  |  |  |  |  | | --- | --- | --- | --- | --- | |  |  |  |  |  | | RAFL08-09-I24 ,At1g52040  jacalin lectin family protein nearly identical to myrosinase-binding protein homolog GI:2997767 from [Arabidopsis thaliana]; contains Pfam profile PF01419 jacalin-like lectin domain; identical to cDNA myrosinase-binding protein homolog GI:2997766 | | | | | | |
|  |  | RAFL09-14-K04 | At1g52040 / jacalin lectin family | |  |  |  |  |  | | --- | --- | --- | --- | --- | |  |  |  |  |  | | At1g52040 ,RAFL09-14-K04  jacalin lectin family protein nearly identical to myrosinase-binding protein homolog GI:2997767 from [Arabidopsis thaliana]; contains Pfam profile PF01419 jacalin-like lectin domain; identical to cDNA myrosinase-binding protein homolog GI:2997766 | | | | | | |
| 3\_6210001\_6240000 | | |  |  | A | B | C | D | P | P' | N |
|  | Cluster:4-1 | |  |  | 2 | 308 | 2 | 4351 | 0.024166461 | 0.07249938 | 3 |
|  |  | RAFL08-11-G19 | At3g18165 / expressed protein | |  |  |  |  |  | | --- | --- | --- | --- | --- | |  |  |  |  |  | | At3g18165 ,RAFL08-11-G19  expressed protein similar to DAM1 (GI:3985930) [Homo sapiens]; contains Pfam profile PF05700: Breast carcinoma amplified sequence 2 (BCAS2) | | | | | | |
|  |  | RAFL05-04-O11 | At3g18190 / chaperonin, putative | |  |  |  |  |  | | --- | --- | --- | --- | --- | |  |  |  |  |  | | At3g18190 ,RAFL05-04-O11  chaperonin, putative similar to SWISS-PROT:P50991- T-complex protein 1, delta subunit (TCP-1-delta) [Homo sapiens]; contains Pfam:PF00118 domain, TCP-1/cpn60 chaperonin family | | | | | | |
| 1\_25740001\_25770000 | | |  |  | A | B | C | D | P | P' | N |
|  | Cluster:8-0 | |  |  | 1 | 108 | 1 | 4553 | 0.0462095 | 0.092419 | 2 |
|  |  | RAFL09-13-J20 | At1g69410 / Eukaryotic initiation factor 5A -related | |  |  |  |  |  | | --- | --- | --- | --- | --- | |  |  |  |  |  | | RAFL09-13-J20 ,At1g69410  eukaryotic translation initiation factor 5A, putative / eIF-5A, putative strong similarity to eukaryotic initiation factor 5A (2) (Nicotiana plumbaginifolia) GI:19702, SP|Q9AXQ6| Eukaryotic translation initiation factor 5A-1 (eIF-5A 1) {Lycopersicon esculentum} | | | | | | |
| 1\_8430001\_8460000 | | |  |  | A | B | C | D | P | P' | N |
|  | Cluster:6-2 | |  |  | 1 | 175 | 0 | 4487 | 0.03774394 | 0.03774394 | 1 |
|  |  | RAFL08-09-I21 | At1g23880 / expressed protein | |  |  |  |  |  | | --- | --- | --- | --- | --- | |  |  |  |  |  | | At1g23880 ,RAFL08-09-I21  NHL repeat-containing protein contains Pfam profile PF01436: NHL repeat | | | | | | |
| 1\_24000001\_24300000 | | |  |  | A | B | C | D | P | P' | N |
|  | Cluster:2-0 | |  |  | 3 | 147 | 11 | 4502 | 0.009149455 | 0.0823451 | 9 |
|  |  | RAFL09-07-F20 | At1g65930 / isocitrate dehydrogenase (NADP+), putative | |  |  |  |  |  | | --- | --- | --- | --- | --- | |  |  |  |  |  | | At1g65930 ,RAFL09-07-F20  isocitrate dehydrogenase, putative / NADP+ isocitrate dehydrogenase, putative strong similarity to isocitrate dehydrogenase SP|Q40345 from [Medicago sativa] | | | | | | |
|  |  | RAFL09-06-L20 | At1g65930 / isocitrate dehydrogenase (NADP+), putative | |  |  |  |  |  | | --- | --- | --- | --- | --- | |  |  |  |  |  | | At1g65930 ,RAFL09-06-L20  isocitrate dehydrogenase, putative / NADP+ isocitrate dehydrogenase, putative strong similarity to isocitrate dehydrogenase SP|Q40345 from [Medicago sativa] | | | | | | |
|  |  | RAFL09-11-A18 | At1g65930 / isocitrate dehydrogenase (NADP+), putative | |  |  |  |  |  | | --- | --- | --- | --- | --- | |  |  |  |  |  | | RAFL09-11-A18 ,At1g65930  isocitrate dehydrogenase, putative / NADP+ isocitrate dehydrogenase, putative strong similarity to isocitrate dehydrogenase SP|Q40345 from [Medicago sativa] | | | | | | |
|  | Cluster:6-1 | |  |  | 4 | 311 | 10 | 4338 | 0.011896291 | 0.10706662 | 9 |
|  |  | RAFL04-09-A20 | At1g65720 / expressed protein | |  |  |  |  |  | | --- | --- | --- | --- | --- | |  |  |  |  |  | | At1g65720 ,RAFL04-09-A20  expressed protein | | | | | | |
|  |  | RAFL04-17-H05 | At1g65650 / expressed protein | |  |  |  |  |  | | --- | --- | --- | --- | --- | |  |  |  |  |  | | At1g65650 ,RAFL04-17-H05  ubiquitin carboxyl-terminal hydrolase family 1 protein similar to 26S proteasome regulatory complex subunit p37A [Drosophila melanogaster] GI:6434962; contains Pfam profile PF01088: Ubiquitin carboxyl-terminal hydrolase, family 1 | | | | | | |
|  |  | RAFL04-20-K18 | At1g65980 / type 2 peroxiredoxin -related | |  |  |  |  |  | | --- | --- | --- | --- | --- | |  |  |  |  |  | | RAFL04-20-K18 ,At1g65980  peroxiredoxin type 2, putative strong similarity to type 2 peroxiredoxin [Brassica rapa subsp. pekinensis] GI:4928472; contains Pfam profile: PF00578 AhpC/TSA (alkyl hydroperoxide reductase and thiol-specific antioxidant) family | | | | | | |
|  |  | RAFL02-10-H06 | At1g65820 / microsomal glutathione s-transferase, putative | |  |  |  |  |  | | --- | --- | --- | --- | --- | |  |  |  |  |  | | RAFL02-10-H06 ,At1g65820  microsomal glutathione s-transferase, putative similar to MGST3\_HUMAN SP:O14880 | | | | | | |
| 2\_16890001\_16920000 | | |  |  | A | B | C | D | P | P' | N |
|  | Cluster:2-1 | |  |  | 2 | 242 | 2 | 4417 | 0.01525385 | 0.045761548 | 3 |
|  |  | RAFL06-13-E16 | At2g40630 / expressed protein | |  |  |  |  |  | | --- | --- | --- | --- | --- | |  |  |  |  |  | | At2g40630 ,RAFL06-13-E16  expressed protein | | | | | | |
|  |  | RAFL04-14-J20 | At2g40660 / methionyl-tRNA synthetase -related | |  |  |  |  |  | | --- | --- | --- | --- | --- | |  |  |  |  |  | | RAFL04-14-J20 ,At2g40660  tRNA-binding region domain-containing protein similar to SP|Q12904 Multisynthetase complex auxiliary component p43 [Contains: Endothelial-monocyte activating polypeptide II (EMAP-II) (Small inducible cytokine subfamily E member 1)] {Homo sapiens}; contains Pfam profile PF01588: Putative tRNA binding domain | | | | | | |
| 1\_660001\_690000 | | |  |  | A | B | C | D | P | P' | N |
|  | Cluster:5-2 | |  |  | 1 | 125 | 0 | 4537 | 0.027021231 | 0.027021231 | 1 |
|  |  | RAFL05-16-O07 | At1g02930 / glutathione transferase, putative | |  |  |  |  |  | | --- | --- | --- | --- | --- | |  |  |  |  |  | | At1g02930 ,RAFL05-16-O07  glutathione S-transferase, putative similar to glutathione S-transferase GI:860955 from [Hyoscyamus muticus] | | | | | | |
| 4\_11040001\_11070000 | | |  |  | A | B | C | D | P | P' | N |
|  | Cluster:7-2 | |  |  | 1 | 63 | 2 | 4597 | 0.040621255 | 0.12186376 | 3 |
|  |  | RAFL05-18-D20 | At4g23060 / expressed protein | |  |  |  |  |  | | --- | --- | --- | --- | --- | |  |  |  |  |  | | At4g23060 ,RAFL05-18-D20  calmodulin-binding family protein contains Pfam profile PF00612: IQ calmodulin-binding motif | | | | | | |
| 4\_6300001\_6330000 | | |  |  | A | B | C | D | P | P' | N |
|  | Cluster:10-2 | |  |  | 1 | 108 | 0 | 4554 | 0.02337551 | 0.02337551 | 1 |
|  |  | RAFL07-14-D12 | At4g12430 / trehalose-6-phosphate phosphatase, putative | |  |  |  |  |  | | --- | --- | --- | --- | --- | |  |  |  |  |  | | At4g12430 ,RAFL07-14-D12  trehalose-6-phosphate phosphatase, putative similar to trehalose-6-phosphate phosphatase (AtTPPB) [Arabidopsis thaliana] GI:2944180; contains Pfam profile PF02358: Trehalose-phosphatase | | | | | | |
| 1\_6600001\_6900000 | | |  |  | A | B | C | D | P | P' | N |
|  | Cluster:9-2 | |  |  | 3 | 64 | 6 | 4590 | 2.2394971E-4 | 0.001567648 | 7 |
|  |  | RAFL09-09-P15 | At1g19180 / expressed protein | |  |  |  |  |  | | --- | --- | --- | --- | --- | |  |  |  |  |  | | RAFL09-09-P15 ,At1g19180  expressed protein | | | | | | |
|  |  | RAFL06-10-F03 | At1g19180 / expressed protein | |  |  |  |  |  | | --- | --- | --- | --- | --- | |  |  |  |  |  | | RAFL06-10-F03 ,At1g19180  expressed protein | | | | | | |
|  |  | RAFL05-02-L02 | At1g19180 / expressed protein | |  |  |  |  |  | | --- | --- | --- | --- | --- | |  |  |  |  |  | | RAFL05-02-L02 ,At1g19180  expressed protein | | | | | | |
| 3\_15900001\_16200000 | | |  |  | A | B | C | D | P | P' | N |
|  | Cluster:8-0 | |  |  | 2 | 107 | 3 | 4551 | 0.005170766 | 0.020683063 | 4 |
|  |  | RAFL06-13-E03 | At3g44300 / nitrilase 2 | |  |  |  |  |  | | --- | --- | --- | --- | --- | |  |  |  |  |  | | RAFL06-13-E03 ,At3g44300  nitrilase 2 (NIT2) identical to SP|P32962 Nitrilase 2 (EC 3.5.5.1) {Arabidopsis thaliana} | | | | | | |
|  |  | RAFL08-10-H06 | At3g44300 / nitrilase 2 | |  |  |  |  |  | | --- | --- | --- | --- | --- | |  |  |  |  |  | | RAFL08-10-H06 ,At3g44300  nitrilase 2 (NIT2) identical to SP|P32962 Nitrilase 2 (EC 3.5.5.1) {Arabidopsis thaliana} | | | | | | |
| 1\_19920001\_19950000 | | |  |  | A | B | C | D | P | P' | N |
|  | Cluster:0-2 | |  |  | 2 | 77 | 1 | 4583 | 8.409994E-4 | 0.0016819988 | 2 |
|  |  | RAFL04-09-M24 | At1g54500 / rubredoxin -related | |  |  |  |  |  | | --- | --- | --- | --- | --- | |  |  |  |  |  | | At1g54500 ,RAFL04-09-M24  rubredoxin family protein similar to SP|P00270 Rubredoxin (Rd) {Desulfovibrio gigas}; contains Pfam profile PF00301: Rubredoxin | | | | | | |
|  |  | RAFL11-03-K23 | At1g54500 / rubredoxin -related | |  |  |  |  |  | | --- | --- | --- | --- | --- | |  |  |  |  |  | | At1g54500 ,RAFL11-03-K23  rubredoxin family protein similar to SP|P00270 Rubredoxin (Rd) {Desulfovibrio gigas}; contains Pfam profile PF00301: Rubredoxin | | | | | | |
| 5\_3390001\_3420000 | | |  |  | A | B | C | D | P | P' | N |
|  | Cluster:8-0 | |  |  | 1 | 108 | 1 | 4553 | 0.0462095 | 0.092419 | 2 |
|  |  | RAFL09-06-K06 | At5g10730 / expressed protein | |  |  |  |  |  | | --- | --- | --- | --- | --- | |  |  |  |  |  | | RAFL09-06-K06 ,At5g10730  expressed protein | | | | | | |
| 5\_13530001\_13560000 | | |  |  | A | B | C | D | P | P' | N |
|  | Cluster:2-2 | |  |  | 1 | 52 | 0 | 4610 | 0.011366073 | 0.011366073 | 1 |
|  |  | RAFL04-16-N11 | At5g35630 / glutamate-ammonia ligase (EC 6.3.1.2) precursor, chloroplast (clone lambdaAtgsl1) (pir||S18600) | |  |  |  |  |  | | --- | --- | --- | --- | --- | |  |  |  |  |  | | At5g35630 ,RAFL04-16-N11  glutamine synthetase (GS2) identical to glutamine synthetase, chloroplast precursor (glutamate-- ammonia ligase, GS2) [Arabidopsis thaliana] SWISS-PROT:Q43127 | | | | | | |
| 1\_2670001\_2700000 | | |  |  | A | B | C | D | P | P' | N |
|  | Cluster:3-0 | |  |  | 2 | 231 | 5 | 4425 | 0.044206142 | 0.2210307 | 5 |
|  |  | RAFL07-11-D02 | At1g08520 / magnesium-chelatase, subunit chlD, chloroplast (Mg-protoporphyrin IX chelatase) (CHLD), putative | |  |  |  |  |  | | --- | --- | --- | --- | --- | |  |  |  |  |  | | RAFL07-11-D02 ,At1g08520  magnesium-chelatase subunit chlD, chloroplast, putative / Mg-protoporphyrin IX chelatase, putative (CHLD) similar to Mg-chelatase SP|O24133 from Nicotiana tabacum, GB:AF014399 GI:2318116 from [Pisum sativum] | | | | | | |
|  |  | RAFL05-02-J02 | At1g08490 / nitrogen fixation protein. putative (NifS) | |  |  |  |  |  | | --- | --- | --- | --- | --- | |  |  |  |  |  | | RAFL05-02-J02 ,At1g08490  cysteine desulfurase, putative similar to nitrogen fixation protein (nifS) GB:D64004 GI:1001701 from [Synechocystis sp]; contains TIGRFAM TIGR01364: phosphoserine aminotransferase; contains Pfam PF00266: aminotransferase, class V | | | | | | |
| 3\_19980001\_20010000 | | |  |  | A | B | C | D | P | P' | N |
|  | Cluster:7-0 | |  |  | 2 | 245 | 2 | 4414 | 0.015618202 | 0.046854608 | 3 |
|  |  | RAFL03-05-E08 | At3g53990 / expressed protein | |  |  |  |  |  | | --- | --- | --- | --- | --- | |  |  |  |  |  | | At3g53990 ,RAFL03-05-E08  universal stress protein (USP) family protein contains Pfam PF00582: universal stress protein family | | | | | | |
|  |  | RAFL02-10-G21 | At3g53990 / expressed protein | |  |  |  |  |  | | --- | --- | --- | --- | --- | |  |  |  |  |  | | At3g53990 ,RAFL02-10-G21  universal stress protein (USP) family protein contains Pfam PF00582: universal stress protein family | | | | | | |
| 5\_15990001\_16020000 | | |  |  | A | B | C | D | P | P' | N |
|  | Cluster:6-1 | |  |  | 2 | 313 | 0 | 4348 | 0.004549907 | 0.004549907 | 1 |
|  |  | RAFL06-16-H05 | At5g40730 / arabinogalactan-protein (AGP24) | |  |  |  |  |  | | --- | --- | --- | --- | --- | |  |  |  |  |  | | RAFL06-16-H05 ,At5g40730  arabinogalactan-protein (AGP24) | | | | | | |
|  |  | RAFL05-07-C04 | At5g40740 / expressed protein | |  |  |  |  |  | | --- | --- | --- | --- | --- | |  |  |  |  |  | | RAFL05-07-C04 ,At5g40740  expressed protein | | | | | | |
| 1\_3390001\_3420000 | | |  |  | A | B | C | D | P | P' | N |
|  | Cluster:3-2 | |  |  | 1 | 36 | 1 | 4625 | 0.015808338 | 0.031616677 | 2 |
|  |  | RAFL04-12-M19 | At1g10360 / glutathione transferase, putative | |  |  |  |  |  | | --- | --- | --- | --- | --- | |  |  |  |  |  | | RAFL04-12-M19 ,At1g10360  glutathione S-transferase, putative similar to glutathione S-transferase (sp|Q03666|GTX4\_TOBAC); similar to EST gb|H36275 gb:AB039930. | | | | | | |
| 3\_4860001\_4890000 | | |  |  | A | B | C | D | P | P' | N |
|  | Cluster:8-0 | |  |  | 1 | 108 | 0 | 4554 | 0.02337551 | 0.02337551 | 1 |
|  |  | RAFL08-09-C10 | At3g14560 / expressed protein | |  |  |  |  |  | | --- | --- | --- | --- | --- | |  |  |  |  |  | | At3g14560 ,RAFL08-09-C10  expressed protein | | | | | | |
| 1\_27510001\_27540000 | | |  |  | A | B | C | D | P | P' | N |
|  | Cluster:5-2 | |  |  | 1 | 125 | 0 | 4537 | 0.027021231 | 0.027021231 | 1 |
|  |  | RAFL05-13-F01 | At1g74100 / sulfotransferase family | |  |  |  |  |  | | --- | --- | --- | --- | --- | |  |  |  |  |  | | RAFL05-13-F01 ,At1g74100  sulfotransferase family protein similar to SP|P52837 Flavonol 4'-sulfotransferase (EC 2.8.2.-) (F4-ST) {Flaveria chloraefolia}; contains Pfam profile PF00685: Sulfotransferase domain | | | | | | |
| 3\_3900001\_4200000 | | |  |  | A | B | C | D | P | P' | N |
|  | Cluster:0-1 | |  |  | 4 | 102 | 15 | 4542 | 7.516408E-4 | 0.0075164083 | 10 |
|  |  | RAFL07-16-P05 | At3g12780 / phosphoglycerate kinase -related | |  |  |  |  |  | | --- | --- | --- | --- | --- | |  |  |  |  |  | | RAFL07-16-P05 ,At3g12780  phosphoglycerate kinase, putative similar to SP|P41758 Phosphoglycerate kinase, chloroplast precursor (EC 2.7.2.3) {Chlamydomonas reinhardtii}; contains Pfam profile PF00162: phosphoglycerate kinase | | | | | | |
|  |  | RAFL09-15-L04 | At3g12780 / phosphoglycerate kinase -related | |  |  |  |  |  | | --- | --- | --- | --- | --- | |  |  |  |  |  | | RAFL09-15-L04 ,At3g12780  phosphoglycerate kinase, putative similar to SP|P41758 Phosphoglycerate kinase, chloroplast precursor (EC 2.7.2.3) {Chlamydomonas reinhardtii}; contains Pfam profile PF00162: phosphoglycerate kinase | | | | | | |
|  |  | RAFL07-14-L16 | At3g12780 / phosphoglycerate kinase -related | |  |  |  |  |  | | --- | --- | --- | --- | --- | |  |  |  |  |  | | At3g12780 ,RAFL07-14-L16  phosphoglycerate kinase, putative similar to SP|P41758 Phosphoglycerate kinase, chloroplast precursor (EC 2.7.2.3) {Chlamydomonas reinhardtii}; contains Pfam profile PF00162: phosphoglycerate kinase | | | | | | |
|  |  | RAFL09-18-L22 | At3g12780 / phosphoglycerate kinase -related | |  |  |  |  |  | | --- | --- | --- | --- | --- | |  |  |  |  |  | | At3g12780 ,RAFL09-18-L22  phosphoglycerate kinase, putative similar to SP|P41758 Phosphoglycerate kinase, chloroplast precursor (EC 2.7.2.3) {Chlamydomonas reinhardtii}; contains Pfam profile PF00162: phosphoglycerate kinase | | | | | | |
|  | Cluster:8-1 | |  |  | 3 | 159 | 16 | 4485 | 0.026495453 | 0.26495454 | 10 |
|  |  | RAFL09-16-C16 | At3g12570 / expressed protein | |  |  |  |  |  | | --- | --- | --- | --- | --- | |  |  |  |  |  | | At3g12570 ,RAFL09-16-C16  expressed protein | | | | | | |
|  |  | RAFL07-09-M01 | At3g12740 / membrane protein common family | |  |  |  |  |  | | --- | --- | --- | --- | --- | |  |  |  |  |  | | RAFL07-09-M01 ,At3g12740  LEM3 (ligand-effect modulator 3) family protein / CDC50 family protein Similar to GI:4585976; GI:4966357; GI:4835763; GI:9757735 from [Arabidopsis thaliana] | | | | | | |
|  |  | RAFL06-16-B16 | At3g12740 / membrane protein common family | |  |  |  |  |  | | --- | --- | --- | --- | --- | |  |  |  |  |  | | RAFL06-16-B16 ,At3g12740  LEM3 (ligand-effect modulator 3) family protein / CDC50 family protein Similar to GI:4585976; GI:4966357; GI:4835763; GI:9757735 from [Arabidopsis thaliana] | | | | | | |
| 4\_1020001\_1050000 | | |  |  | A | B | C | D | P | P' | N |
|  | Cluster:10-1 | |  |  | 2 | 41 | 1 | 4619 | 2.4776903E-4 | 4.9553806E-4 | 2 |
|  |  | RAFL03-07-M07 | At4g02380 / late embryogenesis abundant protein family | |  |  |  |  |  | | --- | --- | --- | --- | --- | |  |  |  |  |  | | RAFL03-07-M07 ,At4g02380  late embryogenesis abundant 3 family protein / LEA3 family protein similar to several small proteins (~100 aa) that are induced by heat, auxin, ethylene and wounding such as Phaseolus aureus indole-3-acetic acid induced protein ARG (SW:32292); contains Pfam profile PF03242: Late embryogenesis abundant protein | | | | | | |
|  |  | RAFL06-13-N20 | At4g02380 / late embryogenesis abundant protein family | |  |  |  |  |  | | --- | --- | --- | --- | --- | |  |  |  |  |  | | RAFL06-13-N20 ,At4g02380  late embryogenesis abundant 3 family protein / LEA3 family protein similar to several small proteins (~100 aa) that are induced by heat, auxin, ethylene and wounding such as Phaseolus aureus indole-3-acetic acid induced protein ARG (SW:32292); contains Pfam profile PF03242: Late embryogenesis abundant protein | | | | | | |
| 5\_16080001\_16110000 | | |  |  | A | B | C | D | P | P' | N |
|  | Cluster:1-1 | |  |  | 2 | 103 | 2 | 4556 | 0.0029258758 | 0.008777628 | 3 |
|  |  | RAFL05-17-L14 | At5g40850 / urophorphyrin III methylase (gb|AAB92676.1) | |  |  |  |  |  | | --- | --- | --- | --- | --- | |  |  |  |  |  | | At5g40850 ,RAFL05-17-L14  urophorphyrin III methylase (UPM1) identical to urophorphyrin III methylase (GI:1146165) [Arabidopsis thaliana]; similar to s-adenosyl-L-methionine-dependent uroporphyrinogen III methyltransferase (GI:1490606) [Arabidopsis thaliana]; similar to Diphthine synthase (Diphtamide biosynthesis methyltransferase) (DPH5) (SP:P32469) [Saccharomyces cerevisiae]; contains Pfam PF00590 : Tetrapyrrole (Corrin/Porphyrin) Methylases domain; contains TIGRFAM PF00590: Tetrapyrrole (Corrin/Porphyrin) Methylases | | | | | | |
|  |  | RAFL11-03-K10 | At5g40850 / urophorphyrin III methylase (gb|AAB92676.1) | |  |  |  |  |  | | --- | --- | --- | --- | --- | |  |  |  |  |  | | At5g40850 ,RAFL11-03-K10  urophorphyrin III methylase (UPM1) identical to urophorphyrin III methylase (GI:1146165) [Arabidopsis thaliana]; similar to s-adenosyl-L-methionine-dependent uroporphyrinogen III methyltransferase (GI:1490606) [Arabidopsis thaliana]; similar to Diphthine synthase (Diphtamide biosynthesis methyltransferase) (DPH5) (SP:P32469) [Saccharomyces cerevisiae]; contains Pfam PF00590 : Tetrapyrrole (Corrin/Porphyrin) Methylases domain; contains TIGRFAM PF00590: Tetrapyrrole (Corrin/Porphyrin) Methylases | | | | | | |
| 1\_25920001\_25950000 | | |  |  | A | B | C | D | P | P' | N |
|  | Cluster:7-2 | |  |  | 1 | 63 | 1 | 4598 | 0.027264666 | 0.05452933 | 2 |
|  |  | RAFL06-15-N08 | At1g69840 / expressed protein | |  |  |  |  |  | | --- | --- | --- | --- | --- | |  |  |  |  |  | | At1g69840 ,RAFL06-15-N08  band 7 family protein strong similarity to hypersensitive-induced response protein [Zea mays] GI:7716466; contains Pfam profile PF01145: SPFH domain / Band 7 family | | | | | | |
| 2\_9300001\_9330000 | | |  |  | A | B | C | D | P | P' | N |
|  | Cluster:5-0 | |  |  | 1 | 76 | 1 | 4585 | 0.032756753 | 0.06551351 | 2 |
|  |  | RAFL11-05-A04 | At2g21970 / stress enhanced protein 2 (SEP2); nuclear gene for chloroplast product | |  |  |  |  |  | | --- | --- | --- | --- | --- | |  |  |  |  |  | | At2g21970 ,RAFL11-05-A04  stress enhanced protein 2 (SEP2) nearly identical to stress enhanced protein 2; SEP2 (GI:7384980) [Arabidopsis thaliana] | | | | | | |
| 3\_5880001\_5910000 | | |  |  | A | B | C | D | P | P' | N |
|  | Cluster:3-0 | |  |  | 1 | 232 | 0 | 4430 | 0.049967833 | 0.049967833 | 1 |
|  |  | RAFL11-02-I18 | At3g17210 / expressed protein | |  |  |  |  |  | | --- | --- | --- | --- | --- | |  |  |  |  |  | | At3g17210 ,RAFL11-02-I18  stable protein 1-related similar to stable protein 1 (GI:13445204) [Populus tremula] PMID:12376651; similar to pop3 peptide GB:AAC26526 from [Populus balsamifera subsp. trichocarpa X Populus deltoides] | | | | | | |
| 2\_7470001\_7500000 | | |  |  | A | B | C | D | P | P' | N |
|  | Cluster:3-0 | |  |  | 2 | 231 | 1 | 4429 | 0.007213332 | 0.014426664 | 2 |
|  |  | RAFL05-07-F05 | At2g17360 / 40S ribosomal protein S4 (RPS4A) | |  |  |  |  |  | | --- | --- | --- | --- | --- | |  |  |  |  |  | | At2g17360 ,RAFL05-07-F05  40S ribosomal protein S4 (RPS4A) contains ribosomal protein S4 signature from residues 8 to 22 | | | | | | |
|  |  | RAFL09-18-O15 | At2g17360 / 40S ribosomal protein S4 (RPS4A) | |  |  |  |  |  | | --- | --- | --- | --- | --- | |  |  |  |  |  | | RAFL09-18-O15 ,At2g17360  40S ribosomal protein S4 (RPS4A) contains ribosomal protein S4 signature from residues 8 to 22 | | | | | | |
|  | Cluster:5-0 | |  |  | 1 | 76 | 2 | 4584 | 0.04873567 | 0.09747134 | 2 |
|  |  | RAFL08-14-M16 | At2g17340 / expressed protein | |  |  |  |  |  | | --- | --- | --- | --- | --- | |  |  |  |  |  | | RAFL08-14-M16 ,At2g17340  pantothenate kinase-related contains Pfam domain, PF01937: Protein of unknown function; supported by tandem duplication of pantothenate kinase -related protein (TIGR\_Ath1:At2g17320) [Arabidopsis thaliana] | | | | | | |
| 1\_21000001\_24000000 | | |  |  | A | B | C | D | P | P' | N |
|  | Cluster:2-0 | |  |  | 10 | 140 | 77 | 4436 | 4.19244E-4 | 0.010900344 | 26 |
|  |  | RAFL07-12-D05 | At1g62380 / 1-aminocyclopropane-1-carboxylate oxidase (ACC oxidase), putative | |  |  |  |  |  | | --- | --- | --- | --- | --- | |  |  |  |  |  | | At1g62380 ,RAFL07-12-D05  1-aminocyclopropane-1-carboxylate oxidase, putative / ACC oxidase, putative nearly identical to ACC oxidase (ACC ox1) GI:587086 from [Brassica oleracea] | | | | | | |
|  |  | RAFL07-11-E17 | At1g62380 / 1-aminocyclopropane-1-carboxylate oxidase (ACC oxidase), putative | |  |  |  |  |  | | --- | --- | --- | --- | --- | |  |  |  |  |  | | At1g62380 ,RAFL07-11-E17  1-aminocyclopropane-1-carboxylate oxidase, putative / ACC oxidase, putative nearly identical to ACC oxidase (ACC ox1) GI:587086 from [Brassica oleracea] | | | | | | |
|  |  | RAFL09-06-C02 | At1g61520 / light-harvesting chlorophyll a/b binding protein | |  |  |  |  |  | | --- | --- | --- | --- | --- | |  |  |  |  |  | | RAFL09-06-C02 ,At1g61520  chlorophyll A-B binding protein / LHCI type III (LHCA3.1) nearly identical to PSI type III chlorophyll a/b-binding protein GI:430947; contains Pfam profile: PF00504 chlorophyll A-B binding protein; similar to PSI type III chlorophyll a/b-binding protein GI:430947 from [Arabidopsis thaliana] | | | | | | |
|  |  | RAFL07-15-M15 | At1g62380 / 1-aminocyclopropane-1-carboxylate oxidase (ACC oxidase), putative | |  |  |  |  |  | | --- | --- | --- | --- | --- | |  |  |  |  |  | | RAFL07-15-M15 ,At1g62380  1-aminocyclopropane-1-carboxylate oxidase, putative / ACC oxidase, putative nearly identical to ACC oxidase (ACC ox1) GI:587086 from [Brassica oleracea] | | | | | | |
|  |  | RAFL07-17-B02 | At1g59359 / 40S ribosomal protein S2 (RPS2B) | |  |  |  |  |  | | --- | --- | --- | --- | --- | |  |  |  |  |  | | RAFL07-17-B02 ,At1g59359  40S ribosomal protein S2 (RPS2B) similar to ribosomal protein S2 GI:430711 from [Drosophila melanogaster] | | | | | | |
|  |  | RAFL08-12-L17 | At1g62380 / 1-aminocyclopropane-1-carboxylate oxidase (ACC oxidase), putative | |  |  |  |  |  | | --- | --- | --- | --- | --- | |  |  |  |  |  | | At1g62380 ,RAFL08-12-L17  1-aminocyclopropane-1-carboxylate oxidase, putative / ACC oxidase, putative nearly identical to ACC oxidase (ACC ox1) GI:587086 from [Brassica oleracea] | | | | | | |
|  |  | RAFL06-07-N12 | At1g63690 / expressed protein | |  |  |  |  |  | | --- | --- | --- | --- | --- | |  |  |  |  |  | | At1g63690 ,RAFL06-07-N12  protease-associated (PA) domain-containing protein contains protease associated (PA) domain, Pfam:PF02225 | | | | | | |
|  |  | RAFL09-06-L01 | At1g62380 / 1-aminocyclopropane-1-carboxylate oxidase (ACC oxidase), putative | |  |  |  |  |  | | --- | --- | --- | --- | --- | |  |  |  |  |  | | RAFL09-06-L01 ,At1g62380  1-aminocyclopropane-1-carboxylate oxidase, putative / ACC oxidase, putative nearly identical to ACC oxidase (ACC ox1) GI:587086 from [Brassica oleracea] | | | | | | |
|  |  | RAFL08-13-D15 | At1g65295 / expressed protein | |  |  |  |  |  | | --- | --- | --- | --- | --- | |  |  |  |  |  | | RAFL08-13-D15 ,At1g65295  expressed protein | | | | | | |
|  |  | RAFL11-07-L13 | At1g62380 / 1-aminocyclopropane-1-carboxylate oxidase (ACC oxidase), putative | |  |  |  |  |  | | --- | --- | --- | --- | --- | |  |  |  |  |  | | RAFL11-07-L13 ,At1g62380  1-aminocyclopropane-1-carboxylate oxidase, putative / ACC oxidase, putative nearly identical to ACC oxidase (ACC ox1) GI:587086 from [Brassica oleracea] | | | | | | |
|  | Cluster:9-1 | |  |  | 7 | 89 | 80 | 4487 | 0.0019192069 | 0.04989938 | 26 |
|  |  | RAFL09-10-F18 | At1g58360 / amino acid permease I (AAP1) | |  |  |  |  |  | | --- | --- | --- | --- | --- | |  |  |  |  |  | | RAFL09-10-F18 ,At1g58360  amino acid permease I (AAP1) identical to amino acid permease I GI:22641 from [Arabidopsis thaliana] | | | | | | |
|  |  | RAFL08-08-O14 | At1g64110 / expressed protein | |  |  |  |  |  | | --- | --- | --- | --- | --- | |  |  |  |  |  | | RAFL08-08-O14 ,At1g64110  AAA-type ATPase family protein contains Pfam domain, PF00004: ATPase, AAA family | | | | | | |
|  |  | RAFL05-18-H15 | At1g64660 / methionine/cystathionine gamma lyase -related | |  |  |  |  |  | | --- | --- | --- | --- | --- | |  |  |  |  |  | | At1g64660 ,RAFL05-18-H15  Cys/Met metabolism pyridoxal-phosphate-dependent enzyme family protein similar to SP|P13254 Methionine gamma-lyase (EC 4.4.1.11) (L-methioninase) {Pseudomonas putida}; contains Pfam profile PF01053: Cys/Met metabolism PLP-dependent enzyme | | | | | | |
|  |  | RAFL04-19-J05 | At1g61800 / glucose-6-phosphate/phosphate-translocator, putative | |  |  |  |  |  | | --- | --- | --- | --- | --- | |  |  |  |  |  | | At1g61800 ,RAFL04-19-J05  glucose-6-phosphate/phosphate translocator, putative similar to glucose-6-phosphate/phosphate-translocator precursor GI:2997591 from [Pisum sativum] | | | | | | |
|  |  | RAFL11-02-N11 | At1g64660 / methionine/cystathionine gamma lyase -related | |  |  |  |  |  | | --- | --- | --- | --- | --- | |  |  |  |  |  | | At1g64660 ,RAFL11-02-N11  Cys/Met metabolism pyridoxal-phosphate-dependent enzyme family protein similar to SP|P13254 Methionine gamma-lyase (EC 4.4.1.11) (L-methioninase) {Pseudomonas putida}; contains Pfam profile PF01053: Cys/Met metabolism PLP-dependent enzyme | | | | | | |
|  |  | RAFL05-08-D17 | At1g63720 / expressed protein | |  |  |  |  |  | | --- | --- | --- | --- | --- | |  |  |  |  |  | | RAFL05-08-D17 ,At1g63720  expressed protein similar to putative protein GB:CAA18164 [Arabidopsis thaliana] | | | | | | |
|  |  | RAFL09-10-F14 | At1g63010 / expressed protein | |  |  |  |  |  | | --- | --- | --- | --- | --- | |  |  |  |  |  | | At1g63010 ,RAFL09-10-F14  SPX (SYG1/Pho81/XPR1) domain-containing protein contains Pfam profile PF03105: SPX domain | | | | | | |
| 1\_1530001\_1560000 | | |  |  | A | B | C | D | P | P' | N |
|  | Cluster:10-0 | |  |  | 1 | 23 | 1 | 4638 | 0.01026841 | 0.02053682 | 2 |
|  |  | RAFL06-10-C16 | At1g05340 / expressed protein | |  |  |  |  |  | | --- | --- | --- | --- | --- | |  |  |  |  |  | | RAFL06-10-C16 ,At1g05340  expressed protein | | | | | | |
|  | Cluster:9-1 | |  |  | 1 | 95 | 1 | 4566 | 0.040755685 | 0.08151137 | 2 |
|  |  | RAFL09-15-I16 | At1g05340 / expressed protein | |  |  |  |  |  | | --- | --- | --- | --- | --- | |  |  |  |  |  | | RAFL09-15-I16 ,At1g05340  expressed protein | | | | | | |
| 1\_7890001\_7920000 | | |  |  | A | B | C | D | P | P' | N |
|  | Cluster:9-1 | |  |  | 1 | 95 | 0 | 4567 | 0.020587604 | 0.020587604 | 1 |
|  |  | RAFL05-02-P11 | At1g22370 / glycosyltransferase family | |  |  |  |  |  | | --- | --- | --- | --- | --- | |  |  |  |  |  | | At1g22370 ,RAFL05-02-P11  UDP-glucoronosyl/UDP-glucosyl transferase family protein glycosyltransferase family | | | | | | |
| 4\_6300001\_6600000 | | |  |  | A | B | C | D | P | P' | N |
|  | Cluster:0-2 | |  |  | 2 | 77 | 4 | 4580 | 0.0040679234 | 0.020339618 | 5 |
|  |  | RAFL06-07-K01 | At4g12800 / probable photosystem I chain XI precursor | |  |  |  |  |  | | --- | --- | --- | --- | --- | |  |  |  |  |  | | At4g12800 ,RAFL06-07-K01  photosystem I reaction center subunit XI, chloroplast (PSI-L) / PSI subunit V identical to Photosystem I reaction center subunit XI, chloroplast precursor (PSI-L) (PSI subunit V) (Swiss-Prot:Q9SUI4) [Arabidopsis thaliana]; contains Pfam profile PF02605: photosystem I reaction center subunit XI; contains 2 transmembrane domains | | | | | | |
|  |  | RAFL06-16-J22 | At4g12800 / probable photosystem I chain XI precursor | |  |  |  |  |  | | --- | --- | --- | --- | --- | |  |  |  |  |  | | At4g12800 ,RAFL06-16-J22  photosystem I reaction center subunit XI, chloroplast (PSI-L) / PSI subunit V identical to Photosystem I reaction center subunit XI, chloroplast precursor (PSI-L) (PSI subunit V) (Swiss-Prot:Q9SUI4) [Arabidopsis thaliana]; contains Pfam profile PF02605: photosystem I reaction center subunit XI; contains 2 transmembrane domains | | | | | | |
| 4\_16650001\_16680000 | | |  |  | A | B | C | D | P | P' | N |
|  | Cluster:3-1 | |  |  | 1 | 215 | 0 | 4447 | 0.04632211 | 0.04632211 | 1 |
|  |  | RAFL05-15-N03 | At4g37660 / ribosomal - like protein | |  |  |  |  |  | | --- | --- | --- | --- | --- | |  |  |  |  |  | | RAFL05-15-N03 ,At4g37660  ribosomal protein L12 family protein ribosomal protein L12, Liberobacter africanum, U09675 | | | | | | |
| 2\_16320001\_16350000 | | |  |  | A | B | C | D | P | P' | N |
|  | Cluster:5-2 | |  |  | 1 | 125 | 0 | 4537 | 0.027021231 | 0.027021231 | 1 |
|  |  | RAFL07-13-I05 | At2g39220 / patatin family | |  |  |  |  |  | | --- | --- | --- | --- | --- | |  |  |  |  |  | | At2g39220 ,RAFL07-13-I05  patatin family protein similar to patatin-like latex allergen [Hevea brasiliensis][PMID:10589016]; contains patatin domain PF01734 | | | | | | |
| 4\_7200001\_7500000 | | |  |  | A | B | C | D | P | P' | N |
|  | Cluster:2-1 | |  |  | 3 | 241 | 7 | 4412 | 0.012910854 | 0.09037598 | 7 |
|  |  | RAFL06-10-J18 | At4g14320 / 60S ribosomal protein L36a/L44 (RPL36aB) | |  |  |  |  |  | | --- | --- | --- | --- | --- | |  |  |  |  |  | | RAFL06-10-J18 ,At4g14320  60S ribosomal protein L36a/L44 (RPL36aB) | | | | | | |
|  |  | RAFL07-16-L09 | At4g14420 / elicitor like protein | |  |  |  |  |  | | --- | --- | --- | --- | --- | |  |  |  |  |  | | At4g14420 ,RAFL07-16-L09  lesion inducing protein-related similar to ORF, able to induce HR-like lesions [Nicotiana tabacum] gi|1762945|gb|AAC49975 | | | | | | |
|  |  | RAFL05-21-A10 | At4g14870 / expressed protein | |  |  |  |  |  | | --- | --- | --- | --- | --- | |  |  |  |  |  | | At4g14870 ,RAFL05-21-A10  expressed protein | | | | | | |
|  | Cluster:1-1 | |  |  | 2 | 103 | 8 | 4550 | 0.02008839 | 0.14061873 | 7 |
|  |  | RAFL06-14-F04 | At4g14890 / ferredoxin family | |  |  |  |  |  | | --- | --- | --- | --- | --- | |  |  |  |  |  | | RAFL06-14-F04 ,At4g14890  ferredoxin family protein similar to SP|P00252 Ferredoxin I from Nostoc muscorum, SP|P00248 Ferredoxin from Mastigocladus laminosus, SP|P00244 Ferredoxin I from Aphanizomenon flos-aquae; contains Pfam profile PF00111 2Fe-2S iron-sulfur cluster binding domain | | | | | | |
|  |  | RAFL07-08-F02 | At4g14360 / dehydration-induced protein-related | |  |  |  |  |  | | --- | --- | --- | --- | --- | |  |  |  |  |  | | At4g14360 ,RAFL07-08-F02  dehydration-responsive protein-related similar to early-responsive to dehydration stress ERD3 protein [Arabidopsis thaliana] GI:15320410; contains Pfam profile PF03141: Putative methyltransferase | | | | | | |
| 3\_570001\_600000 | | |  |  | A | B | C | D | P | P' | N |
|  | Cluster:8-2 | |  |  | 1 | 61 | 2 | 4599 | 0.0393688 | 0.1181064 | 3 |
|  |  | RAFL07-13-P15 | At3g02750 / protein phosphatase 2C (PP2C) | |  |  |  |  |  | | --- | --- | --- | --- | --- | |  |  |  |  |  | | RAFL07-13-P15 ,At3g02750  protein phosphatase 2C family protein / PP2C family protein similar to protein phosphatase-2C; PP2C (GI:3643088) [Mesembryanthemum crystallinum]; contains Pfam PF00481 : Protein phosphatase 2C domain; | | | | | | |
| 1\_3510001\_3540000 | | |  |  | A | B | C | D | P | P' | N |
|  | Cluster:5-0 | |  |  | 1 | 76 | 2 | 4584 | 0.04873567 | 0.146207 | 3 |
|  |  | RAFL07-07-M15 | At1g10670 / ATP citrate-lyase -related | |  |  |  |  |  | | --- | --- | --- | --- | --- | |  |  |  |  |  | | At1g10670 ,RAFL07-07-M15  expressed protein | | | | | | |
| 2\_16140001\_16170000 | | |  |  | A | B | C | D | P | P' | N |
|  | Cluster:2-2 | |  |  | 1 | 52 | 1 | 4609 | 0.022605369 | 0.045210738 | 2 |
|  |  | RAFL04-20-B02 | At2g38750 / annexin -related | |  |  |  |  |  | | --- | --- | --- | --- | --- | |  |  |  |  |  | | At2g38750 ,RAFL04-20-B02  annexin 4 (ANN4) nearly identical to annexin (AnnAt4) [Arabidopsis thaliana] GI:6503084; contains Pfam profile PF00191: Annexin | | | | | | |
| 1\_30030001\_30060000 | | |  |  | A | B | C | D | P | P' | N |
|  | Cluster:9-2 | |  |  | 1 | 66 | 2 | 4594 | 0.042497892 | 0.12749368 | 3 |
|  |  | RAFL05-18-H12 | At1g80840 / WRKY family transcription factor | |  |  |  |  |  | | --- | --- | --- | --- | --- | |  |  |  |  |  | | RAFL05-18-H12 ,At1g80840  WRKY family transcription factor similar to WRKY transcription factor GB:BAA87058 GI:6472585 from [Nicotiana tabacum] | | | | | | |
| 1\_21900001\_22200000 | | |  |  | A | B | C | D | P | P' | N |
|  | Cluster:0-2 | |  |  | 2 | 77 | 2 | 4582 | 0.0016634972 | 0.0049904916 | 3 |
|  |  | RAFL11-11-P13 | At1g60950 / ferrodoxin, chloroplast | |  |  |  |  |  | | --- | --- | --- | --- | --- | |  |  |  |  |  | | RAFL11-11-P13 ,At1g60950  ferredoxin, chloroplast (PETF) identical to FERREDOXIN PRECURSOR GB:P16972 [SP|P16972] from [Arabidopsis thaliana] | | | | | | |
|  |  | RAFL05-18-N22 | At1g60950 / ferrodoxin, chloroplast | |  |  |  |  |  | | --- | --- | --- | --- | --- | |  |  |  |  |  | | RAFL05-18-N22 ,At1g60950  ferredoxin, chloroplast (PETF) identical to FERREDOXIN PRECURSOR GB:P16972 [SP|P16972] from [Arabidopsis thaliana] | | | | | | |
| 5\_24900001\_25200000 | | |  |  | A | B | C | D | P | P' | N |
|  | Cluster:8-0 | |  |  | 2 | 107 | 12 | 4542 | 0.041028306 | 0.49233967 | 12 |
|  |  | RAFL07-11-G07 | At5g63190 / MA3 domain-containing protein | |  |  |  |  |  | | --- | --- | --- | --- | --- | |  |  |  |  |  | | At5g63190 ,RAFL07-11-G07  MA3 domain-containing protein low similarity to programmed cell death 4 protein [Gallus gallus] GI:12958564; contains Pfam profile PF02847: MA3 domain | | | | | | |
|  |  | RAFL04-10-G08 | At5g63190 / MA3 domain-containing protein | |  |  |  |  |  | | --- | --- | --- | --- | --- | |  |  |  |  |  | | At5g63190 ,RAFL04-10-G08  MA3 domain-containing protein low similarity to programmed cell death 4 protein [Gallus gallus] GI:12958564; contains Pfam profile PF02847: MA3 domain | | | | | | |
| 1\_23760001\_23790000 | | |  |  | A | B | C | D | P | P' | N |
|  | Cluster:8-0 | |  |  | 1 | 108 | 0 | 4554 | 0.02337551 | 0.02337551 | 1 |
|  |  | RAFL09-16-D24 | At1g64950 / cytochrome P450, putative | |  |  |  |  |  | | --- | --- | --- | --- | --- | |  |  |  |  |  | | At1g64950 ,RAFL09-16-D24  cytochrome P450, putative similar to cytochrome P450 89A2 (CYPLXXXIX) (SP:Q42602) [Arabidopsis thaliana];similar to cytochrome P450 (GI:438242) [Solanum melongena] | | | | | | |
| 1\_9900001\_10200000 | | |  |  | A | B | C | D | P | P' | N |
|  | Cluster:7-2 | |  |  | 2 | 62 | 8 | 4591 | 0.0077741425 | 0.06219314 | 8 |
|  |  | RAFL07-09-K08 | At1g28380 / expressed protein | |  |  |  |  |  | | --- | --- | --- | --- | --- | |  |  |  |  |  | | At1g28380 ,RAFL07-09-K08  expressed protein | | | | | | |
|  |  | RAFL09-18-C18 | At1g28370 / ethylene responsive element binding factor 11, putative (EREBP11)(ERF11) | |  |  |  |  |  | | --- | --- | --- | --- | --- | |  |  |  |  |  | | RAFL09-18-C18 ,At1g28370  ERF domain protein 11 (ERF11) identical to ERF domain protein 11 (AtERF11) GI:15207789 from [Arabidopsis thaliana] | | | | | | |
| 3\_10020001\_10050000 | | |  |  | A | B | C | D | P | P' | N |
|  | Cluster:8-1 | |  |  | 1 | 161 | 0 | 4501 | 0.034741584 | 0.034741584 | 1 |
|  |  | RAFL08-09-G20 | At3g27210 / expressed protein | |  |  |  |  |  | | --- | --- | --- | --- | --- | |  |  |  |  |  | | RAFL08-09-G20 ,At3g27210  expressed protein | | | | | | |
| 2\_12300001\_12330000 | | |  |  | A | B | C | D | P | P' | N |
|  | Cluster:8-0 | |  |  | 1 | 108 | 1 | 4553 | 0.0462095 | 0.092419 | 2 |
|  |  | RAFL04-17-D16 | At2g28840 / RING zinc finger ankyrin protein -related | |  |  |  |  |  | | --- | --- | --- | --- | --- | |  |  |  |  |  | | At2g28840 ,RAFL04-17-D16  ankyrin repeat family protein contains ankyrin repeats, Pfam:PF00023 | | | | | | |
| 4\_1\_3000000 | | |  |  | A | B | C | D | P | P' | N |
|  | Cluster:8-1 | |  |  | 9 | 153 | 72 | 4429 | 0.0018003528 | 0.04320847 | 24 |
|  |  | RAFL04-16-G03 | At4g02890 / polyubiquitin (UBQ14) | |  |  |  |  |  | | --- | --- | --- | --- | --- | |  |  |  |  |  | | RAFL04-16-G03 ,At4g02890  polyubiquitin (UBQ14) identical to GI:166795; similar to N. sylvestris hexameric polyubiquitin, GenBank accession number M74101 | | | | | | |
|  |  | RAFL05-18-E11 | At4g03430 / pre-mRNA splicing factor -related | |  |  |  |  |  | | --- | --- | --- | --- | --- | |  |  |  |  |  | | RAFL05-18-E11 ,At4g03430  pre-mRNA splicing factor-related similar to pre-mRNA splicing factor pre-mRNA splicing factor prp1 (SP:Q12381) [Fission yeast] | | | | | | |
|  |  | RAFL09-09-O15 | At4g05320 / polyubiquitin UBQ10/SEN3 | |  |  |  |  |  | | --- | --- | --- | --- | --- | |  |  |  |  |  | | At4g05320 ,RAFL09-09-O15  polyubiquitin (UBQ10) (SEN3) senescence-associated protein; identical to GI:870791 | | | | | | |
|  |  | RAFL09-06-I17 | At4g05320 / polyubiquitin UBQ10/SEN3 | |  |  |  |  |  | | --- | --- | --- | --- | --- | |  |  |  |  |  | | At4g05320 ,RAFL09-06-I17  polyubiquitin (UBQ10) (SEN3) senescence-associated protein; identical to GI:870791 | | | | | | |
|  |  | RAFL04-17-I16 | At4g02380 / late embryogenesis abundant protein family | |  |  |  |  |  | | --- | --- | --- | --- | --- | |  |  |  |  |  | | RAFL04-17-I16 ,At4g02380  late embryogenesis abundant 3 family protein / LEA3 family protein similar to several small proteins (~100 aa) that are induced by heat, auxin, ethylene and wounding such as Phaseolus aureus indole-3-acetic acid induced protein ARG (SW:32292); contains Pfam profile PF03242: Late embryogenesis abundant protein | | | | | | |
|  |  | RAFL05-14-O22 | At4g05320 / polyubiquitin UBQ10/SEN3 | |  |  |  |  |  | | --- | --- | --- | --- | --- | |  |  |  |  |  | | At4g05320 ,RAFL05-14-O22  polyubiquitin (UBQ10) (SEN3) senescence-associated protein; identical to GI:870791 | | | | | | |
|  |  | RAFL07-10-D10 | At4g05320 / polyubiquitin UBQ10/SEN3 | |  |  |  |  |  | | --- | --- | --- | --- | --- | |  |  |  |  |  | | At4g05320 ,RAFL07-10-D10  polyubiquitin (UBQ10) (SEN3) senescence-associated protein; identical to GI:870791 | | | | | | |
|  |  | RAFL05-16-P22 | At4g05150 / octicosapeptide/Phox/Bem1p (PB1) domain-containing protein | |  |  |  |  |  | | --- | --- | --- | --- | --- | |  |  |  |  |  | | At4g05150 ,RAFL05-16-P22  octicosapeptide/Phox/Bem1p (PB1) domain-containing protein various predicted proteins contains Pfam profile PF00564: PB1 domain | | | | | | |
|  |  | RAFL08-16-I23 | At4g01120 / G-box binding factor(bZIP protein), putative | |  |  |  |  |  | | --- | --- | --- | --- | --- | |  |  |  |  |  | | RAFL08-16-I23 ,At4g01120  G-box binding factor 2 (GBF2) identical to G-box binding factor 2 (GBF2) SP:P42775 from [Arabidopsis thaliana];contains Pfam profile: PF00170 bZIP transcription factor | | | | | | |
|  | Cluster:10-1 | |  |  | 4 | 39 | 77 | 4543 | 0.006235307 | 0.14964737 | 24 |
|  |  | RAFL05-12-H13 | At4g01020 / helicase domain-containing protein | |  |  |  |  |  | | --- | --- | --- | --- | --- | |  |  |  |  |  | | At4g01020 ,RAFL05-12-H13  helicase domain-containing protein / IBR domain-containing protein / zinc finger protein-related similar to SP|Q14562 ATP-dependent helicase DDX8 (RNA helicase HRH1) (DEAH-box protein 8) {Homo sapiens}; contains Pfam profiles PF04408: Helicase associated domain (HA2), PF00271: Helicase conserved C-terminal domain, PF00097: Zinc finger, C3HC4 type (RING finger), PF01485: IBR domain | | | | | | |
|  |  | RAFL05-14-D24 | At4g05100 / myb DNA-binding protein | |  |  |  |  |  | | --- | --- | --- | --- | --- | |  |  |  |  |  | | At4g05100 ,RAFL05-14-D24  myb family transcription factor (MYB74) contains Pfam profile: PF00249 myb-like DNA-binding domain; identical to cDNA putative transcription factor (MYB74) mRNA, partial cds GI:3941505 | | | | | | |
|  |  | RAFL03-07-M07 | At4g02380 / late embryogenesis abundant protein family | |  |  |  |  |  | | --- | --- | --- | --- | --- | |  |  |  |  |  | | RAFL03-07-M07 ,At4g02380  late embryogenesis abundant 3 family protein / LEA3 family protein similar to several small proteins (~100 aa) that are induced by heat, auxin, ethylene and wounding such as Phaseolus aureus indole-3-acetic acid induced protein ARG (SW:32292); contains Pfam profile PF03242: Late embryogenesis abundant protein | | | | | | |
|  |  | RAFL06-13-N20 | At4g02380 / late embryogenesis abundant protein family | |  |  |  |  |  | | --- | --- | --- | --- | --- | |  |  |  |  |  | | RAFL06-13-N20 ,At4g02380  late embryogenesis abundant 3 family protein / LEA3 family protein similar to several small proteins (~100 aa) that are induced by heat, auxin, ethylene and wounding such as Phaseolus aureus indole-3-acetic acid induced protein ARG (SW:32292); contains Pfam profile PF03242: Late embryogenesis abundant protein | | | | | | |
|  | Cluster:1-0 | |  |  | 7 | 142 | 74 | 4440 | 0.014235923 | 0.34166214 | 24 |
|  |  | RAFL05-01-O08 | At4g01150 / expressed protein | |  |  |  |  |  | | --- | --- | --- | --- | --- | |  |  |  |  |  | | RAFL05-01-O08 ,At4g01150  expressed protein | | | | | | |
|  |  | RAFL05-02-L04 | At4g02530 / chloroplast thylakoid lumen protein | |  |  |  |  |  | | --- | --- | --- | --- | --- | |  |  |  |  |  | | RAFL05-02-L04 ,At4g02530  chloroplast thylakoid lumen protein SP:022773 ;TL16\_ARATH | | | | | | |
|  |  | RAFL05-08-K07 | At4g01310 / ribosomal protein L5p family | |  |  |  |  |  | | --- | --- | --- | --- | --- | |  |  |  |  |  | | RAFL05-08-K07 ,At4g01310  ribosomal protein L5 family protein contains Pfam profiles PF00673: ribosomal L5P family C-terminus, PF00281: ribosomal protein L5 | | | | | | |
|  |  | RAFL07-17-I11 | At4g02290 / glycosyl hydrolase family 9 | |  |  |  |  |  | | --- | --- | --- | --- | --- | |  |  |  |  |  | | RAFL07-17-I11 ,At4g02290  glycosyl hydrolase family 9 protein similar to endo-1,4-beta glucanase; ATCEL2 GI:3132891 from [Arabidopsis thaliana] | | | | | | |
|  |  | RAFL06-16-A22 | At4g05180 / oxygen-evolving complex protein 16, chloroplast precursor (OEC16) | |  |  |  |  |  | | --- | --- | --- | --- | --- | |  |  |  |  |  | | RAFL06-16-A22 ,At4g05180  oxygen-evolving enhancer protein 3, chloroplast, putative (PSBQ2) identical to SP|Q41932 Oxygen-evolving enhancer protein 3-2, chloroplast precursor (OEE3) (16 kDa subunit of oxygen evolving system of photosystem II) (OEC 16 kDa subunit) {Arabidopsis thaliana}; similar to SP|P12301 Oxygen-evolving enhancer protein 3, chloroplast precursor (OEE3) (16 kDa subunit of oxygen evolving system of photosystem II) (OEC 16 kDa subunit) {Spinacia oleracea}; contains Pfam profile PF05757: Oxygen evolving enhancer protein 3 (PsbQ) | | | | | | |
|  |  | RAFL07-07-D15 | At4g04940 / transducin / WD-40 repeat protein family | |  |  |  |  |  | | --- | --- | --- | --- | --- | |  |  |  |  |  | | At4g04940 ,RAFL07-07-D15  transducin family protein / WD-40 repeat family protein contains seven G-protein beta WD-40 repeats | | | | | | |
|  |  | RAFL09-14-J06 | At4g04460 / aspartic protease -related | |  |  |  |  |  | | --- | --- | --- | --- | --- | |  |  |  |  |  | | RAFL09-14-J06 ,At4g04460  aspartyl protease family protein contains Pfam profiles: PF00026 eukaryotic aspartyl protease, PF03489 surfactant protein B, PF05184 saposin-like type B, region 1 | | | | | | |
|  | Cluster:8-2 | |  |  | 4 | 58 | 77 | 4524 | 0.022002848 | 0.52806836 | 24 |
|  |  | RAFL04-14-B14 | At4g05050 / polyubiquitin UBQ11 | |  |  |  |  |  | | --- | --- | --- | --- | --- | |  |  |  |  |  | | At4g05050 ,RAFL04-14-B14  polyubiquitin (UBQ11) identical to GI:304117 | | | | | | |
|  |  | RAFL06-07-F24 | At4g05050 / polyubiquitin UBQ11 | |  |  |  |  |  | | --- | --- | --- | --- | --- | |  |  |  |  |  | | RAFL06-07-F24 ,At4g05050  polyubiquitin (UBQ11) identical to GI:304117 | | | | | | |
|  |  | RAFL06-09-F19 | At4g00430 / transmembrane protein (MIP family) | |  |  |  |  |  | | --- | --- | --- | --- | --- | |  |  |  |  |  | | At4g00430 ,RAFL06-09-F19  plasma membrane intrinsic protein, putative identical to transmembrane protein GI:535780 from [Arabidopsis thaliana]; very strong similarity to SP|Q08733 Plasma membrane intrinsic protein 1C (Transmembrane protein B) (TMP-B) {Arabidopsis thaliana}; contains Pfam profile PF00230: Major intrinsic protein; | | | | | | |
|  |  | RAFL05-16-H03 | At4g00360 / cytochrome p450, putative | |  |  |  |  |  | | --- | --- | --- | --- | --- | |  |  |  |  |  | | At4g00360 ,RAFL05-16-H03  cytochrome P450, putative | | | | | | |
| 3\_210001\_240000 | | |  |  | A | B | C | D | P | P' | N |
|  | Cluster:7-0 | |  |  | 3 | 244 | 2 | 4414 | 0.0013562166 | 0.00406865 | 3 |
|  |  | RAFL08-18-G23 | At3g01540 / DEAD box RNA helicase (DRH1) | |  |  |  |  |  | | --- | --- | --- | --- | --- | |  |  |  |  |  | | At3g01540 ,RAFL08-18-G23  DEAD box RNA helicase (DRH1) identical to RNA helicase DRH1 GB:BAA28347 GI:3149952 [Arabidopsis thaliana]; contains Pfam profiles PF00270: DEAD/DEAH box helicase, PF00271: Helicase conserved C-terminal domain | | | | | | |
|  |  | RAFL07-16-I19 | At3g01540 / DEAD box RNA helicase (DRH1) | |  |  |  |  |  | | --- | --- | --- | --- | --- | |  |  |  |  |  | | At3g01540 ,RAFL07-16-I19  DEAD box RNA helicase (DRH1) identical to RNA helicase DRH1 GB:BAA28347 GI:3149952 [Arabidopsis thaliana]; contains Pfam profiles PF00270: DEAD/DEAH box helicase, PF00271: Helicase conserved C-terminal domain | | | | | | |
|  |  | RAFL05-12-D03 | At3g01540 / DEAD box RNA helicase (DRH1) | |  |  |  |  |  | | --- | --- | --- | --- | --- | |  |  |  |  |  | | RAFL05-12-D03 ,At3g01540  DEAD box RNA helicase (DRH1) identical to RNA helicase DRH1 GB:BAA28347 GI:3149952 [Arabidopsis thaliana]; contains Pfam profiles PF00270: DEAD/DEAH box helicase, PF00271: Helicase conserved C-terminal domain | | | | | | |
| 5\_210001\_240000 | | |  |  | A | B | C | D | P | P' | N |
|  | Cluster:9-1 | |  |  | 1 | 95 | 1 | 4566 | 0.040755685 | 0.08151137 | 2 |
|  |  | RAFL06-11-F15 | At5g01600 / ferritin 1 precursor | |  |  |  |  |  | | --- | --- | --- | --- | --- | |  |  |  |  |  | | RAFL06-11-F15 ,At5g01600  ferritin 1 (FER1) identical to ferritin [Arabidopsis thaliana] GI:1246401, GI:8163920 | | | | | | |
| 3\_18240001\_18270000 | | |  |  | A | B | C | D | P | P' | N |
|  | Cluster:8-2 | |  |  | 1 | 61 | 2 | 4599 | 0.0393688 | 0.1181064 | 3 |
|  |  | RAFL07-12-F11 | At3g49220 / pectinesterase family | |  |  |  |  |  | | --- | --- | --- | --- | --- | |  |  |  |  |  | | At3g49220 ,RAFL07-12-F11  pectinesterase family protein contains Pfam profile: PF01095 pectinesterase | | | | | | |
| 4\_12930001\_12960000 | | |  |  | A | B | C | D | P | P' | N |
|  | Cluster:10-1 | |  |  | 1 | 42 | 1 | 4619 | 0.018359985 | 0.03671997 | 2 |
|  |  | RAFL05-16-H23 | At4g28140 / AP2 domain transcription factor, putative | |  |  |  |  |  | | --- | --- | --- | --- | --- | |  |  |  |  |  | | RAFL05-16-H23 ,At4g28140  AP2 domain-containing transcription factor, putative similar to AP2 domain containing protein RAP2.4 - Arabidopsis thaliana, PID:g2281633 | | | | | | |
| 1\_7560001\_7590000 | | |  |  | A | B | C | D | P | P' | N |
|  | Cluster:3-0 | |  |  | 1 | 232 | 0 | 4430 | 0.049967833 | 0.049967833 | 1 |
|  |  | RAFL05-10-H23 | At1g21640 / expressed protein | |  |  |  |  |  | | --- | --- | --- | --- | --- | |  |  |  |  |  | | At1g21640 ,RAFL05-10-H23  ATP-NAD kinase family protein contains similarity to NAD kinase [Homo sapiens] gi|20070086|gb|AAM01195; contains Pfam domain, PF01513: ATP-NAD kinase | | | | | | |
| 5\_6060001\_6090000 | | |  |  | A | B | C | D | P | P' | N |
|  | Cluster:4-2 | |  |  | 1 | 134 | 0 | 4528 | 0.028951319 | 0.028951319 | 1 |
|  |  | RAFL05-08-B08 | At5g18380 / 40S ribosomal protein S16 (RPS16C) | |  |  |  |  |  | | --- | --- | --- | --- | --- | |  |  |  |  |  | | At5g18380 ,RAFL05-08-B08  40S ribosomal protein S16 (RPS16C) | | | | | | |
| 4\_14550001\_14580000 | | |  |  | A | B | C | D | P | P' | N |
|  | Cluster:1-1 | |  |  | 1 | 104 | 0 | 4558 | 0.022517692 | 0.022517692 | 1 |
|  |  | RAFL04-12-O23 | At4g32330 / expressed protein | |  |  |  |  |  | | --- | --- | --- | --- | --- | |  |  |  |  |  | | RAFL04-12-O23 ,At4g32330  expressed protein | | | | | | |
| 5\_19560001\_19590000 | | |  |  | A | B | C | D | P | P' | N |
|  | Cluster:1-2 | |  |  | 1 | 173 | 0 | 4489 | 0.037315033 | 0.037315033 | 1 |
|  |  | RAFL07-08-E09 | At5g49030 / isoleucyl-tRNA synthetase | |  |  |  |  |  | | --- | --- | --- | --- | --- | |  |  |  |  |  | | RAFL07-08-E09 ,At5g49030  tRNA synthetase class I (I, L, M and V) family protein similar to SP|P41972 Isoleucyl-tRNA synthetase (EC 6.1.1.5) (Isoleucine--tRNA ligase) (IleRS) {Staphylococcus aureus}; contains Pfam profile PF00133: tRNA synthetases class I (I, L, M and V) | | | | | | |
| 4\_10590001\_10620000 | | |  |  | A | B | C | D | P | P' | N |
|  | Cluster:1-0 | |  |  | 1 | 148 | 0 | 4514 | 0.031953678 | 0.031953678 | 1 |
|  |  | RAFL04-16-O12 | At4g21960 / peroxidase, putative | |  |  |  |  |  | | --- | --- | --- | --- | --- | |  |  |  |  |  | | RAFL04-16-O12 ,At4g21960  peroxidase 42 (PER42) (P42) (PRXR1) identical to SP|Q9SB81 Peroxidase 42 precursor (EC 1.11.1.7) (Atperox P42) (PRXR1) (ATP1a/ATP1b) {Arabidopsis thaliana} | | | | | | |
| 1\_29670001\_29700000 | | |  |  | A | B | C | D | P | P' | N |
|  | Cluster:1-0 | |  |  | 2 | 147 | 1 | 4513 | 0.0029792225 | 0.005958445 | 2 |
|  |  | RAFL06-16-K01 | At1g79850 / chloroplast 30S ribosomal protein S17 (CS17) | |  |  |  |  |  | | --- | --- | --- | --- | --- | |  |  |  |  |  | | RAFL06-16-K01 ,At1g79850  30S ribosomal protein S17, chloroplast / CS17 (RPS17) identical to 30S ribosomal protein S17, chloroplast precursor GB:P16180 [Arabidopsis thaliana] | | | | | | |
|  |  | RAFL04-19-L23 | At1g79850 / chloroplast 30S ribosomal protein S17 (CS17) | |  |  |  |  |  | | --- | --- | --- | --- | --- | |  |  |  |  |  | | RAFL04-19-L23 ,At1g79850  30S ribosomal protein S17, chloroplast / CS17 (RPS17) identical to 30S ribosomal protein S17, chloroplast precursor GB:P16180 [Arabidopsis thaliana] | | | | | | |
| 1\_29280001\_29310000 | | |  |  | A | B | C | D | P | P' | N |
|  | Cluster:6-0 | |  |  | 2 | 138 | 4 | 4519 | 0.012401914 | 0.062009573 | 5 |
|  |  | RAFL09-18-I11 | At1g78820 / curculin-like (mannose-binding) lectin family | |  |  |  |  |  | | --- | --- | --- | --- | --- | |  |  |  |  |  | | At1g78820 ,RAFL09-18-I11  curculin-like (mannose-binding) lectin family protein / PAN domain-containing protein similar to S locus glycoprotein [Brassica rapa] GI:12246840; contains Pfam profile PF01453: Lectin (probable mannose binding) | | | | | | |
|  |  | RAFL09-06-A15 | At1g78820 / curculin-like (mannose-binding) lectin family | |  |  |  |  |  | | --- | --- | --- | --- | --- | |  |  |  |  |  | | RAFL09-06-A15 ,At1g78820  curculin-like (mannose-binding) lectin family protein / PAN domain-containing protein similar to S locus glycoprotein [Brassica rapa] GI:12246840; contains Pfam profile PF01453: Lectin (probable mannose binding) | | | | | | |
| 1\_19680001\_19710000 | | |  |  | A | B | C | D | P | P' | N |
|  | Cluster:2-2 | |  |  | 1 | 52 | 0 | 4610 | 0.011366073 | 0.011366073 | 1 |
|  |  | RAFL06-10-O06 | At1g53840 / pectinesterase family | |  |  |  |  |  | | --- | --- | --- | --- | --- | |  |  |  |  |  | | At1g53840 ,RAFL06-10-O06  pectinesterase family protein contains Pfam profile: PF01095 pectinesterase | | | | | | |
| 2\_9990001\_10020000 | | |  |  | A | B | C | D | P | P' | N |
|  | Cluster:1-0 | |  |  | 1 | 148 | 0 | 4514 | 0.031953678 | 0.031953678 | 1 |
|  |  | RAFL09-12-L06 | At2g23600 / hydrolase, alpha/beta fold family | |  |  |  |  |  | | --- | --- | --- | --- | --- | |  |  |  |  |  | | At2g23600 ,RAFL09-12-L06  hydrolase, alpha/beta fold family protein similar to ethylene-induced esterase [Citrus sinensis] GI:14279437, polyneuridine aldehyde esterase [Rauvolfia serpentina] GI:6651393; contains Pfam profile PF00561: hydrolase, alpha/beta fold family | | | | | | |
| Chromosome:1 | | |  |  | A | B | C | D | P | P' | N |
|  | Cluster:0-2 | |  |  | 32 | 47 | 1087 | 3497 | 0.0012182354 | 0.040201765 | 33 |
|  |  | RAFL05-16-F08 | At1g31330 / photosystem I subunit III precursor -related | |  |  |  |  |  | | --- | --- | --- | --- | --- | |  |  |  |  |  | | RAFL05-16-F08 ,At1g31330  photosystem I reaction center subunit III family protein contains Pfam profile: PF02507: photosystem I reaction center subunit III | | | | | | |
|  |  | RAFL08-12-J08 | At1g14345 / expressed protein | |  |  |  |  |  | | --- | --- | --- | --- | --- | |  |  |  |  |  | | At1g14345 ,RAFL08-12-J08  expressed protein contains one transmembrane domain | | | | | | |
|  |  | RAFL04-17-G02 | At1g56340 / calreticulin 1 (CRT1) | |  |  |  |  |  | | --- | --- | --- | --- | --- | |  |  |  |  |  | | At1g56340 ,RAFL04-17-G02  calreticulin 1 (CRT1) identical to calreticulin (crt1) GI:2052379 [Arabidopsis thaliana] | | | | | | |
|  |  | RAFL06-12-L07 | At1g08380 / expressed protein | |  |  |  |  |  | | --- | --- | --- | --- | --- | |  |  |  |  |  | | At1g08380 ,RAFL06-12-L07  expressed protein | | | | | | |
|  |  | RAFL06-09-H06 | At1g29910 / photosystem II type I chlorophyll a /b binding protein, putative | |  |  |  |  |  | | --- | --- | --- | --- | --- | |  |  |  |  |  | | RAFL06-09-H06 ,At1g29910  chlorophyll A-B binding protein 2, chloroplast / LHCII type I CAB-2 / CAB-140 (CAB2A) identical to SP|P04778 Chlorophyll A-B binding protein 2, chloroplast precursor (LHCII type I CAB-2) (CAB-140) (LHCP) {Arabidopsis thaliana} | | | | | | |
|  |  | RAFL07-10-D01 | At1g74470 / geranylgeranyl reductase | |  |  |  |  |  | | --- | --- | --- | --- | --- | |  |  |  |  |  | | At1g74470 ,RAFL07-10-D01  geranylgeranyl reductase identical to geranylgeranyl reductase GB:Y14044 [Arabidopsis thaliana] (involvement: chlorophyll, the tocopherol and the phylloquinone pathways Eur J Biochem 1998 Jan 15;251(1-2):413-7) | | | | | | |
|  |  | RAFL11-11-P13 | At1g60950 / ferrodoxin, chloroplast | |  |  |  |  |  | | --- | --- | --- | --- | --- | |  |  |  |  |  | | RAFL11-11-P13 ,At1g60950  ferredoxin, chloroplast (PETF) identical to FERREDOXIN PRECURSOR GB:P16972 [SP|P16972] from [Arabidopsis thaliana] | | | | | | |
|  |  | RAFL05-18-N22 | At1g60950 / ferrodoxin, chloroplast | |  |  |  |  |  | | --- | --- | --- | --- | --- | |  |  |  |  |  | | RAFL05-18-N22 ,At1g60950  ferredoxin, chloroplast (PETF) identical to FERREDOXIN PRECURSOR GB:P16972 [SP|P16972] from [Arabidopsis thaliana] | | | | | | |
|  |  | RAFL04-19-A20 | At1g12110 / putative NPK1-related protein kinase 2 | |  |  |  |  |  | | --- | --- | --- | --- | --- | |  |  |  |  |  | | At1g12110 ,RAFL04-19-A20  nitrate/chlorate transporter (NRT1.1) (CHL1) identical to nitrate/chlorate transporter SP:Q05085 from [Arabidopsis thaliana]; contains Pfam profile: PF00854 POT family | | | | | | |
|  |  | RAFL06-12-C05 | At1g08380 / expressed protein | |  |  |  |  |  | | --- | --- | --- | --- | --- | |  |  |  |  |  | | RAFL06-12-C05 ,At1g08380  expressed protein | | | | | | |
|  |  | RAFL04-10-O12 | At1g12250 / chloroplast lumen pentapeptide protein, putative | |  |  |  |  |  | | --- | --- | --- | --- | --- | |  |  |  |  |  | | RAFL04-10-O12 ,At1g12250  thylakoid lumenal protein-related weak similarity to SP|O22160 Thylakoid lumenal 15 kDa protein, chloroplast precursor (p15) {Arabidopsis thaliana}; contains Pfam profile PF00805: Pentapeptide repeats (8 copies) | | | | | | |
|  |  | RAFL09-06-G16 | At1g31330 / photosystem I subunit III precursor -related | |  |  |  |  |  | | --- | --- | --- | --- | --- | |  |  |  |  |  | | At1g31330 ,RAFL09-06-G16  photosystem I reaction center subunit III family protein contains Pfam profile: PF02507: photosystem I reaction center subunit III | | | | | | |
|  |  | RAFL08-15-B05 | At1g08380 / expressed protein | |  |  |  |  |  | | --- | --- | --- | --- | --- | |  |  |  |  |  | | RAFL08-15-B05 ,At1g08380  expressed protein | | | | | | |
|  |  | RAFL06-15-G08 | At1g52220 / expressed protein | |  |  |  |  |  | | --- | --- | --- | --- | --- | |  |  |  |  |  | | At1g52220 ,RAFL06-15-G08  expressed protein | | | | | | |
|  |  | RAFL04-19-G19 | At1g74730 / expressed protein | |  |  |  |  |  | | --- | --- | --- | --- | --- | |  |  |  |  |  | | At1g74730 ,RAFL04-19-G19  expressed protein | | | | | | |
|  |  | RAFL06-16-F14 | At1g29920 / photosystem II type I chlorophyll a /b binding protein, putative | |  |  |  |  |  | | --- | --- | --- | --- | --- | |  |  |  |  |  | | At1g29920 ,RAFL06-16-F14  chlorophyll A-B binding protein 165/180, chloroplast / LHCII type I CAB-165/180 identical to SP|P04777 Chlorophyll A-B binding protein 165/180, chloroplast precursor (LHCII type I CAB-165/180) (LHCP) {Arabidopsis thaliana}; similar to photosystem II type I chlorophyll a /b binding protein GI:16364 from [Arabidopsis thaliana] | | | | | | |
|  |  | RAFL05-17-G01 | At1g51400 / photosystem II 5 KD protein | |  |  |  |  |  | | --- | --- | --- | --- | --- | |  |  |  |  |  | | At1g51400 ,RAFL05-17-G01  photosystem II 5 kD protein 100% identical to GI:4836947 (F5D21.10) | | | | | | |
|  |  | RAFL09-10-I02 | At1g29930 / light-harvesting chlorophyll a/b binding protein | |  |  |  |  |  | | --- | --- | --- | --- | --- | |  |  |  |  |  | | RAFL09-10-I02 ,At1g29930  chlorophyll A-B binding protein 2, chloroplast / LHCII type I CAB-2 / CAB-140 (CAB2B) identical to SP|P04778 Chlorophyll A-B binding protein 2, chloroplast precursor (LHCII type I CAB-2) (CAB-140) (LHCP) {Arabidopsis thaliana} | | | | | | |
|  |  | RAFL07-15-H08 | At1g74470 / geranylgeranyl reductase | |  |  |  |  |  | | --- | --- | --- | --- | --- | |  |  |  |  |  | | RAFL07-15-H08 ,At1g74470  geranylgeranyl reductase identical to geranylgeranyl reductase GB:Y14044 [Arabidopsis thaliana] (involvement: chlorophyll, the tocopherol and the phylloquinone pathways Eur J Biochem 1998 Jan 15;251(1-2):413-7) | | | | | | |
|  |  | RAFL06-08-A02 | At1g29920 / photosystem II type I chlorophyll a /b binding protein, putative | |  |  |  |  |  | | --- | --- | --- | --- | --- | |  |  |  |  |  | | At1g29920 ,RAFL06-08-A02  chlorophyll A-B binding protein 165/180, chloroplast / LHCII type I CAB-165/180 identical to SP|P04777 Chlorophyll A-B binding protein 165/180, chloroplast precursor (LHCII type I CAB-165/180) (LHCP) {Arabidopsis thaliana}; similar to photosystem II type I chlorophyll a /b binding protein GI:16364 from [Arabidopsis thaliana] | | | | | | |
|  |  | RAFL04-09-M24 | At1g54500 / rubredoxin -related | |  |  |  |  |  | | --- | --- | --- | --- | --- | |  |  |  |  |  | | At1g54500 ,RAFL04-09-M24  rubredoxin family protein similar to SP|P00270 Rubredoxin (Rd) {Desulfovibrio gigas}; contains Pfam profile PF00301: Rubredoxin | | | | | | |
|  |  | RAFL05-19-G04 | At1g54780 / thylakoid lumen 18.3 kDa protein | |  |  |  |  |  | | --- | --- | --- | --- | --- | |  |  |  |  |  | | RAFL05-19-G04 ,At1g54780  thylakoid lumen 18.3 kDa protein SP:Q9ZVL6 | | | | | | |
|  |  | RAFL02-02-L10 | At1g03130 / photosystem I reaction center subunit II precursor -related | |  |  |  |  |  | | --- | --- | --- | --- | --- | |  |  |  |  |  | | RAFL02-02-L10 ,At1g03130  photosystem I reaction center subunit II, chloroplast, putative / photosystem I 20 kDa subunit, putative / PSI-D, putative (PSAD2) similar to SP|P12353 Photosystem I reaction center subunit II, chloroplast precursor (Photosystem I 20 kDa subunit) (PSI-D) {Spinacia oleracea}; contains Pfam profile PF02531: PsaD | | | | | | |
|  |  | RAFL06-15-B12 | At1g29930 / light-harvesting chlorophyll a/b binding protein | |  |  |  |  |  | | --- | --- | --- | --- | --- | |  |  |  |  |  | | At1g29930 ,RAFL06-15-B12  chlorophyll A-B binding protein 2, chloroplast / LHCII type I CAB-2 / CAB-140 (CAB2B) identical to SP|P04778 Chlorophyll A-B binding protein 2, chloroplast precursor (LHCII type I CAB-2) (CAB-140) (LHCP) {Arabidopsis thaliana} | | | | | | |
|  |  | RAFL05-04-A19 | At1g51400 / photosystem II 5 KD protein | |  |  |  |  |  | | --- | --- | --- | --- | --- | |  |  |  |  |  | | At1g51400 ,RAFL05-04-A19  photosystem II 5 kD protein 100% identical to GI:4836947 (F5D21.10) | | | | | | |
|  |  | RAFL06-12-P20 | At1g28400 / expressed protein | |  |  |  |  |  | | --- | --- | --- | --- | --- | |  |  |  |  |  | | RAFL06-12-P20 ,At1g28400  expressed protein similar to E6 (GI:1000090) [Gossypium barbadense] | | | | | | |
|  |  | RAFL05-01-I05 | At1g54780 / thylakoid lumen 18.3 kDa protein | |  |  |  |  |  | | --- | --- | --- | --- | --- | |  |  |  |  |  | | RAFL05-01-I05 ,At1g54780  thylakoid lumen 18.3 kDa protein SP:Q9ZVL6 | | | | | | |
|  |  | RAFL08-09-H12 | At1g75460 / protease -related | |  |  |  |  |  | | --- | --- | --- | --- | --- | |  |  |  |  |  | | At1g75460 ,RAFL08-09-H12  ATP-dependent protease La (LON) domain-containing protein weak similarity to SP|P36774 ATP-dependent protease La 2 (EC 3.4.21.53) {Myxococcus xanthus}; contains Pfam profile PF02190: ATP-dependent protease La (LON) domain | | | | | | |
|  |  | RAFL09-16-K15 | At1g31330 / photosystem I subunit III precursor -related | |  |  |  |  |  | | --- | --- | --- | --- | --- | |  |  |  |  |  | | At1g31330 ,RAFL09-16-K15  photosystem I reaction center subunit III family protein contains Pfam profile: PF02507: photosystem I reaction center subunit III | | | | | | |
|  |  | RAFL05-09-F03 | At1g31330 / photosystem I subunit III precursor -related | |  |  |  |  |  | | --- | --- | --- | --- | --- | |  |  |  |  |  | | At1g31330 ,RAFL05-09-F03  photosystem I reaction center subunit III family protein contains Pfam profile: PF02507: photosystem I reaction center subunit III | | | | | | |
|  |  | RAFL07-14-F21 | At1g54780 / thylakoid lumen 18.3 kDa protein | |  |  |  |  |  | | --- | --- | --- | --- | --- | |  |  |  |  |  | | RAFL07-14-F21 ,At1g54780  thylakoid lumen 18.3 kDa protein SP:Q9ZVL6 | | | | | | |
|  |  | RAFL11-03-K23 | At1g54500 / rubredoxin -related | |  |  |  |  |  | | --- | --- | --- | --- | --- | |  |  |  |  |  | | At1g54500 ,RAFL11-03-K23  rubredoxin family protein similar to SP|P00270 Rubredoxin (Rd) {Desulfovibrio gigas}; contains Pfam profile PF00301: Rubredoxin | | | | | | |
|  | Cluster:7-1 | |  |  | 84 | 181 | 1035 | 3363 | 0.0037613933 | 0.12412597 | 33 |
|  |  | RAFL11-06-E04 | At1g76920 / F-box protein family (FBX3) | |  |  |  |  |  | | --- | --- | --- | --- | --- | |  |  |  |  |  | | At1g76920 ,RAFL11-06-E04  F-box family protein (FBX3) contains similarity to stamina pistilloidia GI:4101570, the pea ortholog of Fim and UFO from [Pisum sativum] | | | | | | |
|  |  | RAFL08-12-P05 | At1g22710 / sucrose transporter SUC2 (sucrose-proton symporter) | |  |  |  |  |  | | --- | --- | --- | --- | --- | |  |  |  |  |  | | RAFL08-12-P05 ,At1g22710  sucrose transporter / sucrose-proton symporter (SUC2) nearly identical to sucrose-proton symporter SUC2 [Arabidopsis thaliana] GI:407092 | | | | | | |
|  |  | RAFL05-15-K12 | At1g54270 / eukaryotic translation initiation factor 4A-2 (eIF4A-2) | |  |  |  |  |  | | --- | --- | --- | --- | --- | |  |  |  |  |  | | RAFL05-15-K12 ,At1g54270  eukaryotic translation initiation factor 4A-2 / eIF-4A-2 similar to eukaryotic translation initiation factor 4A GI:19696 from [Nicotiana plumbaginifolia] | | | | | | |
|  |  | RAFL02-07-N19 | At1g10760 / SEX1 protein; nuclear gene for chloroplast product | |  |  |  |  |  | | --- | --- | --- | --- | --- | |  |  |  |  |  | | At1g10760 ,RAFL02-07-N19  starch excess protein (SEX1) identical to SEX1 [Arabidopsis thaliana] GI:12044358; supporting cDNA gi|12044357|gb|AF312027.1|AF312027 | | | | | | |
|  |  | RAFL08-10-B15 | At1g22710 / sucrose transporter SUC2 (sucrose-proton symporter) | |  |  |  |  |  | | --- | --- | --- | --- | --- | |  |  |  |  |  | | RAFL08-10-B15 ,At1g22710  sucrose transporter / sucrose-proton symporter (SUC2) nearly identical to sucrose-proton symporter SUC2 [Arabidopsis thaliana] GI:407092 | | | | | | |
|  |  | RAFL08-12-E01 | At1g01550 / expressed protein | |  |  |  |  |  | | --- | --- | --- | --- | --- | |  |  |  |  |  | | At1g01550 ,RAFL08-12-E01  expressed protein | | | | | | |
|  |  | RAFL06-08-E17 | At1g22840 / cytochrome C -related | |  |  |  |  |  | | --- | --- | --- | --- | --- | |  |  |  |  |  | | RAFL06-08-E17 ,At1g22840  cytochrome c, putative similar to cytochrome c [Pumpkin, Winter squash] SWISS-PROT:P00051 | | | | | | |
|  |  | RAFL09-12-N02 | At1g07630 / expressed protein | |  |  |  |  |  | | --- | --- | --- | --- | --- | |  |  |  |  |  | | RAFL09-12-N02 ,At1g07630  protein phosphatase 2C family protein / PP2C family protein similar to protein phosphatase-2c (GI:3608412) [Mesembryanthemum crystallinum]; contains Pfam PF00481 : Protein phosphatase 2C domain | | | | | | |
|  |  | RAFL11-05-C09 | At1g78100 / F-box protein family | |  |  |  |  |  | | --- | --- | --- | --- | --- | |  |  |  |  |  | | RAFL11-05-C09 ,At1g78100  F-box family protein contains F-box domain Pfam:PF00646 | | | | | | |
|  |  | RAFL09-16-J21 | At1g72330 / alanine aminotransferase, putative | |  |  |  |  |  | | --- | --- | --- | --- | --- | |  |  |  |  |  | | At1g72330 ,RAFL09-16-J21  alanine aminotransferase, putative similar to alanine aminotransferase 2 SP|P34106 from Panicum miliaceum, SP|P52894 from Hordeum vulgare, GI:4730884 from Oryza sativa | | | | | | |
|  |  | RAFL05-16-O12 | At1g27930 / expressed protein | |  |  |  |  |  | | --- | --- | --- | --- | --- | |  |  |  |  |  | | RAFL05-16-O12 ,At1g27930  expressed protein contains Pfam profile PF04669: Protein of unknown function (DUF579) | | | | | | |
|  |  | RAFL05-21-L05 | At1g60170 / expressed protein | |  |  |  |  |  | | --- | --- | --- | --- | --- | |  |  |  |  |  | | RAFL05-21-L05 ,At1g60170  pre-mRNA processing ribonucleoprotein binding region-containing protein similar to U4/U6 snRNP-associated 61 kDa protein [Homo sapiens] GI:18249847; contains Pfam profile PF01798: Putative snoRNA binding domain | | | | | | |
|  |  | RAFL09-14-E14 | At1g20560 / AMP-dependent synthetase and ligase family | |  |  |  |  |  | | --- | --- | --- | --- | --- | |  |  |  |  |  | | RAFL09-14-E14 ,At1g20560  AMP-dependent synthetase and ligase family protein similar to AMP-binding protein GI:1903034 from [Brassica napus]; contains Pfam AMP-binding domain PF00501; identical to adenosine monophosphate binding protein 1 AMPBP1 (AMPBP1) GI:20799710 | | | | | | |
|  |  | RAFL08-11-M01 | At1g67580 / protein kinase family | |  |  |  |  |  | | --- | --- | --- | --- | --- | |  |  |  |  |  | | RAFL08-11-M01 ,At1g67580  protein kinase family protein contains protein kinase domain, Pfam:PF00069 | | | | | | |
|  |  | RAFL11-04-K22 | At1g29050 / expressed protein | |  |  |  |  |  | | --- | --- | --- | --- | --- | |  |  |  |  |  | | RAFL11-04-K22 ,At1g29050  expressed protein similar to hypothetical protein GB:AAB67625 GI:2342727 from [Arabidopsis thaliana] | | | | | | |
|  |  | RAFL04-20-P06 | At1g05170 / galactosyltransferase family | |  |  |  |  |  | | --- | --- | --- | --- | --- | |  |  |  |  |  | | RAFL04-20-P06 ,At1g05170  galactosyltransferase family protein | | | | | | |
|  |  | RAFL06-16-D20 | At1g80210 / hypothetical protein | |  |  |  |  |  | | --- | --- | --- | --- | --- | |  |  |  |  |  | | At1g80210 ,RAFL06-16-D20  expressed protein | | | | | | |
|  |  | RAFL02-05-J03 | At1g67730 / short chain dehydrogenase/reductase family protein (b-keto acyl reductase, putative) | |  |  |  |  |  | | --- | --- | --- | --- | --- | |  |  |  |  |  | | RAFL02-05-J03 ,At1g67730  b-keto acyl reductase, putative (GLOSSY8) similar to b-keto acyl reductase GI:2586127 from [Hordeum vulgare] | | | | | | |
|  |  | RAFL07-18-E24 | At1g03400 / 2-oxoglutarate-dependent dioxygenase, putative | |  |  |  |  |  | | --- | --- | --- | --- | --- | |  |  |  |  |  | | RAFL07-18-E24 ,At1g03400  2-oxoglutarate-dependent dioxygenase, putative similar to 2A6 (GI:599622) and tomato ethylene synthesis regulatory protein E8 (SP|P10967); similar to ESTs emb|Z34690, gb|T04168, gb|H37738, gb|T76913, gb|T43801, amd gb|T21964 | | | | | | |
|  |  | RAFL06-16-K07 | At1g73720 / transducin / WD-40 repeat protein family | |  |  |  |  |  | | --- | --- | --- | --- | --- | |  |  |  |  |  | | RAFL06-16-K07 ,At1g73720  transducin family protein / WD-40 repeat family protein contains 5 WD-40 repeats (PF00400); similar to Will die slowly protein (SP:Q9V3J8)[Drosophila melanogaster] | | | | | | |
|  |  | RAFL05-19-L21 | At1g60650 / glycine-rich RNA-binding protein, putative | |  |  |  |  |  | | --- | --- | --- | --- | --- | |  |  |  |  |  | | At1g60650 ,RAFL05-19-L21  glycine-rich RNA-binding protein, putative similar to RNA binding protein(RZ-1) GI:1435061 from [Nicotiana sylvestris]; contains InterPro entry IPR000504: RNA-binding region RNP-1 (RNA recognition motif) (RRM) | | | | | | |
|  |  | RAFL06-09-B20 | At1g80400 / C3HC4-type zinc finger protein family | |  |  |  |  |  | | --- | --- | --- | --- | --- | |  |  |  |  |  | | RAFL06-09-B20 ,At1g80400  zinc finger (C3HC4-type RING finger) family protein low similarity to SP|Q90972 RING finger protein 13 {Gallus gallus}; contains Pfam profile: PF00097 zinc finger, C3HC4 type (RING finger) | | | | | | |
|  |  | RAFL07-08-A08 | At1g06780 / glycosyltransferase family 8 | |  |  |  |  |  | | --- | --- | --- | --- | --- | |  |  |  |  |  | | At1g06780 ,RAFL07-08-A08  glycosyl transferase family 8 protein contains Pfam profile: PF01501 glycosyl transferase family 8 | | | | | | |
|  |  | RAFL09-17-E24 | At1g04140 / transducin / WD-40 repeat protein family | |  |  |  |  |  | | --- | --- | --- | --- | --- | |  |  |  |  |  | | RAFL09-17-E24 ,At1g04140  transducin family protein / WD-40 repeat family protein contains 4 WD-40 repeats (PF00400); similar to neural cell adhesion molecule 2, large isoform precursor gb|M76710 from Xenopus laevis, and beta transducin from S. cerevisiae gb|Q05946. ESTs gb|N65081 gb|Z30910, gb|Z34190, gb|Z34611, gb|R30101, gb|H36304, and gb|N65606 come from | | | | | | |
|  |  | RAFL05-21-H02 | At1g15920 / BTG1 binding factor 1 -related | |  |  |  |  |  | | --- | --- | --- | --- | --- | |  |  |  |  |  | | RAFL05-21-H02 ,At1g15920  CCR4-NOT transcription complex protein, putative similar to SWISS-PROT:Q60809 CCR4-NOT transcription complex, subunit 7 (CCR4-associated factor 1, (CAF1) [Mus musculus] | | | | | | |
|  |  | RAFL09-17-J17 | At1g49160 / protein kinase family | |  |  |  |  |  | | --- | --- | --- | --- | --- | |  |  |  |  |  | | RAFL09-17-J17 ,At1g49160  protein kinase family protein contains protein kinase domain, Pfam:PF00069 | | | | | | |
|  |  | RAFL09-07-D22 | At1g16670 / protein kinase family | |  |  |  |  |  | | --- | --- | --- | --- | --- | |  |  |  |  |  | | RAFL09-07-D22 ,At1g16670  protein kinase family protein contains protein kinase domain, Pfam:PF00069; similar to receptor-like serine/threonine kinase GI:2465923 from [Arabidopsis thaliana] | | | | | | |
|  |  | RAFL04-18-D20 | At1g78570 / NAD-dependent epimerase/dehydratase family | |  |  |  |  |  | | --- | --- | --- | --- | --- | |  |  |  |  |  | | At1g78570 ,RAFL04-18-D20  NAD-dependent epimerase/dehydratase family protein similar to dTDP-glucose 4,6-dehydratase from Aneurinibacillus thermoaerophilus GI:16357461, RmlB from Leptospira borgpetersenii GI:4234803; contains Pfam profile PF01370 NAD dependent epimerase/dehydratase family | | | | | | |
|  |  | RAFL04-19-J24 | At1g20920 / DEAD box RNA helicase, putative | |  |  |  |  |  | | --- | --- | --- | --- | --- | |  |  |  |  |  | | At1g20920 ,RAFL04-19-J24  DEAD box RNA helicase, putative similar to RNA helicase [Rattus norvegicus] GI:897915; contains Pfam profiles PF00270: DEAD/DEAH box helicase, PF00271: Helicase conserved C-terminal domain | | | | | | |
|  |  | RAFL07-14-O09 | At1g49710 / fucosyltransferase, putative | |  |  |  |  |  | | --- | --- | --- | --- | --- | |  |  |  |  |  | | RAFL07-14-O09 ,At1g49710  fucosyltransferase-like protein, putative / FucT2, putative / FucTB, putative (FUT12) identical to Putative fucosyltransferase-like protein (FucTB) (FucT2) (AtFUT12) (Swiss-Prot:Q9FX97) [Arabidopsis thaliana]; similar to glycoprotein 3-alpha-L-fucosyltransferase A (SP:Q9LJK1) [Arabidopsis thaliana]; contains Pfam profile PF00852: Fucosyl transferase | | | | | | |
|  |  | RAFL09-18-J20 | At1g52740 / histone H2A, putative | |  |  |  |  |  | | --- | --- | --- | --- | --- | |  |  |  |  |  | | RAFL09-18-J20 ,At1g52740  histone H2A, putative similar to histone H2A.F/Z Arabidopsis thaliana GI:2407800; contains Pfam profile PF00125 Core histone H2A/H2B/H3/H4 | | | | | | |
|  |  | RAFL05-12-O04 | At1g16010 / expressed protein | |  |  |  |  |  | | --- | --- | --- | --- | --- | |  |  |  |  |  | | At1g16010 ,RAFL05-12-O04  magnesium transporter CorA-like family protein (MRS2-1) low similarity to SP|Q01926 RNA splicing protein MRS2, mitochondrial precursor {Saccharomyces cerevisiae}; contains Pfam profile PF01544: CorA-like Mg2+ transporter protein | | | | | | |
|  |  | RAFL07-13-L09 | At1g33980 / expressed protein | |  |  |  |  |  | | --- | --- | --- | --- | --- | |  |  |  |  |  | | At1g33980 ,RAFL07-13-L09  Smg-4/UPF3 family protein contains Pfam PF03467: Smg-4/UPF3 family; similar to hUPF3B (GI:12232324) [Homo sapiens] | | | | | | |
|  |  | RAFL05-08-M04 | At1g61740 / expressed protein | |  |  |  |  |  | | --- | --- | --- | --- | --- | |  |  |  |  |  | | RAFL05-08-M04 ,At1g61740  expressed protein contains Pfam profile: PF01925 domain of unknown function DUF81; identical to cDNA hypothetical protein, partial GI:4079631 | | | | | | |
|  |  | RAFL03-01-D05 | At1g07080 / expressed protein | |  |  |  |  |  | | --- | --- | --- | --- | --- | |  |  |  |  |  | | RAFL03-01-D05 ,At1g07080  gamma interferon responsive lysosomal thiol reductase family protein / GILT family protein similar to SP|P13284 Gamma-interferon inducible lysosomal thiol reductase precursor {Homo sapiens}; contains Pfam profile PF03227: Gamma interferon inducible lysosomal thiol reductase (GILT) | | | | | | |
|  |  | RAFL08-13-A20 | At1g33230 / expressed protein | |  |  |  |  |  | | --- | --- | --- | --- | --- | |  |  |  |  |  | | At1g33230 ,RAFL08-13-A20  expressed protein | | | | | | |
|  |  | RAFL05-09-P18 | At1g53320 / F-box containing tubby family protein | |  |  |  |  |  | | --- | --- | --- | --- | --- | |  |  |  |  |  | | RAFL05-09-P18 ,At1g53320  F-box family protein / tubby family protein (TULP7) similar to Tubby related protein 2 (Tubby-like protein 2) (P4-6 protein) (Fragment) (SP:P46686) [Mus musculus]; similar to phosphodiesterase (GI:467578) [Mus musculus]; similar to Tubby protein homolog 1. (Swiss-Prot:Q09306) [Caenorhabditis elegans] contains Pfam profile: PF01167: Tub family; contains Pfam PF00646: F-box domain | | | | | | |
|  |  | RAFL08-12-N08 | At1g71040 / multicopper oxidase, type 1 family | |  |  |  |  |  | | --- | --- | --- | --- | --- | |  |  |  |  |  | | At1g71040 ,RAFL08-12-N08  multi-copper oxidase type I family protein similar to SP|P07788 Spore coat protein A {Bacillus subtilis}; contains Pfam profile PF00394: Multicopper oxidase | | | | | | |
|  |  | RAFL04-10-F21 | At1g61250 / secretory carrier membrane protein | |  |  |  |  |  | | --- | --- | --- | --- | --- | |  |  |  |  |  | | At1g61250 ,RAFL04-10-F21  secretory carrier membrane protein (SCAMP) family protein (SC3) contains Pfam domain, PF04144: SCAMP family | | | | | | |
|  |  | RAFL07-18-M23 | At1g10080 / unknown similar to AGI | |  |  |  |  |  | | --- | --- | --- | --- | --- | |  |  |  |  |  | | RAFL07-18-M23 ,At1g10080  http://rarge.gsc.riken.go.jp/microarray/microarray\_data2.pl?ALL\_EX=on&NC\_FLAG=display&LOG=on&ID=RAFL07-18-M23 | | | | | | |
|  |  | RAFL04-13-M11 | At1g04260 / expressed protein | |  |  |  |  |  | | --- | --- | --- | --- | --- | |  |  |  |  |  | | At1g04260 ,RAFL04-13-M11  prenylated rab acceptor (PRA1) family protein weak similarity to prenylated Rab acceptor [Mus musculus] GI:7716652; contains Pfam profile PF03208: Prenylated rab acceptor (PRA1) | | | | | | |
|  |  | RAFL07-11-D20 | At1g16860 / expressed protein | |  |  |  |  |  | | --- | --- | --- | --- | --- | |  |  |  |  |  | | At1g16860 ,RAFL07-11-D20  merozoite surface protein-related contains weak similarity to merozoite surface protein [Plasmodium falciparum] gi|12043655|gb|AAG47601 | | | | | | |
|  |  | RAFL05-08-H19 | At1g68820 / expressed protein | |  |  |  |  |  | | --- | --- | --- | --- | --- | |  |  |  |  |  | | RAFL05-08-H19 ,At1g68820  membrane protein, putative contains 7 transmembrane domains; similar to inhibitor of apoptosis-2 IAP-2 (GI:20043383) [Mamestra configurata nucleopolyhedrovirus] | | | | | | |
|  |  | RAFL07-13-G24 | At1g03040 / bHLH protein | |  |  |  |  |  | | --- | --- | --- | --- | --- | |  |  |  |  |  | | At1g03040 ,RAFL07-13-G24  basic helix-loop-helix (bHLH) family protein component of the pyruvate dehydrogenase complex E3, contains PF|00010 helix-loop-helix DNA-binding domain. ESTs gb|T45640 and gb|T22783 come from this gene | | | | | | |
|  |  | RAFL09-06-D22 | At1g79690 / MutT/nudix family protein | |  |  |  |  |  | | --- | --- | --- | --- | --- | |  |  |  |  |  | | At1g79690 ,RAFL09-06-D22  MutT/nudix family protein contains Pfam NUDIX domain [PF00293]; very low similarity to Chain A and Chain B of Escherichia coli isopentenyl diphosphate:dimethylallyl diphosphate isomerase [gi:15826361] [gi:15826360] | | | | | | |
|  |  | RAFL09-15-I11 | At1g37130 / nitrate reductase 2 (NR2) | |  |  |  |  |  | | --- | --- | --- | --- | --- | |  |  |  |  |  | | RAFL09-15-I11 ,At1g37130  nitrate reductase 2 (NR2) identical to SP|P11035 Nitrate reductase 2 (formerly EC 1.6.6.1) (NR2) {Arabidopsis thaliana} | | | | | | |
|  |  | RAFL09-16-H09 | At1g20980 / SPL1-related protein -related | |  |  |  |  |  | | --- | --- | --- | --- | --- | |  |  |  |  |  | | RAFL09-16-H09 ,At1g20980  SPL1-Related2 protein (SPL1R2) strong similarity to SPL1-Related2 protein [Arabidopsis thaliana] GI:6006427; contains Pfam profile PF03110: SBP domain | | | | | | |
|  |  | RAFL07-07-H04 | At1g22710 / sucrose transporter SUC2 (sucrose-proton symporter) | |  |  |  |  |  | | --- | --- | --- | --- | --- | |  |  |  |  |  | | RAFL07-07-H04 ,At1g22710  sucrose transporter / sucrose-proton symporter (SUC2) nearly identical to sucrose-proton symporter SUC2 [Arabidopsis thaliana] GI:407092 | | | | | | |
|  |  | RAFL06-14-H20 | At1g04850 / expressed protein | |  |  |  |  |  | | --- | --- | --- | --- | --- | |  |  |  |  |  | | RAFL06-14-H20 ,At1g04850  ubiquitin-associated (UBA)/TS-N domain-containing protein weak similarity to SP|P45974 Ubiquitin carboxyl-terminal hydrolase 5 (EC 3.1.2.15) {Homo sapiens}; contains Pfam profile PF00627: UBA/TS-N domain | | | | | | |
|  |  | RAFL04-09-P08 | At1g32490 / RNA helicase, putative | |  |  |  |  |  | | --- | --- | --- | --- | --- | |  |  |  |  |  | | RAFL04-09-P08 ,At1g32490  RNA helicase, putative similar to ATP-dependent RNA helicase #3 [Homo sapiens] GI:3107913; contains Pfam profiles PF04408: Helicase associated domain (HA2), PF00271: Helicase conserved C-terminal domain | | | | | | |
|  |  | RAFL06-15-D19 | At1g13580 / longevity assurance protein -related | |  |  |  |  |  | | --- | --- | --- | --- | --- | |  |  |  |  |  | | RAFL06-15-D19 ,At1g13580  longevity-assurance (LAG1) family protein similar to Alternaria stem canker resistance protein (ASC1) [Lycopersicon esculentum] GI:7688742; contains Pfam profile PF03798: Longevity-assurance protein (LAG1) | | | | | | |
|  |  | RAFL03-07-O21 | At1g19570 / dehydroascorbate reductase, putative | |  |  |  |  |  | | --- | --- | --- | --- | --- | |  |  |  |  |  | | RAFL03-07-O21 ,At1g19570  dehydroascorbate reductase, putative similar to GB:BAA90672 from (Oryza sativa) | | | | | | |
|  |  | RAFL08-10-J10 | At1g04990 / zinc finger protein 2 -related | |  |  |  |  |  | | --- | --- | --- | --- | --- | |  |  |  |  |  | | RAFL08-10-J10 ,At1g04990  zinc finger (CCCH-type) family protein contains Pfam domain, PF00642: Zinc finger C-x8-C-x5-C-x3-H type (and similar) | | | | | | |
|  |  | RAFL09-13-D16 | At1g53210 / expressed protein | |  |  |  |  |  | | --- | --- | --- | --- | --- | |  |  |  |  |  | | RAFL09-13-D16 ,At1g53210  sodium/calcium exchanger family protein / calcium-binding EF hand family protein contains Pfam profiles: PF01699 sodium/calcium exchanger protein, PF00036 EF hand | | | | | | |
|  |  | RAFL09-14-L13 | At1g63420 / expressed protein | |  |  |  |  |  | | --- | --- | --- | --- | --- | |  |  |  |  |  | | RAFL09-14-L13 ,At1g63420  expressed protein | | | | | | |
|  |  | RAFL05-16-L12 | At1g22710 / sucrose transporter SUC2 (sucrose-proton symporter) | |  |  |  |  |  | | --- | --- | --- | --- | --- | |  |  |  |  |  | | At1g22710 ,RAFL05-16-L12  sucrose transporter / sucrose-proton symporter (SUC2) nearly identical to sucrose-proton symporter SUC2 [Arabidopsis thaliana] GI:407092 | | | | | | |
|  |  | RAFL04-13-J08 | At1g80130 / expressed protein | |  |  |  |  |  | | --- | --- | --- | --- | --- | |  |  |  |  |  | | At1g80130 ,RAFL04-13-J08  expressed protein | | | | | | |
|  |  | RAFL08-15-G22 | At1g17440 / expressed protein | |  |  |  |  |  | | --- | --- | --- | --- | --- | |  |  |  |  |  | | At1g17440 ,RAFL08-15-G22  transcription initiation factor IID (TFIID) subunit A family protein similar to SP|Q16514 Transcription initiation factor TFIID 20/15 kDa subunits (TAFII-20/TAFII-15) {Homo sapiens}; contains Pfam profile PF03847: Transcription initiation factor TFIID subunit A | | | | | | |
|  |  | RAFL09-16-K08 | At1g70810 / C2 domain-containing protein | |  |  |  |  |  | | --- | --- | --- | --- | --- | |  |  |  |  |  | | RAFL09-16-K08 ,At1g70810  C2 domain-containing protein similar to zinc finger and C2 domain protein GI:9957238 from [Arabidopsis thaliana] | | | | | | |
|  |  | RAFL06-13-N05 | At1g47240 / NRAMP metal ion transporter 2, putative (NRAMP2) | |  |  |  |  |  | | --- | --- | --- | --- | --- | |  |  |  |  |  | | RAFL06-13-N05 ,At1g47240  NRAMP metal ion transporter 2, putative (NRAMP2) similar to metal transporter Nramp3 [Arabidopsis thaliana] gi|6468012|gb|AAF13278; member of the natural resistance-associated macrophage protein (NRAMP) metal transporter family, PMID:11500563 | | | | | | |
|  |  | RAFL07-13-I02 | At1g30810 / expressed protein | |  |  |  |  |  | | --- | --- | --- | --- | --- | |  |  |  |  |  | | At1g30810 ,RAFL07-13-I02  transcription factor jumonji (jmj) family protein / zinc finger (C5HC2 type) family protein contains similarity to Swiss-Prot:P29375 retinoblastoma-binding protein 2 (RBBP-2) [Homo sapiens]; contains Pfam domains PF02375: jmjN domain and PF02373: jmjC domain; intron between exons 6 and 7 was required to circumvent a frameshift. There could be an underlying sequence error. | | | | | | |
|  |  | RAFL05-09-A05 | At1g63490 / RB-binding protein -related | |  |  |  |  |  | | --- | --- | --- | --- | --- | |  |  |  |  |  | | At1g63490 ,RAFL05-09-A05  transcription factor jumonji (jmjC) domain-containing protein similar to PLU-1 protein (GI:4902724) [Homo sapiens] and PLU1 (GI:22726257) [Mus musculus]; similar to Retinoblastoma-binding protein 2 (RBBP-2) (SP:P29375) {Homo sapiens}; contains Pfam PF02373: jmjC domain | | | | | | |
|  |  | RAFL05-08-K14 | At1g72175 / C3HC4-type zinc finger protein family | |  |  |  |  |  | | --- | --- | --- | --- | --- | |  |  |  |  |  | | At1g72175 ,RAFL05-08-K14  zinc finger (C3HC4-type RING finger) family protein contains Pfam profile: PF00097 zinc finger, C3HC4 type (RING finger) | | | | | | |
|  |  | RAFL02-06-O21 | At1g26665 / expressed protein | |  |  |  |  |  | | --- | --- | --- | --- | --- | |  |  |  |  |  | | At1g26665 ,RAFL02-06-O21  expressed protein | | | | | | |
|  |  | RAFL07-07-K16 | At1g35670 / calcium-dependent protein kinase (CDPK) | |  |  |  |  |  | | --- | --- | --- | --- | --- | |  |  |  |  |  | | RAFL07-07-K16 ,At1g35670  calcium-dependent protein kinase 2 (CDPK2) identical to calcium-dependent protein kinase [Arabidopsis thaliana] gi|604881|dbj|BAA04830; contains protein kinase domain, Pfam:PF00069; contains EF hand domain (calcium-binding EF-hand), Pfam:PF00036, INTERPRO:IPR002048 | | | | | | |
|  |  | RAFL06-07-P06 | At1g72360 / ethylene responsive element binding factor (EREBP), putative | |  |  |  |  |  | | --- | --- | --- | --- | --- | |  |  |  |  |  | | RAFL06-07-P06 ,At1g72360  ethylene-responsive element-binding protein, putative contains Pfam profile: PF00847 AP2 domain; similar to ethylene responsive element binding protein (GI:18496063)[Fagus sylvatica] | | | | | | |
|  |  | RAFL05-09-D08 | At1g66160 / expressed protein | |  |  |  |  |  | | --- | --- | --- | --- | --- | |  |  |  |  |  | | RAFL05-09-D08 ,At1g66160  U-box domain-containing protein similar to immediate-early fungal elicitor protein CMPG1 [Petroselinum crispum] GI:14582200; contains Pfam profile PF04564: U-box domain | | | | | | |
|  |  | RAFL09-16-B05 | At1g79975 / expressed protein | |  |  |  |  |  | | --- | --- | --- | --- | --- | |  |  |  |  |  | | At1g79975 ,RAFL09-16-B05  expressed protein | | | | | | |
|  |  | RAFL06-12-C16 | At1g56200 / expressed protein | |  |  |  |  |  | | --- | --- | --- | --- | --- | |  |  |  |  |  | | RAFL06-12-C16 ,At1g56200  expressed protein | | | | | | |
|  |  | RAFL04-17-L18 | At1g07670 / calcium-transporting ATPase 4, endoplasmic reticulum-type (calcium pump) | |  |  |  |  |  | | --- | --- | --- | --- | --- | |  |  |  |  |  | | At1g07670 ,RAFL04-17-L18  calcium-transporting ATPase 4, endoplasmic reticulum-type (ECA4) identical to SP|Q9XES1 Calcium-transporting ATPase 4, endoplasmic reticulum-type (EC 3.6.3.8) {Arabidopsis thaliana); contains InterPro Accession IPR006069: Cation transporting ATPase | | | | | | |
|  |  | RAFL05-20-M22 | At1g64740 / tubulin alpha-1 chain (TUA1) | |  |  |  |  |  | | --- | --- | --- | --- | --- | |  |  |  |  |  | | RAFL05-20-M22 ,At1g64740  tubulin alpha-1 chain (TUA1) nearly identical to SP|P11139 Tubulin alpha-1 chain {Arabidopsis thaliana} | | | | | | |
|  |  | RAFL05-18-I06 | At1g28710 / expressed protein | |  |  |  |  |  | | --- | --- | --- | --- | --- | |  |  |  |  |  | | RAFL05-18-I06 ,At1g28710  expressed protein similar to GI:2827651, GI:7527728, GI:4406788, GI:6063544, GI:10764853 from [Arabidopsis thaliana] | | | | | | |
|  |  | RAFL06-13-I03 | At1g18360 / hydrolase, alpha/beta fold family | |  |  |  |  |  | | --- | --- | --- | --- | --- | |  |  |  |  |  | | RAFL06-13-I03 ,At1g18360  hydrolase, alpha/beta fold family protein similar to monoglyceride lipase from [Homo sapiens] GI:14594904, [Mus musculus] GI:2632162,[Rattus norvegicus] GI:19697886; contains Pfam profile PF00561: hydrolase, alpha/beta fold family | | | | | | |
|  |  | RAFL09-11-L17 | At1g10410 / expressed protein | |  |  |  |  |  | | --- | --- | --- | --- | --- | |  |  |  |  |  | | RAFL09-11-L17 ,At1g10410  expressed protein similar to ESTs gb|N96021 and gb|N96863 | | | | | | |
|  |  | RAFL07-08-G04 | At1g68550 / AP2 domain transcription factor, putative | |  |  |  |  |  | | --- | --- | --- | --- | --- | |  |  |  |  |  | | RAFL07-08-G04 ,At1g68550  AP2 domain-containing transcription factor, putative contains Pfam profile: PF00847 AP2 domain | | | | | | |
|  |  | RAFL04-17-N08 | At1g63900 / C3HC4-type zinc finger protein family | |  |  |  |  |  | | --- | --- | --- | --- | --- | |  |  |  |  |  | | At1g63900 ,RAFL04-17-N08  zinc finger (C3HC4-type RING finger) family protein contains Pfam profile: PF00097 zinc finger, C3HC4 type (RING finger) | | | | | | |
|  |  | RAFL09-18-B11 | At1g13195 / C3HC4-type zinc finger protein family | |  |  |  |  |  | | --- | --- | --- | --- | --- | |  |  |  |  |  | | RAFL09-18-B11 ,At1g13195  zinc finger (C3HC4-type RING finger) family protein similar to MTD2 [Medicago truncatula] GI:9294812; contains Pfam profile PF00097: Zinc finger, C3HC4 type (RING finger) | | | | | | |
|  |  | RAFL05-16-H11 | At1g15130 / proline-rich protein family | |  |  |  |  |  | | --- | --- | --- | --- | --- | |  |  |  |  |  | | RAFL05-16-H11 ,At1g15130  hydroxyproline-rich glycoprotein family protein | | | | | | |
|  |  | RAFL08-12-O19 | At1g04690 / potassium channel protein, putative | |  |  |  |  |  | | --- | --- | --- | --- | --- | |  |  |  |  |  | | RAFL08-12-O19 ,At1g04690  potassium channel protein, putative nearly identical to K+ channel protein [Arabidopsis thaliana] GI:1063415; contains Pfam profile PF00248: oxidoreductase, aldo/keto reductase family | | | | | | |
|  |  | RAFL05-05-F21 | At1g34370 / zinc finger protein -related | |  |  |  |  |  | | --- | --- | --- | --- | --- | |  |  |  |  |  | | At1g34370 ,RAFL05-05-F21  zinc finger (C2H2 type) family protein contains Pfam domain, PF00096: Zinc finger, C2H2 type | | | | | | |
|  |  | RAFL05-19-K20 | At1g27510 / hypothetical protein | |  |  |  |  |  | | --- | --- | --- | --- | --- | |  |  |  |  |  | | At1g27510 ,RAFL05-19-K20  expressed protein | | | | | | |
|  |  | RAFL09-09-J08 | At1g47530 / ripening regulated protein, putative | |  |  |  |  |  | | --- | --- | --- | --- | --- | |  |  |  |  |  | | RAFL09-09-J08 ,At1g47530  ripening-responsive protein, putative similar to ripening regulated protein DDTFR18 [Lycopersicon esculentum] GI:12231296; contains Pfam profile PF01554: Uncharacterized membrane protein family | | | | | | |
|  |  | RAFL09-10-K04 | At1g26270 / phosphatidylinositol 3- and 4-kinase family | |  |  |  |  |  | | --- | --- | --- | --- | --- | |  |  |  |  |  | | RAFL09-10-K04 ,At1g26270  phosphatidylinositol 3- and 4-kinase family protein similar to phosphatidylinositol 4-kinase type-II beta [Homo sapiens] GI:20159767; contains Pfam profile PF00454: Phosphatidylinositol 3- and 4-kinase | | | | | | |
|  |  | RAFL07-07-A03 | At1g36730 / Eukaryotic translation initiation factor 5 -related | |  |  |  |  |  | | --- | --- | --- | --- | --- | |  |  |  |  |  | | At1g36730 ,RAFL07-07-A03  eukaryotic translation initiation factor 5, putative / eIF-5, putative similar to SP|P55876 Eukaryotic translation initiation factor 5 (eIF-5) {Zea mays}; contains Pfam profiles PF02020: eIF4-gamma/eIF5/eIF2-epsilon, PF01873: Domain found in IF2B/IF5 | | | | | | |
|  | Cluster:0-1 | |  |  | 35 | 71 | 1084 | 3473 | 0.037540954 | 1.2388514 | 33 |
|  |  | RAFL03-06-N04 | At1g55670 / photosystem I subunit V precursor -related | |  |  |  |  |  | | --- | --- | --- | --- | --- | |  |  |  |  |  | | RAFL03-06-N04 ,At1g55670  photosystem I reaction center subunit V, chloroplast, putative / PSI-G, putative (PSAG) identical to SP|Q9S7N7; similar to SP|Q00327 Photosystem I reaction center subunit V, chloroplast precursor (PSI-G) (Photosystem I 9 kDa protein) {Hordeum vulgare}; contains Pfam profile PF01241: Photosystem I psaG / psaK | | | | | | |
|  |  | RAFL04-19-M17 | At1g32060 / phosphoribulokinase precursor | |  |  |  |  |  | | --- | --- | --- | --- | --- | |  |  |  |  |  | | RAFL04-19-M17 ,At1g32060  phosphoribulokinase (PRK) / phosphopentokinase nearly identical to SP|P25697 Phosphoribulokinase, chloroplast precursor (EC 2.7.1.19) (Phosphopentokinase) (PRKASE) (PRK) {Arabidopsis thaliana} | | | | | | |
|  |  | RAFL06-08-C03 | At1g31580 / ORF1 | |  |  |  |  |  | | --- | --- | --- | --- | --- | |  |  |  |  |  | | At1g31580 ,RAFL06-08-C03  expressed protein identical to ORF1 [Arabidopsis thaliana] gi|457716|emb|CAA50905 | | | | | | |
|  |  | RAFL05-16-P12 | At1g67740 / F12A21.13 | |  |  |  |  |  | | --- | --- | --- | --- | --- | |  |  |  |  |  | | RAFL05-16-P12 ,At1g67740  photosystem II core complex proteins psbY, chloroplast (PSBY) / L-arginine metabolising enzyme identical to SP:O49347 Photosystem II core complex proteins psbY, chloroplast precursor (L-arginine metabolising enzyme) (L-AME) [Contains: Photosystem II protein psbY-1 (psbY-A1); Photosystem II protein psbY-2 (psbY-A2)] [Arabidopsis thaliana] | | | | | | |
|  |  | RAFL05-04-D24 | At1g52230 / photosystem I subunit VI precursor | |  |  |  |  |  | | --- | --- | --- | --- | --- | |  |  |  |  |  | | At1g52230 ,RAFL05-04-D24  photosystem I reaction center subunit VI, chloroplast, putative / PSI-H, putative (PSAH2) identical to SP|Q9SUI6; similar to PSI-H precursor [Nicotiana sylvestris] GI:407355; contains Pfam profile PF03244: Photosystem I reaction centre subunit VI | | | | | | |
|  |  | RAFL06-07-I03 | At1g50900 / expressed protein | |  |  |  |  |  | | --- | --- | --- | --- | --- | |  |  |  |  |  | | At1g50900 ,RAFL06-07-I03  expressed protein | | | | | | |
|  |  | RAFL07-07-O16 | At1g75500 / nodulin MtN21 family protein | |  |  |  |  |  | | --- | --- | --- | --- | --- | |  |  |  |  |  | | At1g75500 ,RAFL07-07-O16  nodulin MtN21 family protein similar to MtN21 GB:CAA75575 GI:2598575 from (Medicago truncatula) (Mol. Plant Microbe Interact. 9 (4), 233-242 (1996)); contains Pfam profile PF00892: Integral membrane protein | | | | | | |
|  |  | RAFL05-03-B20 | At1g52400 / glycosyl hydrolase family 1, beta-glucosidase (BG1) | |  |  |  |  |  | | --- | --- | --- | --- | --- | |  |  |  |  |  | | RAFL05-03-B20 ,At1g52400  glycosyl hydrolase family 1 protein / beta-glucosidase, putative (BG1) contains Pfam PF00232 : Glycosyl hydrolase family 1 domain; TIGRFAM TIGR01233: 6-phospho-beta-galactosidase; identical to GI:6651430 from [Arabidopsis thaliana] | | | | | | |
|  |  | RAFL05-18-I22 | At1g44575 / photosystem II 22kDa protein -related | |  |  |  |  |  | | --- | --- | --- | --- | --- | |  |  |  |  |  | | At1g44575 ,RAFL05-18-I22  photosystem II 22kDa protein, chloroplast / CP22 (PSBS) identical to photosystem II 22 kDa protein, chloroplast [precursor] SP:Q9XF91 from [Arabidopsis thaliana]; contains Pfam profile PF00504: Chlorophyll A-B binding protein | | | | | | |
|  |  | RAFL06-15-C10 | At1g75500 / nodulin MtN21 family protein | |  |  |  |  |  | | --- | --- | --- | --- | --- | |  |  |  |  |  | | At1g75500 ,RAFL06-15-C10  nodulin MtN21 family protein similar to MtN21 GB:CAA75575 GI:2598575 from (Medicago truncatula) (Mol. Plant Microbe Interact. 9 (4), 233-242 (1996)); contains Pfam profile PF00892: Integral membrane protein | | | | | | |
|  |  | RAFL05-04-M03 | At1g32470 / glycine cleavage system H protein precursor -related | |  |  |  |  |  | | --- | --- | --- | --- | --- | |  |  |  |  |  | | At1g32470 ,RAFL05-04-M03  glycine cleavage system H protein, mitochondrial, putative similar to SP|P25855 Glycine cleavage system H protein 1, mitochondrial precursor {Arabidopsis thaliana}; contains Pfam profile PF01597: Glycine cleavage H-protein | | | | | | |
|  |  | RAFL05-11-D11 | At1g55490 / RuBisCo subunit binding-protein beta subunit/60 kDa chaperonin beta subunit | |  |  |  |  |  | | --- | --- | --- | --- | --- | |  |  |  |  |  | | RAFL05-11-D11 ,At1g55490  RuBisCO subunit binding-protein beta subunit, chloroplast / 60 kDa chaperonin beta subunit / CPN-60 beta identical to SWISS-PROT:P21240- RuBisCO subunit binding-protein beta subunit, chloroplast precursor (60 kDa chaperonin beta subunit, CPN-60 beta) [Arabidopsis thaliana] | | | | | | |
|  |  | RAFL07-11-O20 | At1g52980 / GTP-binding protein -related | |  |  |  |  |  | | --- | --- | --- | --- | --- | |  |  |  |  |  | | At1g52980 ,RAFL07-11-O20  GTP-binding family protein contains Pfam domain, PF01926: GTPase of unknown function | | | | | | |
|  |  | RAFL11-02-L02 | At1g67090 / ribulose-bisphosphate carboxylase small unit -related | |  |  |  |  |  | | --- | --- | --- | --- | --- | |  |  |  |  |  | | RAFL11-02-L02 ,At1g67090  ribulose bisphosphate carboxylase small chain 1A / RuBisCO small subunit 1A (RBCS-1A) (ATS1A) identical to SP|P10795 Ribulose bisphosphate carboxylase small chain 1A, chloroplast precursor (EC 4.1.1.39) (RuBisCO small subunit 1A) {Arabidopsis thaliana} | | | | | | |
|  |  | RAFL06-13-A08 | At1g44575 / photosystem II 22kDa protein -related | |  |  |  |  |  | | --- | --- | --- | --- | --- | |  |  |  |  |  | | RAFL06-13-A08 ,At1g44575  photosystem II 22kDa protein, chloroplast / CP22 (PSBS) identical to photosystem II 22 kDa protein, chloroplast [precursor] SP:Q9XF91 from [Arabidopsis thaliana]; contains Pfam profile PF00504: Chlorophyll A-B binding protein | | | | | | |
|  |  | RAFL05-04-H06 | At1g32080 / expressed protein | |  |  |  |  |  | | --- | --- | --- | --- | --- | |  |  |  |  |  | | RAFL05-04-H06 ,At1g32080  membrane protein, putative contains 12 transmembrane domains; similar to yohK (GI:405873) [Escherichia coli] | | | | | | |
|  |  | RAFL06-12-A07 | At1g72930 / disease resistance protein (TIR class), putative | |  |  |  |  |  | | --- | --- | --- | --- | --- | |  |  |  |  |  | | RAFL06-12-A07 ,At1g72930  Toll-Interleukin-Resistance (TIR) domain-containing protein domain signature TIR exists, suggestive of a disease resistance protein. | | | | | | |
|  |  | RAFL08-08-D01 | At1g29660 / GDSL-motif lipase/hydrolase protein | |  |  |  |  |  | | --- | --- | --- | --- | --- | |  |  |  |  |  | | RAFL08-08-D01 ,At1g29660  GDSL-motif lipase/hydrolase family protein low similarity to family II lipase EXL1 [Arabidopsis thaliana] GI:15054382; contains InterPro Entry IPR001087 Lipolytic enzyme, G-D-S-L family | | | | | | |
|  |  | RAFL07-10-G07 | At1g52400 / glycosyl hydrolase family 1, beta-glucosidase (BG1) | |  |  |  |  |  | | --- | --- | --- | --- | --- | |  |  |  |  |  | | At1g52400 ,RAFL07-10-G07  glycosyl hydrolase family 1 protein / beta-glucosidase, putative (BG1) contains Pfam PF00232 : Glycosyl hydrolase family 1 domain; TIGRFAM TIGR01233: 6-phospho-beta-galactosidase; identical to GI:6651430 from [Arabidopsis thaliana] | | | | | | |
|  |  | RAFL09-06-P15 | At1g67090 / ribulose-bisphosphate carboxylase small unit -related | |  |  |  |  |  | | --- | --- | --- | --- | --- | |  |  |  |  |  | | At1g67090 ,RAFL09-06-P15  ribulose bisphosphate carboxylase small chain 1A / RuBisCO small subunit 1A (RBCS-1A) (ATS1A) identical to SP|P10795 Ribulose bisphosphate carboxylase small chain 1A, chloroplast precursor (EC 4.1.1.39) (RuBisCO small subunit 1A) {Arabidopsis thaliana} | | | | | | |
|  |  | RAFL06-10-O24 | At1g15820 / chlorophyll a/b-binding protein Lhcb6 | |  |  |  |  |  | | --- | --- | --- | --- | --- | |  |  |  |  |  | | At1g15820 ,RAFL06-10-O24  chlorophyll A-B binding protein, chloroplast (LHCB6) nearly identical to Lhcb6 protein [Arabidopsis thaliana] GI:4741960; contains Pfam profile PF00504: Chlorophyll A-B binding protein | | | | | | |
|  |  | RAFL05-07-J06 | At1g42970 / glyceraldehyde-3-phosphate dehydrogenase | |  |  |  |  |  | | --- | --- | --- | --- | --- | |  |  |  |  |  | | At1g42970 ,RAFL05-07-J06  glyceraldehyde-3-phosphate dehydrogenase B, chloroplast (GAPB) / NADP-dependent glyceraldehydephosphate dehydrogenase subunit B identical to SP|P25857 Glyceraldehyde 3-phosphate dehydrogenase B, chloroplast precursor (EC 1.2.1.13) (NADP-dependent glyceraldehydephosphate dehydrogenase subunit B) {Arabidopsis thaliana} | | | | | | |
|  |  | RAFL03-06-F08 | At1g67090 / ribulose-bisphosphate carboxylase small unit -related | |  |  |  |  |  | | --- | --- | --- | --- | --- | |  |  |  |  |  | | RAFL03-06-F08 ,At1g67090  ribulose bisphosphate carboxylase small chain 1A / RuBisCO small subunit 1A (RBCS-1A) (ATS1A) identical to SP|P10795 Ribulose bisphosphate carboxylase small chain 1A, chloroplast precursor (EC 4.1.1.39) (RuBisCO small subunit 1A) {Arabidopsis thaliana} | | | | | | |
|  |  | RAFL07-08-C08 | At1g64770 / expressed protein | |  |  |  |  |  | | --- | --- | --- | --- | --- | |  |  |  |  |  | | RAFL07-08-C08 ,At1g64770  expressed protein | | | | | | |
|  |  | RAFL02-04-K03 | At1g20340 / plastocyanin | |  |  |  |  |  | | --- | --- | --- | --- | --- | |  |  |  |  |  | | At1g20340 ,RAFL02-04-K03  plastocyanin similar to plastocyanin GI:1865683 from [Arabidopsis thaliana] | | | | | | |
|  |  | RAFL02-06-A08 | At1g06680 / photosystem II oxygen-evolving complex 23 (OEC23) | |  |  |  |  |  | | --- | --- | --- | --- | --- | |  |  |  |  |  | | At1g06680 ,RAFL02-06-A08  photosystem II oxygen-evolving complex 23 (OEC23) JBC 14:211-238 (2002); identical to 23 kDa polypeptide of oxygen-evolving comlex (OEC) GB:CAA66785 GI:1769905 [Arabidopsis thaliana] | | | | | | |
|  |  | RAFL06-13-H16 | At1g11860 / aminomethyltransferase-related precursor protein | |  |  |  |  |  | | --- | --- | --- | --- | --- | |  |  |  |  |  | | At1g11860 ,RAFL06-13-H16  aminomethyltransferase, putative similar to aminomethyltransferase, mitochondrial precursor SP:O49849 from [Flaveria anomala] | | | | | | |
|  |  | RAFL04-15-A14 | At1g12900 / calcium-binding protein, calreticulin -related | |  |  |  |  |  | | --- | --- | --- | --- | --- | |  |  |  |  |  | | At1g12900 ,RAFL04-15-A14  glyceraldehyde 3-phosphate dehydrogenase, chloroplast, putative / NADP-dependent glyceraldehydephosphate dehydrogenase, putative similar to SP|P25856 Glyceraldehyde 3-phosphate dehydrogenase A, chloroplast precursor (EC 1.2.1.13) (NADP-dependent glyceraldehydephosphate dehydrogenase subunit A) {Arabidopsis thaliana}; contains Pfam profiles PF02800: Glyceraldehyde 3-phosphate dehydrogenase C-terminal domain, PF00044: Glyceraldehyde 3-phosphate dehydrogenase NAD binding domain | | | | | | |
|  |  | RAFL04-13-J22 | At1g09750 / expressed protein | |  |  |  |  |  | | --- | --- | --- | --- | --- | |  |  |  |  |  | | At1g09750 ,RAFL04-13-J22  chloroplast nucleoid DNA-binding protein-related contains Pfam profile PF00026: Eukaryotic aspartyl protease;b similar to CND41, chloroplast nucleoid DNA binding protein (GI:2541876) [Nicotiana tabacum] | | | | | | |
|  |  | RAFL09-06-K21 | At1g67090 / ribulose-bisphosphate carboxylase small unit -related | |  |  |  |  |  | | --- | --- | --- | --- | --- | |  |  |  |  |  | | RAFL09-06-K21 ,At1g67090  ribulose bisphosphate carboxylase small chain 1A / RuBisCO small subunit 1A (RBCS-1A) (ATS1A) identical to SP|P10795 Ribulose bisphosphate carboxylase small chain 1A, chloroplast precursor (EC 4.1.1.39) (RuBisCO small subunit 1A) {Arabidopsis thaliana} | | | | | | |
|  |  | RAFL07-07-I23 | At1g56190 / phosphoglycerate kinase -related | |  |  |  |  |  | | --- | --- | --- | --- | --- | |  |  |  |  |  | | RAFL07-07-I23 ,At1g56190  phosphoglycerate kinase, putative similar to SP|P41758 Phosphoglycerate kinase, chloroplast precursor (EC 2.7.2.3) {Chlamydomonas reinhardtii}; contains Pfam profile PF00162: phosphoglycerate kinase | | | | | | |
|  |  | RAFL07-11-K16 | At1g52000 / jacalin lectin family | |  |  |  |  |  | | --- | --- | --- | --- | --- | |  |  |  |  |  | | At1g52000 ,RAFL07-11-K16  jacalin lectin family protein similar to myrosinase binding protein [Brassica napus] GI:1711296, myrosinase-binding protein homolog [Arabidopsis thaliana] GI:2997767; contains Pfam profile: PF01419 jacalin-like lectin domain | | | | | | |
|  |  | RAFL09-16-K05 | At1g09340 / RNA-binding protein -related | |  |  |  |  |  | | --- | --- | --- | --- | --- | |  |  |  |  |  | | RAFL09-16-K05 ,At1g09340  expressed protein | | | | | | |
|  |  | RAFL05-08-B17 | At1g16410 / cytochrome P450, putative | |  |  |  |  |  | | --- | --- | --- | --- | --- | |  |  |  |  |  | | At1g16410 ,RAFL05-08-B17  cytochrome P450, putative similar to gb|AF069494 cytochrome P450 from Sinapis alba and is a member of the PF|00067 Cytochrome P450 family | | | | | | |
|  |  | RAFL04-09-D24 | At1g42970 / glyceraldehyde-3-phosphate dehydrogenase | |  |  |  |  |  | | --- | --- | --- | --- | --- | |  |  |  |  |  | | At1g42970 ,RAFL04-09-D24  glyceraldehyde-3-phosphate dehydrogenase B, chloroplast (GAPB) / NADP-dependent glyceraldehydephosphate dehydrogenase subunit B identical to SP|P25857 Glyceraldehyde 3-phosphate dehydrogenase B, chloroplast precursor (EC 1.2.1.13) (NADP-dependent glyceraldehydephosphate dehydrogenase subunit B) {Arabidopsis thaliana} | | | | | | |
|  | Cluster:9-1 | |  |  | 32 | 64 | 1087 | 3480 | 0.039249726 | 1.2952409 | 33 |
|  |  | RAFL11-12-C18 | At1g06570 / 4-hydroxyphenylpyruvate dioxygenase (HPD) | |  |  |  |  |  | | --- | --- | --- | --- | --- | |  |  |  |  |  | | RAFL11-12-C18 ,At1g06570  4-hydroxyphenylpyruvate dioxygenase (HPD) identical to 4-hydroxyphenylpyruvate dioxygenase (HPD) SP:P93836 [Arabidopsis thaliana (Mouse-ear cress)] | | | | | | |
|  |  | RAFL11-04-I22 | At1g73260 / trypsin inhibitor -related | |  |  |  |  |  | | --- | --- | --- | --- | --- | |  |  |  |  |  | | At1g73260 ,RAFL11-04-I22  trypsin and protease inhibitor family protein / Kunitz family protein similar to trypsin inhibitor propeptide [Brassica oleracea] GI:841208; contains Pfam profile PF00197: Trypsin and protease inhibitor | | | | | | |
|  |  | RAFL11-09-O05 | At1g06570 / 4-hydroxyphenylpyruvate dioxygenase (HPD) | |  |  |  |  |  | | --- | --- | --- | --- | --- | |  |  |  |  |  | | At1g06570 ,RAFL11-09-O05  4-hydroxyphenylpyruvate dioxygenase (HPD) identical to 4-hydroxyphenylpyruvate dioxygenase (HPD) SP:P93836 [Arabidopsis thaliana (Mouse-ear cress)] | | | | | | |
|  |  | RAFL05-18-O20 | At1g29395 / expressed protein | |  |  |  |  |  | | --- | --- | --- | --- | --- | |  |  |  |  |  | | At1g29395 ,RAFL05-18-O20  stress-responsive protein, putative similar to cold acclimation WCOR413-like protein gamma form [Hordeum vulgare] gi|18449100|gb|AAL69988; similar to stress-regulated protein SAP1 [Xerophyta viscosa] gi|21360378|gb|AAM47505 | | | | | | |
|  |  | RAFL05-10-D11 | At1g73480 / hydrolase, alpha/beta fold family | |  |  |  |  |  | | --- | --- | --- | --- | --- | |  |  |  |  |  | | RAFL05-10-D11 ,At1g73480  hydrolase, alpha/beta fold family protein low similarity to monoglyceride lipase from [Homo sapiens] GI:14594904, [Mus musculus] GI:2632162; contains Pfam profile PF00561: hydrolase, alpha/beta fold family | | | | | | |
|  |  | RAFL06-11-B11 | At1g73260 / trypsin inhibitor -related | |  |  |  |  |  | | --- | --- | --- | --- | --- | |  |  |  |  |  | | RAFL06-11-B11 ,At1g73260  trypsin and protease inhibitor family protein / Kunitz family protein similar to trypsin inhibitor propeptide [Brassica oleracea] GI:841208; contains Pfam profile PF00197: Trypsin and protease inhibitor | | | | | | |
|  |  | RAFL08-08-I15 | At1g11360 / expressed protein | |  |  |  |  |  | | --- | --- | --- | --- | --- | |  |  |  |  |  | | RAFL08-08-I15 ,At1g11360  universal stress protein (USP) family protein contains Pfam PF00582: universal stress protein family domain; similar to ethylene-responsive ER6 protein (GI:5669654) [Lycopersicon esculentum] | | | | | | |
|  |  | RAFL09-16-I11 | At1g69490 / No apical meristem (NAM) protein family | |  |  |  |  |  | | --- | --- | --- | --- | --- | |  |  |  |  |  | | At1g69490 ,RAFL09-16-I11  no apical meristem (NAM) family protein similar to N-term half of NAC domain protein NAM [Arabidopsis thaliana] GI:4325282 | | | | | | |
|  |  | RAFL08-19-M03 | At1g07040 / expressed protein | |  |  |  |  |  | | --- | --- | --- | --- | --- | |  |  |  |  |  | | At1g07040 ,RAFL08-19-M03  expressed protein | | | | | | |
|  |  | RAFL08-10-M13 | At1g17020 / oxidoreductase, 2OG-Fe(II) oxygenase family | |  |  |  |  |  | | --- | --- | --- | --- | --- | |  |  |  |  |  | | At1g17020 ,RAFL08-10-M13  oxidoreductase, 2OG-Fe(II) oxygenase family protein similar to flavonol synthase [Petunia x hybrida][GI:311658], leucoanthocyanidin dioxygenase [Malus domestica][SP|P51091]; contains PF03171 2OG-Fe(II) oxygenase superfamily domain | | | | | | |
|  |  | RAFL07-16-P10 | At1g77120 / alcohol dehydrogenase (ADH) | |  |  |  |  |  | | --- | --- | --- | --- | --- | |  |  |  |  |  | | RAFL07-16-P10 ,At1g77120  alcohol dehydrogenase (ADH) identical to alcohol dehydrogenase GI:469467 from (Arabidopsis thaliana) | | | | | | |
|  |  | RAFL05-09-D10 | At1g03220 / expressed protein | |  |  |  |  |  | | --- | --- | --- | --- | --- | |  |  |  |  |  | | At1g03220 ,RAFL05-09-D10  extracellular dermal glycoprotein, putative / EDGP, putative similar to extracellular dermal glycoprotein EDGP precursor [Daucus carota] GI:285741 | | | | | | |
|  |  | RAFL09-10-N03 | At1g53580 / glyoxalase II, putative (hydroxyacylglutathione hydrolase) | |  |  |  |  |  | | --- | --- | --- | --- | --- | |  |  |  |  |  | | RAFL09-10-N03 ,At1g53580  hydroxyacylglutathione hydrolase, putative / glyoxalase II, putative similar to glyoxalase II GI:1644427 from (Arabidopsis thaliana) | | | | | | |
|  |  | RAFL09-15-I16 | At1g05340 / expressed protein | |  |  |  |  |  | | --- | --- | --- | --- | --- | |  |  |  |  |  | | RAFL09-15-I16 ,At1g05340  expressed protein | | | | | | |
|  |  | RAFL09-10-F18 | At1g58360 / amino acid permease I (AAP1) | |  |  |  |  |  | | --- | --- | --- | --- | --- | |  |  |  |  |  | | RAFL09-10-F18 ,At1g58360  amino acid permease I (AAP1) identical to amino acid permease I GI:22641 from [Arabidopsis thaliana] | | | | | | |
|  |  | RAFL05-11-H09 | At1g05680 / glycosyltransferase family | |  |  |  |  |  | | --- | --- | --- | --- | --- | |  |  |  |  |  | | At1g05680 ,RAFL05-11-H09  UDP-glucoronosyl/UDP-glucosyl transferase family protein contains Pfam profile: PF00201 UDP-glucoronosyl and UDP-glucosyl transferase | | | | | | |
|  |  | RAFL04-17-I03 | At1g07040 / expressed protein | |  |  |  |  |  | | --- | --- | --- | --- | --- | |  |  |  |  |  | | At1g07040 ,RAFL04-17-I03  expressed protein | | | | | | |
|  |  | RAFL08-08-O14 | At1g64110 / expressed protein | |  |  |  |  |  | | --- | --- | --- | --- | --- | |  |  |  |  |  | | RAFL08-08-O14 ,At1g64110  AAA-type ATPase family protein contains Pfam domain, PF00004: ATPase, AAA family | | | | | | |
|  |  | RAFL05-18-H15 | At1g64660 / methionine/cystathionine gamma lyase -related | |  |  |  |  |  | | --- | --- | --- | --- | --- | |  |  |  |  |  | | At1g64660 ,RAFL05-18-H15  Cys/Met metabolism pyridoxal-phosphate-dependent enzyme family protein similar to SP|P13254 Methionine gamma-lyase (EC 4.4.1.11) (L-methioninase) {Pseudomonas putida}; contains Pfam profile PF01053: Cys/Met metabolism PLP-dependent enzyme | | | | | | |
|  |  | RAFL05-09-P03 | At1g74020 / strictosidine synthase family | |  |  |  |  |  | | --- | --- | --- | --- | --- | |  |  |  |  |  | | At1g74020 ,RAFL05-09-P03  strictosidine synthase family protein similar to strictosidine synthase [Rauvolfia serpentina][SP|P15324]; contains strictosidine synthase domain PF03088 | | | | | | |
|  |  | RAFL04-19-J05 | At1g61800 / glucose-6-phosphate/phosphate-translocator, putative | |  |  |  |  |  | | --- | --- | --- | --- | --- | |  |  |  |  |  | | At1g61800 ,RAFL04-19-J05  glucose-6-phosphate/phosphate translocator, putative similar to glucose-6-phosphate/phosphate-translocator precursor GI:2997591 from [Pisum sativum] | | | | | | |
|  |  | RAFL05-02-P11 | At1g22370 / glycosyltransferase family | |  |  |  |  |  | | --- | --- | --- | --- | --- | |  |  |  |  |  | | At1g22370 ,RAFL05-02-P11  UDP-glucoronosyl/UDP-glucosyl transferase family protein glycosyltransferase family | | | | | | |
|  |  | RAFL08-09-J19 | At1g02660 / lipase (class 3) family | |  |  |  |  |  | | --- | --- | --- | --- | --- | |  |  |  |  |  | | RAFL08-09-J19 ,At1g02660  lipase class 3 family protein contains Pfam profile PF01764: Lipase | | | | | | |
|  |  | RAFL11-02-N11 | At1g64660 / methionine/cystathionine gamma lyase -related | |  |  |  |  |  | | --- | --- | --- | --- | --- | |  |  |  |  |  | | At1g64660 ,RAFL11-02-N11  Cys/Met metabolism pyridoxal-phosphate-dependent enzyme family protein similar to SP|P13254 Methionine gamma-lyase (EC 4.4.1.11) (L-methioninase) {Pseudomonas putida}; contains Pfam profile PF01053: Cys/Met metabolism PLP-dependent enzyme | | | | | | |
|  |  | RAFL07-13-F20 | At1g78850 / curculin-like (mannose-binding) lectin family | |  |  |  |  |  | | --- | --- | --- | --- | --- | |  |  |  |  |  | | At1g78850 ,RAFL07-13-F20  curculin-like (mannose-binding) lectin family protein low similarity to ser/thr protein kinase from Zea mays [GI:2598067]; contains Pfam lectin (probable mannose binding) domain PF01453 | | | | | | |
|  |  | RAFL05-05-E24 | At1g13990 / expressed protein | |  |  |  |  |  | | --- | --- | --- | --- | --- | |  |  |  |  |  | | At1g13990 ,RAFL05-05-E24  expressed protein | | | | | | |
|  |  | RAFL05-10-D21 | At1g54120 / expressed protein | |  |  |  |  |  | | --- | --- | --- | --- | --- | |  |  |  |  |  | | At1g54120 ,RAFL05-10-D21  expressed protein | | | | | | |
|  |  | RAFL09-10-A12 | At1g68440 / expressed protein | |  |  |  |  |  | | --- | --- | --- | --- | --- | |  |  |  |  |  | | RAFL09-10-A12 ,At1g68440  expressed protein | | | | | | |
|  |  | RAFL05-08-D17 | At1g63720 / expressed protein | |  |  |  |  |  | | --- | --- | --- | --- | --- | |  |  |  |  |  | | RAFL05-08-D17 ,At1g63720  expressed protein similar to putative protein GB:CAA18164 [Arabidopsis thaliana] | | | | | | |
|  |  | RAFL09-10-F14 | At1g63010 / expressed protein | |  |  |  |  |  | | --- | --- | --- | --- | --- | |  |  |  |  |  | | At1g63010 ,RAFL09-10-F14  SPX (SYG1/Pho81/XPR1) domain-containing protein contains Pfam profile PF03105: SPX domain | | | | | | |
|  |  | RAFL05-07-A03 | At1g17870 / expressed protein | |  |  |  |  |  | | --- | --- | --- | --- | --- | |  |  |  |  |  | | At1g17870 ,RAFL05-07-A03  expressed protein contains 6 transmembrane domains; similar to predicted metalloproteases | | | | | | |
|  |  | RAFL09-06-B11 | At1g07720 / beta-ketoacyl-CoA synthase family | |  |  |  |  |  | | --- | --- | --- | --- | --- | |  |  |  |  |  | | RAFL09-06-B11 ,At1g07720  beta-ketoacyl-CoA synthase family protein similar to GB:AAC99312 from [Arabidopsis thaliana] (Plant J. (1999) In press) | | | | | | |
| 1\_21870001\_21900000 | | |  |  | A | B | C | D | P | P' | N |
|  | Cluster:2-2 | |  |  | 1 | 52 | 1 | 4609 | 0.022605369 | 0.045210738 | 2 |
|  |  | RAFL05-19-J02 | At1g60260 / glycosyl hydrolase family 1 | |  |  |  |  |  | | --- | --- | --- | --- | --- | |  |  |  |  |  | | At1g60260 ,RAFL05-19-J02  pseudogene, glycosyl hydrolase family 1 contains Pfam PF00232 : Glycosyl hydrolase family 1 domain; TIGRFAM TIGR01233: 6-phospho-beta-galactosidase; similar to amygdalin hydrolase isoform AH I precursor (GI:16757966) [Prunus serotina]; blastp match of 60% identity and 6.4e-121 P-value to GP|12746303|gb|AAK07429.1|AF321287\_1|AF321287 beta-glucosidase {Musa acuminata} | | | | | | |
|  | Cluster:1-1 | |  |  | 1 | 104 | 1 | 4557 | 0.04453306 | 0.08906612 | 2 |
|  |  | RAFL09-09-H06 | At1g60260 / glycosyl hydrolase family 1 | |  |  |  |  |  | | --- | --- | --- | --- | --- | |  |  |  |  |  | | At1g60260 ,RAFL09-09-H06  pseudogene, glycosyl hydrolase family 1 contains Pfam PF00232 : Glycosyl hydrolase family 1 domain; TIGRFAM TIGR01233: 6-phospho-beta-galactosidase; similar to amygdalin hydrolase isoform AH I precursor (GI:16757966) [Prunus serotina]; blastp match of 60% identity and 6.4e-121 P-value to GP|12746303|gb|AAK07429.1|AF321287\_1|AF321287 beta-glucosidase {Musa acuminata} | | | | | | |
| 2\_480001\_510000 | | |  |  | A | B | C | D | P | P' | N |
|  | Cluster:1-1 | |  |  | 1 | 104 | 1 | 4557 | 0.04453306 | 0.08906612 | 2 |
|  |  | RAFL07-12-M13 | At2g02070 / C2H2-type zinc finger protein -related | |  |  |  |  |  | | --- | --- | --- | --- | --- | |  |  |  |  |  | | At2g02070 ,RAFL07-12-M13  zinc finger (C2H2 type) family protein contains Pfam domain, PF00096: Zinc finger, C2H2 type | | | | | | |
| 3\_9270001\_9300000 | | |  |  | A | B | C | D | P | P' | N |
|  | Cluster:2-1 | |  |  | 3 | 241 | 3 | 4416 | 0.0025160299 | 0.0100641195 | 4 |
|  |  | RAFL04-20-J04 | At3g25530 / gamma hydroxybutyrate dehydrogenase | |  |  |  |  |  | | --- | --- | --- | --- | --- | |  |  |  |  |  | | At3g25530 ,RAFL04-20-J04  6-phosphogluconate dehydrogenase NAD-binding domain-containing protein low similarity to SP|P23523 2-hydroxy-3-oxopropionate reductase (EC 1.1.1.60) (Tartronate semialdehyde reductase) {Escherichia coli}; contains Pfam profile PF03446: NAD binding domain of 6-phosphogluconate dehydrogenase; supporting cDNA gi|15375067|gb|AY044183.1| | | | | | | |
|  |  | RAFL11-12-H04 | At3g25520 / 60S ribosomal protein L5 (RPL5A) | |  |  |  |  |  | | --- | --- | --- | --- | --- | |  |  |  |  |  | | At3g25520 ,RAFL11-12-H04  60S ribosomal protein L5 similar to 60S ribosomal protein L5 GB:P49625 from [Oryza sativa] | | | | | | |
|  |  | RAFL11-07-B21 | At3g25520 / 60S ribosomal protein L5 (RPL5A) | |  |  |  |  |  | | --- | --- | --- | --- | --- | |  |  |  |  |  | | At3g25520 ,RAFL11-07-B21  60S ribosomal protein L5 similar to 60S ribosomal protein L5 GB:P49625 from [Oryza sativa] | | | | | | |
| 2\_16350001\_16380000 | | |  |  | A | B | C | D | P | P' | N |
|  | Cluster:2-0 | |  |  | 2 | 148 | 0 | 4513 | 0.0010281106 | 0.0010281106 | 1 |
|  |  | RAFL09-10-G17 | At2g39310 / jacalin lectin family | |  |  |  |  |  | | --- | --- | --- | --- | --- | |  |  |  |  |  | | RAFL09-10-G17 ,At2g39310  jacalin lectin family protein similar to myrosinase-binding protein homolog [Arabidopsis thaliana] GI:2997767; contains Pfam profile PF01419 jacalin-like lectin domain | | | | | | |
|  |  | RAFL11-09-G01 | At2g39310 / jacalin lectin family | |  |  |  |  |  | | --- | --- | --- | --- | --- | |  |  |  |  |  | | At2g39310 ,RAFL11-09-G01  jacalin lectin family protein similar to myrosinase-binding protein homolog [Arabidopsis thaliana] GI:2997767; contains Pfam profile PF01419 jacalin-like lectin domain | | | | | | |
| 3\_7470001\_7500000 | | |  |  | A | B | C | D | P | P' | N |
|  | Cluster:8-0 | |  |  | 1 | 108 | 0 | 4554 | 0.02337551 | 0.02337551 | 1 |
|  |  | RAFL05-18-O03 | At3g21270 / Dof zinc finger protein | |  |  |  |  |  | | --- | --- | --- | --- | --- | |  |  |  |  |  | | At3g21270 ,RAFL05-18-O03  Dof-type zinc finger domain-containing protein (ADOF2) identical to Dof zinc finger protein ADOF2 GI:3608263 from [Arabidopsis thaliana]; identical to cDNA adof2 mRNA for Dof zinc finger protein GI:3608262; contains Pfam profile PF02701: Dof domain, zinc finger | | | | | | |
| 1\_27000001\_27300000 | | |  |  | A | B | C | D | P | P' | N |
|  | Cluster:9-1 | |  |  | 3 | 93 | 11 | 4556 | 0.0026107915 | 0.026107915 | 10 |
|  |  | RAFL11-04-I22 | At1g73260 / trypsin inhibitor -related | |  |  |  |  |  | | --- | --- | --- | --- | --- | |  |  |  |  |  | | At1g73260 ,RAFL11-04-I22  trypsin and protease inhibitor family protein / Kunitz family protein similar to trypsin inhibitor propeptide [Brassica oleracea] GI:841208; contains Pfam profile PF00197: Trypsin and protease inhibitor | | | | | | |
|  |  | RAFL05-10-D11 | At1g73480 / hydrolase, alpha/beta fold family | |  |  |  |  |  | | --- | --- | --- | --- | --- | |  |  |  |  |  | | RAFL05-10-D11 ,At1g73480  hydrolase, alpha/beta fold family protein low similarity to monoglyceride lipase from [Homo sapiens] GI:14594904, [Mus musculus] GI:2632162; contains Pfam profile PF00561: hydrolase, alpha/beta fold family | | | | | | |
|  |  | RAFL06-11-B11 | At1g73260 / trypsin inhibitor -related | |  |  |  |  |  | | --- | --- | --- | --- | --- | |  |  |  |  |  | | RAFL06-11-B11 ,At1g73260  trypsin and protease inhibitor family protein / Kunitz family protein similar to trypsin inhibitor propeptide [Brassica oleracea] GI:841208; contains Pfam profile PF00197: Trypsin and protease inhibitor | | | | | | |
| 3\_19320001\_19350000 | | |  |  | A | B | C | D | P | P' | N |
|  | Cluster:3-0 | |  |  | 1 | 232 | 0 | 4430 | 0.049967833 | 0.049967833 | 1 |
|  |  | RAFL04-20-A10 | At3g52140 / tetratricopeptide repeat (TPR)-containing protein | |  |  |  |  |  | | --- | --- | --- | --- | --- | |  |  |  |  |  | | At3g52140 ,RAFL04-20-A10  tetratricopeptide repeat (TPR)-containing protein contains Pfam profile PF00515: TPR Domain | | | | | | |
| 3\_17910001\_17940000 | | |  |  | A | B | C | D | P | P' | N |
|  | Cluster:2-1 | |  |  | 2 | 242 | 1 | 4418 | 0.00789916 | 0.01579832 | 2 |
|  |  | RAFL05-09-L11 | At3g48360 / expressed protein | |  |  |  |  |  | | --- | --- | --- | --- | --- | |  |  |  |  |  | | RAFL05-09-L11 ,At3g48360  speckle-type POZ protein-related contains Pfam PF00651 : BTB/POZ domain; similar to Speckle-type POZ protein (SP:O43791) [Homo sapiens] | | | | | | |
|  |  | RAFL07-16-B09 | At3g48360 / expressed protein | |  |  |  |  |  | | --- | --- | --- | --- | --- | |  |  |  |  |  | | RAFL07-16-B09 ,At3g48360  speckle-type POZ protein-related contains Pfam PF00651 : BTB/POZ domain; similar to Speckle-type POZ protein (SP:O43791) [Homo sapiens] | | | | | | |
| 1\_4890001\_4920000 | | |  |  | A | B | C | D | P | P' | N |
|  | Cluster:0-2 | |  |  | 1 | 78 | 1 | 4583 | 0.03360031 | 0.06720062 | 2 |
|  |  | RAFL08-12-J08 | At1g14345 / expressed protein | |  |  |  |  |  | | --- | --- | --- | --- | --- | |  |  |  |  |  | | At1g14345 ,RAFL08-12-J08  expressed protein contains one transmembrane domain | | | | | | |
| 1\_8400001\_8430000 | | |  |  | A | B | C | D | P | P' | N |
|  | Cluster:8-1 | |  |  | 2 | 160 | 1 | 4500 | 0.0035169823 | 0.0070339646 | 2 |
|  |  | RAFL05-01-K23 | At1g23780 / F-box protein family | |  |  |  |  |  | | --- | --- | --- | --- | --- | |  |  |  |  |  | | RAFL05-01-K23 ,At1g23780  F-box family protein contains Pfam PF00646: F-box domain; similar to SP:Q9Y3I1 F-box only protein 7 {Homo sapiens}; similar to SKP1 interacting partner 2 (SKIP2) TIGR\_Ath1:At5g67250 | | | | | | |
|  |  | RAFL05-07-D16 | At1g23800 / mitochondrial aldehyde dehydrogenase (ALDH3) | |  |  |  |  |  | | --- | --- | --- | --- | --- | |  |  |  |  |  | | At1g23800 ,RAFL05-07-D16  aldehyde dehydrogenase, mitochondrial (ALDH3) nearly identical to mitochondrial aldehyde dehydrogenase ALDH3 [Arabidopsis thaliana] gi|19850249|gb|AAL99612; contains Pfam profile PF00171: aldehyde dehydrogenase (NAD) family protein | | | | | | |
| 1\_26160001\_26190000 | | |  |  | A | B | C | D | P | P' | N |
|  | Cluster:1-0 | |  |  | 2 | 147 | 0 | 4514 | 0.0010144025 | 0.0010144025 | 1 |
|  |  | RAFL09-10-F09 | At1g70370 / aromatic rich glyco protein -related | |  |  |  |  |  | | --- | --- | --- | --- | --- | |  |  |  |  |  | | At1g70370 ,RAFL09-10-F09  BURP domain-containing protein / polygalacturonase, putative similar to polygalacturonase isoenzyme 1 beta subunit [Lycopersicon esculentum] GI:170480; contains Pfam profile PF03181: BURP domain | | | | | | |
|  |  | RAFL04-13-K08 | At1g70410 / carbonic anhydrase -related | |  |  |  |  |  | | --- | --- | --- | --- | --- | |  |  |  |  |  | | RAFL04-13-K08 ,At1g70410  carbonic anhydrase, putative / carbonate dehydratase, putative similar to SP|P42737 Carbonic anhydrase 2 (EC 4.2.1.1) (Carbonate dehydratase 2) {Arabidopsis thaliana}; contains Pfam profile PF00484: Carbonic anhydrase | | | | | | |
| 5\_18270001\_18300000 | | |  |  | A | B | C | D | P | P' | N |
|  | Cluster:1-2 | |  |  | 2 | 172 | 0 | 4489 | 0.0013847064 | 0.0013847064 | 1 |
|  |  | RAFL05-04-J09 | At5g45775 / 60S ribosomal protein L11 (RPL11D) | |  |  |  |  |  | | --- | --- | --- | --- | --- | |  |  |  |  |  | | At5g45775 ,RAFL05-04-J09  60S ribosomal protein L11 (RPL11D) | | | | | | |
|  |  | RAFL11-10-E06 | At5g45775 / 60S ribosomal protein L11 (RPL11D) | |  |  |  |  |  | | --- | --- | --- | --- | --- | |  |  |  |  |  | | RAFL11-10-E06 ,At5g45775  60S ribosomal protein L11 (RPL11D) | | | | | | |
| 5\_22800001\_23100000 | | |  |  | A | B | C | D | P | P' | N |
|  | Cluster:5-2 | |  |  | 2 | 124 | 9 | 4528 | 0.03396712 | 0.30570406 | 9 |
|  |  | RAFL03-10-B01 | At5g57120 / expressed protein | |  |  |  |  |  | | --- | --- | --- | --- | --- | |  |  |  |  |  | | At5g57120 ,RAFL03-10-B01  expressed protein weak similarity to SP|Q14978 Nucleolar phosphoprotein p130 {Homo sapiens} | | | | | | |
|  |  | RAFL04-15-B08 | At5g57370 / expressed protein | |  |  |  |  |  | | --- | --- | --- | --- | --- | |  |  |  |  |  | | At5g57370 ,RAFL04-15-B08  expressed protein low similarity to nucleic acid binding protein [Homo sapiens] GI:431953 | | | | | | |
| 1\_28200001\_28500000 | | |  |  | A | B | C | D | P | P' | N |
|  | Cluster:4-2 | |  |  | 2 | 133 | 10 | 4518 | 0.045415256 | 0.4995678 | 11 |
|  |  | RAFL09-17-G19 | At1g76180 / dehydrin -related | |  |  |  |  |  | | --- | --- | --- | --- | --- | |  |  |  |  |  | | RAFL09-17-G19 ,At1g76180  dehydrin (ERD14) identical to SP|P42763 Dehydrin ERD14 {Arabidopsis thaliana} | | | | | | |
|  |  | RAFL05-16-M10 | At1g76200 / expressed protein | |  |  |  |  |  | | --- | --- | --- | --- | --- | |  |  |  |  |  | | RAFL05-16-M10 ,At1g76200  expressed protein | | | | | | |
| 2\_5670001\_5700000 | | |  |  | A | B | C | D | P | P' | N |
|  | Cluster:7-2 | |  |  | 1 | 63 | 0 | 4599 | 0.013725069 | 0.013725069 | 1 |
|  |  | RAFL07-13-F17 | At2g13790 / leucine-rich repeat protein kinase family | |  |  |  |  |  | | --- | --- | --- | --- | --- | |  |  |  |  |  | | At2g13790 ,RAFL07-13-F17  leucine-rich repeat family protein / protein kinase family protein | | | | | | |
| 3\_19800001\_19830000 | | |  |  | A | B | C | D | P | P' | N |
|  | Cluster:8-2 | |  |  | 1 | 61 | 1 | 4600 | 0.026418349 | 0.052836698 | 2 |
|  |  | RAFL03-09-N15 | At3g53420 / plasma membrane intrinsic protein 2A | |  |  |  |  |  | | --- | --- | --- | --- | --- | |  |  |  |  |  | | RAFL03-09-N15 ,At3g53420  plasma membrane intrinsic protein 2A (PIP2A) / aquaporin PIP2.1 (PIP2.1) identical to plasma membrane intrinsic protein 2A SP: P43286 from [Arabidopsis thaliana] | | | | | | |
| 5\_18840001\_18870000 | | |  |  | A | B | C | D | P | P' | N |
|  | Cluster:0-2 | |  |  | 1 | 78 | 1 | 4583 | 0.03360031 | 0.06720062 | 2 |
|  |  | RAFL04-16-N08 | At5g47110 / Lil3 protein | |  |  |  |  |  | | --- | --- | --- | --- | --- | |  |  |  |  |  | | RAFL04-16-N08 ,At5g47110  lil3 protein, putative similar to Lil3 protein [Arabidopsis thaliana] gi|4741966|gb|AAD28780 | | | | | | |
| 1\_4080001\_4110000 | | |  |  | A | B | C | D | P | P' | N |
|  | Cluster:0-2 | |  |  | 1 | 78 | 1 | 4583 | 0.03360031 | 0.06720062 | 2 |
|  |  | RAFL04-19-A20 | At1g12110 / putative NPK1-related protein kinase 2 | |  |  |  |  |  | | --- | --- | --- | --- | --- | |  |  |  |  |  | | At1g12110 ,RAFL04-19-A20  nitrate/chlorate transporter (NRT1.1) (CHL1) identical to nitrate/chlorate transporter SP:Q05085 from [Arabidopsis thaliana]; contains Pfam profile: PF00854 POT family | | | | | | |
| 2\_16200001\_16500000 | | |  |  | A | B | C | D | P | P' | N |
|  | Cluster:2-0 | |  |  | 3 | 147 | 13 | 4500 | 0.013429119 | 0.16114943 | 12 |
|  |  | RAFL09-10-G17 | At2g39310 / jacalin lectin family | |  |  |  |  |  | | --- | --- | --- | --- | --- | |  |  |  |  |  | | RAFL09-10-G17 ,At2g39310  jacalin lectin family protein similar to myrosinase-binding protein homolog [Arabidopsis thaliana] GI:2997767; contains Pfam profile PF01419 jacalin-like lectin domain | | | | | | |
|  |  | RAFL11-09-G01 | At2g39310 / jacalin lectin family | |  |  |  |  |  | | --- | --- | --- | --- | --- | |  |  |  |  |  | | At2g39310 ,RAFL11-09-G01  jacalin lectin family protein similar to myrosinase-binding protein homolog [Arabidopsis thaliana] GI:2997767; contains Pfam profile PF01419 jacalin-like lectin domain | | | | | | |
|  |  | RAFL06-07-L23 | At2g39670 / expressed protein | |  |  |  |  |  | | --- | --- | --- | --- | --- | |  |  |  |  |  | | RAFL06-07-L23 ,At2g39670  radical SAM domain-containing protein similar to hypothetical protein PIR|S76698|S76698 contains Pfam profile PF04055: radical SAM domain protein | | | | | | |
| 3\_7200001\_7500000 | | |  |  | A | B | C | D | P | P' | N |
|  | Cluster:8-1 | |  |  | 2 | 160 | 7 | 4494 | 0.03678091 | 0.29424727 | 8 |
|  |  | RAFL08-14-F20 | At3g20920 / expressed protein | |  |  |  |  |  | | --- | --- | --- | --- | --- | |  |  |  |  |  | | RAFL08-14-F20 ,At3g20920  translocation protein-related contains weak similarity to Drosophila translocation protein 1 (GI:558181) [Drosophila melanogaster] | | | | | | |
|  |  | RAFL05-17-O13 | At3g21230 / 4-coumarate:CoA ligase (4-coumaroyl-CoA synthase) (4CL), putative | |  |  |  |  |  | | --- | --- | --- | --- | --- | |  |  |  |  |  | | At3g21230 ,RAFL05-17-O13  4-coumarate--CoA ligase, putative / 4-coumaroyl-CoA synthase, putative (4CL) similar to 4CL2 [gi:12229665] and 4CL1 [gi:12229649] from [Arabidopsis thaliana], 4CL1 [gi:12229631] from Nicotiana tabacum | | | | | | |
| 4\_7950001\_7980000 | | |  |  | A | B | C | D | P | P' | N |
|  | Cluster:6-2 | |  |  | 1 | 175 | 0 | 4487 | 0.03774394 | 0.03774394 | 1 |
|  |  | RAFL05-13-M21 | At4g15880 / Ulp1 protease family | |  |  |  |  |  | | --- | --- | --- | --- | --- | |  |  |  |  |  | | At4g15880 ,RAFL05-13-M21  Ulp1 protease family protein contains Pfam profile PF02902: Ulp1 protease family, C-terminal catalytic domain; low similarity to sentrin/SUMO-specific protease [Homo sapiens] GI:6906859; identical to cDNA hypothetical protein, partial (1189 bp) GI:2326349 | | | | | | |
| 1\_16860001\_16890000 | | |  |  | A | B | C | D | P | P' | N |
|  | Cluster:8-0 | |  |  | 1 | 108 | 0 | 4554 | 0.02337551 | 0.02337551 | 1 |
|  |  | RAFL05-03-L12 | At1g47128 / cysteine proteinase RD21A | |  |  |  |  |  | | --- | --- | --- | --- | --- | |  |  |  |  |  | | RAFL05-03-L12 ,At1g47128  cysteine proteinase (RD21A) / thiol protease identical to SP|P43297 Cysteine proteinase RD21A precursor (EC 3.4.22.-) {Arabidopsis thaliana}, thiol protease RD21A SP:P43297 from [Arabidopsis thaliana] | | | | | | |
| 3\_18210001\_18240000 | | |  |  | A | B | C | D | P | P' | N |
|  | Cluster:9-1 | |  |  | 1 | 95 | 0 | 4567 | 0.020587604 | 0.020587604 | 1 |
|  |  | RAFL09-07-G15 | At3g49120 / peroxidase, putative | |  |  |  |  |  | | --- | --- | --- | --- | --- | |  |  |  |  |  | | At3g49120 ,RAFL09-07-G15  peroxidase, putative identical to peroxidase [Arabidopsis thaliana] gi|405611|emb|CAA50677 | | | | | | |
| 1\_9960001\_9990000 | | |  |  | A | B | C | D | P | P' | N |
|  | Cluster:7-2 | |  |  | 1 | 63 | 1 | 4598 | 0.027264666 | 0.05452933 | 2 |
|  |  | RAFL07-09-K08 | At1g28380 / expressed protein | |  |  |  |  |  | | --- | --- | --- | --- | --- | |  |  |  |  |  | | At1g28380 ,RAFL07-09-K08  expressed protein | | | | | | |
|  | Cluster:0-2 | |  |  | 1 | 78 | 1 | 4583 | 0.03360031 | 0.06720062 | 2 |
|  |  | RAFL06-12-P20 | At1g28400 / expressed protein | |  |  |  |  |  | | --- | --- | --- | --- | --- | |  |  |  |  |  | | RAFL06-12-P20 ,At1g28400  expressed protein similar to E6 (GI:1000090) [Gossypium barbadense] | | | | | | |
| 1\_22470001\_22500000 | | |  |  | A | B | C | D | P | P' | N |
|  | Cluster:9-1 | |  |  | 1 | 95 | 0 | 4567 | 0.020587604 | 0.020587604 | 1 |
|  |  | RAFL04-19-J05 | At1g61800 / glucose-6-phosphate/phosphate-translocator, putative | |  |  |  |  |  | | --- | --- | --- | --- | --- | |  |  |  |  |  | | At1g61800 ,RAFL04-19-J05  glucose-6-phosphate/phosphate translocator, putative similar to glucose-6-phosphate/phosphate-translocator precursor GI:2997591 from [Pisum sativum] | | | | | | |
| 4\_11820001\_11850000 | | |  |  | A | B | C | D | P | P' | N |
|  | Cluster:0-2 | |  |  | 1 | 78 | 2 | 4582 | 0.049979966 | 0.1499399 | 3 |
|  |  | RAFL04-09-K07 | At4g25050 / acyl carrier protein family | |  |  |  |  |  | | --- | --- | --- | --- | --- | |  |  |  |  |  | | RAFL04-09-K07 ,At4g25050  acyl carrier family protein / ACP family protein similar to Acyl carrier protein, chloroplast precursor from {Spinacia oleracea} SP|P23235, {Casuarina glauca} SP|P93092; contains InterPro accession IPR003881: Isochorismatase | | | | | | |
| 1\_26880001\_26910000 | | |  |  | A | B | C | D | P | P' | N |
|  | Cluster:7-1 | |  |  | 2 | 263 | 3 | 4395 | 0.028701013 | 0.11480405 | 4 |
|  |  | RAFL09-16-J21 | At1g72330 / alanine aminotransferase, putative | |  |  |  |  |  | | --- | --- | --- | --- | --- | |  |  |  |  |  | | At1g72330 ,RAFL09-16-J21  alanine aminotransferase, putative similar to alanine aminotransferase 2 SP|P34106 from Panicum miliaceum, SP|P52894 from Hordeum vulgare, GI:4730884 from Oryza sativa | | | | | | |
|  |  | RAFL06-07-P06 | At1g72360 / ethylene responsive element binding factor (EREBP), putative | |  |  |  |  |  | | --- | --- | --- | --- | --- | |  |  |  |  |  | | RAFL06-07-P06 ,At1g72360  ethylene-responsive element-binding protein, putative contains Pfam profile: PF00847 AP2 domain; similar to ethylene responsive element binding protein (GI:18496063)[Fagus sylvatica] | | | | | | |
| 1\_22800001\_23100000 | | |  |  | A | B | C | D | P | P' | N |
|  | Cluster:10-2 | |  |  | 4 | 105 | 10 | 4544 | 2.3608547E-4 | 0.0023608548 | 10 |
|  |  | RAFL08-15-K01 | At1g62660 / glycosyl hydrolase family 32 | |  |  |  |  |  | | --- | --- | --- | --- | --- | |  |  |  |  |  | | At1g62660 ,RAFL08-15-K01  beta-fructosidase (BFRUCT3) / beta-fructofuranosidase / invertase, vacuolar identical to beta-fructosidase GB:CAA67560 GI:1429209 [Arabidopsis thaliana]; supported by full-length cDNA GI:14517549; identical to cDNA Beta-fructosidase GI:3115854 | | | | | | |
|  |  | RAFL05-03-G20 | At1g62600 / flavin-containing monooxygenase (FMO) family | |  |  |  |  |  | | --- | --- | --- | --- | --- | |  |  |  |  |  | | RAFL05-03-G20 ,At1g62600  flavin-containing monooxygenase family protein / FMO family protein low similarity to flavin-containing monooxygenase 2 from Cavia porcellus [SP|P36366]; contains Pfam profile PF00743 Flavin-binding monooxygenase-like | | | | | | |
|  |  | RAFL04-12-E05 | At1g63010 / expressed protein | |  |  |  |  |  | | --- | --- | --- | --- | --- | |  |  |  |  |  | | At1g63010 ,RAFL04-12-E05  SPX (SYG1/Pho81/XPR1) domain-containing protein contains Pfam profile PF03105: SPX domain | | | | | | |
|  |  | RAFL08-13-K06 | At1g62660 / glycosyl hydrolase family 32 | |  |  |  |  |  | | --- | --- | --- | --- | --- | |  |  |  |  |  | | At1g62660 ,RAFL08-13-K06  beta-fructosidase (BFRUCT3) / beta-fructofuranosidase / invertase, vacuolar identical to beta-fructosidase GB:CAA67560 GI:1429209 [Arabidopsis thaliana]; supported by full-length cDNA GI:14517549; identical to cDNA Beta-fructosidase GI:3115854 | | | | | | |
| 1\_4110001\_4140000 | | |  |  | A | B | C | D | P | P' | N |
|  | Cluster:3-1 | |  |  | 1 | 215 | 0 | 4447 | 0.04632211 | 0.04632211 | 1 |
|  |  | RAFL08-12-B11 | At1g12120 / expressed protein | |  |  |  |  |  | | --- | --- | --- | --- | --- | |  |  |  |  |  | | At1g12120 ,RAFL08-12-B11  expressed protein contains Pfam domain PF05904: Plant protein of unknown function (DUF863) | | | | | | |
| 1\_18930001\_18960000 | | |  |  | A | B | C | D | P | P' | N |
|  | Cluster:1-0 | |  |  | 2 | 147 | 1 | 4513 | 0.0029792225 | 0.005958445 | 2 |
|  |  | RAFL08-09-I24 | At1g52040 / jacalin lectin family | |  |  |  |  |  | | --- | --- | --- | --- | --- | |  |  |  |  |  | | RAFL08-09-I24 ,At1g52040  jacalin lectin family protein nearly identical to myrosinase-binding protein homolog GI:2997767 from [Arabidopsis thaliana]; contains Pfam profile PF01419 jacalin-like lectin domain; identical to cDNA myrosinase-binding protein homolog GI:2997766 | | | | | | |
|  |  | RAFL09-14-K04 | At1g52040 / jacalin lectin family | |  |  |  |  |  | | --- | --- | --- | --- | --- | |  |  |  |  |  | | At1g52040 ,RAFL09-14-K04  jacalin lectin family protein nearly identical to myrosinase-binding protein homolog GI:2997767 from [Arabidopsis thaliana]; contains Pfam profile PF01419 jacalin-like lectin domain; identical to cDNA myrosinase-binding protein homolog GI:2997766 | | | | | | |
|  | Cluster:2-2 | |  |  | 1 | 52 | 2 | 4608 | 0.033719275 | 0.06743855 | 2 |
|  |  | RAFL04-09-C15 | At1g52030 / myrosinase binding protein, putative | |  |  |  |  |  | | --- | --- | --- | --- | --- | |  |  |  |  |  | | At1g52030 ,RAFL04-09-C15  myrosinase-binding protein, putative (F-ATMBP) identical to SP|Q9SAV1 Myrosinase binding protein-like f-AtMBP [Arabidopsis thaliana]; similar to myrosinase binding protein GI:1711295 from [Brassica napus]; contains Pfam PF01419: Jacalin-like lectin domain; identical to cDNA myrosinase-binding protein-like protein (MBP1.2) GI:6760446 | | | | | | |
| 1\_19890001\_19920000 | | |  |  | A | B | C | D | P | P' | N |
|  | Cluster:6-0 | |  |  | 1 | 139 | 0 | 4523 | 0.03002359 | 0.03002359 | 1 |
|  |  | RAFL06-15-K18 | At1g54410 / dehydrin protein family | |  |  |  |  |  | | --- | --- | --- | --- | --- | |  |  |  |  |  | | RAFL06-15-K18 ,At1g54410  dehydrin family protein contains Pfam domain, PF00257: Dehydrin | | | | | | |
| 2\_12900001\_13200000 | | |  |  | A | B | C | D | P | P' | N |
|  | Cluster:3-0 | |  |  | 4 | 229 | 15 | 4415 | 0.013005282 | 0.19507922 | 15 |
|  |  | RAFL09-15-J08 | At2g30620 / histone H1 | |  |  |  |  |  | | --- | --- | --- | --- | --- | |  |  |  |  |  | | At2g30620 ,RAFL09-15-J08  histone H1.2 nearly identical to SP|P26569 Histone H1.2 {Arabidopsis thaliana} | | | | | | |
|  |  | RAFL07-17-K15 | At2g31040 / expressed protein | |  |  |  |  |  | | --- | --- | --- | --- | --- | |  |  |  |  |  | | RAFL07-17-K15 ,At2g31040  ATP synthase protein I -related contains weaks similarity to Swiss-Prot:P08443 ATP synthase protein I [Synechococcus sp.] | | | | | | |
|  |  | RAFL08-14-P10 | At2g30410 / TCP1-chaperonin cofactor A isolog | |  |  |  |  |  | | --- | --- | --- | --- | --- | |  |  |  |  |  | | RAFL08-14-P10 ,At2g30410  tubulin folding cofactor A (KIESEL) identical to cDNA tubulin folding cofactor A, GI:20514256, SP|O04350 Tubulin-specific chaperone A (Tubulin-folding cofactor A) (CFA) (TCP1-chaperonin cofactor A homolog) {Arabidopsis thaliana} | | | | | | |
|  |  | RAFL09-07-B08 | At2g30970 / aspartate aminotransferase, mitochondrial (transaminase A/Asp1) | |  |  |  |  |  | | --- | --- | --- | --- | --- | |  |  |  |  |  | | At2g30970 ,RAFL09-07-B08  aspartate aminotransferase, mitochondrial / transaminase A (ASP1) identical to SP|P46643 Aspartate aminotransferase, mitochondrial precursor (EC 2.6.1.1) (Transaminase A) {Arabidopsis thaliana} | | | | | | |
| 5\_3630001\_3660000 | | |  |  | A | B | C | D | P | P' | N |
|  | Cluster:0-1 | |  |  | 2 | 104 | 3 | 4554 | 0.004895142 | 0.019580567 | 4 |
|  |  | RAFL07-10-E04 | At5g11420 / expressed protein | |  |  |  |  |  | | --- | --- | --- | --- | --- | |  |  |  |  |  | | RAFL07-10-E04 ,At5g11420  expressed protein contains Pfam profile PF04862: Protein of unknown function, DUF642 | | | | | | |
|  |  | RAFL07-11-I06 | At5g11420 / expressed protein | |  |  |  |  |  | | --- | --- | --- | --- | --- | |  |  |  |  |  | | RAFL07-11-I06 ,At5g11420  expressed protein contains Pfam profile PF04862: Protein of unknown function, DUF642 | | | | | | |
| 4\_12450001\_12480000 | | |  |  | A | B | C | D | P | P' | N |
|  | Cluster:0-2 | |  |  | 1 | 78 | 1 | 4583 | 0.03360031 | 0.06720062 | 2 |
|  |  | RAFL05-21-A21 | At4g26850 / expressed protein | |  |  |  |  |  | | --- | --- | --- | --- | --- | |  |  |  |  |  | | RAFL05-21-A21 ,At4g26850  expressed protein | | | | | | |
| 1\_29490001\_29520000 | | |  |  | A | B | C | D | P | P' | N |
|  | Cluster:8-1 | |  |  | 1 | 161 | 0 | 4501 | 0.034741584 | 0.034741584 | 1 |
|  |  | RAFL05-09-G04 | At1g79340 / putative latex-abundant protein | |  |  |  |  |  | | --- | --- | --- | --- | --- | |  |  |  |  |  | | At1g79340 ,RAFL05-09-G04  latex-abundant protein, putative (AMC7) / caspase family protein similar to latex-abundant protein [Hevea brasiliensis] gb:AAD13216; contains Pfam domain, PF00656: ICE-like protease (caspase) p20 domain | | | | | | |
| 5\_8070001\_8100000 | | |  |  | A | B | C | D | P | P' | N |
|  | Cluster:2-0 | |  |  | 1 | 149 | 0 | 4513 | 0.03216813 | 0.03216813 | 1 |
|  |  | RAFL04-20-B22 | At5g24020 / septum site-determining MinD (dbj|BAA90261.1) | |  |  |  |  |  | | --- | --- | --- | --- | --- | |  |  |  |  |  | | RAFL04-20-B22 ,At5g24020  septum site-determining protein (MIND) identical to MinD [Arabidopsis thaliana] GI:6759277; contains Pfam PF00991 : ParA family ATPase | | | | | | |
| 2\_17700001\_17730000 | | |  |  | A | B | C | D | P | P' | N |
|  | Cluster:0-2 | |  |  | 1 | 78 | 1 | 4583 | 0.03360031 | 0.06720062 | 2 |
|  |  | RAFL05-18-J03 | At2g42690 / lipase, putative | |  |  |  |  |  | | --- | --- | --- | --- | --- | |  |  |  |  |  | | RAFL05-18-J03 ,At2g42690  lipase, putative similar to lipase [Dianthus caryophyllus] GI:4103627; contains Pfam profile PF01764: Lipase | | | | | | |
| 1\_29640001\_29670000 | | |  |  | A | B | C | D | P | P' | N |
|  | Cluster:8-1 | |  |  | 1 | 161 | 0 | 4501 | 0.034741584 | 0.034741584 | 1 |
|  |  | RAFL05-03-J16 | At1g79730 / proline-rich protein family | |  |  |  |  |  | | --- | --- | --- | --- | --- | |  |  |  |  |  | | RAFL05-03-J16 ,At1g79730  hydroxyproline-rich glycoprotein family protein contains proline-rich extensin domains, INTERPRO:IPR002965 | | | | | | |
| 4\_13170001\_13200000 | | |  |  | A | B | C | D | P | P' | N |
|  | Cluster:3-1 | |  |  | 1 | 215 | 0 | 4447 | 0.04632211 | 0.04632211 | 1 |
|  |  | RAFL07-11-A20 | At4g28760 / expressed protein | |  |  |  |  |  | | --- | --- | --- | --- | --- | |  |  |  |  |  | | RAFL07-11-A20 ,At4g28760  expressed protein predicted protein. Arabidopsis thaliana | | | | | | |
| 1\_2610001\_2640000 | | |  |  | A | B | C | D | P | P' | N |
|  | Cluster:1-2 | |  |  | 1 | 173 | 0 | 4489 | 0.037315033 | 0.037315033 | 1 |
|  |  | RAFL04-16-H06 | At1g08360 / 60S ribosomal protein L10A (RPL10aA) | |  |  |  |  |  | | --- | --- | --- | --- | --- | |  |  |  |  |  | | At1g08360 ,RAFL04-16-H06  60S ribosomal protein L10A (RPL10aA) similar to 60S ribosomal protein L10A GB:AAC73045 GI:3860277 from [Arabidopsis thaliana] | | | | | | |
| 4\_9000001\_12000000 | | |  |  | A | B | C | D | P | P' | N |
|  | Cluster:6-0 | |  |  | 9 | 131 | 95 | 4428 | 0.0037356832 | 0.104599126 | 28 |
|  |  | RAFL09-09-G01 | At4g19840 / lectin-related | |  |  |  |  |  | | --- | --- | --- | --- | --- | |  |  |  |  |  | | At4g19840 ,RAFL09-09-G01  lectin-related similar to PP2 lectin polypeptide [Cucurbita maxima] GI:410437 | | | | | | |
|  |  | RAFL05-01-C20 | At4g23670 / major latex protein (MLP)-related | |  |  |  |  |  | | --- | --- | --- | --- | --- | |  |  |  |  |  | | At4g23670 ,RAFL05-01-C20  major latex protein-related / MLP-related low similarity to major latex protein {Papaver somniferum}[GI:294060] contains Pfam profile PF00407: Pathogenesis-related protein Bet v I family | | | | | | |
|  |  | RAFL04-10-E08 | At4g25500 / arginine/serine-rich splicing factor RSp40 | |  |  |  |  |  | | --- | --- | --- | --- | --- | |  |  |  |  |  | | At4g25500 ,RAFL04-10-E08  arginine/serine-rich splicing factor RSP40 (RSP40) identical to SP|P92965 Arginine/serine-rich splicing factor RSP40 {Arabidopsis thaliana} | | | | | | |
|  |  | RAFL06-16-E03 | At4g20260 / endomembrane-associated protein | |  |  |  |  |  | | --- | --- | --- | --- | --- | |  |  |  |  |  | | At4g20260 ,RAFL06-16-E03  DREPP plasma membrane polypeptide family protein contains Pfam profile: PF05558 DREPP plasma membrane polypeptide | | | | | | |
|  |  | RAFL04-18-D10 | At4g24520 / NADPH-ferrihemoprotein reductase (NADPH-cytochrome p450 reductase), putative | |  |  |  |  |  | | --- | --- | --- | --- | --- | |  |  |  |  |  | | At4g24520 ,RAFL04-18-D10  NADPH-cytochrome p450 reductase, putative / NADPH-ferrihemoprotein reductase, putative similar to NADPH-ferrihemoprotein reductase NADPH-cytochrome P450 oxydoreductase isoform 1 [Populus balsamifera subsp. trichocarpa x Populus deltoides] GI:13183562, SP|P37116 NADPH-cytochrome P450 reductase (EC 1.6.2.4) (CPR) [Vigna radiata] {Phaseolus aureus} | | | | | | |
|  |  | RAFL04-18-L18 | At4g24120 / expressed protein | |  |  |  |  |  | | --- | --- | --- | --- | --- | |  |  |  |  |  | | At4g24120 ,RAFL04-18-L18  transporter, putative similar to iron-phytosiderophore transporter protein yellow stripe 1 [Zea mays] GI:10770865; contains Pfam profile PF03169: OPT oligopeptide transporter protein | | | | | | |
|  |  | RAFL05-19-A14 | At4g20850 / expressed protein | |  |  |  |  |  | | --- | --- | --- | --- | --- | |  |  |  |  |  | | RAFL05-19-A14 ,At4g20850  subtilase family protein contains similarity to Tripeptidyl-peptidase II (EC 3.4.14.10) (TPP-II) (Tripeptidyl aminopeptidase) (Swiss-Prot:P29144) [Homo sapiens] | | | | | | |
|  |  | RAFL11-12-C05 | At4g20260 / endomembrane-associated protein | |  |  |  |  |  | | --- | --- | --- | --- | --- | |  |  |  |  |  | | RAFL11-12-C05 ,At4g20260  DREPP plasma membrane polypeptide family protein contains Pfam profile: PF05558 DREPP plasma membrane polypeptide | | | | | | |
|  |  | RAFL09-17-I10 | At4g19110 / protein kinase, putative | |  |  |  |  |  | | --- | --- | --- | --- | --- | |  |  |  |  |  | | At4g19110 ,RAFL09-17-I10  protein kinase, putative contains protein kinase domain, Pfam:PF00069 | | | | | | |
| 5\_7020001\_7050000 | | |  |  | A | B | C | D | P | P' | N |
|  | Cluster:9-1 | |  |  | 1 | 95 | 0 | 4567 | 0.020587604 | 0.020587604 | 1 |
|  |  | RAFL05-16-I09 | At5g20830 / sucrose synthase (UDP-glucose-fructose glucosyltransferase/sucrose-UDP glucosyltransferase/SUS1) | |  |  |  |  |  | | --- | --- | --- | --- | --- | |  |  |  |  |  | | At5g20830 ,RAFL05-16-I09  sucrose synthase / sucrose-UDP glucosyltransferase (SUS1) identical to SP|P49040 Sucrose synthase (EC 2.4.1.13) (Sucrose-UDP glucosyltransferase) {Arabidopsis thaliana} | | | | | | |
| 2\_18300001\_18600000 | | |  |  | A | B | C | D | P | P' | N |
|  | Cluster:5-1 | |  |  | 4 | 278 | 9 | 4372 | 0.0060573313 | 0.054515984 | 9 |
|  |  | RAFL08-09-K17 | At2g44670 / senescence-associated protein -related | |  |  |  |  |  | | --- | --- | --- | --- | --- | |  |  |  |  |  | | RAFL08-09-K17 ,At2g44670  senescence-associated protein-related similar to senescence-associated protein SAG102 (GI:22331931) [Arabidopsis thaliana]; | | | | | | |
|  |  | RAFL05-16-D13 | At2g44500 / axi 1 protein from Nicotiana tabacum -related | |  |  |  |  |  | | --- | --- | --- | --- | --- | |  |  |  |  |  | | RAFL05-16-D13 ,At2g44500  expressed protein contains Pfam PF03138: Plant protein family. The function of this family of plant proteins is unknown; previously annotated as 'axi 1 protein from Nicotiana tabacum -related' based on similarity to axi 1 protein (GB:X80301) (GI:559920) from [Nicotiana tabacum], which, due to scienitific fraud was retracted. Retraction in: Schell J. EMBO J 1999 May 17;18(10):2908. PMID:10400497. | | | | | | |
|  |  | RAFL05-19-F23 | At2g44520 / UbiA prenyltransferase family | |  |  |  |  |  | | --- | --- | --- | --- | --- | |  |  |  |  |  | | At2g44520 ,RAFL05-19-F23  UbiA prenyltransferase family protein similar to SP|Q12887 Protoheme IX farnesyltransferase, mitochondrial precursor (EC 2.5.1.-) (Heme O synthase) {Homo sapiens}, SP|P21592 COX10 {Saccharomyces cerevisiae} | | | | | | |
|  |  | RAFL08-18-G13 | At2g44680 / casein kinase II beta chain, putative | |  |  |  |  |  | | --- | --- | --- | --- | --- | |  |  |  |  |  | | RAFL08-18-G13 ,At2g44680  casein kinase II beta chain, putative similar to casein kinase II beta-3 chain (CK II) [Arabidopsis thaliana] SWISS-PROT:O81275 | | | | | | |
| 2\_14490001\_14520000 | | |  |  | A | B | C | D | P | P' | N |
|  | Cluster:4-1 | |  |  | 2 | 308 | 2 | 4351 | 0.024166461 | 0.07249938 | 3 |
|  |  | RAFL11-13-L01 | At2g34520 / ribosomal protein S14p family, mitochondrial precursor | |  |  |  |  |  | | --- | --- | --- | --- | --- | |  |  |  |  |  | | At2g34520 ,RAFL11-13-L01  ribosomal protein S14 mitochondrial family protein identical to ribosomal protein S14 {Arabidopsis thaliana} NCBI\_gi:4583554 | | | | | | |
|  |  | RAFL06-08-D06 | At2g34590 / pyruvate dehydrogenase E1 beta subunit -related | |  |  |  |  |  | | --- | --- | --- | --- | --- | |  |  |  |  |  | | At2g34590 ,RAFL06-08-D06  transketolase family protein similar to SP|O66113 Pyruvate dehydrogenase E1 component, beta subunit (EC 1.2.4.1). {Zymomonas mobilis}; contains Pfam profiles PF02779: Transketolase, pyridine binding domain, PF02780: Transketolase, C-terminal domain | | | | | | |
|  | Cluster:2-2 | |  |  | 1 | 52 | 3 | 4607 | 0.044709165 | 0.13412748 | 3 |
|  |  | RAFL04-17-F09 | At2g34510 / expressed protein | |  |  |  |  |  | | --- | --- | --- | --- | --- | |  |  |  |  |  | | At2g34510 ,RAFL04-17-F09  expressed protein contains Pfam profile PF04862: Protein of unknown function, DUF642 | | | | | | |
| 4\_17310001\_17340000 | | |  |  | A | B | C | D | P | P' | N |
|  | Cluster:2-2 | |  |  | 1 | 52 | 2 | 4608 | 0.033719275 | 0.10115783 | 3 |
|  |  | RAFL05-21-P16 | At4g39460 / mitochondrial carrier protein family | |  |  |  |  |  | | --- | --- | --- | --- | --- | |  |  |  |  |  | | At4g39460 ,RAFL05-21-P16  mitochondrial substrate carrier family protein | | | | | | |
| 5\_25410001\_25440000 | | |  |  | A | B | C | D | P | P' | N |
|  | Cluster:2-0 | |  |  | 2 | 148 | 5 | 4508 | 0.019410595 | 0.11646357 | 6 |
|  |  | RAFL07-09-K24 | At5g64300 / GTP cyclohydrolase II; 3,4-dihydroxy-2-butanone-4-phoshate synthase (emb|CAA03884.1) | |  |  |  |  |  | | --- | --- | --- | --- | --- | |  |  |  |  |  | | At5g64300 ,RAFL07-09-K24  riboflavin biosynthesis protein, putative (RIBA) similar to SP|P47924 {Arabidopsis thaliana}, SP|P51695 Riboflavin biosynthesis protein ribA [Includes: GTP cyclohydrolase II (EC 3.5.4.25); 3,4-dihydroxy-2-butanone 4-phosphate synthase (DHBP synthase)] {Bacillus amyloliquefaciens}; contains Pfam profiles PF00925: GTP cyclohydrolase II, PF00926: 3,4-dihydroxy-2-butanone 4-phosphate synthase | | | | | | |
|  |  | RAFL07-09-O18 | At5g64290 / oxoglutarate/malate translocator, putative | |  |  |  |  |  | | --- | --- | --- | --- | --- | |  |  |  |  |  | | At5g64290 ,RAFL07-09-O18  oxoglutarate/malate translocator, putative similar to SWISS-PROT:Q41364 2-oxoglutarate/malate translocator, chloroplast precursor. [Spinach]{Spinacia oleracea} | | | | | | |
| 5\_22620001\_22650000 | | |  |  | A | B | C | D | P | P' | N |
|  | Cluster:4-2 | |  |  | 2 | 133 | 0 | 4528 | 8.321486E-4 | 8.321486E-4 | 1 |
|  |  | RAFL04-13-A16 | At5g56600 / profilin 5 | |  |  |  |  |  | | --- | --- | --- | --- | --- | |  |  |  |  |  | | At5g56600 ,RAFL04-13-A16  profilin 5 (PRO5) (PRF3) identical to SP|Q9FE63 Profilin 5 {Arabidopsis thaliana} | | | | | | |
|  |  | RAFL04-18-L19 | At5g56670 / 40S ribosomal protein S30 (RPS30C) | |  |  |  |  |  | | --- | --- | --- | --- | --- | |  |  |  |  |  | | RAFL04-18-L19 ,At5g56670  40S ribosomal protein S30 (RPS30C) | | | | | | |
| 5\_3270001\_3300000 | | |  |  | A | B | C | D | P | P' | N |
|  | Cluster:1-2 | |  |  | 2 | 172 | 2 | 4487 | 0.007905076 | 0.023715228 | 3 |
|  |  | RAFL02-07-H03 | At5g10430 / arabinogalactan-protein (AGP4) | |  |  |  |  |  | | --- | --- | --- | --- | --- | |  |  |  |  |  | | RAFL02-07-H03 ,At5g10430  arabinogalactan-protein (AGP4) identical to gi\_3883126\_gb\_AAC77826 | | | | | | |
|  |  | RAFL11-02-F17 | At5g10430 / arabinogalactan-protein (AGP4) | |  |  |  |  |  | | --- | --- | --- | --- | --- | |  |  |  |  |  | | RAFL11-02-F17 ,At5g10430  arabinogalactan-protein (AGP4) identical to gi\_3883126\_gb\_AAC77826 | | | | | | |
| 1\_19500001\_19530000 | | |  |  | A | B | C | D | P | P' | N |
|  | Cluster:8-1 | |  |  | 1 | 161 | 0 | 4501 | 0.034741584 | 0.034741584 | 1 |
|  |  | RAFL05-08-C18 | At1g53400 / expressed protein | |  |  |  |  |  | | --- | --- | --- | --- | --- | |  |  |  |  |  | | RAFL05-08-C18 ,At1g53400  expressed protein | | | | | | |
| 4\_13500001\_13530000 | | |  |  | A | B | C | D | P | P' | N |
|  | Cluster:7-2 | |  |  | 1 | 63 | 0 | 4599 | 0.013725069 | 0.013725069 | 1 |
|  |  | RAFL05-12-O13 | At4g29670 / thioredoxin family | |  |  |  |  |  | | --- | --- | --- | --- | --- | |  |  |  |  |  | | RAFL05-12-O13 ,At4g29670  thioredoxin family protein contains Pfam profile PF00085: Thioredoxin | | | | | | |
| 5\_25020001\_25050000 | | |  |  | A | B | C | D | P | P' | N |
|  | Cluster:3-2 | |  |  | 1 | 36 | 0 | 4626 | 0.007934806 | 0.007934806 | 1 |
|  |  | RAFL11-10-F22 | At5g63160 / expressed protein | |  |  |  |  |  | | --- | --- | --- | --- | --- | |  |  |  |  |  | | At5g63160 ,RAFL11-10-F22  speckle-type POZ protein-related contains Pfam PF00651 : BTB/POZ domain; contains Pfam PF02135 : TAZ zinc finger; similar to Speckle-type POZ protein (SP:O43791) [Homo sapiens] | | | | | | |
| 3\_18150001\_18180000 | | |  |  | A | B | C | D | P | P' | N |
|  | Cluster:10-2 | |  |  | 2 | 107 | 2 | 4552 | 0.0031505034 | 0.009451509 | 3 |
|  |  | RAFL08-12-E20 | At3g48990 / AMP-dependent synthetase and ligase family | |  |  |  |  |  | | --- | --- | --- | --- | --- | |  |  |  |  |  | | At3g48990 ,RAFL08-12-E20  AMP-dependent synthetase and ligase family protein similar to peroxisomal-coenzyme A synthetase (FAT2) [gi:586339] from Saccharomyces cerevisiae; contains Pfam AMP-binding enzyme domain PF00501; identical to cDNA; identical to cDNA adenosine monophosphate binding protein 3 AMPBP3 (AMPBP3)GI:20799714 | | | | | | |
|  |  | RAFL09-06-C22 | At3g48990 / AMP-dependent synthetase and ligase family | |  |  |  |  |  | | --- | --- | --- | --- | --- | |  |  |  |  |  | | At3g48990 ,RAFL09-06-C22  AMP-dependent synthetase and ligase family protein similar to peroxisomal-coenzyme A synthetase (FAT2) [gi:586339] from Saccharomyces cerevisiae; contains Pfam AMP-binding enzyme domain PF00501; identical to cDNA; identical to cDNA adenosine monophosphate binding protein 3 AMPBP3 (AMPBP3)GI:20799714 | | | | | | |
| 5\_7980001\_8010000 | | |  |  | A | B | C | D | P | P' | N |
|  | Cluster:2-1 | |  |  | 2 | 242 | 1 | 4418 | 0.00789916 | 0.01579832 | 2 |
|  |  | RAFL09-15-M15 | At5g23740 / 40S ribosomal protein S11 (RPS11C) | |  |  |  |  |  | | --- | --- | --- | --- | --- | |  |  |  |  |  | | RAFL09-15-M15 ,At5g23740  40S ribosomal protein S11 (RPS11C) | | | | | | |
|  |  | RAFL05-16-O05 | At5g23820 / expressed protein | |  |  |  |  |  | | --- | --- | --- | --- | --- | |  |  |  |  |  | | At5g23820 ,RAFL05-16-O05  MD-2-related lipid recognition domain-containing protein / ML domain-containing protein contains Pfam profile PF02221: ML domain | | | | | | |
| 5\_24300001\_24330000 | | |  |  | A | B | C | D | P | P' | N |
|  | Cluster:9-2 | |  |  | 1 | 66 | 1 | 4595 | 0.028533451 | 0.057066903 | 2 |
|  |  | RAFL02-07-L08 | At5g61160 / transferase family | |  |  |  |  |  | | --- | --- | --- | --- | --- | |  |  |  |  |  | | At5g61160 ,RAFL02-07-L08  transferase family protein similar to anthocyanin 5-aromatic acyltransferase from Gentiana triflora GI:4185599, malonyl CoA:anthocyanin 5-O-glucoside-6'''-O-malonyltransferase from Perilla frutescens GI:17980232, Salvia splendens GI:17980234; contains Pfam profile PF02458 transferase family | | | | | | |
| 4\_5430001\_5460000 | | |  |  | A | B | C | D | P | P' | N |
|  | Cluster:3-0 | |  |  | 2 | 231 | 1 | 4429 | 0.007213332 | 0.014426664 | 2 |
|  |  | RAFL08-18-K16 | At4g10480 / alpha NAC -related | |  |  |  |  |  | | --- | --- | --- | --- | --- | |  |  |  |  |  | | RAFL08-18-K16 ,At4g10480  nascent polypeptide associated complex alpha chain protein, putative / alpha-NAC, putative similar to alpha-NAC, non-muscle form [Mus musculus] GI:1666690; contains Pfam profiles PF01849: NAC domain, PF00627: UBA/TS-N domain | | | | | | |
|  |  | RAFL05-12-D01 | At4g10480 / alpha NAC -related | |  |  |  |  |  | | --- | --- | --- | --- | --- | |  |  |  |  |  | | RAFL05-12-D01 ,At4g10480  nascent polypeptide associated complex alpha chain protein, putative / alpha-NAC, putative similar to alpha-NAC, non-muscle form [Mus musculus] GI:1666690; contains Pfam profiles PF01849: NAC domain, PF00627: UBA/TS-N domain | | | | | | |
| 3\_15870001\_15900000 | | |  |  | A | B | C | D | P | P' | N |
|  | Cluster:6-1 | |  |  | 3 | 312 | 1 | 4347 | 0.0011607888 | 0.0023215776 | 2 |
|  |  | RAFL06-13-M23 | At3g44110 / DnaJ protein AtJ3 | |  |  |  |  |  | | --- | --- | --- | --- | --- | |  |  |  |  |  | | RAFL06-13-M23 ,At3g44110  DNAJ heat shock protein, putative (J3) identical to AtJ3 [Arabidopsis thaliana] GI:2641638, strong similarity to several plant DnaJ proteins from PGR; contains Pfam profiles PF00226 DnaJ domain, PF00684 DnaJ central domain (4 repeats), PF01556 DnaJ C terminal region | | | | | | |
|  |  | RAFL09-17-O15 | At3g44110 / DnaJ protein AtJ3 | |  |  |  |  |  | | --- | --- | --- | --- | --- | |  |  |  |  |  | | RAFL09-17-O15 ,At3g44110  DNAJ heat shock protein, putative (J3) identical to AtJ3 [Arabidopsis thaliana] GI:2641638, strong similarity to several plant DnaJ proteins from PGR; contains Pfam profiles PF00226 DnaJ domain, PF00684 DnaJ central domain (4 repeats), PF01556 DnaJ C terminal region | | | | | | |
|  |  | RAFL08-08-D21 | At3g44110 / DnaJ protein AtJ3 | |  |  |  |  |  | | --- | --- | --- | --- | --- | |  |  |  |  |  | | At3g44110 ,RAFL08-08-D21  DNAJ heat shock protein, putative (J3) identical to AtJ3 [Arabidopsis thaliana] GI:2641638, strong similarity to several plant DnaJ proteins from PGR; contains Pfam profiles PF00226 DnaJ domain, PF00684 DnaJ central domain (4 repeats), PF01556 DnaJ C terminal region | | | | | | |
| 1\_4200001\_4230000 | | |  |  | A | B | C | D | P | P' | N |
|  | Cluster:4-1 | |  |  | 2 | 308 | 0 | 4353 | 0.0044063856 | 0.0044063856 | 1 |
|  |  | RAFL06-10-N03 | At1g12410 / ATP-dependent Clp protease proteolytic subunit (ClpR2) | |  |  |  |  |  | | --- | --- | --- | --- | --- | |  |  |  |  |  | | At1g12410 ,RAFL06-10-N03  ATP-dependent Clp protease proteolytic subunit (ClpP2) identical to nClpP2 GI:5360589 from [Arabidopsis thaliana] | | | | | | |
|  |  | RAFL05-10-E01 | At1g12370 / type II CPD photolyase PHR1 (PHR1) | |  |  |  |  |  | | --- | --- | --- | --- | --- | |  |  |  |  |  | | At1g12370 ,RAFL05-10-E01  type II CPD photolyase PHR1 (PHR1) nearly identical to type II CPD photolyase PHR1 [Arabidopsis thaliana] GI:2984707; similar to class II DNA photolyase (GI:5081541) [Chlamydomonas reinhardtii]; supporting cDNA gi|2984706|gb|AF053365.1|AF053365 | | | | | | |
| 4\_4710001\_4740000 | | |  |  | A | B | C | D | P | P' | N |
|  | Cluster:7-1 | |  |  | 2 | 263 | 1 | 4397 | 0.009291402 | 0.018582804 | 2 |
|  |  | RAFL09-17-I12 | At4g08960 / phosphotyrosyl phosphatase activator protein -related | |  |  |  |  |  | | --- | --- | --- | --- | --- | |  |  |  |  |  | | RAFL09-17-I12 ,At4g08960  phosphotyrosyl phosphatase activator (PTPA) family protein similar to Protein phosphatase 2A, regulatory subunit B' (PP2A, subunit B', PR53 isoform) (Phosphotyrosyl phosphatase activator) (PTPA) (Swiss-Prot:Q28717) [Oryctolagus cuniculus] | | | | | | |
|  |  | RAFL06-11-F02 | At4g08980 / F-box protein family, AtFBW2 | |  |  |  |  |  | | --- | --- | --- | --- | --- | |  |  |  |  |  | | At4g08980 ,RAFL06-11-F02  F-box family protein (FBW2) contains similarity to N7 protein GI:3273101 from [Medicago truncatula] | | | | | | |
| 1\_24870001\_24900000 | | |  |  | A | B | C | D | P | P' | N |
|  | Cluster:10-2 | |  |  | 2 | 107 | 1 | 4553 | 0.0015996902 | 0.0031993804 | 2 |
|  |  | RAFL08-11-D22 | At1g67360 / stress related protein -related | |  |  |  |  |  | | --- | --- | --- | --- | --- | |  |  |  |  |  | | At1g67360 ,RAFL08-11-D22  rubber elongation factor (REF) family protein contains Pfam profile: PF05755 rubber elongation factor protein (REF) | | | | | | |
|  |  | RAFL11-12-J24 | At1g67360 / stress related protein -related | |  |  |  |  |  | | --- | --- | --- | --- | --- | |  |  |  |  |  | | RAFL11-12-J24 ,At1g67360  rubber elongation factor (REF) family protein contains Pfam profile: PF05755 rubber elongation factor protein (REF) | | | | | | |
| 1\_600001\_630000 | | |  |  | A | B | C | D | P | P' | N |
|  | Cluster:10-2 | |  |  | 1 | 108 | 1 | 4553 | 0.0462095 | 0.092419 | 2 |
|  |  | RAFL04-16-I05 | At1g02816 / expressed protein | |  |  |  |  |  | | --- | --- | --- | --- | --- | |  |  |  |  |  | | RAFL04-16-I05 ,At1g02816  expressed protein contains Pfam profile PF04398: Protein of unknown function, DUF538 | | | | | | |
| 3\_2310001\_2340000 | | |  |  | A | B | C | D | P | P' | N |
|  | Cluster:5-0 | |  |  | 1 | 76 | 1 | 4585 | 0.032756753 | 0.06551351 | 2 |
|  |  | RAFL04-17-J04 | At3g07310 / expressed protein | |  |  |  |  |  | | --- | --- | --- | --- | --- | |  |  |  |  |  | | RAFL04-17-J04 ,At3g07310  expressed protein | | | | | | |
| 5\_6390001\_6420000 | | |  |  | A | B | C | D | P | P' | N |
|  | Cluster:2-0 | |  |  | 2 | 148 | 1 | 4512 | 0.003019041 | 0.006038082 | 2 |
|  |  | RAFL11-11-J05 | At5g19120 / conglutin gamma - like protein | |  |  |  |  |  | | --- | --- | --- | --- | --- | |  |  |  |  |  | | RAFL11-11-J05 ,At5g19120  expressed protein low similarity to extracellular dermal glycoprotein EDGP precursor [Daucus carota] GI:285741, SP|P13917 Basic 7S globulin precursor {Glycine max} | | | | | | |
|  |  | RAFL03-09-J09 | At5g19120 / conglutin gamma - like protein | |  |  |  |  |  | | --- | --- | --- | --- | --- | |  |  |  |  |  | | RAFL03-09-J09 ,At5g19120  expressed protein low similarity to extracellular dermal glycoprotein EDGP precursor [Daucus carota] GI:285741, SP|P13917 Basic 7S globulin precursor {Glycine max} | | | | | | |
| 5\_720001\_750000 | | |  |  | A | B | C | D | P | P' | N |
|  | Cluster:3-1 | |  |  | 1 | 215 | 0 | 4447 | 0.04632211 | 0.04632211 | 1 |
|  |  | RAFL04-16-B20 | At5g03120 / expressed protein | |  |  |  |  |  | | --- | --- | --- | --- | --- | |  |  |  |  |  | | At5g03120 ,RAFL04-16-B20  expressed protein | | | | | | |
| 2\_18840001\_18870000 | | |  |  | A | B | C | D | P | P' | N |
|  | Cluster:4-1 | |  |  | 2 | 308 | 3 | 4350 | 0.038523052 | 0.15409221 | 4 |
|  |  | RAFL11-06-I12 | At2g45950 / E3 ubiquitin ligase SCF complex subunit SKP1/ASK1-related | |  |  |  |  |  | | --- | --- | --- | --- | --- | |  |  |  |  |  | | At2g45950 ,RAFL11-06-I12  SKP1 family protein similar to glycoprotein FP21 SP:P52285 from [Dictyostelium discoideum]; contains Pfam profile PF01466: Skp1 family, dimerisation domain | | | | | | |
|  |  | RAFL05-19-H24 | At2g45990 / expressed protein | |  |  |  |  |  | | --- | --- | --- | --- | --- | |  |  |  |  |  | | RAFL05-19-H24 ,At2g45990  expressed protein | | | | | | |
|  | Cluster:3-2 | |  |  | 1 | 36 | 4 | 4622 | 0.039065886 | 0.15626355 | 4 |
|  |  | RAFL06-15-B17 | At2g45960 / plasma membrane intrinsic protein 1B | |  |  |  |  |  | | --- | --- | --- | --- | --- | |  |  |  |  |  | | RAFL06-15-B17 ,At2g45960  plasma membrane intrinsic protein 1B (PIP1B) / aquaporin PIP1.2 (PIP1.2) / transmembrane protein A (TMPA) identical to plasma membrane intrinsic protein 1B SP:Q06611 from [Arabidopsis thaliana] | | | | | | |
| 3\_20100001\_20400000 | | |  |  | A | B | C | D | P | P' | N |
|  | Cluster:1-1 | |  |  | 2 | 103 | 13 | 4545 | 0.04357712 | 0.52292544 | 12 |
|  |  | RAFL09-09-I17 | At3g54920 / polysaccharide lyase family 1 (pectate lyase) | |  |  |  |  |  | | --- | --- | --- | --- | --- | |  |  |  |  |  | | At3g54920 ,RAFL09-09-I17  pectate lyase, putative / powdery mildew susceptibility protein (PMR6) identical to powdery mildew susceptibility protein [Arabidopsis thaliana] GI:22506901; similar to pectate lyase 2 GP:6606534 from [Musa acuminata] | | | | | | |
|  |  | RAFL05-16-C09 | At3g54400 / nucleoid DNA-binding - like protein | |  |  |  |  |  | | --- | --- | --- | --- | --- | |  |  |  |  |  | | RAFL05-16-C09 ,At3g54400  aspartyl protease family protein contains Pfam profile: PF00026 eukaryotic aspartyl protease | | | | | | |
| 2\_15300001\_15600000 | | |  |  | A | B | C | D | P | P' | N |
|  | Cluster:2-0 | |  |  | 3 | 147 | 14 | 4499 | 0.015928537 | 0.20707098 | 13 |
|  |  | RAFL05-07-F16 | At2g37220 / 29 kDa ribonucleoprotein, chloroplast (RNA-binding protein cp29), putative | |  |  |  |  |  | | --- | --- | --- | --- | --- | |  |  |  |  |  | | RAFL05-07-F16 ,At2g37220  29 kDa ribonucleoprotein, chloroplast, putative / RNA-binding protein cp29, putative similar to SP|Q43349 29 kDa ribonucleoprotein, chloroplast precursor (RNA-binding protein cp29) {Arabidopsis thaliana} | | | | | | |
|  |  | RAFL06-08-J02 | At2g37130 / peroxidase, putative (ATP2a) | |  |  |  |  |  | | --- | --- | --- | --- | --- | |  |  |  |  |  | | RAFL06-08-J02 ,At2g37130  peroxidase 21 (PER21) (P21) (PRXR5) identical to SP|Q42580 Peroxidase 21 precursor (EC 1.11.1.7) (Atperox P21) (PRXR5) (ATP2a/ATP2b) {Arabidopsis thaliana} | | | | | | |
|  |  | RAFL08-10-G10 | At2g37220 / 29 kDa ribonucleoprotein, chloroplast (RNA-binding protein cp29), putative | |  |  |  |  |  | | --- | --- | --- | --- | --- | |  |  |  |  |  | | RAFL08-10-G10 ,At2g37220  29 kDa ribonucleoprotein, chloroplast, putative / RNA-binding protein cp29, putative similar to SP|Q43349 29 kDa ribonucleoprotein, chloroplast precursor (RNA-binding protein cp29) {Arabidopsis thaliana} | | | | | | |
| 1\_3900001\_3930000 | | |  |  | A | B | C | D | P | P' | N |
|  | Cluster:6-2 | |  |  | 1 | 175 | 0 | 4487 | 0.03774394 | 0.03774394 | 1 |
|  |  | RAFL05-03-G09 | At1g11630 / pentatricopeptide (PPR) repeat-containing protein | |  |  |  |  |  | | --- | --- | --- | --- | --- | |  |  |  |  |  | | At1g11630 ,RAFL05-03-G09  pentatricopeptide (PPR) repeat-containing protein contains Pfam profile PF01535: PPR repeat | | | | | | |
| 5\_6000001\_9000000 | | |  |  | A | B | C | D | P | P' | N |
|  | Cluster:5-1 | |  |  | 16 | 266 | 125 | 4256 | 0.017493017 | 0.48980445 | 28 |
|  |  | RAFL11-03-N11 | At5g20020 / GTP-binding nuclear protein (RAN-2) | |  |  |  |  |  | | --- | --- | --- | --- | --- | |  |  |  |  |  | | RAFL11-03-N11 ,At5g20020  Ras-related GTP-binding nuclear protein (RAN-2) identical to GTP-binding nuclear protein RAN-2 SP:P41917 from [Arabidopsis thaliana] | | | | | | |
|  |  | RAFL07-17-O15 | At5g19180 / RUB-activating enzyme ECR1 | |  |  |  |  |  | | --- | --- | --- | --- | --- | |  |  |  |  |  | | At5g19180 ,RAFL07-17-O15  ubiquitin activating enzyme, putative (ECR1) identical to putative ubiquitin activating enzyme E1 [Arabidopsis thaliana] GI:2952433; similar to NEDD8 activating enzyme [Mus musculus] GI:17061821 | | | | | | |
|  |  | RAFL07-17-N07 | At5g23890 / expressed protein | |  |  |  |  |  | | --- | --- | --- | --- | --- | |  |  |  |  |  | | At5g23890 ,RAFL07-17-N07  expressed protein weak similarity to SP|P12957 Caldesmon (CDM) {Gallus gallus} | | | | | | |
|  |  | RAFL08-12-D06 | At5g23660 / nodulin MtN3 family protein | |  |  |  |  |  | | --- | --- | --- | --- | --- | |  |  |  |  |  | | At5g23660 ,RAFL08-12-D06  nodulin MtN3 family protein similar to MtN3 GI:1619602 (root nodule development) from [Medicago truncatula] | | | | | | |
|  |  | RAFL08-11-O19 | At5g23670 / serine C-palmitoyltransferase, putative | |  |  |  |  |  | | --- | --- | --- | --- | --- | |  |  |  |  |  | | At5g23670 ,RAFL08-11-O19  serine C-palmitoyltransferase (LCB2) identical to serine palmitoyltransferase [Arabidopsis thaliana] GI:9309380; similar to serine palmitoyltransferase from Solanum tuberosum [GI:4995890], Homo sapiens [SP|O15270], Mus musculus [SP|P97363]; contains Pfam profile PF00155: aminotransferase, classes I and II | | | | | | |
|  |  | RAFL09-16-N12 | At5g19820 / expressed protein | |  |  |  |  |  | | --- | --- | --- | --- | --- | |  |  |  |  |  | | RAFL09-16-N12 ,At5g19820  PBS lyase HEAT-like repeat-containing protein contains Pfam profile: PF03130 PBS lyase HEAT-like repeat | | | | | | |
|  |  | RAFL05-15-P17 | At5g19150 / expressed protein | |  |  |  |  |  | | --- | --- | --- | --- | --- | |  |  |  |  |  | | RAFL05-15-P17 ,At5g19150  carbohydrate kinase family contains Pfam profile PF01256: Carbohydrate kinase | | | | | | |
|  |  | RAFL04-17-B06 | At5g22060 / DnaJ protein, putative | |  |  |  |  |  | | --- | --- | --- | --- | --- | |  |  |  |  |  | | RAFL04-17-B06 ,At5g22060  DNAJ heat shock protein, putative strong similarity to SP|O60884 DnaJ homolog subfamily A member 2 (Dnj3) Homo sapiens, several plant DnaJ proteins from PGR; contains Pfam profiles PF00226 DnaJ domain, PF00684 DnaJ central domain (4 repeats), PF01556 DnaJ C terminal region | | | | | | |
|  |  | RAFL06-07-D07 | At5g20020 / GTP-binding nuclear protein (RAN-2) | |  |  |  |  |  | | --- | --- | --- | --- | --- | |  |  |  |  |  | | RAFL06-07-D07 ,At5g20020  Ras-related GTP-binding nuclear protein (RAN-2) identical to GTP-binding nuclear protein RAN-2 SP:P41917 from [Arabidopsis thaliana] | | | | | | |
|  |  | RAFL07-12-C22 | At5g19130 / GPAA1 - like protein | |  |  |  |  |  | | --- | --- | --- | --- | --- | |  |  |  |  |  | | At5g19130 ,RAFL07-12-C22  GPI transamidase component family protein / Gaa1-like family protein contains Pfam profile: PF04114 Gaa1-like, GPI transamidase component | | | | | | |
|  |  | RAFL07-08-I10 | At5g22460 / esterase/lipase/thioesterase family | |  |  |  |  |  | | --- | --- | --- | --- | --- | |  |  |  |  |  | | At5g22460 ,RAFL07-08-I10  esterase/lipase/thioesterase family protein low similarity to 2-hydroxy-6-oxo-6-phenylhexa-2,4-dienoate hydrolase [Rhodococcus sp. RHA1] GI:8978311, SP|Q02104 Lipase 1 precursor (EC 3.1.1.3) (Triacylglycerol lipase) {Psychrobacter immobilis}; contains Interpro entry IPR000379 | | | | | | |
|  |  | RAFL04-20-B01 | At5g21010 / expressed protein | |  |  |  |  |  | | --- | --- | --- | --- | --- | |  |  |  |  |  | | At5g21010 ,RAFL04-20-B01  speckle-type POZ protein-related contains Pfam PF00651 : BTB/POZ domain; contains Pfam PF00917: MATH domain; similar to Speckle-type POZ protein (SP:O43791) [Homo sapiens] | | | | | | |
|  |  | RAFL04-14-K03 | At5g18590 / RanGAP1 interacting protein | |  |  |  |  |  | | --- | --- | --- | --- | --- | |  |  |  |  |  | | At5g18590 ,RAFL04-14-K03  kelch repeat-containing protein identical to RanGAP1 interacting protein (GI:21950739) [Arabidopsis thaliana]; similar to Tip elongation aberrant protein 1 (Cell polarity protein tea1) (SP:P87061) [Schizosaccharomyces pombe]; contains Pfam PF01344: Kelch motif (5 repeats) | | | | | | |
|  |  | RAFL05-08-E17 | At5g22330 / Ruv DNA-helicase-related protein | |  |  |  |  |  | | --- | --- | --- | --- | --- | |  |  |  |  |  | | At5g22330 ,RAFL05-08-E17  TATA box-binding protein-interacting protein-related similar to TATA box-binding protein-interacting protein SP:O35753 from [ Mus musculus] | | | | | | |
|  |  | RAFL05-15-M17 | At5g23390 / expressed protein | |  |  |  |  |  | | --- | --- | --- | --- | --- | |  |  |  |  |  | | At5g23390 ,RAFL05-15-M17  expressed protein contains Pfam profile: PF04842 plant protein of unknown function (DUF639) | | | | | | |
|  |  | RAFL04-13-H19 | At5g18620 / DNA-dependent ATPase, putative | |  |  |  |  |  | | --- | --- | --- | --- | --- | |  |  |  |  |  | | RAFL04-13-H19 ,At5g18620  DNA-dependent ATPase, putative similar to DNA-dependent ATPase SNF2H [Mus musculus] GI:14028669; contains Pfam profiles PF00271: Helicase conserved C-terminal domain, PF00176: SNF2 family N-terminal domain, PF00249: Myb-like DNA-binding domain | | | | | | |
| 2\_14790001\_14820000 | | |  |  | A | B | C | D | P | P' | N |
|  | Cluster:1-2 | |  |  | 1 | 173 | 0 | 4489 | 0.037315033 | 0.037315033 | 1 |
|  |  | RAFL09-12-E13 | At2g35260 / expressed protein | |  |  |  |  |  | | --- | --- | --- | --- | --- | |  |  |  |  |  | | At2g35260 ,RAFL09-12-E13  expressed protein | | | | | | |
| 5\_26160001\_26190000 | | |  |  | A | B | C | D | P | P' | N |
|  | Cluster:0-2 | |  |  | 1 | 78 | 1 | 4583 | 0.03360031 | 0.06720062 | 2 |
|  |  | RAFL06-08-B20 | At5g66190 / ferredoxin--NADP(+) reductase (adrenodoxin reductase), putative | |  |  |  |  |  | | --- | --- | --- | --- | --- | |  |  |  |  |  | | At5g66190 ,RAFL06-08-B20  ferredoxin--NADP(+) reductase, putative / adrenodoxin reductase, putative strong similarity to Ferredoxin--NADP reductase, chloroplast precursor (EC 1.18.1.2) (FNR) from {Pisum sativum} SP|P10933, {Mesembryanthemum crystallinum} SP|P41343, {Spinacia oleracea} SP|P00455; identical to cDNA ferredoxin-NADP+ reductase precursor (petH) GI:5730138 | | | | | | |
|  | Cluster:8-0 | |  |  | 1 | 108 | 1 | 4553 | 0.0462095 | 0.092419 | 2 |
|  |  | RAFL02-09-F24 | At5g66170 / senescence-associated protein | |  |  |  |  |  | | --- | --- | --- | --- | --- | |  |  |  |  |  | | RAFL02-09-F24 ,At5g66170  senescence-associated family protein contains similarity to ketoconazole resistant protein GI:928938 and senescence-associated protein GI:1046268 from [Arabidopsis thaliana] | | | | | | |
| 2\_16980001\_17010000 | | |  |  | A | B | C | D | P | P' | N |
|  | Cluster:5-0 | |  |  | 1 | 76 | 0 | 4586 | 0.016512975 | 0.016512975 | 1 |
|  |  | RAFL05-18-L20 | At2g40890 / cytochrome P450 98A3 | |  |  |  |  |  | | --- | --- | --- | --- | --- | |  |  |  |  |  | | RAFL05-18-L20 ,At2g40890  cytochrome P450 98A3, putative (CYP98A3) identical to Cytochrome P450 98A3 (SP|O22203) [Arabidopsis thaliana]; similar to gi:17978651 from Pinus taeda | | | | | | |
| 2\_1170001\_1200000 | | |  |  | A | B | C | D | P | P' | N |
|  | Cluster:6-0 | |  |  | 1 | 139 | 0 | 4523 | 0.03002359 | 0.03002359 | 1 |
|  |  | RAFL08-09-A06 | At2g03890 / phosphatidylinositol 3- and 4-kinase family | |  |  |  |  |  | | --- | --- | --- | --- | --- | |  |  |  |  |  | | At2g03890 ,RAFL08-09-A06  phosphatidylinositol 3- and 4-kinase family protein low similarity to phosphatidylinositol 4-kinase type-II beta [Homo sapiens] GI:20159767; contains Pfam profile PF00454: Phosphatidylinositol 3- and 4-kinase | | | | | | |
| 5\_14130001\_14160000 | | |  |  | A | B | C | D | P | P' | N |
|  | Cluster:1-0 | |  |  | 1 | 148 | 0 | 4514 | 0.031953678 | 0.031953678 | 1 |
|  |  | RAFL06-14-K21 | At5g36700 / phosphoglycolate phosphatase, putative | |  |  |  |  |  | | --- | --- | --- | --- | --- | |  |  |  |  |  | | At5g36700 ,RAFL06-14-K21  phosphoglycolate phosphatase, putative similar to phosphoglycolate phosphatase precursor [Chlamydomonas reinhardtii] GI:15982558; contains InterPro accession IPR005834: Haloacid dehalogenase-like hydrolase | | | | | | |
| 3\_5760001\_5790000 | | |  |  | A | B | C | D | P | P' | N |
|  | Cluster:5-0 | |  |  | 1 | 76 | 1 | 4585 | 0.032756753 | 0.06551351 | 2 |
|  |  | RAFL05-17-C14 | At3g16910 / AMP-dependent synthetase and ligase family | |  |  |  |  |  | | --- | --- | --- | --- | --- | |  |  |  |  |  | | RAFL05-17-C14 ,At3g16910  AMP-dependent synthetase and ligase family protein similar to AMP-binding protein GI:1903034 from [Brassica napus]; contains Pfam AMP-binding domain PF00501; identical to cDNA adenosine monophosphate binding protein 7 AMPBP7 (AMPBP7) GI:20799722 | | | | | | |
| 1\_27150001\_27180000 | | |  |  | A | B | C | D | P | P' | N |
|  | Cluster:3-1 | |  |  | 1 | 215 | 0 | 4447 | 0.04632211 | 0.04632211 | 1 |
|  |  | RAFL07-09-L10 | At1g73180 / expressed protein | |  |  |  |  |  | | --- | --- | --- | --- | --- | |  |  |  |  |  | | RAFL07-09-L10 ,At1g73180  eukaryotic translation initiation factor-related similar to eukaryotic translation initiation factor 2A (GI:21956484) [Homo sapiens]; similar to Eukaryotic translation initiation factor 3 subunit 9 (eIF-3 eta) (eIF3 p116) (eIF3 p110) (eIF3b) (Swiss-Prot:P55884) [Homo sapiens] | | | | | | |
| 1\_20010001\_20040000 | | |  |  | A | B | C | D | P | P' | N |
|  | Cluster:0-2 | |  |  | 3 | 76 | 1 | 4583 | 1.850166E-5 | 3.700332E-5 | 2 |
|  |  | RAFL05-19-G04 | At1g54780 / thylakoid lumen 18.3 kDa protein | |  |  |  |  |  | | --- | --- | --- | --- | --- | |  |  |  |  |  | | RAFL05-19-G04 ,At1g54780  thylakoid lumen 18.3 kDa protein SP:Q9ZVL6 | | | | | | |
|  |  | RAFL05-01-I05 | At1g54780 / thylakoid lumen 18.3 kDa protein | |  |  |  |  |  | | --- | --- | --- | --- | --- | |  |  |  |  |  | | RAFL05-01-I05 ,At1g54780  thylakoid lumen 18.3 kDa protein SP:Q9ZVL6 | | | | | | |
|  |  | RAFL07-14-F21 | At1g54780 / thylakoid lumen 18.3 kDa protein | |  |  |  |  |  | | --- | --- | --- | --- | --- | |  |  |  |  |  | | RAFL07-14-F21 ,At1g54780  thylakoid lumen 18.3 kDa protein SP:Q9ZVL6 | | | | | | |
| 4\_1\_300000 | | |  |  | A | B | C | D | P | P' | N |
|  | Cluster:8-2 | |  |  | 2 | 60 | 6 | 4595 | 0.0046263156 | 0.03238421 | 7 |
|  |  | RAFL06-09-F19 | At4g00430 / transmembrane protein (MIP family) | |  |  |  |  |  | | --- | --- | --- | --- | --- | |  |  |  |  |  | | At4g00430 ,RAFL06-09-F19  plasma membrane intrinsic protein, putative identical to transmembrane protein GI:535780 from [Arabidopsis thaliana]; very strong similarity to SP|Q08733 Plasma membrane intrinsic protein 1C (Transmembrane protein B) (TMP-B) {Arabidopsis thaliana}; contains Pfam profile PF00230: Major intrinsic protein; | | | | | | |
|  |  | RAFL05-16-H03 | At4g00360 / cytochrome p450, putative | |  |  |  |  |  | | --- | --- | --- | --- | --- | |  |  |  |  |  | | At4g00360 ,RAFL05-16-H03  cytochrome P450, putative | | | | | | |
| 3\_9000001\_12000000 | | |  |  | A | B | C | D | P | P' | N |
|  | Cluster:5-0 | |  |  | 5 | 72 | 74 | 4512 | 0.0093790805 | 0.21571885 | 23 |
|  |  | RAFL05-10-E23 | At3g27820 / monodehydroascorbate reductase, putative | |  |  |  |  |  | | --- | --- | --- | --- | --- | |  |  |  |  |  | | At3g27820 ,RAFL05-10-E23  monodehydroascorbate reductase, putative similar to cytosolic monodehydroascorbate reductase GB:BAA77214 [Oryza sativa] | | | | | | |
|  |  | RAFL07-12-I09 | At3g29240 / chloroplast lumen common protein family | |  |  |  |  |  | | --- | --- | --- | --- | --- | |  |  |  |  |  | | At3g29240 ,RAFL07-12-I09  expressed protein similar to At1g33780 [Arabidopsis thaliana]; contains Pfam profile PF02622: Uncharacterized ACR, COG1678 | | | | | | |
|  |  | RAFL05-18-K22 | At3g26450 / major latex protein (MLP)-related | |  |  |  |  |  | | --- | --- | --- | --- | --- | |  |  |  |  |  | | RAFL05-18-K22 ,At3g26450  major latex protein-related / MLP-related low similarity to major latex protein {Papaver somniferum}[GI:294060] ; contains Pfam profile PF00407: Pathogenesis-related protein Bet v I family | | | | | | |
|  |  | RAFL09-16-M01 | At3g25830 / myrcene/ocimene synthase, putative | |  |  |  |  |  | | --- | --- | --- | --- | --- | |  |  |  |  |  | | At3g25830 ,RAFL09-16-M01  myrcene/ocimene synthase, putative similar to myrcene/ocimene synthase [Arabidopsis thaliana] GI:9957293; contains Pfam profiles PF03936: Terpene synthase family, metal binding domain, PF01397: Terpene synthase, N-terminal domain | | | | | | |
|  |  | RAFL08-19-G11 | At3g28270 / At14a | |  |  |  |  |  | | --- | --- | --- | --- | --- | |  |  |  |  |  | | At3g28270 ,RAFL08-19-G11  expressed protein similar to At14a protein (GI:11994571 and GI:11994573) [Arabidopsis thaliana] | | | | | | |
|  | Cluster:4-1 | |  |  | 11 | 299 | 68 | 4285 | 0.018645054 | 0.42883623 | 23 |
|  |  | RAFL07-17-D11 | At3g26300 / cytochrome P450 family | |  |  |  |  |  | | --- | --- | --- | --- | --- | |  |  |  |  |  | | At3g26300 ,RAFL07-17-D11  cytochrome P450 family protein contains Pfam profile: PF00067 cytochrome P450 | | | | | | |
|  |  | RAFL07-09-E19 | At3g27700 / RNA recognition motif (RRM) - containing protein | |  |  |  |  |  | | --- | --- | --- | --- | --- | |  |  |  |  |  | | RAFL07-09-E19 ,At3g27700  RNA recognition motif (RRM)-containing protein contains Pfam profile: PF00076 RNA recognition motif | | | | | | |
|  |  | RAFL07-08-F20 | At3g24800 / E3 ubiquitin ligase, PRT1 | |  |  |  |  |  | | --- | --- | --- | --- | --- | |  |  |  |  |  | | RAFL07-08-F20 ,At3g24800  PRT1 protein (PRT1) E3, N-end rule ubiquitin ligase, contains two RING finger domain; identical to PRT1 [Arabidopsis thaliana] GI:3319884 | | | | | | |
|  |  | RAFL05-08-N12 | At3g27080 / TOM20 -related | |  |  |  |  |  | | --- | --- | --- | --- | --- | |  |  |  |  |  | | RAFL05-08-N12 ,At3g27080  mitochondrial import receptor subunit TOM20-3 / translocase of outer membrane 20 kDa subunit 3 (TOM20-3) identical to mitochondrial import receptor subunit TOM20-3 SP:P82874 from [Arabidopsis thaliana] | | | | | | |
|  |  | RAFL05-13-E16 | At3g26420 / glycine-rich RNA-binding protein | |  |  |  |  |  | | --- | --- | --- | --- | --- | |  |  |  |  |  | | At3g26420 ,RAFL05-13-E16  glycine-rich RNA-binding protein similar to RNA-binding protein (RZ-1) GB:BAA12064 [Nicotiana sylvestris]; contains Pfam profile: PF00076 RNA recognition motif. (a.k.a. RRM, RBD, or RNP domain) | | | | | | |
|  |  | RAFL06-07-A02 | At3g27240 / cytochrome c -related | |  |  |  |  |  | | --- | --- | --- | --- | --- | |  |  |  |  |  | | RAFL06-07-A02 ,At3g27240  cytochrome c1, putative cytochrome c1, heme protein, mitochondrial precursor (Clone PC13III) [Solanum tuberosum] SWISS-PROT:P25076 | | | | | | |
|  |  | RAFL03-09-J10 | At3g27925 / DegP protease | |  |  |  |  |  | | --- | --- | --- | --- | --- | |  |  |  |  |  | | RAFL03-09-J10 ,At3g27925  DegP protease, putative SP:022609; almost identical to DegP protease precursor GB:AF028842 from [Arabidopsis thaliana] (J. Biol. Chem. 273 (12), 7094-7098 (1998)) | | | | | | |
|  |  | RAFL06-11-N20 | At3g25220 / immunophilin / FKBP-type peptidyl-prolyl cis-trans isomerase (FKBP15-1) | |  |  |  |  |  | | --- | --- | --- | --- | --- | |  |  |  |  |  | | At3g25220 ,RAFL06-11-N20  FK506-binding protein 2-1 (FKBP15-1) / immunophilin / peptidyl-prolyl cis-trans isomerase / rotamase identical to SP|Q38935 FK506-binding protein 2-1 precursor (EC 5.2.1.8) (Peptidyl-prolyl cis- trans isomerase) (PPiase) (Rotamase) (15 kDa FKBP) (FKBP-15-1) {Arabidopsis thaliana}, immunophilin (FKBP15-1) GB:U52046 [Arabidopsis thaliana] (Proc. Natl. Acad. Sci. U.S.A. 93 (14), 6964-6969 (1996)) | | | | | | |
|  |  | RAFL09-09-A19 | At3g27090 / gda-1 -related | |  |  |  |  |  | | --- | --- | --- | --- | --- | |  |  |  |  |  | | RAFL09-09-A19 ,At3g27090  expressed protein similar to gda-1 [Pisum sativum] GI:2765418 | | | | | | |
|  |  | RAFL05-03-N24 | At3g28710 / adenosine triphosphatase -related | |  |  |  |  |  | | --- | --- | --- | --- | --- | |  |  |  |  |  | | At3g28710 ,RAFL05-03-N24  H+-transporting two-sector ATPase, putative similar to SP|P54641 Vacuolar ATP synthase subunit d (EC 3.6.3.14) (Vacuolar proton pump d subunit) (V-ATPase 41 KDa accessory protein) {Dictyostelium discoideum}; contains Pfam profile PF01992: ATP synthase (C/AC39) subunit | | | | | | |
|  |  | RAFL06-10-D09 | At3g25230 / peptidylprolyl isomerase (ROF1) | |  |  |  |  |  | | --- | --- | --- | --- | --- | |  |  |  |  |  | | RAFL06-10-D09 ,At3g25230  peptidyl-prolyl cis-trans isomerase / FK506-binding protein (ROF1) identical to rotamase FKBP (ROF1) GB:U49453 [Arabidopsis thaliana] (Mol. Gen. Genet. 252 (5), 510-517 (1996)) | | | | | | |
|  | Cluster:0-1 | |  |  | 5 | 101 | 74 | 4483 | 0.03297345 | 0.75838935 | 23 |
|  |  | RAFL05-21-D22 | At3g27830 / 50S ribosomal protein L12-1, chloroplast precursor (CL12-A) | |  |  |  |  |  | | --- | --- | --- | --- | --- | |  |  |  |  |  | | At3g27830 ,RAFL05-21-D22  50S ribosomal protein L12-1, chloroplast (CL12-A) identical to ribosomal protein L12 GB:X68046 [Arabidopsis thaliana] (J. Biol. Chem. 269 (10), 7330-7336 (1994)) | | | | | | |
|  |  | RAFL05-01-B21 | At3g27850 / 50S ribosomal protein L12-3, chloroplast precursor (CL12-C) | |  |  |  |  |  | | --- | --- | --- | --- | --- | |  |  |  |  |  | | RAFL05-01-B21 ,At3g27850  50S ribosomal protein L12-3, chloroplast (CL12-C) identical to ribosomal protein L12 GB:X68046 [Arabidopsis thaliana] (J. Biol. Chem. 269 (10), 7330-7336 (1994)) | | | | | | |
|  |  | RAFL11-10-L16 | At3g27830 / 50S ribosomal protein L12-1, chloroplast precursor (CL12-A) | |  |  |  |  |  | | --- | --- | --- | --- | --- | |  |  |  |  |  | | At3g27830 ,RAFL11-10-L16  50S ribosomal protein L12-1, chloroplast (CL12-A) identical to ribosomal protein L12 GB:X68046 [Arabidopsis thaliana] (J. Biol. Chem. 269 (10), 7330-7336 (1994)) | | | | | | |
|  |  | RAFL09-14-L24 | At3g28220 / expressed protein | |  |  |  |  |  | | --- | --- | --- | --- | --- | |  |  |  |  |  | | At3g28220 ,RAFL09-14-L24  meprin and TRAF homology domain-containing protein / MATH domain-containing protein similar to ubiquitin-specific protease 12 [Arabidopsis thaliana] GI:11993471; contains Pfam profile PF00917: MATH domain | | | | | | |
|  |  | RAFL08-09-L12 | At3g26060 / peroxiredoxin -related | |  |  |  |  |  | | --- | --- | --- | --- | --- | |  |  |  |  |  | | RAFL08-09-L12 ,At3g26060  peroxiredoxin Q, putative similar to peroxiredoxin Q [Sedum lineare] GI:6899842; contains Pfam profile: PF00578 AhpC/TSA (alkyl hydroperoxide reductase and thiol-specific antioxidant) family | | | | | | |
| 4\_15300001\_15600000 | | |  |  | A | B | C | D | P | P' | N |
|  | Cluster:9-2 | |  |  | 2 | 65 | 21 | 4575 | 0.04238358 | 0.7205209 | 17 |
|  |  | RAFL08-11-N01 | At4g34710 / arginine decarboxylase SPE2 | |  |  |  |  |  | | --- | --- | --- | --- | --- | |  |  |  |  |  | | At4g34710 ,RAFL08-11-N01  arginine decarboxylase 2 (SPE2) identical to SP|O23141 Arginine decarboxylase 2 (EC 4.1.1.19) (ARGDC 2) (ADC 2) (ADC-N) {Arabidopsis thaliana} | | | | | | |
|  |  | RAFL09-13-D07 | At4g34710 / arginine decarboxylase SPE2 | |  |  |  |  |  | | --- | --- | --- | --- | --- | |  |  |  |  |  | | RAFL09-13-D07 ,At4g34710  arginine decarboxylase 2 (SPE2) identical to SP|O23141 Arginine decarboxylase 2 (EC 4.1.1.19) (ARGDC 2) (ADC 2) (ADC-N) {Arabidopsis thaliana} | | | | | | |
| 4\_1410001\_1440000 | | |  |  | A | B | C | D | P | P' | N |
|  | Cluster:1-2 | |  |  | 1 | 173 | 0 | 4489 | 0.037315033 | 0.037315033 | 1 |
|  |  | RAFL06-13-C07 | At4g03280 / Rieske FeS protein (component of cytochrome B6-F complex) | |  |  |  |  |  | | --- | --- | --- | --- | --- | |  |  |  |  |  | | At4g03280 ,RAFL06-13-C07  cytochrome B6-F complex iron-sulfur subunit, chloroplast / Rieske iron-sulfur protein / plastoquinol-plastocyanin reductase (petC) identical to gi:9843639; identical to cDNA rieske iron-sulfur protein precursor (petC) GI:5725449 | | | | | | |
| 4\_9300001\_9600000 | | |  |  | A | B | C | D | P | P' | N |
|  | Cluster:8-1 | |  |  | 2 | 160 | 4 | 4497 | 0.016411448 | 0.08205724 | 5 |
|  |  | RAFL09-17-E21 | At4g19410 / pectinacetylesterase, putative | |  |  |  |  |  | | --- | --- | --- | --- | --- | |  |  |  |  |  | | RAFL09-17-E21 ,At4g19410  pectinacetylesterase, putative similar to pectinacetylesterase precursor GI:1431629 from [Vigna radiata] | | | | | | |
|  |  | RAFL09-10-G07 | At4g18950 / protein kinase - like protein | |  |  |  |  |  | | --- | --- | --- | --- | --- | |  |  |  |  |  | | At4g18950 ,RAFL09-10-G07  ankyrin protein kinase, putative similar to ankyrin-kinase [Medicago truncatula] gi|18700701|gb|AAL78674 | | | | | | |
| 4\_16800001\_17100000 | | |  |  | A | B | C | D | P | P' | N |
|  | Cluster:8-0 | |  |  | 4 | 105 | 22 | 4532 | 0.0028403332 | 0.036924332 | 13 |
|  |  | RAFL08-11-O11 | At4g37980 / mannitol dehydrogenase (ELI3-1), putative | |  |  |  |  |  | | --- | --- | --- | --- | --- | |  |  |  |  |  | | At4g37980 ,RAFL08-11-O11  mannitol dehydrogenase, putative (ELI3-1) identical to GI:16267 | | | | | | |
|  |  | RAFL08-08-M18 | At4g37980 / mannitol dehydrogenase (ELI3-1), putative | |  |  |  |  |  | | --- | --- | --- | --- | --- | |  |  |  |  |  | | At4g37980 ,RAFL08-08-M18  mannitol dehydrogenase, putative (ELI3-1) identical to GI:16267 | | | | | | |
|  |  | RAFL09-16-M04 | At4g37980 / mannitol dehydrogenase (ELI3-1), putative | |  |  |  |  |  | | --- | --- | --- | --- | --- | |  |  |  |  |  | | At4g37980 ,RAFL09-16-M04  mannitol dehydrogenase, putative (ELI3-1) identical to GI:16267 | | | | | | |
|  |  | RAFL08-10-N24 | At4g38060 / expressed protein | |  |  |  |  |  | | --- | --- | --- | --- | --- | |  |  |  |  |  | | RAFL08-10-N24 ,At4g38060  expressed protein | | | | | | |
|  | Cluster:7-2 | |  |  | 3 | 61 | 23 | 4576 | 0.0051187878 | 0.06654424 | 13 |
|  |  | RAFL08-17-I12 | At4g38550 / Phospholipase like protein | |  |  |  |  |  | | --- | --- | --- | --- | --- | |  |  |  |  |  | | RAFL08-17-I12 ,At4g38550  expressed protein | | | | | | |
|  |  | RAFL04-09-M02 | At4g38400 / expansin protein family (EXPL2) | |  |  |  |  |  | | --- | --- | --- | --- | --- | |  |  |  |  |  | | At4g38400 ,RAFL04-09-M02  expansin family protein (EXPL2) contains Pfam profile: PF01357 pollen allergen; expansin-like gene, PMID:11641069, www.bio.psu.edu/expansins | | | | | | |
|  |  | RAFL09-15-P17 | At4g38550 / Phospholipase like protein | |  |  |  |  |  | | --- | --- | --- | --- | --- | |  |  |  |  |  | | At4g38550 ,RAFL09-15-P17  expressed protein | | | | | | |
|  | Cluster:1-0 | |  |  | 3 | 146 | 23 | 4491 | 0.048582267 | 0.6315695 | 13 |
|  |  | RAFL06-13-F04 | At4g38160 / expressed protein | |  |  |  |  |  | | --- | --- | --- | --- | --- | |  |  |  |  |  | | At4g38160 ,RAFL06-13-F04  mitochondrial transcription termination factor-related / mTERF-related contains Pfam profile PF02536: mTERF | | | | | | |
|  |  | RAFL06-11-A22 | At4g38770 / proline-rich protein family | |  |  |  |  |  | | --- | --- | --- | --- | --- | |  |  |  |  |  | | At4g38770 ,RAFL06-11-A22  proline-rich family protein (PRP4) similar to proline-rich protein [Arabidopsis thaliana] gi|6782442|gb|AAF28388; contains proline-rich extensin domains, INTERPRO:IPR002965 | | | | | | |
|  |  | RAFL07-14-E15 | At4g38520 / protein phosphatase-2c -related | |  |  |  |  |  | | --- | --- | --- | --- | --- | |  |  |  |  |  | | At4g38520 ,RAFL07-14-E15  protein phosphatase 2C family protein / PP2C family protein similar to Ser/Thr protein phosphatase 2C (PP2C6) (GI:15020818) [Arabidopsis thaliana]; similar to protein phosphatase 2C (GI:3608412) [Mesembryanthemum crystallinum]; contains Pfam PF00481 : Protein phosphatase 2C domain; | | | | | | |
| 3\_10290001\_10320000 | | |  |  | A | B | C | D | P | P' | N |
|  | Cluster:0-1 | |  |  | 2 | 104 | 2 | 4555 | 0.0029812786 | 0.008943836 | 3 |
|  |  | RAFL05-21-D22 | At3g27830 / 50S ribosomal protein L12-1, chloroplast precursor (CL12-A) | |  |  |  |  |  | | --- | --- | --- | --- | --- | |  |  |  |  |  | | At3g27830 ,RAFL05-21-D22  50S ribosomal protein L12-1, chloroplast (CL12-A) identical to ribosomal protein L12 GB:X68046 [Arabidopsis thaliana] (J. Biol. Chem. 269 (10), 7330-7336 (1994)) | | | | | | |
|  |  | RAFL11-10-L16 | At3g27830 / 50S ribosomal protein L12-1, chloroplast precursor (CL12-A) | |  |  |  |  |  | | --- | --- | --- | --- | --- | |  |  |  |  |  | | At3g27830 ,RAFL11-10-L16  50S ribosomal protein L12-1, chloroplast (CL12-A) identical to ribosomal protein L12 GB:X68046 [Arabidopsis thaliana] (J. Biol. Chem. 269 (10), 7330-7336 (1994)) | | | | | | |
| 5\_5910001\_5940000 | | |  |  | A | B | C | D | P | P' | N |
|  | Cluster:1-2 | |  |  | 5 | 169 | 2 | 4487 | 1.3512496E-6 | 2.7024992E-6 | 2 |
|  |  | RAFL09-11-C22 | At5g17920 / 5-methyltetrahydropteroyltriglutamate--homocysteine S-methyltransferase | |  |  |  |  |  | | --- | --- | --- | --- | --- | |  |  |  |  |  | | At5g17920 ,RAFL09-11-C22  5-methyltetrahydropteroyltriglutamate--homocysteine methyltransferase / vitamin-B12-independent methionine synthase / cobalamin-independent methionine synthase (CIMS) identical to SP|O50008 5-methyltetrahydropteroyltriglutamate--homocysteine methyltransferase (EC 2.1.1.14) (Vitamin-B12-independent methionine synthase isozyme) (Cobalamin-independent methionine synthase isozyme) {Arabidopsis thaliana} | | | | | | |
|  |  | RAFL09-10-C09 | At5g17920 / 5-methyltetrahydropteroyltriglutamate--homocysteine S-methyltransferase | |  |  |  |  |  | | --- | --- | --- | --- | --- | |  |  |  |  |  | | At5g17920 ,RAFL09-10-C09  5-methyltetrahydropteroyltriglutamate--homocysteine methyltransferase / vitamin-B12-independent methionine synthase / cobalamin-independent methionine synthase (CIMS) identical to SP|O50008 5-methyltetrahydropteroyltriglutamate--homocysteine methyltransferase (EC 2.1.1.14) (Vitamin-B12-independent methionine synthase isozyme) (Cobalamin-independent methionine synthase isozyme) {Arabidopsis thaliana} | | | | | | |
|  |  | RAFL11-01-K15 | At5g17920 / 5-methyltetrahydropteroyltriglutamate--homocysteine S-methyltransferase | |  |  |  |  |  | | --- | --- | --- | --- | --- | |  |  |  |  |  | | At5g17920 ,RAFL11-01-K15  5-methyltetrahydropteroyltriglutamate--homocysteine methyltransferase / vitamin-B12-independent methionine synthase / cobalamin-independent methionine synthase (CIMS) identical to SP|O50008 5-methyltetrahydropteroyltriglutamate--homocysteine methyltransferase (EC 2.1.1.14) (Vitamin-B12-independent methionine synthase isozyme) (Cobalamin-independent methionine synthase isozyme) {Arabidopsis thaliana} | | | | | | |
|  |  | RAFL06-12-D05 | At5g17920 / 5-methyltetrahydropteroyltriglutamate--homocysteine S-methyltransferase | |  |  |  |  |  | | --- | --- | --- | --- | --- | |  |  |  |  |  | | At5g17920 ,RAFL06-12-D05  5-methyltetrahydropteroyltriglutamate--homocysteine methyltransferase / vitamin-B12-independent methionine synthase / cobalamin-independent methionine synthase (CIMS) identical to SP|O50008 5-methyltetrahydropteroyltriglutamate--homocysteine methyltransferase (EC 2.1.1.14) (Vitamin-B12-independent methionine synthase isozyme) (Cobalamin-independent methionine synthase isozyme) {Arabidopsis thaliana} | | | | | | |
|  |  | RAFL11-06-L17 | At5g17920 / 5-methyltetrahydropteroyltriglutamate--homocysteine S-methyltransferase | |  |  |  |  |  | | --- | --- | --- | --- | --- | |  |  |  |  |  | | At5g17920 ,RAFL11-06-L17  5-methyltetrahydropteroyltriglutamate--homocysteine methyltransferase / vitamin-B12-independent methionine synthase / cobalamin-independent methionine synthase (CIMS) identical to SP|O50008 5-methyltetrahydropteroyltriglutamate--homocysteine methyltransferase (EC 2.1.1.14) (Vitamin-B12-independent methionine synthase isozyme) (Cobalamin-independent methionine synthase isozyme) {Arabidopsis thaliana} | | | | | | |
|  | Cluster:0-2 | |  |  | 2 | 77 | 5 | 4579 | 0.0056326813 | 0.011265363 | 2 |
|  |  | RAFL08-16-E05 | At5g17920 / 5-methyltetrahydropteroyltriglutamate--homocysteine S-methyltransferase | |  |  |  |  |  | | --- | --- | --- | --- | --- | |  |  |  |  |  | | At5g17920 ,RAFL08-16-E05  5-methyltetrahydropteroyltriglutamate--homocysteine methyltransferase / vitamin-B12-independent methionine synthase / cobalamin-independent methionine synthase (CIMS) identical to SP|O50008 5-methyltetrahydropteroyltriglutamate--homocysteine methyltransferase (EC 2.1.1.14) (Vitamin-B12-independent methionine synthase isozyme) (Cobalamin-independent methionine synthase isozyme) {Arabidopsis thaliana} | | | | | | |
|  |  | RAFL09-09-A21 | At5g17920 / 5-methyltetrahydropteroyltriglutamate--homocysteine S-methyltransferase | |  |  |  |  |  | | --- | --- | --- | --- | --- | |  |  |  |  |  | | At5g17920 ,RAFL09-09-A21  5-methyltetrahydropteroyltriglutamate--homocysteine methyltransferase / vitamin-B12-independent methionine synthase / cobalamin-independent methionine synthase (CIMS) identical to SP|O50008 5-methyltetrahydropteroyltriglutamate--homocysteine methyltransferase (EC 2.1.1.14) (Vitamin-B12-independent methionine synthase isozyme) (Cobalamin-independent methionine synthase isozyme) {Arabidopsis thaliana} | | | | | | |
| 5\_15930001\_15960000 | | |  |  | A | B | C | D | P | P' | N |
|  | Cluster:2-0 | |  |  | 1 | 149 | 0 | 4513 | 0.03216813 | 0.03216813 | 1 |
|  |  | RAFL06-13-A19 | At5g40510 / sucrose cleavage protein -related | |  |  |  |  |  | | --- | --- | --- | --- | --- | |  |  |  |  |  | | RAFL06-13-A19 ,At5g40510  expressed protein | | | | | | |
| Chromosome:3 | | |  |  | A | B | C | D | P | P' | N |
|  | Cluster:2-1 | |  |  | 67 | 177 | 850 | 3569 | 0.002774971 | 0.09157405 | 33 |
|  |  | RAFL05-09-L11 | At3g48360 / expressed protein | |  |  |  |  |  | | --- | --- | --- | --- | --- | |  |  |  |  |  | | RAFL05-09-L11 ,At3g48360  speckle-type POZ protein-related contains Pfam PF00651 : BTB/POZ domain; similar to Speckle-type POZ protein (SP:O43791) [Homo sapiens] | | | | | | |
|  |  | RAFL06-08-P20 | At3g07110 / 60S ribosomal protein L13A (RPL13aA) | |  |  |  |  |  | | --- | --- | --- | --- | --- | |  |  |  |  |  | | RAFL06-08-P20 ,At3g07110  60S ribosomal protein L13A (RPL13aA) similar to ribosomal protein L13A GB:O49885 [Lupinus luteus] | | | | | | |
|  |  | RAFL05-03-N22 | At3g15410 / leucine rich repeat protein family | |  |  |  |  |  | | --- | --- | --- | --- | --- | |  |  |  |  |  | | RAFL05-03-N22 ,At3g15410  leucine-rich repeat family protein contains leucine rich-repeat (LRR) domains Pfam:PF00560, INTERPRO:IPR001611; contains similarity to Hcr2-5D [Lycopersicon esculentum] gi|3894393|gb|AAC78596; identical to leucine-rich repeat protein [Arabidopsis thaliana] gi|2760084|emb|CAA76000 | | | | | | |
|  |  | RAFL04-12-O11 | At3g27740 / carbamoyl-phosphate synthase (glutamine-hydrolyzing) (glutamine-dependent carbamoyl-phosphate synthase) small subunit | |  |  |  |  |  | | --- | --- | --- | --- | --- | |  |  |  |  |  | | At3g27740 ,RAFL04-12-O11  carbamoyl-phosphate synthase [glutamine-hydrolyzing] (CARA) / glutamine-dependent carbamoyl-phosphate synthase small subunit identical to carbamoyl phosphate synthetase small subunit GI:2462781 [Arabidopsis thaliana] | | | | | | |
|  |  | RAFL07-10-D04 | At3g61150 / homeodomain protein, GLABRA2 like 1 (HD-GL2-1) | |  |  |  |  |  | | --- | --- | --- | --- | --- | |  |  |  |  |  | | At3g61150 ,RAFL07-10-D04  homeobox-leucine zipper family protein / homeodomain GLABRA2 like protein 1 (HD-GL2-1) similar to Anthocyaninless2 (ANL2) (GP:5702094) Arabidopsis thaliana, EMBL:AF077335 | | | | | | |
|  |  | RAFL09-15-G07 | At3g62250 / ubiquitin extension protein (UBQ5)/40S ribosomal protein S27A (RPS27aC) | |  |  |  |  |  | | --- | --- | --- | --- | --- | |  |  |  |  |  | | RAFL09-15-G07 ,At3g62250  ubiquitin extension protein 5 (UBQ5) / 40S ribosomal protein S27A (RPS27aC) identical to GI:166933, GI:166934 | | | | | | |
|  |  | RAFL07-18-N03 | At3g59780 / expressed protein | |  |  |  |  |  | | --- | --- | --- | --- | --- | |  |  |  |  |  | | RAFL07-18-N03 ,At3g59780  expressed protein | | | | | | |
|  |  | RAFL06-08-B12 | At3g01190 / peroxidase, putative | |  |  |  |  |  | | --- | --- | --- | --- | --- | |  |  |  |  |  | | RAFL06-08-B12 ,At3g01190  peroxidase 27 (PER27) (P27) (PRXR7) identical to SP|Q43735 Peroxidase 27 precursor (EC 1.11.1.7) (Atperox P27) (PRXR7) (ATP12a) {Arabidopsis thaliana} | | | | | | |
|  |  | RAFL07-18-J07 | At3g23530 / cyclopropane synthase, putative | |  |  |  |  |  | | --- | --- | --- | --- | --- | |  |  |  |  |  | | RAFL07-18-J07 ,At3g23530  cyclopropane fatty acid synthase, putative / CPA-FA synthase, putative similar to cyclopropane synthase [Sterculia foetida] GI:21069167; contains Pfam profiles PF02353: Cyclopropane-fatty-acyl-phospholipid synthase, PF01593: amine oxidase, flavin-containing | | | | | | |
|  |  | RAFL04-15-J09 | At3g58140 / phenylalanine-tRNA synthetase-related protein | |  |  |  |  |  | | --- | --- | --- | --- | --- | |  |  |  |  |  | | At3g58140 ,RAFL04-15-J09  phenylalanyl-tRNA synthetase class IIc family protein similar to phenylalanine-tRNA synthetase [Homo sapiens] GI:3983103; contains Pfam profile PF01409: tRNA synthetases class II core domain (F) | | | | | | |
|  |  | RAFL05-07-H16 | At3g53870 / 40S ribosomal protein S3 (RPS3B) | |  |  |  |  |  | | --- | --- | --- | --- | --- | |  |  |  |  |  | | At3g53870 ,RAFL05-07-H16  40S ribosomal protein S3 (RPS3B) ribosomal protein S3a - Xenopus laevis, PIR:R3XL3A | | | | | | |
|  |  | RAFL06-09-H09 | At3g48930 / 40S ribosomal protein S11 (RPS11A) | |  |  |  |  |  | | --- | --- | --- | --- | --- | |  |  |  |  |  | | At3g48930 ,RAFL06-09-H09  40S ribosomal protein S11 (RPS11A) | | | | | | |
|  |  | RAFL06-12-N06 | At3g53580 / diaminopimelate epimerase - like protein | |  |  |  |  |  | | --- | --- | --- | --- | --- | |  |  |  |  |  | | At3g53580 ,RAFL06-12-N06  diaminopimelate epimerase family protein contains Pfam profile PF01678: Diaminopimelate epimerase | | | | | | |
|  |  | RAFL02-02-B08 | At3g02540 / RAD23 -related | |  |  |  |  |  | | --- | --- | --- | --- | --- | |  |  |  |  |  | | At3g02540 ,RAFL02-02-B08  ubiquitin family protein contains Pfam profiles PF00240: Ubiquitin family, PF00627: UBA/TS-N domain; | | | | | | |
|  |  | RAFL02-02-A04 | At3g54890 / light-harvesting chlorophyll a/b binding protein | |  |  |  |  |  | | --- | --- | --- | --- | --- | |  |  |  |  |  | | At3g54890 ,RAFL02-02-A04  chlorophyll A-B binding protein / LHCI type I (CAB) identical to chlorophyll A/B-binding protein [Arabidopsis thaliana] GI:16207; contains Pfam profile: PF00504 chlorophyll A-B binding protein | | | | | | |
|  |  | RAFL07-11-J16 | At3g15950 / expressed protein | |  |  |  |  |  | | --- | --- | --- | --- | --- | |  |  |  |  |  | | RAFL07-11-J16 ,At3g15950  DNA topoisomerase-related similar to DNA topoisomerase IV subunit A (GI:26454107) [Mycoplasma penetrans] | | | | | | |
|  |  | RAFL04-17-H07 | At3g57610 / adenylosuccinate synthetase | |  |  |  |  |  | | --- | --- | --- | --- | --- | |  |  |  |  |  | | At3g57610 ,RAFL04-17-H07  adenylosuccinate synthetase (ADSS) identical to adenylosuccinate synthetase, chloroplast precursor (EC 6.3.4.4) (IMP-- aspartate ligase) (AdSS) (AMPSase) (Swiss-Prot:Q96529) [Arabidopsis thaliana] | | | | | | |
|  |  | RAFL09-06-J23 | At3g16470 / jacalin lectin family | |  |  |  |  |  | | --- | --- | --- | --- | --- | |  |  |  |  |  | | RAFL09-06-J23 ,At3g16470  jacalin lectin family protein contains Pfam profile: PF01419 jacalin-like lectin domain; similar to myrosinase-binding protein homolog [Arabidopsis thaliana] GI:2997767 | | | | | | |
|  |  | RAFL04-13-D06 | At3g23940 / dihydroxyacid dehydratase -related | |  |  |  |  |  | | --- | --- | --- | --- | --- | |  |  |  |  |  | | RAFL04-13-D06 ,At3g23940  dehydratase family contains Pfam profile: PF00920 dehydratase family | | | | | | |
|  |  | RAFL07-18-J24 | At3g02690 / expressed integral membrane protein | |  |  |  |  |  | | --- | --- | --- | --- | --- | |  |  |  |  |  | | RAFL07-18-J24 ,At3g02690  integral membrane family protein similar to PecM protein (GI:5852331) {Vogesella indigofera} and PecM protein (SP:P42194) [Erwinia chrysanthemi] | | | | | | |
|  |  | RAFL06-14-I03 | At3g60770 / 40S ribosomal protein S13 (RPS13A) | |  |  |  |  |  | | --- | --- | --- | --- | --- | |  |  |  |  |  | | RAFL06-14-I03 ,At3g60770  40S ribosomal protein S13 (RPS13A) AtRPS13A mRNA for cytoplasmic ribosomal protein S13, Arabidopsis thaliana,AB031739 | | | | | | |
|  |  | RAFL06-08-B09 | At3g11510 / 40S ribosomal protein S14 (RPS14B) | |  |  |  |  |  | | --- | --- | --- | --- | --- | |  |  |  |  |  | | At3g11510 ,RAFL06-08-B09  40S ribosomal protein S14 (RPS14B) similar to 40S ribosomal protein S14 GB:P19950 [Zea mays] | | | | | | |
|  |  | RAFL05-05-K17 | At3g03780 / methionine synthase -related | |  |  |  |  |  | | --- | --- | --- | --- | --- | |  |  |  |  |  | | RAFL05-05-K17 ,At3g03780  5-methyltetrahydropteroyltriglutamate--homocysteine methyltransferase, putative / vitamin-B12-independent methionine synthase, putative / cobalamin-independent methionine synthase, putative very strong similarity to SP|O50008 5-methyltetrahydropteroyltriglutamate--homocysteine methyltransferase (EC 2.1.1.14) (Vitamin-B12-independent methionine synthase isozyme) (Cobalamin-independent methionine synthase isozyme) {Arabidopsis thaliana}; contains Pfam profile PF01717: Methionine synthase, vitamin-B12 independent | | | | | | |
|  |  | RAFL09-06-A22 | At3g62870 / 60S ribosomal protein L7A (RPL7aB) | |  |  |  |  |  | | --- | --- | --- | --- | --- | |  |  |  |  |  | | RAFL09-06-A22 ,At3g62870  60S ribosomal protein L7A (RPL7aB) 60S RIBOSOMAL PROTEIN L7A - Oryza sativa, SWISSPROT:RL7A\_ORYSA | | | | | | |
|  |  | RAFL09-07-D04 | At3g22230 / 60S ribosomal protein L27 (RPL27B) | |  |  |  |  |  | | --- | --- | --- | --- | --- | |  |  |  |  |  | | At3g22230 ,RAFL09-07-D04  60S ribosomal protein L27 (RPL27B) similar to 60S RIBOSOMAL PROTEIN L27 GB:P41101 from [Solanum tuberosum] | | | | | | |
|  |  | RAFL04-18-D21 | At3g57230 / MADS-box protein | |  |  |  |  |  | | --- | --- | --- | --- | --- | |  |  |  |  |  | | At3g57230 ,RAFL04-18-D21  MADS-box protein (AGL16) MADS-box transcription factor DEFH125 - Antirrhinum majus, PIR:T17029; contains Pfam domain PF00319: SRF-type transcription factor (DNA-binding and dimerisation domain); contains Pfam domain PF01486: K-box region | | | | | | |
|  |  | RAFL06-16-N12 | At3g46740 / chloroplast import-associated channel protein homolog | |  |  |  |  |  | | --- | --- | --- | --- | --- | |  |  |  |  |  | | At3g46740 ,RAFL06-16-N12  chloroplast outer envelope protein, putative similar to chloroplastic outer envelope membrane protein (OEP75) [Pisum sativum] GI:633607; contains Pfam profile PF01103: outer membrane protein, OMP85 family | | | | | | |
|  |  | RAFL07-14-F14 | At3g14840 / receptor-related serine/threonine kinase | |  |  |  |  |  | | --- | --- | --- | --- | --- | |  |  |  |  |  | | At3g14840 ,RAFL07-14-F14  leucine-rich repeat family protein / protein kinase family protein contains Pfam domains PF00560: Leucine Rich Repeat and PF00069: Protein kinase domain; contains 2 predicted transmembrane domains | | | | | | |
|  |  | RAFL04-20-J04 | At3g25530 / gamma hydroxybutyrate dehydrogenase | |  |  |  |  |  | | --- | --- | --- | --- | --- | |  |  |  |  |  | | At3g25530 ,RAFL04-20-J04  6-phosphogluconate dehydrogenase NAD-binding domain-containing protein low similarity to SP|P23523 2-hydroxy-3-oxopropionate reductase (EC 1.1.1.60) (Tartronate semialdehyde reductase) {Escherichia coli}; contains Pfam profile PF03446: NAD binding domain of 6-phosphogluconate dehydrogenase; supporting cDNA gi|15375067|gb|AY044183.1| | | | | | | |
|  |  | RAFL05-08-L19 | At3g43540 / expressed protein | |  |  |  |  |  | | --- | --- | --- | --- | --- | |  |  |  |  |  | | At3g43540 ,RAFL05-08-L19  expressed protein hypothetical protein slr1699 - Synechocystis sp. (strain PCC 6803), PIR:S75306 | | | | | | |
|  |  | RAFL06-10-G07 | At3g11500 / small nuclear ribonucleo protein polypeptide G -related | |  |  |  |  |  | | --- | --- | --- | --- | --- | |  |  |  |  |  | | At3g11500 ,RAFL06-10-G07  small nuclear ribonucleoprotein G, putative / snRNP-G, putative / Sm protein G, putative similar to SWISS-PROT:Q15357 small nuclear ribonucleoprotein G (snRNP-G, Sm protein G, Sm-G, SmG) [Homo sapiens] | | | | | | |
|  |  | RAFL05-20-D01 | At3g51510 / expressed protein | |  |  |  |  |  | | --- | --- | --- | --- | --- | |  |  |  |  |  | | RAFL05-20-D01 ,At3g51510  expressed protein | | | | | | |
|  |  | RAFL09-07-O12 | At3g26520 / tonoplast intrinsic protein, putative | |  |  |  |  |  | | --- | --- | --- | --- | --- | |  |  |  |  |  | | At3g26520 ,RAFL09-07-O12  tonoplast intrinsic protein, putative similar to tonoplast intrinsic protein GI:5081419 from [Brassica napus] | | | | | | |
|  |  | RAFL04-13-N21 | At3g02870 / myo-inositol monophosphatase -related | |  |  |  |  |  | | --- | --- | --- | --- | --- | |  |  |  |  |  | | RAFL04-13-N21 ,At3g02870  inositol-1(or 4)-monophosphatase, putative / inositol monophosphatase, putative / IMPase, putative similar to SP|P54928 Inositol-1(or 4)-monophosphatase 3 (EC 3.1.3.25) (IMPase 3) (IMP 3) (Inositol monophosphatase 3) {Lycopersicon esculentum}; contains Pfam profile PF00459: Inositol monophosphatase family | | | | | | |
|  |  | RAFL05-01-G23 | At3g49600 / ubiquitin-specific protease 26 (UBP26) | |  |  |  |  |  | | --- | --- | --- | --- | --- | |  |  |  |  |  | | At3g49600 ,RAFL05-01-G23  ubiquitin-specific protease 26 (UBP26) similar to GI:11993492; RNA binding protein - Homo sapiens, EMBL:AB016089 (N-terminus), several ubiquitin carboxyl-terminal hydrolases from aa pos. 712 | | | | | | |
|  |  | RAFL06-16-M11 | At3g18780 / actin 2 | |  |  |  |  |  | | --- | --- | --- | --- | --- | |  |  |  |  |  | | At3g18780 ,RAFL06-16-M11  actin 2 (ACT2) identical to SP|Q96292 Actin 2 {Arabidopsis thaliana}; nearly identical to SP|Q96293 Actin 8 [Arabidopsis thaliana] GI:1669387 and to At1g49240 | | | | | | |
|  |  | RAFL11-05-B21 | At3g54210 / ribosomal protein L17 -related protein | |  |  |  |  |  | | --- | --- | --- | --- | --- | |  |  |  |  |  | | At3g54210 ,RAFL11-05-B21  ribosomal protein L17 family protein contains Pfam profile: PF01196 ribosomal protein L17 | | | | | | |
|  |  | RAFL06-13-D11 | At3g14120 / expressed protein | |  |  |  |  |  | | --- | --- | --- | --- | --- | |  |  |  |  |  | | At3g14120 ,RAFL06-13-D11  expressed protein similar to Nuclear pore complex protein Nup107 (Nucleoporin Nup107) (107 kDa nucleoporin) (p105) (Swiss-Prot:P52590) [Rattus norvegicus] | | | | | | |
|  |  | RAFL07-16-B09 | At3g48360 / expressed protein | |  |  |  |  |  | | --- | --- | --- | --- | --- | |  |  |  |  |  | | RAFL07-16-B09 ,At3g48360  speckle-type POZ protein-related contains Pfam PF00651 : BTB/POZ domain; similar to Speckle-type POZ protein (SP:O43791) [Homo sapiens] | | | | | | |
|  |  | RAFL11-12-H04 | At3g25520 / 60S ribosomal protein L5 (RPL5A) | |  |  |  |  |  | | --- | --- | --- | --- | --- | |  |  |  |  |  | | At3g25520 ,RAFL11-12-H04  60S ribosomal protein L5 similar to 60S ribosomal protein L5 GB:P49625 from [Oryza sativa] | | | | | | |
|  |  | RAFL11-07-B21 | At3g25520 / 60S ribosomal protein L5 (RPL5A) | |  |  |  |  |  | | --- | --- | --- | --- | --- | |  |  |  |  |  | | At3g25520 ,RAFL11-07-B21  60S ribosomal protein L5 similar to 60S ribosomal protein L5 GB:P49625 from [Oryza sativa] | | | | | | |
|  |  | RAFL07-10-A20 | At3g54010 / peptidylprolyl isomerase (pasticcino 1) | |  |  |  |  |  | | --- | --- | --- | --- | --- | |  |  |  |  |  | | RAFL07-10-A20 ,At3g54010  peptidyl-prolyl cis-trans isomerase, putative / FK506-binding protein, putative / pasticcino 1-D (PAS1-D) nearly identical to pasticcino 1-D [Arabidopsis thaliana] GI:3080740 | | | | | | |
|  |  | RAFL05-17-P11 | At3g04920 / 40S ribosomal protein S24 (RPS24A) | |  |  |  |  |  | | --- | --- | --- | --- | --- | |  |  |  |  |  | | At3g04920 ,RAFL05-17-P11  40S ribosomal protein S24 (RPS24A) similar to ribosomal protein S19 GB:445612 [Solanum tuberosum] and similar to ribosomal protein S24 GB:4506703 [Homo sapiens] | | | | | | |
|  |  | RAFL07-08-E22 | At3g23530 / cyclopropane synthase, putative | |  |  |  |  |  | | --- | --- | --- | --- | --- | |  |  |  |  |  | | RAFL07-08-E22 ,At3g23530  cyclopropane fatty acid synthase, putative / CPA-FA synthase, putative similar to cyclopropane synthase [Sterculia foetida] GI:21069167; contains Pfam profiles PF02353: Cyclopropane-fatty-acyl-phospholipid synthase, PF01593: amine oxidase, flavin-containing | | | | | | |
|  |  | RAFL06-08-P08 | At3g23390 / 60S ribosomal protein L36a/L44 (RPL36aA) | |  |  |  |  |  | | --- | --- | --- | --- | --- | |  |  |  |  |  | | RAFL06-08-P08 ,At3g23390  60S ribosomal protein L36a/L44 (RPL36aA) similar to ribosomal protein L41 GB:AAA34366 from [Candida maltosa] | | | | | | |
|  |  | RAFL05-12-K04 | At3g23050 / auxin-responsive protein IAA7 (Indoleacetic acid-induced protein 7) | |  |  |  |  |  | | --- | --- | --- | --- | --- | |  |  |  |  |  | | RAFL05-12-K04 ,At3g23050  auxin-responsive protein / indoleacetic acid-induced protein 7 (IAA7) identical to SP|Q38825|AXI7\_ARATH Auxin-responsive protein IAA7 (Indoleacetic acid-induced protein 7) | | | | | | |
|  |  | RAFL09-06-K03 | At3g26070 / plastid-lipid associated protein PAP/fibrillin family | |  |  |  |  |  | | --- | --- | --- | --- | --- | |  |  |  |  |  | | At3g26070 ,RAFL09-06-K03  plastid-lipid associated protein PAP / fibrillin family protein contains Pfam profile PF04755: PAP\_fibrillin | | | | | | |
|  |  | RAFL05-19-H05 | At3g28900 / 60S ribosomal protein L34 (RPL34C) | |  |  |  |  |  | | --- | --- | --- | --- | --- | |  |  |  |  |  | | RAFL05-19-H05 ,At3g28900  60S ribosomal protein L34 (RPL34C) similar to 60S ribosomal protein L34 GB:P41098 [Nicotiana tabacum] | | | | | | |
|  |  | RAFL02-01-G08 | At3g58610 / ketol-acid reductoisomerase | |  |  |  |  |  | | --- | --- | --- | --- | --- | |  |  |  |  |  | | RAFL02-01-G08 ,At3g58610  ketol-acid reductoisomerase identical to ketol-acid reductoisomerase, chloroplast precursor (EC 1.1.1.86) (Acetohydroxy-acid reductoisomerase) (Alpha-keto-beta-hydroxylacil reductoisomerase) (Swiss-Prot:Q05758) [Arabidopsis thaliana] | | | | | | |
|  |  | RAFL06-08-E09 | At3g54640 / tryptophan synthase, alpha subunit (TSA1) | |  |  |  |  |  | | --- | --- | --- | --- | --- | |  |  |  |  |  | | RAFL06-08-E09 ,At3g54640  tryptophan synthase, alpha subunit (TSA1) identical to gi:619753 | | | | | | |
|  |  | RAFL05-18-N15 | At3g15353 / expressed protein | |  |  |  |  |  | | --- | --- | --- | --- | --- | |  |  |  |  |  | | At3g15353 ,RAFL05-18-N15  metallothionein protein, putative | | | | | | |
|  |  | RAFL04-14-L08 | At3g49010 / 60S ribosomal protein L13 (RPL13B)/breast basic conserved protein 1-related (BBC1) | |  |  |  |  |  | | --- | --- | --- | --- | --- | |  |  |  |  |  | | At3g49010 ,RAFL04-14-L08  60S ribosomal protein L13 (RPL13B) / breast basic conserved protein 1-related (BBC1) | | | | | | |
|  |  | RAFL11-07-J05 | At3g16420 / myrosinase binding protein, putative | |  |  |  |  |  | | --- | --- | --- | --- | --- | |  |  |  |  |  | | RAFL11-07-J05 ,At3g16420  jacalin lectin family protein similar to myrosinase binding protein [Brassica napus] GI:1711296; contains Pfam profile: PF01419 jacalin-like lectin domain | | | | | | |
|  |  | RAFL05-21-L03 | At3g23400 / plastid-lipid associated protein PAP/fibrillin family | |  |  |  |  |  | | --- | --- | --- | --- | --- | |  |  |  |  |  | | RAFL05-21-L03 ,At3g23400  plastid-lipid associated protein PAP / fibrillin family protein contains Pfam profile PF04755: PAP\_fibrillin | | | | | | |
|  |  | RAFL05-21-D08 | At3g18490 / chloroplast nucleoid DNA-binding protein -related | |  |  |  |  |  | | --- | --- | --- | --- | --- | |  |  |  |  |  | | At3g18490 ,RAFL05-21-D08  aspartyl protease family protein contains Pfam domain, PF00026: eukaryotic aspartyl protease | | | | | | |
|  |  | RAFL04-10-H14 | At3g49910 / 60S ribosomal protein L26 (RPL26A) | |  |  |  |  |  | | --- | --- | --- | --- | --- | |  |  |  |  |  | | At3g49910 ,RAFL04-10-H14  60S ribosomal protein L26 (RPL26A) 60S RIBOSOMAL PROTEIN L26, Brassica rapa, EMBL:BRD495 | | | | | | |
|  |  | RAFL11-09-J03 | At3g54210 / ribosomal protein L17 -related protein | |  |  |  |  |  | | --- | --- | --- | --- | --- | |  |  |  |  |  | | RAFL11-09-J03 ,At3g54210  ribosomal protein L17 family protein contains Pfam profile: PF01196 ribosomal protein L17 | | | | | | |
|  |  | RAFL05-17-L17 | At3g55280 / 60S ribosomal protein L23A (RPL23aB) | |  |  |  |  |  | | --- | --- | --- | --- | --- | |  |  |  |  |  | | RAFL05-17-L17 ,At3g55280  60S ribosomal protein L23A (RPL23aB) various ribosomal L23a proteins | | | | | | |
|  |  | RAFL04-16-M03 | At3g20790 / expressed protein | |  |  |  |  |  | | --- | --- | --- | --- | --- | |  |  |  |  |  | | At3g20790 ,RAFL04-16-M03  oxidoreductase family protein weak similarity to SP|Q07982 Glucose--fructose oxidoreductase precursor (EC 1.1.99.28) {Zymomonas mobilis}; contains Pfam profiles PF01408: Oxidoreductase family NAD-binding Rossmann fold, PF02894: Oxidoreductase family C-terminal alpha/beta domain | | | | | | |
|  |  | RAFL05-13-D18 | At3g04400 / 60S ribosomal protein L23 (RPL23C) | |  |  |  |  |  | | --- | --- | --- | --- | --- | |  |  |  |  |  | | At3g04400 ,RAFL05-13-D18  60S ribosomal protein L23 (RPL23C) similar to ribosomal protein L17 GB:AAA34113.1 from [Nicotiana tabacum] | | | | | | |
|  |  | RAFL05-13-M17 | At3g53890 / 40S ribosomal protein S21 homolog | |  |  |  |  |  | | --- | --- | --- | --- | --- | |  |  |  |  |  | | RAFL05-13-M17 ,At3g53890  40S ribosomal protein S21 (RPS21B) ribosomal protein S21, cytosolic - Oryza sativa, PIR:S38357 | | | | | | |
|  |  | RAFL08-13-I06 | At3g28180 / glycosyltransferase family 2 | |  |  |  |  |  | | --- | --- | --- | --- | --- | |  |  |  |  |  | | RAFL08-13-I06 ,At3g28180  glycosyl transferase family 2 protein similar to beta-(1-3)-glucosyl transferase GB:AAC62210 GI:3687658 from [Bradyrhizobium japonicum], cellulose synthase from Agrobacterium tumeficiens [gi:710492] and Agrobacterium radiobacter [gi:710493]; contains Pfam glycosyl transferase, group 2 family protein domain PF00535 | | | | | | |
|  |  | RAFL07-12-K04 | At3g20050 / T-complex protein 1, alpha subunit/chaperonin | |  |  |  |  |  | | --- | --- | --- | --- | --- | |  |  |  |  |  | | RAFL07-12-K04 ,At3g20050  T-complex protein 1 alpha subunit / TCP-1-alpha / chaperonin (CCT1) identical to SWISS-PROT:P28769- T-complex protein 1, alpha subunit (TCP-1-alpha) [Arabidopsis thaliana] | | | | | | |
|  |  | RAFL11-10-K08 | At3g22230 / 60S ribosomal protein L27 (RPL27B) | |  |  |  |  |  | | --- | --- | --- | --- | --- | |  |  |  |  |  | | At3g22230 ,RAFL11-10-K08  60S ribosomal protein L27 (RPL27B) similar to 60S RIBOSOMAL PROTEIN L27 GB:P41101 from [Solanum tuberosum] | | | | | | |
|  |  | RAFL05-03-L01 | At3g56340 / 40S ribosomal protein S26 homolog | |  |  |  |  |  | | --- | --- | --- | --- | --- | |  |  |  |  |  | | At3g56340 ,RAFL05-03-L01  40S ribosomal protein S26 (RPS26C) several 40S ribosomal protein S26 | | | | | | |
|  |  | RAFL06-12-K17 | At3g10060 / immunophilin / FKBP-type peptidyl-prolyl cis-trans isomerase, putative | |  |  |  |  |  | | --- | --- | --- | --- | --- | |  |  |  |  |  | | RAFL06-12-K17 ,At3g10060  immunophilin, putative / FKBP-type peptidyl-prolyl cis-trans isomerase, putative Pfam:PF-254: FKBP-type peptidyl-prolyl cis-trans isomerases | | | | | | |
|  |  | RAFL07-09-D05 | At3g60320 / bZIP protein | |  |  |  |  |  | | --- | --- | --- | --- | --- | |  |  |  |  |  | | At3g60320 ,RAFL07-09-D05  expressed protein contains Pfam profiles: PF04782: protein of unknown function (DUF632), PF04783: protein of unknown function (DUF630) | | | | | | |
|  | Cluster:3-2 | |  |  | 14 | 23 | 903 | 3723 | 0.0107122185 | 0.3535032 | 33 |
|  |  | RAFL09-06-N12 | At3g23810 / S-adenosyl-L-homocysteinas -related | |  |  |  |  |  | | --- | --- | --- | --- | --- | |  |  |  |  |  | | At3g23810 ,RAFL09-06-N12  adenosylhomocysteinase, putative / S-adenosyl-L-homocysteine hydrolase, putative / AdoHcyase, putative strong similarity to SP|P50248|SAHH\_TOBAC Adenosylhomocysteinase (EC 3.3.1.1) (S-adenosyl-L-homocysteine hydrolase) (AdoHcyase) {Nicotiana sylvestris}; contains Pfam profile PF00670: S-adenosyl-L-homocysteine hydrolase, NAD binding domain | | | | | | |
|  |  | RAFL06-08-F13 | At3g55360 / 3-oxo-5-alpha-steroid 4-dehydrogenase (steroid 5-alpha-reductase) family | |  |  |  |  |  | | --- | --- | --- | --- | --- | |  |  |  |  |  | | At3g55360 ,RAFL06-08-F13  3-oxo-5-alpha-steroid 4-dehydrogenase family protein / steroid 5-alpha-reductase family protein similar to synaptic glycoprotein SC2 spliced variant from Homo sapiens [EMBL:AF038958], SC2 from Rattus sp. [gi:256994]; contains Pfam 3-oxo-5-alpha-steroid 4-dehydrogenase domain PF02544 | | | | | | |
|  |  | RAFL09-13-P13 | At3g23810 / S-adenosyl-L-homocysteinas -related | |  |  |  |  |  | | --- | --- | --- | --- | --- | |  |  |  |  |  | | RAFL09-13-P13 ,At3g23810  adenosylhomocysteinase, putative / S-adenosyl-L-homocysteine hydrolase, putative / AdoHcyase, putative strong similarity to SP|P50248|SAHH\_TOBAC Adenosylhomocysteinase (EC 3.3.1.1) (S-adenosyl-L-homocysteine hydrolase) (AdoHcyase) {Nicotiana sylvestris}; contains Pfam profile PF00670: S-adenosyl-L-homocysteine hydrolase, NAD binding domain | | | | | | |
|  |  | RAFL07-09-L01 | At3g23810 / S-adenosyl-L-homocysteinas -related | |  |  |  |  |  | | --- | --- | --- | --- | --- | |  |  |  |  |  | | RAFL07-09-L01 ,At3g23810  adenosylhomocysteinase, putative / S-adenosyl-L-homocysteine hydrolase, putative / AdoHcyase, putative strong similarity to SP|P50248|SAHH\_TOBAC Adenosylhomocysteinase (EC 3.3.1.1) (S-adenosyl-L-homocysteine hydrolase) (AdoHcyase) {Nicotiana sylvestris}; contains Pfam profile PF00670: S-adenosyl-L-homocysteine hydrolase, NAD binding domain | | | | | | |
|  |  | RAFL06-11-L24 | At3g46650 / UDP-glycosyltransferase family | |  |  |  |  |  | | --- | --- | --- | --- | --- | |  |  |  |  |  | | At3g46650 ,RAFL06-11-L24  UDP-glucoronosyl/UDP-glucosyl transferase family protein contains Pfam profile: PF00201 UDP-glucoronosyl and UDP-glucosyl transferase | | | | | | |
|  |  | RAFL05-02-I24 | At3g20390 / translational inhibitor protein -related | |  |  |  |  |  | | --- | --- | --- | --- | --- | |  |  |  |  |  | | At3g20390 ,RAFL05-02-I24  endoribonuclease L-PSP family protein contains Pfam domain PF01042: Endoribonuclease L-PSP | | | | | | |
|  |  | RAFL07-08-K09 | At3g16000 / myosin heavy chain-related protein | |  |  |  |  |  | | --- | --- | --- | --- | --- | |  |  |  |  |  | | RAFL07-08-K09 ,At3g16000  matrix-localized MAR DNA-binding protein-related similar to matrix-localized MAR DNA binding protein MFP1 GI:1771158 from [Lycopersicon esculentum] | | | | | | |
|  |  | RAFL07-12-E11 | At3g02230 / reversibly glycosylated polypeptide-1 | |  |  |  |  |  | | --- | --- | --- | --- | --- | |  |  |  |  |  | | At3g02230 ,RAFL07-12-E11  reversibly glycosylated polypeptide-1 (RGP1) identical to reversibly glycosylated polypeptide-1 (AtRGP) [Arabidopsis thaliana] GI:2317729 | | | | | | |
|  |  | RAFL03-08-F09 | At3g25770 / allene oxide cyclase family | |  |  |  |  |  | | --- | --- | --- | --- | --- | |  |  |  |  |  | | RAFL03-08-F09 ,At3g25770  allene oxide cyclase, putative / early-responsive to dehydration protein, putative / ERD protein, putative strong similarity to early-responsive to dehydration (ERD12) protein [GI:15320414]; similar to allene oxide cyclase GI:8977961 from [Lycopersicon esculentum]; contains Pfam profile PF06351: Allene oxide cyclase | | | | | | |
|  |  | RAFL09-16-F08 | At3g23820 / NAD-dependent epimerase/dehydratase family | |  |  |  |  |  | | --- | --- | --- | --- | --- | |  |  |  |  |  | | At3g23820 ,RAFL09-16-F08  NAD-dependent epimerase/dehydratase family protein similar to nucleotide sugar epimerase from Vibrio vulnificus GI:3093975 [PID:g3093975], WbnF [Escherichia coli] GI:5739472, CAPI protein {Staphylococcus aureus} SP|P39858; contains Pfam profile: PF01370 NAD dependent epimerase/dehydratase family | | | | | | |
|  |  | RAFL05-09-N18 | At3g43720 / protease inhibitor/seed storage/lipid transfer protein (LTP) family | |  |  |  |  |  | | --- | --- | --- | --- | --- | |  |  |  |  |  | | RAFL05-09-N18 ,At3g43720  protease inhibitor/seed storage/lipid transfer protein (LTP) family protein contains Pfam protease inhibitor/seed storage/LTP family domain PF00234 | | | | | | |
|  |  | RAFL09-07-D12 | At3g23820 / NAD-dependent epimerase/dehydratase family | |  |  |  |  |  | | --- | --- | --- | --- | --- | |  |  |  |  |  | | At3g23820 ,RAFL09-07-D12  NAD-dependent epimerase/dehydratase family protein similar to nucleotide sugar epimerase from Vibrio vulnificus GI:3093975 [PID:g3093975], WbnF [Escherichia coli] GI:5739472, CAPI protein {Staphylococcus aureus} SP|P39858; contains Pfam profile: PF01370 NAD dependent epimerase/dehydratase family | | | | | | |
|  |  | RAFL09-10-M18 | At3g23810 / S-adenosyl-L-homocysteinas -related | |  |  |  |  |  | | --- | --- | --- | --- | --- | |  |  |  |  |  | | RAFL09-10-M18 ,At3g23810  adenosylhomocysteinase, putative / S-adenosyl-L-homocysteine hydrolase, putative / AdoHcyase, putative strong similarity to SP|P50248|SAHH\_TOBAC Adenosylhomocysteinase (EC 3.3.1.1) (S-adenosyl-L-homocysteine hydrolase) (AdoHcyase) {Nicotiana sylvestris}; contains Pfam profile PF00670: S-adenosyl-L-homocysteine hydrolase, NAD binding domain | | | | | | |
|  |  | RAFL05-07-N10 | At3g55130 / ABC transporter family protein | |  |  |  |  |  | | --- | --- | --- | --- | --- | |  |  |  |  |  | | At3g55130 ,RAFL05-07-N10  ABC transporter family protein breast cancer resistance protein 1 BCRP1, Mus musculus, EMBL:NP\_036050 | | | | | | |
| 3\_4830001\_4860000 | | |  |  | A | B | C | D | P | P' | N |
|  | Cluster:10-1 | |  |  | 1 | 42 | 0 | 4620 | 0.009221531 | 0.009221531 | 1 |
|  |  | RAFL08-11-H16 | At3g14440 / 9-cis-epoxycarotenoid dioxygenase (neoxanthin cleavage enzyme)(NC1)(NCED1), putative | |  |  |  |  |  | | --- | --- | --- | --- | --- | |  |  |  |  |  | | At3g14440 ,RAFL08-11-H16  9-cis-epoxycarotenoid dioxygenase, putative / neoxanthin cleavage enzyme, putative / carotenoid cleavage dioxygenase, putative similar to 9-cis-epoxycarotenoid dioxygenase GB:AAF26356 [GI:6715257][Phaseolus vulgaris] | | | | | | |
| 2\_7680001\_7710000 | | |  |  | A | B | C | D | P | P' | N |
|  | Cluster:10-1 | |  |  | 1 | 42 | 0 | 4620 | 0.009221531 | 0.009221531 | 1 |
|  |  | RAFL08-19-H17 | At2g17840 / senescence-associated protein 12 -related | |  |  |  |  |  | | --- | --- | --- | --- | --- | |  |  |  |  |  | | At2g17840 ,RAFL08-19-H17  senescence/dehydration-associated protein-related (ERD7) similar to senescence-associated protein 12 [Hemerocallis hybrid cultivar] gi|3551958|gb|AAC34857; strong similarity to early-responsive to dehydration stress ERD7 protein [Arabidopsis thaliana] gi|15320412|dbj|BAB63916; identical to cDNA ERD7 partial cds GI:15320411 | | | | | | |
| 1\_16380001\_16410000 | | |  |  | A | B | C | D | P | P' | N |
|  | Cluster:0-1 | |  |  | 2 | 104 | 0 | 4557 | 5.119853E-4 | 5.119853E-4 | 1 |
|  |  | RAFL05-18-I22 | At1g44575 / photosystem II 22kDa protein -related | |  |  |  |  |  | | --- | --- | --- | --- | --- | |  |  |  |  |  | | At1g44575 ,RAFL05-18-I22  photosystem II 22kDa protein, chloroplast / CP22 (PSBS) identical to photosystem II 22 kDa protein, chloroplast [precursor] SP:Q9XF91 from [Arabidopsis thaliana]; contains Pfam profile PF00504: Chlorophyll A-B binding protein | | | | | | |
|  |  | RAFL06-13-A08 | At1g44575 / photosystem II 22kDa protein -related | |  |  |  |  |  | | --- | --- | --- | --- | --- | |  |  |  |  |  | | RAFL06-13-A08 ,At1g44575  photosystem II 22kDa protein, chloroplast / CP22 (PSBS) identical to photosystem II 22 kDa protein, chloroplast [precursor] SP:Q9XF91 from [Arabidopsis thaliana]; contains Pfam profile PF00504: Chlorophyll A-B binding protein | | | | | | |
| 5\_4560001\_4590000 | | |  |  | A | B | C | D | P | P' | N |
|  | Cluster:0-1 | |  |  | 1 | 105 | 1 | 4556 | 0.044952307 | 0.089904614 | 2 |
|  |  | RAFL05-15-H14 | At5g14200 / 3-isopropylmalate dehydrogenase, chloroplast, putative | |  |  |  |  |  | | --- | --- | --- | --- | --- | |  |  |  |  |  | | At5g14200 ,RAFL05-15-H14  3-isopropylmalate dehydrogenase, chloroplast, putative strong similarity to SP|P29102 3-isopropylmalate dehydrogenase, chloroplast precursor {Brassica napus} | | | | | | |
| 5\_6600001\_6630000 | | |  |  | A | B | C | D | P | P' | N |
|  | Cluster:4-2 | |  |  | 1 | 134 | 0 | 4528 | 0.028951319 | 0.028951319 | 1 |
|  |  | RAFL04-17-L09 | At5g19590 / expressed protein | |  |  |  |  |  | | --- | --- | --- | --- | --- | |  |  |  |  |  | | At5g19590 ,RAFL04-17-L09  expressed protein contains Pfam profile PF04398: Protein of unknown function, DUF538 | | | | | | |
| 5\_2580001\_2610000 | | |  |  | A | B | C | D | P | P' | N |
|  | Cluster:3-0 | |  |  | 1 | 232 | 0 | 4430 | 0.049967833 | 0.049967833 | 1 |
|  |  | RAFL04-20-H11 | At5g08100 / asparaginase | |  |  |  |  |  | | --- | --- | --- | --- | --- | |  |  |  |  |  | | At5g08100 ,RAFL04-20-H11  L-asparaginase / L-asparagine amidohydrolase identical to Swiss-Prot:P50287 L-asparaginase (EC 3.5.1.1) (L-asparagine amidohydrolase) [Arabidopsis thaliana] | | | | | | |
| 2\_7500001\_7800000 | | |  |  | A | B | C | D | P | P' | N |
|  | Cluster:8-0 | |  |  | 2 | 107 | 9 | 4545 | 0.025949622 | 0.18164736 | 7 |
|  |  | RAFL05-20-P13 | At2g18050 / histone H1 | |  |  |  |  |  | | --- | --- | --- | --- | --- | |  |  |  |  |  | | RAFL05-20-P13 ,At2g18050  histone H1-3 (HIS1-3) similar to histone H1 [Lycopersicon pennellii] SWISS-PROT:P40267; identical to cDNA histone H1-3 (His1-3) GI:1809314, histone H1-3 [Arabidopsis thaliana] GI:1809305 | | | | | | |
|  |  | RAFL08-16-B13 | At2g17390 / glucanase -related | |  |  |  |  |  | | --- | --- | --- | --- | --- | |  |  |  |  |  | | At2g17390 ,RAFL08-16-B13  ankyrin repeat family protein contains ankyrin repeats, Pfam:PF00023 | | | | | | |
| 4\_15330001\_15360000 | | |  |  | A | B | C | D | P | P' | N |
|  | Cluster:10-1 | |  |  | 1 | 42 | 3 | 4617 | 0.03639058 | 0.14556232 | 4 |
|  |  | RAFL04-14-P24 | At4g34230 / cinnamyl-alcohol dehydrogenase (CAD), putative | |  |  |  |  |  | | --- | --- | --- | --- | --- | |  |  |  |  |  | | RAFL04-14-P24 ,At4g34230  cinnamyl-alcohol dehydrogenase, putative similar to cinnamyl alcohol dehydrogenase, Nicotiana tabacum [SP|P30359], Populus deltoides, PATCHX:G288753 | | | | | | |
|  | Cluster:2-2 | |  |  | 1 | 52 | 3 | 4607 | 0.044709165 | 0.17883666 | 4 |
|  |  | RAFL06-12-L22 | At4g34190 / stress enhanced protein 1 (SEP1) | |  |  |  |  |  | | --- | --- | --- | --- | --- | |  |  |  |  |  | | RAFL06-12-L22 ,At4g34190  stress enhanced protein 1 (SEP1) identical to stress enhanced protein 1 (SEP1) GI:7384978 from [Arabidopsis thaliana] | | | | | | |
| 5\_10410001\_10440000 | | |  |  | A | B | C | D | P | P' | N |
|  | Cluster:1-0 | |  |  | 1 | 148 | 0 | 4514 | 0.031953678 | 0.031953678 | 1 |
|  |  | RAFL08-12-K23 | At5g28500 / expressed protein | |  |  |  |  |  | | --- | --- | --- | --- | --- | |  |  |  |  |  | | At5g28500 ,RAFL08-12-K23  expressed protein predicted proteins, Arabidopsis thaliana and Synechocystis sp. | | | | | | |
| 3\_23280001\_23310000 | | |  |  | A | B | C | D | P | P' | N |
|  | Cluster:10-2 | |  |  | 1 | 108 | 1 | 4553 | 0.0462095 | 0.092419 | 2 |
|  |  | RAFL08-11-G23 | At3g63060 / expressed protein | |  |  |  |  |  | | --- | --- | --- | --- | --- | |  |  |  |  |  | | At3g63060 ,RAFL08-11-G23  circadian clock coupling factor, putative similar to gb:AAK56924 circadian clock coupling factor ZGT {Nicotiana tabacum} | | | | | | |
| 1\_9000001\_9300000 | | |  |  | A | B | C | D | P | P' | N |
|  | Cluster:5-1 | |  |  | 3 | 279 | 7 | 4374 | 0.019107534 | 0.114645205 | 6 |
|  |  | RAFL05-20-P12 | At1g26470 / expressed protein | |  |  |  |  |  | | --- | --- | --- | --- | --- | |  |  |  |  |  | | At1g26470 ,RAFL05-20-P12  expressed protein | | | | | | |
|  |  | RAFL04-13-O14 | At1g26270 / phosphatidylinositol 3- and 4-kinase family | |  |  |  |  |  | | --- | --- | --- | --- | --- | |  |  |  |  |  | | At1g26270 ,RAFL04-13-O14  phosphatidylinositol 3- and 4-kinase family protein similar to phosphatidylinositol 4-kinase type-II beta [Homo sapiens] GI:20159767; contains Pfam profile PF00454: Phosphatidylinositol 3- and 4-kinase | | | | | | |
|  |  | RAFL03-09-A18 | At1g26270 / phosphatidylinositol 3- and 4-kinase family | |  |  |  |  |  | | --- | --- | --- | --- | --- | |  |  |  |  |  | | At1g26270 ,RAFL03-09-A18  phosphatidylinositol 3- and 4-kinase family protein similar to phosphatidylinositol 4-kinase type-II beta [Homo sapiens] GI:20159767; contains Pfam profile PF00454: Phosphatidylinositol 3- and 4-kinase | | | | | | |
| 3\_4500001\_4800000 | | |  |  | A | B | C | D | P | P' | N |
|  | Cluster:3-0 | |  |  | 4 | 229 | 15 | 4415 | 0.013005282 | 0.1430581 | 11 |
|  |  | RAFL06-08-N17 | At3g13920 / eukaryotic translation initiation factor 4A-1 (eIF4A-1) | |  |  |  |  |  | | --- | --- | --- | --- | --- | |  |  |  |  |  | | RAFL06-08-N17 ,At3g13920  eukaryotic translation initiation factor 4A-1 / eIF-4A-1 eIF-4A-1 gi:15293046, gi:15450485; contains Pfam profile PF00270: DEAD/DEAH box helicase; contains Pfam profile PF00271: Helicase conserved C-terminal domain | | | | | | |
|  |  | RAFL07-10-J10 | At3g13920 / eukaryotic translation initiation factor 4A-1 (eIF4A-1) | |  |  |  |  |  | | --- | --- | --- | --- | --- | |  |  |  |  |  | | RAFL07-10-J10 ,At3g13920  eukaryotic translation initiation factor 4A-1 / eIF-4A-1 eIF-4A-1 gi:15293046, gi:15450485; contains Pfam profile PF00270: DEAD/DEAH box helicase; contains Pfam profile PF00271: Helicase conserved C-terminal domain | | | | | | |
|  |  | RAFL07-10-F16 | At3g13930 / acetyltransferase -related | |  |  |  |  |  | | --- | --- | --- | --- | --- | |  |  |  |  |  | | At3g13930 ,RAFL07-10-F16  dihydrolipoamide S-acetyltransferase, putative similar to dihydrolipoamide S-acetyltransferase [Zea mays] GI:5669871; contains Pfam profiles PF00198: 2-oxo acid dehydrogenases acyltransferase (catalytic domain), PF00364: Biotin-requiring enzyme, PF02817: e3 binding domain | | | | | | |
|  |  | RAFL04-20-E04 | At3g14100 / oligouridylate binding protein (UBP1), putative | |  |  |  |  |  | | --- | --- | --- | --- | --- | |  |  |  |  |  | | RAFL04-20-E04 ,At3g14100  oligouridylate-binding protein, putative similar to GB:CAB75429 (GI:6996560) from [Nicotiana plumbaginifolia], contains Pfam profiles: PF00076 RNA recognition motif (3 copies) | | | | | | |
|  | Cluster:4-1 | |  |  | 4 | 306 | 15 | 4338 | 0.03352726 | 0.36879984 | 11 |
|  |  | RAFL07-16-E16 | At3g13930 / acetyltransferase -related | |  |  |  |  |  | | --- | --- | --- | --- | --- | |  |  |  |  |  | | RAFL07-16-E16 ,At3g13930  dihydrolipoamide S-acetyltransferase, putative similar to dihydrolipoamide S-acetyltransferase [Zea mays] GI:5669871; contains Pfam profiles PF00198: 2-oxo acid dehydrogenases acyltransferase (catalytic domain), PF00364: Biotin-requiring enzyme, PF02817: e3 binding domain | | | | | | |
|  |  | RAFL04-12-B09 | At3g14290 / 20S proteasome alpha subunit E2 (PAE2) | |  |  |  |  |  | | --- | --- | --- | --- | --- | |  |  |  |  |  | | At3g14290 ,RAFL04-12-B09  20S proteasome alpha subunit E2 (PAE2) identical to 20S proteasome subunit PAE2 GB:AAC32061 from [Arabidopsis thaliana] | | | | | | |
|  |  | RAFL09-12-A19 | At3g13930 / acetyltransferase -related | |  |  |  |  |  | | --- | --- | --- | --- | --- | |  |  |  |  |  | | RAFL09-12-A19 ,At3g13930  dihydrolipoamide S-acetyltransferase, putative similar to dihydrolipoamide S-acetyltransferase [Zea mays] GI:5669871; contains Pfam profiles PF00198: 2-oxo acid dehydrogenases acyltransferase (catalytic domain), PF00364: Biotin-requiring enzyme, PF02817: e3 binding domain | | | | | | |
|  |  | RAFL11-09-K08 | At3g13920 / eukaryotic translation initiation factor 4A-1 (eIF4A-1) | |  |  |  |  |  | | --- | --- | --- | --- | --- | |  |  |  |  |  | | At3g13920 ,RAFL11-09-K08  eukaryotic translation initiation factor 4A-1 / eIF-4A-1 eIF-4A-1 gi:15293046, gi:15450485; contains Pfam profile PF00270: DEAD/DEAH box helicase; contains Pfam profile PF00271: Helicase conserved C-terminal domain | | | | | | |
| 1\_6690001\_6720000 | | |  |  | A | B | C | D | P | P' | N |
|  | Cluster:8-1 | |  |  | 1 | 161 | 0 | 4501 | 0.034741584 | 0.034741584 | 1 |
|  |  | RAFL05-17-D19 | At1g19400 / expressed protein | |  |  |  |  |  | | --- | --- | --- | --- | --- | |  |  |  |  |  | | At1g19400 ,RAFL05-17-D19  expressed protein | | | | | | |
| 3\_1050001\_1080000 | | |  |  | A | B | C | D | P | P' | N |
|  | Cluster:8-0 | |  |  | 1 | 108 | 1 | 4553 | 0.0462095 | 0.092419 | 2 |
|  |  | RAFL07-08-G02 | At3g04080 / apyrase (Atapy1) | |  |  |  |  |  | | --- | --- | --- | --- | --- | |  |  |  |  |  | | At3g04080 ,RAFL07-08-G02  apyrase (APY1) identical to apyrase (Atapy1) GI:6002631 from [Arabidopsis thaliana] | | | | | | |
| 1\_960001\_990000 | | |  |  | A | B | C | D | P | P' | N |
|  | Cluster:3-0 | |  |  | 1 | 232 | 0 | 4430 | 0.049967833 | 0.049967833 | 1 |
|  |  | RAFL05-08-M23 | At1g03860 / prohibitin 2 -related | |  |  |  |  |  | | --- | --- | --- | --- | --- | |  |  |  |  |  | | RAFL05-08-M23 ,At1g03860  prohibitin, putative similar to SP|P24142 Prohibitin (B-cell receptor associated protein 32) (BAP 32) {Rattus norvegicus}; contains Pfam profile PF01145: SPFH domain / Band 7 family | | | | | | |
| 5\_7530001\_7560000 | | |  |  | A | B | C | D | P | P' | N |
|  | Cluster:3-1 | |  |  | 1 | 215 | 0 | 4447 | 0.04632211 | 0.04632211 | 1 |
|  |  | RAFL09-10-K01 | At5g22740 / glycosyltransferase family 2 | |  |  |  |  |  | | --- | --- | --- | --- | --- | |  |  |  |  |  | | At5g22740 ,RAFL09-10-K01  glycosyl transferase family 2 protein similar to beta-(1-3)-glucosyl transferase GB:AAC62210 GI:3687658 from [Bradyrhizobium japonicum], cellulose synthase from Agrobacterium tumeficiens [gi:710492] and Agrobacterium radiobacter [gi:710493]; contains Pfam glycosyl transferase, group 2 family protein domain PF00535 | | | | | | |
| 3\_17310001\_17340000 | | |  |  | A | B | C | D | P | P' | N |
|  | Cluster:8-2 | |  |  | 1 | 61 | 0 | 4601 | 0.013296161 | 0.013296161 | 1 |
|  |  | RAFL08-18-I10 | At3g47000 / glycosyl hydrolase family 3 | |  |  |  |  |  | | --- | --- | --- | --- | --- | |  |  |  |  |  | | At3g47000 ,RAFL08-18-I10  glycosyl hydrolase family 3 protein beta-D-glucan exohydrolase, Nicotiana tabacum, TREMBL:AB017502\_1 | | | | | | |
| 2\_9450001\_9480000 | | |  |  | A | B | C | D | P | P' | N |
|  | Cluster:8-2 | |  |  | 1 | 61 | 0 | 4601 | 0.013296161 | 0.013296161 | 1 |
|  |  | RAFL05-19-N01 | At2g22430 / homeobox-leucine zipper protein ATHB-6 (HD-Zip transcription factor Athb-6) | |  |  |  |  |  | | --- | --- | --- | --- | --- | |  |  |  |  |  | | At2g22430 ,RAFL05-19-N01  homeobox-leucine zipper protein 6 (HB-6) / HD-ZIP transcription factor 6 identical to homeobox-leucine zipper protein ATHB-6 (HD-ZIP protein ATHB-6) (SP:P46668) [Arabidopsis thaliana] | | | | | | |
| 4\_2550001\_2580000 | | |  |  | A | B | C | D | P | P' | N |
|  | Cluster:9-1 | |  |  | 1 | 95 | 0 | 4567 | 0.020587604 | 0.020587604 | 1 |
|  |  | RAFL05-02-M17 | At4g05020 / NADH dehydrogenase - related | |  |  |  |  |  | | --- | --- | --- | --- | --- | |  |  |  |  |  | | At4g05020 ,RAFL05-02-M17  NADH dehydrogenase-related similar to alternative NADH-dehydrogenase [Yarrowia lipolytica] GI:3718005, 64 kDa mitochondrial NADH dehydrogenase [Neurospora crassa] GI:4753821; contains Pfam profile PF00070: Pyridine nucleotide-disulphide oxidoreductase | | | | | | |
| 1\_3150001\_3180000 | | |  |  | A | B | C | D | P | P' | N |
|  | Cluster:5-2 | |  |  | 2 | 124 | 4 | 4533 | 0.0101192035 | 0.050596017 | 5 |
|  |  | RAFL08-12-B13 | At1g09780 / 2,3-bisphosphoglycerate-independent phosphoglycerate mutase -related | |  |  |  |  |  | | --- | --- | --- | --- | --- | |  |  |  |  |  | | RAFL08-12-B13 ,At1g09780  2,3-biphosphoglycerate-independent phosphoglycerate mutase, putative / phosphoglyceromutase, putative strong similarity to SP|Q42908 2,3-bisphosphoglycerate-independent phosphoglycerate mutase (EC 5.4.2.1) (Phosphoglyceromutase) {Mesembryanthemum crystallinum}; contains Pfam profile PF01676: Metalloenzyme superfamily | | | | | | |
|  |  | RAFL08-13-D11 | At1g09780 / 2,3-bisphosphoglycerate-independent phosphoglycerate mutase -related | |  |  |  |  |  | | --- | --- | --- | --- | --- | |  |  |  |  |  | | RAFL08-13-D11 ,At1g09780  2,3-biphosphoglycerate-independent phosphoglycerate mutase, putative / phosphoglyceromutase, putative strong similarity to SP|Q42908 2,3-bisphosphoglycerate-independent phosphoglycerate mutase (EC 5.4.2.1) (Phosphoglyceromutase) {Mesembryanthemum crystallinum}; contains Pfam profile PF01676: Metalloenzyme superfamily | | | | | | |
| 5\_2430001\_2460000 | | |  |  | A | B | C | D | P | P' | N |
|  | Cluster:1-1 | |  |  | 1 | 104 | 0 | 4558 | 0.022517692 | 0.022517692 | 1 |
|  |  | RAFL05-07-L19 | At5g07690 / myb family transcription factor | |  |  |  |  |  | | --- | --- | --- | --- | --- | |  |  |  |  |  | | RAFL05-07-L19 ,At5g07690  myb family transcription factor (MYB29) similar to myb transcription factor GI:3941436 from [Arabidopsis thaliana] | | | | | | |
| 3\_6240001\_6270000 | | |  |  | A | B | C | D | P | P' | N |
|  | Cluster:8-0 | |  |  | 1 | 108 | 1 | 4553 | 0.0462095 | 0.092419 | 2 |
|  |  | RAFL05-04-F21 | At3g18280 / protease inhibitor/seed storage/lipid transfer protein (LTP) family | |  |  |  |  |  | | --- | --- | --- | --- | --- | |  |  |  |  |  | | RAFL05-04-F21 ,At3g18280  protease inhibitor/seed storage/lipid transfer protein (LTP) family protein similar to TED4 [Zinnia elegans] GI:493721; contains Pfam protease inhibitor/seed storage/LTP family domain PF00234 | | | | | | |
| 1\_19800001\_20100000 | | |  |  | A | B | C | D | P | P' | N |
|  | Cluster:0-2 | |  |  | 5 | 74 | 16 | 4568 | 2.0219552E-5 | 2.6285416E-4 | 13 |
|  |  | RAFL04-09-M24 | At1g54500 / rubredoxin -related | |  |  |  |  |  | | --- | --- | --- | --- | --- | |  |  |  |  |  | | At1g54500 ,RAFL04-09-M24  rubredoxin family protein similar to SP|P00270 Rubredoxin (Rd) {Desulfovibrio gigas}; contains Pfam profile PF00301: Rubredoxin | | | | | | |
|  |  | RAFL05-19-G04 | At1g54780 / thylakoid lumen 18.3 kDa protein | |  |  |  |  |  | | --- | --- | --- | --- | --- | |  |  |  |  |  | | RAFL05-19-G04 ,At1g54780  thylakoid lumen 18.3 kDa protein SP:Q9ZVL6 | | | | | | |
|  |  | RAFL05-01-I05 | At1g54780 / thylakoid lumen 18.3 kDa protein | |  |  |  |  |  | | --- | --- | --- | --- | --- | |  |  |  |  |  | | RAFL05-01-I05 ,At1g54780  thylakoid lumen 18.3 kDa protein SP:Q9ZVL6 | | | | | | |
|  |  | RAFL07-14-F21 | At1g54780 / thylakoid lumen 18.3 kDa protein | |  |  |  |  |  | | --- | --- | --- | --- | --- | |  |  |  |  |  | | RAFL07-14-F21 ,At1g54780  thylakoid lumen 18.3 kDa protein SP:Q9ZVL6 | | | | | | |
|  |  | RAFL11-03-K23 | At1g54500 / rubredoxin -related | |  |  |  |  |  | | --- | --- | --- | --- | --- | |  |  |  |  |  | | At1g54500 ,RAFL11-03-K23  rubredoxin family protein similar to SP|P00270 Rubredoxin (Rd) {Desulfovibrio gigas}; contains Pfam profile PF00301: Rubredoxin | | | | | | |
| 4\_16620001\_16650000 | | |  |  | A | B | C | D | P | P' | N |
|  | Cluster:5-2 | |  |  | 1 | 125 | 0 | 4537 | 0.027021231 | 0.027021231 | 1 |
|  |  | RAFL07-13-E01 | At4g37640 / calcium-transporting ATPase 2, plasma membrane-type (Ca2+-ATPase, isoform 2) | |  |  |  |  |  | | --- | --- | --- | --- | --- | |  |  |  |  |  | | RAFL07-13-E01 ,At4g37640  calcium-transporting ATPase 2, plasma membrane-type / Ca(2+)-ATPase isoform 2 (ACA2) identical to SP|O81108 Calcium-transporting ATPase 2, plasma membrane-type (EC 3.6.3.8) (Ca(2+)-ATPase isoform 2) {Arabidopsis thaliana} | | | | | | |
| 1\_29070001\_29100000 | | |  |  | A | B | C | D | P | P' | N |
|  | Cluster:5-2 | |  |  | 1 | 125 | 0 | 4537 | 0.027021231 | 0.027021231 | 1 |
|  |  | RAFL09-16-F04 | At1g78240 / dehydration-induced protein-related | |  |  |  |  |  | | --- | --- | --- | --- | --- | |  |  |  |  |  | | At1g78240 ,RAFL09-16-F04  dehydration-responsive protein-related similar to early-responsive to dehydration stress ERD3 protein [Arabidopsis thaliana] GI:15320410; contains Pfam profile PF03141: Putative methyltransferase | | | | | | |
| 4\_2580001\_2610000 | | |  |  | A | B | C | D | P | P' | N |
|  | Cluster:8-2 | |  |  | 2 | 60 | 0 | 4601 | 1.7397379E-4 | 1.7397379E-4 | 1 |
|  |  | RAFL04-14-B14 | At4g05050 / polyubiquitin UBQ11 | |  |  |  |  |  | | --- | --- | --- | --- | --- | |  |  |  |  |  | | At4g05050 ,RAFL04-14-B14  polyubiquitin (UBQ11) identical to GI:304117 | | | | | | |
|  |  | RAFL06-07-F24 | At4g05050 / polyubiquitin UBQ11 | |  |  |  |  |  | | --- | --- | --- | --- | --- | |  |  |  |  |  | | RAFL06-07-F24 ,At4g05050  polyubiquitin (UBQ11) identical to GI:304117 | | | | | | |
| 3\_2400001\_2700000 | | |  |  | A | B | C | D | P | P' | N |
|  | Cluster:7-0 | |  |  | 3 | 244 | 12 | 4404 | 0.04162972 | 0.4162972 | 10 |
|  |  | RAFL03-04-J10 | At3g07560 / glycine-rich protein | |  |  |  |  |  | | --- | --- | --- | --- | --- | |  |  |  |  |  | | At3g07560 ,RAFL03-04-J10  glycine-rich protein | | | | | | |
|  |  | RAFL05-21-O04 | At3g08860 / alanine--glyoxylate aminotransferase (beta-alanine-pyruvate aminotransferase/AGT), putative | |  |  |  |  |  | | --- | --- | --- | --- | --- | |  |  |  |  |  | | RAFL05-21-O04 ,At3g08860  alanine--glyoxylate aminotransferase, putative / beta-alanine-pyruvate aminotransferase, putative / AGT, putative similar to similar to SP|Q64565 Alanine--glyoxylate aminotransferase 2, mitochondrial precursor (EC 2.6.1.44) (AGT 2) (Beta-alanine-pyruvate aminotransferase) {Rattus norvegicus}; contains Pfam profile PF00202: aminotransferase, class III | | | | | | |
|  |  | RAFL05-09-B20 | At3g08590 / 2,3-bisphosphoglycerate-independent phosphoglycerate mutase -related | |  |  |  |  |  | | --- | --- | --- | --- | --- | |  |  |  |  |  | | RAFL05-09-B20 ,At3g08590  2,3-biphosphoglycerate-independent phosphoglycerate mutase, putative / phosphoglyceromutase, putative strong similarity to SP|Q42908 2,3-bisphosphoglycerate-independent phosphoglycerate mutase (EC 5.4.2.1) (Phosphoglyceromutase) {Mesembryanthemum crystallinum}; contains Pfam profile PF01676: Metalloenzyme superfamily | | | | | | |
| 5\_23970001\_24000000 | | |  |  | A | B | C | D | P | P' | N |
|  | Cluster:8-0 | |  |  | 4 | 105 | 0 | 4554 | 2.827725E-7 | 2.827725E-7 | 1 |
|  |  | RAFL09-18-G19 | At5g60360 / cysteine proteinase AALP | |  |  |  |  |  | | --- | --- | --- | --- | --- | |  |  |  |  |  | | At5g60360 ,RAFL09-18-G19  cysteine proteinase, putative / AALP protein (AALP) identical to AALP protein GI:7230640 from [Arabidopsis thaliana]; similar to barley aleurain | | | | | | |
|  |  | RAFL05-19-H13 | At5g60360 / cysteine proteinase AALP | |  |  |  |  |  | | --- | --- | --- | --- | --- | |  |  |  |  |  | | At5g60360 ,RAFL05-19-H13  cysteine proteinase, putative / AALP protein (AALP) identical to AALP protein GI:7230640 from [Arabidopsis thaliana]; similar to barley aleurain | | | | | | |
|  |  | RAFL08-12-G17 | At5g60360 / cysteine proteinase AALP | |  |  |  |  |  | | --- | --- | --- | --- | --- | |  |  |  |  |  | | At5g60360 ,RAFL08-12-G17  cysteine proteinase, putative / AALP protein (AALP) identical to AALP protein GI:7230640 from [Arabidopsis thaliana]; similar to barley aleurain | | | | | | |
|  |  | RAFL07-10-L02 | At5g60360 / cysteine proteinase AALP | |  |  |  |  |  | | --- | --- | --- | --- | --- | |  |  |  |  |  | | At5g60360 ,RAFL07-10-L02  cysteine proteinase, putative / AALP protein (AALP) identical to AALP protein GI:7230640 from [Arabidopsis thaliana]; similar to barley aleurain | | | | | | |
| 2\_18810001\_18840000 | | |  |  | A | B | C | D | P | P' | N |
|  | Cluster:8-2 | |  |  | 1 | 61 | 0 | 4601 | 0.013296161 | 0.013296161 | 1 |
[truncated: 2,282,727 more chars]
