# Supplementary material for: A flexible representation of omic knowledge for thorough analysis of microarray data
Source: Plant Methods. 2006 Mar 2;2:5. doi: 10.1186/1746-4811-2-5 (PMC1421397; doi:10.1186/1746-4811-2-5)
Supplement: Additional File 5 — Supplementary Table 5. Ranking result of significant correlations between the "functional Class" of the "Protein-protein interaction and Arabidopsis thaliana gene expression" and the clusters formed by BL-SOM of the microarray probes of expression profile under drought conditions [file 1746-4811-2-5-S5.HTML]

|  |  |  |  |  |  |  |  |  |  |  |  |
| --- | --- | --- | --- | --- | --- | --- | --- | --- | --- | --- | --- |
| Date: | | 2005/06/24 | | | | | | | | | |
| Method: | | Fisher test | | | | | | | | | |
| Cut off P-value: | | 0.05 | | | | | | | | | |
| Target dataset(s): | | BinominalRelations\_of\_WI5 PPI\_WI5 | | | | | | | | | |
| Query dataset(s): | | SOM Cluster | | | | | | | | | |
  | | | | | | | | | | | || F55A11.2 | | | WI5\_id:F55A11.2 |  | A | B | C | D | P | P' | N |
|  | Cluster:10-2 | |  |  | 1 | 108 | 0 | 4554 | 0.02337551 | 0.02337551 | 1 |
|  |  | RAFL11-12-H18 | At4g17530 / ras-related small GTP-binding protein RAB1c | |  |  |  |  |  | | --- | --- | --- | --- | --- | |  |  |  |  |  | | C39F7.4 | | | | | | |
| Y49E10.6 | | | WI5\_id:Y49E10.6 |  | A | B | C | D | P | P' | N |
|  | Cluster:2-0 | |  |  | 1 | 149 | 0 | 4513 | 0.03216813 | 0.03216813 | 1 |
|  |  | RAFL05-02-F20 | At5g65360 / histone H3 | |  |  |  |  |  | | --- | --- | --- | --- | --- | |  |  |  |  |  | | Y49E10.6 | | | | | | |
| F59A2.3 | | | WI5\_id:F59A2.3 |  | A | B | C | D | P | P' | N |
|  | Cluster:2-0 | |  |  | 1 | 149 | 0 | 4513 | 0.03216813 | 0.03216813 | 1 |
|  |  | RAFL08-12-D04 | At5g20290 / 40S ribosomal protein S8 (RPS8A) | |  |  |  |  |  | | --- | --- | --- | --- | --- | |  |  |  |  |  | | F42C5.8 | | | | | | |
| M01F1.2 | | | WI5\_id:M01F1.2 |  | A | B | C | D | P | P' | N |
|  | Cluster:2-0 | |  |  | 3 | 147 | 9 | 4504 | 0.0057971687 | 0.03478301 | 6 |
|  |  | RAFL04-19-O24 | At5g02610 / 60S ribosomal protein L35 (RPL35D) | |  |  |  |  |  | | --- | --- | --- | --- | --- | |  |  |  |  |  | | ZK652.4 | | | | | | |
|  |  | RAFL11-12-M17 | At3g25520 / 60S ribosomal protein L5 (RPL5A) | |  |  |  |  |  | | --- | --- | --- | --- | --- | |  |  |  |  |  | | F54C9.5 | | | | | | |
|  |  | RAFL05-01-F21 | At3g05590 / 60S ribosomal protein L18 (RPL18B) | |  |  |  |  |  | | --- | --- | --- | --- | --- | |  |  |  |  |  | | Y45F10D.12  Eukaryotic ribosomal protein L18 | | | | | | |
|  | Cluster:3-0 | |  |  | 3 | 230 | 9 | 4421 | 0.019373124 | 0.11623875 | 6 |
|  |  | RAFL07-10-D02 | At1g14320 / 60S ribosomal protein L10 (RPL10A)/Wilm's tumor suppressor protein-related | |  |  |  |  |  | | --- | --- | --- | --- | --- | |  |  |  |  |  | | F10B5.1 | | | | | | |
|  |  | RAFL09-10-P09 | At4g36130 / 60S ribosomal protein L8 (RPL8C) | |  |  |  |  |  | | --- | --- | --- | --- | --- | |  |  |  |  |  | | B0250.1 | | | | | | |
|  |  | RAFL09-12-B12 | At4g36130 / 60S ribosomal protein L8 (RPL8C) | |  |  |  |  |  | | --- | --- | --- | --- | --- | |  |  |  |  |  | | B0250.1 | | | | | | |
|  | Cluster:2-1 | |  |  | 3 | 241 | 9 | 4410 | 0.021903817 | 0.13142289 | 6 |
|  |  | RAFL05-17-L17 | At3g55280 / 60S ribosomal protein L23A (RPL23aB) | |  |  |  |  |  | | --- | --- | --- | --- | --- | |  |  |  |  |  | | F52B5.6 | | | | | | |
|  |  | RAFL11-12-H04 | At3g25520 / 60S ribosomal protein L5 (RPL5A) | |  |  |  |  |  | | --- | --- | --- | --- | --- | |  |  |  |  |  | | F54C9.5 | | | | | | |
|  |  | RAFL11-07-B21 | At3g25520 / 60S ribosomal protein L5 (RPL5A) | |  |  |  |  |  | | --- | --- | --- | --- | --- | |  |  |  |  |  | | F54C9.5 | | | | | | |
| Y24F12A.2 | | | WI5\_id:Y24F12A.2 |  | A | B | C | D | P | P' | N |
|  | Cluster:4-0 | |  |  | 2 | 238 | 1 | 4422 | 0.0076462985 | 0.015292597 | 2 |
|  |  | RAFL06-07-B07 | At5g55190 / GTP-binding protein atran3, putative | |  |  |  |  |  | | --- | --- | --- | --- | --- | |  |  |  |  |  | | K01G5.4 | | | | | | |
|  |  | RAFL05-17-C16 | At5g55190 / GTP-binding protein atran3, putative | |  |  |  |  |  | | --- | --- | --- | --- | --- | |  |  |  |  |  | | K01G5.4 | | | | | | |
| B0464.5 | | | WI5\_id:B0464.5 |  | A | B | C | D | P | P' | N |
|  | Cluster:5-0 | |  |  | 4 | 73 | 2 | 4584 | 1.0060772E-6 | 3.0182316E-6 | 3 |
|  |  | RAFL04-15-M13 | At5g56030 / heat shock protein 81-2 (HSP81-2) | |  |  |  |  |  | | --- | --- | --- | --- | --- | |  |  |  |  |  | | C47E8.5 | | | | | | |
|  |  | RAFL07-13-H08 | At5g56010 / heat shock protein, putative | |  |  |  |  |  | | --- | --- | --- | --- | --- | |  |  |  |  |  | | C47E8.5 | | | | | | |
|  |  | RAFL09-06-O18 | At5g56030 / heat shock protein 81-2 (HSP81-2) | |  |  |  |  |  | | --- | --- | --- | --- | --- | |  |  |  |  |  | | C47E8.5 | | | | | | |
|  |  | RAFL05-16-L15 | At5g56010 / heat shock protein, putative | |  |  |  |  |  | | --- | --- | --- | --- | --- | |  |  |  |  |  | | C47E8.5 | | | | | | |
| Y63D3A.4 | | | WI5\_id:Y63D3A.4 |  | A | B | C | D | P | P' | N |
|  | Cluster:5-0 | |  |  | 4 | 73 | 3 | 4583 | 2.3181085E-6 | 9.272434E-6 | 4 |
|  |  | RAFL04-15-M13 | At5g56030 / heat shock protein 81-2 (HSP81-2) | |  |  |  |  |  | | --- | --- | --- | --- | --- | |  |  |  |  |  | | C47E8.5 | | | | | | |
|  |  | RAFL07-13-H08 | At5g56010 / heat shock protein, putative | |  |  |  |  |  | | --- | --- | --- | --- | --- | |  |  |  |  |  | | C47E8.5 | | | | | | |
|  |  | RAFL09-06-O18 | At5g56030 / heat shock protein 81-2 (HSP81-2) | |  |  |  |  |  | | --- | --- | --- | --- | --- | |  |  |  |  |  | | C47E8.5 | | | | | | |
|  |  | RAFL05-16-L15 | At5g56010 / heat shock protein, putative | |  |  |  |  |  | | --- | --- | --- | --- | --- | |  |  |  |  |  | | C47E8.5 | | | | | | |
| C56E6.3 | | | WI5\_id:C56E6.3 |  | A | B | C | D | P | P' | N |
|  | Cluster:4-0 | |  |  | 2 | 238 | 1 | 4422 | 0.0076462985 | 0.015292597 | 2 |
|  |  | RAFL06-07-B07 | At5g55190 / GTP-binding protein atran3, putative | |  |  |  |  |  | | --- | --- | --- | --- | --- | |  |  |  |  |  | | K01G5.4 | | | | | | |
|  |  | RAFL05-17-C16 | At5g55190 / GTP-binding protein atran3, putative | |  |  |  |  |  | | --- | --- | --- | --- | --- | |  |  |  |  |  | | K01G5.4 | | | | | | |
| C47B2.4 | | | WI5\_id:C47B2.4 |  | A | B | C | D | P | P' | N |
|  | Cluster:4-1 | |  |  | 2 | 308 | 2 | 4351 | 0.024166461 | 0.07249938 | 3 |
|  |  | RAFL05-04-G23 | At1g77440 / 20S proteasome beta subunit C (PBC2) | |  |  |  |  |  | | --- | --- | --- | --- | --- | |  |  |  |  |  | | Y38A8.2 | | | | | | |
|  |  | RAFL05-21-D23 | At1g21720 / 20S proteasome beta subunit C (PBC1) | |  |  |  |  |  | | --- | --- | --- | --- | --- | |  |  |  |  |  | | Y38A8.2 | | | | | | |
| C39F7.4 | | | WI5\_id:C39F7.4 |  | A | B | C | D | P | P' | N |
|  | Cluster:4-0 | |  |  | 2 | 238 | 2 | 4421 | 0.0147742275 | 0.044322684 | 3 |
|  |  | RAFL06-07-B07 | At5g55190 / GTP-binding protein atran3, putative | |  |  |  |  |  | | --- | --- | --- | --- | --- | |  |  |  |  |  | | K01G5.4 | | | | | | |
|  |  | RAFL05-17-C16 | At5g55190 / GTP-binding protein atran3, putative | |  |  |  |  |  | | --- | --- | --- | --- | --- | |  |  |  |  |  | | K01G5.4 | | | | | | |
| F22B7.13 | | | WI5\_id:F22B7.13 |  | A | B | C | D | P | P' | N |
|  | Cluster:1-2 | |  |  | 1 | 173 | 0 | 4489 | 0.037315033 | 0.037315033 | 1 |
|  |  | RAFL06-13-M02 | At1g13440 / glyceraldehyde-3-phosphate dehydrogenase -related | |  |  |  |  |  | | --- | --- | --- | --- | --- | |  |  |  |  |  | | F33H1.2  glyceraldehyde 3-phosphate dehydrogenase 4 | | | | | | |
| T09F3.3 | | | WI5\_id:T09F3.3 |  | A | B | C | D | P | P' | N |
|  | Cluster:3-0 | |  |  | 1 | 232 | 0 | 4430 | 0.049967833 | 0.049967833 | 1 |
|  |  | RAFL06-14-K01 | At3g04120 / glyceraldehyde-3-phosphate dehydrogenase C subunit (GapC) | |  |  |  |  |  | | --- | --- | --- | --- | --- | |  |  |  |  |  | | T09F3.3 | | | | | | |
| T13F2.8 | | | WI5\_id:T13F2.8 |  | A | B | C | D | P | P' | N |
|  | Cluster:1-2 | |  |  | 1 | 173 | 0 | 4489 | 0.037315033 | 0.037315033 | 1 |
|  |  | RAFL11-02-K03 | At5g18230 / expressed protein | |  |  |  |  |  | | --- | --- | --- | --- | --- | |  |  |  |  |  | | Y56A3A.1 | | | | | | |
| JC8.3 | | | WI5\_id:JC8.3 |  | A | B | C | D | P | P' | N |
|  | Cluster:3-0 | |  |  | 3 | 230 | 8 | 4422 | 0.015076893 | 0.09046136 | 6 |
|  |  | RAFL07-10-D02 | At1g14320 / 60S ribosomal protein L10 (RPL10A)/Wilm's tumor suppressor protein-related | |  |  |  |  |  | | --- | --- | --- | --- | --- | |  |  |  |  |  | | F10B5.1 | | | | | | |
|  |  | RAFL09-10-P09 | At4g36130 / 60S ribosomal protein L8 (RPL8C) | |  |  |  |  |  | | --- | --- | --- | --- | --- | |  |  |  |  |  | | B0250.1 | | | | | | |
|  |  | RAFL09-12-B12 | At4g36130 / 60S ribosomal protein L8 (RPL8C) | |  |  |  |  |  | | --- | --- | --- | --- | --- | |  |  |  |  |  | | B0250.1 | | | | | | |
|  | Cluster:2-1 | |  |  | 3 | 241 | 8 | 4411 | 0.017076377 | 0.10245825 | 6 |
|  |  | RAFL05-17-L17 | At3g55280 / 60S ribosomal protein L23A (RPL23aB) | |  |  |  |  |  | | --- | --- | --- | --- | --- | |  |  |  |  |  | | F52B5.6 | | | | | | |
|  |  | RAFL11-12-H04 | At3g25520 / 60S ribosomal protein L5 (RPL5A) | |  |  |  |  |  | | --- | --- | --- | --- | --- | |  |  |  |  |  | | F54C9.5 | | | | | | |
|  |  | RAFL11-07-B21 | At3g25520 / 60S ribosomal protein L5 (RPL5A) | |  |  |  |  |  | | --- | --- | --- | --- | --- | |  |  |  |  |  | | F54C9.5 | | | | | | |
|  | Cluster:2-0 | |  |  | 2 | 148 | 9 | 4504 | 0.046735216 | 0.2804113 | 6 |
|  |  | RAFL04-19-O24 | At5g02610 / 60S ribosomal protein L35 (RPL35D) | |  |  |  |  |  | | --- | --- | --- | --- | --- | |  |  |  |  |  | | ZK652.4 | | | | | | |
|  |  | RAFL11-12-M17 | At3g25520 / 60S ribosomal protein L5 (RPL5A) | |  |  |  |  |  | | --- | --- | --- | --- | --- | |  |  |  |  |  | | F54C9.5 | | | | | | |
| C14B9.7 | | | WI5\_id:C14B9.7 |  | A | B | C | D | P | P' | N |
|  | Cluster:1-2 | |  |  | 1 | 173 | 0 | 4489 | 0.037315033 | 0.037315033 | 1 |
|  |  | RAFL06-11-J01 | At1g09690 / 60S ribosomal protein L21 (RPL21C) | |  |  |  |  |  | | --- | --- | --- | --- | --- | |  |  |  |  |  | | C14B9.7 | | | | | | |
| K08E3.7 | | | WI5\_id:K08E3.7 |  | A | B | C | D | P | P' | N |
|  | Cluster:1-0 | |  |  | 2 | 147 | 7 | 4507 | 0.031507306 | 0.15753652 | 5 |
|  |  | RAFL09-11-P11 | At1g20010 / tubulin beta-5 chain (TUB5) | |  |  |  |  |  | | --- | --- | --- | --- | --- | |  |  |  |  |  | | C36E8.5  beta tubulin | | | | | | |
|  |  | RAFL04-16-G05 | At5g44340 / tubulin beta-4 chain (TUB4) | |  |  |  |  |  | | --- | --- | --- | --- | --- | |  |  |  |  |  | | C36E8.5  beta tubulin | | | | | | |
|  | Cluster:2-0 | |  |  | 2 | 148 | 7 | 4506 | 0.031900965 | 0.15950483 | 5 |
|  |  | RAFL08-12-D04 | At5g20290 / 40S ribosomal protein S8 (RPS8A) | |  |  |  |  |  | | --- | --- | --- | --- | --- | |  |  |  |  |  | | F42C5.8 | | | | | | |
|  |  | RAFL04-19-F02 | At4g20890 / tubulin beta-9 chain (TUB9) | |  |  |  |  |  | | --- | --- | --- | --- | --- | |  |  |  |  |  | | C36E8.5  beta tubulin | | | | | | |
| Y82E9BR.3 | | | WI5\_id:Y82E9BR.3 |  | A | B | C | D | P | P' | N |
|  | Cluster:4-1 | |  |  | 2 | 308 | 2 | 4351 | 0.024166461 | 0.07249938 | 3 |
|  |  | RAFL04-15-D14 | At5g08680 / H+-transporting ATP synthase beta chain -related | |  |  |  |  |  | | --- | --- | --- | --- | --- | |  |  |  |  |  | | C34E10.6 | | | | | | |
|  |  | RAFL09-17-J21 | At5g08690 / H+-transporting ATP synthase beta chain (mitochondrial) -related | |  |  |  |  |  | | --- | --- | --- | --- | --- | |  |  |  |  |  | | C34E10.6 | | | | | | |
| F38B7.2 | | | WI5\_id:F38B7.2 |  | A | B | C | D | P | P' | N |
|  | Cluster:4-2 | |  |  | 1 | 134 | 0 | 4528 | 0.028951319 | 0.028951319 | 1 |
|  |  | RAFL05-05-K13 | At3g52590 / ubiquitin extension protein 1 (UBQ1)/60S ribosomal protein L40 (RPL40B) | |  |  |  |  |  | | --- | --- | --- | --- | --- | |  |  |  |  |  | | ZK1010.1 | | | | | | |
| Y56A3A.1 | | | WI5\_id:Y56A3A.1 |  | A | B | C | D | P | P' | N |
|  | Cluster:1-2 | |  |  | 1 | 173 | 0 | 4489 | 0.037315033 | 0.037315033 | 1 |
|  |  | RAFL11-02-K03 | At5g18230 / expressed protein | |  |  |  |  |  | | --- | --- | --- | --- | --- | |  |  |  |  |  | | Y56A3A.1 | | | | | | |
| B0205.3 | | | WI5\_id:B0205.3 |  | A | B | C | D | P | P' | N |
|  | Cluster:2-1 | |  |  | 5 | 239 | 18 | 4401 | 0.0058271554 | 0.08158018 | 14 |
|  |  | RAFL02-10-A09 | At1g33140 / 60S ribosomal protein L9 (RPL90A/C) | |  |  |  |  |  | | --- | --- | --- | --- | --- | |  |  |  |  |  | | R13A5.8 | | | | | | |
|  |  | RAFL07-15-M07 | At1g04480 / 60S ribosomal protein L23 (RPL23A) | |  |  |  |  |  | | --- | --- | --- | --- | --- | |  |  |  |  |  | | B0336.10 | | | | | | |
|  |  | RAFL02-04-I03 | At5g58290 / 26S proteasome AAA-ATPase subunit RPT3 | |  |  |  |  |  | | --- | --- | --- | --- | --- | |  |  |  |  |  | | F23F12.6 | | | | | | |
|  |  | RAFL05-18-P15 | At1g04480 / 60S ribosomal protein L23 (RPL23A) | |  |  |  |  |  | | --- | --- | --- | --- | --- | |  |  |  |  |  | | B0336.10 | | | | | | |
|  |  | RAFL04-18-N22 | At2g44120 / 60S ribosomal protein L7 (RPL7C) | |  |  |  |  |  | | --- | --- | --- | --- | --- | |  |  |  |  |  | | F53G12.10 | | | | | | |
| C49H3.11 | | | WI5\_id:C49H3.11 |  | A | B | C | D | P | P' | N |
|  | Cluster:2-1 | |  |  | 8 | 236 | 13 | 4406 | 5.6352947E-6 | 3.3811768E-5 | 6 |
|  |  | RAFL04-18-N10 | At1g07770 / 40S ribosomal protein S15A (RPS15aA) | |  |  |  |  |  | | --- | --- | --- | --- | --- | |  |  |  |  |  | | F53A3.3 | | | | | | |
|  |  | RAFL09-15-M15 | At5g23740 / 40S ribosomal protein S11 (RPS11C) | |  |  |  |  |  | | --- | --- | --- | --- | --- | |  |  |  |  |  | | F40F11.1 | | | | | | |
|  |  | RAFL05-05-M24 | At5g02960 / 40S ribosomal protein S23 (RPS23B) | |  |  |  |  |  | | --- | --- | --- | --- | --- | |  |  |  |  |  | | F28D1.7 | | | | | | |
|  |  | RAFL07-13-J18 | At2g37270 / 40S ribosomal protein S5 (RPS5A) | |  |  |  |  |  | | --- | --- | --- | --- | --- | |  |  |  |  |  | | T05E11.1 | | | | | | |
|  |  | RAFL06-09-H09 | At3g48930 / 40S ribosomal protein S11 (RPS11A) | |  |  |  |  |  | | --- | --- | --- | --- | --- | |  |  |  |  |  | | F40F11.1 | | | | | | |
|  |  | RAFL06-08-B09 | At3g11510 / 40S ribosomal protein S14 (RPS14B) | |  |  |  |  |  | | --- | --- | --- | --- | --- | |  |  |  |  |  | | F37C12.9 | | | | | | |
|  |  | RAFL05-16-H14 | At1g04270 / 40S ribosomal protein S15 (RPS15A) | |  |  |  |  |  | | --- | --- | --- | --- | --- | |  |  |  |  |  | | F36A2.6 | | | | | | |
|  |  | RAFL04-19-M20 | At1g58380 / 40S ribosomal protein S2 (RPS2A) | |  |  |  |  |  | | --- | --- | --- | --- | --- | |  |  |  |  |  | | C49H3.11 | | | | | | |
|  | Cluster:4-2 | |  |  | 4 | 131 | 17 | 4511 | 0.002741287 | 0.016447721 | 6 |
|  |  | RAFL05-07-M02 | At1g34030 / 40S ribosomal protein S18 (RPS18B) | |  |  |  |  |  | | --- | --- | --- | --- | --- | |  |  |  |  |  | | Y57G11C.16 | | | | | | |
|  |  | RAFL09-17-F16 | At1g07770 / 40S ribosomal protein S15A (RPS15aA) | |  |  |  |  |  | | --- | --- | --- | --- | --- | |  |  |  |  |  | | F53A3.3 | | | | | | |
|  |  | RAFL11-02-D23 | At1g07770 / 40S ribosomal protein S15A (RPS15aA) | |  |  |  |  |  | | --- | --- | --- | --- | --- | |  |  |  |  |  | | F53A3.3 | | | | | | |
|  |  | RAFL05-08-B08 | At5g18380 / 40S ribosomal protein S16 (RPS16C) | |  |  |  |  |  | | --- | --- | --- | --- | --- | |  |  |  |  |  | | T01C3.6 | | | | | | |
|  | Cluster:3-0 | |  |  | 4 | 229 | 17 | 4413 | 0.01857225 | 0.1114335 | 6 |
|  |  | RAFL02-10-H10 | At3g43980 / 40S ribosomal protein S29 (RPS29A) | |  |  |  |  |  | | --- | --- | --- | --- | --- | |  |  |  |  |  | | B0412.4 | | | | | | |
|  |  | RAFL08-09-E20 | At2g41840 / 40S ribosomal protein S2 (RPS2C) | |  |  |  |  |  | | --- | --- | --- | --- | --- | |  |  |  |  |  | | C49H3.11 | | | | | | |
|  |  | RAFL11-04-A02 | At1g07770 / 40S ribosomal protein S15A (RPS15aA) | |  |  |  |  |  | | --- | --- | --- | --- | --- | |  |  |  |  |  | | F53A3.3 | | | | | | |
|  |  | RAFL06-07-B02 | At3g11940 / 40S ribosomal protein S5 (RPS5B) | |  |  |  |  |  | | --- | --- | --- | --- | --- | |  |  |  |  |  | | T05E11.1 | | | | | | |
| C35B1.1 | | | WI5\_id:C35B1.1 |  | A | B | C | D | P | P' | N |
|  | Cluster:0-2 | |  |  | 1 | 78 | 1 | 4583 | 0.03360031 | 0.06720062 | 2 |
|  |  | RAFL04-17-G02 | At1g56340 / calreticulin 1 (CRT1) | |  |  |  |  |  | | --- | --- | --- | --- | --- | |  |  |  |  |  | | Y38A10A.5 | | | | | | |
| F29F11.1 | | | WI5\_id:F29F11.1 |  | A | B | C | D | P | P' | N |
|  | Cluster:5-2 | |  |  | 1 | 125 | 0 | 4537 | 0.027021231 | 0.027021231 | 1 |
|  |  | RAFL07-09-M15 | At3g29360 / UDP-glucose dehydrogenase -related | |  |  |  |  |  | | --- | --- | --- | --- | --- | |  |  |  |  |  | | F29F11.1 | | | | | | |
| R07E3.7 | | | WI5\_id:R07E3.7 |  | A | B | C | D | P | P' | N |
|  | Cluster:5-2 | |  |  | 1 | 125 | 0 | 4537 | 0.027021231 | 0.027021231 | 1 |
|  |  | RAFL07-09-M15 | At3g29360 / UDP-glucose dehydrogenase -related | |  |  |  |  |  | | --- | --- | --- | --- | --- | |  |  |  |  |  | | F29F11.1 | | | | | | |
| T04C10.1 | | | WI5\_id:T04C10.1 |  | A | B | C | D | P | P' | N |
|  | Cluster:8-1 | |  |  | 1 | 161 | 0 | 4501 | 0.034741584 | 0.034741584 | 1 |
|  |  | RAFL08-12-L24 | At3g08720 / ribosomal-protein S6 kinase (ATPK19) -related | |  |  |  |  |  | | --- | --- | --- | --- | --- | |  |  |  |  |  | | T04C10.1 | | | | | | |
| K10H10.2 | | | WI5\_id:K10H10.2 |  | A | B | C | D | P | P' | N |
|  | Cluster:8-0 | |  |  | 1 | 108 | 1 | 4553 | 0.0462095 | 0.092419 | 2 |
|  |  | RAFL09-13-J20 | At1g69410 / Eukaryotic initiation factor 5A -related | |  |  |  |  |  | | --- | --- | --- | --- | --- | |  |  |  |  |  | | T05G5.10  Initiation factor 5A | | | | | | |
| T28A8.7 | | | WI5\_id:T28A8.7 |  | A | B | C | D | P | P' | N |
|  | Cluster:2-1 | |  |  | 2 | 242 | 1 | 4418 | 0.00789916 | 0.01579832 | 2 |
|  |  | RAFL07-15-M07 | At1g04480 / 60S ribosomal protein L23 (RPL23A) | |  |  |  |  |  | | --- | --- | --- | --- | --- | |  |  |  |  |  | | B0336.10 | | | | | | |
|  |  | RAFL05-18-P15 | At1g04480 / 60S ribosomal protein L23 (RPL23A) | |  |  |  |  |  | | --- | --- | --- | --- | --- | |  |  |  |  |  | | B0336.10 | | | | | | |
| R10E11.2 | | | WI5\_id:R10E11.2 |  | A | B | C | D | P | P' | N |
|  | Cluster:2-0 | |  |  | 2 | 148 | 2 | 4511 | 0.00591059 | 0.01182118 | 2 |
|  |  | RAFL03-08-O03 | At1g07940 / elongation factor 1-alpha (EF-1-alpha) | |  |  |  |  |  | | --- | --- | --- | --- | --- | |  |  |  |  |  | | F31E3.5 | | | | | | |
|  |  | RAFL03-06-H07 | At1g33140 / 60S ribosomal protein L9 (RPL90A/C) | |  |  |  |  |  | | --- | --- | --- | --- | --- | |  |  |  |  |  | | R13A5.8 | | | | | | |
|  | Cluster:2-1 | |  |  | 2 | 242 | 2 | 4417 | 0.01525385 | 0.0305077 | 2 |
|  |  | RAFL02-10-A09 | At1g33140 / 60S ribosomal protein L9 (RPL90A/C) | |  |  |  |  |  | | --- | --- | --- | --- | --- | |  |  |  |  |  | | R13A5.8 | | | | | | |
|  |  | RAFL04-18-N22 | At2g44120 / 60S ribosomal protein L7 (RPL7C) | |  |  |  |  |  | | --- | --- | --- | --- | --- | |  |  |  |  |  | | F53G12.10 | | | | | | |
| W06F12.1 | | | WI5\_id:W06F12.1 |  | A | B | C | D | P | P' | N |
|  | Cluster:1-0 | |  |  | 2 | 147 | 7 | 4507 | 0.031507306 | 0.22055113 | 7 |
|  |  | RAFL09-11-P11 | At1g20010 / tubulin beta-5 chain (TUB5) | |  |  |  |  |  | | --- | --- | --- | --- | --- | |  |  |  |  |  | | K01G5.7  tubulin beta-chain | | | | | | |
|  |  | RAFL04-16-G05 | At5g44340 / tubulin beta-4 chain (TUB4) | |  |  |  |  |  | | --- | --- | --- | --- | --- | |  |  |  |  |  | | K01G5.7  tubulin beta-chain | | | | | | |
| K06B4.1 | | | WI5\_id:K06B4.1 |  | A | B | C | D | P | P' | N |
|  | Cluster:5-0 | |  |  | 4 | 73 | 3 | 4583 | 2.3181085E-6 | 9.272434E-6 | 4 |
|  |  | RAFL04-15-M13 | At5g56030 / heat shock protein 81-2 (HSP81-2) | |  |  |  |  |  | | --- | --- | --- | --- | --- | |  |  |  |  |  | | C47E8.5 | | | | | | |
|  |  | RAFL07-13-H08 | At5g56010 / heat shock protein, putative | |  |  |  |  |  | | --- | --- | --- | --- | --- | |  |  |  |  |  | | C47E8.5 | | | | | | |
|  |  | RAFL09-06-O18 | At5g56030 / heat shock protein 81-2 (HSP81-2) | |  |  |  |  |  | | --- | --- | --- | --- | --- | |  |  |  |  |  | | C47E8.5 | | | | | | |
|  |  | RAFL05-16-L15 | At5g56010 / heat shock protein, putative | |  |  |  |  |  | | --- | --- | --- | --- | --- | |  |  |  |  |  | | C47E8.5 | | | | | | |
| C05D9.1 | | | WI5\_id:C05D9.1 |  | A | B | C | D | P | P' | N |
|  | Cluster:4-1 | |  |  | 2 | 308 | 0 | 4353 | 0.0044063856 | 0.0044063856 | 1 |
|  |  | RAFL05-04-G23 | At1g77440 / 20S proteasome beta subunit C (PBC2) | |  |  |  |  |  | | --- | --- | --- | --- | --- | |  |  |  |  |  | | Y38A8.2 | | | | | | |
|  |  | RAFL05-21-D23 | At1g21720 / 20S proteasome beta subunit C (PBC1) | |  |  |  |  |  | | --- | --- | --- | --- | --- | |  |  |  |  |  | | Y38A8.2 | | | | | | |
| F40F11.1 | | | WI5\_id:F40F11.1 |  | A | B | C | D | P | P' | N |
|  | Cluster:2-1 | |  |  | 5 | 239 | 6 | 4413 | 1.3419612E-4 | 5.367845E-4 | 4 |
|  |  | RAFL09-15-M15 | At5g23740 / 40S ribosomal protein S11 (RPS11C) | |  |  |  |  |  | | --- | --- | --- | --- | --- | |  |  |  |  |  | | F40F11.1 | | | | | | |
|  |  | RAFL07-13-J18 | At2g37270 / 40S ribosomal protein S5 (RPS5A) | |  |  |  |  |  | | --- | --- | --- | --- | --- | |  |  |  |  |  | | T05E11.1 | | | | | | |
|  |  | RAFL06-09-H09 | At3g48930 / 40S ribosomal protein S11 (RPS11A) | |  |  |  |  |  | | --- | --- | --- | --- | --- | |  |  |  |  |  | | F40F11.1 | | | | | | |
|  |  | RAFL05-16-H14 | At1g04270 / 40S ribosomal protein S15 (RPS15A) | |  |  |  |  |  | | --- | --- | --- | --- | --- | |  |  |  |  |  | | F36A2.6 | | | | | | |
|  |  | RAFL04-19-M20 | At1g58380 / 40S ribosomal protein S2 (RPS2A) | |  |  |  |  |  | | --- | --- | --- | --- | --- | |  |  |  |  |  | | C49H3.11 | | | | | | |
|  | Cluster:4-0 | |  |  | 3 | 237 | 8 | 4415 | 0.016332459 | 0.065329835 | 4 |
|  |  | RAFL06-07-B07 | At5g55190 / GTP-binding protein atran3, putative | |  |  |  |  |  | | --- | --- | --- | --- | --- | |  |  |  |  |  | | K01G5.4 | | | | | | |
|  |  | RAFL05-17-C16 | At5g55190 / GTP-binding protein atran3, putative | |  |  |  |  |  | | --- | --- | --- | --- | --- | |  |  |  |  |  | | K01G5.4 | | | | | | |
|  |  | RAFL07-07-O08 | At4g30800 / 40S ribosomal protein S11 (RPS11B) | |  |  |  |  |  | | --- | --- | --- | --- | --- | |  |  |  |  |  | | F40F11.1 | | | | | | |
| C07E3.7 | | | WI5\_id:C07E3.7 |  | A | B | C | D | P | P' | N |
|  | Cluster:2-0 | |  |  | 1 | 149 | 0 | 4513 | 0.03216813 | 0.03216813 | 1 |
|  |  | RAFL05-01-F21 | At3g05590 / 60S ribosomal protein L18 (RPL18B) | |  |  |  |  |  | | --- | --- | --- | --- | --- | |  |  |  |  |  | | Y45F10D.12 | | | | | | |
| F28D1.7 | | | WI5\_id:F28D1.7 |  | A | B | C | D | P | P' | N |
|  | Cluster:2-1 | |  |  | 4 | 240 | 2 | 4417 | 1.0099356E-4 | 2.0198712E-4 | 2 |
|  |  | RAFL05-05-M24 | At5g02960 / 40S ribosomal protein S23 (RPS23B) | |  |  |  |  |  | | --- | --- | --- | --- | --- | |  |  |  |  |  | | F28D1.7 | | | | | | |
|  |  | RAFL07-13-J18 | At2g37270 / 40S ribosomal protein S5 (RPS5A) | |  |  |  |  |  | | --- | --- | --- | --- | --- | |  |  |  |  |  | | T05E11.1 | | | | | | |
|  |  | RAFL05-16-H14 | At1g04270 / 40S ribosomal protein S15 (RPS15A) | |  |  |  |  |  | | --- | --- | --- | --- | --- | |  |  |  |  |  | | F36A2.6 | | | | | | |
|  |  | RAFL04-19-M20 | At1g58380 / 40S ribosomal protein S2 (RPS2A) | |  |  |  |  |  | | --- | --- | --- | --- | --- | |  |  |  |  |  | | C49H3.11 | | | | | | |
|  | Cluster:3-0 | |  |  | 2 | 231 | 4 | 4426 | 0.032636177 | 0.06527235 | 2 |
|  |  | RAFL08-09-E20 | At2g41840 / 40S ribosomal protein S2 (RPS2C) | |  |  |  |  |  | | --- | --- | --- | --- | --- | |  |  |  |  |  | | C49H3.11 | | | | | | |
|  |  | RAFL06-07-B02 | At3g11940 / 40S ribosomal protein S5 (RPS5B) | |  |  |  |  |  | | --- | --- | --- | --- | --- | |  |  |  |  |  | | T05E11.1 | | | | | | |
| Y39A1A.19 | | | WI5\_id:Y39A1A.19 |  | A | B | C | D | P | P' | N |
|  | Cluster:5-0 | |  |  | 1 | 76 | 1 | 4585 | 0.032756753 | 0.06551351 | 2 |
|  |  | RAFL09-16-L12 | At1g62560 / flavin-containing monooxygenase (FMO) family | |  |  |  |  |  | | --- | --- | --- | --- | --- | |  |  |  |  |  | | Y39A1A.19 | | | | | | |
| C17G10.2 | | | WI5\_id:C17G10.2 |  | A | B | C | D | P | P' | N |
|  | Cluster:5-0 | |  |  | 4 | 73 | 1 | 4585 | 3.3961462E-7 | 6.7922923E-7 | 2 |
|  |  | RAFL04-15-M13 | At5g56030 / heat shock protein 81-2 (HSP81-2) | |  |  |  |  |  | | --- | --- | --- | --- | --- | |  |  |  |  |  | | C47E8.5 | | | | | | |
|  |  | RAFL07-13-H08 | At5g56010 / heat shock protein, putative | |  |  |  |  |  | | --- | --- | --- | --- | --- | |  |  |  |  |  | | C47E8.5 | | | | | | |
|  |  | RAFL09-06-O18 | At5g56030 / heat shock protein 81-2 (HSP81-2) | |  |  |  |  |  | | --- | --- | --- | --- | --- | |  |  |  |  |  | | C47E8.5 | | | | | | |
|  |  | RAFL05-16-L15 | At5g56010 / heat shock protein, putative | |  |  |  |  |  | | --- | --- | --- | --- | --- | |  |  |  |  |  | | C47E8.5 | | | | | | |
| Y110A7A.14 | | | WI5\_id:Y110A7A.14 |  | A | B | C | D | P | P' | N |
|  | Cluster:4-1 | |  |  | 3 | 307 | 1 | 4352 | 0.0011071523 | 0.0022143046 | 2 |
|  |  | RAFL04-15-N11 | At3g22110 / 20S proteasome alpha subunit C (PAC1) | |  |  |  |  |  | | --- | --- | --- | --- | --- | |  |  |  |  |  | | Y110A7A.14 | | | | | | |
|  |  | RAFL05-04-L16 | At1g79210 / 20S proteasome alpha subunit B, putative | |  |  |  |  |  | | --- | --- | --- | --- | --- | |  |  |  |  |  | | D1054.2 | | | | | | |
|  |  | RAFL05-12-G18 | At4g14800 / 20S proteasome beta subunit D2 (PBD2) | |  |  |  |  |  | | --- | --- | --- | --- | --- | |  |  |  |  |  | | T20F5.2 | | | | | | |
| B0336.10 | | | WI5\_id:B0336.10 |  | A | B | C | D | P | P' | N |
|  | Cluster:2-1 | |  |  | 5 | 239 | 9 | 4410 | 5.1060907E-4 | 0.0030636543 | 6 |
|  |  | RAFL07-15-M07 | At1g04480 / 60S ribosomal protein L23 (RPL23A) | |  |  |  |  |  | | --- | --- | --- | --- | --- | |  |  |  |  |  | | B0336.10 | | | | | | |
|  |  | RAFL05-17-L17 | At3g55280 / 60S ribosomal protein L23A (RPL23aB) | |  |  |  |  |  | | --- | --- | --- | --- | --- | |  |  |  |  |  | | F52B5.6 | | | | | | |
|  |  | RAFL11-12-H04 | At3g25520 / 60S ribosomal protein L5 (RPL5A) | |  |  |  |  |  | | --- | --- | --- | --- | --- | |  |  |  |  |  | | F54C9.5 | | | | | | |
|  |  | RAFL05-18-P15 | At1g04480 / 60S ribosomal protein L23 (RPL23A) | |  |  |  |  |  | | --- | --- | --- | --- | --- | |  |  |  |  |  | | B0336.10 | | | | | | |
|  |  | RAFL11-07-B21 | At3g25520 / 60S ribosomal protein L5 (RPL5A) | |  |  |  |  |  | | --- | --- | --- | --- | --- | |  |  |  |  |  | | F54C9.5 | | | | | | |
|  | Cluster:3-0 | |  |  | 3 | 230 | 11 | 4419 | 0.029778728 | 0.17867237 | 6 |
|  |  | RAFL07-10-D02 | At1g14320 / 60S ribosomal protein L10 (RPL10A)/Wilm's tumor suppressor protein-related | |  |  |  |  |  | | --- | --- | --- | --- | --- | |  |  |  |  |  | | F10B5.1 | | | | | | |
|  |  | RAFL09-10-P09 | At4g36130 / 60S ribosomal protein L8 (RPL8C) | |  |  |  |  |  | | --- | --- | --- | --- | --- | |  |  |  |  |  | | B0250.1 | | | | | | |
|  |  | RAFL09-12-B12 | At4g36130 / 60S ribosomal protein L8 (RPL8C) | |  |  |  |  |  | | --- | --- | --- | --- | --- | |  |  |  |  |  | | B0250.1 | | | | | | |
| Y51H4A.17 | | | WI5\_id:Y51H4A.17 |  | A | B | C | D | P | P' | N |
|  | Cluster:2-1 | |  |  | 3 | 241 | 3 | 4416 | 0.0025160299 | 0.0075480896 | 3 |
|  |  | RAFL06-08-N05 | At5g28540 / luminal binding protein 1 precursor (BiP-1) (AtBP1) | |  |  |  |  |  | | --- | --- | --- | --- | --- | |  |  |  |  |  | | F43E2.8 | | | | | | |
|  |  | RAFL04-18-N22 | At2g44120 / 60S ribosomal protein L7 (RPL7C) | |  |  |  |  |  | | --- | --- | --- | --- | --- | |  |  |  |  |  | | F53G12.10 | | | | | | |
|  |  | RAFL07-11-O19 | At5g28540 / luminal binding protein 1 precursor (BiP-1) (AtBP1) | |  |  |  |  |  | | --- | --- | --- | --- | --- | |  |  |  |  |  | | F43E2.8 | | | | | | |
|  | Cluster:2-0 | |  |  | 2 | 148 | 4 | 4509 | 0.014161414 | 0.04248424 | 3 |
|  |  | RAFL06-10-L13 | At3g09630 / 60S ribosomal protein L4/L1 (RPL4A) | |  |  |  |  |  | | --- | --- | --- | --- | --- | |  |  |  |  |  | | B0041.4 | | | | | | |
|  |  | RAFL04-20-D09 | At5g42020 / luminal binding protein 2 precursor (BiP-2) (AtBP2) | |  |  |  |  |  | | --- | --- | --- | --- | --- | |  |  |  |  |  | | F43E2.8 | | | | | | |
| Y69A2AR.18 | | | WI5\_id:Y69A2AR.18 |  | A | B | C | D | P | P' | N |
|  | Cluster:4-1 | |  |  | 2 | 308 | 2 | 4351 | 0.024166461 | 0.07249938 | 3 |
|  |  | RAFL04-15-D14 | At5g08680 / H+-transporting ATP synthase beta chain -related | |  |  |  |  |  | | --- | --- | --- | --- | --- | |  |  |  |  |  | | C34E10.6 | | | | | | |
|  |  | RAFL09-17-J21 | At5g08690 / H+-transporting ATP synthase beta chain (mitochondrial) -related | |  |  |  |  |  | | --- | --- | --- | --- | --- | |  |  |  |  |  | | C34E10.6 | | | | | | |
| T20F5.2 | | | WI5\_id:T20F5.2 |  | A | B | C | D | P | P' | N |
|  | Cluster:4-1 | |  |  | 8 | 302 | 5 | 4348 | 3.3526283E-7 | 1.3410513E-6 | 4 |
|  |  | RAFL04-15-N11 | At3g22110 / 20S proteasome alpha subunit C (PAC1) | |  |  |  |  |  | | --- | --- | --- | --- | --- | |  |  |  |  |  | | Y110A7A.14 | | | | | | |
|  |  | RAFL05-01-C14 | At5g42790 / 20S proteasome alpha subunit F1 (PAF1) | |  |  |  |  |  | | --- | --- | --- | --- | --- | |  |  |  |  |  | | CD4.6 | | | | | | |
|  |  | RAFL07-08-J17 | At1g13060 / 20S proteasome beta subunit E1 (PBE1) | |  |  |  |  |  | | --- | --- | --- | --- | --- | |  |  |  |  |  | | K05C4.1 | | | | | | |
|  |  | RAFL05-04-G23 | At1g77440 / 20S proteasome beta subunit C (PBC2) | |  |  |  |  |  | | --- | --- | --- | --- | --- | |  |  |  |  |  | | Y38A8.2 | | | | | | |
|  |  | RAFL04-12-B09 | At3g14290 / 20S proteasome alpha subunit E2 (PAE2) | |  |  |  |  |  | | --- | --- | --- | --- | --- | |  |  |  |  |  | | F25H2.9 | | | | | | |
|  |  | RAFL05-04-L16 | At1g79210 / 20S proteasome alpha subunit B, putative | |  |  |  |  |  | | --- | --- | --- | --- | --- | |  |  |  |  |  | | D1054.2 | | | | | | |
|  |  | RAFL05-12-G18 | At4g14800 / 20S proteasome beta subunit D2 (PBD2) | |  |  |  |  |  | | --- | --- | --- | --- | --- | |  |  |  |  |  | | T20F5.2 | | | | | | |
|  |  | RAFL05-21-D23 | At1g21720 / 20S proteasome beta subunit C (PBC1) | |  |  |  |  |  | | --- | --- | --- | --- | --- | |  |  |  |  |  | | Y38A8.2 | | | | | | |
| W09H1.6 | | | WI5\_id:W09H1.6 |  | A | B | C | D | P | P' | N |
|  | Cluster:3-0 | |  |  | 2 | 231 | 0 | 4430 | 0.0024866017 | 0.0024866017 | 1 |
|  |  | RAFL09-10-P09 | At4g36130 / 60S ribosomal protein L8 (RPL8C) | |  |  |  |  |  | | --- | --- | --- | --- | --- | |  |  |  |  |  | | B0250.1 | | | | | | |
|  |  | RAFL09-12-B12 | At4g36130 / 60S ribosomal protein L8 (RPL8C) | |  |  |  |  |  | | --- | --- | --- | --- | --- | |  |  |  |  |  | | B0250.1 | | | | | | |
| Y62E10A.14 | | | WI5\_id:Y62E10A.14 |  | A | B | C | D | P | P' | N |
|  | Cluster:6-1 | |  |  | 2 | 313 | 0 | 4348 | 0.004549907 | 0.004549907 | 1 |
|  |  | RAFL05-08-I15 | At4g24820 / 26S proteasome regulatory subunit (RPN7), putative | |  |  |  |  |  | | --- | --- | --- | --- | --- | |  |  |  |  |  | | F49C12.8 | | | | | | |
|  |  | RAFL07-13-D20 | At4g24820 / 26S proteasome regulatory subunit (RPN7), putative | |  |  |  |  |  | | --- | --- | --- | --- | --- | |  |  |  |  |  | | F49C12.8 | | | | | | |
| F27C1.7 | | | WI5\_id:F27C1.7 |  | A | B | C | D | P | P' | N |
|  | Cluster:4-1 | |  |  | 2 | 308 | 2 | 4351 | 0.024166461 | 0.07249938 | 3 |
|  |  | RAFL04-15-D14 | At5g08680 / H+-transporting ATP synthase beta chain -related | |  |  |  |  |  | | --- | --- | --- | --- | --- | |  |  |  |  |  | | C34E10.6  ATP synthase beta chain | | | | | | |
|  |  | RAFL09-17-J21 | At5g08690 / H+-transporting ATP synthase beta chain (mitochondrial) -related | |  |  |  |  |  | | --- | --- | --- | --- | --- | |  |  |  |  |  | | C34E10.6  ATP synthase beta chain | | | | | | |
| B0041.4 | | | WI5\_id:B0041.4 |  | A | B | C | D | P | P' | N |
|  | Cluster:3-0 | |  |  | 4 | 229 | 9 | 4421 | 0.0030380234 | 0.01822814 | 6 |
|  |  | RAFL07-10-D02 | At1g14320 / 60S ribosomal protein L10 (RPL10A)/Wilm's tumor suppressor protein-related | |  |  |  |  |  | | --- | --- | --- | --- | --- | |  |  |  |  |  | | F10B5.1  ribosomal protein L10 (QM protein) | | | | | | |
|  |  | RAFL09-10-P09 | At4g36130 / 60S ribosomal protein L8 (RPL8C) | |  |  |  |  |  | | --- | --- | --- | --- | --- | |  |  |  |  |  | | B0250.1  Ribosomal Proteins L2 | | | | | | |
|  |  | RAFL08-10-G08 | At3g09630 / 60S ribosomal protein L4/L1 (RPL4A) | |  |  |  |  |  | | --- | --- | --- | --- | --- | |  |  |  |  |  | | B0041.4  ribosomal protein L1 | | | | | | |
|  |  | RAFL09-12-B12 | At4g36130 / 60S ribosomal protein L8 (RPL8C) | |  |  |  |  |  | | --- | --- | --- | --- | --- | |  |  |  |  |  | | B0250.1  Ribosomal Proteins L2 | | | | | | |
|  | Cluster:2-0 | |  |  | 3 | 147 | 10 | 4503 | 0.0073604016 | 0.04416241 | 6 |
|  |  | RAFL04-19-O24 | At5g02610 / 60S ribosomal protein L35 (RPL35D) | |  |  |  |  |  | | --- | --- | --- | --- | --- | |  |  |  |  |  | | ZK652.4  60S ribosomal protein L35 | | | | | | |
|  |  | RAFL06-10-L13 | At3g09630 / 60S ribosomal protein L4/L1 (RPL4A) | |  |  |  |  |  | | --- | --- | --- | --- | --- | |  |  |  |  |  | | B0041.4  ribosomal protein L1 | | | | | | |
|  |  | RAFL11-12-M17 | At3g25520 / 60S ribosomal protein L5 (RPL5A) | |  |  |  |  |  | | --- | --- | --- | --- | --- | |  |  |  |  |  | | F54C9.5 | | | | | | |
|  | Cluster:2-1 | |  |  | 3 | 241 | 10 | 4409 | 0.027396575 | 0.16437945 | 6 |
|  |  | RAFL05-17-L17 | At3g55280 / 60S ribosomal protein L23A (RPL23aB) | |  |  |  |  |  | | --- | --- | --- | --- | --- | |  |  |  |  |  | | F52B5.6  60S ribosomal protein | | | | | | |
|  |  | RAFL11-12-H04 | At3g25520 / 60S ribosomal protein L5 (RPL5A) | |  |  |  |  |  | | --- | --- | --- | --- | --- | |  |  |  |  |  | | F54C9.5 | | | | | | |
|  |  | RAFL11-07-B21 | At3g25520 / 60S ribosomal protein L5 (RPL5A) | |  |  |  |  |  | | --- | --- | --- | --- | --- | |  |  |  |  |  | | F54C9.5 | | | | | | |
| T05G5.3 | | | WI5\_id:T05G5.3 |  | A | B | C | D | P | P' | N |
|  | Cluster:4-2 | |  |  | 1 | 134 | 0 | 4528 | 0.028951319 | 0.028951319 | 1 |
|  |  | RAFL05-18-G17 | At3g48750 / cell division control protein 2 homolog A (CDC2A) | |  |  |  |  |  | | --- | --- | --- | --- | --- | |  |  |  |  |  | | T05G5.3 | | | | | | |
| C36B1.12 | | | WI5\_id:C36B1.12 |  | A | B | C | D | P | P' | N |
|  | Cluster:4-0 | |  |  | 2 | 238 | 1 | 4422 | 0.0076462985 | 0.015292597 | 2 |
|  |  | RAFL06-07-B07 | At5g55190 / GTP-binding protein atran3, putative | |  |  |  |  |  | | --- | --- | --- | --- | --- | |  |  |  |  |  | | K01G5.4 | | | | | | |
|  |  | RAFL05-17-C16 | At5g55190 / GTP-binding protein atran3, putative | |  |  |  |  |  | | --- | --- | --- | --- | --- | |  |  |  |  |  | | K01G5.4 | | | | | | |
| T27F7.3 | | | WI5\_id:T27F7.3 |  | A | B | C | D | P | P' | N |
|  | Cluster:4-1 | |  |  | 2 | 308 | 2 | 4351 | 0.024166461 | 0.07249938 | 3 |
|  |  | RAFL05-14-N10 | At3g11400 / eukaryotic translation initiation factor 3 subunit g (eIF3g) | |  |  |  |  |  | | --- | --- | --- | --- | --- | |  |  |  |  |  | | F22B5.2 | | | | | | |
|  |  | RAFL07-07-F13 | At3g56150 / PROBABLE EUKARYOTIC TRANSLATION INITIATION FACTOR 3 SUBUNIT 8 | |  |  |  |  |  | | --- | --- | --- | --- | --- | |  |  |  |  |  | | T23D8.4 | | | | | | |
| K01G5.7 | | | WI5\_id:K01G5.7 |  | A | B | C | D | P | P' | N |
|  | Cluster:1-0 | |  |  | 2 | 147 | 4 | 4510 | 0.013980698 | 0.05592279 | 4 |
|  |  | RAFL09-11-P11 | At1g20010 / tubulin beta-5 chain (TUB5) | |  |  |  |  |  | | --- | --- | --- | --- | --- | |  |  |  |  |  | | K01G5.7 | | | | | | |
|  |  | RAFL04-16-G05 | At5g44340 / tubulin beta-4 chain (TUB4) | |  |  |  |  |  | | --- | --- | --- | --- | --- | |  |  |  |  |  | | K01G5.7 | | | | | | |
|  | Cluster:2-1 | |  |  | 2 | 242 | 4 | 4415 | 0.0355681 | 0.1422724 | 4 |
|  |  | RAFL05-09-F01 | At5g44340 / tubulin beta-4 chain (TUB4) | |  |  |  |  |  | | --- | --- | --- | --- | --- | |  |  |  |  |  | | K01G5.7 | | | | | | |
|  |  | RAFL06-12-I16 | At2g29550 / tubulin beta-7 chain (TUB7) | |  |  |  |  |  | | --- | --- | --- | --- | --- | |  |  |  |  |  | | K01G5.7 | | | | | | |
| Y37E3.8 | | | WI5\_id:Y37E3.8 |  | A | B | C | D | P | P' | N |
|  | Cluster:2-1 | |  |  | 6 | 238 | 13 | 4406 | 2.9421985E-4 | 0.001765319 | 6 |
|  |  | RAFL02-10-A09 | At1g33140 / 60S ribosomal protein L9 (RPL90A/C) | |  |  |  |  |  | | --- | --- | --- | --- | --- | |  |  |  |  |  | | R13A5.8 | | | | | | |
|  |  | RAFL07-15-M07 | At1g04480 / 60S ribosomal protein L23 (RPL23A) | |  |  |  |  |  | | --- | --- | --- | --- | --- | |  |  |  |  |  | | B0336.10 | | | | | | |
|  |  | RAFL05-17-L17 | At3g55280 / 60S ribosomal protein L23A (RPL23aB) | |  |  |  |  |  | | --- | --- | --- | --- | --- | |  |  |  |  |  | | F52B5.6 | | | | | | |
|  |  | RAFL11-12-H04 | At3g25520 / 60S ribosomal protein L5 (RPL5A) | |  |  |  |  |  | | --- | --- | --- | --- | --- | |  |  |  |  |  | | F54C9.5 | | | | | | |
|  |  | RAFL05-18-P15 | At1g04480 / 60S ribosomal protein L23 (RPL23A) | |  |  |  |  |  | | --- | --- | --- | --- | --- | |  |  |  |  |  | | B0336.10 | | | | | | |
|  |  | RAFL11-07-B21 | At3g25520 / 60S ribosomal protein L5 (RPL5A) | |  |  |  |  |  | | --- | --- | --- | --- | --- | |  |  |  |  |  | | F54C9.5 | | | | | | |
|  | Cluster:3-0 | |  |  | 5 | 228 | 14 | 4416 | 0.0019486465 | 0.011691879 | 6 |
|  |  | RAFL07-10-D02 | At1g14320 / 60S ribosomal protein L10 (RPL10A)/Wilm's tumor suppressor protein-related | |  |  |  |  |  | | --- | --- | --- | --- | --- | |  |  |  |  |  | | F10B5.1 | | | | | | |
|  |  | RAFL09-10-P09 | At4g36130 / 60S ribosomal protein L8 (RPL8C) | |  |  |  |  |  | | --- | --- | --- | --- | --- | |  |  |  |  |  | | B0250.1 | | | | | | |
|  |  | RAFL08-10-G08 | At3g09630 / 60S ribosomal protein L4/L1 (RPL4A) | |  |  |  |  |  | | --- | --- | --- | --- | --- | |  |  |  |  |  | | B0041.4 | | | | | | |
|  |  | RAFL08-13-M06 | At3g11250 / 60S acidic ribosomal protein P0 (RPP0C) | |  |  |  |  |  | | --- | --- | --- | --- | --- | |  |  |  |  |  | | F25H2.10 | | | | | | |
|  |  | RAFL09-12-B12 | At4g36130 / 60S ribosomal protein L8 (RPL8C) | |  |  |  |  |  | | --- | --- | --- | --- | --- | |  |  |  |  |  | | B0250.1 | | | | | | |
|  | Cluster:2-0 | |  |  | 4 | 146 | 15 | 4498 | 0.002734157 | 0.016404942 | 6 |
|  |  | RAFL04-19-O24 | At5g02610 / 60S ribosomal protein L35 (RPL35D) | |  |  |  |  |  | | --- | --- | --- | --- | --- | |  |  |  |  |  | | ZK652.4 | | | | | | |
|  |  | RAFL06-10-L13 | At3g09630 / 60S ribosomal protein L4/L1 (RPL4A) | |  |  |  |  |  | | --- | --- | --- | --- | --- | |  |  |  |  |  | | B0041.4 | | | | | | |
|  |  | RAFL11-12-M17 | At3g25520 / 60S ribosomal protein L5 (RPL5A) | |  |  |  |  |  | | --- | --- | --- | --- | --- | |  |  |  |  |  | | F54C9.5 | | | | | | |
|  |  | RAFL03-06-H07 | At1g33140 / 60S ribosomal protein L9 (RPL90A/C) | |  |  |  |  |  | | --- | --- | --- | --- | --- | |  |  |  |  |  | | R13A5.8 | | | | | | |
| T25C8.2 | | | WI5\_id:T25C8.2 |  | A | B | C | D | P | P' | N |
|  | Cluster:4-2 | |  |  | 1 | 134 | 0 | 4528 | 0.028951319 | 0.028951319 | 1 |
|  |  | RAFL04-20-K07 | At3g53750 / actin (ACT3) | |  |  |  |  |  | | --- | --- | --- | --- | --- | |  |  |  |  |  | | T04C12.6 | | | | | | |
| R07H5.1 | | | WI5\_id:R07H5.1 |  | A | B | C | D | P | P' | N |
|  | Cluster:4-2 | |  |  | 1 | 134 | 0 | 4528 | 0.028951319 | 0.028951319 | 1 |
|  |  | RAFL05-05-K13 | At3g52590 / ubiquitin extension protein 1 (UBQ1)/60S ribosomal protein L40 (RPL40B) | |  |  |  |  |  | | --- | --- | --- | --- | --- | |  |  |  |  |  | | ZK1010.1 | | | | | | |
| C52E4.4 | | | WI5\_id:C52E4.4 |  | A | B | C | D | P | P' | N |
|  | Cluster:6-1 | |  |  | 4 | 311 | 14 | 4334 | 0.029376008 | 0.3525121 | 12 |
|  |  | RAFL08-19-N19 | At3g05530 / 26S proteasome AAA-ATPase subunit RPT5a | |  |  |  |  |  | | --- | --- | --- | --- | --- | |  |  |  |  |  | | F56H1.4 | | | | | | |
|  |  | RAFL05-08-I15 | At4g24820 / 26S proteasome regulatory subunit (RPN7), putative | |  |  |  |  |  | | --- | --- | --- | --- | --- | |  |  |  |  |  | | F49C12.8 | | | | | | |
|  |  | RAFL07-13-D20 | At4g24820 / 26S proteasome regulatory subunit (RPN7), putative | |  |  |  |  |  | | --- | --- | --- | --- | --- | |  |  |  |  |  | | F49C12.8 | | | | | | |
|  |  | RAFL11-04-H03 | At1g20200 / 26S proteasome regulatory subunit S3 (RPN3), putative | |  |  |  |  |  | | --- | --- | --- | --- | --- | |  |  |  |  |  | | C30C11.2 | | | | | | |
| R06C1.3 | | | WI5\_id:R06C1.3 |  | A | B | C | D | P | P' | N |
|  | Cluster:5-0 | |  |  | 1 | 76 | 0 | 4586 | 0.016512975 | 0.016512975 | 1 |
|  |  | RAFL06-07-P18 | At3g60820 / 20S proteasome beta subunit F1 (PBF1) | |  |  |  |  |  | | --- | --- | --- | --- | --- | |  |  |  |  |  | | C02F5.9 | | | | | | |
| ZC504.4 | | | WI5\_id:ZC504.4 |  | A | B | C | D | P | P' | N |
|  | Cluster:5-0 | |  |  | 1 | 76 | 1 | 4585 | 0.032756753 | 0.06551351 | 2 |
|  |  | RAFL09-16-L12 | At1g62560 / flavin-containing monooxygenase (FMO) family | |  |  |  |  |  | | --- | --- | --- | --- | --- | |  |  |  |  |  | | Y39A1A.19  DIMETHYLANILINE MONOOXYGENASE (N-OXIDE FORMING) 5 (EC 1.14.13.8) (HEPATIC FLAVIN-CONTAINING MONOOXYGENASE 5) (FMO 5) (DIMETHYLANILINE OXIDA .. | | | | | | |
| Y46G5A.4 | | | WI5\_id:Y46G5A.4 |  | A | B | C | D | P | P' | N |
|  | Cluster:1-0 | |  |  | 1 | 148 | 0 | 4514 | 0.031953678 | 0.031953678 | 1 |
|  |  | RAFL04-19-J20 | At3g50270 / hydroxycinnamoyl/benzoyltransferase-related | |  |  |  |  |  | | --- | --- | --- | --- | --- | |  |  |  |  |  | | K04G7.10 | | | | | | |
| C18H9.7 | | | WI5\_id:C18H9.7 |  | A | B | C | D | P | P' | N |
|  | Cluster:4-2 | |  |  | 1 | 134 | 0 | 4528 | 0.028951319 | 0.028951319 | 1 |
|  |  | RAFL05-07-M02 | At1g34030 / 40S ribosomal protein S18 (RPS18B) | |  |  |  |  |  | | --- | --- | --- | --- | --- | |  |  |  |  |  | | Y57G11C.16 | | | | | | |
| F07D10.1 | | | WI5\_id:F07D10.1 |  | A | B | C | D | P | P' | N |
|  | Cluster:1-2 | |  |  | 2 | 172 | 1 | 4488 | 0.0040519224 | 0.008103845 | 2 |
|  |  | RAFL05-04-J09 | At5g45775 / 60S ribosomal protein L11 (RPL11D) | |  |  |  |  |  | | --- | --- | --- | --- | --- | |  |  |  |  |  | | F07D10.1 | | | | | | |
|  |  | RAFL11-10-E06 | At5g45775 / 60S ribosomal protein L11 (RPL11D) | |  |  |  |  |  | | --- | --- | --- | --- | --- | |  |  |  |  |  | | F07D10.1 | | | | | | |
| Y79H2A.1 | | | WI5\_id:Y79H2A.1 |  | A | B | C | D | P | P' | N |
|  | Cluster:5-0 | |  |  | 1 | 76 | 2 | 4584 | 0.04873567 | 0.146207 | 3 |
|  |  | RAFL06-07-P18 | At3g60820 / 20S proteasome beta subunit F1 (PBF1) | |  |  |  |  |  | | --- | --- | --- | --- | --- | |  |  |  |  |  | | C02F5.9 | | | | | | |
| R02F11.1 | | | WI5\_id:R02F11.1 |  | A | B | C | D | P | P' | N |
|  | Cluster:2-0 | |  |  | 1 | 149 | 0 | 4513 | 0.03216813 | 0.03216813 | 1 |
|  |  | RAFL03-08-O03 | At1g07940 / elongation factor 1-alpha (EF-1-alpha) | |  |  |  |  |  | | --- | --- | --- | --- | --- | |  |  |  |  |  | | F31E3.5  Elongation factor 1-alpha | | | | | | |
| F32A11.2 | | | WI5\_id:F32A11.2 |  | A | B | C | D | P | P' | N |
|  | Cluster:5-0 | |  |  | 4 | 73 | 2 | 4584 | 1.0060772E-6 | 3.0182316E-6 | 3 |
|  |  | RAFL04-15-M13 | At5g56030 / heat shock protein 81-2 (HSP81-2) | |  |  |  |  |  | | --- | --- | --- | --- | --- | |  |  |  |  |  | | C47E8.5 | | | | | | |
|  |  | RAFL07-13-H08 | At5g56010 / heat shock protein, putative | |  |  |  |  |  | | --- | --- | --- | --- | --- | |  |  |  |  |  | | C47E8.5 | | | | | | |
|  |  | RAFL09-06-O18 | At5g56030 / heat shock protein 81-2 (HSP81-2) | |  |  |  |  |  | | --- | --- | --- | --- | --- | |  |  |  |  |  | | C47E8.5 | | | | | | |
|  |  | RAFL05-16-L15 | At5g56010 / heat shock protein, putative | |  |  |  |  |  | | --- | --- | --- | --- | --- | |  |  |  |  |  | | C47E8.5 | | | | | | |
| K01A2.10 | | | WI5\_id:K01A2.10 |  | A | B | C | D | P | P' | N |
|  | Cluster:7-0 | |  |  | 2 | 245 | 0 | 4416 | 0.002795081 | 0.002795081 | 1 |
|  |  | RAFL05-04-D08 | At1g64520 / 26S proteasome regulatory subunit (RPN12), putative | |  |  |  |  |  | | --- | --- | --- | --- | --- | |  |  |  |  |  | | ZK20.5 | | | | | | |
|  |  | RAFL06-13-G03 | At1g64520 / 26S proteasome regulatory subunit (RPN12), putative | |  |  |  |  |  | | --- | --- | --- | --- | --- | |  |  |  |  |  | | ZK20.5 | | | | | | |
| C04G2.6 | | | WI5\_id:C04G2.6 |  | A | B | C | D | P | P' | N |
|  | Cluster:4-0 | |  |  | 2 | 238 | 1 | 4422 | 0.0076462985 | 0.015292597 | 2 |
|  |  | RAFL06-07-B07 | At5g55190 / GTP-binding protein atran3, putative | |  |  |  |  |  | | --- | --- | --- | --- | --- | |  |  |  |  |  | | K01G5.4 | | | | | | |
|  |  | RAFL05-17-C16 | At5g55190 / GTP-binding protein atran3, putative | |  |  |  |  |  | | --- | --- | --- | --- | --- | |  |  |  |  |  | | K01G5.4 | | | | | | |
| C52B11.2 | | | WI5\_id:C52B11.2 |  | A | B | C | D | P | P' | N |
|  | Cluster:0-2 | |  |  | 1 | 78 | 0 | 4584 | 0.016941883 | 0.016941883 | 1 |
|  |  | RAFL04-17-G02 | At1g56340 / calreticulin 1 (CRT1) | |  |  |  |  |  | | --- | --- | --- | --- | --- | |  |  |  |  |  | | Y38A10A.5 | | | | | | |
| C07H6.5 | | | WI5\_id:C07H6.5 |  | A | B | C | D | P | P' | N |
|  | Cluster:3-0 | |  |  | 2 | 231 | 1 | 4429 | 0.007213332 | 0.014426664 | 2 |
|  |  | RAFL09-10-P09 | At4g36130 / 60S ribosomal protein L8 (RPL8C) | |  |  |  |  |  | | --- | --- | --- | --- | --- | |  |  |  |  |  | | B0250.1 | | | | | | |
|  |  | RAFL09-12-B12 | At4g36130 / 60S ribosomal protein L8 (RPL8C) | |  |  |  |  |  | | --- | --- | --- | --- | --- | |  |  |  |  |  | | B0250.1 | | | | | | |
| ZK1098.4 | | | WI5\_id:ZK1098.4 |  | A | B | C | D | P | P' | N |
|  | Cluster:5-0 | |  |  | 1 | 76 | 2 | 4584 | 0.04873567 | 0.146207 | 3 |
|  |  | RAFL06-07-P18 | At3g60820 / 20S proteasome beta subunit F1 (PBF1) | |  |  |  |  |  | | --- | --- | --- | --- | --- | |  |  |  |  |  | | C02F5.9 | | | | | | |
| ZC434.8 | | | WI5\_id:ZC434.8 |  | A | B | C | D | P | P' | N |
|  | Cluster:5-0 | |  |  | 4 | 73 | 1 | 4585 | 3.3961462E-7 | 6.7922923E-7 | 2 |
|  |  | RAFL04-15-M13 | At5g56030 / heat shock protein 81-2 (HSP81-2) | |  |  |  |  |  | | --- | --- | --- | --- | --- | |  |  |  |  |  | | C47E8.5 | | | | | | |
|  |  | RAFL07-13-H08 | At5g56010 / heat shock protein, putative | |  |  |  |  |  | | --- | --- | --- | --- | --- | |  |  |  |  |  | | C47E8.5 | | | | | | |
|  |  | RAFL09-06-O18 | At5g56030 / heat shock protein 81-2 (HSP81-2) | |  |  |  |  |  | | --- | --- | --- | --- | --- | |  |  |  |  |  | | C47E8.5 | | | | | | |
|  |  | RAFL05-16-L15 | At5g56010 / heat shock protein, putative | |  |  |  |  |  | | --- | --- | --- | --- | --- | |  |  |  |  |  | | C47E8.5 | | | | | | |
| C48B6.3 | | | WI5\_id:C48B6.3 |  | A | B | C | D | P | P' | N |
|  | Cluster:3-0 | |  |  | 1 | 232 | 0 | 4430 | 0.049967833 | 0.049967833 | 1 |
|  |  | RAFL04-16-E04 | At3g51260 / 20S proteasome alpha subunit D (PAD1) | |  |  |  |  |  | | --- | --- | --- | --- | --- | |  |  |  |  |  | | C36B1.4 | | | | | | |
| F10C5.2 | | | WI5\_id:F10C5.2 |  | A | B | C | D | P | P' | N |
|  | Cluster:4-0 | |  |  | 2 | 238 | 1 | 4422 | 0.0076462985 | 0.015292597 | 2 |
|  |  | RAFL06-07-B07 | At5g55190 / GTP-binding protein atran3, putative | |  |  |  |  |  | | --- | --- | --- | --- | --- | |  |  |  |  |  | | K01G5.4 | | | | | | |
|  |  | RAFL05-17-C16 | At5g55190 / GTP-binding protein atran3, putative | |  |  |  |  |  | | --- | --- | --- | --- | --- | |  |  |  |  |  | | K01G5.4 | | | | | | |
| F36A2.6 | | | WI5\_id:F36A2.6 |  | A | B | C | D | P | P' | N |
|  | Cluster:2-1 | |  |  | 8 | 236 | 10 | 4409 | 1.3906676E-6 | 6.953338E-6 | 5 |
|  |  | RAFL04-18-N10 | At1g07770 / 40S ribosomal protein S15A (RPS15aA) | |  |  |  |  |  | | --- | --- | --- | --- | --- | |  |  |  |  |  | | F53A3.3 | | | | | | |
|  |  | RAFL09-15-M15 | At5g23740 / 40S ribosomal protein S11 (RPS11C) | |  |  |  |  |  | | --- | --- | --- | --- | --- | |  |  |  |  |  | | F40F11.1 | | | | | | |
|  |  | RAFL05-05-M24 | At5g02960 / 40S ribosomal protein S23 (RPS23B) | |  |  |  |  |  | | --- | --- | --- | --- | --- | |  |  |  |  |  | | F28D1.7 | | | | | | |
|  |  | RAFL07-13-J18 | At2g37270 / 40S ribosomal protein S5 (RPS5A) | |  |  |  |  |  | | --- | --- | --- | --- | --- | |  |  |  |  |  | | T05E11.1 | | | | | | |
|  |  | RAFL06-09-H09 | At3g48930 / 40S ribosomal protein S11 (RPS11A) | |  |  |  |  |  | | --- | --- | --- | --- | --- | |  |  |  |  |  | | F40F11.1 | | | | | | |
|  |  | RAFL06-08-B09 | At3g11510 / 40S ribosomal protein S14 (RPS14B) | |  |  |  |  |  | | --- | --- | --- | --- | --- | |  |  |  |  |  | | F37C12.9 | | | | | | |
|  |  | RAFL05-16-H14 | At1g04270 / 40S ribosomal protein S15 (RPS15A) | |  |  |  |  |  | | --- | --- | --- | --- | --- | |  |  |  |  |  | | F36A2.6 | | | | | | |
|  |  | RAFL04-19-M20 | At1g58380 / 40S ribosomal protein S2 (RPS2A) | |  |  |  |  |  | | --- | --- | --- | --- | --- | |  |  |  |  |  | | C49H3.11 | | | | | | |
|  | Cluster:4-2 | |  |  | 4 | 131 | 14 | 4514 | 0.0014992869 | 0.0074964343 | 5 |
|  |  | RAFL05-07-M02 | At1g34030 / 40S ribosomal protein S18 (RPS18B) | |  |  |  |  |  | | --- | --- | --- | --- | --- | |  |  |  |  |  | | Y57G11C.16 | | | | | | |
|  |  | RAFL09-17-F16 | At1g07770 / 40S ribosomal protein S15A (RPS15aA) | |  |  |  |  |  | | --- | --- | --- | --- | --- | |  |  |  |  |  | | F53A3.3 | | | | | | |
|  |  | RAFL11-02-D23 | At1g07770 / 40S ribosomal protein S15A (RPS15aA) | |  |  |  |  |  | | --- | --- | --- | --- | --- | |  |  |  |  |  | | F53A3.3 | | | | | | |
|  |  | RAFL05-08-B08 | At5g18380 / 40S ribosomal protein S16 (RPS16C) | |  |  |  |  |  | | --- | --- | --- | --- | --- | |  |  |  |  |  | | T01C3.6 | | | | | | |
|  | Cluster:3-0 | |  |  | 4 | 229 | 14 | 4416 | 0.01067757 | 0.053387847 | 5 |
|  |  | RAFL02-10-H10 | At3g43980 / 40S ribosomal protein S29 (RPS29A) | |  |  |  |  |  | | --- | --- | --- | --- | --- | |  |  |  |  |  | | B0412.4 | | | | | | |
|  |  | RAFL08-09-E20 | At2g41840 / 40S ribosomal protein S2 (RPS2C) | |  |  |  |  |  | | --- | --- | --- | --- | --- | |  |  |  |  |  | | C49H3.11 | | | | | | |
|  |  | RAFL11-04-A02 | At1g07770 / 40S ribosomal protein S15A (RPS15aA) | |  |  |  |  |  | | --- | --- | --- | --- | --- | |  |  |  |  |  | | F53A3.3 | | | | | | |
|  |  | RAFL06-07-B02 | At3g11940 / 40S ribosomal protein S5 (RPS5B) | |  |  |  |  |  | | --- | --- | --- | --- | --- | |  |  |  |  |  | | T05E11.1 | | | | | | |
| Y38F2AL.3 | | | WI5\_id:Y38F2AL.3 |  | A | B | C | D | P | P' | N |
|  | Cluster:4-1 | |  |  | 2 | 308 | 2 | 4351 | 0.024166461 | 0.07249938 | 3 |
|  |  | RAFL04-09-A19 | At4g38510 / probable H+-transporting ATPase | |  |  |  |  |  | | --- | --- | --- | --- | --- | |  |  |  |  |  | | F20B6.2 | | | | | | |
|  |  | RAFL07-17-H08 | At4g38510 / probable H+-transporting ATPase | |  |  |  |  |  | | --- | --- | --- | --- | --- | |  |  |  |  |  | | F20B6.2 | | | | | | |
| F57B9.2 | | | WI5\_id:F57B9.2 |  | A | B | C | D | P | P' | N |
|  | Cluster:1-2 | |  |  | 1 | 173 | 0 | 4489 | 0.037315033 | 0.037315033 | 1 |
|  |  | RAFL11-02-K03 | At5g18230 / expressed protein | |  |  |  |  |  | | --- | --- | --- | --- | --- | |  |  |  |  |  | | Y56A3A.1 | | | | | | |
| F35G12.12 | | | WI5\_id:F35G12.12 |  | A | B | C | D | P | P' | N |
|  | Cluster:5-0 | |  |  | 1 | 76 | 0 | 4586 | 0.016512975 | 0.016512975 | 1 |
|  |  | RAFL06-08-P09 | At1g53750 / 26S proteasome AAA-ATPase subunit RPT1a | |  |  |  |  |  | | --- | --- | --- | --- | --- | |  |  |  |  |  | | C52E4.4 | | | | | | |
| F28D1.2 | | | WI5\_id:F28D1.2 |  | A | B | C | D | P | P' | N |
|  | Cluster:4-1 | |  |  | 2 | 308 | 3 | 4350 | 0.038523052 | 0.15409221 | 4 |
|  |  | RAFL04-15-D14 | At5g08680 / H+-transporting ATP synthase beta chain -related | |  |  |  |  |  | | --- | --- | --- | --- | --- | |  |  |  |  |  | | C34E10.6 | | | | | | |
|  |  | RAFL09-17-J21 | At5g08690 / H+-transporting ATP synthase beta chain (mitochondrial) -related | |  |  |  |  |  | | --- | --- | --- | --- | --- | |  |  |  |  |  | | C34E10.6 | | | | | | |
| Y110A7A.10 | | | WI5\_id:Y110A7A.10 |  | A | B | C | D | P | P' | N |
|  | Cluster:4-1 | |  |  | 2 | 308 | 2 | 4351 | 0.024166461 | 0.07249938 | 3 |
|  |  | RAFL04-15-D14 | At5g08680 / H+-transporting ATP synthase beta chain -related | |  |  |  |  |  | | --- | --- | --- | --- | --- | |  |  |  |  |  | | C34E10.6 | | | | | | |
|  |  | RAFL09-17-J21 | At5g08690 / H+-transporting ATP synthase beta chain (mitochondrial) -related | |  |  |  |  |  | | --- | --- | --- | --- | --- | |  |  |  |  |  | | C34E10.6 | | | | | | |
| F37C12.4 | | | WI5\_id:F37C12.4 |  | A | B | C | D | P | P' | N |
|  | Cluster:2-1 | |  |  | 2 | 242 | 3 | 4416 | 0.024550742 | 0.098202966 | 4 |
|  |  | RAFL11-12-H04 | At3g25520 / 60S ribosomal protein L5 (RPL5A) | |  |  |  |  |  | | --- | --- | --- | --- | --- | |  |  |  |  |  | | F54C9.5 | | | | | | |
|  |  | RAFL11-07-B21 | At3g25520 / 60S ribosomal protein L5 (RPL5A) | |  |  |  |  |  | | --- | --- | --- | --- | --- | |  |  |  |  |  | | F54C9.5 | | | | | | |
| ZK970.4 | | | WI5\_id:ZK970.4 |  | A | B | C | D | P | P' | N |
|  | Cluster:4-1 | |  |  | 2 | 308 | 3 | 4350 | 0.038523052 | 0.15409221 | 4 |
|  |  | RAFL04-09-A19 | At4g38510 / probable H+-transporting ATPase | |  |  |  |  |  | | --- | --- | --- | --- | --- | |  |  |  |  |  | | F20B6.2 | | | | | | |
|  |  | RAFL07-17-H08 | At4g38510 / probable H+-transporting ATPase | |  |  |  |  |  | | --- | --- | --- | --- | --- | |  |  |  |  |  | | F20B6.2 | | | | | | |
| F10B5.1 | | | WI5\_id:F10B5.1 |  | A | B | C | D | P | P' | N |
|  | Cluster:2-1 | |  |  | 6 | 238 | 13 | 4406 | 2.9421985E-4 | 0.001765319 | 6 |
|  |  | RAFL02-10-A09 | At1g33140 / 60S ribosomal protein L9 (RPL90A/C) | |  |  |  |  |  | | --- | --- | --- | --- | --- | |  |  |  |  |  | | R13A5.8 | | | | | | |
|  |  | RAFL07-15-M07 | At1g04480 / 60S ribosomal protein L23 (RPL23A) | |  |  |  |  |  | | --- | --- | --- | --- | --- | |  |  |  |  |  | | B0336.10 | | | | | | |
|  |  | RAFL05-17-L17 | At3g55280 / 60S ribosomal protein L23A (RPL23aB) | |  |  |  |  |  | | --- | --- | --- | --- | --- | |  |  |  |  |  | | F52B5.6 | | | | | | |
|  |  | RAFL11-12-H04 | At3g25520 / 60S ribosomal protein L5 (RPL5A) | |  |  |  |  |  | | --- | --- | --- | --- | --- | |  |  |  |  |  | | F54C9.5 | | | | | | |
|  |  | RAFL05-18-P15 | At1g04480 / 60S ribosomal protein L23 (RPL23A) | |  |  |  |  |  | | --- | --- | --- | --- | --- | |  |  |  |  |  | | B0336.10 | | | | | | |
|  |  | RAFL11-07-B21 | At3g25520 / 60S ribosomal protein L5 (RPL5A) | |  |  |  |  |  | | --- | --- | --- | --- | --- | |  |  |  |  |  | | F54C9.5 | | | | | | |
|  | Cluster:3-0 | |  |  | 5 | 228 | 14 | 4416 | 0.0019486465 | 0.011691879 | 6 |
|  |  | RAFL07-10-D02 | At1g14320 / 60S ribosomal protein L10 (RPL10A)/Wilm's tumor suppressor protein-related | |  |  |  |  |  | | --- | --- | --- | --- | --- | |  |  |  |  |  | | F10B5.1 | | | | | | |
|  |  | RAFL09-10-P09 | At4g36130 / 60S ribosomal protein L8 (RPL8C) | |  |  |  |  |  | | --- | --- | --- | --- | --- | |  |  |  |  |  | | B0250.1 | | | | | | |
|  |  | RAFL08-10-G08 | At3g09630 / 60S ribosomal protein L4/L1 (RPL4A) | |  |  |  |  |  | | --- | --- | --- | --- | --- | |  |  |  |  |  | | B0041.4 | | | | | | |
|  |  | RAFL08-13-M06 | At3g11250 / 60S acidic ribosomal protein P0 (RPP0C) | |  |  |  |  |  | | --- | --- | --- | --- | --- | |  |  |  |  |  | | F25H2.10 | | | | | | |
|  |  | RAFL09-12-B12 | At4g36130 / 60S ribosomal protein L8 (RPL8C) | |  |  |  |  |  | | --- | --- | --- | --- | --- | |  |  |  |  |  | | B0250.1 | | | | | | |
|  | Cluster:2-0 | |  |  | 4 | 146 | 15 | 4498 | 0.002734157 | 0.016404942 | 6 |
|  |  | RAFL04-19-O24 | At5g02610 / 60S ribosomal protein L35 (RPL35D) | |  |  |  |  |  | | --- | --- | --- | --- | --- | |  |  |  |  |  | | ZK652.4 | | | | | | |
|  |  | RAFL06-10-L13 | At3g09630 / 60S ribosomal protein L4/L1 (RPL4A) | |  |  |  |  |  | | --- | --- | --- | --- | --- | |  |  |  |  |  | | B0041.4 | | | | | | |
|  |  | RAFL11-12-M17 | At3g25520 / 60S ribosomal protein L5 (RPL5A) | |  |  |  |  |  | | --- | --- | --- | --- | --- | |  |  |  |  |  | | F54C9.5 | | | | | | |
|  |  | RAFL03-06-H07 | At1g33140 / 60S ribosomal protein L9 (RPL90A/C) | |  |  |  |  |  | | --- | --- | --- | --- | --- | |  |  |  |  |  | | R13A5.8 | | | | | | |
| C56G2.7 | | | WI5\_id:C56G2.7 |  | A | B | C | D | P | P' | N |
|  | Cluster:2-0 | |  |  | 1 | 149 | 0 | 4513 | 0.03216813 | 0.03216813 | 1 |
|  |  | RAFL05-01-F21 | At3g05590 / 60S ribosomal protein L18 (RPL18B) | |  |  |  |  |  | | --- | --- | --- | --- | --- | |  |  |  |  |  | | Y45F10D.12 | | | | | | |
| C47E8.5 | | | WI5\_id:C47E8.5 |  | A | B | C | D | P | P' | N |
|  | Cluster:5-0 | |  |  | 4 | 73 | 7 | 4579 | 2.0782694E-5 | 1.4547886E-4 | 7 |
|  |  | RAFL04-15-M13 | At5g56030 / heat shock protein 81-2 (HSP81-2) | |  |  |  |  |  | | --- | --- | --- | --- | --- | |  |  |  |  |  | | C47E8.5 | | | | | | |
|  |  | RAFL07-13-H08 | At5g56010 / heat shock protein, putative | |  |  |  |  |  | | --- | --- | --- | --- | --- | |  |  |  |  |  | | C47E8.5 | | | | | | |
|  |  | RAFL09-06-O18 | At5g56030 / heat shock protein 81-2 (HSP81-2) | |  |  |  |  |  | | --- | --- | --- | --- | --- | |  |  |  |  |  | | C47E8.5 | | | | | | |
|  |  | RAFL05-16-L15 | At5g56010 / heat shock protein, putative | |  |  |  |  |  | | --- | --- | --- | --- | --- | |  |  |  |  |  | | C47E8.5 | | | | | | |
|  | Cluster:4-2 | |  |  | 2 | 133 | 9 | 4519 | 0.03856442 | 0.26995096 | 7 |
|  |  | RAFL09-17-F16 | At1g07770 / 40S ribosomal protein S15A (RPS15aA) | |  |  |  |  |  | | --- | --- | --- | --- | --- | |  |  |  |  |  | | F53A3.3 | | | | | | |
|  |  | RAFL11-02-D23 | At1g07770 / 40S ribosomal protein S15A (RPS15aA) | |  |  |  |  |  | | --- | --- | --- | --- | --- | |  |  |  |  |  | | F53A3.3 | | | | | | |
| Y63D3A.6 | | | WI5\_id:Y63D3A.6 |  | A | B | C | D | P | P' | N |
|  | Cluster:2-1 | |  |  | 2 | 242 | 1 | 4418 | 0.00789916 | 0.01579832 | 2 |
|  |  | RAFL06-08-N05 | At5g28540 / luminal binding protein 1 precursor (BiP-1) (AtBP1) | |  |  |  |  |  | | --- | --- | --- | --- | --- | |  |  |  |  |  | | C15H9.6  heat shock protein | | | | | | |
|  |  | RAFL07-11-O19 | At5g28540 / luminal binding protein 1 precursor (BiP-1) (AtBP1) | |  |  |  |  |  | | --- | --- | --- | --- | --- | |  |  |  |  |  | | C15H9.6  heat shock protein | | | | | | |
| Y74C10AR.1 | | | WI5\_id:Y74C10AR.1 |  | A | B | C | D | P | P' | N |
|  | Cluster:4-1 | |  |  | 2 | 308 | 2 | 4351 | 0.024166461 | 0.07249938 | 3 |
|  |  | RAFL05-14-N10 | At3g11400 / eukaryotic translation initiation factor 3 subunit g (eIF3g) | |  |  |  |  |  | | --- | --- | --- | --- | --- | |  |  |  |  |  | | F22B5.2 | | | | | | |
|  |  | RAFL07-07-F13 | At3g56150 / PROBABLE EUKARYOTIC TRANSLATION INITIATION FACTOR 3 SUBUNIT 8 | |  |  |  |  |  | | --- | --- | --- | --- | --- | |  |  |  |  |  | | T23D8.4 | | | | | | |
| D2045.2 | | | WI5\_id:D2045.2 |  | A | B | C | D | P | P' | N |
|  | Cluster:4-0 | |  |  | 2 | 238 | 1 | 4422 | 0.0076462985 | 0.015292597 | 2 |
|  |  | RAFL06-07-B07 | At5g55190 / GTP-binding protein atran3, putative | |  |  |  |  |  | | --- | --- | --- | --- | --- | |  |  |  |  |  | | K01G5.4 | | | | | | |
|  |  | RAFL05-17-C16 | At5g55190 / GTP-binding protein atran3, putative | |  |  |  |  |  | | --- | --- | --- | --- | --- | |  |  |  |  |  | | K01G5.4 | | | | | | |
| R07E5.8 | | | WI5\_id:R07E5.8 |  | A | B | C | D | P | P' | N |
|  | Cluster:5-0 | |  |  | 4 | 73 | 1 | 4585 | 3.3961462E-7 | 6.7922923E-7 | 2 |
|  |  | RAFL04-15-M13 | At5g56030 / heat shock protein 81-2 (HSP81-2) | |  |  |  |  |  | | --- | --- | --- | --- | --- | |  |  |  |  |  | | C47E8.5 | | | | | | |
|  |  | RAFL07-13-H08 | At5g56010 / heat shock protein, putative | |  |  |  |  |  | | --- | --- | --- | --- | --- | |  |  |  |  |  | | C47E8.5 | | | | | | |
|  |  | RAFL09-06-O18 | At5g56030 / heat shock protein 81-2 (HSP81-2) | |  |  |  |  |  | | --- | --- | --- | --- | --- | |  |  |  |  |  | | C47E8.5 | | | | | | |
|  |  | RAFL05-16-L15 | At5g56010 / heat shock protein, putative | |  |  |  |  |  | | --- | --- | --- | --- | --- | |  |  |  |  |  | | C47E8.5 | | | | | | |
| F19B10.1 | | | WI5\_id:F19B10.1 |  | A | B | C | D | P | P' | N |
|  | Cluster:6-1 | |  |  | 2 | 313 | 2 | 4346 | 0.024916494 | 0.074749485 | 3 |
|  |  | RAFL05-12-M22 | At2g39760 / expressed protein | |  |  |  |  |  | | --- | --- | --- | --- | --- | |  |  |  |  |  | | ZK858.4 | | | | | | |
|  |  | RAFL11-04-H03 | At1g20200 / 26S proteasome regulatory subunit S3 (RPN3), putative | |  |  |  |  |  | | --- | --- | --- | --- | --- | |  |  |  |  |  | | C30C11.2 | | | | | | |
| F45E1.6 | | | WI5\_id:F45E1.6 |  | A | B | C | D | P | P' | N |
|  | Cluster:2-0 | |  |  | 1 | 149 | 0 | 4513 | 0.03216813 | 0.03216813 | 1 |
|  |  | RAFL05-02-F20 | At5g65360 / histone H3 | |  |  |  |  |  | | --- | --- | --- | --- | --- | |  |  |  |  |  | | F45E1.6  Histone H3 | | | | | | |
| C47E12.5 | | | WI5\_id:C47E12.5 |  | A | B | C | D | P | P' | N |
|  | Cluster:8-0 | |  |  | 1 | 108 | 0 | 4554 | 0.02337551 | 0.02337551 | 1 |
|  |  | RAFL05-03-L12 | At1g47128 / cysteine proteinase RD21A | |  |  |  |  |  | | --- | --- | --- | --- | --- | |  |  |  |  |  | | T03E6.7 | | | | | | |
| Y43B11AR.4 | | | WI5\_id:Y43B11AR.4 |  | A | B | C | D | P | P' | N |
|  | Cluster:2-1 | |  |  | 5 | 239 | 7 | 4412 | 2.202804E-4 | 0.0011014021 | 5 |
|  |  | RAFL04-18-N10 | At1g07770 / 40S ribosomal protein S15A (RPS15aA) | |  |  |  |  |  | | --- | --- | --- | --- | --- | |  |  |  |  |  | | F53A3.3 | | | | | | |
|  |  | RAFL09-15-M15 | At5g23740 / 40S ribosomal protein S11 (RPS11C) | |  |  |  |  |  | | --- | --- | --- | --- | --- | |  |  |  |  |  | | F40F11.1 | | | | | | |
|  |  | RAFL06-09-H09 | At3g48930 / 40S ribosomal protein S11 (RPS11A) | |  |  |  |  |  | | --- | --- | --- | --- | --- | |  |  |  |  |  | | F40F11.1 | | | | | | |
|  |  | RAFL05-16-H14 | At1g04270 / 40S ribosomal protein S15 (RPS15A) | |  |  |  |  |  | | --- | --- | --- | --- | --- | |  |  |  |  |  | | F36A2.6 | | | | | | |
|  |  | RAFL04-19-M20 | At1g58380 / 40S ribosomal protein S2 (RPS2A) | |  |  |  |  |  | | --- | --- | --- | --- | --- | |  |  |  |  |  | | C49H3.11 | | | | | | |
|  | Cluster:3-0 | |  |  | 3 | 230 | 9 | 4421 | 0.019373124 | 0.096865624 | 5 |
|  |  | RAFL02-10-H10 | At3g43980 / 40S ribosomal protein S29 (RPS29A) | |  |  |  |  |  | | --- | --- | --- | --- | --- | |  |  |  |  |  | | B0412.4 | | | | | | |
|  |  | RAFL08-09-E20 | At2g41840 / 40S ribosomal protein S2 (RPS2C) | |  |  |  |  |  | | --- | --- | --- | --- | --- | |  |  |  |  |  | | C49H3.11 | | | | | | |
|  |  | RAFL11-04-A02 | At1g07770 / 40S ribosomal protein S15A (RPS15aA) | |  |  |  |  |  | | --- | --- | --- | --- | --- | |  |  |  |  |  | | F53A3.3 | | | | | | |
|  | Cluster:4-2 | |  |  | 2 | 133 | 10 | 4518 | 0.045415256 | 0.22707628 | 5 |
|  |  | RAFL09-17-F16 | At1g07770 / 40S ribosomal protein S15A (RPS15aA) | |  |  |  |  |  | | --- | --- | --- | --- | --- | |  |  |  |  |  | | F53A3.3 | | | | | | |
|  |  | RAFL11-02-D23 | At1g07770 / 40S ribosomal protein S15A (RPS15aA) | |  |  |  |  |  | | --- | --- | --- | --- | --- | |  |  |  |  |  | | F53A3.3 | | | | | | |
| C43E11.10 | | | WI5\_id:C43E11.10 |  | A | B | C | D | P | P' | N |
|  | Cluster:4-2 | |  |  | 1 | 134 | 0 | 4528 | 0.028951319 | 0.028951319 | 1 |
|  |  | RAFL05-18-G17 | At3g48750 / cell division control protein 2 homolog A (CDC2A) | |  |  |  |  |  | | --- | --- | --- | --- | --- | |  |  |  |  |  | | T05G5.3 | | | | | | |
| C16A11.5 | | | WI5\_id:C16A11.5 |  | A | B | C | D | P | P' | N |
|  | Cluster:4-0 | |  |  | 2 | 238 | 1 | 4422 | 0.0076462985 | 0.015292597 | 2 |
|  |  | RAFL06-07-B07 | At5g55190 / GTP-binding protein atran3, putative | |  |  |  |  |  | | --- | --- | --- | --- | --- | |  |  |  |  |  | | K01G5.4 | | | | | | |
|  |  | RAFL05-17-C16 | At5g55190 / GTP-binding protein atran3, putative | |  |  |  |  |  | | --- | --- | --- | --- | --- | |  |  |  |  |  | | K01G5.4 | | | | | | |
| R12B2.1 | | | WI5\_id:R12B2.1 |  | A | B | C | D | P | P' | N |
|  | Cluster:5-0 | |  |  | 4 | 73 | 6 | 4580 | 1.3392871E-5 | 8.0357226E-5 | 6 |
|  |  | RAFL04-15-M13 | At5g56030 / heat shock protein 81-2 (HSP81-2) | |  |  |  |  |  | | --- | --- | --- | --- | --- | |  |  |  |  |  | | C47E8.5 | | | | | | |
|  |  | RAFL07-13-H08 | At5g56010 / heat shock protein, putative | |  |  |  |  |  | | --- | --- | --- | --- | --- | |  |  |  |  |  | | C47E8.5 | | | | | | |
|  |  | RAFL09-06-O18 | At5g56030 / heat shock protein 81-2 (HSP81-2) | |  |  |  |  |  | | --- | --- | --- | --- | --- | |  |  |  |  |  | | C47E8.5 | | | | | | |
|  |  | RAFL05-16-L15 | At5g56010 / heat shock protein, putative | |  |  |  |  |  | | --- | --- | --- | --- | --- | |  |  |  |  |  | | C47E8.5 | | | | | | |
| T19A6.3 | | | WI5\_id:T19A6.3 |  | A | B | C | D | P | P' | N |
|  | Cluster:8-0 | |  |  | 1 | 108 | 0 | 4554 | 0.02337551 | 0.02337551 | 1 |
|  |  | RAFL05-03-L12 | At1g47128 / cysteine proteinase RD21A | |  |  |  |  |  | | --- | --- | --- | --- | --- | |  |  |  |  |  | | T03E6.7 | | | | | | |
| F48E3.3 | | | WI5\_id:F48E3.3 |  | A | B | C | D | P | P' | N |
|  | Cluster:8-0 | |  |  | 1 | 108 | 1 | 4553 | 0.0462095 | 0.092419 | 2 |
|  |  | RAFL09-13-J20 | At1g69410 / Eukaryotic initiation factor 5A -related | |  |  |  |  |  | | --- | --- | --- | --- | --- | |  |  |  |  |  | | T05G5.10 | | | | | | |
| C23G10.4 | | | WI5\_id:C23G10.4 |  | A | B | C | D | P | P' | N |
|  | Cluster:2-0 | |  |  | 2 | 148 | 5 | 4508 | 0.019410595 | 0.09705298 | 5 |
|  |  | RAFL04-20-D09 | At5g42020 / luminal binding protein 2 precursor (BiP-2) (AtBP2) | |  |  |  |  |  | | --- | --- | --- | --- | --- | |  |  |  |  |  | | C15H9.6 | | | | | | |
|  |  | RAFL03-08-O03 | At1g07940 / elongation factor 1-alpha (EF-1-alpha) | |  |  |  |  |  | | --- | --- | --- | --- | --- | |  |  |  |  |  | | F31E3.5 | | | | | | |
|  | Cluster:2-1 | |  |  | 2 | 242 | 5 | 4414 | 0.04810191 | 0.24050954 | 5 |
|  |  | RAFL06-08-N05 | At5g28540 / luminal binding protein 1 precursor (BiP-1) (AtBP1) | |  |  |  |  |  | | --- | --- | --- | --- | --- | |  |  |  |  |  | | C15H9.6 | | | | | | |
|  |  | RAFL07-11-O19 | At5g28540 / luminal binding protein 1 precursor (BiP-1) (AtBP1) | |  |  |  |  |  | | --- | --- | --- | --- | --- | |  |  |  |  |  | | C15H9.6 | | | | | | |
| W08F4.8 | | | WI5\_id:W08F4.8 |  | A | B | C | D | P | P' | N |
|  | Cluster:5-0 | |  |  | 4 | 73 | 1 | 4585 | 3.3961462E-7 | 6.7922923E-7 | 2 |
|  |  | RAFL04-15-M13 | At5g56030 / heat shock protein 81-2 (HSP81-2) | |  |  |  |  |  | | --- | --- | --- | --- | --- | |  |  |  |  |  | | C47E8.5 | | | | | | |
|  |  | RAFL07-13-H08 | At5g56010 / heat shock protein, putative | |  |  |  |  |  | | --- | --- | --- | --- | --- | |  |  |  |  |  | | C47E8.5 | | | | | | |
|  |  | RAFL09-06-O18 | At5g56030 / heat shock protein 81-2 (HSP81-2) | |  |  |  |  |  | | --- | --- | --- | --- | --- | |  |  |  |  |  | | C47E8.5 | | | | | | |
|  |  | RAFL05-16-L15 | At5g56010 / heat shock protein, putative | |  |  |  |  |  | | --- | --- | --- | --- | --- | |  |  |  |  |  | | C47E8.5 | | | | | | |
| C36E8.5 | | | WI5\_id:C36E8.5 |  | A | B | C | D | P | P' | N |
|  | Cluster:1-0 | |  |  | 2 | 147 | 4 | 4510 | 0.013980698 | 0.05592279 | 4 |
|  |  | RAFL09-11-P11 | At1g20010 / tubulin beta-5 chain (TUB5) | |  |  |  |  |  | | --- | --- | --- | --- | --- | |  |  |  |  |  | | C36E8.5 | | | | | | |
|  |  | RAFL04-16-G05 | At5g44340 / tubulin beta-4 chain (TUB4) | |  |  |  |  |  | | --- | --- | --- | --- | --- | |  |  |  |  |  | | C36E8.5 | | | | | | |
|  | Cluster:2-1 | |  |  | 2 | 242 | 4 | 4415 | 0.0355681 | 0.1422724 | 4 |
|  |  | RAFL05-09-F01 | At5g44340 / tubulin beta-4 chain (TUB4) | |  |  |  |  |  | | --- | --- | --- | --- | --- | |  |  |  |  |  | | C36E8.5 | | | | | | |
|  |  | RAFL06-12-I16 | At2g29550 / tubulin beta-7 chain (TUB7) | |  |  |  |  |  | | --- | --- | --- | --- | --- | |  |  |  |  |  | | C36E8.5 | | | | | | |
| F10G7.4 | | | WI5\_id:F10G7.4 |  | A | B | C | D | P | P' | N |
|  | Cluster:8-0 | |  |  | 1 | 108 | 1 | 4553 | 0.0462095 | 0.092419 | 2 |
|  |  | RAFL05-03-L12 | At1g47128 / cysteine proteinase RD21A | |  |  |  |  |  | | --- | --- | --- | --- | --- | |  |  |  |  |  | | T03E6.7 | | | | | | |
| CD4.6 | | | WI5\_id:CD4.6 |  | A | B | C | D | P | P' | N |
|  | Cluster:4-1 | |  |  | 3 | 307 | 2 | 4351 | 0.0026315711 | 0.0052631423 | 2 |
|  |  | RAFL05-01-C14 | At5g42790 / 20S proteasome alpha subunit F1 (PAF1) | |  |  |  |  |  | | --- | --- | --- | --- | --- | |  |  |  |  |  | | CD4.6 | | | | | | |
|  |  | RAFL05-04-L16 | At1g79210 / 20S proteasome alpha subunit B, putative | |  |  |  |  |  | | --- | --- | --- | --- | --- | |  |  |  |  |  | | D1054.2 | | | | | | |
|  |  | RAFL05-12-G18 | At4g14800 / 20S proteasome beta subunit D2 (PBD2) | |  |  |  |  |  | | --- | --- | --- | --- | --- | |  |  |  |  |  | | T20F5.2 | | | | | | |
|  | Cluster:6-1 | |  |  | 2 | 313 | 3 | 4345 | 0.039689664 | 0.07937933 | 2 |
|  |  | RAFL06-16-H22 | At1g16470 / 20S proteasome alpha subunit B (PAB1) | |  |  |  |  |  | | --- | --- | --- | --- | --- | |  |  |  |  |  | | D1054.2 | | | | | | |
|  |  | RAFL06-12-E24 | At5g42790 / 20S proteasome alpha subunit F1 (PAF1) | |  |  |  |  |  | | --- | --- | --- | --- | --- | |  |  |  |  |  | | CD4.6 | | | | | | |
| C16A3.8 | | | WI5\_id:C16A3.8 |  | A | B | C | D | P | P' | N |
|  | Cluster:4-2 | |  |  | 1 | 134 | 0 | 4528 | 0.028951319 | 0.028951319 | 1 |
|  |  | RAFL04-20-K07 | At3g53750 / actin (ACT3) | |  |  |  |  |  | | --- | --- | --- | --- | --- | |  |  |  |  |  | | T04C12.6 | | | | | | |
| C16A3.9 | | | WI5\_id:C16A3.9 |  | A | B | C | D | P | P' | N |
|  | Cluster:2-0 | |  |  | 2 | 148 | 3 | 4510 | 0.009643516 | 0.028930547 | 3 |
|  |  | RAFL06-08-H02 | At3g04840 / 40S ribosomal protein S3A (RPS3aA) | |  |  |  |  |  | | --- | --- | --- | --- | --- | |  |  |  |  |  | | F56F3.5 | | | | | | |
|  |  | RAFL03-06-H09 | At3g60770 / 40S ribosomal protein S13 (RPS13A) | |  |  |  |  |  | | --- | --- | --- | --- | --- | |  |  |  |  |  | | C16A3.9  40S ribosomal protein S13 | | | | | | |
|  | Cluster:2-1 | |  |  | 2 | 242 | 3 | 4416 | 0.024550742 | 0.07365222 | 3 |
|  |  | RAFL07-15-K08 | At4g34670 / 40S ribosomal protein S3A (RPS3aB) | |  |  |  |  |  | | --- | --- | --- | --- | --- | |  |  |  |  |  | | F56F3.5 | | | | | | |
|  |  | RAFL06-14-I03 | At3g60770 / 40S ribosomal protein S13 (RPS13A) | |  |  |  |  |  | | --- | --- | --- | --- | --- | |  |  |  |  |  | | C16A3.9  40S ribosomal protein S13 | | | | | | |
| F53G2.6 | | | WI5\_id:F53G2.6 |  | A | B | C | D | P | P' | N |
|  | Cluster:4-0 | |  |  | 2 | 238 | 1 | 4422 | 0.0076462985 | 0.015292597 | 2 |
|  |  | RAFL06-07-B07 | At5g55190 / GTP-binding protein atran3, putative | |  |  |  |  |  | | --- | --- | --- | --- | --- | |  |  |  |  |  | | K01G5.4 | | | | | | |
|  |  | RAFL05-17-C16 | At5g55190 / GTP-binding protein atran3, putative | |  |  |  |  |  | | --- | --- | --- | --- | --- | |  |  |  |  |  | | K01G5.4 | | | | | | |
| ZK742.1 | | | WI5\_id:ZK742.1 |  | A | B | C | D | P | P' | N |
|  | Cluster:4-0 | |  |  | 2 | 238 | 1 | 4422 | 0.0076462985 | 0.015292597 | 2 |
|  |  | RAFL06-07-B07 | At5g55190 / GTP-binding protein atran3, putative | |  |  |  |  |  | | --- | --- | --- | --- | --- | |  |  |  |  |  | | K01G5.4 | | | | | | |
|  |  | RAFL05-17-C16 | At5g55190 / GTP-binding protein atran3, putative | |  |  |  |  |  | | --- | --- | --- | --- | --- | |  |  |  |  |  | | K01G5.4 | | | | | | |
| T24H10.3 | | | WI5\_id:T24H10.3 |  | A | B | C | D | P | P' | N |
|  | Cluster:2-0 | |  |  | 1 | 149 | 0 | 4513 | 0.03216813 | 0.03216813 | 1 |
|  |  | RAFL05-02-F20 | At5g65360 / histone H3 | |  |  |  |  |  | | --- | --- | --- | --- | --- | |  |  |  |  |  | | Y49E10.6 | | | | | | |
| F15C11.2 | | | WI5\_id:F15C11.2 |  | A | B | C | D | P | P' | N |
|  | Cluster:4-2 | |  |  | 1 | 134 | 0 | 4528 | 0.028951319 | 0.028951319 | 1 |
|  |  | RAFL05-05-K13 | At3g52590 / ubiquitin extension protein 1 (UBQ1)/60S ribosomal protein L40 (RPL40B) | |  |  |  |  |  | | --- | --- | --- | --- | --- | |  |  |  |  |  | | ZK1010.1 | | | | | | |
| F52B5.6 | | | WI5\_id:F52B5.6 |  | A | B | C | D | P | P' | N |
|  | Cluster:2-1 | |  |  | 7 | 237 | 13 | 4406 | 4.250125E-5 | 2.550075E-4 | 6 |
|  |  | RAFL02-10-A09 | At1g33140 / 60S ribosomal protein L9 (RPL90A/C) | |  |  |  |  |  | | --- | --- | --- | --- | --- | |  |  |  |  |  | | R13A5.8 | | | | | | |
|  |  | RAFL07-15-M07 | At1g04480 / 60S ribosomal protein L23 (RPL23A) | |  |  |  |  |  | | --- | --- | --- | --- | --- | |  |  |  |  |  | | B0336.10 | | | | | | |
|  |  | RAFL05-17-L17 | At3g55280 / 60S ribosomal protein L23A (RPL23aB) | |  |  |  |  |  | | --- | --- | --- | --- | --- | |  |  |  |  |  | | F52B5.6 | | | | | | |
|  |  | RAFL11-12-H04 | At3g25520 / 60S ribosomal protein L5 (RPL5A) | |  |  |  |  |  | | --- | --- | --- | --- | --- | |  |  |  |  |  | | F54C9.5 | | | | | | |
|  |  | RAFL05-18-P15 | At1g04480 / 60S ribosomal protein L23 (RPL23A) | |  |  |  |  |  | | --- | --- | --- | --- | --- | |  |  |  |  |  | | B0336.10 | | | | | | |
|  |  | RAFL04-18-N22 | At2g44120 / 60S ribosomal protein L7 (RPL7C) | |  |  |  |  |  | | --- | --- | --- | --- | --- | |  |  |  |  |  | | F53G12.10 | | | | | | |
|  |  | RAFL11-07-B21 | At3g25520 / 60S ribosomal protein L5 (RPL5A) | |  |  |  |  |  | | --- | --- | --- | --- | --- | |  |  |  |  |  | | F54C9.5 | | | | | | |
|  | Cluster:3-0 | |  |  | 5 | 228 | 15 | 4415 | 0.0024938595 | 0.0149631575 | 6 |
|  |  | RAFL07-10-D02 | At1g14320 / 60S ribosomal protein L10 (RPL10A)/Wilm's tumor suppressor protein-related | |  |  |  |  |  | | --- | --- | --- | --- | --- | |  |  |  |  |  | | F10B5.1 | | | | | | |
|  |  | RAFL09-10-P09 | At4g36130 / 60S ribosomal protein L8 (RPL8C) | |  |  |  |  |  | | --- | --- | --- | --- | --- | |  |  |  |  |  | | B0250.1 | | | | | | |
|  |  | RAFL08-10-G08 | At3g09630 / 60S ribosomal protein L4/L1 (RPL4A) | |  |  |  |  |  | | --- | --- | --- | --- | --- | |  |  |  |  |  | | B0041.4 | | | | | | |
|  |  | RAFL08-13-M06 | At3g11250 / 60S acidic ribosomal protein P0 (RPP0C) | |  |  |  |  |  | | --- | --- | --- | --- | --- | |  |  |  |  |  | | F25H2.10 | | | | | | |
|  |  | RAFL09-12-B12 | At4g36130 / 60S ribosomal protein L8 (RPL8C) | |  |  |  |  |  | | --- | --- | --- | --- | --- | |  |  |  |  |  | | B0250.1 | | | | | | |
|  | Cluster:2-0 | |  |  | 4 | 146 | 16 | 4497 | 0.0033332263 | 0.019999357 | 6 |
|  |  | RAFL04-19-O24 | At5g02610 / 60S ribosomal protein L35 (RPL35D) | |  |  |  |  |  | | --- | --- | --- | --- | --- | |  |  |  |  |  | | ZK652.4 | | | | | | |
|  |  | RAFL06-10-L13 | At3g09630 / 60S ribosomal protein L4/L1 (RPL4A) | |  |  |  |  |  | | --- | --- | --- | --- | --- | |  |  |  |  |  | | B0041.4 | | | | | | |
|  |  | RAFL11-12-M17 | At3g25520 / 60S ribosomal protein L5 (RPL5A) | |  |  |  |  |  | | --- | --- | --- | --- | --- | |  |  |  |  |  | | F54C9.5 | | | | | | |
|  |  | RAFL03-06-H07 | At1g33140 / 60S ribosomal protein L9 (RPL90A/C) | |  |  |  |  |  | | --- | --- | --- | --- | --- | |  |  |  |  |  | | R13A5.8 | | | | | | |
| T22F3.4 | | | WI5\_id:T22F3.4 |  | A | B | C | D | P | P' | N |
|  | Cluster:3-0 | |  |  | 3 | 230 | 8 | 4422 | 0.015076893 | 0.09046136 | 6 |
|  |  | RAFL07-10-D02 | At1g14320 / 60S ribosomal protein L10 (RPL10A)/Wilm's tumor suppressor protein-related | |  |  |  |  |  | | --- | --- | --- | --- | --- | |  |  |  |  |  | | F10B5.1 | | | | | | |
|  |  | RAFL09-10-P09 | At4g36130 / 60S ribosomal protein L8 (RPL8C) | |  |  |  |  |  | | --- | --- | --- | --- | --- | |  |  |  |  |  | | B0250.1 | | | | | | |
|  |  | RAFL09-12-B12 | At4g36130 / 60S ribosomal protein L8 (RPL8C) | |  |  |  |  |  | | --- | --- | --- | --- | --- | |  |  |  |  |  | | B0250.1 | | | | | | |
|  | Cluster:2-1 | |  |  | 3 | 241 | 8 | 4411 | 0.017076377 | 0.10245825 | 6 |
|  |  | RAFL05-17-L17 | At3g55280 / 60S ribosomal protein L23A (RPL23aB) | |  |  |  |  |  | | --- | --- | --- | --- | --- | |  |  |  |  |  | | F52B5.6 | | | | | | |
|  |  | RAFL11-12-H04 | At3g25520 / 60S ribosomal protein L5 (RPL5A) | |  |  |  |  |  | | --- | --- | --- | --- | --- | |  |  |  |  |  | | F54C9.5 | | | | | | |
|  |  | RAFL11-07-B21 | At3g25520 / 60S ribosomal protein L5 (RPL5A) | |  |  |  |  |  | | --- | --- | --- | --- | --- | |  |  |  |  |  | | F54C9.5 | | | | | | |
|  | Cluster:2-0 | |  |  | 2 | 148 | 9 | 4504 | 0.046735216 | 0.2804113 | 6 |
|  |  | RAFL04-19-O24 | At5g02610 / 60S ribosomal protein L35 (RPL35D) | |  |  |  |  |  | | --- | --- | --- | --- | --- | |  |  |  |  |  | | ZK652.4 | | | | | | |
|  |  | RAFL11-12-M17 | At3g25520 / 60S ribosomal protein L5 (RPL5A) | |  |  |  |  |  | | --- | --- | --- | --- | --- | |  |  |  |  |  | | F54C9.5 | | | | | | |
| F38A6.1 | | | WI5\_id:F38A6.1 |  | A | B | C | D | P | P' | N |
|  | Cluster:2-0 | |  |  | 2 | 148 | 2 | 4511 | 0.00591059 | 0.017731769 | 3 |
|  |  | RAFL07-09-P18 | At4g31700 / 40S ribosomal protein S6 (RPS6A) | |  |  |  |  |  | | --- | --- | --- | --- | --- | |  |  |  |  |  | | Y71A12B.1 | | | | | | |
|  |  | RAFL06-08-H02 | At3g04840 / 40S ribosomal protein S3A (RPS3aA) | |  |  |  |  |  | | --- | --- | --- | --- | --- | |  |  |  |  |  | | F56F3.5 | | | | | | |
| C06G3.6 | | | WI5\_id:C06G3.6 |  | A | B | C | D | P | P' | N |
|  | Cluster:5-0 | |  |  | 1 | 76 | 0 | 4586 | 0.016512975 | 0.016512975 | 1 |
|  |  | RAFL06-07-P18 | At3g60820 / 20S proteasome beta subunit F1 (PBF1) | |  |  |  |  |  | | --- | --- | --- | --- | --- | |  |  |  |  |  | | C02F5.9 | | | | | | |
| T20B12.8 | | | WI5\_id:T20B12.8 |  | A | B | C | D | P | P' | N |
|  | Cluster:6-2 | |  |  | 1 | 175 | 0 | 4487 | 0.03774394 | 0.03774394 | 1 |
|  |  | RAFL11-07-O15 | At3g28730 / recombination signal sequence recognition protein -related | |  |  |  |  |  | | --- | --- | --- | --- | --- | |  |  |  |  |  | | T20B12.8 | | | | | | |
| F42A10.3 | | | WI5\_id:F42A10.3 |  | A | B | C | D | P | P' | N |
|  | Cluster:5-0 | |  |  | 4 | 73 | 1 | 4585 | 3.3961462E-7 | 6.7922923E-7 | 2 |
|  |  | RAFL04-15-M13 | At5g56030 / heat shock protein 81-2 (HSP81-2) | |  |  |  |  |  | | --- | --- | --- | --- | --- | |  |  |  |  |  | | C47E8.5 | | | | | | |
|  |  | RAFL07-13-H08 | At5g56010 / heat shock protein, putative | |  |  |  |  |  | | --- | --- | --- | --- | --- | |  |  |  |  |  | | C47E8.5 | | | | | | |
|  |  | RAFL09-06-O18 | At5g56030 / heat shock protein 81-2 (HSP81-2) | |  |  |  |  |  | | --- | --- | --- | --- | --- | |  |  |  |  |  | | C47E8.5 | | | | | | |
|  |  | RAFL05-16-L15 | At5g56010 / heat shock protein, putative | |  |  |  |  |  | | --- | --- | --- | --- | --- | |  |  |  |  |  | | C47E8.5 | | | | | | |
| Y37E3.7 | | | WI5\_id:Y37E3.7 |  | A | B | C | D | P | P' | N |
|  | Cluster:3-0 | |  |  | 1 | 232 | 0 | 4430 | 0.049967833 | 0.049967833 | 1 |
|  |  | RAFL08-13-M06 | At3g11250 / 60S acidic ribosomal protein P0 (RPP0C) | |  |  |  |  |  | | --- | --- | --- | --- | --- | |  |  |  |  |  | | F25H2.10 | | | | | | |
| C47B2.3 | | | WI5\_id:C47B2.3 |  | A | B | C | D | P | P' | N |
|  | Cluster:1-0 | |  |  | 2 | 147 | 4 | 4510 | 0.013980698 | 0.05592279 | 4 |
|  |  | RAFL09-11-P11 | At1g20010 / tubulin beta-5 chain (TUB5) | |  |  |  |  |  | | --- | --- | --- | --- | --- | |  |  |  |  |  | | B0272.1  tubulin beta chain | | | | | | |
|  |  | RAFL04-16-G05 | At5g44340 / tubulin beta-4 chain (TUB4) | |  |  |  |  |  | | --- | --- | --- | --- | --- | |  |  |  |  |  | | B0272.1  tubulin beta chain | | | | | | |
|  | Cluster:2-1 | |  |  | 2 | 242 | 4 | 4415 | 0.0355681 | 0.1422724 | 4 |
|  |  | RAFL05-09-F01 | At5g44340 / tubulin beta-4 chain (TUB4) | |  |  |  |  |  | | --- | --- | --- | --- | --- | |  |  |  |  |  | | B0272.1  tubulin beta chain | | | | | | |
|  |  | RAFL06-12-I16 | At2g29550 / tubulin beta-7 chain (TUB7) | |  |  |  |  |  | | --- | --- | --- | --- | --- | |  |  |  |  |  | | B0272.1  tubulin beta chain | | | | | | |
| H02I12.5 | | | WI5\_id:H02I12.5 |  | A | B | C | D | P | P' | N |
|  | Cluster:6-2 | |  |  | 1 | 175 | 0 | 4487 | 0.03774394 | 0.03774394 | 1 |
|  |  | RAFL11-07-O15 | At3g28730 / recombination signal sequence recognition protein -related | |  |  |  |  |  | | --- | --- | --- | --- | --- | |  |  |  |  |  | | T20B12.8 | | | | | | |
| F37E3.1 | | | WI5\_id:F37E3.1 |  | A | B | C | D | P | P' | N |
|  | Cluster:1-0 | |  |  | 1 | 148 | 0 | 4514 | 0.031953678 | 0.031953678 | 1 |
|  |  | RAFL04-19-J20 | At3g50270 / hydroxycinnamoyl/benzoyltransferase-related | |  |  |  |  |  | | --- | --- | --- | --- | --- | |  |  |  |  |  | | K04G7.10 | | | | | | |
| D1054.2 | | | WI5\_id:D1054.2 |  | A | B | C | D | P | P' | N |
|  | Cluster:4-1 | |  |  | 7 | 303 | 7 | 4346 | 1.22729925E-5 | 7.363795E-5 | 6 |
|  |  | RAFL04-15-N11 | At3g22110 / 20S proteasome alpha subunit C (PAC1) | |  |  |  |  |  | | --- | --- | --- | --- | --- | |  |  |  |  |  | | Y110A7A.14 | | | | | | |
|  |  | RAFL05-01-C14 | At5g42790 / 20S proteasome alpha subunit F1 (PAF1) | |  |  |  |  |  | | --- | --- | --- | --- | --- | |  |  |  |  |  | | CD4.6 | | | | | | |
|  |  | RAFL07-08-J17 | At1g13060 / 20S proteasome beta subunit E1 (PBE1) | |  |  |  |  |  | | --- | --- | --- | --- | --- | |  |  |  |  |  | | K05C4.1 | | | | | | |
|  |  | RAFL05-04-G23 | At1g77440 / 20S proteasome beta subunit C (PBC2) | |  |  |  |  |  | | --- | --- | --- | --- | --- | |  |  |  |  |  | | Y38A8.2 | | | | | | |
|  |  | RAFL05-04-L16 | At1g79210 / 20S proteasome alpha subunit B, putative | |  |  |  |  |  | | --- | --- | --- | --- | --- | |  |  |  |  |  | | D1054.2 | | | | | | |
|  |  | RAFL05-12-G18 | At4g14800 / 20S proteasome beta subunit D2 (PBD2) | |  |  |  |  |  | | --- | --- | --- | --- | --- | |  |  |  |  |  | | T20F5.2 | | | | | | |
|  |  | RAFL05-21-D23 | At1g21720 / 20S proteasome beta subunit C (PBC1) | |  |  |  |  |  | | --- | --- | --- | --- | --- | |  |  |  |  |  | | Y38A8.2 | | | | | | |
| C08E3.9 | | | WI5\_id:C08E3.9 |  | A | B | C | D | P | P' | N |
|  | Cluster:5-0 | |  |  | 1 | 76 | 0 | 4586 | 0.016512975 | 0.016512975 | 1 |
|  |  | RAFL06-07-P18 | At3g60820 / 20S proteasome beta subunit F1 (PBF1) | |  |  |  |  |  | | --- | --- | --- | --- | --- | |  |  |  |  |  | | C02F5.9 | | | | | | |
| F53A3.3 | | | WI5\_id:F53A3.3 |  | A | B | C | D | P | P' | N |
|  | Cluster:5-0 | |  |  | 4 | 73 | 11 | 4575 | 8.174753E-5 | 4.904852E-4 | 6 |
|  |  | RAFL04-15-M13 | At5g56030 / heat shock protein 81-2 (HSP81-2) | |  |  |  |  |  | | --- | --- | --- | --- | --- | |  |  |  |  |  | | C47E8.5 | | | | | | |
|  |  | RAFL07-13-H08 | At5g56010 / heat shock protein, putative | |  |  |  |  |  | | --- | --- | --- | --- | --- | |  |  |  |  |  | | C47E8.5 | | | | | | |
|  |  | RAFL09-06-O18 | At5g56030 / heat shock protein 81-2 (HSP81-2) | |  |  |  |  |  | | --- | --- | --- | --- | --- | |  |  |  |  |  | | C47E8.5 | | | | | | |
|  |  | RAFL05-16-L15 | At5g56010 / heat shock protein, putative | |  |  |  |  |  | | --- | --- | --- | --- | --- | |  |  |  |  |  | | C47E8.5 | | | | | | |
|  | Cluster:2-1 | |  |  | 4 | 240 | 11 | 4408 | 0.006317224 | 0.037903342 | 6 |
|  |  | RAFL04-18-N10 | At1g07770 / 40S ribosomal protein S15A (RPS15aA) | |  |  |  |  |  | | --- | --- | --- | --- | --- | |  |  |  |  |  | | F53A3.3 | | | | | | |
|  |  | RAFL07-13-J18 | At2g37270 / 40S ribosomal protein S5 (RPS5A) | |  |  |  |  |  | | --- | --- | --- | --- | --- | |  |  |  |  |  | | T05E11.1 | | | | | | |
|  |  | RAFL05-16-H14 | At1g04270 / 40S ribosomal protein S15 (RPS15A) | |  |  |  |  |  | | --- | --- | --- | --- | --- | |  |  |  |  |  | | F36A2.6 | | | | | | |
|  |  | RAFL04-19-M20 | At1g58380 / 40S ribosomal protein S2 (RPS2A) | |  |  |  |  |  | | --- | --- | --- | --- | --- | |  |  |  |  |  | | C49H3.11 | | | | | | |
|  | Cluster:3-0 | |  |  | 3 | 230 | 12 | 4418 | 0.03588357 | 0.21530141 | 6 |
|  |  | RAFL08-09-E20 | At2g41840 / 40S ribosomal protein S2 (RPS2C) | |  |  |  |  |  | | --- | --- | --- | --- | --- | |  |  |  |  |  | | C49H3.11 | | | | | | |
|  |  | RAFL11-04-A02 | At1g07770 / 40S ribosomal protein S15A (RPS15aA) | |  |  |  |  |  | | --- | --- | --- | --- | --- | |  |  |  |  |  | | F53A3.3 | | | | | | |
|  |  | RAFL06-07-B02 | At3g11940 / 40S ribosomal protein S5 (RPS5B) | |  |  |  |  |  | | --- | --- | --- | --- | --- | |  |  |  |  |  | | T05E11.1 | | | | | | |
| T17H7.4 | | | WI5\_id:T17H7.4 |  | A | B | C | D | P | P' | N |
|  | Cluster:1-2 | |  |  | 2 | 172 | 2 | 4487 | 0.007905076 | 0.023715228 | 3 |
|  |  | RAFL05-04-J09 | At5g45775 / 60S ribosomal protein L11 (RPL11D) | |  |  |  |  |  | | --- | --- | --- | --- | --- | |  |  |  |  |  | | F07D10.1 | | | | | | |
|  |  | RAFL11-10-E06 | At5g45775 / 60S ribosomal protein L11 (RPL11D) | |  |  |  |  |  | | --- | --- | --- | --- | --- | |  |  |  |  |  | | F07D10.1 | | | | | | |
| ZK652.4 | | | WI5\_id:ZK652.4 |  | A | B | C | D | P | P' | N |
|  | Cluster:2-1 | |  |  | 6 | 238 | 13 | 4406 | 2.9421985E-4 | 0.001765319 | 6 |
|  |  | RAFL02-10-A09 | At1g33140 / 60S ribosomal protein L9 (RPL90A/C) | |  |  |  |  |  | | --- | --- | --- | --- | --- | |  |  |  |  |  | | R13A5.8 | | | | | | |
|  |  | RAFL07-15-M07 | At1g04480 / 60S ribosomal protein L23 (RPL23A) | |  |  |  |  |  | | --- | --- | --- | --- | --- | |  |  |  |  |  | | B0336.10 | | | | | | |
|  |  | RAFL05-17-L17 | At3g55280 / 60S ribosomal protein L23A (RPL23aB) | |  |  |  |  |  | | --- | --- | --- | --- | --- | |  |  |  |  |  | | F52B5.6 | | | | | | |
|  |  | RAFL11-12-H04 | At3g25520 / 60S ribosomal protein L5 (RPL5A) | |  |  |  |  |  | | --- | --- | --- | --- | --- | |  |  |  |  |  | | F54C9.5 | | | | | | |
|  |  | RAFL05-18-P15 | At1g04480 / 60S ribosomal protein L23 (RPL23A) | |  |  |  |  |  | | --- | --- | --- | --- | --- | |  |  |  |  |  | | B0336.10 | | | | | | |
|  |  | RAFL11-07-B21 | At3g25520 / 60S ribosomal protein L5 (RPL5A) | |  |  |  |  |  | | --- | --- | --- | --- | --- | |  |  |  |  |  | | F54C9.5 | | | | | | |
|  | Cluster:3-0 | |  |  | 5 | 228 | 14 | 4416 | 0.0019486465 | 0.011691879 | 6 |
|  |  | RAFL07-10-D02 | At1g14320 / 60S ribosomal protein L10 (RPL10A)/Wilm's tumor suppressor protein-related | |  |  |  |  |  | | --- | --- | --- | --- | --- | |  |  |  |  |  | | F10B5.1 | | | | | | |
|  |  | RAFL09-10-P09 | At4g36130 / 60S ribosomal protein L8 (RPL8C) | |  |  |  |  |  | | --- | --- | --- | --- | --- | |  |  |  |  |  | | B0250.1 | | | | | | |
|  |  | RAFL08-10-G08 | At3g09630 / 60S ribosomal protein L4/L1 (RPL4A) | |  |  |  |  |  | | --- | --- | --- | --- | --- | |  |  |  |  |  | | B0041.4 | | | | | | |
|  |  | RAFL08-13-M06 | At3g11250 / 60S acidic ribosomal protein P0 (RPP0C) | |  |  |  |  |  | | --- | --- | --- | --- | --- | |  |  |  |  |  | | F25H2.10 | | | | | | |
|  |  | RAFL09-12-B12 | At4g36130 / 60S ribosomal protein L8 (RPL8C) | |  |  |  |  |  | | --- | --- | --- | --- | --- | |  |  |  |  |  | | B0250.1 | | | | | | |
|  | Cluster:2-0 | |  |  | 4 | 146 | 15 | 4498 | 0.002734157 | 0.016404942 | 6 |
|  |  | RAFL04-19-O24 | At5g02610 / 60S ribosomal protein L35 (RPL35D) | |  |  |  |  |  | | --- | --- | --- | --- | --- | |  |  |  |  |  | | ZK652.4 | | | | | | |
|  |  | RAFL06-10-L13 | At3g09630 / 60S ribosomal protein L4/L1 (RPL4A) | |  |  |  |  |  | | --- | --- | --- | --- | --- | |  |  |  |  |  | | B0041.4 | | | | | | |
|  |  | RAFL11-12-M17 | At3g25520 / 60S ribosomal protein L5 (RPL5A) | |  |  |  |  |  | | --- | --- | --- | --- | --- | |  |  |  |  |  | | F54C9.5 | | | | | | |
|  |  | RAFL03-06-H07 | At1g33140 / 60S ribosomal protein L9 (RPL90A/C) | |  |  |  |  |  | | --- | --- | --- | --- | --- | |  |  |  |  |  | | R13A5.8 | | | | | | |
| ZC411.1 | | | WI5\_id:ZC411.1 |  | A | B | C | D | P | P' | N |
|  | Cluster:5-0 | |  |  | 1 | 76 | 0 | 4586 | 0.016512975 | 0.016512975 | 1 |
|  |  | RAFL06-07-P18 | At3g60820 / 20S proteasome beta subunit F1 (PBF1) | |  |  |  |  |  | | --- | --- | --- | --- | --- | |  |  |  |  |  | | C02F5.9 | | | | | | |
| B0019.3 | | | WI5\_id:B0019.3 |  | A | B | C | D | P | P' | N |
|  | Cluster:4-0 | |  |  | 2 | 238 | 1 | 4422 | 0.0076462985 | 0.015292597 | 2 |
|  |  | RAFL06-07-B07 | At5g55190 / GTP-binding protein atran3, putative | |  |  |  |  |  | | --- | --- | --- | --- | --- | |  |  |  |  |  | | K01G5.4 | | | | | | |
|  |  | RAFL05-17-C16 | At5g55190 / GTP-binding protein atran3, putative | |  |  |  |  |  | | --- | --- | --- | --- | --- | |  |  |  |  |  | | K01G5.4 | | | | | | |
| F01F1.6 | | | WI5\_id:F01F1.6 |  | A | B | C | D | P | P' | N |
|  | Cluster:9-0 | |  |  | 4 | 28 | 0 | 4631 | 1.8277981E-9 | 1.8277981E-9 | 1 |
|  |  | RAFL08-09-C23 | At1g54100 / aldehyde dehydrogenase, putative (ALDH) | |  |  |  |  |  | | --- | --- | --- | --- | --- | |  |  |  |  |  | | F01F1.6 | | | | | | |
|  |  | RAFL04-09-D07 | At1g54100 / aldehyde dehydrogenase, putative (ALDH) | |  |  |  |  |  | | --- | --- | --- | --- | --- | |  |  |  |  |  | | F01F1.6 | | | | | | |
|  |  | RAFL05-21-E06 | At1g54100 / aldehyde dehydrogenase, putative (ALDH) | |  |  |  |  |  | | --- | --- | --- | --- | --- | |  |  |  |  |  | | F01F1.6 | | | | | | |
|  |  | RAFL08-15-L09 | At1g54100 / aldehyde dehydrogenase, putative (ALDH) | |  |  |  |  |  | | --- | --- | --- | --- | --- | |  |  |  |  |  | | F01F1.6 | | | | | | |
| T01H3.1 | | | WI5\_id:T01H3.1 |  | A | B | C | D | P | P' | N |
|  | Cluster:4-1 | |  |  | 2 | 308 | 2 | 4351 | 0.024166461 | 0.07249938 | 3 |
|  |  | RAFL04-09-A19 | At4g38510 / probable H+-transporting ATPase | |  |  |  |  |  | | --- | --- | --- | --- | --- | |  |  |  |  |  | | F20B6.2 | | | | | | |
|  |  | RAFL07-17-H08 | At4g38510 / probable H+-transporting ATPase | |  |  |  |  |  | | --- | --- | --- | --- | --- | |  |  |  |  |  | | F20B6.2 | | | | | | |
| B0280.5 | | | WI5\_id:B0280.5 |  | A | B | C | D | P | P' | N |
|  | Cluster:4-0 | |  |  | 2 | 238 | 1 | 4422 | 0.0076462985 | 0.015292597 | 2 |
|  |  | RAFL06-07-B07 | At5g55190 / GTP-binding protein atran3, putative | |  |  |  |  |  | | --- | --- | --- | --- | --- | |  |  |  |  |  | | K01G5.4 | | | | | | |
|  |  | RAFL05-17-C16 | At5g55190 / GTP-binding protein atran3, putative | |  |  |  |  |  | | --- | --- | --- | --- | --- | |  |  |  |  |  | | K01G5.4 | | | | | | |
| F58A4.8 | | | WI5\_id:F58A4.8 |  | A | B | C | D | P | P' | N |
|  | Cluster:2-1 | |  |  | 3 | 241 | 7 | 4412 | 0.012910854 | 0.064554274 | 5 |
|  |  | RAFL05-09-F01 | At5g44340 / tubulin beta-4 chain (TUB4) | |  |  |  |  |  | | --- | --- | --- | --- | --- | |  |  |  |  |  | | C36E8.5 | | | | | | |
|  |  | RAFL06-12-I16 | At2g29550 / tubulin beta-7 chain (TUB7) | |  |  |  |  |  | | --- | --- | --- | --- | --- | |  |  |  |  |  | | C36E8.5 | | | | | | |
|  |  | RAFL06-14-I03 | At3g60770 / 40S ribosomal protein S13 (RPS13A) | |  |  |  |  |  | | --- | --- | --- | --- | --- | |  |  |  |  |  | | C16A3.9 | | | | | | |
|  | Cluster:1-0 | |  |  | 2 | 147 | 8 | 4506 | 0.038570926 | 0.19285463 | 5 |
|  |  | RAFL09-11-P11 | At1g20010 / tubulin beta-5 chain (TUB5) | |  |  |  |  |  | | --- | --- | --- | --- | --- | |  |  |  |  |  | | C36E8.5 | | | | | | |
|  |  | RAFL04-16-G05 | At5g44340 / tubulin beta-4 chain (TUB4) | |  |  |  |  |  | | --- | --- | --- | --- | --- | |  |  |  |  |  | | C36E8.5 | | | | | | |
|  | Cluster:2-0 | |  |  | 2 | 148 | 8 | 4505 | 0.039047353 | 0.19523676 | 5 |
|  |  | RAFL03-06-H09 | At3g60770 / 40S ribosomal protein S13 (RPS13A) | |  |  |  |  |  | | --- | --- | --- | --- | --- | |  |  |  |  |  | | C16A3.9 | | | | | | |
|  |  | RAFL04-19-F02 | At4g20890 / tubulin beta-9 chain (TUB9) | |  |  |  |  |  | | --- | --- | --- | --- | --- | |  |  |  |  |  | | C36E8.5 | | | | | | |
| T07C4.1 | | | WI5\_id:T07C4.1 |  | A | B | C | D | P | P' | N |
|  | Cluster:4-2 | |  |  | 2 | 133 | 5 | 4523 | 0.015882019 | 0.0794101 | 5 |
|  |  | RAFL09-17-F16 | At1g07770 / 40S ribosomal protein S15A (RPS15aA) | |  |  |  |  |  | | --- | --- | --- | --- | --- | |  |  |  |  |  | | F53A3.3 | | | | | | |
|  |  | RAFL11-02-D23 | At1g07770 / 40S ribosomal protein S15A (RPS15aA) | |  |  |  |  |  | | --- | --- | --- | --- | --- | |  |  |  |  |  | | F53A3.3 | | | | | | |
|  | Cluster:2-1 | |  |  | 2 | 242 | 5 | 4414 | 0.04810191 | 0.24050954 | 5 |
|  |  | RAFL04-18-N10 | At1g07770 / 40S ribosomal protein S15A (RPS15aA) | |  |  |  |  |  | | --- | --- | --- | --- | --- | |  |  |  |  |  | | F53A3.3 | | | | | | |
|  |  | RAFL07-15-K08 | At4g34670 / 40S ribosomal protein S3A (RPS3aB) | |  |  |  |  |  | | --- | --- | --- | --- | --- | |  |  |  |  |  | | F56F3.5  Ribosomal protein S3a (human) homolog. | | | | | | |
| C47D12.2 | | | WI5\_id:C47D12.2 |  | A | B | C | D | P | P' | N |
|  | Cluster:6-1 | |  |  | 2 | 313 | 2 | 4346 | 0.024916494 | 0.074749485 | 3 |
|  |  | RAFL09-13-A13 | At2g44100 / GDP dissociation inhibitor | |  |  |  |  |  | | --- | --- | --- | --- | --- | |  |  |  |  |  | | Y57G11C.10 | | | | | | |
|  |  | RAFL05-11-L05 | At2g44100 / GDP dissociation inhibitor | |  |  |  |  |  | | --- | --- | --- | --- | --- | |  |  |  |  |  | | Y57G11C.10 | | | | | | |
| F57B9.10 | | | WI5\_id:F57B9.10 |  | A | B | C | D | P | P' | N |
|  | Cluster:6-1 | |  |  | 4 | 311 | 14 | 4334 | 0.029376008 | 0.3525121 | 12 |
|  |  | RAFL08-19-N19 | At3g05530 / 26S proteasome AAA-ATPase subunit RPT5a | |  |  |  |  |  | | --- | --- | --- | --- | --- | |  |  |  |  |  | | F56H1.4 | | | | | | |
|  |  | RAFL05-08-I15 | At4g24820 / 26S proteasome regulatory subunit (RPN7), putative | |  |  |  |  |  | | --- | --- | --- | --- | --- | |  |  |  |  |  | | F49C12.8 | | | | | | |
|  |  | RAFL07-13-D20 | At4g24820 / 26S proteasome regulatory subunit (RPN7), putative | |  |  |  |  |  | | --- | --- | --- | --- | --- | |  |  |  |  |  | | F49C12.8 | | | | | | |
|  |  | RAFL11-04-H03 | At1g20200 / 26S proteasome regulatory subunit S3 (RPN3), putative | |  |  |  |  |  | | --- | --- | --- | --- | --- | |  |  |  |  |  | | C30C11.2 | | | | | | |
| F39B2.2 | | | WI5\_id:F39B2.2 |  | A | B | C | D | P | P' | N |
|  | Cluster:3-0 | |  |  | 3 | 230 | 3 | 4427 | 0.0022016333 | 0.0066048997 | 3 |
|  |  | RAFL04-13-C01 | At1g56070 / elongation factor -related | |  |  |  |  |  | | --- | --- | --- | --- | --- | |  |  |  |  |  | | F25H5.4  Elongation factor Tu family (contains ATP/GTP binding P-loop) | | | | | | |
|  |  | RAFL08-12-J19 | At1g56070 / elongation factor -related | |  |  |  |  |  | | --- | --- | --- | --- | --- | |  |  |  |  |  | | F25H5.4  Elongation factor Tu family (contains ATP/GTP binding P-loop) | | | | | | |
|  |  | RAFL07-10-D07 | At1g56070 / elongation factor -related | |  |  |  |  |  | | --- | --- | --- | --- | --- | |  |  |  |  |  | | F25H5.4  Elongation factor Tu family (contains ATP/GTP binding P-loop) | | | | | | |
|  | Cluster:4-0 | |  |  | 2 | 238 | 4 | 4419 | 0.034489654 | 0.10346896 | 3 |
|  |  | RAFL07-11-O12 | At1g56070 / elongation factor -related | |  |  |  |  |  | | --- | --- | --- | --- | --- | |  |  |  |  |  | | F25H5.4  Elongation factor Tu family (contains ATP/GTP binding P-loop) | | | | | | |
|  |  | RAFL04-17-N12 | At1g56070 / elongation factor -related | |  |  |  |  |  | | --- | --- | --- | --- | --- | |  |  |  |  |  | | F25H5.4  Elongation factor Tu family (contains ATP/GTP binding P-loop) | | | | | | |
| Y71F9AL.13 | | | WI5\_id:Y71F9AL.13 |  | A | B | C | D | P | P' | N |
|  | Cluster:3-0 | |  |  | 3 | 230 | 11 | 4419 | 0.029778728 | 0.23822983 | 8 |
|  |  | RAFL07-10-D02 | At1g14320 / 60S ribosomal protein L10 (RPL10A)/Wilm's tumor suppressor protein-related | |  |  |  |  |  | | --- | --- | --- | --- | --- | |  |  |  |  |  | | F10B5.1 | | | | | | |
|  |  | RAFL09-10-P09 | At4g36130 / 60S ribosomal protein L8 (RPL8C) | |  |  |  |  |  | | --- | --- | --- | --- | --- | |  |  |  |  |  | | B0250.1 | | | | | | |
|  |  | RAFL09-12-B12 | At4g36130 / 60S ribosomal protein L8 (RPL8C) | |  |  |  |  |  | | --- | --- | --- | --- | --- | |  |  |  |  |  | | B0250.1 | | | | | | |
|  | Cluster:2-1 | |  |  | 3 | 241 | 11 | 4408 | 0.033551577 | 0.26841262 | 8 |
|  |  | RAFL05-17-L17 | At3g55280 / 60S ribosomal protein L23A (RPL23aB) | |  |  |  |  |  | | --- | --- | --- | --- | --- | |  |  |  |  |  | | F52B5.6 | | | | | | |
|  |  | RAFL11-12-H04 | At3g25520 / 60S ribosomal protein L5 (RPL5A) | |  |  |  |  |  | | --- | --- | --- | --- | --- | |  |  |  |  |  | | F54C9.5 | | | | | | |
|  |  | RAFL11-07-B21 | At3g25520 / 60S ribosomal protein L5 (RPL5A) | |  |  |  |  |  | | --- | --- | --- | --- | --- | |  |  |  |  |  | | F54C9.5 | | | | | | |
| F21C3.5 | | | WI5\_id:F21C3.5 |  | A | B | C | D | P | P' | N |
|  | Cluster:3-0 | |  |  | 1 | 232 | 0 | 4430 | 0.049967833 | 0.049967833 | 1 |
|  |  | RAFL05-07-D18 | At5g49510 / von Hippel-Lindau binding protein (VHL binding protein; VBP) like | |  |  |  |  |  | | --- | --- | --- | --- | --- | |  |  |  |  |  | | T06G6.9  Human VHL binding protein like | | | | | | |
| Y38A8.2 | | | WI5\_id:Y38A8.2 |  | A | B | C | D | P | P' | N |
|  | Cluster:4-1 | |  |  | 4 | 306 | 2 | 4351 | 2.5832583E-4 | 5.1665166E-4 | 2 |
|  |  | RAFL05-04-G23 | At1g77440 / 20S proteasome beta subunit C (PBC2) | |  |  |  |  |  | | --- | --- | --- | --- | --- | |  |  |  |  |  | | Y38A8.2 | | | | | | |
|  |  | RAFL05-04-L16 | At1g79210 / 20S proteasome alpha subunit B, putative | |  |  |  |  |  | | --- | --- | --- | --- | --- | |  |  |  |  |  | | D1054.2 | | | | | | |
|  |  | RAFL05-12-G18 | At4g14800 / 20S proteasome beta subunit D2 (PBD2) | |  |  |  |  |  | | --- | --- | --- | --- | --- | |  |  |  |  |  | | T20F5.2 | | | | | | |
|  |  | RAFL05-21-D23 | At1g21720 / 20S proteasome beta subunit C (PBC1) | |  |  |  |  |  | | --- | --- | --- | --- | --- | |  |  |  |  |  | | Y38A8.2 | | | | | | |
| F02E9.4 | | | WI5\_id:F02E9.4 |  | A | B | C | D | P | P' | N |
|  | Cluster:1-0 | |  |  | 1 | 148 | 0 | 4514 | 0.031953678 | 0.031953678 | 1 |
|  |  | RAFL02-06-N10 | At5g14920 / expressed protein | |  |  |  |  |  | | --- | --- | --- | --- | --- | |  |  |  |  |  | | F02E9.4 | | | | | | |
| C09D4.5 | | | WI5\_id:C09D4.5 |  | A | B | C | D | P | P' | N |
|  | Cluster:2-1 | |  |  | 6 | 238 | 12 | 4407 | 2.1043592E-4 | 0.0014730515 | 7 |
|  |  | RAFL02-10-A09 | At1g33140 / 60S ribosomal protein L9 (RPL90A/C) | |  |  |  |  |  | | --- | --- | --- | --- | --- | |  |  |  |  |  | | R13A5.8 | | | | | | |
|  |  | RAFL07-15-M07 | At1g04480 / 60S ribosomal protein L23 (RPL23A) | |  |  |  |  |  | | --- | --- | --- | --- | --- | |  |  |  |  |  | | B0336.10 | | | | | | |
|  |  | RAFL05-17-L17 | At3g55280 / 60S ribosomal protein L23A (RPL23aB) | |  |  |  |  |  | | --- | --- | --- | --- | --- | |  |  |  |  |  | | F52B5.6 | | | | | | |
|  |  | RAFL11-12-H04 | At3g25520 / 60S ribosomal protein L5 (RPL5A) | |  |  |  |  |  | | --- | --- | --- | --- | --- | |  |  |  |  |  | | F54C9.5 | | | | | | |
|  |  | RAFL05-18-P15 | At1g04480 / 60S ribosomal protein L23 (RPL23A) | |  |  |  |  |  | | --- | --- | --- | --- | --- | |  |  |  |  |  | | B0336.10 | | | | | | |
|  |  | RAFL11-07-B21 | At3g25520 / 60S ribosomal protein L5 (RPL5A) | |  |  |  |  |  | | --- | --- | --- | --- | --- | |  |  |  |  |  | | F54C9.5 | | | | | | |
|  | Cluster:2-0 | |  |  | 4 | 146 | 14 | 4499 | 0.002213303 | 0.015493121 | 7 |
|  |  | RAFL04-19-O24 | At5g02610 / 60S ribosomal protein L35 (RPL35D) | |  |  |  |  |  | | --- | --- | --- | --- | --- | |  |  |  |  |  | | ZK652.4 | | | | | | |
|  |  | RAFL06-10-L13 | At3g09630 / 60S ribosomal protein L4/L1 (RPL4A) | |  |  |  |  |  | | --- | --- | --- | --- | --- | |  |  |  |  |  | | B0041.4 | | | | | | |
|  |  | RAFL11-12-M17 | At3g25520 / 60S ribosomal protein L5 (RPL5A) | |  |  |  |  |  | | --- | --- | --- | --- | --- | |  |  |  |  |  | | F54C9.5 | | | | | | |
|  |  | RAFL03-06-H07 | At1g33140 / 60S ribosomal protein L9 (RPL90A/C) | |  |  |  |  |  | | --- | --- | --- | --- | --- | |  |  |  |  |  | | R13A5.8 | | | | | | |
| Y54E2A.11 | | | WI5\_id:Y54E2A.11 |  | A | B | C | D | P | P' | N |
|  | Cluster:4-1 | |  |  | 2 | 308 | 2 | 4351 | 0.024166461 | 0.07249938 | 3 |
|  |  | RAFL05-14-N10 | At3g11400 / eukaryotic translation initiation factor 3 subunit g (eIF3g) | |  |  |  |  |  | | --- | --- | --- | --- | --- | |  |  |  |  |  | | F22B5.2  RNA binding protein | | | | | | |
|  |  | RAFL07-07-F13 | At3g56150 / PROBABLE EUKARYOTIC TRANSLATION INITIATION FACTOR 3 SUBUNIT 8 | |  |  |  |  |  | | --- | --- | --- | --- | --- | |  |  |  |  |  | | T23D8.4 | | | | | | |
| Y43E12A.1 | | | WI5\_id:Y43E12A.1 |  | A | B | C | D | P | P' | N |
|  | Cluster:4-2 | |  |  | 1 | 134 | 0 | 4528 | 0.028951319 | 0.028951319 | 1 |
|  |  | RAFL05-18-G17 | At3g48750 / cell division control protein 2 homolog A (CDC2A) | |  |  |  |  |  | | --- | --- | --- | --- | --- | |  |  |  |  |  | | T05G5.3 | | | | | | |
| C06A8.1 | | | WI5\_id:C06A8.1 |  | A | B | C | D | P | P' | N |
|  | Cluster:5-0 | |  |  | 1 | 76 | 2 | 4584 | 0.04873567 | 0.146207 | 3 |
|  |  | RAFL06-07-P18 | At3g60820 / 20S proteasome beta subunit F1 (PBF1) | |  |  |  |  |  | | --- | --- | --- | --- | --- | |  |  |  |  |  | | C02F5.9 | | | | | | |
| K01G5.4 | | | WI5\_id:K01G5.4 |  | A | B | C | D | P | P' | N |
|  | Cluster:4-0 | |  |  | 3 | 237 | 9 | 4414 | 0.020962995 | 0.16770396 | 8 |
|  |  | RAFL06-07-B07 | At5g55190 / GTP-binding protein atran3, putative | |  |  |  |  |  | | --- | --- | --- | --- | --- | |  |  |  |  |  | | K01G5.4 | | | | | | |
|  |  | RAFL05-17-C16 | At5g55190 / GTP-binding protein atran3, putative | |  |  |  |  |  | | --- | --- | --- | --- | --- | |  |  |  |  |  | | K01G5.4 | | | | | | |
|  |  | RAFL07-07-O08 | At4g30800 / 40S ribosomal protein S11 (RPS11B) | |  |  |  |  |  | | --- | --- | --- | --- | --- | |  |  |  |  |  | | F40F11.1 | | | | | | |
|  | Cluster:2-1 | |  |  | 3 | 241 | 9 | 4410 | 0.021903817 | 0.17523053 | 8 |
|  |  | RAFL09-15-M15 | At5g23740 / 40S ribosomal protein S11 (RPS11C) | |  |  |  |  |  | | --- | --- | --- | --- | --- | |  |  |  |  |  | | F40F11.1 | | | | | | |
|  |  | RAFL06-09-H09 | At3g48930 / 40S ribosomal protein S11 (RPS11A) | |  |  |  |  |  | | --- | --- | --- | --- | --- | |  |  |  |  |  | | F40F11.1 | | | | | | |
|  |  | RAFL04-19-M20 | At1g58380 / 40S ribosomal protein S2 (RPS2A) | |  |  |  |  |  | | --- | --- | --- | --- | --- | |  |  |  |  |  | | C49H3.11 | | | | | | |
| T06D8.8 | | | WI5\_id:T06D8.8 |  | A | B | C | D | P | P' | N |
|  | Cluster:7-0 | |  |  | 2 | 245 | 5 | 4411 | 0.049187053 | 0.29512233 | 6 |
|  |  | RAFL05-04-D08 | At1g64520 / 26S proteasome regulatory subunit (RPN12), putative | |  |  |  |  |  | | --- | --- | --- | --- | --- | |  |  |  |  |  | | ZK20.5 | | | | | | |
|  |  | RAFL06-13-G03 | At1g64520 / 26S proteasome regulatory subunit (RPN12), putative | |  |  |  |  |  | | --- | --- | --- | --- | --- | |  |  |  |  |  | | ZK20.5 | | | | | | |
| C53D5.6 | | | WI5\_id:C53D5.6 |  | A | B | C | D | P | P' | N |
|  | Cluster:4-0 | |  |  | 2 | 238 | 1 | 4422 | 0.0076462985 | 0.015292597 | 2 |
|  |  | RAFL06-07-B07 | At5g55190 / GTP-binding protein atran3, putative | |  |  |  |  |  | | --- | --- | --- | --- | --- | |  |  |  |  |  | | K01G5.4 | | | | | | |
|  |  | RAFL05-17-C16 | At5g55190 / GTP-binding protein atran3, putative | |  |  |  |  |  | | --- | --- | --- | --- | --- | |  |  |  |  |  | | K01G5.4 | | | | | | |
| C44H4.5 | | | WI5\_id:C44H4.5 |  | A | B | C | D | P | P' | N |
|  | Cluster:3-0 | |  |  | 2 | 231 | 0 | 4430 | 0.0024866017 | 0.0024866017 | 1 |
|  |  | RAFL09-10-P09 | At4g36130 / 60S ribosomal protein L8 (RPL8C) | |  |  |  |  |  | | --- | --- | --- | --- | --- | |  |  |  |  |  | | B0250.1 | | | | | | |
|  |  | RAFL09-12-B12 | At4g36130 / 60S ribosomal protein L8 (RPL8C) | |  |  |  |  |  | | --- | --- | --- | --- | --- | |  |  |  |  |  | | B0250.1 | | | | | | |
| R151.9 | | | WI5\_id:R151.9 |  | A | B | C | D | P | P' | N |
|  | Cluster:3-0 | |  |  | 1 | 232 | 0 | 4430 | 0.049967833 | 0.049967833 | 1 |
|  |  | RAFL05-07-D18 | At5g49510 / von Hippel-Lindau binding protein (VHL binding protein; VBP) like | |  |  |  |  |  | | --- | --- | --- | --- | --- | |  |  |  |  |  | | T06G6.9 | | | | | | |
| C34E10.6 | | | WI5\_id:C34E10.6 |  | A | B | C | D | P | P' | N |
|  | Cluster:4-1 | |  |  | 2 | 308 | 2 | 4351 | 0.024166461 | 0.07249938 | 3 |
|  |  | RAFL04-15-D14 | At5g08680 / H+-transporting ATP synthase beta chain -related | |  |  |  |  |  | | --- | --- | --- | --- | --- | |  |  |  |  |  | | C34E10.6 | | | | | | |
|  |  | RAFL09-17-J21 | At5g08690 / H+-transporting ATP synthase beta chain (mitochondrial) -related | |  |  |  |  |  | | --- | --- | --- | --- | --- | |  |  |  |  |  | | C34E10.6 | | | | | | |
| ZK930.3 | | | WI5\_id:ZK930.3 |  | A | B | C | D | P | P' | N |
|  | Cluster:6-1 | |  |  | 2 | 313 | 1 | 4347 | 0.013038642 | 0.026077284 | 2 |
|  |  | RAFL06-12-E24 | At5g42790 / 20S proteasome alpha subunit F1 (PAF1) | |  |  |  |  |  | | --- | --- | --- | --- | --- | |  |  |  |  |  | | CD4.6 | | | | | | |
|  |  | RAFL11-07-B22 | At1g14650 / splicing factor -related | |  |  |  |  |  | | --- | --- | --- | --- | --- | |  |  |  |  |  | | W07E6.4  splicing factor | | | | | | |
| F39H11.5 | | | WI5\_id:F39H11.5 |  | A | B | C | D | P | P' | N |
|  | Cluster:6-1 | |  |  | 2 | 313 | 2 | 4346 | 0.024916494 | 0.074749485 | 3 |
|  |  | RAFL06-09-A21 | At5g40580 / 20S proteasome beta subunit B (PBB2) | |  |  |  |  |  | | --- | --- | --- | --- | --- | |  |  |  |  |  | | C47B2.4 | | | | | | |
|  |  | RAFL06-16-H22 | At1g16470 / 20S proteasome alpha subunit B (PAB1) | |  |  |  |  |  | | --- | --- | --- | --- | --- | |  |  |  |  |  | | D1054.2 | | | | | | |
| C34C6.6 | | | WI5\_id:C34C6.6 |  | A | B | C | D | P | P' | N |
|  | Cluster:4-2 | |  |  | 1 | 134 | 0 | 4528 | 0.028951319 | 0.028951319 | 1 |
|  |  | RAFL05-05-K13 | At3g52590 / ubiquitin extension protein 1 (UBQ1)/60S ribosomal protein L40 (RPL40B) | |  |  |  |  |  | | --- | --- | --- | --- | --- | |  |  |  |  |  | | ZK1010.1 | | | | | | |
| T01G9.5 | | | WI5\_id:T01G9.5 |  | A | B | C | D | P | P' | N |
|  | Cluster:2-0 | |  |  | 2 | 148 | 3 | 4510 | 0.009643516 | 0.038574062 | 4 |
|  |  | RAFL06-10-L13 | At3g09630 / 60S ribosomal protein L4/L1 (RPL4A) | |  |  |  |  |  | | --- | --- | --- | --- | --- | |  |  |  |  |  | | B0041.4 | | | | | | |
|  |  | RAFL05-01-F21 | At3g05590 / 60S ribosomal protein L18 (RPL18B) | |  |  |  |  |  | | --- | --- | --- | --- | --- | |  |  |  |  |  | | Y45F10D.12 | | | | | | |
| R09B5.5 | | | WI5\_id:R09B5.5 |  | A | B | C | D | P | P' | N |
|  | Cluster:2-1 | |  |  | 4 | 240 | 4 | 4415 | 4.3340996E-4 | 0.0017336399 | 4 |
|  |  | RAFL07-13-J18 | At2g37270 / 40S ribosomal protein S5 (RPS5A) | |  |  |  |  |  | | --- | --- | --- | --- | --- | |  |  |  |  |  | | T05E11.1 | | | | | | |
|  |  | RAFL11-12-H04 | At3g25520 / 60S ribosomal protein L5 (RPL5A) | |  |  |  |  |  | | --- | --- | --- | --- | --- | |  |  |  |  |  | | F54C9.5 | | | | | | |
|  |  | RAFL04-18-N22 | At2g44120 / 60S ribosomal protein L7 (RPL7C) | |  |  |  |  |  | | --- | --- | --- | --- | --- | |  |  |  |  |  | | F53G12.10 | | | | | | |
|  |  | RAFL11-07-B21 | At3g25520 / 60S ribosomal protein L5 (RPL5A) | |  |  |  |  |  | | --- | --- | --- | --- | --- | |  |  |  |  |  | | F54C9.5 | | | | | | |
|  | Cluster:2-0 | |  |  | 2 | 148 | 6 | 4507 | 0.025339978 | 0.10135991 | 4 |
|  |  | RAFL11-12-M17 | At3g25520 / 60S ribosomal protein L5 (RPL5A) | |  |  |  |  |  | | --- | --- | --- | --- | --- | |  |  |  |  |  | | F54C9.5 | | | | | | |
|  |  | RAFL03-08-O03 | At1g07940 / elongation factor 1-alpha (EF-1-alpha) | |  |  |  |  |  | | --- | --- | --- | --- | --- | |  |  |  |  |  | | F31E3.5 | | | | | | |
| F25H2.9 | | | WI5\_id:F25H2.9 |  | A | B | C | D | P | P' | N |
|  | Cluster:4-1 | |  |  | 2 | 308 | 1 | 4352 | 0.012636807 | 0.025273614 | 2 |
|  |  | RAFL04-12-B09 | At3g14290 / 20S proteasome alpha subunit E2 (PAE2) | |  |  |  |  |  | | --- | --- | --- | --- | --- | |  |  |  |  |  | | F25H2.9 | | | | | | |
|  |  | RAFL05-12-G18 | At4g14800 / 20S proteasome beta subunit D2 (PBD2) | |  |  |  |  |  | | --- | --- | --- | --- | --- | |  |  |  |  |  | | T20F5.2 | | | | | | |
| F56G4.5 | | | WI5\_id:F56G4.5 |  | A | B | C | D | P | P' | N |
|  | Cluster:4-0 | |  |  | 2 | 238 | 1 | 4422 | 0.0076462985 | 0.015292597 | 2 |
|  |  | RAFL06-07-B07 | At5g55190 / GTP-binding protein atran3, putative | |  |  |  |  |  | | --- | --- | --- | --- | --- | |  |  |  |  |  | | K01G5.4 | | | | | | |
|  |  | RAFL05-17-C16 | At5g55190 / GTP-binding protein atran3, putative | |  |  |  |  |  | | --- | --- | --- | --- | --- | |  |  |  |  |  | | K01G5.4 | | | | | | |
| C06E4.2 | | | WI5\_id:C06E4.2 |  | A | B | C | D | P | P' | N |
|  | Cluster:0-2 | |  |  | 1 | 78 | 1 | 4583 | 0.03360031 | 0.06720062 | 2 |
|  |  | RAFL04-17-G02 | At1g56340 / calreticulin 1 (CRT1) | |  |  |  |  |  | | --- | --- | --- | --- | --- | |  |  |  |  |  | | Y38A10A.5 | | | | | | |
| F44G3.9 | | | WI5\_id:F44G3.9 |  | A | B | C | D | P | P' | N |
|  | Cluster:5-0 | |  |  | 1 | 76 | 2 | 4584 | 0.04873567 | 0.146207 | 3 |
|  |  | RAFL06-07-P18 | At3g60820 / 20S proteasome beta subunit F1 (PBF1) | |  |  |  |  |  | | --- | --- | --- | --- | --- | |  |  |  |  |  | | C02F5.9 | | | | | | |
| B0250.1 | | | WI5\_id:B0250.1 |  | A | B | C | D | P | P' | N |
|  | Cluster:2-1 | |  |  | 6 | 238 | 13 | 4406 | 2.9421985E-4 | 0.001765319 | 6 |
|  |  | RAFL02-10-A09 | At1g33140 / 60S ribosomal protein L9 (RPL90A/C) | |  |  |  |  |  | | --- | --- | --- | --- | --- | |  |  |  |  |  | | R13A5.8  Ribosomal protein L9 | | | | | | |
|  |  | RAFL07-15-M07 | At1g04480 / 60S ribosomal protein L23 (RPL23A) | |  |  |  |  |  | | --- | --- | --- | --- | --- | |  |  |  |  |  | | B0336.10 | | | | | | |
|  |  | RAFL05-17-L17 | At3g55280 / 60S ribosomal protein L23A (RPL23aB) | |  |  |  |  |  | | --- | --- | --- | --- | --- | |  |  |  |  |  | | F52B5.6 | | | | | | |
|  |  | RAFL11-12-H04 | At3g25520 / 60S ribosomal protein L5 (RPL5A) | |  |  |  |  |  | | --- | --- | --- | --- | --- | |  |  |  |  |  | | F54C9.5 | | | | | | |
|  |  | RAFL05-18-P15 | At1g04480 / 60S ribosomal protein L23 (RPL23A) | |  |  |  |  |  | | --- | --- | --- | --- | --- | |  |  |  |  |  | | B0336.10 | | | | | | |
|  |  | RAFL11-07-B21 | At3g25520 / 60S ribosomal protein L5 (RPL5A) | |  |  |  |  |  | | --- | --- | --- | --- | --- | |  |  |  |  |  | | F54C9.5 | | | | | | |
|  | Cluster:3-0 | |  |  | 5 | 228 | 14 | 4416 | 0.0019486465 | 0.011691879 | 6 |
|  |  | RAFL07-10-D02 | At1g14320 / 60S ribosomal protein L10 (RPL10A)/Wilm's tumor suppressor protein-related | |  |  |  |  |  | | --- | --- | --- | --- | --- | |  |  |  |  |  | | F10B5.1 | | | | | | |
|  |  | RAFL09-10-P09 | At4g36130 / 60S ribosomal protein L8 (RPL8C) | |  |  |  |  |  | | --- | --- | --- | --- | --- | |  |  |  |  |  | | B0250.1 | | | | | | |
|  |  | RAFL08-10-G08 | At3g09630 / 60S ribosomal protein L4/L1 (RPL4A) | |  |  |  |  |  | | --- | --- | --- | --- | --- | |  |  |  |  |  | | B0041.4 | | | | | | |
|  |  | RAFL08-13-M06 | At3g11250 / 60S acidic ribosomal protein P0 (RPP0C) | |  |  |  |  |  | | --- | --- | --- | --- | --- | |  |  |  |  |  | | F25H2.10 | | | | | | |
|  |  | RAFL09-12-B12 | At4g36130 / 60S ribosomal protein L8 (RPL8C) | |  |  |  |  |  | | --- | --- | --- | --- | --- | |  |  |  |  |  | | B0250.1 | | | | | | |
|  | Cluster:2-0 | |  |  | 4 | 146 | 15 | 4498 | 0.002734157 | 0.016404942 | 6 |
|  |  | RAFL04-19-O24 | At5g02610 / 60S ribosomal protein L35 (RPL35D) | |  |  |  |  |  | | --- | --- | --- | --- | --- | |  |  |  |  |  | | ZK652.4 | | | | | | |
|  |  | RAFL06-10-L13 | At3g09630 / 60S ribosomal protein L4/L1 (RPL4A) | |  |  |  |  |  | | --- | --- | --- | --- | --- | |  |  |  |  |  | | B0041.4 | | | | | | |
|  |  | RAFL11-12-M17 | At3g25520 / 60S ribosomal protein L5 (RPL5A) | |  |  |  |  |  | | --- | --- | --- | --- | --- | |  |  |  |  |  | | F54C9.5 | | | | | | |
|  |  | RAFL03-06-H07 | At1g33140 / 60S ribosomal protein L9 (RPL90A/C) | |  |  |  |  |  | | --- | --- | --- | --- | --- | |  |  |  |  |  | | R13A5.8  Ribosomal protein L9 | | | | | | |
| C23G10.3 | | | WI5\_id:C23G10.3 |  | A | B | C | D | P | P' | N |
|  | Cluster:2-1 | |  |  | 8 | 236 | 10 | 4409 | 1.3906676E-6 | 6.953338E-6 | 5 |
|  |  | RAFL04-18-N10 | At1g07770 / 40S ribosomal protein S15A (RPS15aA) | |  |  |  |  |  | | --- | --- | --- | --- | --- | |  |  |  |  |  | | F53A3.3  40S ribosomal protein | | | | | | |
|  |  | RAFL09-15-M15 | At5g23740 / 40S ribosomal protein S11 (RPS11C) | |  |  |  |  |  | | --- | --- | --- | --- | --- | |  |  |  |  |  | | F40F11.1  ribosomal protein S11 | | | | | | |
|  |  | RAFL05-05-M24 | At5g02960 / 40S ribosomal protein S23 (RPS23B) | |  |  |  |  |  | | --- | --- | --- | --- | --- | |  |  |  |  |  | | F28D1.7  ribosomal protein S23 | | | | | | |
|  |  | RAFL07-13-J18 | At2g37270 / 40S ribosomal protein S5 (RPS5A) | |  |  |  |  |  | | --- | --- | --- | --- | --- | |  |  |  |  |  | | T05E11.1 | | | | | | |
|  |  | RAFL06-09-H09 | At3g48930 / 40S ribosomal protein S11 (RPS11A) | |  |  |  |  |  | | --- | --- | --- | --- | --- | |  |  |  |  |  | | F40F11.1  ribosomal protein S11 | | | | | | |
|  |  | RAFL06-08-B09 | At3g11510 / 40S ribosomal protein S14 (RPS14B) | |  |  |  |  |  | | --- | --- | --- | --- | --- | |  |  |  |  |  | | F37C12.9  Ribosomal protein S14 | | | | | | |
|  |  | RAFL05-16-H14 | At1g04270 / 40S ribosomal protein S15 (RPS15A) | |  |  |  |  |  | | --- | --- | --- | --- | --- | |  |  |  |  |  | | F36A2.6 | | | | | | |
|  |  | RAFL04-19-M20 | At1g58380 / 40S ribosomal protein S2 (RPS2A) | |  |  |  |  |  | | --- | --- | --- | --- | --- | |  |  |  |  |  | | C49H3.11 | | | | | | |
|  | Cluster:4-2 | |  |  | 4 | 131 | 14 | 4514 | 0.0014992869 | 0.0074964343 | 5 |
|  |  | RAFL05-07-M02 | At1g34030 / 40S ribosomal protein S18 (RPS18B) | |  |  |  |  |  | | --- | --- | --- | --- | --- | |  |  |  |  |  | | Y57G11C.16  ribosomal protein S13 | | | | | | |
|  |  | RAFL09-17-F16 | At1g07770 / 40S ribosomal protein S15A (RPS15aA) | |  |  |  |  |  | | --- | --- | --- | --- | --- | |  |  |  |  |  | | F53A3.3  40S ribosomal protein | | | | | | |
|  |  | RAFL11-02-D23 | At1g07770 / 40S ribosomal protein S15A (RPS15aA) | |  |  |  |  |  | | --- | --- | --- | --- | --- | |  |  |  |  |  | | F53A3.3  40S ribosomal protein | | | | | | |
|  |  | RAFL05-08-B08 | At5g18380 / 40S ribosomal protein S16 (RPS16C) | |  |  |  |  |  | | --- | --- | --- | --- | --- | |  |  |  |  |  | | T01C3.6  40S ribosomal protein S16 | | | | | | |
|  | Cluster:3-0 | |  |  | 4 | 229 | 14 | 4416 | 0.01067757 | 0.053387847 | 5 |
|  |  | RAFL02-10-H10 | At3g43980 / 40S ribosomal protein S29 (RPS29A) | |  |  |  |  |  | | --- | --- | --- | --- | --- | |  |  |  |  |  | | B0412.4 | | | | | | |
|  |  | RAFL08-09-E20 | At2g41840 / 40S ribosomal protein S2 (RPS2C) | |  |  |  |  |  | | --- | --- | --- | --- | --- | |  |  |  |  |  | | C49H3.11 | | | | | | |
|  |  | RAFL11-04-A02 | At1g07770 / 40S ribosomal protein S15A (RPS15aA) | |  |  |  |  |  | | --- | --- | --- | --- | --- | |  |  |  |  |  | | F53A3.3  40S ribosomal protein | | | | | | |
|  |  | RAFL06-07-B02 | At3g11940 / 40S ribosomal protein S5 (RPS5B) | |  |  |  |  |  | | --- | --- | --- | --- | --- | |  |  |  |  |  | | T05E11.1 | | | | | | |
| T05E11.1 | | | WI5\_id:T05E11.1 |  | A | B | C | D | P | P' | N |
|  | Cluster:2-1 | |  |  | 8 | 236 | 9 | 4410 | 8.089096E-7 | 4.044548E-6 | 5 |
|  |  | RAFL04-18-N10 | At1g07770 / 40S ribosomal protein S15A (RPS15aA) | |  |  |  |  |  | | --- | --- | --- | --- | --- | |  |  |  |  |  | | F53A3.3 | | | | | | |
|  |  | RAFL09-15-M15 | At5g23740 / 40S ribosomal protein S11 (RPS11C) | |  |  |  |  |  | | --- | --- | --- | --- | --- | |  |  |  |  |  | | F40F11.1 | | | | | | |
|  |  | RAFL05-05-M24 | At5g02960 / 40S ribosomal protein S23 (RPS23B) | |  |  |  |  |  | | --- | --- | --- | --- | --- | |  |  |  |  |  | | F28D1.7 | | | | | | |
|  |  | RAFL07-13-J18 | At2g37270 / 40S ribosomal protein S5 (RPS5A) | |  |  |  |  |  | | --- | --- | --- | --- | --- | |  |  |  |  |  | | T05E11.1 | | | | | | |
|  |  | RAFL06-09-H09 | At3g48930 / 40S ribosomal protein S11 (RPS11A) | |  |  |  |  |  | | --- | --- | --- | --- | --- | |  |  |  |  |  | | F40F11.1 | | | | | | |
|  |  | RAFL06-08-B09 | At3g11510 / 40S ribosomal protein S14 (RPS14B) | |  |  |  |  |  | | --- | --- | --- | --- | --- | |  |  |  |  |  | | F37C12.9 | | | | | | |
|  |  | RAFL05-16-H14 | At1g04270 / 40S ribosomal protein S15 (RPS15A) | |  |  |  |  |  | | --- | --- | --- | --- | --- | |  |  |  |  |  | | F36A2.6 | | | | | | |
|  |  | RAFL04-19-M20 | At1g58380 / 40S ribosomal protein S2 (RPS2A) | |  |  |  |  |  | | --- | --- | --- | --- | --- | |  |  |  |  |  | | C49H3.11 | | | | | | |
|  | Cluster:3-0 | |  |  | 4 | 229 | 13 | 4417 | 0.008637219 | 0.043186095 | 5 |
|  |  | RAFL02-10-H10 | At3g43980 / 40S ribosomal protein S29 (RPS29A) | |  |  |  |  |  | | --- | --- | --- | --- | --- | |  |  |  |  |  | | B0412.4 | | | | | | |
|  |  | RAFL08-09-E20 | At2g41840 / 40S ribosomal protein S2 (RPS2C) | |  |  |  |  |  | | --- | --- | --- | --- | --- | |  |  |  |  |  | | C49H3.11 | | | | | | |
|  |  | RAFL11-04-A02 | At1g07770 / 40S ribosomal protein S15A (RPS15aA) | |  |  |  |  |  | | --- | --- | --- | --- | --- | |  |  |  |  |  | | F53A3.3 | | | | | | |
|  |  | RAFL06-07-B02 | At3g11940 / 40S ribosomal protein S5 (RPS5B) | |  |  |  |  |  | | --- | --- | --- | --- | --- | |  |  |  |  |  | | T05E11.1 | | | | | | |
|  | Cluster:4-2 | |  |  | 3 | 132 | 14 | 4514 | 0.0119851455 | 0.059925728 | 5 |
|  |  | RAFL05-07-M02 | At1g34030 / 40S ribosomal protein S18 (RPS18B) | |  |  |  |  |  | | --- | --- | --- | --- | --- | |  |  |  |  |  | | Y57G11C.16 | | | | | | |
|  |  | RAFL09-17-F16 | At1g07770 / 40S ribosomal protein S15A (RPS15aA) | |  |  |  |  |  | | --- | --- | --- | --- | --- | |  |  |  |  |  | | F53A3.3 | | | | | | |
|  |  | RAFL11-02-D23 | At1g07770 / 40S ribosomal protein S15A (RPS15aA) | |  |  |  |  |  | | --- | --- | --- | --- | --- | |  |  |  |  |  | | F53A3.3 | | | | | | |
| C15H11.7 | | | WI5\_id:C15H11.7 |  | A | B | C | D | P | P' | N |
|  | Cluster:4-1 | |  |  | 7 | 303 | 5 | 4348 | 3.1829013E-6 | 1.2731605E-5 | 4 |
|  |  | RAFL04-15-N11 | At3g22110 / 20S proteasome alpha subunit C (PAC1) | |  |  |  |  |  | | --- | --- | --- | --- | --- | |  |  |  |  |  | | Y110A7A.14  endopeptidase | | | | | | |
|  |  | RAFL05-01-C14 | At5g42790 / 20S proteasome alpha subunit F1 (PAF1) | |  |  |  |  |  | | --- | --- | --- | --- | --- | |  |  |  |  |  | | CD4.6 | | | | | | |
|  |  | RAFL07-08-J17 | At1g13060 / 20S proteasome beta subunit E1 (PBE1) | |  |  |  |  |  | | --- | --- | --- | --- | --- | |  |  |  |  |  | | K05C4.1 | | | | | | |
|  |  | RAFL05-04-G23 | At1g77440 / 20S proteasome beta subunit C (PBC2) | |  |  |  |  |  | | --- | --- | --- | --- | --- | |  |  |  |  |  | | Y38A8.2 | | | | | | |
|  |  | RAFL05-04-L16 | At1g79210 / 20S proteasome alpha subunit B, putative | |  |  |  |  |  | | --- | --- | --- | --- | --- | |  |  |  |  |  | | D1054.2 | | | | | | |
|  |  | RAFL05-12-G18 | At4g14800 / 20S proteasome beta subunit D2 (PBD2) | |  |  |  |  |  | | --- | --- | --- | --- | --- | |  |  |  |  |  | | T20F5.2 | | | | | | |
|  |  | RAFL05-21-D23 | At1g21720 / 20S proteasome beta subunit C (PBC1) | |  |  |  |  |  | | --- | --- | --- | --- | --- | |  |  |  |  |  | | Y38A8.2 | | | | | | |
|  | Cluster:6-1 | |  |  | 3 | 312 | 9 | 4339 | 0.042557452 | 0.17022981 | 4 |
|  |  | RAFL06-16-H22 | At1g16470 / 20S proteasome alpha subunit B (PAB1) | |  |  |  |  |  | | --- | --- | --- | --- | --- | |  |  |  |  |  | | D1054.2 | | | | | | |
|  |  | RAFL08-09-N05 | At4g31300 / 20S proteasome beta subunit A (PBA1) | |  |  |  |  |  | | --- | --- | --- | --- | --- | |  |  |  |  |  | | K08D12.1 | | | | | | |
|  |  | RAFL06-12-E24 | At5g42790 / 20S proteasome alpha subunit F1 (PAF1) | |  |  |  |  |  | | --- | --- | --- | --- | --- | |  |  |  |  |  | | CD4.6 | | | | | | |
| ZK792.8 | | | WI5\_id:ZK792.8 |  | A | B | C | D | P | P' | N |
|  | Cluster:1-0 | |  |  | 2 | 147 | 4 | 4510 | 0.013980698 | 0.05592279 | 4 |
|  |  | RAFL09-11-P11 | At1g20010 / tubulin beta-5 chain (TUB5) | |  |  |  |  |  | | --- | --- | --- | --- | --- | |  |  |  |  |  | | B0272.1 | | | | | | |
|  |  | RAFL04-16-G05 | At5g44340 / tubulin beta-4 chain (TUB4) | |  |  |  |  |  | | --- | --- | --- | --- | --- | |  |  |  |  |  | | B0272.1 | | | | | | |
|  | Cluster:2-1 | |  |  | 2 | 242 | 4 | 4415 | 0.0355681 | 0.1422724 | 4 |
|  |  | RAFL05-09-F01 | At5g44340 / tubulin beta-4 chain (TUB4) | |  |  |  |  |  | | --- | --- | --- | --- | --- | |  |  |  |  |  | | B0272.1 | | | | | | |
|  |  | RAFL06-12-I16 | At2g29550 / tubulin beta-7 chain (TUB7) | |  |  |  |  |  | | --- | --- | --- | --- | --- | |  |  |  |  |  | | B0272.1 | | | | | | |
| F20B6.2 | | | WI5\_id:F20B6.2 |  | A | B | C | D | P | P' | N |
|  | Cluster:4-1 | |  |  | 2 | 308 | 3 | 4350 | 0.038523052 | 0.15409221 | 4 |
|  |  | RAFL04-09-A19 | At4g38510 / probable H+-transporting ATPase | |  |  |  |  |  | | --- | --- | --- | --- | --- | |  |  |  |  |  | | F20B6.2 | | | | | | |
|  |  | RAFL07-17-H08 | At4g38510 / probable H+-transporting ATPase | |  |  |  |  |  | | --- | --- | --- | --- | --- | |  |  |  |  |  | | F20B6.2 | | | | | | |
| T04H1.2 | | | WI5\_id:T04H1.2 |  | A | B | C | D | P | P' | N |
|  | Cluster:2-1 | |  |  | 2 | 242 | 3 | 4416 | 0.024550742 | 0.098202966 | 4 |
|  |  | RAFL07-15-M07 | At1g04480 / 60S ribosomal protein L23 (RPL23A) | |  |  |  |  |  | | --- | --- | --- | --- | --- | |  |  |  |  |  | | B0336.10 | | | | | | |
|  |  | RAFL05-18-P15 | At1g04480 / 60S ribosomal protein L23 (RPL23A) | |  |  |  |  |  | | --- | --- | --- | --- | --- | |  |  |  |  |  | | B0336.10 | | | | | | |
| C15H9.6 | | | WI5\_id:C15H9.6 |  | A | B | C | D | P | P' | N |
|  | Cluster:2-1 | |  |  | 2 | 242 | 1 | 4418 | 0.00789916 | 0.01579832 | 2 |
|  |  | RAFL06-08-N05 | At5g28540 / luminal binding protein 1 precursor (BiP-1) (AtBP1) | |  |  |  |  |  | | --- | --- | --- | --- | --- | |  |  |  |  |  | | C15H9.6 | | | | | | |
|  |  | RAFL07-11-O19 | At5g28540 / luminal binding protein 1 precursor (BiP-1) (AtBP1) | |  |  |  |  |  | | --- | --- | --- | --- | --- | |  |  |  |  |  | | C15H9.6 | | | | | | |
| F42C5.8 | | | WI5\_id:F42C5.8 |  | A | B | C | D | P | P' | N |
|  | Cluster:2-0 | |  |  | 1 | 149 | 0 | 4513 | 0.03216813 | 0.03216813 | 1 |
|  |  | RAFL08-12-D04 | At5g20290 / 40S ribosomal protein S8 (RPS8A) | |  |  |  |  |  | | --- | --- | --- | --- | --- | |  |  |  |  |  | | F42C5.8 | | | | | | |
| K02F2.2 | | | WI5\_id:K02F2.2 |  | A | B | C | D | P | P' | N |
|  | Cluster:4-2 | |  |  | 1 | 134 | 0 | 4528 | 0.028951319 | 0.028951319 | 1 |
|  |  | RAFL05-05-K13 | At3g52590 / ubiquitin extension protein 1 (UBQ1)/60S ribosomal protein L40 (RPL40B) | |  |  |  |  |  | | --- | --- | --- | --- | --- | |  |  |  |  |  | | ZK1010.1 | | | | | | |
| Y92C3B.2 | | | WI5\_id:Y92C3B.2 |  | A | B | C | D | P | P' | N |
|  | Cluster:5-0 | |  |  | 4 | 73 | 1 | 4585 | 3.3961462E-7 | 6.7922923E-7 | 2 |
|  |  | RAFL04-15-M13 | At5g56030 / heat shock protein 81-2 (HSP81-2) | |  |  |  |  |  | | --- | --- | --- | --- | --- | |  |  |  |  |  | | C47E8.5 | | | | | | |
|  |  | RAFL07-13-H08 | At5g56010 / heat shock protein, putative | |  |  |  |  |  | | --- | --- | --- | --- | --- | |  |  |  |  |  | | C47E8.5 | | | | | | |
|  |  | RAFL09-06-O18 | At5g56030 / heat shock protein 81-2 (HSP81-2) | |  |  |  |  |  | | --- | --- | --- | --- | --- | |  |  |  |  |  | | C47E8.5 | | | | | | |
|  |  | RAFL05-16-L15 | At5g56010 / heat shock protein, putative | |  |  |  |  |  | | --- | --- | --- | --- | --- | |  |  |  |  |  | | C47E8.5 | | | | | | |
| Y105E8A.16 | | | WI5\_id:Y105E8A.16 |  | A | B | C | D | P | P' | N |
|  | Cluster:2-1 | |  |  | 8 | 236 | 9 | 4410 | 8.089096E-7 | 4.044548E-6 | 5 |
|  |  | RAFL04-18-N10 | At1g07770 / 40S ribosomal protein S15A (RPS15aA) | |  |  |  |  |  | | --- | --- | --- | --- | --- | |  |  |  |  |  | | F53A3.3 | | | | | | |
|  |  | RAFL09-15-M15 | At5g23740 / 40S ribosomal protein S11 (RPS11C) | |  |  |  |  |  | | --- | --- | --- | --- | --- | |  |  |  |  |  | | F40F11.1 | | | | | | |
|  |  | RAFL05-05-M24 | At5g02960 / 40S ribosomal protein S23 (RPS23B) | |  |  |  |  |  | | --- | --- | --- | --- | --- | |  |  |  |  |  | | F28D1.7 | | | | | | |
|  |  | RAFL07-13-J18 | At2g37270 / 40S ribosomal protein S5 (RPS5A) | |  |  |  |  |  | | --- | --- | --- | --- | --- | |  |  |  |  |  | | T05E11.1 | | | | | | |
|  |  | RAFL06-09-H09 | At3g48930 / 40S ribosomal protein S11 (RPS11A) | |  |  |  |  |  | | --- | --- | --- | --- | --- | |  |  |  |  |  | | F40F11.1 | | | | | | |
|  |  | RAFL06-08-B09 | At3g11510 / 40S ribosomal protein S14 (RPS14B) | |  |  |  |  |  | | --- | --- | --- | --- | --- | |  |  |  |  |  | | F37C12.9 | | | | | | |
|  |  | RAFL05-16-H14 | At1g04270 / 40S ribosomal protein S15 (RPS15A) | |  |  |  |  |  | | --- | --- | --- | --- | --- | |  |  |  |  |  | | F36A2.6 | | | | | | |
|  |  | RAFL04-19-M20 | At1g58380 / 40S ribosomal protein S2 (RPS2A) | |  |  |  |  |  | | --- | --- | --- | --- | --- | |  |  |  |  |  | | C49H3.11 | | | | | | |
|  | Cluster:3-0 | |  |  | 4 | 229 | 13 | 4417 | 0.008637219 | 0.043186095 | 5 |
|  |  | RAFL02-10-H10 | At3g43980 / 40S ribosomal protein S29 (RPS29A) | |  |  |  |  |  | | --- | --- | --- | --- | --- | |  |  |  |  |  | | B0412.4 | | | | | | |
|  |  | RAFL08-09-E20 | At2g41840 / 40S ribosomal protein S2 (RPS2C) | |  |  |  |  |  | | --- | --- | --- | --- | --- | |  |  |  |  |  | | C49H3.11 | | | | | | |
|  |  | RAFL11-04-A02 | At1g07770 / 40S ribosomal protein S15A (RPS15aA) | |  |  |  |  |  | | --- | --- | --- | --- | --- | |  |  |  |  |  | | F53A3.3 | | | | | | |
|  |  | RAFL06-07-B02 | At3g11940 / 40S ribosomal protein S5 (RPS5B) | |  |  |  |  |  | | --- | --- | --- | --- | --- | |  |  |  |  |  | | T05E11.1 | | | | | | |
|  | Cluster:4-2 | |  |  | 3 | 132 | 14 | 4514 | 0.0119851455 | 0.059925728 | 5 |
|  |  | RAFL05-07-M02 | At1g34030 / 40S ribosomal protein S18 (RPS18B) | |  |  |  |  |  | | --- | --- | --- | --- | --- | |  |  |  |  |  | | Y57G11C.16 | | | | | | |
|  |  | RAFL09-17-F16 | At1g07770 / 40S ribosomal protein S15A (RPS15aA) | |  |  |  |  |  | | --- | --- | --- | --- | --- | |  |  |  |  |  | | F53A3.3 | | | | | | |
|  |  | RAFL11-02-D23 | At1g07770 / 40S ribosomal protein S15A (RPS15aA) | |  |  |  |  |  | | --- | --- | --- | --- | --- | |  |  |  |  |  | | F53A3.3 | | | | | | |
| H20J04.5 | | | WI5\_id:H20J04.5 |  | A | B | C | D | P | P' | N |
|  | Cluster:3-0 | |  |  | 1 | 232 | 0 | 4430 | 0.049967833 | 0.049967833 | 1 |
|  |  | RAFL05-07-D18 | At5g49510 / von Hippel-Lindau binding protein (VHL binding protein; VBP) like | |  |  |  |  |  | | --- | --- | --- | --- | --- | |  |  |  |  |  | | T06G6.9 | | | | | | |
| F13B10.2 | | | WI5\_id:F13B10.2 |  | A | B | C | D | P | P' | N |
|  | Cluster:2-1 | |  |  | 6 | 238 | 13 | 4406 | 2.9421985E-4 | 0.001765319 | 6 |
|  |  | RAFL02-10-A09 | At1g33140 / 60S ribosomal protein L9 (RPL90A/C) | |  |  |  |  |  | | --- | --- | --- | --- | --- | |  |  |  |  |  | | R13A5.8 | | | | | | |
|  |  | RAFL07-15-M07 | At1g04480 / 60S ribosomal protein L23 (RPL23A) | |  |  |  |  |  | | --- | --- | --- | --- | --- | |  |  |  |  |  | | B0336.10 | | | | | | |
|  |  | RAFL05-17-L17 | At3g55280 / 60S ribosomal protein L23A (RPL23aB) | |  |  |  |  |  | | --- | --- | --- | --- | --- | |  |  |  |  |  | | F52B5.6 | | | | | | |
|  |  | RAFL11-12-H04 | At3g25520 / 60S ribosomal protein L5 (RPL5A) | |  |  |  |  |  | | --- | --- | --- | --- | --- | |  |  |  |  |  | | F54C9.5 | | | | | | |
|  |  | RAFL05-18-P15 | At1g04480 / 60S ribosomal protein L23 (RPL23A) | |  |  |  |  |  | | --- | --- | --- | --- | --- | |  |  |  |  |  | | B0336.10 | | | | | | |
|  |  | RAFL11-07-B21 | At3g25520 / 60S ribosomal protein L5 (RPL5A) | |  |  |  |  |  | | --- | --- | --- | --- | --- | |  |  |  |  |  | | F54C9.5 | | | | | | |
|  | Cluster:3-0 | |  |  | 5 | 228 | 14 | 4416 | 0.0019486465 | 0.011691879 | 6 |
|  |  | RAFL07-10-D02 | At1g14320 / 60S ribosomal protein L10 (RPL10A)/Wilm's tumor suppressor protein-related | |  |  |  |  |  | | --- | --- | --- | --- | --- | |  |  |  |  |  | | F10B5.1 | | | | | | |
|  |  | RAFL09-10-P09 | At4g36130 / 60S ribosomal protein L8 (RPL8C) | |  |  |  |  |  | | --- | --- | --- | --- | --- | |  |  |  |  |  | | B0250.1 | | | | | | |
|  |  | RAFL08-10-G08 | At3g09630 / 60S ribosomal protein L4/L1 (RPL4A) | |  |  |  |  |  | | --- | --- | --- | --- | --- | |  |  |  |  |  | | B0041.4 | | | | | | |
|  |  | RAFL08-13-M06 | At3g11250 / 60S acidic ribosomal protein P0 (RPP0C) | |  |  |  |  |  | | --- | --- | --- | --- | --- | |  |  |  |  |  | | F25H2.10 | | | | | | |
|  |  | RAFL09-12-B12 | At4g36130 / 60S ribosomal protein L8 (RPL8C) | |  |  |  |  |  | | --- | --- | --- | --- | --- | |  |  |  |  |  | | B0250.1 | | | | | | |
|  | Cluster:2-0 | |  |  | 4 | 146 | 15 | 4498 | 0.002734157 | 0.016404942 | 6 |
|  |  | RAFL04-19-O24 | At5g02610 / 60S ribosomal protein L35 (RPL35D) | |  |  |  |  |  | | --- | --- | --- | --- | --- | |  |  |  |  |  | | ZK652.4 | | | | | | |
|  |  | RAFL06-10-L13 | At3g09630 / 60S ribosomal protein L4/L1 (RPL4A) | |  |  |  |  |  | | --- | --- | --- | --- | --- | |  |  |  |  |  | | B0041.4 | | | | | | |
|  |  | RAFL11-12-M17 | At3g25520 / 60S ribosomal protein L5 (RPL5A) | |  |  |  |  |  | | --- | --- | --- | --- | --- | |  |  |  |  |  | | F54C9.5 | | | | | | |
|  |  | RAFL03-06-H07 | At1g33140 / 60S ribosomal protein L9 (RPL90A/C) | |  |  |  |  |  | | --- | --- | --- | --- | --- | |  |  |  |  |  | | R13A5.8 | | | | | | |
| W06D4.6 | | | WI5\_id:W06D4.6 |  | A | B | C | D | P | P' | N |
|  | Cluster:3-0 | |  |  | 3 | 230 | 4 | 4426 | 0.0037112702 | 0.014845081 | 4 |
|  |  | RAFL04-13-C01 | At1g56070 / elongation factor -related | |  |  |  |  |  | | --- | --- | --- | --- | --- | |  |  |  |  |  | | F25H5.4 | | | | | | |
|  |  | RAFL08-12-J19 | At1g56070 / elongation factor -related | |  |  |  |  |  | | --- | --- | --- | --- | --- | |  |  |  |  |  | | F25H5.4 | | | | | | |
|  |  | RAFL07-10-D07 | At1g56070 / elongation factor -related | |  |  |  |  |  | | --- | --- | --- | --- | --- | |  |  |  |  |  | | F25H5.4 | | | | | | |
|  | Cluster:4-0 | |  |  | 2 | 238 | 5 | 4418 | 0.046670057 | 0.18668023 | 4 |
|  |  | RAFL07-11-O12 | At1g56070 / elongation factor -related | |  |  |  |  |  | | --- | --- | --- | --- | --- | |  |  |  |  |  | | F25H5.4 | | | | | | |
|  |  | RAFL04-17-N12 | At1g56070 / elongation factor -related | |  |  |  |  |  | | --- | --- | --- | --- | --- | |  |  |  |  |  | | F25H5.4 | | | | | | |
| Y49E10.1 | | | WI5\_id:Y49E10.1 |  | A | B | C | D | P | P' | N |
|  | Cluster:7-0 | |  |  | 2 | 245 | 4 | 4412 | 0.036386058 | 0.18193029 | 5 |
|  |  | RAFL05-04-D08 | At1g64520 / 26S proteasome regulatory subunit (RPN12), putative | |  |  |  |  |  | | --- | --- | --- | --- | --- | |  |  |  |  |  | | ZK20.5 | | | | | | |
|  |  | RAFL06-13-G03 | At1g64520 / 26S proteasome regulatory subunit (RPN12), putative | |  |  |  |  |  | | --- | --- | --- | --- | --- | |  |  |  |  |  | | ZK20.5 | | | | | | |
| B0507.1 | | | WI5\_id:B0507.1 |  | A | B | C | D | P | P' | N |
|  | Cluster:2-1 | |  |  | 2 | 242 | 2 | 4417 | 0.01525385 | 0.045761548 | 3 |
|  |  | RAFL11-12-H04 | At3g25520 / 60S ribosomal protein L5 (RPL5A) | |  |  |  |  |  | | --- | --- | --- | --- | --- | |  |  |  |  |  | | F54C9.5  60S ribosomal protein L5 | | | | | | |
|  |  | RAFL11-07-B21 | At3g25520 / 60S ribosomal protein L5 (RPL5A) | |  |  |  |  |  | | --- | --- | --- | --- | --- | |  |  |  |  |  | | F54C9.5  60S ribosomal protein L5 | | | | | | |
| F58F6.4 | | | WI5\_id:F58F6.4 |  | A | B | C | D | P | P' | N |
|  | Cluster:2-0 | |  |  | 1 | 149 | 0 | 4513 | 0.03216813 | 0.03216813 | 1 |
|  |  | RAFL08-12-D04 | At5g20290 / 40S ribosomal protein S8 (RPS8A) | |  |  |  |  |  | | --- | --- | --- | --- | --- | |  |  |  |  |  | | F42C5.8  40S ribosomal protein S8 | | | | | | |
| F33H1.2 | | | WI5\_id:F33H1.2 |  | A | B | C | D | P | P' | N |
|  | Cluster:1-2 | |  |  | 1 | 173 | 0 | 4489 | 0.037315033 | 0.037315033 | 1 |
|  |  | RAFL06-13-M02 | At1g13440 / glyceraldehyde-3-phosphate dehydrogenase -related | |  |  |  |  |  | | --- | --- | --- | --- | --- | |  |  |  |  |  | | F33H1.2 | | | | | | |
| Y48G8AL.8 | | | WI5\_id:Y48G8AL.8 |  | A | B | C | D | P | P' | N |
|  | Cluster:3-0 | |  |  | 3 | 230 | 8 | 4422 | 0.015076893 | 0.09046136 | 6 |
|  |  | RAFL07-10-D02 | At1g14320 / 60S ribosomal protein L10 (RPL10A)/Wilm's tumor suppressor protein-related | |  |  |  |  |  | | --- | --- | --- | --- | --- | |  |  |  |  |  | | F10B5.1 | | | | | | |
|  |  | RAFL09-10-P09 | At4g36130 / 60S ribosomal protein L8 (RPL8C) | |  |  |  |  |  | | --- | --- | --- | --- | --- | |  |  |  |  |  | | B0250.1 | | | | | | |
|  |  | RAFL09-12-B12 | At4g36130 / 60S ribosomal protein L8 (RPL8C) | |  |  |  |  |  | | --- | --- | --- | --- | --- | |  |  |  |  |  | | B0250.1 | | | | | | |
|  | Cluster:2-1 | |  |  | 3 | 241 | 8 | 4411 | 0.017076377 | 0.10245825 | 6 |
|  |  | RAFL05-17-L17 | At3g55280 / 60S ribosomal protein L23A (RPL23aB) | |  |  |  |  |  | | --- | --- | --- | --- | --- | |  |  |  |  |  | | F52B5.6 | | | | | | |
|  |  | RAFL11-12-H04 | At3g25520 / 60S ribosomal protein L5 (RPL5A) | |  |  |  |  |  | | --- | --- | --- | --- | --- | |  |  |  |  |  | | F54C9.5 | | | | | | |
|  |  | RAFL11-07-B21 | At3g25520 / 60S ribosomal protein L5 (RPL5A) | |  |  |  |  |  | | --- | --- | --- | --- | --- | |  |  |  |  |  | | F54C9.5 | | | | | | |
|  | Cluster:2-0 | |  |  | 2 | 148 | 9 | 4504 | 0.046735216 | 0.2804113 | 6 |
|  |  | RAFL04-19-O24 | At5g02610 / 60S ribosomal protein L35 (RPL35D) | |  |  |  |  |  | | --- | --- | --- | --- | --- | |  |  |  |  |  | | ZK652.4 | | | | | | |
|  |  | RAFL11-12-M17 | At3g25520 / 60S ribosomal protein L5 (RPL5A) | |  |  |  |  |  | | --- | --- | --- | --- | --- | |  |  |  |  |  | | F54C9.5 | | | | | | |
| ZK849.2 | | | WI5\_id:ZK849.2 |  | A | B | C | D | P | P' | N |
|  | Cluster:8-0 | |  |  | 1 | 108 | 1 | 4553 | 0.0462095 | 0.092419 | 2 |
|  |  | RAFL05-03-L12 | At1g47128 / cysteine proteinase RD21A | |  |  |  |  |  | | --- | --- | --- | --- | --- | |  |  |  |  |  | | T03E6.7  cathepsin-like protease | | | | | | |
| C04A11.1 | | | WI5\_id:C04A11.1 |  | A | B | C | D | P | P' | N |
|  | Cluster:2-0 | |  |  | 1 | 149 | 0 | 4513 | 0.03216813 | 0.03216813 | 1 |
|  |  | RAFL05-02-F20 | At5g65360 / histone H3 | |  |  |  |  |  | | --- | --- | --- | --- | --- | |  |  |  |  |  | | F45E1.6 | | | | | | |
| C16C8.16 | | | WI5\_id:C16C8.16 |  | A | B | C | D | P | P' | N |
|  | Cluster:4-1 | |  |  | 2 | 308 | 0 | 4353 | 0.0044063856 | 0.0044063856 | 1 |
|  |  | RAFL05-04-G23 | At1g77440 / 20S proteasome beta subunit C (PBC2) | |  |  |  |  |  | | --- | --- | --- | --- | --- | |  |  |  |  |  | | Y38A8.2 | | | | | | |
|  |  | RAFL05-21-D23 | At1g21720 / 20S proteasome beta subunit C (PBC1) | |  |  |  |  |  | | --- | --- | --- | --- | --- | |  |  |  |  |  | | Y38A8.2 | | | | | | |
| B0272.1 | | | WI5\_id:B0272.1 |  | A | B | C | D | P | P' | N |
|  | Cluster:1-0 | |  |  | 2 | 147 | 4 | 4510 | 0.013980698 | 0.05592279 | 4 |
|  |  | RAFL09-11-P11 | At1g20010 / tubulin beta-5 chain (TUB5) | |  |  |  |  |  | | --- | --- | --- | --- | --- | |  |  |  |  |  | | B0272.1 | | | | | | |
|  |  | RAFL04-16-G05 | At5g44340 / tubulin beta-4 chain (TUB4) | |  |  |  |  |  | | --- | --- | --- | --- | --- | |  |  |  |  |  | | B0272.1 | | | | | | |
|  | Cluster:2-1 | |  |  | 2 | 242 | 4 | 4415 | 0.0355681 | 0.1422724 | 4 |
|  |  | RAFL05-09-F01 | At5g44340 / tubulin beta-4 chain (TUB4) | |  |  |  |  |  | | --- | --- | --- | --- | --- | |  |  |  |  |  | | B0272.1 | | | | | | |
|  |  | RAFL06-12-I16 | At2g29550 / tubulin beta-7 chain (TUB7) | |  |  |  |  |  | | --- | --- | --- | --- | --- | |  |  |  |  |  | | B0272.1 | | | | | | |
| C30C11.2 | | | WI5\_id:C30C11.2 |  | A | B | C | D | P | P' | N |
|  | Cluster:6-1 | |  |  | 3 | 312 | 6 | 4342 | 0.018891884 | 0.1133513 | 6 |
|  |  | RAFL05-08-I15 | At4g24820 / 26S proteasome regulatory subunit (RPN7), putative | |  |  |  |  |  | | --- | --- | --- | --- | --- | |  |  |  |  |  | | F49C12.8 | | | | | | |
|  |  | RAFL07-13-D20 | At4g24820 / 26S proteasome regulatory subunit (RPN7), putative | |  |  |  |  |  | | --- | --- | --- | --- | --- | |  |  |  |  |  | | F49C12.8 | | | | | | |
|  |  | RAFL11-04-H03 | At1g20200 / 26S proteasome regulatory subunit S3 (RPN3), putative | |  |  |  |  |  | | --- | --- | --- | --- | --- | |  |  |  |  |  | | C30C11.2 | | | | | | |
| Y57G11C.16 | | | WI5\_id:Y57G11C.16 |  | A | B | C | D | P | P' | N |
|  | Cluster:2-1 | |  |  | 3 | 241 | 3 | 4416 | 0.0025160299 | 0.0075480896 | 3 |
|  |  | RAFL07-13-J18 | At2g37270 / 40S ribosomal protein S5 (RPS5A) | |  |  |  |  |  | | --- | --- | --- | --- | --- | |  |  |  |  |  | | T05E11.1 | | | | | | |
|  |  | RAFL05-16-H14 | At1g04270 / 40S ribosomal protein S15 (RPS15A) | |  |  |  |  |  | | --- | --- | --- | --- | --- | |  |  |  |  |  | | F36A2.6 | | | | | | |
|  |  | RAFL04-19-M20 | At1g58380 / 40S ribosomal protein S2 (RPS2A) | |  |  |  |  |  | | --- | --- | --- | --- | --- | |  |  |  |  |  | | C49H3.11 | | | | | | |
|  | Cluster:3-0 | |  |  | 2 | 231 | 4 | 4426 | 0.032636177 | 0.097908534 | 3 |
|  |  | RAFL08-09-E20 | At2g41840 / 40S ribosomal protein S2 (RPS2C) | |  |  |  |  |  | | --- | --- | --- | --- | --- | |  |  |  |  |  | | C49H3.11 | | | | | | |
|  |  | RAFL06-07-B02 | At3g11940 / 40S ribosomal protein S5 (RPS5B) | |  |  |  |  |  | | --- | --- | --- | --- | --- | |  |  |  |  |  | | T05E11.1 | | | | | | |
| ZK637.8 | | | WI5\_id:ZK637.8 |  | A | B | C | D | P | P' | N |
|  | Cluster:4-1 | |  |  | 2 | 308 | 2 | 4351 | 0.024166461 | 0.07249938 | 3 |
|  |  | RAFL04-09-A19 | At4g38510 / probable H+-transporting ATPase | |  |  |  |  |  | | --- | --- | --- | --- | --- | |  |  |  |  |  | | F20B6.2 | | | | | | |
|  |  | RAFL07-17-H08 | At4g38510 / probable H+-transporting ATPase | |  |  |  |  |  | | --- | --- | --- | --- | --- | |  |  |  |  |  | | F20B6.2 | | | | | | |
| W09D10.3 | | | WI5\_id:W09D10.3 |  | A | B | C | D | P | P' | N |
|  | Cluster:3-0 | |  |  | 1 | 232 | 0 | 4430 | 0.049967833 | 0.049967833 | 1 |
|  |  | RAFL08-13-M06 | At3g11250 / 60S acidic ribosomal protein P0 (RPP0C) | |  |  |  |  |  | | --- | --- | --- | --- | --- | |  |  |  |  |  | | F25H2.10 | | | | | | |
| T04H1.6 | | | WI5\_id:T04H1.6 |  | A | B | C | D | P | P' | N |
|  | Cluster:3-0 | |  |  | 1 | 232 | 0 | 4430 | 0.049967833 | 0.049967833 | 1 |
|  |  | RAFL05-07-D18 | At5g49510 / von Hippel-Lindau binding protein (VHL binding protein; VBP) like | |  |  |  |  |  | | --- | --- | --- | --- | --- | |  |  |  |  |  | | T06G6.9 | | | | | | |
| K05C4.1 | | | WI5\_id:K05C4.1 |  | A | B | C | D | P | P' | N |
|  | Cluster:4-1 | |  |  | 3 | 307 | 1 | 4352 | 0.0011071523 | 0.0022143046 | 2 |
|  |  | RAFL07-08-J17 | At1g13060 / 20S proteasome beta subunit E1 (PBE1) | |  |  |  |  |  | | --- | --- | --- | --- | --- | |  |  |  |  |  | | K05C4.1 | | | | | | |
|  |  | RAFL05-04-L16 | At1g79210 / 20S proteasome alpha subunit B, putative | |  |  |  |  |  | | --- | --- | --- | --- | --- | |  |  |  |  |  | | D1054.2 | | | | | | |
|  |  | RAFL05-12-G18 | At4g14800 / 20S proteasome beta subunit D2 (PBD2) | |  |  |  |  |  | | --- | --- | --- | --- | --- | |  |  |  |  |  | | T20F5.2 | | | | | | |
| C03A7.4 | | | WI5\_id:C03A7.4 |  | A | B | C | D | P | P' | N |
|  | Cluster:4-1 | |  |  | 2 | 308 | 3 | 4350 | 0.038523052 | 0.15409221 | 4 |
|  |  | RAFL05-04-G23 | At1g77440 / 20S proteasome beta subunit C (PBC2) | |  |  |  |  |  | | --- | --- | --- | --- | --- | |  |  |  |  |  | | Y38A8.2  Peptidase | | | | | | |
|  |  | RAFL05-21-D23 | At1g21720 / 20S proteasome beta subunit C (PBC1) | |  |  |  |  |  | | --- | --- | --- | --- | --- | |  |  |  |  |  | | Y38A8.2  Peptidase | | | | | | |
| F32D8.4 | | | WI5\_id:F32D8.4 |  | A | B | C | D | P | P' | N |
|  | Cluster:10-2 | |  |  | 1 | 108 | 0 | 4554 | 0.02337551 | 0.02337551 | 1 |
|  |  | RAFL11-12-H18 | At4g17530 / ras-related small GTP-binding protein RAB1c | |  |  |  |  |  | | --- | --- | --- | --- | --- | |  |  |  |  |  | | C39F7.4 | | | | | | |
| C37C3.6 | | | WI5\_id:C37C3.6 |  | A | B | C | D | P | P' | N |
|  | Cluster:5-0 | |  |  | 4 | 73 | 1 | 4585 | 3.3961462E-7 | 6.7922923E-7 | 2 |
|  |  | RAFL04-15-M13 | At5g56030 / heat shock protein 81-2 (HSP81-2) | |  |  |  |  |  | | --- | --- | --- | --- | --- | |  |  |  |  |  | | C47E8.5  heat shock protein (HSP90) | | | | | | |
|  |  | RAFL07-13-H08 | At5g56010 / heat shock protein, putative | |  |  |  |  |  | | --- | --- | --- | --- | --- | |  |  |  |  |  | | C47E8.5  heat shock protein (HSP90) | | | | | | |
|  |  | RAFL09-06-O18 | At5g56030 / heat shock protein 81-2 (HSP81-2) | |  |  |  |  |  | | --- | --- | --- | --- | --- | |  |  |  |  |  | | C47E8.5  heat shock protein (HSP90) | | | | | | |
|  |  | RAFL05-16-L15 | At5g56010 / heat shock protein, putative | |  |  |  |  |  | | --- | --- | --- | --- | --- | |  |  |  |  |  | | C47E8.5  heat shock protein (HSP90) | | | | | | |
| F27C8.2 | | | WI5\_id:F27C8.2 |  | A | B | C | D | P | P' | N |
|  | Cluster:8-0 | |  |  | 1 | 108 | 1 | 4553 | 0.0462095 | 0.092419 | 2 |
|  |  | RAFL09-13-J20 | At1g69410 / Eukaryotic initiation factor 5A -related | |  |  |  |  |  | | --- | --- | --- | --- | --- | |  |  |  |  |  | | T05G5.10 | | | | | | |
| C56C10.7 | | | WI5\_id:C56C10.7 |  | A | B | C | D | P | P' | N |
|  | Cluster:3-0 | |  |  | 1 | 232 | 0 | 4430 | 0.049967833 | 0.049967833 | 1 |
|  |  | RAFL04-16-E04 | At3g51260 / 20S proteasome alpha subunit D (PAD1) | |  |  |  |  |  | | --- | --- | --- | --- | --- | |  |  |  |  |  | | C36B1.4 | | | | | | |
| C09G4.3 | | | WI5\_id:C09G4.3 |  | A | B | C | D | P | P' | N |
|  | Cluster:2-0 | |  |  | 1 | 149 | 0 | 4513 | 0.03216813 | 0.03216813 | 1 |
|  |  | RAFL03-08-O03 | At1g07940 / elongation factor 1-alpha (EF-1-alpha) | |  |  |  |  |  | | --- | --- | --- | --- | --- | |  |  |  |  |  | | F31E3.5 | | | | | | |
| F35G12.9 | | | WI5\_id:F35G12.9 |  | A | B | C | D | P | P' | N |
|  | Cluster:5-1 | |  |  | 2 | 280 | 2 | 4379 | 0.02015854 | 0.06047562 | 3 |
|  |  | RAFL05-08-F18 | At3g56090 / expressed protein | |  |  |  |  |  | | --- | --- | --- | --- | --- | |  |  |  |  |  | | C54F6.14 | | | | | | |
|  |  | RAFL07-12-N15 | At2g20000 / cell division cycle (CDC) protein - related | |  |  |  |  |  | | --- | --- | --- | --- | --- | |  |  |  |  |  | | Y110A7A.17 | | | | | | |
| B0222.6 | | | WI5\_id:B0222.6 |  | A | B | C | D | P | P' | N |
|  | Cluster:4-0 | |  |  | 2 | 238 | 1 | 4422 | 0.0076462985 | 0.015292597 | 2 |
|  |  | RAFL06-07-B07 | At5g55190 / GTP-binding protein atran3, putative | |  |  |  |  |  | | --- | --- | --- | --- | --- | |  |  |  |  |  | | K01G5.4 | | | | | | |
|  |  | RAFL05-17-C16 | At5g55190 / GTP-binding protein atran3, putative | |  |  |  |  |  | | --- | --- | --- | --- | --- | |  |  |  |  |  | | K01G5.4 | | | | | | |
| T22H2.5 | | | WI5\_id:T22H2.5 |  | A | B | C | D | P | P' | N |
|  | Cluster:5-0 | |  |  | 1 | 76 | 0 | 4586 | 0.016512975 | 0.016512975 | 1 |
|  |  | RAFL04-09-J19 | At1g56450 / 20S proteasome beta subunit G1 (PBG1) | |  |  |  |  |  | | --- | --- | --- | --- | --- | |  |  |  |  |  | | F39H11.5  Yeast NIP80 protein like | | | | | | |
| T24F1.2 | | | WI5\_id:T24F1.2 |  | A | B | C | D | P | P' | N |
|  | Cluster:4-0 | |  |  | 2 | 238 | 1 | 4422 | 0.0076462985 | 0.015292597 | 2 |
|  |  | RAFL06-07-B07 | At5g55190 / GTP-binding protein atran3, putative | |  |  |  |  |  | | --- | --- | --- | --- | --- | |  |  |  |  |  | | K01G5.4 | | | | | | |
|  |  | RAFL05-17-C16 | At5g55190 / GTP-binding protein atran3, putative | |  |  |  |  |  | | --- | --- | --- | --- | --- | |  |  |  |  |  | | K01G5.4 | | | | | | |
| F49C12.8 | | | WI5\_id:F49C12.8 |  | A | B | C | D | P | P' | N |
|  | Cluster:6-1 | |  |  | 3 | 312 | 6 | 4342 | 0.018891884 | 0.1133513 | 6 |
|  |  | RAFL05-08-I15 | At4g24820 / 26S proteasome regulatory subunit (RPN7), putative | |  |  |  |  |  | | --- | --- | --- | --- | --- | |  |  |  |  |  | | F49C12.8 | | | | | | |
|  |  | RAFL07-13-D20 | At4g24820 / 26S proteasome regulatory subunit (RPN7), putative | |  |  |  |  |  | | --- | --- | --- | --- | --- | |  |  |  |  |  | | F49C12.8 | | | | | | |
|  |  | RAFL11-04-H03 | At1g20200 / 26S proteasome regulatory subunit S3 (RPN3), putative | |  |  |  |  |  | | --- | --- | --- | --- | --- | |  |  |  |  |  | | C30C11.2 | | | | | | |
| B0336.2 | | | WI5\_id:B0336.2 |  | A | B | C | D | P | P' | N |
|  | Cluster:4-2 | |  |  | 1 | 134 | 0 | 4528 | 0.028951319 | 0.028951319 | 1 |
|  |  | RAFL05-12-C15 | At1g10630 / ADP-ribosylation factor, putative | |  |  |  |  |  | | --- | --- | --- | --- | --- | |  |  |  |  |  | | B0336.2 | | | | | | |
| ZK675.2 | | | WI5\_id:ZK675.2 |  | A | B | C | D | P | P' | N |
|  | Cluster:1-0 | |  |  | 1 | 148 | 0 | 4514 | 0.031953678 | 0.031953678 | 1 |
|  |  | RAFL04-19-J20 | At3g50270 / hydroxycinnamoyl/benzoyltransferase-related | |  |  |  |  |  | | --- | --- | --- | --- | --- | |  |  |  |  |  | | K04G7.10 | | | | | | |
| D2005.4 | | | WI5\_id:D2005.4 |  | A | B | C | D | P | P' | N |
|  | Cluster:4-0 | |  |  | 2 | 238 | 1 | 4422 | 0.0076462985 | 0.015292597 | 2 |
|  |  | RAFL06-07-B07 | At5g55190 / GTP-binding protein atran3, putative | |  |  |  |  |  | | --- | --- | --- | --- | --- | |  |  |  |  |  | | K01G5.4 | | | | | | |
|  |  | RAFL05-17-C16 | At5g55190 / GTP-binding protein atran3, putative | |  |  |  |  |  | | --- | --- | --- | --- | --- | |  |  |  |  |  | | K01G5.4 | | | | | | |
| Y41C4A.14 | | | WI5\_id:Y41C4A.14 |  | A | B | C | D | P | P' | N |
|  | Cluster:4-1 | |  |  | 2 | 308 | 3 | 4350 | 0.038523052 | 0.15409221 | 4 |
|  |  | RAFL05-04-G23 | At1g77440 / 20S proteasome beta subunit C (PBC2) | |  |  |  |  |  | | --- | --- | --- | --- | --- | |  |  |  |  |  | | Y38A8.2 | | | | | | |
|  |  | RAFL05-21-D23 | At1g21720 / 20S proteasome beta subunit C (PBC1) | |  |  |  |  |  | | --- | --- | --- | --- | --- | |  |  |  |  |  | | Y38A8.2 | | | | | | |
| F10C5.1 | | | WI5\_id:F10C5.1 |  | A | B | C | D | P | P' | N |
|  | Cluster:0-2 | |  |  | 1 | 78 | 2 | 4582 | 0.049979966 | 0.1499399 | 3 |
|  |  | RAFL04-17-G02 | At1g56340 / calreticulin 1 (CRT1) | |  |  |  |  |  | | --- | --- | --- | --- | --- | |  |  |  |  |  | | Y38A10A.5 | | | | | | |
| F26A3.2 | | | WI5\_id:F26A3.2 |  | A | B | C | D | P | P' | N |
|  | Cluster:1-0 | |  |  | 1 | 148 | 0 | 4514 | 0.031953678 | 0.031953678 | 1 |
|  |  | RAFL04-19-J20 | At3g50270 / hydroxycinnamoyl/benzoyltransferase-related | |  |  |  |  |  | | --- | --- | --- | --- | --- | |  |  |  |  |  | | K04G7.10 | | | | | | |
| R12E2.3 | | | WI5\_id:R12E2.3 |  | A | B | C | D | P | P' | N |
|  | Cluster:7-0 | |  |  | 2 | 245 | 5 | 4411 | 0.049187053 | 0.29512233 | 6 |
|  |  | RAFL05-04-D08 | At1g64520 / 26S proteasome regulatory subunit (RPN12), putative | |  |  |  |  |  | | --- | --- | --- | --- | --- | |  |  |  |  |  | | ZK20.5 | | | | | | |
|  |  | RAFL06-13-G03 | At1g64520 / 26S proteasome regulatory subunit (RPN12), putative | |  |  |  |  |  | | --- | --- | --- | --- | --- | |  |  |  |  |  | | ZK20.5 | | | | | | |
| F53G12.10 | | | WI5\_id:F53G12.10 |  | A | B | C | D | P | P' | N |
|  | Cluster:2-1 | |  |  | 4 | 240 | 2 | 4417 | 1.0099356E-4 | 3.0298068E-4 | 3 |
|  |  | RAFL05-17-L17 | At3g55280 / 60S ribosomal protein L23A (RPL23aB) | |  |  |  |  |  | | --- | --- | --- | --- | --- | |  |  |  |  |  | | F52B5.6 | | | | | | |
|  |  | RAFL11-12-H04 | At3g25520 / 60S ribosomal protein L5 (RPL5A) | |  |  |  |  |  | | --- | --- | --- | --- | --- | |  |  |  |  |  | | F54C9.5 | | | | | | |
|  |  | RAFL04-18-N22 | At2g44120 / 60S ribosomal protein L7 (RPL7C) | |  |  |  |  |  | | --- | --- | --- | --- | --- | |  |  |  |  |  | | F53G12.10 | | | | | | |
|  |  | RAFL11-07-B21 | At3g25520 / 60S ribosomal protein L5 (RPL5A) | |  |  |  |  |  | | --- | --- | --- | --- | --- | |  |  |  |  |  | | F54C9.5 | | | | | | |
| F58F12.1 | | | WI5\_id:F58F12.1 |  | A | B | C | D | P | P' | N |
|  | Cluster:4-1 | |  |  | 2 | 308 | 2 | 4351 | 0.024166461 | 0.07249938 | 3 |
|  |  | RAFL04-15-D14 | At5g08680 / H+-transporting ATP synthase beta chain -related | |  |  |  |  |  | | --- | --- | --- | --- | --- | |  |  |  |  |  | | C34E10.6 | | | | | | |
|  |  | RAFL09-17-J21 | At5g08690 / H+-transporting ATP synthase beta chain (mitochondrial) -related | |  |  |  |  |  | | --- | --- | --- | --- | --- | |  |  |  |  |  | | C34E10.6 | | | | | | |
| F28B3.8 | | | WI5\_id:F28B3.8 |  | A | B | C | D | P | P' | N |
|  | Cluster:4-0 | |  |  | 2 | 238 | 1 | 4422 | 0.0076462985 | 0.015292597 | 2 |
|  |  | RAFL06-07-B07 | At5g55190 / GTP-binding protein atran3, putative | |  |  |  |  |  | | --- | --- | --- | --- | --- | |  |  |  |  |  | | K01G5.4 | | | | | | |
|  |  | RAFL05-17-C16 | At5g55190 / GTP-binding protein atran3, putative | |  |  |  |  |  | | --- | --- | --- | --- | --- | |  |  |  |  |  | | K01G5.4 | | | | | | |
| K06H7.6 | | | WI5\_id:K06H7.6 |  | A | B | C | D | P | P' | N |
|  | Cluster:1-2 | |  |  | 1 | 173 | 0 | 4489 | 0.037315033 | 0.037315033 | 1 |
|  |  | RAFL06-11-J01 | At1g09690 / 60S ribosomal protein L21 (RPL21C) | |  |  |  |  |  | | --- | --- | --- | --- | --- | |  |  |  |  |  | | C14B9.7 | | | | | | |
| T04C12.6 | | | WI5\_id:T04C12.6 |  | A | B | C | D | P | P' | N |
|  | Cluster:4-0 | |  |  | 2 | 238 | 2 | 4421 | 0.0147742275 | 0.044322684 | 3 |
|  |  | RAFL06-07-B07 | At5g55190 / GTP-binding protein atran3, putative | |  |  |  |  |  | | --- | --- | --- | --- | --- | |  |  |  |  |  | | K01G5.4 | | | | | | |
|  |  | RAFL05-17-C16 | At5g55190 / GTP-binding protein atran3, putative | |  |  |  |  |  | | --- | --- | --- | --- | --- | |  |  |  |  |  | | K01G5.4 | | | | | | |
| B0035.2 | | | WI5\_id:B0035.2 |  | A | B | C | D | P | P' | N |
|  | Cluster:1-0 | |  |  | 1 | 148 | 0 | 4514 | 0.031953678 | 0.031953678 | 1 |
|  |  | RAFL06-10-E18 | At1g50010 / tubulin alpha-2/alpha-4 chain (TUA2) | |  |  |  |  |  | | --- | --- | --- | --- | --- | |  |  |  |  |  | | F26E4.8  TBA-2 tubulin alpha-2 chain | | | | | | |
| R08D7.3 | | | WI5\_id:R08D7.3 |  | A | B | C | D | P | P' | N |
|  | Cluster:8-0 | |  |  | 1 | 108 | 0 | 4554 | 0.02337551 | 0.02337551 | 1 |
|  |  | RAFL05-03-L12 | At1g47128 / cysteine proteinase RD21A | |  |  |  |  |  | | --- | --- | --- | --- | --- | |  |  |  |  |  | | T03E6.7 | | | | | | |
| T03E6.7 | | | WI5\_id:T03E6.7 |  | A | B | C | D | P | P' | N |
|  | Cluster:8-0 | |  |  | 1 | 108 | 0 | 4554 | 0.02337551 | 0.02337551 | 1 |
|  |  | RAFL05-03-L12 | At1g47128 / cysteine proteinase RD21A | |  |  |  |  |  | | --- | --- | --- | --- | --- | |  |  |  |  |  | | T03E6.7 | | | | | | |
| T28B8.5 | | | WI5\_id:T28B8.5 |  | A | B | C | D | P | P' | N |
|  | Cluster:3-0 | |  |  | 1 | 232 | 0 | 4430 | 0.049967833 | 0.049967833 | 1 |
|  |  | RAFL08-13-M06 | At3g11250 / 60S acidic ribosomal protein P0 (RPP0C) | |  |  |  |  |  | | --- | --- | --- | --- | --- | |  |  |  |  |  | | F25H2.10 | | | | | | |
| K04G7.10 | | | WI5\_id:K04G7.10 |  | A | B | C | D | P | P' | N |
|  | Cluster:1-0 | |  |  | 1 | 148 | 0 | 4514 | 0.031953678 | 0.031953678 | 1 |
|  |  | RAFL04-19-J20 | At3g50270 / hydroxycinnamoyl/benzoyltransferase-related | |  |  |  |  |  | | --- | --- | --- | --- | --- | |  |  |  |  |  | | K04G7.10 | | | | | | |
| F23F12.6 | | | WI5\_id:F23F12.6 |  | A | B | C | D | P | P' | N |
|  | Cluster:6-1 | |  |  | 4 | 311 | 12 | 4336 | 0.019434473 | 0.19434473 | 10 |
|  |  | RAFL08-19-N19 | At3g05530 / 26S proteasome AAA-ATPase subunit RPT5a | |  |  |  |  |  | | --- | --- | --- | --- | --- | |  |  |  |  |  | | F56H1.4 | | | | | | |
|  |  | RAFL05-08-I15 | At4g24820 / 26S proteasome regulatory subunit (RPN7), putative | |  |  |  |  |  | | --- | --- | --- | --- | --- | |  |  |  |  |  | | F49C12.8 | | | | | | |
|  |  | RAFL07-13-D20 | At4g24820 / 26S proteasome regulatory subunit (RPN7), putative | |  |  |  |  |  | | --- | --- | --- | --- | --- | |  |  |  |  |  | | F49C12.8 | | | | | | |
|  |  | RAFL11-04-H03 | At1g20200 / 26S proteasome regulatory subunit S3 (RPN3), putative | |  |  |  |  |  | | --- | --- | --- | --- | --- | |  |  |  |  |  | | C30C11.2 | | | | | | |
| Y47D3A.29 | | | WI5\_id:Y47D3A.29 |  | A | B | C | D | P | P' | N |
|  | Cluster:6-2 | |  |  | 1 | 175 | 0 | 4487 | 0.03774394 | 0.03774394 | 1 |
|  |  | RAFL11-07-O15 | At3g28730 / recombination signal sequence recognition protein -related | |  |  |  |  |  | | --- | --- | --- | --- | --- | |  |  |  |  |  | | T20B12.8 | | | | | | |
| R03G5.2 | | | WI5\_id:R03G5.2 |  | A | B | C | D | P | P' | N |
|  | Cluster:9-0 | |  |  | 4 | 28 | 1 | 4630 | 9.095051E-9 | 1.8190102E-8 | 2 |
|  |  | RAFL08-09-C23 | At1g54100 / aldehyde dehydrogenase, putative (ALDH) | |  |  |  |  |  | | --- | --- | --- | --- | --- | |  |  |  |  |  | | F01F1.6 | | | | | | |
|  |  | RAFL04-09-D07 | At1g54100 / aldehyde dehydrogenase, putative (ALDH) | |  |  |  |  |  | | --- | --- | --- | --- | --- | |  |  |  |  |  | | F01F1.6 | | | | | | |
|  |  | RAFL05-21-E06 | At1g54100 / aldehyde dehydrogenase, putative (ALDH) | |  |  |  |  |  | | --- | --- | --- | --- | --- | |  |  |  |  |  | | F01F1.6 | | | | | | |
|  |  | RAFL08-15-L09 | At1g54100 / aldehyde dehydrogenase, putative (ALDH) | |  |  |  |  |  | | --- | --- | --- | --- | --- | |  |  |  |  |  | | F01F1.6 | | | | | | |
| T10F2.4 | | | WI5\_id:T10F2.4 |  | A | B | C | D | P | P' | N |
|  | Cluster:5-0 | |  |  | 1 | 76 | 0 | 4586 | 0.016512975 | 0.016512975 | 1 |
|  |  | RAFL04-09-J19 | At1g56450 / 20S proteasome beta subunit G1 (PBG1) | |  |  |  |  |  | | --- | --- | --- | --- | --- | |  |  |  |  |  | | F39H11.5 | | | | | | |
| T06G6.9 | | | WI5\_id:T06G6.9 |  | A | B | C | D | P | P' | N |
|  | Cluster:3-0 | |  |  | 1 | 232 | 0 | 4430 | 0.049967833 | 0.049967833 | 1 |
|  |  | RAFL05-07-D18 | At5g49510 / von Hippel-Lindau binding protein (VHL binding protein; VBP) like | |  |  |  |  |  | | --- | --- | --- | --- | --- | |  |  |  |  |  | | T06G6.9 | | | | | | |
| Y15E3A.1 | | | WI5\_id:Y15E3A.1 |  | A | B | C | D | P | P' | N |
|  | Cluster:3-0 | |  |  | 1 | 232 | 0 | 4430 | 0.049967833 | 0.049967833 | 1 |
|  |  | RAFL08-13-M06 | At3g11250 / 60S acidic ribosomal protein P0 (RPP0C) | |  |  |  |  |  | | --- | --- | --- | --- | --- | |  |  |  |  |  | | F25H2.10 | | | | | | |
| C34C12.8 | | | WI5\_id:C34C12.8 |  | A | B | C | D | P | P' | N |
|  | Cluster:4-2 | |  |  | 1 | 134 | 0 | 4528 | 0.028951319 | 0.028951319 | 1 |
|  |  | RAFL04-17-F17 | At4g37910 / heat shock protein mtHsc70-1 | |  |  |  |  |  | | --- | --- | --- | --- | --- | |  |  |  |  |  | | C37H5.8  heat shock 70 protein | | | | | | |
| T05F1.3 | | | WI5\_id:T05F1.3 |  | A | B | C | D | P | P' | N |
|  | Cluster:3-0 | |  |  | 1 | 232 | 0 | 4430 | 0.049967833 | 0.049967833 | 1 |
|  |  | RAFL04-14-I15 | At3g02080 / 40S ribosomal protein S19 (RPS19A) | |  |  |  |  |  | | --- | --- | --- | --- | --- | |  |  |  |  |  | | T05F1.3 | | | | | | |
| F08G2.5 | | | WI5\_id:F08G2.5 |  | A | B | C | D | P | P' | N |
|  | Cluster:2-0 | |  |  | 2 | 148 | 2 | 4511 | 0.00591059 | 0.017731769 | 3 |
|  |  | RAFL06-10-L13 | At3g09630 / 60S ribosomal protein L4/L1 (RPL4A) | |  |  |  |  |  | | --- | --- | --- | --- | --- | |  |  |  |  |  | | B0041.4 | | | | | | |
|  |  | RAFL03-08-O03 | At1g07940 / elongation factor 1-alpha (EF-1-alpha) | |  |  |  |  |  | | --- | --- | --- | --- | --- | |  |  |  |  |  | | F31E3.5 | | | | | | |
| Y17G7B.4 | | | WI5\_id:Y17G7B.4 |  | A | B | C | D | P | P' | N |
|  | Cluster:8-0 | |  |  | 1 | 108 | 1 | 4553 | 0.0462095 | 0.092419 | 2 |
|  |  | RAFL09-13-J20 | At1g69410 / Eukaryotic initiation factor 5A -related | |  |  |  |  |  | | --- | --- | --- | --- | --- | |  |  |  |  |  | | T05G5.10 | | | | | | |
| F29G9.5 | | | WI5\_id:F29G9.5 |  | A | B | C | D | P | P' | N |
|  | Cluster:7-0 | |  |  | 2 | 245 | 5 | 4411 | 0.049187053 | 0.29512233 | 6 |
|  |  | RAFL05-04-D08 | At1g64520 / 26S proteasome regulatory subunit (RPN12), putative | |  |  |  |  |  | | --- | --- | --- | --- | --- | |  |  |  |  |  | | ZK20.5 | | | | | | |
|  |  | RAFL06-13-G03 | At1g64520 / 26S proteasome regulatory subunit (RPN12), putative | |  |  |  |  |  | | --- | --- | --- | --- | --- | |  |  |  |  |  | | ZK20.5 | | | | | | |
| T09B4.8 | | | WI5\_id:T09B4.8 |  | A | B | C | D | P | P' | N |
|  | Cluster:4-2 | |  |  | 2 | 133 | 3 | 4525 | 0.007856598 | 0.031426392 | 4 |
|  |  | RAFL09-17-F16 | At1g07770 / 40S ribosomal protein S15A (RPS15aA) | |  |  |  |  |  | | --- | --- | --- | --- | --- | |  |  |  |  |  | | F53A3.3 | | | | | | |
|  |  | RAFL11-02-D23 | At1g07770 / 40S ribosomal protein S15A (RPS15aA) | |  |  |  |  |  | | --- | --- | --- | --- | --- | |  |  |  |  |  | | F53A3.3 | | | | | | |
| Y38F2AL.4 | | | WI5\_id:Y38F2AL.4 |  | A | B | C | D | P | P' | N |
|  | Cluster:4-1 | |  |  | 2 | 308 | 3 | 4350 | 0.038523052 | 0.15409221 | 4 |
|  |  | RAFL04-09-A19 | At4g38510 / probable H+-transporting ATPase | |  |  |  |  |  | | --- | --- | --- | --- | --- | |  |  |  |  |  | | F20B6.2 | | | | | | |
|  |  | RAFL07-17-H08 | At4g38510 / probable H+-transporting ATPase | |  |  |  |  |  | | --- | --- | --- | --- | --- | |  |  |  |  |  | | F20B6.2 | | | | | | |
| Y57G11C.10 | | | WI5\_id:Y57G11C.10 |  | A | B | C | D | P | P' | N |
|  | Cluster:6-1 | |  |  | 2 | 313 | 1 | 4347 | 0.013038642 | 0.026077284 | 2 |
|  |  | RAFL09-13-A13 | At2g44100 / GDP dissociation inhibitor | |  |  |  |  |  | | --- | --- | --- | --- | --- | |  |  |  |  |  | | Y57G11C.10 | | | | | | |
|  |  | RAFL05-11-L05 | At2g44100 / GDP dissociation inhibitor | |  |  |  |  |  | | --- | --- | --- | --- | --- | |  |  |  |  |  | | Y57G11C.10 | | | | | | |
| T05B11.1 | | | WI5\_id:T05B11.1 |  | A | B | C | D | P | P' | N |
|  | Cluster:5-0 | |  |  | 1 | 76 | 0 | 4586 | 0.016512975 | 0.016512975 | 1 |
|  |  | RAFL04-09-J19 | At1g56450 / 20S proteasome beta subunit G1 (PBG1) | |  |  |  |  |  | | --- | --- | --- | --- | --- | |  |  |  |  |  | | F39H11.5 | | | | | | |
| F37B4.7 | | | WI5\_id:F37B4.7 |  | A | B | C | D | P | P' | N |
|  | Cluster:4-2 | |  |  | 1 | 134 | 0 | 4528 | 0.028951319 | 0.028951319 | 1 |
|  |  | RAFL04-20-K07 | At3g53750 / actin (ACT3) | |  |  |  |  |  | | --- | --- | --- | --- | --- | |  |  |  |  |  | | T04C12.6 | | | | | | |
| F25B4.5 | | | WI5\_id:F25B4.5 |  | A | B | C | D | P | P' | N |
|  | Cluster:1-0 | |  |  | 1 | 148 | 0 | 4514 | 0.031953678 | 0.031953678 | 1 |
|  |  | RAFL04-19-J20 | At3g50270 / hydroxycinnamoyl/benzoyltransferase-related | |  |  |  |  |  | | --- | --- | --- | --- | --- | |  |  |  |  |  | | K04G7.10 | | | | | | |
| F56F3.5 | | | WI5\_id:F56F3.5 |  | A | B | C | D | P | P' | N |
|  | Cluster:2-0 | |  |  | 2 | 148 | 3 | 4510 | 0.009643516 | 0.028930547 | 3 |
|  |  | RAFL06-08-H02 | At3g04840 / 40S ribosomal protein S3A (RPS3aA) | |  |  |  |  |  | | --- | --- | --- | --- | --- | |  |  |  |  |  | | F56F3.5 | | | | | | |
|  |  | RAFL03-06-H09 | At3g60770 / 40S ribosomal protein S13 (RPS13A) | |  |  |  |  |  | | --- | --- | --- | --- | --- | |  |  |  |  |  | | C16A3.9 | | | | | | |
|  | Cluster:2-1 | |  |  | 2 | 242 | 3 | 4416 | 0.024550742 | 0.07365222 | 3 |
|  |  | RAFL07-15-K08 | At4g34670 / 40S ribosomal protein S3A (RPS3aB) | |  |  |  |  |  | | --- | --- | --- | --- | --- | |  |  |  |  |  | | F56F3.5 | | | | | | |
|  |  | RAFL06-14-I03 | At3g60770 / 40S ribosomal protein S13 (RPS13A) | |  |  |  |  |  | | --- | --- | --- | --- | --- | |  |  |  |  |  | | C16A3.9 | | | | | | |
| W05B5.1 | | | WI5\_id:W05B5.1 |  | A | B | C | D | P | P' | N |
|  | Cluster:4-2 | |  |  | 1 | 134 | 0 | 4528 | 0.028951319 | 0.028951319 | 1 |
|  |  | RAFL05-05-K13 | At3g52590 / ubiquitin extension protein 1 (UBQ1)/60S ribosomal protein L40 (RPL40B) | |  |  |  |  |  | | --- | --- | --- | --- | --- | |  |  |  |  |  | | ZK1010.1 | | | | | | |
| F43E2.8 | | | WI5\_id:F43E2.8 |  | A | B | C | D | P | P' | N |
|  | Cluster:2-1 | |  |  | 2 | 242 | 1 | 4418 | 0.00789916 | 0.01579832 | 2 |
|  |  | RAFL06-08-N05 | At5g28540 / luminal binding protein 1 precursor (BiP-1) (AtBP1) | |  |  |  |  |  | | --- | --- | --- | --- | --- | |  |  |  |  |  | | F43E2.8 | | | | | | |
|  |  | RAFL07-11-O19 | At5g28540 / luminal binding protein 1 precursor (BiP-1) (AtBP1) | |  |  |  |  |  | | --- | --- | --- | --- | --- | |  |  |  |  |  | | F43E2.8 | | | | | | |
| ZK1225.6 | | | WI5\_id:ZK1225.6 |  | A | B | C | D | P | P' | N |
|  | Cluster:8-0 | |  |  | 1 | 108 | 1 | 4553 | 0.0462095 | 0.092419 | 2 |
|  |  | RAFL09-13-J20 | At1g69410 / Eukaryotic initiation factor 5A -related | |  |  |  |  |  | | --- | --- | --- | --- | --- | |  |  |  |  |  | | T05G5.10 | | | | | | |
| F55A3.3 | | | WI5\_id:F55A3.3 |  | A | B | C | D | P | P' | N |
|  | Cluster:6-2 | |  |  | 1 | 175 | 0 | 4487 | 0.03774394 | 0.03774394 | 1 |
|  |  | RAFL11-07-O15 | At3g28730 / recombination signal sequence recognition protein -related | |  |  |  |  |  | | --- | --- | --- | --- | --- | |  |  |  |  |  | | T20B12.8 | | | | | | |
| Y54G11A.7 | | | WI5\_id:Y54G11A.7 |  | A | B | C | D | P | P' | N |
|  | Cluster:6-1 | |  |  | 2 | 313 | 0 | 4348 | 0.004549907 | 0.004549907 | 1 |
|  |  | RAFL05-08-I15 | At4g24820 / 26S proteasome regulatory subunit (RPN7), putative | |  |  |  |  |  | | --- | --- | --- | --- | --- | |  |  |  |  |  | | F49C12.8 | | | | | | |
|  |  | RAFL07-13-D20 | At4g24820 / 26S proteasome regulatory subunit (RPN7), putative | |  |  |  |  |  | | --- | --- | --- | --- | --- | |  |  |  |  |  | | F49C12.8 | | | | | | |
| T09A5.10 | | | WI5\_id:T09A5.10 |  | A | B | C | D | P | P' | N |
|  | Cluster:2-0 | |  |  | 2 | 148 | 3 | 4510 | 0.009643516 | 0.028930547 | 3 |
|  |  | RAFL06-10-L13 | At3g09630 / 60S ribosomal protein L4/L1 (RPL4A) | |  |  |  |  |  | | --- | --- | --- | --- | --- | |  |  |  |  |  | | B0041.4 | | | | | | |
|  |  | RAFL04-20-D09 | At5g42020 / luminal binding protein 2 precursor (BiP-2) (AtBP2) | |  |  |  |  |  | | --- | --- | --- | --- | --- | |  |  |  |  |  | | F43E2.8  heat shock protein | | | | | | |
|  | Cluster:2-1 | |  |  | 2 | 242 | 3 | 4416 | 0.024550742 | 0.07365222 | 3 |
|  |  | RAFL06-08-N05 | At5g28540 / luminal binding protein 1 precursor (BiP-1) (AtBP1) | |  |  |  |  |  | | --- | --- | --- | --- | --- | |  |  |  |  |  | | F43E2.8  heat shock protein | | | | | | |
|  |  | RAFL07-11-O19 | At5g28540 / luminal binding protein 1 precursor (BiP-1) (AtBP1) | |  |  |  |  |  | | --- | --- | --- | --- | --- | |  |  |  |  |  | | F43E2.8  heat shock protein | | | | | | |
| C17H12.14 | | | WI5\_id:C17H12.14 |  | A | B | C | D | P | P' | N |
|  | Cluster:4-1 | |  |  | 2 | 308 | 3 | 4350 | 0.038523052 | 0.15409221 | 4 |
|  |  | RAFL04-09-A19 | At4g38510 / probable H+-transporting ATPase | |  |  |  |  |  | | --- | --- | --- | --- | --- | |  |  |  |  |  | | F20B6.2 | | | | | | |
|  |  | RAFL07-17-H08 | At4g38510 / probable H+-transporting ATPase | |  |  |  |  |  | | --- | --- | --- | --- | --- | |  |  |  |  |  | | F20B6.2 | | | | | | |
| K08B12.5 | | | WI5\_id:K08B12.5 |  | A | B | C | D | P | P' | N |
|  | Cluster:4-0 | |  |  | 2 | 238 | 1 | 4422 | 0.0076462985 | 0.015292597 | 2 |
|  |  | RAFL06-07-B07 | At5g55190 / GTP-binding protein atran3, putative | |  |  |  |  |  | | --- | --- | --- | --- | --- | |  |  |  |  |  | | K01G5.4 | | | | | | |
|  |  | RAFL05-17-C16 | At5g55190 / GTP-binding protein atran3, putative | |  |  |  |  |  | | --- | --- | --- | --- | --- | |  |  |  |  |  | | K01G5.4 | | | | | | |
| ZK1098.1 | | | WI5\_id:ZK1098.1 |  | A | B | C | D | P | P' | N |
|  | Cluster:1-0 | |  |  | 1 | 148 | 0 | 4514 | 0.031953678 | 0.031953678 | 1 |
|  |  | RAFL04-19-J20 | At3g50270 / hydroxycinnamoyl/benzoyltransferase-related | |  |  |  |  |  | | --- | --- | --- | --- | --- | |  |  |  |  |  | | K04G7.10 | | | | | | |
| C28H8.12 | | | WI5\_id:C28H8.12 |  | A | B | C | D | P | P' | N |
|  | Cluster:4-1 | |  |  | 2 | 308 | 3 | 4350 | 0.038523052 | 0.15409221 | 4 |
|  |  | RAFL04-15-D14 | At5g08680 / H+-transporting ATP synthase beta chain -related | |  |  |  |  |  | | --- | --- | --- | --- | --- | |  |  |  |  |  | | C34E10.6 | | | | | | |
|  |  | RAFL09-17-J21 | At5g08690 / H+-transporting ATP synthase beta chain (mitochondrial) -related | |  |  |  |  |  | | --- | --- | --- | --- | --- | |  |  |  |  |  | | C34E10.6 | | | | | | |
|  | Cluster:3-2 | |  |  | 1 | 36 | 4 | 4622 | 0.039065886 | 0.15626355 | 4 |
|  |  | RAFL06-08-F13 | At3g55360 / 3-oxo-5-alpha-steroid 4-dehydrogenase (steroid 5-alpha-reductase) family | |  |  |  |  |  | | --- | --- | --- | --- | --- | |  |  |  |  |  | | C15F1.6 | | | | | | |
| M02A10.3 | | | WI5\_id:M02A10.3 |  | A | B | C | D | P | P' | N |
|  | Cluster:1-2 | |  |  | 2 | 172 | 2 | 4487 | 0.007905076 | 0.015810153 | 2 |
|  |  | RAFL05-04-J09 | At5g45775 / 60S ribosomal protein L11 (RPL11D) | |  |  |  |  |  | | --- | --- | --- | --- | --- | |  |  |  |  |  | | F07D10.1  ribosomal protein | | | | | | |
|  |  | RAFL11-10-E06 | At5g45775 / 60S ribosomal protein L11 (RPL11D) | |  |  |  |  |  | | --- | --- | --- | --- | --- | |  |  |  |  |  | | F07D10.1  ribosomal protein | | | | | | |
|  | Cluster:2-1 | |  |  | 2 | 242 | 2 | 4417 | 0.01525385 | 0.0305077 | 2 |
|  |  | RAFL04-18-N22 | At2g44120 / 60S ribosomal protein L7 (RPL7C) | |  |  |  |  |  | | --- | --- | --- | --- | --- | |  |  |  |  |  | | F53G12.10 | | | | | | |
|  |  | RAFL02-03-G08 | At4g18730 / 60S ribosomal protein L11 (RPL11C) | |  |  |  |  |  | | --- | --- | --- | --- | --- | |  |  |  |  |  | | F07D10.1  ribosomal protein | | | | | | |
| F37C12.9 | | | WI5\_id:F37C12.9 |  | A | B | C | D | P | P' | N |
|  | Cluster:2-1 | |  |  | 4 | 240 | 2 | 4417 | 1.0099356E-4 | 2.0198712E-4 | 2 |
|  |  | RAFL07-13-J18 | At2g37270 / 40S ribosomal protein S5 (RPS5A) | |  |  |  |  |  | | --- | --- | --- | --- | --- | |  |  |  |  |  | | T05E11.1 | | | | | | |
|  |  | RAFL06-08-B09 | At3g11510 / 40S ribosomal protein S14 (RPS14B) | |  |  |  |  |  | | --- | --- | --- | --- | --- | |  |  |  |  |  | | F37C12.9 | | | | | | |
|  |  | RAFL05-16-H14 | At1g04270 / 40S ribosomal protein S15 (RPS15A) | |  |  |  |  |  | | --- | --- | --- | --- | --- | |  |  |  |  |  | | F36A2.6 | | | | | | |
|  |  | RAFL04-19-M20 | At1g58380 / 40S ribosomal protein S2 (RPS2A) | |  |  |  |  |  | | --- | --- | --- | --- | --- | |  |  |  |  |  | | C49H3.11 | | | | | | |
|  | Cluster:3-0 | |  |  | 2 | 231 | 4 | 4426 | 0.032636177 | 0.06527235 | 2 |
|  |  | RAFL08-09-E20 | At2g41840 / 40S ribosomal protein S2 (RPS2C) | |  |  |  |  |  | | --- | --- | --- | --- | --- | |  |  |  |  |  | | C49H3.11 | | | | | | |
|  |  | RAFL06-07-B02 | At3g11940 / 40S ribosomal protein S5 (RPS5B) | |  |  |  |  |  | | --- | --- | --- | --- | --- | |  |  |  |  |  | | T05E11.1 | | | | | | |
| C07A12.3 | | | WI5\_id:C07A12.3 |  | A | B | C | D | P | P' | N |
|  | Cluster:0-2 | |  |  | 1 | 78 | 0 | 4584 | 0.016941883 | 0.016941883 | 1 |
|  |  | RAFL04-17-G02 | At1g56340 / calreticulin 1 (CRT1) | |  |  |  |  |  | | --- | --- | --- | --- | --- | |  |  |  |  |  | | Y38A10A.5 | | | | | | |
| C08F8.8 | | | WI5\_id:C08F8.8 |  | A | B | C | D | P | P' | N |
|  | Cluster:5-0 | |  |  | 4 | 73 | 1 | 4585 | 3.3961462E-7 | 6.7922923E-7 | 2 |
|  |  | RAFL04-15-M13 | At5g56030 / heat shock protein 81-2 (HSP81-2) | |  |  |  |  |  | | --- | --- | --- | --- | --- | |  |  |  |  |  | | C47E8.5 | | | | | | |
|  |  | RAFL07-13-H08 | At5g56010 / heat shock protein, putative | |  |  |  |  |  | | --- | --- | --- | --- | --- | |  |  |  |  |  | | C47E8.5 | | | | | | |
|  |  | RAFL09-06-O18 | At5g56030 / heat shock protein 81-2 (HSP81-2) | |  |  |  |  |  | | --- | --- | --- | --- | --- | |  |  |  |  |  | | C47E8.5 | | | | | | |
|  |  | RAFL05-16-L15 | At5g56010 / heat shock protein, putative | |  |  |  |  |  | | --- | --- | --- | --- | --- | |  |  |  |  |  | | C47E8.5 | | | | | | |
| T01C3.6 | | | WI5\_id:T01C3.6 |  | A | B | C | D | P | P' | N |
|  | Cluster:2-1 | |  |  | 2 | 242 | 2 | 4417 | 0.01525385 | 0.045761548 | 3 |
|  |  | RAFL05-16-H14 | At1g04270 / 40S ribosomal protein S15 (RPS15A) | |  |  |  |  |  | | --- | --- | --- | --- | --- | |  |  |  |  |  | | F36A2.6 | | | | | | |
|  |  | RAFL04-19-M20 | At1g58380 / 40S ribosomal protein S2 (RPS2A) | |  |  |  |  |  | | --- | --- | --- | --- | --- | |  |  |  |  |  | | C49H3.11 | | | | | | |
| T06E6.10 | | | WI5\_id:T06E6.10 |  | A | B | C | D | P | P' | N |
|  | Cluster:3-0 | |  |  | 1 | 232 | 0 | 4430 | 0.049967833 | 0.049967833 | 1 |
|  |  | RAFL08-13-M06 | At3g11250 / 60S acidic ribosomal protein P0 (RPP0C) | |  |  |  |  |  | | --- | --- | --- | --- | --- | |  |  |  |  |  | | F25H2.10 | | | | | | |
| C33H5.12 | | | WI5\_id:C33H5.12 |  | A | B | C | D | P | P' | N |
|  | Cluster:1-0 | |  |  | 1 | 148 | 0 | 4514 | 0.031953678 | 0.031953678 | 1 |
|  |  | RAFL04-19-J20 | At3g50270 / hydroxycinnamoyl/benzoyltransferase-related | |  |  |  |  |  | | --- | --- | --- | --- | --- | |  |  |  |  |  | | K04G7.10 | | | | | | |
| ZK1010.1 | | | WI5\_id:ZK1010.1 |  | A | B | C | D | P | P' | N |
|  | Cluster:4-2 | |  |  | 1 | 134 | 0 | 4528 | 0.028951319 | 0.028951319 | 1 |
|  |  | RAFL05-05-K13 | At3g52590 / ubiquitin extension protein 1 (UBQ1)/60S ribosomal protein L40 (RPL40B) | |  |  |  |  |  | | --- | --- | --- | --- | --- | |  |  |  |  |  | | ZK1010.1 | | | | | | |
| T19A5.2 | | | WI5\_id:T19A5.2 |  | A | B | C | D | P | P' | N |
|  | Cluster:5-0 | |  |  | 4 | 73 | 1 | 4585 | 3.3961462E-7 | 6.7922923E-7 | 2 |
|  |  | RAFL04-15-M13 | At5g56030 / heat shock protein 81-2 (HSP81-2) | |  |  |  |  |  | | --- | --- | --- | --- | --- | |  |  |  |  |  | | C47E8.5 | | | | | | |
|  |  | RAFL07-13-H08 | At5g56010 / heat shock protein, putative | |  |  |  |  |  | | --- | --- | --- | --- | --- | |  |  |  |  |  | | C47E8.5 | | | | | | |
|  |  | RAFL09-06-O18 | At5g56030 / heat shock protein 81-2 (HSP81-2) | |  |  |  |  |  | | --- | --- | --- | --- | --- | |  |  |  |  |  | | C47E8.5 | | | | | | |
|  |  | RAFL05-16-L15 | At5g56010 / heat shock protein, putative | |  |  |  |  |  | | --- | --- | --- | --- | --- | |  |  |  |  |  | | C47E8.5 | | | | | | |
| Y48G1A.5 | | | WI5\_id:Y48G1A.5 |  | A | B | C | D | P | P' | N |
|  | Cluster:4-0 | |  |  | 2 | 238 | 1 | 4422 | 0.0076462985 | 0.015292597 | 2 |
|  |  | RAFL06-07-B07 | At5g55190 / GTP-binding protein atran3, putative | |  |  |  |  |  | | --- | --- | --- | --- | --- | |  |  |  |  |  | | K01G5.4 | | | | | | |
|  |  | RAFL05-17-C16 | At5g55190 / GTP-binding protein atran3, putative | |  |  |  |  |  | | --- | --- | --- | --- | --- | |  |  |  |  |  | | K01G5.4 | | | | | | |
| T24B8.1 | | | WI5\_id:T24B8.1 |  | A | B | C | D | P | P' | N |
|  | Cluster:2-1 | |  |  | 7 | 237 | 10 | 4409 | 1.22133315E-5 | 6.106666E-5 | 5 |
|  |  | RAFL02-10-A09 | At1g33140 / 60S ribosomal protein L9 (RPL90A/C) | |  |  |  |  |  | | --- | --- | --- | --- | --- | |  |  |  |  |  | | R13A5.8 | | | | | | |
|  |  | RAFL07-15-M07 | At1g04480 / 60S ribosomal protein L23 (RPL23A) | |  |  |  |  |  | | --- | --- | --- | --- | --- | |  |  |  |  |  | | B0336.10 | | | | | | |
|  |  | RAFL05-17-L17 | At3g55280 / 60S ribosomal protein L23A (RPL23aB) | |  |  |  |  |  | | --- | --- | --- | --- | --- | |  |  |  |  |  | | F52B5.6 | | | | | | |
|  |  | RAFL11-12-H04 | At3g25520 / 60S ribosomal protein L5 (RPL5A) | |  |  |  |  |  | | --- | --- | --- | --- | --- | |  |  |  |  |  | | F54C9.5 | | | | | | |
|  |  | RAFL05-18-P15 | At1g04480 / 60S ribosomal protein L23 (RPL23A) | |  |  |  |  |  | | --- | --- | --- | --- | --- | |  |  |  |  |  | | B0336.10 | | | | | | |
|  |  | RAFL04-18-N22 | At2g44120 / 60S ribosomal protein L7 (RPL7C) | |  |  |  |  |  | | --- | --- | --- | --- | --- | |  |  |  |  |  | | F53G12.10  ribosomal protein | | | | | | |
|  |  | RAFL11-07-B21 | At3g25520 / 60S ribosomal protein L5 (RPL5A) | |  |  |  |  |  | | --- | --- | --- | --- | --- | |  |  |  |  |  | | F54C9.5 | | | | | | |
|  | Cluster:2-0 | |  |  | 4 | 146 | 13 | 4500 | 0.0017651725 | 0.008825863 | 5 |
|  |  | RAFL04-19-O24 | At5g02610 / 60S ribosomal protein L35 (RPL35D) | |  |  |  |  |  | | --- | --- | --- | --- | --- | |  |  |  |  |  | | ZK652.4 | | | | | | |
|  |  | RAFL06-10-L13 | At3g09630 / 60S ribosomal protein L4/L1 (RPL4A) | |  |  |  |  |  | | --- | --- | --- | --- | --- | |  |  |  |  |  | | B0041.4 | | | | | | |
|  |  | RAFL11-12-M17 | At3g25520 / 60S ribosomal protein L5 (RPL5A) | |  |  |  |  |  | | --- | --- | --- | --- | --- | |  |  |  |  |  | | F54C9.5 | | | | | | |
|  |  | RAFL03-06-H07 | At1g33140 / 60S ribosomal protein L9 (RPL90A/C) | |  |  |  |  |  | | --- | --- | --- | --- | --- | |  |  |  |  |  | | R13A5.8 | | | | | | |
|  | Cluster:3-0 | |  |  | 3 | 230 | 14 | 4416 | 0.0498523 | 0.2492615 | 5 |
|  |  | RAFL09-10-P09 | At4g36130 / 60S ribosomal protein L8 (RPL8C) | |  |  |  |  |  | | --- | --- | --- | --- | --- | |  |  |  |  |  | | B0250.1 | | | | | | |
|  |  | RAFL08-10-G08 | At3g09630 / 60S ribosomal protein L4/L1 (RPL4A) | |  |  |  |  |  | | --- | --- | --- | --- | --- | |  |  |  |  |  | | B0041.4 | | | | | | |
|  |  | RAFL09-12-B12 | At4g36130 / 60S ribosomal protein L8 (RPL8C) | |  |  |  |  |  | | --- | --- | --- | --- | --- | |  |  |  |  |  | | B0250.1 | | | | | | |
| C48D5.1 | | | WI5\_id:C48D5.1 |  | A | B | C | D | P | P' | N |
|  | Cluster:7-0 | |  |  | 2 | 245 | 5 | 4411 | 0.049187053 | 0.29512233 | 6 |
|  |  | RAFL05-04-D08 | At1g64520 / 26S proteasome regulatory subunit (RPN12), putative | |  |  |  |  |  | | --- | --- | --- | --- | --- | |  |  |  |  |  | | ZK20.5 | | | | | | |
|  |  | RAFL06-13-G03 | At1g64520 / 26S proteasome regulatory subunit (RPN12), putative | |  |  |  |  |  | | --- | --- | --- | --- | --- | |  |  |  |  |  | | ZK20.5 | | | | | | |
| ZK945.2 | | | WI5\_id:ZK945.2 |  | A | B | C | D | P | P' | N |
|  | Cluster:4-1 | |  |  | 2 | 308 | 2 | 4351 | 0.024166461 | 0.07249938 | 3 |
|  |  | RAFL05-01-C14 | At5g42790 / 20S proteasome alpha subunit F1 (PAF1) | |  |  |  |  |  | | --- | --- | --- | --- | --- | |  |  |  |  |  | | CD4.6 | | | | | | |
|  |  | RAFL05-12-G18 | At4g14800 / 20S proteasome beta subunit D2 (PBD2) | |  |  |  |  |  | | --- | --- | --- | --- | --- | |  |  |  |  |  | | T20F5.2 | | | | | | |
| D1037.4 | | | WI5\_id:D1037.4 |  | A | B | C | D | P | P' | N |
|  | Cluster:6-1 | |  |  | 2 | 313 | 1 | 4347 | 0.013038642 | 0.026077284 | 2 |
|  |  | RAFL09-13-A13 | At2g44100 / GDP dissociation inhibitor | |  |  |  |  |  | | --- | --- | --- | --- | --- | |  |  |  |  |  | | Y57G11C.10 | | | | | | |
|  |  | RAFL05-11-L05 | At2g44100 / GDP dissociation inhibitor | |  |  |  |  |  | | --- | --- | --- | --- | --- | |  |  |  |  |  | | Y57G11C.10 | | | | | | |
| R13A5.8 | | | WI5\_id:R13A5.8 |  | A | B | C | D | P | P' | N |
|  | Cluster:2-1 | |  |  | 4 | 240 | 9 | 4410 | 0.003594625 | 0.02156775 | 6 |
|  |  | RAFL02-10-A09 | At1g33140 / 60S ribosomal protein L9 (RPL90A/C) | |  |  |  |  |  | | --- | --- | --- | --- | --- | |  |  |  |  |  | | R13A5.8 | | | | | | |
|  |  | RAFL05-17-L17 | At3g55280 / 60S ribosomal protein L23A (RPL23aB) | |  |  |  |  |  | | --- | --- | --- | --- | --- | |  |  |  |  |  | | F52B5.6 | | | | | | |
|  |  | RAFL11-12-H04 | At3g25520 / 60S ribosomal protein L5 (RPL5A) | |  |  |  |  |  | | --- | --- | --- | --- | --- | |  |  |  |  |  | | F54C9.5 | | | | | | |
|  |  | RAFL11-07-B21 | At3g25520 / 60S ribosomal protein L5 (RPL5A) | |  |  |  |  |  | | --- | --- | --- | --- | --- | |  |  |  |  |  | | F54C9.5 | | | | | | |
|  | Cluster:2-0 | |  |  | 3 | 147 | 10 | 4503 | 0.0073604016 | 0.04416241 | 6 |
|  |  | RAFL04-19-O24 | At5g02610 / 60S ribosomal protein L35 (RPL35D) | |  |  |  |  |  | | --- | --- | --- | --- | --- | |  |  |  |  |  | | ZK652.4 | | | | | | |
|  |  | RAFL11-12-M17 | At3g25520 / 60S ribosomal protein L5 (RPL5A) | |  |  |  |  |  | | --- | --- | --- | --- | --- | |  |  |  |  |  | | F54C9.5 | | | | | | |
|  |  | RAFL03-06-H07 | At1g33140 / 60S ribosomal protein L9 (RPL90A/C) | |  |  |  |  |  | | --- | --- | --- | --- | --- | |  |  |  |  |  | | R13A5.8 | | | | | | |
|  | Cluster:3-0 | |  |  | 3 | 230 | 10 | 4420 | 0.024273654 | 0.14564192 | 6 |
|  |  | RAFL07-10-D02 | At1g14320 / 60S ribosomal protein L10 (RPL10A)/Wilm's tumor suppressor protein-related | |  |  |  |  |  | | --- | --- | --- | --- | --- | |  |  |  |  |  | | F10B5.1 | | | | | | |
|  |  | RAFL09-10-P09 | At4g36130 / 60S ribosomal protein L8 (RPL8C) | |  |  |  |  |  | | --- | --- | --- | --- | --- | |  |  |  |  |  | | B0250.1 | | | | | | |
|  |  | RAFL09-12-B12 | At4g36130 / 60S ribosomal protein L8 (RPL8C) | |  |  |  |  |  | | --- | --- | --- | --- | --- | |  |  |  |  |  | | B0250.1 | | | | | | |
| K12H4.8 | | | WI5\_id:K12H4.8 |  | A | B | C | D | P | P' | N |
|  | Cluster:8-0 | |  |  | 1 | 108 | 0 | 4554 | 0.02337551 | 0.02337551 | 1 |
|  |  | RAFL05-03-L12 | At1g47128 / cysteine proteinase RD21A | |  |  |  |  |  | | --- | --- | --- | --- | --- | |  |  |  |  |  | | T03E6.7 | | | | | | |
| F10G7.8 | | | WI5\_id:F10G7.8 |  | A | B | C | D | P | P' | N |
|  | Cluster:6-1 | |  |  | 4 | 311 | 14 | 4334 | 0.029376008 | 0.3525121 | 12 |
|  |  | RAFL08-19-N19 | At3g05530 / 26S proteasome AAA-ATPase subunit RPT5a | |  |  |  |  |  | | --- | --- | --- | --- | --- | |  |  |  |  |  | | F56H1.4  ATPase | | | | | | |
|  |  | RAFL05-08-I15 | At4g24820 / 26S proteasome regulatory subunit (RPN7), putative | |  |  |  |  |  | | --- | --- | --- | --- | --- | |  |  |  |  |  | | F49C12.8 | | | | | | |
|  |  | RAFL07-13-D20 | At4g24820 / 26S proteasome regulatory subunit (RPN7), putative | |  |  |  |  |  | | --- | --- | --- | --- | --- | |  |  |  |  |  | | F49C12.8 | | | | | | |
|  |  | RAFL11-04-H03 | At1g20200 / 26S proteasome regulatory subunit S3 (RPN3), putative | |  |  |  |  |  | | --- | --- | --- | --- | --- | |  |  |  |  |  | | C30C11.2 | | | | | | |
| C23G10.8 | | | WI5\_id:C23G10.8 |  | A | B | C | D | P | P' | N |
|  | Cluster:5-0 | |  |  | 1 | 76 | 1 | 4585 | 0.032756753 | 0.06551351 | 2 |
|  |  | RAFL06-07-P18 | At3g60820 / 20S proteasome beta subunit F1 (PBF1) | |  |  |  |  |  | | --- | --- | --- | --- | --- | |  |  |  |  |  | | C02F5.9 | | | | | | |
| C15F1.6 | | | WI5\_id:C15F1.6 |  | A | B | C | D | P | P' | N |
|  | Cluster:3-2 | |  |  | 1 | 36 | 0 | 4626 | 0.007934806 | 0.007934806 | 1 |
|  |  | RAFL06-08-F13 | At3g55360 / 3-oxo-5-alpha-steroid 4-dehydrogenase (steroid 5-alpha-reductase) family | |  |  |  |  |  | | --- | --- | --- | --- | --- | |  |  |  |  |  | | C15F1.6 | | | | | | |
| T11B7.4 | | | WI5\_id:T11B7.4 |  | A | B | C | D | P | P' | N |
|  | Cluster:5-0 | |  |  | 4 | 73 | 7 | 4579 | 2.0782694E-5 | 1.4547886E-4 | 7 |
|  |  | RAFL04-15-M13 | At5g56030 / heat shock protein 81-2 (HSP81-2) | |  |  |  |  |  | | --- | --- | --- | --- | --- | |  |  |  |  |  | | C47E8.5 | | | | | | |
|  |  | RAFL07-13-H08 | At5g56010 / heat shock protein, putative | |  |  |  |  |  | | --- | --- | --- | --- | --- | |  |  |  |  |  | | C47E8.5 | | | | | | |
|  |  | RAFL09-06-O18 | At5g56030 / heat shock protein 81-2 (HSP81-2) | |  |  |  |  |  | | --- | --- | --- | --- | --- | |  |  |  |  |  | | C47E8.5 | | | | | | |
|  |  | RAFL05-16-L15 | At5g56010 / heat shock protein, putative | |  |  |  |  |  | | --- | --- | --- | --- | --- | |  |  |  |  |  | | C47E8.5 | | | | | | |
| R06F6.8 | | | WI5\_id:R06F6.8 |  | A | B | C | D | P | P' | N |
|  | Cluster:1-2 | |  |  | 1 | 173 | 0 | 4489 | 0.037315033 | 0.037315033 | 1 |
|  |  | RAFL06-11-J01 | At1g09690 / 60S ribosomal protein L21 (RPL21C) | |  |  |  |  |  | | --- | --- | --- | --- | --- | |  |  |  |  |  | | C14B9.7  Ribosomal protein L21 | | | | | | |
| C50C3.6 | | | WI5\_id:C50C3.6 |  | A | B | C | D | P | P' | N |
|  | Cluster:1-0 | |  |  | 1 | 148 | 0 | 4514 | 0.031953678 | 0.031953678 | 1 |
|  |  | RAFL04-19-J20 | At3g50270 / hydroxycinnamoyl/benzoyltransferase-related | |  |  |  |  |  | | --- | --- | --- | --- | --- | |  |  |  |  |  | | K04G7.10 | | | | | | |
| D1005.1 | | | WI5\_id:D1005.1 |  | A | B | C | D | P | P' | N |
|  | Cluster:5-0 | |  |  | 1 | 76 | 0 | 4586 | 0.016512975 | 0.016512975 | 1 |
|  |  | RAFL06-07-P18 | At3g60820 / 20S proteasome beta subunit F1 (PBF1) | |  |  |  |  |  | | --- | --- | --- | --- | --- | |  |  |  |  |  | | C02F5.9 | | | | | | |
| Y59H11AM.3 | | | WI5\_id:Y59H11AM.3 |  | A | B | C | D | P | P' | N |
|  | Cluster:3-0 | |  |  | 3 | 230 | 3 | 4427 | 0.0022016333 | 0.0066048997 | 3 |
|  |  | RAFL04-13-C01 | At1g56070 / elongation factor -related | |  |  |  |  |  | | --- | --- | --- | --- | --- | |  |  |  |  |  | | F25H5.4 | | | | | | |
|  |  | RAFL08-12-J19 | At1g56070 / elongation factor -related | |  |  |  |  |  | | --- | --- | --- | --- | --- | |  |  |  |  |  | | F25H5.4 | | | | | | |
|  |  | RAFL07-10-D07 | At1g56070 / elongation factor -related | |  |  |  |  |  | | --- | --- | --- | --- | --- | |  |  |  |  |  | | F25H5.4 | | | | | | |
|  | Cluster:4-0 | |  |  | 2 | 238 | 4 | 4419 | 0.034489654 | 0.10346896 | 3 |
|  |  | RAFL07-11-O12 | At1g56070 / elongation factor -related | |  |  |  |  |  | | --- | --- | --- | --- | --- | |  |  |  |  |  | | F25H5.4 | | | | | | |
|  |  | RAFL04-17-N12 | At1g56070 / elongation factor -related | |  |  |  |  |  | | --- | --- | --- | --- | --- | |  |  |  |  |  | | F25H5.4 | | | | | | |
| C27A2.5 | | | WI5\_id:C27A2.5 |  | A | B | C | D | P | P' | N |
|  | Cluster:3-0 | |  |  | 1 | 232 | 0 | 4430 | 0.049967833 | 0.049967833 | 1 |
|  |  | RAFL04-14-I15 | At3g02080 / 40S ribosomal protein S19 (RPS19A) | |  |  |  |  |  | | --- | --- | --- | --- | --- | |  |  |  |  |  | | T05F1.3 | | | | | | |
| F47G6.1 | | | WI5\_id:F47G6.1 |  | A | B | C | D | P | P' | N |
|  | Cluster:4-0 | |  |  | 2 | 238 | 0 | 4423 | 0.0026385873 | 0.0026385873 | 1 |
|  |  | RAFL08-19-E03 | At1g77510 / protein disulfide isomerase, putative | |  |  |  |  |  | | --- | --- | --- | --- | --- | |  |  |  |  |  | | H06O01.1 | | | | | | |
|  |  | RAFL09-06-G15 | At1g45000 / 26S proteasome regulatory particle triple-A ATPase subunit4 -related | |  |  |  |  |  | | --- | --- | --- | --- | --- | |  |  |  |  |  | | F23F1.8 | | | | | | |
| F25H2.10 | | | WI5\_id:F25H2.10 |  | A | B | C | D | P | P' | N |
|  | Cluster:3-0 | |  |  | 4 | 229 | 8 | 4422 | 0.002188199 | 0.013129194 | 6 |
|  |  | RAFL07-10-D02 | At1g14320 / 60S ribosomal protein L10 (RPL10A)/Wilm's tumor suppressor protein-related | |  |  |  |  |  | | --- | --- | --- | --- | --- | |  |  |  |  |  | | F10B5.1 | | | | | | |
|  |  | RAFL09-10-P09 | At4g36130 / 60S ribosomal protein L8 (RPL8C) | |  |  |  |  |  | | --- | --- | --- | --- | --- | |  |  |  |  |  | | B0250.1 | | | | | | |
|  |  | RAFL08-13-M06 | At3g11250 / 60S acidic ribosomal protein P0 (RPP0C) | |  |  |  |  |  | | --- | --- | --- | --- | --- | |  |  |  |  |  | | F25H2.10 | | | | | | |
|  |  | RAFL09-12-B12 | At4g36130 / 60S ribosomal protein L8 (RPL8C) | |  |  |  |  |  | | --- | --- | --- | --- | --- | |  |  |  |  |  | | B0250.1 | | | | | | |
|  | Cluster:2-1 | |  |  | 3 | 241 | 9 | 4410 | 0.021903817 | 0.13142289 | 6 |
|  |  | RAFL05-17-L17 | At3g55280 / 60S ribosomal protein L23A (RPL23aB) | |  |  |  |  |  | | --- | --- | --- | --- | --- | |  |  |  |  |  | | F52B5.6 | | | | | | |
|  |  | RAFL11-12-H04 | At3g25520 / 60S ribosomal protein L5 (RPL5A) | |  |  |  |  |  | | --- | --- | --- | --- | --- | |  |  |  |  |  | | F54C9.5 | | | | | | |
|  |  | RAFL11-07-B21 | At3g25520 / 60S ribosomal protein L5 (RPL5A) | |  |  |  |  |  | | --- | --- | --- | --- | --- | |  |  |  |  |  | | F54C9.5 | | | | | | |
| T26A5.9 | | | WI5\_id:T26A5.9 |  | A | B | C | D | P | P' | N |
|  | Cluster:2-1 | |  |  | 2 | 242 | 2 | 4417 | 0.01525385 | 0.045761548 | 3 |
|  |  | RAFL07-15-K08 | At4g34670 / 40S ribosomal protein S3A (RPS3aB) | |  |  |  |  |  | | --- | --- | --- | --- | --- | |  |  |  |  |  | | F56F3.5 | | | | | | |
|  |  | RAFL06-08-B09 | At3g11510 / 40S ribosomal protein S14 (RPS14B) | |  |  |  |  |  | | --- | --- | --- | --- | --- | |  |  |  |  |  | | F37C12.9 | | | | | | |
| C36B1.4 | | | WI5\_id:C36B1.4 |  | A | B | C | D | P | P' | N |
|  | Cluster:4-1 | |  |  | 3 | 307 | 3 | 4350 | 0.005004816 | 0.020019265 | 4 |
|  |  | RAFL04-12-B09 | At3g14290 / 20S proteasome alpha subunit E2 (PAE2) | |  |  |  |  |  | | --- | --- | --- | --- | --- | |  |  |  |  |  | | F25H2.9 | | | | | | |
|  |  | RAFL05-04-L16 | At1g79210 / 20S proteasome alpha subunit B, putative | |  |  |  |  |  | | --- | --- | --- | --- | --- | |  |  |  |  |  | | D1054.2 | | | | | | |
|  |  | RAFL05-12-G18 | At4g14800 / 20S proteasome beta subunit D2 (PBD2) | |  |  |  |  |  | | --- | --- | --- | --- | --- | |  |  |  |  |  | | T20F5.2 | | | | | | |
| F59A6.1 | | | WI5\_id:F59A6.1 |  | A | B | C | D | P | P' | N |
|  | Cluster:2-0 | |  |  | 1 | 149 | 0 | 4513 | 0.03216813 | 0.03216813 | 1 |
|  |  | RAFL03-08-O03 | At1g07940 / elongation factor 1-alpha (EF-1-alpha) | |  |  |  |  |  | | --- | --- | --- | --- | --- | |  |  |  |  |  | | F31E3.5 | | | | | | |
| F55H2.2 | | | WI5\_id:F55H2.2 |  | A | B | C | D | P | P' | N |
|  | Cluster:4-1 | |  |  | 2 | 308 | 3 | 4350 | 0.038523052 | 0.15409221 | 4 |
|  |  | RAFL04-09-A19 | At4g38510 / probable H+-transporting ATPase | |  |  |  |  |  | | --- | --- | --- | --- | --- | |  |  |  |  |  | | F20B6.2 | | | | | | |
|  |  | RAFL07-17-H08 | At4g38510 / probable H+-transporting ATPase | |  |  |  |  |  | | --- | --- | --- | --- | --- | |  |  |  |  |  | | F20B6.2 | | | | | | |
| B0412.4 | | | WI5\_id:B0412.4 |  | A | B | C | D | P | P' | N |
|  | Cluster:3-0 | |  |  | 3 | 230 | 3 | 4427 | 0.0022016333 | 0.0044032666 | 2 |
|  |  | RAFL02-10-H10 | At3g43980 / 40S ribosomal protein S29 (RPS29A) | |  |  |  |  |  | | --- | --- | --- | --- | --- | |  |  |  |  |  | | B0412.4  40S ribosomal protein S29 | | | | | | |
|  |  | RAFL08-09-E20 | At2g41840 / 40S ribosomal protein S2 (RPS2C) | |  |  |  |  |  | | --- | --- | --- | --- | --- | |  |  |  |  |  | | C49H3.11 | | | | | | |
|  |  | RAFL06-07-B02 | At3g11940 / 40S ribosomal protein S5 (RPS5B) | |  |  |  |  |  | | --- | --- | --- | --- | --- | |  |  |  |  |  | | T05E11.1  40S ribosomal protein S5 | | | | | | |
|  | Cluster:2-1 | |  |  | 3 | 241 | 3 | 4416 | 0.0025160299 | 0.0050320597 | 2 |
|  |  | RAFL07-13-J18 | At2g37270 / 40S ribosomal protein S5 (RPS5A) | |  |  |  |  |  | | --- | --- | --- | --- | --- | |  |  |  |  |  | | T05E11.1  40S ribosomal protein S5 | | | | | | |
|  |  | RAFL05-16-H14 | At1g04270 / 40S ribosomal protein S15 (RPS15A) | |  |  |  |  |  | | --- | --- | --- | --- | --- | |  |  |  |  |  | | F36A2.6  40S ribosomal protein S15 | | | | | | |
|  |  | RAFL04-19-M20 | At1g58380 / 40S ribosomal protein S2 (RPS2A) | |  |  |  |  |  | | --- | --- | --- | --- | --- | |  |  |  |  |  | | C49H3.11 | | | | | | |
| C37H5.8 | | | WI5\_id:C37H5.8 |  | A | B | C | D | P | P' | N |
|  | Cluster:4-2 | |  |  | 1 | 134 | 0 | 4528 | 0.028951319 | 0.028951319 | 1 |
|  |  | RAFL04-17-F17 | At4g37910 / heat shock protein mtHsc70-1 | |  |  |  |  |  | | --- | --- | --- | --- | --- | |  |  |  |  |  | | C37H5.8 | | | | | | |
| F25H5.4 | | | WI5\_id:F25H5.4 |  | A | B | C | D | P | P' | N |
|  | Cluster:3-0 | |  |  | 3 | 230 | 3 | 4427 | 0.0022016333 | 0.0066048997 | 3 |
|  |  | RAFL04-13-C01 | At1g56070 / elongation factor -related | |  |  |  |  |  | | --- | --- | --- | --- | --- | |  |  |  |  |  | | F25H5.4 | | | | | | |
|  |  | RAFL08-12-J19 | At1g56070 / elongation factor -related | |  |  |  |  |  | | --- | --- | --- | --- | --- | |  |  |  |  |  | | F25H5.4 | | | | | | |
|  |  | RAFL07-10-D07 | At1g56070 / elongation factor -related | |  |  |  |  |  | | --- | --- | --- | --- | --- | |  |  |  |  |  | | F25H5.4 | | | | | | |
|  | Cluster:4-0 | |  |  | 2 | 238 | 4 | 4419 | 0.034489654 | 0.10346896 | 3 |
|  |  | RAFL07-11-O12 | At1g56070 / elongation factor -related | |  |  |  |  |  | | --- | --- | --- | --- | --- | |  |  |  |  |  | | F25H5.4 | | | | | | |
|  |  | RAFL04-17-N12 | At1g56070 / elongation factor -related | |  |  |  |  |  | | --- | --- | --- | --- | --- | |  |  |  |  |  | | F25H5.4 | | | | | | |
| T06E6.2 | | | WI5\_id:T06E6.2 |  | A | B | C | D | P | P' | N |
|  | Cluster:4-2 | |  |  | 1 | 134 | 0 | 4528 | 0.028951319 | 0.028951319 | 1 |
|  |  | RAFL05-18-G17 | At3g48750 / cell division control protein 2 homolog A (CDC2A) | |  |  |  |  |  | | --- | --- | --- | --- | --- | |  |  |  |  |  | | T05G5.3 | | | | | | |
| T10E10.4 | | | WI5\_id:T10E10.4 |  | A | B | C | D | P | P' | N |
|  | Cluster:5-0 | |  |  | 1 | 76 | 0 | 4586 | 0.016512975 | 0.016512975 | 1 |
|  |  | RAFL04-09-J19 | At1g56450 / 20S proteasome beta subunit G1 (PBG1) | |  |  |  |  |  | | --- | --- | --- | --- | --- | |  |  |  |  |  | | F39H11.5 | | | | | | |
| F01E11.1 | | | WI5\_id:F01E11.1 |  | A | B | C | D | P | P' | N |
|  | Cluster:1-2 | |  |  | 2 | 172 | 1 | 4488 | 0.0040519224 | 0.008103845 | 2 |
|  |  | RAFL05-04-J09 | At5g45775 / 60S ribosomal protein L11 (RPL11D) | |  |  |  |  |  | | --- | --- | --- | --- | --- | |  |  |  |  |  | | F07D10.1 | | | | | | |
|  |  | RAFL11-10-E06 | At5g45775 / 60S ribosomal protein L11 (RPL11D) | |  |  |  |  |  | | --- | --- | --- | --- | --- | |  |  |  |  |  | | F07D10.1 | | | | | | |
| Y75B8A.2 | | | WI5\_id:Y75B8A.2 |  | A | B | C | D | P | P' | N |
|  | Cluster:2-0 | |  |  | 1 | 149 | 0 | 4513 | 0.03216813 | 0.03216813 | 1 |
|  |  | RAFL05-01-F21 | At3g05590 / 60S ribosomal protein L18 (RPL18B) | |  |  |  |  |  | | --- | --- | --- | --- | --- | |  |  |  |  |  | | Y45F10D.12 | | | | | | |
| C35B8.2 | | | WI5\_id:C35B8.2 |  | A | B | C | D | P | P' | N |
|  | Cluster:2-0 | |  |  | 1 | 149 | 0 | 4513 | 0.03216813 | 0.03216813 | 1 |
|  |  | RAFL03-08-O03 | At1g07940 / elongation factor 1-alpha (EF-1-alpha) | |  |  |  |  |  | | --- | --- | --- | --- | --- | |  |  |  |  |  | | F31E3.5 | | | | | | |
| H28O16.1 | | | WI5\_id:H28O16.1 |  | A | B | C | D | P | P' | N |
|  | Cluster:4-1 | |  |  | 3 | 307 | 2 | 4351 | 0.0026315711 | 0.007894713 | 3 |
|  |  | RAFL04-12-B09 | At3g14290 / 20S proteasome alpha subunit E2 (PAE2) | |  |  |  |  |  | | --- | --- | --- | --- | --- | |  |  |  |  |  | | F25H2.9 | | | | | | |
|  |  | RAFL04-15-D14 | At5g08680 / H+-transporting ATP synthase beta chain -related | |  |  |  |  |  | | --- | --- | --- | --- | --- | |  |  |  |  |  | | C34E10.6 | | | | | | |
|  |  | RAFL09-17-J21 | At5g08690 / H+-transporting ATP synthase beta chain (mitochondrial) -related | |  |  |  |  |  | | --- | --- | --- | --- | --- | |  |  |  |  |  | | C34E10.6 | | | | | | |
| C04F6.1 | | | WI5\_id:C04F6.1 |  | A | B | C | D | P | P' | N |
|  | Cluster:4-0 | |  |  | 2 | 238 | 1 | 4422 | 0.0076462985 | 0.015292597 | 2 |
|  |  | RAFL06-07-B07 | At5g55190 / GTP-binding protein atran3, putative | |  |  |  |  |  | | --- | --- | --- | --- | --- | |  |  |  |  |  | | K01G5.4 | | | | | | |
|  |  | RAFL05-17-C16 | At5g55190 / GTP-binding protein atran3, putative | |  |  |  |  |  | | --- | --- | --- | --- | --- | |  |  |  |  |  | | K01G5.4 | | | | | | |
| K07A1.1 | | | WI5\_id:K07A1.1 |  | A | B | C | D | P | P' | N |
|  | Cluster:8-0 | |  |  | 1 | 108 | 1 | 4553 | 0.0462095 | 0.092419 | 2 |
|  |  | RAFL09-13-J20 | At1g69410 / Eukaryotic initiation factor 5A -related | |  |  |  |  |  | | --- | --- | --- | --- | --- | |  |  |  |  |  | | T05G5.10 | | | | | | |
| Y38A10A.5 | | | WI5\_id:Y38A10A.5 |  | A | B | C | D | P | P' | N |
|  | Cluster:4-0 | |  |  | 2 | 238 | 3 | 4420 | 0.023792636 | 0.09517054 | 4 |
|  |  | RAFL06-07-B07 | At5g55190 / GTP-binding protein atran3, putative | |  |  |  |  |  | | --- | --- | --- | --- | --- | |  |  |  |  |  | | K01G5.4 | | | | | | |
|  |  | RAFL05-17-C16 | At5g55190 / GTP-binding protein atran3, putative | |  |  |  |  |  | | --- | --- | --- | --- | --- | |  |  |  |  |  | | K01G5.4 | | | | | | |
| F54C9.5 | | | WI5\_id:F54C9.5 |  | A | B | C | D | P | P' | N |
|  | Cluster:2-1 | |  |  | 7 | 237 | 14 | 4405 | 6.093423E-5 | 4.265396E-4 | 7 |
|  |  | RAFL02-10-A09 | At1g33140 / 60S ribosomal protein L9 (RPL90A/C) | |  |  |  |  |  | | --- | --- | --- | --- | --- | |  |  |  |  |  | | R13A5.8 | | | | | | |
|  |  | RAFL07-15-M07 | At1g04480 / 60S ribosomal protein L23 (RPL23A) | |  |  |  |  |  | | --- | --- | --- | --- | --- | |  |  |  |  |  | | B0336.10 | | | | | | |
|  |  | RAFL05-17-L17 | At3g55280 / 60S ribosomal protein L23A (RPL23aB) | |  |  |  |  |  | | --- | --- | --- | --- | --- | |  |  |  |  |  | | F52B5.6 | | | | | | |
|  |  | RAFL11-12-H04 | At3g25520 / 60S ribosomal protein L5 (RPL5A) | |  |  |  |  |  | | --- | --- | --- | --- | --- | |  |  |  |  |  | | F54C9.5 | | | | | | |
|  |  | RAFL05-18-P15 | At1g04480 / 60S ribosomal protein L23 (RPL23A) | |  |  |  |  |  | | --- | --- | --- | --- | --- | |  |  |  |  |  | | B0336.10 | | | | | | |
|  |  | RAFL04-18-N22 | At2g44120 / 60S ribosomal protein L7 (RPL7C) | |  |  |  |  |  | | --- | --- | --- | --- | --- | |  |  |  |  |  | | F53G12.10 | | | | | | |
|  |  | RAFL11-07-B21 | At3g25520 / 60S ribosomal protein L5 (RPL5A) | |  |  |  |  |  | | --- | --- | --- | --- | --- | |  |  |  |  |  | | F54C9.5 | | | | | | |
|  | Cluster:3-0 | |  |  | 5 | 228 | 16 | 4414 | 0.0031419091 | 0.021993365 | 7 |
|  |  | RAFL07-10-D02 | At1g14320 / 60S ribosomal protein L10 (RPL10A)/Wilm's tumor suppressor protein-related | |  |  |  |  |  | | --- | --- | --- | --- | --- | |  |  |  |  |  | | F10B5.1 | | | | | | |
|  |  | RAFL09-10-P09 | At4g36130 / 60S ribosomal protein L8 (RPL8C) | |  |  |  |  |  | | --- | --- | --- | --- | --- | |  |  |  |  |  | | B0250.1 | | | | | | |
|  |  | RAFL08-10-G08 | At3g09630 / 60S ribosomal protein L4/L1 (RPL4A) | |  |  |  |  |  | | --- | --- | --- | --- | --- | |  |  |  |  |  | | B0041.4 | | | | | | |
|  |  | RAFL08-13-M06 | At3g11250 / 60S acidic ribosomal protein P0 (RPP0C) | |  |  |  |  |  | | --- | --- | --- | --- | --- | |  |  |  |  |  | | F25H2.10 | | | | | | |
|  |  | RAFL09-12-B12 | At4g36130 / 60S ribosomal protein L8 (RPL8C) | |  |  |  |  |  | | --- | --- | --- | --- | --- | |  |  |  |  |  | | B0250.1 | | | | | | |
|  | Cluster:2-0 | |  |  | 4 | 146 | 17 | 4496 | 0.0040158513 | 0.028110959 | 7 |
|  |  | RAFL04-19-O24 | At5g02610 / 60S ribosomal protein L35 (RPL35D) | |  |  |  |  |  | | --- | --- | --- | --- | --- | |  |  |  |  |  | | ZK652.4 | | | | | | |
|  |  | RAFL06-10-L13 | At3g09630 / 60S ribosomal protein L4/L1 (RPL4A) | |  |  |  |  |  | | --- | --- | --- | --- | --- | |  |  |  |  |  | | B0041.4 | | | | | | |
|  |  | RAFL11-12-M17 | At3g25520 / 60S ribosomal protein L5 (RPL5A) | |  |  |  |  |  | | --- | --- | --- | --- | --- | |  |  |  |  |  | | F54C9.5 | | | | | | |
|  |  | RAFL03-06-H07 | At1g33140 / 60S ribosomal protein L9 (RPL90A/C) | |  |  |  |  |  | | --- | --- | --- | --- | --- | |  |  |  |  |  | | R13A5.8 | | | | | | |
| C04F12.9 | | | WI5\_id:C04F12.9 |  | A | B | C | D | P | P' | N |
|  | Cluster:2-0 | |  |  | 1 | 149 | 0 | 4513 | 0.03216813 | 0.03216813 | 1 |
|  |  | RAFL05-02-F20 | At5g65360 / histone H3 | |  |  |  |  |  | | --- | --- | --- | --- | --- | |  |  |  |  |  | | Y49E10.6  Core histone H2A/H2B/H3/H4 | | | | | | |
| F26E4.8 | | | WI5\_id:F26E4.8 |  | A | B | C | D | P | P' | N |
|  | Cluster:4-0 | |  |  | 2 | 238 | 2 | 4421 | 0.0147742275 | 0.044322684 | 3 |
|  |  | RAFL06-07-B07 | At5g55190 / GTP-binding protein atran3, putative | |  |  |  |  |  | | --- | --- | --- | --- | --- | |  |  |  |  |  | | K01G5.4 | | | | | | |
|  |  | RAFL05-17-C16 | At5g55190 / GTP-binding protein atran3, putative | |  |  |  |  |  | | --- | --- | --- | --- | --- | |  |  |  |  |  | | K01G5.4 | | | | | | |
| C02F5.9 | | | WI5\_id:C02F5.9 |  | A | B | C | D | P | P' | N |
|  | Cluster:5-0 | |  |  | 1 | 76 | 1 | 4585 | 0.032756753 | 0.06551351 | 2 |
|  |  | RAFL06-07-P18 | At3g60820 / 20S proteasome beta subunit F1 (PBF1) | |  |  |  |  |  | | --- | --- | --- | --- | --- | |  |  |  |  |  | | C02F5.9 | | | | | | |
| F28C6.7 | | | WI5\_id:F28C6.7 |  | A | B | C | D | P | P' | N |
|  | Cluster:2-1 | |  |  | 6 | 238 | 13 | 4406 | 2.9421985E-4 | 0.001765319 | 6 |
|  |  | RAFL02-10-A09 | At1g33140 / 60S ribosomal protein L9 (RPL90A/C) | |  |  |  |  |  | | --- | --- | --- | --- | --- | |  |  |  |  |  | | R13A5.8 | | | | | | |
|  |  | RAFL07-15-M07 | At1g04480 / 60S ribosomal protein L23 (RPL23A) | |  |  |  |  |  | | --- | --- | --- | --- | --- | |  |  |  |  |  | | B0336.10 | | | | | | |
|  |  | RAFL05-17-L17 | At3g55280 / 60S ribosomal protein L23A (RPL23aB) | |  |  |  |  |  | | --- | --- | --- | --- | --- | |  |  |  |  |  | | F52B5.6 | | | | | | |
|  |  | RAFL11-12-H04 | At3g25520 / 60S ribosomal protein L5 (RPL5A) | |  |  |  |  |  | | --- | --- | --- | --- | --- | |  |  |  |  |  | | F54C9.5 | | | | | | |
|  |  | RAFL05-18-P15 | At1g04480 / 60S ribosomal protein L23 (RPL23A) | |  |  |  |  |  | | --- | --- | --- | --- | --- | |  |  |  |  |  | | B0336.10 | | | | | | |
|  |  | RAFL11-07-B21 | At3g25520 / 60S ribosomal protein L5 (RPL5A) | |  |  |  |  |  | | --- | --- | --- | --- | --- | |  |  |  |  |  | | F54C9.5 | | | | | | |
|  | Cluster:3-0 | |  |  | 5 | 228 | 14 | 4416 | 0.0019486465 | 0.011691879 | 6 |
|  |  | RAFL07-10-D02 | At1g14320 / 60S ribosomal protein L10 (RPL10A)/Wilm's tumor suppressor protein-related | |  |  |  |  |  | | --- | --- | --- | --- | --- | |  |  |  |  |  | | F10B5.1 | | | | | | |
|  |  | RAFL09-10-P09 | At4g36130 / 60S ribosomal protein L8 (RPL8C) | |  |  |  |  |  | | --- | --- | --- | --- | --- | |  |  |  |  |  | | B0250.1 | | | | | | |
|  |  | RAFL08-10-G08 | At3g09630 / 60S ribosomal protein L4/L1 (RPL4A) | |  |  |  |  |  | | --- | --- | --- | --- | --- | |  |  |  |  |  | | B0041.4 | | | | | | |
|  |  | RAFL08-13-M06 | At3g11250 / 60S acidic ribosomal protein P0 (RPP0C) | |  |  |  |  |  | | --- | --- | --- | --- | --- | |  |  |  |  |  | | F25H2.10 | | | | | | |
|  |  | RAFL09-12-B12 | At4g36130 / 60S ribosomal protein L8 (RPL8C) | |  |  |  |  |  | | --- | --- | --- | --- | --- | |  |  |  |  |  | | B0250.1 | | | | | | |
|  | Cluster:2-0 | |  |  | 4 | 146 | 15 | 4498 | 0.002734157 | 0.016404942 | 6 |
|  |  | RAFL04-19-O24 | At5g02610 / 60S ribosomal protein L35 (RPL35D) | |  |  |  |  |  | | --- | --- | --- | --- | --- | |  |  |  |  |  | | ZK652.4 | | | | | | |
|  |  | RAFL06-10-L13 | At3g09630 / 60S ribosomal protein L4/L1 (RPL4A) | |  |  |  |  |  | | --- | --- | --- | --- | --- | |  |  |  |  |  | | B0041.4 | | | | | | |
|  |  | RAFL11-12-M17 | At3g25520 / 60S ribosomal protein L5 (RPL5A) | |  |  |  |  |  | | --- | --- | --- | --- | --- | |  |  |  |  |  | | F54C9.5 | | | | | | |
|  |  | RAFL03-06-H07 | At1g33140 / 60S ribosomal protein L9 (RPL90A/C) | |  |  |  |  |  | | --- | --- | --- | --- | --- | |  |  |  |  |  | | R13A5.8 | | | | | | |
| Y71G12B.27 | | | WI5\_id:Y71G12B.27 |  | A | B | C | D | P | P' | N |
|  | Cluster:2-1 | |  |  | 2 | 242 | 3 | 4416 | 0.024550742 | 0.098202966 | 4 |
|  |  | RAFL11-12-H04 | At3g25520 / 60S ribosomal protein L5 (RPL5A) | |  |  |  |  |  | | --- | --- | --- | --- | --- | |  |  |  |  |  | | F54C9.5 | | | | | | |
|  |  | RAFL11-07-B21 | At3g25520 / 60S ribosomal protein L5 (RPL5A) | |  |  |  |  |  | | --- | --- | --- | --- | --- | |  |  |  |  |  | | F54C9.5 | | | | | | |
| F46F11.5 | | | WI5\_id:F46F11.5 |  | A | B | C | D | P | P' | N |
|  | Cluster:4-1 | |  |  | 2 | 308 | 2 | 4351 | 0.024166461 | 0.07249938 | 3 |
|  |  | RAFL04-09-A19 | At4g38510 / probable H+-transporting ATPase | |  |  |  |  |  | | --- | --- | --- | --- | --- | |  |  |  |  |  | | F20B6.2 | | | | | | |
|  |  | RAFL07-17-H08 | At4g38510 / probable H+-transporting ATPase | |  |  |  |  |  | | --- | --- | --- | --- | --- | |  |  |  |  |  | | F20B6.2 | | | | | | |
| F25F8.2 | | | WI5\_id:F25F8.2 |  | A | B | C | D | P | P' | N |
|  | Cluster:9-0 | |  |  | 4 | 28 | 0 | 4631 | 1.8277981E-9 | 1.8277981E-9 | 1 |
|  |  | RAFL08-09-C23 | At1g54100 / aldehyde dehydrogenase, putative (ALDH) | |  |  |  |  |  | | --- | --- | --- | --- | --- | |  |  |  |  |  | | F01F1.6  Aldehyde dehydrogenase | | | | | | |
|  |  | RAFL04-09-D07 | At1g54100 / aldehyde dehydrogenase, putative (ALDH) | |  |  |  |  |  | | --- | --- | --- | --- | --- | |  |  |  |  |  | | F01F1.6  Aldehyde dehydrogenase | | | | | | |
|  |  | RAFL05-21-E06 | At1g54100 / aldehyde dehydrogenase, putative (ALDH) | |  |  |  |  |  | | --- | --- | --- | --- | --- | |  |  |  |  |  | | F01F1.6  Aldehyde dehydrogenase | | | | | | |
|  |  | RAFL08-15-L09 | At1g54100 / aldehyde dehydrogenase, putative (ALDH) | |  |  |  |  |  | | --- | --- | --- | --- | --- | |  |  |  |  |  | | F01F1.6  Aldehyde dehydrogenase | | | | | | |
| W04D2.1 | | | WI5\_id:W04D2.1 |  | A | B | C | D | P | P' | N |
|  | Cluster:4-1 | |  |  | 4 | 306 | 10 | 4343 | 0.011254616 | 0.07878231 | 7 |
|  |  | RAFL07-08-J17 | At1g13060 / 20S proteasome beta subunit E1 (PBE1) | |  |  |  |  |  | | --- | --- | --- | --- | --- | |  |  |  |  |  | | K05C4.1  Proteasome A-type and B-type | | | | | | |
|  |  | RAFL05-04-G23 | At1g77440 / 20S proteasome beta subunit C (PBC2) | |  |  |  |  |  | | --- | --- | --- | --- | --- | |  |  |  |  |  | | Y38A8.2 | | | | | | |
|  |  | RAFL04-18-F12 | At3g59920 / Rab GDP dissociation inhibitor | |  |  |  |  |  | | --- | --- | --- | --- | --- | |  |  |  |  |  | | Y57G11C.10  GDI-1 GDP dissociation inhibitor | | | | | | |
|  |  | RAFL05-21-D23 | At1g21720 / 20S proteasome beta subunit C (PBC1) | |  |  |  |  |  | | --- | --- | --- | --- | --- | |  |  |  |  |  | | Y38A8.2 | | | | | | |
|  | Cluster:2-1 | |  |  | 3 | 241 | 11 | 4408 | 0.033551577 | 0.23486103 | 7 |
|  |  | RAFL11-12-H04 | At3g25520 / 60S ribosomal protein L5 (RPL5A) | |  |  |  |  |  | | --- | --- | --- | --- | --- | |  |  |  |  |  | | F54C9.5 | | | | | | |
|  |  | RAFL04-18-N22 | At2g44120 / 60S ribosomal protein L7 (RPL7C) | |  |  |  |  |  | | --- | --- | --- | --- | --- | |  |  |  |  |  | | F53G12.10 | | | | | | |
|  |  | RAFL11-07-B21 | At3g25520 / 60S ribosomal protein L5 (RPL5A) | |  |  |  |  |  | | --- | --- | --- | --- | --- | |  |  |  |  |  | | F54C9.5 | | | | | | |
| Y45F10D.12 | | | WI5\_id:Y45F10D.12 |  | A | B | C | D | P | P' | N |
|  | Cluster:2-0 | |  |  | 1 | 149 | 0 | 4513 | 0.03216813 | 0.03216813 | 1 |
|  |  | RAFL05-01-F21 | At3g05590 / 60S ribosomal protein L18 (RPL18B) | |  |  |  |  |  | | --- | --- | --- | --- | --- | |  |  |  |  |  | | Y45F10D.12 | | | | | | |
| B0393.1 | | | WI5\_id:B0393.1 |  | A | B | C | D | P | P' | N |
|  | Cluster:4-0 | |  |  | 2 | 238 | 3 | 4420 | 0.023792636 | 0.09517054 | 4 |
|  |  | RAFL06-07-B07 | At5g55190 / GTP-binding protein atran3, putative | |  |  |  |  |  | | --- | --- | --- | --- | --- | |  |  |  |  |  | | K01G5.4 | | | | | | |
|  |  | RAFL05-17-C16 | At5g55190 / GTP-binding protein atran3, putative | |  |  |  |  |  | | --- | --- | --- | --- | --- | |  |  |  |  |  | | K01G5.4 | | | | | | |
| C39E9.14 | | | WI5\_id:C39E9.14 |  | A | B | C | D | P | P' | N |
|  | Cluster:5-0 | |  |  | 1 | 76 | 0 | 4586 | 0.016512975 | 0.016512975 | 1 |
|  |  | RAFL04-09-J19 | At1g56450 / 20S proteasome beta subunit G1 (PBG1) | |  |  |  |  |  | | --- | --- | --- | --- | --- | |  |  |  |  |  | | F39H11.5 | | | | | | |
| F59A2.4 | | | WI5\_id:F59A2.4 |  | A | B | C | D | P | P' | N |
|  | Cluster:5-0 | |  |  | 1 | 76 | 0 | 4586 | 0.016512975 | 0.016512975 | 1 |
|  |  | RAFL06-07-P18 | At3g60820 / 20S proteasome beta subunit F1 (PBF1) | |  |  |  |  |  | | --- | --- | --- | --- | --- | |  |  |  |  |  | | C02F5.9 | | | | | | |
| T05C12.6 | | | WI5\_id:T05C12.6 |  | A | B | C | D | P | P' | N |
|  | Cluster:8-0 | |  |  | 1 | 108 | 1 | 4553 | 0.0462095 | 0.092419 | 2 |
|  |  | RAFL05-03-L12 | At1g47128 / cysteine proteinase RD21A | |  |  |  |  |  | | --- | --- | --- | --- | --- | |  |  |  |  |  | | T03E6.7 | | | | | | |
| B0024.14 | | | WI5\_id:B0024.14 |  | A | B | C | D | P | P' | N |
|  | Cluster:4-2 | |  |  | 1 | 134 | 0 | 4528 | 0.028951319 | 0.028951319 | 1 |
|  |  | RAFL04-20-K07 | At3g53750 / actin (ACT3) | |  |  |  |  |  | | --- | --- | --- | --- | --- | |  |  |  |  |  | | T04C12.6  actin | | | | | | |
| F59A2.1 | | | WI5\_id:F59A2.1 |  | A | B | C | D | P | P' | N |
|  | Cluster:4-0 | |  |  | 2 | 238 | 1 | 4422 | 0.0076462985 | 0.015292597 | 2 |
|  |  | RAFL06-07-B07 | At5g55190 / GTP-binding protein atran3, putative | |  |  |  |  |  | | --- | --- | --- | --- | --- | |  |  |  |  |  | | K01G5.4 | | | | | | |
|  |  | RAFL05-17-C16 | At5g55190 / GTP-binding protein atran3, putative | |  |  |  |  |  | | --- | --- | --- | --- | --- | |  |  |  |  |  | | K01G5.4 | | | | | | |
| ZK20.5 | | | WI5\_id:ZK20.5 |  | A | B | C | D | P | P' | N |
|  | Cluster:6-1 | |  |  | 4 | 311 | 14 | 4334 | 0.029376008 | 0.3525121 | 12 |
|  |  | RAFL08-19-N19 | At3g05530 / 26S proteasome AAA-ATPase subunit RPT5a | |  |  |  |  |  | | --- | --- | --- | --- | --- | |  |  |  |  |  | | F56H1.4 | | | | | | |
|  |  | RAFL05-08-I15 | At4g24820 / 26S proteasome regulatory subunit (RPN7), putative | |  |  |  |  |  | | --- | --- | --- | --- | --- | |  |  |  |  |  | | F49C12.8 | | | | | | |
|  |  | RAFL07-13-D20 | At4g24820 / 26S proteasome regulatory subunit (RPN7), putative | |  |  |  |  |  | | --- | --- | --- | --- | --- | |  |  |  |  |  | | F49C12.8 | | | | | | |
|  |  | RAFL11-04-H03 | At1g20200 / 26S proteasome regulatory subunit S3 (RPN3), putative | |  |  |  |  |  | | --- | --- | --- | --- | --- | |  |  |  |  |  | | C30C11.2 | | | | | | |
| F22B7.5 | | | WI5\_id:F22B7.5 |  | A | B | C | D | P | P' | N |
|  | Cluster:4-2 | |  |  | 1 | 134 | 0 | 4528 | 0.028951319 | 0.028951319 | 1 |
|  |  | RAFL04-17-F17 | At4g37910 / heat shock protein mtHsc70-1 | |  |  |  |  |  | | --- | --- | --- | --- | --- | |  |  |  |  |  | | C37H5.8 | | | | | | |
|  | | | | | | | | | | | |
| Cluster:8-1 | | |  |  | A | B | C | D | P | P' | N |
|  | T04C10.1 | | WI5\_id:T04C10.1 |  | 1 | 161 | 0 | 4501 | 0.034741584 | 0.034741584 | 1 |
|  |  | RAFL08-12-L24 | At3g08720 / ribosomal-protein S6 kinase (ATPK19) -related | |  |  |  |  |  | | --- | --- | --- | --- | --- | |  |  |  |  |  | | T04C10.1 | | | | | | |
| Cluster:0-2 | | |  |  | A | B | C | D | P | P' | N |
|  | C52B11.2 | | WI5\_id:C52B11.2 |  | 1 | 78 | 0 | 4584 | 0.016941883 | 0.016941883 | 1 |
|  |  | RAFL04-17-G02 | At1g56340 / calreticulin 1 (CRT1) | |  |  |  |  |  | | --- | --- | --- | --- | --- | |  |  |  |  |  | | Y38A10A.5 | | | | | | |
|  | C07A12.3 | | WI5\_id:C07A12.3 |  | 1 | 78 | 0 | 4584 | 0.016941883 | 0.016941883 | 1 |
|  |  | RAFL04-17-G02 | At1g56340 / calreticulin 1 (CRT1) | |  |  |  |  |  | | --- | --- | --- | --- | --- | |  |  |  |  |  | | Y38A10A.5 | | | | | | |
|  | C35B1.1 | | WI5\_id:C35B1.1 |  | 1 | 78 | 1 | 4583 | 0.03360031 | 0.06720062 | 2 |
|  |  | RAFL04-17-G02 | At1g56340 / calreticulin 1 (CRT1) | |  |  |  |  |  | | --- | --- | --- | --- | --- | |  |  |  |  |  | | Y38A10A.5 | | | | | | |
|  | C06E4.2 | | WI5\_id:C06E4.2 |  | 1 | 78 | 1 | 4583 | 0.03360031 | 0.06720062 | 2 |
|  |  | RAFL04-17-G02 | At1g56340 / calreticulin 1 (CRT1) | |  |  |  |  |  | | --- | --- | --- | --- | --- | |  |  |  |  |  | | Y38A10A.5 | | | | | | |
|  | F10C5.1 | | WI5\_id:F10C5.1 |  | 1 | 78 | 2 | 4582 | 0.049979966 | 0.1499399 | 3 |
|  |  | RAFL04-17-G02 | At1g56340 / calreticulin 1 (CRT1) | |  |  |  |  |  | | --- | --- | --- | --- | --- | |  |  |  |  |  | | Y38A10A.5 | | | | | | |
| Cluster:4-0 | | |  |  | A | B | C | D | P | P' | N |
|  | F47G6.1 | | WI5\_id:F47G6.1 |  | 2 | 238 | 0 | 4423 | 0.0026385873 | 0.0026385873 | 1 |
|  |  | RAFL08-19-E03 | At1g77510 / protein disulfide isomerase, putative | |  |  |  |  |  | | --- | --- | --- | --- | --- | |  |  |  |  |  | | H06O01.1 | | | | | | |
|  |  | RAFL09-06-G15 | At1g45000 / 26S proteasome regulatory particle triple-A ATPase subunit4 -related | |  |  |  |  |  | | --- | --- | --- | --- | --- | |  |  |  |  |  | | F23F1.8 | | | | | | |
|  | F53G2.6 | | WI5\_id:F53G2.6 |  | 2 | 238 | 1 | 4422 | 0.0076462985 | 0.015292597 | 2 |
|  |  | RAFL06-07-B07 | At5g55190 / GTP-binding protein atran3, putative | |  |  |  |  |  | | --- | --- | --- | --- | --- | |  |  |  |  |  | | K01G5.4 | | | | | | |
|  |  | RAFL05-17-C16 | At5g55190 / GTP-binding protein atran3, putative | |  |  |  |  |  | | --- | --- | --- | --- | --- | |  |  |  |  |  | | K01G5.4 | | | | | | |
|  | F56G4.5 | | WI5\_id:F56G4.5 |  | 2 | 238 | 1 | 4422 | 0.0076462985 | 0.015292597 | 2 |
|  |  | RAFL06-07-B07 | At5g55190 / GTP-binding protein atran3, putative | |  |  |  |  |  | | --- | --- | --- | --- | --- | |  |  |  |  |  | | K01G5.4 | | | | | | |
|  |  | RAFL05-17-C16 | At5g55190 / GTP-binding protein atran3, putative | |  |  |  |  |  | | --- | --- | --- | --- | --- | |  |  |  |  |  | | K01G5.4 | | | | | | |
|  | Y24F12A.2 | | WI5\_id:Y24F12A.2 |  | 2 | 238 | 1 | 4422 | 0.0076462985 | 0.015292597 | 2 |
|  |  | RAFL06-07-B07 | At5g55190 / GTP-binding protein atran3, putative | |  |  |  |  |  | | --- | --- | --- | --- | --- | |  |  |  |  |  | | K01G5.4 | | | | | | |
|  |  | RAFL05-17-C16 | At5g55190 / GTP-binding protein atran3, putative | |  |  |  |  |  | | --- | --- | --- | --- | --- | |  |  |  |  |  | | K01G5.4 | | | | | | |
|  | C56E6.3 | | WI5\_id:C56E6.3 |  | 2 | 238 | 1 | 4422 | 0.0076462985 | 0.015292597 | 2 |
|  |  | RAFL06-07-B07 | At5g55190 / GTP-binding protein atran3, putative | |  |  |  |  |  | | --- | --- | --- | --- | --- | |  |  |  |  |  | | K01G5.4 | | | | | | |
|  |  | RAFL05-17-C16 | At5g55190 / GTP-binding protein atran3, putative | |  |  |  |  |  | | --- | --- | --- | --- | --- | |  |  |  |  |  | | K01G5.4 | | | | | | |
|  | B0019.3 | | WI5\_id:B0019.3 |  | 2 | 238 | 1 | 4422 | 0.0076462985 | 0.015292597 | 2 |
|  |  | RAFL06-07-B07 | At5g55190 / GTP-binding protein atran3, putative | |  |  |  |  |  | | --- | --- | --- | --- | --- | |  |  |  |  |  | | K01G5.4 | | | | | | |
|  |  | RAFL05-17-C16 | At5g55190 / GTP-binding protein atran3, putative | |  |  |  |  |  | | --- | --- | --- | --- | --- | |  |  |  |  |  | | K01G5.4 | | | | | | |
|  | B0222.6 | | WI5\_id:B0222.6 |  | 2 | 238 | 1 | 4422 | 0.0076462985 | 0.015292597 | 2 |
|  |  | RAFL06-07-B07 | At5g55190 / GTP-binding protein atran3, putative | |  |  |  |  |  | | --- | --- | --- | --- | --- | |  |  |  |  |  | | K01G5.4 | | | | | | |
|  |  | RAFL05-17-C16 | At5g55190 / GTP-binding protein atran3, putative | |  |  |  |  |  | | --- | --- | --- | --- | --- | |  |  |  |  |  | | K01G5.4 | | | | | | |
|  | B0280.5 | | WI5\_id:B0280.5 |  | 2 | 238 | 1 | 4422 | 0.0076462985 | 0.015292597 | 2 |
|  |  | RAFL06-07-B07 | At5g55190 / GTP-binding protein atran3, putative | |  |  |  |  |  | | --- | --- | --- | --- | --- | |  |  |  |  |  | | K01G5.4 | | | | | | |
|  |  | RAFL05-17-C16 | At5g55190 / GTP-binding protein atran3, putative | |  |  |  |  |  | | --- | --- | --- | --- | --- | |  |  |  |  |  | | K01G5.4 | | | | | | |
|  | C04F6.1 | | WI5\_id:C04F6.1 |  | 2 | 238 | 1 | 4422 | 0.0076462985 | 0.015292597 | 2 |
|  |  | RAFL06-07-B07 | At5g55190 / GTP-binding protein atran3, putative | |  |  |  |  |  | | --- | --- | --- | --- | --- | |  |  |  |  |  | | K01G5.4 | | | | | | |
|  |  | RAFL05-17-C16 | At5g55190 / GTP-binding protein atran3, putative | |  |  |  |  |  | | --- | --- | --- | --- | --- | |  |  |  |  |  | | K01G5.4 | | | | | | |
|  | C16A11.5 | | WI5\_id:C16A11.5 |  | 2 | 238 | 1 | 4422 | 0.0076462985 | 0.015292597 | 2 |
|  |  | RAFL06-07-B07 | At5g55190 / GTP-binding protein atran3, putative | |  |  |  |  |  | | --- | --- | --- | --- | --- | |  |  |  |  |  | | K01G5.4 | | | | | | |
|  |  | RAFL05-17-C16 | At5g55190 / GTP-binding protein atran3, putative | |  |  |  |  |  | | --- | --- | --- | --- | --- | |  |  |  |  |  | | K01G5.4 | | | | | | |
|  | C36B1.12 | | WI5\_id:C36B1.12 |  | 2 | 238 | 1 | 4422 | 0.0076462985 | 0.015292597 | 2 |
|  |  | RAFL06-07-B07 | At5g55190 / GTP-binding protein atran3, putative | |  |  |  |  |  | | --- | --- | --- | --- | --- | |  |  |  |  |  | | K01G5.4 | | | | | | |
|  |  | RAFL05-17-C16 | At5g55190 / GTP-binding protein atran3, putative | |  |  |  |  |  | | --- | --- | --- | --- | --- | |  |  |  |  |  | | K01G5.4 | | | | | | |
|  | D2005.4 | | WI5\_id:D2005.4 |  | 2 | 238 | 1 | 4422 | 0.0076462985 | 0.015292597 | 2 |
|  |  | RAFL06-07-B07 | At5g55190 / GTP-binding protein atran3, putative | |  |  |  |  |  | | --- | --- | --- | --- | --- | |  |  |  |  |  | | K01G5.4 | | | | | | |
|  |  | RAFL05-17-C16 | At5g55190 / GTP-binding protein atran3, putative | |  |  |  |  |  | | --- | --- | --- | --- | --- | |  |  |  |  |  | | K01G5.4 | | | | | | |
|  | D2045.2 | | WI5\_id:D2045.2 |  | 2 | 238 | 1 | 4422 | 0.0076462985 | 0.015292597 | 2 |
|  |  | RAFL06-07-B07 | At5g55190 / GTP-binding protein atran3, putative | |  |  |  |  |  | | --- | --- | --- | --- | --- | |  |  |  |  |  | | K01G5.4 | | | | | | |
|  |  | RAFL05-17-C16 | At5g55190 / GTP-binding protein atran3, putative | |  |  |  |  |  | | --- | --- | --- | --- | --- | |  |  |  |  |  | | K01G5.4 | | | | | | |
|  | F10C5.2 | | WI5\_id:F10C5.2 |  | 2 | 238 | 1 | 4422 | 0.0076462985 | 0.015292597 | 2 |
|  |  | RAFL06-07-B07 | At5g55190 / GTP-binding protein atran3, putative | |  |  |  |  |  | | --- | --- | --- | --- | --- | |  |  |  |  |  | | K01G5.4 | | | | | | |
|  |  | RAFL05-17-C16 | At5g55190 / GTP-binding protein atran3, putative | |  |  |  |  |  | | --- | --- | --- | --- | --- | |  |  |  |  |  | | K01G5.4 | | | | | | |
|  | T24F1.2 | | WI5\_id:T24F1.2 |  | 2 | 238 | 1 | 4422 | 0.0076462985 | 0.015292597 | 2 |
|  |  | RAFL06-07-B07 | At5g55190 / GTP-binding protein atran3, putative | |  |  |  |  |  | | --- | --- | --- | --- | --- | |  |  |  |  |  | | K01G5.4 | | | | | | |
|  |  | RAFL05-17-C16 | At5g55190 / GTP-binding protein atran3, putative | |  |  |  |  |  | | --- | --- | --- | --- | --- | |  |  |  |  |  | | K01G5.4 | | | | | | |
|  | ZK742.1 | | WI5\_id:ZK742.1 |  | 2 | 238 | 1 | 4422 | 0.0076462985 | 0.015292597 | 2 |
|  |  | RAFL06-07-B07 | At5g55190 / GTP-binding protein atran3, putative | |  |  |  |  |  | | --- | --- | --- | --- | --- | |  |  |  |  |  | | K01G5.4 | | | | | | |
|  |  | RAFL05-17-C16 | At5g55190 / GTP-binding protein atran3, putative | |  |  |  |  |  | | --- | --- | --- | --- | --- | |  |  |  |  |  | | K01G5.4 | | | | | | |
|  | F28B3.8 | | WI5\_id:F28B3.8 |  | 2 | 238 | 1 | 4422 | 0.0076462985 | 0.015292597 | 2 |
|  |  | RAFL06-07-B07 | At5g55190 / GTP-binding protein atran3, putative | |  |  |  |  |  | | --- | --- | --- | --- | --- | |  |  |  |  |  | | K01G5.4 | | | | | | |
|  |  | RAFL05-17-C16 | At5g55190 / GTP-binding protein atran3, putative | |  |  |  |  |  | | --- | --- | --- | --- | --- | |  |  |  |  |  | | K01G5.4 | | | | | | |
|  | Y48G1A.5 | | WI5\_id:Y48G1A.5 |  | 2 | 238 | 1 | 4422 | 0.0076462985 | 0.015292597 | 2 |
|  |  | RAFL06-07-B07 | At5g55190 / GTP-binding protein atran3, putative | |  |  |  |  |  | | --- | --- | --- | --- | --- | |  |  |  |  |  | | K01G5.4 | | | | | | |
|  |  | RAFL05-17-C16 | At5g55190 / GTP-binding protein atran3, putative | |  |  |  |  |  | | --- | --- | --- | --- | --- | |  |  |  |  |  | | K01G5.4 | | | | | | |
|  | F59A2.1 | | WI5\_id:F59A2.1 |  | 2 | 238 | 1 | 4422 | 0.0076462985 | 0.015292597 | 2 |
|  |  | RAFL06-07-B07 | At5g55190 / GTP-binding protein atran3, putative | |  |  |  |  |  | | --- | --- | --- | --- | --- | |  |  |  |  |  | | K01G5.4 | | | | | | |
|  |  | RAFL05-17-C16 | At5g55190 / GTP-binding protein atran3, putative | |  |  |  |  |  | | --- | --- | --- | --- | --- | |  |  |  |  |  | | K01G5.4 | | | | | | |
|  | K08B12.5 | | WI5\_id:K08B12.5 |  | 2 | 238 | 1 | 4422 | 0.0076462985 | 0.015292597 | 2 |
|  |  | RAFL06-07-B07 | At5g55190 / GTP-binding protein atran3, putative | |  |  |  |  |  | | --- | --- | --- | --- | --- | |  |  |  |  |  | | K01G5.4 | | | | | | |
|  |  | RAFL05-17-C16 | At5g55190 / GTP-binding protein atran3, putative | |  |  |  |  |  | | --- | --- | --- | --- | --- | |  |  |  |  |  | | K01G5.4 | | | | | | |
|  | C53D5.6 | | WI5\_id:C53D5.6 |  | 2 | 238 | 1 | 4422 | 0.0076462985 | 0.015292597 | 2 |
|  |  | RAFL06-07-B07 | At5g55190 / GTP-binding protein atran3, putative | |  |  |  |  |  | | --- | --- | --- | --- | --- | |  |  |  |  |  | | K01G5.4 | | | | | | |
|  |  | RAFL05-17-C16 | At5g55190 / GTP-binding protein atran3, putative | |  |  |  |  |  | | --- | --- | --- | --- | --- | |  |  |  |  |  | | K01G5.4 | | | | | | |
|  | C04G2.6 | | WI5\_id:C04G2.6 |  | 2 | 238 | 1 | 4422 | 0.0076462985 | 0.015292597 | 2 |
|  |  | RAFL06-07-B07 | At5g55190 / GTP-binding protein atran3, putative | |  |  |  |  |  | | --- | --- | --- | --- | --- | |  |  |  |  |  | | K01G5.4 | | | | | | |
|  |  | RAFL05-17-C16 | At5g55190 / GTP-binding protein atran3, putative | |  |  |  |  |  | | --- | --- | --- | --- | --- | |  |  |  |  |  | | K01G5.4 | | | | | | |
|  | T04C12.6 | | WI5\_id:T04C12.6 |  | 2 | 238 | 2 | 4421 | 0.0147742275 | 0.044322684 | 3 |
|  |  | RAFL06-07-B07 | At5g55190 / GTP-binding protein atran3, putative | |  |  |  |  |  | | --- | --- | --- | --- | --- | |  |  |  |  |  | | K01G5.4 | | | | | | |
|  |  | RAFL05-17-C16 | At5g55190 / GTP-binding protein atran3, putative | |  |  |  |  |  | | --- | --- | --- | --- | --- | |  |  |  |  |  | | K01G5.4 | | | | | | |
|  | F26E4.8 | | WI5\_id:F26E4.8 |  | 2 | 238 | 2 | 4421 | 0.0147742275 | 0.044322684 | 3 |
|  |  | RAFL06-07-B07 | At5g55190 / GTP-binding protein atran3, putative | |  |  |  |  |  | | --- | --- | --- | --- | --- | |  |  |  |  |  | | K01G5.4 | | | | | | |
|  |  | RAFL05-17-C16 | At5g55190 / GTP-binding protein atran3, putative | |  |  |  |  |  | | --- | --- | --- | --- | --- | |  |  |  |  |  | | K01G5.4 | | | | | | |
|  | C39F7.4 | | WI5\_id:C39F7.4 |  | 2 | 238 | 2 | 4421 | 0.0147742275 | 0.044322684 | 3 |
|  |  | RAFL06-07-B07 | At5g55190 / GTP-binding protein atran3, putative | |  |  |  |  |  | | --- | --- | --- | --- | --- | |  |  |  |  |  | | K01G5.4 | | | | | | |
|  |  | RAFL05-17-C16 | At5g55190 / GTP-binding protein atran3, putative | |  |  |  |  |  | | --- | --- | --- | --- | --- | |  |  |  |  |  | | K01G5.4 | | | | | | |
|  | F40F11.1 | | WI5\_id:F40F11.1 |  | 3 | 237 | 8 | 4415 | 0.016332459 | 0.065329835 | 4 |
|  |  | RAFL06-07-B07 | At5g55190 / GTP-binding protein atran3, putative | |  |  |  |  |  | | --- | --- | --- | --- | --- | |  |  |  |  |  | | K01G5.4 | | | | | | |
|  |  | RAFL05-17-C16 | At5g55190 / GTP-binding protein atran3, putative | |  |  |  |  |  | | --- | --- | --- | --- | --- | |  |  |  |  |  | | K01G5.4 | | | | | | |
|  |  | RAFL07-07-O08 | At4g30800 / 40S ribosomal protein S11 (RPS11B) | |  |  |  |  |  | | --- | --- | --- | --- | --- | |  |  |  |  |  | | F40F11.1 | | | | | | |
|  | K01G5.4 | | WI5\_id:K01G5.4 |  | 3 | 237 | 9 | 4414 | 0.020962995 | 0.16770396 | 8 |
|  |  | RAFL06-07-B07 | At5g55190 / GTP-binding protein atran3, putative | |  |  |  |  |  | | --- | --- | --- | --- | --- | |  |  |  |  |  | | K01G5.4 | | | | | | |
|  |  | RAFL05-17-C16 | At5g55190 / GTP-binding protein atran3, putative | |  |  |  |  |  | | --- | --- | --- | --- | --- | |  |  |  |  |  | | K01G5.4 | | | | | | |
|  |  | RAFL07-07-O08 | At4g30800 / 40S ribosomal protein S11 (RPS11B) | |  |  |  |  |  | | --- | --- | --- | --- | --- | |  |  |  |  |  | | F40F11.1 | | | | | | |
|  | B0393.1 | | WI5\_id:B0393.1 |  | 2 | 238 | 3 | 4420 | 0.023792636 | 0.09517054 | 4 |
|  |  | RAFL06-07-B07 | At5g55190 / GTP-binding protein atran3, putative | |  |  |  |  |  | | --- | --- | --- | --- | --- | |  |  |  |  |  | | K01G5.4 | | | | | | |
|  |  | RAFL05-17-C16 | At5g55190 / GTP-binding protein atran3, putative | |  |  |  |  |  | | --- | --- | --- | --- | --- | |  |  |  |  |  | | K01G5.4 | | | | | | |
|  | Y38A10A.5 | | WI5\_id:Y38A10A.5 |  | 2 | 238 | 3 | 4420 | 0.023792636 | 0.09517054 | 4 |
|  |  | RAFL06-07-B07 | At5g55190 / GTP-binding protein atran3, putative | |  |  |  |  |  | | --- | --- | --- | --- | --- | |  |  |  |  |  | | K01G5.4 | | | | | | |
|  |  | RAFL05-17-C16 | At5g55190 / GTP-binding protein atran3, putative | |  |  |  |  |  | | --- | --- | --- | --- | --- | |  |  |  |  |  | | K01G5.4 | | | | | | |
|  | Y59H11AM.3 | | WI5\_id:Y59H11AM.3 |  | 2 | 238 | 4 | 4419 | 0.034489654 | 0.10346896 | 3 |
|  |  | RAFL07-11-O12 | At1g56070 / elongation factor -related | |  |  |  |  |  | | --- | --- | --- | --- | --- | |  |  |  |  |  | | F25H5.4 | | | | | | |
|  |  | RAFL04-17-N12 | At1g56070 / elongation factor -related | |  |  |  |  |  | | --- | --- | --- | --- | --- | |  |  |  |  |  | | F25H5.4 | | | | | | |
|  | F39B2.2 | | WI5\_id:F39B2.2 |  | 2 | 238 | 4 | 4419 | 0.034489654 | 0.10346896 | 3 |
|  |  | RAFL07-11-O12 | At1g56070 / elongation factor -related | |  |  |  |  |  | | --- | --- | --- | --- | --- | |  |  |  |  |  | | F25H5.4  Elongation factor Tu family (contains ATP/GTP binding P-loop) | | | | | | |
|  |  | RAFL04-17-N12 | At1g56070 / elongation factor -related | |  |  |  |  |  | | --- | --- | --- | --- | --- | |  |  |  |  |  | | F25H5.4  Elongation factor Tu family (contains ATP/GTP binding P-loop) | | | | | | |
|  | F25H5.4 | | WI5\_id:F25H5.4 |  | 2 | 238 | 4 | 4419 | 0.034489654 | 0.10346896 | 3 |
|  |  | RAFL07-11-O12 | At1g56070 / elongation factor -related | |  |  |  |  |  | | --- | --- | --- | --- | --- | |  |  |  |  |  | | F25H5.4 | | | | | | |
|  |  | RAFL04-17-N12 | At1g56070 / elongation factor -related | |  |  |  |  |  | | --- | --- | --- | --- | --- | |  |  |  |  |  | | F25H5.4 | | | | | | |
|  | W06D4.6 | | WI5\_id:W06D4.6 |  | 2 | 238 | 5 | 4418 | 0.046670057 | 0.18668023 | 4 |
|  |  | RAFL07-11-O12 | At1g56070 / elongation factor -related | |  |  |  |  |  | | --- | --- | --- | --- | --- | |  |  |  |  |  | | F25H5.4 | | | | | | |
|  |  | RAFL04-17-N12 | At1g56070 / elongation factor -related | |  |  |  |  |  | | --- | --- | --- | --- | --- | |  |  |  |  |  | | F25H5.4 | | | | | | |
| Cluster:5-2 | | |  |  | A | B | C | D | P | P' | N |
|  | F29F11.1 | | WI5\_id:F29F11.1 |  | 1 | 125 | 0 | 4537 | 0.027021231 | 0.027021231 | 1 |
|  |  | RAFL07-09-M15 | At3g29360 / UDP-glucose dehydrogenase -related | |  |  |  |  |  | | --- | --- | --- | --- | --- | |  |  |  |  |  | | F29F11.1 | | | | | | |
|  | R07E3.7 | | WI5\_id:R07E3.7 |  | 1 | 125 | 0 | 4537 | 0.027021231 | 0.027021231 | 1 |
|  |  | RAFL07-09-M15 | At3g29360 / UDP-glucose dehydrogenase -related | |  |  |  |  |  | | --- | --- | --- | --- | --- | |  |  |  |  |  | | F29F11.1 | | | | | | |
| Cluster:3-2 | | |  |  | A | B | C | D | P | P' | N |
|  | C15F1.6 | | WI5\_id:C15F1.6 |  | 1 | 36 | 0 | 4626 | 0.007934806 | 0.007934806 | 1 |
|  |  | RAFL06-08-F13 | At3g55360 / 3-oxo-5-alpha-steroid 4-dehydrogenase (steroid 5-alpha-reductase) family | |  |  |  |  |  | | --- | --- | --- | --- | --- | |  |  |  |  |  | | C15F1.6 | | | | | | |
|  | C28H8.12 | | WI5\_id:C28H8.12 |  | 1 | 36 | 4 | 4622 | 0.039065886 | 0.15626355 | 4 |
|  |  | RAFL06-08-F13 | At3g55360 / 3-oxo-5-alpha-steroid 4-dehydrogenase (steroid 5-alpha-reductase) family | |  |  |  |  |  | | --- | --- | --- | --- | --- | |  |  |  |  |  | | C15F1.6 | | | | | | |
| Cluster:5-0 | | |  |  | A | B | C | D | P | P' | N |
|  | C08F8.8 | | WI5\_id:C08F8.8 |  | 4 | 73 | 1 | 4585 | 3.3961462E-7 | 6.7922923E-7 | 2 |
|  |  | RAFL04-15-M13 | At5g56030 / heat shock protein 81-2 (HSP81-2) | |  |  |  |  |  | | --- | --- | --- | --- | --- | |  |  |  |  |  | | C47E8.5 | | | | | | |
|  |  | RAFL07-13-H08 | At5g56010 / heat shock protein, putative | |  |  |  |  |  | | --- | --- | --- | --- | --- | |  |  |  |  |  | | C47E8.5 | | | | | | |
|  |  | RAFL09-06-O18 | At5g56030 / heat shock protein 81-2 (HSP81-2) | |  |  |  |  |  | | --- | --- | --- | --- | --- | |  |  |  |  |  | | C47E8.5 | | | | | | |
|  |  | RAFL05-16-L15 | At5g56010 / heat shock protein, putative | |  |  |  |  |  | | --- | --- | --- | --- | --- | |  |  |  |  |  | | C47E8.5 | | | | | | |
|  | T19A5.2 | | WI5\_id:T19A5.2 |  | 4 | 73 | 1 | 4585 | 3.3961462E-7 | 6.7922923E-7 | 2 |
|  |  | RAFL04-15-M13 | At5g56030 / heat shock protein 81-2 (HSP81-2) | |  |  |  |  |  | | --- | --- | --- | --- | --- | |  |  |  |  |  | | C47E8.5 | | | | | | |
|  |  | RAFL07-13-H08 | At5g56010 / heat shock protein, putative | |  |  |  |  |  | | --- | --- | --- | --- | --- | |  |  |  |  |  | | C47E8.5 | | | | | | |
|  |  | RAFL09-06-O18 | At5g56030 / heat shock protein 81-2 (HSP81-2) | |  |  |  |  |  | | --- | --- | --- | --- | --- | |  |  |  |  |  | | C47E8.5 | | | | | | |
|  |  | RAFL05-16-L15 | At5g56010 / heat shock protein, putative | |  |  |  |  |  | | --- | --- | --- | --- | --- | |  |  |  |  |  | | C47E8.5 | | | | | | |
|  | C37C3.6 | | WI5\_id:C37C3.6 |  | 4 | 73 | 1 | 4585 | 3.3961462E-7 | 6.7922923E-7 | 2 |
|  |  | RAFL04-15-M13 | At5g56030 / heat shock protein 81-2 (HSP81-2) | |  |  |  |  |  | | --- | --- | --- | --- | --- | |  |  |  |  |  | | C47E8.5  heat shock protein (HSP90) | | | | | | |
|  |  | RAFL07-13-H08 | At5g56010 / heat shock protein, putative | |  |  |  |  |  | | --- | --- | --- | --- | --- | |  |  |  |  |  | | C47E8.5  heat shock protein (HSP90) | | | | | | |
|  |  | RAFL09-06-O18 | At5g56030 / heat shock protein 81-2 (HSP81-2) | |  |  |  |  |  | | --- | --- | --- | --- | --- | |  |  |  |  |  | | C47E8.5  heat shock protein (HSP90) | | | | | | |
|  |  | RAFL05-16-L15 | At5g56010 / heat shock protein, putative | |  |  |  |  |  | | --- | --- | --- | --- | --- | |  |  |  |  |  | | C47E8.5  heat shock protein (HSP90) | | | | | | |
|  | Y92C3B.2 | | WI5\_id:Y92C3B.2 |  | 4 | 73 | 1 | 4585 | 3.3961462E-7 | 6.7922923E-7 | 2 |
|  |  | RAFL04-15-M13 | At5g56030 / heat shock protein 81-2 (HSP81-2) | |  |  |  |  |  | | --- | --- | --- | --- | --- | |  |  |  |  |  | | C47E8.5 | | | | | | |
|  |  | RAFL07-13-H08 | At5g56010 / heat shock protein, putative | |  |  |  |  |  | | --- | --- | --- | --- | --- | |  |  |  |  |  | | C47E8.5 | | | | | | |
|  |  | RAFL09-06-O18 | At5g56030 / heat shock protein 81-2 (HSP81-2) | |  |  |  |  |  | | --- | --- | --- | --- | --- | |  |  |  |  |  | | C47E8.5 | | | | | | |
|  |  | RAFL05-16-L15 | At5g56010 / heat shock protein, putative | |  |  |  |  |  | | --- | --- | --- | --- | --- | |  |  |  |  |  | | C47E8.5 | | | | | | |
|  | ZC434.8 | | WI5\_id:ZC434.8 |  | 4 | 73 | 1 | 4585 | 3.3961462E-7 | 6.7922923E-7 | 2 |
|  |  | RAFL04-15-M13 | At5g56030 / heat shock protein 81-2 (HSP81-2) | |  |  |  |  |  | | --- | --- | --- | --- | --- | |  |  |  |  |  | | C47E8.5 | | | | | | |
|  |  | RAFL07-13-H08 | At5g56010 / heat shock protein, putative | |  |  |  |  |  | | --- | --- | --- | --- | --- | |  |  |  |  |  | | C47E8.5 | | | | | | |
|  |  | RAFL09-06-O18 | At5g56030 / heat shock protein 81-2 (HSP81-2) | |  |  |  |  |  | | --- | --- | --- | --- | --- | |  |  |  |  |  | | C47E8.5 | | | | | | |
|  |  | RAFL05-16-L15 | At5g56010 / heat shock protein, putative | |  |  |  |  |  | | --- | --- | --- | --- | --- | |  |  |  |  |  | | C47E8.5 | | | | | | |
|  | F42A10.3 | | WI5\_id:F42A10.3 |  | 4 | 73 | 1 | 4585 | 3.3961462E-7 | 6.7922923E-7 | 2 |
|  |  | RAFL04-15-M13 | At5g56030 / heat shock protein 81-2 (HSP81-2) | |  |  |  |  |  | | --- | --- | --- | --- | --- | |  |  |  |  |  | | C47E8.5 | | | | | | |
|  |  | RAFL07-13-H08 | At5g56010 / heat shock protein, putative | |  |  |  |  |  | | --- | --- | --- | --- | --- | |  |  |  |  |  | | C47E8.5 | | | | | | |
|  |  | RAFL09-06-O18 | At5g56030 / heat shock protein 81-2 (HSP81-2) | |  |  |  |  |  | | --- | --- | --- | --- | --- | |  |  |  |  |  | | C47E8.5 | | | | | | |
|  |  | RAFL05-16-L15 | At5g56010 / heat shock protein, putative | |  |  |  |  |  | | --- | --- | --- | --- | --- | |  |  |  |  |  | | C47E8.5 | | | | | | |
|  | R07E5.8 | | WI5\_id:R07E5.8 |  | 4 | 73 | 1 | 4585 | 3.3961462E-7 | 6.7922923E-7 | 2 |
|  |  | RAFL04-15-M13 | At5g56030 / heat shock protein 81-2 (HSP81-2) | |  |  |  |  |  | | --- | --- | --- | --- | --- | |  |  |  |  |  | | C47E8.5 | | | | | | |
|  |  | RAFL07-13-H08 | At5g56010 / heat shock protein, putative | |  |  |  |  |  | | --- | --- | --- | --- | --- | |  |  |  |  |  | | C47E8.5 | | | | | | |
|  |  | RAFL09-06-O18 | At5g56030 / heat shock protein 81-2 (HSP81-2) | |  |  |  |  |  | | --- | --- | --- | --- | --- | |  |  |  |  |  | | C47E8.5 | | | | | | |
|  |  | RAFL05-16-L15 | At5g56010 / heat shock protein, putative | |  |  |  |  |  | | --- | --- | --- | --- | --- | |  |  |  |  |  | | C47E8.5 | | | | | | |
|  | W08F4.8 | | WI5\_id:W08F4.8 |  | 4 | 73 | 1 | 4585 | 3.3961462E-7 | 6.7922923E-7 | 2 |
|  |  | RAFL04-15-M13 | At5g56030 / heat shock protein 81-2 (HSP81-2) | |  |  |  |  |  | | --- | --- | --- | --- | --- | |  |  |  |  |  | | C47E8.5 | | | | | | |
|  |  | RAFL07-13-H08 | At5g56010 / heat shock protein, putative | |  |  |  |  |  | | --- | --- | --- | --- | --- | |  |  |  |  |  | | C47E8.5 | | | | | | |
|  |  | RAFL09-06-O18 | At5g56030 / heat shock protein 81-2 (HSP81-2) | |  |  |  |  |  | | --- | --- | --- | --- | --- | |  |  |  |  |  | | C47E8.5 | | | | | | |
|  |  | RAFL05-16-L15 | At5g56010 / heat shock protein, putative | |  |  |  |  |  | | --- | --- | --- | --- | --- | |  |  |  |  |  | | C47E8.5 | | | | | | |
|  | C17G10.2 | | WI5\_id:C17G10.2 |  | 4 | 73 | 1 | 4585 | 3.3961462E-7 | 6.7922923E-7 | 2 |
|  |  | RAFL04-15-M13 | At5g56030 / heat shock protein 81-2 (HSP81-2) | |  |  |  |  |  | | --- | --- | --- | --- | --- | |  |  |  |  |  | | C47E8.5 | | | | | | |
|  |  | RAFL07-13-H08 | At5g56010 / heat shock protein, putative | |  |  |  |  |  | | --- | --- | --- | --- | --- | |  |  |  |  |  | | C47E8.5 | | | | | | |
|  |  | RAFL09-06-O18 | At5g56030 / heat shock protein 81-2 (HSP81-2) | |  |  |  |  |  | | --- | --- | --- | --- | --- | |  |  |  |  |  | | C47E8.5 | | | | | | |
|  |  | RAFL05-16-L15 | At5g56010 / heat shock protein, putative | |  |  |  |  |  | | --- | --- | --- | --- | --- | |  |  |  |  |  | | C47E8.5 | | | | | | |
|  | F32A11.2 | | WI5\_id:F32A11.2 |  | 4 | 73 | 2 | 4584 | 1.0060772E-6 | 3.0182316E-6 | 3 |
|  |  | RAFL04-15-M13 | At5g56030 / heat shock protein 81-2 (HSP81-2) | |  |  |  |  |  | | --- | --- | --- | --- | --- | |  |  |  |  |  | | C47E8.5 | | | | | | |
|  |  | RAFL07-13-H08 | At5g56010 / heat shock protein, putative | |  |  |  |  |  | | --- | --- | --- | --- | --- | |  |  |  |  |  | | C47E8.5 | | | | | | |
|  |  | RAFL09-06-O18 | At5g56030 / heat shock protein 81-2 (HSP81-2) | |  |  |  |  |  | | --- | --- | --- | --- | --- | |  |  |  |  |  | | C47E8.5 | | | | | | |
|  |  | RAFL05-16-L15 | At5g56010 / heat shock protein, putative | |  |  |  |  |  | | --- | --- | --- | --- | --- | |  |  |  |  |  | | C47E8.5 | | | | | | |
|  | B0464.5 | | WI5\_id:B0464.5 |  | 4 | 73 | 2 | 4584 | 1.0060772E-6 | 3.0182316E-6 | 3 |
|  |  | RAFL04-15-M13 | At5g56030 / heat shock protein 81-2 (HSP81-2) | |  |  |  |  |  | | --- | --- | --- | --- | --- | |  |  |  |  |  | | C47E8.5 | | | | | | |
|  |  | RAFL07-13-H08 | At5g56010 / heat shock protein, putative | |  |  |  |  |  | | --- | --- | --- | --- | --- | |  |  |  |  |  | | C47E8.5 | | | | | | |
|  |  | RAFL09-06-O18 | At5g56030 / heat shock protein 81-2 (HSP81-2) | |  |  |  |  |  | | --- | --- | --- | --- | --- | |  |  |  |  |  | | C47E8.5 | | | | | | |
|  |  | RAFL05-16-L15 | At5g56010 / heat shock protein, putative | |  |  |  |  |  | | --- | --- | --- | --- | --- | |  |  |  |  |  | | C47E8.5 | | | | | | |
|  | K06B4.1 | | WI5\_id:K06B4.1 |  | 4 | 73 | 3 | 4583 | 2.3181085E-6 | 9.272434E-6 | 4 |
|  |  | RAFL04-15-M13 | At5g56030 / heat shock protein 81-2 (HSP81-2) | |  |  |  |  |  | | --- | --- | --- | --- | --- | |  |  |  |  |  | | C47E8.5 | | | | | | |
|  |  | RAFL07-13-H08 | At5g56010 / heat shock protein, putative | |  |  |  |  |  | | --- | --- | --- | --- | --- | |  |  |  |  |  | | C47E8.5 | | | | | | |
|  |  | RAFL09-06-O18 | At5g56030 / heat shock protein 81-2 (HSP81-2) | |  |  |  |  |  | | --- | --- | --- | --- | --- | |  |  |  |  |  | | C47E8.5 | | | | | | |
|  |  | RAFL05-16-L15 | At5g56010 / heat shock protein, putative | |  |  |  |  |  | | --- | --- | --- | --- | --- | |  |  |  |  |  | | C47E8.5 | | | | | | |
|  | Y63D3A.4 | | WI5\_id:Y63D3A.4 |  | 4 | 73 | 3 | 4583 | 2.3181085E-6 | 9.272434E-6 | 4 |
|  |  | RAFL04-15-M13 | At5g56030 / heat shock protein 81-2 (HSP81-2) | |  |  |  |  |  | | --- | --- | --- | --- | --- | |  |  |  |  |  | | C47E8.5 | | | | | | |
|  |  | RAFL07-13-H08 | At5g56010 / heat shock protein, putative | |  |  |  |  |  | | --- | --- | --- | --- | --- | |  |  |  |  |  | | C47E8.5 | | | | | | |
|  |  | RAFL09-06-O18 | At5g56030 / heat shock protein 81-2 (HSP81-2) | |  |  |  |  |  | | --- | --- | --- | --- | --- | |  |  |  |  |  | | C47E8.5 | | | | | | |
|  |  | RAFL05-16-L15 | At5g56010 / heat shock protein, putative | |  |  |  |  |  | | --- | --- | --- | --- | --- | |  |  |  |  |  | | C47E8.5 | | | | | | |
|  | R12B2.1 | | WI5\_id:R12B2.1 |  | 4 | 73 | 6 | 4580 | 1.3392871E-5 | 8.0357226E-5 | 6 |
|  |  | RAFL04-15-M13 | At5g56030 / heat shock protein 81-2 (HSP81-2) | |  |  |  |  |  | | --- | --- | --- | --- | --- | |  |  |  |  |  | | C47E8.5 | | | | | | |
|  |  | RAFL07-13-H08 | At5g56010 / heat shock protein, putative | |  |  |  |  |  | | --- | --- | --- | --- | --- | |  |  |  |  |  | | C47E8.5 | | | | | | |
|  |  | RAFL09-06-O18 | At5g56030 / heat shock protein 81-2 (HSP81-2) | |  |  |  |  |  | | --- | --- | --- | --- | --- | |  |  |  |  |  | | C47E8.5 | | | | | | |
|  |  | RAFL05-16-L15 | At5g56010 / heat shock protein, putative | |  |  |  |  |  | | --- | --- | --- | --- | --- | |  |  |  |  |  | | C47E8.5 | | | | | | |
|  | T11B7.4 | | WI5\_id:T11B7.4 |  | 4 | 73 | 7 | 4579 | 2.0782694E-5 | 1.4547886E-4 | 7 |
|  |  | RAFL04-15-M13 | At5g56030 / heat shock protein 81-2 (HSP81-2) | |  |  |  |  |  | | --- | --- | --- | --- | --- | |  |  |  |  |  | | C47E8.5 | | | | | | |
|  |  | RAFL07-13-H08 | At5g56010 / heat shock protein, putative | |  |  |  |  |  | | --- | --- | --- | --- | --- | |  |  |  |  |  | | C47E8.5 | | | | | | |
|  |  | RAFL09-06-O18 | At5g56030 / heat shock protein 81-2 (HSP81-2) | |  |  |  |  |  | | --- | --- | --- | --- | --- | |  |  |  |  |  | | C47E8.5 | | | | | | |
|  |  | RAFL05-16-L15 | At5g56010 / heat shock protein, putative | |  |  |  |  |  | | --- | --- | --- | --- | --- | |  |  |  |  |  | | C47E8.5 | | | | | | |
|  | C47E8.5 | | WI5\_id:C47E8.5 |  | 4 | 73 | 7 | 4579 | 2.0782694E-5 | 1.4547886E-4 | 7 |
|  |  | RAFL04-15-M13 | At5g56030 / heat shock protein 81-2 (HSP81-2) | |  |  |  |  |  | | --- | --- | --- | --- | --- | |  |  |  |  |  | | C47E8.5 | | | | | | |
|  |  | RAFL07-13-H08 | At5g56010 / heat shock protein, putative | |  |  |  |  |  | | --- | --- | --- | --- | --- | |  |  |  |  |  | | C47E8.5 | | | | | | |
|  |  | RAFL09-06-O18 | At5g56030 / heat shock protein 81-2 (HSP81-2) | |  |  |  |  |  | | --- | --- | --- | --- | --- | |  |  |  |  |  | | C47E8.5 | | | | | | |
|  |  | RAFL05-16-L15 | At5g56010 / heat shock protein, putative | |  |  |  |  |  | | --- | --- | --- | --- | --- | |  |  |  |  |  | | C47E8.5 | | | | | | |
|  | F53A3.3 | | WI5\_id:F53A3.3 |  | 4 | 73 | 11 | 4575 | 8.174753E-5 | 4.904852E-4 | 6 |
|  |  | RAFL04-15-M13 | At5g56030 / heat shock protein 81-2 (HSP81-2) | |  |  |  |  |  | | --- | --- | --- | --- | --- | |  |  |  |  |  | | C47E8.5 | | | | | | |
|  |  | RAFL07-13-H08 | At5g56010 / heat shock protein, putative | |  |  |  |  |  | | --- | --- | --- | --- | --- | |  |  |  |  |  | | C47E8.5 | | | | | | |
|  |  | RAFL09-06-O18 | At5g56030 / heat shock protein 81-2 (HSP81-2) | |  |  |  |  |  | | --- | --- | --- | --- | --- | |  |  |  |  |  | | C47E8.5 | | | | | | |
|  |  | RAFL05-16-L15 | At5g56010 / heat shock protein, putative | |  |  |  |  |  | | --- | --- | --- | --- | --- | |  |  |  |  |  | | C47E8.5 | | | | | | |
|  | T22H2.5 | | WI5\_id:T22H2.5 |  | 1 | 76 | 0 | 4586 | 0.016512975 | 0.016512975 | 1 |
|  |  | RAFL04-09-J19 | At1g56450 / 20S proteasome beta subunit G1 (PBG1) | |  |  |  |  |  | | --- | --- | --- | --- | --- | |  |  |  |  |  | | F39H11.5  Yeast NIP80 protein like | | | | | | |
|  | T10F2.4 | | WI5\_id:T10F2.4 |  | 1 | 76 | 0 | 4586 | 0.016512975 | 0.016512975 | 1 |
|  |  | RAFL04-09-J19 | At1g56450 / 20S proteasome beta subunit G1 (PBG1) | |  |  |  |  |  | | --- | --- | --- | --- | --- | |  |  |  |  |  | | F39H11.5 | | | | | | |
|  | T10E10.4 | | WI5\_id:T10E10.4 |  | 1 | 76 | 0 | 4586 | 0.016512975 | 0.016512975 | 1 |
|  |  | RAFL04-09-J19 | At1g56450 / 20S proteasome beta subunit G1 (PBG1) | |  |  |  |  |  | | --- | --- | --- | --- | --- | |  |  |  |  |  | | F39H11.5 | | | | | | |
|  | C39E9.14 | | WI5\_id:C39E9.14 |  | 1 | 76 | 0 | 4586 | 0.016512975 | 0.016512975 | 1 |
|  |  | RAFL04-09-J19 | At1g56450 / 20S proteasome beta subunit G1 (PBG1) | |  |  |  |  |  | | --- | --- | --- | --- | --- | |  |  |  |  |  | | F39H11.5 | | | | | | |
|  | C06G3.6 | | WI5\_id:C06G3.6 |  | 1 | 76 | 0 | 4586 | 0.016512975 | 0.016512975 | 1 |
|  |  | RAFL06-07-P18 | At3g60820 / 20S proteasome beta subunit F1 (PBF1) | |  |  |  |  |  | | --- | --- | --- | --- | --- | |  |  |  |  |  | | C02F5.9 | | | | | | |
|  | C08E3.9 | | WI5\_id:C08E3.9 |  | 1 | 76 | 0 | 4586 | 0.016512975 | 0.016512975 | 1 |
|  |  | RAFL06-07-P18 | At3g60820 / 20S proteasome beta subunit F1 (PBF1) | |  |  |  |  |  | | --- | --- | --- | --- | --- | |  |  |  |  |  | | C02F5.9 | | | | | | |
|  | D1005.1 | | WI5\_id:D1005.1 |  | 1 | 76 | 0 | 4586 | 0.016512975 | 0.016512975 | 1 |
|  |  | RAFL06-07-P18 | At3g60820 / 20S proteasome beta subunit F1 (PBF1) | |  |  |  |  |  | | --- | --- | --- | --- | --- | |  |  |  |  |  | | C02F5.9 | | | | | | |
|  | R06C1.3 | | WI5\_id:R06C1.3 |  | 1 | 76 | 0 | 4586 | 0.016512975 | 0.016512975 | 1 |
|  |  | RAFL06-07-P18 | At3g60820 / 20S proteasome beta subunit F1 (PBF1) | |  |  |  |  |  | | --- | --- | --- | --- | --- | |  |  |  |  |  | | C02F5.9 | | | | | | |
|  | ZC411.1 | | WI5\_id:ZC411.1 |  | 1 | 76 | 0 | 4586 | 0.016512975 | 0.016512975 | 1 |
|  |  | RAFL06-07-P18 | At3g60820 / 20S proteasome beta subunit F1 (PBF1) | |  |  |  |  |  | | --- | --- | --- | --- | --- | |  |  |  |  |  | | C02F5.9 | | | | | | |
|  | F59A2.4 | | WI5\_id:F59A2.4 |  | 1 | 76 | 0 | 4586 | 0.016512975 | 0.016512975 | 1 |
|  |  | RAFL06-07-P18 | At3g60820 / 20S proteasome beta subunit F1 (PBF1) | |  |  |  |  |  | | --- | --- | --- | --- | --- | |  |  |  |  |  | | C02F5.9 | | | | | | |
|  | F35G12.12 | | WI5\_id:F35G12.12 |  | 1 | 76 | 0 | 4586 | 0.016512975 | 0.016512975 | 1 |
|  |  | RAFL06-08-P09 | At1g53750 / 26S proteasome AAA-ATPase subunit RPT1a | |  |  |  |  |  | | --- | --- | --- | --- | --- | |  |  |  |  |  | | C52E4.4 | | | | | | |
|  | T05B11.1 | | WI5\_id:T05B11.1 |  | 1 | 76 | 0 | 4586 | 0.016512975 | 0.016512975 | 1 |
|  |  | RAFL04-09-J19 | At1g56450 / 20S proteasome beta subunit G1 (PBG1) | |  |  |  |  |  | | --- | --- | --- | --- | --- | |  |  |  |  |  | | F39H11.5 | | | | | | |
|  | ZC504.4 | | WI5\_id:ZC504.4 |  | 1 | 76 | 1 | 4585 | 0.032756753 | 0.06551351 | 2 |
|  |  | RAFL09-16-L12 | At1g62560 / flavin-containing monooxygenase (FMO) family | |  |  |  |  |  | | --- | --- | --- | --- | --- | |  |  |  |  |  | | Y39A1A.19  DIMETHYLANILINE MONOOXYGENASE (N-OXIDE FORMING) 5 (EC 1.14.13.8) (HEPATIC FLAVIN-CONTAINING MONOOXYGENASE 5) (FMO 5) (DIMETHYLANILINE OXIDA .. | | | | | | |
|  | C23G10.8 | | WI5\_id:C23G10.8 |  | 1 | 76 | 1 | 4585 | 0.032756753 | 0.06551351 | 2 |
|  |  | RAFL06-07-P18 | At3g60820 / 20S proteasome beta subunit F1 (PBF1) | |  |  |  |  |  | | --- | --- | --- | --- | --- | |  |  |  |  |  | | C02F5.9 | | | | | | |
|  | Y39A1A.19 | | WI5\_id:Y39A1A.19 |  | 1 | 76 | 1 | 4585 | 0.032756753 | 0.06551351 | 2 |
|  |  | RAFL09-16-L12 | At1g62560 / flavin-containing monooxygenase (FMO) family | |  |  |  |  |  | | --- | --- | --- | --- | --- | |  |  |  |  |  | | Y39A1A.19 | | | | | | |
|  | C02F5.9 | | WI5\_id:C02F5.9 |  | 1 | 76 | 1 | 4585 | 0.032756753 | 0.06551351 | 2 |
|  |  | RAFL06-07-P18 | At3g60820 / 20S proteasome beta subunit F1 (PBF1) | |  |  |  |  |  | | --- | --- | --- | --- | --- | |  |  |  |  |  | | C02F5.9 | | | | | | |
|  | F44G3.9 | | WI5\_id:F44G3.9 |  | 1 | 76 | 2 | 4584 | 0.04873567 | 0.146207 | 3 |
|  |  | RAFL06-07-P18 | At3g60820 / 20S proteasome beta subunit F1 (PBF1) | |  |  |  |  |  | | --- | --- | --- | --- | --- | |  |  |  |  |  | | C02F5.9 | | | | | | |
|  | C06A8.1 | | WI5\_id:C06A8.1 |  | 1 | 76 | 2 | 4584 | 0.04873567 | 0.146207 | 3 |
|  |  | RAFL06-07-P18 | At3g60820 / 20S proteasome beta subunit F1 (PBF1) | |  |  |  |  |  | | --- | --- | --- | --- | --- | |  |  |  |  |  | | C02F5.9 | | | | | | |
|  | Y79H2A.1 | | WI5\_id:Y79H2A.1 |  | 1 | 76 | 2 | 4584 | 0.04873567 | 0.146207 | 3 |
|  |  | RAFL06-07-P18 | At3g60820 / 20S proteasome beta subunit F1 (PBF1) | |  |  |  |  |  | | --- | --- | --- | --- | --- | |  |  |  |  |  | | C02F5.9 | | | | | | |
|  | ZK1098.4 | | WI5\_id:ZK1098.4 |  | 1 | 76 | 2 | 4584 | 0.04873567 | 0.146207 | 3 |
|  |  | RAFL06-07-P18 | At3g60820 / 20S proteasome beta subunit F1 (PBF1) | |  |  |  |  |  | | --- | --- | --- | --- | --- | |  |  |  |  |  | | C02F5.9 | | | | | | |
| Cluster:1-2 | | |  |  | A | B | C | D | P | P' | N |
|  | F01E11.1 | | WI5\_id:F01E11.1 |  | 2 | 172 | 1 | 4488 | 0.0040519224 | 0.008103845 | 2 |
|  |  | RAFL05-04-J09 | At5g45775 / 60S ribosomal protein L11 (RPL11D) | |  |  |  |  |  | | --- | --- | --- | --- | --- | |  |  |  |  |  | | F07D10.1 | | | | | | |
|  |  | RAFL11-10-E06 | At5g45775 / 60S ribosomal protein L11 (RPL11D) | |  |  |  |  |  | | --- | --- | --- | --- | --- | |  |  |  |  |  | | F07D10.1 | | | | | | |
|  | F07D10.1 | | WI5\_id:F07D10.1 |  | 2 | 172 | 1 | 4488 | 0.0040519224 | 0.008103845 | 2 |
|  |  | RAFL05-04-J09 | At5g45775 / 60S ribosomal protein L11 (RPL11D) | |  |  |  |  |  | | --- | --- | --- | --- | --- | |  |  |  |  |  | | F07D10.1 | | | | | | |
|  |  | RAFL11-10-E06 | At5g45775 / 60S ribosomal protein L11 (RPL11D) | |  |  |  |  |  | | --- | --- | --- | --- | --- | |  |  |  |  |  | | F07D10.1 | | | | | | |
|  | M02A10.3 | | WI5\_id:M02A10.3 |  | 2 | 172 | 2 | 4487 | 0.007905076 | 0.015810153 | 2 |
|  |  | RAFL05-04-J09 | At5g45775 / 60S ribosomal protein L11 (RPL11D) | |  |  |  |  |  | | --- | --- | --- | --- | --- | |  |  |  |  |  | | F07D10.1  ribosomal protein | | | | | | |
|  |  | RAFL11-10-E06 | At5g45775 / 60S ribosomal protein L11 (RPL11D) | |  |  |  |  |  | | --- | --- | --- | --- | --- | |  |  |  |  |  | | F07D10.1  ribosomal protein | | | | | | |
|  | T17H7.4 | | WI5\_id:T17H7.4 |  | 2 | 172 | 2 | 4487 | 0.007905076 | 0.023715228 | 3 |
|  |  | RAFL05-04-J09 | At5g45775 / 60S ribosomal protein L11 (RPL11D) | |  |  |  |  |  | | --- | --- | --- | --- | --- | |  |  |  |  |  | | F07D10.1 | | | | | | |
|  |  | RAFL11-10-E06 | At5g45775 / 60S ribosomal protein L11 (RPL11D) | |  |  |  |  |  | | --- | --- | --- | --- | --- | |  |  |  |  |  | | F07D10.1 | | | | | | |
|  | C14B9.7 | | WI5\_id:C14B9.7 |  | 1 | 173 | 0 | 4489 | 0.037315033 | 0.037315033 | 1 |
|  |  | RAFL06-11-J01 | At1g09690 / 60S ribosomal protein L21 (RPL21C) | |  |  |  |  |  | | --- | --- | --- | --- | --- | |  |  |  |  |  | | C14B9.7 | | | | | | |
|  | F33H1.2 | | WI5\_id:F33H1.2 |  | 1 | 173 | 0 | 4489 | 0.037315033 | 0.037315033 | 1 |
|  |  | RAFL06-13-M02 | At1g13440 / glyceraldehyde-3-phosphate dehydrogenase -related | |  |  |  |  |  | | --- | --- | --- | --- | --- | |  |  |  |  |  | | F33H1.2 | | | | | | |
|  | F22B7.13 | | WI5\_id:F22B7.13 |  | 1 | 173 | 0 | 4489 | 0.037315033 | 0.037315033 | 1 |
|  |  | RAFL06-13-M02 | At1g13440 / glyceraldehyde-3-phosphate dehydrogenase -related | |  |  |  |  |  | | --- | --- | --- | --- | --- | |  |  |  |  |  | | F33H1.2  glyceraldehyde 3-phosphate dehydrogenase 4 | | | | | | |
|  | T13F2.8 | | WI5\_id:T13F2.8 |  | 1 | 173 | 0 | 4489 | 0.037315033 | 0.037315033 | 1 |
|  |  | RAFL11-02-K03 | At5g18230 / expressed protein | |  |  |  |  |  | | --- | --- | --- | --- | --- | |  |  |  |  |  | | Y56A3A.1 | | | | | | |
|  | K06H7.6 | | WI5\_id:K06H7.6 |  | 1 | 173 | 0 | 4489 | 0.037315033 | 0.037315033 | 1 |
|  |  | RAFL06-11-J01 | At1g09690 / 60S ribosomal protein L21 (RPL21C) | |  |  |  |  |  | | --- | --- | --- | --- | --- | |  |  |  |  |  | | C14B9.7 | | | | | | |
|  | F57B9.2 | | WI5\_id:F57B9.2 |  | 1 | 173 | 0 | 4489 | 0.037315033 | 0.037315033 | 1 |
|  |  | RAFL11-02-K03 | At5g18230 / expressed protein | |  |  |  |  |  | | --- | --- | --- | --- | --- | |  |  |  |  |  | | Y56A3A.1 | | | | | | |
|  | Y56A3A.1 | | WI5\_id:Y56A3A.1 |  | 1 | 173 | 0 | 4489 | 0.037315033 | 0.037315033 | 1 |
|  |  | RAFL11-02-K03 | At5g18230 / expressed protein | |  |  |  |  |  | | --- | --- | --- | --- | --- | |  |  |  |  |  | | Y56A3A.1 | | | | | | |
|  | R06F6.8 | | WI5\_id:R06F6.8 |  | 1 | 173 | 0 | 4489 | 0.037315033 | 0.037315033 | 1 |
|  |  | RAFL06-11-J01 | At1g09690 / 60S ribosomal protein L21 (RPL21C) | |  |  |  |  |  | | --- | --- | --- | --- | --- | |  |  |  |  |  | | C14B9.7  Ribosomal protein L21 | | | | | | |
| Cluster:3-0 | | |  |  | A | B | C | D | P | P' | N |
|  | F13B10.2 | | WI5\_id:F13B10.2 |  | 5 | 228 | 14 | 4416 | 0.0019486465 | 0.011691879 | 6 |
|  |  | RAFL07-10-D02 | At1g14320 / 60S ribosomal protein L10 (RPL10A)/Wilm's tumor suppressor protein-related | |  |  |  |  |  | | --- | --- | --- | --- | --- | |  |  |  |  |  | | F10B5.1 | | | | | | |
|  |  | RAFL09-10-P09 | At4g36130 / 60S ribosomal protein L8 (RPL8C) | |  |  |  |  |  | | --- | --- | --- | --- | --- | |  |  |  |  |  | | B0250.1 | | | | | | |
|  |  | RAFL08-10-G08 | At3g09630 / 60S ribosomal protein L4/L1 (RPL4A) | |  |  |  |  |  | | --- | --- | --- | --- | --- | |  |  |  |  |  | | B0041.4 | | | | | | |
|  |  | RAFL08-13-M06 | At3g11250 / 60S acidic ribosomal protein P0 (RPP0C) | |  |  |  |  |  | | --- | --- | --- | --- | --- | |  |  |  |  |  | | F25H2.10 | | | | | | |
|  |  | RAFL09-12-B12 | At4g36130 / 60S ribosomal protein L8 (RPL8C) | |  |  |  |  |  | | --- | --- | --- | --- | --- | |  |  |  |  |  | | B0250.1 | | | | | | |
|  | F10B5.1 | | WI5\_id:F10B5.1 |  | 5 | 228 | 14 | 4416 | 0.0019486465 | 0.011691879 | 6 |
|  |  | RAFL07-10-D02 | At1g14320 / 60S ribosomal protein L10 (RPL10A)/Wilm's tumor suppressor protein-related | |  |  |  |  |  | | --- | --- | --- | --- | --- | |  |  |  |  |  | | F10B5.1 | | | | | | |
|  |  | RAFL09-10-P09 | At4g36130 / 60S ribosomal protein L8 (RPL8C) | |  |  |  |  |  | | --- | --- | --- | --- | --- | |  |  |  |  |  | | B0250.1 | | | | | | |
|  |  | RAFL08-10-G08 | At3g09630 / 60S ribosomal protein L4/L1 (RPL4A) | |  |  |  |  |  | | --- | --- | --- | --- | --- | |  |  |  |  |  | | B0041.4 | | | | | | |
|  |  | RAFL08-13-M06 | At3g11250 / 60S acidic ribosomal protein P0 (RPP0C) | |  |  |  |  |  | | --- | --- | --- | --- | --- | |  |  |  |  |  | | F25H2.10 | | | | | | |
|  |  | RAFL09-12-B12 | At4g36130 / 60S ribosomal protein L8 (RPL8C) | |  |  |  |  |  | | --- | --- | --- | --- | --- | |  |  |  |  |  | | B0250.1 | | | | | | |
|  | B0250.1 | | WI5\_id:B0250.1 |  | 5 | 228 | 14 | 4416 | 0.0019486465 | 0.011691879 | 6 |
|  |  | RAFL07-10-D02 | At1g14320 / 60S ribosomal protein L10 (RPL10A)/Wilm's tumor suppressor protein-related | |  |  |  |  |  | | --- | --- | --- | --- | --- | |  |  |  |  |  | | F10B5.1 | | | | | | |
|  |  | RAFL09-10-P09 | At4g36130 / 60S ribosomal protein L8 (RPL8C) | |  |  |  |  |  | | --- | --- | --- | --- | --- | |  |  |  |  |  | | B0250.1 | | | | | | |
|  |  | RAFL08-10-G08 | At3g09630 / 60S ribosomal protein L4/L1 (RPL4A) | |  |  |  |  |  | | --- | --- | --- | --- | --- | |  |  |  |  |  | | B0041.4 | | | | | | |
|  |  | RAFL08-13-M06 | At3g11250 / 60S acidic ribosomal protein P0 (RPP0C) | |  |  |  |  |  | | --- | --- | --- | --- | --- | |  |  |  |  |  | | F25H2.10 | | | | | | |
|  |  | RAFL09-12-B12 | At4g36130 / 60S ribosomal protein L8 (RPL8C) | |  |  |  |  |  | | --- | --- | --- | --- | --- | |  |  |  |  |  | | B0250.1 | | | | | | |
|  | F28C6.7 | | WI5\_id:F28C6.7 |  | 5 | 228 | 14 | 4416 | 0.0019486465 | 0.011691879 | 6 |
|  |  | RAFL07-10-D02 | At1g14320 / 60S ribosomal protein L10 (RPL10A)/Wilm's tumor suppressor protein-related | |  |  |  |  |  | | --- | --- | --- | --- | --- | |  |  |  |  |  | | F10B5.1 | | | | | | |
|  |  | RAFL09-10-P09 | At4g36130 / 60S ribosomal protein L8 (RPL8C) | |  |  |  |  |  | | --- | --- | --- | --- | --- | |  |  |  |  |  | | B0250.1 | | | | | | |
|  |  | RAFL08-10-G08 | At3g09630 / 60S ribosomal protein L4/L1 (RPL4A) | |  |  |  |  |  | | --- | --- | --- | --- | --- | |  |  |  |  |  | | B0041.4 | | | | | | |
|  |  | RAFL08-13-M06 | At3g11250 / 60S acidic ribosomal protein P0 (RPP0C) | |  |  |  |  |  | | --- | --- | --- | --- | --- | |  |  |  |  |  | | F25H2.10 | | | | | | |
|  |  | RAFL09-12-B12 | At4g36130 / 60S ribosomal protein L8 (RPL8C) | |  |  |  |  |  | | --- | --- | --- | --- | --- | |  |  |  |  |  | | B0250.1 | | | | | | |
|  | Y37E3.8 | | WI5\_id:Y37E3.8 |  | 5 | 228 | 14 | 4416 | 0.0019486465 | 0.011691879 | 6 |
|  |  | RAFL07-10-D02 | At1g14320 / 60S ribosomal protein L10 (RPL10A)/Wilm's tumor suppressor protein-related | |  |  |  |  |  | | --- | --- | --- | --- | --- | |  |  |  |  |  | | F10B5.1 | | | | | | |
|  |  | RAFL09-10-P09 | At4g36130 / 60S ribosomal protein L8 (RPL8C) | |  |  |  |  |  | | --- | --- | --- | --- | --- | |  |  |  |  |  | | B0250.1 | | | | | | |
|  |  | RAFL08-10-G08 | At3g09630 / 60S ribosomal protein L4/L1 (RPL4A) | |  |  |  |  |  | | --- | --- | --- | --- | --- | |  |  |  |  |  | | B0041.4 | | | | | | |
|  |  | RAFL08-13-M06 | At3g11250 / 60S acidic ribosomal protein P0 (RPP0C) | |  |  |  |  |  | | --- | --- | --- | --- | --- | |  |  |  |  |  | | F25H2.10 | | | | | | |
|  |  | RAFL09-12-B12 | At4g36130 / 60S ribosomal protein L8 (RPL8C) | |  |  |  |  |  | | --- | --- | --- | --- | --- | |  |  |  |  |  | | B0250.1 | | | | | | |
|  | ZK652.4 | | WI5\_id:ZK652.4 |  | 5 | 228 | 14 | 4416 | 0.0019486465 | 0.011691879 | 6 |
|  |  | RAFL07-10-D02 | At1g14320 / 60S ribosomal protein L10 (RPL10A)/Wilm's tumor suppressor protein-related | |  |  |  |  |  | | --- | --- | --- | --- | --- | |  |  |  |  |  | | F10B5.1 | | | | | | |
|  |  | RAFL09-10-P09 | At4g36130 / 60S ribosomal protein L8 (RPL8C) | |  |  |  |  |  | | --- | --- | --- | --- | --- | |  |  |  |  |  | | B0250.1 | | | | | | |
|  |  | RAFL08-10-G08 | At3g09630 / 60S ribosomal protein L4/L1 (RPL4A) | |  |  |  |  |  | | --- | --- | --- | --- | --- | |  |  |  |  |  | | B0041.4 | | | | | | |
|  |  | RAFL08-13-M06 | At3g11250 / 60S acidic ribosomal protein P0 (RPP0C) | |  |  |  |  |  | | --- | --- | --- | --- | --- | |  |  |  |  |  | | F25H2.10 | | | | | | |
|  |  | RAFL09-12-B12 | At4g36130 / 60S ribosomal protein L8 (RPL8C) | |  |  |  |  |  | | --- | --- | --- | --- | --- | |  |  |  |  |  | | B0250.1 | | | | | | |
|  | F25H2.10 | | WI5\_id:F25H2.10 |  | 4 | 229 | 8 | 4422 | 0.002188199 | 0.013129194 | 6 |
|  |  | RAFL07-10-D02 | At1g14320 / 60S ribosomal protein L10 (RPL10A)/Wilm's tumor suppressor protein-related | |  |  |  |  |  | | --- | --- | --- | --- | --- | |  |  |  |  |  | | F10B5.1 | | | | | | |
|  |  | RAFL09-10-P09 | At4g36130 / 60S ribosomal protein L8 (RPL8C) | |  |  |  |  |  | | --- | --- | --- | --- | --- | |  |  |  |  |  | | B0250.1 | | | | | | |
|  |  | RAFL08-13-M06 | At3g11250 / 60S acidic ribosomal protein P0 (RPP0C) | |  |  |  |  |  | | --- | --- | --- | --- | --- | |  |  |  |  |  | | F25H2.10 | | | | | | |
|  |  | RAFL09-12-B12 | At4g36130 / 60S ribosomal protein L8 (RPL8C) | |  |  |  |  |  | | --- | --- | --- | --- | --- | |  |  |  |  |  | | B0250.1 | | | | | | |
|  | Y59H11AM.3 | | WI5\_id:Y59H11AM.3 |  | 3 | 230 | 3 | 4427 | 0.0022016333 | 0.0066048997 | 3 |
|  |  | RAFL04-13-C01 | At1g56070 / elongation factor -related | |  |  |  |  |  | | --- | --- | --- | --- | --- | |  |  |  |  |  | | F25H5.4 | | | | | | |
|  |  | RAFL08-12-J19 | At1g56070 / elongation factor -related | |  |  |  |  |  | | --- | --- | --- | --- | --- | |  |  |  |  |  | | F25H5.4 | | | | | | |
|  |  | RAFL07-10-D07 | At1g56070 / elongation factor -related | |  |  |  |  |  | | --- | --- | --- | --- | --- | |  |  |  |  |  | | F25H5.4 | | | | | | |
|  | F39B2.2 | | WI5\_id:F39B2.2 |  | 3 | 230 | 3 | 4427 | 0.0022016333 | 0.0066048997 | 3 |
|  |  | RAFL04-13-C01 | At1g56070 / elongation factor -related | |  |  |  |  |  | | --- | --- | --- | --- | --- | |  |  |  |  |  | | F25H5.4  Elongation factor Tu family (contains ATP/GTP binding P-loop) | | | | | | |
|  |  | RAFL08-12-J19 | At1g56070 / elongation factor -related | |  |  |  |  |  | | --- | --- | --- | --- | --- | |  |  |  |  |  | | F25H5.4  Elongation factor Tu family (contains ATP/GTP binding P-loop) | | | | | | |
|  |  | RAFL07-10-D07 | At1g56070 / elongation factor -related | |  |  |  |  |  | | --- | --- | --- | --- | --- | |  |  |  |  |  | | F25H5.4  Elongation factor Tu family (contains ATP/GTP binding P-loop) | | | | | | |
|  | F25H5.4 | | WI5\_id:F25H5.4 |  | 3 | 230 | 3 | 4427 | 0.0022016333 | 0.0066048997 | 3 |
|  |  | RAFL04-13-C01 | At1g56070 / elongation factor -related | |  |  |  |  |  | | --- | --- | --- | --- | --- | |  |  |  |  |  | | F25H5.4 | | | | | | |
|  |  | RAFL08-12-J19 | At1g56070 / elongation factor -related | |  |  |  |  |  | | --- | --- | --- | --- | --- | |  |  |  |  |  | | F25H5.4 | | | | | | |
|  |  | RAFL07-10-D07 | At1g56070 / elongation factor -related | |  |  |  |  |  | | --- | --- | --- | --- | --- | |  |  |  |  |  | | F25H5.4 | | | | | | |
|  | B0412.4 | | WI5\_id:B0412.4 |  | 3 | 230 | 3 | 4427 | 0.0022016333 | 0.0044032666 | 2 |
|  |  | RAFL02-10-H10 | At3g43980 / 40S ribosomal protein S29 (RPS29A) | |  |  |  |  |  | | --- | --- | --- | --- | --- | |  |  |  |  |  | | B0412.4  40S ribosomal protein S29 | | | | | | |
|  |  | RAFL08-09-E20 | At2g41840 / 40S ribosomal protein S2 (RPS2C) | |  |  |  |  |  | | --- | --- | --- | --- | --- | |  |  |  |  |  | | C49H3.11 | | | | | | |
|  |  | RAFL06-07-B02 | At3g11940 / 40S ribosomal protein S5 (RPS5B) | |  |  |  |  |  | | --- | --- | --- | --- | --- | |  |  |  |  |  | | T05E11.1  40S ribosomal protein S5 | | | | | | |
|  | W09H1.6 | | WI5\_id:W09H1.6 |  | 2 | 231 | 0 | 4430 | 0.0024866017 | 0.0024866017 | 1 |
|  |  | RAFL09-10-P09 | At4g36130 / 60S ribosomal protein L8 (RPL8C) | |  |  |  |  |  | | --- | --- | --- | --- | --- | |  |  |  |  |  | | B0250.1 | | | | | | |
|  |  | RAFL09-12-B12 | At4g36130 / 60S ribosomal protein L8 (RPL8C) | |  |  |  |  |  | | --- | --- | --- | --- | --- | |  |  |  |  |  | | B0250.1 | | | | | | |
|  | C44H4.5 | | WI5\_id:C44H4.5 |  | 2 | 231 | 0 | 4430 | 0.0024866017 | 0.0024866017 | 1 |
|  |  | RAFL09-10-P09 | At4g36130 / 60S ribosomal protein L8 (RPL8C) | |  |  |  |  |  | | --- | --- | --- | --- | --- | |  |  |  |  |  | | B0250.1 | | | | | | |
|  |  | RAFL09-12-B12 | At4g36130 / 60S ribosomal protein L8 (RPL8C) | |  |  |  |  |  | | --- | --- | --- | --- | --- | |  |  |  |  |  | | B0250.1 | | | | | | |
|  | F52B5.6 | | WI5\_id:F52B5.6 |  | 5 | 228 | 15 | 4415 | 0.0024938595 | 0.0149631575 | 6 |
|  |  | RAFL07-10-D02 | At1g14320 / 60S ribosomal protein L10 (RPL10A)/Wilm's tumor suppressor protein-related | |  |  |  |  |  | | --- | --- | --- | --- | --- | |  |  |  |  |  | | F10B5.1 | | | | | | |
|  |  | RAFL09-10-P09 | At4g36130 / 60S ribosomal protein L8 (RPL8C) | |  |  |  |  |  | | --- | --- | --- | --- | --- | |  |  |  |  |  | | B0250.1 | | | | | | |
|  |  | RAFL08-10-G08 | At3g09630 / 60S ribosomal protein L4/L1 (RPL4A) | |  |  |  |  |  | | --- | --- | --- | --- | --- | |  |  |  |  |  | | B0041.4 | | | | | | |
|  |  | RAFL08-13-M06 | At3g11250 / 60S acidic ribosomal protein P0 (RPP0C) | |  |  |  |  |  | | --- | --- | --- | --- | --- | |  |  |  |  |  | | F25H2.10 | | | | | | |
|  |  | RAFL09-12-B12 | At4g36130 / 60S ribosomal protein L8 (RPL8C) | |  |  |  |  |  | | --- | --- | --- | --- | --- | |  |  |  |  |  | | B0250.1 | | | | | | |
|  | B0041.4 | | WI5\_id:B0041.4 |  | 4 | 229 | 9 | 4421 | 0.0030380234 | 0.01822814 | 6 |
|  |  | RAFL07-10-D02 | At1g14320 / 60S ribosomal protein L10 (RPL10A)/Wilm's tumor suppressor protein-related | |  |  |  |  |  | | --- | --- | --- | --- | --- | |  |  |  |  |  | | F10B5.1  ribosomal protein L10 (QM protein) | | | | | | |
|  |  | RAFL09-10-P09 | At4g36130 / 60S ribosomal protein L8 (RPL8C) | |  |  |  |  |  | | --- | --- | --- | --- | --- | |  |  |  |  |  | | B0250.1  Ribosomal Proteins L2 | | | | | | |
|  |  | RAFL08-10-G08 | At3g09630 / 60S ribosomal protein L4/L1 (RPL4A) | |  |  |  |  |  | | --- | --- | --- | --- | --- | |  |  |  |  |  | | B0041.4  ribosomal protein L1 | | | | | | |
|  |  | RAFL09-12-B12 | At4g36130 / 60S ribosomal protein L8 (RPL8C) | |  |  |  |  |  | | --- | --- | --- | --- | --- | |  |  |  |  |  | | B0250.1  Ribosomal Proteins L2 | | | | | | |
|  | F54C9.5 | | WI5\_id:F54C9.5 |  | 5 | 228 | 16 | 4414 | 0.0031419091 | 0.021993365 | 7 |
|  |  | RAFL07-10-D02 | At1g14320 / 60S ribosomal protein L10 (RPL10A)/Wilm's tumor suppressor protein-related | |  |  |  |  |  | | --- | --- | --- | --- | --- | |  |  |  |  |  | | F10B5.1 | | | | | | |
|  |  | RAFL09-10-P09 | At4g36130 / 60S ribosomal protein L8 (RPL8C) | |  |  |  |  |  | | --- | --- | --- | --- | --- | |  |  |  |  |  | | B0250.1 | | | | | | |
|  |  | RAFL08-10-G08 | At3g09630 / 60S ribosomal protein L4/L1 (RPL4A) | |  |  |  |  |  | | --- | --- | --- | --- | --- | |  |  |  |  |  | | B0041.4 | | | | | | |
|  |  | RAFL08-13-M06 | At3g11250 / 60S acidic ribosomal protein P0 (RPP0C) | |  |  |  |  |  | | --- | --- | --- | --- | --- | |  |  |  |  |  | | F25H2.10 | | | | | | |
|  |  | RAFL09-12-B12 | At4g36130 / 60S ribosomal protein L8 (RPL8C) | |  |  |  |  |  | | --- | --- | --- | --- | --- | |  |  |  |  |  | | B0250.1 | | | | | | |
|  | W06D4.6 | | WI5\_id:W06D4.6 |  | 3 | 230 | 4 | 4426 | 0.0037112702 | 0.014845081 | 4 |
|  |  | RAFL04-13-C01 | At1g56070 / elongation factor -related | |  |  |  |  |  | | --- | --- | --- | --- | --- | |  |  |  |  |  | | F25H5.4 | | | | | | |
|  |  | RAFL08-12-J19 | At1g56070 / elongation factor -related | |  |  |  |  |  | | --- | --- | --- | --- | --- | |  |  |  |  |  | | F25H5.4 | | | | | | |
|  |  | RAFL07-10-D07 | At1g56070 / elongation factor -related | |  |  |  |  |  | | --- | --- | --- | --- | --- | |  |  |  |  |  | | F25H5.4 | | | | | | |
|  | C07H6.5 | | WI5\_id:C07H6.5 |  | 2 | 231 | 1 | 4429 | 0.007213332 | 0.014426664 | 2 |
|  |  | RAFL09-10-P09 | At4g36130 / 60S ribosomal protein L8 (RPL8C) | |  |  |  |  |  | | --- | --- | --- | --- | --- | |  |  |  |  |  | | B0250.1 | | | | | | |
|  |  | RAFL09-12-B12 | At4g36130 / 60S ribosomal protein L8 (RPL8C) | |  |  |  |  |  | | --- | --- | --- | --- | --- | |  |  |  |  |  | | B0250.1 | | | | | | |
|  | T05E11.1 | | WI5\_id:T05E11.1 |  | 4 | 229 | 13 | 4417 | 0.008637219 | 0.043186095 | 5 |
|  |  | RAFL02-10-H10 | At3g43980 / 40S ribosomal protein S29 (RPS29A) | |  |  |  |  |  | | --- | --- | --- | --- | --- | |  |  |  |  |  | | B0412.4 | | | | | | |
|  |  | RAFL08-09-E20 | At2g41840 / 40S ribosomal protein S2 (RPS2C) | |  |  |  |  |  | | --- | --- | --- | --- | --- | |  |  |  |  |  | | C49H3.11 | | | | | | |
|  |  | RAFL11-04-A02 | At1g07770 / 40S ribosomal protein S15A (RPS15aA) | |  |  |  |  |  | | --- | --- | --- | --- | --- | |  |  |  |  |  | | F53A3.3 | | | | | | |
|  |  | RAFL06-07-B02 | At3g11940 / 40S ribosomal protein S5 (RPS5B) | |  |  |  |  |  | | --- | --- | --- | --- | --- | |  |  |  |  |  | | T05E11.1 | | | | | | |
|  | Y105E8A.16 | | WI5\_id:Y105E8A.16 |  | 4 | 229 | 13 | 4417 | 0.008637219 | 0.043186095 | 5 |
|  |  | RAFL02-10-H10 | At3g43980 / 40S ribosomal protein S29 (RPS29A) | |  |  |  |  |  | | --- | --- | --- | --- | --- | |  |  |  |  |  | | B0412.4 | | | | | | |
|  |  | RAFL08-09-E20 | At2g41840 / 40S ribosomal protein S2 (RPS2C) | |  |  |  |  |  | | --- | --- | --- | --- | --- | |  |  |  |  |  | | C49H3.11 | | | | | | |
|  |  | RAFL11-04-A02 | At1g07770 / 40S ribosomal protein S15A (RPS15aA) | |  |  |  |  |  | | --- | --- | --- | --- | --- | |  |  |  |  |  | | F53A3.3 | | | | | | |
|  |  | RAFL06-07-B02 | At3g11940 / 40S ribosomal protein S5 (RPS5B) | |  |  |  |  |  | | --- | --- | --- | --- | --- | |  |  |  |  |  | | T05E11.1 | | | | | | |
|  | C23G10.3 | | WI5\_id:C23G10.3 |  | 4 | 229 | 14 | 4416 | 0.01067757 | 0.053387847 | 5 |
|  |  | RAFL02-10-H10 | At3g43980 / 40S ribosomal protein S29 (RPS29A) | |  |  |  |  |  | | --- | --- | --- | --- | --- | |  |  |  |  |  | | B0412.4 | | | | | | |
|  |  | RAFL08-09-E20 | At2g41840 / 40S ribosomal protein S2 (RPS2C) | |  |  |  |  |  | | --- | --- | --- | --- | --- | |  |  |  |  |  | | C49H3.11 | | | | | | |
|  |  | RAFL11-04-A02 | At1g07770 / 40S ribosomal protein S15A (RPS15aA) | |  |  |  |  |  | | --- | --- | --- | --- | --- | |  |  |  |  |  | | F53A3.3  40S ribosomal protein | | | | | | |
|  |  | RAFL06-07-B02 | At3g11940 / 40S ribosomal protein S5 (RPS5B) | |  |  |  |  |  | | --- | --- | --- | --- | --- | |  |  |  |  |  | | T05E11.1 | | | | | | |
|  | F36A2.6 | | WI5\_id:F36A2.6 |  | 4 | 229 | 14 | 4416 | 0.01067757 | 0.053387847 | 5 |
|  |  | RAFL02-10-H10 | At3g43980 / 40S ribosomal protein S29 (RPS29A) | |  |  |  |  |  | | --- | --- | --- | --- | --- | |  |  |  |  |  | | B0412.4 | | | | | | |
|  |  | RAFL08-09-E20 | At2g41840 / 40S ribosomal protein S2 (RPS2C) | |  |  |  |  |  | | --- | --- | --- | --- | --- | |  |  |  |  |  | | C49H3.11 | | | | | | |
|  |  | RAFL11-04-A02 | At1g07770 / 40S ribosomal protein S15A (RPS15aA) | |  |  |  |  |  | | --- | --- | --- | --- | --- | |  |  |  |  |  | | F53A3.3 | | | | | | |
|  |  | RAFL06-07-B02 | At3g11940 / 40S ribosomal protein S5 (RPS5B) | |  |  |  |  |  | | --- | --- | --- | --- | --- | |  |  |  |  |  | | T05E11.1 | | | | | | |
|  | T22F3.4 | | WI5\_id:T22F3.4 |  | 3 | 230 | 8 | 4422 | 0.015076893 | 0.09046136 | 6 |
|  |  | RAFL07-10-D02 | At1g14320 / 60S ribosomal protein L10 (RPL10A)/Wilm's tumor suppressor protein-related | |  |  |  |  |  | | --- | --- | --- | --- | --- | |  |  |  |  |  | | F10B5.1 | | | | | | |
|  |  | RAFL09-10-P09 | At4g36130 / 60S ribosomal protein L8 (RPL8C) | |  |  |  |  |  | | --- | --- | --- | --- | --- | |  |  |  |  |  | | B0250.1 | | | | | | |
|  |  | RAFL09-12-B12 | At4g36130 / 60S ribosomal protein L8 (RPL8C) | |  |  |  |  |  | | --- | --- | --- | --- | --- | |  |  |  |  |  | | B0250.1 | | | | | | |
|  | JC8.3 | | WI5\_id:JC8.3 |  | 3 | 230 | 8 | 4422 | 0.015076893 | 0.09046136 | 6 |
|  |  | RAFL07-10-D02 | At1g14320 / 60S ribosomal protein L10 (RPL10A)/Wilm's tumor suppressor protein-related | |  |  |  |  |  | | --- | --- | --- | --- | --- | |  |  |  |  |  | | F10B5.1 | | | | | | |
|  |  | RAFL09-10-P09 | At4g36130 / 60S ribosomal protein L8 (RPL8C) | |  |  |  |  |  | | --- | --- | --- | --- | --- | |  |  |  |  |  | | B0250.1 | | | | | | |
|  |  | RAFL09-12-B12 | At4g36130 / 60S ribosomal protein L8 (RPL8C) | |  |  |  |  |  | | --- | --- | --- | --- | --- | |  |  |  |  |  | | B0250.1 | | | | | | |
|  | Y48G8AL.8 | | WI5\_id:Y48G8AL.8 |  | 3 | 230 | 8 | 4422 | 0.015076893 | 0.09046136 | 6 |
|  |  | RAFL07-10-D02 | At1g14320 / 60S ribosomal protein L10 (RPL10A)/Wilm's tumor suppressor protein-related | |  |  |  |  |  | | --- | --- | --- | --- | --- | |  |  |  |  |  | | F10B5.1 | | | | | | |
|  |  | RAFL09-10-P09 | At4g36130 / 60S ribosomal protein L8 (RPL8C) | |  |  |  |  |  | | --- | --- | --- | --- | --- | |  |  |  |  |  | | B0250.1 | | | | | | |
|  |  | RAFL09-12-B12 | At4g36130 / 60S ribosomal protein L8 (RPL8C) | |  |  |  |  |  | | --- | --- | --- | --- | --- | |  |  |  |  |  | | B0250.1 | | | | | | |
|  | C49H3.11 | | WI5\_id:C49H3.11 |  | 4 | 229 | 17 | 4413 | 0.01857225 | 0.1114335 | 6 |
|  |  | RAFL02-10-H10 | At3g43980 / 40S ribosomal protein S29 (RPS29A) | |  |  |  |  |  | | --- | --- | --- | --- | --- | |  |  |  |  |  | | B0412.4 | | | | | | |
|  |  | RAFL08-09-E20 | At2g41840 / 40S ribosomal protein S2 (RPS2C) | |  |  |  |  |  | | --- | --- | --- | --- | --- | |  |  |  |  |  | | C49H3.11 | | | | | | |
|  |  | RAFL11-04-A02 | At1g07770 / 40S ribosomal protein S15A (RPS15aA) | |  |  |  |  |  | | --- | --- | --- | --- | --- | |  |  |  |  |  | | F53A3.3 | | | | | | |
|  |  | RAFL06-07-B02 | At3g11940 / 40S ribosomal protein S5 (RPS5B) | |  |  |  |  |  | | --- | --- | --- | --- | --- | |  |  |  |  |  | | T05E11.1 | | | | | | |
|  | M01F1.2 | | WI5\_id:M01F1.2 |  | 3 | 230 | 9 | 4421 | 0.019373124 | 0.11623875 | 6 |
|  |  | RAFL07-10-D02 | At1g14320 / 60S ribosomal protein L10 (RPL10A)/Wilm's tumor suppressor protein-related | |  |  |  |  |  | | --- | --- | --- | --- | --- | |  |  |  |  |  | | F10B5.1 | | | | | | |
|  |  | RAFL09-10-P09 | At4g36130 / 60S ribosomal protein L8 (RPL8C) | |  |  |  |  |  | | --- | --- | --- | --- | --- | |  |  |  |  |  | | B0250.1 | | | | | | |
|  |  | RAFL09-12-B12 | At4g36130 / 60S ribosomal protein L8 (RPL8C) | |  |  |  |  |  | | --- | --- | --- | --- | --- | |  |  |  |  |  | | B0250.1 | | | | | | |
|  | Y43B11AR.4 | | WI5\_id:Y43B11AR.4 |  | 3 | 230 | 9 | 4421 | 0.019373124 | 0.096865624 | 5 |
|  |  | RAFL02-10-H10 | At3g43980 / 40S ribosomal protein S29 (RPS29A) | |  |  |  |  |  | | --- | --- | --- | --- | --- | |  |  |  |  |  | | B0412.4 | | | | | | |
|  |  | RAFL08-09-E20 | At2g41840 / 40S ribosomal protein S2 (RPS2C) | |  |  |  |  |  | | --- | --- | --- | --- | --- | |  |  |  |  |  | | C49H3.11 | | | | | | |
|  |  | RAFL11-04-A02 | At1g07770 / 40S ribosomal protein S15A (RPS15aA) | |  |  |  |  |  | | --- | --- | --- | --- | --- | |  |  |  |  |  | | F53A3.3 | | | | | | |
|  | R13A5.8 | | WI5\_id:R13A5.8 |  | 3 | 230 | 10 | 4420 | 0.024273654 | 0.14564192 | 6 |
|  |  | RAFL07-10-D02 | At1g14320 / 60S ribosomal protein L10 (RPL10A)/Wilm's tumor suppressor protein-related | |  |  |  |  |  | | --- | --- | --- | --- | --- | |  |  |  |  |  | | F10B5.1 | | | | | | |
|  |  | RAFL09-10-P09 | At4g36130 / 60S ribosomal protein L8 (RPL8C) | |  |  |  |  |  | | --- | --- | --- | --- | --- | |  |  |  |  |  | | B0250.1 | | | | | | |
|  |  | RAFL09-12-B12 | At4g36130 / 60S ribosomal protein L8 (RPL8C) | |  |  |  |  |  | | --- | --- | --- | --- | --- | |  |  |  |  |  | | B0250.1 | | | | | | |
|  | B0336.10 | | WI5\_id:B0336.10 |  | 3 | 230 | 11 | 4419 | 0.029778728 | 0.17867237 | 6 |
|  |  | RAFL07-10-D02 | At1g14320 / 60S ribosomal protein L10 (RPL10A)/Wilm's tumor suppressor protein-related | |  |  |  |  |  | | --- | --- | --- | --- | --- | |  |  |  |  |  | | F10B5.1 | | | | | | |
|  |  | RAFL09-10-P09 | At4g36130 / 60S ribosomal protein L8 (RPL8C) | |  |  |  |  |  | | --- | --- | --- | --- | --- | |  |  |  |  |  | | B0250.1 | | | | | | |
|  |  | RAFL09-12-B12 | At4g36130 / 60S ribosomal protein L8 (RPL8C) | |  |  |  |  |  | | --- | --- | --- | --- | --- | |  |  |  |  |  | | B0250.1 | | | | | | |
|  | Y71F9AL.13 | | WI5\_id:Y71F9AL.13 |  | 3 | 230 | 11 | 4419 | 0.029778728 | 0.23822983 | 8 |
|  |  | RAFL07-10-D02 | At1g14320 / 60S ribosomal protein L10 (RPL10A)/Wilm's tumor suppressor protein-related | |  |  |  |  |  | | --- | --- | --- | --- | --- | |  |  |  |  |  | | F10B5.1 | | | | | | |
|  |  | RAFL09-10-P09 | At4g36130 / 60S ribosomal protein L8 (RPL8C) | |  |  |  |  |  | | --- | --- | --- | --- | --- | |  |  |  |  |  | | B0250.1 | | | | | | |
|  |  | RAFL09-12-B12 | At4g36130 / 60S ribosomal protein L8 (RPL8C) | |  |  |  |  |  | | --- | --- | --- | --- | --- | |  |  |  |  |  | | B0250.1 | | | | | | |
|  | Y57G11C.16 | | WI5\_id:Y57G11C.16 |  | 2 | 231 | 4 | 4426 | 0.032636177 | 0.097908534 | 3 |
|  |  | RAFL08-09-E20 | At2g41840 / 40S ribosomal protein S2 (RPS2C) | |  |  |  |  |  | | --- | --- | --- | --- | --- | |  |  |  |  |  | | C49H3.11 | | | | | | |
|  |  | RAFL06-07-B02 | At3g11940 / 40S ribosomal protein S5 (RPS5B) | |  |  |  |  |  | | --- | --- | --- | --- | --- | |  |  |  |  |  | | T05E11.1 | | | | | | |
|  | F37C12.9 | | WI5\_id:F37C12.9 |  | 2 | 231 | 4 | 4426 | 0.032636177 | 0.06527235 | 2 |
|  |  | RAFL08-09-E20 | At2g41840 / 40S ribosomal protein S2 (RPS2C) | |  |  |  |  |  | | --- | --- | --- | --- | --- | |  |  |  |  |  | | C49H3.11 | | | | | | |
|  |  | RAFL06-07-B02 | At3g11940 / 40S ribosomal protein S5 (RPS5B) | |  |  |  |  |  | | --- | --- | --- | --- | --- | |  |  |  |  |  | | T05E11.1 | | | | | | |
|  | F28D1.7 | | WI5\_id:F28D1.7 |  | 2 | 231 | 4 | 4426 | 0.032636177 | 0.06527235 | 2 |
|  |  | RAFL08-09-E20 | At2g41840 / 40S ribosomal protein S2 (RPS2C) | |  |  |  |  |  | | --- | --- | --- | --- | --- | |  |  |  |  |  | | C49H3.11 | | | | | | |
|  |  | RAFL06-07-B02 | At3g11940 / 40S ribosomal protein S5 (RPS5B) | |  |  |  |  |  | | --- | --- | --- | --- | --- | |  |  |  |  |  | | T05E11.1 | | | | | | |
|  | F53A3.3 | | WI5\_id:F53A3.3 |  | 3 | 230 | 12 | 4418 | 0.03588357 | 0.21530141 | 6 |
|  |  | RAFL08-09-E20 | At2g41840 / 40S ribosomal protein S2 (RPS2C) | |  |  |  |  |  | | --- | --- | --- | --- | --- | |  |  |  |  |  | | C49H3.11 | | | | | | |
|  |  | RAFL11-04-A02 | At1g07770 / 40S ribosomal protein S15A (RPS15aA) | |  |  |  |  |  | | --- | --- | --- | --- | --- | |  |  |  |  |  | | F53A3.3 | | | | | | |
|  |  | RAFL06-07-B02 | At3g11940 / 40S ribosomal protein S5 (RPS5B) | |  |  |  |  |  | | --- | --- | --- | --- | --- | |  |  |  |  |  | | T05E11.1 | | | | | | |
|  | T24B8.1 | | WI5\_id:T24B8.1 |  | 3 | 230 | 14 | 4416 | 0.0498523 | 0.2492615 | 5 |
|  |  | RAFL09-10-P09 | At4g36130 / 60S ribosomal protein L8 (RPL8C) | |  |  |  |  |  | | --- | --- | --- | --- | --- | |  |  |  |  |  | | B0250.1 | | | | | | |
|  |  | RAFL08-10-G08 | At3g09630 / 60S ribosomal protein L4/L1 (RPL4A) | |  |  |  |  |  | | --- | --- | --- | --- | --- | |  |  |  |  |  | | B0041.4 | | | | | | |
|  |  | RAFL09-12-B12 | At4g36130 / 60S ribosomal protein L8 (RPL8C) | |  |  |  |  |  | | --- | --- | --- | --- | --- | |  |  |  |  |  | | B0250.1 | | | | | | |
|  | T04H1.6 | | WI5\_id:T04H1.6 |  | 1 | 232 | 0 | 4430 | 0.049967833 | 0.049967833 | 1 |
|  |  | RAFL05-07-D18 | At5g49510 / von Hippel-Lindau binding protein (VHL binding protein; VBP) like | |  |  |  |  |  | | --- | --- | --- | --- | --- | |  |  |  |  |  | | T06G6.9 | | | | | | |
|  | R151.9 | | WI5\_id:R151.9 |  | 1 | 232 | 0 | 4430 | 0.049967833 | 0.049967833 | 1 |
|  |  | RAFL05-07-D18 | At5g49510 / von Hippel-Lindau binding protein (VHL binding protein; VBP) like | |  |  |  |  |  | | --- | --- | --- | --- | --- | |  |  |  |  |  | | T06G6.9 | | | | | | |
|  | T06E6.10 | | WI5\_id:T06E6.10 |  | 1 | 232 | 0 | 4430 | 0.049967833 | 0.049967833 | 1 |
|  |  | RAFL08-13-M06 | At3g11250 / 60S acidic ribosomal protein P0 (RPP0C) | |  |  |  |  |  | | --- | --- | --- | --- | --- | |  |  |  |  |  | | F25H2.10 | | | | | | |
|  | F21C3.5 | | WI5\_id:F21C3.5 |  | 1 | 232 | 0 | 4430 | 0.049967833 | 0.049967833 | 1 |
|  |  | RAFL05-07-D18 | At5g49510 / von Hippel-Lindau binding protein (VHL binding protein; VBP) like | |  |  |  |  |  | | --- | --- | --- | --- | --- | |  |  |  |  |  | | T06G6.9  Human VHL binding protein like | | | | | | |
|  | H20J04.5 | | WI5\_id:H20J04.5 |  | 1 | 232 | 0 | 4430 | 0.049967833 | 0.049967833 | 1 |
|  |  | RAFL05-07-D18 | At5g49510 / von Hippel-Lindau binding protein (VHL binding protein; VBP) like | |  |  |  |  |  | | --- | --- | --- | --- | --- | |  |  |  |  |  | | T06G6.9 | | | | | | |
|  | T06G6.9 | | WI5\_id:T06G6.9 |  | 1 | 232 | 0 | 4430 | 0.049967833 | 0.049967833 | 1 |
|  |  | RAFL05-07-D18 | At5g49510 / von Hippel-Lindau binding protein (VHL binding protein; VBP) like | |  |  |  |  |  | | --- | --- | --- | --- | --- | |  |  |  |  |  | | T06G6.9 | | | | | | |
|  | C27A2.5 | | WI5\_id:C27A2.5 |  | 1 | 232 | 0 | 4430 | 0.049967833 | 0.049967833 | 1 |
|  |  | RAFL04-14-I15 | At3g02080 / 40S ribosomal protein S19 (RPS19A) | |  |  |  |  |  | | --- | --- | --- | --- | --- | |  |  |  |  |  | | T05F1.3 | | | | | | |
|  | W09D10.3 | | WI5\_id:W09D10.3 |  | 1 | 232 | 0 | 4430 | 0.049967833 | 0.049967833 | 1 |
|  |  | RAFL08-13-M06 | At3g11250 / 60S acidic ribosomal protein P0 (RPP0C) | |  |  |  |  |  | | --- | --- | --- | --- | --- | |  |  |  |  |  | | F25H2.10 | | | | | | |
|  | C48B6.3 | | WI5\_id:C48B6.3 |  | 1 | 232 | 0 | 4430 | 0.049967833 | 0.049967833 | 1 |
|  |  | RAFL04-16-E04 | At3g51260 / 20S proteasome alpha subunit D (PAD1) | |  |  |  |  |  | | --- | --- | --- | --- | --- | |  |  |  |  |  | | C36B1.4 | | | | | | |
|  | C56C10.7 | | WI5\_id:C56C10.7 |  | 1 | 232 | 0 | 4430 | 0.049967833 | 0.049967833 | 1 |
|  |  | RAFL04-16-E04 | At3g51260 / 20S proteasome alpha subunit D (PAD1) | |  |  |  |  |  | | --- | --- | --- | --- | --- | |  |  |  |  |  | | C36B1.4 | | | | | | |
|  | T28B8.5 | | WI5\_id:T28B8.5 |  | 1 | 232 | 0 | 4430 | 0.049967833 | 0.049967833 | 1 |
|  |  | RAFL08-13-M06 | At3g11250 / 60S acidic ribosomal protein P0 (RPP0C) | |  |  |  |  |  | | --- | --- | --- | --- | --- | |  |  |  |  |  | | F25H2.10 | | | | | | |
|  | Y37E3.7 | | WI5\_id:Y37E3.7 |  | 1 | 232 | 0 | 4430 | 0.049967833 | 0.049967833 | 1 |
|  |  | RAFL08-13-M06 | At3g11250 / 60S acidic ribosomal protein P0 (RPP0C) | |  |  |  |  |  | | --- | --- | --- | --- | --- | |  |  |  |  |  | | F25H2.10 | | | | | | |
|  | Y15E3A.1 | | WI5\_id:Y15E3A.1 |  | 1 | 232 | 0 | 4430 | 0.049967833 | 0.049967833 | 1 |
|  |  | RAFL08-13-M06 | At3g11250 / 60S acidic ribosomal protein P0 (RPP0C) | |  |  |  |  |  | | --- | --- | --- | --- | --- | |  |  |  |  |  | | F25H2.10 | | | | | | |
|  | T05F1.3 | | WI5\_id:T05F1.3 |  | 1 | 232 | 0 | 4430 | 0.049967833 | 0.049967833 | 1 |
|  |  | RAFL04-14-I15 | At3g02080 / 40S ribosomal protein S19 (RPS19A) | |  |  |  |  |  | | --- | --- | --- | --- | --- | |  |  |  |  |  | | T05F1.3 | | | | | | |
|  | T09F3.3 | | WI5\_id:T09F3.3 |  | 1 | 232 | 0 | 4430 | 0.049967833 | 0.049967833 | 1 |
|  |  | RAFL06-14-K01 | At3g04120 / glyceraldehyde-3-phosphate dehydrogenase C subunit (GapC) | |  |  |  |  |  | | --- | --- | --- | --- | --- | |  |  |  |  |  | | T09F3.3 | | | | | | |
| Cluster:9-0 | | |  |  | A | B | C | D | P | P' | N |
|  | F25F8.2 | | WI5\_id:F25F8.2 |  | 4 | 28 | 0 | 4631 | 1.8277981E-9 | 1.8277981E-9 | 1 |
|  |  | RAFL08-09-C23 | At1g54100 / aldehyde dehydrogenase, putative (ALDH) | |  |  |  |  |  | | --- | --- | --- | --- | --- | |  |  |  |  |  | | F01F1.6  Aldehyde dehydrogenase | | | | | | |
|  |  | RAFL04-09-D07 | At1g54100 / aldehyde dehydrogenase, putative (ALDH) | |  |  |  |  |  | | --- | --- | --- | --- | --- | |  |  |  |  |  | | F01F1.6  Aldehyde dehydrogenase | | | | | | |
|  |  | RAFL05-21-E06 | At1g54100 / aldehyde dehydrogenase, putative (ALDH) | |  |  |  |  |  | | --- | --- | --- | --- | --- | |  |  |  |  |  | | F01F1.6  Aldehyde dehydrogenase | | | | | | |
|  |  | RAFL08-15-L09 | At1g54100 / aldehyde dehydrogenase, putative (ALDH) | |  |  |  |  |  | | --- | --- | --- | --- | --- | |  |  |  |  |  | | F01F1.6  Aldehyde dehydrogenase | | | | | | |
|  | F01F1.6 | | WI5\_id:F01F1.6 |  | 4 | 28 | 0 | 4631 | 1.8277981E-9 | 1.8277981E-9 | 1 |
|  |  | RAFL08-09-C23 | At1g54100 / aldehyde dehydrogenase, putative (ALDH) | |  |  |  |  |  | | --- | --- | --- | --- | --- | |  |  |  |  |  | | F01F1.6 | | | | | | |
|  |  | RAFL04-09-D07 | At1g54100 / aldehyde dehydrogenase, putative (ALDH) | |  |  |  |  |  | | --- | --- | --- | --- | --- | |  |  |  |  |  | | F01F1.6 | | | | | | |
|  |  | RAFL05-21-E06 | At1g54100 / aldehyde dehydrogenase, putative (ALDH) | |  |  |  |  |  | | --- | --- | --- | --- | --- | |  |  |  |  |  | | F01F1.6 | | | | | | |
|  |  | RAFL08-15-L09 | At1g54100 / aldehyde dehydrogenase, putative (ALDH) | |  |  |  |  |  | | --- | --- | --- | --- | --- | |  |  |  |  |  | | F01F1.6 | | | | | | |
|  | R03G5.2 | | WI5\_id:R03G5.2 |  | 4 | 28 | 1 | 4630 | 9.095051E-9 | 1.8190102E-8 | 2 |
|  |  | RAFL08-09-C23 | At1g54100 / aldehyde dehydrogenase, putative (ALDH) | |  |  |  |  |  | | --- | --- | --- | --- | --- | |  |  |  |  |  | | F01F1.6 | | | | | | |
|  |  | RAFL04-09-D07 | At1g54100 / aldehyde dehydrogenase, putative (ALDH) | |  |  |  |  |  | | --- | --- | --- | --- | --- | |  |  |  |  |  | | F01F1.6 | | | | | | |
|  |  | RAFL05-21-E06 | At1g54100 / aldehyde dehydrogenase, putative (ALDH) | |  |  |  |  |  | | --- | --- | --- | --- | --- | |  |  |  |  |  | | F01F1.6 | | | | | | |
|  |  | RAFL08-15-L09 | At1g54100 / aldehyde dehydrogenase, putative (ALDH) | |  |  |  |  |  | | --- | --- | --- | --- | --- | |  |  |  |  |  | | F01F1.6 | | | | | | |
| Cluster:4-1 | | |  |  | A | B | C | D | P | P' | N |
|  | T20F5.2 | | WI5\_id:T20F5.2 |  | 8 | 302 | 5 | 4348 | 3.3526283E-7 | 1.3410513E-6 | 4 |
|  |  | RAFL04-15-N11 | At3g22110 / 20S proteasome alpha subunit C (PAC1) | |  |  |  |  |  | | --- | --- | --- | --- | --- | |  |  |  |  |  | | Y110A7A.14 | | | | | | |
|  |  | RAFL05-01-C14 | At5g42790 / 20S proteasome alpha subunit F1 (PAF1) | |  |  |  |  |  | | --- | --- | --- | --- | --- | |  |  |  |  |  | | CD4.6 | | | | | | |
|  |  | RAFL07-08-J17 | At1g13060 / 20S proteasome beta subunit E1 (PBE1) | |  |  |  |  |  | | --- | --- | --- | --- | --- | |  |  |  |  |  | | K05C4.1 | | | | | | |
|  |  | RAFL05-04-G23 | At1g77440 / 20S proteasome beta subunit C (PBC2) | |  |  |  |  |  | | --- | --- | --- | --- | --- | |  |  |  |  |  | | Y38A8.2 | | | | | | |
|  |  | RAFL04-12-B09 | At3g14290 / 20S proteasome alpha subunit E2 (PAE2) | |  |  |  |  |  | | --- | --- | --- | --- | --- | |  |  |  |  |  | | F25H2.9 | | | | | | |
|  |  | RAFL05-04-L16 | At1g79210 / 20S proteasome alpha subunit B, putative | |  |  |  |  |  | | --- | --- | --- | --- | --- | |  |  |  |  |  | | D1054.2 | | | | | | |
|  |  | RAFL05-12-G18 | At4g14800 / 20S proteasome beta subunit D2 (PBD2) | |  |  |  |  |  | | --- | --- | --- | --- | --- | |  |  |  |  |  | | T20F5.2 | | | | | | |
|  |  | RAFL05-21-D23 | At1g21720 / 20S proteasome beta subunit C (PBC1) | |  |  |  |  |  | | --- | --- | --- | --- | --- | |  |  |  |  |  | | Y38A8.2 | | | | | | |
|  | C15H11.7 | | WI5\_id:C15H11.7 |  | 7 | 303 | 5 | 4348 | 3.1829013E-6 | 1.2731605E-5 | 4 |
|  |  | RAFL04-15-N11 | At3g22110 / 20S proteasome alpha subunit C (PAC1) | |  |  |  |  |  | | --- | --- | --- | --- | --- | |  |  |  |  |  | | Y110A7A.14  endopeptidase | | | | | | |
|  |  | RAFL05-01-C14 | At5g42790 / 20S proteasome alpha subunit F1 (PAF1) | |  |  |  |  |  | | --- | --- | --- | --- | --- | |  |  |  |  |  | | CD4.6 | | | | | | |
|  |  | RAFL07-08-J17 | At1g13060 / 20S proteasome beta subunit E1 (PBE1) | |  |  |  |  |  | | --- | --- | --- | --- | --- | |  |  |  |  |  | | K05C4.1 | | | | | | |
|  |  | RAFL05-04-G23 | At1g77440 / 20S proteasome beta subunit C (PBC2) | |  |  |  |  |  | | --- | --- | --- | --- | --- | |  |  |  |  |  | | Y38A8.2 | | | | | | |
|  |  | RAFL05-04-L16 | At1g79210 / 20S proteasome alpha subunit B, putative | |  |  |  |  |  | | --- | --- | --- | --- | --- | |  |  |  |  |  | | D1054.2 | | | | | | |
|  |  | RAFL05-12-G18 | At4g14800 / 20S proteasome beta subunit D2 (PBD2) | |  |  |  |  |  | | --- | --- | --- | --- | --- | |  |  |  |  |  | | T20F5.2 | | | | | | |
|  |  | RAFL05-21-D23 | At1g21720 / 20S proteasome beta subunit C (PBC1) | |  |  |  |  |  | | --- | --- | --- | --- | --- | |  |  |  |  |  | | Y38A8.2 | | | | | | |
|  | D1054.2 | | WI5\_id:D1054.2 |  | 7 | 303 | 7 | 4346 | 1.22729925E-5 | 7.363795E-5 | 6 |
|  |  | RAFL04-15-N11 | At3g22110 / 20S proteasome alpha subunit C (PAC1) | |  |  |  |  |  | | --- | --- | --- | --- | --- | |  |  |  |  |  | | Y110A7A.14 | | | | | | |
|  |  | RAFL05-01-C14 | At5g42790 / 20S proteasome alpha subunit F1 (PAF1) | |  |  |  |  |  | | --- | --- | --- | --- | --- | |  |  |  |  |  | | CD4.6 | | | | | | |
|  |  | RAFL07-08-J17 | At1g13060 / 20S proteasome beta subunit E1 (PBE1) | |  |  |  |  |  | | --- | --- | --- | --- | --- | |  |  |  |  |  | | K05C4.1 | | | | | | |
|  |  | RAFL05-04-G23 | At1g77440 / 20S proteasome beta subunit C (PBC2) | |  |  |  |  |  | | --- | --- | --- | --- | --- | |  |  |  |  |  | | Y38A8.2 | | | | | | |
|  |  | RAFL05-04-L16 | At1g79210 / 20S proteasome alpha subunit B, putative | |  |  |  |  |  | | --- | --- | --- | --- | --- | |  |  |  |  |  | | D1054.2 | | | | | | |
|  |  | RAFL05-12-G18 | At4g14800 / 20S proteasome beta subunit D2 (PBD2) | |  |  |  |  |  | | --- | --- | --- | --- | --- | |  |  |  |  |  | | T20F5.2 | | | | | | |
|  |  | RAFL05-21-D23 | At1g21720 / 20S proteasome beta subunit C (PBC1) | |  |  |  |  |  | | --- | --- | --- | --- | --- | |  |  |  |  |  | | Y38A8.2 | | | | | | |
|  | Y38A8.2 | | WI5\_id:Y38A8.2 |  | 4 | 306 | 2 | 4351 | 2.5832583E-4 | 5.1665166E-4 | 2 |
|  |  | RAFL05-04-G23 | At1g77440 / 20S proteasome beta subunit C (PBC2) | |  |  |  |  |  | | --- | --- | --- | --- | --- | |  |  |  |  |  | | Y38A8.2 | | | | | | |
|  |  | RAFL05-04-L16 | At1g79210 / 20S proteasome alpha subunit B, putative | |  |  |  |  |  | | --- | --- | --- | --- | --- | |  |  |  |  |  | | D1054.2 | | | | | | |
|  |  | RAFL05-12-G18 | At4g14800 / 20S proteasome beta subunit D2 (PBD2) | |  |  |  |  |  | | --- | --- | --- | --- | --- | |  |  |  |  |  | | T20F5.2 | | | | | | |
|  |  | RAFL05-21-D23 | At1g21720 / 20S proteasome beta subunit C (PBC1) | |  |  |  |  |  | | --- | --- | --- | --- | --- | |  |  |  |  |  | | Y38A8.2 | | | | | | |
|  | K05C4.1 | | WI5\_id:K05C4.1 |  | 3 | 307 | 1 | 4352 | 0.0011071523 | 0.0022143046 | 2 |
|  |  | RAFL07-08-J17 | At1g13060 / 20S proteasome beta subunit E1 (PBE1) | |  |  |  |  |  | | --- | --- | --- | --- | --- | |  |  |  |  |  | | K05C4.1 | | | | | | |
|  |  | RAFL05-04-L16 | At1g79210 / 20S proteasome alpha subunit B, putative | |  |  |  |  |  | | --- | --- | --- | --- | --- | |  |  |  |  |  | | D1054.2 | | | | | | |
|  |  | RAFL05-12-G18 | At4g14800 / 20S proteasome beta subunit D2 (PBD2) | |  |  |  |  |  | | --- | --- | --- | --- | --- | |  |  |  |  |  | | T20F5.2 | | | | | | |
|  | Y110A7A.14 | | WI5\_id:Y110A7A.14 |  | 3 | 307 | 1 | 4352 | 0.0011071523 | 0.0022143046 | 2 |
|  |  | RAFL04-15-N11 | At3g22110 / 20S proteasome alpha subunit C (PAC1) | |  |  |  |  |  | | --- | --- | --- | --- | --- | |  |  |  |  |  | | Y110A7A.14 | | | | | | |
|  |  | RAFL05-04-L16 | At1g79210 / 20S proteasome alpha subunit B, putative | |  |  |  |  |  | | --- | --- | --- | --- | --- | |  |  |  |  |  | | D1054.2 | | | | | | |
|  |  | RAFL05-12-G18 | At4g14800 / 20S proteasome beta subunit D2 (PBD2) | |  |  |  |  |  | | --- | --- | --- | --- | --- | |  |  |  |  |  | | T20F5.2 | | | | | | |
|  | H28O16.1 | | WI5\_id:H28O16.1 |  | 3 | 307 | 2 | 4351 | 0.0026315711 | 0.007894713 | 3 |
|  |  | RAFL04-12-B09 | At3g14290 / 20S proteasome alpha subunit E2 (PAE2) | |  |  |  |  |  | | --- | --- | --- | --- | --- | |  |  |  |  |  | | F25H2.9 | | | | | | |
|  |  | RAFL04-15-D14 | At5g08680 / H+-transporting ATP synthase beta chain -related | |  |  |  |  |  | | --- | --- | --- | --- | --- | |  |  |  |  |  | | C34E10.6 | | | | | | |
|  |  | RAFL09-17-J21 | At5g08690 / H+-transporting ATP synthase beta chain (mitochondrial) -related | |  |  |  |  |  | | --- | --- | --- | --- | --- | |  |  |  |  |  | | C34E10.6 | | | | | | |
|  | CD4.6 | | WI5\_id:CD4.6 |  | 3 | 307 | 2 | 4351 | 0.0026315711 | 0.0052631423 | 2 |
|  |  | RAFL05-01-C14 | At5g42790 / 20S proteasome alpha subunit F1 (PAF1) | |  |  |  |  |  | | --- | --- | --- | --- | --- | |  |  |  |  |  | | CD4.6 | | | | | | |
|  |  | RAFL05-04-L16 | At1g79210 / 20S proteasome alpha subunit B, putative | |  |  |  |  |  | | --- | --- | --- | --- | --- | |  |  |  |  |  | | D1054.2 | | | | | | |
|  |  | RAFL05-12-G18 | At4g14800 / 20S proteasome beta subunit D2 (PBD2) | |  |  |  |  |  | | --- | --- | --- | --- | --- | |  |  |  |  |  | | T20F5.2 | | | | | | |
|  | C05D9.1 | | WI5\_id:C05D9.1 |  | 2 | 308 | 0 | 4353 | 0.0044063856 | 0.0044063856 | 1 |
|  |  | RAFL05-04-G23 | At1g77440 / 20S proteasome beta subunit C (PBC2) | |  |  |  |  |  | | --- | --- | --- | --- | --- | |  |  |  |  |  | | Y38A8.2 | | | | | | |
|  |  | RAFL05-21-D23 | At1g21720 / 20S proteasome beta subunit C (PBC1) | |  |  |  |  |  | | --- | --- | --- | --- | --- | |  |  |  |  |  | | Y38A8.2 | | | | | | |
|  | C16C8.16 | | WI5\_id:C16C8.16 |  | 2 | 308 | 0 | 4353 | 0.0044063856 | 0.0044063856 | 1 |
|  |  | RAFL05-04-G23 | At1g77440 / 20S proteasome beta subunit C (PBC2) | |  |  |  |  |  | | --- | --- | --- | --- | --- | |  |  |  |  |  | | Y38A8.2 | | | | | | |
|  |  | RAFL05-21-D23 | At1g21720 / 20S proteasome beta subunit C (PBC1) | |  |  |  |  |  | | --- | --- | --- | --- | --- | |  |  |  |  |  | | Y38A8.2 | | | | | | |
|  | C36B1.4 | | WI5\_id:C36B1.4 |  | 3 | 307 | 3 | 4350 | 0.005004816 | 0.020019265 | 4 |
|  |  | RAFL04-12-B09 | At3g14290 / 20S proteasome alpha subunit E2 (PAE2) | |  |  |  |  |  | | --- | --- | --- | --- | --- | |  |  |  |  |  | | F25H2.9 | | | | | | |
|  |  | RAFL05-04-L16 | At1g79210 / 20S proteasome alpha subunit B, putative | |  |  |  |  |  | | --- | --- | --- | --- | --- | |  |  |  |  |  | | D1054.2 | | | | | | |
|  |  | RAFL05-12-G18 | At4g14800 / 20S proteasome beta subunit D2 (PBD2) | |  |  |  |  |  | | --- | --- | --- | --- | --- | |  |  |  |  |  | | T20F5.2 | | | | | | |
|  | W04D2.1 | | WI5\_id:W04D2.1 |  | 4 | 306 | 10 | 4343 | 0.011254616 | 0.07878231 | 7 |
|  |  | RAFL07-08-J17 | At1g13060 / 20S proteasome beta subunit E1 (PBE1) | |  |  |  |  |  | | --- | --- | --- | --- | --- | |  |  |  |  |  | | K05C4.1  Proteasome A-type and B-type | | | | | | |
|  |  | RAFL05-04-G23 | At1g77440 / 20S proteasome beta subunit C (PBC2) | |  |  |  |  |  | | --- | --- | --- | --- | --- | |  |  |  |  |  | | Y38A8.2 | | | | | | |
|  |  | RAFL04-18-F12 | At3g59920 / Rab GDP dissociation inhibitor | |  |  |  |  |  | | --- | --- | --- | --- | --- | |  |  |  |  |  | | Y57G11C.10  GDI-1 GDP dissociation inhibitor | | | | | | |
|  |  | RAFL05-21-D23 | At1g21720 / 20S proteasome beta subunit C (PBC1) | |  |  |  |  |  | | --- | --- | --- | --- | --- | |  |  |  |  |  | | Y38A8.2 | | | | | | |
|  | F25H2.9 | | WI5\_id:F25H2.9 |  | 2 | 308 | 1 | 4352 | 0.012636807 | 0.025273614 | 2 |
|  |  | RAFL04-12-B09 | At3g14290 / 20S proteasome alpha subunit E2 (PAE2) | |  |  |  |  |  | | --- | --- | --- | --- | --- | |  |  |  |  |  | | F25H2.9 | | | | | | |
|  |  | RAFL05-12-G18 | At4g14800 / 20S proteasome beta subunit D2 (PBD2) | |  |  |  |  |  | | --- | --- | --- | --- | --- | |  |  |  |  |  | | T20F5.2 | | | | | | |
|  | T01H3.1 | | WI5\_id:T01H3.1 |  | 2 | 308 | 2 | 4351 | 0.024166461 | 0.07249938 | 3 |
|  |  | RAFL04-09-A19 | At4g38510 / probable H+-transporting ATPase | |  |  |  |  |  | | --- | --- | --- | --- | --- | |  |  |  |  |  | | F20B6.2 | | | | | | |
|  |  | RAFL07-17-H08 | At4g38510 / probable H+-transporting ATPase | |  |  |  |  |  | | --- | --- | --- | --- | --- | |  |  |  |  |  | | F20B6.2 | | | | | | |
|  | ZK945.2 | | WI5\_id:ZK945.2 |  | 2 | 308 | 2 | 4351 | 0.024166461 | 0.07249938 | 3 |
|  |  | RAFL05-01-C14 | At5g42790 / 20S proteasome alpha subunit F1 (PAF1) | |  |  |  |  |  | | --- | --- | --- | --- | --- | |  |  |  |  |  | | CD4.6 | | | | | | |
|  |  | RAFL05-12-G18 | At4g14800 / 20S proteasome beta subunit D2 (PBD2) | |  |  |  |  |  | | --- | --- | --- | --- | --- | |  |  |  |  |  | | T20F5.2 | | | | | | |
|  | Y82E9BR.3 | | WI5\_id:Y82E9BR.3 |  | 2 | 308 | 2 | 4351 | 0.024166461 | 0.07249938 | 3 |
|  |  | RAFL04-15-D14 | At5g08680 / H+-transporting ATP synthase beta chain -related | |  |  |  |  |  | | --- | --- | --- | --- | --- | |  |  |  |  |  | | C34E10.6 | | | | | | |
|  |  | RAFL09-17-J21 | At5g08690 / H+-transporting ATP synthase beta chain (mitochondrial) -related | |  |  |  |  |  | | --- | --- | --- | --- | --- | |  |  |  |  |  | | C34E10.6 | | | | | | |
|  | C34E10.6 | | WI5\_id:C34E10.6 |  | 2 | 308 | 2 | 4351 | 0.024166461 | 0.07249938 | 3 |
|  |  | RAFL04-15-D14 | At5g08680 / H+-transporting ATP synthase beta chain -related | |  |  |  |  |  | | --- | --- | --- | --- | --- | |  |  |  |  |  | | C34E10.6 | | | | | | |
|  |  | RAFL09-17-J21 | At5g08690 / H+-transporting ATP synthase beta chain (mitochondrial) -related | |  |  |  |  |  | | --- | --- | --- | --- | --- | |  |  |  |  |  | | C34E10.6 | | | | | | |
|  | ZK637.8 | | WI5\_id:ZK637.8 |  | 2 | 308 | 2 | 4351 | 0.024166461 | 0.07249938 | 3 |
|  |  | RAFL04-09-A19 | At4g38510 / probable H+-transporting ATPase | |  |  |  |  |  | | --- | --- | --- | --- | --- | |  |  |  |  |  | | F20B6.2 | | | | | | |
|  |  | RAFL07-17-H08 | At4g38510 / probable H+-transporting ATPase | |  |  |  |  |  | | --- | --- | --- | --- | --- | |  |  |  |  |  | | F20B6.2 | | | | | | |
|  | Y69A2AR.18 | | WI5\_id:Y69A2AR.18 |  | 2 | 308 | 2 | 4351 | 0.024166461 | 0.07249938 | 3 |
|  |  | RAFL04-15-D14 | At5g08680 / H+-transporting ATP synthase beta chain -related | |  |  |  |  |  | | --- | --- | --- | --- | --- | |  |  |  |  |  | | C34E10.6 | | | | | | |
|  |  | RAFL09-17-J21 | At5g08690 / H+-transporting ATP synthase beta chain (mitochondrial) -related | |  |  |  |  |  | | --- | --- | --- | --- | --- | |  |  |  |  |  | | C34E10.6 | | | | | | |
|  | T27F7.3 | | WI5\_id:T27F7.3 |  | 2 | 308 | 2 | 4351 | 0.024166461 | 0.07249938 | 3 |
|  |  | RAFL05-14-N10 | At3g11400 / eukaryotic translation initiation factor 3 subunit g (eIF3g) | |  |  |  |  |  | | --- | --- | --- | --- | --- | |  |  |  |  |  | | F22B5.2 | | | | | | |
|  |  | RAFL07-07-F13 | At3g56150 / PROBABLE EUKARYOTIC TRANSLATION INITIATION FACTOR 3 SUBUNIT 8 | |  |  |  |  |  | | --- | --- | --- | --- | --- | |  |  |  |  |  | | T23D8.4 | | | | | | |
|  | Y74C10AR.1 | | WI5\_id:Y74C10AR.1 |  | 2 | 308 | 2 | 4351 | 0.024166461 | 0.07249938 | 3 |
|  |  | RAFL05-14-N10 | At3g11400 / eukaryotic translation initiation factor 3 subunit g (eIF3g) | |  |  |  |  |  | | --- | --- | --- | --- | --- | |  |  |  |  |  | | F22B5.2 | | | | | | |
|  |  | RAFL07-07-F13 | At3g56150 / PROBABLE EUKARYOTIC TRANSLATION INITIATION FACTOR 3 SUBUNIT 8 | |  |  |  |  |  | | --- | --- | --- | --- | --- | |  |  |  |  |  | | T23D8.4 | | | | | | |
|  | F27C1.7 | | WI5\_id:F27C1.7 |  | 2 | 308 | 2 | 4351 | 0.024166461 | 0.07249938 | 3 |
|  |  | RAFL04-15-D14 | At5g08680 / H+-transporting ATP synthase beta chain -related | |  |  |  |  |  | | --- | --- | --- | --- | --- | |  |  |  |  |  | | C34E10.6  ATP synthase beta chain | | | | | | |
|  |  | RAFL09-17-J21 | At5g08690 / H+-transporting ATP synthase beta chain (mitochondrial) -related | |  |  |  |  |  | | --- | --- | --- | --- | --- | |  |  |  |  |  | | C34E10.6  ATP synthase beta chain | | | | | | |
|  | Y54E2A.11 | | WI5\_id:Y54E2A.11 |  | 2 | 308 | 2 | 4351 | 0.024166461 | 0.07249938 | 3 |
|  |  | RAFL05-14-N10 | At3g11400 / eukaryotic translation initiation factor 3 subunit g (eIF3g) | |  |  |  |  |  | | --- | --- | --- | --- | --- | |  |  |  |  |  | | F22B5.2  RNA binding protein | | | | | | |
|  |  | RAFL07-07-F13 | At3g56150 / PROBABLE EUKARYOTIC TRANSLATION INITIATION FACTOR 3 SUBUNIT 8 | |  |  |  |  |  | | --- | --- | --- | --- | --- | |  |  |  |  |  | | T23D8.4 | | | | | | |
|  | C47B2.4 | | WI5\_id:C47B2.4 |  | 2 | 308 | 2 | 4351 | 0.024166461 | 0.07249938 | 3 |
|  |  | RAFL05-04-G23 | At1g77440 / 20S proteasome beta subunit C (PBC2) | |  |  |  |  |  | | --- | --- | --- | --- | --- | |  |  |  |  |  | | Y38A8.2 | | | | | | |
|  |  | RAFL05-21-D23 | At1g21720 / 20S proteasome beta subunit C (PBC1) | |  |  |  |  |  | | --- | --- | --- | --- | --- | |  |  |  |  |  | | Y38A8.2 | | | | | | |
|  | F58F12.1 | | WI5\_id:F58F12.1 |  | 2 | 308 | 2 | 4351 | 0.024166461 | 0.07249938 | 3 |
|  |  | RAFL04-15-D14 | At5g08680 / H+-transporting ATP synthase beta chain -related | |  |  |  |  |  | | --- | --- | --- | --- | --- | |  |  |  |  |  | | C34E10.6 | | | | | | |
|  |  | RAFL09-17-J21 | At5g08690 / H+-transporting ATP synthase beta chain (mitochondrial) -related | |  |  |  |  |  | | --- | --- | --- | --- | --- | |  |  |  |  |  | | C34E10.6 | | | | | | |
|  | Y110A7A.10 | | WI5\_id:Y110A7A.10 |  | 2 | 308 | 2 | 4351 | 0.024166461 | 0.07249938 | 3 |
|  |  | RAFL04-15-D14 | At5g08680 / H+-transporting ATP synthase beta chain -related | |  |  |  |  |  | | --- | --- | --- | --- | --- | |  |  |  |  |  | | C34E10.6 | | | | | | |
|  |  | RAFL09-17-J21 | At5g08690 / H+-transporting ATP synthase beta chain (mitochondrial) -related | |  |  |  |  |  | | --- | --- | --- | --- | --- | |  |  |  |  |  | | C34E10.6 | | | | | | |
|  | F46F11.5 | | WI5\_id:F46F11.5 |  | 2 | 308 | 2 | 4351 | 0.024166461 | 0.07249938 | 3 |
|  |  | RAFL04-09-A19 | At4g38510 / probable H+-transporting ATPase | |  |  |  |  |  | | --- | --- | --- | --- | --- | |  |  |  |  |  | | F20B6.2 | | | | | | |
|  |  | RAFL07-17-H08 | At4g38510 / probable H+-transporting ATPase | |  |  |  |  |  | | --- | --- | --- | --- | --- | |  |  |  |  |  | | F20B6.2 | | | | | | |
|  | Y38F2AL.3 | | WI5\_id:Y38F2AL.3 |  | 2 | 308 | 2 | 4351 | 0.024166461 | 0.07249938 | 3 |
|  |  | RAFL04-09-A19 | At4g38510 / probable H+-transporting ATPase | |  |  |  |  |  | | --- | --- | --- | --- | --- | |  |  |  |  |  | | F20B6.2 | | | | | | |
|  |  | RAFL07-17-H08 | At4g38510 / probable H+-transporting ATPase | |  |  |  |  |  | | --- | --- | --- | --- | --- | |  |  |  |  |  | | F20B6.2 | | | | | | |
|  | Y41C4A.14 | | WI5\_id:Y41C4A.14 |  | 2 | 308 | 3 | 4350 | 0.038523052 | 0.15409221 | 4 |
|  |  | RAFL05-04-G23 | At1g77440 / 20S proteasome beta subunit C (PBC2) | |  |  |  |  |  | | --- | --- | --- | --- | --- | |  |  |  |  |  | | Y38A8.2 | | | | | | |
|  |  | RAFL05-21-D23 | At1g21720 / 20S proteasome beta subunit C (PBC1) | |  |  |  |  |  | | --- | --- | --- | --- | --- | |  |  |  |  |  | | Y38A8.2 | | | | | | |
|  | C17H12.14 | | WI5\_id:C17H12.14 |  | 2 | 308 | 3 | 4350 | 0.038523052 | 0.15409221 | 4 |
|  |  | RAFL04-09-A19 | At4g38510 / probable H+-transporting ATPase | |  |  |  |  |  | | --- | --- | --- | --- | --- | |  |  |  |  |  | | F20B6.2 | | | | | | |
|  |  | RAFL07-17-H08 | At4g38510 / probable H+-transporting ATPase | |  |  |  |  |  | | --- | --- | --- | --- | --- | |  |  |  |  |  | | F20B6.2 | | | | | | |
|  | F20B6.2 | | WI5\_id:F20B6.2 |  | 2 | 308 | 3 | 4350 | 0.038523052 | 0.15409221 | 4 |
|  |  | RAFL04-09-A19 | At4g38510 / probable H+-transporting ATPase | |  |  |  |  |  | | --- | --- | --- | --- | --- | |  |  |  |  |  | | F20B6.2 | | | | | | |
|  |  | RAFL07-17-H08 | At4g38510 / probable H+-transporting ATPase | |  |  |  |  |  | | --- | --- | --- | --- | --- | |  |  |  |  |  | | F20B6.2 | | | | | | |
|  | F55H2.2 | | WI5\_id:F55H2.2 |  | 2 | 308 | 3 | 4350 | 0.038523052 | 0.15409221 | 4 |
|  |  | RAFL04-09-A19 | At4g38510 / probable H+-transporting ATPase | |  |  |  |  |  | | --- | --- | --- | --- | --- | |  |  |  |  |  | | F20B6.2 | | | | | | |
|  |  | RAFL07-17-H08 | At4g38510 / probable H+-transporting ATPase | |  |  |  |  |  | | --- | --- | --- | --- | --- | |  |  |  |  |  | | F20B6.2 | | | | | | |
|  | ZK970.4 | | WI5\_id:ZK970.4 |  | 2 | 308 | 3 | 4350 | 0.038523052 | 0.15409221 | 4 |
|  |  | RAFL04-09-A19 | At4g38510 / probable H+-transporting ATPase | |  |  |  |  |  | | --- | --- | --- | --- | --- | |  |  |  |  |  | | F20B6.2 | | | | | | |
|  |  | RAFL07-17-H08 | At4g38510 / probable H+-transporting ATPase | |  |  |  |  |  | | --- | --- | --- | --- | --- | |  |  |  |  |  | | F20B6.2 | | | | | | |
|  | C28H8.12 | | WI5\_id:C28H8.12 |  | 2 | 308 | 3 | 4350 | 0.038523052 | 0.15409221 | 4 |
|  |  | RAFL04-15-D14 | At5g08680 / H+-transporting ATP synthase beta chain -related | |  |  |  |  |  | | --- | --- | --- | --- | --- | |  |  |  |  |  | | C34E10.6 | | | | | | |
|  |  | RAFL09-17-J21 | At5g08690 / H+-transporting ATP synthase beta chain (mitochondrial) -related | |  |  |  |  |  | | --- | --- | --- | --- | --- | |  |  |  |  |  | | C34E10.6 | | | | | | |
|  | F28D1.2 | | WI5\_id:F28D1.2 |  | 2 | 308 | 3 | 4350 | 0.038523052 | 0.15409221 | 4 |
|  |  | RAFL04-15-D14 | At5g08680 / H+-transporting ATP synthase beta chain -related | |  |  |  |  |  | | --- | --- | --- | --- | --- | |  |  |  |  |  | | C34E10.6 | | | | | | |
|  |  | RAFL09-17-J21 | At5g08690 / H+-transporting ATP synthase beta chain (mitochondrial) -related | |  |  |  |  |  | | --- | --- | --- | --- | --- | |  |  |  |  |  | | C34E10.6 | | | | | | |
|  | C03A7.4 | | WI5\_id:C03A7.4 |  | 2 | 308 | 3 | 4350 | 0.038523052 | 0.15409221 | 4 |
|  |  | RAFL05-04-G23 | At1g77440 / 20S proteasome beta subunit C (PBC2) | |  |  |  |  |  | | --- | --- | --- | --- | --- | |  |  |  |  |  | | Y38A8.2  Peptidase | | | | | | |
|  |  | RAFL05-21-D23 | At1g21720 / 20S proteasome beta subunit C (PBC1) | |  |  |  |  |  | | --- | --- | --- | --- | --- | |  |  |  |  |  | | Y38A8.2  Peptidase | | | | | | |
|  | Y38F2AL.4 | | WI5\_id:Y38F2AL.4 |  | 2 | 308 | 3 | 4350 | 0.038523052 | 0.15409221 | 4 |
|  |  | RAFL04-09-A19 | At4g38510 / probable H+-transporting ATPase | |  |  |  |  |  | | --- | --- | --- | --- | --- | |  |  |  |  |  | | F20B6.2 | | | | | | |
|  |  | RAFL07-17-H08 | At4g38510 / probable H+-transporting ATPase | |  |  |  |  |  | | --- | --- | --- | --- | --- | |  |  |  |  |  | | F20B6.2 | | | | | | |
| Cluster:1-0 | | |  |  | A | B | C | D | P | P' | N |
|  | C36E8.5 | | WI5\_id:C36E8.5 |  | 2 | 147 | 4 | 4510 | 0.013980698 | 0.05592279 | 4 |
|  |  | RAFL09-11-P11 | At1g20010 / tubulin beta-5 chain (TUB5) | |  |  |  |  |  | | --- | --- | --- | --- | --- | |  |  |  |  |  | | C36E8.5 | | | | | | |
|  |  | RAFL04-16-G05 | At5g44340 / tubulin beta-4 chain (TUB4) | |  |  |  |  |  | | --- | --- | --- | --- | --- | |  |  |  |  |  | | C36E8.5 | | | | | | |
|  | K01G5.7 | | WI5\_id:K01G5.7 |  | 2 | 147 | 4 | 4510 | 0.013980698 | 0.05592279 | 4 |
|  |  | RAFL09-11-P11 | At1g20010 / tubulin beta-5 chain (TUB5) | |  |  |  |  |  | | --- | --- | --- | --- | --- | |  |  |  |  |  | | K01G5.7 | | | | | | |
|  |  | RAFL04-16-G05 | At5g44340 / tubulin beta-4 chain (TUB4) | |  |  |  |  |  | | --- | --- | --- | --- | --- | |  |  |  |  |  | | K01G5.7 | | | | | | |
|  | C47B2.3 | | WI5\_id:C47B2.3 |  | 2 | 147 | 4 | 4510 | 0.013980698 | 0.05592279 | 4 |
|  |  | RAFL09-11-P11 | At1g20010 / tubulin beta-5 chain (TUB5) | |  |  |  |  |  | | --- | --- | --- | --- | --- | |  |  |  |  |  | | B0272.1  tubulin beta chain | | | | | | |
|  |  | RAFL04-16-G05 | At5g44340 / tubulin beta-4 chain (TUB4) | |  |  |  |  |  | | --- | --- | --- | --- | --- | |  |  |  |  |  | | B0272.1  tubulin beta chain | | | | | | |
|  | B0272.1 | | WI5\_id:B0272.1 |  | 2 | 147 | 4 | 4510 | 0.013980698 | 0.05592279 | 4 |
|  |  | RAFL09-11-P11 | At1g20010 / tubulin beta-5 chain (TUB5) | |  |  |  |  |  | | --- | --- | --- | --- | --- | |  |  |  |  |  | | B0272.1 | | | | | | |
|  |  | RAFL04-16-G05 | At5g44340 / tubulin beta-4 chain (TUB4) | |  |  |  |  |  | | --- | --- | --- | --- | --- | |  |  |  |  |  | | B0272.1 | | | | | | |
|  | ZK792.8 | | WI5\_id:ZK792.8 |  | 2 | 147 | 4 | 4510 | 0.013980698 | 0.05592279 | 4 |
|  |  | RAFL09-11-P11 | At1g20010 / tubulin beta-5 chain (TUB5) | |  |  |  |  |  | | --- | --- | --- | --- | --- | |  |  |  |  |  | | B0272.1 | | | | | | |
|  |  | RAFL04-16-G05 | At5g44340 / tubulin beta-4 chain (TUB4) | |  |  |  |  |  | | --- | --- | --- | --- | --- | |  |  |  |  |  | | B0272.1 | | | | | | |
|  | W06F12.1 | | WI5\_id:W06F12.1 |  | 2 | 147 | 7 | 4507 | 0.031507306 | 0.22055113 | 7 |
|  |  | RAFL09-11-P11 | At1g20010 / tubulin beta-5 chain (TUB5) | |  |  |  |  |  | | --- | --- | --- | --- | --- | |  |  |  |  |  | | K01G5.7  tubulin beta-chain | | | | | | |
|  |  | RAFL04-16-G05 | At5g44340 / tubulin beta-4 chain (TUB4) | |  |  |  |  |  | | --- | --- | --- | --- | --- | |  |  |  |  |  | | K01G5.7  tubulin beta-chain | | | | | | |
|  | K08E3.7 | | WI5\_id:K08E3.7 |  | 2 | 147 | 7 | 4507 | 0.031507306 | 0.15753652 | 5 |
|  |  | RAFL09-11-P11 | At1g20010 / tubulin beta-5 chain (TUB5) | |  |  |  |  |  | | --- | --- | --- | --- | --- | |  |  |  |  |  | | C36E8.5  beta tubulin | | | | | | |
|  |  | RAFL04-16-G05 | At5g44340 / tubulin beta-4 chain (TUB4) | |  |  |  |  |  | | --- | --- | --- | --- | --- | |  |  |  |  |  | | C36E8.5  beta tubulin | | | | | | |
|  | C33H5.12 | | WI5\_id:C33H5.12 |  | 1 | 148 | 0 | 4514 | 0.031953678 | 0.031953678 | 1 |
|  |  | RAFL04-19-J20 | At3g50270 / hydroxycinnamoyl/benzoyltransferase-related | |  |  |  |  |  | | --- | --- | --- | --- | --- | |  |  |  |  |  | | K04G7.10 | | | | | | |
|  | Y46G5A.4 | | WI5\_id:Y46G5A.4 |  | 1 | 148 | 0 | 4514 | 0.031953678 | 0.031953678 | 1 |
|  |  | RAFL04-19-J20 | At3g50270 / hydroxycinnamoyl/benzoyltransferase-related | |  |  |  |  |  | | --- | --- | --- | --- | --- | |  |  |  |  |  | | K04G7.10 | | | | | | |
|  | B0035.2 | | WI5\_id:B0035.2 |  | 1 | 148 | 0 | 4514 | 0.031953678 | 0.031953678 | 1 |
|  |  | RAFL06-10-E18 | At1g50010 / tubulin alpha-2/alpha-4 chain (TUA2) | |  |  |  |  |  | | --- | --- | --- | --- | --- | |  |  |  |  |  | | F26E4.8  TBA-2 tubulin alpha-2 chain | | | | | | |
|  | F25B4.5 | | WI5\_id:F25B4.5 |  | 1 | 148 | 0 | 4514 | 0.031953678 | 0.031953678 | 1 |
|  |  | RAFL04-19-J20 | At3g50270 / hydroxycinnamoyl/benzoyltransferase-related | |  |  |  |  |  | | --- | --- | --- | --- | --- | |  |  |  |  |  | | K04G7.10 | | | | | | |
|  | K04G7.10 | | WI5\_id:K04G7.10 |  | 1 | 148 | 0 | 4514 | 0.031953678 | 0.031953678 | 1 |
|  |  | RAFL04-19-J20 | At3g50270 / hydroxycinnamoyl/benzoyltransferase-related | |  |  |  |  |  | | --- | --- | --- | --- | --- | |  |  |  |  |  | | K04G7.10 | | | | | | |
|  | F26A3.2 | | WI5\_id:F26A3.2 |  | 1 | 148 | 0 | 4514 | 0.031953678 | 0.031953678 | 1 |
|  |  | RAFL04-19-J20 | At3g50270 / hydroxycinnamoyl/benzoyltransferase-related | |  |  |  |  |  | | --- | --- | --- | --- | --- | |  |  |  |  |  | | K04G7.10 | | | | | | |
|  | F37E3.1 | | WI5\_id:F37E3.1 |  | 1 | 148 | 0 | 4514 | 0.031953678 | 0.031953678 | 1 |
|  |  | RAFL04-19-J20 | At3g50270 / hydroxycinnamoyl/benzoyltransferase-related | |  |  |  |  |  | | --- | --- | --- | --- | --- | |  |  |  |  |  | | K04G7.10 | | | | | | |
|  | C50C3.6 | | WI5\_id:C50C3.6 |  | 1 | 148 | 0 | 4514 | 0.031953678 | 0.031953678 | 1 |
|  |  | RAFL04-19-J20 | At3g50270 / hydroxycinnamoyl/benzoyltransferase-related | |  |  |  |  |  | | --- | --- | --- | --- | --- | |  |  |  |  |  | | K04G7.10 | | | | | | |
|  | ZK1098.1 | | WI5\_id:ZK1098.1 |  | 1 | 148 | 0 | 4514 | 0.031953678 | 0.031953678 | 1 |
|  |  | RAFL04-19-J20 | At3g50270 / hydroxycinnamoyl/benzoyltransferase-related | |  |  |  |  |  | | --- | --- | --- | --- | --- | |  |  |  |  |  | | K04G7.10 | | | | | | |
|  | ZK675.2 | | WI5\_id:ZK675.2 |  | 1 | 148 | 0 | 4514 | 0.031953678 | 0.031953678 | 1 |
|  |  | RAFL04-19-J20 | At3g50270 / hydroxycinnamoyl/benzoyltransferase-related | |  |  |  |  |  | | --- | --- | --- | --- | --- | |  |  |  |  |  | | K04G7.10 | | | | | | |
|  | F02E9.4 | | WI5\_id:F02E9.4 |  | 1 | 148 | 0 | 4514 | 0.031953678 | 0.031953678 | 1 |
|  |  | RAFL02-06-N10 | At5g14920 / expressed protein | |  |  |  |  |  | | --- | --- | --- | --- | --- | |  |  |  |  |  | | F02E9.4 | | | | | | |
|  | F58A4.8 | | WI5\_id:F58A4.8 |  | 2 | 147 | 8 | 4506 | 0.038570926 | 0.19285463 | 5 |
|  |  | RAFL09-11-P11 | At1g20010 / tubulin beta-5 chain (TUB5) | |  |  |  |  |  | | --- | --- | --- | --- | --- | |  |  |  |  |  | | C36E8.5 | | | | | | |
|  |  | RAFL04-16-G05 | At5g44340 / tubulin beta-4 chain (TUB4) | |  |  |  |  |  | | --- | --- | --- | --- | --- | |  |  |  |  |  | | C36E8.5 | | | | | | |
| Cluster:2-1 | | |  |  | A | B | C | D | P | P' | N |
|  | T05E11.1 | | WI5\_id:T05E11.1 |  | 8 | 236 | 9 | 4410 | 8.089096E-7 | 4.044548E-6 | 5 |
|  |  | RAFL04-18-N10 | At1g07770 / 40S ribosomal protein S15A (RPS15aA) | |  |  |  |  |  | | --- | --- | --- | --- | --- | |  |  |  |  |  | | F53A3.3 | | | | | | |
|  |  | RAFL09-15-M15 | At5g23740 / 40S ribosomal protein S11 (RPS11C) | |  |  |  |  |  | | --- | --- | --- | --- | --- | |  |  |  |  |  | | F40F11.1 | | | | | | |
|  |  | RAFL05-05-M24 | At5g02960 / 40S ribosomal protein S23 (RPS23B) | |  |  |  |  |  | | --- | --- | --- | --- | --- | |  |  |  |  |  | | F28D1.7 | | | | | | |
|  |  | RAFL07-13-J18 | At2g37270 / 40S ribosomal protein S5 (RPS5A) | |  |  |  |  |  | | --- | --- | --- | --- | --- | |  |  |  |  |  | | T05E11.1 | | | | | | |
|  |  | RAFL06-09-H09 | At3g48930 / 40S ribosomal protein S11 (RPS11A) | |  |  |  |  |  | | --- | --- | --- | --- | --- | |  |  |  |  |  | | F40F11.1 | | | | | | |
|  |  | RAFL06-08-B09 | At3g11510 / 40S ribosomal protein S14 (RPS14B) | |  |  |  |  |  | | --- | --- | --- | --- | --- | |  |  |  |  |  | | F37C12.9 | | | | | | |
|  |  | RAFL05-16-H14 | At1g04270 / 40S ribosomal protein S15 (RPS15A) | |  |  |  |  |  | | --- | --- | --- | --- | --- | |  |  |  |  |  | | F36A2.6 | | | | | | |
|  |  | RAFL04-19-M20 | At1g58380 / 40S ribosomal protein S2 (RPS2A) | |  |  |  |  |  | | --- | --- | --- | --- | --- | |  |  |  |  |  | | C49H3.11 | | | | | | |
|  | Y105E8A.16 | | WI5\_id:Y105E8A.16 |  | 8 | 236 | 9 | 4410 | 8.089096E-7 | 4.044548E-6 | 5 |
|  |  | RAFL04-18-N10 | At1g07770 / 40S ribosomal protein S15A (RPS15aA) | |  |  |  |  |  | | --- | --- | --- | --- | --- | |  |  |  |  |  | | F53A3.3 | | | | | | |
|  |  | RAFL09-15-M15 | At5g23740 / 40S ribosomal protein S11 (RPS11C) | |  |  |  |  |  | | --- | --- | --- | --- | --- | |  |  |  |  |  | | F40F11.1 | | | | | | |
|  |  | RAFL05-05-M24 | At5g02960 / 40S ribosomal protein S23 (RPS23B) | |  |  |  |  |  | | --- | --- | --- | --- | --- | |  |  |  |  |  | | F28D1.7 | | | | | | |
|  |  | RAFL07-13-J18 | At2g37270 / 40S ribosomal protein S5 (RPS5A) | |  |  |  |  |  | | --- | --- | --- | --- | --- | |  |  |  |  |  | | T05E11.1 | | | | | | |
|  |  | RAFL06-09-H09 | At3g48930 / 40S ribosomal protein S11 (RPS11A) | |  |  |  |  |  | | --- | --- | --- | --- | --- | |  |  |  |  |  | | F40F11.1 | | | | | | |
|  |  | RAFL06-08-B09 | At3g11510 / 40S ribosomal protein S14 (RPS14B) | |  |  |  |  |  | | --- | --- | --- | --- | --- | |  |  |  |  |  | | F37C12.9 | | | | | | |
|  |  | RAFL05-16-H14 | At1g04270 / 40S ribosomal protein S15 (RPS15A) | |  |  |  |  |  | | --- | --- | --- | --- | --- | |  |  |  |  |  | | F36A2.6 | | | | | | |
|  |  | RAFL04-19-M20 | At1g58380 / 40S ribosomal protein S2 (RPS2A) | |  |  |  |  |  | | --- | --- | --- | --- | --- | |  |  |  |  |  | | C49H3.11 | | | | | | |
|  | C23G10.3 | | WI5\_id:C23G10.3 |  | 8 | 236 | 10 | 4409 | 1.3906676E-6 | 6.953338E-6 | 5 |
|  |  | RAFL04-18-N10 | At1g07770 / 40S ribosomal protein S15A (RPS15aA) | |  |  |  |  |  | | --- | --- | --- | --- | --- | |  |  |  |  |  | | F53A3.3  40S ribosomal protein | | | | | | |
|  |  | RAFL09-15-M15 | At5g23740 / 40S ribosomal protein S11 (RPS11C) | |  |  |  |  |  | | --- | --- | --- | --- | --- | |  |  |  |  |  | | F40F11.1  ribosomal protein S11 | | | | | | |
|  |  | RAFL05-05-M24 | At5g02960 / 40S ribosomal protein S23 (RPS23B) | |  |  |  |  |  | | --- | --- | --- | --- | --- | |  |  |  |  |  | | F28D1.7  ribosomal protein S23 | | | | | | |
|  |  | RAFL07-13-J18 | At2g37270 / 40S ribosomal protein S5 (RPS5A) | |  |  |  |  |  | | --- | --- | --- | --- | --- | |  |  |  |  |  | | T05E11.1 | | | | | | |
|  |  | RAFL06-09-H09 | At3g48930 / 40S ribosomal protein S11 (RPS11A) | |  |  |  |  |  | | --- | --- | --- | --- | --- | |  |  |  |  |  | | F40F11.1  ribosomal protein S11 | | | | | | |
|  |  | RAFL06-08-B09 | At3g11510 / 40S ribosomal protein S14 (RPS14B) | |  |  |  |  |  | | --- | --- | --- | --- | --- | |  |  |  |  |  | | F37C12.9  Ribosomal protein S14 | | | | | | |
|  |  | RAFL05-16-H14 | At1g04270 / 40S ribosomal protein S15 (RPS15A) | |  |  |  |  |  | | --- | --- | --- | --- | --- | |  |  |  |  |  | | F36A2.6 | | | | | | |
|  |  | RAFL04-19-M20 | At1g58380 / 40S ribosomal protein S2 (RPS2A) | |  |  |  |  |  | | --- | --- | --- | --- | --- | |  |  |  |  |  | | C49H3.11 | | | | | | |
|  | F36A2.6 | | WI5\_id:F36A2.6 |  | 8 | 236 | 10 | 4409 | 1.3906676E-6 | 6.953338E-6 | 5 |
|  |  | RAFL04-18-N10 | At1g07770 / 40S ribosomal protein S15A (RPS15aA) | |  |  |  |  |  | | --- | --- | --- | --- | --- | |  |  |  |  |  | | F53A3.3 | | | | | | |
|  |  | RAFL09-15-M15 | At5g23740 / 40S ribosomal protein S11 (RPS11C) | |  |  |  |  |  | | --- | --- | --- | --- | --- | |  |  |  |  |  | | F40F11.1 | | | | | | |
|  |  | RAFL05-05-M24 | At5g02960 / 40S ribosomal protein S23 (RPS23B) | |  |  |  |  |  | | --- | --- | --- | --- | --- | |  |  |  |  |  | | F28D1.7 | | | | | | |
|  |  | RAFL07-13-J18 | At2g37270 / 40S ribosomal protein S5 (RPS5A) | |  |  |  |  |  | | --- | --- | --- | --- | --- | |  |  |  |  |  | | T05E11.1 | | | | | | |
|  |  | RAFL06-09-H09 | At3g48930 / 40S ribosomal protein S11 (RPS11A) | |  |  |  |  |  | | --- | --- | --- | --- | --- | |  |  |  |  |  | | F40F11.1 | | | | | | |
|  |  | RAFL06-08-B09 | At3g11510 / 40S ribosomal protein S14 (RPS14B) | |  |  |  |  |  | | --- | --- | --- | --- | --- | |  |  |  |  |  | | F37C12.9 | | | | | | |
|  |  | RAFL05-16-H14 | At1g04270 / 40S ribosomal protein S15 (RPS15A) | |  |  |  |  |  | | --- | --- | --- | --- | --- | |  |  |  |  |  | | F36A2.6 | | | | | | |
|  |  | RAFL04-19-M20 | At1g58380 / 40S ribosomal protein S2 (RPS2A) | |  |  |  |  |  | | --- | --- | --- | --- | --- | |  |  |  |  |  | | C49H3.11 | | | | | | |
|  | C49H3.11 | | WI5\_id:C49H3.11 |  | 8 | 236 | 13 | 4406 | 5.6352947E-6 | 3.3811768E-5 | 6 |
|  |  | RAFL04-18-N10 | At1g07770 / 40S ribosomal protein S15A (RPS15aA) | |  |  |  |  |  | | --- | --- | --- | --- | --- | |  |  |  |  |  | | F53A3.3 | | | | | | |
|  |  | RAFL09-15-M15 | At5g23740 / 40S ribosomal protein S11 (RPS11C) | |  |  |  |  |  | | --- | --- | --- | --- | --- | |  |  |  |  |  | | F40F11.1 | | | | | | |
|  |  | RAFL05-05-M24 | At5g02960 / 40S ribosomal protein S23 (RPS23B) | |  |  |  |  |  | | --- | --- | --- | --- | --- | |  |  |  |  |  | | F28D1.7 | | | | | | |
|  |  | RAFL07-13-J18 | At2g37270 / 40S ribosomal protein S5 (RPS5A) | |  |  |  |  |  | | --- | --- | --- | --- | --- | |  |  |  |  |  | | T05E11.1 | | | | | | |
|  |  | RAFL06-09-H09 | At3g48930 / 40S ribosomal protein S11 (RPS11A) | |  |  |  |  |  | | --- | --- | --- | --- | --- | |  |  |  |  |  | | F40F11.1 | | | | | | |
|  |  | RAFL06-08-B09 | At3g11510 / 40S ribosomal protein S14 (RPS14B) | |  |  |  |  |  | | --- | --- | --- | --- | --- | |  |  |  |  |  | | F37C12.9 | | | | | | |
|  |  | RAFL05-16-H14 | At1g04270 / 40S ribosomal protein S15 (RPS15A) | |  |  |  |  |  | | --- | --- | --- | --- | --- | |  |  |  |  |  | | F36A2.6 | | | | | | |
|  |  | RAFL04-19-M20 | At1g58380 / 40S ribosomal protein S2 (RPS2A) | |  |  |  |  |  | | --- | --- | --- | --- | --- | |  |  |  |  |  | | C49H3.11 | | | | | | |
|  | T24B8.1 | | WI5\_id:T24B8.1 |  | 7 | 237 | 10 | 4409 | 1.22133315E-5 | 6.106666E-5 | 5 |
|  |  | RAFL02-10-A09 | At1g33140 / 60S ribosomal protein L9 (RPL90A/C) | |  |  |  |  |  | | --- | --- | --- | --- | --- | |  |  |  |  |  | | R13A5.8 | | | | | | |
|  |  | RAFL07-15-M07 | At1g04480 / 60S ribosomal protein L23 (RPL23A) | |  |  |  |  |  | | --- | --- | --- | --- | --- | |  |  |  |  |  | | B0336.10 | | | | | | |
|  |  | RAFL05-17-L17 | At3g55280 / 60S ribosomal protein L23A (RPL23aB) | |  |  |  |  |  | | --- | --- | --- | --- | --- | |  |  |  |  |  | | F52B5.6 | | | | | | |
|  |  | RAFL11-12-H04 | At3g25520 / 60S ribosomal protein L5 (RPL5A) | |  |  |  |  |  | | --- | --- | --- | --- | --- | |  |  |  |  |  | | F54C9.5 | | | | | | |
|  |  | RAFL05-18-P15 | At1g04480 / 60S ribosomal protein L23 (RPL23A) | |  |  |  |  |  | | --- | --- | --- | --- | --- | |  |  |  |  |  | | B0336.10 | | | | | | |
|  |  | RAFL04-18-N22 | At2g44120 / 60S ribosomal protein L7 (RPL7C) | |  |  |  |  |  | | --- | --- | --- | --- | --- | |  |  |  |  |  | | F53G12.10  ribosomal protein | | | | | | |
|  |  | RAFL11-07-B21 | At3g25520 / 60S ribosomal protein L5 (RPL5A) | |  |  |  |  |  | | --- | --- | --- | --- | --- | |  |  |  |  |  | | F54C9.5 | | | | | | |
|  | F52B5.6 | | WI5\_id:F52B5.6 |  | 7 | 237 | 13 | 4406 | 4.250125E-5 | 2.550075E-4 | 6 |
|  |  | RAFL02-10-A09 | At1g33140 / 60S ribosomal protein L9 (RPL90A/C) | |  |  |  |  |  | | --- | --- | --- | --- | --- | |  |  |  |  |  | | R13A5.8 | | | | | | |
|  |  | RAFL07-15-M07 | At1g04480 / 60S ribosomal protein L23 (RPL23A) | |  |  |  |  |  | | --- | --- | --- | --- | --- | |  |  |  |  |  | | B0336.10 | | | | | | |
|  |  | RAFL05-17-L17 | At3g55280 / 60S ribosomal protein L23A (RPL23aB) | |  |  |  |  |  | | --- | --- | --- | --- | --- | |  |  |  |  |  | | F52B5.6 | | | | | | |
|  |  | RAFL11-12-H04 | At3g25520 / 60S ribosomal protein L5 (RPL5A) | |  |  |  |  |  | | --- | --- | --- | --- | --- | |  |  |  |  |  | | F54C9.5 | | | | | | |
|  |  | RAFL05-18-P15 | At1g04480 / 60S ribosomal protein L23 (RPL23A) | |  |  |  |  |  | | --- | --- | --- | --- | --- | |  |  |  |  |  | | B0336.10 | | | | | | |
|  |  | RAFL04-18-N22 | At2g44120 / 60S ribosomal protein L7 (RPL7C) | |  |  |  |  |  | | --- | --- | --- | --- | --- | |  |  |  |  |  | | F53G12.10 | | | | | | |
|  |  | RAFL11-07-B21 | At3g25520 / 60S ribosomal protein L5 (RPL5A) | |  |  |  |  |  | | --- | --- | --- | --- | --- | |  |  |  |  |  | | F54C9.5 | | | | | | |
|  | F54C9.5 | | WI5\_id:F54C9.5 |  | 7 | 237 | 14 | 4405 | 6.093423E-5 | 4.265396E-4 | 7 |
|  |  | RAFL02-10-A09 | At1g33140 / 60S ribosomal protein L9 (RPL90A/C) | |  |  |  |  |  | | --- | --- | --- | --- | --- | |  |  |  |  |  | | R13A5.8 | | | | | | |
|  |  | RAFL07-15-M07 | At1g04480 / 60S ribosomal protein L23 (RPL23A) | |  |  |  |  |  | | --- | --- | --- | --- | --- | |  |  |  |  |  | | B0336.10 | | | | | | |
|  |  | RAFL05-17-L17 | At3g55280 / 60S ribosomal protein L23A (RPL23aB) | |  |  |  |  |  | | --- | --- | --- | --- | --- | |  |  |  |  |  | | F52B5.6 | | | | | | |
|  |  | RAFL11-12-H04 | At3g25520 / 60S ribosomal protein L5 (RPL5A) | |  |  |  |  |  | | --- | --- | --- | --- | --- | |  |  |  |  |  | | F54C9.5 | | | | | | |
|  |  | RAFL05-18-P15 | At1g04480 / 60S ribosomal protein L23 (RPL23A) | |  |  |  |  |  | | --- | --- | --- | --- | --- | |  |  |  |  |  | | B0336.10 | | | | | | |
|  |  | RAFL04-18-N22 | At2g44120 / 60S ribosomal protein L7 (RPL7C) | |  |  |  |  |  | | --- | --- | --- | --- | --- | |  |  |  |  |  | | F53G12.10 | | | | | | |
|  |  | RAFL11-07-B21 | At3g25520 / 60S ribosomal protein L5 (RPL5A) | |  |  |  |  |  | | --- | --- | --- | --- | --- | |  |  |  |  |  | | F54C9.5 | | | | | | |
|  | F53G12.10 | | WI5\_id:F53G12.10 |  | 4 | 240 | 2 | 4417 | 1.0099356E-4 | 3.0298068E-4 | 3 |
|  |  | RAFL05-17-L17 | At3g55280 / 60S ribosomal protein L23A (RPL23aB) | |  |  |  |  |  | | --- | --- | --- | --- | --- | |  |  |  |  |  | | F52B5.6 | | | | | | |
|  |  | RAFL11-12-H04 | At3g25520 / 60S ribosomal protein L5 (RPL5A) | |  |  |  |  |  | | --- | --- | --- | --- | --- | |  |  |  |  |  | | F54C9.5 | | | | | | |
|  |  | RAFL04-18-N22 | At2g44120 / 60S ribosomal protein L7 (RPL7C) | |  |  |  |  |  | | --- | --- | --- | --- | --- | |  |  |  |  |  | | F53G12.10 | | | | | | |
|  |  | RAFL11-07-B21 | At3g25520 / 60S ribosomal protein L5 (RPL5A) | |  |  |  |  |  | | --- | --- | --- | --- | --- | |  |  |  |  |  | | F54C9.5 | | | | | | |
|  | F37C12.9 | | WI5\_id:F37C12.9 |  | 4 | 240 | 2 | 4417 | 1.0099356E-4 | 2.0198712E-4 | 2 |
|  |  | RAFL07-13-J18 | At2g37270 / 40S ribosomal protein S5 (RPS5A) | |  |  |  |  |  | | --- | --- | --- | --- | --- | |  |  |  |  |  | | T05E11.1 | | | | | | |
|  |  | RAFL06-08-B09 | At3g11510 / 40S ribosomal protein S14 (RPS14B) | |  |  |  |  |  | | --- | --- | --- | --- | --- | |  |  |  |  |  | | F37C12.9 | | | | | | |
|  |  | RAFL05-16-H14 | At1g04270 / 40S ribosomal protein S15 (RPS15A) | |  |  |  |  |  | | --- | --- | --- | --- | --- | |  |  |  |  |  | | F36A2.6 | | | | | | |
|  |  | RAFL04-19-M20 | At1g58380 / 40S ribosomal protein S2 (RPS2A) | |  |  |  |  |  | | --- | --- | --- | --- | --- | |  |  |  |  |  | | C49H3.11 | | | | | | |
|  | F28D1.7 | | WI5\_id:F28D1.7 |  | 4 | 240 | 2 | 4417 | 1.0099356E-4 | 2.0198712E-4 | 2 |
|  |  | RAFL05-05-M24 | At5g02960 / 40S ribosomal protein S23 (RPS23B) | |  |  |  |  |  | | --- | --- | --- | --- | --- | |  |  |  |  |  | | F28D1.7 | | | | | | |
|  |  | RAFL07-13-J18 | At2g37270 / 40S ribosomal protein S5 (RPS5A) | |  |  |  |  |  | | --- | --- | --- | --- | --- | |  |  |  |  |  | | T05E11.1 | | | | | | |
|  |  | RAFL05-16-H14 | At1g04270 / 40S ribosomal protein S15 (RPS15A) | |  |  |  |  |  | | --- | --- | --- | --- | --- | |  |  |  |  |  | | F36A2.6 | | | | | | |
|  |  | RAFL04-19-M20 | At1g58380 / 40S ribosomal protein S2 (RPS2A) | |  |  |  |  |  | | --- | --- | --- | --- | --- | |  |  |  |  |  | | C49H3.11 | | | | | | |
|  | F40F11.1 | | WI5\_id:F40F11.1 |  | 5 | 239 | 6 | 4413 | 1.3419612E-4 | 5.367845E-4 | 4 |
|  |  | RAFL09-15-M15 | At5g23740 / 40S ribosomal protein S11 (RPS11C) | |  |  |  |  |  | | --- | --- | --- | --- | --- | |  |  |  |  |  | | F40F11.1 | | | | | | |
|  |  | RAFL07-13-J18 | At2g37270 / 40S ribosomal protein S5 (RPS5A) | |  |  |  |  |  | | --- | --- | --- | --- | --- | |  |  |  |  |  | | T05E11.1 | | | | | | |
|  |  | RAFL06-09-H09 | At3g48930 / 40S ribosomal protein S11 (RPS11A) | |  |  |  |  |  | | --- | --- | --- | --- | --- | |  |  |  |  |  | | F40F11.1 | | | | | | |
|  |  | RAFL05-16-H14 | At1g04270 / 40S ribosomal protein S15 (RPS15A) | |  |  |  |  |  | | --- | --- | --- | --- | --- | |  |  |  |  |  | | F36A2.6 | | | | | | |
|  |  | RAFL04-19-M20 | At1g58380 / 40S ribosomal protein S2 (RPS2A) | |  |  |  |  |  | | --- | --- | --- | --- | --- | |  |  |  |  |  | | C49H3.11 | | | | | | |
|  | C09D4.5 | | WI5\_id:C09D4.5 |  | 6 | 238 | 12 | 4407 | 2.1043592E-4 | 0.0014730515 | 7 |
|  |  | RAFL02-10-A09 | At1g33140 / 60S ribosomal protein L9 (RPL90A/C) | |  |  |  |  |  | | --- | --- | --- | --- | --- | |  |  |  |  |  | | R13A5.8 | | | | | | |
|  |  | RAFL07-15-M07 | At1g04480 / 60S ribosomal protein L23 (RPL23A) | |  |  |  |  |  | | --- | --- | --- | --- | --- | |  |  |  |  |  | | B0336.10 | | | | | | |
|  |  | RAFL05-17-L17 | At3g55280 / 60S ribosomal protein L23A (RPL23aB) | |  |  |  |  |  | | --- | --- | --- | --- | --- | |  |  |  |  |  | | F52B5.6 | | | | | | |
|  |  | RAFL11-12-H04 | At3g25520 / 60S ribosomal protein L5 (RPL5A) | |  |  |  |  |  | | --- | --- | --- | --- | --- | |  |  |  |  |  | | F54C9.5 | | | | | | |
|  |  | RAFL05-18-P15 | At1g04480 / 60S ribosomal protein L23 (RPL23A) | |  |  |  |  |  | | --- | --- | --- | --- | --- | |  |  |  |  |  | | B0336.10 | | | | | | |
|  |  | RAFL11-07-B21 | At3g25520 / 60S ribosomal protein L5 (RPL5A) | |  |  |  |  |  | | --- | --- | --- | --- | --- | |  |  |  |  |  | | F54C9.5 | | | | | | |
|  | Y43B11AR.4 | | WI5\_id:Y43B11AR.4 |  | 5 | 239 | 7 | 4412 | 2.202804E-4 | 0.0011014021 | 5 |
|  |  | RAFL04-18-N10 | At1g07770 / 40S ribosomal protein S15A (RPS15aA) | |  |  |  |  |  | | --- | --- | --- | --- | --- | |  |  |  |  |  | | F53A3.3 | | | | | | |
|  |  | RAFL09-15-M15 | At5g23740 / 40S ribosomal protein S11 (RPS11C) | |  |  |  |  |  | | --- | --- | --- | --- | --- | |  |  |  |  |  | | F40F11.1 | | | | | | |
|  |  | RAFL06-09-H09 | At3g48930 / 40S ribosomal protein S11 (RPS11A) | |  |  |  |  |  | | --- | --- | --- | --- | --- | |  |  |  |  |  | | F40F11.1 | | | | | | |
|  |  | RAFL05-16-H14 | At1g04270 / 40S ribosomal protein S15 (RPS15A) | |  |  |  |  |  | | --- | --- | --- | --- | --- | |  |  |  |  |  | | F36A2.6 | | | | | | |
|  |  | RAFL04-19-M20 | At1g58380 / 40S ribosomal protein S2 (RPS2A) | |  |  |  |  |  | | --- | --- | --- | --- | --- | |  |  |  |  |  | | C49H3.11 | | | | | | |
|  | F13B10.2 | | WI5\_id:F13B10.2 |  | 6 | 238 | 13 | 4406 | 2.9421985E-4 | 0.001765319 | 6 |
|  |  | RAFL02-10-A09 | At1g33140 / 60S ribosomal protein L9 (RPL90A/C) | |  |  |  |  |  | | --- | --- | --- | --- | --- | |  |  |  |  |  | | R13A5.8 | | | | | | |
|  |  | RAFL07-15-M07 | At1g04480 / 60S ribosomal protein L23 (RPL23A) | |  |  |  |  |  | | --- | --- | --- | --- | --- | |  |  |  |  |  | | B0336.10 | | | | | | |
|  |  | RAFL05-17-L17 | At3g55280 / 60S ribosomal protein L23A (RPL23aB) | |  |  |  |  |  | | --- | --- | --- | --- | --- | |  |  |  |  |  | | F52B5.6 | | | | | | |
|  |  | RAFL11-12-H04 | At3g25520 / 60S ribosomal protein L5 (RPL5A) | |  |  |  |  |  | | --- | --- | --- | --- | --- | |  |  |  |  |  | | F54C9.5 | | | | | | |
|  |  | RAFL05-18-P15 | At1g04480 / 60S ribosomal protein L23 (RPL23A) | |  |  |  |  |  | | --- | --- | --- | --- | --- | |  |  |  |  |  | | B0336.10 | | | | | | |
|  |  | RAFL11-07-B21 | At3g25520 / 60S ribosomal protein L5 (RPL5A) | |  |  |  |  |  | | --- | --- | --- | --- | --- | |  |  |  |  |  | | F54C9.5 | | | | | | |
|  | F10B5.1 | | WI5\_id:F10B5.1 |  | 6 | 238 | 13 | 4406 | 2.9421985E-4 | 0.001765319 | 6 |
|  |  | RAFL02-10-A09 | At1g33140 / 60S ribosomal protein L9 (RPL90A/C) | |  |  |  |  |  | | --- | --- | --- | --- | --- | |  |  |  |  |  | | R13A5.8 | | | | | | |
|  |  | RAFL07-15-M07 | At1g04480 / 60S ribosomal protein L23 (RPL23A) | |  |  |  |  |  | | --- | --- | --- | --- | --- | |  |  |  |  |  | | B0336.10 | | | | | | |
|  |  | RAFL05-17-L17 | At3g55280 / 60S ribosomal protein L23A (RPL23aB) | |  |  |  |  |  | | --- | --- | --- | --- | --- | |  |  |  |  |  | | F52B5.6 | | | | | | |
|  |  | RAFL11-12-H04 | At3g25520 / 60S ribosomal protein L5 (RPL5A) | |  |  |  |  |  | | --- | --- | --- | --- | --- | |  |  |  |  |  | | F54C9.5 | | | | | | |
|  |  | RAFL05-18-P15 | At1g04480 / 60S ribosomal protein L23 (RPL23A) | |  |  |  |  |  | | --- | --- | --- | --- | --- | |  |  |  |  |  | | B0336.10 | | | | | | |
|  |  | RAFL11-07-B21 | At3g25520 / 60S ribosomal protein L5 (RPL5A) | |  |  |  |  |  | | --- | --- | --- | --- | --- | |  |  |  |  |  | | F54C9.5 | | | | | | |
|  | B0250.1 | | WI5\_id:B0250.1 |  | 6 | 238 | 13 | 4406 | 2.9421985E-4 | 0.001765319 | 6 |
|  |  | RAFL02-10-A09 | At1g33140 / 60S ribosomal protein L9 (RPL90A/C) | |  |  |  |  |  | | --- | --- | --- | --- | --- | |  |  |  |  |  | | R13A5.8  Ribosomal protein L9 | | | | | | |
|  |  | RAFL07-15-M07 | At1g04480 / 60S ribosomal protein L23 (RPL23A) | |  |  |  |  |  | | --- | --- | --- | --- | --- | |  |  |  |  |  | | B0336.10 | | | | | | |
|  |  | RAFL05-17-L17 | At3g55280 / 60S ribosomal protein L23A (RPL23aB) | |  |  |  |  |  | | --- | --- | --- | --- | --- | |  |  |  |  |  | | F52B5.6 | | | | | | |
|  |  | RAFL11-12-H04 | At3g25520 / 60S ribosomal protein L5 (RPL5A) | |  |  |  |  |  | | --- | --- | --- | --- | --- | |  |  |  |  |  | | F54C9.5 | | | | | | |
|  |  | RAFL05-18-P15 | At1g04480 / 60S ribosomal protein L23 (RPL23A) | |  |  |  |  |  | | --- | --- | --- | --- | --- | |  |  |  |  |  | | B0336.10 | | | | | | |
|  |  | RAFL11-07-B21 | At3g25520 / 60S ribosomal protein L5 (RPL5A) | |  |  |  |  |  | | --- | --- | --- | --- | --- | |  |  |  |  |  | | F54C9.5 | | | | | | |
|  | F28C6.7 | | WI5\_id:F28C6.7 |  | 6 | 238 | 13 | 4406 | 2.9421985E-4 | 0.001765319 | 6 |
|  |  | RAFL02-10-A09 | At1g33140 / 60S ribosomal protein L9 (RPL90A/C) | |  |  |  |  |  | | --- | --- | --- | --- | --- | |  |  |  |  |  | | R13A5.8 | | | | | | |
|  |  | RAFL07-15-M07 | At1g04480 / 60S ribosomal protein L23 (RPL23A) | |  |  |  |  |  | | --- | --- | --- | --- | --- | |  |  |  |  |  | | B0336.10 | | | | | | |
|  |  | RAFL05-17-L17 | At3g55280 / 60S ribosomal protein L23A (RPL23aB) | |  |  |  |  |  | | --- | --- | --- | --- | --- | |  |  |  |  |  | | F52B5.6 | | | | | | |
|  |  | RAFL11-12-H04 | At3g25520 / 60S ribosomal protein L5 (RPL5A) | |  |  |  |  |  | | --- | --- | --- | --- | --- | |  |  |  |  |  | | F54C9.5 | | | | | | |
|  |  | RAFL05-18-P15 | At1g04480 / 60S ribosomal protein L23 (RPL23A) | |  |  |  |  |  | | --- | --- | --- | --- | --- | |  |  |  |  |  | | B0336.10 | | | | | | |
|  |  | RAFL11-07-B21 | At3g25520 / 60S ribosomal protein L5 (RPL5A) | |  |  |  |  |  | | --- | --- | --- | --- | --- | |  |  |  |  |  | | F54C9.5 | | | | | | |
|  | Y37E3.8 | | WI5\_id:Y37E3.8 |  | 6 | 238 | 13 | 4406 | 2.9421985E-4 | 0.001765319 | 6 |
|  |  | RAFL02-10-A09 | At1g33140 / 60S ribosomal protein L9 (RPL90A/C) | |  |  |  |  |  | | --- | --- | --- | --- | --- | |  |  |  |  |  | | R13A5.8 | | | | | | |
|  |  | RAFL07-15-M07 | At1g04480 / 60S ribosomal protein L23 (RPL23A) | |  |  |  |  |  | | --- | --- | --- | --- | --- | |  |  |  |  |  | | B0336.10 | | | | | | |
|  |  | RAFL05-17-L17 | At3g55280 / 60S ribosomal protein L23A (RPL23aB) | |  |  |  |  |  | | --- | --- | --- | --- | --- | |  |  |  |  |  | | F52B5.6 | | | | | | |
|  |  | RAFL11-12-H04 | At3g25520 / 60S ribosomal protein L5 (RPL5A) | |  |  |  |  |  | | --- | --- | --- | --- | --- | |  |  |  |  |  | | F54C9.5 | | | | | | |
|  |  | RAFL05-18-P15 | At1g04480 / 60S ribosomal protein L23 (RPL23A) | |  |  |  |  |  | | --- | --- | --- | --- | --- | |  |  |  |  |  | | B0336.10 | | | | | | |
|  |  | RAFL11-07-B21 | At3g25520 / 60S ribosomal protein L5 (RPL5A) | |  |  |  |  |  | | --- | --- | --- | --- | --- | |  |  |  |  |  | | F54C9.5 | | | | | | |
|  | ZK652.4 | | WI5\_id:ZK652.4 |  | 6 | 238 | 13 | 4406 | 2.9421985E-4 | 0.001765319 | 6 |
|  |  | RAFL02-10-A09 | At1g33140 / 60S ribosomal protein L9 (RPL90A/C) | |  |  |  |  |  | | --- | --- | --- | --- | --- | |  |  |  |  |  | | R13A5.8 | | | | | | |
|  |  | RAFL07-15-M07 | At1g04480 / 60S ribosomal protein L23 (RPL23A) | |  |  |  |  |  | | --- | --- | --- | --- | --- | |  |  |  |  |  | | B0336.10 | | | | | | |
|  |  | RAFL05-17-L17 | At3g55280 / 60S ribosomal protein L23A (RPL23aB) | |  |  |  |  |  | | --- | --- | --- | --- | --- | |  |  |  |  |  | | F52B5.6 | | | | | | |
|  |  | RAFL11-12-H04 | At3g25520 / 60S ribosomal protein L5 (RPL5A) | |  |  |  |  |  | | --- | --- | --- | --- | --- | |  |  |  |  |  | | F54C9.5 | | | | | | |
|  |  | RAFL05-18-P15 | At1g04480 / 60S ribosomal protein L23 (RPL23A) | |  |  |  |  |  | | --- | --- | --- | --- | --- | |  |  |  |  |  | | B0336.10 | | | | | | |
|  |  | RAFL11-07-B21 | At3g25520 / 60S ribosomal protein L5 (RPL5A) | |  |  |  |  |  | | --- | --- | --- | --- | --- | |  |  |  |  |  | | F54C9.5 | | | | | | |
|  | R09B5.5 | | WI5\_id:R09B5.5 |  | 4 | 240 | 4 | 4415 | 4.3340996E-4 | 0.0017336399 | 4 |
|  |  | RAFL07-13-J18 | At2g37270 / 40S ribosomal protein S5 (RPS5A) | |  |  |  |  |  | | --- | --- | --- | --- | --- | |  |  |  |  |  | | T05E11.1 | | | | | | |
|  |  | RAFL11-12-H04 | At3g25520 / 60S ribosomal protein L5 (RPL5A) | |  |  |  |  |  | | --- | --- | --- | --- | --- | |  |  |  |  |  | | F54C9.5 | | | | | | |
|  |  | RAFL04-18-N22 | At2g44120 / 60S ribosomal protein L7 (RPL7C) | |  |  |  |  |  | | --- | --- | --- | --- | --- | |  |  |  |  |  | | F53G12.10 | | | | | | |
|  |  | RAFL11-07-B21 | At3g25520 / 60S ribosomal protein L5 (RPL5A) | |  |  |  |  |  | | --- | --- | --- | --- | --- | |  |  |  |  |  | | F54C9.5 | | | | | | |
|  | B0336.10 | | WI5\_id:B0336.10 |  | 5 | 239 | 9 | 4410 | 5.1060907E-4 | 0.0030636543 | 6 |
|  |  | RAFL07-15-M07 | At1g04480 / 60S ribosomal protein L23 (RPL23A) | |  |  |  |  |  | | --- | --- | --- | --- | --- | |  |  |  |  |  | | B0336.10 | | | | | | |
|  |  | RAFL05-17-L17 | At3g55280 / 60S ribosomal protein L23A (RPL23aB) | |  |  |  |  |  | | --- | --- | --- | --- | --- | |  |  |  |  |  | | F52B5.6 | | | | | | |
|  |  | RAFL11-12-H04 | At3g25520 / 60S ribosomal protein L5 (RPL5A) | |  |  |  |  |  | | --- | --- | --- | --- | --- | |  |  |  |  |  | | F54C9.5 | | | | | | |
|  |  | RAFL05-18-P15 | At1g04480 / 60S ribosomal protein L23 (RPL23A) | |  |  |  |  |  | | --- | --- | --- | --- | --- | |  |  |  |  |  | | B0336.10 | | | | | | |
|  |  | RAFL11-07-B21 | At3g25520 / 60S ribosomal protein L5 (RPL5A) | |  |  |  |  |  | | --- | --- | --- | --- | --- | |  |  |  |  |  | | F54C9.5 | | | | | | |
|  | Y51H4A.17 | | WI5\_id:Y51H4A.17 |  | 3 | 241 | 3 | 4416 | 0.0025160299 | 0.0075480896 | 3 |
|  |  | RAFL06-08-N05 | At5g28540 / luminal binding protein 1 precursor (BiP-1) (AtBP1) | |  |  |  |  |  | | --- | --- | --- | --- | --- | |  |  |  |  |  | | F43E2.8 | | | | | | |
|  |  | RAFL04-18-N22 | At2g44120 / 60S ribosomal protein L7 (RPL7C) | |  |  |  |  |  | | --- | --- | --- | --- | --- | |  |  |  |  |  | | F53G12.10 | | | | | | |
|  |  | RAFL07-11-O19 | At5g28540 / luminal binding protein 1 precursor (BiP-1) (AtBP1) | |  |  |  |  |  | | --- | --- | --- | --- | --- | |  |  |  |  |  | | F43E2.8 | | | | | | |
|  | Y57G11C.16 | | WI5\_id:Y57G11C.16 |  | 3 | 241 | 3 | 4416 | 0.0025160299 | 0.0075480896 | 3 |
|  |  | RAFL07-13-J18 | At2g37270 / 40S ribosomal protein S5 (RPS5A) | |  |  |  |  |  | | --- | --- | --- | --- | --- | |  |  |  |  |  | | T05E11.1 | | | | | | |
|  |  | RAFL05-16-H14 | At1g04270 / 40S ribosomal protein S15 (RPS15A) | |  |  |  |  |  | | --- | --- | --- | --- | --- | |  |  |  |  |  | | F36A2.6 | | | | | | |
|  |  | RAFL04-19-M20 | At1g58380 / 40S ribosomal protein S2 (RPS2A) | |  |  |  |  |  | | --- | --- | --- | --- | --- | |  |  |  |  |  | | C49H3.11 | | | | | | |
|  | B0412.4 | | WI5\_id:B0412.4 |  | 3 | 241 | 3 | 4416 | 0.0025160299 | 0.0050320597 | 2 |
|  |  | RAFL07-13-J18 | At2g37270 / 40S ribosomal protein S5 (RPS5A) | |  |  |  |  |  | | --- | --- | --- | --- | --- | |  |  |  |  |  | | T05E11.1  40S ribosomal protein S5 | | | | | | |
|  |  | RAFL05-16-H14 | At1g04270 / 40S ribosomal protein S15 (RPS15A) | |  |  |  |  |  | | --- | --- | --- | --- | --- | |  |  |  |  |  | | F36A2.6  40S ribosomal protein S15 | | | | | | |
|  |  | RAFL04-19-M20 | At1g58380 / 40S ribosomal protein S2 (RPS2A) | |  |  |  |  |  | | --- | --- | --- | --- | --- | |  |  |  |  |  | | C49H3.11 | | | | | | |
|  | R13A5.8 | | WI5\_id:R13A5.8 |  | 4 | 240 | 9 | 4410 | 0.003594625 | 0.02156775 | 6 |
|  |  | RAFL02-10-A09 | At1g33140 / 60S ribosomal protein L9 (RPL90A/C) | |  |  |  |  |  | | --- | --- | --- | --- | --- | |  |  |  |  |  | | R13A5.8 | | | | | | |
|  |  | RAFL05-17-L17 | At3g55280 / 60S ribosomal protein L23A (RPL23aB) | |  |  |  |  |  | | --- | --- | --- | --- | --- | |  |  |  |  |  | | F52B5.6 | | | | | | |
|  |  | RAFL11-12-H04 | At3g25520 / 60S ribosomal protein L5 (RPL5A) | |  |  |  |  |  | | --- | --- | --- | --- | --- | |  |  |  |  |  | | F54C9.5 | | | | | | |
|  |  | RAFL11-07-B21 | At3g25520 / 60S ribosomal protein L5 (RPL5A) | |  |  |  |  |  | | --- | --- | --- | --- | --- | |  |  |  |  |  | | F54C9.5 | | | | | | |
|  | B0205.3 | | WI5\_id:B0205.3 |  | 5 | 239 | 18 | 4401 | 0.0058271554 | 0.08158018 | 14 |
|  |  | RAFL02-10-A09 | At1g33140 / 60S ribosomal protein L9 (RPL90A/C) | |  |  |  |  |  | | --- | --- | --- | --- | --- | |  |  |  |  |  | | R13A5.8 | | | | | | |
|  |  | RAFL07-15-M07 | At1g04480 / 60S ribosomal protein L23 (RPL23A) | |  |  |  |  |  | | --- | --- | --- | --- | --- | |  |  |  |  |  | | B0336.10 | | | | | | |
|  |  | RAFL02-04-I03 | At5g58290 / 26S proteasome AAA-ATPase subunit RPT3 | |  |  |  |  |  | | --- | --- | --- | --- | --- | |  |  |  |  |  | | F23F12.6 | | | | | | |
|  |  | RAFL05-18-P15 | At1g04480 / 60S ribosomal protein L23 (RPL23A) | |  |  |  |  |  | | --- | --- | --- | --- | --- | |  |  |  |  |  | | B0336.10 | | | | | | |
|  |  | RAFL04-18-N22 | At2g44120 / 60S ribosomal protein L7 (RPL7C) | |  |  |  |  |  | | --- | --- | --- | --- | --- | |  |  |  |  |  | | F53G12.10 | | | | | | |
|  | F53A3.3 | | WI5\_id:F53A3.3 |  | 4 | 240 | 11 | 4408 | 0.006317224 | 0.037903342 | 6 |
|  |  | RAFL04-18-N10 | At1g07770 / 40S ribosomal protein S15A (RPS15aA) | |  |  |  |  |  | | --- | --- | --- | --- | --- | |  |  |  |  |  | | F53A3.3 | | | | | | |
|  |  | RAFL07-13-J18 | At2g37270 / 40S ribosomal protein S5 (RPS5A) | |  |  |  |  |  | | --- | --- | --- | --- | --- | |  |  |  |  |  | | T05E11.1 | | | | | | |
|  |  | RAFL05-16-H14 | At1g04270 / 40S ribosomal protein S15 (RPS15A) | |  |  |  |  |  | | --- | --- | --- | --- | --- | |  |  |  |  |  | | F36A2.6 | | | | | | |
|  |  | RAFL04-19-M20 | At1g58380 / 40S ribosomal protein S2 (RPS2A) | |  |  |  |  |  | | --- | --- | --- | --- | --- | |  |  |  |  |  | | C49H3.11 | | | | | | |
|  | T28A8.7 | | WI5\_id:T28A8.7 |  | 2 | 242 | 1 | 4418 | 0.00789916 | 0.01579832 | 2 |
|  |  | RAFL07-15-M07 | At1g04480 / 60S ribosomal protein L23 (RPL23A) | |  |  |  |  |  | | --- | --- | --- | --- | --- | |  |  |  |  |  | | B0336.10 | | | | | | |
|  |  | RAFL05-18-P15 | At1g04480 / 60S ribosomal protein L23 (RPL23A) | |  |  |  |  |  | | --- | --- | --- | --- | --- | |  |  |  |  |  | | B0336.10 | | | | | | |
|  | Y63D3A.6 | | WI5\_id:Y63D3A.6 |  | 2 | 242 | 1 | 4418 | 0.00789916 | 0.01579832 | 2 |
|  |  | RAFL06-08-N05 | At5g28540 / luminal binding protein 1 precursor (BiP-1) (AtBP1) | |  |  |  |  |  | | --- | --- | --- | --- | --- | |  |  |  |  |  | | C15H9.6  heat shock protein | | | | | | |
|  |  | RAFL07-11-O19 | At5g28540 / luminal binding protein 1 precursor (BiP-1) (AtBP1) | |  |  |  |  |  | | --- | --- | --- | --- | --- | |  |  |  |  |  | | C15H9.6  heat shock protein | | | | | | |
|  | F43E2.8 | | WI5\_id:F43E2.8 |  | 2 | 242 | 1 | 4418 | 0.00789916 | 0.01579832 | 2 |
|  |  | RAFL06-08-N05 | At5g28540 / luminal binding protein 1 precursor (BiP-1) (AtBP1) | |  |  |  |  |  | | --- | --- | --- | --- | --- | |  |  |  |  |  | | F43E2.8 | | | | | | |
|  |  | RAFL07-11-O19 | At5g28540 / luminal binding protein 1 precursor (BiP-1) (AtBP1) | |  |  |  |  |  | | --- | --- | --- | --- | --- | |  |  |  |  |  | | F43E2.8 | | | | | | |
|  | C15H9.6 | | WI5\_id:C15H9.6 |  | 2 | 242 | 1 | 4418 | 0.00789916 | 0.01579832 | 2 |
|  |  | RAFL06-08-N05 | At5g28540 / luminal binding protein 1 precursor (BiP-1) (AtBP1) | |  |  |  |  |  | | --- | --- | --- | --- | --- | |  |  |  |  |  | | C15H9.6 | | | | | | |
|  |  | RAFL07-11-O19 | At5g28540 / luminal binding protein 1 precursor (BiP-1) (AtBP1) | |  |  |  |  |  | | --- | --- | --- | --- | --- | |  |  |  |  |  | | C15H9.6 | | | | | | |
|  | F58A4.8 | | WI5\_id:F58A4.8 |  | 3 | 241 | 7 | 4412 | 0.012910854 | 0.064554274 | 5 |
|  |  | RAFL05-09-F01 | At5g44340 / tubulin beta-4 chain (TUB4) | |  |  |  |  |  | | --- | --- | --- | --- | --- | |  |  |  |  |  | | C36E8.5 | | | | | | |
|  |  | RAFL06-12-I16 | At2g29550 / tubulin beta-7 chain (TUB7) | |  |  |  |  |  | | --- | --- | --- | --- | --- | |  |  |  |  |  | | C36E8.5 | | | | | | |
|  |  | RAFL06-14-I03 | At3g60770 / 40S ribosomal protein S13 (RPS13A) | |  |  |  |  |  | | --- | --- | --- | --- | --- | |  |  |  |  |  | | C16A3.9 | | | | | | |
|  | M02A10.3 | | WI5\_id:M02A10.3 |  | 2 | 242 | 2 | 4417 | 0.01525385 | 0.0305077 | 2 |
|  |  | RAFL04-18-N22 | At2g44120 / 60S ribosomal protein L7 (RPL7C) | |  |  |  |  |  | | --- | --- | --- | --- | --- | |  |  |  |  |  | | F53G12.10 | | | | | | |
|  |  | RAFL02-03-G08 | At4g18730 / 60S ribosomal protein L11 (RPL11C) | |  |  |  |  |  | | --- | --- | --- | --- | --- | |  |  |  |  |  | | F07D10.1  ribosomal protein | | | | | | |
|  | B0507.1 | | WI5\_id:B0507.1 |  | 2 | 242 | 2 | 4417 | 0.01525385 | 0.045761548 | 3 |
|  |  | RAFL11-12-H04 | At3g25520 / 60S ribosomal protein L5 (RPL5A) | |  |  |  |  |  | | --- | --- | --- | --- | --- | |  |  |  |  |  | | F54C9.5  60S ribosomal protein L5 | | | | | | |
|  |  | RAFL11-07-B21 | At3g25520 / 60S ribosomal protein L5 (RPL5A) | |  |  |  |  |  | | --- | --- | --- | --- | --- | |  |  |  |  |  | | F54C9.5  60S ribosomal protein L5 | | | | | | |
|  | T01C3.6 | | WI5\_id:T01C3.6 |  | 2 | 242 | 2 | 4417 | 0.01525385 | 0.045761548 | 3 |
|  |  | RAFL05-16-H14 | At1g04270 / 40S ribosomal protein S15 (RPS15A) | |  |  |  |  |  | | --- | --- | --- | --- | --- | |  |  |  |  |  | | F36A2.6 | | | | | | |
|  |  | RAFL04-19-M20 | At1g58380 / 40S ribosomal protein S2 (RPS2A) | |  |  |  |  |  | | --- | --- | --- | --- | --- | |  |  |  |  |  | | C49H3.11 | | | | | | |
|  | T26A5.9 | | WI5\_id:T26A5.9 |  | 2 | 242 | 2 | 4417 | 0.01525385 | 0.045761548 | 3 |
|  |  | RAFL07-15-K08 | At4g34670 / 40S ribosomal protein S3A (RPS3aB) | |  |  |  |  |  | | --- | --- | --- | --- | --- | |  |  |  |  |  | | F56F3.5 | | | | | | |
|  |  | RAFL06-08-B09 | At3g11510 / 40S ribosomal protein S14 (RPS14B) | |  |  |  |  |  | | --- | --- | --- | --- | --- | |  |  |  |  |  | | F37C12.9 | | | | | | |
|  | R10E11.2 | | WI5\_id:R10E11.2 |  | 2 | 242 | 2 | 4417 | 0.01525385 | 0.0305077 | 2 |
|  |  | RAFL02-10-A09 | At1g33140 / 60S ribosomal protein L9 (RPL90A/C) | |  |  |  |  |  | | --- | --- | --- | --- | --- | |  |  |  |  |  | | R13A5.8 | | | | | | |
|  |  | RAFL04-18-N22 | At2g44120 / 60S ribosomal protein L7 (RPL7C) | |  |  |  |  |  | | --- | --- | --- | --- | --- | |  |  |  |  |  | | F53G12.10 | | | | | | |
|  | T22F3.4 | | WI5\_id:T22F3.4 |  | 3 | 241 | 8 | 4411 | 0.017076377 | 0.10245825 | 6 |
|  |  | RAFL05-17-L17 | At3g55280 / 60S ribosomal protein L23A (RPL23aB) | |  |  |  |  |  | | --- | --- | --- | --- | --- | |  |  |  |  |  | | F52B5.6 | | | | | | |
|  |  | RAFL11-12-H04 | At3g25520 / 60S ribosomal protein L5 (RPL5A) | |  |  |  |  |  | | --- | --- | --- | --- | --- | |  |  |  |  |  | | F54C9.5 | | | | | | |
|  |  | RAFL11-07-B21 | At3g25520 / 60S ribosomal protein L5 (RPL5A) | |  |  |  |  |  | | --- | --- | --- | --- | --- | |  |  |  |  |  | | F54C9.5 | | | | | | |
|  | JC8.3 | | WI5\_id:JC8.3 |  | 3 | 241 | 8 | 4411 | 0.017076377 | 0.10245825 | 6 |
|  |  | RAFL05-17-L17 | At3g55280 / 60S ribosomal protein L23A (RPL23aB) | |  |  |  |  |  | | --- | --- | --- | --- | --- | |  |  |  |  |  | | F52B5.6 | | | | | | |
|  |  | RAFL11-12-H04 | At3g25520 / 60S ribosomal protein L5 (RPL5A) | |  |  |  |  |  | | --- | --- | --- | --- | --- | |  |  |  |  |  | | F54C9.5 | | | | | | |
|  |  | RAFL11-07-B21 | At3g25520 / 60S ribosomal protein L5 (RPL5A) | |  |  |  |  |  | | --- | --- | --- | --- | --- | |  |  |  |  |  | | F54C9.5 | | | | | | |
|  | Y48G8AL.8 | | WI5\_id:Y48G8AL.8 |  | 3 | 241 | 8 | 4411 | 0.017076377 | 0.10245825 | 6 |
|  |  | RAFL05-17-L17 | At3g55280 / 60S ribosomal protein L23A (RPL23aB) | |  |  |  |  |  | | --- | --- | --- | --- | --- | |  |  |  |  |  | | F52B5.6 | | | | | | |
|  |  | RAFL11-12-H04 | At3g25520 / 60S ribosomal protein L5 (RPL5A) | |  |  |  |  |  | | --- | --- | --- | --- | --- | |  |  |  |  |  | | F54C9.5 | | | | | | |
|  |  | RAFL11-07-B21 | At3g25520 / 60S ribosomal protein L5 (RPL5A) | |  |  |  |  |  | | --- | --- | --- | --- | --- | |  |  |  |  |  | | F54C9.5 | | | | | | |
|  | F25H2.10 | | WI5\_id:F25H2.10 |  | 3 | 241 | 9 | 4410 | 0.021903817 | 0.13142289 | 6 |
|  |  | RAFL05-17-L17 | At3g55280 / 60S ribosomal protein L23A (RPL23aB) | |  |  |  |  |  | | --- | --- | --- | --- | --- | |  |  |  |  |  | | F52B5.6 | | | | | | |
|  |  | RAFL11-12-H04 | At3g25520 / 60S ribosomal protein L5 (RPL5A) | |  |  |  |  |  | | --- | --- | --- | --- | --- | |  |  |  |  |  | | F54C9.5 | | | | | | |
|  |  | RAFL11-07-B21 | At3g25520 / 60S ribosomal protein L5 (RPL5A) | |  |  |  |  |  | | --- | --- | --- | --- | --- | |  |  |  |  |  | | F54C9.5 | | | | | | |
|  | M01F1.2 | | WI5\_id:M01F1.2 |  | 3 | 241 | 9 | 4410 | 0.021903817 | 0.13142289 | 6 |
|  |  | RAFL05-17-L17 | At3g55280 / 60S ribosomal protein L23A (RPL23aB) | |  |  |  |  |  | | --- | --- | --- | --- | --- | |  |  |  |  |  | | F52B5.6 | | | | | | |
|  |  | RAFL11-12-H04 | At3g25520 / 60S ribosomal protein L5 (RPL5A) | |  |  |  |  |  | | --- | --- | --- | --- | --- | |  |  |  |  |  | | F54C9.5 | | | | | | |
|  |  | RAFL11-07-B21 | At3g25520 / 60S ribosomal protein L5 (RPL5A) | |  |  |  |  |  | | --- | --- | --- | --- | --- | |  |  |  |  |  | | F54C9.5 | | | | | | |
|  | K01G5.4 | | WI5\_id:K01G5.4 |  | 3 | 241 | 9 | 4410 | 0.021903817 | 0.17523053 | 8 |
|  |  | RAFL09-15-M15 | At5g23740 / 40S ribosomal protein S11 (RPS11C) | |  |  |  |  |  | | --- | --- | --- | --- | --- | |  |  |  |  |  | | F40F11.1 | | | | | | |
|  |  | RAFL06-09-H09 | At3g48930 / 40S ribosomal protein S11 (RPS11A) | |  |  |  |  |  | | --- | --- | --- | --- | --- | |  |  |  |  |  | | F40F11.1 | | | | | | |
|  |  | RAFL04-19-M20 | At1g58380 / 40S ribosomal protein S2 (RPS2A) | |  |  |  |  |  | | --- | --- | --- | --- | --- | |  |  |  |  |  | | C49H3.11 | | | | | | |
|  | T04H1.2 | | WI5\_id:T04H1.2 |  | 2 | 242 | 3 | 4416 | 0.024550742 | 0.098202966 | 4 |
|  |  | RAFL07-15-M07 | At1g04480 / 60S ribosomal protein L23 (RPL23A) | |  |  |  |  |  | | --- | --- | --- | --- | --- | |  |  |  |  |  | | B0336.10 | | | | | | |
|  |  | RAFL05-18-P15 | At1g04480 / 60S ribosomal protein L23 (RPL23A) | |  |  |  |  |  | | --- | --- | --- | --- | --- | |  |  |  |  |  | | B0336.10 | | | | | | |
|  | C16A3.9 | | WI5\_id:C16A3.9 |  | 2 | 242 | 3 | 4416 | 0.024550742 | 0.07365222 | 3 |
|  |  | RAFL07-15-K08 | At4g34670 / 40S ribosomal protein S3A (RPS3aB) | |  |  |  |  |  | | --- | --- | --- | --- | --- | |  |  |  |  |  | | F56F3.5 | | | | | | |
|  |  | RAFL06-14-I03 | At3g60770 / 40S ribosomal protein S13 (RPS13A) | |  |  |  |  |  | | --- | --- | --- | --- | --- | |  |  |  |  |  | | C16A3.9  40S ribosomal protein S13 | | | | | | |
|  | F56F3.5 | | WI5\_id:F56F3.5 |  | 2 | 242 | 3 | 4416 | 0.024550742 | 0.07365222 | 3 |
|  |  | RAFL07-15-K08 | At4g34670 / 40S ribosomal protein S3A (RPS3aB) | |  |  |  |  |  | | --- | --- | --- | --- | --- | |  |  |  |  |  | | F56F3.5 | | | | | | |
|  |  | RAFL06-14-I03 | At3g60770 / 40S ribosomal protein S13 (RPS13A) | |  |  |  |  |  | | --- | --- | --- | --- | --- | |  |  |  |  |  | | C16A3.9 | | | | | | |
|  | Y71G12B.27 | | WI5\_id:Y71G12B.27 |  | 2 | 242 | 3 | 4416 | 0.024550742 | 0.098202966 | 4 |
|  |  | RAFL11-12-H04 | At3g25520 / 60S ribosomal protein L5 (RPL5A) | |  |  |  |  |  | | --- | --- | --- | --- | --- | |  |  |  |  |  | | F54C9.5 | | | | | | |
|  |  | RAFL11-07-B21 | At3g25520 / 60S ribosomal protein L5 (RPL5A) | |  |  |  |  |  | | --- | --- | --- | --- | --- | |  |  |  |  |  | | F54C9.5 | | | | | | |
|  | F37C12.4 | | WI5\_id:F37C12.4 |  | 2 | 242 | 3 | 4416 | 0.024550742 | 0.098202966 | 4 |
|  |  | RAFL11-12-H04 | At3g25520 / 60S ribosomal protein L5 (RPL5A) | |  |  |  |  |  | | --- | --- | --- | --- | --- | |  |  |  |  |  | | F54C9.5 | | | | | | |
|  |  | RAFL11-07-B21 | At3g25520 / 60S ribosomal protein L5 (RPL5A) | |  |  |  |  |  | | --- | --- | --- | --- | --- | |  |  |  |  |  | | F54C9.5 | | | | | | |
|  | T09A5.10 | | WI5\_id:T09A5.10 |  | 2 | 242 | 3 | 4416 | 0.024550742 | 0.07365222 | 3 |
|  |  | RAFL06-08-N05 | At5g28540 / luminal binding protein 1 precursor (BiP-1) (AtBP1) | |  |  |  |  |  | | --- | --- | --- | --- | --- | |  |  |  |  |  | | F43E2.8  heat shock protein | | | | | | |
|  |  | RAFL07-11-O19 | At5g28540 / luminal binding protein 1 precursor (BiP-1) (AtBP1) | |  |  |  |  |  | | --- | --- | --- | --- | --- | |  |  |  |  |  | | F43E2.8  heat shock protein | | | | | | |
|  | B0041.4 | | WI5\_id:B0041.4 |  | 3 | 241 | 10 | 4409 | 0.027396575 | 0.16437945 | 6 |
|  |  | RAFL05-17-L17 | At3g55280 / 60S ribosomal protein L23A (RPL23aB) | |  |  |  |  |  | | --- | --- | --- | --- | --- | |  |  |  |  |  | | F52B5.6  60S ribosomal protein | | | | | | |
|  |  | RAFL11-12-H04 | At3g25520 / 60S ribosomal protein L5 (RPL5A) | |  |  |  |  |  | | --- | --- | --- | --- | --- | |  |  |  |  |  | | F54C9.5 | | | | | | |
|  |  | RAFL11-07-B21 | At3g25520 / 60S ribosomal protein L5 (RPL5A) | |  |  |  |  |  | | --- | --- | --- | --- | --- | |  |  |  |  |  | | F54C9.5 | | | | | | |
|  | W04D2.1 | | WI5\_id:W04D2.1 |  | 3 | 241 | 11 | 4408 | 0.033551577 | 0.23486103 | 7 |
|  |  | RAFL11-12-H04 | At3g25520 / 60S ribosomal protein L5 (RPL5A) | |  |  |  |  |  | | --- | --- | --- | --- | --- | |  |  |  |  |  | | F54C9.5 | | | | | | |
|  |  | RAFL04-18-N22 | At2g44120 / 60S ribosomal protein L7 (RPL7C) | |  |  |  |  |  | | --- | --- | --- | --- | --- | |  |  |  |  |  | | F53G12.10 | | | | | | |
|  |  | RAFL11-07-B21 | At3g25520 / 60S ribosomal protein L5 (RPL5A) | |  |  |  |  |  | | --- | --- | --- | --- | --- | |  |  |  |  |  | | F54C9.5 | | | | | | |
|  | Y71F9AL.13 | | WI5\_id:Y71F9AL.13 |  | 3 | 241 | 11 | 4408 | 0.033551577 | 0.26841262 | 8 |
|  |  | RAFL05-17-L17 | At3g55280 / 60S ribosomal protein L23A (RPL23aB) | |  |  |  |  |  | | --- | --- | --- | --- | --- | |  |  |  |  |  | | F52B5.6 | | | | | | |
|  |  | RAFL11-12-H04 | At3g25520 / 60S ribosomal protein L5 (RPL5A) | |  |  |  |  |  | | --- | --- | --- | --- | --- | |  |  |  |  |  | | F54C9.5 | | | | | | |
|  |  | RAFL11-07-B21 | At3g25520 / 60S ribosomal protein L5 (RPL5A) | |  |  |  |  |  | | --- | --- | --- | --- | --- | |  |  |  |  |  | | F54C9.5 | | | | | | |
|  | C36E8.5 | | WI5\_id:C36E8.5 |  | 2 | 242 | 4 | 4415 | 0.0355681 | 0.1422724 | 4 |
|  |  | RAFL05-09-F01 | At5g44340 / tubulin beta-4 chain (TUB4) | |  |  |  |  |  | | --- | --- | --- | --- | --- | |  |  |  |  |  | | C36E8.5 | | | | | | |
|  |  | RAFL06-12-I16 | At2g29550 / tubulin beta-7 chain (TUB7) | |  |  |  |  |  | | --- | --- | --- | --- | --- | |  |  |  |  |  | | C36E8.5 | | | | | | |
|  | K01G5.7 | | WI5\_id:K01G5.7 |  | 2 | 242 | 4 | 4415 | 0.0355681 | 0.1422724 | 4 |
|  |  | RAFL05-09-F01 | At5g44340 / tubulin beta-4 chain (TUB4) | |  |  |  |  |  | | --- | --- | --- | --- | --- | |  |  |  |  |  | | K01G5.7 | | | | | | |
|  |  | RAFL06-12-I16 | At2g29550 / tubulin beta-7 chain (TUB7) | |  |  |  |  |  | | --- | --- | --- | --- | --- | |  |  |  |  |  | | K01G5.7 | | | | | | |
|  | C47B2.3 | | WI5\_id:C47B2.3 |  | 2 | 242 | 4 | 4415 | 0.0355681 | 0.1422724 | 4 |
|  |  | RAFL05-09-F01 | At5g44340 / tubulin beta-4 chain (TUB4) | |  |  |  |  |  | | --- | --- | --- | --- | --- | |  |  |  |  |  | | B0272.1  tubulin beta chain | | | | | | |
|  |  | RAFL06-12-I16 | At2g29550 / tubulin beta-7 chain (TUB7) | |  |  |  |  |  | | --- | --- | --- | --- | --- | |  |  |  |  |  | | B0272.1  tubulin beta chain | | | | | | |
|  | B0272.1 | | WI5\_id:B0272.1 |  | 2 | 242 | 4 | 4415 | 0.0355681 | 0.1422724 | 4 |
|  |  | RAFL05-09-F01 | At5g44340 / tubulin beta-4 chain (TUB4) | |  |  |  |  |  | | --- | --- | --- | --- | --- | |  |  |  |  |  | | B0272.1 | | | | | | |
|  |  | RAFL06-12-I16 | At2g29550 / tubulin beta-7 chain (TUB7) | |  |  |  |  |  | | --- | --- | --- | --- | --- | |  |  |  |  |  | | B0272.1 | | | | | | |
|  | ZK792.8 | | WI5\_id:ZK792.8 |  | 2 | 242 | 4 | 4415 | 0.0355681 | 0.1422724 | 4 |
|  |  | RAFL05-09-F01 | At5g44340 / tubulin beta-4 chain (TUB4) | |  |  |  |  |  | | --- | --- | --- | --- | --- | |  |  |  |  |  | | B0272.1 | | | | | | |
|  |  | RAFL06-12-I16 | At2g29550 / tubulin beta-7 chain (TUB7) | |  |  |  |  |  | | --- | --- | --- | --- | --- | |  |  |  |  |  | | B0272.1 | | | | | | |
|  | C23G10.4 | | WI5\_id:C23G10.4 |  | 2 | 242 | 5 | 4414 | 0.04810191 | 0.24050954 | 5 |
|  |  | RAFL06-08-N05 | At5g28540 / luminal binding protein 1 precursor (BiP-1) (AtBP1) | |  |  |  |  |  | | --- | --- | --- | --- | --- | |  |  |  |  |  | | C15H9.6 | | | | | | |
|  |  | RAFL07-11-O19 | At5g28540 / luminal binding protein 1 precursor (BiP-1) (AtBP1) | |  |  |  |  |  | | --- | --- | --- | --- | --- | |  |  |  |  |  | | C15H9.6 | | | | | | |
|  | T07C4.1 | | WI5\_id:T07C4.1 |  | 2 | 242 | 5 | 4414 | 0.04810191 | 0.24050954 | 5 |
|  |  | RAFL04-18-N10 | At1g07770 / 40S ribosomal protein S15A (RPS15aA) | |  |  |  |  |  | | --- | --- | --- | --- | --- | |  |  |  |  |  | | F53A3.3 | | | | | | |
|  |  | RAFL07-15-K08 | At4g34670 / 40S ribosomal protein S3A (RPS3aB) | |  |  |  |  |  | | --- | --- | --- | --- | --- | |  |  |  |  |  | | F56F3.5  Ribosomal protein S3a (human) homolog. | | | | | | |
| Cluster:10-2 | | |  |  | A | B | C | D | P | P' | N |
|  | F55A11.2 | | WI5\_id:F55A11.2 |  | 1 | 108 | 0 | 4554 | 0.02337551 | 0.02337551 | 1 |
|  |  | RAFL11-12-H18 | At4g17530 / ras-related small GTP-binding protein RAB1c | |  |  |  |  |  | | --- | --- | --- | --- | --- | |  |  |  |  |  | | C39F7.4 | | | | | | |
|  | F32D8.4 | | WI5\_id:F32D8.4 |  | 1 | 108 | 0 | 4554 | 0.02337551 | 0.02337551 | 1 |
|  |  | RAFL11-12-H18 | At4g17530 / ras-related small GTP-binding protein RAB1c | |  |  |  |  |  | | --- | --- | --- | --- | --- | |  |  |  |  |  | | C39F7.4 | | | | | | |
| Cluster:2-0 | | |  |  | A | B | C | D | P | P' | N |
|  | T24B8.1 | | WI5\_id:T24B8.1 |  | 4 | 146 | 13 | 4500 | 0.0017651725 | 0.008825863 | 5 |
|  |  | RAFL04-19-O24 | At5g02610 / 60S ribosomal protein L35 (RPL35D) | |  |  |  |  |  | | --- | --- | --- | --- | --- | |  |  |  |  |  | | ZK652.4 | | | | | | |
|  |  | RAFL06-10-L13 | At3g09630 / 60S ribosomal protein L4/L1 (RPL4A) | |  |  |  |  |  | | --- | --- | --- | --- | --- | |  |  |  |  |  | | B0041.4 | | | | | | |
|  |  | RAFL11-12-M17 | At3g25520 / 60S ribosomal protein L5 (RPL5A) | |  |  |  |  |  | | --- | --- | --- | --- | --- | |  |  |  |  |  | | F54C9.5 | | | | | | |
|  |  | RAFL03-06-H07 | At1g33140 / 60S ribosomal protein L9 (RPL90A/C) | |  |  |  |  |  | | --- | --- | --- | --- | --- | |  |  |  |  |  | | R13A5.8 | | | | | | |
|  | C09D4.5 | | WI5\_id:C09D4.5 |  | 4 | 146 | 14 | 4499 | 0.002213303 | 0.015493121 | 7 |
|  |  | RAFL04-19-O24 | At5g02610 / 60S ribosomal protein L35 (RPL35D) | |  |  |  |  |  | | --- | --- | --- | --- | --- | |  |  |  |  |  | | ZK652.4 | | | | | | |
|  |  | RAFL06-10-L13 | At3g09630 / 60S ribosomal protein L4/L1 (RPL4A) | |  |  |  |  |  | | --- | --- | --- | --- | --- | |  |  |  |  |  | | B0041.4 | | | | | | |
|  |  | RAFL11-12-M17 | At3g25520 / 60S ribosomal protein L5 (RPL5A) | |  |  |  |  |  | | --- | --- | --- | --- | --- | |  |  |  |  |  | | F54C9.5 | | | | | | |
|  |  | RAFL03-06-H07 | At1g33140 / 60S ribosomal protein L9 (RPL90A/C) | |  |  |  |  |  | | --- | --- | --- | --- | --- | |  |  |  |  |  | | R13A5.8 | | | | | | |
|  | F13B10.2 | | WI5\_id:F13B10.2 |  | 4 | 146 | 15 | 4498 | 0.002734157 | 0.016404942 | 6 |
|  |  | RAFL04-19-O24 | At5g02610 / 60S ribosomal protein L35 (RPL35D) | |  |  |  |  |  | | --- | --- | --- | --- | --- | |  |  |  |  |  | | ZK652.4 | | | | | | |
|  |  | RAFL06-10-L13 | At3g09630 / 60S ribosomal protein L4/L1 (RPL4A) | |  |  |  |  |  | | --- | --- | --- | --- | --- | |  |  |  |  |  | | B0041.4 | | | | | | |
|  |  | RAFL11-12-M17 | At3g25520 / 60S ribosomal protein L5 (RPL5A) | |  |  |  |  |  | | --- | --- | --- | --- | --- | |  |  |  |  |  | | F54C9.5 | | | | | | |
|  |  | RAFL03-06-H07 | At1g33140 / 60S ribosomal protein L9 (RPL90A/C) | |  |  |  |  |  | | --- | --- | --- | --- | --- | |  |  |  |  |  | | R13A5.8 | | | | | | |
|  | F10B5.1 | | WI5\_id:F10B5.1 |  | 4 | 146 | 15 | 4498 | 0.002734157 | 0.016404942 | 6 |
|  |  | RAFL04-19-O24 | At5g02610 / 60S ribosomal protein L35 (RPL35D) | |  |  |  |  |  | | --- | --- | --- | --- | --- | |  |  |  |  |  | | ZK652.4 | | | | | | |
|  |  | RAFL06-10-L13 | At3g09630 / 60S ribosomal protein L4/L1 (RPL4A) | |  |  |  |  |  | | --- | --- | --- | --- | --- | |  |  |  |  |  | | B0041.4 | | | | | | |
|  |  | RAFL11-12-M17 | At3g25520 / 60S ribosomal protein L5 (RPL5A) | |  |  |  |  |  | | --- | --- | --- | --- | --- | |  |  |  |  |  | | F54C9.5 | | | | | | |
|  |  | RAFL03-06-H07 | At1g33140 / 60S ribosomal protein L9 (RPL90A/C) | |  |  |  |  |  | | --- | --- | --- | --- | --- | |  |  |  |  |  | | R13A5.8 | | | | | | |
|  | B0250.1 | | WI5\_id:B0250.1 |  | 4 | 146 | 15 | 4498 | 0.002734157 | 0.016404942 | 6 |
|  |  | RAFL04-19-O24 | At5g02610 / 60S ribosomal protein L35 (RPL35D) | |  |  |  |  |  | | --- | --- | --- | --- | --- | |  |  |  |  |  | | ZK652.4 | | | | | | |
|  |  | RAFL06-10-L13 | At3g09630 / 60S ribosomal protein L4/L1 (RPL4A) | |  |  |  |  |  | | --- | --- | --- | --- | --- | |  |  |  |  |  | | B0041.4 | | | | | | |
|  |  | RAFL11-12-M17 | At3g25520 / 60S ribosomal protein L5 (RPL5A) | |  |  |  |  |  | | --- | --- | --- | --- | --- | |  |  |  |  |  | | F54C9.5 | | | | | | |
|  |  | RAFL03-06-H07 | At1g33140 / 60S ribosomal protein L9 (RPL90A/C) | |  |  |  |  |  | | --- | --- | --- | --- | --- | |  |  |  |  |  | | R13A5.8  Ribosomal protein L9 | | | | | | |
|  | F28C6.7 | | WI5\_id:F28C6.7 |  | 4 | 146 | 15 | 4498 | 0.002734157 | 0.016404942 | 6 |
|  |  | RAFL04-19-O24 | At5g02610 / 60S ribosomal protein L35 (RPL35D) | |  |  |  |  |  | | --- | --- | --- | --- | --- | |  |  |  |  |  | | ZK652.4 | | | | | | |
|  |  | RAFL06-10-L13 | At3g09630 / 60S ribosomal protein L4/L1 (RPL4A) | |  |  |  |  |  | | --- | --- | --- | --- | --- | |  |  |  |  |  | | B0041.4 | | | | | | |
|  |  | RAFL11-12-M17 | At3g25520 / 60S ribosomal protein L5 (RPL5A) | |  |  |  |  |  | | --- | --- | --- | --- | --- | |  |  |  |  |  | | F54C9.5 | | | | | | |
|  |  | RAFL03-06-H07 | At1g33140 / 60S ribosomal protein L9 (RPL90A/C) | |  |  |  |  |  | | --- | --- | --- | --- | --- | |  |  |  |  |  | | R13A5.8 | | | | | | |
|  | Y37E3.8 | | WI5\_id:Y37E3.8 |  | 4 | 146 | 15 | 4498 | 0.002734157 | 0.016404942 | 6 |
|  |  | RAFL04-19-O24 | At5g02610 / 60S ribosomal protein L35 (RPL35D) | |  |  |  |  |  | | --- | --- | --- | --- | --- | |  |  |  |  |  | | ZK652.4 | | | | | | |
|  |  | RAFL06-10-L13 | At3g09630 / 60S ribosomal protein L4/L1 (RPL4A) | |  |  |  |  |  | | --- | --- | --- | --- | --- | |  |  |  |  |  | | B0041.4 | | | | | | |
|  |  | RAFL11-12-M17 | At3g25520 / 60S ribosomal protein L5 (RPL5A) | |  |  |  |  |  | | --- | --- | --- | --- | --- | |  |  |  |  |  | | F54C9.5 | | | | | | |
|  |  | RAFL03-06-H07 | At1g33140 / 60S ribosomal protein L9 (RPL90A/C) | |  |  |  |  |  | | --- | --- | --- | --- | --- | |  |  |  |  |  | | R13A5.8 | | | | | | |
|  | ZK652.4 | | WI5\_id:ZK652.4 |  | 4 | 146 | 15 | 4498 | 0.002734157 | 0.016404942 | 6 |
|  |  | RAFL04-19-O24 | At5g02610 / 60S ribosomal protein L35 (RPL35D) | |  |  |  |  |  | | --- | --- | --- | --- | --- | |  |  |  |  |  | | ZK652.4 | | | | | | |
|  |  | RAFL06-10-L13 | At3g09630 / 60S ribosomal protein L4/L1 (RPL4A) | |  |  |  |  |  | | --- | --- | --- | --- | --- | |  |  |  |  |  | | B0041.4 | | | | | | |
|  |  | RAFL11-12-M17 | At3g25520 / 60S ribosomal protein L5 (RPL5A) | |  |  |  |  |  | | --- | --- | --- | --- | --- | |  |  |  |  |  | | F54C9.5 | | | | | | |
|  |  | RAFL03-06-H07 | At1g33140 / 60S ribosomal protein L9 (RPL90A/C) | |  |  |  |  |  | | --- | --- | --- | --- | --- | |  |  |  |  |  | | R13A5.8 | | | | | | |
|  | F52B5.6 | | WI5\_id:F52B5.6 |  | 4 | 146 | 16 | 4497 | 0.0033332263 | 0.019999357 | 6 |
|  |  | RAFL04-19-O24 | At5g02610 / 60S ribosomal protein L35 (RPL35D) | |  |  |  |  |  | | --- | --- | --- | --- | --- | |  |  |  |  |  | | ZK652.4 | | | | | | |
|  |  | RAFL06-10-L13 | At3g09630 / 60S ribosomal protein L4/L1 (RPL4A) | |  |  |  |  |  | | --- | --- | --- | --- | --- | |  |  |  |  |  | | B0041.4 | | | | | | |
|  |  | RAFL11-12-M17 | At3g25520 / 60S ribosomal protein L5 (RPL5A) | |  |  |  |  |  | | --- | --- | --- | --- | --- | |  |  |  |  |  | | F54C9.5 | | | | | | |
|  |  | RAFL03-06-H07 | At1g33140 / 60S ribosomal protein L9 (RPL90A/C) | |  |  |  |  |  | | --- | --- | --- | --- | --- | |  |  |  |  |  | | R13A5.8 | | | | | | |
|  | F54C9.5 | | WI5\_id:F54C9.5 |  | 4 | 146 | 17 | 4496 | 0.0040158513 | 0.028110959 | 7 |
|  |  | RAFL04-19-O24 | At5g02610 / 60S ribosomal protein L35 (RPL35D) | |  |  |  |  |  | | --- | --- | --- | --- | --- | |  |  |  |  |  | | ZK652.4 | | | | | | |
|  |  | RAFL06-10-L13 | At3g09630 / 60S ribosomal protein L4/L1 (RPL4A) | |  |  |  |  |  | | --- | --- | --- | --- | --- | |  |  |  |  |  | | B0041.4 | | | | | | |
|  |  | RAFL11-12-M17 | At3g25520 / 60S ribosomal protein L5 (RPL5A) | |  |  |  |  |  | | --- | --- | --- | --- | --- | |  |  |  |  |  | | F54C9.5 | | | | | | |
|  |  | RAFL03-06-H07 | At1g33140 / 60S ribosomal protein L9 (RPL90A/C) | |  |  |  |  |  | | --- | --- | --- | --- | --- | |  |  |  |  |  | | R13A5.8 | | | | | | |
|  | M01F1.2 | | WI5\_id:M01F1.2 |  | 3 | 147 | 9 | 4504 | 0.0057971687 | 0.03478301 | 6 |
|  |  | RAFL04-19-O24 | At5g02610 / 60S ribosomal protein L35 (RPL35D) | |  |  |  |  |  | | --- | --- | --- | --- | --- | |  |  |  |  |  | | ZK652.4 | | | | | | |
|  |  | RAFL11-12-M17 | At3g25520 / 60S ribosomal protein L5 (RPL5A) | |  |  |  |  |  | | --- | --- | --- | --- | --- | |  |  |  |  |  | | F54C9.5 | | | | | | |
|  |  | RAFL05-01-F21 | At3g05590 / 60S ribosomal protein L18 (RPL18B) | |  |  |  |  |  | | --- | --- | --- | --- | --- | |  |  |  |  |  | | Y45F10D.12  Eukaryotic ribosomal protein L18 | | | | | | |
|  | F38A6.1 | | WI5\_id:F38A6.1 |  | 2 | 148 | 2 | 4511 | 0.00591059 | 0.017731769 | 3 |
|  |  | RAFL07-09-P18 | At4g31700 / 40S ribosomal protein S6 (RPS6A) | |  |  |  |  |  | | --- | --- | --- | --- | --- | |  |  |  |  |  | | Y71A12B.1 | | | | | | |
|  |  | RAFL06-08-H02 | At3g04840 / 40S ribosomal protein S3A (RPS3aA) | |  |  |  |  |  | | --- | --- | --- | --- | --- | |  |  |  |  |  | | F56F3.5 | | | | | | |
|  | R10E11.2 | | WI5\_id:R10E11.2 |  | 2 | 148 | 2 | 4511 | 0.00591059 | 0.01182118 | 2 |
|  |  | RAFL03-08-O03 | At1g07940 / elongation factor 1-alpha (EF-1-alpha) | |  |  |  |  |  | | --- | --- | --- | --- | --- | |  |  |  |  |  | | F31E3.5 | | | | | | |
|  |  | RAFL03-06-H07 | At1g33140 / 60S ribosomal protein L9 (RPL90A/C) | |  |  |  |  |  | | --- | --- | --- | --- | --- | |  |  |  |  |  | | R13A5.8 | | | | | | |
|  | F08G2.5 | | WI5\_id:F08G2.5 |  | 2 | 148 | 2 | 4511 | 0.00591059 | 0.017731769 | 3 |
|  |  | RAFL06-10-L13 | At3g09630 / 60S ribosomal protein L4/L1 (RPL4A) | |  |  |  |  |  | | --- | --- | --- | --- | --- | |  |  |  |  |  | | B0041.4 | | | | | | |
|  |  | RAFL03-08-O03 | At1g07940 / elongation factor 1-alpha (EF-1-alpha) | |  |  |  |  |  | | --- | --- | --- | --- | --- | |  |  |  |  |  | | F31E3.5 | | | | | | |
|  | B0041.4 | | WI5\_id:B0041.4 |  | 3 | 147 | 10 | 4503 | 0.0073604016 | 0.04416241 | 6 |
|  |  | RAFL04-19-O24 | At5g02610 / 60S ribosomal protein L35 (RPL35D) | |  |  |  |  |  | | --- | --- | --- | --- | --- | |  |  |  |  |  | | ZK652.4  60S ribosomal protein L35 | | | | | | |
|  |  | RAFL06-10-L13 | At3g09630 / 60S ribosomal protein L4/L1 (RPL4A) | |  |  |  |  |  | | --- | --- | --- | --- | --- | |  |  |  |  |  | | B0041.4  ribosomal protein L1 | | | | | | |
|  |  | RAFL11-12-M17 | At3g25520 / 60S ribosomal protein L5 (RPL5A) | |  |  |  |  |  | | --- | --- | --- | --- | --- | |  |  |  |  |  | | F54C9.5 | | | | | | |
|  | R13A5.8 | | WI5\_id:R13A5.8 |  | 3 | 147 | 10 | 4503 | 0.0073604016 | 0.04416241 | 6 |
|  |  | RAFL04-19-O24 | At5g02610 / 60S ribosomal protein L35 (RPL35D) | |  |  |  |  |  | | --- | --- | --- | --- | --- | |  |  |  |  |  | | ZK652.4 | | | | | | |
|  |  | RAFL11-12-M17 | At3g25520 / 60S ribosomal protein L5 (RPL5A) | |  |  |  |  |  | | --- | --- | --- | --- | --- | |  |  |  |  |  | | F54C9.5 | | | | | | |
|  |  | RAFL03-06-H07 | At1g33140 / 60S ribosomal protein L9 (RPL90A/C) | |  |  |  |  |  | | --- | --- | --- | --- | --- | |  |  |  |  |  | | R13A5.8 | | | | | | |
|  | T01G9.5 | | WI5\_id:T01G9.5 |  | 2 | 148 | 3 | 4510 | 0.009643516 | 0.038574062 | 4 |
|  |  | RAFL06-10-L13 | At3g09630 / 60S ribosomal protein L4/L1 (RPL4A) | |  |  |  |  |  | | --- | --- | --- | --- | --- | |  |  |  |  |  | | B0041.4 | | | | | | |
|  |  | RAFL05-01-F21 | At3g05590 / 60S ribosomal protein L18 (RPL18B) | |  |  |  |  |  | | --- | --- | --- | --- | --- | |  |  |  |  |  | | Y45F10D.12 | | | | | | |
|  | C16A3.9 | | WI5\_id:C16A3.9 |  | 2 | 148 | 3 | 4510 | 0.009643516 | 0.028930547 | 3 |
|  |  | RAFL06-08-H02 | At3g04840 / 40S ribosomal protein S3A (RPS3aA) | |  |  |  |  |  | | --- | --- | --- | --- | --- | |  |  |  |  |  | | F56F3.5 | | | | | | |
|  |  | RAFL03-06-H09 | At3g60770 / 40S ribosomal protein S13 (RPS13A) | |  |  |  |  |  | | --- | --- | --- | --- | --- | |  |  |  |  |  | | C16A3.9  40S ribosomal protein S13 | | | | | | |
|  | F56F3.5 | | WI5\_id:F56F3.5 |  | 2 | 148 | 3 | 4510 | 0.009643516 | 0.028930547 | 3 |
|  |  | RAFL06-08-H02 | At3g04840 / 40S ribosomal protein S3A (RPS3aA) | |  |  |  |  |  | | --- | --- | --- | --- | --- | |  |  |  |  |  | | F56F3.5 | | | | | | |
|  |  | RAFL03-06-H09 | At3g60770 / 40S ribosomal protein S13 (RPS13A) | |  |  |  |  |  | | --- | --- | --- | --- | --- | |  |  |  |  |  | | C16A3.9 | | | | | | |
|  | T09A5.10 | | WI5\_id:T09A5.10 |  | 2 | 148 | 3 | 4510 | 0.009643516 | 0.028930547 | 3 |
|  |  | RAFL06-10-L13 | At3g09630 / 60S ribosomal protein L4/L1 (RPL4A) | |  |  |  |  |  | | --- | --- | --- | --- | --- | |  |  |  |  |  | | B0041.4 | | | | | | |
|  |  | RAFL04-20-D09 | At5g42020 / luminal binding protein 2 precursor (BiP-2) (AtBP2) | |  |  |  |  |  | | --- | --- | --- | --- | --- | |  |  |  |  |  | | F43E2.8  heat shock protein | | | | | | |
|  | Y51H4A.17 | | WI5\_id:Y51H4A.17 |  | 2 | 148 | 4 | 4509 | 0.014161414 | 0.04248424 | 3 |
|  |  | RAFL06-10-L13 | At3g09630 / 60S ribosomal protein L4/L1 (RPL4A) | |  |  |  |  |  | | --- | --- | --- | --- | --- | |  |  |  |  |  | | B0041.4 | | | | | | |
|  |  | RAFL04-20-D09 | At5g42020 / luminal binding protein 2 precursor (BiP-2) (AtBP2) | |  |  |  |  |  | | --- | --- | --- | --- | --- | |  |  |  |  |  | | F43E2.8 | | | | | | |
|  | C23G10.4 | | WI5\_id:C23G10.4 |  | 2 | 148 | 5 | 4508 | 0.019410595 | 0.09705298 | 5 |
|  |  | RAFL04-20-D09 | At5g42020 / luminal binding protein 2 precursor (BiP-2) (AtBP2) | |  |  |  |  |  | | --- | --- | --- | --- | --- | |  |  |  |  |  | | C15H9.6 | | | | | | |
|  |  | RAFL03-08-O03 | At1g07940 / elongation factor 1-alpha (EF-1-alpha) | |  |  |  |  |  | | --- | --- | --- | --- | --- | |  |  |  |  |  | | F31E3.5 | | | | | | |
|  | R09B5.5 | | WI5\_id:R09B5.5 |  | 2 | 148 | 6 | 4507 | 0.025339978 | 0.10135991 | 4 |
|  |  | RAFL11-12-M17 | At3g25520 / 60S ribosomal protein L5 (RPL5A) | |  |  |  |  |  | | --- | --- | --- | --- | --- | |  |  |  |  |  | | F54C9.5 | | | | | | |
|  |  | RAFL03-08-O03 | At1g07940 / elongation factor 1-alpha (EF-1-alpha) | |  |  |  |  |  | | --- | --- | --- | --- | --- | |  |  |  |  |  | | F31E3.5 | | | | | | |
|  | K08E3.7 | | WI5\_id:K08E3.7 |  | 2 | 148 | 7 | 4506 | 0.031900965 | 0.15950483 | 5 |
|  |  | RAFL08-12-D04 | At5g20290 / 40S ribosomal protein S8 (RPS8A) | |  |  |  |  |  | | --- | --- | --- | --- | --- | |  |  |  |  |  | | F42C5.8 | | | | | | |
|  |  | RAFL04-19-F02 | At4g20890 / tubulin beta-9 chain (TUB9) | |  |  |  |  |  | | --- | --- | --- | --- | --- | |  |  |  |  |  | | C36E8.5  beta tubulin | | | | | | |
|  | F42C5.8 | | WI5\_id:F42C5.8 |  | 1 | 149 | 0 | 4513 | 0.03216813 | 0.03216813 | 1 |
|  |  | RAFL08-12-D04 | At5g20290 / 40S ribosomal protein S8 (RPS8A) | |  |  |  |  |  | | --- | --- | --- | --- | --- | |  |  |  |  |  | | F42C5.8 | | | | | | |
|  | C09G4.3 | | WI5\_id:C09G4.3 |  | 1 | 149 | 0 | 4513 | 0.03216813 | 0.03216813 | 1 |
|  |  | RAFL03-08-O03 | At1g07940 / elongation factor 1-alpha (EF-1-alpha) | |  |  |  |  |  | | --- | --- | --- | --- | --- | |  |  |  |  |  | | F31E3.5 | | | | | | |
|  | F59A2.3 | | WI5\_id:F59A2.3 |  | 1 | 149 | 0 | 4513 | 0.03216813 | 0.03216813 | 1 |
|  |  | RAFL08-12-D04 | At5g20290 / 40S ribosomal protein S8 (RPS8A) | |  |  |  |  |  | | --- | --- | --- | --- | --- | |  |  |  |  |  | | F42C5.8 | | | | | | |
|  | T24H10.3 | | WI5\_id:T24H10.3 |  | 1 | 149 | 0 | 4513 | 0.03216813 | 0.03216813 | 1 |
|  |  | RAFL05-02-F20 | At5g65360 / histone H3 | |  |  |  |  |  | | --- | --- | --- | --- | --- | |  |  |  |  |  | | Y49E10.6 | | | | | | |
|  | F59A6.1 | | WI5\_id:F59A6.1 |  | 1 | 149 | 0 | 4513 | 0.03216813 | 0.03216813 | 1 |
|  |  | RAFL03-08-O03 | At1g07940 / elongation factor 1-alpha (EF-1-alpha) | |  |  |  |  |  | | --- | --- | --- | --- | --- | |  |  |  |  |  | | F31E3.5 | | | | | | |
|  | R02F11.1 | | WI5\_id:R02F11.1 |  | 1 | 149 | 0 | 4513 | 0.03216813 | 0.03216813 | 1 |
|  |  | RAFL03-08-O03 | At1g07940 / elongation factor 1-alpha (EF-1-alpha) | |  |  |  |  |  | | --- | --- | --- | --- | --- | |  |  |  |  |  | | F31E3.5  Elongation factor 1-alpha | | | | | | |
|  | C35B8.2 | | WI5\_id:C35B8.2 |  | 1 | 149 | 0 | 4513 | 0.03216813 | 0.03216813 | 1 |
|  |  | RAFL03-08-O03 | At1g07940 / elongation factor 1-alpha (EF-1-alpha) | |  |  |  |  |  | | --- | --- | --- | --- | --- | |  |  |  |  |  | | F31E3.5 | | | | | | |
|  | F58F6.4 | | WI5\_id:F58F6.4 |  | 1 | 149 | 0 | 4513 | 0.03216813 | 0.03216813 | 1 |
|  |  | RAFL08-12-D04 | At5g20290 / 40S ribosomal protein S8 (RPS8A) | |  |  |  |  |  | | --- | --- | --- | --- | --- | |  |  |  |  |  | | F42C5.8  40S ribosomal protein S8 | | | | | | |
|  | Y75B8A.2 | | WI5\_id:Y75B8A.2 |  | 1 | 149 | 0 | 4513 | 0.03216813 | 0.03216813 | 1 |
|  |  | RAFL05-01-F21 | At3g05590 / 60S ribosomal protein L18 (RPL18B) | |  |  |  |  |  | | --- | --- | --- | --- | --- | |  |  |  |  |  | | Y45F10D.12 | | | | | | |
|  | C56G2.7 | | WI5\_id:C56G2.7 |  | 1 | 149 | 0 | 4513 | 0.03216813 | 0.03216813 | 1 |
|  |  | RAFL05-01-F21 | At3g05590 / 60S ribosomal protein L18 (RPL18B) | |  |  |  |  |  | | --- | --- | --- | --- | --- | |  |  |  |  |  | | Y45F10D.12 | | | | | | |
|  | F45E1.6 | | WI5\_id:F45E1.6 |  | 1 | 149 | 0 | 4513 | 0.03216813 | 0.03216813 | 1 |
|  |  | RAFL05-02-F20 | At5g65360 / histone H3 | |  |  |  |  |  | | --- | --- | --- | --- | --- | |  |  |  |  |  | | F45E1.6  Histone H3 | | | | | | |
|  | C04A11.1 | | WI5\_id:C04A11.1 |  | 1 | 149 | 0 | 4513 | 0.03216813 | 0.03216813 | 1 |
|  |  | RAFL05-02-F20 | At5g65360 / histone H3 | |  |  |  |  |  | | --- | --- | --- | --- | --- | |  |  |  |  |  | | F45E1.6 | | | | | | |
|  | C04F12.9 | | WI5\_id:C04F12.9 |  | 1 | 149 | 0 | 4513 | 0.03216813 | 0.03216813 | 1 |
|  |  | RAFL05-02-F20 | At5g65360 / histone H3 | |  |  |  |  |  | | --- | --- | --- | --- | --- | |  |  |  |  |  | | Y49E10.6  Core histone H2A/H2B/H3/H4 | | | | | | |
|  | Y49E10.6 | | WI5\_id:Y49E10.6 |  | 1 | 149 | 0 | 4513 | 0.03216813 | 0.03216813 | 1 |
|  |  | RAFL05-02-F20 | At5g65360 / histone H3 | |  |  |  |  |  | | --- | --- | --- | --- | --- | |  |  |  |  |  | | Y49E10.6 | | | | | | |
|  | C07E3.7 | | WI5\_id:C07E3.7 |  | 1 | 149 | 0 | 4513 | 0.03216813 | 0.03216813 | 1 |
|  |  | RAFL05-01-F21 | At3g05590 / 60S ribosomal protein L18 (RPL18B) | |  |  |  |  |  | | --- | --- | --- | --- | --- | |  |  |  |  |  | | Y45F10D.12 | | | | | | |
|  | Y45F10D.12 | | WI5\_id:Y45F10D.12 |  | 1 | 149 | 0 | 4513 | 0.03216813 | 0.03216813 | 1 |
|  |  | RAFL05-01-F21 | At3g05590 / 60S ribosomal protein L18 (RPL18B) | |  |  |  |  |  | | --- | --- | --- | --- | --- | |  |  |  |  |  | | Y45F10D.12 | | | | | | |
|  | F58A4.8 | | WI5\_id:F58A4.8 |  | 2 | 148 | 8 | 4505 | 0.039047353 | 0.19523676 | 5 |
|  |  | RAFL03-06-H09 | At3g60770 / 40S ribosomal protein S13 (RPS13A) | |  |  |  |  |  | | --- | --- | --- | --- | --- | |  |  |  |  |  | | C16A3.9 | | | | | | |
|  |  | RAFL04-19-F02 | At4g20890 / tubulin beta-9 chain (TUB9) | |  |  |  |  |  | | --- | --- | --- | --- | --- | |  |  |  |  |  | | C36E8.5 | | | | | | |
|  | T22F3.4 | | WI5\_id:T22F3.4 |  | 2 | 148 | 9 | 4504 | 0.046735216 | 0.2804113 | 6 |
|  |  | RAFL04-19-O24 | At5g02610 / 60S ribosomal protein L35 (RPL35D) | |  |  |  |  |  | | --- | --- | --- | --- | --- | |  |  |  |  |  | | ZK652.4 | | | | | | |
|  |  | RAFL11-12-M17 | At3g25520 / 60S ribosomal protein L5 (RPL5A) | |  |  |  |  |  | | --- | --- | --- | --- | --- | |  |  |  |  |  | | F54C9.5 | | | | | | |
|  | JC8.3 | | WI5\_id:JC8.3 |  | 2 | 148 | 9 | 4504 | 0.046735216 | 0.2804113 | 6 |
|  |  | RAFL04-19-O24 | At5g02610 / 60S ribosomal protein L35 (RPL35D) | |  |  |  |  |  | | --- | --- | --- | --- | --- | |  |  |  |  |  | | ZK652.4 | | | | | | |
|  |  | RAFL11-12-M17 | At3g25520 / 60S ribosomal protein L5 (RPL5A) | |  |  |  |  |  | | --- | --- | --- | --- | --- | |  |  |  |  |  | | F54C9.5 | | | | | | |
|  | Y48G8AL.8 | | WI5\_id:Y48G8AL.8 |  | 2 | 148 | 9 | 4504 | 0.046735216 | 0.2804113 | 6 |
|  |  | RAFL04-19-O24 | At5g02610 / 60S ribosomal protein L35 (RPL35D) | |  |  |  |  |  | | --- | --- | --- | --- | --- | |  |  |  |  |  | | ZK652.4 | | | | | | |
|  |  | RAFL11-12-M17 | At3g25520 / 60S ribosomal protein L5 (RPL5A) | |  |  |  |  |  | | --- | --- | --- | --- | --- | |  |  |  |  |  | | F54C9.5 | | | | | | |
| Cluster:5-1 | | |  |  | A | B | C | D | P | P' | N |
|  | F35G12.9 | | WI5\_id:F35G12.9 |  | 2 | 280 | 2 | 4379 | 0.02015854 | 0.06047562 | 3 |
|  |  | RAFL05-08-F18 | At3g56090 / expressed protein | |  |  |  |  |  | | --- | --- | --- | --- | --- | |  |  |  |  |  | | C54F6.14 | | | | | | |
|  |  | RAFL07-12-N15 | At2g20000 / cell division cycle (CDC) protein - related | |  |  |  |  |  | | --- | --- | --- | --- | --- | |  |  |  |  |  | | Y110A7A.17 | | | | | | |
| Cluster:4-2 | | |  |  | A | B | C | D | P | P' | N |
|  | C23G10.3 | | WI5\_id:C23G10.3 |  | 4 | 131 | 14 | 4514 | 0.0014992869 | 0.0074964343 | 5 |
|  |  | RAFL05-07-M02 | At1g34030 / 40S ribosomal protein S18 (RPS18B) | |  |  |  |  |  | | --- | --- | --- | --- | --- | |  |  |  |  |  | | Y57G11C.16  ribosomal protein S13 | | | | | | |
|  |  | RAFL09-17-F16 | At1g07770 / 40S ribosomal protein S15A (RPS15aA) | |  |  |  |  |  | | --- | --- | --- | --- | --- | |  |  |  |  |  | | F53A3.3  40S ribosomal protein | | | | | | |
|  |  | RAFL11-02-D23 | At1g07770 / 40S ribosomal protein S15A (RPS15aA) | |  |  |  |  |  | | --- | --- | --- | --- | --- | |  |  |  |  |  | | F53A3.3  40S ribosomal protein | | | | | | |
|  |  | RAFL05-08-B08 | At5g18380 / 40S ribosomal protein S16 (RPS16C) | |  |  |  |  |  | | --- | --- | --- | --- | --- | |  |  |  |  |  | | T01C3.6  40S ribosomal protein S16 | | | | | | |
|  | F36A2.6 | | WI5\_id:F36A2.6 |  | 4 | 131 | 14 | 4514 | 0.0014992869 | 0.0074964343 | 5 |
|  |  | RAFL05-07-M02 | At1g34030 / 40S ribosomal protein S18 (RPS18B) | |  |  |  |  |  | | --- | --- | --- | --- | --- | |  |  |  |  |  | | Y57G11C.16 | | | | | | |
|  |  | RAFL09-17-F16 | At1g07770 / 40S ribosomal protein S15A (RPS15aA) | |  |  |  |  |  | | --- | --- | --- | --- | --- | |  |  |  |  |  | | F53A3.3 | | | | | | |
|  |  | RAFL11-02-D23 | At1g07770 / 40S ribosomal protein S15A (RPS15aA) | |  |  |  |  |  | | --- | --- | --- | --- | --- | |  |  |  |  |  | | F53A3.3 | | | | | | |
|  |  | RAFL05-08-B08 | At5g18380 / 40S ribosomal protein S16 (RPS16C) | |  |  |  |  |  | | --- | --- | --- | --- | --- | |  |  |  |  |  | | T01C3.6 | | | | | | |
|  | C49H3.11 | | WI5\_id:C49H3.11 |  | 4 | 131 | 17 | 4511 | 0.002741287 | 0.016447721 | 6 |
|  |  | RAFL05-07-M02 | At1g34030 / 40S ribosomal protein S18 (RPS18B) | |  |  |  |  |  | | --- | --- | --- | --- | --- | |  |  |  |  |  | | Y57G11C.16 | | | | | | |
|  |  | RAFL09-17-F16 | At1g07770 / 40S ribosomal protein S15A (RPS15aA) | |  |  |  |  |  | | --- | --- | --- | --- | --- | |  |  |  |  |  | | F53A3.3 | | | | | | |
|  |  | RAFL11-02-D23 | At1g07770 / 40S ribosomal protein S15A (RPS15aA) | |  |  |  |  |  | | --- | --- | --- | --- | --- | |  |  |  |  |  | | F53A3.3 | | | | | | |
|  |  | RAFL05-08-B08 | At5g18380 / 40S ribosomal protein S16 (RPS16C) | |  |  |  |  |  | | --- | --- | --- | --- | --- | |  |  |  |  |  | | T01C3.6 | | | | | | |
|  | T09B4.8 | | WI5\_id:T09B4.8 |  | 2 | 133 | 3 | 4525 | 0.007856598 | 0.031426392 | 4 |
|  |  | RAFL09-17-F16 | At1g07770 / 40S ribosomal protein S15A (RPS15aA) | |  |  |  |  |  | | --- | --- | --- | --- | --- | |  |  |  |  |  | | F53A3.3 | | | | | | |
|  |  | RAFL11-02-D23 | At1g07770 / 40S ribosomal protein S15A (RPS15aA) | |  |  |  |  |  | | --- | --- | --- | --- | --- | |  |  |  |  |  | | F53A3.3 | | | | | | |
|  | T05E11.1 | | WI5\_id:T05E11.1 |  | 3 | 132 | 14 | 4514 | 0.0119851455 | 0.059925728 | 5 |
|  |  | RAFL05-07-M02 | At1g34030 / 40S ribosomal protein S18 (RPS18B) | |  |  |  |  |  | | --- | --- | --- | --- | --- | |  |  |  |  |  | | Y57G11C.16 | | | | | | |
|  |  | RAFL09-17-F16 | At1g07770 / 40S ribosomal protein S15A (RPS15aA) | |  |  |  |  |  | | --- | --- | --- | --- | --- | |  |  |  |  |  | | F53A3.3 | | | | | | |
|  |  | RAFL11-02-D23 | At1g07770 / 40S ribosomal protein S15A (RPS15aA) | |  |  |  |  |  | | --- | --- | --- | --- | --- | |  |  |  |  |  | | F53A3.3 | | | | | | |
|  | Y105E8A.16 | | WI5\_id:Y105E8A.16 |  | 3 | 132 | 14 | 4514 | 0.0119851455 | 0.059925728 | 5 |
|  |  | RAFL05-07-M02 | At1g34030 / 40S ribosomal protein S18 (RPS18B) | |  |  |  |  |  | | --- | --- | --- | --- | --- | |  |  |  |  |  | | Y57G11C.16 | | | | | | |
|  |  | RAFL09-17-F16 | At1g07770 / 40S ribosomal protein S15A (RPS15aA) | |  |  |  |  |  | | --- | --- | --- | --- | --- | |  |  |  |  |  | | F53A3.3 | | | | | | |
|  |  | RAFL11-02-D23 | At1g07770 / 40S ribosomal protein S15A (RPS15aA) | |  |  |  |  |  | | --- | --- | --- | --- | --- | |  |  |  |  |  | | F53A3.3 | | | | | | |
|  | T07C4.1 | | WI5\_id:T07C4.1 |  | 2 | 133 | 5 | 4523 | 0.015882019 | 0.0794101 | 5 |
|  |  | RAFL09-17-F16 | At1g07770 / 40S ribosomal protein S15A (RPS15aA) | |  |  |  |  |  | | --- | --- | --- | --- | --- | |  |  |  |  |  | | F53A3.3 | | | | | | |
|  |  | RAFL11-02-D23 | At1g07770 / 40S ribosomal protein S15A (RPS15aA) | |  |  |  |  |  | | --- | --- | --- | --- | --- | |  |  |  |  |  | | F53A3.3 | | | | | | |
|  | Y43E12A.1 | | WI5\_id:Y43E12A.1 |  | 1 | 134 | 0 | 4528 | 0.028951319 | 0.028951319 | 1 |
|  |  | RAFL05-18-G17 | At3g48750 / cell division control protein 2 homolog A (CDC2A) | |  |  |  |  |  | | --- | --- | --- | --- | --- | |  |  |  |  |  | | T05G5.3 | | | | | | |
|  | K02F2.2 | | WI5\_id:K02F2.2 |  | 1 | 134 | 0 | 4528 | 0.028951319 | 0.028951319 | 1 |
|  |  | RAFL05-05-K13 | At3g52590 / ubiquitin extension protein 1 (UBQ1)/60S ribosomal protein L40 (RPL40B) | |  |  |  |  |  | | --- | --- | --- | --- | --- | |  |  |  |  |  | | ZK1010.1 | | | | | | |
|  | B0024.14 | | WI5\_id:B0024.14 |  | 1 | 134 | 0 | 4528 | 0.028951319 | 0.028951319 | 1 |
|  |  | RAFL04-20-K07 | At3g53750 / actin (ACT3) | |  |  |  |  |  | | --- | --- | --- | --- | --- | |  |  |  |  |  | | T04C12.6  actin | | | | | | |
|  | C16A3.8 | | WI5\_id:C16A3.8 |  | 1 | 134 | 0 | 4528 | 0.028951319 | 0.028951319 | 1 |
|  |  | RAFL04-20-K07 | At3g53750 / actin (ACT3) | |  |  |  |  |  | | --- | --- | --- | --- | --- | |  |  |  |  |  | | T04C12.6 | | | | | | |
|  | C18H9.7 | | WI5\_id:C18H9.7 |  | 1 | 134 | 0 | 4528 | 0.028951319 | 0.028951319 | 1 |
|  |  | RAFL05-07-M02 | At1g34030 / 40S ribosomal protein S18 (RPS18B) | |  |  |  |  |  | | --- | --- | --- | --- | --- | |  |  |  |  |  | | Y57G11C.16 | | | | | | |
|  | B0336.2 | | WI5\_id:B0336.2 |  | 1 | 134 | 0 | 4528 | 0.028951319 | 0.028951319 | 1 |
|  |  | RAFL05-12-C15 | At1g10630 / ADP-ribosylation factor, putative | |  |  |  |  |  | | --- | --- | --- | --- | --- | |  |  |  |  |  | | B0336.2 | | | | | | |
|  | R07H5.1 | | WI5\_id:R07H5.1 |  | 1 | 134 | 0 | 4528 | 0.028951319 | 0.028951319 | 1 |
|  |  | RAFL05-05-K13 | At3g52590 / ubiquitin extension protein 1 (UBQ1)/60S ribosomal protein L40 (RPL40B) | |  |  |  |  |  | | --- | --- | --- | --- | --- | |  |  |  |  |  | | ZK1010.1 | | | | | | |
|  | C34C6.6 | | WI5\_id:C34C6.6 |  | 1 | 134 | 0 | 4528 | 0.028951319 | 0.028951319 | 1 |
|  |  | RAFL05-05-K13 | At3g52590 / ubiquitin extension protein 1 (UBQ1)/60S ribosomal protein L40 (RPL40B) | |  |  |  |  |  | | --- | --- | --- | --- | --- | |  |  |  |  |  | | ZK1010.1 | | | | | | |
|  | C34C12.8 | | WI5\_id:C34C12.8 |  | 1 | 134 | 0 | 4528 | 0.028951319 | 0.028951319 | 1 |
|  |  | RAFL04-17-F17 | At4g37910 / heat shock protein mtHsc70-1 | |  |  |  |  |  | | --- | --- | --- | --- | --- | |  |  |  |  |  | | C37H5.8  heat shock 70 protein | | | | | | |
|  | C37H5.8 | | WI5\_id:C37H5.8 |  | 1 | 134 | 0 | 4528 | 0.028951319 | 0.028951319 | 1 |
|  |  | RAFL04-17-F17 | At4g37910 / heat shock protein mtHsc70-1 | |  |  |  |  |  | | --- | --- | --- | --- | --- | |  |  |  |  |  | | C37H5.8 | | | | | | |
|  | T06E6.2 | | WI5\_id:T06E6.2 |  | 1 | 134 | 0 | 4528 | 0.028951319 | 0.028951319 | 1 |
|  |  | RAFL05-18-G17 | At3g48750 / cell division control protein 2 homolog A (CDC2A) | |  |  |  |  |  | | --- | --- | --- | --- | --- | |  |  |  |  |  | | T05G5.3 | | | | | | |
|  | F37B4.7 | | WI5\_id:F37B4.7 |  | 1 | 134 | 0 | 4528 | 0.028951319 | 0.028951319 | 1 |
|  |  | RAFL04-20-K07 | At3g53750 / actin (ACT3) | |  |  |  |  |  | | --- | --- | --- | --- | --- | |  |  |  |  |  | | T04C12.6 | | | | | | |
|  | ZK1010.1 | | WI5\_id:ZK1010.1 |  | 1 | 134 | 0 | 4528 | 0.028951319 | 0.028951319 | 1 |
|  |  | RAFL05-05-K13 | At3g52590 / ubiquitin extension protein 1 (UBQ1)/60S ribosomal protein L40 (RPL40B) | |  |  |  |  |  | | --- | --- | --- | --- | --- | |  |  |  |  |  | | ZK1010.1 | | | | | | |
|  | F38B7.2 | | WI5\_id:F38B7.2 |  | 1 | 134 | 0 | 4528 | 0.028951319 | 0.028951319 | 1 |
|  |  | RAFL05-05-K13 | At3g52590 / ubiquitin extension protein 1 (UBQ1)/60S ribosomal protein L40 (RPL40B) | |  |  |  |  |  | | --- | --- | --- | --- | --- | |  |  |  |  |  | | ZK1010.1 | | | | | | |
|  | W05B5.1 | | WI5\_id:W05B5.1 |  | 1 | 134 | 0 | 4528 | 0.028951319 | 0.028951319 | 1 |
|  |  | RAFL05-05-K13 | At3g52590 / ubiquitin extension protein 1 (UBQ1)/60S ribosomal protein L40 (RPL40B) | |  |  |  |  |  | | --- | --- | --- | --- | --- | |  |  |  |  |  | | ZK1010.1 | | | | | | |
|  | F22B7.5 | | WI5\_id:F22B7.5 |  | 1 | 134 | 0 | 4528 | 0.028951319 | 0.028951319 | 1 |
|  |  | RAFL04-17-F17 | At4g37910 / heat shock protein mtHsc70-1 | |  |  |  |  |  | | --- | --- | --- | --- | --- | |  |  |  |  |  | | C37H5.8 | | | | | | |
|  | C43E11.10 | | WI5\_id:C43E11.10 |  | 1 | 134 | 0 | 4528 | 0.028951319 | 0.028951319 | 1 |
|  |  | RAFL05-18-G17 | At3g48750 / cell division control protein 2 homolog A (CDC2A) | |  |  |  |  |  | | --- | --- | --- | --- | --- | |  |  |  |  |  | | T05G5.3 | | | | | | |
|  | T05G5.3 | | WI5\_id:T05G5.3 |  | 1 | 134 | 0 | 4528 | 0.028951319 | 0.028951319 | 1 |
|  |  | RAFL05-18-G17 | At3g48750 / cell division control protein 2 homolog A (CDC2A) | |  |  |  |  |  | | --- | --- | --- | --- | --- | |  |  |  |  |  | | T05G5.3 | | | | | | |
|  | T25C8.2 | | WI5\_id:T25C8.2 |  | 1 | 134 | 0 | 4528 | 0.028951319 | 0.028951319 | 1 |
|  |  | RAFL04-20-K07 | At3g53750 / actin (ACT3) | |  |  |  |  |  | | --- | --- | --- | --- | --- | |  |  |  |  |  | | T04C12.6 | | | | | | |
|  | F15C11.2 | | WI5\_id:F15C11.2 |  | 1 | 134 | 0 | 4528 | 0.028951319 | 0.028951319 | 1 |
|  |  | RAFL05-05-K13 | At3g52590 / ubiquitin extension protein 1 (UBQ1)/60S ribosomal protein L40 (RPL40B) | |  |  |  |  |  | | --- | --- | --- | --- | --- | |  |  |  |  |  | | ZK1010.1 | | | | | | |
|  | C47E8.5 | | WI5\_id:C47E8.5 |  | 2 | 133 | 9 | 4519 | 0.03856442 | 0.26995096 | 7 |
|  |  | RAFL09-17-F16 | At1g07770 / 40S ribosomal protein S15A (RPS15aA) | |  |  |  |  |  | | --- | --- | --- | --- | --- | |  |  |  |  |  | | F53A3.3 | | | | | | |
|  |  | RAFL11-02-D23 | At1g07770 / 40S ribosomal protein S15A (RPS15aA) | |  |  |  |  |  | | --- | --- | --- | --- | --- | |  |  |  |  |  | | F53A3.3 | | | | | | |
|  | Y43B11AR.4 | | WI5\_id:Y43B11AR.4 |  | 2 | 133 | 10 | 4518 | 0.045415256 | 0.22707628 | 5 |
|  |  | RAFL09-17-F16 | At1g07770 / 40S ribosomal protein S15A (RPS15aA) | |  |  |  |  |  | | --- | --- | --- | --- | --- | |  |  |  |  |  | | F53A3.3 | | | | | | |
|  |  | RAFL11-02-D23 | At1g07770 / 40S ribosomal protein S15A (RPS15aA) | |  |  |  |  |  | | --- | --- | --- | --- | --- | |  |  |  |  |  | | F53A3.3 | | | | | | |
| Cluster:6-1 | | |  |  | A | B | C | D | P | P' | N |
|  | Y62E10A.14 | | WI5\_id:Y62E10A.14 |  | 2 | 313 | 0 | 4348 | 0.004549907 | 0.004549907 | 1 |
|  |  | RAFL05-08-I15 | At4g24820 / 26S proteasome regulatory subunit (RPN7), putative | |  |  |  |  |  | | --- | --- | --- | --- | --- | |  |  |  |  |  | | F49C12.8 | | | | | | |
|  |  | RAFL07-13-D20 | At4g24820 / 26S proteasome regulatory subunit (RPN7), putative | |  |  |  |  |  | | --- | --- | --- | --- | --- | |  |  |  |  |  | | F49C12.8 | | | | | | |
|  | Y54G11A.7 | | WI5\_id:Y54G11A.7 |  | 2 | 313 | 0 | 4348 | 0.004549907 | 0.004549907 | 1 |
|  |  | RAFL05-08-I15 | At4g24820 / 26S proteasome regulatory subunit (RPN7), putative | |  |  |  |  |  | | --- | --- | --- | --- | --- | |  |  |  |  |  | | F49C12.8 | | | | | | |
|  |  | RAFL07-13-D20 | At4g24820 / 26S proteasome regulatory subunit (RPN7), putative | |  |  |  |  |  | | --- | --- | --- | --- | --- | |  |  |  |  |  | | F49C12.8 | | | | | | |
|  | ZK930.3 | | WI5\_id:ZK930.3 |  | 2 | 313 | 1 | 4347 | 0.013038642 | 0.026077284 | 2 |
|  |  | RAFL06-12-E24 | At5g42790 / 20S proteasome alpha subunit F1 (PAF1) | |  |  |  |  |  | | --- | --- | --- | --- | --- | |  |  |  |  |  | | CD4.6 | | | | | | |
|  |  | RAFL11-07-B22 | At1g14650 / splicing factor -related | |  |  |  |  |  | | --- | --- | --- | --- | --- | |  |  |  |  |  | | W07E6.4  splicing factor | | | | | | |
|  | D1037.4 | | WI5\_id:D1037.4 |  | 2 | 313 | 1 | 4347 | 0.013038642 | 0.026077284 | 2 |
|  |  | RAFL09-13-A13 | At2g44100 / GDP dissociation inhibitor | |  |  |  |  |  | | --- | --- | --- | --- | --- | |  |  |  |  |  | | Y57G11C.10 | | | | | | |
|  |  | RAFL05-11-L05 | At2g44100 / GDP dissociation inhibitor | |  |  |  |  |  | | --- | --- | --- | --- | --- | |  |  |  |  |  | | Y57G11C.10 | | | | | | |
|  | Y57G11C.10 | | WI5\_id:Y57G11C.10 |  | 2 | 313 | 1 | 4347 | 0.013038642 | 0.026077284 | 2 |
|  |  | RAFL09-13-A13 | At2g44100 / GDP dissociation inhibitor | |  |  |  |  |  | | --- | --- | --- | --- | --- | |  |  |  |  |  | | Y57G11C.10 | | | | | | |
|  |  | RAFL05-11-L05 | At2g44100 / GDP dissociation inhibitor | |  |  |  |  |  | | --- | --- | --- | --- | --- | |  |  |  |  |  | | Y57G11C.10 | | | | | | |
|  | C30C11.2 | | WI5\_id:C30C11.2 |  | 3 | 312 | 6 | 4342 | 0.018891884 | 0.1133513 | 6 |
|  |  | RAFL05-08-I15 | At4g24820 / 26S proteasome regulatory subunit (RPN7), putative | |  |  |  |  |  | | --- | --- | --- | --- | --- | |  |  |  |  |  | | F49C12.8 | | | | | | |
|  |  | RAFL07-13-D20 | At4g24820 / 26S proteasome regulatory subunit (RPN7), putative | |  |  |  |  |  | | --- | --- | --- | --- | --- | |  |  |  |  |  | | F49C12.8 | | | | | | |
|  |  | RAFL11-04-H03 | At1g20200 / 26S proteasome regulatory subunit S3 (RPN3), putative | |  |  |  |  |  | | --- | --- | --- | --- | --- | |  |  |  |  |  | | C30C11.2 | | | | | | |
|  | F49C12.8 | | WI5\_id:F49C12.8 |  | 3 | 312 | 6 | 4342 | 0.018891884 | 0.1133513 | 6 |
|  |  | RAFL05-08-I15 | At4g24820 / 26S proteasome regulatory subunit (RPN7), putative | |  |  |  |  |  | | --- | --- | --- | --- | --- | |  |  |  |  |  | | F49C12.8 | | | | | | |
|  |  | RAFL07-13-D20 | At4g24820 / 26S proteasome regulatory subunit (RPN7), putative | |  |  |  |  |  | | --- | --- | --- | --- | --- | |  |  |  |  |  | | F49C12.8 | | | | | | |
|  |  | RAFL11-04-H03 | At1g20200 / 26S proteasome regulatory subunit S3 (RPN3), putative | |  |  |  |  |  | | --- | --- | --- | --- | --- | |  |  |  |  |  | | C30C11.2 | | | | | | |
|  | F23F12.6 | | WI5\_id:F23F12.6 |  | 4 | 311 | 12 | 4336 | 0.019434473 | 0.19434473 | 10 |
|  |  | RAFL08-19-N19 | At3g05530 / 26S proteasome AAA-ATPase subunit RPT5a | |  |  |  |  |  | | --- | --- | --- | --- | --- | |  |  |  |  |  | | F56H1.4 | | | | | | |
|  |  | RAFL05-08-I15 | At4g24820 / 26S proteasome regulatory subunit (RPN7), putative | |  |  |  |  |  | | --- | --- | --- | --- | --- | |  |  |  |  |  | | F49C12.8 | | | | | | |
|  |  | RAFL07-13-D20 | At4g24820 / 26S proteasome regulatory subunit (RPN7), putative | |  |  |  |  |  | | --- | --- | --- | --- | --- | |  |  |  |  |  | | F49C12.8 | | | | | | |
|  |  | RAFL11-04-H03 | At1g20200 / 26S proteasome regulatory subunit S3 (RPN3), putative | |  |  |  |  |  | | --- | --- | --- | --- | --- | |  |  |  |  |  | | C30C11.2 | | | | | | |
|  | F39H11.5 | | WI5\_id:F39H11.5 |  | 2 | 313 | 2 | 4346 | 0.024916494 | 0.074749485 | 3 |
|  |  | RAFL06-09-A21 | At5g40580 / 20S proteasome beta subunit B (PBB2) | |  |  |  |  |  | | --- | --- | --- | --- | --- | |  |  |  |  |  | | C47B2.4 | | | | | | |
|  |  | RAFL06-16-H22 | At1g16470 / 20S proteasome alpha subunit B (PAB1) | |  |  |  |  |  | | --- | --- | --- | --- | --- | |  |  |  |  |  | | D1054.2 | | | | | | |
|  | F19B10.1 | | WI5\_id:F19B10.1 |  | 2 | 313 | 2 | 4346 | 0.024916494 | 0.074749485 | 3 |
|  |  | RAFL05-12-M22 | At2g39760 / expressed protein | |  |  |  |  |  | | --- | --- | --- | --- | --- | |  |  |  |  |  | | ZK858.4 | | | | | | |
|  |  | RAFL11-04-H03 | At1g20200 / 26S proteasome regulatory subunit S3 (RPN3), putative | |  |  |  |  |  | | --- | --- | --- | --- | --- | |  |  |  |  |  | | C30C11.2 | | | | | | |
|  | C47D12.2 | | WI5\_id:C47D12.2 |  | 2 | 313 | 2 | 4346 | 0.024916494 | 0.074749485 | 3 |
|  |  | RAFL09-13-A13 | At2g44100 / GDP dissociation inhibitor | |  |  |  |  |  | | --- | --- | --- | --- | --- | |  |  |  |  |  | | Y57G11C.10 | | | | | | |
|  |  | RAFL05-11-L05 | At2g44100 / GDP dissociation inhibitor | |  |  |  |  |  | | --- | --- | --- | --- | --- | |  |  |  |  |  | | Y57G11C.10 | | | | | | |
|  | F10G7.8 | | WI5\_id:F10G7.8 |  | 4 | 311 | 14 | 4334 | 0.029376008 | 0.3525121 | 12 |
|  |  | RAFL08-19-N19 | At3g05530 / 26S proteasome AAA-ATPase subunit RPT5a | |  |  |  |  |  | | --- | --- | --- | --- | --- | |  |  |  |  |  | | F56H1.4  ATPase | | | | | | |
|  |  | RAFL05-08-I15 | At4g24820 / 26S proteasome regulatory subunit (RPN7), putative | |  |  |  |  |  | | --- | --- | --- | --- | --- | |  |  |  |  |  | | F49C12.8 | | | | | | |
|  |  | RAFL07-13-D20 | At4g24820 / 26S proteasome regulatory subunit (RPN7), putative | |  |  |  |  |  | | --- | --- | --- | --- | --- | |  |  |  |  |  | | F49C12.8 | | | | | | |
|  |  | RAFL11-04-H03 | At1g20200 / 26S proteasome regulatory subunit S3 (RPN3), putative | |  |  |  |  |  | | --- | --- | --- | --- | --- | |  |  |  |  |  | | C30C11.2 | | | | | | |
|  | F57B9.10 | | WI5\_id:F57B9.10 |  | 4 | 311 | 14 | 4334 | 0.029376008 | 0.3525121 | 12 |
|  |  | RAFL08-19-N19 | At3g05530 / 26S proteasome AAA-ATPase subunit RPT5a | |  |  |  |  |  | | --- | --- | --- | --- | --- | |  |  |  |  |  | | F56H1.4 | | | | | | |
|  |  | RAFL05-08-I15 | At4g24820 / 26S proteasome regulatory subunit (RPN7), putative | |  |  |  |  |  | | --- | --- | --- | --- | --- | |  |  |  |  |  | | F49C12.8 | | | | | | |
|  |  | RAFL07-13-D20 | At4g24820 / 26S proteasome regulatory subunit (RPN7), putative | |  |  |  |  |  | | --- | --- | --- | --- | --- | |  |  |  |  |  | | F49C12.8 | | | | | | |
|  |  | RAFL11-04-H03 | At1g20200 / 26S proteasome regulatory subunit S3 (RPN3), putative | |  |  |  |  |  | | --- | --- | --- | --- | --- | |  |  |  |  |  | | C30C11.2 | | | | | | |
|  | ZK20.5 | | WI5\_id:ZK20.5 |  | 4 | 311 | 14 | 4334 | 0.029376008 | 0.3525121 | 12 |
|  |  | RAFL08-19-N19 | At3g05530 / 26S proteasome AAA-ATPase subunit RPT5a | |  |  |  |  |  | | --- | --- | --- | --- | --- | |  |  |  |  |  | | F56H1.4 | | | | | | |
|  |  | RAFL05-08-I15 | At4g24820 / 26S proteasome regulatory subunit (RPN7), putative | |  |  |  |  |  | | --- | --- | --- | --- | --- | |  |  |  |  |  | | F49C12.8 | | | | | | |
|  |  | RAFL07-13-D20 | At4g24820 / 26S proteasome regulatory subunit (RPN7), putative | |  |  |  |  |  | | --- | --- | --- | --- | --- | |  |  |  |  |  | | F49C12.8 | | | | | | |
|  |  | RAFL11-04-H03 | At1g20200 / 26S proteasome regulatory subunit S3 (RPN3), putative | |  |  |  |  |  | | --- | --- | --- | --- | --- | |  |  |  |  |  | | C30C11.2 | | | | | | |
|  | C52E4.4 | | WI5\_id:C52E4.4 |  | 4 | 311 | 14 | 4334 | 0.029376008 | 0.3525121 | 12 |
|  |  | RAFL08-19-N19 | At3g05530 / 26S proteasome AAA-ATPase subunit RPT5a | |  |  |  |  |  | | --- | --- | --- | --- | --- | |  |  |  |  |  | | F56H1.4 | | | | | | |
|  |  | RAFL05-08-I15 | At4g24820 / 26S proteasome regulatory subunit (RPN7), putative | |  |  |  |  |  | | --- | --- | --- | --- | --- | |  |  |  |  |  | | F49C12.8 | | | | | | |
|  |  | RAFL07-13-D20 | At4g24820 / 26S proteasome regulatory subunit (RPN7), putative | |  |  |  |  |  | | --- | --- | --- | --- | --- | |  |  |  |  |  | | F49C12.8 | | | | | | |
|  |  | RAFL11-04-H03 | At1g20200 / 26S proteasome regulatory subunit S3 (RPN3), putative | |  |  |  |  |  | | --- | --- | --- | --- | --- | |  |  |  |  |  | | C30C11.2 | | | | | | |
|  | CD4.6 | | WI5\_id:CD4.6 |  | 2 | 313 | 3 | 4345 | 0.039689664 | 0.07937933 | 2 |
|  |  | RAFL06-16-H22 | At1g16470 / 20S proteasome alpha subunit B (PAB1) | |  |  |  |  |  | | --- | --- | --- | --- | --- | |  |  |  |  |  | | D1054.2 | | | | | | |
|  |  | RAFL06-12-E24 | At5g42790 / 20S proteasome alpha subunit F1 (PAF1) | |  |  |  |  |  | | --- | --- | --- | --- | --- | |  |  |  |  |  | | CD4.6 | | | | | | |
|  | C15H11.7 | | WI5\_id:C15H11.7 |  | 3 | 312 | 9 | 4339 | 0.042557452 | 0.17022981 | 4 |
|  |  | RAFL06-16-H22 | At1g16470 / 20S proteasome alpha subunit B (PAB1) | |  |  |  |  |  | | --- | --- | --- | --- | --- | |  |  |  |  |  | | D1054.2 | | | | | | |
|  |  | RAFL08-09-N05 | At4g31300 / 20S proteasome beta subunit A (PBA1) | |  |  |  |  |  | | --- | --- | --- | --- | --- | |  |  |  |  |  | | K08D12.1 | | | | | | |
|  |  | RAFL06-12-E24 | At5g42790 / 20S proteasome alpha subunit F1 (PAF1) | |  |  |  |  |  | | --- | --- | --- | --- | --- | |  |  |  |  |  | | CD4.6 | | | | | | |
| Cluster:8-0 | | |  |  | A | B | C | D | P | P' | N |
|  | C47E12.5 | | WI5\_id:C47E12.5 |  | 1 | 108 | 0 | 4554 | 0.02337551 | 0.02337551 | 1 |
|  |  | RAFL05-03-L12 | At1g47128 / cysteine proteinase RD21A | |  |  |  |  |  | | --- | --- | --- | --- | --- | |  |  |  |  |  | | T03E6.7 | | | | | | |
|  | K12H4.8 | | WI5\_id:K12H4.8 |  | 1 | 108 | 0 | 4554 | 0.02337551 | 0.02337551 | 1 |
|  |  | RAFL05-03-L12 | At1g47128 / cysteine proteinase RD21A | |  |  |  |  |  | | --- | --- | --- | --- | --- | |  |  |  |  |  | | T03E6.7 | | | | | | |
|  | T03E6.7 | | WI5\_id:T03E6.7 |  | 1 | 108 | 0 | 4554 | 0.02337551 | 0.02337551 | 1 |
|  |  | RAFL05-03-L12 | At1g47128 / cysteine proteinase RD21A | |  |  |  |  |  | | --- | --- | --- | --- | --- | |  |  |  |  |  | | T03E6.7 | | | | | | |
|  | T19A6.3 | | WI5\_id:T19A6.3 |  | 1 | 108 | 0 | 4554 | 0.02337551 | 0.02337551 | 1 |
|  |  | RAFL05-03-L12 | At1g47128 / cysteine proteinase RD21A | |  |  |  |  |  | | --- | --- | --- | --- | --- | |  |  |  |  |  | | T03E6.7 | | | | | | |
|  | R08D7.3 | | WI5\_id:R08D7.3 |  | 1 | 108 | 0 | 4554 | 0.02337551 | 0.02337551 | 1 |
|  |  | RAFL05-03-L12 | At1g47128 / cysteine proteinase RD21A | |  |  |  |  |  | | --- | --- | --- | --- | --- | |  |  |  |  |  | | T03E6.7 | | | | | | |
|  | F10G7.4 | | WI5\_id:F10G7.4 |  | 1 | 108 | 1 | 4553 | 0.0462095 | 0.092419 | 2 |
|  |  | RAFL05-03-L12 | At1g47128 / cysteine proteinase RD21A | |  |  |  |  |  | | --- | --- | --- | --- | --- | |  |  |  |  |  | | T03E6.7 | | | | | | |
|  | ZK849.2 | | WI5\_id:ZK849.2 |  | 1 | 108 | 1 | 4553 | 0.0462095 | 0.092419 | 2 |
|  |  | RAFL05-03-L12 | At1g47128 / cysteine proteinase RD21A | |  |  |  |  |  | | --- | --- | --- | --- | --- | |  |  |  |  |  | | T03E6.7  cathepsin-like protease | | | | | | |
|  | F48E3.3 | | WI5\_id:F48E3.3 |  | 1 | 108 | 1 | 4553 | 0.0462095 | 0.092419 | 2 |
|  |  | RAFL09-13-J20 | At1g69410 / Eukaryotic initiation factor 5A -related | |  |  |  |  |  | | --- | --- | --- | --- | --- | |  |  |  |  |  | | T05G5.10 | | | | | | |
|  | K07A1.1 | | WI5\_id:K07A1.1 |  | 1 | 108 | 1 | 4553 | 0.0462095 | 0.092419 | 2 |
|  |  | RAFL09-13-J20 | At1g69410 / Eukaryotic initiation factor 5A -related | |  |  |  |  |  | | --- | --- | --- | --- | --- | |  |  |  |  |  | | T05G5.10 | | | | | | |
|  | ZK1225.6 | | WI5\_id:ZK1225.6 |  | 1 | 108 | 1 | 4553 | 0.0462095 | 0.092419 | 2 |
|  |  | RAFL09-13-J20 | At1g69410 / Eukaryotic initiation factor 5A -related | |  |  |  |  |  | | --- | --- | --- | --- | --- | |  |  |  |  |  | | T05G5.10 | | | | | | |
|  | T05C12.6 | | WI5\_id:T05C12.6 |  | 1 | 108 | 1 | 4553 | 0.0462095 | 0.092419 | 2 |
|  |  | RAFL05-03-L12 | At1g47128 / cysteine proteinase RD21A | |  |  |  |  |  | | --- | --- | --- | --- | --- | |  |  |  |  |  | | T03E6.7 | | | | | | |
|  | F27C8.2 | | WI5\_id:F27C8.2 |  | 1 | 108 | 1 | 4553 | 0.0462095 | 0.092419 | 2 |
|  |  | RAFL09-13-J20 | At1g69410 / Eukaryotic initiation factor 5A -related | |  |  |  |  |  | | --- | --- | --- | --- | --- | |  |  |  |  |  | | T05G5.10 | | | | | | |
|  | Y17G7B.4 | | WI5\_id:Y17G7B.4 |  | 1 | 108 | 1 | 4553 | 0.0462095 | 0.092419 | 2 |
|  |  | RAFL09-13-J20 | At1g69410 / Eukaryotic initiation factor 5A -related | |  |  |  |  |  | | --- | --- | --- | --- | --- | |  |  |  |  |  | | T05G5.10 | | | | | | |
|  | K10H10.2 | | WI5\_id:K10H10.2 |  | 1 | 108 | 1 | 4553 | 0.0462095 | 0.092419 | 2 |
|  |  | RAFL09-13-J20 | At1g69410 / Eukaryotic initiation factor 5A -related | |  |  |  |  |  | | --- | --- | --- | --- | --- | |  |  |  |  |  | | T05G5.10  Initiation factor 5A | | | | | | |
| Cluster:6-2 | | |  |  | A | B | C | D | P | P' | N |
|  | Y47D3A.29 | | WI5\_id:Y47D3A.29 |  | 1 | 175 | 0 | 4487 | 0.03774394 | 0.03774394 | 1 |
|  |  | RAFL11-07-O15 | At3g28730 / recombination signal sequence recognition protein -related | |  |  |  |  |  | | --- | --- | --- | --- | --- | |  |  |  |  |  | | T20B12.8 | | | | | | |
|  | T20B12.8 | | WI5\_id:T20B12.8 |  | 1 | 175 | 0 | 4487 | 0.03774394 | 0.03774394 | 1 |
|  |  | RAFL11-07-O15 | At3g28730 / recombination signal sequence recognition protein -related | |  |  |  |  |  | | --- | --- | --- | --- | --- | |  |  |  |  |  | | T20B12.8 | | | | | | |
|  | F55A3.3 | | WI5\_id:F55A3.3 |  | 1 | 175 | 0 | 4487 | 0.03774394 | 0.03774394 | 1 |
|  |  | RAFL11-07-O15 | At3g28730 / recombination signal sequence recognition protein -related | |  |  |  |  |  | | --- | --- | --- | --- | --- | |  |  |  |  |  | | T20B12.8 | | | | | | |
|  | H02I12.5 | | WI5\_id:H02I12.5 |  | 1 | 175 | 0 | 4487 | 0.03774394 | 0.03774394 | 1 |
|  |  | RAFL11-07-O15 | At3g28730 / recombination signal sequence recognition protein -related | |  |  |  |  |  | | --- | --- | --- | --- | --- | |  |  |  |  |  | | T20B12.8 | | | | | | |
| Cluster:7-0 | | |  |  | A | B | C | D | P | P' | N |
|  | K01A2.10 | | WI5\_id:K01A2.10 |  | 2 | 245 | 0 | 4416 | 0.002795081 | 0.002795081 | 1 |
|  |  | RAFL05-04-D08 | At1g64520 / 26S proteasome regulatory subunit (RPN12), putative | |  |  |  |  |  | | --- | --- | --- | --- | --- | |  |  |  |  |  | | ZK20.5 | | | | | | |
|  |  | RAFL06-13-G03 | At1g64520 / 26S proteasome regulatory subunit (RPN12), putative | |  |  |  |  |  | | --- | --- | --- | --- | --- | |  |  |  |  |  | | ZK20.5 | | | | | | |
|  | Y49E10.1 | | WI5\_id:Y49E10.1 |  | 2 | 245 | 4 | 4412 | 0.036386058 | 0.18193029 | 5 |
|  |  | RAFL05-04-D08 | At1g64520 / 26S proteasome regulatory subunit (RPN12), putative | |  |  |  |  |  | | --- | --- | --- | --- | --- | |  |  |  |  |  | | ZK20.5 | | | | | | |
|  |  | RAFL06-13-G03 | At1g64520 / 26S proteasome regulatory subunit (RPN12), putative | |  |  |  |  |  | | --- | --- | --- | --- | --- | |  |  |  |  |  | | ZK20.5 | | | | | | |
|  | F29G9.5 | | WI5\_id:F29G9.5 |  | 2 | 245 | 5 | 4411 | 0.049187053 | 0.29512233 | 6 |
|  |  | RAFL05-04-D08 | At1g64520 / 26S proteasome regulatory subunit (RPN12), putative | |  |  |  |  |  | | --- | --- | --- | --- | --- | |  |  |  |  |  | | ZK20.5 | | | | | | |
|  |  | RAFL06-13-G03 | At1g64520 / 26S proteasome regulatory subunit (RPN12), putative | |  |  |  |  |  | | --- | --- | --- | --- | --- | |  |  |  |  |  | | ZK20.5 | | | | | | |
|  | T06D8.8 | | WI5\_id:T06D8.8 |  | 2 | 245 | 5 | 4411 | 0.049187053 | 0.29512233 | 6 |
|  |  | RAFL05-04-D08 | At1g64520 / 26S proteasome regulatory subunit (RPN12), putative | |  |  |  |  |  | | --- | --- | --- | --- | --- | |  |  |  |  |  | | ZK20.5 | | | | | | |
|  |  | RAFL06-13-G03 | At1g64520 / 26S proteasome regulatory subunit (RPN12), putative | |  |  |  |  |  | | --- | --- | --- | --- | --- | |  |  |  |  |  | | ZK20.5 | | | | | | |
|  | R12E2.3 | | WI5\_id:R12E2.3 |  | 2 | 245 | 5 | 4411 | 0.049187053 | 0.29512233 | 6 |
|  |  | RAFL05-04-D08 | At1g64520 / 26S proteasome regulatory subunit (RPN12), putative | |  |  |  |  |  | | --- | --- | --- | --- | --- | |  |  |  |  |  | | ZK20.5 | | | | | | |
|  |  | RAFL06-13-G03 | At1g64520 / 26S proteasome regulatory subunit (RPN12), putative | |  |  |  |  |  | | --- | --- | --- | --- | --- | |  |  |  |  |  | | ZK20.5 | | | | | | |
|  | C48D5.1 | | WI5\_id:C48D5.1 |  | 2 | 245 | 5 | 4411 | 0.049187053 | 0.29512233 | 6 |
|  |  | RAFL05-04-D08 | At1g64520 / 26S proteasome regulatory subunit (RPN12), putative | |  |  |  |  |  | | --- | --- | --- | --- | --- | |  |  |  |  |  | | ZK20.5 | | | | | | |
|  |  | RAFL06-13-G03 | At1g64520 / 26S proteasome regulatory subunit (RPN12), putative | |  |  |  |  |  | | --- | --- | --- | --- | --- | |  |  |  |  |  | | ZK20.5 | | | | | | |
