# Supplementary material for: A flexible representation of omic knowledge for thorough analysis of microarray data
Source: Plant Methods. 2006 Mar 2;2:5. doi: 10.1186/1746-4811-2-5 (PMC1421397; doi:10.1186/1746-4811-2-5)
Supplement: Additional File 6 — Supplementary Table 6. Ranking result of significant correlations between the "functional Class" of the "Comparison between gene expression of Arabidopsis thaliana and Zinia elegans" and the clusters formed by BL-SOM of the microarray probes of expression profile under drought conditions [file 1746-4811-2-5-S6.HTML]

|  |  |  |  |  |  |  |  |  |  |  |  |
| --- | --- | --- | --- | --- | --- | --- | --- | --- | --- | --- | --- |
| Date: | | 2005/06/24 | | | | | | | | | |
| Method: | | Fisher test | | | | | | | | | |
| Cut off P-value: | | 0.05 | | | | | | | | | |
| Target dataset(s): | | microarray data of zinnia ARRAY\_ZE\_00001 | | | | | | | | | |
| Query dataset(s): | | SOM Cluster | | | | | | | | | |
  | | | | | | | | | | | || C3 | | | GeneTree cluster\_id:C3 |  | A | B | C | D | P | P' | N |
|  | Cluster:2-1 | |  |  | 3 | 241 | 7 | 4412 | 0.012910854 | 0.077465124 | 6 |
|  |  | RAFL05-13-M17 | At3g53890 / 40S ribosomal protein S21 homolog | |  |  |  |  |  | | --- | --- | --- | --- | --- | |  |  |  |  |  | | At3g53890 ,Z1565  40S ribosomal protein S21 homolog [Arabidopsis thaliana] | | | | | | |
|  |  | RAFL05-17-F03 | At2g32060 / 40S ribosomal protein S12 (RPS12C) | |  |  |  |  |  | | --- | --- | --- | --- | --- | |  |  |  |  |  | | Z827 ,At2g32060  ribosomal protein S12 [Hordeum vulgare] | | | | | | |
|  |  | RAFL07-13-J18 | At2g37270 / 40S ribosomal protein S5 (RPS5A) | |  |  |  |  |  | | --- | --- | --- | --- | --- | |  |  |  |  |  | | Z5061 ,At2g37270  putative 40S ribosomal protein S5 [Oryza sativa] | | | | | | |
| B2 | | | GeneTree cluster\_id:B2 |  | A | B | C | D | P | P' | N |
|  | Cluster:1-0 | |  |  | 1 | 148 | 0 | 4514 | 0.031953678 | 0.031953678 | 1 |
|  |  | RAFL05-05-C12 | At3g53460 / 29 kDa ribonucleoprotein, chloroplast (RNA-binding protein cp29) | |  |  |  |  |  | | --- | --- | --- | --- | --- | |  |  |  |  |  | | At3g53460 ,Z5969  29kD B ribonucleoprotein [Nicotiana sylvestris] | | | | | | |
| A2 | | | GeneTree cluster\_id:A2 |  | A | B | C | D | P | P' | N |
|  | Cluster:2-1 | |  |  | 2 | 242 | 2 | 4417 | 0.01525385 | 0.045761548 | 3 |
|  |  | RAFL09-15-F15 | At4g30190 / ATPase 2, plasma membrane-type (proton pump 2) (proton-exporting ATPase), putative | |  |  |  |  |  | | --- | --- | --- | --- | --- | |  |  |  |  |  | | At4g30190 ,Z2090  plasma membrane proton ATPase [Kosteletzkya virginica] | | | | | | |
|  |  | RAFL06-15-O15 | At5g09220 / amino acid permease 2 (AAP2) | |  |  |  |  |  | | --- | --- | --- | --- | --- | |  |  |  |  |  | | Z6876 ,At5g09220  amino acid carrier [Ricinus communis] | | | | | | |
| E4 | | | GeneTree cluster\_id:E4 |  | A | B | C | D | P | P' | N |
|  | Cluster:4-0 | |  |  | 3 | 237 | 10 | 4413 | 0.026236488 | 0.2098919 | 8 |
|  |  | RAFL08-08-F03 | At5g19690 / oligosaccharyl transferase STT3-related protein | |  |  |  |  |  | | --- | --- | --- | --- | --- | |  |  |  |  |  | | Z2346 ,At5g19690  oligosaccharyl transferase STT3-like protein [Arabidopsis thaliana] | | | | | | |
|  |  | RAFL07-09-G10 | At3g18660 / glycogenin glucosyltransferase (glycogenin) - related | |  |  |  |  |  | | --- | --- | --- | --- | --- | |  |  |  |  |  | | At3g18660 ,Z6956 ,Z850  hypothetical protein [Arabidopsis thaliana]  unknown protein [Arabidopsis thaliana] | | | | | | |
|  |  | RAFL07-14-E01 | At2g38080 / laccase (diphenol oxidase), putative | |  |  |  |  |  | | --- | --- | --- | --- | --- | |  |  |  |  |  | | Z741 ,At2g38080 ,Z832  diphenol oxidase [Nicotiana tabacum]/laccase | | | | | | |
|  | Cluster:7-0 | |  |  | 3 | 244 | 10 | 4406 | 0.028285021 | 0.22628017 | 8 |
|  |  | RAFL05-17-H06 | At4g16190 / cysteine proteinase | |  |  |  |  |  | | --- | --- | --- | --- | --- | |  |  |  |  |  | | At4g16190 ,Z8955  putative preprocysteine proteinase [Nicotiana tabacum] | | | | | | |
|  |  | RAFL11-12-D09 | At4g16190 / cysteine proteinase | |  |  |  |  |  | | --- | --- | --- | --- | --- | |  |  |  |  |  | | At4g16190 ,Z8955  putative preprocysteine proteinase [Nicotiana tabacum] | | | | | | |
|  |  | RAFL08-11-G18 | At4g16190 / cysteine proteinase | |  |  |  |  |  | | --- | --- | --- | --- | --- | |  |  |  |  |  | | At4g16190 ,Z8955  putative preprocysteine proteinase [Nicotiana tabacum] | | | | | | |
| C1 | | | GeneTree cluster\_id:C1 |  | A | B | C | D | P | P' | N |
|  | Cluster:1-2 | |  |  | 7 | 167 | 9 | 4480 | 7.691689E-7 | 5.3841823E-6 | 7 |
|  |  | RAFL09-11-C22 | At5g17920 / 5-methyltetrahydropteroyltriglutamate--homocysteine S-methyltransferase | |  |  |  |  |  | | --- | --- | --- | --- | --- | |  |  |  |  |  | | At5g17920 ,Z4970  cobalamine-independent methionine synthase [Solenostemon scutellarioides] | | | | | | |
|  |  | RAFL11-01-K15 | At5g17920 / 5-methyltetrahydropteroyltriglutamate--homocysteine S-methyltransferase | |  |  |  |  |  | | --- | --- | --- | --- | --- | |  |  |  |  |  | | At5g17920 ,Z4970  cobalamine-independent methionine synthase [Solenostemon scutellarioides] | | | | | | |
|  |  | RAFL09-10-C09 | At5g17920 / 5-methyltetrahydropteroyltriglutamate--homocysteine S-methyltransferase | |  |  |  |  |  | | --- | --- | --- | --- | --- | |  |  |  |  |  | | At5g17920 ,Z4970  cobalamine-independent methionine synthase [Solenostemon scutellarioides] | | | | | | |
|  |  | RAFL06-12-D05 | At5g17920 / 5-methyltetrahydropteroyltriglutamate--homocysteine S-methyltransferase | |  |  |  |  |  | | --- | --- | --- | --- | --- | |  |  |  |  |  | | At5g17920 ,Z4970  cobalamine-independent methionine synthase [Solenostemon scutellarioides] | | | | | | |
|  |  | RAFL11-06-L17 | At5g17920 / 5-methyltetrahydropteroyltriglutamate--homocysteine S-methyltransferase | |  |  |  |  |  | | --- | --- | --- | --- | --- | |  |  |  |  |  | | At5g17920 ,Z4970  cobalamine-independent methionine synthase [Solenostemon scutellarioides] | | | | | | |
|  |  | RAFL05-21-E19 | At1g48630 / WD-40 repeat auxin-dependent protein ARCA, putative | |  |  |  |  |  | | --- | --- | --- | --- | --- | |  |  |  |  |  | | At1g48630 ,Z1752  G protein beta subunit-like [Medicago sativa] | | | | | | |
|  |  | RAFL09-11-K06 | At4g13930 / hydroxymethyltransferase | |  |  |  |  |  | | --- | --- | --- | --- | --- | |  |  |  |  |  | | At4g13930 ,Z5266  hydroxymethyltransferase [Arabidopsis thaliana] | | | | | | |
|  | Cluster:0-2 | |  |  | 2 | 77 | 14 | 4570 | 0.029166238 | 0.20416367 | 7 |
|  |  | RAFL08-16-E05 | At5g17920 / 5-methyltetrahydropteroyltriglutamate--homocysteine S-methyltransferase | |  |  |  |  |  | | --- | --- | --- | --- | --- | |  |  |  |  |  | | At5g17920 ,Z4970  cobalamine-independent methionine synthase [Solenostemon scutellarioides] | | | | | | |
|  |  | RAFL09-09-A21 | At5g17920 / 5-methyltetrahydropteroyltriglutamate--homocysteine S-methyltransferase | |  |  |  |  |  | | --- | --- | --- | --- | --- | |  |  |  |  |  | | At5g17920 ,Z4970  cobalamine-independent methionine synthase [Solenostemon scutellarioides] | | | | | | |
| C5 | | | GeneTree cluster\_id:C5 |  | A | B | C | D | P | P' | N |
|  | Cluster:2-1 | |  |  | 3 | 241 | 2 | 4417 | 0.0013085117 | 0.0039255354 | 3 |
|  |  | RAFL07-15-M07 | At1g04480 / 60S ribosomal protein L23 (RPL23A) | |  |  |  |  |  | | --- | --- | --- | --- | --- | |  |  |  |  |  | | Z611 ,At1g04480  putative putative 60S ribosomal protein L17 [Arabidopsis thaliana] | | | | | | |
|  |  | RAFL05-18-P15 | At1g04480 / 60S ribosomal protein L23 (RPL23A) | |  |  |  |  |  | | --- | --- | --- | --- | --- | |  |  |  |  |  | | Z611 ,At1g04480  putative putative 60S ribosomal protein L17 [Arabidopsis thaliana] | | | | | | |
|  |  | RAFL06-15-B04 | At1g14810 / aspartate-semialdehyde dehydrogenase -related | |  |  |  |  |  | | --- | --- | --- | --- | --- | |  |  |  |  |  | | At1g14810 ,Z1806  aspartate-semialdehyde dehydrogenase, putative [Arabidopsis thaliana] | | | | | | |
| C2 | | | GeneTree cluster\_id:C2 |  | A | B | C | D | P | P' | N |
|  | Cluster:1-0 | |  |  | 2 | 147 | 4 | 4510 | 0.013980698 | 0.05592279 | 4 |
|  |  | RAFL05-14-L16 | At3g19820 / cell elongation protein (DWARF1) (DIMINUTO) (DIM) | |  |  |  |  |  | | --- | --- | --- | --- | --- | |  |  |  |  |  | | At3g19820 ,Z3771  brassinosteroid biosynthetic protein LKB [Pisum sativum]/DIMINUTO/Dwarf1 | | | | | | |
|  |  | RAFL04-18-H22 | At1g01300 / chloroplast nucleoid DNA binding protein -related | |  |  |  |  |  | | --- | --- | --- | --- | --- | |  |  |  |  |  | | At1g01300 ,Z6332  chloroplast nucleoid DNA binding protein, putative [Arabidopsis thaliana] | | | | | | |
|  | Cluster:4-0 | |  |  | 2 | 238 | 4 | 4419 | 0.034489654 | 0.13795862 | 4 |
|  |  | RAFL09-14-G07 | At4g37870 / phosphoenolpyruvate carboxykinase (ATP) -related protein | |  |  |  |  |  | | --- | --- | --- | --- | --- | |  |  |  |  |  | | Z446 ,At4g37870  phosphoenolpyruvate carboxykinase [Flaveria trinervia] | | | | | | |
|  |  | RAFL05-14-F18 | At4g37870 / phosphoenolpyruvate carboxykinase (ATP) -related protein | |  |  |  |  |  | | --- | --- | --- | --- | --- | |  |  |  |  |  | | Z446 ,At4g37870  phosphoenolpyruvate carboxykinase [Flaveria trinervia] | | | | | | |
| D5 | | | GeneTree cluster\_id:D5 |  | A | B | C | D | P | P' | N |
|  | Cluster:1-0 | |  |  | 1 | 148 | 0 | 4514 | 0.031953678 | 0.031953678 | 1 |
|  |  | RAFL09-10-F09 | At1g70370 / aromatic rich glyco protein -related | |  |  |  |  |  | | --- | --- | --- | --- | --- | |  |  |  |  |  | | Z3858 ,At1g70370  polygalacturonase isoenzyme 1 beta subunit [Arabidopsis thaliana] | | | | | | |
| A1 | | | GeneTree cluster\_id:A1 |  | A | B | C | D | P | P' | N |
|  | Cluster:1-2 | |  |  | 13 | 161 | 77 | 4412 | 2.3164921E-5 | 6.0228794E-4 | 26 |
|  |  | RAFL08-12-B15 | At5g46110 / phosphate/triose-phosphate translocator, putative | |  |  |  |  |  | | --- | --- | --- | --- | --- | |  |  |  |  |  | | Z6046 ,At5g46110  triose phosphate/phosphate translocator [Flaveria pringlei] | | | | | | |
|  |  | RAFL11-12-B10 | At5g66570 / photosystem II oxygen-evolving complex 33 (OEC33) | |  |  |  |  |  | | --- | --- | --- | --- | --- | |  |  |  |  |  | | At5g66570 ,Z7352  33kDa precursor protein of oxygen-evolving complex [Solanum tuberosum] | | | | | | |
|  |  | RAFL08-10-G06 | At5g01530 / light-harvesting chlorophyll a/b binding protein | |  |  |  |  |  | | --- | --- | --- | --- | --- | |  |  |  |  |  | | Z3440 ,At5g01530  Chlorophyll a/b-binding protein CP29 precursor [Zea mays] | | | | | | |
|  |  | RAFL03-03-A07 | At5g66570 / photosystem II oxygen-evolving complex 33 (OEC33) | |  |  |  |  |  | | --- | --- | --- | --- | --- | |  |  |  |  |  | | At5g66570 ,Z7352  33kDa precursor protein of oxygen-evolving complex [Solanum tuberosum] | | | | | | |
|  |  | RAFL06-15-G17 | At5g01530 / light-harvesting chlorophyll a/b binding protein | |  |  |  |  |  | | --- | --- | --- | --- | --- | |  |  |  |  |  | | Z3440 ,At5g01530  Chlorophyll a/b-binding protein CP29 precursor [Zea mays] | | | | | | |
|  |  | RAFL07-18-K16 | At5g03760 / glycosyltransferase family 2 | |  |  |  |  |  | | --- | --- | --- | --- | --- | |  |  |  |  |  | | At5g03760 ,Z2945  putative protein [Arabidopsis thaliana] | | | | | | |
|  |  | RAFL09-06-C15 | At5g01530 / light-harvesting chlorophyll a/b binding protein | |  |  |  |  |  | | --- | --- | --- | --- | --- | |  |  |  |  |  | | Z3440 ,At5g01530  Chlorophyll a/b-binding protein CP29 precursor [Zea mays] | | | | | | |
|  |  | RAFL11-03-I17 | At5g01530 / light-harvesting chlorophyll a/b binding protein | |  |  |  |  |  | | --- | --- | --- | --- | --- | |  |  |  |  |  | | Z3440 ,At5g01530  Chlorophyll a/b-binding protein CP29 precursor [Zea mays] | | | | | | |
|  |  | RAFL09-11-O13 | At3g46780 / expressed protein | |  |  |  |  |  | | --- | --- | --- | --- | --- | |  |  |  |  |  | | Z5531 ,At3g46780  putative protein [Arabidopsis thaliana] | | | | | | |
|  |  | RAFL07-16-C20 | At5g01530 / light-harvesting chlorophyll a/b binding protein | |  |  |  |  |  | | --- | --- | --- | --- | --- | |  |  |  |  |  | | Z3440 ,At5g01530  Chlorophyll a/b-binding protein CP29 precursor [Zea mays] | | | | | | |
|  |  | RAFL09-15-H22 | At1g05190 / ribosomal protein L6p family | |  |  |  |  |  | | --- | --- | --- | --- | --- | |  |  |  |  |  | | At1g05190 ,Z3804  putative chloroplast 50S ribosomal protein, L6 [Arabidopsis thaliana] | | | | | | |
|  |  | RAFL03-06-H04 | At5g46110 / phosphate/triose-phosphate translocator, putative | |  |  |  |  |  | | --- | --- | --- | --- | --- | |  |  |  |  |  | | Z6046 ,At5g46110  triose phosphate/phosphate translocator [Flaveria pringlei] | | | | | | |
|  |  | RAFL05-08-N13 | At4g02530 / chloroplast thylakoid lumen protein | |  |  |  |  |  | | --- | --- | --- | --- | --- | |  |  |  |  |  | | At4g02530 ,Z6019  predicted protein of unknown function [Arabidopsis thaliana] | | | | | | |
|  | Cluster:0-1 | |  |  | 10 | 96 | 80 | 4477 | 3.0162279E-5 | 7.8421924E-4 | 26 |
|  |  | RAFL06-13-A08 | At1g44575 / photosystem II 22kDa protein -related | |  |  |  |  |  | | --- | --- | --- | --- | --- | |  |  |  |  |  | | Z7449 ,At1g44575  precursor of photosystem II subunit (22KDa) [Nicotiana tabacum] | | | | | | |
|  |  | RAFL07-12-E12 | At2g21330 / fructose-bisphosphate aldolase, putative | |  |  |  |  |  | | --- | --- | --- | --- | --- | |  |  |  |  |  | | Z3569 ,At2g21330  homologous to plastidic aldolases [Solanum tuberosum] | | | | | | |
|  |  | RAFL05-18-I22 | At1g44575 / photosystem II 22kDa protein -related | |  |  |  |  |  | | --- | --- | --- | --- | --- | |  |  |  |  |  | | Z7449 ,At1g44575  precursor of photosystem II subunit (22KDa) [Nicotiana tabacum] | | | | | | |
|  |  | RAFL09-17-N23 | At2g21330 / fructose-bisphosphate aldolase, putative | |  |  |  |  |  | | --- | --- | --- | --- | --- | |  |  |  |  |  | | Z3569 ,At2g21330  homologous to plastidic aldolases [Solanum tuberosum] | | | | | | |
|  |  | RAFL07-18-J01 | At2g21330 / fructose-bisphosphate aldolase, putative | |  |  |  |  |  | | --- | --- | --- | --- | --- | |  |  |  |  |  | | Z3569 ,At2g21330  homologous to plastidic aldolases [Solanum tuberosum] | | | | | | |
|  |  | RAFL06-08-I11 | At2g46820 / expressed protein | |  |  |  |  |  | | --- | --- | --- | --- | --- | |  |  |  |  |  | | Z302 ,At2g46820  unknown protein [Arabidopsis thaliana] | | | | | | |
|  |  | RAFL07-12-M09 | At2g21330 / fructose-bisphosphate aldolase, putative | |  |  |  |  |  | | --- | --- | --- | --- | --- | |  |  |  |  |  | | Z3569 ,At2g21330  homologous to plastidic aldolases [Solanum tuberosum] | | | | | | |
|  |  | RAFL07-18-C20 | At2g21330 / fructose-bisphosphate aldolase, putative | |  |  |  |  |  | | --- | --- | --- | --- | --- | |  |  |  |  |  | | Z3569 ,At2g21330  homologous to plastidic aldolases [Solanum tuberosum] | | | | | | |
|  |  | RAFL08-09-L12 | At3g26060 / peroxiredoxin -related | |  |  |  |  |  | | --- | --- | --- | --- | --- | |  |  |  |  |  | | At3g26060 ,Z7102  peroxiredoxin Q [Sedum lineare] | | | | | | |
|  |  | RAFL08-18-C10 | At2g21330 / fructose-bisphosphate aldolase, putative | |  |  |  |  |  | | --- | --- | --- | --- | --- | |  |  |  |  |  | | Z3569 ,At2g21330  homologous to plastidic aldolases [Solanum tuberosum] | | | | | | |
|  | Cluster:0-2 | |  |  | 6 | 73 | 84 | 4500 | 0.0039265547 | 0.102090426 | 26 |
|  |  | RAFL07-14-F21 | At1g54780 / thylakoid lumen 18.3 kDa protein | |  |  |  |  |  | | --- | --- | --- | --- | --- | |  |  |  |  |  | | Z3344 ,At1g54780  unknown protein [Arabidopsis thaliana] | | | | | | |
|  |  | RAFL05-19-G04 | At1g54780 / thylakoid lumen 18.3 kDa protein | |  |  |  |  |  | | --- | --- | --- | --- | --- | |  |  |  |  |  | | Z3344 ,At1g54780  unknown protein [Arabidopsis thaliana] | | | | | | |
|  |  | RAFL04-16-N08 | At5g47110 / Lil3 protein | |  |  |  |  |  | | --- | --- | --- | --- | --- | |  |  |  |  |  | | Z2874 ,At5g47110  Lil3 protein [Arabidopsis thaliana] | | | | | | |
|  |  | RAFL05-01-I05 | At1g54780 / thylakoid lumen 18.3 kDa protein | |  |  |  |  |  | | --- | --- | --- | --- | --- | |  |  |  |  |  | | Z3344 ,At1g54780  unknown protein [Arabidopsis thaliana] | | | | | | |
|  |  | RAFL05-17-B17 | At3g50820 / photosystem II oxygen-evolving complex 33 (OEC33) | |  |  |  |  |  | | --- | --- | --- | --- | --- | |  |  |  |  |  | | Z9108 ,At3g50820  oxygen-evolving enhancer protein 1, chloroplast precursor [Solanum tuberosum] | | | | | | |
|  |  | RAFL09-06-E16 | At2g26080 / glycine dehydrogenase (decarboxylating) (glycine decarboxylase/glycine cleavage system P-protein), putative | |  |  |  |  |  | | --- | --- | --- | --- | --- | |  |  |  |  |  | | Z6158 ,At2g26080  P-protein of the glycine cleavage system [Flaveria pringlei] | | | | | | |
|  | Cluster:2-2 | |  |  | 4 | 49 | 86 | 4524 | 0.018504295 | 0.48111168 | 26 |
|  |  | RAFL05-14-K24 | At1g18060 / expressed protein | |  |  |  |  |  | | --- | --- | --- | --- | --- | |  |  |  |  |  | | Z3053 ,At1g18060  unknown protein [Arabidopsis thaliana] | | | | | | |
|  |  | RAFL06-12-L22 | At4g34190 / stress enhanced protein 1 (SEP1) | |  |  |  |  |  | | --- | --- | --- | --- | --- | |  |  |  |  |  | | Z7039 ,At4g34190  stress enhanced protein 1; SEP1 [Arabidopsis thaliana] | | | | | | |
|  |  | RAFL06-10-H13 | At5g42650 / allene oxide synthase / cytochrome P450 74A | |  |  |  |  |  | | --- | --- | --- | --- | --- | |  |  |  |  |  | | At5g42650 ,Z7649  rubber particle protein [Parthenium argentatum] | | | | | | |
|  |  | RAFL05-12-G03 | At5g42650 / allene oxide synthase / cytochrome P450 74A | |  |  |  |  |  | | --- | --- | --- | --- | --- | |  |  |  |  |  | | At5g42650 ,Z7649  rubber particle protein [Parthenium argentatum] | | | | | | |
|  | Cluster:1-0 | |  |  | 7 | 142 | 83 | 4431 | 0.024142498 | 0.627705 | 26 |
|  |  | RAFL06-16-H24 | At5g11450 / oxygen-evolving complex related protein | |  |  |  |  |  | | --- | --- | --- | --- | --- | |  |  |  |  |  | | At5g11450 ,Z3468  putative protein [Arabidopsis thaliana] | | | | | | |
|  |  | RAFL04-15-H06 | At3g25920 / 50S ribosomal protein L15, chloroplast precursor (CL15) | |  |  |  |  |  | | --- | --- | --- | --- | --- | |  |  |  |  |  | | Z4749 ,At3g25920  Plastid ribosomal protein CL15 [Pisum sativum] | | | | | | |
|  |  | RAFL05-02-L04 | At4g02530 / chloroplast thylakoid lumen protein | |  |  |  |  |  | | --- | --- | --- | --- | --- | |  |  |  |  |  | | At4g02530 ,Z6019  predicted protein of unknown function [Arabidopsis thaliana] | | | | | | |
|  |  | RAFL05-08-K07 | At4g01310 / ribosomal protein L5p family | |  |  |  |  |  | | --- | --- | --- | --- | --- | |  |  |  |  |  | | At4g01310 ,Z6123  plastid ribosomal protein PRPL5 [Spinacia oleracea] | | | | | | |
|  |  | RAFL05-04-C24 | At3g13120 / chloroplast 30S ribosomal protein S10, putative | |  |  |  |  |  | | --- | --- | --- | --- | --- | |  |  |  |  |  | | At3g13120 ,Z2819  plastid ribosomal protein S10 precursor [Mesembryanthemum crystallinum] | | | | | | |
|  |  | RAFL05-17-D18 | At5g55220 / trigger factor-related protein | |  |  |  |  |  | | --- | --- | --- | --- | --- | |  |  |  |  |  | | At5g55220 ,Z4062  trigger factor-like protein [Arabidopsis thaliana] | | | | | | |
|  |  | RAFL05-02-P08 | At3g01480 / thylakoid lumen rotamase | |  |  |  |  |  | | --- | --- | --- | --- | --- | |  |  |  |  |  | | Z1615 ,At3g01480  thylakoid lumen rotamase [Spinacia oleracea] | | | | | | |
|  | Cluster:9-2 | |  |  | 4 | 63 | 86 | 4510 | 0.039566863 | 1.0287384 | 26 |
|  |  | RAFL05-02-L02 | At1g19180 / expressed protein | |  |  |  |  |  | | --- | --- | --- | --- | --- | |  |  |  |  |  | | Z684 ,Z931 ,Z3688 ,At1g19180  unknown protein [Arabidopsis thaliana]  unknown protein [Arabidopsis thaliana] | | | | | | |
|  |  | RAFL09-09-P15 | At1g19180 / expressed protein | |  |  |  |  |  | | --- | --- | --- | --- | --- | |  |  |  |  |  | | Z684 ,Z931 ,Z3688 ,At1g19180  unknown protein [Arabidopsis thaliana]  unknown protein [Arabidopsis thaliana] | | | | | | |
|  |  | RAFL06-10-F03 | At1g19180 / expressed protein | |  |  |  |  |  | | --- | --- | --- | --- | --- | |  |  |  |  |  | | Z684 ,Z931 ,Z3688 ,At1g19180  unknown protein [Arabidopsis thaliana]  unknown protein [Arabidopsis thaliana] | | | | | | |
|  |  | RAFL05-03-O21 | At5g05600 / oxidoreductase, 2OG-Fe(II) oxygenase family | |  |  |  |  |  | | --- | --- | --- | --- | --- | |  |  |  |  |  | | Z7465 ,At5g05600  leucoanthocyanidin dioxygenase-like protein [Arabidopsis thaliana] | | | | | | |
|  | | | | | | | | | | | |
| Cluster:7-0 | | |  |  | A | B | C | D | P | P' | N |
|  | E4 | | GeneTree cluster\_id:E4 |  | 3 | 244 | 10 | 4406 | 0.028285021 | 0.22628017 | 8 |
|  |  | RAFL05-17-H06 | At4g16190 / cysteine proteinase | |  |  |  |  |  | | --- | --- | --- | --- | --- | |  |  |  |  |  | | At4g16190 ,Z8955  putative preprocysteine proteinase [Nicotiana tabacum] | | | | | | |
|  |  | RAFL11-12-D09 | At4g16190 / cysteine proteinase | |  |  |  |  |  | | --- | --- | --- | --- | --- | |  |  |  |  |  | | At4g16190 ,Z8955  putative preprocysteine proteinase [Nicotiana tabacum] | | | | | | |
|  |  | RAFL08-11-G18 | At4g16190 / cysteine proteinase | |  |  |  |  |  | | --- | --- | --- | --- | --- | |  |  |  |  |  | | At4g16190 ,Z8955  putative preprocysteine proteinase [Nicotiana tabacum] | | | | | | |
| Cluster:1-0 | | |  |  | A | B | C | D | P | P' | N |
|  | C2 | | GeneTree cluster\_id:C2 |  | 2 | 147 | 4 | 4510 | 0.013980698 | 0.05592279 | 4 |
|  |  | RAFL05-14-L16 | At3g19820 / cell elongation protein (DWARF1) (DIMINUTO) (DIM) | |  |  |  |  |  | | --- | --- | --- | --- | --- | |  |  |  |  |  | | At3g19820 ,Z3771  brassinosteroid biosynthetic protein LKB [Pisum sativum]/DIMINUTO/Dwarf1 | | | | | | |
|  |  | RAFL04-18-H22 | At1g01300 / chloroplast nucleoid DNA binding protein -related | |  |  |  |  |  | | --- | --- | --- | --- | --- | |  |  |  |  |  | | At1g01300 ,Z6332  chloroplast nucleoid DNA binding protein, putative [Arabidopsis thaliana] | | | | | | |
|  | A1 | | GeneTree cluster\_id:A1 |  | 7 | 142 | 83 | 4431 | 0.024142498 | 0.627705 | 26 |
|  |  | RAFL06-16-H24 | At5g11450 / oxygen-evolving complex related protein | |  |  |  |  |  | | --- | --- | --- | --- | --- | |  |  |  |  |  | | At5g11450 ,Z3468  putative protein [Arabidopsis thaliana] | | | | | | |
|  |  | RAFL04-15-H06 | At3g25920 / 50S ribosomal protein L15, chloroplast precursor (CL15) | |  |  |  |  |  | | --- | --- | --- | --- | --- | |  |  |  |  |  | | Z4749 ,At3g25920  Plastid ribosomal protein CL15 [Pisum sativum] | | | | | | |
|  |  | RAFL05-02-L04 | At4g02530 / chloroplast thylakoid lumen protein | |  |  |  |  |  | | --- | --- | --- | --- | --- | |  |  |  |  |  | | At4g02530 ,Z6019  predicted protein of unknown function [Arabidopsis thaliana] | | | | | | |
|  |  | RAFL05-08-K07 | At4g01310 / ribosomal protein L5p family | |  |  |  |  |  | | --- | --- | --- | --- | --- | |  |  |  |  |  | | At4g01310 ,Z6123  plastid ribosomal protein PRPL5 [Spinacia oleracea] | | | | | | |
|  |  | RAFL05-04-C24 | At3g13120 / chloroplast 30S ribosomal protein S10, putative | |  |  |  |  |  | | --- | --- | --- | --- | --- | |  |  |  |  |  | | At3g13120 ,Z2819  plastid ribosomal protein S10 precursor [Mesembryanthemum crystallinum] | | | | | | |
|  |  | RAFL05-17-D18 | At5g55220 / trigger factor-related protein | |  |  |  |  |  | | --- | --- | --- | --- | --- | |  |  |  |  |  | | At5g55220 ,Z4062  trigger factor-like protein [Arabidopsis thaliana] | | | | | | |
|  |  | RAFL05-02-P08 | At3g01480 / thylakoid lumen rotamase | |  |  |  |  |  | | --- | --- | --- | --- | --- | |  |  |  |  |  | | Z1615 ,At3g01480  thylakoid lumen rotamase [Spinacia oleracea] | | | | | | |
|  | B2 | | GeneTree cluster\_id:B2 |  | 1 | 148 | 0 | 4514 | 0.031953678 | 0.031953678 | 1 |
|  |  | RAFL05-05-C12 | At3g53460 / 29 kDa ribonucleoprotein, chloroplast (RNA-binding protein cp29) | |  |  |  |  |  | | --- | --- | --- | --- | --- | |  |  |  |  |  | | At3g53460 ,Z5969  29kD B ribonucleoprotein [Nicotiana sylvestris] | | | | | | |
|  | D5 | | GeneTree cluster\_id:D5 |  | 1 | 148 | 0 | 4514 | 0.031953678 | 0.031953678 | 1 |
|  |  | RAFL09-10-F09 | At1g70370 / aromatic rich glyco protein -related | |  |  |  |  |  | | --- | --- | --- | --- | --- | |  |  |  |  |  | | Z3858 ,At1g70370  polygalacturonase isoenzyme 1 beta subunit [Arabidopsis thaliana] | | | | | | |
| Cluster:4-0 | | |  |  | A | B | C | D | P | P' | N |
|  | E4 | | GeneTree cluster\_id:E4 |  | 3 | 237 | 10 | 4413 | 0.026236488 | 0.2098919 | 8 |
|  |  | RAFL08-08-F03 | At5g19690 / oligosaccharyl transferase STT3-related protein | |  |  |  |  |  | | --- | --- | --- | --- | --- | |  |  |  |  |  | | Z2346 ,At5g19690  oligosaccharyl transferase STT3-like protein [Arabidopsis thaliana] | | | | | | |
|  |  | RAFL07-09-G10 | At3g18660 / glycogenin glucosyltransferase (glycogenin) - related | |  |  |  |  |  | | --- | --- | --- | --- | --- | |  |  |  |  |  | | At3g18660 ,Z6956 ,Z850  hypothetical protein [Arabidopsis thaliana]  unknown protein [Arabidopsis thaliana] | | | | | | |
|  |  | RAFL07-14-E01 | At2g38080 / laccase (diphenol oxidase), putative | |  |  |  |  |  | | --- | --- | --- | --- | --- | |  |  |  |  |  | | Z741 ,At2g38080 ,Z832  diphenol oxidase [Nicotiana tabacum]/laccase | | | | | | |
|  | C2 | | GeneTree cluster\_id:C2 |  | 2 | 238 | 4 | 4419 | 0.034489654 | 0.13795862 | 4 |
|  |  | RAFL09-14-G07 | At4g37870 / phosphoenolpyruvate carboxykinase (ATP) -related protein | |  |  |  |  |  | | --- | --- | --- | --- | --- | |  |  |  |  |  | | Z446 ,At4g37870  phosphoenolpyruvate carboxykinase [Flaveria trinervia] | | | | | | |
|  |  | RAFL05-14-F18 | At4g37870 / phosphoenolpyruvate carboxykinase (ATP) -related protein | |  |  |  |  |  | | --- | --- | --- | --- | --- | |  |  |  |  |  | | Z446 ,At4g37870  phosphoenolpyruvate carboxykinase [Flaveria trinervia] | | | | | | |
| Cluster:2-1 | | |  |  | A | B | C | D | P | P' | N |
|  | C5 | | GeneTree cluster\_id:C5 |  | 3 | 241 | 2 | 4417 | 0.0013085117 | 0.0039255354 | 3 |
|  |  | RAFL07-15-M07 | At1g04480 / 60S ribosomal protein L23 (RPL23A) | |  |  |  |  |  | | --- | --- | --- | --- | --- | |  |  |  |  |  | | Z611 ,At1g04480  putative putative 60S ribosomal protein L17 [Arabidopsis thaliana] | | | | | | |
|  |  | RAFL05-18-P15 | At1g04480 / 60S ribosomal protein L23 (RPL23A) | |  |  |  |  |  | | --- | --- | --- | --- | --- | |  |  |  |  |  | | Z611 ,At1g04480  putative putative 60S ribosomal protein L17 [Arabidopsis thaliana] | | | | | | |
|  |  | RAFL06-15-B04 | At1g14810 / aspartate-semialdehyde dehydrogenase -related | |  |  |  |  |  | | --- | --- | --- | --- | --- | |  |  |  |  |  | | At1g14810 ,Z1806  aspartate-semialdehyde dehydrogenase, putative [Arabidopsis thaliana] | | | | | | |
|  | C3 | | GeneTree cluster\_id:C3 |  | 3 | 241 | 7 | 4412 | 0.012910854 | 0.077465124 | 6 |
|  |  | RAFL05-13-M17 | At3g53890 / 40S ribosomal protein S21 homolog | |  |  |  |  |  | | --- | --- | --- | --- | --- | |  |  |  |  |  | | At3g53890 ,Z1565  40S ribosomal protein S21 homolog [Arabidopsis thaliana] | | | | | | |
|  |  | RAFL05-17-F03 | At2g32060 / 40S ribosomal protein S12 (RPS12C) | |  |  |  |  |  | | --- | --- | --- | --- | --- | |  |  |  |  |  | | Z827 ,At2g32060  ribosomal protein S12 [Hordeum vulgare] | | | | | | |
|  |  | RAFL07-13-J18 | At2g37270 / 40S ribosomal protein S5 (RPS5A) | |  |  |  |  |  | | --- | --- | --- | --- | --- | |  |  |  |  |  | | Z5061 ,At2g37270  putative 40S ribosomal protein S5 [Oryza sativa] | | | | | | |
|  | A2 | | GeneTree cluster\_id:A2 |  | 2 | 242 | 2 | 4417 | 0.01525385 | 0.045761548 | 3 |
|  |  | RAFL09-15-F15 | At4g30190 / ATPase 2, plasma membrane-type (proton pump 2) (proton-exporting ATPase), putative | |  |  |  |  |  | | --- | --- | --- | --- | --- | |  |  |  |  |  | | At4g30190 ,Z2090  plasma membrane proton ATPase [Kosteletzkya virginica] | | | | | | |
|  |  | RAFL06-15-O15 | At5g09220 / amino acid permease 2 (AAP2) | |  |  |  |  |  | | --- | --- | --- | --- | --- | |  |  |  |  |  | | Z6876 ,At5g09220  amino acid carrier [Ricinus communis] | | | | | | |
| Cluster:2-2 | | |  |  | A | B | C | D | P | P' | N |
|  | A1 | | GeneTree cluster\_id:A1 |  | 4 | 49 | 86 | 4524 | 0.018504295 | 0.48111168 | 26 |
|  |  | RAFL05-14-K24 | At1g18060 / expressed protein | |  |  |  |  |  | | --- | --- | --- | --- | --- | |  |  |  |  |  | | Z3053 ,At1g18060  unknown protein [Arabidopsis thaliana] | | | | | | |
|  |  | RAFL06-12-L22 | At4g34190 / stress enhanced protein 1 (SEP1) | |  |  |  |  |  | | --- | --- | --- | --- | --- | |  |  |  |  |  | | Z7039 ,At4g34190  stress enhanced protein 1; SEP1 [Arabidopsis thaliana] | | | | | | |
|  |  | RAFL06-10-H13 | At5g42650 / allene oxide synthase / cytochrome P450 74A | |  |  |  |  |  | | --- | --- | --- | --- | --- | |  |  |  |  |  | | At5g42650 ,Z7649  rubber particle protein [Parthenium argentatum] | | | | | | |
|  |  | RAFL05-12-G03 | At5g42650 / allene oxide synthase / cytochrome P450 74A | |  |  |  |  |  | | --- | --- | --- | --- | --- | |  |  |  |  |  | | At5g42650 ,Z7649  rubber particle protein [Parthenium argentatum] | | | | | | |
| Cluster:1-2 | | |  |  | A | B | C | D | P | P' | N |
|  | C1 | | GeneTree cluster\_id:C1 |  | 7 | 167 | 9 | 4480 | 7.691689E-7 | 5.3841823E-6 | 7 |
|  |  | RAFL09-11-C22 | At5g17920 / 5-methyltetrahydropteroyltriglutamate--homocysteine S-methyltransferase | |  |  |  |  |  | | --- | --- | --- | --- | --- | |  |  |  |  |  | | At5g17920 ,Z4970  cobalamine-independent methionine synthase [Solenostemon scutellarioides] | | | | | | |
|  |  | RAFL11-01-K15 | At5g17920 / 5-methyltetrahydropteroyltriglutamate--homocysteine S-methyltransferase | |  |  |  |  |  | | --- | --- | --- | --- | --- | |  |  |  |  |  | | At5g17920 ,Z4970  cobalamine-independent methionine synthase [Solenostemon scutellarioides] | | | | | | |
|  |  | RAFL09-10-C09 | At5g17920 / 5-methyltetrahydropteroyltriglutamate--homocysteine S-methyltransferase | |  |  |  |  |  | | --- | --- | --- | --- | --- | |  |  |  |  |  | | At5g17920 ,Z4970  cobalamine-independent methionine synthase [Solenostemon scutellarioides] | | | | | | |
|  |  | RAFL06-12-D05 | At5g17920 / 5-methyltetrahydropteroyltriglutamate--homocysteine S-methyltransferase | |  |  |  |  |  | | --- | --- | --- | --- | --- | |  |  |  |  |  | | At5g17920 ,Z4970  cobalamine-independent methionine synthase [Solenostemon scutellarioides] | | | | | | |
|  |  | RAFL11-06-L17 | At5g17920 / 5-methyltetrahydropteroyltriglutamate--homocysteine S-methyltransferase | |  |  |  |  |  | | --- | --- | --- | --- | --- | |  |  |  |  |  | | At5g17920 ,Z4970  cobalamine-independent methionine synthase [Solenostemon scutellarioides] | | | | | | |
|  |  | RAFL05-21-E19 | At1g48630 / WD-40 repeat auxin-dependent protein ARCA, putative | |  |  |  |  |  | | --- | --- | --- | --- | --- | |  |  |  |  |  | | At1g48630 ,Z1752  G protein beta subunit-like [Medicago sativa] | | | | | | |
|  |  | RAFL09-11-K06 | At4g13930 / hydroxymethyltransferase | |  |  |  |  |  | | --- | --- | --- | --- | --- | |  |  |  |  |  | | At4g13930 ,Z5266  hydroxymethyltransferase [Arabidopsis thaliana] | | | | | | |
|  | A1 | | GeneTree cluster\_id:A1 |  | 13 | 161 | 77 | 4412 | 2.3164921E-5 | 6.0228794E-4 | 26 |
|  |  | RAFL08-12-B15 | At5g46110 / phosphate/triose-phosphate translocator, putative | |  |  |  |  |  | | --- | --- | --- | --- | --- | |  |  |  |  |  | | Z6046 ,At5g46110  triose phosphate/phosphate translocator [Flaveria pringlei] | | | | | | |
|  |  | RAFL11-12-B10 | At5g66570 / photosystem II oxygen-evolving complex 33 (OEC33) | |  |  |  |  |  | | --- | --- | --- | --- | --- | |  |  |  |  |  | | At5g66570 ,Z7352  33kDa precursor protein of oxygen-evolving complex [Solanum tuberosum] | | | | | | |
|  |  | RAFL08-10-G06 | At5g01530 / light-harvesting chlorophyll a/b binding protein | |  |  |  |  |  | | --- | --- | --- | --- | --- | |  |  |  |  |  | | Z3440 ,At5g01530  Chlorophyll a/b-binding protein CP29 precursor [Zea mays] | | | | | | |
|  |  | RAFL03-03-A07 | At5g66570 / photosystem II oxygen-evolving complex 33 (OEC33) | |  |  |  |  |  | | --- | --- | --- | --- | --- | |  |  |  |  |  | | At5g66570 ,Z7352  33kDa precursor protein of oxygen-evolving complex [Solanum tuberosum] | | | | | | |
|  |  | RAFL06-15-G17 | At5g01530 / light-harvesting chlorophyll a/b binding protein | |  |  |  |  |  | | --- | --- | --- | --- | --- | |  |  |  |  |  | | Z3440 ,At5g01530  Chlorophyll a/b-binding protein CP29 precursor [Zea mays] | | | | | | |
|  |  | RAFL07-18-K16 | At5g03760 / glycosyltransferase family 2 | |  |  |  |  |  | | --- | --- | --- | --- | --- | |  |  |  |  |  | | At5g03760 ,Z2945  putative protein [Arabidopsis thaliana] | | | | | | |
|  |  | RAFL09-06-C15 | At5g01530 / light-harvesting chlorophyll a/b binding protein | |  |  |  |  |  | | --- | --- | --- | --- | --- | |  |  |  |  |  | | Z3440 ,At5g01530  Chlorophyll a/b-binding protein CP29 precursor [Zea mays] | | | | | | |
|  |  | RAFL11-03-I17 | At5g01530 / light-harvesting chlorophyll a/b binding protein | |  |  |  |  |  | | --- | --- | --- | --- | --- | |  |  |  |  |  | | Z3440 ,At5g01530  Chlorophyll a/b-binding protein CP29 precursor [Zea mays] | | | | | | |
|  |  | RAFL09-11-O13 | At3g46780 / expressed protein | |  |  |  |  |  | | --- | --- | --- | --- | --- | |  |  |  |  |  | | Z5531 ,At3g46780  putative protein [Arabidopsis thaliana] | | | | | | |
|  |  | RAFL07-16-C20 | At5g01530 / light-harvesting chlorophyll a/b binding protein | |  |  |  |  |  | | --- | --- | --- | --- | --- | |  |  |  |  |  | | Z3440 ,At5g01530  Chlorophyll a/b-binding protein CP29 precursor [Zea mays] | | | | | | |
|  |  | RAFL09-15-H22 | At1g05190 / ribosomal protein L6p family | |  |  |  |  |  | | --- | --- | --- | --- | --- | |  |  |  |  |  | | At1g05190 ,Z3804  putative chloroplast 50S ribosomal protein, L6 [Arabidopsis thaliana] | | | | | | |
|  |  | RAFL03-06-H04 | At5g46110 / phosphate/triose-phosphate translocator, putative | |  |  |  |  |  | | --- | --- | --- | --- | --- | |  |  |  |  |  | | Z6046 ,At5g46110  triose phosphate/phosphate translocator [Flaveria pringlei] | | | | | | |
|  |  | RAFL05-08-N13 | At4g02530 / chloroplast thylakoid lumen protein | |  |  |  |  |  | | --- | --- | --- | --- | --- | |  |  |  |  |  | | At4g02530 ,Z6019  predicted protein of unknown function [Arabidopsis thaliana] | | | | | | |
| Cluster:0-2 | | |  |  | A | B | C | D | P | P' | N |
|  | A1 | | GeneTree cluster\_id:A1 |  | 6 | 73 | 84 | 4500 | 0.0039265547 | 0.102090426 | 26 |
|  |  | RAFL07-14-F21 | At1g54780 / thylakoid lumen 18.3 kDa protein | |  |  |  |  |  | | --- | --- | --- | --- | --- | |  |  |  |  |  | | Z3344 ,At1g54780  unknown protein [Arabidopsis thaliana] | | | | | | |
|  |  | RAFL05-19-G04 | At1g54780 / thylakoid lumen 18.3 kDa protein | |  |  |  |  |  | | --- | --- | --- | --- | --- | |  |  |  |  |  | | Z3344 ,At1g54780  unknown protein [Arabidopsis thaliana] | | | | | | |
|  |  | RAFL04-16-N08 | At5g47110 / Lil3 protein | |  |  |  |  |  | | --- | --- | --- | --- | --- | |  |  |  |  |  | | Z2874 ,At5g47110  Lil3 protein [Arabidopsis thaliana] | | | | | | |
|  |  | RAFL05-01-I05 | At1g54780 / thylakoid lumen 18.3 kDa protein | |  |  |  |  |  | | --- | --- | --- | --- | --- | |  |  |  |  |  | | Z3344 ,At1g54780  unknown protein [Arabidopsis thaliana] | | | | | | |
|  |  | RAFL05-17-B17 | At3g50820 / photosystem II oxygen-evolving complex 33 (OEC33) | |  |  |  |  |  | | --- | --- | --- | --- | --- | |  |  |  |  |  | | Z9108 ,At3g50820  oxygen-evolving enhancer protein 1, chloroplast precursor [Solanum tuberosum] | | | | | | |
|  |  | RAFL09-06-E16 | At2g26080 / glycine dehydrogenase (decarboxylating) (glycine decarboxylase/glycine cleavage system P-protein), putative | |  |  |  |  |  | | --- | --- | --- | --- | --- | |  |  |  |  |  | | Z6158 ,At2g26080  P-protein of the glycine cleavage system [Flaveria pringlei] | | | | | | |
|  | C1 | | GeneTree cluster\_id:C1 |  | 2 | 77 | 14 | 4570 | 0.029166238 | 0.20416367 | 7 |
|  |  | RAFL08-16-E05 | At5g17920 / 5-methyltetrahydropteroyltriglutamate--homocysteine S-methyltransferase | |  |  |  |  |  | | --- | --- | --- | --- | --- | |  |  |  |  |  | | At5g17920 ,Z4970  cobalamine-independent methionine synthase [Solenostemon scutellarioides] | | | | | | |
|  |  | RAFL09-09-A21 | At5g17920 / 5-methyltetrahydropteroyltriglutamate--homocysteine S-methyltransferase | |  |  |  |  |  | | --- | --- | --- | --- | --- | |  |  |  |  |  | | At5g17920 ,Z4970  cobalamine-independent methionine synthase [Solenostemon scutellarioides] | | | | | | |
| Cluster:0-1 | | |  |  | A | B | C | D | P | P' | N |
|  | A1 | | GeneTree cluster\_id:A1 |  | 10 | 96 | 80 | 4477 | 3.0162279E-5 | 7.8421924E-4 | 26 |
|  |  | RAFL06-13-A08 | At1g44575 / photosystem II 22kDa protein -related | |  |  |  |  |  | | --- | --- | --- | --- | --- | |  |  |  |  |  | | Z7449 ,At1g44575  precursor of photosystem II subunit (22KDa) [Nicotiana tabacum] | | | | | | |
|  |  | RAFL07-12-E12 | At2g21330 / fructose-bisphosphate aldolase, putative | |  |  |  |  |  | | --- | --- | --- | --- | --- | |  |  |  |  |  | | Z3569 ,At2g21330  homologous to plastidic aldolases [Solanum tuberosum] | | | | | | |
|  |  | RAFL05-18-I22 | At1g44575 / photosystem II 22kDa protein -related | |  |  |  |  |  | | --- | --- | --- | --- | --- | |  |  |  |  |  | | Z7449 ,At1g44575  precursor of photosystem II subunit (22KDa) [Nicotiana tabacum] | | | | | | |
|  |  | RAFL09-17-N23 | At2g21330 / fructose-bisphosphate aldolase, putative | |  |  |  |  |  | | --- | --- | --- | --- | --- | |  |  |  |  |  | | Z3569 ,At2g21330  homologous to plastidic aldolases [Solanum tuberosum] | | | | | | |
|  |  | RAFL07-18-J01 | At2g21330 / fructose-bisphosphate aldolase, putative | |  |  |  |  |  | | --- | --- | --- | --- | --- | |  |  |  |  |  | | Z3569 ,At2g21330  homologous to plastidic aldolases [Solanum tuberosum] | | | | | | |
|  |  | RAFL06-08-I11 | At2g46820 / expressed protein | |  |  |  |  |  | | --- | --- | --- | --- | --- | |  |  |  |  |  | | Z302 ,At2g46820  unknown protein [Arabidopsis thaliana] | | | | | | |
|  |  | RAFL07-12-M09 | At2g21330 / fructose-bisphosphate aldolase, putative | |  |  |  |  |  | | --- | --- | --- | --- | --- | |  |  |  |  |  | | Z3569 ,At2g21330  homologous to plastidic aldolases [Solanum tuberosum] | | | | | | |
|  |  | RAFL07-18-C20 | At2g21330 / fructose-bisphosphate aldolase, putative | |  |  |  |  |  | | --- | --- | --- | --- | --- | |  |  |  |  |  | | Z3569 ,At2g21330  homologous to plastidic aldolases [Solanum tuberosum] | | | | | | |
|  |  | RAFL08-09-L12 | At3g26060 / peroxiredoxin -related | |  |  |  |  |  | | --- | --- | --- | --- | --- | |  |  |  |  |  | | At3g26060 ,Z7102  peroxiredoxin Q [Sedum lineare] | | | | | | |
|  |  | RAFL08-18-C10 | At2g21330 / fructose-bisphosphate aldolase, putative | |  |  |  |  |  | | --- | --- | --- | --- | --- | |  |  |  |  |  | | Z3569 ,At2g21330  homologous to plastidic aldolases [Solanum tuberosum] | | | | | | |
| Cluster:9-2 | | |  |  | A | B | C | D | P | P' | N |
|  | A1 | | GeneTree cluster\_id:A1 |  | 4 | 63 | 86 | 4510 | 0.039566863 | 1.0287384 | 26 |
|  |  | RAFL05-02-L02 | At1g19180 / expressed protein | |  |  |  |  |  | | --- | --- | --- | --- | --- | |  |  |  |  |  | | Z684 ,Z931 ,Z3688 ,At1g19180  unknown protein [Arabidopsis thaliana]  unknown protein [Arabidopsis thaliana] | | | | | | |
|  |  | RAFL09-09-P15 | At1g19180 / expressed protein | |  |  |  |  |  | | --- | --- | --- | --- | --- | |  |  |  |  |  | | Z684 ,Z931 ,Z3688 ,At1g19180  unknown protein [Arabidopsis thaliana]  unknown protein [Arabidopsis thaliana] | | | | | | |
|  |  | RAFL06-10-F03 | At1g19180 / expressed protein | |  |  |  |  |  | | --- | --- | --- | --- | --- | |  |  |  |  |  | | Z684 ,Z931 ,Z3688 ,At1g19180  unknown protein [Arabidopsis thaliana]  unknown protein [Arabidopsis thaliana] | | | | | | |
|  |  | RAFL05-03-O21 | At5g05600 / oxidoreductase, 2OG-Fe(II) oxygenase family | |  |  |  |  |  | | --- | --- | --- | --- | --- | |  |  |  |  |  | | Z7465 ,At5g05600  leucoanthocyanidin dioxygenase-like protein [Arabidopsis thaliana] | | | | | | |
