# Supplementary figures and images for: A flexible representation of omic knowledge for thorough analysis of microarray data
Source: Plant Methods. 2006 Mar 2;2:5. doi: 10.1186/1746-4811-2-5 (PMC1421397; doi:10.1186/1746-4811-2-5)

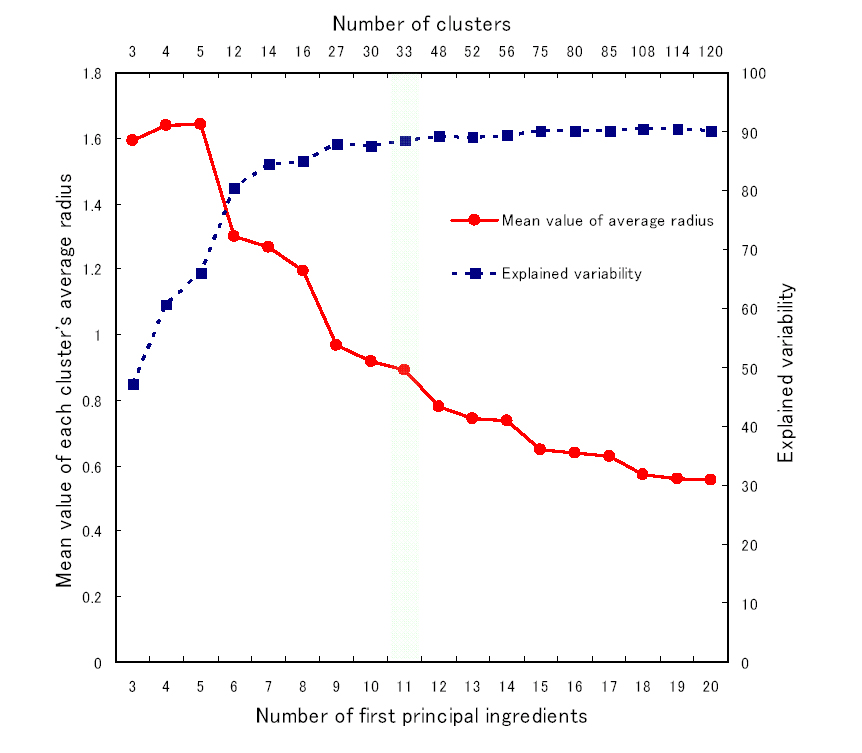

Supplement: Additional File 7 — The relationship between the number of the first principal ingredient in the BL-SOM clusters and the index of clustering. The first principal ingredient = 3 to 20 (clustering 3 to 120) of BL-SOM clustering of the microarray data under drought stress and the index of clustering (the "mean value of each cluster's average radius" and "explained variability") is plotted. It is used for analysis because the cluster with X = 11, clustering = 33, separated enough. [file 1746-4811-2-5-S7.jpeg]
